# Genome-based taxonomic classification of the genus *Sulfitobacter* along with the proposal of a new genus *Parasulfitobacter* gen. nov. and exploring the gene clusters associated with sulfur oxidation

**DOI:** 10.1186/s12864-024-10269-3

**Published:** 2024-04-22

**Authors:** Xiaokun Xu, Mengdan He, Qingjie Xue, Xiuzhen Li, Ang Liu

**Affiliations:** 1 Department of Pathogenic Biology, College of Basic Medicine, Jining Medical University, 272067 Jining, Shandong P. R. China; 2 School of Basic Medical Sciences, Shandong Second Medical University, 261042 Weifang, Shandong P. R. China

**Keywords:** *Parasulfitobacter* gen. nov., *Parasulfitobacter algicola* comb. nov., Sulfur oxidation system, *Sulfitobacter*, DMSP degradation

## Abstract

**Background:**

The genus *Sulfitobacter*, a member of the family *Roseobacteraceae*, is widely distributed in the ocean and is believed to play crucial roles in the global sulfur cycle. However, gene clusters associated with sulfur oxidation in genomes of the type strains of this genus have been poorly studied. Furthermore, taxonomic errors have been identified in this genus, potentially leading to significant confusion in ecological and evolutionary interpretations in subsequent studies of the genus *Sulfitobacter*. This study aims to investigate the taxonomic status of this genus and explore the metabolism associated with sulfur oxidation.

**Results:**

This study suggests that *Sulfitobacter algicola* does not belong to *Sulfitobacter* and should be reclassified into a novel genus, for which we propose the name *Parasulfitobacter* gen. nov., with *Parasulfitobacter algicola* comb. nov. as the type species. Additionally, enzymes involved in the sulfur oxidation process, such as the sulfur oxidization (Sox) system, the disulfide reductase protein family, and the sulfite dehydrogenase (SoeABC), were identified in almost all *Sulfitobacter* species. This finding implies that the majority of *Sulfitobacter* species can oxidize reduced sulfur compounds. Differences in the modular organization of *sox* gene clusters among *Sulfitobacter* species were identified, along with the presence of five genes with unknown function located in some of the *sox* gene clusters. Lastly, this study revealed the presence of the demethylation pathway and the cleavage pathway used by many *Sulfitobacter* species to degrade dimethylsulfoniopropionate (DMSP). These pathways enable these bacteria to utilize DMSP as important source of sulfur and carbon or as a defence strategy.

**Conclusions:**

Our findings contribute to interpreting the mechanism by which *Sulfitobacter* species participate in the global sulfur cycle. The taxonomic rearrangement of *S. algicola* into the novel genus *Parasulfitobacter* will prevent confusion in ecological and evolutionary interpretations in future studies of the genus *Sulfitobacter*.

**Supplementary Information:**

The online version contains supplementary material available at 10.1186/s12864-024-10269-3.

## Background

The genus *Sulfitobacter* is a member of the family *Roseobacteraceae* of the class *Alphaproteobacteria*. This genus was first proposed by Sorokin [1] describing two strains of heterotrophic bacteria with high sulfide oxidase activity isolated from the H_2_S-O_2_ interface of the Black Sea. As of March 10, 2023, the genus *Sulfitobacter* comprised 24 validated species according to NCBI taxonomy and LPSN (https://lpsn.dsmz.de/genus/sulfitobacter). Members of the family *Roseobacteraceae* constitute up to 20% of coastal marine bacterial populations [2], making this family one of the most abundant groups of marine bacteria. *Sulfitobacter* strains are abundant and widely distributed across diverse ocean habitats [3], including seawater [4, 5], sediment [6], tidal flat [7], starfish [8], seagrass [8], brown algae [9] and coral [10]. Besides oxidizing sulfite and thiosulfate, *Sulfitobacter* strains also have the capability to degrade diatom-derived dimethylsulfoniopropionate (DMSP), an organic sulfur-containing compound presents globally in very large amounts (10^9^ tons or more per year) [11]. As a result, *Sulfitobacter* strains are considered significant contributors to the organic sulfur cycle in marine environments. Strains of *Sulfitobacter* also attract much attention as they could produce bioactive metabolites [12–14], accumulate tungsten [15], mitigate harmful algal blooms [16] and degrade hydrocarbon [17].

Taxonomy forms the fundamental basis for microbiology, and current microbial taxonomy relies solely on an approach known as polyphasic taxonomy [18]. Over the past several decades, 16S rRNA gene phylogeny has played a central role in polyphasic taxonomy. Recently, with advancements in next-generation whole-genome sequencing, more accurate genetic and phylogenetic methods such as phylogenomic analysis [19], average amino acid identity (AAI) [20], average nucleotide identity (ANI) [21], and digital DNA–DNA hybridization (dDDH) [22] have been adopted, significantly enhancing the accuracy of taxonomic assignments. As a result, many earlier taxonomic classifications have undergone re-evaluation and modification using genome-based analysis [23, 24]. It has been reported that compared to genome-based phylogenetic analyses, 16S rRNA gene-based phylogeny lacks the resolution necessary for proper phylogenetic reconstruction in *Roseobacteraceae* species [25]. In this study, the taxonomic status of the genus *Sulfitobacter* and the metabolism associated with organic sulfur cycling were explored based on genome analysis. The taxonomy of the genus *Sulfitobacter* has been re-evaluated, proposing its re-classified into *Sulfitobacter* sensu stricto, along with the establishment of a novel genus, *Parasulfitobacter* gen. nov. Our findings aim to offer a deeper insight into the genus *Sulfitobacter*, provide guidance for future taxonomic endeavors related to this genus, and mitigate potential inaccuracies in taxonomic classification.

## Methods

### 16S rRNA gene and genome sequences collection

The 16S rRNA gene sequences of the type strains of the validated species within the genus *Sulfitobacter* and closely related species were downloaded from EzBioCloud database, and their accession numbers were shown in Fig. S1. Additionally, 18 genome sequences of type strains within the genus *Sulfibobacter* were downloaded from the NCBI GenBank assembly database. For a comprehensive phylogenomic analysis of the genus *Sulfitobacter*, 17 genome sequences of related type strains within the genera *Roseobacter*, *Pseudosulfitobacter*, *Ruegeria*, *Roseivivax*, *Pelagimonas*, *Litorivita*, *Arenibacterium*, *Yoonia*, *Loktanella*, and *Brevirhabdus* were obtained from the NCBI GenBank assembly database. The genome sequence of *Hyphomonas polymorpha* PS728^T^ was also downloaded and employed as the outgroup in the phylogenomic analysis. Details regarding the genome sequence properties of the mentioned 36 type strains were presented in Table S1 within the supplemental material. To calculate AAI values between the genome sequences of *S. algicola* 1151^T^ and the type strains of the type species within the genera belonging to the family *Roseobacteraceae*, 129 genome sequences of type strains of the type species within the genera belonging to family *Roseobacteraceae* were downloaded from the GenBank assembly database. The accession numbers of the genome sequences of these 129 type strains were listed in Table S2 in the supplemental material.

### 16S rRNA gene-based and genome-based phylogenetic analysis

Multiple sequence alignment of the obtained 16S rRNA gene sequences was conducted using the Muscle program [26] integrated in MEGA software version X [27]. A phylogenetic tree was then established utilizing the maximum-likelihood (ML) method with MEAG X. The selected substitution model for this analysis was Kimura 2-parameter and Gama Distributed with Invariant sites (K2 + G + I), and the tree supported topologies were evaluated through bootstrap values calculated based on 1000 replications. In an effort to comprehensively analyze the taxonomy of the genus *Sulfitobacter*, genome-based phylogenetic trees were reconstructed using three sets of sequences: the nucleotide sequence of an up-to-date bacterial core gene set (UBCG) [19] consisting of 92 genes, the amino acid sequence of UBCG, and the amino acid sequence of single-copy orthologous clusters (OCs) comprising 488 proteins. For the reconstruction of the phylogenomic tree based on the nucleotide sequence of UBCG, a codon-based alignment file (Additional file 2) was generated using a JAVA program [19] with the ‘-a codon’ option from the 36 genome sequences (Table S1). This file was utilized to construct a ML tree by PhyML 3.0 [28] with the selected substitution model being GTR, and the tree supported topologies were evaluated through bootstrap values calculated based on 100 replications. For the reconstruction of the phylogenomic tree based on the amino acid sequence of UBCG, an alignment file (Additional file 3) was generated using the same JAVA program [19] with the ‘-a aa’ option. The resulting file was utilized to construct a ML tree following the same steps as with the nucleotide sequence of UBCG except that the selected substitution model was LG. During reconstruction of the phylogenomic tree based on OCs, the amino acid sequences were identified by comparing whole protein sequences pairwise with the execution of Proteinortho version 6 [29] with the command ‘-e = 1e-5 -cov = 50 -identity = 50’. Subsequently, single-copy OCs were filtered using an in-house perl script (Additional file 4), and the resulting file (Additional file 5) was used to construct a ML tree following the same steps as with the amino acid sequence of UBCG.

### Calculation of genome-based similarity indices for taxa delineation

AAI was computed using the CompareM (https://github.com/dparks1134/ CompareM) program with the parameters of 40% amino acid identity and 50% coverage length. Alignment fractions (AF) and genome-wide ANI (gANI) values were calculated through the Microbial Species Identifier (MiSI) method using ANIcalculator 2014 − 127, version 1.0 (https://ani.jgi.doe.gov/html/anicalculator.php) [30]. Percentage of conserved proteins (POCP) was calculated based on an approach described by Qin et al. [31]..

### Comparative genome analysis

Annotated genome files obtained from the NCBI GenBank assembly database were manually reviewed to identify genes related to sulfite oxidation and DMSP degradation pathways. Functional annotation of Open Reading Frames (ORFs) was also conducted using the KEGG automatic annotation server (KASS v2.1, https://www.genome.jp/tools/kaas/) [32] with the KEGG database (http://www.genome.jp/kegg/). The functional annotated genes were categorized using KEGG orthology (KO) numbers. Preparation of the Venn diagram and identification of the core genomes were conducted using EVenn (http://www.ehbio.com/test/venn) [33]. Genes sharing KEGG orthologs in the genomes of all strains were classified as the core genome.

### Analysis of the phenotypic characteristics

Phenotypic characteristics of the *Sulfitobacter* species were collected and reviewed from the original descriptions in various studies.

## Results and discussions

### Phylogenetic and phylogenomic analysis of the genus ***Sulfitobacter***

In order to assess the effectiveness of 16S rRNA gene sequence-based phylogenetic reconstruction in the taxonomy of the *Sulfitobacter* species, a ML tree was reconstructed based on 16 S rRNA sequences of the type strains within the genus *Sulfitobacter* and closely related genera (Fig. S1). Within this ML tree, the genus *Sulfitobacter* appears paraphyletic due to the presence of type strains from the genera *Roseobacter* and *Arenibacterium*. Furthermore, the ML tree demonstrates inadequate bootstrap support for the majority of branches. This observation leads us to conclude that 16S rRNA gene sequences lack the resolution required for precise phylogenetic reconstruction within *Sulfitobacter* species.

**Fig. 1:**
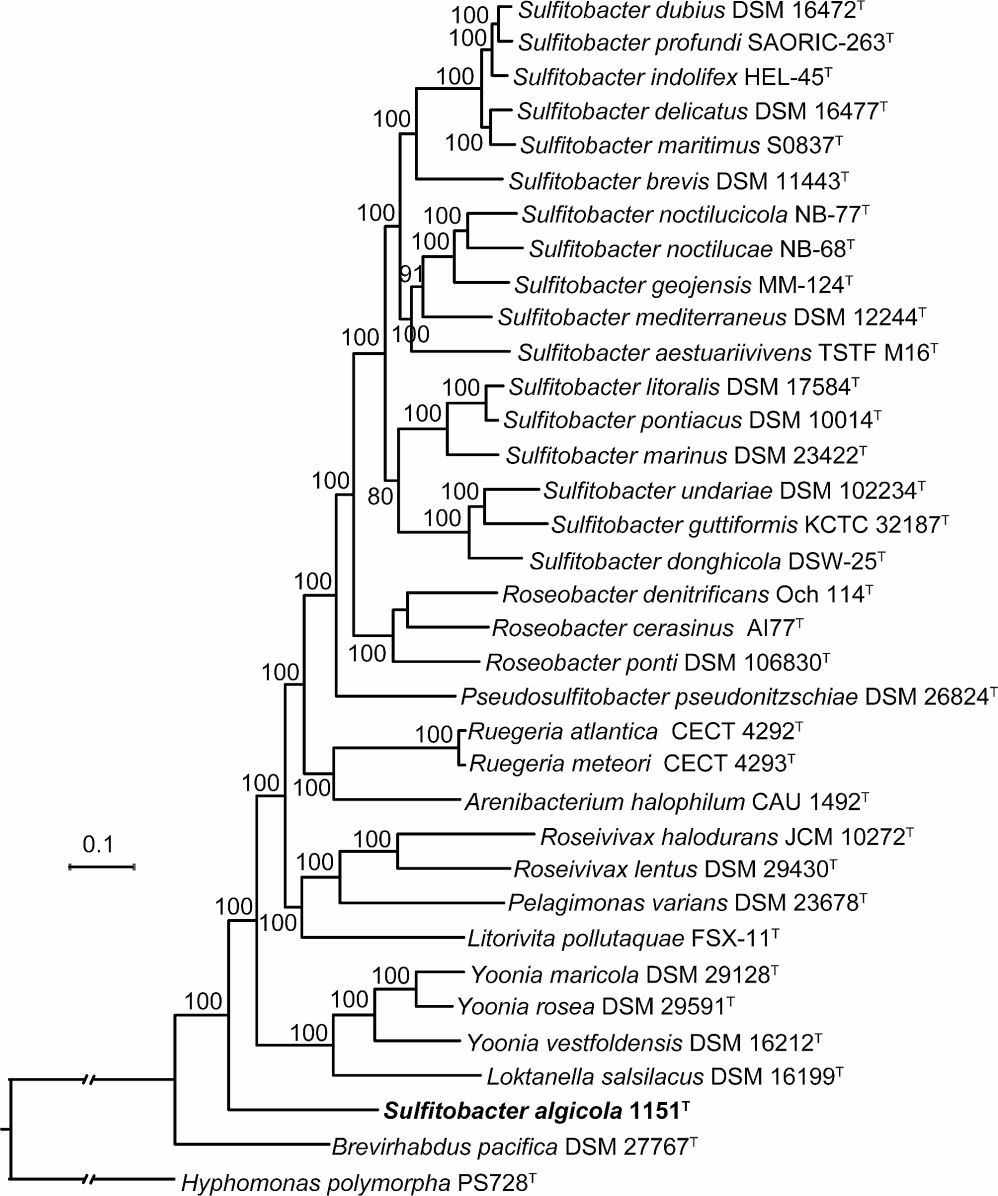
Maximum-likelihood phylogenetic tree based on the amino acid sequences of OCs consisting of 488 proteins of the type strains of validated species of the genus *Sulfitobacter* and members of closely related taxa whose genome sequences were available. *H. polymorpha* PS728^T^ is used as an outgroup. *S. algicola* 1151^T^ is shown in bold. Bootstrap percentages (> 70%) based on 100 replicates are shown at nodes. Bar, 0.1 substitutions per nucleotide position

**Fig. 2:**
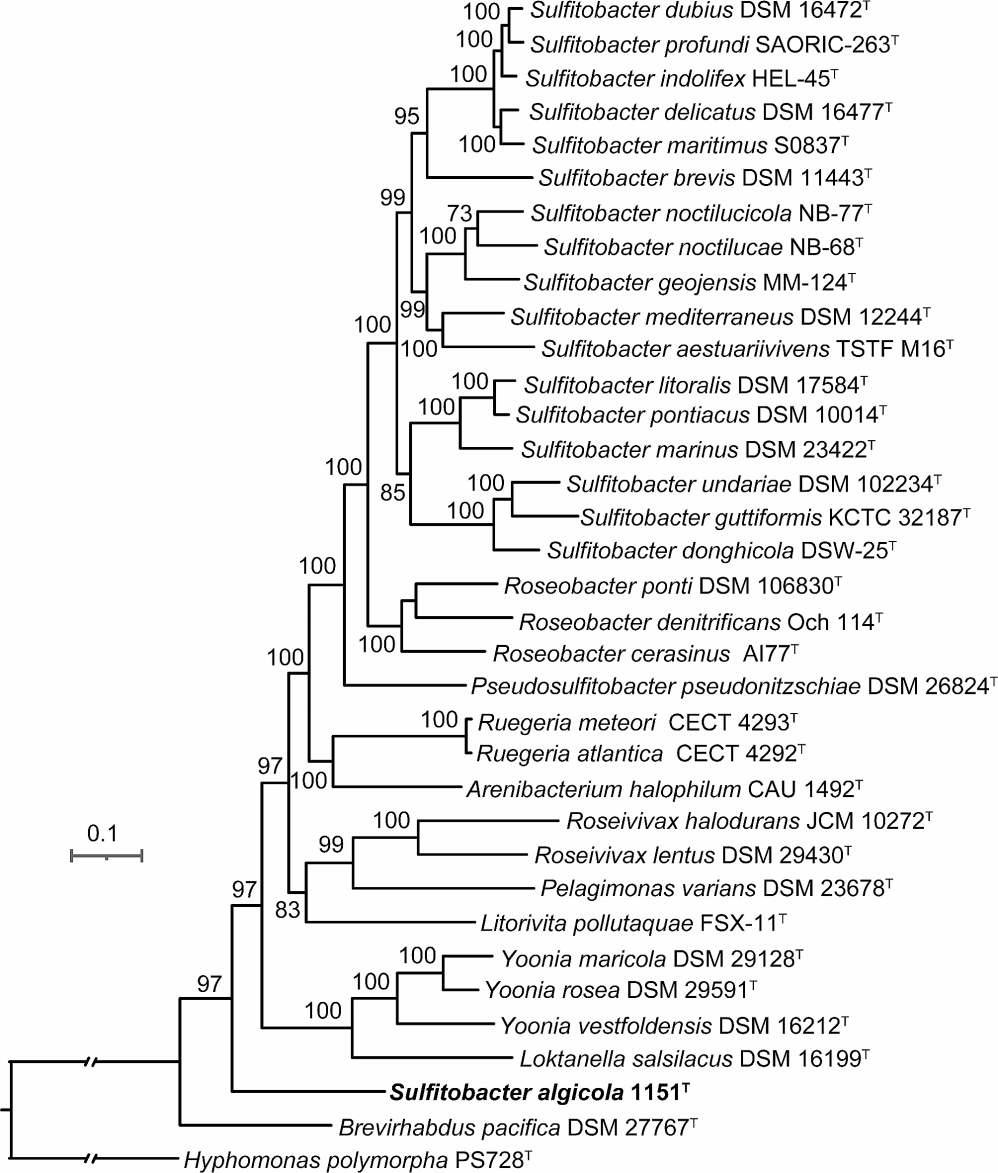
Maximum-likelihood phylogenetic tree based on the amino acid sequences of UBCG of the type strains of validated species of the genus *Sulfitobacter* and members of closely related taxa whose genome sequences were available. *H. polymorpha* PS728^T^ is used as an outgroup. *S. algicola* 1151^T^ is shown in bold. Bootstrap percentages (> 70%) based on 100 replicates are shown at nodes. Bar, 0.1 substitutions per nucleotide position

It is accepted that the multigene-based phylogenomic approach is much more consistent and dependable, thus being the preferred method for inferring phylogenetic relationships among prokaryotes. In this investigation, genome-based phylogeny is used as the primary guideline for revisiting the taxonomic status of the genus *Sulfitobacter*. Phylogenomic trees were reconstructed using three sets of sequences: the amino acid sequence of OCs, the amino acid sequence of UBCG, and the nucleotide sequence of UBCG. The three phylogenomic trees (Figs. 1 and 2, and Fig. S2) display robust bootstrap support for the majority of the branches, suggesting that phylogenomic analysis should be more suitable for inferring relationships among *Sulfitobacter* species. All trees show that the majority of *Sulfitobacter* species, including the type species *S. pontiacus* DSM 10,014^T^, clustered together, except for *S. algicola* 1151^T^. In both the phylogenomic trees based on the amino acid sequences of OCs (Fig. 1) and UBCG (Fig. 2), *S. algicola* 1151^T^ forms a distinct branch quite far away from other *Sulfitobacter* type strains. In the phylogenomic tree based on the nucleotide sequence of UBCG (Fig. S2), *S. algicola* 1151^T^ forms a cluster with *Pelagimonas varians* DSM 23,678^T^ and *Litorivita pollutaquae* FSX-11^T^ with a very low bootstrap support value (7%), and this cluster is also quite far away from other *Sulfitobacter* species. The extended branch length indicates a distant genomic relationship between *S. algicola* 1151^T^ and *P. varians* DSM 23,678^T^ as well as *L. pollutaquae* FSX-11^T^. Based on these phylogenomic trees, it is suggested that *S. algicola* does not belong to the genus *Sulfitobacter* and should be reclassified into a novel genus. The phylogemonic trees also show that *Pseudosulfitobacter pseudonitzschiae* DSM 26,824^T^ forms a separate cluster from the primary *Sulfitobacter* clade, supporting the proposal that this species does not belong to *Sulfitobacter* and should be reclassified into the novel genus *Pseudosulfitobacter* [34]. Moreover, the three phylogemonic trees demonstrate that genera such as *Roseobacter*, *Ruegeria*, *Roseivivax* and *Yoonia* are monophyletic, suggesting that these genera are well defined.

### Assessment of genome-based similarity indexes for genus

AAI is the most widely used genomic amino acid-level comparison for demarcating genera. In addition, percentage of conserved proteins (POCP), alignment fractions (AF) and genome-wide ANI (gANI) are also suggested to be used for genus delineation. As prokaryotic taxa display a continuum of AAI, POCP, AF and gANI values, the discrete boundaries for genus are difficult to define. Results of Luo et al. [20]. indicate that AAI values among members of related but different genera typically range between 60 and 80%, with a maximum not exceeding 85%. In a taxonomic study of species of the roseobacter group, Wirth et al. [35]. employed a gradient of AAI to delimit genera that is defined by two values: a minimum value (80%) below which separating species into different genera should be considered and a maximum value (85%) above which combining species into the same genus should be considered. Nicholson et al. [36]. proposed a similar strategy in using AAI to delimit genera: AAI between the type strain of one species and the type strain of the type species of one genus should be greater than 76% so as to assign this species to the same genus. In addition, the AAI among all type strains of one specific genus should be greater than 74%. AAI has been applied for delimiting genera in various prokaryotic families, including *Flavobacteriaceae* [37], *Roseobacteraceae* [35], *Colwelliaceae* [38] and *Weeksellaceae* [36]. It is recommended for application in other prokaryotic genera as well [35]. POCP has also been used to estimate evolutionary distance between two strains, with a cut-off for prokaryotic genera set at 50% [31]. However, many studies argued that this 50% POCP cut-off might be overly conservative [35]. Consequently, while POCP has been commonly used to estimate evolutionary distance, the 50% cut-off for genera is often not applied. In this study, POCP is employed for estimating evolutionary distance without applying the 50% cutoff. It is reported that a combination of gANI and AF between two genomes has been shown to accurately reflect genomic relatedness, aiding in the delineation of species or genus [30]. Barco et al. [39]. reported that the AF values of the estimated genus inflection points have a mean of 0.333, with a median of 0.349. Additionally, the gANI values of the estimated genus inflection points have a mean of 73.10%, with a median of 73.08%. In this study, AAI, POCP, gANI and AF were all used for analyzing the genomic relatedness of different species.

The AAI value between *S. algicola* 1151^T^ and the type strain of the type species of *Sulfitobacter*, *S. pontiacus* DSM 10,014^T^, was calculated to be 68.0%, apparently below the 76.0% threshold. AAI values between *S. algicola* 1151^T^ and the other 16 type strains of the genus *Sulfitobacter* ranged from 67.3 to 68.5%, again falling below the suggested 74.0% cutoff. Consequently, the AAI indexes support our proposal derived from phylogenomic analysis that *S. algicola* does not belong to *Sulfitobacter*. The AAI values between *S. pontiacus* DSM 10,014^T^ and the other 16 type strains of the genus *Sulfitobacter* ranged from 73.5 to 92.4%, indicating a closer genomic relatedness with *S. pontiacus* DSM 10,014^T^ than with *S. algicola* 1151^T^. Althrough AAI values between *S. pontiacus* DSM 10,014^T^ and some of these 16 type strains were calculated to be lower than 76.0%, as the phylogenomic trees show that all of the selected type strains of *Sulfitobacter* other than *S. algicola* 1151^T^ cluster together, we think there is no necessity to split these *Sulfibacter* species into different genera.

The POCP values between *S. algicola* 1151^T^ and the selected 17 type strains of *Sulfitobacter* ranged from 51.8 to 57.1%, while the POCP values among the 17 type strains of *Sulfitobacter* were calculated to be between 61.9 and 84.9%. The relatively lower POCP values suggest that *S. algicola* 1151^T^ exhibits reduced genomic relatedness with the 17 selected type strains of *Sulfitobacter*, supporting our proposal derived from phylogenomic analysis that *S. algicola* does not belong to *Sulfitobacter*.

The gANI and AF values between *S. algicola* 1151^T^ and *S. pontiacus* DSM 10,014^T^ were calculated to be 71.835% and 0.325, respectively, both lower than the reported genus inflection points of 73.08% and 0.333. The gANI and AF values between *S. pontiacus* DSM 10,014^T^ and the 16 type strains of the genus *Sulfitobacter* were calculated to be between 74.17 and 87.19% and 0.495–0.81, higher than the reported genus inflection points. Consequently, gANI and AF values further support our proposal that *S. algicola* 1151^T^ does not belong to the genus *Sulfitobacter*.

The phylogenomic analysis suggests that *S. algicola* 1151^T^ should be reclassified into a novel genus. To further analyze this proposal, the available genomes of the type strains of the type species of the family *Roseobacteraceae* from GenBank assembly database were downloaded. Subsequently, the AAI values between all the selected type strains and *S. algicola* 1151^T^ were calculated. These AAI values ranged from 55.62 to 69.52%, apparently lower than the 76% threshold, supporting the proposal derived from phylogenomic analysis that *S. algicola* should be reclassified into a novel genus.

### Genomic and phenotypic features analysis

Genomic and phenotypic features have also been widely used in bacterial taxonomy. In this study, we carefully reviewed the genomic and phenotypic features of the *Sulfitobacter* species. The distinctive characteristics between *S. algicola* 1151^T^ and the selected 17 type strains of *Sulfitobacter* are listed in Table 1. Significant differences were observed in features such as growth temperature, polar lipid composition, fatty acid compositions and genomic DNA G + C content. Specifically, the grow temperature of *S. algicola* 1151^T^ is higher than that of the selected 17 type strains of *Sulfitobacter*. The proportion of C_20:1_*ω7c* in the fatty acid profile of *S. algicola* 1151^T^ was measured to be 29.7%, whereas C_20:1_*ω7c* was not detected or the proportion was measured to be less than 0.5% in the fatty acid profiles of the selected 17 type strains of *Sulfitobacter*. The proportion of C_18:0_ in the fatty acid profile of *S. algicola* 1151^T^ was measured to be 11.7%, whereas C_18:0_ was not detected or the proportion was measured to be less than 2.0% in the fatty acid profiles of the selected 17 type strains of *Sulfitobacter*. The proportion of summed feature 8 (C_18:1_*ω*7*c* and/or C_18:1_*ω*6*c*) in the fatty acid profile of *S. algicola* 1151^T^ was measured to be 44.1%, whereas the proportions of summed feature 8 in the fatty acid profiles of the selected 17 type strains of *Sulfitobacter* ranged between 50.0 and 89.6%. The genomic DNA G + C content of *S. algicola* 1151^T^ was measured to be 51.8 mol%, whereas the genomic DNA G + C contents of the selected 17 type strains of *Sulfitobacter* range from 54.7 mol% to 61.2 mol%. In summary, results of the genomic and phenotypic features analysis support our proposal that *S. algicola* does not belong to the genus *Sulfitobacter*.

**Table 1:** Characteristics that differentiate *Sulfitobacter algicola* 1151^T^ and the selected 17 type strains of *Sulfitobacter*

Characteristic	1	2
Growth at 10ºC	-^a^	+^b^
Growth at 37ºC	+^a^	V(-)^b^
Nitrate reduction	-^a^	V(-)^b^
Catalase	+^a^	V(+)^b^
Oxygen requirement	Aerobic^a^	Aerobic or facultatively aerobic^b^
Major fatty acids		
Content of summed feature 8 (%)	44.1^a^	50.0-89.6^b^
Content of C_20:1_*ω7c* (%)	29.7^a^	< 0.5^b^
Content of C_18:0_ (%)	11.7^a^	ND-2.0^b^
Contain DPG as the major polar lipid	-^a^	V(+)^b^
*sox* gene cluster	-	+^c^
G + C (mol%) calculated from the genomes	51.8	54.7–61.2

Strains: 1, *S. algicola* 1151^T^; 2, the selected 17 type strains of *Sulfitobacter* including *S. brevis* DSM 1143^T^, *S. delicatus* DSM 16,477^T^, *S. dubius* DSM 16,472^T^, *S. indolifex* HEL-45^T^, *S. aestuariivivens* TSTF-M16^T^, *S. geojensis* MM-124^T^, *S. maritimus* S0837^T^, *S. profundi* SAORIC-263^T^, *S. mediterraneus* DSM 12,244^T^, *S. noctilucae* NB-68^T^, *S. noctilucicola* NB-77^T^, *S. donghicola* DSW-25^T^, *S. guttiformis* KCTC 32,187^T^, *S. undariae* DSM 102,234^T^, *S. litoralis* DSM 17,584^T^, *S. marinus* DSM 23,422^T^ and *S. pontiacus* DSM 10,014^T^. +, positive test result; -, negative test result; V, variable results in different species; V(+),variable results in different species and most are positive; V(+),variable results in different species and most are negative, ND, not detected

^a^, data from [51]

^b^, data from [1, 4–9, 13, 43, 52–55]

^c^, except for *S. guttiformis* KCTC 32,187^T^

To investigate the metabolic features of *S. algicola* 1151^T^ and the selected 17 type strains of *Sulfitobacter*, we conducted functional analyses utilizing KEGG database categories. The overall relative abundances of KEGG functional genes in both *S. algicola* 1151^T^ and the selected 17 type strains of *Sulfitobacter* were found to be similar (Fig. S3). Carbohydrate metabolism, amino acid metabolism, membrane transport, transport and catabolism, and translation-associated genes exhibit high abundance in both *S. algicola* 1151^T^ and the selected 17 type strains. The core genome of the selected 17 type strains of *Sulfitobacter* comprises 1090 genes (Fig. S4), with translation, amino acid metabolism and carbohydrate metabolism-associated genes being notably abundant in the core genome (Fig. S5).

### Sulfur oxidation and DMSP degradation pathways analysis

Sulfur oxidation is a critical component of the Earth’s sulfur cycle. The sulfur element can exist in a variety of oxidation states ranging from − 2 to + 6, yielding various sulfur compounds such as thiosulfate (S_2_O_3_^2−^), sulfite (SO_3_^2−^), sulfide (S_2_^2−^) and sulfate (SO_3_^2−^). Reduced sulfur compounds are oxidized to sulfur or sulfate by a community of bacteria which are called sulfur-oxidizing bacteria (SOB). Within this bacterial community, various enzymes and proteins involved in sulfur oxidation have been discovered. A central sulfur oxidization pathway, known as the sulfur oxidization (Sox) system, possesses the capability to oxidize thiosulfate, sulfide, sulfite, and elemental sulfur to sulfate [40]. Additionally, some other enzymes participating in sulfur oxidation have also been identified. For sulfide oxidation, there are two related enzymes that belong to the disulfide reductase protein family: flavocytochrome *c* sulfide dehydrogenase (FCC) and sulfide: quinone reductase (SQR). Oxidation of sulfur to sulfite by the dissimilatory sulfite reductase (rDSR) system has been experimentally proven [41]. Regarding the oxidation of sulfite to sulfate, two pathways have been reported: one (SorAB) involves a sulfite dehydrogenase reducing cytochrome *c*, while the other (SoeABC) involves reducing a quinone [42].

**Fig. 3:**
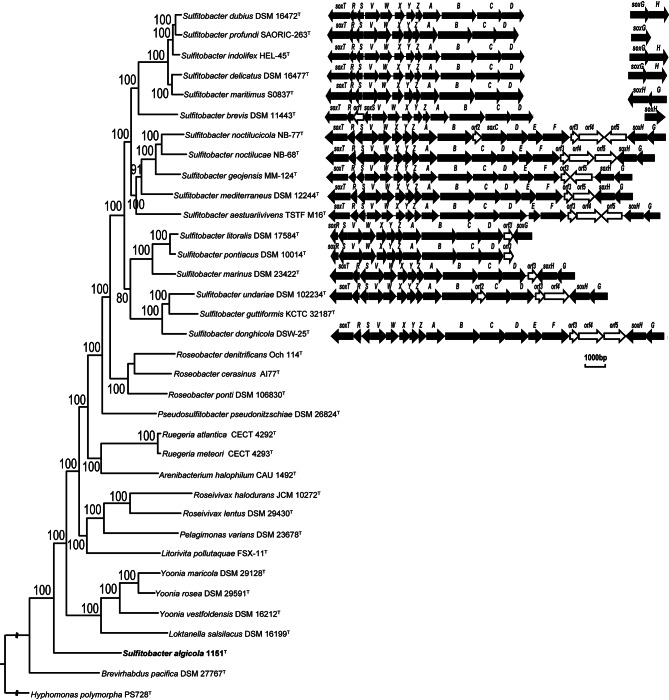
The modular organization of *sox* gene cluster of the selected 17 type strains of *Sulfitobacter* and *S. algicola* 1151^T^. The five genes with unknown function (*orf1-5*) are marked by hollow arrows

It has been documented that certain *Sulfitobacter* species are positive for oxidizing reduced sulfur compounds [1, 43]. In our investigation, we searched the enzymes involved in the sulfur oxidation process in the genomes of *Sulfitobacter* species. Initially, our focus was on the *sox* gene cluster. It was found that the *sox* gene cluster could be identified in all the 17 type strains of *Sulfitobacter* species except for *S. guttiformis* KCTC 32,187^T^. Notably, the *sox* gene cluster could not be identified in *S. algicola* 1151^T^. The modular organization of the *sox* gene clusters varies among the selected type species (Fig. 3). All identified *sox* gene clusters comprise *soxRSVWXYZABCD* genes, with the presence of *soxT*, *soxE, soxF*, *soxG* and *soxH* in some of these clusters (Fig. 3, Table S3). Additionally, five genes (*orf1-5*) with unknown function were identified in certain *sox* gene clusters (Fig. 3, Table S3). In the *sox* gene cluster of *S. brevis* DSM 1143^T^, *orf1*, the product of which is annotated as heme-binding protein, is positioned between *soxR* and *soxS*. In the *sox* gene clusters of *S. noctilucicola* NB-77^T^ and *S. undariae* DSM 102,234^T^, *orf2*, the product of which is annotated as DsrE family protein, occupies the space between *soxB* and *soxC*. In the *sox* gene clusters of *S. noctilucicola* NB-77^T^, *S. noctilucae* NB-68^T^, *S. aestuariivivens* TSTF-M16^T^ and *S. donghicola* DSW-25^T^, *orf3*, *orf4* and *orf5*, the products of which are annotated as DUF302 domain-containing protein, 5-aminolevulinate synthase and YeeE/YedE family protein, respectively, are situated between *soxF* and *soxH*. In the *sox* gene clusters of *S. geojensis* MM-124^T^ and *S. mediterraneus* DSM 12,244^T^, *orf3* and *orf5* are situated between *soxF* and *soxH*. In the *sox* gene cluster of *S. undariae* DSM 102,234^T^, *orf3* and *orf4* are situated between *soxF* and *soxH*. In the *sox* gene cluster of *S. marinus* DSM 23,422^T^, *orf3* is positioned between *soxF* and *soxH*. In the *sox* gene cluster of *S. litoralis* DSM 17,584^T^, *orf3* is located between *soxF* and *soxG*. Lastly, in the *sox* gene cluster of *S. pontiacus* DSM 10,014^T^, *orf3* is positioned downstream of *soxD*. The physiological functions of these five genes in sulfur oxidation warrant further research. In the *sox* gene clusters of *S. dubius* DSM 16,472^T^, *S. profundi* SAORIC-263^T^, *S. indolifex* HEL-45^T^, *S. delicatus* DSM 16,477^T^, *S. maritimus* S0837^T^ and *S. brevis* DSM 1143^T^, certain genes, namely *soxF* and/or *soxH*, locate separately from the other genes of this gene cluster. Conversely, in the remaining selected *Sulfitobacter* species, all genes of the *sox* cluster are co-located (Fig. 3, Table S3).

Regarding sulfide oxidation, the genes (*soxEF*) encoding flavocytochrome *c* sulfide dehydrogenase (FCC) were identified in six type strains of *Sulfitobacter*, namely *S. noctilucae* NB-68^T^, *S. noctilucicola* NB-77^T^, *S. geojensis* MM-124^T^, *S. mediterraneus* DSM 12,244^T^, *S. aestuariivivens* TSTF-M16^T^ and *S. donghicola* DSW-25^T^, but not in the other 11 type strains of *Sulfitobacter* or *S. algicola* 1151^T^ (Fig. 3, Table S3). The gene encoding sulfide: quinone reductase was not identified in the selected 17 type strains of *Sulfitobacter* or *S. algicola* 1151^T^. Concerning sulfite oxidation, three genes (*soeABC*) encoding a sulfite dehydrogenase were identified in the selected type strains of *Sulfitobacter* species, excluding *S. pontiacus* DSM 10,014^T^ and *S. litoralis* DSM 17,584^T^ (Table S4). Notably, *soeABC* were also identified in *S. algicola* 1151^T^. Genes encoding the dissimilatory sulfite reductase (rDSR) system that could be used for oxidizing sulfur to sulfite were not identified in any of the selected type strains. These findings suggest that nearly all *Sulfitobacter* species possess enzymes facilitating sulfite oxidation, which will offer energy for their survival.

The sulfonium compound DMSP is produced in the oceans at petagram levels mainly by marine phytoplankton, macroalgae and bacteria for its anti-stress functions [44, 45]. DMSP could be utilized as important sulfur and carbon sources by many bacteria, among which the *Roseobacteraceae* species and SAR11 clade are the most prominent members [46]. The genus *Sulfitobacter* belongs to *Roseobacteraceae* and it is reported that some *Sulfitobacter* strains are involved in DMSP degradation. For instance, *Sulfitobacter* sp. EE-36 possesses a DMSP lyase (DddL), which facilitates the conversion of DMSP into the gas dimethylsulfide (DMS) [47]. Moreover, it is reported that *Sulfitobacter* sp. D7 could consume and metabolize algal DMSP to produce high amounts of methanethiol, and DMSP could mediate the bacterial virulence of *Sulfitobacter* sp. D7 against an oceanic bloom-forming phytoplankter [48]. Therefore, our investigation delves into the genomes of *Sulfitobacter* species to identify enzymes involved in DMSP degradation.

**Fig. 4:**
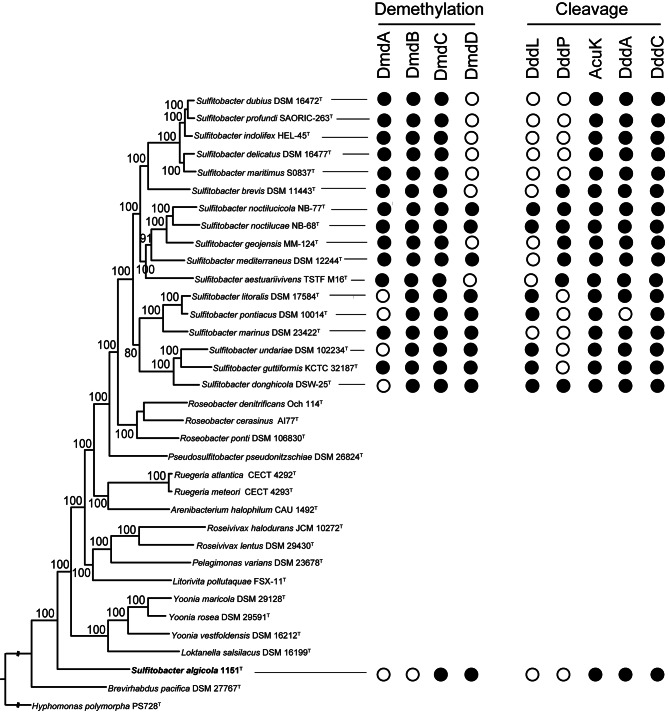
The distribution of enzymes involved in DMSP degradation in the selected 17 type strains of *Sulfitobacter* and *S. algicola* 1151^T^. Filled cycle indicates that the corresponding enzyme is present and the opened cycle indicates that the corresponding enzyme is absent

It is reported that bacteria employ two pathways for DMSP decomposition [49]: the demethylation pathway and the cleavage pathway (Fig. S6). Our findings reveal that genes encoding enzymes involved in DMSP degradation could be detected in all of the selected 17 type strains of *Sulfitobacter* and *S. algicola* 1151^T^ (Fig. 4, Table S5). Some strains possess all the complete two pathways while the other strains do not. In the demethylation pathway, *S. mediterraneus* DSM 12,244^T^, *S. noctilucae* NB-68^T^, *S. noctilucicola* NB-77^T^, *S. guttiformis* KCTC 32,187^T^ and *S. marinus* DSM 23,422^T^ were identified to have all the four genes encoding DmdA, DmdB, DmdC and DmdD responsible for degrading DMSP to acetaldehyde and methanethiol. However, the complete demethylation pathway was not identified in the other type strains of the selected type stains of *Sulfitobacter* species, as they lack either DmdD or DmdA (Fig. 4, Table S5). In the case of *S. algicola* 1151^T^, neither DmdA nor DmdB was identified.

In the cleavage pathway, the gene encoding DddD, responsible for directly degrading DMSP to DMS and 3-hydroxypropionic acid (3-HP), was not identified in the selected 17 type strains of *Sulfitobacter* or *S. algicola* 1151^T^. However, genes encoding DddL/DddP, AcuK, DddA and DddC, capable of degrading DMSP to DMS and acetyl-CoA, were identified in 10 type strains of the genus *Sulfitobacter*, These include *S. brevis* DSM 1143^T^, *S. aestuariivivens* TSTF-M16^T^, *S. geojensis* MM-124^T^, *S. mediterraneus* DSM 12,244^T^, *S. noctilucae* NB-68^T^, *S. noctilucicola* NB-77^T^, *S. donghicola* DSW-25^T^, *S. guttiformis* KCTC 32,187^T^, *S. undariae* DSM 102,234^T^ and *S. litoralis* DSM 17,584^T^. The complete cleavage pathway was not identified in the other selected type strains of the genus *Sulfitobacter* or *S. algicola* 1151^T^ as they lack DddL/DddP or DddA (Fig. 4, Table S5). These findings suggest that members of the genus *Sulfitobacter* decompose DMSP in either cleavage pathway or demethylation pathway. Notably, seven type strains of the genus *Sulfitobacter* contain DddL (Fig. 4, Table S5), a membrane-associated DMSP lyase capable of breaking down DMSP into DMS and acrylate. This indicates that these strains may also employ DMSP degradation as a defense strategy by shifting the predation pressure to non-DddL-containing bacteria [50].

## Conclusion

In this study, based on 18 publically available genomes labeled as type strains of *Sulfitobacter*, we delved into the taxonomic status of this genus and its involvement in organic sulfur cycling. Employing whole-genome phylogeny as a guideline, and supplementing it with pairwise genome comparisons, our study suggests that *S. algicola* should be reclassified into a novel genus, for which the name *Parasulfitobacter* gen. nov. is proposed. This proposal finds support in the analysis of genomic and phenotypic features. Employing such an approach ensures a consistent and reliable classification of the genus *Sulfitobacter*, a group of bacteria that is both abundant and widely distributed, garnering increasing interest in terms of organic sulfur cycling, bioactive metabolites and biotechnical investigations.

This study also highlights the widespread presence of the *sox* gene cluster in nearly all the type strains of *Sulfitobacter* species, indicating the potential of the majority of *Sulfitobacter* species to oxidize reduced sulfur compounds, thereby deriving energy for their survival. Furthermore, our findings reveal the identification of both the demethylation pathway and the cleavage pathway for degrading DMSP in many *Sulfitobacter* species. This suggests that these bacteria can utilize DMSP as important sulfur and carbon sources or employ it as a defense strategy. These insights contribute to our understanding of how *Sulfitobacter* species participate in global sulfur cycle.

Description of *Parasulfitobacter* gen. nov. and *Parasulfitobacter algicola* comb. nov. are shown in Table 2.

**Table 2:** Description of *Parasulfitobacter* gen. nov. and *Parasulfitobacter algicola* sp. nov

Guiding Code for Nomenclature	ICNP	ICNP
Nature of the type material	Species	Strain
Genus name	*Parasulfitobacter*	-
Species name	-	*Parasulfitobacter algicola*
Genus status	gen. nov.	-
Genus etymology	Pa.ra.sul.fi.to.bac’ter. Gr. prep. *para*, besides, near, like; N.L. fem. n. *Sulfitobacter* a bacteria generic name; N.L. fem. n. *Parasulfitobacter*, besides the genus *Sulfitobacter*, referring to the close relationship to this genus	-
Type species of the genus	*Parasulfitobacter algicola*	-
Specific epithet	-	*algicola*
Species status	-	sp. nov.
Species etymology	-	al.gi’co.la. L. fem. n. alga seaweed, *alga*; L. suff. -*cola* inhabitant, dweller; N.L. masc. n. *algicola* alga-dweller
Designation of the Type Strain	-	1151^T^
Strain Collection Numbers	-	KCTC 72,513^T^ = MCCC 1H00384^T^
Type Genome, MAG or SAG accession Nr.	-	JABUFE000000000
Genome status	-	Incomplete
Genome size	-	3,967
GC mol%	-	51.8
16 S rRNA gene accession nr	-	MN508060
Description of the new taxon and diagnostic traits	Cells are Gram-stain-negative, aerobic, oval-rod-shaped, non-flagellated and non-motile. Cells are 0.5–0.6 μm in width and 1.2–2.4 μm in size. NaCl is required for growth. Positive for oxidase and catalase activities, but negative for nitrate reduction activity. The sole respiratory quinone is Q-10. The major fatty acids are summed feature 8 (C_18:1_*ω*6*c* and/or C_18:1_*ω*7*c*), C_20:1_*ω*7*c* and C_18:0_. The major polar lipids are phosphatidylglycerol, phosphatidylethanolamine and phosphatidylcholine. The DNA G + C content is 51.8 mol%. A member of the family *Roseobacteraceae*, class *Alphaproteobacteria* according to 16 S rRNA gene sequence analysis and phylogenomics. The type species (and currently sole species) for the genus is *Parasulfitobacter algicola*.	Basonym: *Sulfitobacter algicola* Park et al. 2022. The description is the same to Wang et al. [51]. for *Sulfitobacter algicola*. Genome based analysis provided strong evidence for placement of this species in the genus *Parasulfitobacter*. The type strain is strain 1151^T^ (= KCTC 72,513^T^ = MCCC 1H00384^T^).
Country of origin	-	China
Region of origin	-	Coast of Weihai, China
Source of isolation	-	Marine green algae
Latitude	-	37°34′12″N
Longitude	-	122°9′0″E
Number of strains in study	-	1
Information related to the Nagoya Protocol	-	MCCC and KCTC give free access to genetic resources

### Electronic supplementary material

Below is the link to the electronic supplementary material.

Supplementary Material 1

Supplementary Material 2

Supplementary Material 3

Supplementary Material 4

Supplementary Material 5

## Data Availability

All genome sequences used in this study are publicly available in the NCBI database. All genes related to sulfur oxidizing are listed. All these data are documented in the supplementary file.
