# Supplementary material for: Genome-based taxonomic classification of the genus Sulfitobacter along with the proposal of a new genus Parasulfitobacter gen. nov. and exploring the gene clusters associated with sulfur oxidation
Source: BMC Genomics. 2024 Apr 22;25:389. doi: 10.1186/s12864-024-10269-3 (PMC11034169; doi:10.1186/s12864-024-10269-3)
Supplement: Supplementary file 1 — Supplementary Material 1 [file 12864_2024_10269_MOESM1_ESM.docx]

Supplementary materials

**Genome-based taxonomic classification of the genus *Sulfitobacter* along with the proposal of a new genus *Parasulfitobacter* gen. nov. and exploring the gene clusters associated with** **sulfur oxidation**

Xiaokun Xu^a^, Mengdan He^b^, Qingjie Xue^a^, Xiuzhen Li^a^, Ang Liu^a,^*

**Affiliation:**

^a^ Department of Pathogenic Biology, College of Basic Medicine, Jining Medical University, Jining 272067, Shandong, P. R. China

^b^ College of basic Medicine, Weifang Medical University, Weifang 261042, Shandong, P. R. China

***Correspondence:**

Ang Liu, E-mail: liuang08@163.com

**Key words:**

*Parasulfitobacter* gen. nov.; *Parasulfitobacter algicola* comb. nov.; sulfur oxidation system; *Sulfitobacter*; DMSP degradation

**Supplementary Table S1** Genome accession numbers and G+C contents of the type strains used for conducting the phylogenomic analysis

| Organism | Strain | G+C content (mol%) | Accession number |
| --- | --- | --- | --- |
| *Sulfitobacter brevis* | DSM 1143 | 58.4 | FOMW00000000.1 |
| *Sulfitobacter delicatus* | DSM 16477 | 60.8 | FNBP00000000.1 |
| *Sulfitobacter dubius* | DSM 16472 | 60.2 | FOPG00000000.1 |
| *Sulfitobacter indolifex* | HEL-45 | 59.5 | ABID00000000.1 |
| *Sulfitobacter aestuariivivens* | TSTF-M16 | 59.3 | JACTAG000000000.1 |
| *Sulfitobacter geojensis* | MM-124 | 57.8 | JASE00000000.1 |
| *Sulfitobacter maritimus* | S0837 | 59.8 | JABVBB000000000.1 |
| *Sulfitobacter profundi* | SAORIC-263 | 61.2 | MVIQ00000000.1 |
| *Sulfitobacter mediterraneus* | DSM 12244 | 58.1 | QBKU00000000.1 |
| *Sulfitobacter noctilucae* | NB-68 | 58.3 | JASC00000000.1 |
| *Sulfitobacter noctilucicola* | NB-77 | 57.1 | JASD00000000.1 |
| *Sulfitobacter donghicola* | DSW-25 | 55.2 | JASF00000000.1 |
| *Sulfitobacter guttiformis* | KCTC 32187 | 56.1 | JASG00000000.1 |
| *Sulfitobacter undariae* | DSM 102234 | 54.7 | JACIEI000000000.1 |
| *Sulfitobacter litoralis* | DSM 17584 | 58.5 | FNJD00000000.1 |
| *Sulfitobacter marinus* | DSM 23422 | 56.9 | FPAJ00000000.1 |
| *Sulfitobacter pontiacus* | DSM 10014 | 60.3 | FNNB00000000.1 |
| *Sulfitobacter algicola* | 1151 | 51.8 | JABUFE000000000.1 |
| *Pseudosulfitobacter pseudonitzschiae* | DSM 26824 | 61.7 | FQVP00000000.1 |
| *Arenibacterium halophilum* | CAU 1492 | 63.1 | VCPC00000000.1 |
| *Brevirhabdus pacifica* | DSM 27767 | 66.6 | PGFI00000000.1 |
| *Litorivita pollutaquae* | FSX-11 | 58.7 | QFVT00000000.1 |
| *Loktanella salsilacus* | DSM 16199 | 60.0 | FOTF00000000.1 |
| *Pelagimonas varians* | DSM 23678 | 55.2 | QKMF00000000.1 |
| *Roseivivax halodurans* | JCM 10272 | 66.3 | JALZ00000000.1 |
| *Roseivivax lentus* | DSM 29430 | 66.1 | FTOQ00000000.1 |
| *Roseobacter cerasinus* | AI77 | 61.0 | BLIV00000000.1 |
| *Roseobacter denitrificans* | OCh 114 | 58.9 | CP000362.1, CP000464.1, CP000465.1, CP000466.1, CP000467.1 |
| *Roseobacter litoralis* | Och-149 | 57.2 | CP002623.1, CP002626.1, CP002625.1 CP002624.1 |
| *Roseobacter ponti* | DSM 106830 | 60.5 | CP048788.1, CP048789.1 |
| *Ruegeria atlantica* | CECT 4292 | 56.4 | CYPU00000000.1 |
| *Ruegeria meteori* | CECT 4293 | 56.5 | CYPS00000000.1 |
| *Yoonia maricola* | DSM 29128 | 56.2 | PGTY00000000.1 |
| *Yoonia rosea* | DSM 29591 | 57.7 | FTPR00000000.1 |
| *Yoonia vestfoldensis* | DSM 16212 | 61.8 | ARNL00000000.1 |
| *Hyphomonas polymorpha* | PS728 | 62.3 | ARYM00000000.1 |

**Supplementary Table S2** Genome accession numbers of the type species of the family *Roseobacteraceae*

| Organism | Strain | Accession number |
| --- | --- | --- |
| *Actibacterium mucosum* | KCTC 23349 | JFKE00000000.1 |
| *Aestuariivita boseongensis* | BS-B2 | JXYH00000000.1 |
| *Aestuarium zhoushanense* | G7 | CP021114.1, CP021115.1, CP021116.1, CP021117.1 |
| *Agaricicola taiwanensis* | CCM 7684 | BMCP00000000.1 |
| *Albibacillus kandeliae* | J95 | QEHM00000000.1 |
| *Aliiroseovarius pelagivivens* | CECT 8811 | OMOI00000000.1 |
| *Aliiruegeria lutimaris* | DSM 25294 | FNEK00000000.1 |
| *Aliishimia ponticola* | MYP11 | SRKY00000000.1 |
| *Allosediminivita pacifica* | DSM 29329 | QBKN00000000.1 |
| *Antarcticimicrobium sediminis* | S4J41 | SMFP00000000.1 |
| *Antarctobacter heliothermus* | DSM 11445 | FZON00000000.1 |
| *Aquicoccus porphyridii* | L1 8-17 | VINQ00000000.1 |
| *Aquimixticola soesokkakensis* | CECT 8620 | FWFS00000000.1 |
| *Arenibacterium halophilum* | CAU 1492 | VCPC00000000.1 |
| *Ascidiaceihabitans donghaensis* | CECT 8599 | OMOR00000000.1 |
| *Boseongicola aestuarii* | CECT 8489 | FXXQ00000000.1 |
| *Brevirhabdus pacifica* | DSM 27767 | PGFI00000000.1 |
| *Carideicomes alvinocaridis* | SCR17 | SRJI00000000.2 |
| *Celeribacter neptunius* | DSM 26471 | FORH00000000.1 |
| *Chachezhania antarctica* | SM1703 | QZEX00000000.1 |
| *Citreimonas salinaria* | DSM 26880 | FNPF00000000.1 |
| *Cognatishimia maritima* | DSM 28223 | FQWM00000000.1 |
| *Cognatiyoonia koreensis* | DSM 17925 | FOIZ00000000.1 |
| *Cribrihabitans marinus* | DSM 29340 | FNYD00000000.1 |
| *Dinoroseobacter shibae* | DSM 16493 | CP000830.1, CP000831.1, CP000832.1, CP000833.1, CP000834.1, CP000835.1 |
| *Donghicola eburneus* | DSM 29127 | FOXY00000000.1 |
| *Epibacterium ulvae* | U95 | PHJF00000000.1 |
| *Falsihalocynthiibacter arcticus* | PAMC 20958 | CP014327.1, CP014328.1 |
| *Falsiruegeria mediterranea* | CECT 7615 | ONZG00000000.1 |
| *Flavimaricola marinus* | CECT 8899 | FXZK00000000.1 |
| *Fluviibacterium aquatile* | SM1902 | SMZO00000000.1 |
| *Halocynthiibacter namhaensis* | RA2-3 | JWIF00000000.1 |
| *Hasllibacter halocynthiae* | DSM 29318 | PVTT00000000.1 |
| *Histidinibacterium lentulum* | B17 | RDRB00000000.1 |
| *Jannaschia helgolandensis* | DSM 14858 | FNZQ00000000.1 |
| *Jhaorihella thermophila* | DSM 23413 | FNVD00000000.1 |
| *Kandeliimicrobium roseum* | XY-R6 | PNOS00000000.1 |
| *Kangsaoukella pontilimi* | GH1-50 | WUPT00000000.1 |
| *Leisingera methylohalidivorans* | DSM 14336 | CP006773.1, CP006774.1, CP006775.1 |
| *Lentibacter algarum* | DSM 24677 | FNPR00000000.1 |
| *Limimaricola hongkongensis* | DSM 17492 | APGJ00000000.1 |
| *Litoreibacter albidus* | DSM 26922 | FNOI00000000.1 |
| *Litorivita pollutaquae* | FSX-11 | QFVT00000000.1 |
| *Loktanella salsilacus* | DSM 16199 | FOTF00000000.1 |
| *Lutimaribacter saemankumensis* | DSM 28010 | FNEB00000000.1 |
| *Maliponia aquimaris* | CECT 8898 | FXYF00000000.1 |
| *Mameliella alba* | DSM 26384 | QAOY00000000.1 |
| *Mangrovicoccus ximenensis* | T1lg56 | QBKZ00000000.1 |
| *Marinibacterium profundimaris* | 22II1-22F33 | AQQR00000000.1 |
| *Marinovum algicola* | FF3 | FNYY00000000.1 |
| *Maritimibacter alkaliphilus* | HTCC2654 | AAMT00000000.1 |
| *Marivita cryptomonadis* | CL-SK44 | JFKD00000000.1 |
| *Marivivens donghaensis* | KCTC 42776 | JALNPR000000000.1 |
| *Meinhardsimonia xiamenensis* | CGMCC 1.10789 | FNFV00000000.1 |
| *Meridianimarinicoccus roseus* | TG-679 | QGKU00000000.1 |
| *Monaibacterium marinum* | C7 | OCTN00000000.1 |
| *Neptunicoccus sediminis* | CY02 | MDVX00000000.1 |
| *Nereida ignava* | DSM 16309 | FORZ00000000.1 |
| *Nioella nitratireducens* | SSW136 | MNBW00000000.1 |
| *Oceanibium sediminis* | O448 | QGNX00000000.1 |
| *Oceanicola granulosus* | HTCC2516 | AAOT00000000.1 |
| *Oceaniglobus indicus* | 1-19b | NMZM00000000.1 |
| *Octadecabacter arcticus* | 238 | CP003742.1, CP003743.1, CP003744.1 |
| *Pacificibacter maritimus* | DSM 104731 | RKQK00000000.1 |
| *Pacificitalea manganoxidans* | DY25 | CP021404.1, CP021405.1, CP021406.1, CP021407.1, CP021408.1, CP021409.1 |
| *Paenimaribius caenipelagi* | JBTF-M29 | VFSV00000000.1 |
| *Palleronia marisminoris* | DSM 26347 | FOPF00000000.1 |
| *Parasedimentitalea marina* | W43 | CP033219.1, CP033220.1, CP033221.1, CP033222.1, CP033223.1 |
| *Pelagicola litoralis* | CL-ES2 | SULI00000000.1 |
| *Pelagimonas varians* | DSM 23678 | QKMF00000000.1 |
| *Pelagivirga sediminicola* | BH-SD19 | QCYH00000000.1 |
| *Pelagovum pacificum* | SM1903 | CP065915.1, CP065916.1, CP065917.1 |
| *Phaeobacter gallaeciensis* | DSM 26640 | CP006966.1, CP006973.1, CP006972.1, CP006970.1, CP006971.1, CP006969.1 |
| *Phycocomes zhengii* | LMIT002 | QDFI00000000.1 |
| *Planktomarina temperata* | RCA23 | CP003984.1 |
| *Planktotalea frisia* | DSM 23709 | QKZM00000000.1 |
| *Pontibaca methylaminivorans* | DSM 21219 | FTPS00000000.1 |
| *Pontivivens insulae* | CECT 8812 | OMKW00000000.1 |
| *Poseidonocella pacifica* | DSM 29316 | FOJU00000000.1 |
| *Primorskyibacter sedentarius* | DSM 104836 | SLZU00000000.1 |
| *Profundibacter amoris* | BAR1 | CP032125.1 |
| *Profundibacterium mesophilum* | KAUST100406-0324 | APKE00000000.1 |
| *Pseudaestuariivita atlantica* | 22II-S11-z3 | AQQZ00000000.1 |
| *Pseudodonghicola xiamenensis* | DSM 18339 | AUBS00000000.1 |
| *Pseudohalocynthiibacter aestuariivivens* | BS-W9 | JAGFNU000000000.1 |
| *Pseudooceanicola atlanticus* | 22II-s11g | AQQX00000000.1 |
| *Pseudooctadecabacter jejudonensis* | CECT 8397 | FWFT00000000.1 |
| *Pseudophaeobacter arcticus* | DSM 23566 | AXBF00000000.1 |
| *Pseudoponticoccus marisrubri* | SJ5A-1 | LPXO00000000.1 |
| *Pseudoprimorskyibacter insulae* | CECT 8871 | OMOJ00000000.1 |
| *Pseudopuniceibacterium sediminis* | CY03 | QWJJ00000000.1 |
| *Pseudoroseicyclus aestuarii* | CECT 9025 | QJTE00000000.1 |
| *Pseudoruegeria aquimaris* | CECT 7680 | FWFQ00000000.1 |
| *Pseudosulfitobacter pseudonitzschiae* | DSM 26824 | FQVP00000000.1 |
| *Pukyongiella litopenaei* | SH-1 | CP027665.1, CP043619.1, CP043620.1, CP043621.1, CP043622.1, CP043623.1 |
| *Puniceibacterium antarcticum* | SM1211 | AWWI00000000.1 |
| *Rhodophyticola porphyridii* | MA-7-27 | RCNT00000000.1 |
| *Rhodosalinus sediminis* | WDN1C137 | QOHR00000000.1 |
| *Rhodovulum sulfidophilum* | DSM 1374 | CP015418.1, CP015419.1, CP015420.1 |
| *Roseibacterium elongatum* | DSM 19469 | CP004372.1 |
| *Roseicyclus mahoneyensis* | DSM 16097 | QGGW00000000.1 |
| *Roseisalinus antarcticus* | CECT 7023 | FWFZ00000000.1 |
| *Roseivivax halodurans* | JCM 10272 | JALZ00000000.1 |
| *Roseobacter litoralis* | Och-149 | CP002623.1, CP002626.1, CP002625.1, CP002624.1 |
| *Roseovarius tolerans* | DSM 11457 | FOBO00000000.1 |
| *Rubellimicrobium thermophilum* | DSM 16684 | AOLV00000000.1 |
| *Rubricella aquisinus* | DSM 103377 | JACIJS000000000.1 |
| *Ruegeria atlantica* | CECT 4292 | CYPU00000000.1 |
| *Sagittula stellata* | E-37 | AAYA00000000.1 |
| *Salinihabitans flavidus* | DSM 27842 | FODS00000000.1 |
| *Salipiger mucosus* | DSM 16094 | APVH00000000.1 |
| *Sedimentitalea nanhaiensis* | DSM 24252 | AXBG00000000.1 |
| *Sediminimonas qiaohouensis* | DSM 21189 | AUIJ00000000.1 |
| *Shimia marina* | DSM 26895 | FOMU00000000.1 |
| *Silicimonas algicola* | KC90 | CP034588.1 |
| *Sulfitobacter pontiacus* | DSM 10014 | FNNB00000000.1 |
| *Tateyamaria omphalii* | KCTC 12333 | BMWT00000000.1 |
| *Thalassobius mediterraneus* | DSM 16398 | FTNX00000000.1 |
| *Thalassococcus halodurans* | DSM 26915 | FNUZ00000000.1 |
| *Thiosulfatihalobacter marinus* | GL-11-2 | JAECRZ000000000.1 |
| *Tranquillimonas alkanivorans* | DSM 19547 | FOXA00000000.1 |
| *Tritonibacter horizontis* | O3.65 | LPUY00000000.1 |
| *Tropicibacter naphthalenivorans* | DSM 19561 | FWXX00000000.1 |
| *Tropicimonas isoalkanivorans* | DSM 19548 | FOLG00000000.1 |
| *Vannielia-litorea* | DSM 29440 | FSRL00000000.1 |
| *Wenxinia marina* | DSM 24838 | AONG00000000.1 |
| *Yangia pacifica* | DSM 26894 | FOZW00000000.1 |
| *Yoonia vestfoldensis* | DSM 16212 | ARNL00000000.1 |
| *Youngimonas vesicularis* | CC-AMW-E | SSMD00000000.1 |

**Supplementary Table S3** Locus_tags of the genes of sox cluster of *Sulfitobacter algicola* 1151^T^ and the selected 17 type strains of *Sulfitobacter*

S Strains: 1, *S. dubius* DSM 16472^T^; 2, *S. profundi* SAORIC-263^T^; 3, *S. indolifex* HEL-45^T^; 4, *S. delicatus* DSM 16477^T^; 5, *S. maritimus* S0837^T^; 6, *S. brevis* DSM 1143^T^; 7, *S. noctilucicola* NB-77^T^; 8, *S. noctilucae* NB-68^T^; 9, *S. geojensis* MM-124^T^; 10, *S. mediterraneus* DSM 12244^T^; 11, *S. aestuariivivens* TSTF-M16^T^; 12, *S. litoralis* DSM 17584^T^; 13, *S. pontiacus* DSM 10014^T^; 14, *S. marinus* DSM 23422^T^; 15, *S. undariae* DSM 102234^T^; 16, *S. guttiformis* KCTC 32187^T^; 17, *S. donghicola* DSW-25^T^; 18, *S. algicola* 1151^T^

| Strain | *soxT* | *orf1* | *soxR* | *soxS* | *soxV* | *soxW* | *soxX* | *soxY* | *soxZ* | *soxA* | *soxB* | *orf2* | | *soxC* | *soxD* | *soxE* | *soxF* | *orf3* | *orf4* | *orf5* | *soxG* | *soxH* |
| --- | --- | --- | --- | --- | --- | --- | --- | --- | --- | --- | --- | --- | --- | --- | --- | --- | --- | --- | --- | --- | --- | --- |
| 1 | BM229_RS01380 |  | BM229_RS01385 | BM229_RS01390 | BM229_RS01395 | BM229_RS01400 | BM229_RS01405 | BM229_RS01410 | BM229_RS01415 | BM229_RS01420 | BM229_RS01425 |  | BM229_RS01430 | | BM229_RS01435 | - | - |  |  |  | BM229_RS03470 | BM229_RS03475 |
| 2 | B1030_RS02990 |  | B1030_RS02985 | B1030_RS02980 | B1030_RS02975 | B1030_RS02970 | B1030_RS02965 | B1030_RS02960 | B1030_RS02955 | B1030_RS02950 | B1030_RS02945 |  | B1030_RS02940 | | B1030_RS02935 | - | - |  |  |  | B1030_RS01060 | - |
| 3 | OIHEL45_RS12760 |  | OIHEL45_RS12755 | OIHEL45_RS12750 | OIHEL45_RS12745 | OIHEL45_RS12740 | OIHEL45_RS12735 | OIHEL45_RS12730 | OIHEL45_RS12725 | OIHEL45_RS12720 | OIHEL45_RS12715 |  | OIHEL45_RS12710 | | OIHEL45_RS12705 | - | - |  |  |  | OIHEL45_RS10740 | OIHEL45_RS10735 |
| 4 | BLS57_RS07880 |  | BLS57_RS07885 | BLS57_RS07890 | BLS57_RS07895 | BLS57_RS07900 | BLS57_RS07905 | BLS57_RS07910 | BLS57_RS07915 | BLS57_RS07920 | BLS57_RS07925 |  | BLS57_RS07930 | | BLS57_RS07935 | - | - |  |  |  | BLS57_RS09780 | BLS57_RS09785 |
| 5 | HTT03_RS04835 |  | HTT03_RS04830 | HTT03_RS04825 | HTT03_RS04820 | HTT03_RS04815 | HTT03_RS04810 | HTT03_RS04805 | HTT03_RS04800 | HTT03_RS04795 | HTT03_RS04790 |  | HTT03_RS04785 | | HTT03_RS04780 |  |  |  |  |  | HTT03_RS03015 | HTT03_RS03020 |
| 6 | BM174_RS09865 | BM174_RS09875 | BM174_RS09870 | BM174_RS09880 | BM174_RS09885 | BM174_RS09890 | BM174_RS09895 | BM174_RS09900 | BM174_RS09905 | BM174_RS09910 | BM174_RS09915 |  | BM174_RS09920 | | BM174_RS09925 | - | - |  |  |  | - | BM174_RS12100 |
| 7 | Z946_RS0110910 |  | Z946_RS0110915 | Z946_RS0110920 | Z946_RS0110925 | Z946_RS0110930 | Z946_RS0110935 | Z946_RS0110940 | Z946_RS0110945 | Z946_RS0110950 | Z946_RS0110955 | Z946_RS0110960 | Z946_RS0110965 | | Z946_RS0110970 | Z946_RS0110975 | Z946_RS0110980 | Z946_RS0110985 | Z946_RS0110990 | Z946_RS0110995 | Z946_RS0111005 | Z946_RS0111000 |
| 8 | Z945_RS0115250 |  | Z945_RS0115255 | Z945_RS0115260 | Z945_RS0115265 | Z945_RS0115270 | Z945_RS0115275 | Z945_RS0115280 | Z945_RS0115285 | Z945_RS0115290 | Z945_RS0115295 |  | Z945_RS0115300 | | Z945_RS0115305 | Z945_RS0115310 | Z945_RS0115315 | Z945_RS0115320 | Z945_RS0115325 | Z945_RS0115330 | Z945_RS0115340 | Z945_RS0115335 |
| 9 | Z947_RS0107010 |  | Z947_RS0107005 | Z947_RS0107000 | Z947_RS0106995 | Z947_RS0106990 | Z947_RS0106985 | Z947_RS0106980 | Z947_RS0106975 | Z947_RS0106970 | Z947_RS0106965 |  | Z947_RS0106960 | | Z947_RS0106955 | Z947_RS0106950 | Z947_RS0106945 | Z947_RS0106940 | - | Z947_RS0106935 | Z947_RS0106925 | Z947_RS0106930 |
| 10 | C8N31_RS11355 |  | C8N31_RS11350 | C8N31_RS11345 | C8N31_RS11340 | C8N31_RS11335 | C8N31_RS11330 | C8N31_RS11325 | C8N31_RS11320 | C8N31_RS11315 | C8N31_RS11310 |  | C8N31_RS11305 | | C8N31_RS11300 | C8N31_RS11295 | C8N31_RS11290 | C8N31_RS11285 | - | C8N31_RS11280 | C8N31_RS11270 | C8N31_RS11275 |
| 11 | H9Q16_RS19595 |  | H9Q16_RS19600 | H9Q16_RS19605 | H9Q16_RS19610 | H9Q16_RS19615 | H9Q16_RS19620 | H9Q16_RS19625 | H9Q16_RS19630 | H9Q16_RS19635 | H9Q16_RS19640 |  | H9Q16_RS19645 | | H9Q16_RS19650 | H9Q16_RS19655 | H9Q16_RS19660 | H9Q16_RS19665 | H9Q16_RS19670 | H9Q16_RS19675 | H9Q16_RS19685 | H9Q16_RS19680 |
| 12 | - |  | BLQ98_RS11465 | BLQ98_RS11470 | BLQ98_RS11475 | BLQ98_RS11480 | BLQ98_RS11485 | BLQ98_RS11490 | BLQ98_RS11495 | BLQ98_RS11500 | BLQ98_RS11505 | - | BLQ98_RS11510 | | BLQ98_RS11515 | - | - | BLQ98_RS11520 |  |  | BLQ98_RS11525 | - |
| 13 | - |  | BLW19_RS07110 | BLW19_RS07115 | BLW19_RS07120 | BLW19_RS07125 | BLW19_RS07130 | BLW19_RS07135 | BLW19_RS07140 | BLW19_RS07145 | BLW19_RS07150 |  | BLW19_RS07155 | | BLW19_RS07160 | - | - | BLW19_RS07165 |  |  | - | - |
| 14 | BM347_RS13655 |  | BM347_RS13660 | BM347_RS13665 | BM347_RS13670 | BM347_RS13675 | BM347_RS13680 | BM347_RS13685 | BM347_RS13690 | BM347_RS13695 | BM347_RS13700 |  | BM347_RS13705 | | BM347_RS13710 | - | - | BM347_RS13715 |  |  | BM347_RS13725 | BM347_RS13720 |
| 15 | GGR95_RS07180 |  | GGR95_RS07185 | GGR95_RS07190 | GGR95_RS07195 | GGR95_RS07200 | GGR95_RS07205 | GGR95_RS07210 | GGR95_RS07215 | GGR95_RS07220 | GGR95_RS07225 | GGR95_RS07230 | GGR95_RS07235 | | GGR95_RS07240 | - | - | GGR95_RS07245 | GGR95_RS07250 | - | GGR95_RS07260 | GGR95_RS07255 |
| 16 | - |  | - | - | - | - | - | - | - | - | - | - | - | | - | - | - |  |  |  | - | - |
| 17 | Z948_RS0114995 |  | Z948_RS0115000 | Z948_RS0115005 | Z948_RS0115010 | Z948_RS0115015 | Z948_RS0115020 | Z948_RS0115025 | Z948_RS0115030 | Z948_RS0115035 | Z948_RS0115040 |  | Z948_RS0115045 | | Z948_RS0115050 | Z948_RS0115055 | Z948_RS0115060 | Z948_RS0115065 | Z948_RS0115070 | Z948_RS0115075 | Z948_RS0115085 | Z948_RS0115080 |
| 18 | - |  | - | - | - | - | - | - | - | - | - | - | - | | - | - | - |  |  |  | - | - |

**Supplementary Table S4** Locus_tags of *SoeABC* genes of *Sulfitobacter algicola* 1151^T^ and the selected 17 type strains of *Sulfitobacter*.

Strains: 1, *S. dubius* DSM 16472^T^; 2, *S. profundi* SAORIC-263^T^; 3, *S. indolifex* HEL-45^T^; 4, *S. delicatus* DSM 16477^T^; 5, *S. maritimus* S0837^T^; 6, *S. brevis* DSM 1143^T^; 7, *S. noctilucicola* NB-77^T^; 8, *S. noctilucae* NB-68^T^; 9, *S. geojensis* MM-124^T^; 10, *S. mediterraneus* DSM 12244^T^; 11, *S. aestuariivivens* TSTF-M16^T^; 12, *S. litoralis* DSM 17584^T^; 13, *S. pontiacus* DSM 10014^T^; 14, *S. marinus* DSM 23422^T^; 15, *S. undariae* DSM 102234^T^; 16, *S. guttiformis* KCTC 32187^T^; 17, *S. donghicola* DSW-25^T^; 18, *S. algicola* 1151^T^

| Strain | *SoeA* | *SoeB* | *SoeC* |
| --- | --- | --- | --- |
| 1 | BM229_RS09455 | BM229_RS09460 | BM229_RS09465 |
| 2 | B1030_RS04725 | B1030_RS04730 | B1030_RS04735 |
| 3 | OIHEL45_RS03685 | OIHEL45_RS03690 | OIHEL45_RS03695 |
| 4 | BLS57_RS01220 | BLS57_RS01225 | BLS57_RS01230 |
| 5 | HTT03_RS15530 | HTT03_RS15535 | HTT03_RS15540 |
| 6 | BM174_RS05615 | BM174_RS05620 | BM174_RS05625 |
| 7 | Z946_RS0106560 | Z946_RS0106565 | Z946_RS0106570 |
| 8 | Z945_RS0107605 | Z945_RS0107610 | Z945_RS0107615 |
| 9 | Z947_RS0120085 | Z947_RS0120095 | Z947_RS0120100 |
| 10 | C8N31_RS01120 | C8N31_RS01125 | C8N31_RS01130 |
| 11 | H9Q16_RS12805 | H9Q16_RS12810 | H9Q16_RS12815 |
| 12 | - | - | - |
| 13 | - | - | - |
| 14 | BM347_RS14060 | BM347_RS14065 | BM347_RS14070 |
| 15 | GGR95_RS00665 | GGR95_RS00660 | GGR95_RS00655 |
| 16 | Z949_RS0115570 | Z949_RS0115575 | Z949_RS0115580 |
| 17 | Z948_RS0109235 | Z948_RS0109230 | Z948_RS0109225 |
| 18 | HRQ87_RS04735 | HRQ87_RS04740 | HRQ87_RS04745 |

**Supplementary Table S5** List of protein_ids of DMSP related enzymes in *Sulfitobacter algicola* 1151^T^ and the selected 17 type strains of *Sulfitobacter*.

Strains: 1, *S. dubius* DSM 16472^T^; 2, *S. profundi* SAORIC-263^T^; 3, *S. indolifex* HEL-45^T^; 4, *S. delicatus* DSM 16477^T^; 5, *S. maritimus* S0837^T^; 6, *S. brevis* DSM 1143^T^; 7, *S. noctilucicola* NB-77^T^; 8, *S. noctilucae* NB-68^T^; 9, *S. geojensis* MM-124^T^; 10, *S. mediterraneus* DSM 12244^T^; 11, *S. aestuariivivens* TSTF-M16^T^; 12, *S. litoralis* DSM 17584^T^; 13, *S. pontiacus* DSM 10014^T^; 14, *S. marinus* DSM 23422^T^; 15, *S. undariae* DSM 102234^T^; 16, *S. guttiformis* KCTC 32187^T^; 17, *S. donghicola* DSW-25^T^; 18, *S. algicola* 1151^T^. DddD, DddQ, DddW, DddY were not identified in any of the strains

| Strain | DmdA | DmdB | DmdC | DmdD | DddL | DddP |  | AcuK | DddA | DddC |
| --- | --- | --- | --- | --- | --- | --- | --- | --- | --- | --- |
| 1 | WP_093926163.1 | WP_093926328.1 | WP_093926949.1 | - | - | - |  | WP_093927533.1 | WP_093927492.1 | WP_093925996.1 |
| 2 | WP_132443413.1 | WP_132443335.1 | WP_132444318.1 | - | - | - |  | WP_067622966.1 | WP_132443895.1 | WP_132443534.1 |
| 3 | WP_007119686.1 | WP_007119563.1 | WP_007119073.1 | - | - | - |  | WP_007118329.1 | WP_040700324.1 | WP_007119922.1 |
| 4 | WP_093741561.1 | WP_093742013.1 | WP_093740169.1 | - | - | - |  | WP_093738654.1 | WP_093738558.1 | WP_093741116.1 |
| 5 | WP_174858431.1 | WP_174858311.1 | WP_174859853.1 | - | - | - |  | WP_174860849.1 | WP_174860803.1 | WP_174858638.1 |
| 6 | WP_093922302.1 | WP_093922457.1 | WP_093923199.1 | - | - | WP_093924908.1 |  | WP_093924481.1 | WP_093922287.1 | WP_093923771.1 |
| 7 | WP_025057289.1 | WP_025057411.1 | WP_025054439.1 | WP_193789251.1 | WP_025056738.1 | WP_025056532.1 |  | WP_025055403.1 | WP_025056562.1 | WP_025054026.1 |
| 8 | WP_025052029.1 | WP_025052937.1 | WP_025050748.1 | WP_025053001.1 | WP_025053558.1 | WP_025052576.1 |  | WP_025051932.1 | WP_025052030.1 | WP_025052721.1 |
| 9 | WP_025044868.1 | WP_025044711.1 | WP_025045691.1 | - | - | WP_025044182.1 |  | WP_025043050.1 | WP_025042324.1 | WP_025044498.1 |
| 10 | WP_025049182.1 | WP_025046243.1 | WP_025049774.1 | WP_025046848.1 | - | WP_025048733.1 |  | WP_025049248.1 | WP_025046635.1 | WP_025048302.1 |
| 11 | WP_191074170.1 | WP_191074280.1 | WP_191075319.1 | - | - | WP_191075532.1 |  | WP_191076080.1 | WP_191075931.1 | WP_191073965.1 |
| 12 | - | WP_093732352.1 | WP_093733594.1 | WP_093733011.1 | WP_093733284.1 | - |  | WP_093734393.1 | WP_093732962.1 | WP_093733053.1 |
| 13 | - | WP_037944362.1 | WP_074634798.1 | WP_009826810.1 | WP_074635571.1 | - |  | WP_005850125.1 | - | WP_074635076.1 |
| 14 | WP_093915133.1 | WP_093915164.1 | WP_093915603.1 | WP_093916960.1 | - | - |  | WP_093916350.1 | WP_093917712.1 | WP_093914796.1 |
| 15 | - | WP_184567784.1 | WP_184567784.1 | WP_184563288.1 | WP_184567256.1 | - |  | WP_184565021.1 | WP_184568193.1 | WP_184565883.1 |
| 16 | WP_025062221.1 | WP_025061757.1 | WP_025063223.1 | WP_025064410.1 | WP_025061449.1 | - |  | WP_025064018.1 | WP_025063816.1 | WP_025061880.1 |
| 17 | - | WP_025057894.1 | WP_025059776.1 | WP_025058442.1 | WP_025058113.1 | WP_025057920.1 |  | WP_025058977.1 | WP_025059162.1 | WP_025057798.1 |
| 18 | - | - | WP_174139036.1 | WP_174135728.1 | - | - |  | WP_174139723.1 | WP_174135967.1 | WP_174139457.1 |





**Fig. S1** Maximum-likelihood phylogenetic tree based on 16S rRNA gene sequences of all the type strains of validated species of the genus *Sulfitobacter* and members of closely related taxa. *H. polymorpha* PS728^T^ is used as an outgroup. *S. algicola* 1151^T^ is shown in bold. Bootstrap percentages (>70%) based on 1000 replicates are shown at nodes. Bar, 0.02 substitutions per nucleotide position





**Fig. S2** Maximum-likelihood phylogenetic tree based on the nucleotide sequences of UBCG of the type strains of validated species of the genus *Sulfitobacter* and members of closely related taxa whose genome sequences were available. *H. polymorpha* PS728^T^ is used as an outgroup. *S. algicola* 1151^T^ is shown in bold. Bootstrap percentages (>70%) based on 100 replicates are shown at nodes. Bar, 0.1 substitutions per nucleotide position


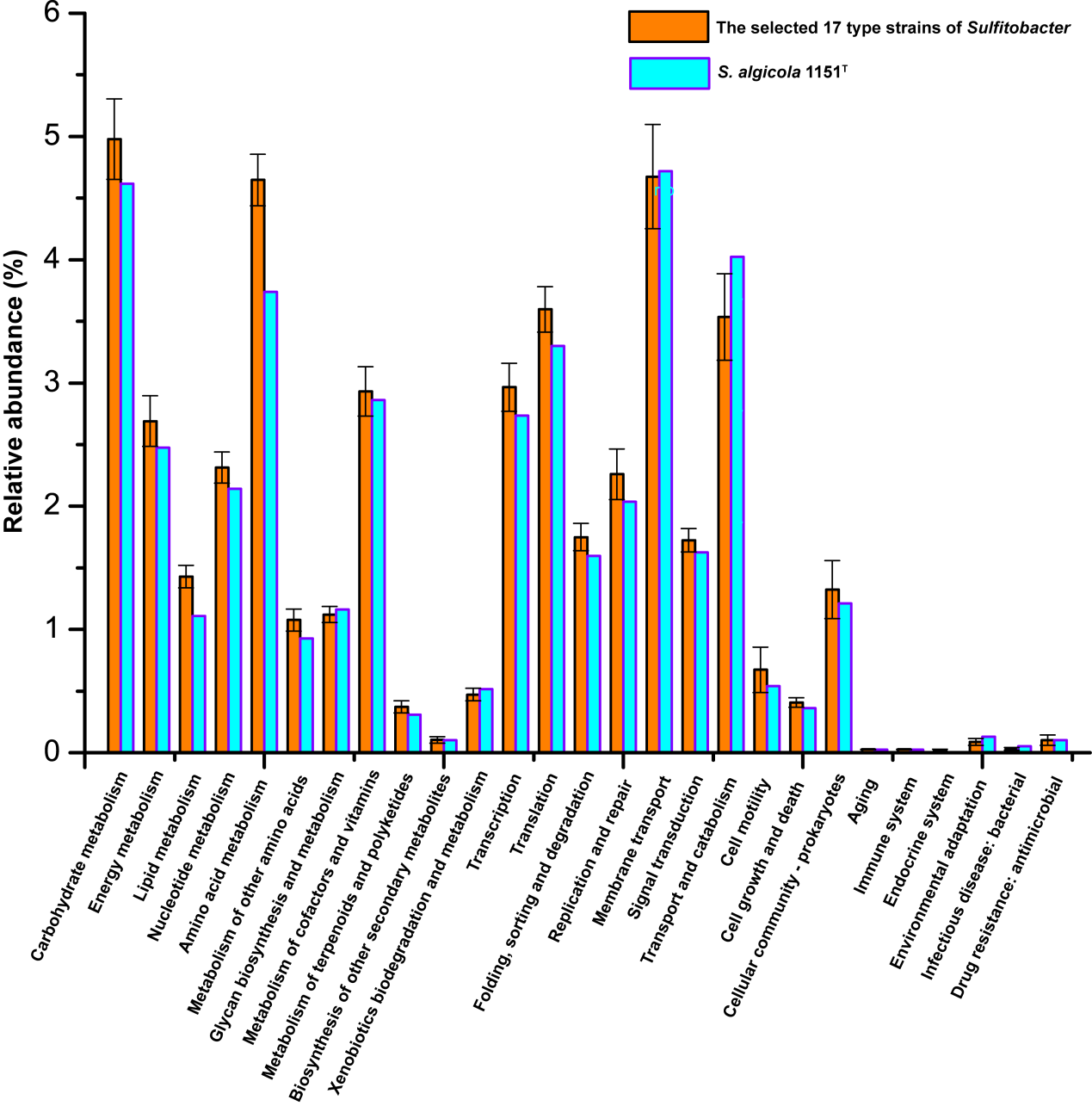


**Fig. S3** Relative abundances of KEGG functional genes identified in genomes of *S. algicola* 1151^T^ and the selected 17 type strains of *Sulfitobacter* at the secondary level. The relative abundances are expressed as the percentage of genes assigned to their respective KEGG categories relative to the total genes within each genome. The data are indicated as mean values of relative abundances in the selected 17 type strains of *Sulfitobacter*, with error bars representing standard deviations.


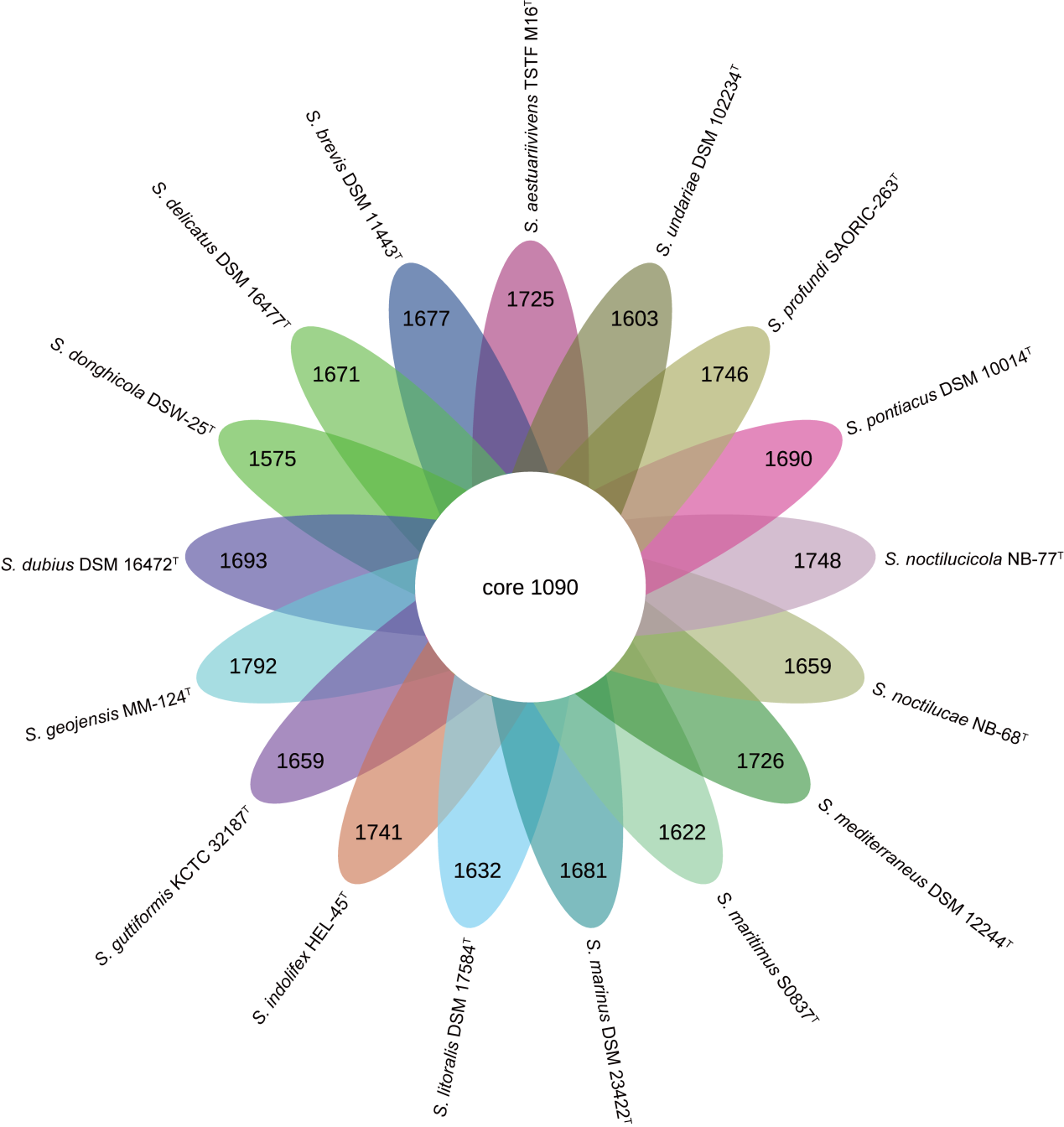


**Fig. S4** Venn diagram displaying the common and unique genes numbers among the selected 17 type strains of *Sulfitobacter*.


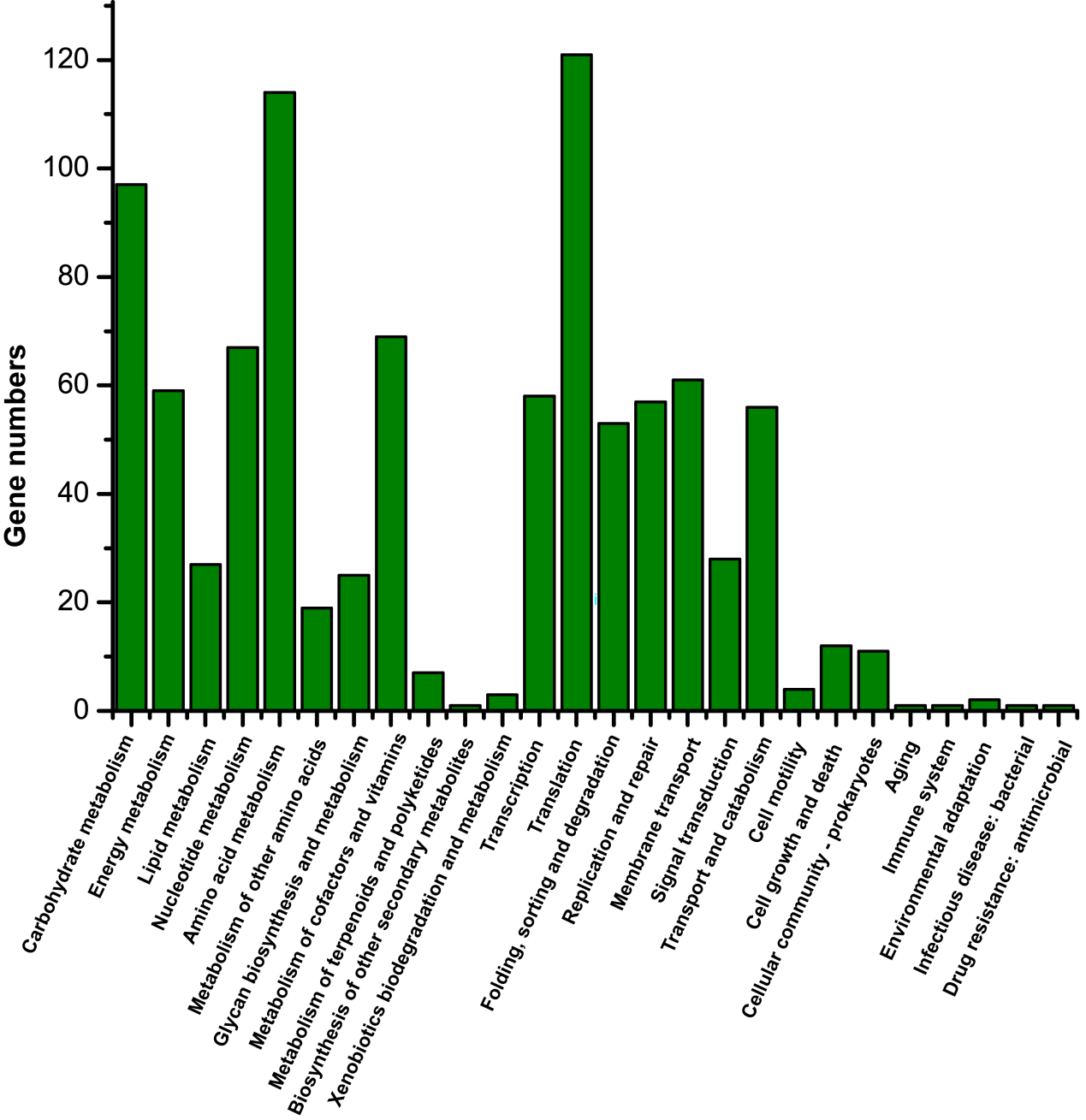


**Fig. S5** Numbers of KEGG functional genes identified in the core genome of the selected 17 type strains of *Sulfitobacter* at the secondary level.





**Fig. S6** DMSP degradation pathways. The enzymes identified in *Sulfitobacter* species are shown in black, and the DddD enzyme that was not identified in *Sulfitobacter* species is shown in gray. MMPA = 3-methylmercaptopropionate; MTA-CoA = methylthioacrylyl-CoA; 3-HP = 3-hydroxypropionic acid; MalSA = malonate semialdehyde; THF = tetrahydrofolat
