# Supplementary material for: Genome-based taxonomic classification of the genus Sulfitobacter along with the proposal of a new genus Parasulfitobacter gen. nov. and exploring the gene clusters associated with sulfur oxidation
Source: BMC Genomics. 2024 Apr 22;25:389. doi: 10.1186/s12864-024-10269-3 (PMC11034169; doi:10.1186/s12864-024-10269-3)
Supplement: Supplementary file 2 — Supplementary Material 2 [file 12864_2024_10269_MOESM2_ESM.docx]

>'Brevirhabdus-pacificaDSM-27767'

ATGAACGCCAAGGAACTCAACGACAAGACCCCCGACCAGCTGAAGGATCAGCTGGCCGAGCTGAAGAAGGAAGCGTTCAACCTGCGCTTCCAGCAGGCTACGCAACAGCTTGAGAACACCGCACGCATGCGCACCGTCCGTCGCGACGTTGCCCGTGTGAAGACCGTTCTCAACCAGAAGGCCGCTGCTGCGGCCGCAGAAGAATAAATGGCCAGCGCAGATCTTCTCACCATGACAAGCAAGACTTCCCCCGACAAGCAGAAGGCCCTGGACAGCGCGCTCGCGCAGATCGAGCGGCAGTTCGGCAAAGGCTCGATCATGAAGCTGGGCGCCGACAACCCGGTGCAGGAGATCGAGGCCACTTCGACTGGCTCTCTGGGGCTGGATATCGCCCTGGGCATCGGCGGCATCCCCAAGGGGCGCATCGTCGAGATCTACGGCCCCGAAAGCTCGGGCAAGACCACGCTGACGCTGCATTGCGTGGCGGAAGAACAGAAAAAGGGCGGCGTCTGCGCCTTTGTCGATGCCGAGCATGCCCTTGATCCGCAATATGCCAAGAAGCTGGGCGTCGACCTGGACGAGTTGCTGATCTCGCAGCCCGACACCGGCGAGCAGGCGCTTGAGATCGTCGACACGCTGGTGCGTTCGGGGGCCGTCAACATGGTCGTCGTCGACAGCGTCGCGGCCCTGACGCCGAAGTCCGAGCTTGAGGGCGAGATGGGCGATTCCAGCGTCGGCGTCCACGCCCGCCTGATGAGCCAGGCGATGCGCAAGCTGACCGGGTCGATCAGCCGCTCCAAGTGTACCGTCATCTTCATCAACCAGATCCGCATGAAGATCGGCGTCATGTTCGGCAGCCCCGAGACGACGACGGGCGGCAACGCGCTGAAATTCTACAGTTCGGTTCGTCTGGACATCCGCCGCATCGGCGCCATCAAGGACCGCGACGAGGTGGTCGGCAACACCACCAAGGTCAAGGTCGTCAAGAACAAGGTGGCCCCGCCGTTCAAGCAGGTGGAATTCGACATCATGTATGGCGAAGGCATCTCGAAGATGGGCGAGCTTCTGGACCTGGGCGTCAAGGCCGGCGTGGTCGAGAAGTCGGGCAGCTGGTTCAGCTATGGCGATGAGCGCATCGGTCAGGGCCGCGAGAATGCCAAGGCCTTCCTGAAGGCCAACAACCGCATCGCCCTGTCGATCGAGGACAAGATCCGCGCCGCCCACGGGCTGGAATTCGACGAAGAGGCGGGCGAC------GACGTGGTCGAGGCCTGAATGTCCCGCCTGAACACCGCCGAGGTCGAACCCAAATGGCAAAGCGCCTGGGAAGAGGCCGGCGTCTTCACCGCGACCCGCGACGAGTCGAAGCCGAAATACTACGTGCTCGAGATGTTCCCCTATCCCTCGGGGCGGATCCACATGGGCCACGTGCGCAACTACACGATGGGCGACGTGATCGCGCGCTACAAGATCTCGACCGGGCACAACGTCCTGCATCCGATGGGCTGGGACGCCTTCGGCATGCCGGCCGAGAACGCCGCAATCGAACGCGGCGGCCATCCCGCCACCTGGACCTATGACAACATCGCGGACATGCGCGCCCAGATGAAGCCGCTGGGCCTCAGCATCGACTGGAGCCGCGAGTTCGCGACCTGCGACCCCGAGTATTACGGCCAGCAGCAGGCGATGTTCATCGACATGCTCGACAAGGGGCTGGTCTATCGCAAGAACGCGGTGGTCAACTGGGACCCGGTGGACATGACCGTGCTGGCCAACGAACAGGTGATCGACGGGCGGGGCTGGCGTTCGGACGCGCCGGTCGAACGGCGCGAGCTGACCCAGTGGTTCTTCCGCATCTCGGATTTCGCCGACGAGCTGCTCTCGGCGCTGGACGGTCTGGACAACTGGCCCGAGAAGGTCCGCACCATGCAGGCCAACTGGATCGGCCGGTCGCGCGGTCTGGAATTCGCGTTCCAGCTGACGGCCCCCACCAATGGCTTTGCCGAGCTGCCGGTCTACACCACCCGGCCCGACACCCTGCTGGGCGCCAGCTTCGTCGGTATCTCGCCCGACCATCCGCTGGCCAAGTCGCTGGAAGAGGGCAACCCCGAGCTTGCGGCCTTCAGCGCCGATTGCCGCCGCATGGGCACCTCTGAGGCCGATATGGAGAAGGCCGAGAAAAAGGGCTTTGATACAGGGCTGCGGGTGCGCCACCCGCTGAACCCCGCGTGGGAGCTGCCGGTTTGGGTCGCCAACTTCATCCTGATGGATTACGGCACCGGCGCCGTTTTCGCCTGCCCGGCCCACGATCAGCGGGATCTGGATTTCTGTCGCAAATACGACTTGCCGGTGACGGACACGTTCTTCGCCCTCGACAACCCCCGCCCCGTCGAGGACGAGGCCTTCGTGCCCCCCAAGACCGAGCCCGTGCGCTGGGTCGATCATTTTGCCGGGCTGGACGTCGCCACCGGCCAGGAGGCGATCGACGCCACCATCGACCTTGCCGAGAAACAGGGCTGGGGCAAGGGCGTCACCCAGTTCCGCCTGCGCGACTGGGGCCTGTCGCGGCAACGCTACTGGGGCTGCCCGATCCCGGTCGTCCACTGCGACGCCTGCGGCGTGGTGCCCGAGAAAAAGGAAAACCTGCCGATCGAACTGCCGCGCGACGTCAGCTTCGACAAGCCCGGCAACCCGCTGGACCGGCACCCGACATGGCGCGACTGTGCCTGCCCCGCCTGCGGCCAGCCCGCCAAGCGCGAAACCGACACGATGGACACGTTCGTCGACAGCTCGTGGTATTACGCGCGCTTCACCTCGCCCCGGGCCGCGACGCCCACCGATGCCGAGGACGCGGCCTACTGGATGAATGTCGACCAGTATATCGGCGGGATCGAACACGCGATCCTGCACCTGCTTTACTCGCGCTTCTTTGCCCGCGCGATGAACCAGACCGGCCACCTGCCCGAAAAGGCGATCGAGCCGTTTAACGCGCTTTTCACCCAAGGCATGGTGACCCACGAGATCTATGTCACCCAGGATGACAAGGGCCGCCCGGTCTACCATCTGCCCGAATCCATCACCGAGTCCGGCGCGCGCGAGTCGGGGGCCCCCGTGCAGGTGATCCCCTCGGCCAAGATGTCGAAATCCAAGAAGAACGTGGTCGACCCGGTCAACATCATCGAGGCTTACGGCGCCGATACGGCCCGTTGGTTCATCCTGTCCGACTCGCCCCCCGAGCGCGATGTCGAATGGACCGCCGCCGGGGCCGAGGCCGCCTTCCGTCACCTCAACCGGGTCTGGCGCATTGCCACCGACGCCGCCGAGGCATCG---GATGCCGCCGGGGATGCCGACCAGGCGCTTGACCGCGCCACCCACCGGGCCATCGCCGACGTGACCGAGGGTGTCGAGAACTTCGCCTTCAACGCGGCCGTCGCCAAGCTTTACGGGTTCACCAACACGCTGGCGAAATCCAAGGCCAGCGGGCAGGCGCGCCGCCGCGCTGTCGCGACGCTGGCCCAGCTGATGAGCCCGATGACCCCGCACCTGTCCGAGGAGATCTGGGCGCTGCTGGGCAACGTGGGGCTGGTGGTGCGGGCGCCCTGGCCCAAGGCCGACCCTGCCCTGCTGGTCGAGGACAGCGTCACCCTGCCCATCCAGATCAATGGCAAGCGCAAGTCCGAGATCACCGTGGCCGCGGACCTGCCCCGCGAAGAGGTTGAAAAGATCGCCCTTGCGGATAAAGCTGTCCAGAAGGCGCTGGCCGGTGGTCAGCCGCGCAAGCTGATCGTGGTTCCGGGCCGGATTGTGAATGTCGTCATCTGAATGGCTATTACTTCTGCAAACCAGCTGGAGCTGTTGCAGACCGCCGAGGCGGTCGCGCGCGAGAAGATGATCGACCCGTCGCTCGTGATCGAGGCGATGGAAGAAAGCCTGGCCCGGGCCGCCAAGAGCCGTTACGGCGCCGAGATGGACATCCGCGTCAGCATCGACCGCAAGACCGGACGTGCCACCTTCACCCGCGTCCGCACCGTTGTGGAAGATGACGCGGTCGAGAACTACCAGGCCGAGCTGACCGTCAAGGAAGCCAAGCAGTATAAAGAGGACCCCCAGATCGGCGACGAGATCGTCGATGAGGTTCCCCCCGTCGAGATGGGCCGCATCGCCGCCCAGTCCGCCAAGCAGGTGATCCTGCAGAAGGTCCGCGAAGCCGAGCGTGACCGCCAGTACGAGGAATTCAAGGACCGCGCCGGCACCATCATCAACGGTGTCGTCAAGCGCGAGGAATACGGCAACGTCATCGTCGACATCGGCCGTGGCGAGGCTGTCCTGCGCCGCAACGAGAAGATCGGCCGCGAAAGCTACCGGCCCAACGACCGCATCCGCGTCTACATCAAGGACGTGCGCCGCGAACCCCGTGGCCCGCAGATCTTCCTCAGCCGCACCGCGCCCGAGTTCATGGCCGAGCTGTTCAAGATGGAAGTGCCCGAGATCTACGACGGCATCATCGAGATCAAGGCCGTCGCCCGCGATCCGGGCTCCCGCGCCAAGATCGCCGTCATCTCCTACGACAGCTCGATCGACCCCGTCGGCGCCTGCGTCGGCATGCGCGGCAGCCGGGTTCAGGCTGTCGTCAACGAGCTTCAGGGCGAGAAGATCGACATCATCCCGTGGAACGAGGATCAGCCGACCTTCCTTGTGAACGCGCTGCAGCCCGCCGAGGTCACCAAGGTGGTTCTGGATGAAGAGGCCGAGCGCATCGAGGTCGTCGTGCCCGACGAGCAGCTGTCGCTGGCCATCGGCCGTCGCGGTCAGAACGTGCGTCTGGCCAGCCAGCTGACCGGCCTCGACATCGACATCATGACCGAGGAAGAGGAAAGCAAGCGCCGCCAGGCCGAGTTTGCCGAGCGCACGCAGCTCTTCATGGACACGCTGGATCTGGACGAGTTCTTCGCCCAGCTTCTGGTCTCGGAAGGCTTCACCAACCTCGAGGAAGTCGCCTATGTCGACGCCGACGAGTTGCTGGTGATCGACGGAGTCGACAGCGACACCGCCGGCGAGCTTCAGGCCCGCGCCCGCGACCACCTGGAGGCGATCAACAAGAAGGCGCTGGAGCGCGCCGCCGAGCTTGGCGTCGACCAGAGCCTGGTTGATTTCGAGGGGCTGACCCCCCAGATGGTAGAAGCGCTGGCCGAGGACGGTATCCTGTCGCTCGAAGATTTCGCGACCTGCGCCGACTGGGAACTGGCCGGCGGCTGGACCACGGTCGAAGGCGAGCGGGTCAAGGATGACGGCCTGCTGGAGAAATTCGACGTTTCGCTGGAAGAGGCCCAGGACATGGTTATGACCGCCCGCGTTATGCTGGGCTGGGTCGATCCGACCGAGATGCTGGCCGAGGATACCGGCGAGGACGCCGAGGGCGAAGAGGGCCAGGCCGCCGAAGGCGACGCCTGAATGGCACATAAAAAAGCAGGCGGTTCATCCCGCAACGGCCGCGACTCTGCCGGCCGTCGCCTCGGCGTCAAGAAATTCGGTGGCGAAGCCGTCATCCCGGGCAACATCATCGTGCGTCAGCGCGGCACCAAGTGGTGGCCGGGCGAGGGCGTCGGCCTGGGCAAGGATCACACCATCTTCGCGACCGCCGAAGGTCACGTGACCTTCCACAAGGGTCTGAAGGGTCGCACCTTCATTTCGGTTATGCCAGCGGCCGAGGCCGCCGAGTAAATGGCGAAACGGTGGTATTCGGTAAGCGTCCTCTCGAACTTCGAGAAAAAGATCGCCGAGCAGATCCGGCATGACGTGGCCGCCGCCGGTCTTGAGGACGAGATCGAGGAAGTGCTGGTCCCCACCGAAGAGGTGATCGAGGTCCGGCGCGGCAAGAAGGTGACCGCCGAGCGGCGCTTCATGCCCGGCTATGTGCTGGTGCGGATGGAGATGTCGGATCGCGGCTATCACCTGATCAACTCGATCAACCGGGTGACCGGGTTCCTCGGTCCCCAGGGCAAGCCGATGCCCATGCGCGACGCCGAGGTGAACCAGATCCTCAACCGCGTCGAGGAAGGCCAGGAGGCCCCGCGTCAACTGATCTCCTTCGAGGTCGGCGAGAAGGTCAAGGTCAACGGCGGACCCTTCGAAGGGTTCGACGGTATGGTCGAGGAAGTGGACGACGACAACCAGCGCCTCAAGGTGTCGGTGTCGATCTTCGGCCGGGCGACCCCGGTCGAACTGGAATACACGGAAGTCTCGAAAGAGATCTGAATGAGTTTCACGCTCGCCATCGTGGGGCGCCCCAACGTGGGCAAGTCCACCCTGTTCAACCGCCTCGTGGGGCGGCGCCTTGCGCTGGTCGACGATCAGCCCGGCGTCACCCGCGACCTGCGCGAGGGCGAGGCCCGGCTTGGCGACCTGCGTTTCACCGTCGTCGACACCGCCGGCCTTGAAGAGGCCACGGACCAGAGCCTTCAGGGCCGCATGCGCCGGCTGACCGAGCGCGCGGTCGACATGGCCGACATCTGCCTGTTCATGATCGACGCGCGCGTGGGCATTACCCCGACCGACCAGGTCTTCGCCGAGATCCTGCGCAAGCGGTCGGCCCACGTGATCGTGGCCGCCAACAAAGCCGAGGGGCGGGCCGGCGAGGCCGGGATGCTGGAGGCCTACTCGCTGGGTCTTGGCGAGCCCCTGCGCCTGTCGGCCGAACATGGCGAGGGGATGGAGGACCTGTTGGCCGTCCTGACCCCGCTGGCCGACGAGTTCGCCCTTAAGGCCCAGGACAGCGCCCCCGAGACCGACGTGGAACTGGACGAGGAAGGCGGCGAGGAGGATCCCAGCCCCCGGCCCAGCCGCGCGAAACCGCTCCAGGTTGCCGTGGTTGGCCGGCCCAATGCCGGCAAATCGACCCTGATCAACGCCATCCTGGGCGAGGACCGCCTGTTGACCGGACCCGAGGCCGGGATCACCCGCGACGCCATCTCGGTCACGACCGAATGGGACGGC---GCCCCGGTGCGCATCTTCGACACCGCCGGCATGCGCAAGCGCGCCAAGGTGCAGGACAAGCTTGAGAAGCTGTCGGTCTCGGACGGGCTGCGCGCCGTGCGCTTCGCCGAGGTAGTCGTGGTGCTGCTGGACGTGGAGATCCCCTTCGAGCAGCAAGACCTGCGCATCGCCGACCTGGCCGAGCGCGAGGGCCGCGCCGTGGTGATCGCGGTCAACAAGTGGGATATCGAGACCGACCGCCAAAACAAGCTCAAGGAGCTGAAGGAAGAGTTCACGCGTCTGCTGCCCCAGCTTCGCGGCGCGCCGCTGATCACCGTCTCGGCCAAGACCGGGCGGGGACTCGACCGGTTGCAGGAAGCGATCCTGAAGGCACATGAGGTCTGGAACCGGCGGGTGACAACGGCGACGCTGAACCGCTGGCTGATCGGGATGCTGGAGGCGCACCCGCCCCCCGCGCCGGGCGGGCGGCGGATCAAGCTGCGCTACATGACCCAGGCCAAGACCCGGCCGCCGGGTTTCGTGGTCATGTGCTCGCACCCCGAGAAACTGCCGGAAAGCTATTCGCGGTATCTTGTCAACGGGTTGCGTGACAGCTTTGACATGCCGGGGACGCCGATCCGCCTGACCTTCCGCAGCCAGGCCGACAAGAACCCCTACAAGGACCGCAAGAAATCCACACCCTCGCGCCTGCGCAAGCACCTGGGCAAGGGCCGCGCAGACTAGATGCGGATCGTCTTCATGGGCACGCCCGATTTCTCGGTCCCCGTCCTGGACGCGCTGATCGCGGCCGGGCACGAGATCGCCGCCGTCTACTGCCAGCCGCCCCGCCCGGCGGGTCGGGGCAAGAAACCGCGCCCCACCCCCGTCCATGCCCGGGCCGAGGCGCTGGGCCTTGAGGTGCGCCACCCCGAAACCCTGCGCGACGCGGATGAACAGGCGCGCTTCGCGGCGCTTGAGGCTGACGTGGCGGTGGTCGTGGCCTATGGCCTGCTGCTGCCGCAGGCGGTGCTGGATGCGCCGCGTCACGGGTGCCTGAACATCCACGCCAGCCTTCTGCCGCGCTGGCGAGGGGCGGCGCCCATCCACCGGGCGATCATGGCGGGCGACGCGCGCACCGGCGTCTGCATCATGCAGATGGAAGCCGGGCTCGACACCGGGCCGGTGCTGTTGCGGGCCGAGATCCCGATCGGACCCACAGACACCACCGGCGATCTGCACGACGCGCTGTCGGCGCTGGGCGCGGCCCGGATCGTCGAGGCGCTGGAGCGGCTGGAGGGGCTGACCCCCGTGCCCCAGCCCGAAGAGGGCGTGACTTACGCGGCCAAGATCGACAAGGCCGAGGCGCGGATCGACTGGTCCCGTCCGGCCGCAGAGGTAGATCGCAAAATCAGGGGCCTGTCGCCCTTTCCCGGCGCCTGGACCGAGATGGCGGGCGAACGGGTGAAACTGCTGCGCTCGCGCGCGGAGGGCGGGGCAGGTGCCCCAGGCGAGGTTCTG---ACCGGCTTTACCATCGCCTGTGGCGAAGGTGCGGTCGAGGTGCTGGAAGCCCAGCGCCAGGGCAAGCGGGCGGCGGGCGCGGCCGAATTCCTGCGCGGCAACACCTTGCCT------GAGCGAATGGGCGAC---TGAATGAGCGACTCTACCGGCTACCAGGTTCTGGCCCGCAAATACCGGCCCGCCACATTCGCTGACCTGATCGGACAGGACGCGATGGTGCGCACCCTGCGCAACGCCTTTGCCGCCGACCGCATCGCCCAGGCCTTCATCCTGACCGGCATCCGGGGCACCGGCAAAACGACGACGGCGCGGATCATCGCCAAGGGCATGAACTGTGTCGGCACCGATGGCGAGGGCGGCCCCACGACCGATCCCTGCGGCACCTGCGAGCATTGCGTCGCCATCGCCGAGGGGCGCCACGTCGACGTGATGGAGATGGACGCCGCCAGCCGCACCGGCGTCGGCGACATCCGCGAGATCATCGACAGCGTGCATTACCGGGCCGCCAGCGCCCGCTACAAGATCTACATCATCGACGAGGTTCACATGCTGTCCAACAGCGCGTTCAACGCGCTGCTGAAGACGCTGGAAGAACCCCCCGCCCACGTGAAGTTCATCTTCGCCACCACCGAGATCCGCAAGGTGCCCGTCACCGTGCTGTCGCGCTGTCAGCGCTTCGACCTGCGCCGGATCGAGCCCGAGGTGATGATCGCCCATCTACAATCCATCGCCGGCAAGGAAAGCGCCCAGATCAACGACGACGCGCTGGCGCTGATCACCCGCGCCGCCGAGGGATCGGTGCGCGACGCCATGAGCCTTCTGGATCAGGCCATCAGCCACGGCGCGGGCGAGACCACCGCCGATCAGGTGCGCGCCATGCTGGGTCTGGCTGACCGGGGCCGGGTGCTGGACCTTTACGAGCTGGTGATGGCCGGCGACGCGGCGGGTGCGCTGAACGAGCTGTCGGCACAGTATTCCGACGGGGCCGACCCGCTGGCGGTGCTGCGCGATCTGGCCGAGATCACCCATTGGATCTCGGTCATCAAGATCACCCCCGAGGCGGCGGAGGATCCCACCGTCGGCCCCGACGAGCGCGCGCGCGGCCAAGGCCTGGCCCAGCGTCTGCCGATGCGGGCGATGACGCGCATGTGGCAGATGCTGCTGAAGGCGCTCGAGGAGGTCTCGGCCGCACCCAACGCCATGATGGCCGCCGAGATGGCGGTGATCCGGCTGACCCATGTCTCGACCCTGCCTTCGCCCGAGGAATTGATCCGCAGGCTTCAGGAC---ACGCCCGCCCCGCCCGCCGCGTTTGCCCCTGGCGGTGGCGCCGCGCCCGGCCCCTCGGCCGGCGGCCAAAGTGGGTCGCAGCAGGGCTCGGCCAGCGGCTCGGCCCCCGTTACGGCTGGCCCCGCAACCGCCGCCGCCCCCTCTCAGCAAACAGCCCTTGCCCGCTTTGCCACCTTCCAGTCAGTGGTCGAACTGATCCGTCAGAACCGGGACGTGAAACTTCTGGTCGAGGTCGAGACCACGCTGCGGCTGGTGTCCTACCAGCCCGGCCGCATCGAGTTTCAGCCCGATGACCGCGCCCCTCGCGATCTGGCCCAACGCCTTGGCCAGCGGCTTCAGGGCTGGACGGGCGCCCGCTGGGCCGTCAGCGTCACCGCCGAGGGCGGAGGCGAGACCATCGCCGAGGGCCGCGACGCCGAGCGCCTGGCGATGGAGCGAGAGGCCGCCAAGCACCCGCTGGTGCAGGCCGTGATGGACGCCTTTCCCGGGGCGAAGATTTCCAACGTAATCCCCTTCGAGACCATCCAGGCCGAGGCCGCGATCCAGGCCCTGCCCGAGGTGGACGACGAATGGGACCCCTTCGAAGAAACATAGATGCCCAAGATGAAGACCAAGTCGAGCGCCAAGAAGCGCTTCAAGATGACGGCCTCGGGCCGCGTCAAGGCAGGCCAGGCCGGCAAACGGCACGGCATGATCAAGCGGACGAACAAGTTCCTGCGGGACGCACGCGGCACCACGCTGCTGAGCAAGCCCGACGAGAACATCGTCAAAAAATACATGCCTTACGCGCGCTGAATGAACCTTTTTGCCGAGATGCGCGCCCTTGTGCTCGACAGTCTTTCCGATCTTCAGGATGCGGGCGGGCTGCCGCGCGACCTGGACATGGCGAATGTCGCGGTCGAGCCGCCGCGCGACGCCGCACACGGGGACATGGCCACCAACGCCGCGATGGTGCTGGCAAAGCCGGCCGGCATGAAACCCCGCGACATCGCCGACGCGCTGGCCGCGCGGCTGCTGGAGGATGGGCGCATCGCCTCGGCCGATGTGGCGGGTCCGGGTTTTTTGAACCTGCGGCTGTCGCCCGCCGTCTGGCAGGGTGTGGTCCGCGCGGCCCTGCGCGAGGGTGTCGATTTCGGGCGCTCGGACATGGGGCAGGGGCGCAAGATCAATGTCGAATACGTCTCGGCCAACCCGACCGGTCCGCTGCACGTGGGTCATACCCGTGGCGCGGTCTTCGGCGATGCGCTGTGCAGCCTGCTGGATTTCTCGGGCCATGACGTGACCCGGGAATACTATGTCAACGACGGCGGCGCCCAGGTCGACGTGCTCGCGCGCTCGGTCTACCTGCGCTACCTCGAGGCCCACGGTCAGGAAGTCGCCTTCGAGGACGGAACCTATCCCGGCGACTATCTGATCGAGGTCGGTCAGGCGCTGAAGGACAAGGTGGGTGACGCCTATGTCGGCAAGGGCGAGGACGCCTGGCTGGAAGAGGTGCGTGATTTCGCGACCGATGCGATGCTGGGCCTGATCCGCGAGGATCTGCATCTGCTGGGCGTGAAGATGGACAATTTCTTCTCGGAAAAATCCCTTTACGGCACCGGCCGGATCGAGGCCGCGCTGAACAGCCTCGAGACCAAGGGCCTGATCTACGAGGGCGTGCTGGAGCCGCCGAAGGGCAAGACCCCCGAGGATTGGGAGCCGCGCGAGCAGACGCTTTTCCGCTCGACCGCCCATGGCGACGACGTTGACCGCCCGGTGAAGAAATCCGACGGCAGCTGGACCTATTTCGCGCCCGACATCGCCTATCACTTCGACAAGATCGAGCGCGGCTATGACGAGTTGATCGACGTCTTCGGCGCCGATCACGGCGGTTACGTCAAGCGCATGAAGGCTGCCGTGTCGGCCCTGTCGGACGGGGCCGTGCCGCTGGACATCAAGCTGACCCAGCTGGTGCGCCTGTTCAAGAACGGCGAGCCTTTCAAGATGTCCAAGCGTGCGGGCACCTTCATCACCCTGCGCGACGTGGTCGAGCAGGTGGGCGCCGATGTCACCCGTTTCGTCATGCTGACGCGCAAGAACGACGCGCCGCTGGATTTCGACTTTGCCCGGGTGCTGGAGCAGTCCAAGGACAACCCGGTCTTCTACGTGCAATACGCCCATGCGCGGATCCGTTCGGTCATGCGCAAGGCCGAGGCCGCGGGCATCGCCTCGGACGCCGCGACGCTGGCCGGGGCGGATCTGGACCTTGCCTCGGACCCAGCCGAACTGGCGCTGGCGGCCAAGCTGGCCGAGTGGCCGCGCCTGATCGAGATCGCCGCCCGCACCCACGAGCCCCATCGCGTCGCCTTCTACCTCTACGAGCTGGCGTCCGAGCTGCACGGCCTTTGGAACCTGGGCAATGCCCGCCCCGAGCTGCGCTTTCTGCAGGAAGGTGACATTGCTGCAACGCAGGCGAAAATGGCGCTTATCCAGGCGACGGCCGTTGTTATTTCCGCCGGCCTTGGTATTCTTGGCGTGACCCCGGTGGAAGAGATGCGCTGAATGGCCGCCGACGATCCGCTTCCCCCGCTGCGCCAGGTGATCGCGGCCCACGGGCTGTCGGCGCGCAAGTCACTGGGACAGAACTTTCTGCTGGACCTGAACCTGACGGCCAAGATCGCCCGGCAGGCCGGCGATCTTGCAGGCTCGGACGTGCTGGAGGTGGGTCCCGGCCCGGGCGGTCTGACCCGGGGCCTGCTGGCCGAGGGGGCGCGCAAGGTGCTGGCGATCGAGAAGGACAGCCGCTGCCTGCCGGCGCTGGAAGAGATTTCGGCGGCCTACCCCGGTCGGTTGGAGGTGCTGAACGCCGACGCGCTGGAGATCGACCCCACCGCCCACCTGACCGCGCCCATCCGGGTGGTGGCCAACCTGCCCTACAACGTCGGCACCGAGCTGCTGGTGCGCTGGCTGACACCGCGCGACTGGCCCCCGTTCTGGAGCAGCCTGACCCTGATGTTCCAGAAAGAGGTGGCCGAGCGTATCGTCGCCCGGCCCGGATCGAAGGCCTACGGGCGTCTGGCCCTCCTGGCCCAATGGCGGGCCGATCCGGCCATCGTCATGCACCTGCCGCCACAGGCTTTCATCCCCGCGCCCAAGGTCCATTCCGCCGTGGTCCACCTGACCCGGCTGGACGCGCCCCGGTATCCCGCCGACGCGGCGATCCTGTCGCGCATCACCAAGGCCGCCTTTGGACAGCGCCGCAAGATGCTGCGCGCCAGCCTGAAGGGCACGGTGCCGGACGTGGAGAACGTCTTGAGGGACGTGGGCATCGACCCCACCGCCCGGGCCGAGACGCTGGACCTCGAAGCCTTCTGCGCCCTGGCCCGCCGGGTCGAGGAGCTGTGAATGGATATTATCGCACAGATCGAAGCCGAGCAGATCGAAGCTCTCGGCAAGACCTTCCCCGATTTCAAGGCCGGCGACACCGTTCGCGTCGGCTTCAAGGTGACCGAGGGCACCCGCACCCGCGTGCAGAACTATGAAGGCGTCTGCATTTCGCGCCGCAACGGTGCCGGCATCGCCGGGTCCTTCACCGTGCGCAAGATCTCGTTCGGCGAGGGCGTGGAGCGCGTGTTCCCCCTGCACTCGACCAACATCGACAGCATCGAGGTCGTCCGCCGCGGCAAGGTGCGTCGCGCCAAGCTCTACTATCTTCGCTCGCGTCGCGGCAAATCTGCCCGGATCGCGGAAAAGACCAACTACAAGCCGCTGTCGGGCGCAAAAGCGTAAATGGCAAACAGCAAAAGAACCCTGTTTCTGAAGCGCCGCCTGCGCGTTCGGAACAAGCTGCGGAAGATGAACGCCGGGCGTCCGCGCCTGTCGGTTCACCGCTCGAACAAGAACATCAGCGTGCAGCTGATCGACGATGTGAACGGCGTCACGCTCGCCTCGGCCTCCTCGCTGGAGAAGGACCTGGGCGTTCTGGGCAAGAACAACGTCGAAGCGGCGGCGAAGGTGGGCACCGCCATCGCCGAGCGCGCGAAAAAGGCCGGGGTCGAAGAATGCTACTTCGACCGTGGTGGTTTCCTGTTTCACGGGAAGGTCAAGGCACTTGCCGACGCCGCCCGTGAAAACGGCCTGAAGTTCTGAATGTTTGCGGTCCTCAAGACCGGTGGCAAGCAGTACAAGGTGCAGAGCGGCGACGTTCTGCGTGTTGAGAAGCTGGCCGCCCAAGCGGGTGACACCGTTCAGTTCAACGACATCATGATGCTGGGTGGCGACAAGCCCGTGGTTGGCGCTCCGCTGATCTCCGGTGCGGCCGTGCAGGCCGAGGTCATCGACCAGATCAAGGGCGAGAAGGTCATCCATTTCGTCAAGCGCCGCCGGAAGCATTCGTCCAAGCGCACCAAGGGCCACCGCCAGCAACTGACCCTGCTGCGCGTGACCGAGATCCTGGCCGAAGGTGGCGACAAGTCGGGCGTCAAGGCCGCTGTCGGTGCCGGTTCGGTTGCCGGTGTT---------GCCGCTGCTGCCGCC------GCGCCGAAGACCCCCGCCAAGAAGGCCGCCGCGCCCAAGGCTGAAGCACCCAAGGCCGAGGCCGCCGCGCCCAAGGCCGCAAAGGCCGACGACAAGGCTGACGATCTCAAGAAGCTGTCGGGCGTTGGTCCCGCGCTTGAGAAGAAGCTGCACGAAGCCGGTGTCACCACCTTCGCACAGATCGCCGGCTGGTCCGCCGCCGACATCGCCGAGATGGACGAGAAGCTGTCCTTCAAGGGCCGCATCGAGCGCGAGGGCTGGGTCGACCAGGCCAAAGAGCTTACCAAAGGCTAAATGTCCCGAGTCAAAGGTGGCACGACCACTCACGCCCGTCACAAGAAGGTCATCAAGGCAGCCAAAGGTTACTATGGCCGTCGCAAGAACGCCTTCCGCACCGCAACCCAGGCCGTCGACAAGGCGAACCAGTACGCGACACGCGATCGCAAGGCCCGCAAGCGCAATTTCCGCGCCCTGTGGATCCAGCGAATCAACGCCGCCGTCCGTCTGCATGACGACAGCCTGACCTACTCGCGCTTCATCAACGGTCTCGGCCTCGCCGGTATCGAAGTTGACCGCAAGGTTCTGGCCGACCTGGCCGTGCACGAGCCCGAAGCATTCGGCGCCATCGTCGACCAGGCGAAAGCCGCGCTGAAC---TGAATGCAGGTCACCGAGACCAAGAACGAAGGCCTGGAGCGGGCATATACCATCACTGTGACGGCCAAGGAGCTGGACGACAAGGTCGGCGAGAAGCTGGTCGAAGCGCAGCCCGAGGTCGAGCTGAAGGGCTTCCGCAAGGGCAAGGTGCCGATGGCGCTGCTCAAGAAGCAGTTCGGTCAGCGCCTGCTGGGCGAAGCCATGCAGGAATCGGTCGATGCGGCCATGGCCAAGCATTTCGAGGACAGCGGCGACCGCCCCGCGTTCCAGCCCAAGGTCGAGATGACCAACCAGGACTGGAAAGAGGGTGACGATGTCGTCGTCGACATGTCCTACGAGGCGCTGCCCGAGGTGCCCGACGCCGACTTCAGCAAGATCAAGCTGGAGAAGATGGTCGTGAAGCCCGAGGAATCCGCCGTCGACGACGCGCTGAAGAACCTGGCCGAGTCGGCCCAGAACTTCACCGACCGCGACGCCAAGGAGAAATCGCAGGACGGCGACCAGGTCGTGATCGACTTCCTGGGCAAGGTCGACGGCGAAGCCTTCGAGGGCGGCGCTGCCGAGGACTATCCGCTGGTGCTGGGCTCCAACTCGTTCATCCCCGGTTTCGAGGAGCAGCTGGTTGGCAAGAAAGCCGGTGAAGAGGTCGAGGTGAAGGTCTCGTTCCCCGGGGAATACGGTGCCGCGCACCTGGCCGGCAAGGACGCCGTGTTCGAGTGCAAGGTGAAGGCCGTGAAGGCCCCCGCCGCCGCCGAGATCGACGACGAGCTGGCCAAGAAATACGGCTCGGAAAGCCTTGACGCCCTCAAGGAGCAGATCCGCGAACGCCTGGCCGCCGAGTATAACGGTGCCGCCCGTGCCGTCATGAAGCGCAAGCTGCTGGACGAGCTGGACGAGCTGGTCAGCTTCGAGCTGCCGCCGACCCTGGTCGACGCCGAGGCCAAGCAGATCGCCCATCAGCTGTGGCACGAGGAAAACCCTGACGTCCAAGGCCACGATCACCCCGAGATCGAGACGACCGACGAGCACAAGAAGCTGGCCGAGCGCCGCGTCCGTCTTGGCCTGCTGCTGGCCGAGCTGGGCCGCAAGCAGGAGATCACCGTCAACGATCAGGAACTGACTCAGGCCATCATGAACCAGGCCCGGCAGTACCCCGGTCAGGAACGCCAGTTCTTCGAGTTCGCGCAGCAGAACGCCGAGTTCCGCCAGCAGATCCAGGCGCCGATCTTCGAAGACAAGGTCGTCGACTACATCTTCGAGTTGGCCAAGGTCGAGGAAAAGGAAGTCACCAAGGAAGAGCTTGAGAAAGCCGTTGAGGAGCTGGAGCAGGAGTGAATGAAACTTCACGAACTTCACGACAATCCCGGCGCAACCAAGAAGCGCAAGCGCGTTGGCCGCGGCCCGGGTTCCGGCACCGGCAAGACCGCCGGCCGTGGTATCAAGGGTCAGAAGTCCCGTTCGGGCGTCTCGATCAATGGCTACGAGGGTGGCCAAATGCCGCTCTACCAGCGGCTTCCCAAGCGTGGCTTCACCCCGCCGAACCGCAAGAAATTCGCCGTCCTGAACCTGAGCCTGCTTCAGAAGTTCCTGGACGACAAGAAGATCGACGGCTCGAAGGCCATCACCGAGGACGTCCTGGTCGAATCGGGCCTGGTGCGTCGCAAGCTCGACGGTGTCCGCATCCTCGCCAAGGGCGAGTTCAACGCCAAGGTGAACCTGGAAGTCACCGGCGCCTCCAAGTCGGCCATCGAGGCTGTCGAGAAGGCCGGCGGCTCGCTCAAGGTTTCGACCCCGGCGGCGACTTCCGAGCAAGCGTAAATGATCCAGATGCAGACCAATCTGGATGTCGCTGACAACTCCGGCGCTCGCCGGGTGCAGTGCATCAAGGTCCTGGGTGGTTCCAAGCGTAAATACGCCTCCGTCGGCGACATCATTGTCGTCTCGGTGAAGGAAGCCATCCCGCGCGGCCGCGTGAAGAAAGGTGACGTCCGCAAGGCCGTCGTCGTGCGCACCGCCAAGGAAGTCCGTCGCGAAGACGGCACCGCAATCCGGTTCGACCGGAACGCGGCCGTCATCCTCAACAACAACAACGAGCCCGTCGGCACCCGTATCTTCGGGCCGGTGGTTCGTGAGTTGCGCGCGAAGAACTTCATGAAGATCATCTCGCTGGCTCCGGAGGTTCTCTGAATGCGTCACGCACGAGGCTACCGCCGCCTGAACCGCACCCATGAACACCGCAAGGCGCTGTTCGCGAACATGGCCGGCTCGCTCATCGAACATGAGCAGATCAAGACCACCCTGCCCAAGGCGAAGGAACTTCGCCGCATCGTCGAAAAGCTCATCACTCTGGGCAAGCGCGGCGATCTGCACGCCCGCCGCCAGGCCGGTGCACAGCTGAAGCAGGACGAATATGTCGCCAAGCTGTTCGACGTGCTCGGCCCCCGCTACGCCGAGCGTCAGGGCGGCTATGTCCGCGTCCTGAAGGCCGGCTTCCGCTATGGTGACATGGCGCCGATGGCGATCATCGAATTCGTCGACCGCGATGTCGACGCCAAGGGCGCCGGCGACCGCGCCCGCCTTGAGGCCGAGGAAGCCGCCGAA---TAAATGCTTCAGCCAAAGCGCACAAAATTCCGCAAGCAGCACAAGGGCCGTATCCACGGCGAAGCCAAGGGTGGCTCCACCCTGAACTTCGGCACCTTCGGCCTCAAGGCCACGCAGCCCGAGCGGATCACTGCACGTCAGATCGAGGCGGCTCGCCGCGCGATGACGCGCCACATGAAACGTCAGGGCCGTGTCTGGATCCGTATCTTCCCCGATACGCCGGTCACCTCGAAGCCGACCGAAGTCCGTATGGGTAAAGGTAAGGGTTCGGTCGATTACTGGGCCGCCAAGGTCAAGCCCGGCCGGGTGATGTTCGAGATCGACGGTGTTTCCGAAGCCGTCGCGCGCGAGGCCCTGCGCCTGGCCGCGATGAAGCTGCCGATCAAGACCCGCACCGTGGTCCGCGAAGACTGGTGAATGAGCGACATCCGACTGACGAACACGGCGACACGCAGCAAGGAGCTGTTCCGCCCCATCGATCCGCAGAACGTGCGGATGTATGTCTGTGGGCCGACGGTCTATGACCGCGCCCACATGGGCAACGCCCGTCCCGCCATCGTTTTCGACGTGCTGTTCCGGCTGCTGCGCCATGTCTACGGGCCGGAGCATGTCCGCTACGTGCGCAACATCACCGACATCGACGACAAGATAAACGCCCGCGCCGCCGAGACGGGCCGCCCCATCCGCGAGATCACGGACGAGACGGCGGCCTGGTATCTTGAGGACATGGCCGCCCTGGGCAACCTGACGCCCACGGTCACCCCCCGCGCCACCGAATTTGTCGACGGCATGATCGCCATGATCGAGCAGCTCATCGCCTCGGGCCATGCGTATGAGGCCGAGGGGCACGTCCTCTTCTCGGTCGAAAGCTATGACCGATACGGCCGGCTGTCGGGCCGTTCGATCGATGACATGATCGCCGGGGCACGGGTCGAGGTGGCGCCCTACAAGCGCAATCCGATGGATTTCGTCCTGTGGAAACCCTCGGGCCCCGATCTGCCGGGCTGGGACAGCCCATGGGGCAGGGGGCGGCCCGGTTGGCACATCGAATGCTCGGCCATGAGCCGCGACCTGCTGGGCGAAAGCTTCGACATCCACGGCGGCGGCAACGACCTGATGTTTCCCCACCATGAGAACGAGATCGCCCAAAGCTGTTGTGCCAACCCGAATTCGGATTTCGCCCGGGTCTGGATGCATAACGAGATGCTTCAGGTCGAGGGCAAGAAGATGTCCAAGTCGCTGGGCAATTTCTTCACCGTGCGCGACCTGATCGACCAGGGCATCCCCGGCGAGGTGATCCGCTTCGTCTTCCTGTCGACCCACTACGGCAAGCCAATGGACTGGACCGAGCGCAAGGCCCAGGAGGCCGCGGCGACCCTGCGCAAGTGGCGCGCCCTGTGCCGGGGCGTGACGGCTGCACCGCAGCCCGACGCCGCCGTGCTGGCCTGCCTGGCCGATGATCTGAATACCGCCGGAGCCATCACCGAGATGCACCGCCTGGCAAGCGCCGGGCAGGCGGCCGAGCTGCTGGCTTCGGCCGCGATGCTGGGTCTGCTGGAGCCGGGAATGGGCGATTGGGCCGAAGCCCCCGCCGCCGATCTCAGCGCCTTTGCCGAGGCGCTGGCCGCGCGCCGGGCCGAGGCGATGGTCAGCAAGGATTTCTCGCAGGTCGATGCGCTGAAGCAGGCGCTTGTCGCCGCCGGCGTCGATGTCCGCATGAGCAAGACGGGCGTGGACCTTGTTCCCGGGCCCGGCTTCGATCCCGCCGCCATCGAGGGGCTGATCTGAATGGCCAAG------------CCGAAGTCCGATCCCAACTACAAGGTCGTCGCCGAGAACCGGCGCGCGCGCTACGATTACGCGATCGAGGAGGATCTCGAATGCGGGATCATGCTCGAGGGCTCCGAGGTGAAGGCCCTGCGCCAGAAATCGGCCAACATCGCCGAGAGCTATGCCGCCGTCGAGGACGGTGAGCTGTGGCTGATCAACTCCTACATCGCGCCCTACGAGCAGGCCCGCACCTTCGGCCACGACGAGCGGCGGCGCCGCAAGCTGCTGGTTTCCCGCAAGGAGCTGGCGCGCCTGTGGAACGCCACCAAGCGCGAGGGGATGACCCTGGTCCCCCTGGTGCTCTACTTCAACCATCGCGGCATGGCGAAGATCAAGATCGCCATCGCCAAGGGCAAGAAGAACCAGGACAAGCGCGCCAACGAGGCCAAGCGCGACTGGCAGCGTCAGAAGGCGCGGTTGTTGCGCCAGGGC---TAGATGAGCGCGAAGGCTGAACATTACGACGTGATCCGCAAGCCGATCATCACCGAAAAGGCAACCATGGCATCCGACGCGGGCGCTGTTGTCTTCGAGGTGGCGATGGACTCCAACAAGCCGCAGATCAAGGCGGCTGTCGAGGGTCTGTTCGGCGTCAAGGTGAAGGCGGTCAACACCACCATCACCAAGGGCAAGGTCAAGAAGTTCCGCGGCCAGCCTGGCCGCCGCAAGGACGTCAAGAAAGCGTATGTCACCCTCGAAGAGGGCAATACGATAGACGTCACTACGGGCCTCTGAATGGGTAAAGAGAAAAATCCCCGCCGCGTGGCCGACAACGAAGCACTCGCCAAGACGCGCATGCTTCGGACGTCCCCGCAGAAACTGAACCTCGTCGCCGCCATGATCCGCGGCAAGAAGGTGGACAAGGCCCTGGCCGACCTCACCTTCTCGAAGAAGCGGATCGCGGCCGACGTCAAGAAATGCCTTCAGTCGGCAATCGCCAACGCGGAAAACAACCATAACCTGGATGTTGACGAGCTGGTCGTGGCCGAAGCCTATGTCGGCAAGAACCTGACCATGAAGCGCGGTCGTCCGCGGGCTCGTGGCCGGTTCGGCAAGATCGTCAAGCCGTTCTCGGAACTCACCATCAAGGTCCGTCAGGTCGAGGAGCAAGCGTAAATGGCATCCGCAGCAGAGCAAATGGCAGCGAACATGAGCTGGGGGGCCTTCGGAAAGGCTACCGAGCTGAAGCAGCGCATCCTCTTCACGCTGGCTCTTCTCATTGTCTATCGCCTCGGCACCTACATTCCCGTCCCCGGCATCGACGGCACGGCCCTGCGCCAGTTCGTGCAGGAAGCCGCAACCGGCCTGGGCGGCATCCTCAACATGTTCACCGGCGGCGCCATCGGCCGTATGGGCATCTTTGCCCTGGGGATCATGCCCTACATCTCGGCCTCGATCATCGTGCAGCTTCTGACCGCGATGGTCCCCTCGCTTGAGCAGCTCAAGAAAGAGGGTGAGCAGGGCCGCAAGAAGATCAACCAGTATACCCGCTACGGCACCGTCTTTCTGGCCACGTTCCAGGCATACGGTCTGGCCGCCAGCCTCGAGGCCGGCGACCTGGCGACCGATCCAGGCCTCTATTTCAAGGCATCCGCCGTCATCACGCTTGTGGGCGGCACGATGTTCCTGATGTGGCTGGGCGAACAGATCACCTCGCGCGGGATCGGCAACGGCATCTCGCTGATCATCTTCGTCGGCATCATCGCCGAAGTCCCCGCCGCCCTGGCACAGTTCTTCGCCTCGGGCCGCTCGGGCGCGCTGAGCCCCGCGGTGATCGTGGGCGTCATCATCATGGTGATCCTGACCATCGCCTTCGTGGTGTTCATGGAACGCGCCCTGCGCAAGATCCACATCCAGTATCCGCGCCGTCAGGTGGGGATGAAGGTCTATGACGGCGGCTCGTCGCACCTGCCGATCAAGGTCAACCCGGCCGGCGTGATCCCCGCGATCTTCGCCAGCTCGCTGCTGCTGCTGCCGACCACGATCAGCACCTTCTCGGGCAGCCAGACCGGCCCGGTGATGTCGACCATCCTGGCCTACTTCGGCCCCGGACAGCCGCTCTACCTGCTGTTCTTCGCCGCCATGGTGATCTTCTTCACCTTCTTCTACACCGCCAACGTCGCGTTCAAGACCGACGACGTGGCCGAGAACCTGAAGAACCAGAACGGCTTTATCCCGGGGATCCGACCCGGCAAGCGCACCGAGGAATACCTGGATTACGTGGTCAACCGCATCCTGGTCCTGGGCGCAGGCTACTTGACCCTGGTGACGCTGCTGCCCGAGATCCTGCGCAGCCAGCTGGCGATCCCGTTCTACTTCGGCGGCACCTCGGTTCTGATCGTGGTCTCGGTGACGATGGACACGATCCAGCAGGTCCAGAGCCATCTTCTGGCGCATCAGTACGAGGGCCTGATCGAAAAATCTCAACTGCGCGGC---------AAGCGTCGCGGCAAGAAGGGGACAGCTAGACGATGAATGCAATTTCTCGACCTCGCCAAAGTGTATATCCGATCCGGCGGCGGCGGCGGTGGTGCCGTCAGTTTCCGCCGCGAGAAATACATCGAATATGGCGGTCCCGACGGCGGTGACGGCGGCAAGGGCGGGTCGGTCGTGGCCGAGGCGGTCGAGGGTCTGAACACCCTGATCGACTTCCGCTACCAGCAGCATTTCTTCGCCAAGAACGGCGTGCCCGGCATGGGCAAGCAACGCACCGGCGCCGATGGCGAGGACGTGATCCTGCGGGTGCCCGTCGGCACCGAGATCCTTGACGAGGACGAGGAGACGGTGATCGCCGACCTGACCGAGGTCGGGCAGCGCGTGGTGCTGGCCAGGGGCGGCAACGGCGGCTTCGGCAACCTGCATTTCAAATCCTCGACCAACCAGGCGCCGCGCCGGGCCAACGCCGGCCAGCCGGGGGTGGAGCGCACGATCTGGCTGCGTCTGAAGCTGATCGCCGACGCGGGCCTGCTGGGCCTGCCCAACGCGGGCAAGTCGACCTTCCTTGCTGCCAGCTCCAACGCCCGGCCCAAGATCGCCGATTACCCCTTCACCACGCTGCACCCTAACCTCGGCGTCGTCGGCATCGACGAGGTCGAATTCGTGATGGCCGACATTCCCGGTCTGATCGAGGGCGCGCACGAGGGCAGGGGCATCGGCGACCGCTTTCTGGGCCATGTGGAACGCTGCTCGGTCCTGCTGCACTTGGTCGACGGCACCTCCGAGGATGTGGCCGAGGATTACCGCGTCATCATCCACGAGCTGGAATCCTATGGCGGGGCGCTGGCCGACAAGCCGCGTATCACGGCACTGAACAAGATCGACGCGCTGGACGACGAGGAGCGGGCCGAGAAGAAGGCCGCGCTTGAGGCCGCCACCGGCGGCAACGTCCTGATGATGTCCGGCGTCAGCCGCGAGGGCCTGCCCGAGGTGCTGCGCGCCGTGCGGGCGCGGATCGACGCCGACAAGCTCCGCCAGCGAAAGGCTGAGGAGCCCGAGTCGTGGCGGCCCTGAATGGCTGCCAAACTCAAGAAGGGCGACAAGGTCGTCGTGCTGGCCGGTAAGGACAAGGGCAAGGAGGGTGAGATCACCCGCGTCATGCCTGCGGCCAACAAGGCCATCGTGGACGGTGTGAACGTGGCGATCCGCCATACCCGTCAAAGCCAGAACAGCCAGGGCGGCCGCGTGCCGACCCCGATGCCGATCGATCTTTCGAACCTGGCACTGCTGGACTCCAACGGCAAGGCAACCCGCGTCGGCTTCCGCACGGAAGACGGCAAGAAGGTGCGCTTTGCCAAGACCACGGGGGACGTGATC------TGAATGTTGCGCTCTGGTGTAATCGCAAAGAAGGTCGGGATGACCCGCCTTTTCATGGACGACGGGAAGCAGATTCCCGTGACGGTCCTTCAACTGGACAAGCTTCAGGTCGTGGGCACGCGCACCGCGGACGAGCACGGCTATTCGGCCGTTCAGCTCGGCGCCGGTACCGCCAAGGCCAAGCGCGTCTCTAAGGCCATGCGCGGCGTTTTCTCCGCCGTGAAGGTCGAACCCAAGCGTAAGATCGCGGAATTCCGCGTGGCGCCCGAGAACCTGATCGAGGTCGGCGAGGAAATCACCGCCAACCATTACTTCGAAGGTCAGTTCGTGGACGTCTCGGGCACCTCGATCGGTAAGGGCTTTGCCGGTGCCATGAAGCGGCACAACTTCGGCGGCCTGCGCGCCACGCACGGTGTCTCGATCAGCCACCGTTCGCACGGCTCCACCGGTCAGTGTCAGGACCCCGGCCGGGTGTTCAAGGGCAAGAAGATGGCCGGTCACATGGGCGCCGCCCGCGTGACCACGCAGAACCTGCAGGTCATCCGCACCGACGCCGACCGTGGCCTGATCATGGTCAAGGGCGCTGTCCCCGGCTCCAAGGGTGGCTGGGTCACGATCAAGGATGCGGTCAAGAAGCCGATCCCCGAGAACGTGATCTATCCCGCCGCGCTGAAGTCGGCCGCCGAGGAAGCCGAGCGTCTGGCCAAGGAAGCTGCCGAGCAGGCCGCTGCCGAAGCCGAAGCCGCCGAGAAGGCCGCCGCCGAGGCTGCCGCTGCCGAGCAGGCCGCCGCGCTGAAGGAGGCCGAGGCCTCT---------------GACGACACCGCCGCGCCCGAAGGAGGCGACAACAATGAAAGC------TGAATGGCACTCAAGTCGTACAAACCGACGACGCCGGGCCAGCGTGGGCTGGTTCTGATCGACCGTTCGGAGCTTTGGAAAGGACGTCCTGTCAAATCCCTCACCGAGGGTCTGACGAAGAAGGGCGGCCGGAACAACACCGGACGGATTACAATGCGCCGCCGCGGCGGTGGCGCAAAGCGTCTCTACCGGATCGTGGATTTCAAGCGCACGAAACTGGACATGACGGCGACCGTCGAGCGGATCGAATACGATCCGAACCGGACCGCCTTCATCGCCCTGATCAAATACGAAGATGGTCAGCAGGCATACATCCTGGCGCCCCAGCGTCTTGCCGTCGGTGACAGCGTTGTCGCCTCGGCCAAGGCCGACGTGAAGCCCGGCAACGCGATGCCCTTCTCGGGCCTGCCGATCGGTACGATCGTCCACAACATCGAGCTGAAGCCCGGCAAAGGTGGCCAGATCGCCCGCGCCGCCGGTACCTACGCCCAGTTCGTCGGTCGTGACGGTGGCTACGCCCAGATCCGCCTGAGCTCGGGCGAGCTTCGCATGGTCCGTCAGGAATGCATGTGCACCGTCGGTGCCGTGTCGAACCCCGACAACTCCAACCAGAACCTCGGCAAGGCCGGTCGCAACCGTCACAAGGGCATCCGCCCGTCGGTCCGCGGTGTGGTCATGAACCCGGTCGATCACCCCCATGGTGGTGGTGAAGGCCGGACCTCGGGTGGTCGTCACCCGGTCACGCCCTGGGGCAAGCCCACAAAGGGTGCCCGCACCCGGAACAAGAACAAGGCGTCCAGCAAGCTGATCATCCGCTCGCGTCACGCCAAGAAGAAGGGCCGGTAAATGCTGGACGCTGCGACCTATACCCCGCGTCTCAAGACGCTCTACACCGACGAGATCCGCGCCAAGCTGAAGGAAGAATTCGGCTACGCGAACGAGATGCAGATCCCGCGTCTCGACAAGATCGTTCTCAACATCGGCTGTGGCGCCGAGGCGGTCCGCGACACCAAGAAGGCCAAATCCGCTCAGGAAGACCTGACCGCGATTGCCGGCCAGAAGGCGCTGATCACCAAGGCCAAGAAATCCATCGCCGGCTTCCGTGTCCGCGAAGAGATGCCGCTGGGCGCCAAGGTGACCCTGCGCGGCGAGCGCATGTACGAATTCCTCGATCGCCTGATCACCGTCGCAATGCCCCGTATCCGGGACTTCCGCGGCGTGTCCGGCAAGAGCTTCGACGGTCGTGGCAACTACGCCACCGGCCTGAAAGAGCACATCGTGTTCCCCGAGATCAACTTCGACAAGGTCGATGAGGTCTGGGGTATGGATATCGTCATCTGCACCACCGCAGGTACCGACGCAGAAGCCAAGGCGCTGTTGAAGCATTTCAACATGCCCTTCAACAGCTGAATGAAAGCTGATGCGATCAAACTTGATGGCGCCAAGGCCGGCTCGGTCGAGCTGGACGACGCGATCTTCGGGCTCGAGCCCCGCGCAGACATCCTGCACCGTGTGGTCCGCTGGCAGCGCAACAACGCGCAGCAGGGCACCCACAAGGTCAAGACCCGGTCCGAGACCAGCTACTCGACCAAGAAGATCTATCGCCAGAAGGGCACCGGCGGCGCACGCCACGGTGACCGGAACGCGCCGATCTTCCGTAAGGGTGGTATCTACAAGGGTCCGACCCCGCGCAGCCACGGCCACGAGCTGACCAAGAAGTTCCGCAAGCTGGGCCTGCGCCATGCGCTGTCCGCCAAGCAATCCGCGGGCGAGCTGGTCATCCTGGACACGCTGGAAAAGGACGCCAAGACGGCCGTCCTGTCCCGGCAGGTCAAGGACCTGGGCTGGAAGCGCGCCCTGGTCATCGATGGCGCGACCGTGGACGAGAACTTCGCCCGCGCCGCACGCAACCTCGACGGTGTCGATGTGCTGCCGTCGATGGGCGCAAACGTCTATGACATCCTGCGGAGCGATACCCTCGTTCTGACCAAGGCGGGTGTCGAAGCACTGGAGGCTCGACTGAAATGAATGGAAAACGTGGTTCTCGTCATTCATCTGCTTCTGGCCATGGCCCTGATCGGGGTCGTGCTGCTGCAACGGTCCGAGGGCGGCGGCCTGGGGATCGGCGGCGGGGGCGGCGGTGTCGTGTCGGGCCGCTCGGCTGCGACGGCGCTGGGCAAGATCACCTGGTTCCTGGCGGCGGGCTTCATCGCGACATCCATCACGCTGACAATCTTTGCGGCACAGAATTCGGCAGGTCAGTCGGTCATCGACCGGCTGGCCCCGGCCCCCGCGGCCGAGTCGGGCGCCACCGACGGCGCGCTGCCGCCGGCATCGGATCTGCTTCCGCCCTCCAGCGGCTCGGCCACGCCGCTGACGCCGCCGCGCGCCTAGATGTCTTTCTTCAAGAAACTCAAAGACCGGATGCTGAAATCCTCCTCGCGGCTCGAGGCGGGGCTGGACGCGATCGTCGAGGAGGGGCAGGAGCCTTCGGGCCAGGCCCCGGCCGCGCCGCCCTCGGACGCC------------------------------------------GCACCGGCGCCGGCCCCGGTGCCTCCGGCTGATCCGGCG---------------------------------AGCCCCGCGACGGCGCCGTCCGGGGCGCCCGTCCCT------------------------------------------------------------GCGGCCCCCACCCCCGAGGCCCAAACTCCCGTCTCCCCA---------------------------GAGCCCGAAAGCAAGGGCGGCCTGATCGACCGGCTGATGGGGCGC---GGCGGCCAGGAGGTTGCCCGGCGCACCCTGGACGATCCCATGCTGGAACAGCTTGAGGAACTGCTGATCACCGCCGACATGGGCGTCGATACCGCCCTGCGCGTCACCGCCAACATCGCCGAGAGCCATTTCGGCAAGCGTGTCTCGGCCGAAGAGGTCAAGCGGCTGATGGCCGAGGAGATCGCCCGCATCATGGAGCCGGTGGCCCAGCCGATGCCGATCTACTCCAAAAAGCCCCAGGTCGTGCTGGTGGTCGGGGTCAACGGCTCGGGCAAGACCACGACTATCGGCAAGCTGGCCAGCCAGTTCCGCGCCGTCGGCAAGTCGGTCATCATCGCGGCGGGCGACACGTTTCGCGCCGCCGCGGTCGAGCAGCTTCAGGTCTGGGGCGAACGGGCCGGCGTGCCGGTGCTGACCGCCCCCGAGGGCAGCGACCCCGCCAGCCTGGCCTTCGACGCGATGCTTAAGGCCGAGGCCGAGGGCGCCGACCTGCTGCTGATCGACACCGCGGGACGGTTGCAGAACCGCGCCGACCTGATGGAAGAACTGGCCAAGATCGTCCGCGTCATCCGCAAGCGCGATCCCGAGGCCCCTCACAACACCCTGCTGGTGCTGGACGCCACCACCGGCCAGAACGCCCTGAGCCAGGTCGAGACCTTCCGCAAGCTGGCGGATGTCTCGGGTCTGGTGATGACCAAGCTGGACGGCACCGCCCGGGGCGGCGTGCTGGTGGCGCTGGCCGACCGCTTCGGCCTGCCAATCCACGCCATCGGCGTGGGCGAGCAGATCGACGACCTGGCGCCCTTCGATCCCGAGGATTACGCCTGTGCCCTGACGGGCCTGGAGGTTTGAATGACCGATGACACTTGGGGACGTGTTAGCAAGCTTTTGAAAAGGTCGGTGGGGAACAACAATTACACAACCTGGATCAAGCCGCTTCGATTTCTGAAGGAAGAGGCGGGCGTCGTGACCTTCGGCGTTCCGACCGTCTTTATCGGCGACTGGGTGTCGCGGAACTTCGGGGATCAGATCCGCCAGCACCTGATGGCTGAGGGCGAGGCTGTGGAACGGTTGCGTTTCGTTGTTCCGCCGGCCCAGGCCAGCGCCGACCAGGCCCCCGCGGGCAAGGCCCAGGCCGCACGCGCCGGCCGTGACCCCGAGGACAGCGCCGCCTCGGTCATCCCGGGGGCGCCGCTCGATGCCCGCTTCACCTTCGACAGCTTCGTCGTCGGCAAGCCGAACGAGCTGGCAAACGCCGCCGCGCGGCGGGTTGCCTCCTCGGGGCCCGTGACGTTCAACCCGCTTTTCCTTTATGGCGGCGTCGGTCTCGGCAAGACCCACCTGATGCACGCCATCGCGTGGGAGCTTCAGGCCCGCAGCCCCGAAAAGAAGGTCGTGTATCTCTCGGCTGAACAGTTCATGTACCGCTTCGTGCAATCCCTGCGCGACCGCTCGACCATGGATTTCAAACAGCTATTCCGCTCGGTCGATGTTCTGATGGTGGATGACGTTCAGTTCATCGCCGGCAAGAACTCCACCCAGGACGAATTCTTCCATACCTTCAACGCGTTGGTCGATCAGGGCCGGCAGATCATCATCTCGGGCGACCGCTCGCCCTCGGAGATGGACGGCATGGACGAGCGCATCCGCTCGCGGCTGCAATGGGGTCTGGTCGTCGACCTGCACCCGACCGACTACGAACTGCGCCTCGGGATCCTGCAGTCCAAGGTCGAGCAGTATCGCCGCGACTATCCGCAACTGGATATCGCCAACGGCGTGCTGGAATTCCTGGCGCATCGCATCTCGACCAACGTGCGGGTGCTGGAAGGCGCGCTGACGCGCCTCTTCGCCTTCGCCTCTCTTGTGGGCCGCGAGATCACGCTGGACCTGACCCAGGACTGCCTGGCCGACATTCTGCGCGCCAGCGACCGTAAGGTCACGATCGACGAAATCATCCGCAAGGTCAGCGAGCATTACAACATCCGCATGTCGGACATCCTCAGCCCCAAGCGGACGCGCACCATTGCCCGCCCGCGCCAGGTGGCGATGTATCTCGCCAAGCAGCTGACCTCGCGGTCGCTGCCCGAGATCGGCCGGCGTTTCGGCGGTCGCGACCACACCACGGTGCTGCACGCGGTCCGCAAGATCGAGGAGCTGCGGTCGATCGACAACCAGATCGACGAGGATGTGGAACTTTTGCGCCGCATGCTCGAGGCATGAATGCTGGGTCTCGGAACCATCGCCAAGAAAGTATTCGGAACGCCAAATGACCGCGAGGTGAAAGCCCGCAGGCCGCTTGTCGAAAAGATCAACGCCCTCGAGCCACAGTTCACCGACCTCACCGATGAGCAGATCATCGAGAAGACCGCCGAGTTCCGCGAGCGCCTGGCCAAGGGCGAAGCCCTGGACGACCTGCTGCCCGAGGCCTTCGCCAACGTGCGCGAAGCGGCCCGCCGCACCCTGGGCCTGCGGGCCTTCGACGTGCAGCTCATGGGCGGGATCTTCCTGCACCAGGGCAACATCGCCGAGATGAAAACCGGCGAGGGCAAGACGCTGATGGCGACCTTCCCGGCCTATCTGAACGCCCTGACGGGGCGCGGCGTGCACATCGTCACGGTCAACGACTACCTGGCGCGGCGCGATGCCGAATGGATGGGCAAGGTCTATGCCGCCCTGGGCCTGACCACCGGCGTGGTCTACCCCCAGCAGCCCGAGCAGGAAAAGGCGGAGGCCTACGGCGCCGACGTCACCTACGCCACCAACAACGAGCTGGGTTTCGACTACCTTCGGGACAACATGCGCATGGATCTCGACGAGATGAACCAGCGCGACCACTACTTCGCCATCGTCGACGAGGTCGATTCGATCCTGATCGACGAGGCGCGCACGCCGCTGATCATCTCGGGCCCCTCCGAGGACCGGAGCGAACTTTACAAGACCATCGACACGGTGGTCCCCAGCCTCTCGGACGAGCATTTCACCATCGACGAGAAGACCCGCAACGTCACCTACACCGACGAGGGCAACGAGTTCCTGGAACAGGAGCTGCTGCGTCAGGGGATCCTGCCGGAGGGTCAATCCCTCTATGACCCCGAGTCGACGACGCTGGTGCACCACGTCACCCAGGCCCTGCGCGCCCACAAGCTGTTCCAGCGCGACAAGGATTACATCGTCCGCGATGACGAGGTCGTGCTGATCGACGAATTCACCGGCCGCATGATGTCGGGTCGCCGCCTGTCGGACGGCCTGCACCAGGCGATCGAGGCCAAGGAGAACGTCTCGATCCAGCCCGAGAACGTGACCCTGGCATCGGTCACCTTCCAGAACTATTTCCGGCTTTACGACAAGCTGGGCGGCATGACCGGCACCGCCGCGACCGAGGCCGAGGAATTCGCCGAGATCTACAAGCTGGGCGTGATCGAGGTGCCGACCAACCGGCCCATCGCCCGCGTCGACGAGCATGACGCCGTCTACCGGACCGCCAAGGAAAAATACGACGCCATCCGCGAGACGATCGAGGAGGCCCACAAGAAGGGTCAGCCGATCCTCGTCGGCACCACCTCGATCGAGAAGTCCGAGTTCCTGTCGCAGATGCTCAAGGAACGCGGCGTGCCCCACAACGTCCTCAACGCCCGCCAGCATGAGCAGGAGGCCGCCATCGTCGCCGACGCCGGCAAGCCGGGCGCTGTGACCATCGCCACCAACATGGCCGGTCGCGGCACCGACATCCAGCTTGGCGGCAACGTCGACATGAAGGTGCTGGAGGCCCTGGCCACCGACCCCGAGGCCGATCCCGCCGCCCTGCGCGAACGCGCCGAGGCCGAGGTCGCCGGCGACAAGAAGAAGGTGCTGGAGGCAGGCGGCCTGTTCGTTCTGGCCACCGAGCGGCACGAAAGCCGCCGCATCGACAACCAGCTGCGCGGCCGCTCGGGCCGTCAGGGCGACCCCGGCCGCTCGGCCTTCTACCTCAGCCTTGAAGATGACCTGATGCGGATCTTCGGGTCCGAGCGTCTGGACAACGTGCTGGGCAAGCTGGGCATGAAGGAAGGCGAGGCGATCATCCACCCATGGGTGAACAAGAGCCTCGAGAAGGCCCAGGCCAAGGTCGAGGCGCGCAACTTCGACATCCGCAAGCAACTGCTGAAGTTCGACGACGTGATGAACGACCAGCGCAAGGCCATCTTCGGCCAGCGTCTGGAGATCATGGAAAGCAAAGACGTCAACGAGATTGTCGAGGATATGCGCCACCAGGTCATCGACGACCTGGTCGATTTCTACATCCCCGCCCGGTCCTACGCCGACCAGTGGGATGGCGAGGGGCTTTATGCCGCTGTCATCGAGAAGCTGGGCGTCGACGCCCCCGTCATCGCCTGGACGCAGGAAGAAGGGGTCGATGATTCCGACATCCGCGAGCGTCTTTACAAGGCAACGGACGAGTTCATGGCCGGCAAGGCCGCCAAGTTCGGCCCCGAGCAGATGCGCCGTATCGAAAAACAGGTCCTGCTGCAGACCATCGACGCCAAGTGGCGCGAGCATCTTGTGACGCTGGAGCATCTGCGCTCGGTCGTGGGGTTCCGCGGCTATGCACAGCGCGATCCGCTCAACGAGTACAAGAACGAAAGCTTCCAGCTTTTCGAGAGCCTGCTGAACTCCCTCCGCGAGGAAGTGACCGAGAAGCTGGCACAGCTTCGCCCGCTCAGCGAGGAAGAGCAGAAGCAGATGCTGGCCCAGCTGATCGAACAGCAGCGTGCCTTGCAGGGCGCCGGCGCGCAACCC---------------GCCGCACCGGCGGGCCCGCAG---GCGGCCGCACAGCCTGACGGGGCCGGAGAGACAGCCGTCGCCGAGGTGGGGCGCAACGATCCCTGCCCCTGTGGCTCGGGCAAGCGCTACAAGCATTGCCACGGTGCCGTCACCTGAGTGAACCTTTCGGCTGAACTCGACCAATTCTCCAAGCGGATCGGCTATCGCTTCAGAAAGCCGGAGCGCCTGGTCGAGTCTGTCACCCACGCCTCGATCTCGACCGCGACCCGCCCCGACAACCAGCGCTACGAATTCCTGGGCGACCGAGTGCTGGGCCTGGTCATGGCCGAGGCGCTGCTGAAGGCAGATCCCGCGGCCTCGGAGGGGCGGCTGGCGCCCCGGTTCAACGCGTTGGTGCGCAAGGAGACCTGCGCCGAAGTGGCCCGCGATGCCGACCTGGGCAGCGTGCTGAAGCTTGGCCGTTCAGAGCAGATGTCGGGCGGCCGCCGCAAGGAGGCCCTGCTTGCCGACGCCATGGAAGCGGTGATCGCGGCGGTCTACCTGGACGGCGGGTTCGAGGCGGCGCGCGACATGATCCTGCGCCTCTGGGGCGACCGGATCGAGAAGGTCGAGGCCGACGCCCGCGATCCCAAGACCGCGTTGCAGGAATGGGCCCAGGCCCGGGGGCAGCAGCCCCCCGCCTACCGCGAGGTCGGCCGCGACGGCCCCGACCACGCGCCGCAATTCACCATCGAGGCCCGGCTTGCCGATGGCCTGAGCGAACAGGCGACGGCCGGCTCCAAGCGCCAGGCCGAACAGGCGGTGGCGCGCAAACTTCTGGAACGGGTTGAAAAGAATGGCTGAATGGCAAAGACCGGAAAACGCACAGCAGCCGCCAAGGCCGCCTTCGCCGACAAGCACGACCTCACCGTCGAGGAGGCGATTGCCCTGATTAAGGACAACTCCAAGACCAAATTCGACGAGACCGTCGAGATTGCGATGAACCTGGGTGTCGACCCGCGCCACGCCGACCAGATGGTCCGCGGCAAGGTGACCCTGCCCAACGGCACCGGCAAGACCGTTCGCGTCGCCGTCTTCGCCCGTGGCGCCAAGGCAGACGAAGCCAAGGAAGCCGGTGCGGACATCGTTGGTGCCGAGGACCTGATGGAGACCATCCAGGGCGGCGAGATCAACTTCGAGCGTTGCATCGCCACGCCCGACATGATGCCGATCGTCGGCCGCCTGGGTAAGGTCCTGGGCCCGCGCAACCTGATGCCGAACCCCAAGGTGGGTACGGTCACCATGGATGTCGCCGAAGCCGTCAAGGCCGCCAAGGGCGGCGAGGTCCAGTTCCGCGCCGAGAAGGCCGGTGTGGTCCACGCCGGCGTCGGCAAGGCCTCGTTCGAGGTCGACAAGCTGGTCGAGAACGTCCGCGCCTTCGTGGACGCGGTCGCCAAGGCCAAGCCGACAGGTGCCAAGGGCGCCTACATGAAGAAGGTTTCGCTGAGCTCCACCATGGGGCCTGGCGTGTCGGTGGACATCACGTCCGCAACCGGCAACTGAATGGCAGACGATATCGAAATCGATCTGGACGATCTTGGCCATCGCATGGATGGCGCCATGACCGCGCTGCGCTCGGAATTCCAGAGCCTGCGCACCGGGCGGGCCTCGGCCTCGATGCTGGATACGATCACGGTCAACGCCTATGACACGGTCACCCCGCTCAACCAGGTGGGCACGGTTAACGTCCCCGAACCGCGGATGCTTACCGTCACCGTCTGGGACAAGCAGCTGGTCAACAAGGTCGAAAAGGCGATCCGCGAGTCGGGGCTGGGGATCAACCCGGTCATGGACGGCACGGTCATCCGCCTGCCGATCCCCGAGCTGAACGAGGAACGCCGCCGCGAGCTGACCAAGATCGCCGCCCAATATGCCGAGGCCGCCCGCGTGGCGGTGCGCAACGTGCGCCGCGACGGGATGGACCAGATCAAGAATGCCAAGTCCGCGGGCATGAGCGAGGACGACCAGAAGATCTGGTCCGGCGAGATCCAGGAGATGACGGACAAGCACATCGCCGCCATCGACGCGGCGCTGGAGTCCAAGCAGGCCGAGATCATGCAGGTCTGAATGGCCAAGAAAGTAGCTGGTACGATGAAGCTCCAGGTCCCCGCGGGCCAGGCAAACCCGTCGCCGCCCGTGGGCCCCGCCCTGGGTCAGCGCGGCATCAACATCATGGAATTCTGCAAGGCGTTCAACGCCAAGACGCAGGAGATGGAGCAGGGTGCCCCCTGTCCGACCGTGATCACCTACTACCAGGACAAGTCCTTCACCATGGACATCAAGACGCCCCCGGCGTCCTACCTGCTGAAGAAGGCCGCCAAGCTGAAGTCCGGCGCCAAGACCCCCAGCCGTGAGACCGCCGGTTACGTGACCGTCGCTCAGGTGCGCGAGATCGCCGAGACCAAGATGAAGGACCTCAGCGCGAACGACATCGAGGGTGCAATGCAGATCATCCTGGGCTCGGCCCGTTCGATGGGTATCGAGGTGAAATAAATGTTCGAGACGCTATCCGAACGCCTCTCCGGTGTATTCGACAAGCTCGGCAAGCAAGGCGCCCTGAGCGACGAGGATGTAAAGACCGCCCTGCGCGAAGTCCGCGTGGCGCTGCTGGAGGCCGACGTCTCGCTTCCCGTGGCCCGCGACTTTGTCGCCGCCGTCCAGGAAAAGGCCACCGGCCAGGCAGTCACCCGCTCGGTCACGCCGGGCCAGCAGGTCGTCAAGATCGTCCATGACGAACTGGTCCATGTCCTGGCCGGCGACGGCACCGCCGGTCAGCTGAAGATCGACAACCCGCCCGCGCCGATCCTGATGGTCGGCCTGCAGGGTTCGGGCAAGACGACCACCACCGCCAAGCTGGCCAAGCGGCTGAAGGAAAAGTCGGGCAAGCGGGTGCTGATGGCTTCGCTTGACGTGAACCGCCCCGCCGCGATGGAACAGCTTGCCGTTCTGGGCGTGCAGGTCGGCGTCGACACCCTGCCCATCGTCAAGGGCCAGACCCCGGTCGAGATCGCCCGCCGCGCCAAGCAGCAGGCGACGATGGGCGGCTATGACGTCTACATGCTGGACACCGCCGGGCGCCTCTCCATCGACGAGGCGCTGATGGCCGAGGTCGAGGCCGTCCGCGACGTAACCTCCCCGCGCGAGACCATGCTGGTCGTCGACGGCCTGACCGGCCAGGACGCCGTTCACACCGCCGAGAATTTCGACGCCCGCATCGGCATCTCGGGCGTCGTGCTGACCCGGATGGACGGCGACGGCCGCGGCGGTGCTGCCCTTTCCATGCGCGCCGTCACCGGCAAGCCGATCAAGTTCGTCGGCCTTGGCGAGAAGCTGGACGCGCTGGAAGAGTTCCACCCCGAGCGGGTCGCCGGCCGCATCCTTGGCATGGGCGATATCGTCAGCCTGGTCGAAAAGGCCCAGGAGACGATCGAGGCCGAACAGGCCGAGCGCATGATGAAGCGCTTCCAGAAGGGTCAGTTCAACATGAACGACCTGAAGTCCCAGATCGAGCAGATGCAGAAGCTGGGCGGCATGGAAGGCGTGATGGGCATGCTGCCGGGCATGAAGAAGATGTCCAAGCAGGTCGAGGCCGCCGGCATGGACGATACCCTGCTGAAACGTCAGATCGCCCTGATCCAGTCGATGACCAAGAAGGAACGCGCCAACCCGCAGCTCCTTCAGGCCAGCCGCAAGAAGCGCATCGCCAAGGGCGCGGGGCTTGAGGTCAGCGAGCTGAACAAGCTGCTGAAGATGCACCGCCAGATGGCGGACATGATGAAGAAGATGGGAAAAATGGGCAAAAAGGGCATGATGCGCGGCGCCTTGGGCCAGATGTTCGGCAAGGGCGGC------CCCAGC------------CAGGCCGATATCGAGGCCGCGAAGGCCCAGATGGGCGGCGCTGGCGGCCAGATGCCCGCGGGCCTGCCCGGCATGGGCGGCGGTCTTGGCCTGCCCCCCGGCCTGTCCGGTTTCGGGAAGAAGAAATGAATGGTTCCAGCAGACAAACTCGCCCAGATACTCGAGCGGTTTCAGTATATCGAAGCCCGCATGTCCCAGGGCTCG------GGCGATATCGCGCAGCTTGGCCGCGAGTATGCGGCCCTGCGCCCCGTGGTCGAACAGGTCGAGGGGTACAACCGCCTGATCGCCGATATCGCCGAGGCCGAGGCGATGCTGGACGACCCCGAGATGCGCGCCCTGGCCGAAGATGAACTGCCGGCCCTGCGCACCCGTCTGCCCGAGGCCGAGGCCGCCGTGCGCCTGGCCCTTCTTCCCAAGGACGCCGCCGACGAACGCGCCGCCATCGTCGAGATCCGCCCCGGCACCGGCGGTGAGGAGGCAGCACTCTTCGCCGGCGACCTGTGGCGCATGTATCAGCGCTACGCCGAAGGGCGCGGCTGGTCCGTCTCGGTCATCGAGGAAAGCCTGACCGAGCTGGGCGGCCTGAAGGAGCTTGTCGCCAACGTGCGCGGGCAGGGGGTCTTTGCCCGGCTGAAGTTCGAATCCGGCGTCCACCGGGTGCAGCGCGTGCCCTCGACCGAATCCGGCGGCCGCATTCACACATCGGCCGCCACCGTCGCCGTCCTGCCCGAGGCCGAGGAAGTCGATATTGACATCCCCTCCACCGACATCCGCATCGACACGATGCGCGCCTCGGGGGCCGGGGGGCAGCACGTGAACACGACCGACTCGGCCGTGCGGATCACCCACATCCCCAGCGGCATCGTCGTCACCAGCTCCGAGAAATCCCAGCACCGCAACCGCGAGATCGCGATGCAGGTGCTGCGCGCCCGGCTTTACGACGCCGAGCGTCAGAAGGTCGACAGCGCCCGCGCCGCCGACCGCAAGGCGCAGGTGGGCTCGGGCGACCGGTCCGAGCGGATCCGCACCTACAACTTCCCCCAAGGGCGGATGACCGACCACCGCATCAACCTGACCCTCTACAAGCTGGATCAGGTGATGCAGGGGGACCTGGACGAGATCATCGACTCGCTGATCGAGGAGGATCAGGCCCGCCGCCTGGCCGAGATGGAGCAATGAGTGGATAGAGCCCAGAAAGAAAAATTGGTCGAAGAGCTCGGCCAAATCTTCGAAAGCTCTGGCGTCGTTGTGGTTGCCCACTACGAAGGTCTCACGGTTGCCGAAATGCAGGATCTGCGGGCGCGTGCGCGCGACGCAGGTGGTGCAGTTCGCGTTGCCAAGAACAAGCTCGCCAAGATCGCCCTTGATGGAAAGCCCTGCGCAAGCATTGCCGACTATCTGACGGGCATGACCGTTCTGACCTATTCCGAGGACCCCGTGGCAGCAGCCAAGGTGGCCGAGGACTTCGCCAAGGAGAACAAAAAGTTCGAAATCCTTGGCGGTGCTATGGGCGAGAACGCTCTGGACCGGGCCGGTGTCGAGGCCGTGTCGAAAATGCCGTCGCGCGAGGAGCTTATTGCTACCATCGCTGGCATGATCGGTGCGCCTGCTTCGAACATCGCCGGTGCGATTGGCGCGCCTGCTTCGAACATCGCGAGCATTCTTTCGACAATCGAAGAGCGCGCGGAAGCGTGAATGAAAACCTTTACCGCCACTCCGGCGGATATCGACAAGAAATGGATCCTGATCGACGCCGAGGGCGTCGTTCTTGGCCGTCTTGCCGCTATCGTCGCCACCCGCCTTCGCGGCAAGCACAAGCCCAGCTTCACCCCGTCCCAGGACATGGGTGACAATGTCATCGTGATCAACGCCGACAAGATCCAGCTGACCGGCAACAAGCGCATGAAGCCCAACTACTGGCACACCGGTTATCCGGGCGGCATCAAGTCCCGCACCACCGGCCAGATCCTGGAAGGCGAGCACCCCGAGCGCGTCGTCATCCAGGCCGTCAAGCGCATGCTGCCCGGTGGCAAGCTGAGCCGTCAGCAGATGACCAACCTTCGCGTCTATGCCGGTGCCGAGCACGGCCACGAGGCCCAGAGCCCCGAAGTCCTGGACGTGAAATCCATGAACTCCAAGAACACGCGGAGC---TGAGTGACGGCGGCCATGGCCGCCATCCTGGGGGCCGCCGACGATCCTGACCCCTCCCGCCCACCCACCGCCCTCGGGCTTGCCGTCTCGGGCGGCAGCGACTCGCTGGCTCTGATGCACCTGGCCGCCGCTTGGGCGGCGCCCAGGGCTATCCGCCTGCGGGTGGCAACCGTGGATCACCGCCTGCGCCCCGAATCCGGGACGGAAGCGCGGGAGGTGGCCCACGCCGCGGCCGCGCTGGACCTTCCCCATGAGATCCTGGAATGGACCGACGGCCCCGGCGCCGGCAACCTTCAAGCCAATGCCCGCGATGCCCGCCGCCGCCTGCTGGGCGACTGGGCCGCGCGGCACGGGCTGACGGGGGTGCTGACCGGCCACACCGCCGACGACCAGGCCGAGACGGTGCTGTTGCGCCTGGCGCGCGGCTCGGGCGTGGACGGCCTGGCGGCGATGCGGCCGGGCCGCCCGGGGCGATCCCTT---TTTCTGCGTCCGCTATTGGGGCACCGCCGCGAGGAGCTGCGCGACCTTCTGCGTGCCAAGGGCCTGACCTGGGCCGAGGATCCGGGCAATGAAGATTCCGTCTATGACCGGGTCAAGGCGCGTCGGGCGCTGGCGCTGCTGGCCCCGCTTGGCATCGATGTCGAGGGGGTGAACCGCACGGCCGATGCCATGGCCCGCGCCCGCGAGGCACTGGAGCGGCGGGGCGCCGAGGCCGCCGCCGCCATGACCCGCGAGGAGGGCGCCGACCTGCTGATCGAGGTCGCGCCCTGGCGTCTGTTGGACGACGAGACGCGCCTGCGCCTTCTGGCGGCGGGGCTGATGTGGGTCGGCGGTCAGGACTACCGCCCCCGCCTGCGCGCGCTGGAAGACACGGTCCGCGCCGCGGCCGCTGGCCGGCGCGCCACGCTTCACGGGGTCGTGATACATCCTGCCGCAGGGTGGCTGCGCCTTTACCGCGAGCCCTCGGCGCTGATCGGGGTGCGGGCGCGGCCCGGCGACACATGGGACAACCGCTGGATCCTGGCGCGCGAGGCGGGCACCACCGAGGGGGCACAGGTCGAAATGCTTGGCGAAGAAGGGCTTACCGCCCTT---GATCCGCGCCCCGCGGGCGTTCCGCGCGACAGCCTGCTGGGCCTTCCGGCCGTCTGGCGGGCGGGGCGGGTGATCTGTGTGCCGCACCTCGGGATGGGCGGCGGATTTCGCGCAACCCGCGCAGGGGAGCAGGAA---TTCACCCGGACGTTGTTATCTCATTGAATGGCTGATCTGAAGAAACTTGCTGAAGAGATCGTGGGTCTGACCCTTCTCGAAGCACAAGAACTGAAAACCATCCTCAAGGACGAGTACGGCATCGAGCCCGCTGCTGGCGGCGCTGTCATGATGGCAGGTCCTGCTGACGCCGGCGACGCAGGCGAGGAACAGACCGAGTTTGACGTGATCCTGAAGGCCGCTGGCGCCCAGAAGATCAACGTCATCAAAGAAGTCCGCGCCATCACCGGCCTGGGCCTGAAAGAAGCGAAAGAGCTGGTTGAAGCCGGTGGAAAAGTCAAGGAAGGCGTTTCCAAGGAAGAAGCCGAAGACATCAAGGGCAAGCTGGAAGCAGCTGGCGCCGAGATCGAGCTCAAGTAAATGTCTCGTATTGGTAAGAAACCGGTCGAGCTGCCGTCGGGTGTTTCGGCAACCGTCTCCGGCCAGACCGTCGAAGTGAAGGGCCCCAAGGGTACCCGCAGCTTCACTGCAACCGACGATGTCACCATTTCGGTGGACGACAACGTCATCTCGATCGCGCCCCGTGGCTCGTCCAAGCGCGCCCGCCAGCAGTGGGGCATGTCCCGCACGATGGTCGGCAACCTGGTGACCGGCGTCTCCACCGGCTTCAAGAAAGAGCTGGAGATCAACGGTGTGGGTTACCGCGCGCAGATGCAGGGCAACACCCTGAAGCTGAGCCTGGGCCTGAGCCATGACGTGAATTTCGAAGTGCCCGAGGGCGTCACCGTGACGGCCCCCAAGCAAACCGAGATCGTCGTGGAAGGCATCGATCAGCAACTCGTCGGCCAGGTCGCGGCCAATATTCGCGAATGGCGGAAGCCCGAGCCCTACAAGGGCAAAGGCATCAAGTACAAGGACGAGTACATCTTCCGCAAGGAAGGCAAGAAGAAGTAAATGGACGTTATCCTTCTAGAGCGTGTGGCCAAGCTGGGTCAGATGGGCGAAGTCGTTTCCGTCAAGGAAGGCTATGCGCGCAACTACCTGCTGCCTCAGGGCAAGGCCCGTCGCGCCTCCGATGCCAACCTCAAGATGTTCGAAGAGCAGAAGGCCCAGCTCGAGGCCCGCAACCTCGAGACCAAGAAAGAGGCCGATAGCCTCGCCGCCAAGATCGACGATCAGCAGTATGTCGTGATTCGCTCGGCCTCCGACGCCGGCGCGCTCTACGGCTCGGTCACCACCCGTGACGCTGCCGACGCAATCAACGAAGACGGCGTGTCGATCGACCGCAAGCAGATCGTTCTGTCCGAGCCCATCAAGTATCTGGGCCTGCACACCGTCACCGTCGTCCTCCACCCCGAGGTCACCGCGTCGGTCAAGCTGAACGTCGCCCGCTCGCCCGAAGAGGCCGAGCTTCAGGCGTCGGGCAAGTCGATCCAGGAACTGGCAGCCGAGGAAGAGGCCGAGGCCGAATTCGAGATCGCCGAGCTCTTCGACGATATCGGAGCCGCCGGCCTCGACGACGATGATCGC------------------GACGAGCGCGACAGCGACGACGAGACCGAAGAGAAC---TGAATGAGCGATCAAGACGGCAAGAAAACTCTCGGTCTTCGCGGCGGACCTCGCAGCGGTCAGGTTAAGCAAAGCTTCAGCCATGGCCGGACGAAGAACGTCGTGGTGGAAACCAAACGCAAACGCGTCGTGGTTCCCAAGCCGGGTGCTTCCAAGGCCGCCGGCGCCGCGTCGAACCGCGGGGGTGACGCCTCCAAGCGTCCCGCCGGGATTACCGATGCCGAGATGGAGCGTCGCCTGAAGGCGTTGCAGGCCGCCAAGGCCCGCGAAGCCGAAGAGGCCGAGCGCCGCGAGCGCGAGGAGCGCGAGCGCGCCGAAGACCGCGAACGCCGCCGCGCCGAGGCCGAGGCCAAGGAACGCGAAGAGCGTGAGCGCGAAGAGCGTGCCCGCCAGAAGGCCGAAGAGGACGAGCGCAAGCAGCGCGAGGCCGAGGAAGCCAAGCAG---GCCGCACAGCCCGCA------CCGCAGGAACGCGCCGAGCCCCAGGCTGACGCCGGTCCCGCCGCAACGCCCCGCAAGGAGCGGGACGACCGTCCCAAGCGCGAAGCCAAGGGCGCCGGCGACCGC------CGCCGCTCGGGCAAGCTGACGCTGAACCAGGCGCTTTCGGGCGGCGAGGGTGGCCGTCAGCGGTCCATGGCCGCGATGAAGCGCAAGCAGGAACGCGCCCGCCAGAAGGCGATGGGTCAGAACGTCGAGCGCGAAAAGGTCGTGCGCAACGTCAACCTGCCCGAGGCGATCACCGTCCAGGAACTTGCCAACCGCATGGCAGAGCGTGTGGCCGACGTGGTCAAGTCGCTGATGACCAGCGGCATCATGGCGACCCAGAACCAGACCATCGACGCCGATACCGCCGAGCTCATCATCGAAGAGTTCGGCCACAAGGTCGTGCGCGTTTCGGATGCCGACGTCGAGCAGGTCATCGACACGATCGACGACAAGCCCGAGGATCTGCGCAACCGTCCGCCGGTCATCACCGTCATGGGTCACGTCGACCACGGCAAGACCTCGTTGCTGGACGCGATCCGCAACGCCAAGGTCGTCGCGGGCGAAGCCGGCGGTATCACCCAGCACATCGGCGCCTACCAGGTGCAGGCGGCCGACGGCCAGATCCTGACCTTCCTGGATACGCCCGGCCACGCGGCCTTCACCTCGATGCGTGCCCGTGGTGCGCAGGTGACCGACATCGTGGTCCTGGTGGTCGCGGCCGATGACGCCGTGATGCCCCAGACCGTCGAAGCCATCCACCATGCCAAGGCGGCCGGCGTTCCGATGATCGTCGCGATCAACAAGATCGACCGTCATGAGGCCAACCCCGACAAGGTGCGCACCGATCTGCTGCAGCACGAAGTCGTGGTCGAGAAGATGTCCGGCGACGTGCAGGATGTCGAGGTTTCGGCCATCAAGGGCACCGGCCTGGACGAACTGCTTGAGGCGATTGCCCTGCAGGCAGAGATCCTGGAACTGAAAGCCAACCCCGACCGTGCCGCCTCCGGCGCCGTGATCGAGGCCCAGCTGGACGTCGGCCGCGGTCCCGTGGCCACCGTCCTGGTTCAGAACGGCACCCTGCGCCGCGGCGACATCTTCGTCGTGGGCGAGCAGTACGGCAAGGTTCGCGCCCTGATCAACGACAAGGGCGAGCGGGTCGACGAAGCCGGCCCCTCGGTCCCGGTCGAGGTTCTCGGCCTCAACGGCACCCCCGAGGCCGGTGACGTGCTCAACGTCGTCGAGACCGAGGCACAGGCCCGCGAGATCGCCGAATACCGCGAGAAGGCGGCCAAGGAAAAGCGCGCCGCTGCCGGTGCCGCGACGACCCTCGAACAGCTCATGGCCAAGGCCAAGGACGACGAGAACGTCTCGGAGATGCCGATCCTGGTCAAAGCCGACGTTCAGGGTTCCGCCGAAGCCATCGTTCAGGCGATGGAGAAGATCGGGAACGAGGAGGTGCGCGTGCGCGTCCTGCATTCCGGCGTCGGCGCGATCACTGAATCCGACATCGGCCTGGCCGAAGCCTCGGGCGCGCCGGTCTTCGGCTTCAACGTCCGTGCCAATGCCTCGGCCCGCAACAGCGCCAACCAGAAGGGCGTGGAGATCCGCTACTACTCGGTGATCTACGACCTTGTGGACGACGTGAAAGCGGCCGCATCCGGCCTGCTGTCGGCAGAGGTTCGCGAGAACTTCATCGGCTACGCCGAGATCAAGGACGTGTTCAAGGTGTCCAACGTCGGCAAGGTCGCCGGCTGTCTGGTCACCGAGGGTGTTGCCCGCCGTTCGGCCGGTGTGCGCCTGCTGCGCGACAACGTGGTGATCCACGAGGGCACGCTGAAGACGCTCAAGCGCTTCAAGGACGAGGTTGCAGAGGTCCAGTCCGGCCAGGAATGCGGTATGGCCTTCGAGAACTACGACGATATCCGTGCCGGCGATGTCATCGAGATCTTCGAGCGCGAAGAGGTCGAGCGTACCCTGAGCTAAATGGCCAAGCAGAAGAAGACCCCCCGGCCCAAGGCGGAAACCCCCAAGGGATTCCGCGATTATTTCGGGGCCGAGGTGCAGGGCCGCAAGGCGATGCTGGACATCATCGGGCAGGTCTATCACCGCTATGGGTTCGACGCGCTGGAAAGCTCGGCCGTCGAGACGGTCGAGGCGCTGGGCAAGTTCCTTCCCGATGTCGACCGCCCCAACGCCGGCGTCTTCGCCTGGCAGGAGGATGCCGAGGGTGAGAAACCCGGCGACTGGCTGGCGCTGCGCTATGACCTGACAGCGCCTCTGGCACGGGTCTATGCCCAGCACCGCAACGATCTGCCCACCCCATACCGCCGCTATGCGATGGGCCCGGTCTGGCGCAACGAAAAGCCGGGGCCGGGGCGTTTCCGCCAGTTCTATCAATGCGATGCCGACACGGTTGGCGCGCCCTCGGTCGCCGCCGACGCCGAGATCTGCGCCATGCTGTCCGACACGCTGGAAGCGGTTGGCATCCAGCGCGGCGACTACATCGTGCGCGTCAACAACCGCAAGGTTCTGAACGGGGTGATGGAGGTGGCCGGCGTGGCCGATCCCGACTTCGCCGATGAGCGCGGCATCGTGCTGCGGGCCATCGACAAGCTGGACCGGCTGGGGACGGGGGGCGTGCGCGCCCTGCTGGGCGCGGGTCGCGAGGACGAGAGCGGCGATTACACCAAGGGCGCGGGCCTCTCGGAGGCGCAGGCCGATGTCGTCATGGGCTTCATGGAGGCGCGGCGCGAGGACGGGGCGGCCACCGTGGCGCGGCTGTCCGAGCTGGTCGGGGACTCGGTCGTCGGGCGCGAGGGCGTGGACGAGCTGCGCCAGATCGCGGATCTGCTGGCCGCCCAGGGCTATGGCCCGGACCGGATCGTCATCGACCCTTCGGTCGTGCGCGGGCTGGGCTATTACACCGGCCCGGTGTTCGAGGCCGAGCTGACCTTCGAGATCCTGGACGAAAAGGGCCGCAAGCGGCAGTTCGGCTCGGTCGCGGGGGGCGGACGCTATGACGATCTGGTCAAGCGGTTCACCGGTCAGTCGGTCCCGGCCACTGGCGTTTCGATCGGCGTGGACCGCCTGCTGGCGGCGCTGGCCGCCAAGGGGCTGGGGGAGACCGCTGAAGAGGGTCCCGTCGTCGTCACGGTCATGGACCGTGAGCGCATGGCCGATTACCAGTCGATGGTCGCCGAGCTGCGCAATGCCGGCATCCGTGCCGAGGTCTACCTTGGCAACCCCAAGAACTTCGGCAACCAGTTGAAATATGCCGACCGGCGGGGCGCCCCCGTCGCCGTCATCCAGGGCGGGGACGAGGCCGCCCGCGGCGTGGTGCAGATCAAGGACCTGAAGCTGGGCGCCGAGATCGCCCAAAGCGCCAGCCTGGAGGAGTGGAAGGCCCAGCCCGCCCAGTCCGAGGTGCCCCGCACCGATCTGGTCGCCGCCGTGCGCGGCATTCTGGCCCGGACCGCCTGAATGGATGAACTGAGGCAGAAATACCTTGCCGCGACCGCCGATGCCGCCGATGAGGCAGCACTTGAGGCGATCCGCGTCCAGGCGCTGGGCAAGAAGGGCGAGATCAGCCTGAAGATGCGCGAGCTGGGCAAGATGACCCCGGAAGAGCGCACGACCGCCGGCCCCGCCCTCAACGCCCTCAAGGACGAGATCAACTCGGCCCTGGCCGCCAAGCGCGCCGCCCTGGGCGATGCCGCGCTTGAGGCCCGTCTGCGCGACGAGTGGCTGGACGTGACCCTGCCCGGGCGCCCGCGCCGCACAGGCACGATCCACCCGATCAGCCAGGTCACCGAAGAGGTTACCGCGATCTTCGCCGACATGGGCTTCTCGGTCGCCGAGGGTCCCCAGATCGAAAGCGACTGGTTCAATTTCGACGCGCTGAACATCCCCGGCCACCACCCGGCCCGCGCCGAGATGGATACGTTCTACATGGCCCGCGCCGAAGGGGACGATCGTCCGCCCCACGTCCTGCGCACCCACACCAGCCCGGTCCAGATCCGCACGATGCAGGAGCGTGGCGCGCCCCTGCGGATCATCGCGCCCGGCCGCGTCTATCGCTGCGACTACGACCAGACCCACACGCCGATGTTCCACCAGGTCGAGGGGCTTGCCCTGGACAAGGATCTGTCGATGGCGAACCTGAAATGGGTGCTGGAGGAGTTCGTCCGCGCCTTCTTCGAAGTTGACGAGGTCGAGCTTCGGTTCCGCGCCAGCCATTTCCCGTTCACCGAACCCTCTGCCGAGGTCGACATCCGCTGTTCCTGGGAGGGTGGTCAGCTTCGCGTAGGCGAGGGTGACGACTGGCTTGAGATCCTGGGCAGCGGCATGGTCCACCCCAAGGTCCTGGCCGCCGGCGGCATCGACGCCGACACCTGGCAGGGCTTTGCGTTCGGCATGGGGATCGACCGGCTGGCAATGCTGAAATACGGCATCCCCGACCTGCGGGCCTTTTTCGACAGCGATCTGCGCTGGCTACGCCACTATGGCTTTGCCTCGCTGGACCAGCCGACCCTGCGCGGCGGCCTCAGCCGCTGAATGGAGCTGGCCGAGGACGCCGGGCTGGATCTGGTCGAGATTTCCCCCAACGCCGCGCCGCCGGTGTGCAAGATCATGGATTTCGGCAAGTTCAAATACGAACAGCAGAAGCGCGAATCCGAAGCGCGCAAGAAGCAGAAGACCATCGAGGTGAAGGAGGTGAAGTTCCGCCCCAACACCGATACGCACGACTATGAAGTCAAGATGCGCAACGTCTTCCGCTTCCTCGAGGCCGGCGACAAGGTGAAAATCACCCTGCGCTTCCGTGGCCGCGAGATGGCGCACCAGAACCTGGGCCGCGAGCTGCTGGAGCGGGTTGCCGAAGACGTCAAGGAACTGGGCAAGGTCGAGAACATGCCCAAGATGGAAGGTCGTCAGATGATCATGATGATCGGCCCCATCGCCAAGTAAATGAAATTCACGGTATCCTGGCTTGGGGATCATCTGGAAACCGACGCCTCGGTCGACGAGATCTGCGACACGCTGACCGACTTGGGGCTTGAGGTCGAAGGGGTCGAGAACCCCGCCGACACGCTGGGCGCCTTCCGCATCTGCCGCGTGATCGAGGCCGGACCCCATCCCGATGCCGACCGTCTGCGCCTGTGCCGGGTCGAAACCCCCCAGGGCGAGGTGCAGGTTGTCTGCGGGGCGCCCAACGCGCGCACCGGGCTGGTGGGCGTCTTCGCCCCCGTCGGCACCCATGTCCCCGGCACCGGCGTCGATCTGAAGCCCGGCGTCATCCGGGGCGTGGAATCGAACGGCATGCTGTGTTCCGAGCGCGAGCTGATGCTGTCGGACAACCATGATGGCATCATCGACCTGCCCGAGGCGCCGCTGGGCGAAAGGTTCATCGACTACCGGGGCGTCAAC---------------GACCCGGTGATCGAGATTGCGATCACCCCCAACCGCCCCGACGCGCTGGGCGTTCGCGGGATCGCCCGCGACCTGGCCGCCCGCGGCCTCGGCCGGCTGAAGGACGCGCCCGCCGTCAGCGTCGCTTCGGGCGGTCCCAGCCCGATCAACGTCTCCATCGATGACGACGTGCTGGACGGTTGCCCGGTGTTCTGCGGCCGGATGATCCGGGGCGTGCGCAACGGCCCCAGCCCCGAGTGGATGCAAAAGCGCCTGCGCGCCATCGGGCTGCGGCCGATCTCGGCGCTGGTCGATGTGACCAACTTCCTGACCTATGACCGCAACCGCCCCCTGCACGTCTTCGATGTCGACAAGGTTCAGGGGGACCTGCGGGTTCATTACGCCAAGGGCGGCGAGCGGCTGGTCGCGCTGGACGACAAGGAATACGAACTGGGCGCCGGGATGATGGTGATCTCGGACGACCAGGGCGTCGAGAGCATCGCAGGCATCATGGGCGGCGCGCCCACCGGCTGCACCGAGGAGACGGTCAACGTCTTCGTCGAAAGCGCCTACTGGAACCCGGTGGCGATCGCCCACGCAGGCCGCGCGCTCAAGATCAACTCGGATGCGCGTTACCGTTTTGAACGGGGGATCGACCCTGCCTTCACGCCCGAGGGGCTGGATATCGCGACCGCGCTGATCCTCGAGATCTGCGGCGGCGAGGCTTCGGAGGTCGTCTGCGCCGGTGCCGTGCCCGACACCGACCGCGCCTACCGGCTGGACACCGACCGGGTGCAGTCGCTGGTCGGCATGGACATTCCCGCCGACACCCAGCGCCAGACCCTGACCGCCCTCGGCTTCCGCCTCGAGGGGGACATGGCCCATGTTCCCAGCTGGCGCCCCGATGTGATGGGACAGGCCGACCTGGTCGAGGAAGTGGCGCGCATCGCCTCGCTGACCCGCCTTGAGGGGCGGCCGATGCCGCGCGTG---CCGGGCGTTCCGCGCCCCATCCTCACCCCGCTGCAGCGTCGCGAATCCGCGGCCCGTCGCGCCGGCGCGGCGCTGGGTTACAACGAATGCGTCACCTACAGCTTCATCGACCAGCCGACAGCGGCGATGTTCGGCGGCGGCGACTTTGCCACCATGCTGGAAAACCCGATCTCCAGCGACATGAGCCACATGCGCCCCTCGCTGCTGCCGGGGCTGCTTCAGGCCGCTGCGCGCAACCAGGCCCGGGGTTTCACCGACCTGGCCCTGTTCGAGGTCGGCCCCGTCTTCGAGGGCGGTGAGCCCGAGGATCAGCGCTTGCAGATGTCAGGCCTGCTGGTCGGTCACAACTCGCCGCGCGAACCCCATGGCGGCCGCCGCCCGGTCGACATCTACGACGCCAAGGCCGACGCCGAGGCCGTGCTGGCCGAGCTGGGCGCACCCGCCAAGGTTCAGATCGCCCGCGGTGCCGAGGAATGGTGGCATCCCGGCCGCCACGGCGTGATGCGTCTGGGACCCAAAAAGGTCCTGGCCGTCTTCGGCGAGCTGCATCCCAAGGTGCTGAACGCGCTGGACATCAAGGGCCCGGCGGTGGCTTTCACCATCTGGCCGTCCGAGGTGCCGGTGCCCAAAGCCAAGGGCGCGACCCGCCCGGCGCTGGAGATGCGCGACCTTCAGGCGGTCGAGCGTGACTTCGCCTTCGTCGTCGACGAGGGGGTCGAGGCGCTGGGCCTCGTCAACGCCGCGGCCGGCGCCAACAAGGCCCTGATCGAGGATGTCCGCGTCTTCGACGAGTTCACCGGCACCCAGATGGGCGAGGGCAAGAAATCCCTGGCGATCACCGTCCGGCTTCAACCCCGCGACAAGACCCTGACCGAGGCCGATATCGAGGCCGTCAGCGCCAAGATTGTGGAGAAGGTCGCGAAGGCCACGGGCGGCACGCTGCGCGGCTGAATGCACGCTTTCCGCACCCATACCTGCGCCGATCTCGACAAATCCAACGTCGGCGATACCGTCCGCCTGTCGGGCTGGGTCCACCGGGTGCGCGACCACGGCGGCATCCTGTTCATCGACCTGCGTGACCATTACGGCGTGACCCAGGTGCTGGCCGACCCCGACAGCCCCGTTTTCGCCGATGTCGAGAAGGTGCGCAGCGAATGGTGCATCCGCATCGACGGCACCGTGAAGGCCCGCGACCCCGAGCTGGTGAACCCCAAGATCCCCACCGGCGAGGTCGAGGTCTTCGTACGCGACATCGAGGTTCTGGGCGAGGCTGCGGAACTGCCGCTGATGGTCTTCGGCGATCAGGAATACCCCGAAGAGACGCGTCTGCGCTATCGCTTCCTGGACCTGCGCCGCGAGAAGCTTCAGCGCAACATGACCCTGCGCTCGGACGTGGTGGCCTCGATCCGCAAGCGGATGTGGGGTGCGGATTTCCGCGAGTATCAGACGCCGATCATCACCGCGTCCTCTCCCGAGGGCGCGCGCGACTTTCTGGTGCCCTCGCGCCTGCATCCCGGCAAGTTCTACGCGCTGCCCCAGGCACCGCAGCAGTTCAAACAGCTGATCATGGTCTCGGGCTTCGACAAGTATTTCCAGATCGCCCCCTGTTTCCGCGATGAAGATCCGCGCGCCGACCGTTCGCCCACCGATTTCTACCAGCTCGACATGGAGATGTCGTTCGTCACCCAGCAGGACGTGTTCGACACGATCCAGCCCGTGGTGCAGGGCATCTTCGAGGAATTCGGCGGCGGCCGCAAAGTCGACACCACGTGGGAGCAGATCAGCTATCGCGACGCGGCCATGTGGTATGGCACCGACAAGCCCGACCTGCGCAACCCGATCAAGATGCAGGACGTGTCCGAGCATTTCCGCGGCTCGGGCTTTGCCATCTTCGCCAAACTGCTGGAACAGGACGGCACCCAGATCCGCGCCATCCCCGCCCCCAAGGGCGGCAGCCGCAAGTTCTGCGACCGCATGAACGCCTTCGCCCAGAAAGAGGGTCTGCCCGGCATGGGCTACATCTTCTGGCGCGAGACC---------GACGGCCAGATGGAGGCCGCCGGCCCCCTCGCCAAGAACATCGGCCCCGAGCGGACCGAGGCCATCCGCCAGCAACTTGACCTGGGCGTGGGCGACGCGGCCTTCTTCCTTGGCGGCAAGCCGTCGGCCTTCGAGACCGTCGCCGGCAAGGCCCGCACCGAGATCGGCAACGAGCTTGGGCTCACCGACACCGACCGCTTTGCATTCGCCTGGATCGTCGACTTCCCGATGTACGAGAAGGACGAGGAGACCGGCGCGATCGACTTCTCGCACAACCCCTTCTCGATGCCCCAGGGCGGGCTGGAGGCGCTGCAGGGCGATCCGCTGGAGGTCCTTGGCTACCAGTATGACCTTGCCTGCAACGGCTACGAGCTGATCTCGGGCGCGATCCGGAACCACAAGCTGGACATCATGTACAAGGCGTTCGAGCTGGCGGGCTATGGCCCCGAAGAGGTCGACAAGCGCTTCGGCGGCATGGTCAAGGCGTTCCGCTACGGCGCACCGCCCCACGGCGGTTGCGCGGCCGGCATCGACCGGATCGTGATGCTGCTGGCCGACGAGGCCAACATCCGCGAGGTCATCATGTTCCCGATGAACCAGCGCGCCGAGGACCTGATGATGGAGGCTCCCTCGGAGCCCACCAACGAGCAGCTACGCGAATTGCGCCTGCGGGTCATCCCCGCCGAGTGAATGACCGATCTGTCCCATATCCGCAATTTTTCGATCGTGGCCCATATCGACCACGGCAAATCCACCTTGGCCGACCGGCTGATCCAGTCCACTGGCACCGTCCAGGACAGGGACATGAAGGAACAGTTGCTGGACGCGATGGATATCGAGCGCGAGCGCGGGATCACCATCAAGGCCAACACGGTCCGCATCGACTATGTTGCCGACGACGGGCGCAAGTATGTGCTGAACCTGATCGACACGCCCGGCCACGTCGACTTCGCCTATGAGGTCAGCCGTTCGATGCGCGCGGTCGAAGGATCGTTGCTGGTCGTCGACAGCACCCAGGGTGTCGAGGCGCAGACCCTCGCCAACGTCTATCAGGCCATCGACGCCGACCACGAGATCGTGCCCGTCCTCAACAAGATCGACCTGCCGGCCGCCGATTGCGAGCGTGTGGCCGAGCAGATCGAGGATGTGATCGGCATCGACGCTTCGGGCGCCATTCAGGTCAGCGCCAAGACCGGCATCGGCATCCACGAGACGCTGGAGGCCATCGTCAAGCTGCTGCCGCCGCCTCAGGGTGAGCGGGACGCGCCGCTGAAGGCCATGCTGGTCGACAGCTGGTACGATGCCTATCTGGGCGTAATCGTTCTGGTGCGCATCATCGACGGCGTGCTGAAAAAGGGCGACCGGATCCGCATGATGCACAACGACTCGATCCACCATGTGGACCGGATCGGCGTCTTCCGCCCGCCGATGCAGACCGTGGACGAGCTGGGCCCCGGCGAGATCGGCTTTCTGACCGCCTCGATCAAGCAGGTGCGCGACACCCGCGTCGGCGACACCATCACCCACGAGAAGAAGGGTGCCGACAAGGCGCTGCCGGGCTTCAAGCCCAGCCAGCCGGTGGTCTTCTGCGGCCTCTTCCCCGTCGACAACGCCGAGTTCGAGGACCTGCGCGACGCGATCGAGAAGCTGGCGCTGAACGACGCCTCCTTCAGCTTCGAGATGGAGACCTCGGCGGCGCTGGGCTTCGGCTTCCGCTGCGGCTTTCTGGGGCTTTTGCACCTTGAGGTGATCCGCGACCGGATCGAGCGGGAATATGACATCGACCTCATCACCACCGCGCCCAGCGTTATCTACAACGTCCACATGCGCGACGGCGAGATGATCGAGCTTCACAACCCCGCCGACATGCCCGACCTGACCCACGTGGACCATATCGAGGAGCCGCGGATCAAGGCGACCATCCTTGTCCCGGACGAGTTTCTGGGCGACGTGCTGAAGCTGTGTCAGGACCGGCGCGGCATCCAGATGGACCTGACCTATGCCGGCAGCCGGGCGATGGTGGTCTATGACCTGCCGCTGAACGAGGTGGTGTTCGACTTCTACGACCGGCTGAAGTCGGTGACGAAGGGCTATGCCAGCTTCGATTATCAGATGATCGGCTACCGTCAGGACCACCTGGTGAAGATGCAGATCCTGGTCAATGACGAGCCTGTGGACGCGCTTTCCACCATGGTTCACCGCGACCGGGCCGAGACCCGGGGCCGCGCCATGGTCGAGAAGCTGAAGGACCTGATCCCGCGCCACATGTTCAAGATCCCGATCCAGGCCGCCATCGGCGGCCGCATCATCGCCCGCGAGACCCTGGCCGCCCTGCGCAAGGACGTGACCGCCAAATGCTATGGCGGGGACGCGACGCGCAAGAAGAAGCTGTTGGAGAAGCAGAAGGCGGGCAAAAAGAAGATGCGCCAGTTCGGCAAGGTCGACATCCCGCAGGAAGCGTTCATTAATGCGCTGAAGATGGATGGGTGAATGAGTCTGCCCCCCGGTTTCATCGACGAGCTGCGCACGCGGCTCAGCCTCTCCCAGGTCGTCGGGCGCAAGGTCATGTGGGACCCGCGCAAGTCGAACCAGGGCAAGGGCGATTTCTGGGCGCCCTGTCCCTTCCACCAGGAAAAGAGCGCCTCGTTCCACGTCGATGATCGCAAGGGCTTCTATTACTGCTTCGGCTGCCAGGCCAAGGGCGACGCCATCTCCTTCGTGCGCGAGACCGAGAACGTGGGCTTCATGGAGGCGATCCGCATCCTGGCGCAGGAAGCCGGCCTGCCGGTTCCCCAAAGCGACCCGCGTGCCCAGGCCCGTACCGACCGGCGCTCGGCTCTGGCCGAGGTCATGGAACAGGCGGTGCGCTTCTTCACGATGCAGCTTGGCACCGCGGCGGGAGGGGCGGCGCGGCAATATCTTGATGGGCGCGGCCTCGACGAGGGGGCGCGCAAGCGCTTTGCCATCGGCTACGCCCCCGACGCGCGCCAGGCGCTCTGGTCGCACCTGACCGGAGCGGGGGTGGCGCCCGACATGATCGTCGACGCGGGCCTCGCGGCGCGGCCCGACGATGGTGGCGCCCCCTACGACCGCTTCCGCGGGCGCATCATCTTCCCCATCCGCGACGGGCGCGGGCGGACCATCGCGCTGGGCGGGCGCGCCATGAGCGCCAATGCCCGCGCCAAGTATCTCAACTCCCCCGAGACCGAGCTTTTCGACAAGGGGCGCAGCCTCTACAACCTCGGCCCTGCCCGGGAGGCCGCCGGAAAGCGTGGCACCCTGATCGTGGCCGAGGGCTATATGGACGTGATTGCCCTTGTTCAGGCCGGGTTCGAGGCCACCGTCGCCCCCCTGGGCACCGCGATCACCGAGAACCAGCTTCGGATGCTGTGGCAGATCTCGCCCGAGCCGGTGATCGCCCTCGACGGCGATGCCGCCGGCCTGCGCGCGGCCCAACGACTGATCGACCTGGCGCTGCCCCTGCAGGAGGCTGGCCAATCCCTGCGCTTTGCCATCCTGCCCGAGGGCAAGGACCCTGACGATCTGATCCGTGCCGAGGGGGCGCAGGCCATGCAGCGCGTGGTCGATGGCGCGGTGCCCATGGTGCGCCTGCTCTGGCAGCGCGAGACCGAGGGCCGCGTCTTCGACAGCCCCGAGCGCAAGGCCGCTCTCGACAAGTCCCTGCGCGACATCCTGCGCCGGATCCGCGACCGCTCGATCCGCGGCCATTACGCCGACGAGATCAAGCGCCTGCGCTGGGATCTTTTCGATCCCAACCGCGGCTCGGGCCAGGGTTTGGGCCAGGGCGGGGGGCGCGGCGGCAACCGCCGGTGGGGCGACGCGCCTGCCCAGCCCCTTGATTCCACCCGCCGATCGCTTCTCGCCATG---GCCGAGCCGGGGGTCGAGGAGCACCTTCGCGAGGCGGTGATCCTGGCCGCGCTGGTCGTGCATCCCGCCATCCTGCGCGATTTCGAGGCCGAGCTGGAAACGCTGGAACTGGCC---CCCGCCCACGAGCCCGTGCGCGCGGCGCTGCTGCGTCACGGCTTTGCCGATCTTGCCGAGCTGCCCGAGCGGATCGCCACCGACGCCGGC---GGCGCCCTTGAAAAGCTGTTCGCCCTGAGCCATGTCCAGATCTCCCCCCCGTTCAGGCACCGGGACGACGCCGAGCTCGCGGCGCAGACCGTGCGCCAGGAACTGTCGAAACTGGCCTCGCGCCGGGGGGTGCAAAGGGAGATAGACGAGGCGTCGCAGGACCTCGAGGGGCTGGCCGACGAGGGGCTGACATGGCGCCTCGGACAGGCCGCCGAGGCCCGCGACAGGGCCGAAAGATGGGGCGTGGACGACAGT------CCTGATATGGGCGAAGATCGCGACGCCCTGTCGAAG------------ATGCTGCAAGACCTCATCGACGGTGAGGTTTGGGTAAAGAAAACAAGG------TAAATGTGCGCCGACGCCCCCGATTACAAGACCACCCTGAACCTTCCCCAGACCGAATTTCCCATGCGCGCGGGCCTGCCCAAGCGCGAACCCGGCTGGCTGGAGCGGTGGGCGCGGATCGGTGTCTACGAGCGGCTGCGCGACAAGGAGACCCGTCAGCCCTTCACCCTGCATGACGGCCCTCCCTACGCCAACGGGCACCTGCACATCGGTCACGCGCTGAACAAGATCCTCAAGGACATGGTGATCCGCAGCCAGCAGATGTCGGGCCGGGACGCGCGTTACATCCCCGGCTGGGACTGCCACGGCCTGCCGATCGAATGGAAGATCGAAGAGAAGTATCGCCAGAAGGGTCTGGACAAGGACGCCGTGCCGATCGTCGATTTCCGTCAGGAATGCCGCAGCTTCGCCGAAGGCTGGGTCGACATCCAGCGCGACGAGTTCAAGCGCATCGGCATCACCGGCAACTGGGACAACCCCTACCTCACGATGGATTTCCACGCCGAGCGTGTCATCGCCGAGGAGTTCCAGAAATTCCTGATGAACGGCACCCTCTATCGTGGCTCCAAGCCCGTGATGTGGAGCCCGGTGGAAAAGACCGCCCTTGCCGAGGCCGAGGTCGAATACCACGACCACAAGAGCCACACGATCTGGGTGCCCTTCAAGATCCGCGAAGGC------GCGCCCGCCGACATGGCCGACGCCCGCGTCGTGATCTGGACCACCACGCCCTGGACCATCCCCTCGAACAAGGCCGTCGCCTTCAACCCCAAGATCGCCTACGGCCTCTACCGGGTCGACGGGACCGAGGAGGAAAGCTGGACGGCGCAAGGCGACCTTTACCTGCTGGCCGACAAGCTGGCAGATGAGGTGCTGTCCCGCGCCCGCGTGACCGCCAGC------ACCCGCCTGCGCGATGTCAGCGCCGACGAGCTGTCGGGCCTCACCCTCATCCACCCCTTCAACGGGGTCGAGGGTGCCGAGGGCTTCTGGGATTATGACGTGCCGATGATCGACGGCGATCATGTCACCGACGATGCCGGAACCGGCTTCGTGCATACCGCCCCCAGCCACGGCGCAGACGATTTCGAATGTTTCGTCCGCCGCAACTGGCTGGACCGG---ATGACCTACAACGTGGGCGAGGAAAGCGAATTCCTGGACCATGTGCCGTTCTTCGCCGGGCTTCAGGTCTTTGACCGCAAGGGCAAGGAAGGCAAGGCCAACGCCGCCGTCATCGACAAGCTGGTGGCCGCCAGCGGGCTGATCGCGCGTGGCCGCGTCACCCACAGCTATCCCCATTCCTGGCGCTCCAAGGCGCCGGTGATCTTCCGCAACACGCCCCAATGGTTCGCCGCCATCGACCGCGCCGTCGGCGACGGCCAGGACGAGTATGGCACCACCATCCGCGAACGGGCGCTGACCTCGATCGACAAGGTGACATGGACGCCCAAGACCGGGCGCAACCGGCTCTACTCGATGATCGAGGCGCGGCCCGACTGGGTCCTGTCGCGCCAGCGTGCCTGGGGCGTGCCCCTGACCTGCTTCGTCAAGAAGGACACCCAGCCCACCGATCCCGACTTTCTGCTGCGCGATCCCGTCGTCAACGCCCGCATCACCGAGGCCTTCGAGGCCGAGGGCGCCGACGCCTGGTACAAGGACGGCGCCAAGGCCCGCTTCCTCGGCGACGACCACGACCACGACGCCTACGAGCAGGTGTTCGACATCCTCGACGTGTGGTTCGACTCGGGCTCCACCCACGCCTTTGTTCTGCGCGACCGCGAGGACGGGTCCGAGGACGGTTTGGCCGATCTCTACCTCGAGGGCACGGACCAGCACCGCGGCTGGTTCCACTCGTCGATGCTGCAGGCCTGCGGCACCCGTGGCCGCGCCCCCTATCGCGGCGTGCTGACCCACGGCTTCACCCTCGACGGCAAGGGCAACAAGATGTCCAAGTCGCTGGGCAACACCATCGCCCCCGAGGCGGTAATCAAGCAATACGGCGCCGACATCCTGCGGCTTTGGGTGGCCCAGTCGGACTATACCGCCGACCTGCGCATCGGCGACGAGATCCTGAAGGGTGTCGCCGACAGCTACCGCCGCCTGCGCAACACCATGCGCTTCATGCTGGGCAGCCTGTCCGGCTTCTCCGAGGCCGATCGCATCGCCCCCGAGGACATGCCCGAGCTTGAACGCTGGGTGCTGCACCGTCTGGCGGAGCTCGACCACCGGGTGCGCACGGGCTACGCCGCCTATGACTTCCAGGGCGTGTTTCAGGCGCTGTTCAACTTCGCCACGGTGGATCTTTCGTCCTTCTACTTCGACATCCGCAAGGACGTGCTCTACTGCGACGGGGACACGGTGGAACGCCGCGCTGCGCGCAGCGTGCTCGACATCCTCTATCACCGGCTGACCACATGGCTGGCGCCGATCCTGGTCTTCACGATGGAGGAGGTCTGGCTGGAGCGGTTCCCCGGTGACGCAAGCTCCGTCCACCTTCAGGACATCCCCGCCACCCCCGGCGACTGGCGCGACGATCCGCTGGCCGCCAAATGGGCCGGCGTGCGGCGGGTGCGCCGGGTCGTGACCGCCGCCCTCGAGGAACAGCGCCGCGACAAGGTCATCGGCGCCTCGCTCGAAGCCGCGCCCGTGGTCCATGTCACCGATCCGGCGGTGCTGGCCCAGCTGCGCACGCTGCCCTTCGCCGACATCTGCATCACCTCGGCGGTGGAACTGACGGGCGATCCCATCCCCGACGAGGCCTTCCGCCTGCCCGAGATCGACGGCGTCGGCGTGGTCTTCGAACAGGCCGAGGGCGAAAAGTGCCAGCGTTGCTGGAAGATCCTGCCCGACGTGGGCCACCACGCCCACGCGCAGGTCTGCGGCCGCTGCGACGCGGCCCTCAAGTGAGCCATTCAAAGCCAGAACATCCGTATTCGCCTGAAGGCGTTTGACTACCGGGTGCTGGATTCCTCGACCCAGGAAATCGTAAATACGGCCAAGCGGACCGGCGCGCAGGTGCGCGGACCGATCCCGCTGCCGAACAAGATCGAGAAGTTCACCGTTCTGCGTGGACCGCACATCGACAAGAAATCCCGTGATCAGTTCGAGATCCGCACGCACAAGCGCCTGCTCGACATCATCGATCCGACCCCCCAGACCGTGGACGCGCTGATGAAGCTCGACCTGGCTGCCGGTGTCGACGTCGAGATCAAGGTT------TAAATGGCTGAAGATATCAAAACTCTGGACGGCCTGCGCGACGCCGTCACCGGTGGCGTTCAGGGCACC---------------GCCGACATGATCAACCGCGAGCCCCAGCGCGACGAGCTGGGCCGTTCCTACGCCACCGGCAAGCGCAAGGACGCGGTCGCCCGCGTCTGGATCAAGCCCGGTTCGGGCAAGGTCACCGTCAACGGCAAGCCGATCAACGCGTATTTCGCGCGCCCCGTGCTTCAGATGATCCTCAAGCAGCCGTTCCAGGTTGCTGGCGTGGAAGGTGAATTCGACGTCACCGCGACCGTCAAGGGCGGTGGTCTTTCGGGTCAGGCCGGTGCGGTCAAGCACGGCATCTCGAAGGCGCTGCAGCTTTACGAACCCTCGCTGCGTGGCGCGCTGAAGGCCGCCGGCTTCCTGACCCGCGACAGCCGCGTGGTCGAGCGGAAGAAATACGGCCGCGCCAAGGCCCGCAAGAGCTTCCAGTTCTCCAAGCGCTGAATGCCAACGATCCAACAGCTGATCCGCAAGCCGCGGCAGCCCAAAGTCAAACGCTCGAAGTCGCTGCACCTGGAGGGTTGCCCCCAGAAGCGCGGCGTCTGCACGCGAGTCTACACGACCACACCGAAGAAGCCGAACTCGGCCATGCGGAAGGTTGCCAAGGTGCGCCTGACCAATGGCTTCGAGGTCATCAGCTACATTCCCGGTGAATCCCACAACCTTCAGGAGCACTCTGTGGTCCTGATCCGCGGCGGCCGGGTCAAAGACCTTCCGGGTGTCCGTTACCACATCCTGCGCGGTGTTCTGGATACCCAGGGCGTCAAAGACCGTAAGCAACGCCGTTCGAAATACGGCGCCAAGCGTCCGAAGTAAATGGCACGAGATCGTCGTCAAACGAAGCGCAAGGTTTCCAAGAACATCGCCACCGGCGTTGCGCATGTGAACTCTTCGTTCAACAACACCAAGATCCTCATCTCCGACGTTCAGGGCAACGCGATCGCGTGGTCCTCGGCCGGCACGATGGGTTTCAAGGGCAGCCGTAAATCCACCCCCTATGCCGCCCAGATGGCCGCAGAGGACGTGGGCAAGAAGGCCCAGGAACACGGCGTCAAGACGCTGGAAGTCGAAGTGCAGGGCCCCGGTTCGGGCCGCGAGAGCGCCCTGCGCGCTCTGGCCGCTGCCGGCTTCAACATCACGGCCATCCGTGATGTGACCCCGATCGCCCACAACGGCGTGCGCCCCCCCAAGCGGCGCCGCGTCTGAATGCGGTCG---CGCAGGGGTTTGTTGCTTATCCTGTCGTCACCATCGGGTGCCGGCAAATCCACCCTGGCCAACCGCCTGCGCAACTGGGACCCCGATATCGTCTTCTCCGTCTCGGCCACGACCCGCGCCCCCCGACCCGGGGAGCTTGACGGACGCGAGTATTATTTCCGCAGCCGCGAGTCCTTCCTGGCCATGGTCGAGGATGGTGACATGCTGGAACATGCGGAGGTTTTCGGCAATCTCTACGGCTCGCCCCAGGGTCCCGTCGAAGAGGCGATCGTCGAGGGGCGCGACGTGCTGTTCGACATCGACTGGCAGGGCGGTCAGCAGATCCGCAATTCGGCCCTGGCCGAGGATGTGGTGTCGATCTTCATCCTGCCCCCCTCGATCGCCGAGCTTGAGCGCCGCCTGCGGTCGCGCGACCAGGACAGCGATGAGGTGATCGCCGCCCGCATGCAGAAAAGCCGCGACGAGATCAGCCACTGGGCCGAATATGACTATGTCCTGGTCAACCGCGACCTCGACGAGACCGAGATGAAACTGCGCGCGATCCTGCAGGCCGAGCGTCTGCGCCGCAGCCGCCAGGTGGGGCTGGTGGATCTGGTGCGCGAACTGAACAAAGAATTCGGAGACAGG---TGAATGGCTCAATCCTACCTTGGCCAGAAACGACTCCGCAAATATTACGGCAAGATCCGCGAAGTCCTGGAGATGCCGAATCTCATCGAGGTTCAGAAATCCTCGTATGACCTGTTCCTGAAATCCGGTGATCAGACCGAGCCGATGGACGGCGAAGGCATCAAGGGTGTCTTCCAGTCGGTCTTCCCGATCAAAGATTTCAACGAGACCGCCGTGCTTGAGTTCGTCAAGTACGAGCTGGAAAAGCCGAAATACGACGTCGAGGAATGTCAGCAGCGTGACATGACCTACAGCGCACCCCTGAAGGTGACGCTGCGCCTGATCGTGTTCGATGTCGACGAGGACACCGGCGCCAAGTCGGTCAAGGACATCAAGGAGCAGGACGTGTTCATGGGCGACATGCCCCTGATGACGCCCAACGGCACCTTCGTCGTCAACGGAACCGAGCGCGTGATCGTCAGCCAGATGCACCGTTCGCCCGGTGTGTTCTTCGACCACGACAAGGGCAAGACCCACTCGTCGGGCAAGCTGCTGTTCGCCTGCCGCATCATCCCCTACCGCGGTTCCTGGCTCGACTTCGAGTTCGACGCCAAGGACATCGTGTTCGCCCGCATCGACCGTCGCCGCAAGCTGCCGGTGACGACCCTGCTCTATGCCCTCGGGCTGGACCAGGAAGGTATCATGGATGCCTATTACGATACGGTTACCTACCGTCTGGAAAAGAACAAAGGCTGGGCCACCAAGTTCTTCCCCGAGCGGATTCGCGGCACCCGCCCGACCTATGACATCGTCGACGCCGGTTCGGGCGAGGTGATCGCCGAGGCCGGCAAGAAGGTCACCCCGCGCGCGGTCAAGCAACTGATCGACAAGGGTGATGTCACCGAGATCCTGGTTCCCTACGACCAGATCGTCGGTCGCTTCGTCGCCAAGGACATCATCAACGAGGACACCGGCGCGATCTATGTCGAGGCCGGCGATGAGCTGACGCAGGAGTTCAACAAGGAAGGCGAACTCACCGGCGGCAGCCTCAAGGACCTGACCGACGCGGGCATCACCGAGATCCCGGTGCTGGACATCGACAACATCAACGTCGGTCCCTACATCCGCAACACGATGGTGGTCGACAAGAACATGGGCCGCGACACCGCGCTCATGGACATCTACCGCGTGATGCGTCCGGGCGAGCCGCCCACCGTCGAGGCCGCAAGCCAGCTGTTCGACACGCTGTTCTTCGATTCCGAGCGTTACGACCTGTCGGCCGTGGGCCGGGTCAAGATGAACATGCGTCTGGCACTGGATGCGCCCGACACCATGCGCACCCTGCGCCGCGAAGACATCATCGCCTGCATCAAGGCGCTGGTCGAACTGCGTGACGGCAAGGGCGACATCGACGACATCGACCACCTGGGCAACCGTCGCGTCCGTTCCGTCGGCGAGCTGATGGAAAACCAGTATCGCGTCGGCCTGCTGCGGATGGAGCGTGCCATCAAGGAGCGCATGTCCTCGGTCGAGATCGACACCGTGATGCCGCAGGACCTGATCAACGCCAAGCCCGCCGCGGCTGCGGTGCGCGAGTTCTTCGGCTCCAGCCAGCTGTCGCAGTTCATGGACCAGACCAACCCGCTGTCCGAAGTGACGCACAAGCGTCGCCTGTCGGCGCTTGGGCCGGGCGGTCTGACCCGCGAGCGCGCCGGCTTCGAGGTGCGCGACGTGCACCCGACCCACTATGGCCGGATGTGCCCGATCGAAACGCCGGAAGGCCCGAACATCGGTCTGATCAACAGCCTCGCCACCTTCGCCCGTGTCAACAAGTACGGCTTCATCGAGACGCCTTATCGCAAGGTCGAGAACGGCAAGGTCACCGATGACGTGAGCTACATGTCGGCGACCGAAGAGATGCGTCACACCGTGGCCCAGGCCAACGCCAAGCTCGACGAGCAGGGCCGTTTCGTGAACGACCTGGTCTCGACCCGTCAGGCCGGCGAATACATGCTCCAGCCCAACGAGAACGTGGACCTCATCGACGTCAGCCCCAAGCAGCTGGTGTCGGTCGCGGCCTCGCTGATCCCGTTCCTGGAAAACGACGACGCCAACCGCGCCCTCATGGGCTCGAACATGCAGCGTCAGGCGGTTCCCCTGCTTCAGGCCGAGGCACCCCTGGTGGGCACCGGCATCGAAGAGATCGTGGCCCGCGACTCGGGCGCTGCGATCATGGCCCGTCGCGGCGGCATCATCGACCAGGTGGACAGCACCCGTATCGTTGTGCGCGCCACCCAGGATCTGGAGCCGGGCGATCCCGGCGTGGACATCTACCGTCTGCGCAAGTTCCAGCGTTCGAACCAGAACACCTGCATCAACCAGCGTCCGCTGGTGAAGGTGGGCGACACGGTCGGCAAGAACGAGGTCATCGCCGATGGCCCCTCGACCGATCTGGGCGAACTGGCCCTCGGCAAGAACGTGGTCGTCGCGTTCATGCCCTGGAACGGCTACAACTACGAGGACTCGATCCTGATCTCCGAGCGGATCGTGCGCGATGACGTCTTCACCTCGATCCACATCGAGGAGTTCGAGGTCGCCGCCCGCGACACCAAGCTCGGGCCGGAAGAGATCACCCGCGACATCCCCAACGTCGGCGAGGAGGCCCTGCGCAACCTCGACGAAGCGGGCATCGTCTACATCGGTGCCGACGTCGGTCCGGGCGACATCCTTGTGGGCAAGATCACCCCCAAGGGCGAAAGCCCCATGACCCCCGAGGAAAAGCTGCTTCGCGCCATCTTCGGCGAGAAGGCTTCGGACGTGCGCGACACTTCGCTGCGCCTGCCGCCGGGTGACTACGGCACGATCGTCGAGGTCCGCGTCTTCAACCGTCACGGTGTCGACAAGGACGAGCGCGCCCTGCAGATCGAGCGCGAGGAAGTCGAGCGTCTGGCCCGTGACCGGGACGACGAGCTGGCGATCCTGGATCGCAACATCTACGCCCGTCTGAAAACCCAGATCCTCGGCAAGACCGCGGTCAAGGGCCCCAAGGGCGTCAAGCCCAACTCGGAGATCACCGAGGAGCTTCTGGAAACGCTCAGCCGCGGCCTGTGGTGGCAGCTGGCGCTGAAGGAAGAGGCCGACGCAAGCATCGTCGAGGCCCTGAACGAGCAGTATCAGGCGCAGAAGCTGTCGCTGGATGCCCGTTTCGAGGACAAGGTCGAGAAGGTTCGCCGCGGCGACGACCTGCCCCCCGGCGTGATGAAGATGGTCAAGGTCTTCGTGGCCGTGAAGCGCAAGCTTCAGCCCGGCGACAAGATGGCCGGCCGTCACGGCAACAAGGGTGTCATCTCGAAGGTCGTTCCGATGGAGGATATGCCCTTCCTCGGTGACGGCACGCCTGTCGACTTCGTGCTGAACCCGCTGGGCGTTCCCTCGCGGATGAACGTCGGTCAGATCCTCGAGACCCACATGGGTTGGGCCGCACGCGGTCTGGGCGAGCAGATTGGCGAAGCCCTGGGTGAATACCGCCGTTCCGGCGACATGACCCCGGTGCGCGACGCGATGAAGATCGCCTATGGCGACGATGTCTACGACGAGGGCATCGCCGACATGGACGAGACCCGTCTGGTCGAGGCCGCCGACAACGTGGTGCGTGGTGTTCCCATCGCGACCCCCGTCTTCGACGGTGCCAAGGAAGCCGATGTCAACGACGCCCTTCAGCGGGCCGGGTTCGACATGTCGGGCCAGTCGGTCCTGTTCGATGGCCGCACCGGCGAGCAATTCGCCCGGCAGGTCACAGTGGGCGTCAAGTACCTGCTGAAACTGCACCACCTGGTGGACGACAAGATCCACGCCCGTTCGACCGGCCCCTACAGCCTGGTTACCCAGCAGCCGCTGGGTGGTAAGGCCCAGTTCGGCGGTCAGCGCTTCGGTGAGATGGAGGTCTGGGCCCTGGAAGCCTATGGCGCCGCCTACACCCTGCAGGAGATGCTGACGGTGAAGTCGGACGACGTCGCAGGCCGCACCAAGGTCTACGAGAGCATCGTCAAGGGCGAGGACAACTTCGAGGCCGGCGTGCCGGAATCGTTCAACGTTCTGGTCAAGGAGGTCCGGGGTCTGGGCCTCAACATGGAACTCCTGGACGCGGAGGGCGACGACTAAATGCCGCTTTACGAGCATGTGTTCATCTCGCGTCAGGACCTGTCGAACACGCAGGCCGAAGGCCTCGTCGAACATTTCGGCACCGTCCTCTCGGACAACGGTGGCAAGGTCATCGAAAGCGAGTACTGGGGCGTCAAGACGATGTCCTACAAGATCAACAAGAACCGCAAGGGCCACTACGCGTTCCTGCGGACCGACGCCCCCGCCCCTGCCGTGCAGGAAATGGAGCGCCTGATGCGCCTGCATGACGACGTGATGCGCGTTCTGACCATCAAGGTCGACGAGCACGCCGAAGGTCCCTCGGTGCAGATGCAAAAGCGCGACGAG------------CGCGAACGTCGCTAAGGCGCCGCCGACACGCCGGTCGAGGCGCTGGACGAGAAGACCGCCCGCGCCGAGCTGGCCCGCCTTGCCGCGCGCATCGCAGAGGCCGACCGCGCCTATCACACCGACGACGCCCCCGTCATCGACGACGCGACCTACGACGCCCTGCGCCGCCGCAACGAAGCGATCGAGGCGCGCTTTCCGGCGCTGAAACGCCCCGACAGCCCATCGGAAAAGGTCGGCGGCACCATCGCCGAGGGGTTTTCCAAGGTCGAGCATGAGCAGCGGATGCTGTCCCTCTCGAACGCCTTCGAGGACGAGGAGATCACCGAGTTCGTCGAGCGTGTCCGCAAGTACCTGGGCCTTGCCCGCGACGCCGAGCTTGCCTTCACGGCCGAACCGAAGATCGACGGCCTGTCCCTGTCGCTGCGCTATGAAAAGGGCGTTCTGGTGACCGCCGCCACCCGCGGCGACGGGTCGGTGGGCGAGAATGTCACCGCCAACGCCCGCACCATCCAGGGCCTGCCCCAGAAGCTGTCCGGGGCGCCCGATCTGCTGGAAGTGCGCGGCGAGGTCTACATGCGCCACGACGATTTCGCCGAGCTGAACCGCACCCAGCTTGCCCAGGGCAAGAAACCCTTTGCCAATCCGCGCAACGCCGCCGCCGGATCCCTGCGCCAGCTAGACGCCGAGATCACCCGCGCCCGGCCGCTGGCTTTCTTCGCCTATGGCTGGGGCAGCCTTTCGGAGCCTTTGGCCGACACCCAGATGGGCGCCATCGAGCGGCTTGCCGCGCTGGGGTTCGAGACCAACCCCCTGACCCGCAGGCTGTCCACGCCCGAGGATCTGCTGGCCCATTATCACCGGATCGAGGAGGACCGCGCGACCCTCGGCTACGACATCGACGGCGTCGTCTACAAGGTCGACGACCTGGAGCTTCAGCGCCGCCTTGGCTTCCGCTCGACCACGCCCCGCTGGGCGCTGGCGCACAAATTCCCGGCCGAGCTGGCCTGGACCCGGCTGGAAGGGATCGACATTCAGGTCGGGCGCACCGGCGCGCTGTCGCCCGTGGCCCGGCTTCGCCCCGTCACGGTGGGCGGCGTGGTTGTCGCCAATGCCACCCTGCACAACGAGGATTACATCGCCGGCCGCGACAGCCGCGGCGAGGAGATCCGCGAGGGCAAGGACATCCGCATCGGCGACCGGGTCCAGGTCTACCGCGCCGGCGACGTCATTCCCAAGATCGCCGATGTCGACCTGGCCGCTAGGCCCGATGGGGCCGAGCCTTACGTCTTTCCCGATACCTGTCCCGAATGCGGATCGGAGGCGATCCGCGAGGAGGGCGACGCTGTGCGGCGCTGCACCGGCGGGCTGATCTGCCCGGCCCAGGCGGTCGAGCGCCTGCGCCATTTTGTCAGCCGCGCGGCCTTCGACATCGAGGGGCTGGGCGCGCGCCAGGTGGAGGCGCTTTACCGCGACGAATGGATCGCCGAGCCGGCCGACATCTTCCGCCTGCGCGCGCGTTACGGCTCGGGGCCGCGACAGCTCAAGAACCGCGAAGGTTGGGGCGAGAAATCCGCCGCCAACCTGCTCAACGCAATCGACGAGCGGCGCCGCATCCCCCTCAACCGGCTGATCTTCGCCCTGGGCATTCGCCACGTGGGCGAGAATGCCGCCAACCTTCTGGCCCGCCACTACGGCACCTGGGAGGCCTTCGCCCAGGCGATGGATGCCGCCGCCGAAGGGGACGCGCCCGCCTGGCAGGCGCTGAACGACATCGACGGGGTGGGGGAGGTCATGGCGACCTCGCTCGTGACGACGTTGCAGCAGGACCGGGAACGCGCCTCGATCGAGCGTCTGGTCGCCGAGCTGGAGGTCGAAGCCGTCGCGGCCCCCGATCCCGGCGACAGCGTTGTCGCGGGCAAGACCGTCGTCTTCACCGGCAAGCTGGAGCGGATGACCCGGGCCGAGGCCAAGTCCCGGGCCGAGGCCTTGGGCGCCAAGGTGGCAGGCTCGGTCTCGGCGCGGACGGATTACCTGGTGGCGGGGCCTGGCGCCGGATCCAAGGCGACCAAGGCCGCCGAGCTGGGGGTCGAGGTGCTGGACGAGGACGCCTGGCTGACCCTCACGGGCGGGCTTTAGATGATCCACAAGAACTGGCAAGAGCTGATCAAACCGACCCAGCTTGACGTCAAACCCGGCAACGACCCCTCGCGCGAAGCGACCGTCGTGGCCGAACCGCTGGAGCGTGGCTTCGGCCTGACCATGGGCAACGCCCTGCGCCGGGTGCTGATGAGCTCGCTTCAGGGTGCCGCCATCACCAGCGTCCAGATCGACAACGTGCTGCACGAATTCTCGTCCATCTCTGGTGTTCGCGAAGACGTGACCGACGTGGTCCTGAACCTCAAGGGCGTCGCCGTCCGCATGGAGGTCGAAGGCCCCAAGCGTCTGTCGGTCAATGCCAAGGGCCCCGGTGTCGTCACTGCCGGTGACATCGCCGAGACCGCCGGCATCGAGATCCTGAACAAGGATCACGTGATCTGCCACCTCGACGAGGGTGCCGATCTGTTCATGGAACTGACGGTCAACACCGGCAAGGGCTATGTCAGCGCGGACAAGAACCGCCCCGAGGACGCCCCTATCGGCCTGATTCCGATCGACGCGATCTACTCGCCCGTGAAGAAGGTCAGCTATGACGTGCAGCCCACCCGCGAGGGCCAAGTGCTGGACTATGACAAGCTGACGCTCAAGCTGGTGACCGACGGCTCGATCACCCCCGACGACGCCGTGGCCTATGCCGCGCGCATCCTGCAGGACCAGCTGTCGGTCTTCGTCAACTTCGACGAGCCCGAGGCCGCCCGCAGCCAGGACGACGAGGACGATCTGGAGTTCAACCCGCTTCTGCTGAAGAAGGTGGACGAGCTGGAACTGTCGGTGCGTTCGGCAAACTGCCTGAAGAACGACAACATCGTCTACATCGGCGACCTGATCCAGAAGACCGAAGCCGAGATGCTCCGCACCCCGAACTTCGGCCGCAAGTCGCTGAACGAGATCAAGGAAGTGCTGTCCGGGATGGGTCTCCACCTGGGCATGGATATCGTGGACTGGCCGCCCGACAACATCGAGGAACTGGCCAAGAAATACGAAGACCACCTCTGAATGGCAAGAGAAGACAATCGTCGGGGAAATCGTCGCGAG---CGCGAAGAAACCCCCGAATTCGCCGATCGTCTCGTGGCGATCAACCGGGTCAGCAAGACGGTCAAGGGCGGTAAGCGCTTCGGCTTCGCTGCTCTGGTCGTCGTCGGTGACCAGCGCGGTCGCGTGGGCTTCGGCAAGGGCAAGGCCAAGGAGGTCCCCGAGGCCATCCGCAAGGCCACCGAGCAAGCCAAGCGTCAGATGGTCCGCGTACCGCTGCGCGAGGGTCGTACCCTGCACCACGACATCGAGGGCCGTCACGGCGCTGGTAAGGTCGTGATGCGGACCGCCCCCCAGGGTACCGGCATCATCGCCGGTGGTCCGATGCGCGCCGTGTTCGAGATGCTGGGCGTTCAGGACGTGGTCGCCAAGTCGATCGGGTCCCAGAACCCCTACAACATGATCCGCGCGACGCTGAACGGCCTTGGCCGCGAGGCTTCGCCCCGTTCGGTCGCGCAGCGTCGCGGCAAGAAGGTCGCGGACATCCTCAAGAAG------------------CCCGAAGCCGAAGCGGCCTCCGAGTCGTAAATGAACGATCCTATCGGCGATATGCTCACCCGGATCCGCAACGCACAGCTGCGCGGCAAGTCCACGGTTGAAACCCCCGCCTCCAAGCTTCGCGCCTGGGTGCTCGACGTGCTGGCCGACGAAGGCTACATCCGCGGCTACGAGAAGACGACCGGCAAGGACGGCCACCCGGCCCTGTCGATCAGCCTGAAGTACTACGAAGGCACCCCGGTGATCCGCGAGATCAAGCGGGTCTCGAAGCCCGGCCGTCGTGTCTACATGGGCGTCAAGGACATCCCCTCGGTCCGTCAGGGCCTGGGTGTCTCGATCGTCTCCACGCCTCGCGGCGTGATGTCGGATGCAAATGCGCGCACTGCCAATGTTGGTGGTGAAGTGCTTTGCACGGTCTTCTAAATGCACGACATCCGCGCCATCCGCGAGAACCCGGACGCTTTCGACGCCGCCCTCTCCCGCCTGGGGCTGACCAACCCCTCGGCCGAGATCCTGCAGATCGACGCCGCCCGCCGCGAGGCCATCGCCCAGGCCGAGGAGGCGCAGGCCGCGCGCAACGCGGCCTCCAAGGAGGTCGGCGCCGCCAAGGCAAGCGGCAACGAGGACGAGTTCAACCGCCTGCGCGCGCTGGTAGCCGAGAAAAAGCAGCAGATTGCCGATCTGGAAGAAAAGGCCCGCGAGGGCGACGCCAAGCTGCGCGATCTTCTGATGCGCCTGCCCAACCTGCCCCTCGACGAAGTGCCCGACGGCACCGACGAGGAGGACAACGTCGAGCTGCACCGCCGCGGCACGCCCCCCGCCTTCGACTTCAAGCCGCTGGAGCATTACCAGATCCCCGCCGCCGTGCCGGGACTGGACTTCGAGAGCGCCGCGCGGCTTTCGGGATCGCGCTTCGTGGTGCTGCGCGGGGCGATGGCGCGGGTGCACCGGGCGCTGGCGCAATTCATGCTGGACGTACATGCCACCGAAAACGGCCTGGAAGAGACCTGGACCCCCGTGCTCGTGCGCGAAGAGATGATGTATGGCACCGGCCAGCTGCCCAAGTTCGGCGAAGACAGCTACCAGACCACCAATGGCTGGTGGCTGGTGCCCACCGCCGAGGTGACGCTGACCAACACCGTCAACGGCCAGACCGTGGACGAATCCGCCCTGCCCCTGCGCCTTTGCGCCCACACTCAGTGCTTCCGCTCGGAAGCGGGCAGCGCCGGGCGCGACACCTCGGGGATGCTGCGCCAGCATCAGTTCGAGAAGGTCGAGATGGTCTCGATCACCCATCCCGACACATCCCGCGACGAGCTGGACCGCATGACCCGCTGCGCCGAGGATATCCTGGACCGGCTGGGCCTGGCCTATCGCACGGTGGTCCTGTGCACCGGCGACATGGGGTTCGGCGCCCGCCGCACCCACGACATCGAGGTCTGGCTGCCCGGTCAGGACACCTACCGCGAGATCAGCTCGATCTCGCTGGTGGGGGATTTCCAGGCCCGGCGCATGAATGCCCGCTTCCGCCCCGAGGGCGGCGGCAAGCCCGAGTTCCTGCACACGCTGAACGGCTCGGGCCTGGCGGTGGGCCGGACGCTGATCGCCGTGCTCGAGAACGGCCAGCAGGAAGACGGCTCTGTCCTGCTGCCCGAGGTTCTGCACCCCTACCTGCGGGGCGCCACCCGCGTCACCCCCGAGGGCGCGTTGACCTGAATGGCGATCACAGCTGCACAGGTAAAAGAGCTGCGCGACACCACCGGCGCAGGCATGATGGACGCCAAGAAGGCGCTGACCGAGAACAATGGCGACATGGAAGCCGCCGTCGACTGGCTGCGGACCAAGGGTCTGGCGAAGGCCGCCAAGAAGTCCGGCCGTACCGCCGCCGAGGGCCTGGTCGCCGTCGCTATCGAAGGTGGCGAAGCCGTCGCCCTCGAGGTGAACGCCGAGACCGACTTCGTCGCCAAGAACGCCGATTTCCAGGCGATGGTCGCTGATTTCTCGAAAGCCGCCCTGAAGGTCGGTTCGGTCGATGAGCTGAAGGCCAGCGACATCAACGGCAAGAAGGTCGAGGACATCCTGACCGACAAGATCGCAACCGTGGGCGAGAACATGGCGATCCGCCGGATGGCCAAGATCAGCGGTGAGACCGTCACCGCCTACGTTCACAACCAAGCTGCCGAGAACATGGGCAAGATCGGCGTGCTGATCGCCATGAACGGCACCGACAACGGCATCGGCCGTCAGATCGCAATGCACGTCGCCGCCGCCAACCCCGCATCGCTGGGCGAAGCCGACCTGGACCAGGCCCTGGTCGAGCGCGAGAAAAGCGTCCTGACCGAGCAGGCCCGTGAATCCGGCAAGCCCGAGCAGGTCATCGAGAAGATGATCGAAGGCCGCATGAAGAAGTTCCTCTCCGAAGTGACCCTTCTGGGCCAGGCTTTCGTCATCAACCCCGACCAGACCGTGGCCGAGGCCGCCAAGGAAGCCGGCGTCGAGATCACCGGTTTCGTGCGCCTGGAAGTGGGCGAGGGGATCGAGAAGGAAGCCGAGAACTTCGCCGAGGAAGTCGCGAAGATGAACGCC---TGAATGAACCAGGAACTGACAAACAACCCGTTCAACCCGCTGACCCCGCCGAAGCAGTTCGACGAGATCAAGGTGTCGCTGGCCTCGCCCGAGCGGATCCTCTCGTGGTCCTTCGGTGAGATCAAGAAGCCCGAAACCATCAACTACCGTACGTTCAAGCCCGAGCGTGACGGCCTGTTCTGCGCGCGTATCTTTGGCCCGATCAAGGATTACGAATGTCTGTGCGGCAAGTACAAGCGGATGAAGTATCGCGGCGTCGTCTGCGAGAAATGCGGTGTGGAAGTCACCCTGCAGAAGGTCCGTCGCGAGCGGATGGGCCACATCGAACTGGCCTCGCCCGTCGCGCACATCTGGTTCCTCAAGTCGCTGCCCTCGCGCATCGGCCTGATGCTGGACATGACCCTGCGCGATCTTGAGCGGATCCTGTATTTCGAGAACTACGTCGTCATCGAGCCGGGCCTGACGGACCTCACCTATGGTCAGCTGATGACCGAGGAAGAGTTCCTGGACGCCCAGGACGCCTACGGCACCGACGCCTTCACCGCCGGCATCGGCGCCGAGGCCATCCGCGAGATGCTGGCCGCGATCGACCTCGAGTCCGAGGCCGAGCAGCTGCGCGCCGATCTGGCCGAGGCCACCGGCGAGCTGAAGCCCAAGAAGATCATCAAGCGCCTGAAGATCGTCGAGAACTTCATCGAGTCGGGCAACCGCCCCGAGTGGATGGTCCTGACCGTGATCCCCGTGATCCCGCCCGAGCTGCGCCCGCTGGTGCCGCTGGACGGTGGCCGCTTTGCGACCTCGGACCTCAACGACCTTTACCGTCGCGTCATCAACCGCAACAACCGTCTGAAGCGCCTGATCGAGCTTCGTGCGCCCGACATCATCGTGCGCAACGAAAAGCGGATGCTGCAGGAATCGGTCGACGCTCTGTTCGACAACGGTCGCCGTGGCCGGGTCATCACCGGTGCCAACAAGCGCCCGCTGAAGTCGCTGTCGGACATGCTCAAGGGCAAGCAGGGCCGCTTCCGCCAGAACCTTCTGGGCAAGCGGGTCGACTTCTCGGGCCGTTCGGTCATCGTGACCGGACCGGAGCTCAAGCTGCACCAGTGCGGCCTGCCCAAGAAGATGGCGCTGGAGCTGTTCAAGCCGTTCATCTACTCGCGGCTCGAGGCGAAGGGCCTCAGCTCGACCGTGAAGCAGGCCAAGAAGCTCGTTGAAAAGGAACGCCCCGAGGTCTGGGACATCCTGGACGAGGTGATCCGCGAGCATCCGGTGATGCTGAACCGTGCACCCACCCTGCACCGTCTTGGCATCCAGGCGTTCGAGCCCATCCTCATCGAAGGTAAGGCGATCCAGCTGCACCCGCTCGTCTGCTCGGCCTTCAACGCCGACTTCGACGGTGACCAGATGGCCGTTCACGTGCCTCTTTCGCTGGAAGCCCAGCTGGAAGCACGCGTGCTGATGATGTCCACGAACAACGTTCTGTCGCCCGCCAACGGCGCACCGATCATCGTGCCGTCGCAGGACATGATCCTGGGTCTCTACTACATCACGCTGGAGCGTGAGGGCATGAAGGGCGAGGGCATGGTCTTCGCCGATGTGGACGAGGTGCAGCACGCGCTGGACGCAGGCGAGGTGCACCTGCACTCGAAGATCACCGCGCGTCTCAAGCAGATCGACGACGAGGGCAACGAGGTCATGCGCCGGTTCGAAACCACGCCGGGCCGCGTCCGTCTGGGTGCGCTTCTGCCGATGAACGCCAAGGCGCCCTTCGATCTGGTGAACCGTCTGCTGCGCAAGAAAGAGGTGCAGCAGGTCATCGACACCGTCTACCGCTACTGCGGTCAGAAAGAGTCGGTCATCTTCTGCGACCAGATCATGACCACCGGTTTCCGCGAGGCTTTCCGGGCCGGCATCTCGTTCGGCAAGGACGACATGACCATCCCCGAAGCCAAGTGGAAGATCGTGGACGACGTCCGCGGTCAGGTGAAAGAGTTCGAACAGCAGTACATGGACGGCCTGATCACCCAGGGCGAGAAGTACAACAAGGTCGTCGACGCCTGGTCCAAGTGCTCGGACGAGGTTGCGGCCGCCATGATGTCCGAGATCTCGGCCGTTCGTAAGGACGACGCCGGTGCCGAGATGGAGCCGAACTCGGTCTACATGATGTCCCACTCGGGTGCGCGTGGTTCGCCCGCCCAGATGAAACAGCTGGGCGGCATGCGCGGCCTCATGGCCAAGCCCTCGGGCGAGATCATCGAGACGCCGATCATCTCGAACTTCAAGGAAGGTCTGACCGTTCTTGAATACTTCAACTCGACCCACGGTGCCCGTAAGGGTCTGGCCGATACCGCGCTCAAGACTGCGAACTCGGGCTACCTGACCCGCCGTCTGGTGGACGTGGCACAGGACTGCATCGTGCGTCAGCACGACTGTGGCACCGATCTGGCCGTCACCGCTGAGCCTGCCGTCAACGACGGCGAGGTCGTGTCGTCGATGGCCGAGCGGATCCTTGGCCGTGTCGCCGCCGAGAACGTCCTGATGCCGGGCACCGACGAGGTGCTTCTGTCCAAGGGCGAGCTGATCGACGAGCGCAAGGCCGACGCGGTCGAGCAGGCCGGTGTCCTGCGGATGCGTATCCGCAGCCCGCTGACCTGTGAGGCAGAAGAAGGCGTCTGCGCCATGTGCTACGGGCGCGACCTTGCCCGCGGCACGATCGTCAACGAAGGCGAGGCCGTCGGTATCATCGCGGCCCAGTCCATCGGCGAGCCCGGCACGCAGCTGACGATGCGGACCTTCCACATCGGCGGTATTGCACAGGGTGGTCAGCAGTCCTTCCTCGAGGCGTCGCAAGACGGCAAGATCGAGTACCGCAACGCCGTTGTCCTGAAGAACGACGCCGGCGAGACCATCGTCATGGGCCGGAACATGATCCTGGCGATCGTCGACGGTGACGGCGCCGAGCGGGCCAGCCACAAGCTGGGCTACGGCACCAAGATCTTCGTCGAGGACGGCGCCAAGGTGTCCCGTGGCGACAAGCTGTTCGAATGGGATCCCTACACCCTGCCGATCATCGCCGAGAAGGCCGGTAAGGCCAAGTTCGTGGACCTCGTGAGCGGCATCGCCGTGCGCGACGAGACGGACGATGCCACCGGCATGACCCAGAAGATCGTCATGGACTGGCGCGCGGCCCCCAAGGGCAACGAGCTCAAGCCCGAGATCCTGATCGTCGGCGAGGATGGCGAGCCTGTCCGCAACGACAACGGCAACCCGGTCACCTACCCGATGTCGGTGGACGCCGTTCTGTCCGTCGAGGAAGGGCAGGAGGTCCGTGCCGGTGACGTGGTCGCGCGCATTCCGCGCGAAGGTGCGAAGACCAAGGACATCACCGGTGGTCTGCCGCGTGTGGCCGAACTCTTCGAGGCACGTCGCCCCAAGGATCACGCGATCATCGCCGAGATCGACGGCTACGTCCGCTTCGGTCGCGACTACAAGAACAAGCGTCGCATCACGATCGAACCGGCGGACGAGTCGATGGAGCCCGTCGAATACATGGTGCCCAAGGGCAAGCACATTCCCGTCGCGGAAGGTGACTTCGTCAACAAGGGCGACTACATCATGGACGGCAACCCCGCGCCCCACGACATCCTGTCGATCATGGGTGTCGAGGCTCTGGCTGAATACATGATCAACGAGGTGCAGGACGTCTATCGCCTTCAGGGCGTTAAGATCAACGACAAGCACATCGAGGTCATCGTGCGCCAGATGCTCCAGAAGTGGGAGATCTCGGACAGCGGCGAGACGACGCTGCTCAAGGGCGAGCATGTCGACAAGGCCGAGTTCGATGCAGCCAACGACAAGGCCATTGCCCGCGGCGGTCGCCCCGCCCAGGGCGAGCCGATCCTGCTAGGGATCACCAAGGCCTCGCTGCAGACGCGCTCGTTCATCTCGGCGGCGTCCTTCCAGGAGACGACCCGCGTTCTTACCGAGGCTTCGGTTCAGGGCAAGCGCGACAAGCTGGTCGGCCTCAAGGAGAACGTCATCGTGGGTCGCCTGATCCCCGCCGGCACGGGTGGTGCTACCAAGCGCGTCCGCCAGATCGCGACCGAGCGGGACCGCAAGGTCATCGAGCAGCGCCAGGCCGAGGCCGAGGCCGCGCTGGCGCTGAACGCGCCGGACGAGGCGGCGGTCGAG---------------------GATGGCTTCGGCATGGCCCCCGAAAGCCGCGACTAAATGTCCCGTCGTCACGCCGCAGAAAAACGCGAAGTCCTGCCCGATGCTAAGTACAGCGATCGGGTTCTGACGAAATTCATGAACAACCTGATGATCGACGGCAAGAAATCTGTCGCCGAATCCATCGTCTACAACGCCATGGAGCGGGTCGAGGAGCGTCTGAAGCGCGCCCCGATCGAAGTGTTCCACGAGGCGCTGGACAACGTGAAGCCCTCGGTCGAGGTGCGCTCGCGCCGCGTCGGTGGTGCCACGTATCAGGTTCCCGTCGAGGTCCGCCCCGAGCGCCGCGAAGCCCTGGCCATCCGTTGGCTGATCAAGGCTGCCCGCGCCCGGAACGAGAACACGATGGAAGAGCGCCTTGCAGGCGAGCTGAGCGATGCGGTCAACAGCCGCGGCACGGCCGTCAAGAAGCGCGAAGACACCCACAAGATGGCCGACGCGAACAAAGCGTTCAGCCATTACCGCTGGTAAATGCCCCATGCCCATACCGACCGCTCC---------CCGGTGATGCACGCGCCCGCGCCCGACGTGCGCAACCGCAAGAAACTGGAGGGCGGTCGCCGCTTCAAGCTGGAGACCGAGTTCAGCCCCGCCGGCGACCAGCCGACGGCGATTGCCGAGCTGTCGGCCGGCGTCATGGCCGGCGAGCGGGATCAGGTCCTGCTGGGCGCCACGGGCACGGGCAAGACCTTCACGATGGCCAAGATCATCGAGGAGACCCAGCGCCCGGCGATCATCCTGGCGCCGAACAAGACCCTTGCCGCCCAGCTTTACGGCGAGTTCAAGGGCTTCTTCCCCGACAACGCGGTCGAGTATTTCGTCAGCTACTACGACTACTACCAGCCCGAGGCCTACGTGCCCCGCTCGGACACCTATATCGAGAAGGAATCCCAGATCAACGAACAGATCGACCGGATGCGCCACTCGGCCACGCGGGCGCTTCTGGAACGCGATGACGTGATCATCGTGGCCTCGGTCTCGTGCATCTACGGTATCGGTTCGGTGGAAACCTATGGGGCCATGACCCAGGATCTGATCGCGGGTCAGGAATACGACCAGCGGGCGATCATCGCCGATCTGGTGGCGCAGCAGTATCGCCGCAACGATCAGGCGTTCCAGCGCGGCACCTTCCGGGTGCGGGGCGACAGCCTCGAGATCTTTCCCGCCCACCTCGACAGTCGCGCTTGGCGGCTGTCGTTCTTCGGCAACGAGCTGGAAAGCATCACCGAGTTCGACCCCCTGACCGGCGAGAAGACCGACACGTTCCAGCAGATCCGCGTCTACGCCAACTCGCACTACGTCACGCCCAAGCCGACGATGAAACAGGCCGTCAATTCGATCAAGAAAGAGCTGCGCCAGCGGCTGGACCAGCTTGTGGCCGACGGCAAGCTGCTGGAAGCCCAGCGGCTGGAGCAGCGCACCAATTTCGACATCGAGATGCTGGAGGCCACGGGCGTCTGCAACGGCATCGAGAACTACTCGCGCTACCTCACCGGCCGCGCCCCCGGAGAGCCGCCCCCCACCCTGTTCGAATTCATTCCCGACAACGCCATCGTCTTCGCCGACGAGTCCCACGTCAGCGTGCCCCAGATCGGCGGCATGTATAAAGGCGACTACCGGCGCAAGTTCACGCTGGCCGAGCACGGGTTCCGCCTGCCGTCCTGCATGGACAACCGCCCCCTCAAGTTCGAGGAATGGGACGCCATGCGCCCGCAATCGGTCTTCGTCAGCGCGACCCCCGCCGGGTGGGAGCTGGAGCAGGCCGGCGGCGTCTTCACCGAACAGGTGATCCGCCCCACCGGCCTTCTGGACCCCGAGATCGAGATCCGCCCCGTCGGCACGCAGGTCGACGACCTGCTGGACGAGGTGCGCCGCGTCACCGCCGCCGGCTACCGCACGCTGGTCACCACCCTGACCAAGCGCATGGCCGAGGATCTGACCGAATACATGCATGAACAGGGCATCAAGGTGCGCTACATGCACAGCGACATCGACACGCTGGAACGCATCGAGATCCTGCGCGACCTGCGGCTGGGGGCGTTCGACGTGTTGATCGGCATCAACCTTCTGCGCGAGGGTCTGGATATTCCCGAATGCGGGCTGGTCGCGATCCTTGATGCCGACAAGGAAGGCTTCCTGCGCTCGGAAACCTCGCTGATCCAGACCATCGGCCGGGCCGCGCGGAACGTGGACGCCCGCGCGATCCTTTACGCCGACCGCATCACCGGCAGCATGGAACGCGCCATGCGCGAGACCGAGCGTCGGCGCGAAAAGCAGATCGCCTACAACACCGAACACGGCATAACCCCCGCCACCGTCAAGAAGAACGTCGAGGATATTTTGGCCGGCCTCTATCAGGGCGACGTGGACATGAACCGCGTCACCGCCAAGATCGACGCGCCGATGGCCGGTGCAAACCTTCAGGCCCATCTGGACGGGCTGCGCGACAAGATGCGCAAGGCCGCCGAGAACCTCGAGTTCGAAGAGGCCGCCCGCCTGCGCGACGAGGTCAAGCGGCTGGAAACCGTCGATCTGGTGGTCAGCGACGATCCCCTGGCCCGCCAGCAGGCGGTCGACCGGGCGGTCGATGCCGCGCAAAAGGCCTCGGGCCGGTCCACGGCCGGGCGTGGCGGCATGCGGGGCGGCGTCAAGCGGCGGAAGGGC---TGAATGGCCGCAAAACCGTTTTTCCGCCGCCGCAAGGTCTGCCCCTTCTCGGGCGACAACGCACCCAAGATCGACTACAAAGACACCCGTCTGCTGCAGCGCTACATCTCCGAGCGTGGCAAGATCGTGCCCTCGCGGATCACCGCAGTCTCCGCCAAGAAACAGCGCGAGCTGGCCCGTGCGATCAAGCGCGCCCGCTTCCTCGCCCTGCTTCCCTACGCCGTGAAATAAATGCCCAAACGTATCCTCAACGGCACCGTCACCAGCGACCAGAACGAGCAGACTGTCACGGTCCTGGTCGAGCGTCGCTACACGCATCCTCTGCTGAACAAGACCGTCCGCGCTTCGAAGAAGTACCGCGCGCACGATCCGAAGAACGAATTCAAGGTCGGTGACAAGGTCCGCATCCAGGAGTGCGCGCCGATTTCGAAAACCAAGCGTTGGGAGGTGGTCGCCAAC------TAAATGGCTAACTCGCCTCAGGCAAAAAAACGCGCGCGCCAGAATGAGCGCCGCCAGAACGTGAACAAGGCCCGCCGTTCGCGGATCCGGACCTTCCTCCGCAAGGTCGAGGAAGCGATTACCTCCGGCGACGCGGATGCCGCCAAGACCGCCCTTCAGCAGGCCCAGCCCGAGCTGATGCGCGGCGTTACCAAAGGCGTGATGCACAAGAGCACCGCCTCGCGCAAGATGTCGCGCCTGAACAGCCGGGTCAAAGCCCTGGCCTGAATGTCGCGTTCTGTATGGAAGGGCCCTTTTGTCGACAGCTATGTCCTCAAGAAGGCCGAGAAGACCAAAGAATCCGGTCGCAACGAAGTCATCAAGATCTGGTCGCGCCGCTCGACGATCCTGCCCCAGTTCGTGGGTCTGACGTTTGGCGTCTACAACGGCCAGAAGCACATCCCGGTCAACGTGACCGAGGACATGATCGGCCAGAAGTTCGGTGAATACTCGCCGACGCGGACCTACTACGGTCACGCTGCCGACAAGAAATCGAAGCGGAAGTAAGTGGCACGTATTGCTGGCGTCAACATCCCGACCGGGAAACGCGTCCCCATCGCCCTCACCTACATCACCGGTATCGGCAACACCTCGGCCCGCGCAATCTGCGACGCCGTGGGTATCGAACCGACCCGCCGCGTCAACGAGCTGAGCGACGCCGAAGTGCTTCAGATCCGCGAGCACATCGACGCCAACTTCACCGTTGAAGGCGACCTGCGCCGCGAAGTGCAGATGAACGTCAAGCGTCTGATGGACCTGGGCGCCTACCGCGGCCTGCGTCATCGTCGCAACCTGCCCGTGCGCGGCCAGCGCACCCACACCAACGCCCGCACCCGCAAGGGCCCGGCGAAGCCGATCGCCGGCAAGAAGAAATAAATGTCCATGAAAATCCGTCTCGCCCGTGGTGGCTCCAAGAAGCGCCCCCACTATTCCATCGTCGCCGCCGACAGCCGCATGGCCCGCGACGGCCGCTTCAAGGAGAAGCTGGGCACCTACAACCCGCTCCTGCCCAAGGACAGCGAAGAGCGCGTCAAGATGGACGTCGAGCGCGTTCAGTACTGGCTGGACCAGGGCGCCCAGCCCACCGACCGCGTCAGCCGTTTCCTGGAGGCCGCCGGCCTGAAGGACAAGACCGAGCGCAACAACCCCAACAAAGCCAAGCCCGGCAAGAAGGCGACCGAGCGCGCCGAGGAGAAGGCCGCCAAGGCAGCCGAGGCCGCCGAGGCGACCGCCGCCGATGCCGAGGAAGCCGCAGCCGAG---TGAATGTCGATCACCGTCGAAGACAAGCAGCGCCTGATGAAGGAATTCGCAACCAAGGAAGGCGACACCGGTTCGCCCGAAGTCCAGGTTGCCATCCTGTCCAGCCGCATCGCCACCCTGACCGAGCATTTCAAGACCCACAAGAAGGACAACCACGGCCGCCGTGGTCTTCTGAAGATGGTCGCCCAGCGCCGCAAGCTGCTGGACTACACCCGCGCCAAGGACGAGGCCCGTTACCAGGACCTCATCAAGCGCCTGGGCCTGCGCCGCTGAATGCCCAGCCTGAACGAGATCCGATCCACCTTCCTGAACTACTTCGAACGCCAGGGTCACCAGGTCGTGCCCAGCTCTCCGCTGGTGCCGCGCAACGACCCGACGCTGATGTTCGCCAACTCGGGCATGGTGCAGTTCAAGAACCTGTTCACGGGGGTCGAAAAGCGCGACTACACCCGCGCCACCACGGCCCAGAAATGCGTGCGCGCCGGCGGCAAGCACAACGACCTGGACAACGTGGGCTATACCGCCCGGCACCACACCTTCTTTGAGATGCTGGGGAATTTCAGCTTCGGCGATTATTTCAAGAACGAAGCCATCCCCTTCGCATGGGAGATGATCACCAAGGAGCTGGACATCCCCAAGGACCGGCTGGTCGTCACCGTCTACCACGACGATGACGAGGCCGCCGAGATCTGGAAGAAGGTCGCGGGCATTTCGGACAACCGGATTATTCGCATCGCCACCGACGACAATTTCTGGATGATGGGGCCGACCGGGCCCTGCGGACCCTCGTCCGAGATCTTCTTCGACCACGGCGACCACATCTGGGGTGGCCCTCCGGGCAGCCCCGAGGAGGATGGTGACCGCTTCGTCGAGATCTGGAACCTGGTCTTCATGCAATATGAGCAGTTCGAGGACGGCACCCGTGAGCCGCTGCCGAACCAGTCCATCGACACCGGCATGGGGATCGAACGGGTGGCGGCCCTGTTGCAGGGCACCAACGACAACTATGCGACCGACCTCATGCGCTCGCTGATCGAGGCTTCGGCCCACGCCACCTCGACCGACCCCGATGGGCCCGGCAAGACCCATCACCGGGTGATCGCCGATCACCTGCGCTCGACCTCGTTCCTGATTGCCGACGGGGTGATGCCGTCGAACGACGGGCGCGGCTATGTCCTGCGCCGGATCATGCGCCGCGCCATGCGTCACGCGCATCTGCTGGGCTCGCAGGATCCGGTGATGCACCGTCTGGTGCCGGCGCTGGTGCAGCAGATGGGCGCCGCTTACCCCGAGCTGGGCCAGGCCCAGGCGCTGATCCAGGAGACCCTGCGCGCCGAGGAGACCCGTTTCAAGCAGACGCTTGAGCGCGGCCTGCGCCTGCTGGACGACGAGCTGTCGGACCTGCCCGAGGATGCGCCGCTGCCGGGCGAAGCTGCCTTCAAGCTTTACGACACCTTCGGCTTTCCGCTGGACCTGACCCAGGACGCCCTGCGTGAAAAGGGCCGCAGCGTCGATACCGATGGCTTTGACGCCGCCATGGCCGAACAGAAGGCCAAGGCGCGCGCCGCCTGGTCCGGTTCGGGCGAGACGGCGGATGCCTCTCTTTGGTTCGATCTGGCCGAAAAGCACGGCACGACCGAGTTCCTGGGCTATGACACCGAAGTGGCCGAGGGCCAGTTGCTGGCCATCGTGCGCGATGGTGCCGCGGTGAAGACGGCCGAGGCCGGCCAGGAGGTGCAGTTCGTCCTGAACCAGACACCGTTCTACGCCGAATCGGGCGGCCAGGTCGGCGACCAGGGCGAGATCCGCACCGAAACGGGCGCCGCCCGCATCACCGACACCCGCAAGACCGCCGGTGTCTTCATCCACATGGGCCAGGTTACCGAGGGCACGATCGAGACGGGGCAGGGCGCCGAGCTGGAGGTGGACCACTCCCGCCGCAGCGCCATCCGCGCCAACCACTCGGCCACGCACCTGCTGCACGAGGCCCTGCGCCGGACGCTGGGCGATCATGTGGCGCAGCGCGGATCGCTCAACGCCCATGACCGGCTGCGGTTCGATTTCAGCCACGGCAAGGCCCTGAGCCGCGAAGAGCTGGATCAGGTCGAGACCGAGGTGAACGAGTTCATCCGCCGCAACGAAAAGGTCGAGACTCGCATCATGACCCCCGACGACGCCCGCGCGATCGGCGCGCAGGCGCTTTTCGGCGAGAAATACGGCGACGAGGTGCGCGTGGTCTCGATGGGCTCGCTGTCGGGTTCGGGCAAGGGCGCCTCGGGCGATACCTATTCGCTGGAGCTTTGCGGCGGCACGCACGTTCGCCAGACCGGCGACATCGGCATGTTCGTCCTGCTGGGCGACAGCGCCTCAAGCTCGGGCGTGCGGCGGATCGAGGCCCTGTCGGGGGCCGATGCCTTCCGCTACCTGGCCGATCAGGGCAAGCACCTGTCGGACGCAGCATTGGCCCTGAAGGCCCGGCCCGACGAGGTGCCCGAGCGTGTCCGCGCCCTGCTGGATGAGCGCAAGGCGCTGACCAACGAGCTGGCGCAGCTGCGGCGTGAACTGGCGATGGGTGGCGGTGGCGGCGCGGCGCAGCCCGAGGCGCAGGAGGTCAACGGCATCCGCTTTGCGGCCCATGTGGTCAATGGCGTCACCGGCAAGGATCTTCCGGCGCTGGTGGACGAGCACAAGGCGCGGCTGGACAGCGGCGCGGTACTGCTGATCGCCGACACCGGCGGCAAGGCGGCCGTGGCCGCGGGCGTCACCAAGGACCTGACCGACCGGCTGTCGGCGGTGGATCTGGTTCGCGCGGCGGTGGCCGAACTGGGCGGGAAGGGCGGCGGCGGCCGGCCCGACATGGCCCAGGGCGGCGGCCGCGACACGGCCAATTCCGATGCCGCGATCGCCGCGGCACGCAGCCACCTGGAGACGTGAATGGGTTTCAAGACCGGGATCGTCGGGCTTCCGAATGTGGGCAAATCCACGCTGTTCAATGCGCTGACGCGCACGGCTGCCGCCCAGGCGGCGAACTTTCCCTTCTGCACGATCGAGCCCAATGTGGGCGAGGTGGCCGTGCCCGACGCGCGGCTCGACAAGCTGGCCGCGATCGCCAGTTCCAAGCAGATCATCCCGACCCGCATGACCTTCGTCGATATCGCCGGCCTGGTGAAGGGTGCCTCCAAGGGCGAGGGGCTGGGCAACCAGTTCCTGGCCAACATCCGCGAGTGCGACGCCATCGCCCATGTGCTGCGCTGTTTCGAGAATGACGACATCACCCATGTCGACGGGCGCGTCGACCCGGTCGAGGATGCCGAGACCATCGAGACCGAGCTGATGCTGGCGGACATGGAATCGATCGAGAAGCGGCTTCAGAACCTGCAGCGCAAGTTGAAGGGCAACGACAAGGATGCCGCCCAGCAGGACCGCCTGCTGCGCCGGGCGCTGGCCGCCCTGGAAGAGGGGCGCCCCGCCCGCACCGTCGAGATCGACGCCGAGGACGAGAAGGCCTGGGCGATGCTTCAGCTTCTGACCTCCAAGCCCGTCCTCTATGTCTGCAACGTCGACGAGGCCAGCGCCGCCACCGGCAATGACCAGACCCGCCGCGTCGCCGAGATGGCCGAGGCGCAGGGCGCGGCCCATGTCGTGATCTCGGCCGCGATCGAGGAAGAGATCAGCCAGCTCGACCCCGAGGAGGCGCGCGAGTTCCTGGAGGAGCTGGGGCTGGAGGAGGCGGGCCTCGACCGTCTGATCCGGGCGGGCTACGAGTTGCTGGACCTGCAGACCTATTTCACTGTCGGACCCAAGGAAGCACGGGCCTGGACCGTGCCCGCGGGTTCCACCGCGCCGAAGGCCGCCGGTGTCATCCACGGCGATTTCGAGCGCGGTTTCATCCGGGCCGAGACCATCGCCTACGACGATTATATCGCCAACAACGGCGAGCAGGGGGCCAAGGACGCCGGCAAGCTGCGGGTCGAGGGCAAGAGCTACATCGTCAAGGATGGCGATGTGCTGCACTTCCTTTTCAACGCCTGAATGGGTTGGAAACGTCTCGAGGAGATGGATCTGCGCGGCAAGCGCGTGCTGACGCGGGTGGACATCAATGTCCCCGTCAGCGACGGGCGCGTCAGCGACGCCACCCGGATCGAGCGGATCGTGCCCACCGTCAATGCCATTCTCGAGGCTGGAGGCACGCCGATCCTGCTGGCCCATTTCGGCCGCCCCAAAGGCCGGCCCGACCCCGCCCTGTCGCTGGGCGTTGTCCTGCCGGCCCTCGAACGCGCCCTGGGCCGCGGGGTCTTCTTCGTCGAGGCCCCGATCGGCGCCAGGGAAGAGATCGCCTCTATCCGCGCCAAGGACGTTATCCTGCTGGAGAACGTGCGCTTCTATCCCGGCGAGACCGCCAACGAGGACGGGTTCTGCGATGCCCTGGCCGCCCTGGGCGACGTCTATTGCAACGACGCTTTCTCTGCCGCCCATCGCGCCCACGCCTCGACCGAGGGGCTGGCCCGCCGCCTGCCCGCCTGCGCCGGCCGGCTGATGGAGGCCGAGCTTTCGGCGCTGGAGGCCGCCCTGGGCAAGCCCGCCCGCCCCGTCGCCGCCGTGGTCGGCGGCGCCAAGGTCTCGACCAAGCTTGACCTGCTGTCGAACCTGGTGACACGGGTCGACCACCTGATCATCGGCGGCGGCATGGCCAACACGTTCCTGCATGCGCAAGGCGTCGCCATCGGCACGTCGCTGGCCGAGAAGGATCTTGCCGACACCGCCCGCCGCATCCTCGACGAGGCCGAGCGCGCCGGCTGCACCATCCACCTTCCCGTAGACCTGGTCGTTGCCCGCGAATTCCGCGCCGAGGCCCCGCACGAGATCGTCGCACGC---------GACGAGTGCCCGCCCGACGCGATGATCCTCGATGCCGGCCCCGAAAGCGTGCGCGCCATCGTTGAGCTTCTGGGCCGCTGCAAGACCCTCGTCTGGAACGGCCCGCTGGGCGCGTTCGAGATCCCGCCCTTCGACACCGCCACCAACGGCGCCGCGCGCGAGGCCGCGGTTCTGACAGCCGCCGGGCAACTCGTCTCGGTCGCCGGCGGCGGCGACACGGTGGCCGCCCTCAACCAGGCCGGGGCCGCCGATGGCTTCAGCTATGTCTCGACCGCGGGGGGCGCCTTCCTGGAATGGATGGAGGGCAAGACCCTGCCCGGCGTCGCCGCCCTGGGGGGCTGAATGGCGCTCCCCGATTTCACCATGCGTCAGCTGCTTGAAGCTGGCGTTCACTTCGGTCACCAGACCCAGCGTTGGAACCCCCGCATGGGCGAGTTCATCTATGGTGACAAGAACGGCATTCACATTCTCGACCTGACGCAGACCGTCCCGATGCTGGATCAGGCCCTGCAGGTCGTGCGCGACACCGTCGCCAAGGGCGGCCGCATCCTGTTCGTCGGCACCAAGCGTCAGGCCCAGAAGCCCGTCGCAGACGCCGCCGAGCGTTGCGCACAATACTACATGAACCACCGCTGGCTGGGCGGCACGCTGACCAACTGGAAAACCGTGTCGAACTCGATCAACCGCCTCAAGGCGATCGACGAGCAGATGCAGAACGGCGTTGAAGGCCTGACCAAGAAAGAGCGTCTGGGCATGGAGCGCGAGCAGGTCAAGCTGGAAGCCTCGCTTGGCGGTATCCGCGAGATGGGTGGCGTTCCCGATCTGCTGTTCGTCATCGACGTGAACAAGGAAGATCTGGCCATCGCCGAAGCCAAGAAGCTGGGCATTCCGGTCGTCGCCGTCGTCGACACCAACGCCTCGCCCGATGGCGTCGACTACATCATCCCCGGCAACGATGACGCGGCCCGCGCCATCGCTCTCTACTGTGACCTGGTCAGCCGCGCTGCCCTGGACGGCATGTCCGCCCAGATGGGCGCCGCTGGTTTCGACCTGGGTGCCATGGAAGAGGCCCCCGAGGAAGAGGCCGTCGCCGAGGAAGGCAATGCCTCCTCGGAGACCGTGGCCGACGACGCCGTCGCCAAGGACGCGGAAAACTGAGTGACCAAACGCACGTCTGCCAAGTACAAGCTCGATCGCCGCATGGGCGAGAACATCTGGGGCCGCTCCAAGTCGCCCGTCGTCCGCCGCGAATACGGCCCCGGCCAGCACGGTCAGCGCCGCAAGGGCAAACTGTCCGATTTCGGTATCCAGCTGCGCGCCAAGCAGAAGCTCAAGGGCTACTACGGCGACCTGACCGAGAAGCAGTTCCGCCGCATCTATGCCGAGGCCGAGCGCGTCAAGGGCGACACCGGTGAGAACCTCATCGGCCTGCTCGAGCGCCGCCTGGACGCCATCGTCTACCGCGCCAAGTTCGTGCCGACCGTTTTCGCCGCGCGCCAGTTCGTGAACCACGGCCACGTCCGTGTGAACGGCAAGAAGGTCAACATCCCCTCCTACCGCGTGAAGGAAGGCGACGTGATCGAGGTCCGCGACCGTTCCAAGCAGATGGCCGTCCTGCTCGAGGCCGTCCAGCTGGCCGAGCGTGACGTCCCCGACTACCTCGAGGTCGATCACTCCAAGATGACCGCCACCTTCGTGCGCACCCCGGCGCTGGGCGACGTGCCCTACCCGGTGATGATGGAGCCGAACCTGGTCGTGGAATTCTACGCGAAGAACTGAATGGGTCAAAAAGTCAATCCGATCGGCATGCGCCTCCAGGTCAACCGCACCTGGGACAGCCGCTGGTACGCCGACACCAAGGATTACGGTGATCTGCTTCTCGAGGACATCAAGATCCGCGAGTTCATCAAGGAAGAGTGCAAGCAGGCCGGTATCTCGAAGGTCATCATCGAGCGTCCGCACCGCAAGTGCCGCGTCACCATCCATACCGCCCGCCCCGGTGTCATCATCGGCAAGAAGGGCGCGGATATCGAGACGCTGCGCAAGAAGCTCGCCTCGATGACCGCAAGCGAACTGCACCTCAACATCCTCGAGGTGCGCAAGCCCGAGCTGGACGCCGCCCTGGTGGCCGAAAGCATCGCGCAGCAGCTTGAGCGTCGTGTTTCGTTCCGTCGCGCCATGAAGCGCTCGGTCCAGAACGCAATGCGCATGGGTTCGCTGGGCATCCGGGTCAACGTCGCCGGCCGTCTGGGCGGCGCCGAGATCGCCCGGACCGAATGGTACCGCGAGGGCCGCGTGCCCCTGCACACCCTTCGCGCCGACATCGACTACGCCCTGGCCGAAGCCAAGACCCCTTACGGGATCATCGGCATCAAGGTCTGGATCTTCAAGGGCGAGATCATGGAGCACGACCCGTCGGCCCGTGACCGCAAGCAACAGGAACTGCAGGAAGGGCCCGCACCTCGCGGTCCG------CGCCGGTAAATGGGCCGCAGACGCAAGGGACGCGACATTTCGGGCTGGGTGGTGATCGACAAGCCCGCCGGCATCAGCTCGACCGCCGTGGTGAACAAGCTGCGCTGGGCCTTTGATGCCAAGAAGGCCGGCCATGCCGGCACCTTGGACCCCGAGGCGACCGGAGTGCTGGCCGTGGCGTTGGGCGAGGCGACCAAGACCGTTCCCTACGTGACCGATGCGCTCAAGGCCTATCGCTTCACCGTGCGTCTGGGCCAGACCACCAACACCGACGACGCCGAGGGCGAGGTCATCGCCGAAAGCGACCTGCGCCCCAGCGACGCCGAGATCGACGCTGCCCTGCCCGCCTTTCGCGGCCATATCATGCAGGTCCCGCCCCAGTTCTCGGCCGTGAAGGTCGACGGCGAGAGGGCCTATGCCCTTGCCCGCGGCGGCGAGGAGCTGGAGCTGGCCGCCCGCCCCCTTTGGGTCGAGGAGCTGAGTTTGGTCGAGCGGCCCGATGCAGATCACGTCACCCTCGAGATGGTCTGCGGCAAGGGCGGCTATGTCCGGTCGATCGCACGGGACCTGGGCCAGGCGCTGGGTTGCGGCGGCCACGTCCGCGAGCTGCGCCGAACCTGGTCCGGCCCCTTCGATGCCGAGGACGGCCTGAGCATGGACCAGGTCGAGGAACTGGCGCGCACGCCCGCGCTGGACGACCACCTGCTGCCGCTGGAACTGGGCCTAGCCGACCTGCCGGAATTGCCTACCACTGCCGAGGGTGCGACGCGCCTGCGCAACGGCAATCCGGGCATGGTCCTGACCTCGGACGCCGAGTATGGCGACGAGGCATGGGCCTCGTTCGAGGGGCGCGCCATCGCCGTCGGCACCTACCGCGCCGGAGAGCTGCACCCCAGCCGCGTCTTCGTCGGC------TGACCGCACCTGATCGGGCTCACCGGTTCCATCGGCATGGGAAAGTCGACGACGGCGGGACTTTTCGCCGCCGAAGGGGTGCCGGTCTGGGATGCCGATGCGGCGGTGCACCGCCTTTATGGTCCTGGCGGGGCTGCCGTCGCCCCACTGGCGCGGATCTGTCCCGATGCGATTGTCGAGGATGCGGTGGACCGCCAGCGCCTCAAGGACTGGATCGCCCGGGACGACGCCGCCCTGCCCCGGATCGAGGCGATCGTTCACCCGCTGGTCGGCGCCGATCGCGCCGCATTTCTGGACGAGGCCGCGGCAGACATCGTGGTTCTTGATATCCCGCTGATCTTCGAAACCGGCGCCGCCGACCGTTTCGACACGCTGGTCGTGGTCAGCGCGCCGGCCGAGGTGCAGCGCGCCCGCGTCCTCGCCCGGGGCACCATGACCGAGGCCGAGTTCGAGAATATCCTGGCCCGCCAGGTTCCCGATGCCGAAAAGCGCCGCCGCGCCGATCACGTCATTCCGACCACGACCCTTGAAGCCGCCGCCGCCGCCGTGCGACAGATCCTTGAGCAGATCAGGGGAAAT---CGCGATGCGTGAATGACGCCGGTTGACGCCCTGATCGAGGACGACCGCTGGTGCGCACTGGGGCTTGAGGATCTGGCCGCCCGCGCCGCCGGCGCCGTGCTGACGCATCTCGACCTGGATCCCGAGGAATTCGAGATCAGCCTGCTGGGCTGTGATGACGCGCGCATCGCCGTTCTGAACGAGGAGTTTCGGGGCAAGCCCGCACCCACCAACGTGCTGAGCTGGCCGGCCGAGGATCTGGCCCCCGAAAGCCCCGGCGCCATTCCGCCGCCCCCCGAACCGATGTTCGACGGC------GCGTTAGGTGACATCGCCATCGCCTATGACACCTGCCTGCGCGAGGCCGAGGAACAGGGCAAACCGCTGGAAAACCACCTGCTGCACCTGCTGGCGCATGCGACGTTGCACCTGCTGGGCTATGACCACGAGACGGACGAAGATGCCGCAGTGATGGAACGTCTCGAGCGGGAAATACTTGTTTCCCTGGGTGTGCCTGATCCATATTCAGGCCAGACGGCCTGAATGGCACGGTTCGTTTTCATCACGGGTGGGGTTGTTTCGTCGTTGGGCAAAGGCCTGGCATCGGCGGCGCTTGGATCGCTTCTGCAGGCGCGCGGCTTCTCGGTCCGTCTGCGCAAGCTTGACCCCTATCTCAACGTGGACCCGGGCACGATGTCGCCCTTCGAGCATGGCGAGGTCTTCGTCACCGACGATGGTGCCGAGACCGACCTCGACCTTGGCCATTACGAGCGTTTCACCGGCGTCGCCGCCCGCAAGACCGACAGCGTCTCCTCGGGCCGGATCTATTCCAACGTGCTGGAGAAGGAGCGTCGCGGCGACTACCTGGGCAAGACGATCCAGGTCATTCCCCACGTCACCAACGAGATCAAGGACTTCATCTCCATCGGGGAGGACGAGGTCGATTTCATGCTGTGCGAGATCGGCGGCACCGTCGGCGACATCGAAGGCCTGCCCTTCTTCGAGGCGATCCGTCAGTTCGCCCAGGACAAGCCGCGCGGGCAATGCATCTTCATGCATCTCACGCTGCTGCCCTGGATCGGTGCCTCGGGCGAGCTGAAGACAAAGCCCACCCAGCACTCGGTCAAGGAGCTGCGTTCGATCGGGATCGCGCCGGACATCCTGGTCTGTCGCTCGGACAAGCCCATCCCCGAGAAGGAGCGCGAGAAGATCGCCCTCTTCTGCAACGTCCGCAAGGAGGACGTGATCGCCGCGCCCGACCTGCGCTCGATCTACGAGGCGCCTCTGGCCTATCACCGCGAGGGGCTGGACCAGGCGGTTCTGGACGCCTTCGGCATCCACCCCGCGCCAGCGCCCAACTTGTCGATCTGGGAAGACGTGGCCGACCGGGTCTTCAACCCCGAGGGCGAGGTCCGCGTCGCCATCGTCGGCAAGTACACCCAGCTTGAGGATGCCTATAAATCCATCGCCGAGGCGCTGACCCACGGCGGCATGGCCAACCGGGTCCGCGTCAAGATCGAGTGGATCGACGCCGAGATCTTCGAGCGCGAGGATCCCGCCCCCCACCTGGAACGGTTCCACGCCATCCTGGTGCCCGGCGGCTTTGGCGAACGGGGCACCGAGGGCAAGATCAAGGCCGTAGAGTTCGCCCGCACCCGCAAGGTCCCCTACCTGGGGATCTGCCTGGGCATGCAGATGGCCGTGATCGAGGCCGCCCGCAACGTCGCCGGCATGACCACCGCCGGGTCCGAGGAGTTCGACCACGAGGCGGGCGAGAAGCGGTTCGAGCCGGTGATCTTCCACCTCAAGGAATGGATCAAGGACAACGAGACGATTGCCCGCACCGTGCTGGACGACAAGGGCGGCACCATGCGCCTGGGCGAATACGACGCCACGCTGACCGAAGGCTCGAACGTGGCCTCGGTTTACGGCACCCGCCACATCAAGGAACGCCACCGCCACCGCTACGAGGTCGACATCAAGTACCGCGAAGCGCTGGAGAAGGTCGGGCTGTGCTTCTCGGGCCTGTCGCCCGACGGCCGCCTGCCCGAAATCGTCGAGTGGAAGGACCATCCGTGGTTCATCGGCGTGCAGTTCCACCCCGAGCTGAAGTCCAAGCCCTTCGCGCCCCATCCCCTGTTCCGCGACTTCATCCGCGCGGCCAAGGATACCTCGCGCCTGGTCTGA---ATGGACAAGCTGACAGTGCTGGGGCTGGAAAGCAGCTGCGACGACACGGCCGCGGCCGTGGTCGAGCTGACGCGCGCCGCCGACGGGCAGGTGCTGGCCTCGGTCGTGCACGGGCAGACGGACCTTCACGCGGCCTTTGGCGGCGTTGTCCCCGAACTGGCCGCGCGGGCCCATGCCGAAAAGCTGGACCTGGCCGTGGAACAGGCTCTTTCACAGGCGGAAGTGCCGCTTTCGGCGGTGGATGCGATCGCCGTCACCTCGGGCCCGGGCCTGATTGGCGGGGTGATGTCGGGGGTGATGTGCGCGCGCGGCCTGGCCGCCGGCGCGGGCCTGCCGCTGATCGGGGTCAACCACCTGGCGGGTCACGCCCTGACCCCGCGCATGACCGATGGGCTGGAATTTCCTTATCTGATGCTGCTGGTTTCGGGCGGGCACTGTCAGTTCCTGCTGGTGCGCGGCGTCGACAGCTTCACCCGGCTGGGCGGCACGATCGACGATGCCCCCGGCGAGGCTTTTGACAAATGCGCCCGCCTGCTGGGCCTGGAACAACCCGGCGGCCCGGCGGTGGAGCGCGAGGCACGCGCTGGCGATCCGACCGCCCACCGCCTGCCGCGCCCCCTGCTGGACCGGCCCGGCTGCGACATGTCCTTTTCCGGGCTGAAGACCGCCCTACTACGCGCCCGGGACGGGATCGTCGCGCAGAAAGGCGGGCTGTCGGTCCAGGACCGGGCCGATCTGTGCGCCGCCTTCCAGGCCGCCGCATCGGACGTGCTGATCGAGAAGACCCGCCGCGCCCTGGATGTGGCGGCCGGAGCCTGCGGCGGGGTCCCCGCCCTTGCGGTGGCCGGCGGCGTTGCCGCCAATACGGCCCTTCGCAAGGGCTTGCAGTCGCTCGCCGACGCGCGCGGCATCGCCCTGGTCGCCCCGCCGTTGCGCTATTGCACCGACAATGCCGCGATGATCGCATGGGCCGGCGCCGAACGGCTTGCCGCCGACCTTGTCGAGCCGCAGGATCTGGTTCCGCGGCCGCGCTGGCCGCTTGATCGCACCAGCCCCAGCCTTCTGGGTTCGGGCAAGAAAGGGGCCAAGGCATGA---------------------------------------------------------------------------------------------------------------------------------------------------------------------------------------------------------------------------------------------------------------------------------------------------------------------------------------------------------------------------------------------------------------

>'Su-undariaeDSM-102234'

ATGATCGCCAAAGAACTACACGACAAGACGCCGGATCAACTCCGTGACGAACTTGTGAACCTGAAAAAAGAAGCGTTCAACCTGCGTTTCCAACAGGCCACTGGCCAATTGGAAAACCCAGCACGTCTGCGTACCGTTAAGCGTGACGTTGCTCGTGTTAAAACAGTATTGAACCAAAAAGCTGCAACCGCAGCGACAGACGCGTAAATGGCAACGGCAGATCTTTTGAATATGGAT---AAGAAATCGGCAGACAAGCAAAAGGCGCTCGATAGCGCGCTGGCGCAAATCGAACGGCAGTTCGGTAAAGGCTCAATCATGAAGCTGGGAACAGCGGGCGCGATACAAGATATCACGGCGTGCTCTACCGGTTCCTTGGGGCTTGATATCGCATTAGGCATCGGCGGCCTGCCAATGGGCCGTATCATTGAAATTTACGGGCCTGAATCTTCTGGTAAAACAACGCTGACGCTGCATTGCGTTGCGGAACAGCAAAAGATGGGCGGCGTTTGCGCGTTTGTGGATGCAGAACATGCGCTTGATCCGCAATACGCTAAAAAGCTGGGCGTCGACATTGACGAATTGCTGATCTCACAGCCCGACACGGGCGAGCAGGCTCTCGAGATTGTGGACACGCTGGTACGTTCTGGCGCCGTGAACATGGTTGTGGTCGATTCGGTTGCGGCACTGACGCCAAAGTCCGAACTTGAAGGCGAAATGGGCGATAGTTCTGTTGGTGTTCAGGCGCGTTTGATGAGTAAAGCAATGCGCAAGCTGACCAGCTCTATTTCACGTTCGAACTGCATGGTGATCTTTATCAACCAAATCCGGATGAAAATTGGTGTTATGTTTGGCTCTCCTGAAACGACAACAGGTGGCAACGCGCTTAAATTCTATTCCTCTGTACGTCTGGATATCCGCCGCATCGGTTCTCTCAAGGACCGCGACGAAGTGGTGGGCAACCAGACCCGCGTGAAGGTTGTGAAAAACAAAGTGGCAGCGCCGTTTAAGCAGGTCGAATTTGATATCATGTACGGTGAAGGCATCTCCAAAATGGGCGAATTGCTGGACATGGGCGTGAAGGCCGGAATCGTGGACAAATCAGGTAGTTGGTTCTCTTGTGGGGACGAGCGGATCGGGCAGGGGCGTGAGAATGCCAAAACCTACCTGCGTGAACATCCAGAGATGGCAATGGATATCGAAGACAAAATCCGCGCTTCGCATGGTCTTGACTTCAACGGTTCTGAAAACTTGGATCCTGATATTTTGGACGATTGAATGACCCGCTATACCCCCTCCGAGATCGAAGCACGCTGGCAAGCCGCTTGGGAGCAGAACGAAATCTTCAAGGCTGTCCGCTCGGCCGACAAGCCAAAGTATTATGTGTTGGAAATGTTCCCCTATCCGTCGGGCCGCATCCATATGGGCCACGTGCGCAACTATACACTGGGCGACGTGATTGCGCGGTACAAGCTGGCCAAGGGTTTTAACGTCTTGCACCCGATGGGATGGGATTCCTTCGGTCTGGCCGCTGAAAACGCCGCGATGCAAAAGGGTATCCACCCCGGCGAATGGACCTTCCAGAACATCGAAGACATGAAGAATCAGATGAAGCCGCTGGGTTTCTCGCTGGATTGGTCGCGTGAGATTGCAACCTGCCACCCTGATTACTACCAACACCAGCAGGCCATGTTCATTGATATGATCGAAGCGGGCCTGATCTATCGCAAGAACGCAGTGGTGAACTGGGACCCCGTCGATATGACCGTTCTGGCCAACGAACAGGTTGAACAGGGCCGCGGCTGGCGTTCTGGCGCATTAGTTGAACGCCGCGAACTGACGCAATGGTTCTTCAAAATCTCCGATTACTCGGATGAATTGTTGGGTGCGCTGGACACGCTGGAAAACTGGCCCGCCAAAGTACGTTTGATGCAGGAAAACTGGATCGGCAAATCGCGCGGTTTGCAATTCTCGTTCTCGACCGTGAATGCGCCCGGCGGACATGACCAGATTGAGGTTTACACAACCCGCCCCGACACCCTGTTAGGCGCGTCCTTTGTTGGAATTTCTCCTGATCACCCGATTGCCAAGCTGCTAGAGCGTGACAGTGCCGATGTCGCCGCGTTTGTCGCCGAGTGCCGCAAAGGCGGCACAACGGAAGAAGCCATTGAAACAGGTGAGAAGCTGGGTATGGACACAGGGATTCGCGTCCGCCATCCCTTTGACACCTCCAAGGAACTACCCGTCTACATCGCCAACTTTATCTTGATGGAATACGGCACAGGCGCGATCTTTGGCTGTCCTGCCCATGATCAGCGTGATTTCGAGTTTGCCACAAAATACGACCTGCCGATCATTTCGACATACCTGCCGTCCGAGGATGCCTCAGAAGAATTGACCGAAGCCTATGTGCCGCAGAAAACCGAAAAGGTATTCTATAACCGTGGTTTCTCAGGTGAACCGTGGCAGACAGGCCTAGAAGCCATCGACGCTGCCATCGCATTTTGCGAATCTCAAGGTGTTGGCCAAGGTGTGACCAAGTTCCGCCTGCGGGATTGGGGCCTGTCGCGCCAACGTTATTGGGGGTGCCCGATCCCAGTTGTGCATTGCGACGACTGCGGCGTGGTGCCTGAAAAGAAAGAAAACCTTCCGGTTAAGCTGCCCGAAGATGTCACCTTTGACATTCCAGGCAACCCGCTGGACCGCCACACAGAATGGCGCACCACACCTTGCCCGTCTTGTGGCAAAGCTGCACAGCGTGAAACCGACACCATGGATACTTTTGTGGATTCGTCTTGGTATTACGCACGTTTCACCGCGCCACACGCCGATACGCCCACAAATATGGATGACGCCGAATACTGGATGAACGTCGATCAGTACATCGGCGGTGTTGAACACGCGATTCTCCACCTGCTTTATGCCCGCTTCTTTGCGCGGGCGATGCAGATCACAGGCCATTTGCCGCAAAAAGCGATTGAGCCGTTTAACGCGCTGTTCACCCAAGGCATGGTGACACATGCGATTTATAAAACCATCGGTGCCGATGACCGCCCCGTCTTCCATTACCCCGAGGAAGTGAAAGGTGATCAGGCCTTCGAAGGTGGTGCAGAGGTTGAAATCATCCCTTCCGCCAAAATGTCCAAGTCCAAAAACAACGTGGTCGACCCGCTGAACATCATTTCTGCATTTGGCGCTGATACAGCCCGTTGGTTTGTTCTGAGCGATTCGCCCCCCGAACGCGATGTCGAGTGGACTGCCTCGGGTGCGGAAGCGGCATATAAACATTTGGGCCGTGTTTGGAACATCTGCGACAAGATCAGCCAGATGGAAGACAGCGATGCAGGCGACGACGATAAAGAATTGCTCAAGCAAATGCACAAAGCGACCCATGACGTGACCATGGCGATTGAAAGCTTTGGCTTTAACGCGGCCATCGCCAAGCTGTACGGCTTTACCGCCACATTGCAAAAATCCAAAGCAAGTAAGGCCGCGCAGCGCGAAGCAGTCATGACGCTTGCGCAGTTGATGTCCCCGATGACGCCGCATTTGGCCGAAGACATCTGGGCCAATCAAGGCGGCGAAGGGCTGATTGCCACTGCGCCTTGGCCTGTCGCTGATGAAGCCATGTTAAAAGACGATACCGTCACCCTGCCCATTCAGGTCAACGGCAAACGCCGCGGCGAAATTGATGTGCCTGCGGATATGCCAAAAGACGAAGTTGAAAAGCTGGCTCTTGCTCATGAGGCTGTGATCCGTATCCTTGACGGTGGAACACCCAAGAAGGTGATCGTTGTACCGGGACGGATTGTGAATGTCGTTGTTTAAATGGCCATTACATCCGCAAACCAGTTGGAGCTGCTGCAAACTGCCGAAGCAGTGGCCCGCGAAAAAATGATCGACCCAGCGTTGGTTATTGACGCGATGGAAGAATCTCTGGCCCGTGCTGCCAAGTCCCGCTATGGCGCGGAAATGGACATTCGTGTGGCGATCGACCGCAAAACAGGCCGCGCGACATTCACCCGCGTTCGTACCGTTGTTGCGGATGATGAGCTGGAAAACTATCAAGCTGAATTTACGGTTGAGCAAGCCAAGCAGTATATGGCTGATCCAGAAATCGGTCAAACCTATGTTGAAGAAGTGCCGCCAGTTGAAATGGGCCGTATCGCGGCACAGTCTGCCAAGCAGGTGATCCTTCAAAAGGTCCGCGAAGCCGAGCGTGACCGCCAGTATGAAGAATTCAAAGATCGCGCAGGCACAATCGTCAACGGTTTGGTCAAACGCGAAGAATACGGCAACGTTATTGTTGATGTGGGCGCTGGCGAAGCGATCCTGCGCCGCAACGAGAAAATCGGCCGCGAATCATACCGCCCGAACGATCGTATCCGCGTTTACATCAAGGATGTCCGTCGCGAGCAGCGCGGCCCACAGATTTTCCTGAGCCGTACAGATCCGCAATTCATGGCTGAATTGTTCAAGATGGAAGTGCCGGAAATCTATGACGGCATCATCGAGATCAAAGCTGTGGCCCGTGATCCGGGCTCGCGCGCTAAGATTGCTGTGATCAGCTATGATAACTCTATCGACCCTGTCGGCGCTTGCGTTGGTATGCGCGGTAGCCGTGTTCAGGCGGTTGTGAACGAACTTCAGGGTGAAAAGATCGACATCATTCCTTGGAATGACGATCAGCCGACTTTCCTTGTGAACGCGTTGCAACCAGCAGAGGTTTCCAAGGTTGTTCTGGATGAGGAAGCCGGCAAGATCGAAGTTGTTGTTCCTGCTGAGCAATTGTCGTTGGCGATTGGCCGTCGCGGTCAAAACGTACGTCTGGCCAGCCAACTGACTGGCCTCGACATTGACATCATGACCGAAGAGCAAGACAGCGCGCGCCGTCAGGCCGAGTTTGAACTGCGCACAAAATTGTTCATGGATAACCTTGATTTGGACGAGTTCTTTGCCCAACTACTGGTATCCGAAGGGTTCACGAACCTAGAAGAAGTCGCCTATGTCGAAGTTGACGAACTGCTGGTGATTGACGGTGTTGACGAAGCGACTGCGGGTGAATTGCAAGCCCGTGCCCGTGATGTTTTGGAAGCACAGAGTAAAGCCGCATTGGATGCCGCACGTGCGTTGGGCGTAGAAGACAGCCTGTTTGAATTTGAGGGCCTTACACCCCAGATGATTGAAGCATTGGCAAAAGACGATGTTAAAACGCTGGAAGATTTCGCAACCTGTGCGGATTGGGAATTGGCGGGTGGCTGGACCACTGTGAACGGTGAACGCGTCAAAGACGAAGGCACATTGGAAGCCTTTGAAATGTCATTGGAAGACGCGCAAAAGCTGATCATGACAGCCCGCGTATTGCTGGGTTGGGTTGATCCCGCTGAACTGGAAGCCGAT---GTTGAAGAAGACATCGACGCA------------GACGAGGAGGCCGAGGCCTGAATGGCACACAAAAAAGCAGGCGGCTCATCCCGTAACGGACGCGACTCAGCTGGTCGTCGTCTTGGCGTTAAAAAATACGGCGGCGAAGTTGTCGTTCCCGGCAACATCATCGTGCGTCAGCGCGGCACTACATTCTGGCCAGGCCAGAACGTAGGCATGGGCAAAGATCACACGATCTTCGCAACTGTTGACGGCAATGTTCAGTTCCACAAGGGCCTGAAAAACCGCACGTTCATCTCCATCGTACCGGCGGCAGAAGCTGCTGAATAAATGGCAAAACGGTGGTATTCGGTCAGTGTCCTTTCCAACTTTGAGAAAAAGATCGCCGAACAGATTCGCACAACGGTTGCTGAACTGGAATTGGAAGATCAGATCGACGAAGTGCTGGTTCCGACCGAAGAAGTGATCGAAATCCGTCGCGGTAAGAAAGTCACAACCGAACGTCGCTTTATGCCGGGGTATGTGCTGGTGCACATGGAGATGTCCGATCGTGGCTATCACCTGATCAACTCGATCAACCGCGTCACAGGGTTCTTGGGCCCACAAGGCCGTCCAATGCCGATGCGCGATGCCGAGGTAACTGCGATCCTTGGCCGTGTTCAAGAGGGCGAAGAAGCACCCCGTACGCTTATTCACTTTGAAATTGGTGAGCGCGTTAAGGTTGCTGACGGTCCGTTCGAAGACTTCGACGGCATGGTTGAGCAAGTTGACGAAGAAAACCAGAAGCTCAAAGTTATGGTGTCCATCTTTGGTCGCGAGACTCCGGTTGAGCTGGACTTCACTCAGGTGAATAAGCAAGTTTAGATGTCATTCTCCCTCGCTATCGTGGGCCGCCCCAATGTGGGCAAATCCACGCTGTTTAATCGTCTTGTTGGCAAACGCCTTGCCTTGGTGGATGACCAGCCCGGCGTCACACGCGACTTGCGTGAAGGGGCCGCGCGGCTTGCTGATTTGCGCTTTACTGTCATTGATACCGCAGGTTTAGAGGATGTCACCGACGACAGCCTACAGGGCCGCATGCGCCGTCTGACAGAACGCGCTGTGGATATGGCGGACGTTTGTCTGTTCATGATTGATGCACGCACAGGCCTGACGCCAACCGATCTCGTGTTTGCAGACATCTTGCGCAAACGCTCCGCCAATGTGATTCTTGCCGCGAACAAAGCCGAAGGCTCTGCCGCTGATGCGGGCGTGATCGAGGCTTATAACCTTGGTTTGGGCGAACCGATCCGCCTTTCCGCCGAACATGGCGAAGGGTTGAACGATCTTTACACCCAGTTGATGCCACTTGCGGATGCTTTTGCTGAAAAAGCCCGCGATGATGCGCCCGAAGTTGATGTGGATGTCAGCGACGAAGATGAAGACGAGGACGCGATGCCCGTCCCAACACGGGCAAAGCCGTTGCAAGTGGCCGTTGTGGGCCGCCCGAATGCAGGTAAATCGACGCTGATCAACCAGATCGTCAAAGAAGACCGTCTGCTGACAGGCCCCGAAGCGGGGATTACCCGCGATGCGATCTCGCTTATGACCGAGTGG------AATGTGCCGATGCGTATCTTTGATACGGCTGGCATGCGTAAAAAGGCCAAGGTCCAAGAAAAGCTGGAAAAACTTAGCGTCAGTGACGGTTTGCGTGCGGTGAAATTCGCCGAAGTTGTTGTGGTGTTGTTAGACGCCGAAATCCCCTTTGAGCAGCAAGATTTGCGCATCGCTGATCTGGCCGAGCGTGAAGGCCGCGCTGTGGTGATTGCGGTGAACAAATGGGACGTCGAAGAAAACCGTCAGGAAAAGCTCAAAGAACTCAAAGAGAGCTTTGAACGCCTGTTGCCGCAGTTGCGCGGTGCGCCGCTGATTACAGTGTCGGCGAAAACGGGCAGGGGGCTGGACCGCCTGCACCAAGCGATTATGCGCGCCTATGAGATGTGGAACCGTCGTGTGACCACGGCGCAATTGAACCGCTGGCTCTCGGGTATGATGGAGGCGCATCCGCCCCCCGCACCCCAAGGTAAGCGCATTAAGATGAAATACATGACACAGGCCAAAACCCGTCCGCCGGGCTTTGTTGTGATGTGTAGCCATCCGGATAAATTGCCCGAAAGCTATAGCCGCTATCTGGTGAACGGTTTGCGGGTTGATTTTGACATGCCCGGCACGCCAATTCGTTTGTGGATGCGGGGGCAGTCAGACGCCAACCCGTTTAAGGGCCGCAAAAAGGCACCGCCGTCCAAGCTGCGTAAACACACAGCCAGCAAACGCAGAGATTAAATGCGGATTGTGTTTATGGGCACACCGGAGTTTTCGGTGACGGTACTGGATGCGCTGGTTGAGGCAGGTCACGAGATTGCGGCCGTTTATTCGCAGCCCCCGCGTCCTGCCGGGCGCGGAAAAAAGGACCGCCCGACGCCGGTTCACGCGCGTGCCGAAGCCTTGGGTCTGGAGGTGCGTACGCCAGTTTCGCTCAAGACCCCCGAGGCGCTAGCAGAGTTTTCCGCGCTGACGGCGGATGTGGCCGTTGTTGTGGCTTATGGCCTGATTCTGCCCCAAGCCATTCTGGATGCGCCCGCACAGGGCTGTTTGAATATTCACGCCAGCCTGCTGCCGCGTTGGCGGGGGGCCGCCCCCATTCACCGTGCTATTATGGCGGGGGATGCGCAAACGGGTGTGTGCATCATGCAGATGGAGGCAGGGCTGGATACGGGCCCTGTTTTGTTGTGTGAAAATCTGGATATCGGCGCTGAAGAAACAACGGCGCAATTGCATGACCGCCTGAGCATTCTGGGCGCGGCGGCTATCGTTGAGGCGTTGGCGAGTTTGGACGATTTGACGCCACAGGTTCAGCCCGAAGAGGGCGTGACTTATGCCGCCAAGATCGACAAAGCCGAGGCGCGGATTGACTGGCACCTACCTGCTGGGCAAGTAGATCGCATGATCCGTGGATTGTCGCCATTTCCGGGGGCGTGGTTTGAACACGGCGGTGTTCGCGTCAAAGTACTGGGGTCGCGGTTGGTAACAGGTGCGGGCGCGGCTGGCATTGTGCTGGACGAGGCGCTGCATGTGGCCTGCGGCGAGGGGGCGGTTGCACTGACCCGCTTGCAAAAGGCGGGCAAAGGGGCGCAGGATGTTGAGGTGTTTCAACGCGGCATGCAGATCGCCGTTGGCGCCAACTTGAACGAAGGATAGATGACCGACACCGCCGCCTACCGCGTACTTGCCCGCAAATACCGCCCCGAAACCTTTGCCGATCTGGTGGGGCAAGAGGCGATGGTGCGTACGCTTAAAAACGCATTTGCGGCAGACCGCATCGCGCAGGCGTTCATCATGACAGGTATTCGCGGCACGGGTAAAACGACCACCGCACGGATCATTGCCAAAGGCATGAACTGTATCGGTCCTGACGGCACCTCTGGCCCCACCACAGAACCTTGCGGCGTCTGTGAACATTGCACCGCCATCATGGAAGGCCGCCATGTCGATGTGATCGAGATGGACGCCGCGTCAAACACCGGCGTCGCCAACATCCGTGAAATCATCGACAGCGTACATTACCGCGCCGCCTCTGCCCGCTACAAAGTCTACATCATCGATGAAGTTCACATGCTGTCCACTGGCGCGTTCAACGCGCTGCTAAAGACATTGGAAGAACCACCAGAGCACGTCAAATTCATCTTTGCGACCACGGAAATTCGCAAAGTCCCCGTCACTGTCCTGTCGCGATGCCAGCGGTTTGACCTGCGCCGAATTGAACCCGAGGTGATGATTGCCCTGCTGCGCAAAATCGCCACGGCCGAAGCGGCTGATATCGCCGATGACGCGCTGGCGCTGATCACCCGCGCTGCCGAAGGCTCGGCCCGCGATGCGACTTCTTTGTTAGACCAAGCCATTAGTCACGGCGCAGGCGAAACAACCGCCATGCAGGTCCGCGCCATGCTGGGCCTTGCAGACCGCGCCCGTGTTCTGGATTTGCTGGATATGATCTTGCGGGGCGATGCCGCCAGCGCCCTCACTGAAATCGGCGCCCAATACGCCGAGGGCGCAGACCCGATGGCCGTACTGCGCGATCTGGCCGAAATCACCCATTGGGTTAGCGTGGTGAAAATCACGCCCGATGCCGCCGAAGACCCGACCATTTCCCCCGAAGAGCGGGACCGTGGCCGCGTTATGGCCGATGCGCTTGCAATCCGTGTTTTGACCCGCTTGTGGCAGATGCTGCTCAAGGCACTGGAGGAAGTTGCAAGCGCGCCCAATGCCATGATGGCTGCCGAAATGGCAATCATCCGCCTCACCCATGTTGCTGATCTGCCCTCGCCAGAAGAATTGCTGCGCACGTTGCAGAAC---ACACCTGCACCA------CCCGCTGGAGGCAACGGCCCCTCA------------------AGCGGCCAAGCAGTGCAGCACAGCGCACCCCAAATGACGTCCACGCCCAACGCTTCGGGCCAAGCCACCGCTCTTGCGGTTGATCCTGCTTCAGCACTCGCAGCGTTTCCAACCTTTGAACATGTCCTTGAGCTGATCCGCCACAACCGTGACGTCAAGCTACTGGTCGAAGTAGAAACGTCTTTGCAACTGGCCGCCTATCAACCGGGCCGGGTGGAATTTGTGCCCACCGACACCGCCCCGCGCGATCTGGCTCAACGGCTTGGCGCAAAACTCCAACTTTGGACAGGCAACAGATGGGCCGTGACAGTCGTCAATACTGGCGGCGCGCCGACCATCGCCTCGCTCCGTGACGCCAAAGACAACGCCATGCGCGCCGAAGCAGAAGCCCACCCGCTCATGATGGCCGTGCTGGCCCAGTTCCCACGGGCGAAAATCACTGCGATCCGCACAGCCGCCGACATTGCCGCCGCTGCCGTATCCGAGGCCCTGCCAGAGGTCGAAGACGAATGGGATCCTTTCGAGGACAGCTGAATGCCCAAGATGAAGACGAAATCGAGCGCAAAGAAGCGCTTTAAGATCTCGGCGACTGGTAAGGTCATCGGCGGTCAGGCTGGTAAACAGCACGGCATGATCAAGCGGAGCAACAAATTCATCCGTAACGCACGCGGCACGACAGCATTGTCAGCGCCAGATGCCAAGATCATCAAGGGCTTTATGCCCTACGACCGCTAAATGAACCTTTTTGCCGATATCCGTACCCTAGTTCTGAGCTCCGTTGACGCGATGGTTGCCTCTGGTGACCTGCCTGCAGGTTTGACCACTGACAACATTACAGCAGAACCACCGCGAGATGCCGCGCATGGGGACATGGCGACAAATGCGGCGATGGTATTGGCAAAGCCGGCAGGCATGAAACCCCGCGACATTGCCGAAAAACTGGCCGTGATTTTGGTGCAAGACCCTCGTGTCACCAGCGCCGATGTCGCCGGACCGGGTTTTTTGAACCTGCGGTTGGCAGATGGCGTGTGGCAGCAAGTGGCCAGCACAGTTCTGGATGCTGTTACGGATTATGGCCGTGGCGATCTGGGCACGGGCAAAACTGTTAACGTCGAATATGTTTCTGCAAACCCGACTGGACCGCTGCATGTGGGCCATACCCGTGGCGCGGTTTTCGGTGATGCGTTGGCCAGCCTGCTGGATTTCGCAGGTTGGGACGTGACCCGCGAATATGTGATCAATGATGGCGGCGGGCAGATTGGCGTGCTCGCACGGTCGGTCTATTTGCGGTATCTTGAGGCACACGGCCAAGAAGTGGCCTTCCCCGATGGGACATATCCCGGTGATTATCTGATCCCCGTTGGGCAAAAGCTGAAAGATAAAGTCGGCGATCAATATATTGACCAACCCGAAGATGTCTGGCTGGGTCCGATTGGCGATTTTGCTACAGATGAGATGATGGACCTGATCCGCGATGATCTGGCGCAGCTTGGTGTCAGGATGGACCGTTTTTTCTCGGAGAAGTCGCTGTACAACACAGGCAAGATCGAAGCCTGTCTGAAAAAGCTCGATGATATGGGCCTGATCTATCGCGGGACTCTTGAGCCGCCAAAAGGCAAGTTGCCTGATGATTATGAGGCCCGCGAACAGACGCTGTTCAAATCCACCGACTTTGGCGATGACCAAGACCGCGCCATCCAAAAACATGACGGCGCATGGACCTATTTCGCCCCTGATATTGCCTACCACAATGATAAGGTCGAGCGCGGCTATGATCAGTTGATCAATGTCTTTGGCGCTGATCATGGCGGTTATGTCAAACGGATGAAAGCCGCAGTTCACGCGTTGTCTGATGGCAAGGTGCCATTGGACATCAAGCTAACACAACTGGTTAAGCTGTTCAAAAACGGTGAAGAATTCAAAATGTCCAAACGGGCGGGGAACTTTGTTCTGCTTAGTGATCTGATCAAGGAAGTGGGCAAGGATGTGACCCGCTTTGTGATGCTGACACGCAAAAATGACGCGCCGCTGGACTTTGATTTTAACAAGGTGATGGAGCAATCGCGCGAAAACCCCGTGTTCTACGTGCAATACGCCCACGCCCGTGTGGCAAGTGTGATGCGCAAAGCTACCGAGGCGGGCATCGACGTGAGCGATGCTGCGCTGAAAGCCGCTGACCTGAGCAAGCTGGATCACACCGCCGAACTGGCCTTGCTGCGCAAAGTTGCGGAATGGCCCCGTCTGGTTGAAACTGCGGCGCGCAGCAACGAGCCGCACCGCATCGCCTTTTACCTCTATGAATTGGCGGGCGATTTGCATGGTTTCTGGAACCTTGGCAACGCCGAGACAGGTCTGCGGTTCATCCAAGAGGACGATCCTGCAACATCACAAGCGAAAATCGCCCTTGCACGCTCCGTTGCGATTGTAATTGCGGCTGGCTTGGGTATCCTTGGCGTAACTCCTGCGGAAGAGATGCGTTAAATGAGCACAATAGACAACCTTCCCTCCTTGCGTCAGGTAATTGACGATCATGGCCTTCAAGCACGTAAATCCCTTGGCCAGAACTTTTTGCTGGACCTCAACCTGACGGCAAAAATTGCCCGCCAAGCGGGGGATTTGACACAATGTGATGTGCTGGAAATTGGCCCCGGTCCCGGTGGCTTGACACGTGGATTACTGGCCGAGGGAGCGCGCCGTGTGCTGGCAATTGAAAAAGACAGCCGCTGCATGCCTGCGCTGGCCGAAATTGCCGCCGCCTATCCAGACCGCCTTCAGGTCATCGACGGGGACGCACTAGAGGTGAACCCCCTAGCCCATCTAACCCCGCCGATCCGCGTCGCCGCCAATCTGCCCTATAACATCGGCACGGAATTGCTGGTGCGCTGGCTAACCCCGCCTGAATGGCCCCCGTTCTGGCAGAGCCTGACCCTGATGTTCCAACGTGAAGTCGCAGAACGCATTGTCGCGGCACCAGGGTCCAAGGCTTACGGGCGTCTTGCCCTGTTGGCGCAATGGCGCGCCGATGCAAAGATCGTGATCAACCTGCCACCCGAAGCCTTCAGCCCGCCGCCAAAAGTGTCCAGTGCCGTGGTCCACCTGACCGCCCTGCCCGAGCCGCGCTTTCCTGCTGATGCTGCAATCCTGTCGCGTACGGTTGCTGCCGCATTCAACCAGCGCCGCAAGATGCTGCGTGCAGCCTTGAAAGGCACGACCCCAGACATCGAGGACCGCCTGATCGCCGCAGGGCTAAAGCCAACGGACCGCGCAGAGCAAATATCACTGGAAGGATTTTGTGCGCTGGCACGTGAGATCGCTAAGTCCTGAATGAACCTGATCGCAGAAATCGAGGCGGAACATATCGCCGAACTCGCCAAGGAAATCCCTGATTTCCGCGCCGGTGATACAATTCGCGTTGGCTTTAAAGTCACCGAAGGTACACGTACCCGCGTACAGAACTACGAAGGTGTTTGCATCAGCCGCAAGCACGGCAAAGGCATCGCCGGCGCATTCACAGTTCGCAAAATCTCATTCGGCGAAGGTGTAGAGCGCGTATTCCCGCTGCACTCCACCAACATCGACAGCATCACCGTGGTTCGCCGTGGTCGCGTGCGTCGCGCCAAACTGTATTACTTGCGTGAGCGTCGTGGTAAATCCGCACGTATCATCGAGAACACACACTACAAGCCGCTCAAGGGG---------TAAATGGCAAACAGCAAAAGACAACTGTTCATCAAACGCCGCCTGCGCGTTCGGAACAAACTTCGTCGCACGAACCGTGGGCGCATGCGCCTCAGCGTGCACCGCTCCAACAAGAACATCAGCGTTCAGCTGATCGACGATGTGAACGGCGTTACAGTCGCTTCCGCCTCGTCGCTTGAAAAAGATCTTGGCGTTGTGGGTAAAAACAACATCGAAGCAGCCACAAAAGTAGGCGCGGCAATTGCCGAGCGTGCGAAAAAGGCCGGTGTTGAAGTTGCCTATTTCGATCGCGGCGGTTTCCTCTTCCACGGTAAAGTGAAGGCCTTGGCCGACGCAGCCCGTGAAGGTGGTTTGAAGATCTAAATGTTTGCGGTCCTGAAAACAGGCGGTAAGCAATATAAAGTTCAAGCGGGCGATATGCTCCGTGTGGAACGTATTGCGGCATCTGCCGGTGAGACAGTTCAATTCAACGAAGTGCTGATGCTGGGTGGCGATAACCCAACTGTTGGCGCGCCAATGATCGAAGACGCCGGCGTTCAAGCCGAAGTGGTTGATCAGATCAAAGGCGAAAAAGTCATCCACTTCGTTAAACGTCGTCGGAAGCACTCTTCCAAGCGTACAAAAGGCCACCGTCAGAAGCTGACATTGGTTAAGATCACCGAGATCCTCGCATCTGGCGCAGGTAAATCCGGCGTTGCAGCTGCAATCGGTACAGGTTCCGTTTCTGCGGCAGCCGTTGCTGCC------------AAAGCAGCCAAGCCTGCTAAAGCCGAAGCG---------CCTAAAGCAGAG------AAAGCTGAAGCCAAAGCGAAAAAAGCTGCTAAAGCTGAA---GGC---GATGATCTGTCCGAAATCTCTGGCGTTGGTCCAGTTATCGTGGGCAAACTGAACGATGCGGGTATCACCACATTCGCTCAGATCGCTGCATGGACTGATGCAGACGTAGAAGAGATCGAAGAGAAACTGTCGTTCAAAGGTCGTGTCGGTCGTGAAGACTGGATTGCACAGGCCAAAGTTCTGGCAAAAGGCTAAATGTCCCGTACAAAAGGTGGTACAGTTACCCACGCCCGTCACCGTAAGGTCGTCAAGGCAGCAAAAGGTTATTACGGCCGTCGCAAGAGTACCTTTAAGGTCGCGCGTCAGGCAGTAGACAAAGCCAACCAATACGCAACCCGTGACCGTAAGGTTCGCAAGCGGAACTTCCGCGCATTGTGGATCCAGCGTATCAACGCGGCTGTTCGCGCCCATGATGCAGAGCTGACATATTCACGCTTCATCAATGGTCTGAACCTTGCCGGTATCGAAGTTGACCGCAAAGTTCTGGCTGATCTGGCCGTGAACGAACCAGAAGCATTTACAGCGATTGTAAAGCAAGCTCAGGCGTCGCTGGCAGCCTAAATGCAGGTCAAAGAGACGCTTAACGAAGGTCTGAAACGCGGCTATACAATCAACATCACAGCAGCCGAGCTGGATGTGAAAGTTGATGAAAAGCTGAAAGAAGCCCAGTCCGAAGTCGAGATGAAGGGTTTCCGTAAAGGTAAAGTACCAATGGCGCTGCTGAAAAAGCAGTTTGGTCCAAAGGTTCTGGGCGAAGCGATGCAAGAAGCCGTTGATGGCGCGATGAACGACCATTTTGAGTCGACCGGTGACCGTCCTGCGATGCAGCCTGACGTTAAAATGACCAACGATGACTGGAAAGAGGGCGACGACGTCGAAGTCGAAATGTCCTACGAAAAGCTGCCAGCGATTCCAGACGTTGACCTGAGCAAAATCTCTCTTGAGAAAATGGTTGTTAAAGCAGACGAAGCTTCCATTGATGAAGCTCTGGCATCTTTGGCTGAAACAGCTCAAGATTTCAAAGCGCGCAAAAAAGGCTCCAAAGCCAAAGACGGTGATCAAATCGTTCTGGACTTTGTTGGTAAAGTTGACGGCGAAGCCTTTGAAGGTGGCGCGGCAGAAGATTACCCGCTGGTACTCGGCTCCAACTCTTTCATCCCCGGTTTTGAAGAGCAGCTGGTTGGCGTGAAAGCCGAAGAAGAAAAAGACGTCACAGTGAACTTCCCTGATGAATATCAGGCAGAGCACCTTGCCGGCAAAGAGGCTGTTTTCTCTTGCACCATCAAAGAAGTTAAAGAGCCTGTAGCGGCTGAAATTAACGACGAGATGGCGAAGAAATTCGGTGCCGAAGACCTTGCTGCGCTGAAAGTGCAGATTGGTGAGCGTCTGGAAGCCGAATATGTTGGCGCGTCCCGCGCTGTCATGAAGCGCGGTCTGCTCGACGCGCTGGACGGTCTGGTTGACTTTGATCTGCCACCTTCCTTGCTGGATGCAGAAGCAGGCCAGATTGCGCACCAGCTGTGGCATGAAGATAACCCAGAAGTCGAAGGCCACGATCACCCCGAGATCGAAACCACAGACGAGCACAAGAAACTGGCCGCACGTCGCGTTCGTTTGGGCCTGTTGTTGGCCGAACTGGGACAGAAAGCCGAAGTGGAAGTCACTGACGCAGAAATGACCCAAGCGATCATGAACCAAGCGCGCCAGTACCCGGGTCAGGAACGTCAGTTCTTTGAGTTCGTGCAGCAAAACCAGCAGATGCAACAGCAGATGCGTGCGCCGATCTTTGAAGACAAAGTTGTCGACTATGTGTTTGAGCAAGCCACTGTTGCAGAGAAAGAAGTCTCTAAAGACGATCTGCAAAAGGCTGTTGAAGCACTGGAAGAAGAATAAATGAAACTTCACGAACTTTCAGATAATGCAGGCGCAACGAAACCACGCAAACGCGTAGGCCGTGGTCCTGGTTCCGGCACCGGTAAAATGGGTGGCCGTGGTATCAAAGGTCAAAAATCCCGCTCAGGTGTGGCGATCAAAGGGTACGAAGGCGGCCAGATGCCCCTCTACCAACGTCTGCCAAAGCGTGGCTTTACCAAGCCGAACCGCAAAACATACTCTGCTCTGAACCTGGGTCTGATCCAGAAGTTCGTTGACGCAGGTAAATTGGATATTTCTGCTGTAATCACCGAAGATGCACTGGTTGCATGCGGTGTTCTGCGTCGCAAGCGTGACGGCATCCGTATCCTTGCAAAAGGCGACGTGACATCCAAGCTGAACCTTGACGTAACTGGCGCATCAAAATCTGCAATCGAAGCAGTTGAAAAAGCAGGCGGCAGCCTGACTGTCAAAGCTGCGGCGGCTGTTGAAGCGTCCGAATAAATGATCCAGATGCAGACCAACTTGGATGTTGCTGACAACAGTGGCGCTCGCCGCGTTCAGTGCATCAAGGTCCTGGGTGGTTCCAAGCGTAAGTACGCATCCGTGGGCGACGTTATTGTCGTCTCGGTAAAGGAAGCCATTCCACGCGGCCGTGTAAAGAAGGGTGACGTGCGTAAAGCCGTTGTCGTTCGTACCGCTAAAGAAGTTCGTCGTGACGATGGCACAGCCATTCGTTTTGACCGCAACGCAGCTGTTATCTTGAATAACAACAACGAGCCAGTTGGCACCCGTATCTTCGGGCCAGTTGTTCGTGAATTGCGCGGCAAAAACTTCATGAAAATCATCTCACTCGCTCCGGAGGTGCTGTAAATGCGTCACGCACGTGGATACCGCCGTCTTAACCGCACACATGAGCACCGCAAGGCGCTCTGGGCCAACATGGCCGGCTCGCTGATTGAGCATGAGCAAATCAAGACAACATTGCCAAAAGCAAAAGAATTGCGCCCGATCATCGAGAAGATGATCACGCTCGCGAAACGTGGCGATTTGCACGCCCGTCGTCAGGCCCGCGCACGTCTGAAAGAAGATCAGTACGTTACAAAATTGTTCGACATCCTCGGACCACGCTACAAAGACCGCCAAGGTGGTTACGTGCGCGTTCTGAAAGCGGGCTTCCGCTATGGTGACATGGCGCCTATGGCGATCATCGAATTCGTTGACCGCGACCGCGACGCCAAAGGCGCAGCTGACAAAGCCCGCGTTGCAGCAGAAGAAGCCGCAGAA---TAAATGCTACAGCCAAAACGTACGAAATTCCGTAAGCAGTTCAAAGGCTCCATCAAGGGCCTTGCGAAGGGCGGGTCTGACCTGAACTTCGGCACTTACGGCCTCAAAGCAGTTGAGCCTGAGCGTGTAACAGCGCGTCAGATCGAAGCTGCACGTCGTGCCATGACGCGTCACATGAAGCGTCAAGGCCGTGTCTGGATCCGCATTTTCCCAGATGTACCCGTAACCTCCAAGCCCGTCGAAGTTCGTATGGGTAAAGGTAAAGGTTCCGTAGACTTCTGGGCCTGTAAAGTGAAACCAGGTCGCGTGATGTTTGAAATCGATGGCGTCAACGACGACATCGCCCGTGAGGCCCTGCGCCTTGCTGCGATGAAGCTGCCGATCAAAACACGCGTGATTGTGCGCGAAGACTGGTAAATGACGACACTCAAGCTGCACAACACAAAGACCCGCAAGCGCGAGGAATTTGTGCCGATTGATCCGCAAAACGTGCGGATGTATGTCTGTGGCCCCACGGTGTATGACCGCGCCCATATTGGTAATGCGCGGCCTGTGATTGTGTTTGACGTGCTCAACCGCCTGCTGCGTCACGTATATGGGGAAAGCAACGTCACCTATGTGCGCAACTTCACCGATATCGACGACAAAATTAACGCCCGCGCGGCGCGGGATGGCAAAGACATCTCCGAGATCACAGCGCAGACGACGCAGTGGTTCCTTGACGATATGGCGGCTGTTGGCGCGATAGAGCCCGATCACATGCCCCGCGCCACGGCCTATGTGCCGCAAATGATCGCAATGATCGAAGACCTGATCGCCAAAGGCCACGCTTATGCGGCAGAGGGCCATGCGCTGTTTGCCGTAGATAGCTATAAGGACTACGGTGCGCTGTCTGGGCGCAGCACTGACGATATGATTGCAGGCGCACGGGTAGAGATCGCCCCGTACAAGCGCAATCCGATGGATTTTGTCCTCTGGAAACCCTCGGATGCCGACACGCCGGGATGGGATAGCCCGTGGGGCCGTGGGCGTCCGGGCTGGCATATCGAATGCTCTGCCATGGCGCATGAGCTGCTGGGCACACATTTTGACATCCACGGTGGTGGAAATGACCTGATGTTCCCGCACCATGAGAACGAGATCGCCCAAAGCTGCTGCGCGGGG------GATGATTTTGCCAATGTCTGGCTGCACAATGAGATGTTGCAGGTAGAGGGCAAGAAGATGTCCAAATCGCTGGGTAACTTCTTCACCGCGCGTGATTTACTAGACAAGGGTGTACCGGGAGAGGTGATCCGCTTTGTGATGCTGTCGACGCATTATCGCAAGCCGATGGATTGGACTGAGAAAAAGGCACAAGAGGCATCTCGGTCATTGAAAAAATGGCGGGACCTTACCGCAGATATTGAAGCCGCACCGTCAATTCCGGCGGTGGTTTTGACTACGCTTTGCGATGACTTGAATACGGCTGGCGTCCTCACACTGCTACATGAATTTGCAAGTAATGGCGATCTGGCCTCCTTGAAGGCGAGCGCGCAGCTATTGGGACTGCTTACAGAGGAATTAGGGGGCTGGACTGAACGCGGCGGTGCTCTTTTGGACGGTTGGACTGAGAGACTTACCGTTGCGCGGGAAAAAGCGATGGAAACGAAAGACTTTGTAGAAGTTGATCGGATCAAGTTACTTCTGACTGACGCTGGTGTGAAAGTTCAAATGGGTAAAGAGGGGATCGTCTTGGCTGCTGGTCCTGAGGTTGATCTGGCTAAACTTGAGGCCCTCAAATGAATGGCACAGGTAAAATCATCATCCAAATCCGACCCTAACTATAAGGTCATCGCAGAAAACCGCCGCGCGCGGTTTGATTATGCAATCGAGGACGATGTCGAATGCGGCATTATTCTGGAAGGGTCCGAGGTCAAGAGCCTGCGTATGGGCGGCTCTAACATCGCCGAGAGCTATGCAGCGGTGGAGGATGGTGAACTGTGGTTGGTGAACAGCTACATCGCGCCCTACAAACAGGCCAAGACATTTGGCCATGAAGAGCGCCGTCGCCGCAAGCTGCTGGTCAGCCGCAAGCAACTGGTAGAGATGTGGAACGCTACTCAGCGTAAGGGGATGACCCTTGTGCCGTTGGTGATGTATTTCAACCATCGCGGCAAGGCCAAGATCAAAATCGGCATCGCCAAAGGTAAACAGCTGCACGATAAGCGTGCGGACTCTGCCAAACGGGATTGGTCACGCCAGAAGTCACGCCTGCTTAAAGAACACAGCTAAATGAGCGCGAAACCAGAACATTACGACGTGATTCGCAAGCCAATCATCACCGAAAAAGCAACAATGGCTTCCGAGCAAAACGCTGTTGTTTTTGAAGTAGCGATCGACAGCAATAAGCCAATGATCAAAGAAGCTGTTGAAGCTCTCTTTGGTGTCAAAGTGAAGGCCGTGAACACGTCCATCACCAAAGGCAAAGTAAAGCGCTTCCGCGGTCAGCTGGGCACACGTAAAGACGTGAAAAAAGCCTATGTGACGCTGGAAGAAGGCAACACGATCGACGTATCCACCGGTCTCTAAATGAGCAAGGATAAAAATCCCCGCCGCGTGGCCGACAATGAAGCACGTGCAAAACTGCGCATGTTGAAAACGTCCCCGCAGAAACTGAACCTCGTTGCGGCAATGATCCGTGGCAAGAAAGTGGACAAGGCCCTGACGGACCTGACGTTCTCCAAAAAGCGTGTCGCTATCGACGTGAAGAAATGCCTTCAGTCCGCGATCGCCAATGGCGAAAACAACCACAATCTTGATGTGGACGAACTGGTCGTGGCAGAAGCCTATGTCGGCAAAAACATGACACTCAAGCGTGGTCGTCCACGTGCGCGTGGCCGTTTCGGCAAGATCATGAAGCCGTTTGCGGAAATCACAATCGTCGTGCGTCAGGTTGAGGAGCAAGCATAAATGGTATCTGCCGTCGAGAATATGGCCGCTAACTCAAGTTGGTCCGCCTTTGGCAAGGCAACCGACCTGCGCCACCGCATTTTGTTCACGCTGGGCTTGCTCATCGTCTACCGTCTGGGGACATTTATCCCCGTGCCCGGTATCGACGGTGCCGCATTACGCCAGTTTATGGATAGTGCGGGGCAGGGCATTGGCGGCATGGTTTCCATGTTCACCGGTGGGGCGCTTGGCCGTATGGGTATCTTTGCGCTTGGTATCATGCCTTACATCTCCGCTTCGATTGTTGTGCAGCTTTTGACATCTATGGTGCCATCGCTGGAGCAGCTGAAGAAAGAGGGCGAACAAGGCCGTAAGAAGATCAACCAATATACCCGCTGGGGTACGGTGGCGCTTGCCACGGTGCAGTCCTACGGTTTGGCTGTGTCGCTTGAGGCAGGTGATATCGCGTCTGATCCGGGCATGTATTTCCGCATTGCGTGTATGATCACGCTGATCGGCGGCACGATGTTCCTGATGTGGCTGGGTGAGCAGATCACAGCACGCGGCATCGGCAACGGCATCTCTTTGATCATTTTCGTCGGCATCATCGCCGAAGTCCCCGCCGCCATCGCACAGTTCTTTGTGTCTGGTCGTTCCGGTGCAATCAGCCCTGCGGTGATTGTCGCTGTGCTGGCTATGGTGGTGCTAACGATCATGTTTGTGGTGTTTATGGAGCGCGCCCTGCGTAAGATTCACATCCAATACCCGCGCCGTCAGGTCGGTATGAAGGTCTATGACGGTGGCTCCAGCCACTTGCCGATCAAGGTTAACCCAGCAGGTGTTATCCCCGCGATCTTCGCAAGCTCGCTGCTGCTGTTGCCGATCACGATCAGCACGTTCTCTGGCGATACAACCAGCCCCGTTATGTCTTGGTTGCTTGCCAACTTCGGTCCGGGCCAGCCGCTGTATCTGCTGTTCTTCATCGCGATGATTGTTTTCTTTGCGTATTTCTACACTTTCAACGTCTCGTTCAAACCCGATGACGTTGCGGATAACCTGAAAAACCAAAACGGTTTTGTACCGGGCATTCGCCCCGGCAAAAAGACCGCCGAATACCTTGAGTACGTCACCAACCGTATTCTTGTCTTGGGCTCTGCTTATCTGGCAGCGGTTTGTATTCTTCCTGAAATTCTGCGTGGTCAGTTCGCTGTACCTTTCTACTTTGGTGGCACATCCGTGTTGATCATTGTTTCTGTAACGATGGATACGATCCAGCAGGTACAAAGCCATCTTTTGGCACATCAATATGAAGGTCTTCTGGAAAAGTCGCAATTGCGCGGCAAATCCGGCAAAGGCCGCAAAAAACGGAGCCCGGCGCGCAAATGAATGAAGTTCCTCGATCTCGCCAAAGTTTACATCCGCTCCGGTGCCGGTGGCGGCGGCTGTATCTCGTTTCGGCGGGAAAAATACATCGAATACGGCGGCCCTGATGGCGGTGATGGCGGCGGCGGCGGCACAGTTTGGGCTGTGGCTGTGGACGGTCTGAACACCTTGATCGATTTTCGTTACCAACAGCACTTTTTTGCCAAAAACGGTCAGCCGGGCATGGGTAAGCAGCGTACAGGTAAAGATGGCGATGACATCATCTTGCGTGTTCCTGTAGGCACCGAAATTCTGGACGAAGACCAAGAAACCGTTCTTGCCGATATGACCGAACTGGGCCAGCGCGTAGAGTTGGCACGTGGAGGCAACGGTGGTTGGGGTAACCTGCACTTTAAATCCGCCACCAACCAAGCGCCACGCCGTTCTAACCCCGGTCAAGACGGGGTCGAGCGGACGTTGTGGCTGCGGCTCAAGCTGATTGCGGATGTGGGCCTGTTGGGCCTACCCAATGCCGGTAAATCAACCTTTTTGGCAGCGACTTCAAATGCACGGCCTAAAATTGCGGATTATCCGTTTACCACACTTCACCCGAACTTGGGCGTGGTAGGTGTTGATAACACTGAATTTGTTGTTGCCGACATCCCCGGTTTGATTGAGGGAGCCCATGACGGGCGTGGACTGGGTCATCGCTTTTTGGGCCATGTTGAGCGGTGCGCCGTGCTGTTGCATTTGGTCGATGGAACATCCGAGACGATCACCGAGGATTACCAGACCATCATCGGTGAACTGGAAGCCTATGGCGGAGAGTTGGCCGACAGGCCCCGTGTGACGGTCTTGAACAAGATTGATGCTCTGGATGAGGAGACGCTGGCAAGTGCTTCTGCTGAATTGAGAAAAGCGTGTTCTGGAGAAGTGATGTTGATGTCGGGCGTGGCCAAGACAAACACCGTCGAAGTCCTGCGTGCCCTGCGTGCCGAGATCGACGACAACCGTCTTCGCCAGACCAAAGGCGAGGAGGAAGCGCCTTGGCAACCTTAAATGGCTGCTAAGCTGAAAAAAGGTGACACTGTCATCGTATTGGCCGGTAAGGACAAGGGCAAAGAAGGCACAATCGCCTCCGTTGATCCCAAGACCAACAAAGCTGTTGTAGACGGTGTTAACGTTTACCTCCGCGCCACACGCCAAACACAAACATCTCAGGGCGGTCGCATCCCTAAATCGATGCCAATCGACCTGAGCAACCTCGCAATCAAGGATGCCAACGGCAAGCCTTCACGCGTTGGTTTCAAAATGGACGGCGAAAACAAAGTCCGTTTCGCCAAGACAACAGGGGACGTAATC------TGAATGTTGCGCTCAGGCGTTATTGCAAAAAAGATGGGCATGACCCGTCTGTTCATGGAAGACGGTAAGCAGATTCCTGTAACCGTTCTTCAGCTCGATAACCTTCAGGTTGTTGCACAGCGTACAATCGAACGGGACGGCTATGTAGCTGTTCAGCTCGGTGCAGGTACAGCAAAAGTGAAACGTACATCGCAAGCGATGCGCGGTCACTTTGCAGCAGCAAAGGTTGAACCAAAGCGTAAGGTTGTTGAATTCCGCGTTGACGCGGATGCGATGCTTCCAGTTGGCGAAGAAATTATCGCGGATCACTACTTTGCAGGTCAATACGTTGACGTTGCGGGTACTTCCATCGGTAAGGGCTTTCAGGGCGCCATGAAGCGTCACAACTTTGGCGGTCTGCGTGCGACACACGGTGTTTCCGTGTCTCACCGTTCACACGGCTCCACGGGCCAGTGTCAGGATCCAGGTAAAGTTTTCAAAGGCAAGAAAATGGCTGGTCACATGGGCTCCGCTCGCGTGACAACGCAGAACCTTGAGGTTGTTAAAACCGATACGGCGCGTGGCTTGATCATGGTCAAAGGCGCGGTTCCTGGTTCCAAAGGTGGCTGGGTGACTGTTAAGGATGCGGTTAAAAAACCGTTCCCTGACGCAGCAATCGTTCCTGGCGCTCTTGCATCCGCTGCACGTGAAGCCGCGAAAGCAGCCGAAGAAGCAGCCGCAGCCGCAGCAGCCGAGGCAGAAGCCGCAGCAGTA------------GAAGCTGCCGCAGCCGAGCAGGCCGCAATGGAAGCAGCAGAAAAC---------GCAGAAGCTACGCCAGATGCTGTAGCCGAAGCA---GAAAAGAAAGAAGGTGACGCATGAATGGCACTCAAGTCGTACAAACCGACGACGCCAGGCCAGCGTGGGCTGGTACTGATCGACCGTTCGGAGCTTTGGAAAGGCCGCCCAGTAAAGGCCCTCACTGAGGGACTTCACAAGCATGGCGGACGGAACAACACCGGACGGATCACAATGCGTCGCAAAGGTGGTGGTGCAAAGCGCCTCTACCGTATCGTAGATTTCAAACGCAACAAACTGGACGTCACCGCGACGATCATGCGTATTGAATATGACCCTAACCGGACTGCGTTTATCGCGCTGGTTAAATACGAAGATGGTGAGCAGGCCTATATCCTCGCGCCTCAGCGTATCGCAATCGGTGACCAAGTCGTGGCATCCGCCAAGGCCGACATCAAACCCGGTAACGCAATGCCTTTCTCCGGCATGCCAATCGGTACAATCATTCACAACATCGAGATGAAGCCAGGCAAAGGCGGTCAGATCGCCCGTGCAGCCGGTACTTACGCCCAATTCGTTGGTCGTGATGGTGGCTACGCACAGATTCGTCTGTCATCCGGTGAATTGCGTCTTGTCCGTCAGGAATGCATGGCCACCGTCGGTGCCGTGTCCAACCCTGACAACTCCAACCAGAACTACGGTAAAGCGGGCCGCATGCGTCACAAGGGCGTTCGCCCAAGTGTTCGTGGTGTCGTTATGAACCCGATCGATCACCCGCATGGTGGTGGTGAAGGTCGTACGTCTGGTGGTCGTCATCCGGTTACTCCATGGGGTAAACCAACTAAGGGTGCCAAGACACGTAACAAGAAAAAAGCGTCAAGTGCTCTGATCATCAGATCGCGCCACGCCAAGAAGAAGGGGCGTTAAATGCTCGATACTGCAGCTTATACACCCCGCCTCAAGGCCGACTTCAAAAACCGCATTCGTGCGGCTTTGAAAGAAGAATTCGGCTATACCAACGACATGCAGATCCCGCGTTTGGACAAGATCGTCCTGAACATCGGTTGTGGCGCAGAAGCTGTCCGTGACAGCAAAAAAGCCAAATCCGCTCAGGAAGACCTGACACTGATCGCAGGCCAGAAAGCACTGACAACCATCGCCAAGAAATCCATTGCGGGTTTCCGGGTACGGGAAGAAATGCCACTGGGCGCGAAGGTTACACTTCGCGGTGACCGGATGTACGAATTCCTTGATCGTCTGATCACAGTTGCAATGCCACGTATCCGCGACTTCCGCGGCATCAACGGCAAATCTTTTGATGGCAACGGCAACTATGCCATGGGCCTGAAAGAGCACCTCGTGTTCCCCGAAATCGATTTTGATAAAATCGACGAAAACTGGGGCATGGACATCGTAATCGCTACGACAGCGAAAACCGACGCCGAAGCGAAGGCAATGTTGAAACTGTTCAACATGCCCTTCAACTCATAAATGAAACTAGATGTCATCAATCTCGAAGGCGCTTCCGCCGGATCGATCGACCTGGACGAGGCCCTGTTTGGTCTTGAGCCACGTGCCGACATCCTGCACCGTGTTGTGCGCTGGCAGCGTAACAACGCGCAGGCCGGTACTCACAAGGTCAAGACGCGTCGTGAAGTCAGCTATTCCACCAAAAAGATCTATCGCCAAAAAGGCACCGGTGGCGCACGCCACGGCGCTCGCTCGGCACCGATCTTTCGTGGGGGCGGTATCTACAAGGGTCCTACACCCCGTAGCCACGGCCACGAACTGACTAAGAAGTTCCGTAAATTGGGTCTGCGCATGGCGCTCTCCGCAAAGGCAAAAGCCGGCGCGTTGGTCATCATCGACGATGCAGCATCCAACGGTAAAACATCTGCTCTGGCCAAACAGGTTAAATCCTTGGGTTGGAAGCGGGCGTTGATCATTGACGGTGCATCCGTGAATGAGAACTTCCTGCAAGCCGCGCGCAACATTGAAGGTTTGGACATCCTGCCAACAATGGGCGCTAACGTATATGATATCCTGAAGCGTGACACACTCGTGATCACAAAAGCAGGCATCGAAGCACTGGAGGCCCGTTTGAAATGAAACGAAAATGTCATCCTGATCATCCACCTCATCCTCGCGCTTGGCCTCATCGCCGTAGTGCTGTTGCAACGCTCCGAAGGTGGCGGGCTTGGCATG---GGCGGCGGCGGCGGTGCAAATTCCGGCCGCCCAGCAGCCAGCCCGATGGCGAAAGTAACTTGGATTTTGGGCCTTGCCTTTGTGGTGACGTCTATCGCGCTGACAATCACAAGCGCACAAAAATCCGCAGGGGTTTCCGTATTGGACCGCCTGACAGATTCGCCTCCCGCGCTGGAGCAGAGCGATCCCGCTGCGGCACAAGGCTTGGGCGATCTTTTGCCACCCACACAGGGCGACAACGCACCGCTGGTCCCAACAGTCGACTAAGTGGCGTTTTTCAAGAAACTCAAAGACCGTTTGTTCACCTCCTCGTCCAAGATTGATGAAGGGTTAGAGGCGATTGTCAGTGATGGCGGTCAGGCCGATGTGCCTGCGGTA---------------------GAAACCCCAGCGCAGGTTGTGGAGGTTACACCCGAAGCGGCGCAAGAGGCAGCTCAGGAGGTCCCGGACTTAGACACGGCAGTGCATGCGGCACTTGAAGAAAAACAGCGCCAAGAGGCGCAAGCCGAGGCTGTAGAGGAACCGCAAGCG---------------------------------------CCAGAGCCCACG---------GTAGAACCGCTGCGTACGACACTTACACCTGTTGCACCTGTGCTGGAGGAAGCGCCAACG------GAGCCCGTTGCCAAACGCGGTGTTCTGGGCCGTCTGATGGGGCGCGGTGCGGATGGCGAAGTTGTACGCCGCGAGTTGGATGACGCGATGTTGGAGCAGCTAGAAGAGCTGTTGATTGCGTCGGATATGGGGGTTGATACGGCCTTGCGGGTCACGTCCAACATGGCCGAAGGACGTTTGGGTAAAAAATTGTCTGTGCAGGAAATCAAAACCCTTATGGCAGATGAAATTGCGCGAATCATGGAACCCGTGGCCAAGCCGCTGCCGCTTTATGCCAAAACACCTCAGGTGGTTCTGGTTGTAGGCGTGAACGGATCGGGCAAAACCACAACCATCGGCAAGCTGGCCAGCCAGTTTCGTGGTGCGGGCAAAAAAGTGGTGATCGCAGCAGGGGATACGTTCCGTGCTGCCGCTGTTGAGCAATTGCAAGTTTGGGGCGAGCGCGCAGGCGTACCTGTGTTGACCGCGCCCCATGGCAGTGACCCTGCCAGTCTGGCCTTTGACGCGATGGTGCGTGCCGAGGCGGATGGTGCTGACCTGTTGATGATCGACACGGCAGGGCGGTTGCAAAATCGCGGCGATCTGATGGAAGAACTGGCCAAAATTGTAAGGGTTATTCGCAAGAAAGACCCCGAAGCGCCGCATAACACTTTGCTGGTGCTGGATGCGACCACGGGCCAAAACGCCATTAATCAAGTGAAGGTGTTCCAAGACATCTCGGATGTGTCGGGTCTTGTTATGACCAAACTGGACGGCACAGCCAAAGGCGGTGTGTTGGTGTCGCTGGCGGATAAATTCGGCCTGCCGATCCACGCGATTGGCGTCGGCGAACAGATTGATGATCTGCAACCCTTTGACCCGCAAGAATTTGCAGACGCGTTGACAGGGTTGGACCGCTAAATGACACGGGACGAATGGGGCAGCGTTAAGCAACGACTTCTTAAAACAGTCGGGCAAAACAACTATACAACTTGGATTGACCCTCTGCTTTTGGGTGCGCTTGATTCCGGCATTGCCACCGTGAATGTCCCGACGACATTCTTTGGTAACTACGTGAGTCAAAATTTCGCTGACCTCATCCTGCATGAGATGCGTGCTTTTGATGATTCCCTATCGCGTTTGAAATTTGAAGTTGCAGGA------------AAAACCGCCAAGCCTGCTACCCGCCAGACAGATGCCATCGCTGCCCCACGTTCAACG---CCAAAAGCGACGACTACATATACTGCCCCGCTGGAACGCCGCTTTTCGTTTGATACTTTTGTTGTCGGGAAGCCGAACGAATTGGCCCATGCGGCTGCCCGCCGAGTTGCCGAAGGTGGCCCTGTATCGTTCAACCCCCTGTTTCTGTATGGCGGCGTTGGCCTTGGCAAAACCCACCTTATGCATGCCATCGCGCAAGAACTTCAAATCCGTAAGCCCGAATTGAACGTGCTCTATCTGTCGGCAGAACAATTCATGTACCGCTTTGTTCAGGCTTTGCGTGACCGCAAGATGATGGATTTCAAAGAAATCTTCCGTACAGTGGATGTGCTGATGGTGGATGACGTTCAGTTCATTGCAGGCAAGGACAGCACGCAAGAAGAGTTTTTCCACACTTTTAACGCGTTAGTGGATCAAAATAAGCAGATCATCATTTCTGCTGACCGCGCCCCGCAAGACATCAAAGACCTTGAAGACCGCGTGAAGTCGCGTCTGCAATGCGGTTTGGTGGTTGATCTTCACCCCACTGACTATGAATTGCGTCTTGGCATCTTGCAGACCAAAGTTGACACGCACCGCGCCTCTTATCCCGAGCTGGCGATGGAGGATGGTATCCTTGAATTCCTCGCACACCGCATCTCGACCAACGTGCGCGTGCTAGAAGGTGCGCTGACGCGGCTGTTTGCCTTTGCCTCACTAGTTGGCCGTAAAATTGATATGGAGCTTACCCAAGATTGTCTGGCTGATGTGCTGCGTGCATCCGAACGGAAAGTCACCGTTGAGGAAATCCAGCGTAAGGTCTCGGATCACTATAACATCCGCTTGTCTGATATGGTCGGCCCCAAACGTCTGCGCAGCTATGCAAGGCCCCGTCAGGTTGCGATGTATTTGTGCAAGCACATGACCAGCCGTTCACTGCCCGAAATCGGGCGTCGGTTTGGCGGCCGCGATCACACCACCGTTATGCACGGGGTGAAACGTATCGAAGAGCTGAAAATCTCGGACGGTCAGATCGCTGAAGATCTTGAACTTTTGCGTCGCGCATTAGAGGCATAAATGCTGGGACTCGGAACACTCACCAAAAAAGTTTTTGGTTCGCCTAATGGTCGCAAGATCAAAGCCGTCCGCCCGCTGGTTGAAAAGATCAACGCGCTAGAGCCAGAGTTTGAGAAACTCTCGGATGAGGCAATCAAGGATAAAACCGAGGAATTGTCCGCCCGTGCTTTGAAAGGCGAAAGCCTTGATGCATTGCTTCCTGAAGCCTTTGCCAACTGCCGTGAGGGTGCGCGCCGTACTTTGGGACTGCGCGCCTTTGATACCCAGTTGATGGGTGCGATTTTCTTGCATCAAGGCAACGTGGCCGAGCAAAAGACAGGTGAAGGTAAAACCCTAACCGCGACATTTGCGGCTTATCTCAATGCGCTTACTCACAAAGGCGTGCACATTGTTACAGTAAACGAATATCTGGTGAAACGTGATGCGGAATGGATGAGCAAAGTCTTTGCTTCGTTGGGTCTCACGACCGGCTATATCATTCCGAACATGCCCGATGATCTAAAGCGTCATGCCTATGAATGCGATATCACCTACGCTACCAACAATGAACTTGGCTTTGACTATCTTCGCGACAATATGAAAGCCGAGCTGAGCGAGATTTTCCAAAAGCAACATAACTTTGCGATTGTGGATGAGGTTGACAGTATCCTGATTGACGAAGCGCGTACGCCTTTGATTATTTCCGGTCCGTCTGATGACCGTTCAGACATGTATCAGACCATTGATACGATTATCCCGACGCTGCTGCCCGAGCATTATGAACTGGATGAGAAAACCCGCGGTGTGACCTTTACCGACGAGGGTATTGAGTTCCTCGAAGAACAATTGCGCGCCCGTGAGTTGTTGGAAGGTGAAGCGACGCTTTATGATCCTGAAAGCACGTCTTTGGTGCATCACGTAAACCAAGGTTTGCGCGCGCATACCCTGTTTGAAAAAGACAAAGACTATATTGTGCGTGACGGCGACGTTGTTTTGATCGATGAATTTACCGGCCGCATGATGGCTGGTCGTCGTTTGGGGGATGGCCTGCATCAGGCGATTGAAGCCAAAGAAGGTGTGAAAATTCAGCCCGAAAACGTCACATTGGCGTCTGTGACCTTCCAAAACTATTTCCGCCTTTACGATAAGCTCGCAGGGATGACAGGCACGGCCCTGACCGAAGCAGATGAATTTATGGAAATCTACGGGTTGGGTGTTGTCGAAGTACCTACCAACGTGCCTGTGGCGCGTCAGGATGCGGATGATGCCGTCTACCGCACTGTTGCAGAGAAATATGCTGCGTTGATCGAAGACACAAAAGAGGCGCACGCCAAAGGCCAGCCTTGCCTTGTGGGGACAACCTCAATCGAGAAGTCCGAGATGTTGAGTAAGCTGCTGGAAAAAGAAGGCATCAAGCATAGTGTTTTGAACGCCCGCCAGCACGAGCAAGAAGCACAAATTGTCGGCGACGCTGGAAAATTCGGTGCCGTGACTATTGCCACCAACATGGCGGGTCGTGGTACTGACATTCAGTTGGGTGGTAACGTTGAGCTGAAAGTTCTGGATGCATTGGCCGCCGATCCCGAGGCCGATCCAGTTGCTGTTCGCGCCCGTATCGAAGCCGAACACGCTGATGAAAAGAAAAAGGTTCTTGAGGCAGGTGGCCTATACGTTCTGGCCTCCGAGCGTCACGAAAGCCGCCGGATTGATAACCAGTTGCGCGGTCGGTCTGGCCGTCAGGGTGATCCGGGACGTTCTTCGTTCTACCTGTCTCTCGAAGATGACCTGATGCGCATTTTCGGTTCTGACCGATTGGAAAAAGTTCTGACATCGTTGGGCCTGAAAGAGGGCGAAGCGATCATCCACCCTTGGGTGAATAAGTCTCTTGAGCGTGCGCAAGCCAAGGTCGAGGGCCGCAACTTTGACATGCGTAAGCAGTTGTTGAAGTTTGACGACGTGATGAACGAACAACGTAAAGTCATCTTCGGGCAACGCCGTGAAATCATGGAATCACAGGATTTGCATGAAATCACCGAAGATATGCGTCATCAGATGGTCGATGATTTGATTGACCAGTATATGCCGGTCAATACTTATGCAGATCAGTGGGACACACAGGGGTTTTACGCCGCTGTGATTGAGCAGTTGGGCGTTGATGTGCCCGTGATTGCATGGTGTGAAGAAGACGGCGTAGATGATGACATCATTCGTGATCGCTTGATCGAAGCAACAGATAAATTGATGGCCGAAAAGACTGAAAAATTCGGTCCTGAAAATATGCGCAACATCGAAAAGCATTTCTTGTTGCAAACGATTGATGCAAAATGGCGTGAGCACCTTTTGACGCTTGAGCACCTGCGCTCGGTTGTGGGTTTCCGCGGGTATGCGCAACGTGATCCGCTGAACGAATATAAAAACGAATCGTTCCAGTTGTTTGAAAGCATGCTGGACAGCTTGCGTTCAGATGTGACGCAGCGCTTGTCACGCATCGAGCCGCCAAGTGAAGAACAACAGCAACGGCTGATGGAGCAGATGTTGGCACAGCAACGGGCGGCGGACGACGCGGTCGATGATGCCGTGGATCAG---------GCCGAAGCGGCAGAAGCC------ATTGACGGGTTCGATGAAAATGACCCTTCTACATGGGGGTCACCCGCAAGGAATGATCTGTGCCCCTGTGGTTCTGGCAAAAAGTTCAAGCACTGCCACGGGCAACTCACCTAAGTGAAGTTATCGGCGGAACTCAAAGCGTTCGAAGGGCGACTGGGCTATCAGTTTGAAAAGCCTAAACTGCTGAACGAAGCTGTAACCCATGCGTCGATGACGACACCCAACCGTGACGATAACCAAAGGTTGGAGTTTCTGGGTGATAGGGTGCTAGGCTTGGTTATGGCCGAAGCCCTCCTGAACCTCGATACTGGCGCATCAGAGGGCCAATTGGCCCCGCGCTTTAACGCCTTGGTGCGCAAAGAAGCCTGTGCTGATGTCGCCCGCGAGATTGATATTGGTGCGGTTCTGCGTTTGGGCCGCTCTGAGATGCTATCGGGGGGGCGGCGCAAGCAAGCACTGCTGGGCGATGCGATGGAAGCTGTGATTGCGGCGGTCTATGTGGATGGCGGGTTTGACGCCGCCCGCGACATGATCATCCGGCTGTGGGGCACGCGGACAACTTCGGTGAAAGAAGATGCCCGTGATGCCAAAACATCATTGCAAGAATGGGCGCAGGCGCGCGGGCTGGAGCCGCCACAATATGTGTTGGAAAAACGTAGCGGCCCAGACCACGCACCGATCTTTACAATAGCCGCACGCCTGTCCACAGGACAGACTGCCAGTGCGACAGCAGGCGCGAAACGCGCCGCAGAACAAGACGCCGCAGCATCGCTGTTGGCGCAATTGGAGCAAGAGATATGAATGGCTAAACTTGGTAAGCGCACAGTCGCAGCTCGCGAAGCATTCGTTGGCAAAGAGAACCTCACAGTTGAAGATGCTGTGGCACTGATCAAAGGTAACGCAAACGCAAAGTTTGACGAAACCATCGAGATCGCAATGAACCTCGGCATTGACCCACGTCATGCTGACCAAATGGTTCGCGGCGTTGTTGGCCTGCCAAACGGCACGGGCAAAGACGTTCGAGTTGCTGTTTTTGCACGCGGCCCCAAAGCCGACGAAGCAAAAGAAGCTGGCGCGGATATCGTAGGCGCAGAAGACCTGATGGAAATCGTTCAGGGCGGCAAGATCGACTTTGATCGTTGCATCGCAACACCTGACATGATGCCTGTTGTTGGTCGTCTGGGCAAAGTGCTCGGCCCACGCAACCTGATGCCAAACCCAAAAGTCGGTACTGTGACTATGGATGTGGCTGCTGCTGTTAAAGCGGCCAAAGGTGGCGAAGTGCAGTTCAAAGCTGAAAAGGGCGGTGTCGTCCATGCAGGCGTTGGCAAAGCATCTTTTGACGCGGCCAAACTGGTTGAAAACATCCGCGCATTTGTAAGCGCAGTTGCCAAAGCACGTCCAACAGGCGCCAAAGGTGCCTATATGACAAAGATCGCATTGAGCTCCACAATGGGCCCGGGCGTGACTGTTTCAGTGGATAACGCTGCATCCGAGTAAATGTCAGAAGATTTTATGCTCGACACCGATGATCTTGATCGGCGCATGAAGGGTGCAATTGCATCCATGCGCACAGAATTTGCATCCCTGCGCACAGGCCGTGGGTCGGCCTCTATGCTGGAACCTGTGATGGTAGATGCCTATGGCCAGATGACACCGATCAATCAGGTTGGCACAGTCAACGTGCCAGAGCCGCGTATGGTAACGGTGAACGTTTGGGATAAAGCTTTGGTTGGTAAGGTCGAAAAGGCCATTCGTGAAAGCGGTTTGGGCATCAACCCACAGCTGAACGGCACAATCATTATGCTGCCGATTCCCGAGTTAAACGAAGAGCGCCGCGCACAGTTGAGCAAAGTTGCGGGCGGTTATGCCGAGAATGCCCGTGTGAGTATCCGTAACGTTCGCCGTGATGGTATGGATCAGATCAAAAAAGCCAAAGCCGATGGCATGTCCGAAGACGATCAAAAGATCTGGGAAGACGAAGTTCAGGAACTGACCAATAAGTTCATCAAAGCTATTGATGAAGGGCTTGAGACGAAACAAGCTGAAATCATGCAGGTTTAAATGGCCAAGAAACTCGTCGGTACGATGAAGTTGCAAGTTAAAGCGGGTCAAGCAAACCCGTCCCCGCCAGTCGGTCCAGCATTGGGTCAACGCGGCATCAACATCATGGAATTCTGTAAGGCGTTCAACGCTAAAACTGCAGATCTGGAGCCAGGTGCGCCTTGCCCAACTGTGATCAGCTACTATCAGGACAAGTCCTTCACAATGGACATCAAGACGCCACCAGCGTCCTACTTCCTGAAGAAAGCTGCCAAAGTGAACTCCGGCGCGAAAACACCAAGCCGTGAAACTGTTGGTACCATCACAGCCAAGCAACTGCGCGAAATCGCAGAAGCAAAAATGGCCGATCTGTCTGCGAATGACGTAGAGCAGGCGATGAAAATCATTATGGGCTCCGCAAAGTCCATGGGCATCGAGGTTAAGTAAATGTTTGAGAATCTCAGCGAACGCCTCTCCGGTGTCTTTGACCGCCTGACCAAACAGGGCGCGCTCTCGGAAGATGACGTAAAAACAGCCCTGCGCGAAGTGCGTGTTGCCCTGCTTGAGGCTGACGTTTCGCTGCCGGTCGCACGTGATTTTGTGAACGCGGTGCAAGAAAAGGCCACTGGCCAAGCCGTCACCAAATCCATCACGCCCGGCCAACAGGTTGTCAAAATCGTTCATGACGCCCTGATCGACACGCTGCGCGGCGAAGGTGAACCGGGTGCGCTGAAAATCGACAGCCCGCCAGCACCGATTCTCATGGTGGGTTTGCAAGGCTCAGGTAAAACCACAACCACGGCCAAACTCGCAAAACGTCTGAAAGAAAAAGACGGCAAGCGTGTGTTGATGGCATCGCTTGACGTGAACCGCCCTGCCGCAATGGAACAGTTGGCGATTCTGGGTGTGCAGATCGGTGTGGACACTCTGCCCATCGTCAAAGGCGAAACGCCCGTCCAGATCGCCAAACGCACCAAGACACAGGCGTCTTTGGGTGGCTATGACGTCTATATGCTGGATACCGCTGGTCGCTTGTCCATCGACGAAGAGTTGATGCAACAAGTCGAAGCCGTGCGCGATGTGGTTACGCCCCGCGAAACACTGTTGGTGGTTGATGGCCTGACAGGCCAAGACGCCGTGCAAACCGCGCAAAACTTTAACGACCGTATCGGTATTTCCGGCGTTGTCCTGACCCGTATGGACGGTGACGGCCGCGGTGGTGCTGCGCTATCGATGCGTGCCGTTACAGGCAAGCCCATCAAATACGTCGGCCTTGGCGAAAAGATGGACTCGCTGGAAACTTTCGAGCCAGAGCGCGTTGCAGGCCGTATCCTTGGCATGGGCGACATTGTTGCGCTGGTTGAGAAAGCCCAAGAAACAATCGAGGCCGAACAAGCCGAAAAGATGATGCGCCGCATGGCGAAGGGTCAGTTCAACATGAACGACCTCAAAATGCAGCTGGAACAGATGATCAAAATGGGCGGCATGCAAGGCATGATGGGCATGATGCCCGGCATGGGCAAAATGGCCAAACAGGTCGAAGACGCCGGTCTTGATGACAAAGTGCTGAAACAACAAATCGCCCTGATCAACTCTATGACCAAAAAAGAGCGCGCCAACCCCGCGCTGCTGCAAGCCAGCCGCAAAAAGCGGATCGCCAAAGGCGCAGGCATGCAGGTGTCTGACCTGAACAAGCTGATGAAAATGCAGCGCCAGATGTCCGACATGATGAAGAAAATGGGCAAA---GGCAAAGGCGGCATGATGAAAGCCGCAATGAAACAGATGATGGGCAAAGGCGGCATGGACCCTGCGGCAATGGCGCAAGGCATGGACCCCAAAGCACTGGAAGCAGCCGCAAAACAAATGGGCGGCAAACTGCCCGGT------------TTGGGCGGCGGCATGGGCCTGCCTGCGGGCCTGTCAGGTTTCGGCAAAAAGAAATGA---------ATGGACCGCCTTGCACAAATCACCGCCCGTTTCGAGTACCTCGAAGCGGCCATGTCCACTGCC------GGTGGCGATATTTCCAAGCTCGCTAAAGAATACAGCGATCTACGCCCTGTGGTGGAGGAAATCTCATCTTACCGCGTGTTGCTAGATAATCTGGAGGGCGCCAAAGAGATGTTGGCCGATCCCGATATGAAGGACATGGCCCGCGAAGAGATCAGCGATATTGAGGCCGCTCTGCCCGCTGCCGAGGCGTCATTGCAATTGGCACTGCTGCCCAAAGATGAGGCAGATGCGCGGCCCGCGATGTTGGAAATTCGCCCGGGTACAGGCGGTGACGAAGCAGCGTTGTTTGCCGCGGATCTTTTGCGCATGTACCACCGCTATGCTGAATCGCGCGGGTGGAAGCTGGATATCATCGAAGAGCAAACAACCGAATTGGGCGGCATCAAAGAAGTTGTGGCGCATATCACAGGCGAAAACGTTTTTGCGCGCTTGAAGTACGAGAGCGGCGTGCACCGTGTGCAACGTGTGCCCAGCACCGAAAGCGGCGGGCGCATTCACACCTCGGCGGCCACAGTAGCGGTTTTACCCGAAGCGGAAGATGTGGATATCCAGATCGCAACCACGGATATTCGCATTGATACATACCGTAGTTCGGGTGCTGGGGGGCAGCACGTCAATACCACCGATTCCGCCGTGCGGATCACCCATATTCCCACAGGGATTGTTGTCACCAGTTCCGAGAAATCCCAACACCGTAACCGCGAGATTGCGATGCAGGTGCTGAAAACGCGGCTTTATGATGCCGAACGCCAGCGGATTGATACAGAACGTTCGGACAGCCGCGCCAGCCAAGTCGGCAGCGGTGACCGCTCAGAGCGCATTCGCACCTATAACTTCCCCCAAGGGCGGATGACGGATCACCGGATAAACCTGACGCTCTATAAACTGGATGCTGTCATGCAAGGTGATCTGGATGAGATCGTGGATGCGCTTACGGCGGATGCGCAGGCGCAAATGCTGGCGGAGATGGGGCAATGAGTGGATAGAGCACAAAAAGAACAGTTGGTCGACGAACTCGGCCAAATCTTTGAAAGCTCTGGCGTAGTAGTGGTTAGCCACTACGTCGGTCTGACAGTTGCTGAAATGCAGGATCTTCGCGCGCGGGCAACCGCTGCGGGTGGGTCTGTGCGTGTTGCCAAAAACCGGCTCGCCAAGATCGCCCTTGAGGGCAAGCCATGCGCAAGCATTGCTGACCTTCTGACGGGTATGACCGTTCTGACCTATTCTGAGGATCCTGTAGCTGCGGCTCGGGTTGCTCAAGAATTCGCCAAAGAGAATGACAAACTTGTTATTCTCGGTGGTGCAATGGGTGAGAATGCGTTGGACGCCGCCGGTGTTGAAGCCGTGTCCAAGATGCCTTCGCGCGAGGAGCTTATCTCCACGATCGCTGGCATGCTGGGCGCACCTGCTTCCAACATCGCGGGGGCCATTGGCGCACCTGCAAGCAACATCGCATCCATCTTGTCTACGATCGAAGACAAGGCTGCG---TAAATGAAAACCTTTTCTGCAACACCGGCAGACATCGACAAAAAATGGATCATCATTGATGCTGAAGGCATCGTGCTGGGCCGTTTGGCATCGATCATCGCCATGCGCTTGCGCGGCAAGCACAAGCCTTCTTTCACACCTCACATGGATTGCGGCGATAACGTCATCGTAATCAACGCCGAGAAAATCCAAATGACCGGCAAGAAGCGTGAAGAAATGTTCTATTGGCACACCGGCCACCCAGGCGGGATCAAAGAACGCTCCAAGCAAGACATTCTGGATGGCAAGCACCCAGAGCGTATCGTGACTTTGGCCGTTAAGCGCATGCTGCCCGGCAACCGTCTGAGCCGCCAGATCATGACCAACCTGCGTGTATACGCCGGTGGTGAGCACCCCCATGAGGCACAAGAGCCAACCGTTCTGGATGTTGCATCCATGAACAAGAAAAACACGCGGAGCGCATGAATGCAGCAGGAAGATGCGCCGCTGCTGCACGCTGTCGATGTTGGTTTTGGTGCCGAACCTCCTAAACGTATCGGAATCGCTGTGTCGGGCGGGGGGGATAGTGTCGCGTTGCTGCATTTGTTTGCGCGCTGGTCTGCCCAATGTAATCACCCCATTGCTGCCGTTACTGTAGATCACGGCCTGCGCCCTGAGAGCCGCGCCGAGGCGGAAGGCGTAGCCGCGCTGTGCCAAAAACTGGGCGTTTCACACGATATTCTGACATGGGAGCGGCCTGAAGGGGCGGGTAATCTGCCTGCTGCTGCCCGTGACGGGCGCTATGCCCTGATGGCGGATTGGGCTAAGGCGCATGACATCGGCGGTATCGCGGTGGGCCACACGATTGATGACGGTGCCGAGAATTTTATAATGCGCTTGGGCCGTGCGGCAGGTATCGACGGTCTGGCCCAGATGGTGCCGCTGTTTGAGCGTTATGGTCTGTGTTGGGCAAGGCCGCTTTGGCAGTTTCATCGCGGGGCATTACGCGACTACTTGCTACGGCAGGATGTATCATGGGCAGAAGATCCTAGTAATGATGACCCGCGTTACCTGCGGACAAAGGCTCGGCGGCTCCTTCCTCAGTTAAAAGAGCTGGGGGTGGATGCACAGAGCATCCAACAATCAGCATCCGCGCTGCGAATGGCGCAATCGGCGCTGCAACATTATACTGTCAAAGAGGCCGAGACCCATGTGAAGGAAGTGGCAGGTGATATCCTGATCCCTCAGGTCATTGTGCCGCACATTCCCAGCGATATTGAGCGGCGGTTACTTGTTGCTGCTGTGCAATGGGTCGGATCAAATCCATACCCCCCGCGTAAGGAATTCGCGACCACGCTGGAGTTTACGCTGTCACAACAGCAGCGGTTGACGGTTGCAGGCTGCCTCGTCGCGCTGCGAAAAGGGTTTTTCCACATTACCCGCGAATATAACGCGGTGAAGGATTTAGCAGGCCCGACCGATGCCGTGTGGGATACACGTTGGCGGTTGCATGGGCCCCAT------GCGCCTGATCTAGAGGTGCGCGCCTTGGGAGAG---GCCGTGTCCGAGCTGCCTGATTGGCGGGCAACGGGCCTTCCAAGGCCGACATTAATGGCTTCGCCAGCGGTTTGGCGGGGTAAAACCCTCGTTGCGGCACCTTTGGCGAAATATAATGTAGATTGGACTGCGCAGATCGTCGCAGAT---------TTCACCTCATTCCTGCTTTCGCATTGAATGGCTGATCTGAAAAAACTGGCAGAAGACATCGTTGGTCTGACACTGCTTGAAGCACAAGAACTGAAAACAATCCTCAAAGACGAGTACGGCATCGAGCCTGCAGCCGGTGGCGCAGTCATGATGGCGGGCCCTGCAGACGGCGCAGCCGCTGCTGAGGAAAAAACTGAATTCGACGTCGTTCTGAAGAACGCCGGCGCATCCAAAATCAACGTGATCAAAGAAGTTCGCGGCATCACAGGCCTTGGCCTGAAAGAAGCCAAAGACTTGGTTGAAGCCGGTGGCAAAATCAAAGAAGGCGTTGATAAAGCCGAAGCAGACGAAATCAAAGCCAAGCTGGAAGCAGCTGGCGCAGAAGTCGAGCTGGCCTAAATGTCTCGTATTGGTAAAAAAGCGGTCAACCTGCCCAGCGGCGTTTCTGCCTCTGTCAGTGGCCAAACGATTGAAGTGAAAGGCCCTAAAGGGGTCCGTACCTTCAGCGCAACCGACGATGTCACGATGACCGTCGAGGAAAACGCCGTAAGCGTTACCCCTCGCGGTAAATCCAAGCGCGCGCGTCAGCAGTGGGGCATGTCCCGCACTATGATCGCTAACTTGGTGACTGGCGTTACCGACGGTTTCAAGAAAGAGCTTGAGATCCAAGGTGTTGGTTACCGTGCTGCCATGACTGGCAACACATTGAAACTGAACCTCGGCTTGTCCCATGACGTCGATTACATCCCGCCAGCAGGCGTGACTGTAACGGCGCCAAAGCAAACCGAAATCATTGTGGAAGGCATTGACGAACAACTTGTTGGTCAAGTCGCTGCGAACATTCGCGCATGGCGTAAGCCCGAGCCCTATAAGGGCAAAGGCATCCGCTATAAGGGTGAATTCGTCTTCCGCAAAGAAGGCAAGAAGAAGTAAATGAAAGTTATCCTTCTCCAACGTGTTGCGAAACTGGGCCAAATGGGTGACGTCGTTGACGTTAAGCCAGGCCATGCACGCAACTTCTTGCTGCCACAGCAAAAAGCTCTGACCGCCTCCAAAGCAAACATCGAAGCTTTTGAAGGTCAGAAAGCACAGCTCGAAGCACAGAACCTCGAAACCAAAGCAGAAGCCGAAGCAATGGCCGAACGTCTTGGTGGCCAGCAGTTCGTTGTGATTCGTTCCGCTTCCGACGCAGGCGCGCTTTACGGTTCCGTTACAACACGTGACGCATCCGATGCCGCAACTGCCGAAGGCTTCACCGTTGACCGCAAGCAGGTCGTTTTGATCGCGCCTATCAAATACTTGGGCATCCACGAAGTGAAGGTTATTCTTCACCCAGAGGTTGAAGCGACAATCGAGATGAACGTTGCACGTTCACCTGAAGAAGCTGAATTGCAAGCAGCCGGTAAATCCATTCAAGAACTCGCAGCGGAAGAAGAAGCCGCAGCAGAATTCGAGATTTCCGAACTGTTTGACGATCTCGGCTCCGCCGCGTCCGACGATGACGATGCAGCGCCTGCCGCA---------------------GCTGCAGACGAAGAAGCT------TAAATGAGCGATAGTGACGGCAGAAAAACATTGGGTCTG---GGCGGTTCGCGTCCCAGCAACGTAAAGCAGAGCTTTAGCCACGGACGTACAAAAAACGTCGTCGTGGAAACTAAGCGCAAACGCGTTGTGGTACCAAAGCCTGGTGGCCAAAAACCAACCGGTCCGGGTGCGGGCCCTGTAGGCGATCCTTCACGGCGCCCCGCAGGCATCACCGATTCAGAAATGGAACGCCGCCTGAAGGCTGTACAAGCCGCGAAAGCGCGTGAAGTAGAAGAAGCCGCAGCACGCGCTGCGGAAGAAAAAGCACGCGCAGAAGATCGCGAGCGCCGTCGCGCCGAGATGGAAGCGAAAGAGCAAGAAGATCGCGAGCGCGAAGAGAGCCTCAAGGCGAAAGCCGAGGAAGAAGAGCGCGCGAAACGTGCTGCGGAAGCCGCTGCACAGGCTGCACCTGCTCCTTCT---GAGCCAGCTCAAGCACGCGCAACGCCGAACAAAGCGCTTCCTGCTGCTACGCCGCGTAAAACCGAGCGTGACCGCGAAGAAACAAACAAAAAGAATCGCCAAGACGACGAT---CGTCGTTCAGGTAAACTGACAGTAAACCAAGCGCTGCGTGGCGGTGAAGGTGGCCGTCAGCGTTCCATGGCGCAGATGAAGCGTAAGCAAGACCGCGCACGTGCAAAAGCGATGGGCGGTAACGTCGAGCGCGAAAAGATCATTCGTGACGTTCAATTGCCACCAGCAATTGTTGTGTCCGAACTGGCCGCACGTATGGCTGAGAAAACTGGCGCTGTTGTGAAAGCGTTGATGCAATCCGGTCTGATGGTAACGCAAAACGAAACCATCGATGCGGATACAGCGGAACTGATCATCGAAGAATTCGGCCACAAAGTGGTTCGCGTTTCCGATGCTGACGTTGAAGATGTGATCAAAATCGAAGTCGATGACGAAGGCGATCTGCGGTCACGTCCACCTGTGATCACCATCATGGGTCACGTTGACCACGGCAAAACATCCCTTCTGGATGCGATCCGTAACGCCAAGGTTGTTTCCGGCGAAGCGGGTGGCATCACCCAGCATATCGGTGCCTATCAGGTGACTACCGATGGCGGCCAAGTGTTGTCCTTCCTTGATACTCCGGGCCACGCGGCCTTTACCTCCATGCGTTCGCGCGGTGCACAGGTAACGGATATTGTGGTTCTGGTGGTCGCGGCGGATGACGCTGTTATGCCTCAGACAATCGAGGCGATTGCCCACGCGAAAGCGGCTAAAGTGCCGATGATCGTGGCGATCAACAAGATCGACAAACCAGCGGCTGATGCCGACCGTGTCCGCGCTGCTTTGTTGCAGCACGAAGTTATCGTTGAGAAGATGTCTGGCGATGTGCAGGACGTTGAAGTTTCAGCGGTCACTGGCCAAGGTCTGGATGAACTGCTGGAAGCGATTGCATTGCAGTCCGAGATCCTTGAACTGAAAGCAAACCCGAACCGCGCTGCCGTTGGTGCCGTGATCGAGGCACAGCTTGATGTGGGTCGTGGTCCAGTGGCGACGGTTCTGGTGCAAAACGGTACATTGCGTCAGGGTGACATCTTTGTTGTAGGTGAGCAGTACGGTAAGGTTCGTGCGTTGATTGACGACCAAGGCAACCGCGTAAAAGAAGCTGGCCCTTCTGTTCCTGTAGAGGTTCTGGGCTTGAATGGTACGCCAGAAGCGGGTGACGTTCTGAACGTGACATCCACCGAAGCGCAGGCGCGTGAGATTGCGGAATACCGTGCAAACGCCGCTAAAGACAAACGCGCTGCTGCGGGTGCCGCAACGACGTTGGAACAGTTGATGGCAAATGCCAAAGCTGATGAAAACGTTTCTGAACTGCCTATTCTGGTCAAAGCGGATGTGCAGGGTTCTGCCGAAGCGATTGTTCAGGCAATGGAGAAAATCGGTAACGATGAAGTCCGTGTACGGGTTCTTCACTCTGGTGTTGGTGCGATCACTGAAACAGATGTGGGCCTTGCCGAAGCTTCGGGCGCGCCGATCATTGGCTTTAACGTCCGTGCCAACGCCTCTGCGCGTAACACGGCAAACCAGAAGGGTGTCGAGATCCGCTACTACTCGATTATCTACGATCTGGTGGACAACGTAAAAGCGGCGGCTTCCGGCTTGCTCAGTAACGAGATCAAAGAAACCTTCATTGGCTACGCAAGCATCAAAGAAGTGTTCAAGGTCACAGGCATCGGCAAAGTGGCTGGCTGTCTGGTTACCGAAGGGGTGGCGCGCCGCTCTGCTGGTGTACGTCTGCTGCGGGATAACGTTGTTATTCACGAAGGTACGCTTAAGACGCTCAAGCGCTTTAAGGATGAAGTACCAGAAGTACAGTCCGGTCAGGAATGTGGTATGGCCTTCGAAAACTACGAAGACATCCGCCCTGACGATGTGATCGAAATCTTCACCCGTGAAGAAGTGACACGCACGCTGAGCTAAATGGCCAAGCCTAAAAAGACCCCGCGCCCAAAGGCGGAAACGCCCCGTGGTTTTCGCGATTATTTCGGCACCGAGGTGACCCAGCGCGCCGAGATGCTGGCAAAGATTGCAGGGGTTTATCATCGTTATGGTTTTGACGCGCTCGAAAGCGCTGGCGTTGAGAAGGTTGAAGCGCTGGGTAAGTTCCTGCCTGACGTGGACCGCCCCAATGAAGGCGTGTTTGCATGGCAAGAGGACGCTGAGGCAGAGAAGCCCGGTGACTGGCTGGCACTGCGCTATGATCTGACGGCGCCGTTGGCCCGTGTGTATGCGCAGCACCGCAATGAATTGCCGACGCCGTACCGCCGCTATGCGATGGGGCCTGTTTGGCGCAATGAAAAGCCCGGCCCTGGTCGCTTTCGCCAGTTTTATCAATGTGATGCTGATACGGTTGGTGCGGGTTCCGTTGTTGCCGACGCCGAGATTTGTGCGATGTTGTCGGATTGCCTTGAGGAAGTCGGGATCGAGCGCGGTGATTACATCGTGCGCGTGAACAACCGCAAAGTGTTGAACGGTGTGCTGGAGGTTGCTGGCCTGTCGGGAGATGACAAAGAGAACGCGCGCGGGATCGTGCTGCGTGCTATTGATAAACTGGACCGCCTTGGCGTGGACGGTGTGCGGGCGCTGCTAGGGGCAGGGCGCAAAGACGATAGCGGCGATTTCACCGACGGTGCGGGGCTGGATGATGCGGCGGCTGATGTTGTCATGGGCTTTATGGATGCCAAGCGCGACGATGGCGCGGCGACCTGTGCGCGGTTGCGTGAATTGGTCGGCGATAGTGTTGTTGGCCTTGAAGGTGTGGCCGAGTTGGAAACCATTGCCGAATTGTTAAGCGCTGGCGGCTACGGACCGGATCGGATCGAAATTGATCCCTCCGTTGTGCGTGGCCTTGGCTACTATACTGGCCCTGTCTACGAAGCCGAACTCACTTTTGAAATCAAAGACGAAAAAGGCCGCCCACGCAACTTTGGTTCGGTTGCGGGCGGTGGGCGATATGACGATCTGGTCAAACGCTTTACCGGACAAGAAGTCCCTGCAACGGGCGTTTCAATCGGTGTCGACCGTCTGCTGGCTGCCCTACACGCCAAGGGGCGGATGGACACGCAAGCCGAAGGCCCTGTTGTTGTCACAGTGATGGATAAGGCGCGCATGGCCGATTATCAGGCGATGGTGGCTGAACTGCGTCAGGCGGGTATTCGTGCGGAAGTATACTTGGGAAATCCCAAGAACTTTGGCAACCAATTGAAATACGCCGATAAACGCGGCAGCCCTGTGGCCATCATTGAAGGCGATGAAGAGCATCAAAATGGCATGATACAAATCAAGGATTTGGTGCTGGGTGCGAAAATCGCAGAAAATGCGACACTGGAAGAATGGCGCGACAGGCCCAGCCAATACGAAGTGCCGCGTGCCGATCTGGTGACACGTGTCCGCGAAATACTGGAGCTGTACCGCTGAATGGACGATCTCAAGCAAAAATACCTTTCCCAGATTGCAAATGCGTCTGATGAATCCGGATTGGAAGACATCCGGTTGGCCGCTGTCGGCAAAAAGGGCGAAGTTGCTCTGAAAATGCGTGAACTGGGCAAGATGACACCAGAAGAACGCCAGACTGCAGGCCCTGCGCTGAATGCGCTTAAAGATGAAATTAACTCGGCTTTGGCGGCGAAGAAGGCGGCACTGGGCGATGCGGCATTGGATGAGCGCCTGCGCAGTGAGTGGCTGGACGTGACGCTGCCGTCACGCGGGCGGCCCATGGGCACGATTCACCCTGTGTCTCAGGTTACCGAAGAACTGACAGCGATCTTTGCTGAAATGGGCTTTTCCGTTGCCGAAGGTCCGCGCATTGATACCGATTGGTATAACTTTGACGCGCTGAACATTCCGGGCCACCACCCTGCACGCGCCGAGATGGACACGTTTTACATGGCCCGCGCTGAAGGTGACGACCGCCCGCCGCATGTGCTGCGCACCCATACTTCGCCTGTGCAAATCCGCACGATGGAGGCCGAAGGCGCGCCGCTGCGCATCATCTGTCCCGGTGGTGTGTACCGCGCTGACTATGACCAGACCCACACGCCGATGTTCCATCAGGTAGAAGGTTTGGCGATTGATAAAGACATCTCCATGGCGAACCTGAAATGGACGCTGGAGGAATTCTTCTCGGCTTTCTTCGAGATCGACGGCATCAAAACCCGCTTCCGCGCCTCGCATTTCCCCTTCACCGAACCATCGGCGGAAGTGGATATTCAATGTTCATGGGTCGATGGCCAATTGCGCATTGGCGAAGGCGACGGATGGATGGAAGTGCTGGGTTCTGGCATGGTCCACCCCAAAGTGCTGGCCGCTGGCGGGATCGATCCCGAGGTTTATCAAGGTTTTGCCTTTGGCATGGGGATCGACCGTATTGCGATGCTGAAATACGGCATCCCCGATTTGCGGGCGTTCTTTGATTCAGATTTGCGTTGGCTGCGGCACTATGGCTTCGCGAGCTTGGATCAGCCGAATTTGCATGGTGGGTTGAGTAGGTGAATGGTCATGGCGGAAGAGGCAGGACTGGATCTGGTAGAGATTTCTCCCAATGCCAACCCGCCCGTGTGTAAAATCATGGATTTCGGCAAGTTCAAATACGAGACGCAAAAACGCGAAGCCGAAGCGCGTAAAAAGCAAAAGATCATTGAGATCAAAGAAATCAAGTTCCGCCCGAATACCGACAGCGGCGACTATGAAATCAAAATGCGTAACGTTTTCAAATTTCTGGCAAACGGCGACAAGGTGAAAATCACCCTGCGTTTCCGTGGCCGCGAAATGGCACACCAGAACCTCGGCCGCGAGCTGCTGGAACGTGTTGCAGCCGACACCAAAGACTTTGGCAAAGTTGAAAACTTTCCGAAAATGGAAGGTCGCCAAATGGTCATGCTGATCGGCCCGCTGCCCGCCTAAATGAAATTCACACTCTCTTGGCTCAAAGAGCACCTCGACACCACGGCTTCCGTGGATGAGATCACCTATGCCCTGACCGATCTGGGTCTGGAAGTGGAGGGGGTCGAAGACCGTGGCGCGAAGCTGCGCGATTTTACCCTTGGTTTTGTGAAATCCGCTGAAAAGCATCCCGATGCGGACCGCCTGCGCGTGTGTCAGGTGGAAACCGACGAAGGCCTGCAACAGATCATCTGTGGCGCACCCAATGCGCGCGAAGGCATCACCGTTGTGATCGCCAAGCCCGGCGTGTACGTGCCCGGCATTGATACCACCATTGGCGTGGGCAAAATCCGCGGGATTGAAAGCTTTGGCATGATGGCCTCCGAGCGCGAGCTGGAGCTGTCCGAAGAGCATGACGGGATCATTGAACTTCCGTCTGGTAATGTCGGCGACAGCTTTACCGATTGGCTGGCCGAAAATGACCCTGCCAAAGTTGACCCTGTGATCGAAATCGCCATCACGCCGAACCGTCCAGACGCCCTTGGCGTGCGCGGTATTGCGCGTGATCTGGCGGCGCGTGGTTTGGGCAAGCTCAAGCAGCGTGACGTAGATGTGGTGGCGGGGGCGTTCCCTTGTCCTGTCAGCGTCAGCATTGACGACGACACGCTGGAGCACTGTCCTGTGTTCTTTGGCCGTGTCATTCGCGGCGTGAAAAACGGGCCAAGCCCTGTTTGGTTGCAAGACCGTCTGCGTGCCATTGGCCTGCGGCCGATCAGCTTTTTGGTGGATGTGACCAACTTTTTCACCTTTGACCGCAACCGCCCGCTGCATGTGTTTGACGCGGATAAGATTGCAGGCGACTTGCGCGTTCACCGCGCCAAAGGTGGTGAGACCTTGGTCGCGTTGGACGACAAAGAATACACCCTGCAAGACAGCATGATCGTGATCTCGGACGCGAATGGGGTTGAGAGCATTGGCGGCGTGATGGGCGGTTTGGCCACTGGCGTGACCGAAGACACCGTGAATGTCTTCCTTGAAGCAGCGTATTTTGATCCGGTGCGTACAGCCTACACGGGCCGCGCGCTCAAGATCAATTCGGATGCGCGATATCGCTTTGAACGCGGGATTGACCCCGAGTGGACGCCATACGGGATTGAGCACGCAACCCAGATGATCCTTGATCATGCGGGCGGTGAAGCCTCGGACGTGGTGAGTGCGGGTAAAGTGCCTGACACCAGCCGCGCCTACCGTCTGAATGCGAAACGTGTGGTTTCCTTGGTTGGTATGGAAATCCCTGAATCGACCCAGCGTCAGACGCTGACGTCTTTGGGTTTCCGTTTGGAAGGCGACATGGCGCATGTGCCCAGCTGGCGCCCCGATGTTCAGGGCGAGGCCGATCTGGTGGAAGAAGTGGCGCGGATTGCGTCCCTGACCAAGTTGGAAGGCGTGCCTTTGCCGCGTCTGACAACAGGCGTGCCGCGCCCCGTGATGTCACCTGTGCAGCGCCGCGAAGTTGCGGCTCGGCGTACGGCGGCGGCATTGGGCTATCATGAATGTGTGACCTATAGCTTCATTGACCAGCCCTCGGCGGCTCTGTTTGGTGGGGGCACTGATGCAACCCGTCTGGAAAACCCGATCAGCAATGACATGAGCCACATGCGCCCGGCCTTGCTGCCCGGTCTGCTGGCTGCCGCAGCACGCAATCAGGCACGTGGTTTTGCAGATATGGCGCTGTTTGAAGTTGGCCCTGCCTTTAGTGGCGGTGAACCGGGCGAACAGCACATGCTTGTTTCTGGCCTGTTGGTGGGGCGTACTGGTCCCAAAGACGTGTTGGGTGCTGCGCGTGACGTAGACGTGTTTGACGTCAAAGCGGACGCCGAGGCCATTCTGGCGGCAATCGGCGCACCGGCCAAAGTGCAAATTCTGCGCGGCGCTGATGATTGTTGGCATCCCGGCCGTCACGGCAAGGTTTGTCTTGGTCCCAAGAAAGTGCTGGCCATCTTTGGCGAAGTACATCCACGTATCTTGGCCGCGATGGATGTGAAGGGGCCTGCAATGGCCTTTACGATCTGGCCTGCCGAAGTGCCGCTGCCGCGCAAAGCAGGGGCCACACGCCCCGCGCTGAAGGTCAGTGATTTGCAAGCGGTCGAGCGTGACTTTGCCTTTGTTGTAGATGCGGATGTAGAAGCCCTGACGTTGGTTAACGCGGCGATGGGTGCGGATAAGGTATTGATCGAAGACGTGCGTGTGTTTGACGAATTCATCGGTGGCAGTCTGGGTGAGGGGAAAAAATCCCTTGCGTTGACCGTACGCATGCAACCGCATGAACAAACGTTGAAAGATGCGGATATCGAAGCTGTGGGGGCCAAGGTTGTTGAAAAAGTGACCAAGGCCACAGGCGGGGTCTTGCGCGGGTGAATGCACGCCTATCGTAACCAGACTTGCGCCGACCTAAACACAACAAACGTGGGGGACAAAGTCCGCCTTTCCGGTTGGGTTCACCGTGTCCGCGATCACGGGGGCGTTTTATTCATCGACCTACGTGACCATTATGGGATCACCCAATTGATTTGCGATGGCGATTCACCTGCATTCGCTGAGCTGGAAAAAGTTCGTGCTGAATGGTGCATCCGTGTGGACGGCGACGTAAAAGCGCGTGCAGCTGAACTGGTGAACGAAAAAATTCCGACGGGCGGTATTGAGATCTATGTCCGTGACGTTGAAGTCCTTGGGCAAGTAAACGATTTGCCATTGCAGGTTTTCGGTGAGCAAGAATACCCAGAGGAAACCCGTCTGCGCTATCGTTACCTTGATCTGCGTCGCGAGAAGATGCAGCGCAACATGATCTTGCGCTCAGATGTGGTTAGCTCGATCCGTCAGCGCATGTGGGATCGGAAGTTTAAAGAATTCCAAACGCCAATCATTACCGCCTCCTCTCCTGAAGGGGCGCGCGACTTTTTGGTGCCGTCACGTTTGCACCCTGGTAAGTTTTACGCACTACCTCAAGCACCACAGCAGTTTAAGCAATTGTTGATGGTATCGGGTTTCGATAAATACTTCCAAATTGCGCCGTGCTTCCGTGATGAAGATCCGCGCGCAGATCGGTCGCCAACAGATTTCTACCAACTCGACCTTGAGATGTCGTTTGTTGAGCAGCAAGATGTGTTCGACACGATCCAGCCTGTGCTGACAGGCATCTTTGAAGAATTCGGCGGTGGCAAAGCTGTGGATCAGGAATGGCCGCAAATTTCTTATAAGGATGCCGCGCTCTGGTATGGTTCAGACAAACCTGATTTACGCAACCCGATCAAGATGCAGGTTGTTTCCGAACACTTCGCAGGTTCCGGCTTTGCAATTTTTGCAAAACTGCTGGAGCAGGAAGGCACCCAAATCCGTGCGATCCCAGCACCTACTGGTGGTAGCCGCAAGTTCTGTGACCGGATGAATGCCTTTGCACAAAAAGAAGGCCTGCCCGGCATGGGGTATATCTTCTGGCGTGATCAA---GGTGAG------GGCATGGAAGCGGCTGGCCCACTGGCCAAGAACATTGGGCCCGAGCGCACCGAAGCCATTCGCCAGCAACTCGGGCTGGGCGTCGGTGACGCGGCCTTCTTCCTTGGTGGTAAGCCGAAAGCATTTGAATCTGTTGCTGGCCGCGCCCGGAATGTGATCGGAGAAGAACTGGGCCTGACAGATCAGAACCGCTTTGCCTTCGCTTGGATCGTGGATTTCCCGATCTACGAAAAAGATGAGACAACGGGCAAGATTGACTTTGAACACAACCCTTTCTCCATGCCCCAGGGCGGGATGGAGGCACTCAACGGTGATCCGTTGGAAGTGTTGGGCTATCAATATGATCTGGCATGTAACGGGTATGAATTGGTTTCGGGCGCGATCAGGAACCACCGTCCCGAGATTATGTTCAAAGCCTTCGAAATTGCCGGTTATGGCCCCGAAGAAGTGCGCAAGCGTTTTGGTGGCATGGTCAACGCATTCCAATACGGTGCTCCACCCCACGGCGGTTGTGCGGCAGGGATTGACCGTATCGTGATGTTGCTCGCCGAAGAGAGCAACATCCGTGAAGTGATCTTGTTCCCGATGAACCAGCGTGCCGAAGATCTGATGATGAATGCACCAAACGATCCAATGCCGGATCAACTGATGGAATTGGGTCTGCGGGTTATCCCACAAGACTAAATGACACCACTTTCACACATCCGCAATTTCTCCATCGTCGCTCACATTGACCATGGCAAATCCACCCTCGCTGATCGTCTCATCCAAGAGACGAAGACGGTCGCTGTGCGCGATATGAAAGCGCAGATGCTGGACAGCATGGACATCGAGCGCGAGCGCGGAATTACGATCAAAGCACAGACAGTGCGGATCAACTATCAGGCGCTCAATGGTGAGGAATATGTCCTCAACTTGATCGACACCCCCGGTCACGTCGATTTTGCCTATGAAGTCTCCCGCTCGATGCGTGCGGTTGAGGGGTCATTGTTGGTGGTGGACAGCACGCAGGGCGTTGAGGCGCAGACGCTTGCCAACGTTTACCATGCTCTGGATGCAGATCATGAGATCGTGCCCGTCCTGAACAAGATTGACCTGCCTGCGACAGACTGTGACCGTGTTGCGGAACAGATCGAAGACGTGATCGGCATTGACGCCTCTCAGGCGATCCGCGTGTCTGCCAAGACGGGCGAAGGCATTGTTGAGACACTGGAAGCCGTGGTGCATAGGCTACCAGCGCCGACAGGCACATTGGATGCGCCGCTAAAAGCAATGCTGGTTGATTCATGGTACGACAGCTATCTGGGCGTGATCGTTCTGGTGCGGATCATGGATGGCCAGTTGAAAAAGGGCGACCGCATCACGATGATGCAAAACGGCTCTATTCACCACGTAGACCGCATCGGTGTTTTCCGCCCCGCGATGACAGAGATAGACGTGCTGGGACCCGGCGAAATCGGGTTCCTCACCGCGTCGATCAAGCAGGTGCGTGATACCCGTGTGGGGGATACGATCACCCATGATAAAAAAGGCTGTGAAACTGCGCTGCCGGGCTTTAAGCCATCGCAGCCTGTGGTTTTCTGTGGCCTGTTCCCCGTTGATGCAGCGCTGTTCGAAGACCTGCGCGATAGCATCGAAAAACTGGCGTTGAACGACGCGAGCTTTAGCTATGAGATGGAAACATCCGCTGCACTTGGCTTTGGTTTCCGCTGTGGCTTTCTTGGTCTGCTGCACCTCGAAGTGATCCGTGACCGGATCGAGCGTGAATACGATATTGATCTGATCACTACAGCGCCGAGCGTTATCTATAATATCCACATGAAAGACGGGACGATGCAGCAATTGCACAACCCCGCTGACATGCCTGATCTGACCTTTGTCGAGCATCTTGAGGAGCCACGGATCAAGGCGACAATCCTTGTGCCTGATGAATACCTCGGTGACGTACTTAAGCTTTGCCAAGACCGTCGCGGTATCCAGATGGATCTGACCTATGCCGGTTCGCGTGCGATGGTTGTCTATGACCTGCCGTTGAACGAGGTTGTGTTTGATTTTTACGACCGTCTGAAATCAGTGACCAAAGGCTATGCATCGTTTGACTATCAGATGATCGGCTACCGAGAAGACGCGCTGGTCAAGATGTCCATCCTTGTCAACGATGAACCCGTCGATGCCTTGTCCACCATGGTTCACCGCGACCGCGCCGAAATGCGGGGCCGCGCCATGGTTGAAAAGCTCAAAGACCTGATCCCGCGCCACATGTTCAAAATCCCGATCCAAGCGGCAATTGGCGGCAAGGTAATTGCACGCGAAACTTTGAGCGCCATGCGCAAAGACGTGACGGCCAAATGTTATGGTGGTGACGCCTCGCGTAAGCGCAAGCTGCTGGACAAGCAGAAAGCGGGTAAGAAGAAGATGCGCCAATTCGGGTCAGTGAACATCCCGCAAGAAGCGTTTATCTCTGCGCTGAAGATGGACGGGTGAATGAGTCTTCCACCCGGTTTCCTTGATGAATTGCGCACACGCTCCAGCCTGTCTCAGGTTGTGGGGCGTAAAGTCATTTGGGATGCGCGTAAGTCCAATCAGGGCAAGGGTGATATGTGGGCACCATGCCCCTTTCACCACGAAAAATCCGCCAGTTTTCATGTAGATGACCGCAAGGGTTTTTATTATTGCTTTGGCTGTCACGCCAAAGGCGATGCCATTTCTTTTGTGCGCGAGACAGAAAATGTCAGCTTTATGGAAGCGGTAGAGATACTTGCGCGTGAAGCCGGGATGCCGATCCCGCAACGTGACCCGCAAGCGCAAGAAAAAGCCGAGAAACGTACAGAACTCGTGGATGTGATGGAATTGGCAGTCCGCTGGTTCCGGTTGCAGTTGCGCACAGGGGCTGCCTCGGCGGCGCGGGAGTATCTGGAGAAGCGCGGGTTGAACCAAGAGGTCTGCGACCGTTGGGAGATCGGCTTTGCGCCTGATAGCTGGCAGGGCTTGTGGGACGCGCTGAAAGGCAAGGACATCGCAGATGAGTTGATCATCGGTGCGGGTCTGGCCAAACCTTCCAGTAAAGGCGGCAAGCCCTATGACACGTTTCGCGGGCGGATCATGTACCCGATCCGTGATGCGCGGGGCCGTGCGATTGCCTTTGGCGGGCGTGCGATGGACCCTGAGGATAAGGCGAAATACCTCAATTCTCCCGAGACCGAATTGTTTGATAAGGGCCGCAGCCTTTACAACGTGAAGGAGGCGCGGGTGGCAGCGGGCAAGGGACAACCCTTGCTGGTGGCCGAAGGATACATGGATGTGATCGCGCTGTCCGAAGCGGGGTTTGGCGCGTCCGTTGCGCCGCTTGGCACGGCGATTACTGAAAACCAGTTGGCGATGCTGTGGCGGATCAGCGACGAGCCGATCATCACATTGGATGGCGATACGGCTGGCCAGCGCGCTGCGCTGCGCTTGATCGATCTGGCGCTGCCTTTGCTAGAGGCGGGGCGCTCTTTGCGCTTTGCCATGATGCCAGAGGGCAAAGACCCCGATGATTTGCTGAAATCTTCTGGTGCGGGGGCGGTACAGGCGTTGCTGGACGCGGCTGTGCCTATGGTGCAACTGCTGTGGCAACGCGAGGTTGAGGGCAAAGTCTTTGACAGCCCCGAACGCAAAGCGGCGCTGGATAAGAACCTGCGTGAAAAAATCAAACTGATCAAAGACCCCAGCATCCGCAGCCACTATGGCCAAGCGATCAAGGACATGCGGTGGGACCTGTTTCGCGCCAAA------------GGCAGTTTT---------GCGCCACGTCCATGGAAGTCGCAGTGGGGCAAAGCGCCGCAAGGGCCATCAGCGGGGGCCAAATCCTCTATCTTGGCGACTGCGGCAGATGCCCGCGCGACGGATCATTTGCGCGAAGCCGTTATTTTGGCGGCCTGCATCAGTTGTCCAGAAGCAGTCGAGCCGTTTGAAAGCGGGCTGGAGACGATGGCCTGTCTTGATGCCGAGCACGCCCGGATGCGGGACATGGTTTTACGGTATGCGCCTGCTGGCGCTGCGGTGTTGCGGGGGCAAATTTCGGATGTTTTAGGGCCTGATGCACTTGAAAACCTGCTAACGCAGCGCCATGTCGCTATTACGCCGTGCATTCGCAGGCCCGGTGATGTGGAGCTCACGGGCATGACAATCGCTGAAGAACTGGCCAAGCTCAAAGCGGTGCGGGGGCTTCAGGAAGAAGTGAATGATGCGGCCGATGACCTAAGCGGTGAGGCGGATGAGGGGCTGACGTGGCGATTGGCCGAAGCTGCAAAAGCAGCGGATCGTGCGCATCGCGCTGGTCAGGAAGACAAAGCGGAATATGTTATCGCGGATAATGGTGCACGGCTGGATCGTGATCAGGTGACCCTGAGCCGTAACATGTTTGACCAAATCGACTTTTCCAAAGGTGGGAAAAAAGACTAAATGTGCGCCGAGACCCCAGACTATAAATCTACTTTGAACCTGCCAAAAACCGATTTCCCAATGCGTGCGGGATTGCCCAAACGTGAGCCAATGTGGCTGGAACAGTGGAATGAAATCGGCATCTATGACCGCCTGCGCGAAAAGGAGACACGCGAACCGTTCACCCTGCATGATGGCCCCCCCTATGCCAACGGGCATTTGCACATCGGTCACGCGCTGAACAAAACCATCAAAGACATGATCGTGCGCAGCCACCAGATGATGGGCCGCGATGCGCGTTACATCCCGGGCTGGGATTGCCATGGTCTGCCGATCGAATGGAAGATCGAAGAACAGTACCGTAAAAAAGGCCGCGACAAGGACCAAGTGCCGATCAATGAATTCCGTGGTGAATGCCGCGAGTTTGCCAAAGGCTGGGTGGACATCCAGCGTGAGGAATTCAAACGCCTTGGGGTTCAGGGAAACTGGGATAAGCCATACCTGACAATGGATTTCCACGCCGAGCGCGTGATCGCCGAAGAATTCATGAAATTCTTGATGAACGGCACGCTGTACCAAGGCTCTAAACCTGTGATGTGGTCGCCTGTTGAGCAGACCGCATTGGCCGAGGCCGAGGTTGAGTATCACGACAAAGACAGTTTCACGATCTGGGTGAAGTTTAAAGTTGTTGGTGGT---------------GATCTGGAAGATGCGCAGGTTGTCATCTGGACAACGACGCCATGGACAATGCCATCGAACAAAGCAGTCGTTTACGGCGCGGGTATTTCTTATGGCCTGTATGAAATCACTGGCACGCCTGACGAATGCTGGGTGAATGTGGGCGATAAATATCTGCTGGCCGATAATATGGCGGCGGATGTTCTGGGGCGTGCACGTCTGGAGGAAGGGCAGTGGGCCCGTGTCCGTGATGTGACCACTGCTGAACTTGAAACCATCAGCCTCAAGCACCCGCTGCATGGTGTCGACGGCGGCAACGGCGAATGGGATGATCTGCGCGATTTCCGCGCCGCTGATTTTGTGACCGACACTGATGGTACGGGCTTTGTGCATTGCGCGCCAAGCCATGGTTTGGATGAATATGATCTCTACCGTGATCTGGGCATGCTGGACCAAGTTATCACGTATAATGTGATGCCCGATGGTCGTTACCGCGATGATTTGCCGCTGTTTGGCGGCAAGGCGATCCTCAAGCCGAACGGCAAAGAGGGCAACGCCAACGCTGCTGTGATCGACAAGCTGGTCGAAGTCGGTGGCCTGCTGGCGCGTGGCAAGATCAAACACAGCTACCCACACAGCTGGCGCTCTAAAGCGCCCGTGATCTATCGCAACACTCCGCAATGGTTTGCCGCGATTGATAAAACTGTGGGTGATGGTCAGGACCAATTCGGCACCACCATCCGTGAACGTGCGCTGACCGAGATCGACAACGTCAACTGGACGCCAAAATCAGGCCGCAACCGCCTGCACGCGATGATGGAGGCACGCCCAGATTGGGTGCTGTCGCGCCAACGTGCATGGGGTGTGCCGCTCACCTGCTTTACGCGCAAAGGTGTGCTGCCGACGGATGCGGATTTCCTGCTGCGCAATGCAGAGGTGAACCAGCGTATTGTTGAGGCGTTTGAAGTTGAAGGTGCGGATGCGTGGTATGAGGACGGCGCAAAGGCGCGTTTCCTTGACGGTATCGTGAACCCTGATGATTACGATCAAGTCACCGATATTCTGGACGTGTGGTTCGACTCTGGTTCGACTCATGCCTTCACTCTGCGTGATCGCGAAGATGGCTCAGAGGATGGCATCGCAGATGTCTATATGGAAGGCACCGACCAGCACCGCGGCTGGTTCCACTCGTCCTTGCTGCAATCGGTTGGCACCACGGGCCGCGCGCCGTACCGCAATGTTGTGACGCATGGCTTTACGTTGGATTCAAAGGGCATGAAGATGTCCAAATCCATCGGCAATACCATCGTACCCGAGAAAATCATCCAGCAATACGGGGCGGACATCCTGCGCCTTTGGGTGGCCCAGACGGATTACACCAACGATCAGCGCATCGGTGATGAAATCCTCAAAGGCACCTCGGACAGTTATCGCCGCTTGCGCAACACCATGCGCTATATGCTGGGTGCCTTGTCTGATTTCACCGAGGCAGACCGCGTAGACGCCGCTGATATGCCCGAGTTGGAGCGTTGGGTGCTGCACCGTCTGGCCGAGTTGGATACAGTGGTACGTGACGGCTATTCGCGCTTTGATTTCCAAGGCGTTTTCCGCGCGATCTTTGAATTCGCCACGCTGGATTTGTCGGCCTTCTACTTTGATATCCGCAAAGACGCGCTGTATTGTGATGGCGATACGCTGCGCCGCCGTGCGGCACGCACGGTGTTGGATCTGTTGTTCCACCGTCTGACAACTTGGTTGGCACCCGTTCTGGTGTTCACCATGGAAGAAGTCTGGCTGGAGCGTATGGGTGGCGAAGGCAGCTCGGTTCACCTGCAAGATATTCCTGCCACGCCTGAGGGTTGGCTGGATACGGAATTGGCCGCAAAATGGGCCAAGGTCCGTGCGGCGCGCCGTGTGGTGACTGCCGCTTTGGAAGTACAGCGTACAGCCAAGGTAATTGGTGCCTCGCTTGAGGCGGCTCCGGTTGTCTATGTTGAAGACGCAGCGCAGCGTGCGGCGTTGGAGAGTGTGTCGTTTGATGATGTGACCATCACCAGCCAGATCACTGTCACAGGCGATGCAGCACCTGAGGATGCGTTCCGCATGCCCGAAACCGACGGTGTGGCCGTGTCCTTTGTTCTGGCCCAAGGCGAGAAATGTGCGCGGTGCTGGAAAGTTCTGCCAGATGTTGGAACCCACAAGCATGCAGGCGTTTGTGCCCGCTGTGACGATGCGGTT---TAAATGGCTGCTAGCCAAAACATCCGCATCCGTCTTAAGGCGTTTGATTATCGGGTGCTTGATGCTTCCACACAGGAAATCGTCAACACCGCCAAGCGCACAGGCGCTTCTGTTCGCGGCCCCATTCCGCTGCCGAACAAAATCGAAAAATTCACCGTTTTGCGTGGTCCCCACGTAGACAAAAAATCCCGTGACCAGTTCGAGATCCGTACGCACAAGCGTATGCTGGATATCGTTGATCCGACTCCCCAGACCGTTGACGCGCTGATGAAGCTCGACCTCGCCGCTGGTGTGGACGTCGAGATCAAGCTGCAGTCATAAATGTCTGATCAAATCAACACACTCGAAGATCTC---------------GCATCTGTAGCCGGCGTTGCCGCAACTCCAGAAGTTGAACTGACACCACGTGAGCCCGTTCGTGACGAATTCGGCCGCGCTTATGCAACCGGCAAACGTAAAGACGCTGTTGCACGCGTCTGGATCAAGCCAGGTTCCGGTAAGGTTATCGTAAACGGCAAGCCGCAAAACGAATATTTTGCGCGCCCTGTTTTGCAGATGATCCTGCAGCAGCCTTTCGGCATCACCGGTACGGATGGCCAATTTGACGTCTACGCGACAGTCAAAGGCGGCGGTCTTTCTGGTCAAGCGGGCGCGGTTAAGCACGGTGTTTCCAAAGCACTGCAGCTGTATGATCCCTCCTTGCGTGGCGCATTGAAAGCCGCTGGCTTCCTGACACGCGACAGCCGCGTTGTTGAGCGTAAGAAATACGGTAAAGCAAAAGCGCGTAAGAGCTTCCAGTTCTCCAAGCGTTAAATGCCAACAATTCAGCAGCTGATCCGCAAACCGCGCCAGCCGAAACGCAAAACATCGAAATCCATGCACCTGCAGGAATGCCCGCAGAAGCGTGGCGTATGTACGCGCGTTTATACAACCACACCTAAAAAGCCTAACTCCGCGATGCGGAAAGTGGCCAAGGTTCGCTTGACCAACGGTTTTGAGGTCATCTCCTACATCGGCGGCGAAAGCCACAACCTTCAGGAACACTCTGTGGTTCTGATCCGTGGCGGCCGTGTAAAAGACCTTCCAGGTGTACGTTACCACGTCGTTCGCGGTGTTCTCGATACGCAAGGCGTTAAAGATCGTAAGCAGCGTCGCTCCAAGTACGGCGCCAAGCGTCCGAAGTAAATGGCACGCGAAGCAAAACGCACCAAAAAGAAGGTCTCCAAGAACATCGCCGCAGGTGTGGCGCATGTGAACTCTTCTTTCAACAACACAAAAATCCTGATCTCCGACGTACAGGGCAACGCCATTGCATGGTCGTCCGCTGGTACAATGGGCTTCAAAGGGTCGCGTAAATCTACTCCTTACGCAGCTCAGATGGCCGCCGAAGATGCAGGCAAAAAAGCACAAGACCACGGCGTGAAAACGCTGGAAGTCGAAGTGCAAGGTCCAGGTTCGGGCCGTGAAAGCGCATTGCGCGCTCTGGCAGCTGCCGGTTTCAACATCACCTCTATCCGTGATGTAACACCAATGGCACACAACGGTTGCCGTCCGCCAAAACGCCGCCGCGTTTAAATGAAC---GACCGACGCGGCCTTTTGATCATCCTTTCCTCACCTTCCGGTGCAGGTAAATCCACACTCGCCAAGCGGCTGATGGTTTGGGATGAAACCCTGAGTTTTTCCGTGTCCGCCACCACCCGCGCGCCCCGCGAAGGTGAGGTGGACGGCAAGGACTATCGCTTTGTGCAAGAAGATACTTTCCGCCAATGGGTGGGAGAGGGCGAAATGTTGGAGCACGCACATGTCTTTGGTAATTTCTACGGCTCACCAAAGGCGCCTGTCGAAGAAGCCATTAATGCTGGTAATGACGTGCTGTTCGACATCGATTGGCAGGGTGCACAGCAAATCCGCAACTCTCCTCTGGGGCTTTATACGCTGTCGATTTTCCTGCTTCCACCCTCTATCGCCGAGCTACACCGCAGGTTGATCAGCCGCGGCCAAGACGACGAAGCGACAATTTCCAAGCGGATGCAAAAAAGCTGGGATGAGATCAGCCATTGGGACGGGTATGATTATGTTCTGGTAAACGATGATCTGGATGTCACAGAACTGAAACTTAAGACCATTATATCCGCCGAGCGGCTGAAACGCACCCAGCAGCCCCAGCTGAGTGACATCGCACGGCGTCTGCAATCACAATTCGAGGATTTGAAATGAATGGCACAATCCTTCCTTGGCCAGAAGCGTTTACGTAAATATTATGGCAAAATCCGCGAAGTGCTGGAAATGCCGAACCTCATTGAGGTTCAGAAGTCTTCATATGACTTGTTCCTGAATTCAGGCGATGCTGAAACCCCCACCGATGGTGATGGTATTCAGGGCGTTTTCCAGTCGGTTTTCCCGATTAAGGATTTCAACGAGACATCCATTCTTGAGTACGTAAAATACGAGCTCGAAAAGCCGAAATACGACGTAGAGGAATGCCAGCAGCGCGACATGACATATGCCGCACCGCTTAAGGTCACGCTCCGTCTGATCGTGTTTGATGTGGATGAAGATACTGGCGCCAAGTCGGTCAAGGACATCAAAGAGCAAGACGTCTTTATGGGCGACATGCCGTTGATGACGCCAAACGGTACGTTTGTTGTAAACGGTACCGAGCGTGTTATCGTGTCCCAGATGCACCGTTCACCGGGTGTGTTCTTTGACCACGATAAAGGTAAAACGCACTCTTCAGGTAAACTCCTGTTCGCTTGCCGTATTATTCCTTACCGCGGTTCGTGGTTGGATTTTGAATTCGACGCAAAAGACATCGTGTTCTGCCGTATTGACCGTCGTCGCAAGCTGCCTGTCACCACGCTGCTGTATTCTCTTGGCCTTGACCAAGAAGCGATCATGGATGCCTATTACAACACCGTTACCTACCGTCTTGAGGCGGGCAAGGGCTGGGTTGCACCGTTCTTCCCGGAGCGGGTTCGTGGCACACGTCCCACCTATGATATCGTAGACGCAGATAGCGGTGAGATCCTGTTCGAAGCAGGTAAGAAAGTGACGCCTCGCGCGGTTAAGAAATTGATCGACGAAGGCAACGTGACATCCTTGCTGCTGCCTTACGATCACATTCAGGGCACGTTCGTATCCAAAGACATCATCAACGAAGAAACGGGCGCGATTTACGTCGAGGCCGGTGACGAGATGACACTGGAGTATGACAAAGACGGCACGTTGATCGGTGGCACAGCCAAAGAACTGATCGACGCGGGCATCACCGAAATCCCATTGTTGGATATCGATAACGTCAACGTTGGCCCCTATATGCGTAACACCATGGCGGCAGATAAAAACATGAACCGCGACACTGCGCTCATGGATATCTACCGCGTGATGCGTCCGGGTGAGCCGCCCACAGTTGAAGCCGCGTCGAACCTGTTCGACACGCTGTTCTTCGATTCCGAGCGTTATGACCTGTCGGCTGTTGGTCGTGTGAAGATGAACATGCGTTTGGCATTGGAAAAAGAAGACACCCAGCGCACGTTGGACCGCGATGATATCGTTGCATGTATCAAAGCGCTGGTTGACCTGCGCGATGGCCGTGGCGACATCGACGACATTGACCACCTTGGTAACCGTCGTGTGCGTTCCGTTGGCGAATTGATGGAAAACCAGTACCGTGTTGGCCTGCTCCGTATGGAGCGTGCGATCAAGGAACGTATGTCTTCTGTCGAAATCGACACCGTCATGCCACAAGACTTGATCAACGCAAAACCAGCGGCTGCTGCGGTACGTGAATTCTTCGGCTCTTCGCAGCTGTCGCAGTTTATGGACCAAACCAACCCGCTGTCCGAGGTCACGCACAAGCGTCGCCTTTCAGCGCTTGGACCAGGTGGTCTGACACGTGAGCGTGCAGGCTTTGAAGTACGTGACGTTCACCCGACCCACTATGGTCGGATGTGTCCGATTGAAACGCCTGAAGGTCCAAACATTGGTTTGATCAACTCGCTGGCTACTTTTGCCCGCGTGAACAAATATGGTTTCATCGAAACACCTTACCGCAAGGTAAAAGACGGTATCGTTTCGGATGACGTTCAATACATGTCCGCGACCGAAGAAATGCGCCACACTGTGGCGCAGGCCAACGCGCACCTTGATGAAAACATGAAGTTCGTAAACGATCTGGTTTCGACACGTAAATCGGGTGACTATACACTGTCACCTTCGATGAACGTAGACCTGATCGACGTTTCGCCAAAGCAGTTGGTCTCGGTCGCTGCGTCTTTGATCCCCTTCCTTGAGAATGACGATGCGAACAGGGCCTTGATGGGCTCGAACATGCAACGTCAGGCTGTGCCATTGCTGCGTGCAGAAGCGCCGCTGGTGGGTACAGGTATCGAAGAAGTTGTTGCACGCGATTCCGGTGCTGCCTATATGGCCCGCCGCGCTGGCGTGATTGACCAAGTGGATGCGACACGTATCGTTATCCGCGCAACAGAAGACCTTGAGTTGGGCGACGCAGGCGTAGACATCTACCGCATGCGCAAGTTCCAACGCTCTAACCAGAACACCTGCATCAACCAGCGTCCGCTGGTGAAAGTGGGTGAGACGGTTACAAAAGGTCAGGTTATTGCTGACGGTCCATCCACAGACATGGGTGAACTGGCTCTTGGTAAAAACGTGATTGTCGCGTTTATGCCTTGGAATGGTTATAACTACGAAGACTCCATCCTGATCTCCGAGCGTGTATCGCGTGACGACGTGTTTACTTCGATCCACATCGAAGAATTTGAAGTCGCCGCCCGTGATACCAAGCTTGGGCCAGAGGAAATCACACGCGATATTCCAAACGTTGGTGAAGAAGCGCTGCGCAACCTCGACGAGGCTGGCATCGTGTATATCGGTGCAGACGTTGAGCCGGGTGACATTCTGGTTGGTAAGATCACGCCAAAAGGCGAAAGCCCGATGACGCCGGAAGAGAAACTCTTGCGCGCCATCTTTGGTGAAAAAGCATCGGACGTGCGTGATACCTCGCTGCGTGTGAAACCGGGTGATTTCGGTACGGTCGTAGAAGTACGTGTGTTCAACCGCCACGGTGTGGAAAAAGACGAACGTGCGCTGCAAATCGAGCGTGAAGAAGTCGAACGTCTGGCGCGTGACCGCGACGATGAGATGGCGATCCTTGATCGCAACATCTTTGCGCGTCTGCGTGAAGTGATCCTTGGCAAAGTAGCTGTCAAAGGTCCACGCGGCGTCAAGCCAAACGCACAGATCACCGAAGAAGTGTTGGAAGTCCTGACACGCGGTCAGTGGTGGCAGATCGCCCTCGAAGACGAGGACGATGCGAAAATCGTTGAAGCGTTGAACGAGCAGTACGAGATCCAGAAACGGGCCTTGGATGCGCGCTTTGAGGACAAAGTCGAGAAAGTACGCCGTGGCGATGATCTGCCCCCAGGTGTGATGAAGATGGTCAAAGTATTCGTAGCGGTGAAGCGCAAGCTGCAGCCGGGCGATAAAATGGCCGGTCGTCACGGGAACAAGGGTGTTATCTCCAAAGTGGTCCCGATGGAGGACATGCCGTTCCTCGAAGACGGTACACCGGTCGATTTCTGTCTGAACCCGCTGGGTGTTCCATCGCGTATGAACGTTGGTCAGATTTTGGAAACACACATGGGCTGGGCCGCACGCGGTCTGGGCATCAACGTGGACGAGGCGCTGCAAGAATATAAGCGTTCCGGCGATATGACCCCTGTGCGTGAAGCGATGAAGCTGGCCTACGGCGATGATGTCTACGACGAAGGCATCGCTGGTATGGAAGAGGATGACCTTCTGGAAGCGGCGGGCAACGTGACCCGCGGTGTGCCAATTGCGACACCTGTTTTTGACGGGGCCAAGGAAGCGGACGTAAATGACAGCCTTGCACGTGCAGGCTTCGATACATCCGGTCAATCGGTTCTGTTCGATGGTCGCACAGGCGAGCAGTTTAGCCGCAAGGTTACTGTTGGCGTGAAATACCTGCTGAAACTGCACCACCTTGTGGATGACAAAATCCACGCGCGTTCGACCGGACCATACTCGCTGGTTACCCAGCAGCCATTGGGCGGTAAAGCACAGTTCGGTGGTCAGCGCTTTGGTGAGATGGAAGTCTGGGCACTTGAAGCCTACGGCGCTGCATACACCTTGCAGGAAATGCTGACCGTGAAATCGGATGACGTTGCTGGCCGTACAAAAGTGTACGAGAGCATCGTCAAGGGCGAGGACAACTTCGAGGCAGGTATTCCTGAATCGTTCAACGTTCTGGTCAAAGAAGTCCGGGGCCTCGGCCTGAACATGGAACTCCTGGATGCGGAGGATGAAGAGTAGATGCCGCTATATGAGCATGTGATGATCGCGCGTCAGGACTTGTCCAATACACAAGCCGAAGGCCTCATCGAACATTTTGGTACAGTTCTGTCCGACAACGGCGGAGCACTCGTGGATAGCGAGTACTGGGGCGTCAAAACGATGGCCTATAAAATCAACAAGAACCGCAAAGGCCACTATGCCTTCTTGCGCTCTGATGCACCGGCAACTGCCGTGCAAGAGATGGAACGCCTGATGCGTCTGCACGACGATGTTATGCGTGTTCTGACCATCAAAGTTGATGCGCACAAAGAGCTGCCATCGGTTCAGATGCAAAAACGTGACGAGCGCCCAGAGCGCCGTGAACGTCGCTGAGACATTTCCGAACTCTGCGTAGATACTCTCAACGAAGAACAGGCGCGGACCGCGCTCGAAAACCTTGCTGCGCAGTTGGTTGCAGCAAACACAGCGTATCATACCAACGATGCACCTGAAATTTCCGACGCAGAATTTGACCTGCTCAAACGCCTGAACGCAGATATTGAAAAGCGTTTTCCAAACCTCAAACGAAGCGACAGCCCCAGTGATCAAGTTGGTGCAGCTGTCGCAGCGGGCTTTGGAAAAATCACCCATAGCGTCGCCATGCTGTCGCTCTCCAATGCTTTTAGCGACGAAGATGTGCAGGAGTTTGACGGATCTGTTCGTAAGTACCTTGGTTTGGCTGCGGATGCTTCGCTGTCGTTCACGGCAGAACCAAAGATTGATGGCCTTTCGCTTTCGTTGCGGTATGAAAATGGCATCTTGGTACAGGCCGCCACGCGGGGCGATGGATCGGTTGGTGAAAACGTTACGGCCAATGCGCGCACAATCTCTGATGTACCTCACGAAATCACAGGCGCGCCAGAGGTTCTGGAAGTACGCGGCGAAGTTTACATGAGCCATGCGGATTTTGCGGCCCTTAATGCAAGGCAAGAAGAACGTGGCGGCAAGACATTTGCAAACCCGCGCAATGCTGCTGCGGGATCGCTACGTCAACTGGATGCCGAGATTACACGCTCGCGACCTTTGCGGTTTTTCGCATATGCTTGGGGCAGTATTTCGGCCCCCTTAGGGAACACACAATGGGAGTCGATTGAACATCTTTCCAAACTTGGCTTTTCGACAAACCCGCTGACCGCGCTCTGCGCGGGACCTACGGAAATGATCAACCACTACCGCGATATCGAAGCACAGCGCGCGGGTTTGGGGTATGACATTGACGGTGTTGTCTACAAAGTAAACGATTTGGCGTTACAAAACCGCCTTGGATTTCGGTCGACCACACCCCGCTGGGCCATCGCGCATAAATTCCCGGCTGAACTTTCTTGGACACGCCTTGAAGGGATAGATATTCAAGTGGGCCGCACAGGTGCGCTCAGCCCTGTGGCGCGACTGACGCCCGTGACTGTGGGCGGTGTGGTTGTCTCAAATGCGACGCTGCACAATGAGGATTACATCAAAGGGTTGGATAGCAAAGGCCAAGTGATCCGTGAGGGCAAAGATATTCGAATTGGTGATCTGGTTCAGGTCTACCGTGCGGGCGATGTGATCCCCAAGGTGGCGGATGTCGATATCACCAAACGCCTCGATGGTGTCACGCCTTTTGAATTCCCACAAACCTGTCCGCAATGCGGTAGCGATGCCATCCGCGAACCGGGGGATGCGGTGCGTAGATGTACGGGCGGGCTGATTTGCCCTGCGCAAGCTGTCGAAAAGCTCAAGCACTTTGTATCGCGGGGCGCCTTTGATATTGACGGTCTGGGCGCCAAGCAGGTTGAACAGTTCCATACCGATGGATGGGTGACCGAACCTGCGGATATCTTCACCCTCCAAGAACGTTTCGGCAGCGGCGTGCAGCAATTGAAGAACCGCGAAGGCTGGGGCGATAAATCGGCGGCGGGGCTGTTTGCTGCCATTGATGCCAAACGTAAAATCCCGCTTGCCCGTCTGTTATTTGGTTTGGGCATCCGCCATGTGGGCGAATCCGCGTCAAACATGATTGCGCTGCACTATGGCACATGGGCGGCATTGGAGCAGGCAATGGACGCAGCGCAACCGCAAGAGGGCGCTGCATGGGATGATTTGGTTGGTATTGATGGCATGGGCGCGGTGATGGCTGGGTCTCTTGTCGGTGCCTTTGCACAAGAGGCAGAGCGTGCTTCGATCAATCGGCTGGTGGCGCAACTGGATGTCCAAGAGGCCGTGCGCGCCGACACTTCTGGTTCACCTGTTGCGGGCAAGACCGTGGTGTTTACAGGCACCCTTGAAAAGATGACCCGCGCCGAAGCCAAAGCCAAAGCGGAGAGGTTGGGTGCAAAGGTATCGGGGAGCGTGAGCGCCAAAACCGATATTTTGGTTGCTGGACCCGGTGCCGGATCAAAGGAAAAGAAGGCGATAGAATTGGGCATCCAGATATTGGATGAAGACGGCTGGCTTGGGCTGGTTGACGGGCTATGAATGATCCACAAAAATTGGGCCGAACTGATCAAGCCAACACAGCTTGAAGTTAAACCCGGAAATGATCCTGCACGTCAGGCGACAGTGATTGCAGAGCCGCTTGAGCGTGGTTTTGGTCTCACAATGGGCAACGCGCTGCGCCGCGTTCTGATGTCCTCGCTGCAAGGCGCGGCAATCACATCCGTACAAATCGACAACGTTCTGCACGAGTTCTCGTCAGTTGCTGGCGTTCGTGAAGATGTCACCGACATCATCTTGAACCTCAAAGGCGTTTCCATCCGTATGGAAGTCGAAGGACCAAAGCGTCTGTCCATCTCCGCAAAAGGCCCGGGCGTTGTGACGGCTGGCGATATCGGCGAAACCGCTGGCATCGAAATCCTGAACCGCGACCATGTTATCTGCCACCTCGACGACGGTGCAGACATCTACATGGAACTGATGGTCAACACTGGCAAGGGCTATGTCTCTGCTGACAAGAACAAGCCAGAAGACGCGCCAATCGGTCTTATCCCGATCGATGCGATCTACTCACCTGTGAAAAAAGTCAGCTATGATGTTCAGCCTACCCGCGAAGGTCAGGTTCTGGACTATGACAAACTGACAATGAAAGTCGAAACCGACGGCTCCCTGACGCCTGACGATGCAGTGGCATTTGCCGCACGTATCCTTCAGGATCAATTGGGCATCTTCGTGAACTTCGACGAACCAGAATCAGCTTCCCGTCAGGACGACGACGATGGCCTCGAGTTCAACCCGCTTCTGCTCAAGAAAGTGGACGAGCTGGAACTGTCTGTACGTTCCGCCAACTGCCTGAAGAACGACAATATCGTGTATATTGGCGATCTGATCCAGAAGACCGAAGCCGAAATGCTGCGCACACCGAACTTTGGCCGCAAGTCCTTGAACGAGATCAAGGAAGTGTTGTCAGGCATGGGTCTGCACCTCGGCATGGACGTTGAGGACTGGCCACCAGAGAACATCGAAGATCTGGCCAAGAAGTTCGAAGACAACTTCTAAATGGCCAGAGATGACAACCGGGGTGGCAACCGCCGCAACCAGCGCGAAGAAGCGCCAGAATTCGCAGACCGCCTTGTCGCGATCAACCGCGTTTCCAAAACTGTAAAAGGTGGTAAGCGCTTTGGCTTCGCCGCTCTTGTAGTGGTTGGTGACCAAAAAGGTCGTGTGGGTTTTGGCAAAGGTAAAGCCAAAGAAGTGCCTGAAGCGATTCGTAAAGCAACTGAGCAAGCAAAACGCCAAATGATCCGCGTTCAGCTTCGCGAAGGCCGCACGTTGCACCATGATATGTCTGGCCGTCACGGCGCAGGCAAAGTGATCATGCGCACAGCACCAGAAGGTACTGGTATTATTGCTGGTGGTCCAATGCGTGCCGTATTCGAAATGCTCGGCGTCAAAGACGTTGTTTCCAAATCGATCGGTTCACAAAACCCTTACAACATGATCCGCGCCACCATGGACGGATTGAAAAAAGAACAGTCCCCACGTTCTGTCGCGCAGCGTCGCGGTAAGAAAGTGGCAGACATTCTGCCGAAGCGTGAAGATGCATCTGATTCTTCTGCTCAAGTCGCTGAGGAGGCATAAATGACCGACCCTATCGCAGATATGCTGACACGCATCCGCAACAGCCAGTTGCGCGGCAAATCCACAGTCATGACACCAGGTTCAAAACTGCGTGCATGGGTGTTGGACGTACTCGCCGACGAAGGCTACATTCGTGGCTATGAAAAAACGACAGGCGCCGATGGCCATCCAGCTATCGAAATCAGCCTGAAGTACTACGAGGGCGAACCTGTTATTCGTGAGTTGAAGCGGGTTTCCAAACCCGGTCGCCGCGTCTACATGGCCGCACAAGACCTCCCATCCGTCCGTCAGGGCTTGGGTGTGTCGATTGTCTCCACCCCACGGGGTGTGATGTCGGACGCAAGCGCACGCGCAGCCAACGTTGGCGGCGAAGTGCTTTGCACCGTATTCTAAATGCATGACATTCGTGCCATACGCGAAAACCCCGATGCTTTTGACGCCGCTTTGGCGCGGCGCGGCGATGCGCCTTTGTCCTCGTCTATTCTGGAGCTGGACACAGCACGCCGCACCAAAATTCAGGCCGCCGAGGCCGCCCAAGCCGAACAAAACGCCGCCTCCAAAAACGTAGGTGCCGCCAAGGCCAAAGGCGATGACGCCGAGTTCGAGCGTCTGCGCGCGCTGGTGTCCGAGAAAAAGGCCGAAGTTGCCGCCATGCAGGCCGAAGCCAAGGATCTAGATGTCCAGCTGACGGATATGCTGGCCCGTATCCCGAACACCCCTGCCGATGACGTGCCTGATGGCGCGGATGAAGCCGCGAATGTCGAGGTCAAGCGCTGGGGCGATGTGCGTGCCTTTGACTTCGCACCGCGTGAACACTTTGATCTGGATAGCGTCGCCGCCTCCATGGATTTTGAAACCGCCGCGAAAACATCGGGTGCGCGTTTTGTGATGTTGAAGCGTGGCGTGGCCCGCGTGCACCGTGCGCTGGCGCAATTCATGCTGGATACACATGTAGACGAAAACGGCCTGACCGAAGTCAACTCTCCGGTTCTGGTGCGCGACGAAGCCATGTATGGTACTGATAAGTTGCCTAAATTTGGCGAAGACAGCTACCGCACCGAAGAAGGCATGTGGCTGGTCCCTACCTCCGAAGTACCGCTGACCTATACAGTCGCGGGTGACACGCTGGACGCCTCCGATCTGCCCCGCCGCATGACGTCACACACGCTGTGCTTCCGTTCCGAAGCGGGATCAGCGGGCCGTGATACCTCGGGTATGTTACGCCAGCACCAGTTCGAAAAAGTCGAAATGGTGTCGATCACCCTGCCCGACGAGTCGGACGCCGAGCAAAAACGCATGCTGGGCTGTGCCGAGGGTATTTTGGAAAAACTGGGTATCCCCTATCGCACGTTAATCTTGTGCACAGGTGATATGGGCTTTGGCGCGCGCCGCACCTTTGATATCGAAGCATGGTTGCCCGGTCAGAACGCTTACCGCGAGATCAGTTCGGTTTCCACAACAGGTGATTTTCAGGCGCGCCGCATGAACGCACGTTTCAAACCTGCCGAGGGCGGCAAGCCCCAGTTTGTGCATACGCTGAACGGTTCTGGCCTTGCTGTGGGGCGTTGTTTGATTGCTGTGCTGGAAAATGGCCAGCAGGCAGACGGCACCGTCAAACTCCCCGCGGTTCTGGCCCCCTATCTGGGCGGCAAAACTGTGCTGGGCCTTGATGGGCAACTTGCGTAAATGGCAATTACAGCATCCATGGTCAAAGAACTCCGCGACACGACCGGCGCGGGCATGATGGACGCCAAAAAGGCACTGACAGAAAACAATGGTGACATGGAAGCCTCTGTTGACTGGCTGCGCACCAAAGGTTTGGCAAAAGCCGCCAAGAAATCCGGCCGTACAGCGGCAGAGGGTCTTGTAGCTGTTCAAGTTGAAGGCGGTCGCGGTGTTGCGGTTGAAGTTAACTCTGAAACCGACTTTGTTGGTAAAAACGCTGACTTCCAGAAAATGGTTGCTGGCATTGCCAAAGTTGCTGTTTCCGCAGCAGATATCGACGCGTTGAAAGCAGCAGACATGGGCGGCAAAACTGTCGAACAGACTGTTACAGATGCCGTAGCCGTTATCGGTGAAAACATGTCCGTACGTCGTATGAACTCTATCGAAGGTGACTTGGTTGTGTCTTACGTTCACAACGCAGCGGCACCTGGCATGGGCAACATCGGTGTTCTGGTCGCAATGACCGGCGGCGACGAAGCCTTTGGTAAACAGATTGCGATGCACATTGCTGCAACCAACCCTGCTTCCTTGTCCGAGGAAGATCTTGATCCAGCCGTGATCGAGAAAGAAAAGCAGGTCCAGATCGATATCGCTAAGGAATCAGGCAAGCCTGACGCCGTGATCGAAAAAATGATCGTTGGCCGTATGCAAAAGTACATGTCCGAAGTGACACTGGTAAACCAAGCATTTGTGATCAACCCTGACCTGACAGTTGCAGCAGCAGCCAAAGAAGCAGGCGCGACAATCACTGGGTTTGTACGCCTTGAAGTCGGCGAAGGCATTGAAGTTGTGAAAGAAGATTTCGCAGCTGAAGTTGCAAAAGTTTCTCAAGGCTAAATGAACCAGGAACTTACAAACAACCCGTTCAACCCGGTTGCACCTACAAAAACGTTTGACGAGATCAAGGTTTCCCTTGCTTCGCCAGAGCGTATTTTGTCGTGGTCTTTCGGTGAGATCAAAAAGCCAGAAACCATCAACTATCGTACGTTCAAGCCAGAGCGTGACGGTCTGTTCTGTGCGCGTATCTTTGGACCAATCAAAGACTACGAATGTCTGTGCGGCAAATACAAACGCATGAAGTATCGCGGCGTTGTCTGCGAAAAATGTGGTGTTGAAGTTACGCTGCAAAAAGTGCGCCGTGACCGTATGGGCCACATCGAACTGGCATCGCCAGTGGCACACATCTGGTTCCTCAAGTCTCTGCCATCGCGGATTGGCTTGATGCTGGACATGACCCTGCGTGATCTTGAGCGTGTTCTCTACTTTGAAAACTACGTTGTCATCGAGCCGGGCCTGACGGACCTCACCTACGGTCAAATGCTGACCGAAGAAGAGTATATGGATGCGCAAGACGGCTATGGCATGGATGCGTTCACCGCTAACATCGGTGCAGAAGCGATCCGTGAAATGCTGGCCGCGATCGACCTCGAAGCCGAGGCCGACCAACTGCGTGAAGAGCTGAAAGTCGCCACAGGTGAGTTGAAGCCGAAGAAGATCATCAAACGCCTCAAAGTGGTTGAGTCCTTCCTTGAATCTGGCAACCGTCCTGAGTGGATGGTTCTGACAGTGATCCCCGTGATCCCGCCAGAACTGCGCCCACTGGTACCGCTGGATGGTGGCCGCTTTGCGACCTCCGATCTGAACGACCTGTACCGCCGCGTAATCAACCGTAACAACCGTTTGAAGCGTCTGATCGAACTGCGCGCGCCCGATATCATCGTGCGTAACGAAAAACGTATGTTGCAGGAATCTGTGGATGCGTTGTTTGACAACGGCCGTCGTGGCCGTGTGATCACGGGTGCCAACAAACGTCCGTTGAAATCACTCTCCGACATGCTGAAAGGTAAGCAAGGTCGCTTCCGTCAGAACCTTTTGGGTAAGCGCGTCGACTTCTCTGGCCGATCAGTCATTGTGACGGGCCCCGAGTTGAAGCTGCACCAATGTGGTCTGCCGAAAAAGATGGCCTTGGAGCTGTTCAAGCCGTTCATCTACTCGCGCCTTGAAGCAAAAGGTCTGTCTTCGACAGTCAAGCAAGCCAAAAAGCTGGTGGAAAAAGAGCGTCCCGAAGTGTGGGACATCTTGGATGAAGTTATCCGCGAACACCCAGTCATGCTGAACCGTGCGCCTACCTTGCACCGTCTTGGTATTCAGGCGTTTGAACCCGTCTTGATCGAGGGTAAAGCCATCCAGCTGCACCCGCTGGTTTGTTCGGCCTTTAACGCTGACTTTGACGGCGACCAAATGGCGGTTCACGTGCCGCTTTCGCTGGAAGCCCAGCTGGAAGCACGCGTCTTGATGATGTCCACGAACAACGTTCTGTCGCCTGCAAACGGCGCGCCGATCATTGTTCCTTCACAGGATATGATCTTGGGCCTCTATTACGTCACGCTTGAGCGTGAAGGTATGAAAGGTCAGGGCAAAGTGTTCGGCACCGTCGACGAAGTGCAGCACGCACTCGACGCGGGTGAAGTACATCTGCACGCCAAAATCCAAGCGCGGATCAAACAGATCGATAACGAAGGCAACGAAGTTATCATTCGTTTCGACACCACTCCGGGCCGTATGCGTCTGGGTGCCTTGCTGCCGCTGAACGCAAAAGCGCCGTTTGACCTGGTCAACCGTTTGCTGCGTAAGAAAGAAGTGCAGCAGGTCATCGATACGGTTTACCGTTATTGTGGTCAGAAAGAATCCGTCATCTTCTGTGACCAGATCATGACATTGGGTTTCCGCGAAGCTTTCAAAGCCGGGATTTCGTTTGGTAAAGACGACATGTTGATCCCAGACAGCAAATGGCCACTGGTTGAAGAAACCCGTGACCAAGTGCGCGACTTTGAACAGCAGTACATGGACGGCCTGATTACTCAGGGTGAAAAGTACAACAAAGTGGTCGATGCATGGTCAAAGTGTAACGACAAAGTCACCGACGCGATGATGGGTGCGATTTCCGATACCACATACGCCGAAGACGGTTCCGAAAATGAACCAAACTCGGTTTACATGATGGCTCACTCCGGTGCGCGTGGTTCGGTTACGCAGATGAAACAGCTGGGCGGTATGCGTGGTCTGATGGCCAAGCCGAACGGCGATATCATCGAAACACCGATCATTTCGAACTTTAAAGAAGGTCTGACCGTTCTTGAGTACTTCAACTCGACACACGGTGCGCGGAAAGGTCTGTCGGATACGGCTCTGAAAACGGCTAACTCCGGTTACCTGACACGTCGTCTGGTGGATGTGGCGCAAGATTGCATCGTTCGTATGCATGATTGTGGTACAGACGTTGCGATCACAGCAACCGCTGCGGTCAACGATGGCGAAGTTGTTTCTTCGCTGGCCGAGCGTCTGTTGGGTCGTGTTGTTGCAGAAGACATCATGCGTCCAGGTACAGAAGAAGTTCTGATCGCCAACGGCACAATCGTTGACGAACGTCTGGCTGACATCATCGACGAAGCCTCTGTTGCCTCTGCACGGATCCGTTCACCACTGACATGTGAGGCCGAAGAAGGCGTCTGCGCAATGTGCTACGGTCGTGACCTTGCACGTGGTACACTTGTGAACCAAGGTGAAGCGGTGGGTATCATCGCGGCGCAGTCGATTGGTGAACCGGGTACACAGCTGACAATGCGGACATTCCACATTGGTGGCGTTGCGCAGGGTGGCCAGCAGTCCTTCCAAGAAGCAGGTCAGGCCGGTAAAATCCGCTTTGAGAACAGCAACACGCTGCAAAACTCTTCGGATGAAACCATGGTCATGGGCCGCAACATGAAACTGTTGATCATCGATGAAAACGGTGATGAGCGAGCGAGCCACAAAGTGGGTTACGGTACCAAGCTGTTTGTCAAAGAGGGCCAGTCTATTGCCCGTGGCGACAAACTGTACGAATGGGACCCCTACACGCTGCCGATTATTGCGGAAGCCAAAGGTATTGCCAAACACGTCGATCTGATCTCAGGCATCTCTGTCAAAGACGAGACCGATGACGCGACAGGCATGACCCAGAAAATCGTGATCGATTGGCGCTCTGCCGCCAAAGGTAACGAGCTGAAGCCAGAAATCATTCTGGTTGATGCAGATGGTGAACCTGTCCGTAACTCTGCTGGCAATCCGATCACCTACCCGATGTCCGTAGACGCGATTATGTCTATGGAAGAAGGTAGCGACGTTGAAGCGGGTGACGTTCTGGCGCGTATTCCGCGTGAAGGCGCCAAGACCAAGGACATTACCGGTGGTCTGCCACGTGTTGCGGAACTCTTTGAGGCACGCCGCCCCAAAGACCACGCGATCATCGCGGAAATCGACGGCTATGTGCGTTACGGCAAAGACTACAAGAACAAGCGCCGCATCGCGATCGAGAGCTCGGAAGATCCGGATCACAAGGTCGAATACATGGTGCCCAAGGGCAAGCACATCCCAGTGGCCGAAGGCGACTTTGTCCAGAAGGGCGATTACATCATGGACGGCAATCCAGCGCCGCATGACATTCTCGCCATTATGGGTGTCGAAGCTTTGGCTGACTATATGATCGACGAAGTTCAGGACGTTTACCGCCTGCAAGGTGTGAAGATTAACGACAAACACATCGAAGTCATCGTGCGTCAAATGTTGCAGAAATGGGAAATCCAGGAATCTGGTGATACCACGCTGCTCAAAGGCGAACACGTAGACAAGCTGGAATTCGACCAAGCCAACGAAAAGGCGATCTCTAAAGGGGGCCGTGTTGCCAAAGGCGAACCGATCCTCTTGGGTATCACCAAGGCGTCGCTGCAAACCCGCAGCTTCATCTCGGCGGCATCCTTCCAAGAGACCACACGTGTTCTGACCGAGGCATCTGTCCAAGGTAAGCGCGATAAACTGGTTGGCTTGAAAGAGAACGTCATCGTGGGTCGTCTGATCCCTGCGGGTACTGGTGGGGCAACCCAACAGATGCGCCGTGTGGCTGCTGATCGTGATAACGTTGTTGTCGAAGCACGCCGTATCGAAGCGGAAAAAGCTGCTGCACTTGCTGCACCAGTTGCGCCTGCGTCCGATGTGGTTGGCGGCGATGTGTTCTCTGACACGTCCGGGGACGAGGAAAGCCGCGATTAAATGTCACGCCGTCACGCCGCTGAAAAACGCGAAGTACTGCCAGACGCCAAGTTCGGCGATCTGGTTTTGACCAAATTCATGAACAACCTGATGATCGATGGTAAGAAATCGACCGCAGAGCGTATCGTGTACAACGCATTCGATCGCGTTGAAGCCAAAATCAAACGCGCACCAGTGGAAGTATTCCACGAAGCGCTTGAAAACATCCAGCCATCCGTCGAAGTGCGCTCGCGCCGCGTCGGTGGTGCGACTTATCAGGTTCCAGTAGAAGTTCGCCCAGAGCGCCGCGTTGCTCTTGCGATCCGCTGGTTGATCAAAGCCGCACGTGCGCGCAACGAAAATACCATGGAAGAGCGCCTTGCAGGCGAGCTGATGGACGCAGTTCAGTCCCGTGGTACAGCCGTTAAAAAGCGTGAAGATACGCATAAAATGGCCGACGCGAACAAAGCATTCAGCCACTACCGCTGGTAAATGCCCTATGCGCAAACCGACAAAAGCGAAGCGACACCCATTCTCGCTAATCCAGCGCCTGACGTACGCACCCGTCCCAAGCTTGAGGGCGGTAAGACATTTGTGCTTAAAACCGAATTCGAACCCGCAGGCGATCAACCGACGGCCATCAAGGAACTCTCCGAGGGTATCCGTAACGGTGAGCGCAATCAGGTGCTGTTGGGGGCCACGGGCACAGGCAAGACCTTTACCATGGCCAAGATGATCGAAGAAACGCAACGACCCGCGATCATCCTTGCACCGAATAAGACGCTCGCGGCACAGTTGTATGGTGAGTTTAAGGGGTTCTTTCCTGATAATGCGGTCGAATATTTCGTGAGTTACTACGACTATTATCAACCCGAAGCTTATGTTGCCCGCTCGGATACTTTCATTGAGAAAGAGAGCCAAATCAACGAACAGATTGACCGGATGCGCCACTCGGCCACGCGGGCCTTGTTGGAGCGGGATGATGTGATCATCGTGGCGTCGGTATCGTGTATCTATGGTATCGGCTCGGTTGAGACTTACGGCGCGATGACGCAGGATTTGACCGTTGGCAATAGCTATGACCAGCGGCAGGTGATTGCCGATCTTGTTGCGCAGGCCTACAAACGCAACGATGCCTCTTTCCAGCGTGGTGCGTTCCGCGTGCGCGGCGACAGCCTAGAAATTTTCCCTGCCCACCTTGATGATCGCGCTTGGAAATTGTCTTTCTTTGGCGAGGAACTGGAAAGCATTACAGAGTTTGATCCGCTTACGGGTGAAAAGACAGGCAACATGGACCAAGTGCGCATCTATGCGAATTCGCACTATGTGACGCCCAAGCCCACGATGAACCAAGCGGTGATCGGCATTAAAAAAGAGCTGCGCATGCGGTTGGATCAACTGGTAGGTGAGGGCAAATTGCTAGAGGCGCAGCGGCTGGAACAGCGCTGTAACTTTGATCTGGAGATGCTGGAGGCCACCGGCGTGTGCAACGGGATCGAGAACTATTCGCGTTATCTGACGGGCCGTGCGCCCGGAGAGCCACCCCCGACATTGTTTGAATTTATCCCCGACAATGCAATTGTTTTTGCGGATGAATCCCACGTCAGCGTGCCCCAGATTGGTGGCATGTACAAAGGTGACTTTAGGCGAAAAATGACACTTGCTGAACACGGCTTCCGCTTGCCGTCATGTATGGATAACCGGCCTCTCAAGTTTGAGGAATGGGACGCCATGCGGCCGCAATCTGTGTTTGTATCTGCCACCCCCGCCAATTGGGAGATGGAGCAAACGGGCGGTGTGTTCACCGAGCAGATCATTCGCCCCACAGGTCTGATTGATCCGGTCATCGAAATCCGCCCTGTAGAGATGCAAGTGGATGACCTGTTGGATGAAGTGCGCAAAGTGGCCGCCGAAGGGATGCGGACGTTATGCACCACGCTGACCAAACGCATGGCCGAGGATTTGACGGAATACATGCACGAACAAGGTATTCGTGTGCGCTATATGCACAGTGACATCGACACGATTGAACGGATCGAAATTCTGCGCGATTTGCGGCTGGGTGCTTTTGATGTGCTGATCGGGATTAACCTGCTGCGCGAAGGTTTGGACATTCCAGAATGTGGGCTTGTCGCGATCCTTGATGCCGACAAAGAGGGGTTTTTGCGCTCTGAAACATCACTGATCCAGACCATTGGCCGTGCTGCGCGTAATTCTGAGGGTCGCGTGATCATGTATGCGGATCGCATTACTGGCAGCATGGAGCGCGCCATGGGCGAGACAGAGCGCCGCCGCGTCAAGCAGCTTGCCTATAATGAAGAACACGGCATCACTCCGATGACTGTGAAAAAGAACGTCGAGGATATTTTGGCTGGTCTTTACAAAGGCGACACAGATCAATCGCGCGTCACAGCCAAGATCGATAATCCGCTGGCGGGTGGCAACCTGCAATCTGTTCTGGAAGGGCTGCGCACAGACATGCGCAAAGCAGCCGAAAACCTTGAATTTGAAGAGGCCGCACGCCTGCGTGATGAAGTGAAGCGGCTTGAATCCGTTGACCTTGCAATCGCGGATGATCCAATGGCGCGGCAATATGCGGTTGAAAAAGCGGTGGGCGAGGCCCGCGTTAAATCAGGCCGTAGCACAATGGGCCGTGGTGGTATGCGTGGCGGGGTAAAGCGGCGG---AGCCGTTAAATGGCTGCAAAACCATTTTTCCGCCGTCGCAAAGTGTGCCCCTTCTCGGGTGATAACGCTCCGGCGATCGACTACAAAGACACACGTCTTCTGCAACGCTACATCTCCGAGCGTGGCAAAATCGTGCCTTCCCGTATCACCGCAGTATCTGCGAAAAAGCAGCGTGAATTGGCCCGTGCCATCAAACGCGCCCGCTTCCTCGCCCTGCTGCCCTATGCTGTAAACTAAATGCCAAAGCGTATCCTGACAGGCACCGTAACCTCGGACGCCAACGAACAAACAGTAACCGTATCCGTAGAGCGTCGCTTTACACACCCGGTTCTGAAAAAGACCATTCGTAAGTCCAAAAAATATCGGGCGCACGATGAAAACAACACTTTCAAAGTGGGCCAGCAGGTCCGCATTATTGAATGTGCACCACGTTCCAAAACCAAGCGTTGGGAAGTAATCACTGCGGAAGCATGAATGGCCAATACACCACAAGCAAAGAAACGCGCACGTCAGAACGAAAAGCGTTTTGCTATCAACAAAGCACGCCGTTCACGTATCCGTACGTTCATCCGCAAAGTTGAAGAAGCAATCGAATCTGGCGTAAAAGACGATGCAGTTGCGGCACTGAAAGCAGCACAGCCAGAACTGATGCGGGGCGTCACAAAAGGCGTTTACCACAAGAACACAGCATCGCGCAAAATGTCGCGTCTGGCTGCACGGGTAAAAGCAATCGCTTAAATGTCTCGTTCAGTATGGAAAGGTCCTTTTGTTGACTCTTATGTCCTCAAAAAGGCCGAAGCCTCCCGCGAGAGCGGTCGTAACGAAGTGATCAAGATCTGGTCGCGTCGTTCCACGATCCTGCCGCAGTTTGTTGGCCTGACGTTTGGCGTCTATAACGGTCACAAACACATCCCTGTAAACGTCACAGAAGACATGATTGGTCAGAAGTTTGGTGAGTTCTCACCAACGCGGACCTATTACGGTCACGCAGCTGACAAAAAAGCGAAGCGGAAATAAGTGGCACGTATTGCCGGCGTAAACATCCCGACTGCAAAGCGGGTTCCAATCGCCCTCACATATATCACCGGTATCGGTAACTCTTCGGCTCAGGCCATTTGCGAAGCTGTAAAAATCGATTTTACACGTCGCATCAACGAGTTGTCTGATGACGAAATCTTGAAAATCCGCGAGTACATCGACGAGCACTATACCGTCGAAGGCGACCTGCGCCGTGACACACAGATGAACATCAAACGTTTGATGGATCTGGGGTGCTACCGTGGCCTGCGTCACCGTCGTAACTTGCCTGTACGCGGTCAGCGTACTCACACCAACGCTCGCACTCGCAAAGGCCCTGCTAAGGCCATTGCTGGTAAGAAGAAATAAATGTCCATGAAAATTCGCCTCGCCCGCGGTGGCTCCAAAAAACGCCCCTTCTACCGTATCGTTGCAGCCGACAGCCGTATGCCACGTGATGGCCGCTACATCGAAAAGCTGGGCACATACGCACCATTGCTGCCAAAAGACAGCGAAGACCGTGTTAAAATGAACATGGAGCGCGTCGAGTACTGGTTGGGTCAAGGCGCACAGCCAACAGACCGTATCCAGCGCATGCTGGAAGCTGCCGGCGTTCGCCCGAAGACAGAGCGCAACAACCCTAAAAAGGGTACACCAGGCAAAAAAGCGCAAGACCGCGTAGAAGAAAAAGCAGCCAAGGCAACAGCAGCGGCCGAAGCAGCAGCAGCGCCAGCAGAAGAAGCGGCAGCTGAAGAATAAATGTCGATTACGAAAGAAGAAAAAGCCCGCTTGATGAGCGAATTTGGTGCCAAAGAAGGCGATACAGGTTCCCCAGAAGTACAAGTGGCAGTTCTTAGCTCGCGCATCGCGACACTGACAGAGCACTTCAAAACACACAAAAAAGACAACCACGGTCGCCGTGGCCTGTTGAAAATGGTTGCGACACGCCGCAAGCTTTTGGACTATGTTAAGTCCAAAGATGAGGCACGTTACCAAGACTTGATCAAACGTCTGGGCCTGCGCCGCTAAATGCAAACGCTTAACGAAATCCGGTCAAGTTTTCTGAACTATTTCGGGGATAACGGCCACGCAATCGTGCCGTCCAGCCCTTTGGTGCCACGTAACGACCCTACGCTCATGTTCACAGCCGCTGGAATGGTACAGTTCAAAAACCTGTTCACTGGTGTTGAAACACGTGATTATTCCCGTGCCACAAGCGCACAAAAATGTGTGCGCGCTGGCGGCAAGCATAATGATCTCGACAATGTGGGTTACACGGCACGCCACCACACGTTTTTTGAGATGCTGGGCAACTTCAGCTTTGGCGACTACTTTAAAGAAGACGCTATTCCAATGGCTTGGGACCTGTTAACCAAGGTTTTCGGCATTGATGCCTCGCGCCTGTTGGTGACCGTTTACCACACGGATGACGAAGCGGTTGAAATTTGGAAGAAACATGCAGGTCTGAGTGATGATCGCATCATTCGTATCGCCACAGATGATAACTTCTGGTCCGCTGGTCCGACAGGCCCCTGTGGCCCTTGCACCGAGATTTTCTATGATCACGGCGATCACATCTGGGGCGGACCTCCGGGATCGCCTGAGGAAGATGGCGACCGTTTTGTGGAAATTTGGAACCTCGTGTTCATGCAATATGAACAGTTCGAGGATGGCACACGCCGTGATCTGCCTAACAAATCGATTGATACGGGCATGGGGATCGAGCGGGTTGCGGCCTTGCTTCAAGGGACCAATGATAACTACGCAACGGATTTGATGCGTAGCCTGATCGAGGCCTCTGCAAATGCGACCAGCAGCGATCCAGATGGCCCCGGCAAAACCCACCACCGCGTGATTGCGGATCACCTGCGTTCTACTTCGTTTTTGATGGCCGATGGCGTCATGCCGAGTAACGATGGCCGGGGATATGTTCTGCGGCGTATTATGCGCCGTGCCATGCGTCACGCGCATTTGCTGGGTGTCCAAGACCCGTTGATGCACCGTCTTGTGCCCGCCTTGGTCGCTCAGATGGGCCAAGCCTATCCAGAGCTGGGCCAAGCTCAAAGCATGATTGAGCAAACTTTGCTCCAAGAGGAAACGCGTTTCCGTCAAACGCTTGATCGGGGTCTCAAGTTGCTGGATGAGGAGCTAGTCTTGCTGCCAGAAGGCGCAAATCTGCCCGGCGCGTCGGCTTTTAAGCTTTATGATACATTCGGTTTCCCGCTTGATCTGACCCAAGACGCCTTGCGCGAAAAAGGCCGTGCGGTCGACACGGACGGGTTTGATACTGCAATGGCCGAGCAAAAGGCAAAAGCGCGTGCCGCTTGGTCTGGTTCAGGTGAGGCCGCAGATGCTGCGATCTGGTTTGACGTTGCTGACAAATCCGGCCTGACCGAATTTTTGGGATATGACACTGAAACCGCTGAAGGCAAAATTGCCGCATTGGTCCAAGATGGCAAGGCCGTCGTACAGGCGAATGCAGGCGACGAGGTGCAGATCGCGCTGAACCAAACGCCATTTTATGCCGAAAGCGGCGGCCAATTGGGCGATACAGGGCTGATCACAACCCAAACCGGCACTGCGCGTATTACAGACACGCGTAAGGCTGCGGGTGTAATTATTCACTTTGCAAAAGTTGAAAAGGGTAGCATTTCCAAGGATCAATCAGCCGTTCTTGATGTGGATCACACGCGCCGCACTGCGATCCGCGCAAACCATTCCGCAACCCACTTGCTGCACGAAGCCTTGCGTCACGCCTTGGGTGATCATGTCGCGCAGCGCGGATCGTTGAATGCACAAGACCGTCTGCGTTTTGATTTCAGTCACAACGAAGCGATCAGCTCTGACGATCTTGCGCGGGTAGAGACAGAGGTGAATGCGTATATTCGCCAAAACACCCCCGTTGAGACCCGTATCATGACACCGGATGACGCACGTGCCCTTGGCGCGCAGGCGCTGTTTGGTGAAAAATACGGCGACGAAGTGCGCGTTGTGTCGATGGGCCTGCAAGATGGGTCAAAGAAAGGGGCGGATGGTAACACCTATTCGCTGGAGCTTTGCGGGGGCACACATGTGCGCCGTACGGGCGATATTGGTGCCTTTGTGATGTTGGGCGACAGTGCCAGCAGTGCAGGCGTTCGCCGGATAGAGGCGCTGACAGGCGAAGCTGCTCTAAAGCATTTGCGCGATCAGGACAGCCTGTTGGCTCAAACCGCCTTGGAACTCAAAAGCCCATCATCTTCTGTGCCAGAACGGGTCCGCGCCTTGATGGATGAACGCCGCAGCCTTGCGAATGAGGTTGCACAATTGCGCCGTGAATTGGCAATGTCTGGT------GGTAGTAGCGATCAAGATGTGCGTGAAATCAACGGGGTATCCTTCGTTTCTCAGGTCCTGACTGGTATCACAGGTAAAGACTTGCCTGCCTTGATGGATGAACACAAAGCCCGCATTGGCTCGGGTGCTGTGCTTCTTATTGCCGATACGGGTGACAAGGCCGCTGTTGCCGCAGGTGTCACCGATGATCTCAAAGGCACGCTCTCTGCTGTTGATATCCTACGTGCCGCTGTGGCCGAATTGGGGGGTAAGGGCGGCGGTGGTCGTCCTGATATGGCTCAGGGTGGTGGGGCCTCGGCCGAGAACGCACAAGCGGCCATTGCCGCCGCAGAAACCGTGATTAAAGGATAAATGGGTTTCAAAATGGGTATCGTCGGTCTGCCGAATGTTGGCAAATCAACGCTTTTCAACGCGCTTACTCGCACGGCCGCCGCGCAGGCTGCTAACTTCCCGTTCTGCACGATTGAGCCGAACGTCGGCGAGGTTGCTGTACCGGACGCGCGTCTGGATAAATTAGTCGAAATCGCCAAGTCCCAATCCATCATCCCAACGCGGATGACCTTTGTGGATATCGCGGGTCTGGTCAAAGGCGCGTCCAAAGGCGAAGGCTTGGGCAACCAGTTCCTTGCCAACATCCGCGAGACTGATGCAATTGCGCACGTTCTGCGTTGCTTTGAGGATGATGATGTCACCCATGTCGAAGACCGTGTTGATCCTGTGGCCGATGCTGAAACCATCGACACGGAATTGATGCTGGCCGATCTGGAGAGCATCGAGAAACGTCGTGCAGGTCTGGTGCGTAAAATCAAAGGCAATGACAAAGACGCCGTGCAGCAGGACCGCCTTTTGGCTCAAGCCCAAGCCGCCATTGAAGACGGTCAACCCGCCCGTGTGGTTGAAGTAGACGAAGATGACGCGAAGGCATGGCGGATGCTGCAACTGCTGACCACCAAACCCGTTCTGTATGTGTGTAACGTGGGCGTAGATGACGCTGCCAAAGGCAATGCACATTCTGCCGCTGTTGCCGCCATGGCCGAAGCGCAAGGCAATTCCGCGGTGGTTATCTCGGCCCAGATCGAAGAAGAGATCAGCCAGCTTGATGCAGATGACGCGCAGATGTTCCTTGATGATATGAACCTCGAAGAAGCAGGGTTGGATCGCTTGATCCGAGCGGGCTATGAGCTACTGCACCTTGAAACATATTTCACCGTTGGCCCGAAAGAAGCCCGCGCGTGGACCATCAAACAGGGCACGTCAGCCCCCAAAGCCGCAGGCGTCATTCACGGCGATTTTGAAAAAGGCTTCATCCGCGCCGAGACGATTGCCTATGATGATTTCGTCACGCTAGGCGGCGAAGGCCCTGCAAAAGAAGCAGGTAAAATGCGTGCAGAAGGCAAAAGCTATATCGTCAAAGATGGCGACGTGCTGCACTTCTTGTTTAACACCTAAATGGGCTGGAAATCACTTGATGACATGGACCTGCACGGCAAGCGGGTTCTGGTACGCGTCGATATCAACGTTCCCGTTGAAAAAGGCCAAGTCACAGATGCCACGCGAATCGAACGGATTGTACCAACGGTGCATGATATTCTGGCGAAAGGTGGCTCGCCCATTTTGCTGGCCCATTTTGGCCGCCCCAAAGGCAAGGTCAACCTGGACATGAGCCTGCGCCAAGTGGTCCCTGCCCTGCGCCACGCGCTGATGCGCTCGGTTGCATTGGTAGAGACCTTGGAGGCTGCCGAAAAGATGACCGCCGAAGTGGCCGCCGCCGAGGTTGAGCTGATCGAGAACATCCGCTTCCATGCGGGCGAGGAAGCCAACGATCCTGAATTTGCACAGCGTCTGGCAAAATTGGGCGATGTGTATTGCAACGATGCGTTCTCTGCAGCGCACCGCGCCCATGCCTCGACCGAAGGCATTGCGAAATATCTGCCGTCATGCGCAGGCCGTTTGATGCAGGCCGAACTGTCGGCACTGGAATCTGCGTTGTCCAAACCCGAACGCCCTGTTGGGGCGGTTGTGGGTGGTGCCAAAGTTTCGACCAAGATCGCGCTGCTGGAGAACCTAGTGAACCGTCTGGACGTGCTAGTTATTGGCGGCGGCATGGCCAATACATTCCTTGTGGCGCAAGGCGCGCAATTGGGCGCATCTTTGTGCGAGATGGATTACTTGGACACCGCACGCGATATTATGGCGCAAGCCGCCAAGGCGGGTTGCCGCGTGATCCTGCCCGTAGATGGCTTGGTTGCGACCGAATTCAAATCCGGTGCCGCGCATGAGGTTGCCCTGCTTGGGCCAGATACAGTTCTGGATGCGGATCAAATGGTATTGGATGCAGGCCCCGAAACCATCAAACAGATCAAGATCGCCTTTGAAGGATTGAAAACACTGATCTGGAACGGACCTATGGGCGCGTTTGAAATCGCCCCCTTTGATACGGCCACCGTTGCGGCGGCCCAATGCGCAGCGGAACGCACCCGCAATGGCGCGTTGATTTCTGTTGCGGGTGGCGGTGATACCGTCGCGGCGTTGAACCAAGCGGGCGTGGCGGATGACTTCACCTATATCTCCACCGCAGGCGGTGCCTTTTTGGAGTGGATGGAAGGGAAAACCTTGCCCGGCGTGGCCGCATTGATAAAGTGAATGGCTCTTCCTGAGTTCTCCATGCGCCAATTGCTCGAAGCAGGCGTACACTTTGGTCACCAGACACAGCGCTGGAACCCACGCATGGGCCCGTACATCTACGGCGCGCGTAACGGCATTCACATCATGGATTTGACACAGACCGTCCCAATGTTGGAAGACGCTCTAAAAATCATCCGTGATACAGTCGCCAAAGGCGGCAGCGTTCTTTTCGTTGGTACCAAGCGTCAGGCTGCACAGCCGATCGCCGAAGCCGCCGAAAAATGCGCACAGTATTACATGAACCACCGTTGGTTGGGCGGCACGCTCACAAACTGGCAGACTGTTTCTAAATCCATCCAGCGTCTGAAGCACATCGATGAGCAATCTGAAATGGGTTTCTCTGGCCTGACGAAAAAAGAACGTCTGGGCATGGAACGTGACCAAGGCAAGCTTGAAGCTTCCCTCGGTGGTATCCGCGAAATGGGCGGCCGTCCTGACTTGATCTTTGTCATCGACGTTCGCAAAGAGCAGCTGGCAATCGCAGAAGCCAACAAGCTGGGTATTCCAGTTGTGGCCGTGGTTGACACAAACTGCTCACCAGACGGCATCGATTACATCATTCCGGGCAACGATGACGCGGCACGCGCTATCGCACTCTACACCGATCTGGCAGCACGTGCTGCTCTTGACGGTATGTCCGCACAGCTGGGCGCCGCTGGCGTTGATCTGGGCGCGATGGAAGAAGCGCCAATGGAAGAAGCACTC---------------------GCTGAAGAAGCGGCTGCAGAAGCTCCTGCCGCAAGC---------TAAGTGACTAAACGTACAGCTGCCAAGCATAAAATTGACCGTCGCATGGGCGAAAACATCTGGGGCCGCGCAAAATCCCCAGTTAACCGTCGTGAATATGGCCCCGGCCAGCACGGTCAGCGCCGTAAAGGCAAGATTTCCGATTTCGGTATCCAGTTGCGTGCCAAGCAGAAGCTGAAAGGCTACTACGGCGACCTGACCGAGAAGCAGTTCCGTCGCATTTACGGCGAAGCCGAGCGTGTTAAAGGCGATACAGGTGAAAACTTGATCGGTCTGCTGGAACGCCGTCTGGACGCGGTTGTGTACCGCGCCAAGTTCGTTGCGACAGTTTTTGCTGCACGTCAATTCGTAAACCACCGCCACGTACGCGTGAACGGCAAGTTGGTTAACATTCCTTCCTACCGTGTAAAAGAAGGTGACGTGATCGAAGTACGTGACCGTTCCAAGCAAATGGTTGCGCTGGTTGAAGCCACACAATTGGCTGAACGCGATGTGCCTGACTACCTCGAAGTTGATCACTCCAAAATGACAGCGACATTCGTACGCACCCCTGCTTTGGGCGACGTGCCGTACCCTGTTGTTATGGAACCAAACCTCGTCGTGGAATTCTACGCGAAGAACTAAATGGGTAATAAAGTCAATCCAATCGGCATGCGCCTTCAGGTTAACCGTACGTGGGATAGCCGCTGGTATGCTGACACCAAAGACTTTGGTGACCTTCTTCTCGAAGACCTTGCGATCCGCAAGTTCATCAAAAAAGAATGCCATCAGGCGGGTATCTCCCGTGTGATCATCGAACGTCCGCACAAAAAGTGCCGCGTTACGATCCACACAGCGCGCCCAGGTGTTATCATCGGCAAGAAAGGTGCAGACATCGAAGGTCTGCGCAAGAAGCTGGCGGCATTCACCGCGTCTGAATTGCACCTCAACATTGTTGAAGTTCGCAAGCCAGAGCTGGATGCAGCACTTGTTGGTGAATCCATCGCACAACAGCTGGAACGTCGGGTTTCTTTCCGTCGCGCCATGAAACGTGCGGTACAGAACGCAATGCGTATGGGTGCCCTTGGTATCCGTGTGAACCTTGCTGGCCGTCTTGGCGGTGCAGAAATCGCGCGTACCGAATGGTACCGTGAAGGTCGCGTTCCGCTCCACACACTGCGTGCTGACATCGACTATGCACATGTTGAAGCAATGACTGCCTATGGCATCATCGGCATCAAGACGTGGATCTTCAAAGGCGAAATCATGGAACATGATCCAGCTGCCCGTGACCGTAAGGCACAAGAAATGCAAGACGGCCCAGCACCTCGTGGTGCCGGCGGTCGGCGTTAAATGGCACGCAGACGCAAAGGGCGCGATATTTCTGGCTGGGTTGTGATCGACAAACCCGCAGGACCGACATCGACAACCGTGGTCAACAAAGTGCGTTGGGCATTTGACGCGCGCAAAGCAGGTCATGCAGGCACGTTGGACCCTGACGCGACAGGCGTTCTGGCCATCGCGCTGGGTGAGGCTACCAAAACGGTTCCTTACATCACCGATGCGCTTAAGGCTTATGTGTTCACCGTGCGTTTGGGGGTTTCCACCAATACCGATGATGCCGAAGGCGAAGTTCTTGCCACGACCGATCTACGCCCTGATGACAGCGCGATCAAAGAGGCTCTGAACGGTTTTGTCGGTGACATCGAGCAAGTGCCACCGCAGTTTTCTGCCGTCAAAATTGACGGAGAGCGGGCCTATAAGCGCGCCCGCGATGGGGAACAGATGGAGATTGCGGCACGCCCGCTGTTTGTCGAAAGCCTGATCATGTTGGACCGCCCCGATGTGGATCACGTGACGCTAGAGATGGTGTGTGGCAAAGGCGGCTATGTCCGCTCGATTGCCCGCGATCTGGGCGAGGTTTTGGGCTGCAAGGCCCATGTCCGCGAATTGCGCCGCACGTGGTCTGGGCCGTTTGACGTAGATAACGCGATTACGCTCGAACAGGTTGACGAGATGGCGAAAACCTTGGCCCTTGATGAATTCCTCGGACCGCTTGAAATGGGGTTGCAGGACTTGCCACAGGTCACTGCCAGTGCCGAGGGGGCGAACAAGCTGCGCCATGGTAATCCCGGTATGGTGTTTGCCAAAGACGTTGAATACGGGGATGAATGCTGGGCCGTATATGAGGGGCAAGCCGTTGCCGTTGGCACATATCGTGCGGGTGAACTGCACCCTTCACGTGTGTTTAAC---ACTGGCTAAATGTTTGTTCTTGGTCTCACAGGTTCCATTGGCATGGGGAAATCCACCACTGCCCAAATGTTTGTCGAATTAGGCTGCGCGCTGTGGGATGCGGACGCGGCTGTGCACCGGTTGTATGCGCAGGGTGGCGCTGCGGTTGCGCCTTTTGCTGCTGCCTTTCCTGATGCTATCGTTGACGGCGCTGTGTCGCGTCTCGCATTGAAAGACATCATTGGCCGCGATCCTGCGGCGTTGAAGCAGATAGAACAGATCGTACATCCGTTGGTAGGTGAGGACCGTGCCGATTTCCTTACCCAAACCACTGCCGATATTGTGGTTCTGGATATTCCACTTTTGTTTGAAACAGGCGGCGATGCGCGTGTGGATGCGACGGCCTGCGTGTTCACCGATGACGCCACCCAAGAGGCGCGGGTGATGGCACGCGGAACAATGACGCGTGAGCAGTTTTTATCGATTAAAGCCAAGCAAATGCCAGCCGCTGAAAAATGCACCCGTGCGACCTATGTGATCCAGACGGATACGCTTGAACACGCGCGCACGCAGGTGCAGAACGTGGTTGAAACCATACGGAGCCAGCTAAATGCG---TGAATGGACGCGCTTGATATCGTTTTTGAGGCCGGTGCTTGGAGCACGGACGAGTTTACTCCGCTGGCGCAAGGGGCAGTGATTGCTACCTTGCGCCATATGGCGCTTGAGGCTGACGCCTGCGAGATCACGCTTCTTGCCTGCGATGATGCACGTATTGCTGAGTTAAACGCAGAATTCCGCGGCAAACCAACGGCCACAAATGTGCTCAGTTGGCCAGCGCAGCCCGTTGCGCCCCCGTCTGAGGGGCAAGCACCACCAGCAGTTGAACGCGGGTTTGATGACATGTTTGAATTGGGCGACATCGCCCTCAGCTATGAGACGTGTGCACGTGAGGCTGCCGAATCAGGCAAACCCTTCAGTGACCATCTGACGCATCTGATTGTGCACGGAGTGTTACATTTACTCGGCTATGATCATGAAACTGACGGCGATGCAGCGCTTATGGAGGGGTTGGAGGTTGAAATTCTTGGTAATCTCGGGCTGGATGACCCATATAGCGTTGATATTATATAGATGGCACGCTATATTTTCATCACCGGCGGTGTTGTATCCTCACTTGGCAAAGGTCTGGCATCCGCAGCCCTTGGCGCATTGCTTCAGGCACGTGGATTTTCTGTCCGCCTGCGCAAGCTTGACCCCTACCTTAACGTTGATCCCGGCACGATGAGCCCGTTTGAACATGGCGAAGTGTTTGTCACGGATGACGGCGCTGAAACCGATCTGGATCTGGGCCACTATGAGCGGTTCACAGGCGTTTCTGCGCGCAAAACTGATTCCGTCTCATCGGGGCGCATCTATTCTACCGTTCTGGAAAAAGAACGCCGTGGCGATTATCTGGGCAAAACCATTCAGGTGATTCCGCATGTAACAAATGAGATCAAAGATTTCCTTGCTGTGGGTGAAGACGAAGTTGATTTCATGCTTTGCGAAATCGGCGGTACGGTAGGTGACATCGAAGGCCTGCCCTTCTTTGAAGCGATCCGCCAGTTCAGCCACGACAAACCGCGTGGCCAATGTATCTTTATGCACCTCACCCTGCTGCCCTATCTGGCCGCTTCGGGTGAATTGAAAACAAAGCCCACCCAGCACTCGGTCAAAGAACTGCAAAGCATCGGCATCGCGCCCGACATCCTTGTTTGCCGCTCTGAACATCCGATCCCCGAAAAAGAGCGCGAGAAAATCGCCCTGTTCTGTAACGTGCGCAAAGACTCTGTTGTGGCGGCATATGATCTGAAATCCATCTATGAAGCCCCGCTGGCCTATCATGCACAAGGTCTGGATCAGGCAGTATTGGACGCGTTTGATATCTCCCCTGCCCCGCGCCCCGATCTGTCCGTTTGGAATGATGTCTATGACCGCATTCACAACACCGACGGCGAAGTGAAAGTGGCGATTGTTGGTAAATACACCCAATTGGGCGATGCCTATAAATCCATCGCCGAGGCGCTGACACATGGCGGCATGGCCAACCGTGTACGGGTGAACGTGGAATGGGTCGACGCCGAAGTCTTTGATTCAGACGATGTGGCCCAACATCTGGAAGGCTATCACGCCATCCTTGTTCCCGGCGGCTTTGGCGAACGCGGCACAGAGGGTAAAATCAAAGCAGCCCAATACGCCCGTGAAAACAAAGTACCCTATCTGGGCATCTGTCTGGGCATGCAAATGGCCGTGATCGAAGCCGCGCGCAACGTTGCGGGCCTGTCCACTGCTGGTTCCGAAGAATTCGACCACGAATCGGGCAAACGCCGCTTTGAACCTGTGGTCTATCACCTCAAGGAATGGGTGCAGGGCAATCACAAGGTTGAGCGCAAAGTCGGCGATGACAAAGGCGGCACGATGCGTCTGGGTGCCTATGATGCGACACTCAAAGAAGGCAGCAAGGTTGCCGAAGTCTATGGCACCACTGCAATCGATGAACGCCATCGCCACCGCTATGAGGTCGACATCGCCTACCGCGAACAGCTTGAGGCCGCAGGCATGACCTTCTCGGGCATGTCGCCTGATGGCAAATTGCCTGAAATCGTGGAATGGTCTGATCATCCTTGGTTCATCGGCGTGCAATTCCACCCAGAGCTGAAATCAAAGCCTTTCAAACCGCACCCGCTGTTTAAAGACTTTGTACGCGCCGCCAAAGACATGTCGCGGCTGGTTTAA---ATGACTTCCCTCACCCTCTTAGGTATTGAAAGCAGTTGCGATGACACCGCCGCCGCCGTTTTGCGC---GGGCAAGCAGGGGATGCGCAGGTTCTCTCCTCTATTGTGATGGGGCAAACCGCGTTGCATGCAGATTTCGGCGGTGTCGTGCCAGAAATCGCAGCACGTGCGCATGCTGAAAAGCTGGATTTATGTGTTGCGCAGGCACTTGAGGCGGCAGAGCTTAGCCTTGATGACATTGATGCTGTCGCGGTCACGGCTGGCCCGGGGCTGATCGGGGGCGTTGTATCCGGGGTCATGTGCGCCAAAGGCATTGCACTGGGGCGGGGCCTGCCGCTGTACGGTGTGAACCATCTGGCGGGGCATGCCCTAACACCGCGCCTGACAGATGGCGTGACCTTTCCGTACCTGATGTTGCTTGTTTCTGGCGGGCATTGCCAATTCCTTATCGTTTCGGGGCCAGATCAATTCCAGCGCCTTGGCGGTACGATTGATGACGCACCGGGCGAAGCCTTTGACAAAGTCGCACGTTTGATTTCCCTTCCCCAACCGGGTGGCCCTGCGATTGAAACCCGCGCCAAGGCTGGCGATTCAAAACGGTTCAAACTGCCGCGTCCGTTGCTGGACCGTCAAGGATGCGACATGTCATTTTCGGGCCTGAAAACCGCCGTGCTGCGCCAGCGCGATCATCTACTGAAGGAT---GGCACGTTATCTGCCCAAGATCAGGCAGACCTTGCCGCAGGGTTCCAAGCCGCTGTTGTGGATGTGCTGGCCGAAAAAACTCGCCGCGCCTTGGCGGTTTATGCCCCGATGACAGAT---ACGCCCAGCATTTGTGTCGCGGGTGGCGTTGCCGCGAACATGGCCATTCGTAGCGCATTGGAAACGGTTGCACACAAATTTGATGCCGCGTTCATCGCGCCGCCGCTGGCCCTATGCACGGATAACGCTGCGATGATCGCCTATGCCGCGTTGGAACAGATGGGCACCCGTGCGCCAGACGGCATGGACCTGTCGGCGCGTCCGCGCTGGCCATTGGATCAAAGCGCGCCCGCGATGTTGGGTAGTGGCAAAAAGGGAGCAAAAGCATGAATGGCAAAGAACAAATCTCATGGGGGCGCTGGCCCCACTCAACGGCAACTGCGTGTAGGCGAGACAACCCGCCGCGCGTTAAGCGAAATCCTTGCACGTGGCGATGTTCACGATCCCGAGCTGAACCGCATGTCCATCACCGTGGGCGAAGTGCGCATGTCGAATGACCTGCGCATCGCAACGGTGTATGTGCTGCCCTTGGGCGGGATCGGCAAAGAAGACGTGCTTAAAATGCTGGCTCGCAACAATTACGAGTTGCGCAGGATGGTTGCAAAAAGGCTCACGCTGAAATTCGCACCCGAGTTACGTTTCCAATTGGACATGACTTTTGACCAAATGGACGAAACCAGCCGGATGTTGTCACAGGATATTGTGCGCCGCGATGCGGATGCGGATTGA

>'Su-dubiusDSM-16472'

GTGAAAGCCAGCGAACTGCACGACAAGACGCCGGACCAGCTCCGCGACGAGCTTGTGAACCTGAAAAAAGAATCCTTCAACTTGCGTTTTCAGCAGGCCACCGGCCAGCTGGAGAACCCTGCACGTTTGAAGACCGTGAAGCGTGACGTGGCCCGTGTCCACACCGTGCTGAACCAGAAAGCCGCTGCTGCGGCAGCCGAA---TAAATGGCAATGGCAGATCTTTTGACAATGGACAGCAAGAAAACCGCGGAAAAGCAAAAGGCGCTCGACAGCGCGCTGGCCCAGATCGAACGTCAGTTCGGCAAAGGCTCGATCATGAAATTGGGCGCCGAAGGGGCGATCCAAGACATCAAGGCCAGCTCAACAGGCTCGCTTGGCCTCGATATCGCGCTTGGCATTGGCGGTTTGCCGATGGGGCGTATCATCGAGATTTACGGCCCGGAATCTTCGGGTAAAACCACGCTGACCCTGCACTGCGTGGCGGAACAGCAAAAAGCTGGCGGCGTTTGCGCCTTCGTCGATGCGGAGCACGCGCTTGACCCACAATATGCGAAAAAGCTTGGCGTGGATATTGATGAACTGCTGATTTCGCAGCCTGACACAGGCGAACAGGCGTTGGAGATTACTGATACGCTCGTGCGCTCGGGTGCGGTGAATATGGTTATCGTCGACTCGGTGGCGGCTTTGACACCAAAATCCGAACTCGAAGGCGAAATGGGTGACAGCAGCGTCGGCGTGCAGGCACGACTGATGAGCCAAGCCATGCGGAAGCTCACCGGTTCGATTAGCCGCTCCAATTGTATGGTGATCTTCATCAACCAGATCAGGATGAAGATCGGCGTCATGTTCGGCTCGCCCGAGACGACGACCGGCGGTAATGCGTTGAAATTCTATTCGTCTGTCCGGCTCGACATTCGCCGCATCGGCGCATTGAAGGACCGCGATGAAGTTGTCGGCAATGCGACGAAGGTTAAGGTCGTCAAGAACAAGGTTGCGCCGCCCTTCAAACAGGTCGAGTTCGACATCATGTATGGCGAAGGTATTTCGAAAATGGGCGAATTGCTTGATCTTGGGGTCAAGGCTGGCGTGGTCGATAAGTCAGGATCATGGTTCAGCTACGGTGATGAGCGGATCGGGCAGGGGCGTGAGAACGCTAAGAACTTCCTGAAAGAAAATACCGCCATGGCCTCCGAGATCGAAGACAAGATCCGCGCGGCACATGGGTTGGATTTTGATGGATCCGGTGGTGACGACGCGGATATCCTTGAAGCGTAAATGCCCCGCTATACCCCCGCCGAGATCGAAGCCCGCTGGCAGCAAGCCTGGGAAAAGGACGGCATTTTTCAGGCCGTTCGCAACGCTGACAAGCCGAAGTACTATGTGCTTGAGATGTTCCCCTACCCCTCGGGTCGCATCCACATGGGCCATGTGCGCAACTACACGATGGGCGACGTGATCGCGCGCTACAAGATCGCAACAGGGCATAACGTGCTGCACCCGATGGGCTGGGACGCCTTTGGCATGCCCGCCGAAAACGCCGCCATGGCCATTGGCGGCCACCCGGCGGATTGGACCTATGACAACATCGCCGAGATGAAGAAGCAGATGAAACCTCTAGGCCTGTCGATCGACTGGTCGCGTGAAATCGCCACCTGCCACCCCGGTTACTACGGTCAGCAGCAAGCGCTGTTTCTCGACTTCCTCAAAGAGGGTCTGGTCTACCGCAAGAACGCCGTGGTAAACTGGGACCCGGTCGATATGACCGTGCTGGCCAACGAGCAGGTCGAAAACGGCTGCGGCTGGCGCTCTGGTGCGCCGGTGGAGCGGCGTGAGTTGACGCAATGGTTCTTCAAGATTTCGGACCACTCCGAGGAACTGCTCTCTGCGCTCGACAGTCTCGACAACTGGCCCGCCAAGGTCAAACTGATGCAGGCCAACTGGATCGGCAAATCGCGCGGGCTGCAGTTTGCCTTCTCGACCATTGAAGCGCCCGAGGGTTTTGACCGGATCGAGGTCTATACAACCCGCCCCGACACGCTGCTGGGTGCCTCCTTCGTCGGCATCTCGCCCGACCATCCGCTCGCCAAGACGCTGGAGCGGGACGACGAAGCCGTCGCCGCCTTCTGTGCCGAATGCCGCAAGGGCGGCACCACCGAGGAAGCCATCGAAACCGCCGAGAAGCTGGGCTATGACACCGGCATCCGCGTGCGCCATCCCTTTGACACGGCACATGAATTGCCGGTCTATATCGCGAACTTCATCTTGATGGACTACGGCACCGGCGCGATTTTTGGCTGCCCCGGCCACGACCAGCGCGATTTCGATTTCGCCAGCAAATACGACCTGCCGATCATCTCGACCTTCCTGCCTTCGGAAGACGCCTCGCCCAAACTGGCCGAGGCCTTCGTGCCGCAGAAGTCGGAAAAGGTGTTCTACAACCGTGGCTTCGCGGGCAACCAGTGGCAGACCGGCGAAGACGCCGTCGACGCCGCCATCGCCTTTTGCGAGGAAAACGGCATTGGCCAAGGCGTCACCAAATACCGTCTGCGGGACTGGGGCCTCTCGCGTCAGCGCTACTGGGGCTGCCCGATCCCGGTCATTCATTGCGATGACTGCGGCGTGGTGCCCGAAAAGAAAGAGAACCTGCCGATCGAGCTGCCCTACGATGTCACCTTTGACACCCCCGGCAACCCGCTTGACCGCCACCCGACATGGCGCAACTGCGCCTGCCCCGCCTGCGGCAAGGACGCCCTGCGCGAGACCGACACGATGGACACCTTCGTCGACAGCTCCTGGTATTTCGCCCGTTTCACAGCTCCGCGCGCGGAGACGCCGACCGTCATGGAAGACGCGCAATATTGGATGAACGTCGATCAATATATCGGCGGCATCGAACACGCGATCCTGCACCTGCTCTACTCGCGCTTCTTTGCCCGTGCGATGCAGATCACCGGCCACCTGCCCGAAAGCGCTGTTGAGCCCTTCGACGCGCTCTTTACCCAAGGCATGGTGACGCATGAGATCTACCAGACGCGCGACGGCAATGGCCGCCCGGTCTATCACCTCCCCGAAGAGGTGACCGAGGGCAAATTG---GCAGACGGCACCGAGGTCGAGATCATCCCCTCCGCCAAAATGTCGAAGTCCAAGAAAAACGTCGTCGACCCGCTGCACATCATCTCGAACTACGGCGCCGACACCGCGCGTTGGTTCGTGCTAAGCGATTCGCCCCCCGAGCGGGACGTGGAATGGACCGCCAGCGGCGCCGAGGCGTCTTACAAGCACCTGTCGCGCGTGTGGAACATCTGCGACCGCGTGAGCGAAATGGACCGCGATGCAACCGGCACCGGCGATGATGACCTGCTCCGCGCCATGCACAAGACGATCCATGACGTAACGATGGGCGTGGAATCCTTCGGCTTTAACGCGGCGATTGCCAAGCTCTATGCCTTCACCGCCACTTTGCAGAAATCCAAGGCGGGCTATGCCGCGCAGCGCGAAGCGATCATGACGCTGGCGCAGTTGATGTCGCCGATGACCCCGCACCTGGCCGAAGACATCTGGGCGCATCAAGGCGGCGAAGGGCTGATCACCAAGGCACCATGGCCCAAGGCCGATGAGAAGATGCTGGTCGATGACACTGTGACCCTGCCGATCCAGATCAACGGCAAACGCCGGGCGGAAATTCAGGTGCCCGCCGACATGCCGAAGGAAGAGGTTGAAAAAATCGCGCTGGCGCATGAAGCTGTCATTCGAACGCTGGACGGGGCCACACCGAAAAAGGTCATCGTCGTGCCCGGACGGATTGTGAATGTCGTTGCCTAAATGGCCATTACATCTGCAAACCAGCTTGAGCTGTTGCAAACCGCCGAGGCGGTGGCGCGCGAGAAGATGATCGACCCCGGTCTGGTCGTCGAAGCGATGGAAGAATCCCTCGCCCGTGCCGCCAAGTCCCGCTACGGCGCGGAAATGGACATCCGGGTCGACATCGACCGCAAGACCGGCAAAGCGACATTTACCCGCGTTCGCACCGTGGTCGAGGATGATGAGCTTGAGAATTATCAGGCTGAGTTCACCGTCGATCAGGCCAAGCAGTATATGGCTGACCCCAAGGTCGGCGATACTTACGTCGAAGAAGTGCCCCCGGTTGAGATGGGCCGGATTGCGGCGCAGTCGGCCAAACAGGTGATTTTGCAGAAGGTCCGCGAAGCTGAGCGTGACCGCCAGTTCGAAGAGTTCAAAGACCGCGCCGGTACAATCATCAACGGTCTGGTCAAGCGCGAGGAATACGGCAACGTCATCGTCGATGTGGGCGCTGGCGAAGCGATCCTGCGTCGGAACGAAAAGATCGGCCGCGAGAGCTATCGCCCGAACGACCGTATCCGCGTCTACATCAAAGACGTGCGTCGCGAGCAGCGTGGTCCGCAGATTTTCCTGAGCCGCACCGCGCCTGAGTTCATGGCCGAACTGTTCAAGATGGAAGTGCCGGAAATCTATGACGGCATCATCGAGATCAAGGCGGTCGCCCGCGACCCCGGTTCGCGCGCCAAGATTGCTGTGATTTCGCATGACGGCTCCATCGACCCTGTCGGTGCCTGCGTTGGTATGCGCGGCTCCCGCGTTCAGGCCGTCGTGAACGAGCTTCAGGGCGAGAAGATCGACATCATTCCGTGGAACGAAGACCAGCCGACCTTCCTTGTGAACGCGCTTCAGCCTGCTGAAGTGTCGAAAGTGGTTCTGGACGAAGAAGCCGGCAAGATCGAAGTCGTCGTGCCCGAAGAGCAGTTGTCGCTTGCGATTGGCCGCCGTGGTCAAAACGTGCGTCTGGCCAGCCAGCTGACCGGCCTCGATATTGACATCATGACCGAAGAGCAGGAAAGCCAGCGCCGTCAGGCCGAGTTCGAACTGCGCACCAAGCTGTTCATGGACAACCTCGATCTGGACGAATTCTTTGCCCAGTTGCTGGTTTCCGAAGGTTTCACCAACCTCGAAGAAGTGGCCTACGTCGAAGTCGACGAGTTGCTGGTCATCGACGGTGTCGACGAAGACACCGCTGGCGAGTTGCAGGCCCGTGCCCGTGATGTGCTTGAGGCGCAGAACAAAGCGGCGCTTGATAACGCCCGCGCCTTGGGTGTCGAAGACAGCCTGGTTGAATTTGAGGGTCTGACCCCCCAAATGTTAGAAGCGCTGGCAAAAGACGATGTAAAGACCTTGGAAGATTTCGCCACCTGTGCCGATTGGGAACTGGCTGGCGGCTGGACCACGGTCAACGGCGAGCGTGTTAAGGACGATGGTGCGCTTGAGCCCTTTGAGGTCAGCCTTGAAGACGCCCAAGCTATGATCATGACCGCCCGTGTCATGCTGGGCTGGGTCGACCCGACCGAGCTTGAAGCCGACGCGGACGAAGACGACGTAGAAACAGACGGCGAGAACGCCGAGGAGGCCGAGGCCTAAATGGCACATAAAAAAGCAGGCGGTTCATCCCGTAACGGGCGCGACTCAGCTGGTCGTCGCCTTGGCGTTAAGAAATATGGCGGCGAAGCCGTTATCCCTGGCAACATCATCGTGCGTCAGCGCGGCACCAAGTTTTGGCCAGCCGAAGGCGTCGGCATGGGTAAAGATCACACGATCTTTGCTGTTGTAGACGGTGCTGTGACCTTCCACAAAGGTTTGAAAAACCGCACGTTTATTTCGGTTCTGCCACGCGCGGAGGCCGCTGAATAAATGGCAAAACGGTGGTATTCGGTCAGTGTCCTGTCGAACTTCGAGAAGAAGATCGCCGAGCAGATCCGCGCGTCCGTGGCCGAGCAGGAACTTGAAGACCAGATTGACGAAGTGCTGGTGCCCACCGAAGAGGTGATTGAGGTCCGGCGCGGCAAGAAGGTGACAACCGAGCGTCGCTTCATGCCCGGCTATGTGCTGGTGCATATGGAAATGTCCGACGCGGGCTATCACTTGATCAACTCGATCAACCGCGTCACCGGTTTCTTGGGCCCGCAAGGCCGTCCGATGCCGATGCGCGATGCCGAGGTTCAGGCTATCCTGGGTCGCGTGCAGGAAGGCGAAGAAGCCCCGCGCACGCTGATCCACTTCGAGATCGGCGAAAAGGTTAAGGTTGCCGACGGTCCGTTCGAGGACTTCGACGGCATGATCGAAGAGGTCGACGAGGATAACCAGCGCCTTAAGGTGTCGGTCTCGATCTTTGGTCGGGAAACCCCGGTCGAATTGGAATTCACGCAGGTCAACAAGCAGATCTGAATGTCCTTTACCCTTGCCATCGTGGGCCGCCCGAACGTCGGCAAATCCACGCTGTTCAACCGACTTGTCGGCAAGCGTCTTGCGCTGGTCGACGACCAGCCGGGCGTGACCCGCGACCTGCGCGAAGGTGCCGCCAAACTTGCGGACCTGCGTTTCACCGTGGTCGACACCGCGGGGCTTGAAGAGGTCACCGACGACAGCCTTCAAGGCCGTATGCGTCGCCTGACAGAGCGCGCGGTTGATATGGCCGACATCTGCCTGTTCATGATCGACGCCCGTGTTGGGGTGACCCCATCCGATATGGTTTTTGCTGAGATTCTGCGCAAAAAATCCGCGCATGTGATCCTTGCCGGCAACAAAGCCGAGGGCAAAGCCGCCGATGCCGGAATGATCGAAGCCTATTCGCTGGGTCTGGGAGAGCCGATCCGCATGTCAGCCGAACATGGTGAGGGGTTGAACGACCTTTACACGATGCTGATGCCGCTGGCTGACGAATACGAAGACCGCGCGGTCAAGGACGCACCTGAAACGGATGTGGACCTGCCCGAGGATGACGACGATCTTGAGACCGTCCCGATGCCGACGCGAGCCAAACCTTTGCAGGTCGCCGTGGTGGGCCGCCCCAATGCGGGTAAATCGACGTTGATTAACCAGATTTTGGGCGAAGACCGTCTGCTGACAGGGCCAGAGGCCGGGATCACCCGTGATGCGATTTCGCTTATGACCGATTGGGCAGGGCCGGTGCCGATGCGCATTTTCGACACTGCCGGGATGCGCAAAAAGGCCAAGGTTCAAGAAAAACTTGAAAAGCTGAGTGTCAGCGATGGCCTGCGCGCGGTGAAGTTTGCCGAAGTTGTGGTGGTCCTGCTTGACGCCGAAATCCCGTTTGAGCAGCAAGACCTGCGCATTGCCGATTTGGCCGAGCGTGAGGGTCGTGCGGTCGTTGTTGCCGTGAACAAATGGGACATCGAAGAAAACCGTCAAGAAAAACTCCGCGATCTCAAAGAGAGTTTTGAGCGTCTGCTGCCGCAGTTACGCGGAGCGCCACTGATTACCGTCAGTGCGAAAACGGGCAGGGGGCTTGACCGCCTTCAGGCCGCGATCATGCGTGCCTATGAGACATGGAACCGCCGTGTGACCACTGCGCAACTGAACCGCTGGCTCTCGGGCATGTTGGAGGCGCATCCGCCCCCCGCACCGCAGGGCAAACGGATCAAGTTGCGTTATATGACCCAAGCCAAAACGCGCCCGCCGGGTTTCGTGGTGATGTGCAGCCATCCCGACAAGGTGCCTGACAGCTATTCTCGCTATCTGGTCAATGGGTTGCGGGTGGACTTTGACATGCCCGGCACGCCGATACGCCTGTGGATGCGTGGCCAATCCGATGCGAATCCCTATAAAAACCGCAAGAAGGCGGCCCCATCGAAGCTGCGCAAGCACACGGCCGGGCGGCGTAAAGACTAAATGCGACTGATCTTCATGGGCACGCCCGATTTTTCGGTGCCGATCCTCGATGCGCTGGTGCAGGCTGGGCATGACATCTGCGCGGTCTATTGCCAGCCGCCACGCCCCGCCGGGCGCGGCAAGAAACCCCGCCCCAGCCCGGTACAGCAACGTGCCGAGGAGATTGGTCTGCTGGTGCGCCATCCGGTCTCGCTCAAACACCCCGAACCGCAGGAAGAATTCGCCGAGCTTGATGCAGATGCGGCGGTGGTCGTGGCCTACGGTCTGATCTTGCCGCAGGCCATTCTGGACGCGCCGAAACAGGGCTGCCTGAACATCCACGCAAGCCTGCTGCCGCGCTGGCGCGGGGCCGCGCCCATCCACCGTGCGATCATGGCGGGGGATGAAAAGACCGGCGTTTGCATCATGCAAATGGAAGCGGGGTTGGACACTGGGCCCGTGCTGCTCTGCGAAGAGACCAAGATCGGTGCGGCTGAGACCACGGCCGAACTGCACGACCGGCTGAGCGCCATGGGCGCGGTGGCGATCAACAAGGCGCTGTCGCAATTGTTGCAGCTTACCCCGCAGCCCCAGCCCGAAGAAGGCGTCACCTATGCGGCCAAGATCGACAAGGCCGAGGCCAAGATCGACTGGACCCGCCCTGCGGTTGAGGTGGATCGGCTGATCCGTGGCCTTTCACCCTTTCCCGGGGCGTGGTTCGAGATTGACGGACAAAGGGTCAAAGTGCTGGGATCGGTGCTGGACGAAGGCACAGGTGCTGCGGGTGAGGTCTTGTCGGATGATCTGCGCATTGCCTGCGGCGAGGGTGCCGTGCGACTTACGCGCTTGCAGCGCGCGGGCAAGGGAGCGCAGGACGCGGATGTGTTTCAGCGCGGCGCGCAGATCGCGGTGGGGCGGCGCTTGGGCGAAGGCTGAATGACAGATACATCCGCCTACCGCGTTCTGGCCCGCAAATACCGGCCCGAAACCTTTGCCGATCTTGTCGGCCAAGACGCCATGGTGCGCACGCTCAAGAACGCTTTTGCCGCCGACCGGATCGCGCAGGCCTTTGTGATGACAGGCATTCGCGGCACCGGCAAAACGACGACGGCGCGGATCATCGCCAAGGGGATGAACTGTATCGGGCCAGACGGCAACGGCGGGCCGACGACGGATCCTTGCGGCGTATGCGAACATTGCACCGCGATTATGGAAGGTCGACACGTTGATGTGATGGAGATGGACGCCGCCTCGAACACCGGCGTCGCCAACATCCGCGAGATCATTGATTCGGTACACTACCGCGCTGCGTCGGCCCGCTACAAAGTCTATATTATCGATGAAGTACACATGCTCTCCACCGGTGCGTTCAACGCGCTGCTGAAAACGTTGGAAGAGCCACCCGAACACGTCAAATTCATCTTCGCCACCACCGAGATCCGCAAGGTGCCGGTGACGGTGCTGTCGCGCTGTCAGCGATTTGACCTGCGCCGGATTGAGCCCGAAGTGATGATTGTTCTGCTGCGCAAAATCGCCACAGCCGAGGGGGCCGAGATCACCGACGACGCGCTGGCGCTCATCACCCGCGCTGCCGAAGGCTCGGCCCGTGATGCGACCTCGCTGCTGGATCAAGCGATCAGCCACGGCGCGGGCGAAACCGGCGCCGAACAGGTGCGCGCCATGCTGGGGCTGGCCGACCGGGGCCGGGTGCTGGACCTCTTTGACATGGTGCTGCGTGGTGACGCAGGCGCGGCGCTGACGGAACTCAGCGGCCAATACGCCGATGGCGCTGACCCAATGGCGGTGCTACGCGATCTGGCCGAGATCACCCACTGGGTGTCGGTCGTTAAAATCACACCTGATGCCGCCGAAGACCCTACAATCGCCCCGGAAGAACGCGCCCGTGGTCAGCAAATGGCCGAGACCCTGCCGATGCGCGTGCTGACCCGCCTGTGGCAAATGCTGCTCAAAGCGCTCGACGAAGTCGCCGCCGCCCCCAACGCGATGATGGCCGCCGAAATGGCCGTGATCCGTCTGACCCATGTGGCCGACCTGCCGTCGCCCGAGGAACTGGTGCGCAAGCTGCAAAACAGCACGCCACCCCCGGCCCCCCCCGTCGGTGGTGGCGGTGGCGGCAACGGCGCGCCCCAAGGCGGCGCGCAGGCCGTGCAACACGCCCAGCAGCGCATGGCATCGAACCCCGGTCCACAGGGGCAGACCATCGCCTTGGCGCAAGACCTGAATGCCGCGTTGGCCCGCTTCCCGACATTCGAACATGTGGTTGAGCTGATCCGCGTGAACCGCGATGTGAAACTGCTGGTCGAGGTCGAAACCTGCCTGCAACTGGCCGCCTATCAGCCCGGTCGCATCGAATTCGTCCCCACCGACGACGCCCCGCGCGATCTGGCGCAACGGCTGGGGCAAAAGCTGCAACTCTGGACCGGCAACCGCTGGGCGGTGTCGCTGGTCAACGAGGGCGGCGCCGAAACCATCGCGCAGATCCGAGACGCGCGCGAACTGGCGCTCAAGAAACAAGCCCAAGAACACCCGATGATGCAGGCCGTGTTGGCACAGTTCCCCAAGGCCCGCATCACCGCAATCCGCACCCCCGAAGACATCGCCGCCGCCGCCACGGCAGAGGCGCTGCCCGAGGTCGAAGACGAATGGGATCCGTTCGAGGACGGTTGAATGCCCAAGATGAAGACGAAATCGAGCGCCAAAAAGCGCTTTAAGGTCTCGGCGACTGGTAAGGTCATCGGCAGCCAAGCTGGCAAACAGCACGGCATGATCAAACGGACCAACAAGTTCATCCGTAACGCACGCGGCACGACAGCACTGTCCGAGCCCGATGCAAAGATCATCAAGGGCTTCATGCCCTACGCCCGCTGAATGAACCTATTTGCCGAAATTCGCCATCTCATCATTGCGACACTGGAACAGATGGTCGCGCAGGATGCTCTGCCTGCGACCCTGAACTTTGACCCGATCACCGCCGAGCCGCCGCGTGACCCCGCGCATGGCGACATGGCGACCAACGCCGCGATGGTGCTGGCCAAGCCCGCAGGCATGAAGCCGCGCGACATCGCCGAAGCATTGGCCGCGCAATTGCAAAACGATCCGCGCATCACCTCTGCCGAAGTGGCCGGTCCGGGCTTTATCAACCTCCGCCTTGCGCCTTCGGTCTGGCAGAATGTGGCACGGCAGGTGCTCAGCCAAGGCACCGACTTTGGCCGCGCGATCCTTGGCGCCGGCCAACGGGTGAACGTCGAATATGTCTCGGCCAACCCAACCGGCCCGCTGCACGTGGGCCACACCCGTGGTGCGGTCTTTGGCGACGCGCTGGCAAGCTTGCTGGATTTCGCAGGCTACGACGTGACACGAGAATACTATATCAACGACGGTGGCGCGCAGGTCGATGTGCTCGCACGGTCCGTGTATCTGCGCTACCTCGAAGCCAATGGCAAAGAGGTGGCCTTCCCCGATGGCACATACCCCGGGGACTACCTGATCCCGCTGGGCGAGGCGCTGGCGAAAATGTATGGCGACAAGCTGGTCGACCAGCCTGAAAGCGAGTGGCTCGATCATATCCGTGAGTTCGCGACCGACGCGATGATGAACTTGATCCGTGAGGATCTGGCGGCCCTCGGCGTTGAGATGGACGTCTTCTACAGCGAGAAATCCCTCTACGGCACCGGCCAGATCGAAGCGGCGATTGCTTCGCTCGAAGCCAAAGGGCTAATCTACGAAGGTGTGCTGGAGCCGCCAAAAGGCAAGAAGCCCGAAGATTGGGAGCCGCGCGAACAGACGTTGTTCAAATCCACCGAACACGGCGATGACGTGGACCGCCCCGTCAAGAAATCCGACGGCTCGTGGACCTATTTCGCCCCTGACATCGCCTATCACTACGACAAGGTCAGCCGCGACTTTGACATGCTGATCGACGTCTTCGGCGCGGACCATGGCGGCTATGTCAAACGGATGAAAGCGGCGGTGTCGGCATTGAGCGATGGTCATGTGCCGCTCGACATCAAGCTGACGCAACTGGTGAAACTCTATAAAAACGGCGAGCCGTTTAAGATGTCCAAACGGGCAGGGACTTTTGTGACCCTGCGCGATGTGGTCGATCAGGTTGGGCCGGACGTTACGCGCTTTGTCATGCTGACGCGCAAAAACGACGCGATGCTCGATTTCGACTTCGACAAGGTGCTGGAGCAAAGCCGCGAGAATCCGGTGTTCTATGTGCAATACGCCCATGCGCGGGTGGCATCGATCCTGCGCAAAGCCCAAGAGGCCGGGGTCGAGGTTGCCGATGCCACGCTGATGGATGCGGATCTGACCAAGCTGGATCATGATGCAGAACTTGGCCTGCTGCGCAAACTGGCCGAATGGCCGCGTCTGGTCGAAACCGCCGCGCGCAGCAACGAGCCGCACCGGGTGGCCTTCTACCTCTATGAACTCGCGTCCGATTTCCACAGCCTTTACCACTTGGGCCGTTCCGAAGACGGGCTGCGTGCGTTGCAAGAAGGGGACGCTGCCACATCGCAGGCAAAAATCGCCCTGTCGCGTGCCGTTGCGATTGTAATCGCGGCGGGTCTTGGTATTCTTGGCGTCACACCGGCACAAGAGATGCGATAAATGAGTGCAATCGACAACCTTCCGCCGCTGCGCGAGGTGATCAACACACACGAACTAGCCGCGCGGAAATCGCTGGGCCAGAACTTCCTGCTGGATCTGAACCTCACGGCCAAGCTCGCACGGCAAGCCGGGGATATGGCCGATTGCGACGTGCTTGAAATCGGCCCCGGCCCCGGTGGCCTCACCCGTGGACTGCTGGCCGAAGGCGCACGTCATGTGCTGGCGATCGAGAAGGACCGCCGCTGCCTGCCCGCGCTGGCTGAAGTGGCGGAACACTACCCCGGTCGCCTGACGGTCATCGAAGGCGACGCGCTGGAGATCGACCCGCTGAGCCACCTGACCCCGCCGATCCGCGTGGCTGCCAACCTGCCCTATAACGTCGGGACCGAATTGCTGGTGCGCTGGCTCACCCCGCCCGAGTGGCCCCCCTTCTGGCAGAGCCTGACGCTGATGTTCCAACGCGAAGTGGCCGAGCGGATCGTGGCGACGCCCGGCTCCAAAGCCTACGGGCGGCTGGCCGTGCTGGCACAGTGGCGCAGCGATGCGCGGATCGTCATGCAATTGCCGCCCGGGGCTTTCACGCCGCCGCCAAAGGTCTCGTCGAGCGTGGTGCATCTGACCGCCCTGCCCGAGCCGCGCTATCCGGCTGATCCCGCCGTGCTCAGCCGCGTGGTCGCGATGGCGTTCAATCAACGCCGCAAAATGCTGCGTTCGGCGCTTAAAGGGGCGGCACCCGACATTGAGGATCGCCTGCAGGCCGCTGGGCTGAAACCCACCGACCGAGCCGAGCAAATCCCGCTGGAGGGTTTCTGCGCCCTTGCCCGCGAAATCGCCAAGGGGTGAATGAACCTGATCGCAGAGATCGAGGCGGAACAAGTCGCCGAACTGGGGAAAGAGATCCCCGACTTCCGTGCCGGTGATACCATTCGTGTTGGTTTTAAAGTCACCGAAGGCACCCGCACCCGTGTGCAGAACTACGAAGGCGTCTGCATTTCGCGCAAAAATGGCCACGGCATTGCCGGGTCATTCACTGTGCGCAAAATTTCCTTTGGCGAAGGCGTGGAGCGTGTGTTCCCCCTGCATTCGACCAACATCGACAGCATCACCGTTGTTCGCCGTGGCCGCGTGCGTCGCGCCAAGCTGTACTACCTGCGCAGCCGTCGCGGCAAATCCGCACGTATTGTCGAAAACGCTCACTACAAGCCCCGCGCGAACGCA------TAAATGGCAAATACCAAAAGACAGCTGTTCCTGAAACGCCGCATGCGCGTTCGGAACAAACTCCGCAAAGTGAACGCCGGGCGCATGCGCCTGTCGGTACACCGTTCGTCCAAGAACATCTCGGCTCAGCTGATCGACGATGTGAACGGCGTTACACTCGCATCCGCCTCCACCATGGAGAAGGATCTGGGCGTCGTTGGCAAAAACAACGTCGAAGCGGCAACCAAGGTCGGCGCATTGATCGCCGAGCGTGCCAAGAAAGCTGGCGTGGAAGAAGCATACTTCGACCGTGGCGGTTTCTTGTTCCACGGCAAAGTGAAAGCTTTGGCCGAAGCGGCCCGCGAAGGCGGTCTGAAGATCTGAATGTTTGCGGTCATCAAGACAGGCGGCAAGCAATACAAAGTGCAATCCGGCGATATGCTGCGGGTTGAACGTATTGCGGCCAATGCTGGCGAAACAGTCCAGTTCAACGAAGTTCTGATGCTCGGCGGCGACAGCCCTGTGCTCGGCGCGCCTATGGTTAAAGATGCAGGCGTTCAGGCCGAAGTCGTTGACCAGATCAAAGGCGAGAAGGTTATCAACTTCGTCAAGCGTCGCCGGAAGCACTCTTCCAAGCGTACCAAAGGTCACCGTCAGAAACTGACCCTGATCAAGATCACCGATATCCTCGCCTCTGGTGCAGAGAAGTCGGGCGTTGCTGCTGCCATCGGTACCGGCTCCGTAAGCGCT------------GCCGCTGTTGCCGCG------GCGAAGCCGGCCAAAACCAAGAAGGCCGCCGCACCCAAAGCG---------AAGGCTGAAAAAACCGCGCCTAAGGCCAAGAAGGCCGACGCGGGTTCTGACGACTTGAAAGAGCTGAGCGGCGTTGGCCCGGCACTTGAGAAGAAGCTGCACGAAGCGGGGGTCACATCCTTTGCGCAGATCGCAGCATGGACCGAAGCGGATATCGCTGAAGTTGACGAGAAACTGTCTTTCAAAGGCCGTATCCAGCGTGAAGGCTGGGTCGATCAGGCCAAAGAAAAGACCAAAGGCTAAATGTCCCGAGTAAAAGGTGGTACAGTCACCCACGCGCGTCACAAAAAGATCATCAAAGCCGCAAAAGGCTATTATGGTCGGCGCAAGAACGTCTTTAAGGTCGCCACACAGGCGGTCGACAAGGCCAACCAATATGCAACCCGTGACCGCAAGAACCGCAAGCGCAACTTCCGCGCTCTGTGGATCCAGCGGATCAACGCCGCCGTGCGCAGCCACGACGAAGCGCTGACATACAGCCGCTTCATCAACGGTCTGTCGCTGGCCGGTATCGAAGTGGACCGTAAGGTTCTGGCCGATCTGGCCGTGCATGAGCCCGAAGCCTTTGGCGCGATCGTCAAACAGGCGCAGGACGCACTGGCAGCCTAAATGCAGGTCAACGAGACGCTGAACGAAGGTCTGAAACGCGGTTACAACATCACCGTGACCGCCGCCGAACTGGAAGCCAAGGTCAACGAAAAGCTGGCCGAAGCGCAGCCCGAAGTCGAAATGAAGGGCTTCCGCAAGGGCAAGGTTCCGATGGCACTGCTGAAAAAGCAGTTTGGCCAGCGGATCATGGGCGAAGCGATGCAGGAAAGCATCGATGGCGCCATGGCAGAGCATTTTGAAAAGTCCGGCGATCGCCCCGCGATGCAGCCCGAAGTCAAAATGACCAACGAAGACTGGAAAGAAGGCGATGACGTCGAAGTTTCCATGGCTTACGAAAAACTGCCGGAAATCCCCGAAGTCGATCTGAGCAAGATCGAGCTGGAAAAGATGGTCGTTAAGGCCGATGACGCCGCCGTCGAAGAAGCGCTGGCAAGCCTTGCCGAAACTGCACAAGACTTCAAAGCCCGCAAAGAGGGTGCCAAAGCGGAAGATGGCGATCAGGTTGTGATCGACTTCAAAGGCTCCGTCGACGGTGAAGAATTCGAAGGCGGTGCGGCGGAAGATTACCCGCTGGTGCTGGGCTCCAACTCCTTCATCCCCGGCTTCGAAGAGCAGTTGGTCGGCGTGAAGGCGGGCGAAGAAAAGTCCGTCGTCGTGAACTTCCCCGAAGAGTATCAGGCTGAGCATCTGGCCGGTAAGGAAGCGACATTCGCTTGCACCGTTAAAGAAGTGAAAGAGCCTGTTGCTGCTGAAGTAAACGACGAGATGGCCAAGAAATTCGGCGCCGAGGATCTCGACGCGCTGAAAGGTCAGATCGCTGAGCGTCTGGAAGCGGAATATGCTGGTGCTTCGCGCGCTGTGATGAAGCGTAACCTGCTGGACGCGCTGGACAAAGAGGTCAGCTTTGACCTGCCGCCGTCGCTGGTTGACGCCGAAGCCAAGCAGATCGCGCATCAGCTGTGGCACGAGGAAAACCCCGAAGTTGAGGGCCATGATCACCCCGAGGTTGAGCCGACAGACGAGCACAAGACACTGGCCGAGCGCCGCGTCCGTCTGGGCCTGCTTCTGGCCGAACTGGGTCAAAAGGCCGAGGTTCAGGTGACCGATGCCGAGATGACCCAAGCGATCATGAACCAGGCGCGTCAGTACCCGGGTCAAGAGCGCCAGTTCTTTGAGTTCGTGCAGCAAAACCAGCAGATGCAGCAGCAAATGCGTGCGCCGATCTTTGAAGACAAGGTTGTCGATCACGTTGTTGAGCAAGCCAAAGTGACCGAGAAAGAGATCTCCAAAGACGAGCTTCAAAAAGCTGTTGAGGAACTGGAGGACGAATAAATGAAACTGCACGAACTTTCCGACAACGAAGGCGCAACCAAGAAGCGTATGCGCGTCGGCCGTGGCCCTGGCTCCGGCAAGGGTAAAATGGGTGGCCGTGGTATCAAAGGTCAAAAGTCCCGTTCCGGCGTGGCGATCAAGGGCTTTGAAGGCGGCCAGATGCCGATCTATCAGCGTCTGCCCAAGCGTGGCTTTAACAAGCCCAACCGCAAGTCTTTCGCTGTTGTGAACCTAGGCCTGATCCAGAAGTTCATCGACGCCAAGAAGATCGACGCTGGCAACACCATCGACGGTGCTGCTCTAGTTGCGTCCGGTCTGGTTCGTCGTGAGCTGGACGGTATCCGCGTTCTGGCCAAAGGTGACATCACCTCTAAGATCGACCTGAACGTTGCCGGCGCTTCTCAGTCTGCCATCGAGGCGGTCGAGAAGGCAGGCGGCAAGGTGACTGTGACTGCCCCCAAGGCGGTCGAAGCATCCGAGTAAATGATCCAGATGCAGACCAACCTGGATGTTGCTGACAACAGCGGCGCGCGCCGTGTTCAGTGCATCAAGGTCTTGGGTGGTTCCAAGCGTAAATACGCATCCGTCGGCGACATCATTGTCGTGTCGGTCAAGGAAGCCATCCCTCGCGGTCGTGTGAAAAAGGGCGACGTCCGTAAGGCCGTTGTCGTTCGCACCGCCAAAGAAGTTCGCCGCGACGATGGCACCGCCATCCGTTTCGACCGCAACGCTGCTGTTATCCTGAATAACAACAACGAGCCTGTCGGCACCCGTATCTTCGGGCCGGTTGTTCGTGAGCTGCGCGCGAAGAACTTCATGAAAATCATCTCGCTCGCTCCGGAGGTGCTGTAAATGCGTCACGCACGTGGATACCGCCGCCTGAACCGTACACATGAACACCGCAAGGCGCTGTTCTCGAACATGGCAGGCTCGCTCATCGAGCATGAGCAAATCAAAACAACCTTGCCGAAAGCCAAGGAACTGAAGCCGATCATCGAAAAGATGATCACGCTGGCCAAACGTGGCGATCTGCACGCCCGCCGTCAGGCCGCAAGCAAGCTGAAAGAAGACCAGTATGTCGCGAAACTGTTCGACATCCTCGGCCCGCGCTACAAAGACCGCCAAGGTGGTTACGTTCGCGTGCTGAAAGCTGGCTTCCGCTATGGTGACATGGCGCCTATGGCGATCATCGAATTCGTCGACCGCGACCGCGACGCCAAAGGCGCCGCAGACAAAGCGCGTCTGGCTGAAGAAGAAGCCGCAGAA---TAAATGCTTCAACCAAAGCGTACTAAATTCCGTAAGCAGTTTAAGGGCTCGATCAAGGGTCTGGCAAAGGGCGGGTCTGACCTGAACTTTGGCACCTACGGCCTGAAGGCACTGCAGCCGGAGCGGGTTACAGCCCGTCAAATCGAAGCGGCACGCCGCGCCATGACGCGTCACATGAAGCGTCAAGGCCGTGTCTGGATCCGCATCTTCCCGGACGTTCCGGTCACCTCTAAGCCCGTCGAAGTTCGTATGGGTAAAGGTAAAGGTTCCGTCGACTTCTGGGCAGCCAAGGTCAAGCCGGGCCGCATCATGTTTGAAATCGACGGTGTCGGTGAAGACGTGGCACGCGAAGCCCTGCGTCTGGCAGCCATGAAGCTGCCGATCAAGACCCGGGTTGTCGTCCGCGAGGACTGGTAAATGACCACGATCAAGCTGCACAACACCGCCACCCGGAAGAAAGAGGATTTCACGCCGATTGATGCGCGGAATGTGCGGATGTATGTCTGCGGCCCCACGGTCTATGACCGGGCGCACCTGGGCAACGCGCGGCCCGTGATCGTGTTTGATGTGCTCTTCCGGCTGCTGCGGCACGTCTATGGGCCCGAACACGTTACATACGTGCGCAATTTCACCGATGTAGATGACAAGATCAACGCCCGCGCCGCGACAAGCGGGCGCAGCATTGGCGATATCACAGCCGAGACGACCCAGTGGTATCTCGACGACATGGCCGCCGTCGGTGCGCTGGAGCCCGACCACATGCCTCGCGCGACACAGTATATCCCCCAGATGGTCGCGATGATCCGCGGGCTGATCGACGAGGGCTACGCCTATGAGGCCGAGGGCCATGTGCTGTTCCGGGTGCGCAAATACGCCGAATATGGCGCGCTTTCTGGCCGGTCGGTCGACGACATGATCGCAGGCGCGCGGGTTGAAGTCGCGCCTTACAAAGAAGACCCGATGGATTTCGTACTCTGGAAACCTTCGGATGAGGCCACGCCCGGTTGGGAGTCGCCTTGGGGGCAGGGCCGTCCGGGCTGGCATATCGAATGCTCCGCCATGGCGCATGACCTGCTGGGGGAGCGGTTCGACATCCACGGCGGTGGGATCGACCTGCAGTTCCCGCACCACGAGAATGAGATCGCCCAGTCGAAATGCGCGGGC------CACGGGTTCGCCAACTACTGGCTGCACAATGAGATGTTGCAGGTCGAGGGCAAGAAGATGTCCAAGTCCTTGGGCAATTTCTTTACCGTCCGCGATCTGCTGGACCAAGGCGTGCCGGGGGAGGTCATCCGTTTCGTGATGCTCTCGACCCATTACCGCAAGCCGATGGACTGGACTGAGAAAAAGCGTGAGGAGGCTGAGAAGACCCTTCGCAAATGGTATCTTCAGGCGGCTGAGGCTGAGGCCGCGACGCCT---TCACCGGACCTCGTCACACTCTTGGCGGATGACCTCAACACCCACGGTGCGTTGACCGAATGCCACCGGCTGTCGAATGCGGGCGATACGGCAGGTCTGCGCGGGGCGTTGAAGCTTTTGGGGCTGCTGGATGATCTGATCCCCGAATGGGCCGCGGTGCAGGCGCTGGATCTCAGCGATGTCGAGGCTTTTCTGAGTGATGCCCATGCCACCGCGATGGAGACCAAAGATTTCGCGCAGGTCGACCGGATCAAAACCGCGCTCACCTCTGTCGGGATCGAAGTCCAGATGAGCAAGGACGGCGTGAAACTGACCCCACCGCCCGGCTTTGATCGGGCGCAGTTGGAGGGGTTGTTGTGAATGTCACAGGTCAAATCCAGCTCCAAATCCGATCCGAACTACAAGGTGATCGCCGAAAACCGCCGCGCGCGGTTTGATTACGCTATCGAAGAAGACATCGAATGCGGCATCATCCTTGAAGGGTCCGAGGTCAAATCTCTGCGCGAAGGTGGGGCGAATATCGCCGAGAGTTACGCCGCCGTGGAGGATGGCGAGCTGTGGTTGGTGAATTCCTATGTGGCGCCCTACAAGCAGGCCAAGACCTTTCAGCATGAGGAACGCCGTCGCCGTAAGTTGTTGGTGAGCCGCAAGCAATTGGCGGACCTGTGGAACGCGACGCAGCGCAAGGGCATGACGCTGGTGCCGCTGGTGATGTATTTCAACCATCGCGGCATGGCGAAGATCAAGATCGGCGTGGCCAAGGGTAAGAAGCTGCATGACAAGCGCGAGACGGCGGCCAAGCGCGATTGGTCGCGGCAGAAGCAGCGGTTGTTGAAGGATCACGGCTGAATGAGCGCCAAGCATGAACATTACGATGTGATCCGCAAGCCGATCATCACCGAGAAAGCAACAATGGCGTCCGAACAGAACGCTGTCGTTTTCGAAGTGGCGATCGAGAGCAACAAGCCAATGATCAAAGAGGCCGTTGAGGCGCTCTTTAACGTCAAGGTGAAGGCCGTGAACACGTCCATCACCAAAGGCAAGGTCAAGCGTTTCCGGGGCCAGATGGGCCGCCGTAAAGACGTGAAAAAGGCTTATGTGACGCTCGAAGAAGGCAACACAATCGACGTATCCACCGGGCTGTAAATGAGCAAGGATAAGAATCCCCGCCGCGTGGCAGACAACGAAGCAATGGCAAAACTGCGCATGCTTCGCACCAGCCCGCAGAAACTGAACCTGGTTGCAGCTCTGATCCGTGGCAAGTCCGTGGACAAAGCGTTGACCGACCTCACCTTCTCCAAGAAGCGGGTCGCGCAGGACGTGAAGAAATGTCTTCAGTCCGCGATTGCTAACGCCGAGAACAACCACAACCTGGACGTCGATGAGCTCATCGTGGCCGAGGCCTATGTCGGTAAGAACCTGACCATGAAGCGCGGTCGCCCACGTGCCCGTGGCCGTTTCGGCAAGATCATCAAGCCGTTTGCCGAGATCACGATCAAAGTGCGTCAAGTTGAGGAGCAAGCCTGAATGGTATCTGCCGTCGAAAACATGGCCGCGAACACGAGCTGGGCCGCATTGGGCAAGGCAACTGACCTGCGCAACCGGATCCTGTTCACGCTCGGGCTGCTTATCGTTTACCGCCTTGGCACCTTTATCCCGGTGCCCGGCATCGATGGTGGGGCGCTGCGTGACTTCATGGAAAGCGCGGGCCAAGGCATCGGCGGCATGGTGTCGATGTTTACCGGCGGCGCGCTAGGCCGGATGGGTATCTTCGCTCTGGGCATCATGCCTTATATTTCGGCCTCGATCATCGTTCAGCTGCTGACCTCCATGGTTCCGGCGCTGGAGCAGTTGAAAAAAGAGGGCGAGCAGGGACGCAAGAAGATTAACCAGTACACACGCTACGGCACCGTGGCGCTGGCGACTTTGCAGTCCTATGGTCTCGCGGTCAGTCTTCAGGCGGGTGACATTGTTGCTGACGGGCAGATGGGCTTTGGCTTTGCCTGTATGATCACGCTCGTCGGCGGCACTATGTTCCTGATGTGGCTCGGTGAGCAAATCACCGCACGCGGCATCGGCAACGGTATCTCGCTGATCATCTTCGTCGGCATTATCGCCGAAGTCCCCGCCGCCATAGCCCAGTTCTTTGCTTCGGGCCGCTCTGGCGCGATCAGCCCTGCGGTGATTGTTGGTGTGCTGGTCATGGTGATCGCGACGATCATGTTCGTGGTCTTCATGGAGCGCGCGTTGCGCAAGATCCATATCCAGTACCCCCGTCGCCAAGTCGGCATGAAGATGTATGACGGCGGCACGTCGCACCTGCCGATCAAAGTGAACCCCGCAGGCGTGATCCCGGCAATCTTTGCCTCCTCACTGCTGCTTCTGCCAGTGACGGTCAGCACATTCTCGGGCAACTCCACCAGCCCTGTGATGTCTTGGCTCTTGGCGAACTTTGGCCCCGGCCAGCCGCTTTATTTGCTGTTCTTCGTGGCGATGATCGTCTTCTTTGCCTATTTCTATACGTTCAACGTCAGCTTCAAACCCGATGACGTGGCCGACAACCTGAAGAACCAGAACGGTTTCGTGCCGGGCATCCGTCCGGGCAAACGCACCGCCGAGTATCTTGAGTATGTCGTGAATCGCATACTGGTCTTGGGTTCGTTCTACCTCGCTGCGGTCTGTGTTCTGCCGGAAATTCTGCGCGGTCAGTTCGCTATTCCCTTCTACTTCGGCGGTACATCGGTGCTGATCGTCGTGTCGGTGACGATGGACACCATCCAACAGGTGCAGAGCCATCTGCTCGCGCATCAATACGAAGGTTTGCTTGAACGCTCGCAATTGCGCGGCAAAGGTGCGGGTACACGCAAGAAACGGAGCCCCGTACGTCGATGAATGAAGTTTCTCGATCTGTGCAAAGTCTATATTCGATCCGGTGGAGGCGGTGGCGGATGCGTGTCGTTTCGCCGCGAAAAATATATCGAATATGGTGGTCCGGATGGCGGTGATGGTGGTACGGGCGGTTCTGTCTGGGCCGAAGCGGTCGATGGGCTGAACACGCTGATAGACTTCCGCTATCAGCAGCACTTCTTTGCTAAAAGTGGCCAGCCCGGCATGGGCAAACAGCGGACCGGTAAGGACGGCGACGATATCATCCTGCGGGTGCCCGTGGGCACGGAGATCCTCGACGAGGATCAGGAGACCGTCATCGCCGACCTGACCGAGCTGGGGCAGCGCGTGCAGCTTGCGCGCGGTGGCAATGGCGGCTGGGGTAACTTGCATTTCAAATCCGCGACCAACCAAGCGCCCCGTCGGGCAAACCCCGGTCAAGAAGGCGTCGAACGCACGCTCTGGCTGAGGTTGAAGTTGATCGCTGACGTAGGTCTGTTGGGCTTACCGAATGCGGGTAAATCGACGTTTCTTGCTGCTACTTCCAACGCGCGGCCCAAAATTGCGGATTACCCATTCACCACGCTGCATCCGAACCTTGGGGTTGTGGGAGTGGACAACACGGAATTCGTCGTAGCAGATATTCCCGGCCTTATCGAAGGGGCCTCCGAAGGGCGCGGCTTAGGCGATCTTTTCCTAGGCCATGTGGAGCGTTGCGCGGTGTTGTTGCACCTGATCGACGGTACATCCGAGACTGTTGCCGAAGATTATCATACGATCATCGGCGAGCTGGAGGCCTATGGCGGCGATTTGGCAGAGAAACCGCGGGTCACAGTGCTTAACAAGGTTGATGCATTGGATGAAGATGAGCGTGCAACACGTTTGAAAGAATTGCAAAAAGCCTGCGGTGGCGATGTTATGATGATGTCAGGCGTGGCCGGTGAGGGCGTCATTGAGGTGCTTCGCACCCTGCGCCAGAATATCGACGATGACCGCCTGCGTTTCCGCATCTCCGAGGAAGAAGAGACTTGGCAACCCTAAATGGCTGCTAAACTTCGCAAAGGTGACAAGGTCATCGTCCTGTCCGGCAAGGACAAGGGCAAGACGGGCACCATCTCGTCTGTTGACCCCAAGTCGAACAAGGCGATCGTTGACGGCGTGAAAATCGCCATCCGCGCCACACGCCAGACACAGACATCTCAAGGCGGCCGCATCCCCAAGGCGATGCCGATCGACCTGAGCAACCTCGCACTGGTGGATGCCAATGGCAAAGCCACCCGCGTGGGTTTCAAAATCGAAGGCGACAAAAAAGTGCGCTTTGCCAAGACCACGGGGGACGTGATCGATGCTTGAATGTTGCGCTCAGGCGTTATTGCAAAAAAAGTCGGCATGACCCGGCTGTTCATGGAAGACGGCAAGCAGATTCCTGTGACCGTTCTCCAGTTGGACAAGCTTCAGGTTGTCGCACAGCGCACCGCAGACCGTGACGGCTACACCGCCGTTCAGCTGGGTGCCGGTTCGGCGAAAGCCAAACGCACAAGCCAAGCCATGCGTGGCCATTTCGCAGCAGCGAAAGTGGAACCCAAGCGCAAGGTTGCTGAATTCCGCGTCGATGCGGAAGCGATGCTGGAAGTCGGTGAGGAAATCATCGCGGACCATTACTTCGCAGGTCAGTACGTTGACGTTGCAGGCACTTCGATCGGTAAAGGTTTTGCCGGTGCGATGAAGCGTCACAACTTTGGCGGTCTGCGGGCGACACACGGTGTTTCCATCAGCCACCGTTCGCACGGTTCCACAGGCCAGTGTCAGGATCCCGGCAAGGTTTTCAAAGGTAAGAAAATGGCCGGTCACATGGGCGCTGCCCGTGTCACCACGCAGAACCTCGAGGTCGTCAAGACTGACAGCGCGCGTGGCCTGATCATGGTTAAAGGTGCCGTTCCTGGCTCCAAAGGTGGCTGGGTCACCGTCAAGGATGCGGTCAAAAAGCCGTTCCCCGAAGACGCGATTCTGCCCGCCGCTCTGAAATCCGCCGCTGAAGAAGCCGCGAAAGCCGCCGAAGAGGCAGCCGCCGCAGCCGCAGCAGAGGCAGAAGCTGAAGCCAAGCGTTTGGCCGAAGAGCAAGCCGCACAGGAAGCCGAAGCGCTGAAAGCCGCAGAAGCTGAAATCGCAGCTGAGGGTTCGGATGCCGACAATTCCGATGCCGACGACAAGAAAGAAGGTGACGCATGAATGGCACTCAAGTCGTACAAACCGACGACGCCAGGCCAGCGTGGACTGGTACTGATCGACCGTTCGGAGCTGTGGAAAGGCCGCCCGGTCAAAGCCCTTACTGAGGGTTTGACCAAATCTGGCGGTCGGAACAACACCGGACGAATCACAATGCGTCGTACAGGTGGTGGTGCAAAGCGCCTCTACCGTATCGTTGATTTCAAGCGTAACAAGCTGGACATGTCCGCTGTTGTCGCGCGGATCGAATATGACCCCAACCGGACCGCTTTCATCGCACTGATCCAATACGAAGACGGCGAGCAGGCCTACATCCTGGCCCCCCAGCGTCTGGCCATCGGCGACAAGATCATCGCCGGCGCCAAAGTGGACATCAAACCCGGTAACGCGATGCCTTTCTCGGGCATGCCAATCGGTACGATCGTCCACAACATCGAGATGAAGCCCGGTAAGGGCGGTCAGATCGCACGTGCCGCCGGCACCTACGCCCAGTTCGTGGGTCGTGACGGTGGCTACGCTCAGATCCGTCTGAGCAGCGGCGAGCTGCGTCTCGTGCGTCAGGAATGCATGGCCACCGTTGGTGCTGTGTCTAACCCCGACAACTCCAACCAGAACTACGGTAAAGCGGGCCGCATGCGTCACAAGGGCATCCGTCCTTCTGTACGTGGTGTGGTGATGAACCCGATCGACCACCCGCACGGCGGTGGTGAAGGCCGGACCTCTGGTGGTCGTCACCCGGTTACTCCTTGGGGTAAGCCGACGAAGGGTGCCAAGACCCGCAACAAGAACAAAGCGTCCAGCAAGCTTATCATCCGCTCGCGTCACGCCAAGAAGAAGGGGCGTTAAATGCTTGATACCGCAACCTACACACCCCGCCTTCAGGCCGAATACCGCGAGAAGATCCGCGCCGCCCTGAAAGAGGAATTCGGCTACAAGAACGACATGATGATCCCCAAGCTGGACAAAATCGTTCTGAACATCGGCTGTGGTGCCGAGGCCGTACGTGACAGCAAGAAAGCCAAGTCGGCTCAGGAAGACTTGACCGTGATTGCAGGCCAGAAGGCCCTGACAACCGTGGCCAAGAAATCCATCGCTGGTTTCCGCGTCCGTGAGGAAATGCCGCTGGGTGCGAAAGTAACCCTGCGCGGTGACCGCATGTACGAATTCCTTGACCGTCTGATCACGATCGCAATGCCCCGTATCCGCGACTTCCGCGGCGTGCCAGGCAAAAGCTTTGACGGCCGTGGCAACTACGCCATGGGCTTGAAAGAGCATATCGTGTTCCCCGAAATCGACTTCGACAAAGTTGACGAGACTTGGGGTATGGACATCGTGATCGCCACCACGGCGAAAACCGACGCTGAAGCCAAGGCGCTGTTGAAAGCTTTCAACATGCCCTTCAATTCATAAATGAAACTCGATGTCATCAAACTCGACGGTGGCAAGGCCGGGTCCGTAGACCTGGACGAAGCACTGTTCGGCCTTGAGCCGCGTGCCGACATCCTGCACCGCGTCGTGCGCTGGCAGCGTAACAACGCGCAGCAGGGTACGCACAAGGTCAAGACGCGGTCCGAGACCAGTTACTCGACCAAGAAGATCTATCGCCAAAAAGGCACCGGCGGCGCACGCCACGGTGACCGTAACGCGCCGATCTTCCGTAAGGGTGGTATCTACAAGGGTCCAACCCCGCGTAGCCACGGCCACGAGCTGACCAAGAAGTTCCGCAAGCTGGGTCTGCGCCACGCGCTCAGCGCCAAAGCAAAAGCCGGTTCCTTGGTCATCATCGACGAAGCAACGTCCGAAGGCAAAACAGCCGCTCTGGCCAAGCAGGTTTCGAACCTGGGTTGGAAGCGCACGCTGGTCATCGATGGCGCTTCGGTCAACGAGAACTTCGCACAGGCCGCACGCAACATCGAAGGTTTGGATATCCTGCCGTCGATGGGCGCAAACGTCTATGACATCCTCAAGCGTGACACTCTGGTGATCACCAAAGCGGGGATCGAAGCATTGGAGGCACGCCTGAAATGAATGGAAAATGTCGTTCTGATCATTCACCTGATTCTGGCCCTTGGCCTGATTGCTGTGGTGCTGCTGCAACGGTCTGAGGGCGGTGGCCTTGGCATC---GGTGGTGGCGGCGGCGCTGTTTCTGGCCGCTCGGCTGCTACCGCTTTGGGCAAGATTACCTGGATCCTCGCCGCTGGTTTCATTGCAACATCGATCACGTTGACCATCATCGCGGCTGAGAAATCGGCGGGCTCGTCGGTTATTGACCGGTTGAGCGCGACACCGCCCGCACAGAACCAAGATGGCAGCCCTGCCCTGCCCGCAGGCGATGATCTGCTGCCGCCTTCTGCGGATGGCAACACACCGCTGGTCCCTGCGGCCGATTAAATGGCCTTCTTCACGAAGCTCAAGGACCGCTTGTTCAAATCCTCGTCCAAGATCGACGAGGGGCTAGAGGCGATTGTCAGCGATGGCGGCGAAACAGAGGCC---------GAAGCGGCCCCCGTGGATGCGGACCAGCCCGGCGCGGCCTCTGATGTGATGCCCGACGGGCAGACCCCCGAACCCGCGCCGATGCCAGAA------CCGACG------CCCGCGCCGATGCCAGAGCCGACG---GCGCCCGCCACCATTCCCGAGCCCACCCCTCAGCCTGCAGATCCGGAGCCCGCGCCCGAACCCGAGCAGGTGCCGCCCGAACCCGCTCCCGATGAAGTGCCGCCCCTGCGCCAAGCCATGACCCCGGTCGCCCCTGCGCTTGACGAA------GCA---GAGGCCGCTCCTGCGAAGCCAGGCCTTCTGGGCCGTTTGATGGGCCGCAGCACGGCGCAGCCTGTGGTGCGCCGCGCGCTGGACGATGAGATGCTCGAACAGCTCGAAGAACTGCTGATCAGTGCTGACATGGGCGTCGACACCGCGCTGCGGGTCACGGCCAATATGGCCGAAGGGCGCTTTGGCAAGAAACTCTCGGTGGCGGAGATCAAACAACTGCTCGCCAGCGAAGTCAGCCGCATCATGGAGCCTGTGGCGCGGCCCTTGCCGATCTATTCCAAGACCCCGCAGGTGGTGCTGGTGGTCGGCGTGAACGGCTCTGGCAAGACGACAACCATCGGCAAACTGGCATCACAATTCCGCGCGGCGGGCAAGAAGGTCGTAATCGCCGCTGGCGACACCTTCCGCGCTGCTGCGGTGGAGCAATTGCAGGTCTGGGGCGAACGCGCAGGCGTGCCGGTTTTGACCGCAGCGCAGGGCAGCGACCCGGCCAGCCTTGCCTTCGACGCCATGGGCCGCGCGCAGGAGGAAGGCGCTGATCTGTTGCTGATCGACACCGCAGGCCGTTTGCAAAACCGGGGCGACCTGATGGAGGAATTGGCCAAGATCGTGCGCGTCATCCGTAAGAAGGACGAGACCGCGCCGCATAACACCCTATTGGTGCTGGACGCGACGACCGGGCAAAACGCACTGAACCAAGTGAAGGTTTTTCAAGAGATTTCCGACGTCAGCGGTCTGGTTATGACCAAGTTGGATGGCACCGCCAAGGGGGGCGTTCTGGTGGCGCTGGCGGATCGTTTCGGCCTGCCGATCCATGCCGTGGGCCTGGGGGAGCAGATTGATGATCTGTCGCCCTTCGACCCGGATGAATTTGCCGCTGCTTTGGTGGGCACGGATGCCTAAATGACATTAGATCACTGGGGCGAGATCAAAGAGCGGCTTCTGAAGACCGTTGGGCAAAACAACTACACCACTTGGATTGAGCCGCTTGTGCCCGGCGATGTCGAAGATGGAATCGTGACACTCAAGGTGCCCACGAACTTCTTTGGCAATTACGTCAGCCAGAACTTCTCGGACCTGATCTTGCATGAAATTAACGCCGCTGGCACCGATGCCACGCGCCTGAATTTTGCGCTGAACCAGCAGCCCGCCAATGCCGCCGACAAGCCCGCCCCAGCTGCGCGGCAGACCACCGCCGCTGCAAAGCCC------GCCGCCAATAGCCAGCTGAGCACTGCCCCGCTCGACCCGCGCTTTAGCTTTGACAACTTTGTTGTTGGCAAACCAAACGAACTGGCCCATGCCGCCGCGCGCCGTGTTGCCGAAGGTGGCCCGGTCACGTTCAACCCGCTGTTCCTCTATGGCGGTGTCGGTCTGGGTAAGACCCACCTGATGCACGCGATCGCCCGCGAGCTGCATGAGCGCAAGCCCGAGATGAACGTGCTTTATCTTTCGGCCGAGCAATTCATGTACCGTTTTGTGCAGGCGCTGCGCGACCGCAAGATGATGGACTTCAAAGAGATCTTCCGTTCGGTCGACGTGCTGATGGTCGATGACGTGCAGTTCATCGCGGGCAAGGATTCAACGCAGGAAGAGTTCTTCCACACGTTCAATGCGCTCGTGGACCAGCACAAACAGATCATTATCTCTGCCGACCGCGCACCGGGTGAGATCAAAGACCTCGAAGATCGGGTGAAATCACGCCTGCAGTGCGGCTTGATCGTGGACCTGCACCCGACGGACTATGAGCTTCGCCTCGGCATTCTGCAAAGCAAGGTCGAAGTGCAGCGCAAGACATATCCGGACCTCGAAGTTGCCGATGGCGTTTTGGAATTCCTCGCGCATCGCATTACCTCGAACGTCCGCGTGCTCGAAGGTGCGCTGACCCGTCTGTTCGCTTTCGCCTCGCTCGTGGGCCGTGAGATCGACATGGGCCTGACCCAAGATTGTCTGGCCGACGTGCTGCGCGCCTCAGAGCGCAAAATTTCGGTCGAGGAAATCCAGCGGAAAGTGTCGGATCACTACAACATCCGTCTGAGCGATATGATCGGCCCCAAGCGTCTGCGCAGCTATGCCCGCCCACGTCAGGTGGCGATGTATCTGTGCAAGCAGATGACCAGCCGCTCCTTGCCAGAGATTGGCCGCCGCTTTGGCGGGCGTGATCATACCACCGTCATGCATGGTGTGCGCCGGATCGAAGAACTGAAGGTTTCCGACGGTCAGATCGCCGAGGATCTGGAACTGCTGCGCCGGGCGCTCGAATCCTGAATGCTGGGTATCGGAACAATCGCCAAAAAGGTCTTTGGCACGCCAAACGACCGGAAGATCAAGGCGACCCGTCCGCTTGTGGCACGGATCAATGCGCTGGAGCCTGAGTTTGAAAAGCTGAGCGACGAAGAGATCAAGGCGCGCACGGAAGAACTAGCCAAACGCGCAAATGCGGGTGAAAGCCTTGACGATCTGCTGCCTGAGGCTTTTGCCAACTGCCGGGAAGCGGCCCGCCGCACCCTTGGCTTGCGGGCTTTCGACACGCAGCTTTTGGGCGGGATTTTCCTGCATCAGGGCAATATCGCCGAACAAAAGACAGGTGAAGGCAAGACCCTGACTGCGACTTTCGCAGCCTACCTCAACGGGCTCACGCATAAAGGTGTGCATGTGGTAACGGTGAACGAATACCTTGCCAAACGTGACGCCGAATGGATGAGCAAAGTATTTGGCGCCTTGGGACTGACCACCGGGGTCGCTTATTCCGGCATGCCCGAAGACCAGAAGCGCGCGGCCTATGCCTGCGACATCACCTATGCCACCAACAACGAGTTGGGTTTCGACTACCTGCGCGACAACATGAAATCGAACCTGTCGGACATGCTGCAACACGGGCATAACTTTGCCATCGTGGACGAAGTCGACAGCATTCTGATCGACGAAGCGCGGACGCCGCTGATCATCTCCGGCCCCTCGCAGGATCGTTCTGAGATGTATCAGATCATCGACACGCTGATCCCCTCGTTGACCGAAGAGCATTACGAGCTCGACGAGAAAACCCGCAACGTGACCTTCACCGATGAGGGCAATGAGTTCCTCGAAGAGCAGTTGCGCGCCCGTGATCTGATCGAAGAGGGGATGACCCTTTATGATCCCGAAAGCACGACCATCGTGCACCACGTCAACCAAGGTCTGCGTGCGCATAAACTGTTCCAGCGCGACAAAGACTACATCGTCCGCGATGGCGCTGTGACGTTGATCGACGAATTCACCGGTCGCATGATGCCGGGCCGCCGCCTGTCGGATGGCCTGCACCAAGCGATTGAAGCCAAAGAGGGCGTCGACATCCAGCCCGAGAACGTGACCCTCGCAAGCGTTACTTTCCAGAACTACTTCCGTCTTTACGACAAGCTGGGCGGCATGACCGGCACGGCCCTGACCGAAGCCGAGGAATTCGCCGAGATCTACGGTCTTGGCGTTGTCGAAGTGCCGACCAACGTGCCGATTGCCCGTGTCGACGAAGACGACGCCGTTTACCGCACCGCGCGCGAGAAATACGAGGCGATGATTGAAAAGGTGAAGGAGGCCCACGCCAAGGGTCAGCCCTGCCTTGTCGGCACCACCTCGATCGAAAAGTCCGAACAGCTGAGCGCTATGCTGACGGCCGATGGGATCGCGCATAATGTGCTTAACGCCCGCCAGCACGAGCAAGAAGCACAGATCATCGCCGATGCTGGTAAGCTGGGTGCCGTGACCATCGCCACCAACATGGCGGGTCGCGGCACCGACATCCAGCTGGGCGGCAACGTTGAGTTGAAAGTGTTGGATGCACTGGACGCTGACCCCGAAGCTGACCCGGCGAACATCCGTGCCCAGATCGAGGCGCAGCACGCCGAGGAAAAACAGAAGGTGCTAGAGGCCGGTGGTCTCTACGTTCTGGCGTCTGAGCGTCACGAGAGCCGCCGCATCGACAACCAGCTACGCGGCCGTTCGGGCCGTCAGGGCGATCCGGGCCGCACCTCTTTCTTCCTGTCGCTTGAAGATGATCTGATGCGCATCTTCGGCTCGGAACGGCTCGAGAAAGTGCTGACCACGCTCGGCCTCAAAGAAGGCGAAGCGATTGTGCACCCTTGGGTAAACAAGTCGCTTGAGCGCGCGCAGGCGAAGGTCGAAGGTCGCAACTTTGACATCCGTAAGCAGTTGTTGAAGTTCGACGATGTGATGAACGAGCAGCGCAAAGTCATCTTTGGCCAGCGCCGCGAGATCATGGAGGCGGAGAACCTTAATGAGATCGTCACAGACATGCGCGAGCAGGTGATCGACGATTTGATCGACACCTATATGCCGCCCAAGACCTACGCCGATCAGTGGGACACCCAAGGTTTCTATGCCGCGGTGATCGAACAGCTGAACGTCGACGTGCCGATCATCGCATGGTGTGAAGAAGATGGCGTGGATGACGAAGTGATCCGCGAGCGTCTGATGAAAGCCACCGATGAGCTGATGGCCAAAAAGGCCGAGGCCTTTGGCGAAGAGAACATGCGCAACATCGAGAAACAGTTGCTGTTGCAGGCCATCGACACCAAATGGCGCGACCACCTGCTGACGCTGGAGCATCTGCGCTCGGTCGTGGGCTTTCGCAGCTACGCGCAGCGCGATCCGCTGAACGAGTACAAGAACGAAGCATTCCAACTGTTTGAGACGATGCTCGACAGTCTGCGGCAGGACGTGACCCAGAAGCTGGGCCAAATCCAACCGATGAGCGAAGAGCAGCGCCGCGAGATGATGCAGGAAATGGCTGACCGTCAGGCCGCGATGCAAGCCGCCGCGACCGAGGCTGCCGATGAGGCA------TCTGAGCAGGCAGAGGCT---GCCGCACCGGGCTTTGATGAGAATGATCCGTCCACATGGGGCAACCCAGGCCGGAACGATCTCTGCCCCTGTGGCTCGGGCAAAAAGTTCAAACATTGCCACGGCGAAATCAAATAGATGAAGCTAAGCGGAGACCTCAAAGCCTTCGAGGCGCGGATCGGCCATCACTTCGGCAAGCCGGAACTGCTGGTTCGCGCGGTGACCCATGCCTCCATGTCCTCGGCCAACCGCGACGACAACCAGCGGCTGGAGTTTCTGGGCGACCGGGTGCTGGGCCTTGTCATGGCCGAGGCGCTGCTGGCGCTGGATCCCGGCGCGACCGAGGGGCAGTTGGCCCCGCGGTTCAACGCATTGGTGCGCAAGGAAACCTGCGCCGATGTCGCGCGTGAGATTGATCTAGGCAAGGTGCTGAAATTGGGCCGGTCTGAGATGATTTCGGGCGGGCGGCGCAAGCAGGCGCTGTTGGGCGACGCTATCGAGGCGGTGATTGCCGCCGTTTATCTGGATGGCGGGTTTGACGCGGCCAAAGACCTCGTGTTGCGGCTGTGGGGCAACCGGCTGAAGACGGTTAAAGAAGACGCCCGCGACGCCAAAACCGCGTTGCAGGAATGGGCACAAGCACGCGGCCTGACCCCGCCGCGCTATGTCCAGACCGACCGCAGCGGCCCCGACCATGCGCCGGTCTTTACCATTACCGCGCGGCTGGACAACGGCGCCGAAGCCGCCGCCACCGCGCCCTCAAAACGTGCCGCCGAACAGGCCGCCGCCACCACGCTGTTGCGGCAATTGGAGAAGAACTCATGAATGGCAAAACTTGGAAAACGCACCCGCGCCGCCCGTGAAGCCTTTGCTGGCAAAGAAGAGATCACCGTTGAAGAAGCGGTAAGCCTCATCAAAGCAAACGCAAACGCAAAGTTCGACGAAACCATCGAGATCGCGATGAACCTCGGTGTTGACCCGCGCCACGCAGACCAAATGGTCCGCGGTGTTGTCGGCCTGCCCAACGGCACCGGCAAAACAATGCGCGTTGCTGTCTTCGCCCGTGGCGCGAAAGCTGAAGAAGCCGAAAAGGCTGGCGCAGATATCGTTGGTGCAGAAGACCTGATGGAAACCGTGCAGTCCGGCAAGATCGACTTTGATCGTTGCATCGCGACACCCGACATGATGCCGATCGTTGGCCGTCTGGGTAAAGTGCTTGGCCCCCGTAACCTGATGCCGAACCCCAAGGTTGGCACCGTGACCATGGACGTCGCCGACGCGGTGAAAGCGGCCAAGGGTGGTGAAGTTCAGTTCAAAGCCGAAAAGGGCGGTGTCGTCCATGCAGGCGTTGGCAAACTGTCCTTCGACGAAGCCAAGCTGGCCGAGAACATCCGTGCCTTTGTTGGCGCGGTGTCCAAAGCCAAGCCTGCCGGTGCCAAAGGCACATACATGAAAAAGATCAACCTGAGCTCCACAATGGGCCCGGGCGTGTCGGTCGCTGTCGAGAACGCAACCGCCGAGTGAATGTCAGACGAATTTATGCTCGATACCGATGATCTTGAACGGCGGATGAACGGCGCAATTGCGTCGCTTCGGACCGAGTTCGCATCGCTGCGCACGGGGCGTGGCTCGGCCTCCATGCTGGAGCCGGTGATGGTTGAGGCCTACGGCCAGATGACCCCGGTGAACCAAGTGGGCACCGTCAACGTGCCAGAGCCGCGCATGGTCACGATCAACGTCTGGGACAAGGGTCTTGTTGGCAAGGTCGAGAAAGCCATTCGTGAAAGCGGTCTGGGCATCAACCCGCAGCTCAATGGCACCATCATCATGCTGCCGATCCCTGAGCTGAACGAAGAGCGCCGCACCCAGTTGACCAAGGTCGCGGGCCAATATGCCGAACACGCCCGCGTCTCGATCCGCAACATCCGTCGCGACGGGATGGACCAGATCAAGAAGGCCAAGAACGACGGCATGTCCGAAGATGACCAGAAAATCTGGGAAGGCGAAGTGCAAGACCTGACCAACCGCTTCATCACCCTCATCGACGACCAGCTTGAGACGAAACAGGCCGAAATCATGCAAGTTTGAATGGCCAAGAAACTCGTCGGTACGATGAAGTTGCAAGTTAAAGCGGGTCAAGCAAACCCGTCCCCGCCCGTGGGCCCAGCCCTCGGTCAGCGCGGCATTAACATCATGGAATTCTGCAAGGCGTTCAACGCCAAGACCGCAGACATGGAGCCGGGCGCACCGTGCCCCACCGTGATCAGCTACTATCAGGACAAGTCCTTCACCATGGACATCAAGACGCCCCCGGCGTCCTACTACCTGAAAAAAGCTGCCAAGGTGAACTCTGGCGCGAAAACGCCGAGCCGTGAAACCGTTGGCACCGTGACCACCAAGCAGCTGCGCGAGATCGCAGAAGCAAAAGCCGCCGACCTGTCCGCGAACGACGTGGAAGCGGCGATGAAAATCATTCTGGGTTCTGCCCGGTCCATGGGCATCGAGGTGAAGTAAATGTTTGAAAATCTCAGCGAACGGCTCTCTGGTGTCTTTGACCGCCTGACCAAACAAGGCGCGCTCAGCGACGAAGATGTCAAAACCGCCCTGCGTGAAGTGCGCGTCGCCCTGCTGGAGGCTGACGTTTCGCTGCCCGTGGCGCGCGATTTCGTCAAAGCGGTACAGGACAAGGCGACCGGCCAAGCGGTGACCAAATCGGTCACGCCCGGCCAGCAGGTTGTGAAGATCGTCCATGACGCGCTGATCGACACGCTGAAAGGCGAAGGCGAACCCGGGGCGCTCAAGATCGACAGCCCGCCCGCCCCGATCCTGATGGTCGGCCTACAGGGCGGTGGTAAGACGACCACCACCGCGAAACTGGCCAAGCGTTTGAAAGAGCGTGACGGCAAGCGCGTGCTGATGGCCTCGCTCGACGTGAACCGCCCGGCGGCGATGGAGCAACTTGCCATCCTCGGCACCCAGATCGGCGTCGACACGCTACCGATCGTCAAGGGCGAAAGCCCCGTCCAGATTGCCAAGCGCGCCAAGACGCAGGCGGGTTTGGGCGGCTATGACGTCTATATGCTCGACACTGCGGGCCGTCTGTCTATCGACGAAGAGTTGATGCAGCAGGTCAAAGCCGTGCGCGACGTGGCCAATCCACGTGAAACATTGCTCGTCGTCGACGGCTTGACCGGCCAAGACGCCGTGCACACCGCCGAGAACTTTGACGAGCGTATCGGCATCACCGGTGTCGTGCTGACCCGGATGGACGGCGATGGCCGTGGCGGTGCGGCTCTCTCGATGCGTGCTGTGACCGGCAAGCCGATCAAGTTCGTCGGTCTTGGCGAAAAGATGGACGCACTTGAGACTTTCGAGCCTGAGCGCATCGCGGGCCGTATCCTCGGCATGGGCGATATCGTCGCGCTGGTCGAAAAGGCCCAAGAAACCATCGAGGCCGAACAGGCCGAGAAGATGATGAAGCGCATGGCCAAGGGTCAGTTCAACATGAACGACCTGAAGATGCAGCTTGAGCAGATGATCAAGATGGGCGGCATGCAGGGCATGATGGGTATGATGCCCGGCATGGGGAAAATGGCGAAACAGGTCGAAGACGCCGGTTTCGACGACAAGATCCTCAAGCAGCAGATCGCCCTGATCCAGTCCATGACCAAGAAAGAGCGCGCCAACCCTGCCCTGCTTCAGGCCAGCCGCAAAAAGCGTATCGCCAAGGGTGCCGGCATGGAGGTCTCCGACCTCAACAAGCTGATGAAGATGCACCGCCAGATGTCCGACATGATGAAGAAGATGGGCAAAATGGGCAAAGGCGGCATGCTCAAGCAGGCCATGAAAGGCATGATGGGCAAGGGCGGCATGGACCCCTCC------------CAGATGGACCCCAAGGCGCTCGAAGCGGCGGCCAAGCAGATGGGCGGCAAGCTGCCCGGCGGTCTGGGCGGCATGGGCGGCGGCATGGGCCTGCCCGGCGGACTCAGCGGTTTCGGGAAAAAGAAATGAATGATCCCGATGCACCGCCTCGCGCAAATCTCGCAACGCTTCCAGTTTCTGGAGGCCAGCATGTCTGCGGGCTCCGATGGGGCCGATTTTTCAGCCCTCGCCAAGGAATACAGCGATCTCAAGCCCGTGGTGGATCAAATTGATCTCTACCAGCAGCTTCAGCGCGACCTTGAAGAAGCCGAGTTGATGCTGAAAGACCCCGAGATGGCAGAGCTTGCCCGCGAAGAACTGCCTCGTCTAAAGGCGCGTCTGCCGGAGGTCGAACAGGGATTGCAGCTAGCGCTTCTGCCGCGCGATTCCGCCGATGCGAAGCCCGCGATGCTGGAGATCCGCCCCGGCACGGGCGGAGATGAGGCGGCGCTGTTCGCGGGCGACCTGCTGCGCATGTACCAACGCTACGCCGAAGCGCGGGGCTGGGGGTTCGACCTGATTGAAAGCCAGATGACCGAGTTGGGCGGCGTCAAGGAAGTCGTGGCCCATATCACCGGGCAGAACGTTTTTGCCCGGCTGAAGTTCGAAAGCGGCGTGCACCGTGTCCAGCGCGTGCCCTCGACCGAAAGCGGCGGGCGCATTCATACCTCAGCGGCAACCGTGGCGGTGCTGCCCGAGGCTGAAGACGTGGATATCGAGATCAACGCCAATGACCTGCGGATCGATACAATGCGCTCCTCGGGTGCGGGCGGCCAGCACGTTAATACCACCGATTCTGCCGTGCGCATCACCCATATTCCGACCGGGATTGTGGTTACATCGTCGGAAAAATCGCAGCACCGAAACCGCGACAAGGCGATGCAGGTGCTCAAAGCGCGGCTCTATGACATGGAGCGCAGCCGGATCGACAGCGCACGTTCCGCCGACCGCGCGGCGCAGGTGGGCACGGGCGACCGGTCCGAGCGAATCCGAACCTATAACTTCCCCCAAGGGCGGATGACCGACCACCGGATCAACCTGACGCTCTACCGTCTTGAGGCCGTGATGCAGGGCGATCTGGATGAGATCGTCGATGCCCTGACCGCCGACGCCCAAGCACGTCAATTGTCGGAGATGGAGGGGTGAGTGGATAGAGCACAAAAAGAACAGCTGGTCGACGAACTCGGCCAGATCTTTGAAAGCTCTGGCGTCGTTGTGGTTAGCCACTACGTCGGTTTGACAGTTGCCGAAATGCAGGACCTTCGGGCGCGCGCACGCGCTGCCGGTGGGGCCGTGCGTGTTGCCAAAAACAGGCTCGCCAAGATCGCCCTTGAGGGCAAGCCATGCGAAAGCATCGCTGACCTTCTGACGGGTATGACCGTTCTGACCTATTCTGAGGATCCTGTGGCTGCGGCCAAGGTTGCTCAGGAATTCTCCAAGGAAAACCCAAAGCTGGTGATCCTCGGTGGTTCCATGGGTGAGAACGCGTTGGACGCCGCTGGTGTCGAAGCCGTGTCGAAAATGCCTTCGCGGGAGGAGCTTATCTCCACCATCGCGGGCATGCTCGGCGCCCCTGCTTCGAACATCGCCGGGGCCATTGGCGCACCTGCAAGCAACATCGCATCCATCTTGTCCACGATCGAGGACAAGGCGGCTGCGTAAATGAAAACCTTTTCTGCGACACCGGCAGATATCGACAAGAAATGGATCATCATCGACGCCGAAGGCGTCGTGCTGGGCCGTCTCGCCTCGATCATCGCCATGCGCCTGCGCGGCAAACACAAGCCGTCCTTCACGCCGCATATGGATTGCGGCGACAACGTGATCGTCATCAACGCCGAGAAGGTGCAGATGACCGGCAAGAAGCGCGAAGAGCACTTCTACTGGCACACCGGCCACCCCGGCGGGATCAAATCGCGCACTAAGGAGCAAATCCTTGAAGGCGCGCATCCTGAGCGTGTTGTGACCCAAGCGGTCAAGCGCATGCTGCCCGGCAACCGCCTGAGCCGCCAGATCATGACCAACCTGCGCGTCTATGCAGGTAGCGATCACCCCCATGAGGCCCAGAGCCCCGAAGTTCTGGACGTTAAGTCCATGAACAAGAAAAACACGCGGAGTGCATGAATGACTGCCGCTGATGCAGCATTGGCCGAGGGGCTGCTGGCAGGGCTGGGCGGCGCGCTGCCGCCTCGCCTTGGCGTTGCCGTTTCGGGTGGGGGCGATTCCATGGCGTTGCTTTCCCTTCTGCACGGGCTCTGCAAGGTTGCGGGCACACATCTCGAAGTTGTGACGGTCGATCATGGACTGCGACCCGAAGCAGCGGCAGAGGCAGATCTTGTCGCACGCTGCGCGGGCGATTTGGGGCTGCACCACGAGACGTTGCAATGGCGCGGGTGGGACGGGCAGGGCAATCTGCAAAACGCCGCGCGTCACGCGCGCTATGCGCTGATGGCCGATTGGGCGGCGCGGCGTGATTTGCCTTGCATTGCCCTTGGCCATACGGCGGATGATCAGGCCGAGACTGTCTTGATGCGATTGGCCCGCCGGGCGGGCGTGGATGGGCTGGCCGCGATGGCCCCGCAAAGCCAGCGGCAGGGTGTCACATGGCTGCGCCCCCTGCTGTCTGCCCGGCGCGAGGCTTTGCGCGATTATCTGCGTCGCGCGGGGCTTGAATGGGTTGATGACCCCAGCAACGATGACCCGCGTTATACACGCATTCAGACCCGCCAAACCCTAGCTGCCCTCGCCCCACTCGGCCTTGATGTTGAAACGCTGGCCGAGGTGGCAAGCAACATGGCCCGCGCCCGTGACGCACTCGACCAGCAAGCCGACCACGCCGCTGGCAATATTCTGCGCATGGAGGCAGGGGCCTGGGTGATCAGGGCCGATGCCTTTTTCGCCGAGCCGGAAGAAATCCGCCGTCGTTTGATGATCCGCGCGCTTGGGCAGATTAGTGGCGGTGCCTATCCTCCGCGTCGCGGGCCGGTGGCGGCGCTGATTGCTGGGCTGGCAAACGGGCAGGGCGCGACTCTGGATGGGTGCCAAACCCTGCTCCGGCGCGGTGAAATCTGGGTGTTTCGCGAATATAACGCGGTACGCAATCTTCATGTGCCTGCGGATCATCTGTGGGACGGGCGTTGGCGCGCGGTGCCGTCAACGGTGTTT---ATGGGGGCCGAATTGCGTGCATTGGGGCCAGAGGGTCTTGCATATTGTCCTGATTGGCGCAGCTTCGGGCGGCCCCGTGCGATGCTTTTGTCGACCCCGGCGGTCTGGCAGGGGGGGCGGCTGCTTGCAGCGCCCCTTGCTGGTCTGGACGAGAAATGGCATGTGCTGCTGGAACGCGATGCCGGTTGGCCTAAAACCACGCCATTATCGCATTGAATGGCTGATCTGAAAAAACTGGCAGAAGACATCGTTGGTCTGACTCTGCTTGAAGCACAAGAACTGAAAACCATCCTCAAAGACGAGTACGGCATCGAGCCCGCCGCTGGCGGCGCAGTGATGATGGCTGGTCCTGCTGATGGCGGCGCTGCCGAAGAAGAAAAGACCGAATTCGACGTCGTTCTGAAGAACGCCGGCGCATCCAAGATCAACGTGATCAAGGAAGTCCGCGGCATCACAGGTCTCGGCCTGAAAGAAGCCAAAGACCTGGTCGAAGCCGGTGGCAAGATCAAAGAAGGCGTCGACAAAGCCGAAGCCGAAGACATCAAAGGCAAGCTGGAAGCAGCTGGCGCCGAAGTCGAGCTGGCCTAAATGTCTCGAATTGGTAAGAAACCTGTCGCCATGCCTTCGGGCGTCTCGGCAGAGGTAAGCGGTCAGACGATTGAAGTGAAGGGCCCTAAGGGGACCCGCACCTTCCGTGCGACTGACGACGTGACGCTGACGGTCGAAGACAGCGCAATCACTGTGACACCGCGCGGCAAGTCCAAGCGCGCACGTCAGCAGTGGGGCATGTCCCGGACTATGATCGAAAACCTGGTGACCGGTGTCACCACGGGCTTCAAGAAAGAGCTTGAGATCCAAGGTGTTGGTTATCGTGCTGCCATCAACGGCAACACGCTGCGCCTGAACCTCGGTCTCAGCCACGATGTTGACTATGTTGCGCCCGAGGGCGTCACCGTAACAGCACCGAAGCAGACCGAAATCGTTGTGGAAGGCATTGACGAACAGCTTGTTGGTCAGGTCGCTGCGAACATCCGCGCTTGGCGCAAGCCCGAGCCCTATAAGGGCAAAGGCATCCGCTATAAGGGTGAGTTCGTGTTCCGCAAAGAAGGCAAGAAGAAGTAAATGCAAGTTATCCTTCTGGAACGTGTGGCCAAACTGGGCCAGATGGGCGAAGTCGTGGACGTAAAGTCTGGCTACGCTCGCAACTATCTTCTGCCCCAAGGCAAAGCGCTGTCGGCCTCCAAAGCCAACGTCGAAGCCTTTGAAGGTCAGAAAGCTCAGCTTGAGGCGCAAAACCTCGAGACCAAAAAAGAAGCCGAAGCCATGGCCGAAAAGCTGAACGGTCAGCAATTCGTCGTGATTCGCTCTGCTTCCGACGCTGGCGCGCTTTATGGTTCCGTCACCACCCGTGACGCCGCCGAAGCCGCCACCGAAGAAGGTTTCTCGGTTGACCGCAAGCAGGTCGTTCTGGGCCAGCCCATCAAGTATCTGGGCGTCCACGAAGTGCAGGTCGTTCTGCACCCCGAAGTGACCGCCACAATCGAGCTGAACGTCGCACGTTCGCCCGAAGAGGCCGAGCTTCAGGCATCCGGCAAATCGATTCAGGAATTGGCCGCCGAAGAGGAAGCCGCTGCTGAATTCGAAATCTCCGAACTGTTTGACGACATCGGTTCCGCCGCTGACGAA---GACGGCGACTCGGACGTTGTGCGCACCCCCGAGGGCGACGCACAGGACGACAGCAACAGC------TGAATGAGCGATAGTGACGGCAAGAAAACATTGGGTCTGCGCGGCGGCGCACGTCCGGGGAATGTAAAGCAAAGCTTTAGCCACGGGCGGACCAAGAACGTTGTGGTCGAAACCAAGCGCAAGCGTGTTGTGGTGCCCAAGCCGGGTGGCCAGAAACCAACCGGGCCGGGTGCCGGGCCGATTGGGGATCCCAAGAAACGTCCCGCAGGCATCACCGATGCCGAGATGGAGCGTCGTCTGAAAGCTGTTCAGGCTGCCAAAGCCCGTGAGGTTGAGGAAGCCGCAGCACGTGAGGCCGAGGAAAAGGCCCGCGCCGAAGAGCGTGAGCGCCGCCGGGCCGAGATTGAAGCCAAAGAACGCGAAGAGCGTGAGCGCGAGGAAAGCCTTAAGGCGAAAGCCGAGGAAGACGCGCGCCGCAAAGCAGAGGCCGAAGCG---------GCTGCTGCTGCTCCTGCGCCAGAACCTGCCGCCGCGCGTGAGCCGGGCAACAAGCCCATGCCCGCCGCAACGCCGCGCAAAACAGAGCGTGACCGCGACGAAACCAAGAAGCGTAGCAAGGGTGGCGACAGC---CGCCGTTCTGGTAAGCTGACAGTGAACCAAGCCCTCAACGGTGGTGAAGGTGGCCGTCAGCGTTCCATGGCGCAGATGAAGCGCAAACAAGAGCGTGCGCGTCAAAAAGCCATGGGTGGTCAGGTTGAGCGCGAGAAGATTGTGCGCAACGTCAATCTGCCCCCGGCAATTGTTGTATCCGAGCTTGCCAACCGTATGGCCGAAAAGACTGGCGCTGTTGTAAAAGCGTTGATGCAGAACGGCATGATGGTCACGCAGAATGAAACCATCGACGCGGATACCGCTGAACTCATCATCGAAGAGTTTGGCCACAAGGTTGTGCGCGTCAGCGACTCTGATGTCGAAGATGTCATCAAAGAGATCGTAGACAAGCCCGAAGACCTGCAAGGTCGTCCACCTGTGATCACGATCATGGGCCACGTTGACCACGGTAAAACATCGCTGCTCGACGCGATCCGTAATGCGAAAGTGGTTGCTGGCGAAGCCGGCGGGATCACCCAGCACATCGGTGCCTATCAGGTCACGACAGACAATGGTGCGGTGCTGTCCTTCCTCGACACACCCGGCCACGCGGCCTTTACCTCCATGCGTTCGCGCGGTGCTCAGGTAACAGATATCGTGGTTCTGGTGGTCGCGGCGGATGACGCCGTGATGCCGCAGACGATCGAAGCGATTGCCCACGCGAAAGCGGCCAAAGTGCCGATGATCGTGGCGATCAACAAGATCGATAAGCCTGCTGCGAACCCTGATAAGGTCCGGACGGATCTGCTTCAGCACGAAGTCATCGTCGAAAAGATGTCTGGCGATGTGCAGGATGTCGAAGTCTCGGCCGCAACGGGCCAAGGTCTGGACGAGTTGCTCGAAGCGATTGCGCTGCAGTCCGAAATCCTCGAACTGAAGGCGAACCCAGATCGGGCCGCTGTGGGTGCGGTGATCGAAGCGCAGCTTGATGTGGGCCGCGGCCCTGTTGCGACTGTTCTGGTTCAGAACGGCACGCTGCGTCAGGGTGATATCTTTGTTGTGGGTGAGCAGTACGGTAAGGTCCGTGCGCTGATCAATGACCAAGGCGAGCGCGTCAAAGAAGCAGGTCCTTCGGTACCGGTTGAGGTGCTTGGCATCAACGGCACGCCAGAGGCCGGTGACGTTCTGAACGTCACTGAAACCGAAGCGCAGGCCCGCGAAATCGCTGAATACCGCGCCAACGCAGCCAAGGACAAACGTGCCGCTGCTGGCGCTGCGACGACGCTGGAACAGCTTATGGCCAATGCCAAGGCGAACGAAGACGTCAGCGAGTTGCCGATCTTGGTCAAAGCCGACGTGCAAGGCTCTGCCGAAGCGATCGTTCAGGCGATGGAGAAAATCGGCAACGACGAAGTCCGCGTTCGGGTTCTGCACTCCGGTGTGGGCGCGATCACCGAGACGGATGTCGGTCTGGCCGAAGCTTCCGGCGCACCGATCATGGGCTTCAACGTCCGCGCCAACGCCTCGGCGCGGAACACGGCAAACCAGAAGGGCGTCGAAATCCGCTACTACTCGGTCATCTATGACCTTGTGGATGACGTCAAAGCGGCTGCAAGCGGCCTGCTCAGCGCCGAGATCAAAGAAAACTTCATTGGCTATGCCAACATCAAAGAAGTCTTCAAGGTCACCGGTGTGGGTAAAGTTGCGGGCTGTCTGGTCACCGAAGGCGTCGCTCGACGCAGCGCCGGTGTGCGTCTGCTGCGCGACAACGTCGTGATCCACGAAGGTACGCTGAAAACGCTCAAGCGCTTCAAAGACGAAGTGCCTGAAGTCCAGTCCGGGCAGGAGTGCGGCATGGCGTTTGAGAACTACGATGACATCCGTCCCGACGATGTGATCGAGATCTTCGAGCGCGAAGAAGTGACCCGCACATTGGCCTAAATGGCAAAGCCCAAGAAAACCCCCCGTCCCAAGGCGCAGACCCCAAAGGGGTTCCGCGATTATTTCGGGACCGAGGTGACCCATCGGGCCGAGATGCTCAGCAAGATTGCCGCAGTTTACCACCGCTATGGGTTCGACGCGCTGGAAAGCTCGGGCGTGGAGACGGTCGAGGCGCTTGGCAAGTTTCTCCCCGATGTGGACCGCCCCAACGAGGGTGTCTTTGCATGGCAGGAAGATTCCGAAGGCGACAAGCCCGGCGATTGGCTGGCGCTGCGCTATGATCTGACAGCGCCCTTGGCGCGGGTCTATGCCCAACACCGCAACGATCTGCCGATGCCCTACCGCCGCTATGCGATGGGTCCGGTTTGGCGCAACGAAAAGCCGGGGCCGGGGCGGTTTCGTCAGTTTTATCAGTGTGATGCGGATACCGTAGGCGCGCCATCAGTGGCAGCGGATGCCGAGATTTGCGCGATGCTGGCCGATTGTTTGGAAGAGGTCGGGATTGCGCGCGGCGACTATGTGGTGCGCGTGAACAACCGCAAGGTTCTGAACGGCGTGCTGGAAGTCGCTGGCCTTGCCGGTGACGACAAGGATATCGAGCGCGGCATCGTGCTGCGCGCCATCGACAAGTTGGACCGCCTCGGCCCGGAGGGCGTGCGCGCCTTGCTGGGGGACGGCCGCAAGGACGACAGCGGCGATTTCACCAAGGGCGCAGGTCTGGCAGACGCGCAGGCCGATGTGGTCATGGGCTTCATGCAGGCCAAGCGCGACAGCGGGGCCGAGACTGTGGCGCGGCTGCGCGAGTTGGTCGCGGGCTCCGATGTCGGTGTGCAGGGTGTGGATGAGCTTGAGTTGATCTCTGACCTTCTGGCCGCGGGCGGCTATGGCCCTGATCGGGTTGAGATTGATCCGTCGGTCGTGCGCGGGCTTGGCTATTACACCGGTCCGGTGTTCGAGGCCGAGCTGACCTTCGAGATCAAAGACGAAAAAGGCCGCGCGCGGAACTTCGGATCGGTCGCGGGCGGTGGGCGCTACGACGATCTGGTGAAACGCTTCACCGGGCAGGAAGTGCCAGCAACCGGCGTCTCCATCGGTGTCGACCGTCTGCTCGCCGCGCTGGCCGCCAAAGGGCGGCTTGAGGCCGAAGCGACAGGCCCCGTCGTCGTCACGGTGATGGACCGGGACCGCATGGCCGATTATCAAGCAATGGTCGCGGAACTGCGGCAGGCGGGCATCCGGGCAGAGGTCTATCTGGGCAACCCCAAGAACTTCGGCAACCAGATGAAATACGCCGACAAGCGGCAGAGCCCGGTTGTGGTCATCGAAGGCGGTGACGAGAAAGACCGCGGCGTGGTGCAGATCAAGGACATGGTGCTGGGCGCGCAACTGGCGCAGGAGGCCAGCCATGACGAGTGGAAAGAGCGCAAAAACCAATATGAGGTAAAGCGCAGCGATCTGGTGCAGGAAGTGCGCAATATTCTGGATCGGACCAACTGAATGGACGATCTGAAGCAAAAGTACCTGAGCCAAATCGCCGATGCCGGTGACGAATCCGCATTGGAAGACATCCGGCTGGCTGCCGTCGGCAAGAAGGGCGAGGTCGCGCTAAAGATGCGCGAGCTGGGCAAGATGACCCCCGAAGAGCGCCAGACCGCTGGCCCCGCGCTGAACGCGCTGAAGGATGAGATCAACTCGGCGCTTGCCGCCAAGAAGGCAGCACTTGGCGATGCCGCGCTTGATGACCGTCTGCGCAGCGAATGGCTCGACGTGACCCTGCCCACGCGGCACCAGCGTCAGGGCAGCATCCACCCGGTCAGCCAAGTGACCGAGGAGTTGACCGCAATCTTCGCCGAGATGGGTTTCTCGGTCGCCGAAGGCCCGCGCATCGACACCGATTGGTACAACTTCGACGCGCTGAACATCCCCGGCCACCACCCCGCCCGGGCAGAGATGGACACGTTTTACATGGCCCGTGCCGAGGGCGACGAGCGCGCCCCCCACGTGCTGCGCACCCACACCTCGCCCGTGCAAATCCGCACCATGGAGGCCGAAGGCGCGCCCCTGCGCATCATCTGCCCGGGCGGTGTCTACCGCGCCGACTACGACCAGACCCACACGCCGATGTTCCATCAGGTCGAAGGTCTGGCGATCGACAAGGATATCTCGATGGCGAACCTGAAATGGACGCTTGAGGAATTCTTTGCTGCCTTTTTCGAGATCGACGGCATCAAAACCCGCTTCCGCGCGTCGCACTTCCCCTTCACCGAGCCTTCGGCTGAGGTCGACATTCAATGTTCTTGGGTTGACGGTCAGTTGCGTATTGGCGAGGGCGACGGCTGGCTGGAAGTGCTCGGCTCCGGCATGGTGCACCCCAAGGTGTTGCAGGCGGGCGGGATTGACCCGAATGAGTGGCAGGGCTTTGCCTTTGGCATGGGGATCGACCGGATCGCGATGCTGAAATACGGCATTCCGGATTTGCGGGCGTTCTTTGATTCAGATTTGCGGTGGTTGCGGCATTATGGGTTCGCGAGCTTGGATCAGCCGAATTTGCATGGTGGGTTGTCGCGTTAAATGGAAATGGCCGACGAGGCCGGGCTCGACCTTGTCGAGATTTCGCCAAACGCCAACCCGCCCGTGTGCAAGATCATGGACTTCGGCAAGTTCAAATATGAACAGCAAAAGCGCGAGAGCGAAGCCCGTAAGAAACAGAAGATCATCGAGATCAAAGAAGTCAAATTCCGTCCGAATACGGATACGAATGACTACGATGTGAAGATGCGCAATGTCTTCAAGTTTCTTGAAGGTGGCGACAAGGTTAAGGTGACTTTGCGGTTCCGTGGCCGTGAAATGGCGCACCAGAACCTTGGTCGTGAGCTTTTGGAACGCGTTGCTGCGGATACCAAAGAAATTGGCCGGGTTGAGAACTTCCCCAAGATGGAAGGCCGCCAGATGGTCATGGTTATCGGGCCCCTGCCTAACTAAATGAAATTCACCCTCTCCTGGCTCAAGGACCATCTCGACACCACGGCGTCGATTGATGAGATCACCTATGCCCTCACCGACCTCGGCCTCGAAGTCGAAGGCGTCGAGAACCCGGCGGCGAAGCTGGCCGACTTTACCCTCGGCTATGTGCAATCCGCCGAGAAACACCCCGATGCCGACCGGCTGAACGTCTGTCAGGTGGAAACCGACGAAGGCGTCATGCAGATCATCTGCGGCGCGCCGAATGCCCGCCCGGGCATCACCGTCGTCGTGGCCAAGCCGGGCGTCTATGTGCCGGGGATCGACACCACCATCGGCGTCGGCAAAATTCGCGGCGTTGAGAGCTTTGGCATGATGGCCTCCGAGCGCGAGATGGAGCTGAGCGAGGAGCACGACGGCATCATCGAGCTGCCCTCGGGCAAGCCCGGCGACCGTTTCATTGACTGGCTGGCCGAGAATGATCCGGCAAAGGTCGACCCGGTGATCGAGATCGCCATCACGCCCAACCGCCCGGATGCGCTCGGCGTGCGCGGCATTGCGCGCGACCTCGCGGCGCGGGGCTTGGGCAAGCTGAAGCCGCGTGATTGCGATGCGGTTGAGGGCAGCTTTGCCTCGCCGATCAGCGTCAGCATTGACGACGACACGCTGGACGGCTGTCCAGTATTCTATGGCCGCGTGATCCGGGGCGTGAAAAACGGCCCCAGCCCGCAGTGGTTGCAAGATTGCCTGCGTGCAATCGGGCTGCGTCCGATCTCTTTCCTTGTCGACGTGACGAACTTCTTCACCTTTGACCGCAACCGCCCGCTGCACGTCTTTGACGCGGATAAGGTCAAAGGCAACCTGCGCGTCCACCGCGCCAAGGGCGGCGAAGAGATCGTGGCGCTGGATGAAAAGACCTATACCCTGCAAGCGGGGCAGATGGTCATCTCCGACGACAGCGGCGTGGAAAGCATCGCGGGCATCATGGGCGGCGAGGCCACGGGCGTGACCGAAGACACGGTGAACGTCTTTGTTGAAAGCGCCTATTGGGACCCGGTTCAGATCGCCTATGCGGGCCGTGCGCTCAAGATCAACTCGGACGCCCGTTATCGTTTCGAGCGTGGTGTGGACCCGGCATGGACGCCCTATGGTATCGAACACGCCACGCGGATGATCTTGGATCACGCGGGCGGCGAGGCCTCCGAGGTCGTGGTCGCGGGCAAGGTGCCGGACACGTCGCGCGCTTACAAGTTGGACGCGGCCAAGGTGCAATCTCTGGTCGGCATGACGATCCCCGAGAGCGACCAGCGCCAGACGCTCACCGCGCTTGGCTTCCAACTCGACGGCGACATGGCACAGGTGCCAAGCTGGCGTCCTGACGTGCAGGGTGAGGCCGATCTGGTGGAGGAGGTTGCACGGATTGCGTCCCTCACCAAGCTCGAAGGCAAGCCGCTGCCGCGCCTGACAACAGGCGTGCCGCGCCCCGTGCTCTCGCCCATGCAGCGCCGCGTCGTGACCGCACGGCGCACTGCGGCGGCGCTCGGCTACAACGAATGTGTGACCTACAGTTTCATCGACCAAGCCTCTGCTGCGCTCTTTGGTGGTGGCACAGACGAGACGCGCCTCGAAAACCCGATCTCGTCGGACATGAGCCATATGCGCCCCGATCTGCTGCCCGGTCTGCTGCAAACTGCCGCCCGCAATCAGGCGCGCGGCTTTGCCGATATGGCGCTGTTTGAGGTCGGCCCCGCGTTCAGCGGCGGTGAGCCGGGCGAAGAGCAGATCATGGTCAGCGGCCTGTTGGTGGGTCGCACCGGTCCGCGTGACGTGCATGGCGCGGCGCGTGCGGTTGATGTGTTTGACGCCAAAGCCGATGCCGAGGCCGTGCTGGCCGCCATCGGTGCGCCCGCCAAGGTGCAGGTCCTGCGCGGCGCGGCGGATTGGTGGCATCCGGGCCGTCACGGCAAAATCTGCCTTGGCCCGAAAAAGGTGCTGGGCGTCTTTGGCGAAGTGCATCCGCGTGTGTTGGCTGCGATGGACGTGAAAGGCCCGGCAATGGCTTTCACCATCTGGCCCACCGAAGTGCCGCTGCCGCGCAAATCCGGTGCCACCCGCCCGGCGTTGCAGATCAGCGACCTTCAGGCAGTCGAGCGTGACTTTGCCTTTGTCGTCGACGCGGATGTAGAGGCGCTGACGCTGGTTAACGCGGCCAAAGGGGCCGACAAGAGCCTGATCGAAGACGTGCGTGTCTTTGATGAGTTCATCGGCGGCAGCGTTGGTGAAGGCAAAAAATCCCTCGCCATTACCGTGCGTTTGCAGCCGAGTGACAAGACGCTCAAAGATGCCGATATCGAAGCGGTTGGTGCTAAGGTCGTTGAGAAAGTCACCAAAGCCACCGGCGGCCTGCTGCGCGGCTGAATGCACGCCTATCGCAGCCATACCTGTGCCGATCTGAGCCTTGAGAACAAGGGTGACAATGTGCGCCTGTCCGGCTGGGTGCACCGGGTACGGGATCACGGCGGCGTGCTGTTTCTCGATCTGCGCGACCATTACGGCATTACTCAAGTGATCTGCGACCCCGACAGCCCCGCCTTCGCCGAAATGGAGAAGGTCCGCGCGGAATGGTGCGTGCGCATCGACGGCACGGTAAAGGCGCGGGACGAAAGCTTGGTTAATCCCAAGCTGCCGACAGGCGCGATCGAAGTCTATGCGCGTGAAATCGAAGTGCTGGGCTCTGCTGCCGAACTGCCGCTGCAAGTGTTCGGCGATCAGGAATACCCTGAGGAAACGCGCCTGCGGTACCGTTACCTCGACCTGCGTCGTGAGAAGATGCAAAAGAACATGACACTGCGCTCGGACGTTGTGACCTCGATCCGCAAGCGCATGTGGGACCAGAACTTCCGTGAGTTCCAGACCCCAATCATCACCGCCTCTTCCCCTGAAGGGGCTCGCGACTTCCTCGTGCCGTCGCGCCTGCATCCGGGCAAGTTCTACGCGCTGCCGCAGGCGCCGCAGCAGTTCAAACAGCTACTGATGGTCTCGGGCTTTGACAAATACTTCCAAATCGCGCCCTGTTTCCGCGACGAGGATCCGCGTGCCGATCGGTCGCCCACCGATTTCTACCAGCTCGACATGGAGATGTCCTTTGTCACCCAGCAGGATGTGTTCGACACGATCCAGCCGGTGATCGCGGGCATTTTTGAGGAGTTTGGCGGCGGCAAGAAGGTCGACGAGACTTGGGAGCAGATTTCCTATAAGGACGCGGCGCTGTGGTATGGCTCGGACAAGCCCGACCTGCGCAACCCGATTAAGATGCAGGTCGTGTCCGACCATTTCCGCGATTCCGGTTTCGCGATCTTTGCCAAGCTGCTGGAGCAGGACGGCACCGAGATTCGCGCAATCCCTGCGCCCACGGGCGGCAGCCGCAAGTTCTGTGACCGCATGAACGCCTTCGCTCAGAAGGAAGGTCTGCCCGGCATGGGCTATATCTTCTGGCGCGATCAGGGC---------AACGGCATGGAAGCCGCTGGCCCCTTGGCCAAGAACATCGGCCCTGAGCGCACCGAGGCGATCCGCCAGCAGTTGAATCTGGGCGTTGGCGATGCGGCCTTCTTCCTTGGCGGCAAGCCGAAGTCTTTCGAGGCGGTCGCAGGCCGCGCGCGGAACGTCATTGGCGAAGAGCTGAACCTCACCGACAAGGAACGTTTCGCTTTTGCTTGGATCGTGGACTTCCCGATCTACGAGCGGGACGAAGAGACCGGCAAGATCGACTTCGAGCACAACCCCTTCTCCATGCCGCAGGGCGGGATGGAAGCGCTACAGGGCGATCCGCTGAAGGTGTTGGGCTATCAATATGACCTCGCCTGCAACGGCTATGAGTTGGTGTCGGGTGCTATCCGGAACCACAAGCCCGAGATTATGTTCAAAGCGTTTGAGATCGCGGGCTATGGCGAAGATGAGGTGCGCAAGCGCTTTGGCGGCATGGTCAACGCATTCCAATACGGCGCCCCGCCCCACGGTGGCTGTGCCGCGGGCATCGACCGTATCGTCATGCTGCTGGCCGAAGAGGCGAACATCCGCGAAGTCATTCTCTTCCCGATGAACCAGCGCGCCGAAGACCTGATGATGAATGCGCCGTCCGAGCCCACCAGCGATCAGCTGATGGAGCTTGGCCTGCGGGTCATCCCGCAGGAGTGAATGACACCGCTTGAAAACATCCGCAATTTCTCCATCGTGGCGCATATCGACCACGGCAAATCCACGCTGGCCGACCGGTTGATCCAACTCACCGGCACGGTCGCCGAACGCGACATGCAAAGCCAGCTTCTGGACAACATGGATATCGAGCGGGAGCGGGGCATCACCATCAAGGCCAACACCGTCCGCATCGAATACCCTGCCAAGGACGGCAAAACCTATGTGCTGAACCTGATCGACACGCCCGGACACGTCGACTTCGCCTATGAGGTCGCGCGCTCCATGCATGCGGTCGAAGGCTCGCTGCTGGTCGTCGACGCCACTCAGGGCGTCGAGGCGCAGACGCTGGCCAATGTCTATACCGCCATCGATGCGGATCATGAGATTGTGCCGGTGTTGAACAAGGTCGACCTGCCCGCCTCTGACCCCGACCGCGTGCGCGAACAGATCGAGGACGTGATCGGCATCGACGCCTCCGAGGCCTGCCTGATCTCAGCCAAGACCGGCGTCGGCATCCCCGATGTGTTGGAAGCGATCGTCAATAAACTGCCCGCGCCCGGCGGCGACCCCGATGCGCCACTCAAGGCGATGCTGGTAGACAGTAAATATGACCAATACCTTGGCGTCATCTGTATCGTCCGGATCATCGACGGCACCCTGAAAAAGGGCGACCGCATTCGCATGATGAAGACCGGCGGCACCTATGACGTGGACGATGTGGGCGTTTACCGGCCCAAGATGACCGGCGTCGAAAGCCTCGGCCCCGGCGAGATCGGTTATCTCAACGCCTCGATCAAACAGGTCCGCGACACCCGCGTCGGCGATACGATCACCCATGAGAAGCGCAAGTGTGAAACCCCGCTGCCGGGCTTTAAGCCGTCCGTGCCGGTGGTTTTCTGTGGTCTCTTCCCGGTCGACGCCAATGACTTCGAAGACATGCGCGATGCGATCGAAAAGCTCGCCCTGAACGATGCCTCCTTCACCTATGAGATGGAAACATCTGCCGCGCTCGGCTTTGGCTTCCGCTGCGGTTTCCTTGGCCTGCTGCACCTCGAAGTGATCCGCGACCGGCTTGAGCGTGAGTATGACATCGACCTGATCACCACCGCCCCTTCGGTGATCTATCACGTCCACATGAAGGACGGCACGATGCAGGAGCTGCACAACCCCGCCGACATGCCCGACATGACGTTGGTGGACCACATGCAAGAGCCGCGGATCAAAGCGACGATCCTCGTGCCCGACGAATACCTCGGCGACGTGCTGAAGCTCTGCCAAGACCGCCGCGGCATTCAGGAAGACCTGACCTATGCCGGTTCGCGGGCGATGGTCGTCTATGACCTGCCGCTGAACGAGGTGGTGTTTGATTTCTACGACCGGCTGAAGTCGGTCACCAAGGGTTACGCTAGTTTTGACTACCAGATGATCGGCTACCGGCAGGATAATTTGGTCAAGATGCAGATCCTCGTGAACGACGAGCCGGTGGATGCGCTGTCGACCATGGTGCACCGCGACCGCGCCGAGATGCGCGGCCGGGCGATGGTGGAAAAGCTCAAAGACCTGATCCCGCGCCACATGTTCAAGATCCCGATCCAGGCGGCCATCGGTGGCAAGGTGATCGCGCGCGAGACGCTCTCGGCCATGCGCAAGGACGTGACGGCGAAGTGTTATGGCGGCGACGCCACGCGGAAGAAGAAGCTGCTGGAGAAGCAGAAGGCCGGTAAGAAGAAGATGCGCCAGTTCGGGAAAGTGGATATCCCGCAGGAGGCGTTTATTTCCGCTCTAAAAATGGACAGCTAAATGAGCCTGCCCCCTGGATTTTTGGAAGAACTGCGTGACCGCGCCAGCCTGTCTCAGGTGGTGGGGCGTAAGGTCATATGGGACAACCGAAAGTCCAATCAGGGCAAAGGCGACATGTGGGCGCCGTGCCCCTTCCATCAGGAAAAATCCGCCAGCTTTCACGTCGATGACCGCAAAGGGTTCTACTACTGCTTTGGTTGCCACGCGAAGGGGGATGCGATCTCTTTCGTGCGCGAGACCGAGAATGTGAGCTTTATGGAGGCGGTGGAGATCATCGCCCGCGAAGTCGGCATGCCGGTGCCCAAGCAAGACCCCCGTGCGCAGGCCAAGGCCGACAAGCGTACCCAACTGGCCGAGGTGATGGAGCAAGCGGTGCAGTGGTTCCGCCTGCAATTGCGCACCGGGGCTGCGGGGGCGGCGCGGGACTATCTGGCCAAGCGCGGCCTCTCGGAACAGGCGCAGGCGCATTGGGAGATTGGCTTTGCGCCGAACAGTTGGCAGGGGCTTTGGGATGCGCTCAAATCCAAGGGCGTCGCGGATGAGTTGATCCTTGCTGCGGGGCTGGCGAAACCCTCGTCCAAGGGCGGCAAGCCCTATGACACTTTCCGCGGGCGCATCATGTTCCCGATCCGCGATGCGCGGGGGCGGGCGATCGCTTTTGGCGGTCGCGCGATGGACCCGGAGGATAAGGCGAAATACCTCAACTCGCCTGAGACCGAACTCTTCGACAAGGGCCGCAGCCTTTACAACGTGAAAGACGCCCGCGCCGCCGCCGGTCGGGGTCAGCCGCTGATCGTCGCCGAAGGCTATATGGATGTGATTGCCCTGCACGGGGCGGGGTTTGAAGGCGCCGTGGCCCCGCTTGGCACCGCGATTACCGAAAACCAGTTGCAGATGCTCTGGCGGATCGCGCCCGAGCCGATTATCACGCTGGACGGCGATGCGGCGGGCCAGCGCGCGGCGCTTCGATTGATCGATCTGGCCCTGCCGCTGTTGGAGGCCGGGCAAAGCCTGCGTTTCGCGGTGATGCCCGAGGGGCAGGATCCCGACGATCTGCTGCGCGCGCAGGGGGCGGGGGCCTTGCAAAAACTCTTGGACAACGCCCTACCCATGGTGCGCCTGCTGTGGCAGCGCGAGACCGAGGGCAAGGTTTTCGACAGCCCCGAACGCAAGGCCGCGCTGGACAAAAGCCTGCGCGAAAAGATCAAGCTGATCCGCGACCCGTCGATCCGCAGCCACTACGGGCAAGAGATCAAAGACCTGCGGTGGGACCTGTTTCGCCCGCAAAAGAAACGCCCTAAAGGGCCGGGCAAG------GGCAAAGGCGGCAAGGGCAGCTGGGGT---GCCCCGCTGGCCCCATTGGCCAGCACCAAAGCCTCGGCCCTCGTGGCGATGAGCGATGAGCAGGTGGGCATGCATCTGCGCGAGGCGGTAATCCTCGTCGCCCTCGTGGACTGCCCTCAGATGATCGAAACCTTCGAGACCGGGTTGGAGGGGATGGGCTGCGCGGACCCCGACCATGCGCGGCTGCGCGACCTGCTGCTGCGCTTTGGCCATGCCGGTGAAGATGTGCTGCGCGAAGAGATTTCCTATTCCCTCGGCTGGGAGACACTTGAAAACATGAAGGGGCAACGCCACGTTGCGATCACGCCCTGCATCCGCAATCCCGGCAATGTCGAAATGACCCGGCTGACCGTGGCGGAGGAACTGGCCAAACTGGACGCTGCCCGCGGGCTGAACGAGGAAATTGCAGAAGCCGTAGAAGACCTAAGCGGCCCGGCAGACGAGGGGGTCACTTGGCGTCTGAGCGAAGCAGCCCGCGCCGCCGACATGGCCCGGCGCAGCGCACAAGAAGACAACGGTGAATTTGACGTTGGCGACAACGGCATGACCATGGACCGCGATGAGCGCAGTGCACTGGATGCGCTGCTCGGAACGATTAAATATGAGAAGTCGAAGGGTCGCGGATGAATGTGCGCCGAGACCCCCGACTATAAAGCCACGTTGAACCTGCCCAAAACCGATTTCCCGATGCGCGCTGGACTGCCCAAGCGCGAGCCCGCTTGGCTGGAACGCTGGGAGAAAATCGGCGTCTATGACCGCCTGCGCGAGAAAGAGGGTCGTGAGCCTTTCACCCTGCACGATGGCCCTCCCTACGCCAACGGGAACCTGCACATCGGTCACGCGCTGAACAAGACGATCAAGGACATGATCGTGCGCAGCCACCAGATGATGGGCTATGATGCGCGCTATATCCCCGGCTGGGATTGCCACGGCCTGCCGATCGAATGGAAGATCGAAGAGCAGTACCGCAAGAAGGGCCGCGACAAGGATCAGGTGCCGATCAACGAATTTCGCGCCGAATGCCGCGAGTTTGCGCGTGGCTGGGTTGACGTACAGCGCGAAGAATTCAAGCGGTTGGGCATTACCGGCAATTGGGAAAACCCGTACCTGACGATGGACTTCCACGCCGAGCGCGTGATCGCTGAGGAATTCATGAAGTTCCTGATGAACGGGACGCTTTATCAAGGCTCCAAACCCGTGATGTGGTCGCCGGTCGAAAAGACCGCGCTGGCCGAGGCGGAGGTCGAGTATCACGACAAGGAAAGCCACACCGTTTGGGTGAAGTTTAAGGTCGTC------GAAACCGGCGATCGGACGCTCGACGGGGCAAATGTGGTGATCTGGACGACCACGCCTTGGACCATGCCCTCGAACAAGGCCGTGGTTTACGGCGAGAGCATCTCTTATGGTCTGTATGAAATCACCGGCCGACCCGAGGAATGCTGGGTCAGCATCGGTGACCGCTATCTGCTGGCTGACGATCTGGCTGCGGATGTATTTGCCCGCGCGCGTCTGGACGAGGGGATGTACCGCCGTCTTTGCGATGTCACGCAGGAAGATTTAGCCAAGATTAAGCTTCAGCACCCGCTCGCCGGGGCCGAGGGCGCGAACGGCGAATGGGACGACCTGCGCGATTTCCGCGCGGCGGATTTCGTCACCTCGGACGAGGGCACGGGCTTCGTGCATTGCGCACCTTCCCATGGCCTCGAGGAATACGAACTCTACCGTGACCTTGGCATGCTGCCGCAGGTCATCACCTATAACGTCATGGAAGACGGTCGTTTCCGCGACGATCTGCCGTTCTTTGGCGGCAAAGCGATCCTCAAGCCGAACGGCAAGGAGGGTAACGCGAACAGCGCGATCATCGACAAGCTGGTCGAGGTCGGCGGTCTGCTGGCGCGTGGCAAGATCAAGCACAGCTACCCGCACAGCTGGCGCTCCAAGGCGCCGGTGATTTATCGCAACACGCCGCAGTGGTTTGCCGCCATCGACAAGGAGGTCGGCGACGGACTGGACCAGAACGGCAAGACGATCCGCGAGCGCGCGCTGACGTGTATCGACAAGGTCAACTGGGTGCCGAAATCCGGCCGCAACCGTCTGCATTCGATGATGGAGGCGCGGCCCGACTGGGTGCTCAGCCGCCAGCGCGCCTGGGGCGTGCCGCTGACCTGTTTCGTGCGCAGGGGTGTCGCTCCGACGGATGAGAACTTCCTGCTGCGCAACGAAGCAGTGAACCAGCGTATCACCGAGGCTTTCGAGACCGAAGGCGCGGATGCGTGGTATGCGGAAGGCGCGAAGGAGCGGTTCCTCGAAGGCATCGTTGATCCTGCAGAGTTCGATCAGGTGACCGACATCCTCGACGTTTGGTTCGACAGCGGTTCGACCCATGCCTTCACCCTGCGCGACCGTGAAGACGGGACCGAGGACGGCATCGCAGACGTTTACATGGAGGGCACCGACCAGCACCGCGGCTGGTTCCATTCGTCGCTGCTGCAATCCGTCGGCACCACGGGCCGCGCGCCGTATCGCAATGTGGTGACCCATGGTTTCACGTTGGACGCCAAGGGCATGAAGATGTCCAAATCCATCGGCAACACCATCGTGCCCGAGAAGATCGTGCAGCAGTATGGCGCGGATATCCTGCGTCTCTGGGTGGCGCAGACCGATTACACCGCCGACCAACGCATCGGGGATGAGATCCTCAAAGGTGTGGCCGACAGCTATCGCCGCCTGCGCAACACCATGCGCTATATGCTCGGCGCATTGAATGATTTCAGCGAGGCGGACCGCGTTGATCCCGCCGATATGCCCGAGTTGGAGCGGTGGGTGCTGCACCGGGTGGCGGAACTCGACAAGGTTGTACGCGACGGCTTTGCGCGCTTTGATTTTCAGGGCGTGTTTCAGGCGGTGTTCACCTTTGCGACGGTCGATCTCTCGGCGTTCTACTTCGATATCCGCAAGGATGCGCTCTATTGCGATGGCGATACCCTGCGCCGCCGCGCCGCGCGCACGGTGCTGGATATCCTGTTCCACCGTCTGACCACATGGCTTGCACCTGTGCTGGTCTTCACGATGGAGGAAGTCTGGCTGGAGCGTTTCCCGGGCGAGGAATCCTCCGTGCATCTGGTGGATATGCCCGAAACGCCAGAGGCTTGGCTAAACCCGGAGCTGGCGGCGAAATGGTCCAAGGTCCGCGCGGCGCGGCGTGTGGTGACCGCGGCGCTCGAAGTGCAGCGGACCGAGAAGGTGATCGGTGCCTCGCTCGAAGCGGCCCCCGTTGTGCATGTCGACGACGCGGCGCAGCGCGAGGCGTTGGAGAGCGTGTCTTTCGAAGACGTAGCCATCACCTCTGACATCACCGTAACCGGCGATGCAGCTCCGGCTGAGGCTTTCCGCATGCCCGAGGCGCAAGGCGTCGCCGTGGTGTTCGAGAAAGCCGAAGGCGCCAAATGCGAGCGTTGCTGGAAGGTGCTGCCGGATGTCGGCACGCATGAGCATCCCGGCGTTTGCGGGCGCTGTGATGAAGCGGTGCGTTAAGCCGCTCCAAGCCAGAACATCCGCATTCGCCTGAAGGCGTTTGACTATCGGGTGCTCGATTCTTCTACACAGGAAATCGTCAGCACCGCGAAGCGCACAGGCGCTTCGGTTCGCGGACCCATTCCGCTGCCGAACAAAATCGAGAAATTCACCGTTCTGCGTGGTCCCCACGTTGACAAGAAATCCCGTGACCAGTTCGAAATCCGCACGCACAAGCGTCTGCTGGATATCATTGATCCGACCCCCCAGACCGTGGACGCGCTGATGAAGCTCGACCTCGCCGCTGGCGTGGATGTCGAGATCAAGCTGCAATCGTAAATGGCTGACGAAATCAAAACACTTGAAGGTCTCGAAGCTGCTGTGACCGGCGGCGTACAGGGCACCGAA------------ACCGAAATGACCCCGCGTGAGCCGGTTCGTGACGAACTGGGCCGCGCCTATGCCACCGGCAAACGTAAAGACGCGGTCGCCCGCGTTTGGATCAAGCCCGGCTCCGGCAAGGTTATCGTGAACGGCAAGCCGCAGAACGAATACTTCGCACGTCCTGTGCAGCAGCTGATCCTCGCGCAGCCTTTCGGCATCACCAACACCGAAGGTCAGTTCGACGTGTTCGCAACTGTCAAAGGCGGCGGTCTGTCCGGTCAAGCGGGCGCGGTCAAGCACGGCATCTCCAAAGCGCTGCAGCTTTACGATCCCTCCCTGCGCGGTGCGCTGAAAGCGGCAGGCTTCCTGACCCGTGACAGCCGCGTCGTCGAGCGGAAGAAATACGGTAAGGCCAAAGCGCGTCGGAGCTTCCAGTTCTCCAAGCGTTAAATGCCAACGATCCAACAGCTGATCCGCAAGCCGCGGCAGCCGAAAGTCAAACGTTCGAAATCCATGCACCTGCAGGAGTGCCCGCAAAAGCGTGGCGTCTGCACACGGGTTTACACAACAACACCCAAGAAGCCGAACTCGGCCATGCGTAAGGTTGCCAAAGTGCGCCTGACCAATGGTTTCGAAGTCATCTCCTACATCCCGGGTGAGAGCCACAACCTTCAGGAACACTCCGTGGTTCTGATCCGCGGCGGTCGTGTAAAAGACCTTCCCGGTGTGCGTTACCACATCCTGCGCGGTGTTCTCGATACCCAAGGTGTCAAGGACCGGAAGCAGCGTCGCTCCAAGTACGGTGCGAAGCGTCCGAAGTAAATGGCACGCGATAAGACAAAGACCAAGCGTAAGGTCTCCAAGAACATCGCCGCAGGTGTGGCGCATGTGAACTCAAGCTTTAACAACACCAAGATCCTGATCTCGGATGTTCAAGGCAACGCAATCTCCTGGTCCTCCGCTGGCACCATGGGCTTCAAAGGGTCGCGTAAGTCGACACCTTACGCAGCTCAGATGGCCGCAGAAGATGCTGGCCGCAAAGCTCAGGATCACGGCGTCAAGACGCTGGAAGTCGAAGTGCAAGGCCCCGGTTCCGGCCGTGAATCCGCACTGCGCGCTCTGGCTGCTGCCGGTTTCAACATCACGTCGATCCGTGATGTGACCCCGATGGCCCACAACGGTTGCCGCCCGCCCAAACGCCGCCGCGTTTAAACAAAGCCAAACCGACGCGGCCTTCTGATCATCCTCAGTTCCCCCTCCGGGGCGGGCAAATCGACCATGGCCCACGCCCTGCGCGCATGGGATCCGACGATCAACTTCTCGGTCTCTGCCACCACCCGCGCGCCCCGCCCGGGCGAAGAGGACGGCACGGATTACCGCTTTGTCGGCGAAGAGGATTTCCGTCAGGCCGTGGCTGAGGGCGAAATGCTGGAACACGCCCATGTCTTCGGCAACTTCTATGGCTCCCCCAAAGCCCCCGTGCAGGCGGCGATTGATCAGGGGCAGGACATCCTTTTCGACATCGACTGGCAGGGCGCCCAGCAGATCCGTAACTCTGACCTGAACACGCACACGCTGTCGATTTTCCTTCTGCCGCCCTCGATCACCGAGTTGAAGCGCCGTTTGGAAAGCCGGGGGCAGGACGATGCCGAGACCATTGCCAAGCGCATGGGCAAAAGCTGGGATGAGATCAGCCACTGGGATGGCTATGACTTCGTGCTGGTCAACGACGATCTCGACCAGACCGAGGCGCGGCTGAAATCCATCATCACCGCCGCGCGGCTGCGGCTGAGCCAACAGCCCGCCATCAAGGATCACGTCCGCCGTCTGCAATCTGAATTTGAGGATTTGAAATGAATGGCACAATCCTTCCTTGGCCAGAAACGTCTGCGTAAATATTACGGCAAAATCCGCGAAGTGCTGGAGATGCCGAACCTCATCGAGGTTCAGAAATCCTCTTACGATCTGTTCTTGAATTCCGGCGACGCCGAGACCCCCACCGATGGTGAAGGTATCACAGGCGTTTTCCAGTCGGTTTTCCCGATCAAAGATTTCAATGAGACCTCCGTGCTTGAATACGTCAAGTACGAGCTCGAAAAGCCGAAATACGATGTTGAGGAATGTCAGCAGCGTGACATGACCTACAGCGCGCCACTTAAGGTGACGCTGCGCCTCATCGTGTTTGATGTGGACGAAGACACCGGTGCCAAGTCGGTCAAAGACATCAAAGAACAAGACGTGTTCATGGGCGACATGCCCCTGATGACGCCGAACGGCACTTTCGTCGTAAACGGCACCGAGCGTGTGATCGTATCCCAGATGCACCGCTCCCCGGGCGTGTTCTTTGACCACGACAAGGGCAAAACCCACTCTTCGGGTAAGCTCCTGTTCGCTTGCCGCATCATCCCATACCGCGGTTCCTGGCTGGACTTCGAATTCGACGCCAAAGACATCGTCTTTGCCCGGATCGACCGCCGCCGCAAACTGCCTGTGACGACCTTGCTCTATGCCCTCGGGCTGGACCAAGAGGCGATCATGAACGCCTACTACAAGACGGTCACCTACACGCTTGAGAAGAACAAGGGCTGGGTTGCACCCTTCTTCCCCGACCGCGTGCGCGGCACCCGTCCGACCTATGATCTGGTGGATGCAGCGACAGGTGAAATCCTGTTCGAAGCGACCAAAAAGGTCACCCCTCGCGCCGTCAAAAAGCTGCTCGACGAAGGCAAGGTCAAAGACCTGCTGCTGCCTTTCGATCACATCGTTGGCAAGTTCGTCGCGCGTGACATCATCAACGAAGAAACCGGTGCGATTTACGTCGAAGCCGGTGACGAGTTGACGCTGGAATACGACAAAGACGGCACCCTGATCGGCGGCACCGCGAAAGAGCTGATCGACGCGGGCATCACCGAGATTCCGCTGCTGGACATCGACAACGTCAACGTCGGTCCTTACATGCGCAACACCATGGCGATGGACAAAAACATGAACCGCGACACCGCGCTCATGGACATCTACCGCGTCATGCGCCCGGGCGAGCCGCCCACCGTTGAGGCCGCGTCCGCGCTCTTTGACACGCTGTTCTTTGATAGCGAACGCTATGACCTCTCGGCTGTTGGCCGCGTGAAAATGAACATGCGTCTGGCGCTTGATAAGCCCGACACACAGCGCACGCTGGACCGTGACGACATCGTCGCTTGTATCAAAGCGCTGGTTGACCTGCGCGATGGCCGTGGCGACATCGACGACATTGACCACCTCGGCAACCGTCGTGTGCGTTCGGTCGGCGAATTGATGGAAAACCAGTACCGCGTTGGCCTGCTGCGCATGGAGCGCGCGATCAAAGAGCGGATGTCCTCCGTCGAGATCGACACCGTGATGCCGCAAGACCTGATCAACGCCAAGCCAGCAGCGGCTGCGGTGCGTGAATTCTTTGGCTCTTCGCAGCTGTCGCAGTTCATGGACCAAACCAACCCGCTCTCCGAAGTGACGCACAAGCGTCGTCTCTCGGCGCTTGGGCCGGGCGGTTTGACACGTGAGCGTGCGGGCTTTGAAGTGCGCGACGTTCACCCGACCCACTATGGTCGGATGTGCCCGATTGAAACGCCGGAAGGGCCGAACATTGGTCTGATCAACTCGCTGGCCACTTTTGCCCGCGTGAACAAATACGGCTTCATCGAAACGCCTTACCGCGTTGTCAAAGACAGCACCGTCACCGACGAAGTGCACTACATGTCCGCGACCGAGGAAATGCGCCACACCGTGGCTCAGGCAAACGCCAACCTCGACGAAAACATGAAGTTCGTGAACGAACTGGTTTCGACCCGTCAGTCCGGCGACTACACGCTGGCGCCGACGGAAAACGTGGACCTGATCGACGTTTCGCCCAAGCAGTTGGTCTCGGTCGCTGCATCGCTGATCCCGTTCCTCGAAAACGACGACGCCAACCGCGCTCTCATGGGCTCGAACATGCAACGTCAGGCCGTACCGCTTCTGCAAGCTGAGGCGCCGCTTGTCGGCACCGGCATCGAAGAAGTCGTGGCGCGCGATTCCGGGGCGGCCTACACGGCACGCCGCGCGGGTATCATCGACCAAGTCGATGCCTCCCGTATCGTGATCCGGGCCACCGAAGACCTTGAGTTGGGCGACGCGGGTGTGGACATCTACCGCATGCGCAAGTTCCAGCGCTCGAACCAGAACACCTGCATCAACCAGCGTCCGCTGGTGAAAGTGGGCGAGAAGGTCACCAAGGGTCAGGTCATCGCGGATGGTCCGTCGACCGATATGGGGGAACTGGCGCTCGGCAAGAACGTCGTCGTCGCCTTCATGCCGTGGAATGGCTACAACTACGAAGACTCCATCCTGATCTCCGAGCGGATTTCCCGTGACGACGTCTTCACCTCAATCCACATCGAGGAATTTGAAGTCGCCGCGCGTGACACGAAGCTTGGGCCAGAAGAGATCACCCGAGACATTCCCAACGTCGGCGAGGAAGCCCTTCGCAACCTCGACGAAGCCGGTATCGTGTATATCGGTGCGGACGTTGAGCCGGGCGACATCCTTGTGGGTAAGATCACACCGAAAGGCGAAAGCCCGATGACGCCGGAAGAAAAGCTTCTGCGCGCCATCTTCGGTGAGAAAGCCTCTGACGTGCGCGACACCTCGTTGCGTGTGAAGCCGGGCGATTTCGGCACTGTTGTCGAAGTGCGTGTCTTCAACCGTCACGGCGTTGAGAAAGACGAGCGTGCGCTGCAGATCGAGCGTGAGGAAGTTGAACGTCTGGCCCGTGACCGGGACGACGAATTGGCGATCCTTGACCGCAACATCTATGCGCGTCTCAAGGACATGATCCTTGGCAAAATCGCCGTCAAAGGCCCCAAAGGCGTGAAGGCGAACAGCCAGATCACCGAGGAACTGCTGGAAACCCTGACCCGTGGTCAGTGGTGGCAGTTGGCCCTTGAGGACGAGGATGACGCGAAGATCGTCGAAGCCCTGAACGAGCAGTATGAGATCCAGAAGCGGACCTTGGATGCGCGTTTCGAGGACAAGGTCGAGAAAGTACGTCGTGGTGACGATCTGCCCCCGGGTGTGATGAAGATGGTCAAAGTGTTCGTGGCGGTGAAGCGTAAGCTGCAGCCGGGCGACAAGATGGCCGGTCGTCACGGGAACAAGGGTGTGATCTCGAAAGTGGTGCCGATGGAGGACATGCCGTTCCTCGCCGATGGTACCCCGGTCGACTTCTGCCTTAACCCTCTGGGTGTTCCATCGCGGATGAACGTTGGTCAGATTCTTGAGACACACATGGGCTGGGCCGCACGCGGTCTGGGCATCAACATCGACGAGGCGCTGCAAGAGTATAAGCGTTCCGGCGATCTGACACCTGTGCGTGAAGCAATGCAGCTGGCCTATGGTGATGATGTCTACGAAGAGGGCATCACCGGCATGGACGAGGACACGCTTCTTGAAGTGGCCGACAACGTGCGCCGCGGTGTGCCGATCGCCACACCGGTCTTTGACGGTGCCAAGGAAGCGGACGTGAATGACAGCCTCAAGCGGGCCGGGTTCGACACCTCCGGTCAGTCGGTGCTGTTCGATGGTCGTACAGGTGAGCAGTTTGCCCGCCCCGTGACCGTCGGCGTCAAGTACCTGCTCAAGCTGCACCACTTGGTGGACGACAAGATCCACGCACGTTCGACCGGGCCGTACTCGCTCGTCACACAGCAGCCGCTGGGTGGTAAAGCACAGTTCGGTGGCCAGCGCTTTGGTGAGATGGAAGTCTGGGCTCTCGAAGCCTACGGCGCCGCTTACACATTGCAGGAAATGCTGACCGTCAAGTCGGACGACGTCGCGGGCCGGACCAAGGTCTATGAAAGCATCGTCAAGGGCGAGGATAACTTCGAAGCGGGCATTCCAGAGAGCTTTAACGTTCTCGTCAAGGAAGTGCGCGGCCTCGGCCTGAATATGGAACTCCTGGATGCGGAGGAGGATGAGTGAATGCCGCTTTATGAGCATGTTATGATTGCGCGTCAGGACCTGTCCAACACGCAAGCAGAAGGCCTCATCGAACACTTTGGCACCGTCCTCGCGGACAACGACGGCAAGCTCGTCGACAGCGAGTACTGGGGCGTCAAAACGATGGCCTACAAGATCAACAAAAACCGCAAGGGCCACTATGCCTTCCTGCGTTCGGACGCCCCCGCGACCGCCGTGCAGGAAATGGAGCGCCTGATGCGCCTGCATGATGACGTGATGCGCGTTCTGACCATCAAAGTTGATGAGCACAAAGAACTGCCGTCCGTACAGATGCAAAAGCGTGACGAGCGGCCCGACCGCCGCGAACGCCGTTGAGTGACATCCCAGATCGAGGTGGCAGCGCTGGACAAGGCGCAAGCAGAAACGGAACTGGCGCGGTTGGCAGAGTTGCTGAGTGCGGCCAACACGGCCTATCATACGGAAGATGCGCCCGAAATCTCGGATGCGGAATATGATGCGCTGAAGCGGCGCAACGCAGCAATCGAACAGCGTTTCCCCGAACTAAAACGAAGCGATAGCCCCTCGGAGCAAGTCGGTGCACCGGTAGCCGACGGCTTTGGCAAGGTGCGCCATGCGGTGTCGATGCTGTCGCTGGCCAATGCCTTTGACGCAGAGGATGTGACAGAGTTCGATGCGCGGATTCGTAAATACCTGGGGCTTGGGGCCAAAGCGCCGCTGGCCTATACTGCGGAGCCAAAGATCGATGGTCTGTCGCTCTCTCTGCGCTATGAGAACGGGGTGCTCAAGCAGGCCGCCACCCGCGGCGATGGTTCGGTGGGGGAGAATGTCACCGCCAATGCGCGCACCATTGCCGACATTCCGCATGAACTAAAGAACGCGCCAGACCTGCTGGAGGTGCGCGGCGAAGTCTACATGAGCCATGCCGATTTCGCGGCGCTGAACGCGCGGCAGGCCGAAACGGGGGGCAAGACCTTTGCCAATCCGCGCAACGCCGCCGCCGGATCGTTGCGGCAGCTTGATGCGGAAATCACCCGTGCGCGTCCGCTTCGGTTCTTTGCCTATGCTTGGGGCGCGCTCTCGACCCCGCTGGCCGAAACGCAAAAGGGCGCGATCGACCGGTTGGCGGAACTGGGATTTTCCACCAACCCGCTGACTGCGCTTTGTGATGGTCCCAACGACATGGTGGCCCATTATGAGCAGATCGAAGCGCAGCGCGCCACGCTTGGCTATGATATCGATGGTGTGGTCTACAAGGTCGATGACCTCGCCCTACAGGAACGCCTCGGCTTTCGCTCGACAACGCCCCGCTGGGCAATCGCGCATAAATTCGCGGCTGAACTGGCTTGGACACGGCTGGAAGGCATTGACATTCAGGTTGGCCGCACCGGGGCGCTGTCCCCCGTGGCGCGGTTGCAGCCGGTCACAGTCGGCGGGGTCGTCGTCTCCAACGCGACGCTGCACAATGAGGATTACATCAAGGGGCTCGACAGTAAGGGTGCTGAGATCCGGGGTGGTAAAGACGTGCGCGTTGGCGATTGGGTGCAAATCTACCGCGCGGGCGATGTGATCCCCAAAGTGGCGGATGTTGACCTATCCAAACGCCCCGAGGATGCGGCGCCCTTCGTTTTTCCCACCACCTGCCCGGAATGCGGCTCGGATGCGATCCGTGAGCCGGGCGATGCGGTGCGGCGCTGCACAGGCGGGCTGATTTGCCCCGCGCAGGCCGTTGAAAAGCTGAAACATTTCGTCGCGCGCGGGGCCTTTGACATCGAAGGGCTCGGGGCCAAACAGGTAGAGCAATTCTACCACGACGGTTGGATCGCGGAGCCTGCGGATATTTTCACTTTGAAAGAGCGCTATGGTAGCGGCGTGCAGCAGTTGAAGAACCGCGAAGGCTGGGGGCCGAAGTCCGCAGACAACCTATTCCAAGCCATTGAAGATAAGCGCGAAATCCCGATGGCGCGGCTGATCTTTGCCCTCGGCATCCGCCATGTGGGCGAAGCGGCGTCGAACCTTATCGCATTGCATTACGGCGACTGGGACAGTTTTGAGGCCGCCATGGCCGAAGCGCGCGGGCTGGAGGGCCCCGCGTGGGATGACCTGATCGGTGTCGATGGCGTTGGCAGTGTTATGGCAGGCTCGCTGGTTTCGGCCTTCGCGCAGGAGGCCGAGCGCGCCTCCATCGACCGGCTGGTCGCGCATCTGACCGTGGTTCCAGCGGAACGACCCGATACCGAAGGCAGCCCGGTGGCGGGCAAAACGGTCGTTTTCACCGGCACCTTGGAGAAGATGAGCCGCGCCGAGGCCAAGGCACGGGCGGAGCGTTTGGGGGCCAAGGTCTCAGGATCGGTCAGCGCCAAGACTGACATTCTCGTGGCCGGTCCGGGGGCAGGCTCCAAAGCCAAGAAAGCTGCCGATTTGGGGATTGAGACTTTGGACGAGGACGGCTGGCTCGCCTTGATCGAAGGCAAATGAATGATCCACAAAAATTGGGCTGAATTGATCAAGCCGCAACAGCTGGACGTCAAGCCGGGCAATGACCCAGCCCGTCAAGCAACTGTTATGGCAGAACCGCTGGAGCGGGGCTTTGGCCTGACGCTCGGCAACGCGCTGCGCCGCGTGCTGATGTCGTCGCTCCAGGGTGCGGCAATCACATCCGTGCAGATCGACAACGTGCTGCACGAGTTTTCCTCCGTGGCCGGTGTGCGCGAAGACGTGACCGACATCATCCTGAACCTCAAAGGCGTTAGCCTGCGCATGGAAGTCGAAGGGCCCAAGCGCCTGTCGATCTCTGCGAAGGGTCCGGGCGTTGTCACTGCCGGTGACATCTCCGAATCCGCCGGTATCGAGATTCTGAACCGCGAGCATGTGATCTGCCACCTTGACGATGGTGCAGATGTCTACATGGAACTGACCGTCAACACTGGCAAGGGCTATGTCTCTGCCGACAAGAACAAGCCGGAAGATGCGCCCATCGGTCTGATCCCGATCGACGCGATTTATTCGCCGGTCAAGAAGGTCTCCTATGACGTGCAGCCCACCCGTGAGGGCCAGGTGCTGGACTATGATAAGCTGACCATGAAAGTCGAAACAGACGGTTCCATCACGCCGGATGACGCCGTAGCCTTCGCCGCGCGCATCCTGCAGGATCAGCTGGGCATCTTCGTCAACTTCGACGAGCCGGAATCGGCGTCCCGTCAGGACGACGACGATGGTCTTGAGTTCAACCCGTTGCTGCTCAAGAAAGTCGACGAGTTGGAACTGTCTGTACGTTCGGCAAACTGCCTGAAGAACGACAACATCGTCTATATCGGCGACCTGATCCAGAAGACCGAAGCCGAAATGCTGCGCACGCCGAACTTTGGCCGCAAGTCTTTGAACGAGATCAAGGAAGTGCTGTCGGGTATGGGCCTGCACCTTGGCATGGACGTCGAGGACTGGCCGCCAGAGAACATCGAAGACCTCGCCAAGAAGTTCGAGGATTCGTTCTAAATGGCAAGAGATGACAACCGTGGGGGCAACCGCCGCAACCAGCGCGACGAGACCCCGGAATTCGCTGACCGTCTGGTCGCGATCAACCGCGTGTCCAAGACCGTTAAGGGTGGTAAGCGTTTCGGCTTTGCCGCACTTGTAGTCGTGGGCGATCAAAAAGGCCGCGTCGGCTTTGGTAAGGGTAAAGCGAAAGAAGTGCCCGAGGCCATCCGCAAGGCGACTGAGCAAGCCAAGCGTCAGATGATCCGCGTGCAGCTGCGCGAAGGTCGTACGCTGCACCATGACATGCACGGCCGCCACGGCGCCGGCAAAGTCATCATGCGTACAGCACCTGAAGGTACCGGTATCATCGCCGGTGGTCCGATGCGTGCCGTATTCGAGATGCTGGGCGTAAAGGACGTTGTGTCCAAGTCGGTCGGTTCGCAGAACCCATACAACATGATCCGCGCCACTTTGGACGGTCTGAGAAAAGAGCAATCGCCCCGTTCCGTCGCGCAACGTCGCGGCAAGAAAGTGGCTGACATTCTGCCCAAGCGTGATGACAACGTAGAGTCCTCCGCACAAGTGGCTGAGGAGGCATAAATGAACGATCCTATCGCAGATATGCTGACACGCATCCGTAACTCTTCGCTGCGCGGCAAATCCACCGTATCCACACCAGCTTCCAAGCTGCGTGCATGGGTGTTGGACGTGCTGGCCGACGAAGGCTACATCCGCGGCTACGAAAAGGTGACGGGCGCCGATGGCCACCCCGCCATCGAGATCAGCCTCAAGTACTACGAAGGCGAGCCTGTTATTCGCGAATTGAAACGGGTTTCCAAGCCCGGTCGTCGCGTTTACATGGCCGTCAATGACATTCCTGTTGTCCGTCAGGGCCTCGGTGTGTCGATTGTCTCCACCTCCAAAGGTGTGATGTCGGACGCATCTGCACGCTCTGCCAATGTTGGCGGCGAAGTGCTCTGCACCGTATTCTAAATGCACGATATCCGCGCCATCCGCGACAATCCCGAGGCTTTTGATGCCGCTCTTGCCCGCCGGGGCGAAGCTGCAATGTCTGAGGCTGTGCTGTCCCTTGATGCCGCGCGCCGCGCCAAGATCGCTGCGGCTGAAACGGCAAAAGCCGAGCAAAACAAGGCCAGCAAGGAAGTCGGCGCCGCCAAGGCCAAAGGCGACGAGGCAGAGTTTGACCGTCTGCGGGCCTTGGTCAGCGACAAAAAGGCCGAAGTTGCCGCGATGAATACCGAGGCGCAGGAGTTGGATGCAAAGCTGACGGACATGCTGGCGCGCATCCCGAACAGCCCCGCCGATGATGTGCCGCAGGGCGCGAACGAAGAAGACAACGTTGAGGTGAAGGTCTGGGGCGATAAACCCAGTTTTGATTTCACCCCGGTTGAGCATTACGAGATCGCAAGCGTCAAACCCGGCATGGATTTCGAGACGGCCTCGAAAACATCGGGGGCGCGTTTTGTCATGCTGAAAGGCGGCGTGGCCCGTATTCACCGGGCATTGGCGCAATTCATGATCGACACCCATGTAGACGAGAACGGGCTGACGGAATACAACACCCCTGTTCTTGTCAACAATGCAGCAATGTACGGTACAGATAAACTGCCGAAGTTTGGCGGCGATTCATTCGATACGCAAGAAGACTATTGGCTAATCCCAACCTCGGAAGTCACGCTTACTTATAGCATTGCCGGGGAAACGCTCGAAGAACGATCACTACCCATGCGCATGACTGCCCATACGCTTTGCTTCCGCTCTGAGGCCGGGAGTGCTGGTCGTGACACGGCAGGCATGTTGCGCCAGCACCAGTTTGAGAAGGTCGAGATGGTGTCGATCACCCATCCCGATGAAAGCGACGCCGAGCAGCAGCGCATGGTCGGCTGTGCCGAAGGTATCCTTGAGAAACTCGGCGTGCCCTATCGTACCGTGATCCTCTGCACAGGTGACATGGGCTTTGGCGCGCGCCGCACCTATGACATCGAGGCTTGGGTGCCGGGGCAGAATTGTTACCGCGAGATTTCGTCGGTCTCGACCACTGGCGATTTTCAAGCGCGGCGGATGAATGCGCGGTTCAAGCCTGGGGGCGGCGGCAAGCCGCAGTTCGTGCATACGCTGAACGGTTCCGGCTTGGCTGTTGGGCGCTGCCTGATTGCGGTGCTTGAGAATGGCCAGCAGGCCGACGGGTCAGTGATACTGCCCGAGGTGCTTGCGCCATATCTAGGCGGCAAGACGACACTGACGGCGGAGGGTGTGCTCGCCTAAATGGCAATCACAGCATCCATGGTCAAGGAACTGCGCGACAGCACCGGCGCAGGCATGATGGACGCCAAGAAGGCGTTGACCGAAAGCAATGGCGACATGGAAGCCGCCGTTGACTGGCTGCGCACCAAAGGTCTGGCCAAAGCGGCCAAAAAATCCGGCCGTACAGCAGCCGAAGGTCTTGTGGCCGTTAAAGTTGAAGGCGGTCACGGTGTCGCGGTTGAAGTGAACTCCGAAACCGATTTCGTCGGCAAAAACGCCGAGTTCCAGTCCATGGTCAGCAACATCGCCGATGCAGCGCTGAAAGTTGATGATGTTGAAGCGCTGAAAGCGGCTGAGATCAATGGCAAGACCGTTGAAACCACGCTGACCGACGCGATCGCCAAAATCGGTGAGAACATGTCCCTGCGCCGTATGCAGAGCATTGATGGCGAGACCGTTGTCTCCTACGTGCACAACGCAGCTGCGCCCGGCATGGGCAAGATCGGCGTTCTGGTCGCCATGAACGGTGGTAACGAAGAGTTCGGCAAGCAGGTCGCGATGCACATCGCCGCTGTGAACCCGGCGTCGCTGTCCGAAGCTGACCTTGACCCGGCCGTTGTCGAGAAAGAAAAGCAAGTTCAGATCGACATCGCCCGTGAAAGCGGCAAGCCCGAAGCCGTGATCGAAAAGATGATCGTGGGCCGGATGCAGAAGTATATGTCCGAAGTGACATTGCTGAACCAATCCTTCGTCGTGAATCCTGACCTCTCCGTAGGCAAGGCCGCTGAAGAAGTTGGTGCAACCATCACTGGTTTTGTGCGTCTTGAAGTTGGCGAAGGCATCGAAGTCGTCAAAGAAGACTTCGCCGCCGAGGTGGCCAAAGCAGCAAAAGGCTAAATGAACCAGGAACTGACAAACAACCCGTTCAACCCGCTGACGCCGCAAAAAGCGTTTGACGAAATCAAGGTCTCTTTGGCCTCGCCAGAGCGGATCCTCAGCTGGTCCTTCGGTGAGATCAAAAAGCCGGAAACCATCAACTACCGGACGTTCAAGCCCGAGCGTGACGGCCTGTTCTGTGCGCGTATCTTTGGCCCGATCAAAGACTACGAATGTCTTTGCGGCAAATATAAGCGGATGAAGTATCGCGGCGTTGTCTGCGAGAAATGCGGTGTCGAAGTGACGCTGCAAAAGGTCCGCCGTGAGCGCATGGGCCACATCGAACTGGCGTCGCCAGTGGCGCATATCTGGTTCCTCAAGTCGCTGCCCTCGCGCATCGGCCTGATGCTGGACATGACCCTGCGCGATCTGGAGCGTGTTCTCTACTTTGAGAACTACGTGGTGATCGAGCCGGGCCTTACCGATCTTCAATACGGTCAGATGATGACCGAAGAAGAGTACATGGACGCGCAGGATGCCTATGGCATGGACGCGTTCACCGCCAATATCGGTGCCGAAGCGATCCGCGAAATGCTGGCCGCCATTGATCTGGAAGCCGAAGCCGAGACCCTGCGCGCTGATCTGAAAGAGGCGACAGGCGAGCTGAAGCCCAAGAAGATCATCAAGCGTCTGAAAGTGGTTGAGTCGTTCCTCGAGTCGGGCAACCGTCCTGAGTGGATGGTCATGACCGTGATCCCGGTGATCCCGCCAGAACTGCGCCCGCTGGTGCCGCTGGATGGTGGCCGTTTCGCGACCTCCGACCTCAACGACCTCTACCGCCGCGTGATCAACCGGAACAACCGTTTGAAGCGTCTCATTGAGCTGCGCGCGCCTGACATCATCGTTCGTAACGAAAAGCGGATGCTGCAGGAATCCGTCGACGCACTGTTCGACAACGGCCGCCGTGGCCGCGTCATCACCGGCGCCAACAAGCGCCCGCTGAAGTCGCTGTCCGACATGCTGAAAGGCAAACAGGGTCGCTTCCGTCAGAACCTTTTGGGTAAGCGCGTCGACTTCTCGGGCCGTTCGGTGATTGTGACCGGCCCGGAGCTGAAGCTGCACCAATGTGGTCTGCCGAAGAAGATGGCTTTGGAGCTTTTCAAGCCGTTCATCTACTCGCGGCTTGAGGCCAAAGGTCTGTCCTCCACCGTGAAACAGGCGAAAAAGCTGGTTGAAAAAGAGCGTCCCGAGGTTTGGGACATCCTTGATGAGGTGATCCGCGAACACCCCGTCATGCTCAACCGCGCGCCGACGCTTCACCGTCTTGGCATTCAGGCGTTCGAGCCCGTTCTGATCGAAGGTAAAGCGATCCAGCTTCACCCGCTGGTTTGCTCGGCTTTTAACGCTGACTTTGACGGTGACCAGATGGCCGTTCACGTGCCGCTCTCGCTGGAAGCCCAGCTCGAAGCGCGTGTTCTGATGATGTCCACGAACAACGTTCTGTCGCCTGCAAACGGCGCGCCGATCATCGTGCCGTCGCAGGATATGATCTTGGGTCTCTACTACACGACCCTTGAGCGCCAAGGCATGGTTGGCGAAGGCATGGTCTTCGGCTCCGTCGACGAGGTGCAGCACGCCCTCGACGCCGGCGCGGTTCACCTGCACTCGAAAATCAAGGCACGGATCAAACAGATCGATGCCGAAGGCAACGAAGTGATGATGCGCTTTGACACGACCCCCGGTCGTGTGCGTCTTGGTGCCTTGCTGCCGTTGAACGCCAAAGCGCCGTTTGACTTGGTCAACCGTCTGCTGCGTAAGAAAGAAGTGCAGCAGGTCATCGACACCGTCTACCGTTATTGCGGTCAGAAAGAGTCGGTTATTTTCTGTGACCAGATCATGACGATGGGTTTCCGTGAAGCTTTCAAAGCGGGCATCTCCTTCGGCAAAGACGACATGCTGATCCCCGACACCAAATGGCCGCTGGTTGAAGAGACCCGCGAGCAGGTGAAGGACTTTGAGCAGCAGTATATGGACGGCCTGATCACTCAGGGCGAAAAGTACAACAAAGTCGTCGATGCATGGTCGAAGTGTAACGACAAAGTCACCGAAGCGATGATGGGCTCCATCTCGGCCACCACGTACCACGAGAATGGGTCCGAAAAGGAGCCGAACTCGGTCTACATGATGGCCCACTCTGGTGCGCGTGGTTCGGTTACTCAGATGAAGCAGCTGGGCGGGATGCGCGGTCTGATGGCGAAGCCGAACGGCGACATCATTGAGACGCCGATCATCTCGAACTTTAAAGAAGGTCTGACCGTTCTTGAGTACTTCAACTCGACACACGGTGCGCGGAAAGGTCTGTCGGATACCGCTCTGAAAACAGCGAACTCCGGTTACCTGACACGCCGTCTGGTGGACGTGGCGCAGGACTGTATCGTGCGTATGCACGACTGTGGCACCGAAACCGCGATCACTGCCGAAGCGGCTGTGAACGATGGCGAAGTCGTCTCCTCGCTGGCCGAGCGTCTGTTGGGCCGTGTTGCAGCGGATGACATTCTGGCGCCAGGCACCGAGGAGGTCATCGTGCCCGCCGGTGGTCTGATCGACGAGCGGATGGCCGATGCGATTGATGCGGCGGCTGTTCAGGTTGCCCGTATCCGTAGCCCGCTGACATGTGAGGCCGAAGAAGGCGTCTGTGCCATGTGCTATGGTCGTGACCTTGCACGCGGTACGCTGGTGAACCAAGGTGAGGCCGTTGGCATCATCGCGGCCCAGTCGATTGGTGAGCCAGGTACACAGCTGACGATGCGGACGTTCCACATCGGCGGCGTTGCACAGGGTGGCCAGCAGTCCTTCCAAGAGGCCAGCCAGTCCGGTAAAATCGTGTTCGAAAACGCTCAGACGTTGGAAAACAGCTCTGGCGAGATCCTCGTCATGGGTCGGAACATGAAGCTGTCGATCGTTGACGAAAGCGGTGATGAGCGGTCCAGCCACAAAGTCGGCTACGGTACCAAGCTCTTCGTCAAGGATGGCGATACCATTGCGCGCGGCGACAAGCTGTTCGAGTGGGATCCCTATACCCTGCCGATCATCGCCGAGAAGCCAGGTATGGCCAAATACGTCGACCTTGTGTCGGGCATCGCTGTCAAAGACGACACTGATGACGCGACAGGCATGACCCAGAAGATTGTGATCGACTGGCGCGCGGCCCCCAAAGGCAACGAGCTTAAGCCTGAGATCATTCTGGTTGGTGACGATGGCGAACCGGTCCGCAACGACGCGGGCAACCCGGTGACCTATCCAATGTCCGTGGATGCCGTTCTGTCCGTTGAGGATCAGACCGAAATCCAAGCGGGTGACATCATCGCGCGTATCCCGCGTGAAGGTGCCAAGACCAAGGACATTACCGGTGGTCTGCCTCGTGTGGCCGAACTCTTTGAGGCACGTCGCCCCAAAGATCACGCCATCATCGCGGAAATCGACGGCTATGTGCGCTTCGGCAAGGACTACAAGAACAAGCGCCGCATCGCGATTGAAAGCTCGGATGATCCGGACGTGAAGGTTGAATACATGGTGCCCAAGGGGAAACACATCCCCGTTGCCGAAGGCGACTTCGTGCAGAAGGGTGACTACATCATGGACGGCAACCCCGCGCCGCACGACATTCTGGCGATTATGGGCGTCGAGGCCTTGGCCGAGTACATGATTGACGAGGTGCAGGACGTTTACCGCCTGCAGGGTGTGAAGATTAACGACAAACACATCGAAGTCATCGTCCGTCAGATGCTGCAGAAGTGGGAAATCCAAGAAAGCGGCGACACCACGCTGCTGAAGGGCGAACACGTCGACAAGCAGGAGTTCGACACCGCCAACGAGAAGGCGCTGAAGAAGGGTGGCCGTCCTGCCAAGGGCGAACCGATCCTGCTGGGCATTACCAAGGCGTCGCTTCAGACCCGCAGCTTTATCTCTGCCGCGTCCTTCCAGGAAACAACGCGCGTGCTGACCGAAGCTTCGGTTCAGGGCAAGCGGGACAAGCTGGTTGGCCTGAAAGAGAACGTCATTGTGGGCCGTTTGATCCCGGCGGGTACTGGTGGGGCAACCCAGCAGATGCGCAAAGTGGCGACAGACCGCGACAACGTTGTGATCGAAGCGCGCCGCGAAGAAGCCGAGGCGGCAGCCGCTCTGGCCGCTCCGACCGCTTCTACGGACGACGTCGTTGGTGGCGATGTGTTCAACACGCCAGTGGGTGACGACGAAAGCCGCGATTGAATGTCACGCCGCCACGCCGCTGAAAAACGCGAAGTCCTGCCAGACGCCAAATACGGCGATCTGGTTCTCACCAAATTCATGAACAACCTGATGATCGACGGCAAGAAATCTGTCGCCGAGCGCATCGTCTACAACGCGATGACCCGCGTCGAAGACAAGATCAAGCGCGCCCCGATCGAGGTGTTCCACGAAGCACTTGAAAACATCCAGCCGTCCGTCGAAGTTCGTTCGCGTCGCGTTGGTGGTGCCACCTATCAGGTGCCAGTCGAAGTGCGCCCCGAGCGCCGTCAGGCGCTGGCGATCCGCTGGTTGATCAAAGCCGCGCGCGCCCGCAACGAAAACACCATGGAAGAGCGTCTTGCAGGCGAGCTGATGGACGCCGTACAGTCCCGTGGTACTGCCGTTAAAAAGCGCGAAGATACGCACAAGATGGCCGACGCCAACAAAGCGTTCAGCCATTACCGCTGGTAAATGCCCTATGCCCATTCCGACAAATCCGAGGGGATGCCGATGCTGGCAAACCCCGCGCCAGACGTGCGCAGCCGCCCCAAGCTGGAGGGCGGCCATAAGTTCAAGCTGGTGACCGAGTTCGCTCCGGCGGGCGACCAGCCCACCGCAATCAAAGAATTGACCGAAGGGGTGAACTCGGGCGAGCGCGATCAGGTGCTTCTAGGCGCGACCGGCACGGGCAAGACTTTCACCATGGCCAAGGTGATCGAGGAAACGCAACGCCCGGCGATCATCCTTGCGCCGAACAAGACGCTGGCGGCGCAGCTTTACGGCGAATTTAAGGGCTTCTTCCCCGACAACGCGGTGGAATATTTCGTCTCATACTATGACTACTACCAGCCCGAAGCCTATGTCGCGCGGTCCGATACCTTCATCGAGAAGGAATCCCAGATCAACGAACAGATCGACCGGATGCGCCACTCCGCCACCCGCGCGCTTTTGGAGCGTGACGACGTGATTATTGTCGCCTCGGTGTCGTGCATTTATGGTATCGGTAGCGTCGAAACCTACGGCGCGATGACCCAAGATCTGAAAGCCGGTGAAAGCTACGACCAGCGCAAAGTCATCGCCGATCTGGTGGCGCAGCAGTACAAACGCAATGACGCGGCCTTCCAACGTGGTTCATTCCGGGTACGCGGCGACAGTCTCGAAATCTTCCCGGCCCACCTTGATGACCGCGCATGGCGGCTGTCCTTCTTTGGTGAAGAGTTGGAGAGCATCACCGAGTTCGACCCTCTGACGGGCGAAAAGACCGACACTTTCGACCAAATCCGCGTCTATGCGAACAGCCACTATGTGACGCCGAAGCCAACGATGTCTCAGGCGATCATCGGGATCAAGAAAGAGCTGCGCACGCGGTTGGATCAACTGGTCGCCGACGGCAAACTGCTTGAGGCGCAGCGGCTTGAGCAACGGACGAACTTCGACCTCGAGATGCTGGAGGCCACCGGCGTCTGCAACGGGATCGAAAACTACTCGCGCTACCTCACAGGCCGCGCGCCCGGGGAGCCGCCCCCCACCCTGTTCGAATTCATCCCTGACAACGCCATCGTTTTTGCGGATGAATCCCACGTTTCGGTCCCGCAGATCGGCGGCATGTACAAGGGCGACTATCGGCGCAAATTCACGCTCGCCGAACACGGCTTCCGTCTGCCGTCCTGCATGGACAACCGCCCGCTGAAGTTCGAGGAATGGGACGCGATGCGCCCGCAGTCGGTCTTTGTCTCGGCCACGCCCGCCGCGTGGGAGATCGAGCAAACCGGAGGCGTATTCACCGAACAAATCATTCGCCCGACAGGTCTGATCGACCCGCAAATTGAGATCCGCCCAGTTGAAATGCAGGTCGACGATCTGCTTGATGAGGTGCGCAAAGTCGCCGCCGATGGCTACCGGACCCTGTGTACCGTGCTAACCAAGCGGATGGCCGAAGACCTGACCGAATATATGCACGAACAGGGCATCCGCGTGCGCTACATGCACAGTGACATCGACACGATCGAGCGGATCGAAATTCTGCGCGACTTGCGTTTGGGCGCCTTTGACGTGCTGATCGGGATCAACCTATTGCGGGAGGGGCTGGACATCCCCGAATGTGGGCTGGTGGCCATTCTGGACGCGGACAAGGAGGGCTTCCTGCGCTCTGAAACCTCGCTCATTCAGACCATTGGCCGGGCCGCGCGAAACGCCGAAGGCCGCGTAATCATGTATGCCGACCGCATCACCGGCAGTATGGAACGGGCGATGGGCGAAACCGACCGCCGCCGCGCCAAGCAGCTGGCCTATAACGAAGAACACGGCATCACGCCCGCCACGGTCAAAAAGAACGTCGATGACATTCTGGCCGGCCTTTACAAAGGTGATGTCGATATGAACCGGGTCACTGCCAAGGTCGACAACCCCCTAGCGGGCGGCAACCTTCAAACTGTGCTTGATGGGCTGAGGGTCGACATGCGCAAAGCTGCCGAGAACCTTGAGTTCGAAGAAGCCGCAAGGCTGCGCGATGAGGTCAAGCGGCTGGAGGCAGTCGATCTGGCTGTGGCCGACGATCCGATGGCCCGTCAACAAGCGGTGGACCGCGCTGTCGATGCCGCTCAAAAAGCGTCGGGGCGCAGCACCTCTGGCCGAGGCGGCATGCGTGGCGGCAACGTGAAA------CGGCGCTAAATGGCTGCAAAACCATTTTTCCGTCGTCGTAAAGTGTGCCCCTTCTCGGGCGACAACGCACCTGCGATTGACTACAAGGACACACGTCTGCTGCAACGCTACATCTCTGAGCGTGGCAAGATCGTTCCTTCCCGTATCACCGCCGTATCGGCCAAGAAACAACGCGAACTGGCCCGTGCCATCAAACGCGCTCGTTTCCTCGCCCTGCTGCCCTACGCCGTTAAGTAAATGCCCAAGCGTATCCTTACAGGCACCGTGACATCGGACGCCAACGCACAAACAGTTTCCGTTTCCGTAGAGCGCCGCTTTACGCATCCGGTTCTGAAAAAGACCATCCGTAAGTCCAAGAAGTACCGGGCTCACGATGAGAACAACACATACAAAGTGGGCGACAGCGTTCGCATCATCGAGTGTGCACCAAAGTCGAAAACCAAACGTTGGGAAGTTTTGACTTCGGACAAGTGAATGGCAAACACAACACAATCCGCAAAGCGCGCACGTCAAAACGAGAAACGTTTTGCGATCAACAAAGCCCGTCGTTCGCGCATCCGCACCTACCTGCGCAAAGTCGAAGAAGCTATCACCTCCGGTGATAAAGAAGCCGCAACCGCAGCTCTGAAAGCCGCCCAGCCCGAGCTGATGCGCGGCGTCACAAAAGGCGTTTTCCACAAAAACACCGCGTCGCGCAAAATGTCGCGTCTGGCCGCACGGGTCAAAGCACTGGGTTAAATGGCTCGTTCAGTATGGAAAGGTCCTTTTGTTGACTCTTATGTCCTCAAAAAGGCAGAGGCTTCCCGCGAGGGCGGCCGTAACGAAGTGATCAAGATCTGGTCGCGCCGCAGCACGATCCTGCCCCAGTTCGTGGGTCTGACGTTTGGCGTGTACAACGGTCATAAGCACATCCCTGTTAACGTCAGCGAAGACATGATCGGTCAGAAGTTCGGTGAGTACTCCCCGACTCGGACCTACTACGGTCATGCCGCCGACAAAAAAGCGAAGCGGAAATAAGTGGCACGTATTGCCGGCGTAAACATCCCGACTGCAAAGCGGGTTCCAATCGCCCTCACCTATATCACCGGTATTGGCACCTCCTCCGCCAAAGCCATTTGCGAAGCCGTTGGCATCGACGCGACCCGTCGCGTTAACGAACTCTCCGACGCCGAAGTTCTGGCCGTGCGTGAGCACATCGACGCCAACTACACCGTCGAAGGCGACCTGCGTCGTGACACGCAGATGAACATCAAGCGTCTGATGGACCTTGGTTGCTACCGTGGCCTGCGCCATCGTCGTAACCTGCCCGTCCGTGGTCAGCGTACCCACACCAATGCTCGCACCCGCAAAGGCCCCGCAAAGGCCATTGCTGGCAAGAAGAAATAAATGGCTATGAAAATTCGTCTCGCCCGCGGCGGCTCCAAAAAGCGCCCCTTCTACCGTATCGTTGCTGCTGACAGCCGCATGCCGCGCGATGGCCGCTTCATCGAGAAGCTGGGCACATACAACCCGCTGCTGCCCAAAGACAGCGAAGACCGCGTGAAAATGGACGTTGAAAAGATCGAAGCATGGATTGCCAAGGGCGCACAGCCGACCGAGCGCGTTGTCCGCATGCTGGAAGCCGCTGGCGTTCGCGAAAAGACCGAGCGTAACAACCCCAAAAAGGGCACACCGGGCAAGAAAGCCCAAGAGCGCGTGCAGGAAAAAGCCGACAAAGCCGCTGCCGCTGCCGAAGCTGCCAACGCACCTGCCGAAGAAGCTTCCGCAGAA---TAAATGTCGATTACTGCTGAAGAAAAAGCAAAAGTCATGAAAGATTTCGGCACCAAAGAAGGCGATACAGGTTCGCCCGAAGTACAGGTTGCCATTCTGACCTCGCGCATCGTCACGCTGACCGAGCACTTCAAGACCCACAAAAAAGACAACCACGGCCGCCGTGGTCTTTTGAAAATGGTCGCGACGCGCCGCAAGCTGCTGGATTACGTTAAGGCCAAAGATGAGTCCCGTTATCAGGACCTGATCAAGCGCCTCGGCCTGCGCCGCTAAATGAAGACGCTGAATGAAATTCGCTCAACTTTTCTGAATTATTTCGATGCGCAGGGGCATCAGATCGTGCCCTCCAGCCCATTGGTGCCGCGCAATGACCCGACGTTGATGTTCACGGCTGCCGGTATGGTGCAGTTCAAGAATCTCTTTACCGGGGTCGAAACCCGCGATTATAGCCGCGCCACGTCGGCCCAGAAATGTGTGCGCGCGGGCGGGAAACATAACGATCTCGACAATGTGGGTTATACTGCACGGCACCACACGTTCTTCGAGATGTTGGGCAACTTCAGTTTCGGGGATTACTTTAAATCCGAAGCGATCCCCTTTGCCTGGGACCTGCTGACCAAGGAATTCGGCATTGACCCGAACCGCCTGCTGGTCACGGTTTACCATACGGATGAAGAAGCGGTGAAAATCTGGAAAGCCCACACCGGCCTGCCGGATGACCGCATTATCCGCATCGCCACAGACGATAATTTCTGGTCGGCTGGCCCAACCGGCCCCTGTGGGCCCTGTACTGAAATCTTCTACGACCACGGCGACCATATTTGGGGTGGCCCCCCCGGATCACCGGAGGAAGATGGTGACCGTTTTGTCGAGATCTGGAACCTTGTTTTCATGCAATACGAGCAGTTCGAGGACGGCACGCGCCAGCCGTTGCCGAACCAGTCGATTGACACCGGCATGGGGATCGAGCGGGTCGCGGCGTTGCTGCAGGGCACGAATGACAACTACGCCACCGATCTGATGCGCAGCCTGATTGAGGCCAGTGCGCATGCGTCTTCGACCGATCCCGACGGGCCGGGCAAGACCCACCACCGGGTGATTGCGGACCACCTGCGTTCGACCTCCTTCCTTATTGCCGACGGGGTTATGCCCTCGAACGAGGGCCGCGGCTATGTATTGCGCCGGATCATGCGTCGCGCCATGCGGCACGCACACTTGCTGGGCGTTAAGGACCCGTTAATGCATCAACTGGTACCGTCGCTGGTGCAGCAGATGGGTGCGGCTTACCCTGAATTGGGGCAGGCCCAATCGCTCATTCGTGAGACGTTGTTGCTGGAGGAAACCCGTTTCCGCCAAACGCTCGACCGGGGTTTGAAGCTGTTGGACGATGAGTTGAGCTCTCTCCCAGAGGGGGCGACCCTGCCGGGGGAGGCCGCGTTTAAGCTTTATGATACCTACGGGTTCCCACTTGACCTTACGCAGGACGCGTTGCGGGAAAAGGGACGTGCGGTTGACACCGATGGGTTCGACACTGCGATGCAGGCACAAAAGGCCAAAGCCCGCGCCGCTTGGGCCGGGTCGGGCGAAGCGGCGGATGCGACTGTCTGGTTTGACGTAGCTGACAAGAGCGGCACCACTGAATTCTTGGGCTATGACACTGAAAGCGCGGAAGGTCAGATCGTTGCCTTGGTACAGGGCAGTGACAAAGTCGATTCTGCAGCCGTTGGCAGCGACGTGCAAGTCGCGCTGAACCAGACACCTTTCTACGCTGAAAGCGGCGGGCAGGTTGGCGATACTGGCGTGATCCGCACCCAAAGCGGCATCGTCAATGTTACGGACACGCGTAAATCTGCGGGCGTTTTCGTGCATTTCGGCCATGTCGTCGAAGGCGAAGTGAAACCCGGCCAGACGGCGGTTCTTAATGTCGACCCGGCGCGGCGTACCGCGATCCGTGCCAATCACTCGGCCACGCACCTGTTGCATGAGGCGTTGCGCAACGCACTTGGAGACCATGTTTCGCAGCGTGGATCGTTGAATGCGCACGACCGGCTGCGGTTCGATTTTTCGCATGCCAAGGGCTTAACGCAGGAAGAGCTGAGCCAGGTCGAGCGCGAGGTGAATGATTATATCCGCCAAAACACGCCGGTTGAGACGCGGATCATGACCCCGGATGATGCCCGTCGCATGGGCGCACAGGCGCTTTTCGGTGAGAAATACGGCGATGAAGTGCGGGTTGTCTCAATGGGACAGCTTGAAGGCTCGGGTAAGGGCAGCGATAAATCAACCTATTCGCTTGAGCTTTGCGGCGGCACCCATGTGCGGCAGACAGGCGATATCGGCGCTTTTGTTCTACTGGGCGATAGTGCCAGCAGTGCGGGCGTCCGCCGGATTGAAGCGCTGACTGGTACAGAAGCATTGGCTTGGTTGCGTGAGCAAGAGGCCGCCTTGAGCCGCGTCGCAGCGGAGTTGAAAACCTCGACAAGCGATGTGCCTGATCGTGTCCGCGCGCTTCTTGACGAACGCCGCAGCCTCTCCAACGAAGTGGCCCAGTTGCGCCGCGAATTGGCGATGTCT---GGTGGCGGCGCGGCAGCACCCGAAGCACGCGAAGTGAATGGTGTGAGGTTCGTCGGCCAAGTGTTGAGCGGCGTGACGGGCAAAGACTTGCCGGGTCTGGTGGACGAACATAAGGCCAAACTGGGTTCAGGCGCGGTGCTGTTGATTGCCGATACGGGCGGCAAAGCAGCGGTTGCAGGCGGCGTTACCAAAGATTTGACAGATCGCCTTTCGGCGGTCGATATGGTGAAGGCCGCGGTGGCTGAACTGGGCGGTAAAGGCGGCGGTGGCCGTCCTGATATGGCACAGGGCGGCGGTGCTTCGGCAGAGAATGCAGAGGCCGCGATCGCTGCCGCTGAAAATATCTTGAAAGGATAAATGGGTTTCAAAATGGGCATCGTCGGTCTGCCGAACGTCGGCAAATCGACCCTTTTCAACGCGCTGACCCGCACCGCAGCGGCACAGGCGGCGAATTTTCCGTTCTGCACGATCGAGCCCAATGTCGGCGAAGTGGCCGTGCCCGACGCGCGGCTCGACACGCTGGCCGAAATCGCCAAGTCGAAAAGCATCATCCCTACGCGCATGACCTTTGTCGATATCGCTGGTCTGGTGAAGGGCGCGTCGAAAGGCGAAGGCTTGGGCAATCAGTTCCTCGCCAACATCCGCGAAGTCGACGCTATCGCCCATGTGCTGCGCTGCTTTGAAGACGGCGATGTCACCCATGTCGAAGGCCGCGTGGACCCGGTGGCGGATGCCGAAACCATCGACACCGAACTGATGCTGGCCGACATTGAAAGCATCGAGAAACGCTTGCAAAACATCGTGCGTAAAGTGCGCGGCGGCGACAAGGAAGCGGTGCAGCAGGAACGTCTGATGCGCATGGCGCTTGAGGCATTGGAGGCTGGCAACCCCGCGCGCGTGGTTGAGGTAGACGAAGATGACGCCAAAGCATGGCGGATGCTGCAACTGCTGACCACGAAACCGGTGCTCTACGTCTGCAACGTAGGCGAGGCGGAAGCCGCTGAAGGCAACGCGCATTCCGCAAAAGTGGCCGAAATGGCCGCAGCGCAAGGCAATTCGCATGTCGTAATTTCGGCCCAAATCGAAGAAGAAATCAGCCAGCTAGAGGCCGAAGAGGCCGAGATGTTCCTCGAAGAGATGGGGCTGAAAGAGGCCGGTCTCGATCGTTTGATCCGTGCTGGCTATGAGCTTTTGCATCTTGAGACCTACTTCACCGTCGGCCCCAAGGAAGCGCGCGCTTGGACGATCAAATCCGGCACCTCTGCTCCGAAAGCCGCTGGTGTGATTCACGGCGATTTCGAAAAGGGATTCATCCGGGCCGAAACCATCGCCTATGATGATTTTGTATGCCTTGGCGGCGAAGGCCCAGCCAAGGAGGCGGGCAAGATGCGTGCTGAAGGTAAGAGCTATATCGTCAAGGATGGCGACGTGCTGCACTTCCTTTTCAACACGTAAATGGGCTGGAAATCACTGGACGACATGGACCTGAACGCCAAGCGCGTACTGCTGCGGGTCGACATCAACGTACCCGTAGAGGATGGCCGCGTGACCGATGCCACGCGGATTGAGCGGATCGTGCCCACAGTGAACGACATTCTCTCGCGCGGTGGCAAGGTGACACTGCTGGCGCATTTCGGGCGACCTAAGGGCAAGGTTGTCGAGGAGATGAGCCTAAAACAGGTCCTTCCCGCACTGGAAAACGCTTTGGGTCGTGACGTGGCCTTTGTCCCGTCGCTTGAGGCCGCAGCT---------------GGGGCGCAGGATGATCTGCAACTCATGGAAAACATCCGGTTCTACCCAGGTGAAGAAGCGAATGATGCAGAGTTCGCCCAACGCCTAGCTGATCTGGGTGATGTCTATTGCAACGACGCCTTCTCGGCTGCGCACCGCGCCCATGCATCGACGGAAGCGCTGGCGCGGCTGCTGCCCGCCTGCGCCGGCCGTTTGATGCAAGCTGAACTTTCGGCGCTAGAGGCGGCTTTGGCCAAGCCAGAGCGCCCGGTGGGTGCCGTTGTTGGCGGGGCCAAGGTCTCGACCAAGATCGCGTTGTTGGAAAACCTCGTGAACCGATTGGATGTGCTGGTGATCGGCGGCGGTATGGCGAACACCTTCCTCGCCGCACTTGGCGCAGATTTGGGCAAATCCTTGGAAGAGCCTGACTATTACAGCACCGCCAAAGACATCATGGCGCAGGCGGACAAGGCGGGTTGCCGGGTGATCTTGCCCGTTGACGGTTTGGTCGCGCGCGACTTTGCCAAAGGTGCCGCGCATGAGGTGGCCCAATTGGGCCCCGACGCGAAATTGGCCGCCGATCAAATGGTGCTGGATGCGGGCCCCGATACTGTCGCCCTTGTCGAGGCCGCTTTTGCCGGGCTGCGCACGCTGATCTGGAACGGGCCAATGGGGGCCTTTGAGATCCCGCCGTTCGACACTGCCACTGTTGCTGCCGCCCGCGCGGCGGCCCAGCAAACCCGCGATGGCACGCTGACCTCGGTTGCGGGTGGTGGTGACACGGTTGCGGCACTTAACCAAGCGGGTGTGGCGGATGACTTTACCTATATCTCCACTGCGGGGGGCGCGTTCCTGGAATGGATGGAAGGCAAAACCCTGCCCGGCGTCGCGGCATTGGGCGGATAAATGGCTCTTCCTGAGTTCTCCATGCGCCAATTGCTTGAAGCAGGCGTACACTTTGGTCACCAGACACAGCGCTGGAACCCCCGCATGGGCCCGTACATCTACGGCGCACGCAACGGCATCCACATCATGGACCTCACGCAAACCGTTCCCATGCTGGACGATGCGCTGAAAGTGATCCGTGACACCGTCGCCAAAGGCGGCAGCGTTCTCTTCGTCGGCACCAAGCGTCAGGCTGCCCAGCCGATCGCCGAAGCCGCAGAGAAATGCGCACAGTATTACATGAACCACCGTTGGCTCGGCGGTACGCTGACCAACTGGCAGACCGTTTCGCAGTCGATCAACCGTCTGAAGAGCATCGACGAGCAGTCCGAGCGTGGCTTTGAAGGCCTGACCAAGAAAGAGCGTCTTGGCATGGAACGCGACCAGTTCAAACTGGAAGCATCGCTGGGCGGTATCCGCGAAATGGGCGGTCGTCCTGACCTTCTGTTCGTCATCGACGTGAAAAAAGAAGCATTGGCGATTGCCGAAGCCAACAAACTGGGCATCCCAGTTGTGGCCGTGGTTGACACCAACTGCTCGCCCGACGGCATCGACTACATCATTCCCGGCAACGACGACGCGGCCCGCGCCATCTCGCTTTACTGCGATCTGGCAGCCCGTGCGGCACTTGACGGCATGTCCGCTCAGCTGGGTGCGGCAGGCGTTGACCTTGGCGCCATGGAAGAAGCACCCGAAGAAGAAGCCATGACCGCAGAAAGCAACGCTTCGGAAGAAACTCTGCATGACGATGCCATGGGCAAAGACGCGGAATCCTAAGTGACCAAACGCACAGCTGCCAAGCACAAACTAGACCGCCGCATGGGCGAAAACATCTGGGGCCGTCCGAAGTCCCCGGTGAACCGTCGTGAATACGGCCCCGGCCAGCACGGTCAGCGCCGTAAAGGCAAGATTTCCGATTTCGGTATTCAGCTGCGCGCCAAGCAGAAGCTCAAGGGCTACTACGGCGACCTGACCGAAAAGCAGTTCCGCCGCATCTACGGCGAAGCCGAGCGTGTTAAAGGCGATACAGGTGAAAACCTGATCGGTCTGCTGGAGCGTCGTCTGGACGCCGTTGTGTACCGCGCCAAGTTCGTTGCGACCGTTTTCGCTGCGCGCCAGTTCGTAAACCACGGCCACGTTCGTGTGAACGGCAAGAAAGTGAACATCCCCTCCTACCGCGTGAAAGAGGGTGACGTCATCGAAGTGCGTGACCGTTCCAAGCAGTTGGCTTCCGTTCTGGAAGCGGTTCAGCTGCCCGAGCGTGACGTGCCTGACTACCTTGAGACGGATCACTCCAAGCTGACAGCAACCTTCGTGCGCACACCCGGCCTGTCCGACGTGCCATACCCGGTTGTGATGGAGCCTAACCTCGTCGTGGAATTCTACGCGAAGAACTAAATGGGTAACAAAGTAAATCCGATCGGTATGCGTCTTCAGGTGAACCGCACCTGGGACAGCCGCTGGTACGCCGACACCAAGGATTACGGTGATCTTCTTCTCGAAGACCTCGCAATCCGCGACTTCATCAAGAAAGAGTGCCACCAAGCTGGTGTTGCCCGTGTGATCATCGAGCGTCCGCACAAAAAGTGCCGCGTCACGATCCACACAGCACGCCCCGGTGTCATCATTGGCAAGAAAGGCGCGGACATCGAGACGCTGCGCCAGAAGATCGCCAAGATGACCAACTCGGAACTGCACCTCAACATCGTTGAGATCCGCAAGCCCGAGCTAGACGCACATCTGGTTGGTGAGAGCATTGCACAGCAGCTGGAGCGCCGGGTTTCTTTCCGCCGCGCCATGAAACGTGCCGTGCAGAACGCCATGCGCATGGGCGCACTGGGCATCCGCGTGAACCTCGCGGGTCGTCTTGGTGGTGCTGAAATCGCGCGTACCGAATGGTACCGTGAGGGTCGCGTGCCTCTGCACACATTGCGTGCCGACATCGATTACGCACATGTCGAAGCGGCCACCGCTTACGGCATCATCGGGATCAAGACATGGATCTTCAAAGGCGAGATCATGGAACATGACCCCGCCGCGCGTGACCGTAAGGCACAGGAACTCCAAGACGGCCCAGCACCTCGCGGTGCCGGCGGTCGTCGCTAAATGGCACGCAAACGCAAGGGTCGCGATATTTCAGGTTGGCTGGTTGTAGACAAACCCGCCGGGCCGACATCGACCGCCGTGGTCAACAAGGTTCGCTGGGCGCTCGAAGCCAAGAAGGCGGGCCACGCAGGCACCCTTGACCCCGAAGCGACCGGTGTTCTGGCCATCGCCTTGGGCGAAGCGACCAAGACCGTGCCCTATATCACCGACGCGCTCAAAGCCTATGAGTTCACCGTGCGTCTGGGCATTGCCACCAACACCGACGATGCCGAAGGCGAGGTCATCGGCACCTCCGATTTGCGCCCCGACGATGCAGCCATCAAGGACGCGCTGAGCGATTTCATCGGGGACATCCAGCAGGTGCCGCCGCAGTTCTCTGCCGTGAAGATCGACGGCCAGCGCGCCTATAAACGCGCCCGCGATGGCGAAGAGATGGACATCGCCGCCCGCCCGCTTTGGGTAGAAAGCCTGCTGCTGCTCGACCGGCCCGACGCCGATCACGTCACGCTTGAAATGGTCTGCGGCAAGGGCGGCTATGTCCGCTCCATTGCCCGCGATCTTGGGCAGAAGCTTGGTTGCCTCGGTCATGTCCGCGAACTGCGCCGCACGTGGTCCGGCCCGTTTGAGGCGGCGAATGCCCTGACACTGGCACAGATCGACGAGATCGCACGAACGCCAGAACTCGACACGCATCTGCTGCCGCTGGCCGAAGGATTGGTTGAGTTGCCGGAGGTCAAAGCCACCCCCGAAGGCGCGACGCGTCTGCGCAACGGCAACCCCGGCATGGTGATCGCCCATGACGTGGAATACGGCGATGAATGCTGGGCCTCCCTCGACGGCCGGCCCGTGGCCGTGGGCCGCTTCAAGGCGGGCGAATTGCACCCCAGCCGGGTGTTTAACCTATCCTCGTGAATGGTTCTCCTCGGGCTGACCGGGTCGATTGGCATGGGCAAGTCGACCACGGCACAGATGTTTGCCGAGCAAGGCTGCGCCGTCTGGGATGCCGATGCAGCGGTACACCGGCTCTATGCCGCCGGTGGTGCGGCGGTCGCGCCGATGCAGGCGGCGTTTCCGACAGCGATTGAACATGGCGCGGTCTCTCGCGTCGCGTTGAAAGAGATCATCGCCGCCGACCCCACCGCCCTGCCCCGGATTGAGGCCATCGTTCATCCGCTTGTCGCGCAAGATCGCGCCGCGTTCCTTTCCAGTGCAACCAATGACATCGCCGTGCTCGACATTCCGCTGCTGTTTGAGACTGGCGGCAACGCTGCGATGGACGCGGTGGTCTGCGTCACCATCCCCGACGCCGTCCAACGCGACCGCGTGCTTGTCCGTGGCACCATGACCGAGGCGCAATTCGATGCCATCCGCGCCAAGCAGATGCCCGCCAAAGAGAAGTGCGCCCGTTCAGACTATGTAATAGTGACCGACACGCTCGACCATGCCCGCGCGCAGGTGCGGGATGTGATCCGCGACATTAGGGAAAAGCTACGCCATGCGTGAATGGAAGATTTCGACCTCGTGATTGAGGACGACCGCTGGAACGCGGTCGACCTTGAACCGCTCGCTCATGCGGCGGCACGCGCCACTCTGGGGCATCTCGGCCTCAACGCCGAGGCGGCAGAGATGACCCTGCTGGCCTGCGATGACGCCCGCATTGCTGTACTGAACGAAGACTTTCGCGGCAAAGCGCGCGCCACTAATGTGCTGAGCTGGCCCGCAGAAGAACGGGGTGCCGCCGCACCGGGCGGCGATCCGCTGCCCGTCTCCCCCGGCATTGACGGAATGCTCGAACTGGGCGACATCGCTTTGGCCTATGAGACCTGTGCCGCCGAAGCTAAAGCGGCAGACAAGCCTCTGGCCGCGCATGTGACCCATCTCATCGTACACGGGCTGTTGCATCTTCTGGGCTATGACCATGAAAATGACCCCGACGCGACGTTGATGGAAGGGTTAGAGCGCGAAATACTTGGCAAAATGGGTTATGATGACCCATATAGGGAAAATGGGCCTTAAATGGCACGCTATATTTTCATCACCGGCGGTGTGGTCTCGTCTCTGGGCAAGGGGCTGGCCTCGGCAGCCTTGGGCGCTTTGCTTCAGGCACGCGGCTTTTCGGTACGTCTGCGCAAGCTGGACCCTTATCTGAACGTCGATCCCGGCACGATGTCGCCCTTTGAGCATGGCGAAGTTTTCGTCACAGACGATGGCGCGGAAACCGACCTTGATCTAGGCCACTACGAACGCTTCACCGGCGTTGCCGCACGCAAGACCGACAGCATCAGTTCGGGCCGGGTCTACTCCACCGTGCTAGAGAAGGAACGCCGCGGCGATTACCTAGGGAAAACCATTCAGGTCATTCCGCATGTGACCAACGAAATCAAAGACTTCATCGACATTGGTGATGACGAGGTCGATTTCATGCTTTGTGAAATCGGCGGCACCGTGGGCGATATCGAAGGTCTGCCCTTTTTCGAAGCGATCCGTCAGTTCAGCCAAGACAAGCCGCGTGGGCAGTGTATCTTTATGCACCTCACGTTGCTGCCCTTCGTTAAGGCCAGCGGCGAGTTGAAAACCAAGCCGACGCAGCACTCGGTCAAGGAACTGCGCTCTATCGGTATTGCGCCCGACATTCTGGTTTGCCGTTCCGAAGGGCCGATCCCCGCGAAAGAGCGCGAGAAGCTGGCGCTGTTCTGCAATGTACGCCCCGATAGCGTAATTGCTGCGCAGGACCTGAAATCAATCTACGAAGCGCCGCTTGCCTATCACCGCGAGGGGTTGGATCAAGCGGTGCTGGATGCCTTCCAGATCACCCCGGCCCCCAGGCCAAACCTGTCACGTTGGGAAGATGTGGCCGACCGTATCTACAACCCTGAAGGCGAAGTGAAGGTGGCCATCGTTGGCAAATACACCCAACTTGAAGACGCCTATAAATCCATTGCCGAGGCGCTGACCCATGGCGGCATGGCGAACCGGGTCAAGGTAAAGGTCGAATGGGTCGACGCCGAGATTTTCGACAGCGAAGACGCCGGTCCCCATCTCGAGGGTTTCCACGCGATCCTCGTCCCCGGCGGCTTTGGCGAACGCGGCACCGAAGGCAAAATCAAAGCGGCGAAATATGCGCGTGAACACAAGGTGCCCTACCTTGGCATTTGTCTTGGTATGCAAATGGCGGTGATCGAAGCCGCGCGGAATGTCGCGGGCCTGAAAACCGCAGGCTCCGAAGAATTTGACCATGAAGCCGGGAAAAAGCGTTTCGAGCCGGTGGTTTATCACCTGAAAGAATGGGTGCAAGGCAACCATAAAGTCGCGCGTAAGGTGGACGACGATAAGGGCGGCACCATGCGTCTGGGTGCCTATGACGCGACGCTGGTCGAAGGGTCGCGCGTGGCCGAAGCCTATGGCACCACTACGATCGATGAACGTCACCGTCACCGTTATGAGGTCGACATCGCCTATAAGGAACAGCTTGAGAAGGCTGGGCTGAAATTCTCGGGCATGTCGCCGGATGGCAAACTGCCAGAGATCGTAGAATGGTCGGATCACCCGTGGTTCATCGGGGTGCAGTTCCACCCCGAGCTGAAGTCAAAGCCGTTTGATCCGCATCCCTTGTTCAAGGATTTCGTGCGCGCCGCGAAGGATGTGTCGCGGTTGGTATAACAATCCCAGACACGCCTGATCCTTGGTTTGGAAAGCAGCTGCGACGATACGGCAGCAGCCGTAGTGCGTATTGAAGAAGACGGACGCGGCACGGTTCTGGCGTCGGTTGTTGCGGGCCAGACCGAATTGCACGCCGATTTTGGCGGTGTCGTTCCTGAAATCGCCGCCCGCGCCCATGCAGAGAAATTAGATCACTGTGTAGAGGATGCACTGGCCGGGGCCGGGATCACCCTGCCGCAGATCGACGCCATCGCCGTTACTGCGGGGCCGGGGTTGATCGGCGGTGTGGTCTCGGGCGTGATGTGCGCCAAGGGACTGTCAGCGGCGACGGGCAAGCCGCTTTACGGGGTCAACCATCTGGCCGGCCACGCGCTGACGCCGCGACTGACTGATGATGTGCCCTATCCCTATTTGATGCTACTGGTCTCGGGCGGTCATTGCCAGTTCCTGCTGGTCCGCGGGCCTGAGAGTTTCGATCGCCTCGGCGGCACAATTGATGATGCGCCGGGCGAGGCATTTGACAAGGTCGCGCGGCTATTGGGCCTGCCGCAGCCCGGTGGACCTTCGATCGAGAAATGCGCGCAGGAGGGCGATGCCAAACGCTTCGCCCTGCCGCGTCCGCTGTTGGATCGTGAAGGGTGCGACATGTCCTTTTCGGGTCTGAAAACCGCTGTGCTGCGCGCCCGTGACAAATGTGTGGCCGCCCATGGCGGGCTGACGCGGCAAGATCAGGCGGATTTAGCGGCAGGGTTCCAAGCCGCCGTGGTTGAGGTGTTGGCCCATAAGACGCGCCGCGCCTTTGCAGAGTATCCGGCAGAGGGCGCG------CGGGGTCTGTGCGTAGCGGGGGGTGTCGCTGCTAATCAGTCAATTCGTGCAGCGTTAGAAACTGTTGCTGCCGAGCAAGAGGCACGTTTTGTCGCTCCGCCGCTGGCGCTTTGCACTGATAACGCCGCGATGATCGCTTTCGCAGCCGGAGAGCAAGCGGTACTGCGCGCCCCCGATGATCTGACCCTTTCGGCCCGCCCGCGTTGGCCATTAGACACTGCGCGGCCGTCGATGTTGGGCAGCGGCAAGAAGGGAGCCAAAGCGTGA---------------------------------------------------------------------------------------------------------------------------------------------------------------------------------------------------------------------------------------------------------------------------------------------------------------------------------------------------------------------------------------------------------------

>'Pelagimonas-variansDSM-23678'

ATGAACGCCCAAGAACTTATGGACAAGACACCGGATCAACTCCGGGAAGATCTCGTTCAGCTCAAAAAGGAGGCCTTCAACCTCCGCTTCCAAGCCGCATCTGGCGCGCTGGAAAACACTGCTCGCATGCGCGAAGTTCGCCGCAGCGTTGCCCGTGTAAACACCATTTTGAATCAAAAAGCGGCAGCTGCCGCAGCGGAGGCGTAAATGGCAACGGCAGATCTCCTCACAATG---AACAAAAAGACCGCGGACAAGCAAAAGGCGCTCGATAGCGCTCTGGCCCAGATCGAACGTCAATTCGGCAAAGGGTCAATCATGAAGCTGGGTGGCGAAAACGCCATTCGGGACATCGAAGCGACATCAACGGGCTCTCTTGGATTGGATATCGCCCTCGGTATTGGCGGTTTGCCCAAAGGCCGGATCATTGAAATCTATGGCCCTGAAAGCTCCGGTAAAACCACGCTGACCTTGCATGTGGTTGCAGAGGAACAAAAAAAGGGCGGTGTTTGCGCCTTTGTGGACGCCGAGCACGCGCTTGATCCGCAATATGCCAAGAAATTGGGCGTCGATCTGGATGAATTGCTGATTTCGCAACCCGATACTGGGGAACAAGCGCTTGAGATTGTGGACACGCTGGTCCGCTCCGGGGCAGTCAACCTTGTTGTGGTCGATTCGGTTGCGGCTTTGACACCAAAGTCCGAACTTGAGGGCGACATGGGTGACAGCAGTGTCGGCGTTCAGGCTCGCTTGATGAGCCAGGCGATGCGCAAACTAACCGGATCAATCAGCCGTTCGAATTGTATGGTTGTTTTCATCAACCAGATCAGGATGAAGATCGGCGTGATGTTTGGCTCGCCCGAAACCACAACTGGTGGCAACGCGTTAAAGTTTTACAGCTCGGTGCGATTGGACATTCGTCGGATTGGCGCGATCAAGGATCGTGACGAAGTTGTAGGTAACCAAACCCGCGTCAAAGTGGTTAAGAACAAAGTCGCACCACCGTTTAAAGTGGTTGAATTCGACATCATGTATGGCGAAGGCATCTCCAAAATGGGTGAATTGCTGGATCTTGGGGTTAAGGCCGGTGTGGTTGAAAAATCCGGGGCTTGGTATTCCTACGGGGATGAGCGGATCGGGCAGGGGCGTGAAAATGCCAAAAACTATCTGCGAGAGAATAGCCGCATCTCTTATGAGATTGAGGACAAGATTCGCGCGGCGCACGGGCTTGAATTTGAT---------------GATCCTGATTTGGTCGAAGACTGAATGTCGCTGTACGACCCCGCCAAAACCGAACCAAAATGGCAAGCCGCCTGGGATGAGGCTGGCACATTCAAAGCCACAATGACGGGCGACAAGCCCAAATATTATGTGCTGGAAATGTTCCCGTATCCATCAGGGCGCATCCATATTGGCCATGTGCGCAACTACACGATGGGCGACGTGATCGCGCGCTACAAGCTGGCGACGGGTCATAATGTATTGCACCCCATGGGGTTTGACGCCTTTGGTATGCCTGCGGAAAACGCTGCGATGGCCATCGGCGGGCATCCCAAGACCTGGACCTATGAGAACATCGACACGATGGTCGGCCAGATGAAGCCTCTGGGCTTTGGCCTTGATTGGTCGCGTATGTTCGCAACCTGCGATCCTGAATATTATGGTCAGCAACAGGCGCTGTTCATCGATTTTCTGGCCAAAGACTTGGTTTATCGCAAAAATGCGGTTGTGAACTGGGATCCGGTGGACATGACCGTGTTGGCCAACGAGCAGGTCATTGACGGCAAGGGCTGGCGGTCTGGTGCCGAAGTCGAGCGGCGCGAGCTGACGCAGTGGTTCTTCAAGATCTCGGATTATTCCGAAGAGCTTCTGGATGCCTTGGACACGCTGGAAAACTGGCCGGCCAAGGTCCGTTTGATGCAGGAAAACTGGATCGGTAAATCGCGCGGTCTGGAGTTCGGTTTCGAGCGTGTGGAC---------GGCGAAGAGCCGATCACCGTTTATACAACGCGCCCGGATACGCTGATGGGCGCGTCCTTTGTTGGTATTTCGCCAGATCATCCGATTGCCAAGAAACTGGAAGCGGAAAACCCTGAAATCGCAGCAGAAATTGCTGAGATGCGCAAAGGCGGCACCACGGCCGAGGCTTTGGAAAAGGCTGAAAAGCGCGGCGTTGATACCGGTATCCGTACCAAGCATCCGCTGGATTCCAATTGGGAACTGCCGGTCTGGATCGCCAACTTTATCTTGATGGACTATGGCACGGGCGCGATCTTTGGCTGCCCTGCGCATGACCAGCGCGACTATGAATTCGCATCAAAGTATGAACTGCCGATCGTTCCGGTGTTT------------------GAGGAAGAAATTACCGAAGCCTTCGTTCCCGCGAAAACCGAAAAGGTCACCTTCTTGCGCGGCTTTGCCGGCGCGACCGAACAGACCGGTGACGAAGCCGTGGATGCGGCCATTGCTGCCGCCGAAAGAGACGGGTGGGGCAGCGGTGTAACCAAATTCCGCCTGCGCGATTGGGGCCTGTCCCGCCAGCGTTATTGGGGCTGCCCGATTCCGGTTGTGCATTGCGACGATTGCGGTGTGGTGCCTGAAAAGAAAGAAAACCTGCCGATTGAATTGCCCGATGATGTGACATTGGACATTCCGGGTAATCCGCTGGACCGCCATCCGACGTGGCGCGATTGTGCCTGCCCGTCTTGCGGCAAGCCCGCGCAGCGCGAAACCGATACAATGGATACCTTTGTTGACAGCTCTTGGTATTTCGCCCGTTTCACTGCGCCGGACGCCAAGACGCCAACCGATATGGAAGCCGCGTCCTATTGGATGAACGTCGATCAGTATATCGGTGGGATCGAGCACGCGATTTTACACCTGCTGTATTCGCGGTTCTTTGCGCGCGCGATGAACATCACCGGCCATTTGCCCGATTCCGCGCGTGAACCGTTTCACGCACTGTTCACCCAAGGCATGGTGACCCACGCCATTTACATGACGACAGGCGAAGATGGCCGTCCGGTTTATCATTATCCAGAGGATGTTCGCGATGGCGGCGGTTTCAAGGATGGCACTCAGGTCAAAATCATCCCGTCTGCCAAGATGTCGAAATCCAAAAACAACGTTGTTGATCCGGTCGAGATCATCAAAGCCTTTGGCGCTGACACTGCGCGTTGGTTTGTCCTTAGCGATTCTCCCCCAGAGCGTGATGTCGAATGGACTGCGTCAGGCGCGGAAGCGGCCAGCAAACACCTGAATCGCGTTTGGATGCTGTGCGACAAGATCGGCAAAATGCCTGCCGATTATGCAGGCCGCGACAACGAAGAGCTGATGCGCGAAATGCACAAGGCCATTCACGATGTGACCATGGCCATCGACAGCTTTGGCTTTAATGCGGCCATTGCAAAACTGTACGGCTTTGCATCGACGATTGCGAAATCCGACGCCGGAACCAACGAGAAGAAGCTCGCGATCAAGACATTGGCGCAGTTGATGTCTCCGATGACGCCGCATTTGGCTGAAGATATCTGGGCGCATCAGGGCGGCGAAGGCTTGATCGCAAATGCGCCGTGGCCGGTTGCTGACGAAAAACTGATGGTCGAAGACAGTGTGACCCTGCCTATCCAGATCAACGGCAAGCGCCGCGGCGAAATGAAGGTGCCGCGCGATATGCCCAAAGACGAGGTTGAAAAAGCGGCGCTTGCACATGAAGCTGTGGTAAAGGCCCTTGATGGGGGCCAGCCGAAAAAGCTGATCGTAGTGCCGGGGCGGATTGTGAATGTGGTTGTCTAAATGGCAATCACATCTGCCAACCAGCTGGAACTACTGCAGACCGCCGAAGCGGTTGCGCGCGAAAAGATGATCGACCCCGGTCTGGTTATCGAAGCGATGGAAGAATCGCTCGCTCGGGCGGCAAAGTCGCGTTACGGCGCAGAAATGGACATTCGCGTGTCTATCGACCGCAAGAACGGTAAAGCCACCTTTACCCGTGTCCGTACCGTGGTCGCGGAAGAAGATCTGGAAAACTACCAGTCTGAATTCACCGTTGAGCAAGCCAAACAATACATGGCTGAACCAACAATCGGCGACACCTATGTCGAAGAAGTGCCCCCAGTGGAACTTGGCCGTATCGCGGCGCAATCGGCAAAGCAGGTTATCTTGCAAAAGGTGCGTGAAGCCGAACGTGATCGTCAGTTCGAAGAATTCCAGGATCGCAACGGCACCATTATCAATGGCTCTGTCAAACGCGAAGAATATGGCAACGTCATCGTTGATGTTGGTCGCGGCGAAGGCATGTTGCGCCGCAACGAAAAAATCGGCCGCGAAAGCTATCGCCCCGGTGATCGTATCCGCTGCTTCATCAAGGACGTTCGCCGCGAGACCCGTGGCCCGCAGATCTTCCTGAGCCGCACAGCGCCTGAATTCATGGCCGAGTTGTTCAAAATGGAAGTGCCAGAAATCTATGATGGCATTATCGAAATCAAAGCTGTAGCCCGTGACCCCGGTTCGCGTGCAAAGATCGCTGTGATCTCTCATGATGGCGGCATTGATCCTGTCGGGGCCTGCGTTGGTATGCGCGGCAGCCGTGTGCAGGCTGTTGTGAACGAACTGCAAGGCGAAAAAATCGACATCATTCCATGGAATGAAGATGTTCCGACCTTCCTTGTGAACGCGCTGCAGCCCGCAGAAGTCACCAAAGTGGTTCTGGATGAAGAAGCCGAGCGTATCGAAGTCGTGGTTCCGGATGAACAGCTGTCGCTGGCCATCGGCCGCCGTGGACAAAACGTGCGTCTGGCCTCTCAGCTGACGGGTCTTGATATCGACATCATGACCGAAGAAGAAGAATCCAAGCGCCGTCAGGCAGAATTCGAAACACGCACCAAGCTGTTCATCGAAACGCTGGATGTCGACGAATTCTTTGCTCAATTGCTGGTTTCCGAAGGCTTCACCAATCTCGAAGAAGTTGCCTACGTTGAAAAAGACGAACTGCTGGTGATTGACGGTGTTGACGACGACACCGCAAACGAGCTGCAGACCCGTGCACGTGAGTTCTTGGAAGCACAAGCAAAGAAAGCCCTGGAGCGGGCACAAGAATTGGGCGTTCAGGACAACCTGATCGGTTTCGAAGGTCTAACGCCACAAATGGTCGAAGCTTTGGCTGAAGATGGTGTTTTGTCTCTCGAAGATTTCGCCACTTGTGCGGATTGGGAACTGGCCGGTGGCTGGACAACCGAAGATGGCAAGCGCGTCAAAGACGACGGTCTGCTTGAGAAATTTGATCTAAGCTTGGAAGACGCACAGACTATGGTCATGACCGCGCGCGTTCTGCTGGGTTGGGTTGACCCAACCGAGCTGGAACCCGAC---------------GCCGACGGTGACGAAGGCGAATTTGAAAGCGAAGAGGACTAAATGGCACATAAAAAAGCTGGCGGTTCCTCCCGCAACGGTCGCGACTCAGCTGGTCGCCGTCTTGGTGTGAAATTGTACGGTGGCCAAGCGGCTATTCCGGGCAATATCGTCGTGCGTCAGCGCGGAACCAAGTTTTGGCCGGGCGAAGGCGTAGGCATGGGCAAAGATCACACAATCTTTGCAACTGTCGAAGGCGCAGTCACCTTCCGCAAAGGCCTGAAAGGCCGAACCTTTATTTCGGTGCTTCCAGTGGCGGAGGCCGCTGAATAAATGGCAAAGCGGTGGTATTCGGTCAGTGTTCTCTCGAACTTTGAGAAGAAGATTGCGGAACAGATCCGGACGGCGGTTGAGGAGCAGGAGCTTCATGACCAGATCGACGAAGTTCTCGTTCCTACCGAAGAGGTGATCGAGGTGCGTCGCGGCAAAAAGGTGACAACCGAACGTCGCTTTATGCCTGGTTACGTGCTGGTACACATGGAAATGTCGGACCAAGGGTATCATCTGATCAACTCAATCAACCGGGTGACTGGTTTCTTGGGTCCGCAGGGGCGTCCGATGCCCATGCGCGATGCCGAAGTCGATGCAATGATCGGCCGCGTTCAAGAGAACGAAGAATCGCCGCGTCTCGAGATCCGCTTTGAGACTGGTGAAAAAGTCAAAGTCACCGACGGTCCGTTCGAAGGCTTTGATGGCATGGTCGAAGGCGTTGACGATGACAACCAGCGCCTGCGCGTTTCGGTATCGATCTTTGGTCGTGAAACACCGGTTGAGCTGGAATTCACACAGGTCACTAAAGAAATGTAAATGAGTTTTACGCTTGCCATTGTCGGCCGCCCCAATGTGGGTAAGTCGACGCTGTTTAATCGCCTAGTCGGAAAACGCCTCGCGTTGGTCGATGACCAACCGGGTGTAACGCGTGATTTACGTGAAGGGGATGCCAGAGTTGGCGATATCCGCTTTACGGTGATCGACACCGCGGGCCTTGAAAACGCCAATGACGAATCGCTGCCTGCCCGCATGCGCCGCCTGACAGAGCGCGCTGTGGATATGGCCGACGTCTGCCTGTTTCTTGTCGACGCCCGCGCCGGTATTCTGGCCGATGATATTGTGTTTGCGGAAATCTTGCGCAGGCGGGCCGGAAAGGTGATTTTGGCCGCCAACAAGGCAGAGGGTGCCGCTGCCGACGCCGGCGTGATCGAGGCCTACAGCCTTGGCCTTGGTGAACCGATCCGCCTTTCTGCCGAACATGGCGAAGGGTTGAATGATCTGTATTCCTTTTTGTTGCCGATAGAAGAAGAATTCAAAGCCCGCGCTGACGCGAACGCCCCAGATATCGAAGTCGAAGTCGGAGATGATGATCCGGAC------GCGCCGCGTCCGATCACGGCGGACAGGCCTTTGCAAATTGCCGTTGTTGGCCGTCCGAATGCCGGCAAGTCGACACTGATCAACCAGATCATCGGCGAAGAACGCCTTCTGACCGGCCCCGAAGCCGGGATCACCCGAGATGCCATTTCCGTTCAAAAGGAATGGGATGGG---GTGCACATGCGGATCTTTGACACTGCCGGTATGCGCAAGCGCGCCAAAGTGCAAAAGAAGTTGGAAAAACTATCGGTTTCCGATGGCATCCGCGCGATCAAATTTGCCGAAGTTGTCGTGGTCTTGCTAGACGCCGAAATCCCTTTCGAGGTCCAAGACCTGAAAATCGCCGATTTGGCTGAACGTGAAGGCCGCGCCGTGGTTATCGCGGTCAACAAATGGGACATTGAAGACGAAAAGCAAGAAAAGCTGCGCAATATGCGAGAGGCCTTTGAACGTCTCCTGCCGCAGCTGCGCGGCGCGCCTTTGGTTACCGTCTCTGCCCGTACCGGCAAGGGACTTGATCGCCTACAGGCGGCCATTATGAAGGCGCATACCGTTTGGAACCGCCGCGTTTCCACCGGAGAATTGAACCGCTGGCTGTCCGATATGATCGAACGGCACCCGCCGCCCGCACCGCAGGGCAAGCGGATCAAGTTGCGGTATATTACACAGGCCAAAACCCGTCCGCCGGGCTTTGTTGTCATGTGTTCGCACCCTGACAAGCTGCCAACAAGCTATTCGCGCTATTTGGTCAACGGCATGCGCGAAGATTTTGACATGCCAGGCACGCCGATCCGCTTGGTTATGCGCGGCCAAAGCGATCAGAACCCGTTCAAGGGACGGCGTAAAAAGAACGCTGGCGCGCTTAAAAAGCACTTGGGCAGCTTGCCAAAAGCTTAGATGCGTGTGATCTTTATGGGAAGTCCGGATTTCTCCGTACCCGTGCTTGAGGCCTTGGTGGAGGCCGGACATGAGATTGCAGCGGTTTATTGTCAGCCGCCACGTCCCGCCGGACGCGGCAAGAAAGACAGACCGACAGCCGTTCATGCGCGCGCAGTTAAGATGGGGCTAGAAGTGCGCCATCCCGTTTCACTCAAGGGGGATGCGGAACGGGAAGATTTTGCAGCGCTTGGGGCGGATGTTGCAGTTGTTGTTGCCTATGGGTTGATCTTGCCACAGGCGATTTTGGATGCGCCTGAATTGGGGTGCTTGAATATCCATGCGTCGCTTTTGCCGCGTTGGCGCGGCGCGGCCCCCATTCACCGTGCCATCCTCGAGGGGGACGCAGAAACCGGAGTTTGCATTATGCAAATGGAGGCAGGGTTGGACACCGGACCGGTTTTACTGACGGAATCCCTGCGTATCGGCGACGAAGAAACTACAGGGGCCCTGCACGACCGTTTGTCGGAGATGGGGGCACGAATGATCTGTAAGGCGCTGGGCCAGCTGGGCAATCTGAGCCCGCAGGTCCAACCAGAGGATGGCGTCACCTATGCCGCCAAGATCGACAAATCCGAAGCCGCAGTGGACTGGAGCAAGCCCGCCGAAGTGATTTCCCGGCAAATTCGCGGCTTGTCGCCCTTTCCCGGCGTTTGGACCTTGCAAGACGGTCAGCGAATCAAGCTGCTGGGCGCTCGTTTGACTACGGGACAGGGCAATCCTGGCGAAGCGTTGGATGATGCAATGACAATTGCTTGCGGACAGGGCGCGGTTCAGATCACTCGGGCCCAGCGTGCGGGAAAGGGTGCACAAGACACTGACGTTTTCTTGCGCGGCAATCCGGTCGCCAAAGGCACCGTGCTAGGCAGT---TAAATGACCGACACGCCCGAATATCAGGTTCTTGCCCGCAAATACCGTCCAGAGACCTTTGTTGATCTGGTCGGCCAAGACCCGATGGTGCGCACGCTGCGCAACGCGTTCGAGGCGGACCGCATTGCTCAGGCCTTTGTGATGACAGGCATTCGCGGCACCGGCAAAACCACCACTGCCCGCATCATCGCCAAAGGCATGAATTGCATCGGCGCAGACGGAAACGCTGGCCCCACCACCGATCCATGCGGCATCTGTGAACACTGCGTCGCCATCATGGAAGGCCGCCATGTCGACGTGATGGAAATGGACGCGGCATCAAACACCGGTGTCGCCAATATCCGCGAAATCATCGATTCTGTGCACTATCGCGCGGCTTCTGCCCGCTACAAAGTCTATATCATCGACGAAGTGCATATGTTGTCGACCGGTGCATTCAACGCCTTGCTCAAAACGCTCGAAGAGCCACCGGCGCATGTCAAATTCATTTTCGCGACCACGGAAATCCGCAAGGTGCCCGTGACGGTACTGTCGCGTTGCCAGCGGTTTGACCTGCGCCGGATCGAACCCGAAGTCATGATTGCCATGCTGCGAAAAATCGCGACCGGTGAAAATGCCCAGATCACCGACGACGCTTTGGCCCTGATTTGCCGCGCAGCCGAAGGATCGGCGCGCGATGCAACATCGCTGCTTGATCAGGCCATCTCCCATGGAGCAGGCGAGACAACTGCCGATCAAATCCGCGCCATGTTAGGACTGGCGGATCGCGGCCGCGTTATGGATTTGTTCGAACGGATCATGCGCGGTGATGCGGCAGGCGCGCTACAAGAGCTGTCGAGCCAATACGCCGACGGAGCAGACCCGCTGGCCGTCTTGCGCGACCTGGCCGAACTGACACACTGGGTTTCGGTGGTGAAAATCACCCCTGACGCAGCCGAAGACCCCACGATCGCACCCGATGAACGCACCCGCGGATTGGCTTTTGCCGACGGGCTTGGCATGCGTCCCCTGTCGCGAGCCTGGCAAATGCTGCTCAAGGCGATTGAAGAGGTCTCCAACGCCCCGTCTTCGATGATGGCGGCTGAAATGGCCGTGATCCGGTTGACCCATGTTGCCGATCTGCCGTCACCCGAAGAATTGATCCGCAAACTTCAAGACACGCCGCCACCCACTGCTCCG------GGGCCCCAAGGAGGCGGGATGGCGCAACCAAGCCATAACACCTCAGCCCAAGGGCTACCGGCACCACCGGGCACGTCTGGCCCGGTGGCCTATGCCGGCGGTGCGGCGGCAGCCGTGGCCGTCGATCAAGCCTTGGCCAAATATCCCACGTTTGAACATGTGTTGGAGCTGATCCGGATCAACCGCGATGTCAAATTGCTGGTCGACGTAGAGGGCAGCGTTCGATTGGCCGCCTATCAACCCGGCCGGATAGAGTTTGCACCGGCGGACAATGCCTCGCCCGATTTGGCGGCACGGATCGGCGGGGCCTTGCAACGCTGGACCGGAAACCGTTGGGCAGTTTCAATCGTCAGTGATTGCAATGCGCCAACCATTGTCGAACGCCGCGATGCTGCGAAACTCGCCTTGCATGCCAAAGCCGAAGAACACCCGCTGGTTCAGGCCGTCATCGCCGCCTTTCCCAAAGCCGAAATCGTTGATGTCCGGACTGCCGAAGACATCGCCGCCGTCGCGCTTGAGGAGTCTTTGGCGGAAGTCGAAGACGAATGGGATCCGTTTGAAGAAGACTAGATGCCCAAGATGAAGACGAAATCGAGCGCCAAAAAGCGCTTCAAGGTCACTGCGACCGGTAAAGTGATGGGTGGTCAGGCTGGCAAACGCCACGGCATGATCAAACGCACACGCAAATTCATCCGCGATGCTCGCGGCACCACGACATTGTCTGCACCTGACGCGAAAACCGTCAAGTCATTCATGCCGTACGACCGCTAAATGAACCTCTTTACAGATATCCGCGAGCTGGTGCTCGCTTCGATCAATGCCATGCAAGCCGACGGCGCGCTTCCGGGAGAGTTGAACCTCGCTCCGGTGACGGTTGAGCCGCCGCGCGATCCTGCCCATGGCGATATGGCGACCAATGCCGCGATGGTGCTGGCCAAACCGGCCAAGCAAAAGCCACGCGACATTACACAAGCACTGGCGGCCAAACTGGCTGAGGATGACCGCATCCAGAGCGCAGAAGTGGCAGGCCCTGGTTTTCTGAACCTGCGCTTGTCGCCGGCTTTGTGGCAAAAAGTGCCCGCCGAGATCCTGAAGACTGGCACCGATTATGGCCGCTCTGACATGGGCGCGGGCAAGAAAGTCAACGTTGAATACGTCAGCGCCAATCCGACCGGTCCAATGCATGTTGGCCACGCGCGCGGCGCAATTGTTGGCGATGCGATGGCCTCTTTGCTGGATTTCGCAGGATTTGACGTAACACGCGAATACTACATCAACGACGGCGGCGCGCAGGTGGATGTTTTGGCGCGCTCTGTCTATTTGCGCTACCTCGAAGCACATGGCCAAGAGGTCGAATTTCCTGAAGGCACCTATCCGGGTGATTACCTTGTTGCGGTGGGTCAAGCGCTCAAGGACAAGGTTGGCGATGCCTATATCGATCAGCCCGAAGATGTCTGGCTGGCGGATGTTCGTGTCTACTCCACGGATGCGATGATGGATTTGATCCGTGAAGACCTCAAAGCTTTGGGCGTTGAGATGGATTACTTCTTCTCCGAAAAATCGCTGTACGGAACCGGTAAGATCGAGGCGGCGATTGACAGTCTGCGTGACAAGGATCTGATCTATCGCGGGGTGCTTGAACCGCCCAAGGGTAAAAAGCCCGAAGATTGGGAACCGCGCGAACAGACTTTGTTCAAATCAACTGAGCATGGCGATGATGTTGATCGCCCTATCCAGAAATCGGACGGCGGCTGGACCTATTTTGCCCCCGACATTGCCTATCACTACGACAAGGTCGAGCGCGGTTACGATCTGCTGATCGATATTTTCGGGGCCGATCACGGCGGCTATGTTAAACGGATGAAGGCGGCGGTTTCGGCACTGTCGGGGGGCACTGTGCCACTTGATATCAAGCTGATCCAGCTGGTGAAACTGTTCAAAAACGGCGAGCCGTTCAAAATGTCCAAGCGGGCAGGGACCTTTATCACGCTGCGCGATCTGGTTGAAATGGTTGGTGCAGACGTAGCGCGTTTCCATATGTTGACCCGCAAATCGGACGCCTCTTTGGACTTTGACTTCGACAAAGTTGTCGAACAGAGCAGTGAAAACCCTGTGTTCTATGTGCAATATGCGCATTCCCGGATTTGCTCTGTTTTACGCAATGCAGCAGCGCAGGGCATCTCCACCGATGACGCAACATTGGCGGGTGCGGATTTGAGCAATCTG---CATGAAGCTGAAATTGCCTTGATCGCCAAGCTGGCTGACTGGCCGCGTCAGGTGGAAATTGCGGCCCGCGTGCACGAACCGCACCGTATTGCGACCTACCTTCAAGAGCTGGCCTCGGACCTGCACGGTCACTATAACCGCGGCAACAAAGAGACCGAGCTGCGCTTTTTGCAAGAT---GACGCCAATGTCACACTGACCAAAATTGCGCTGGCGCGGGCCGTTGCAGTTGTTATTTCCGCCGGTCTTGGTATCTTGGGCGTAACTCCAGCCACAGAAATGCGCTAAATGACCCAAATTGATTCACTACCGCCTCTGCGCGATGTCATCACCAGCCATGATTTAAAGGCCCGCAAGGCGCTTGGGCAAAACTTTTTGCTCGACCTCAATCTCACCTCCCGTATTGCGCGCATCCCCGGAGACCTGTCCCAAACCGATGTGATCGAGGTCGGTCCCGGCCCCGGCGGATTGACACGCGGCTTGCTGGCCGAAGGTGCACGCCACGTGCTGGCCATCGAAAAAGACAGCCGCTGCCTGCCGGCGCTTCAACAAATCGCTGATATCTATCCCGACCGATTTACCTTTGTCGAAGGCGATGCCCTGAAAATTGATCCGCTTGAGCATCTGACACCGCCCATCGCGATCTGCGCCAACTTGCCCTACAATGTCGGCACCGAACTACTGGTGCGCTGGTTGACCCCCAAAGAATGGCCGCCCTTTTGGTCATCCTTGACCTTGATGTTCCAAAAGGAAGTTGCCGAGCGGATCATCGCCCAACCCGGATCAAAGGCCTATGGCCGCTTGGCGATCCTTGCGCAATGGCGCGCCGATCCACGTATCGCGCTGACCTTGCCACCCGAAGCTTTTTCGCCGCCGCCCAAAGTGCATTCCGCCGTGGTACATCTTACCGCCCTGCCCGAGCCGCGCTTTCCGGCCAAAGCCAAGGTTTTGGAACGTGTTGTCGCTGCGGCCTTTAACCAGCGCCGAAAAATGCTGCGCTCTGCCCTCAAAACCTTGTCTCCGGATATTGAGGATCGTCTGCTGGCTGCGGGGATCAAACCCACAGAGCGCGCCGAACAGGTCCCGCTAGAAGCCTTCTGCGCCCTAGCGCGACAATTCGAAGACGACTAAATGAACCTGATCGCACAGCTCGAAGCGGAGCAAATTGCTGCGCTGGGGAAAGACATCCCCGACTTCAAAGCCGGCGACACCATTCGCGTAGGCTTTAAGGTGACCGAAGGTACACGTAGCCGTGTACAGAACTACGAAGGTGTTTGCATCAGCCGTAAAAACGGCAAGGGCATCTCGGGTTCGTTCACCGTTCGCAAGATTTCCTTTGGTGAAGGCGTGGAACGTGTGTTCCCACTGCATTCCACCAACATCGAATACATTGAAGTTGTTCGCCGTGGCCGTGTGCGCCGCGCCAAGCTTTACTATCTGCGTTCGCGTCGTGGCAAATCTGCCCGTATCGCAGAAGATTCAACCTACAAGCCCCTCAAGGCCAAGTCA---TAAATGGCAAACAGCAAACGTGTACTGTTCCAAAAGCGCCGCATGCGCGTCCGGAACAAACTCCGCAAGGTCAACGCTGGTCGCGTTCGTCTTTCGGTTCACCGTTCGAACAAGAACATCAGTGTTCAGCTCATCGACGACGTGGCAGGCCGCACTTTGGCAGCTGCATCGACACTCGAGCCAACTCTGGGCCTTGTCGGCAAGAACAACGTCGAAGCCGCAACCAAAGTGGGTGCTTTGATCGCCGAGCGTGCGAAAGCCGCTGGTGTAGAGGAAGCATACTTCGATCGTGGTGGTTTCTTGTTCCACGGCAAAGTGAAGGCCTTGGCCGACGCTGCGCGTGAAGGTGGTTTGAAGATCTAAATGTTCGCGGTACTCAAGACCGGCGGCAAGCAATATAAAGTCCAGGCGGGTGATATCCTCCGTGTGGAAAAGCTGGCTGCTGATGCGGGCGAAACCGTCCAGTTCAACGACATTTTGATGATTGGCGGCGAAAGCACTGCCATTGGCGCGCCTTTTGTGGCTGGCGCTGGTGTTCAGGCGGAAATCGTCGACCAGATCAAAGGCGAAAAGACCATCAACTTCGTCATGCGTCGCCGTAAGCACGGCTCGCAGCGTACCAAGGGTCACCGTCAGCAGCTTACTTTGCTGCGTATCACAGACATCTTGGCCTCTGGCGCAGATGCATCCGGCGTAAAAGCTGCTGTTGGCGCGGGCTCCGCACCA---------------------------------------------------------AAAGCCGCTGCTCCGGCCGCTGCTCCTCAAAAAGCG---------GCTGCGGCTGCTGGTGCA---------------GATGATCTGACCGAGATCACTGGTGTCGGCCCCGCCGCCGCCAAAAAGCTGGTAGACGCCGGCATCACAACTTTTGCACAACTTGCAGCAGTTGACGCGGAC---------------------------ACCTTTGAAGGTGCCAAAGTCAAAGCTGAGTGGGTTGAGCAAGCCAAAACACTGGCC------TAAATGTCCCGAGTTAAAGGCGGAACCGTCGCGCACGCGCGTCATAAAAAGGTAATCAAGCAGGCCAAAGGTTATTATGGCCGCCGCAAGAATACCTTCAAGGTAGCAGCACAGGCAGTCGACAAGGCGAACCAATACGCCACACGCGACCGTCATAACCGCAAGCGCAATTTCCGCGCGCTGTGGATTCAGCGTATCAACGCGGCTGTACGTGCTCACGACGAAGCACTGACATACTCCAAGTTCATCAATGGTCTGAACCTGGCCGGCATCGAAGTAGACCGTAAGGTTCTTGCCGATCTGGCCGTACACGAGCCCGAAGCATTTGCTGCGATCGTGGATCAAGCCAAAGGCGCTCTGGCCGCTTAAATGCAGGTCACCGAGACCCTGAACGAAGGCTTGAAGCGCGGTTATTCGATCGTCGTATCAGCCGCTGAGCTGGACGAAAAAGTCACTGCCAAGCTGGTAGAAGCGCAGCCCGAAGTTGAGCTGAAGGGCTTTCGCAAGGGCAAGGTCCCTATGCCATTGCTGAAAAAGCAGTTCGGCCAGCGCCTGATGGGCGAAGCGATGCAAGAAGCCATCGACGGTGCCATGAACAAGCATTTTGAAGACAGTGGCGATCGTCCTGCGTTGCAGCCAGAAATCAAAATGACCAACGATGACTGGAAAGAGGGCGACGACATCAACGTCGACATGTCCTACGAAGCATTGCCCGTTGTTCCGGAAATCGATCTGTCGGTCATCGCAGTCGAAAAACTGGTTGTCAAAGCAGACGACGCAAGCGTTGACGAAGCGCTGGCTTCTTTGGCCGAAACCGCTCAGGATTTCGAAACC------AAAGACGGCGCAGCTGAAGATAAAGACCAGGTTGTCATCGACTTCTTGGGCAAAGTTGACGGCGAAGCCTTTGAAGGCGGCGCGGCTGAGGATTACCCTCTGGCCCTCGGAACCGGTTCCTTCATTCCGGGTTTCGAAGAGCAGCTGATCGGTGTCAAAGCCGGCGACGAAAAGAACGTTGAAGTCAAATTCCCCGAGGAATATGGCGCGGAAAACCTGGCCGGCAAAGACGCTGTGTTCGAGGTCAAAGTCAAAGAAGTCAAAGCGCCCAAAGCTGCTGAGATCAATGACGAGCTGGCCACCAAATTCGGTGCCGAAGACCTTGATGGCCTGAAGGGTCAAATCCGTGAGCGTCTTGAAGCTGAATACACTGGTGCCGCACGTCAGGTGATGAAGCGCGCAATGTTGGACAAGCTGGACGATCTGGTTTCCTTCGATCTGCCGCCGTCGCTGGTCGAAGCCGAAGCTGGCCAGATTGCGCACCAGCTGTGGCACGAAGAAAACCCCGACGTTCAGGGCCACGACCACGATCCGGTTGTCCCAACAGACGAGCATAACAAACTGGCCGAACGCCGCGTGCGTCTAGGCTTGTTGTTGGCCGAACTGGGTCAGAAAGCCGAAGTTGAAGTCAGCGAAGCGGAAATGACTCAAGCGATCATGAACCAGGCGCGTCAGTACCCGGGTCAAGAGCGCCAGTTCTTTGACTACGTCCGTGAGAACCCGCAGATGCAGCAACAGATGCGTGCGCCGATCTTTGAAGACAAAGTGATCGACCACATTGCTGAAAAAGCTGAAGTGACTGAAAAAGAGATCTCCAAAGACGATCTGCAAAAAGCAGTTGAAGCGCTCGACGACGAGTAAATGAAACTCAATGAACTGCGTGATAACGATGGCGCAACAAAACGCCGCAAACGCGTTGGCCGTGGTGCCGGTTCGGGCATGGGTAAGACCGGTGGCCGTGGTATCAAGGGTCAAAAATCCCGCTCGGGTGTAGCCATCAATGGTTACGAAGGCGGTCAGATGCCTTTGTACCAACGTCTGCCTAAGCGCGGCTTCAACAAGCCGAACCGCAAGGCATGGGCCGTTATCAACTTGGGCCTGATCCAGAAATTCGTAGACGCCGGCAAATTGACTGCGGGC---GATATTTCCGAAGATACACTGGTGTCTTCCGGTCTGGTTCGCCGCAAGCTGGACGGTATTCGCGTTCTTGCAAAAGGCGAAGTTACTGGCGCTCTGACCATCAACGTAACCGGCGCTTCCAAGTCCGCAATCGCGGCTGTTGAGGCAAAAGGTGGCGCGCTGACAGTCACGAACGCGGCAGCAGCGGAA---------TAAATGATCCAGATGCAGACCAATCTGGATGTAGCTGACAACTCCGGTGCCCGGAAAGTTCAGTGCATCAAGGTCCTCGGCGGCTCGCATCGCCGCTACGCATCGGTCGGCGATATCATCGTCGTATCGGTCAAAGAGGCCATTCCACGTGGTCGCGTTAAAAAGGGTGACGTCCGTAAGGCCGTTGTCGTTCGCACCGCAAAAGAAGTTCGTCGTGATGATGGCACCGCCATCCGTTTTGACCGCAACGCTGCAGTGATCTTGAACACTGCAGGTGAGCCGGTTGGCACGCGTATCTTTGGCCCAGTCGTTCGTGAACTGCGCGCAAAGAACTTCATGAAGATCATCTCACTCGCCCCGGAGGTGCTGTAAATGCGTCATAAACGTGGATATCGCCGCCTTAATCGTACACATGAGCACCGCAAAGCGTTGTTCTCGAACATGGCAGGCTCGCTCATCGAACATGAGCAGATCAAAACCACTTTGCCAAAAGCAAAAGAACTCAAGCGTATCATCGACAAGCTGATCACCTTGGGCAAGCGCGGCGATCTGCACGCGCGCCGTCAGGCTGCAGCTCAGCTGAAAGAAGACAAGGACGTTGCAAAACTGTTCGAGATCCTTGGCCCACGTTACGCCGAGCGTTCGGGCGGCTATTGCCGTGTTCTGAAAGCTGGCTTCCGTTACGGTGACATGGCACCCATGGCGATCATCGAATTGGTTGACCGCGACGTAGACGCTAAAGGCAAGGCTGACAAAGCCCGCTTGGAAGAAGCAGAAGCTGCTGACGCATAAATGCTTCAGCCAAAGCGTACTAAATTCCGCAAGATGTTCAAAGGCCGTATCAAAGGCCTTGCCAAAGGTGGTTCCGATGTAACGTTCGGCACTTACGGTCTTAAGGCTCTTGAGCCTGAGCGCGTAACTGCCCGTCAGATCGAAGCTGCACGTCGTGCCATGACTCGTCATATGAAGCGTCAAGGTAACGTTTGGATCCGCATCTTCCCAGACACACCTATCACTGCCAAGCCTATCGAAGTTCGTATGGGTAAAGGTAAGGGTTCTGTGGACCGTTGGGCCTGTAAGGTTAAGCCGGGCCGCATCATGTTCGAAATCGACGGTGTATCCGAAGATGTCGCCCGTGAGGCTCTGCGTCTGGCCGCGATGAAACTGCCGGTGAAAACCCGCGTAGTCATCCGCGAAGACTGGTAAATGGACCCGATTTTTCTGACCAACAGCAAGACACGCAAAAAGGAACGCTTTGTTCCGATTGATCCGAAAAATGTGCGGCTCTATTTGTGTGGACCGACGGTCTATGACCGTGCGCATTTGGGCAATGCCCGTCCGGTTCTGGTCTTTGATGTTTTGCAACGTCTGCTGCGCCACGTCTATGGCGCGGATCATGTGACCTATGTGCGCAATTTCACCGATGTGGACGACAAAATCAACGCAGCGCGGAAAGAGGCAGGCGCGCAAGTGCATGAACGTTCAAGCGAGACCATTGACTGGTATCATCAGGATATGGATGCACTAGGGGCGGCGCGCCCGGACCATGAACCGCGTGCTACCGAATATATCGATCAGATGGTTACAATGATTGCCGACCTCATCGCCAAGGGGCATGCCTATGAGGCAAAGGGCCATGTTCTGTTCCGCGTCCGGTCTTACAAGGATTATGGTCAGCTGTCAGGCCGGTCTGTTGATGACATGATTGCTGGCGCGCGGGTCGAAGTGGCCCCGTTCAAGGAAGACCCGATGGATTTTGTGCTATGGAAGCCGTCCACGGATGACCTGCCGGGCTGGCCGTCCCCTTGGGGCAGGGGCCGCCCGGGTTGGCATATCGAATGTTCCGCTATGGCGGATGATTTGCTTTGGAAAAAATTTGACATTCATGGCGGTGGCAATGATCTGACTTTTCCTCACCATGAAAATGAAATTGCCCAAAGCTGCTGCGCCCACAGCGAAGTTGGTTTCGCCAATGTCTGGCTGCACAACGAGATGTTGCAGGTCGAGGGCAAGAAGATGTCCAAGTCGCTGGGCAACTTCTTTACCGTCCGCGATTTGTTGGATCAGGGCATTCCGGGCGAAGTGATCCGCTTTGTGATGCTGTCGACGCACTACCGCAAACCGATGGATTGGACTGAGAAAAAGGCTCAGCATGCTAATAGCACTTTGGACAGTTTTGTAAGCCAAACAGTTAACATGGAACCTGCAAAAGACATTCCTGTCGAATTTTTAGAAGCCCTAGCTGATGATCTAAACACCTCCAAAGCAATTACCGTTTTGTACAGTTTGGCCAAGAGATTTGAT------GAATTGCTCGCGTCACTTTTGTTCTTAGGG------------------GGTGGTTGGGTTGGAAACAATACG------TTGGAAGAACTGGTATTTGAATTGGAGAAGCTTAGAGCCGAAGCCATGGAAACCAAGGATTTCTCCCAAGTCGACCGGTTGAAAACCGCCTTGATTGACGCAGGGGTCGAAGTACGGATGTCCAAAGCCGGCGTAGAGCTGGTGTCGGGTCCAAATTTCGATGCGGCCAAGTTGGAGGGGCTG---TAAATGGCTAAG---------AAACCCGTATCAGACCCGAATTATAAAGTGATCGCCGAAAACCGGCGTGCGCGCTATGACTATGCTATCGCTGATGACATCGAGTGCGGAATCATTCTGGCCGGGTCAGAGGTGAAGTCCCTGCGCGAAAACAGCGCAAATATTGCCGAAAGCTATGCGGCCGTAGAGGACGGCGAGTTGTGGCTCGTGAACTCTTACATTGCCCCCTACACACGTGCC---ATGTTCAGCCACGAAGAACGCAAACGACGCAAGCTGTTGGTATCGCGCAAAGAACTGTCGCGGCTGTGGAATGAAACCCAGCGCAAGGGCATGACGCTTGTTCCGTTGGTGATGTATTTCAATCATACCGGCAAAGTTAAGATCAAAATCGGCATCGCCAAAGGTAAGAAGAATCACGACAAACGTGAAACTGATGCCAAGCGTGATTGGGGCCGCCAAAAGGCGCGACTGCTAAAGCAAAGT---TAAATGAGCGCGAAAGCACAACACTACGACATCGTTCGCAAGCCGATCGTAACCGAGAAAACCACAATGGCATCCGAAAACGGTGCAGTGGTTTTCGAAGTCGCCATCGATAGCAACAAGCCACAGATCAAAGAGGCCGTTGAGGCTCTCTTTGGTGTGAAGGTGAAAGCGGTTAACACCACAATCACCAAAGGCAAAGTCAAGCGTTTCAAGGGCACCACCGGTCGCCGTAAAGACGTTAAAAAGGCCTATGTAATGCTCGAAGAGGGCAACACCATCGACGTGACCACTGGTCTCTAAATGGGCAAGGATAAAAATCCCCGCCGCGTGGCGGACAACGAAGCGATGGCAAAGCTGCGCATGCTGCGCACCTCGCCGCAGAAACTCAACCTTGTTGCCCAACTTATTCGTGGCAAAAAAGTTGATCGCGCTCTTGTCGATCTGACCTTCTCTAAGAAGCGGATCGCCGAAGATGTGAAAAAGTGCCTGCAGTCCGCCATTGCAAACGCTGAGAACAATCACGGTCTGGACGTTGACGAACTCGTCGTCGCCGAGGCCTATGTTGGTAAGAACCTGATCATGAAGCGCGGTCGTCCTCGGGCGCGTGGTCGTTTTGGTCGGATCAACAAGCCGTTCTCGGAACTCACCATCAAGGTGCGTCAGGTTGAGGAGCAAGCCTAAATGGTATCCGCAGCAGAGCAAATGGCCGCAAACACAAGTTGGGCGGCACTGGGCAAAGCAACCGACCTGCGCAATCGCATCCTCTTTACCCTTGGCCTATTAATCGTCTACCGCCTCGGCACCTTCATTCCCGTTCCGGGAATTGACGGCGCTGCTTTGCAGGAATTCGTTGATCAGGCCGGGCAAGGGATCGCTGGCATTGTGTCGATGTTCACAGGGGGCGCGCTTGGCCGCATGGGCATCTTTGCCCTCGGTATTATGCCCTATATTTCCGCCTCCATTATCATTCAGTTGCTGACGGCCATGGTGCCGGCACTTGAGCAGTTGAAGAAAGAAGGCGAACAGGGCCGTAAAAAAATCAACCAGTACACACGCTACGGCACGGTGGTTCTGGCGACTTTTCAGGCTTACGGCCTTGCGGCCTCGCTTGAGGCGGGCGACCTGGCTACCGATCCCGGTTGGTATTTCCGCGCAGCCTGTGTGATCACCCTTGTGGGCGGCACCATGTTCCTGATGTGGCTGGGTGAGCAAATCACCGCACGCGGTATCGGCAACGGCATCTCGCTGATCATCTTCGTCGGTATCATCGCCGAAGTTCCTGCCGCTTTGGCGCAGTTCTTTGCCTCTGGCCGCTCTGGTGCGATCAGCCCTGCAGTGATTGTCGGTGTCATGCTGATGGTTGTGGCGGTGATTGCCTTTGTGGTCTTTATGGAACGGGCGCTTCGAAAGATCGCCATCCAGTATCCCCGCCGTCAGGTGGGCATGAAGATGACAGAAGCGCAACAAAGCCATCTGCCACTCAAAGTGAACCCGGCGGGCGTCATTCCTGCGATCTTTGCCAGTTCTTTGCTGTTGCTACCGGTGACGATCAGCACCTTCTCCGGCTCTGAAACCGGTCCGATTATGTCGACAGTTCTGGCCTACTTTGGTCCCGGACAGCCGCTATATCTGTTGTTCTTTGCGTCGATGATCGTCTTCTTTGCGTATTTCTATACGTTCAATGTGTCGTTCAAACCTGATGAAGTTGCGGACAACCTGAAGAATCAGAACGGTTTTGTTCCGGGCATTCGCCCAGGCAAAAAGACAGCTGAGTATCTTGAGTATGTCGTGAACCGTTTACTTGTTGTTGGCGCTGCATATTTGGCTGCTGTTTGTTTGCTGCCGGAAATTCTGCGGGCTCAATTCAGCATTCCGTTCTACTTTGGCGGAACTTCGGTCCTTATTGTCGTTAGCGTGACAATGGACACTATTCAGCAAGTGCAAAGTCATCTTCTCGCACATCAGTACGAAGGTCTTATTCAGAAGTCGCAGCTTCGCGGTAAAGGT---AAGGCTCGTAAGCGGAAAGGGCCGGCGCGCAAATGAATGAAATTCCTCGATCTTTGCAAAGTCTACATCCGCTCTGGTGGCGGGGGCAATGGCTGCATCAGTTTCCGCCGCGAGAAGTTCATCGAATACGGTGGGCCGGATGGCGGCGATGGCGGACGGGGCGGCAGTGTCTGGGTCGAGGCCGTAGACGGCTTGAACACATTGATCGATTTTCGGTATCAGCAGCATTGGTTTGCTGGCAACGGGCAGTCTGGTGCGGGCCGTGGGCGTACTGGCAAAGATGGTGAAGACATCATTTTGCGCGCGCCGGTTGGCACCGAAATTATCGACGAAGATCAGGAAACTGTAATTGCCGACCTGACCGAACTGGGACAGCGCGTTTTGCTGGCCAAAGGCGGCAATGGCGGCTGGGGCAACCTGCATTTCAAAACCTCGACCAATCAGGCACCGCGCCGGGCCAATCCGGGCCAGGACGAGATCAACCGCACCCTTTGGCTGCGCCTGAAATTGATCGCTGACGTGGGTCTTGTGGGTTTGCCAAATGCCGGTAAATCGACTTTTTTGGCTGCGACATCAAACGCGCGCCCCAAGATCGCCGATTATCCGTTTACAACGCTACACCCCAATCTTGGGGTTGTGCTGGTTGATGGCAATGAATTTGTCGTCGCGGATATTCCCGGCCTGATCGAAGGTGCCCATGAAGGGCGTGGTCTTGGTGATCTGTTTTTGGGCCATGTCGAACGCTGCGCCGTGTTGCTGCACTTGATCGACGGCAGCTCAGGCAGTTTGCTTGAGGATTACAAAACGATTTGCTCCGAACTGGAAGCTTATGGCGAAGGGTTGGCTGACAAGCCCCGCGTCACGGTTCTGAACAAGATTGATACGTTGGACGCAGAAGAACGCGCGTTCTTGAAGGACGAGCTGGAAGCGATTGGCGCTGAAAACGTGCTGCTGATGTCAGGTGCTTCTGGCGAAGGCGTAACGGATGTTTTGCGCGCACTGCGCCCCCATGTCGACGCCCGCCGCGCTGCGGATCGCCCTGTTGAGGAGGACGGGACTTGGCATCCCTGAATGGCTGCTAAGTTGAAAAAGGGTGACAAAGTCATCGTTCTTGCTGGCAAAGACAAAGGCAAAACCGGCGAGATCACTTCGGTGAACCCAGCTGCTGGTAAAGCCATTGTTGACGGCGTGAATGTTGCGATCCGTCACCAGCGTCAGTCGCAAACAGCTCAGGGCGGCCGCCTTCCTAAGGCCCTGCCAATCCAGCTGTCGAACCTGGCACTTGTTGATGCAAACGGCAAAGCAACCCGCGTTGGCTTCCGCATGGAAGGTGACAAGAAGGTTCGTTTCGCCAAGACCACAGGGGATGTAATCGATGCTTGA---ATGCGCTCTGGTATTATCGCAAAGAAAGTCGGGATGACTCGCGTCTTCATGGAAGATGGCAAGCAGATCCCAGTGACTGTTCTACAACTCGAAAACCTGCAGGTTGTTGCTCAGCGTACTGCTGAAGACAATGGTTACACAGCTGTTCAGCTGGGTGCCGGCACAGCCAAGGTTAAACGCACAAGCAAAGCCATGCGTGGTCACTTCGCCAAGGCGAATGTTGAACCCAAGCGCAAGGTTGCTGAATTCCGCGTTGCTCCTGAAAACATGATCGAAGTGGGCGCGGAAATTACCGCTGAACACTATAACGAGGGCCAGTACGTTGACGTATCCGGCATCTCGATCGGTAAAGGTTTTCAAGGCGGCATGAAGCGTTGGAACTTTGGCGGTCTGCGCGCGACACACGGTGTTTCGATCAGCCACCGTTCGCACGGTTCGACAGGTCAGTGTCAGAATCCCGGTAAGGTTTTCAAAGGCAAGAAAATGGCCGGTCACATGGGTGCAGCCCGTGTTACCACCCAAAACCTTCAGGTTGTCAAAGCTGACGCGGATCGCGGCATCTTGATGATCAAAGGCGCCGTTCCTGGTTCCAAAGGTGGTTGGGTTACGGTCAAAGACGCCGTGAAAAAGCCGCTGCCCGATGACGTGCCAACACCGGCCGGTCTTCGCGCAACTGCC---------------------------------------GCACCTGCAGAAGCTCCTGCAGAAGGAGGT---------------------------------------------------GAAGCA------------------------------------------------------------------TGAATGGCATTAAAGTCGTATAAGCCGACTACGCCAGGCCAGCGTGGGCTGGTTCTGATCGACCGTTCGGAGCTGTGGAAAGGTCGTCCTGTCAAGGCACTCACTGAGGGTCTGACTAAAAAGGGCGGTCGTAACAACACCGGACGGATCACGATGCGTCGCAAAGGCGGTGGGGCAAAGCGCCTCTATCGTATCGTAGATTTTAAGCGGAACAAATTCGACGTGACAGCAACTGTTGCACGTATTGAATATGACCCTAACCGGACCGCATTTATCGCACTCGTTCAATATGACGATGGCGAACAGGCCTATATTCTTGCACCTCAGCGTCTCGCCATTGGTGACCGCGTTGTTGCAAGCGCCAAGGCCGACATCAAGCCCGGTAACGCGATGCCCTTCAGCGGCATGCCGATCGGTACTATTGTTCACAACATCGAAATGAAGCCTGGTAAAGGTGGTCAAATCGCGCGTGCAGCTGGTACTTATGCCCAGTTTGTTGGTCGTGACGGTGGCTACGCCCAGATCCGCCTCAGCTCGGGTGAACTGCGCTTGGTTCGTCAGGAATGCATGGCCACCATCGGTGCTGTGTCCAACCCTGACAACTCAAACCAGAACTTCGGTAAAGCCGGCCGTATGCGCCACAAAGGCATCCGTCCGAGCGTTCGTGGTGTCGTAATGAACCCAATCGATCACCCACATGGTGGTGGTGAAGGCCGGACATCGGGTGGTCGTCACCCTGTTTCGCCTTGGGGTAAACCGACCAAGGGCAAGCGTACCCGCAATACCAACAAAGCGTCGCAGAAGCTGATCATCCGCTCGCGCCACGCCAAGAAGAAGGGACGCTAAATGCTTGATGAGCAAAAGTATACCCCGCGTCTTAAGACGAAGTTCCGCGACGAAATCCGCGCCAAGCTGAAAGAAGAATTCGGCTACAAGAACGACATGCAGATCCCGCGTCTGGATAAAATCGTTCTGAACATTGGTTGTGGTGCCGAAGCGGTACGTGATTCCAAGAAAGCCAAATCGGCGATCGAAGATCTGACAACAATCGCTGGTCAACAGGCCGTCGGTACAAAGGCCAAGAACAGCCACGCGCCGTTCCGCCTGCGTGAAGGCATGATCATTGGTACAAAGGTAACCCTTCGCGGTGACCGTATGTACGAATTTCTTGACCGTCTGACCACTGTTGCAATGCCTCGCATTCGTGACTTCCGTGGCGTGAAA---CCTTCGTTTGACGGTCGTGGCAACTTTGCCATGGGCCTGAAGGAACACATCGTCTTCCCCGAGATTGATTTCGACAAAGTCGACGAAGTCTGGGGCATGGACATCATCATCACCACGACCGCGGACGACGACGCGGAAGCCAAAGCGCTGTTGAAGCATTTCAACATGCCTTTCAACGCGTAAATGAAACTTGATGTGATCAAACTTGACGGCGCGTCCGTAGGTTCGGTGGATCTGGACGAAGCACTGTTCGGCGTTGAGCCGCGTGCAGACATCCTTCACCGTGTGGTTCGCTGGCAGCGTAACAACGCGCAGGCCGGTACCCACAAGGTAAAGACCCGCTCGGAAGTTAGCTACTCGACCAAGAAGATCTATCGCCAAAAAGGCACCGGTGGCGCACGTCACGGTTCCCGCAAGGCGCCCATCTTCCGTAAAGGTGGCATCTATAAGGGTCCAACACCGCGTTCGCACGGTCACGACCTGCAAAAGAAAGTCCGCGCACTTGGTCTGAAAATGGCTTTGTCTGCTAAGGCTGCAACTGGTTCGCTGGTTGTGATCGAGGACATCAACACCGACGGCAAGACCAAGACCTTGGCAAATCAGATCAAGGCTCTGGGTTGGAAGCGCGCGCTGGTCATCGACGGAGCTTCGGTTAACGAAGAATTCCTGAAGGCCGCTCGCAACATCGAAGGTCTGGACGTTCTGCCCACCATGGGTGCCAACGTTTATGACATCCTGCGCCGTGACACGCTTGTGCTCACAAAAGCGGGTGTCGAAGCACTGGAGGCTCGCCTGAAATGAATGGAAAACGTAATTCTCATTGTCCACCTTCTTCTTGCGCTAGGCCTTATTGGCGTAGTGCTCTTGCAGCGTTCCGAAGGCGGTGGCCTTGGGATGGGCGGCGGCGGCGGCGGGGCCATGAGCCAACGCTCAGCTGCGACAGCCATGGGCAAAGTGACCTGGGCCCTTGCCATTGCTTTTATCATCACCTCGATTTCTTTGACTGTCATCTCCGCGAAAAACGCTTCTGGCTCTTCGATCTTGGATCGTGTTGGCGGTGCTCCGGCTGTAGAAGAAACAGCTGATCCTGCTGGTGAA---TTGACCGACGCATTGTTGCCGCCTCCTTCGGCTGATGATGCGCCTTTGGTGCCAACCGCCGACTAAATGTCCTTTTTCGGCAAACTCAAAAACAAATTGTTCAAATCCTCTTCCAAATTGGATGAGGGACTGGATGCGATTGTCCAAGATGGTGGC------GAAGACGAGGTGGATGTTCCTGCGCCGGAGCCGGTTAAAACACCG---------------------------------ATACCTAACGACGCTGAAATCCCAACCGATGAATCCGAGTCCGAGGTGGCACCAGACCAAGAGACAGATTTAGACCCGTTGGCTGAG------------------------------------------GCACCGGAAGAAGCATCC---------------------------------------------------------------------------------------------------------GGTATTTTGGGCCGCCTGATGGGGCGTGGCGGGGAAAGTGCCGACCTTCGCCGTGTGCTTGACGATGACATGCTTGAAAGCCTTGAGGAGCTGTTGATCCAGTCCGATATGGGGGTTGATACAGCCCTGCGCGTGTCTGCCAATATCGCCGAAGGGCGGATGGGCAAGAAGCTGTCTGTGACCGAAATCAAAGGGCTTTTGGCCGGCGAAATTGCCCGCATCATGGAGCCGGTGGCAAAACCTTTGCCGCTATACGCAACGAAACCACAGGTGGTTTTGGTGGTCGGGGTGAATGGATCGGGCAAAACCACAACCATCGGCAAGCTGGCGTCCCAGTTCCGCGCGGCCGGAAAAAAGGTGGTGATCGCTGCGGGTGATACGTTCCGCGCTGCAGCGGTAGAACAATTGCAAGTCTGGGGGGACCGCGCGGGCGTGCCGGTTTTGACCGCACCCGAAGGGTCGGATCCCGCGAGCCTTGCCTATGATGCGATGACCAAAGCCCAAGAAGACGGGGCTGATCTGTTGATGATTGATACCGCGGGACGATTGCAAAACCGTCAGGATTTGATGGAAGAGTTGGCCAAGATTGTCCGGGTGATCCGCAAGAAAGACCCTGACGCGCCGCACAACACATTGTTGGTGCTGGATGCCACCACGGGCCAAAACGCTGTGACGCAGGTCGAAGTGTTCCGGAAAATTTCGGATGTATCGGGGTTGGTGATGACCAAACTGGATGGAACAGCGCGCGGCGGCGTCTTGGTGTCGCTTGCCGATAAATTTGGCCTGCCGATCCATGCGATTGGTGTCGGGGAACAAATTGATGATTTGGCCCCGTTCGATCCCGAAGAGTTTGCGGCTGCGCTGACCGGTCTTGATGCCTGAATGACAAAAGAACAATGGGGCGCGTTGCAAGAGCAGATCTGCAATACGGTTGGGGACAATAACTATAAAACCTGGATCAAGCCGCTTTCTTTTGCTGGAATGAACGACGGCGTCGTTACACTTCATGCGCCGACAAGCTTTTTTGGCAGCTATGTATTGCAAAACTACGGGGACATGCTTCTGGCGCAGATTTCAACTGTTGCGCCAACTGTACGCCGCATTTCCTATGCCGTT---------------------------GAGCAAGCCAGCGCTTCTAGCGAACCCAAGGCACCGGCCAAGCCGGCCGAAGCCGCGCCCAAGACACTTCCGGGTGCGCCATTGGATGGCCGGTTTACCTTCGACAACTTCGTCGTCGGAAAGCCAAACGAATTGGCCCATGCCGCCGCCAAACGTGTGGCCGAAGGTGGCCCGGTGACCTTCAACCCCCTGTTTTTGTATGGCGGCGTAGGTCTGGGTAAGACGCACCTGATGCACGCCATTGCGCACGAACTCAGCGCCCGAAATCCACATCTTTCTGTGCTGTATCTGTCAGCAGAACAATTCATGTATCGCTTTGTGCAATCCTTGCGCGAACGCAAGATGATGGATTTCAAGGAGATGTTCCGCTCCGTTGATGTTTTGATGGTGGATGACGTTCAATTCATTGCCGGCAAAGACAGCACTCAAGAAGAGTTCTTTCACACCTTCAACGCCCTTGTTGATCAAAACAAACAGATCATCATCTCGGCCGACCGGGCCCCGGATGAAATCAAGGATCTTGAGAACCGCATCCGGTCCCGTCTGCAATCTGGTTTGGTGGTGGATCTGCACCCTACCGATTACGAACTGCGTCTTGGCATTTTGCAGACCAAGGTCGAAACTTATCGCACCATGTACCCTAGCCTGTCGATCGATTCTGGCGTGTTGGAATTCCTTGCACATCGGATCAGCACAAACGTGCGTGTCCTTGAGGGGGCTTTGACCCGTTTGTTCGCTTTTGCGTCACTGGTCGGAAAGCCGATCAATATGGATCTGGTCCAAGACAGTCTTTCAGATGTTCTGCGTGCTTCTGAACGTAAAATTTCGATCGATGAAATTCAGCGCCGTGTCGCAGAGCACTACAATATTCGCCTCAGCGATATGATTGGCCCCAAGCGGGTGCGCAACTTTGCCCGCCCTCGCCAGATCGCAATGTATTTGTGCAAACAGCTTACATCCCGGTCCTTGCCGGAGATTGGCCGACGGTTTGGTGGACGTGATCACACGACTGTCATGCACGGGGTTCGGCGGATAGAAGAGCTGCGCGTACAAGATGGACAAATCGACGAAGATGTCGAAATGCTACGCAGGGCGCTAGAAGCCTAA------ATGTTTGGAAAAATCGCGCGGAAGGTCTTCGGTACTCCGAATGACCGTAAGATCAAGGCCACCCGACCGCTGGTGCAGCAGATCAACGCGCTGGAGGCCGAGTTTGAAAAACTGGGTGATGCCGGCCTGATCGAAAAGACAGAAGAATTCAGGAAACGTGTCGCCGATGGCGAATCGTTGGATTCTCTTTTACCCGAAGCCTTTGCCAACTGCCGCGAGGCCGCCAAGCGCGCCTTGGGACTGCGTGCTTTTGATGTGCAGCTGATGGGCGGGATCTTCCTGCATCAAGGCAATATCTCGGAAATGAAAACCGGTGAGGGCAAGACCCTCATGGCCACCTTTGCCGCCTATTTGAACGCTTTGACCGGGCGCGGCGTGCATGTTGTGACGGTAAACGACTATCTGGCCCGCCGTGATGCGGATTGGATGAGCAAAGTTTACGGTGCCTTAGGGCTGACCACTGGCGTTGTCTATCCACGTCAGGAAGATGGCGAGAAGAAAGAGGCCTACGCCTCAGACATCACCTATGCCACCAACAATGAGCTGGGGTTTGACTATCTGCGCGACAATATGAAATCTGAACTCGATCAGATTTATCAGCGCGACCACTATTTCGCTATCGTTGACGAAGTCGATTCAATCCTTGTTGACGAAGCGCGGACACCGCTGATTATTTCCGGCCCTGCCGAAGATCGATCAGAAATGTATGTCCAGATCGACAAGTTGATCCCGGATCTGGACCCTGACCACTATACCGTCGATGAAAAGACCCGAAACGTGACCTTCACGGATGAAGGCAACGAATTTCTGGAACAGCATCTTCAGGCCCGCGGCATTTTGCCCGAAGGTCAATCGCTTTATGATCCGGAATCGACGACGATTGTTCATCACGTGAATCAAGGCCTGCGCGCTCATGTGCTATTCACAAAGGACAAAGACTACATTGTCCGTGACCGCGAAGTTGTCCTGATCGACGAATTCACCGGTCGCATGATGGCCGGCCGCCGCTTGTCCGAGGGCTTGCATCAAGCGATCGAAGCCAAAGAAAACTGTGACATTCAGGCCGAGAACGTCACGCTGGCATCGGTAACCTTTCAAAACTACTTCCGCCTGTATGACAAGCTGGGCGGTATGACAGGTACGGCTGCAACGGAAGCCGAAGAATTCGCTGAAATCTATGGCCTCGGTGTTGTCGAAGTGCCAACCAACCGCCCAATCGCCCGTGTCGATGAAGACGACAAAGTCTACCGCACCGCGCGCGAGAAGTTTGAAGCCATCGTGGAAGAGGTCAAAATCGCCCATGAGAAGGGCCAGCCGGTTCTTGTCGGCACGACCTCAATCGAGAAGTCGGAAATGCTGAGCAATTTGCTCAAAGACGCCGGTTTGCCGCACAATGTCTTGAACGCGCGCCAGCACGAACAAGAAGCCCAGATTGTTGCCGATGCAGGCAAGCGCGGAGCGGTGACCATCGCGACCAACATGGCTGGCCGCGGCACGGACATCAAATTGGGCGGGAACGTTGACTTTACCGTCATGGAAGCAATCGCGGCTGACCCCGAAGGTGACCCCGAAGCTATCCGTACACGAATTGAAGAAGAGCACAAAACCGACGAAGCGGCCGTCAAAGAGGCCGGTGGCCTTTATGTCTTGGCGACAGAACGTCACGAAAGCCGCCGCATCGACAACCAGTTGCGCGGCCGCTCGGGCCGTCAGGGTGACCCGGGCCGGTCTTCTTTCTTCTTGAGCCTAGAAGATGACCTAATGCGCATCTTTGGTTCTGAACGCTTGGAAAAAGTGCTGTCGACTCTGGGCATGAAAGAAGGCGAAGCAATTGTTCACCCGTGGGTAAACAAATCGCTGGAGCGCGCTCAGGCCAAGGTCGAAGGTCGCAATTTTGACATCCGCAAGCAGCTGTTGAAATTCGACGATGTGATGAACGATCAGCGTAAAGTGATCTTTTCCCAGCGCCGCGAGATCATGGAAGCCGAGGATCTTTCGGAAATCGTGCAAGATATGCGCACCGATGTGATCGACGAACTGGTCGATATTTATGCACCTGCAAAGTCATATGCCGAACAGTGGGACATGCCGGGGCTATATGCCGCGAGTATTGAAAAACTGGGTGTTGATCTGCCGATTATTGCATGGGCCGACGAAGACGGCGTGGATCAAACTGTGATCCGCGAACGTTTGGAAGAAGCAACCGACAAGGCAATGGCCGAAAAAGCCGAGGCCTTTGGCCCGACAATCATGCGCCAGATTGAAAAGCAGTTCTTGCTGAATACCATCGACGGCAAATGGCGTGAGCATCTCCTGACTCTTGAGCACTTGCGGTCAGTTGTTGGGTTCCGTGGCTATGCCCAGCGTGACCCGCTGAACGAATACAAGACCGAAGCCTTTCAATTGTTTGAAAATCTTCTTGATGGTTTGCGCGAAACTGTGACGACTCAGCTGTCTCAAGTGCGCCCGATGAACGAAGAAGAGCGCGCCGCAATGATTGCCCAGGAAGAACAGCGGCAAAAAATGGCGCAGATGATGGCCCAGCAATCGACACCTGACGCCACTTCCGCTGAACGC---------CCATTGATTGCAGGTTTTGACGAAACCGATCCGGCAACGTGGGGCAATCCCGGCCGGAATGATGCATGCCCCTGCGGCTCTGGTGACAAATTCAAACACTGCCACGGGAAAATT---TAGATGAAACAATCAGCCGAGCTTATCGCCCTACAAGACCGGCTTGGATACACGTTTACCAAGCCGGAACTGCTGCGCCAATCTGTGACGCACAGTTCAATGTCGAGCCCGGGCAGAAATGATAACCAACGGTTGGAATTTCTGGGGGACCGGGTGCTGGGCCTTGTAATGTCCGAAGCGCTTTTACTGCATGATCAGCAGGCAACTGAGGGGGTGTTGGCCCCCCGCTTTAACGCTTTGGTGCGCAAGGAGACCTGCGCCGATGTTGCACGGCAGATTGATCTCGGTGCGGCACTCAAACTCGGGCGTTCTGAACAAATGTCTGGCGGACGGCGGAAATTGGCATTGCTCGGCGATGCCATGGAGGCGGTGATTGCTGCCGTCTATATGGATGGCGGATTTGAAACTGCCCGCACGCTCATCCTACGGCTGTGGGGCACCCGCATCGAGGCCGTTGAAGACGACGCCAAAGACGCCAAGACCAGCTTGCAAGAATGGGCACAGGCCCGCGCGCAAGAACCGCCCAGATACATATTGGCTGAACGGTCCGGCCCCGATCATGCCCCTGTGTTTACGATTCAGGCGGTCCTTGCTTCGGGCGAAACATCAACCGCCACGGCGGGATCAAAACGACAAGCGGAACAAGCGGCTGCAAAGGCCTTGCTCGACAAACTGGGA---------TAAATGGCAAATCTCGGAAAACGCACCCGCGCAGCGCGCGAAGCTTTCGCTGGCAAAGACAACCTGACTGTCGAGGACGCTGTTGCGCTTCTCAAAGGGAACGCAAACGCCAAATTTGACGAAACCATTGAAATCGCGATGGCGTTGGGCGTTGACCCCCGTCACGCTGACCAAATGGTTCGCGGTGTTGTCAGCCTGCCAAACGGCACTGGCAAAACCGTCCGCGTCGCTGTTTTCGCTCGTGGCCCAAAGGCCGAAGAAGCGCAGGCTGCTGGTGCGGACATCGTTGGCGCAGAAGATCTGATGGAAGCCGTTCAAGGCGGCACCATTGATTTTGATCGCTGCATTGCGACACCTGACATGATGCCCATCGTTGGTCGTCTTGGTAAGGTCCTTGGCCCGCGGAACCTGATGCCAAACCCCAAGGTTGGTACAGTGACCATGGACGTAGCCCAAGCTGTCAAAGACAGCAAAGGTGGCCAGGTTCAGTTCAAAGCTGAAAAAGCTGGTGTTGTGCATGCTGGTGTTGGCAAAGCGTCCTTCGACGAAGCCAAGCTGGTCGAAAACATCCGTGCCTTTGTTGGTGCCGTTTCGGCCGCCAAGCCTTCGGGTTCCAAAGGGACTTACATGAAGAAGATCTCTGTAAGCTCGACAATGGGCCCAGGCGTGACACTTGATGTGAACAGCGCAGTCGGAAGCTAAATGTCCGAAGATTTCGAACTCGATACTGATGATCTGATCCGCCGTATGGATGGCGCAATGGCAAACCTGCGGACGGAGTTTGCATCTCTGCGCACCGGCCGTGCTTCTGCGTCCATGCTGGAGCCGATCGACGTCGATGCCTATGGCTCGATGACGCCCATCAACCAAGTCGGTACTGTCAATGTGCCGGAGCCGCGTATGGTTACCATCAATGTGTGGGACAAGGGCTTGGTTGGCAAAGTGGAAAAAGCGATCCGCGAATCCGGTTTGGGTATCAATCCGCAGCTGAATGGCACAATCATTATGCTGCCGATCCCCGAACTGAACGAGGAACGCCGCCGCGAGCTGGGAAAAGTTGCCGGCAACTATGCGGAACATGCCCGTGTGTCGGTCCGCAATGTGCGCCGCGACGGCATGGACAAAATCAAAAAAGCCAAAAACGACGGCATGTCCGAAGACGACCAAAAACTTTGGGAATCCGAAGTTCAGGATATGACGAATACCTACATTAAAAAGGTCGATGATCTTCTTGAAACAAAACAAGAAGATATCATGCAAGTTTAAATGGCCAAGAAGCTAGCTGGCACGATGAAGCTGCAGATTGCAGCCGGTAAAGCCAACCCAAGCCCCCCCGTCGGTCCGGCGCTGGGTCAGCGCGGCATCAACATCATGGAATTCTGTAAGGCGTTCAACGCCAAGACGCAGGAAATGGAGCCCGGCGCGCCGTGCCCGACCGTGATCACTTACTATCAGGACAAGTCCTTCACTATGGACATCAAGACGCCACCTGCGTCTTACTACCTGAAAAAAGCAGCCGGCCTGAAGCGCGGTGCTGAGAACCCCGGCCGTGAGACCGTGGCATCGGTAACTGTTGCTCAGGTGCGTGAAATCGCCGAAGCCAAAATGAAGGACCTGTCGGCGAACGACGTCGAGGCCGCAATGTTGATCATCGTCGGCTCCGCCCGTTCGATGGGCATCGAGGTGAAATAAATGTTTGAAAATCTGTCCGAACGCCTGGGCGGCGTCTTTGATCGACTGACCAAGCAAGGTGCGCTGTCTGCTGACGACGTAAAAACCGCAATGCGCGAAGTGCGGGTCGCTTTGCTAGAGGCCGATGTCTCGCTACCGGTTGCCCGCCAGTTCATCAAAGCCGTCGAGAAAAAAGCGACAGGCGCGTCCGTGACCAAATCGGTCACACCCGGTCAGCAGGTTGTCAAAATCGTCCATGACGAATTGATCGCCGTACTGGCAGGTGAGGGTGAGCCCGGTTCGCTCAAAATCGACAATGCCCCCGCACCGATCTTAATGGTTGGCCTACAGGGGTCTGGTAAGACCACGACCACTGCCAAACTGGCCAAGCGCCTGACAGAAAGAGACGGCAAAAAAGTTCTGATGGCCTCGTTGGATGTCAATCGTCCGGCGGCGATGGAACAGTTAGAGATCTTGGGCAAGCAGATTGGGGTAACCACGCTACCCATCGTCAAAGGCGAAGATCCGGTTGCCATCGCCAAGCGTGCAAAAACGCAGGCGTCTTTGGGTGGCTATGACGTCTATATGCTCGATACTGCTGGCCGTTTGTCCATCGATGAAGAATTGATGGCGCAGGTTGAAGCTGTTCGCGATGTCGCCAATCCGCGTGAAACGCTGCTGGTCGTCGACGGCCTGACGGGCCAGGATGCCGTGCACACCGCGGAAAACTTTGATGACCGTATCGGTATTTCCGGCGTTGTACTGACCCGGATGGATGGCGACGGTCGTGGCGGTGCAGCCCTGTCCATGCGCGCCGTTACCGGCAAGCCGATCAAGTTCGTCGGCCTTGGCGAAAAAATGGAAGCGCTTGAAACCTTTGAGCCAGAGCGCGTTGCCGGCCGTATTCTCGGCATGGGCGATATCGTGGCTTTGGTCGAAAAGGCCCAAAGCACGCTTGAGGCCGAGCAAGCCGAACGAATGATGAAGCGGTTTTCAAAGGGTCAGTTCAATATGAACGACCTCAAGATGCAGCTTGAGCAGATGATCAAGATGGGCGGCATGGAGGGCATGATGCAAATGATGCCCGGTATGGGCAAAATGGCCAAGCAGGTTGGCGATGCCGGTATGGACGACAAAGTGCTGAAACAGCAAATCGCGCTGATTCAATCCATGACCAAACGCGAACGTGCCAACCCACAGCTTTTGCAGGCCAGCCGCAAGAAACGCATCGCAGCCGGTGCTGGTATGCAGGTGTCGGACCTGAACAAGCTGCTGAAAATGCAGCGGCAAATGTCTGATATGATGAAGAAAATGGGCAAGATGGGCAAAGGTGGCATGCTGAAGCAAGCCATGAAGGGGATGATGGGCAAGGCTGGAATGGACCCGTCC------------CAGATGGACCCTGCTGCAATGGAAGCGGCTGCCAAACAGCTGGGTTCACGCATGCCCGGCGGCCTTCCCGGTATG---GGCGGCATGGGCTTGCCCGGCGGCTTGTCTGGTTTGGGAAAAAAGAAGTAAATGATCCCATTTGAACGCTTGCAGAAAATCAAAGAACGTCTTGAGTATGTTGAGGCCCGCATGGCCCTCGGCGAA------GGCGATATTGCGCAGCTTGGCCGTGAATATTCAGAGCTGAAACCGGTTGTGGACCAGATCCTTGACTGGGAACGTCTGGTTGCCGATCTCGCTGAGGCCGAAGCGATGCTGGATGATCCAGAGATGCGCGAACTTGCTGAAGAAGAACTGCTCGGCCTGCGCGAACGCCTGCCAGAGGTCGAACACGCGGTTCAGTTGTCTTTGTTGCCCAAAGACGCAGCAGACGCCCGCCCGGCTATGCTGGAAATTCGTCCCGGAACCGGCGGCGATGAGGCGGCCTTGTTCGCCGGTGACCTCGCGCGGATGTACCAACGCTATGTTGAGCAACACGGCTGGCGCTGGGAAGTCATCGAAGAGGCGACATCAGAGCTGGGCGGTATCCGAGAGCTGGTGGTGCGTATCAAAGGCGACGGTGTTTTTGCGCGGCTCAAGTTTGAATCCGGTGTGCATAGGGTGCAGCGGGTTCCGGAAACGGAATCGGGGGGGCGAATTCACACCTCGGCAGCCACGGTTGCGGTACTACCCGAGGCCGAGGAAGTTGATATTGCGATCAATCCCGGCGACATTCGGATCGATACGATGCGTGCCTCCGGGTCTGGAGGGCAGCACGTGAACACCACGGATTCGGCCGTGCGGATAACCCACATGCCTTCCGGGATTGTTGTCACCAGCTCGGAAAAATCACAGCACCGGAACCGCGATATTGCAATGCAGGTTCTGCGGGCGCGCTTGTTTGATCTGGAACGCCAGAAGGTTGCGGATGAGCGATCTGCGCACCGCAAATCGCAAGTCGGGTCCGGAGACAGGTCCGAGCGAATCAGAACCTATAACTTCCCGCAGGGGCGTCTGACCGATCATCGCATCGGGCTGACCCTGTACAAGCTTGACCAGATCATGCAGGGCGATCTGGATGACATCATCGATGCGTTGACAGCGGAGCATCAAGCCGCGTTGTTGGCGGAATTGGGGACTTAAGTGGATAGAGCACAAAAAGAGAAGTTGGTCGACGAGCTCGGCCAGATCTTTGACAGCTCTGGCGTCGTAGTGGTTGCCCGCTACGAGGGTCTGACAGTTGCAGACATGCAGAGCCTGCGCGGGATTGCCCGTGAAGCGGAAGCTTCGGTTCGTGTCGCCAAGAACAGGCTTGCCAGAATTGCCGTTAAGGGCACTCAGTGCGAAAGCATCGATGAGTTCCTGGGCGGTATGACCGTTCTTACCTTCTCTGAGGACCCCGTGGCAGCTGCCAAGGTCGTCGAAGACTTCGCCAAGACGAACAAAAAGTTCGAAATTCTTGGTGGTGTAATGGGTGGAACGGCTCTTGACCGGGCTGGTGTTGCGGCCGTGTCGAAAATGCCTTCGCGTGACGAGCTTATTGCTTCGATCGTTGGCTGCATCGGCGCACCTGCATCTAACATCGCCGGCGCAATTGGCGCACCTGCTTCTAACATCGCATCCATTCTCTCGACTATCGAAGAGAAAGCGGAAGCGTAAATGAAAACCTTCTCTGCTAAACCAGCAGATATCGAGAAGAAGTGGATCATCATCGACGCCGAAGGCGTGGTGCTGGGCCGTCTTGCCTCGATTATCGCTGTTCGCTTGCGTGGCAAGCACAAGCCTTCTTTCACGCCTCACATGGATTGTGGTGACAATGTCATCGTCATCAATGCTGAAAAAGTACAAATGACCGGCAAGAAGCGCGAAGAAAACTTCTACTGGCACACCGGTCACCCCGGTGGGATCAAATCGCGCACCAAAGCCGAGATCCTCGAAGGCAAGCATCCTGAGCGCGTTGTCACTCAGGCGGTCAAGCGTATGCTGCCAGGCAACCGCCTGTCGCGTCAGATCATGACCAACCTGCGCGTCTATGCCGGCGCCGAGCACCCCCACGAGGCCCAGAGCCCCGAAGTGCTTGACGTCAAATCGATGAACAAGAAAAACACGCGGGTA---TAA---------------------------------ATGGGCGGTCTGTTGGGGCCGGATTTTCCCGGTGACATCGGTCTGGCCGTGTCTGGTGGCGGCGACAGTATGGCGATGCTGACCCTTGCGCACAACTGGACACGTGTCTGGGGGGTGCGGTTATGGGTTGTCACGATAGACCACGGGCTACGCCGCGAAAGCGCGACCGAGGCCGCGATGGTCGCGCAGGAATGTGCGGCGCTTGGCTGGCCACATGCAACGGTGAAATGGCAT---TGGGACGGCTCAGGGAATTTGCAGGATGCGGCCCGTCAGGCCCGATTGGACCTGATTGATCGCTGGCGCGGTGTG------TTGCGCCATGTTTTGATGGCCCATACCAAGGATGATTTGTCAGAGACATTTTTGATGCGTTTCAAGCGCGGTTCGGGCGTCGATGGATTGTCGGCGATGCGAGATGAAATGCAATCGGACAGCTTTGAAGTGTTGCGCCCCTGTTTGTCGATGCGTCGCGAAGACCTGCGCCACTATCTGACTGTGCTCAAGACCCCCTGGGCCGAAGATCCCTCAAATCTTGATCCCAAATATGAACGTGTGCGCGTGCGGCAGTCCTTGCCGGACTTGGACAGGCTTGGCCTTAGCGTTGATTTGATTGCCGAGACTGCGAAAAGGTTGCGTCGCGCACAAGAGGCATTGCAGCAACGTGCGGTGCAGGTCTGGCGCGACATTGGCCATGCGCGAACAGGCGACATTCTATTAACCCGATCAGGTTTCGATGGCGTTGAACGGGAAACGCAACTTCGGCTCTTGGCAGCTGGGCTTCAATATGTGTCCTCGGCACCGTACCGGCCACGGGCAGAGCCTCTAGAAGCGCTGCTTGATCGGCTGTTGGGCGGCGGCGGCGGCACCTTGCACGGATGCGAATGTCGGGCGGAAAAGGAACAATTGCGTATTTTTCGGGAAGAAAAACCCCTGAAGAATCTGACCGATCCGCAACACGGGTTTTGGGATCAAAGGTGGCGAATCCCTGCACCGCTG------CCTGACGGCGCGCAGATTCGCAATCTGGGTGATGAGGGATGGCGATTGATTGAAAATAAGCGGGATACCGCAATACCTTATCATGCAGCGCGCAGCTTGCCTGCCGTTTGGAAAGGTGATGTTTTATTGGCCTGTGACGCATTTGGCGTCGGGCCGGGGGGGGGCTTGGCGTTATGGCGTGCGGGGCACAGCTTTGCCCGCTTCCTTCTTTCGCATTGAATGGCTGATCTGAAAGCACTTGCTGAAAGCATCGTGGGTCTGACCCTGCTGGAAGCACAAGAACTGAAAACTATCCTCAAGGACGAGTATGGCATCGAGCCCGCAGCTGGCGGCGCAGTTGTTATGGCTGGTCCAGCAGACGCTGGCGAAGCGGCTGAGGAAAAAACTGAATTTGACGTGATCCTGAAGGCCGCTGGCCCTAAGAAAATCAACGTCATCAAAGAAGTCCGCGGCATCACCGGTCTGGGCCTGAAAGAAGCTAAAGAGCTGGTTGAAGCCGGCGGCAAAGTCAAAGAAGGCGTTTCCAAGGACGAAGCAGAAGAGCTTAAGAAAAAGCTCGAAGAAGCTGGCGCTGAAGTCGAAGTTAAGTAAATGTCTCGTATTGGGAAAAAACCGGTTGAGCTGCCTTCTGGTGTGACAGCATCTGTTTCGGGTCAGGTTGTTGAAGTTAAGGGGCCGAAAGGCGTCCAGACTTTCACTGCCACCGATGATGTTTCAATCACCGTTGAAGACAGTGCCGTAAAAGTTGCACCGCGTGGCAAGTCAAAGCGCGCGCGTCAGCAATGGGGTATGTCCCGCACAGTGGTGGCCAATTTGGTCCACGGTGTGCAGAACACCTTCAAGAAAGAGCTTGAGATCCACGGCGTTGGTTATCGTGCAGCTGTTCAGGGCAAAACCCTGAAGCTGAACCTTGGCTACTCGCATGACGTGGACTTTGCTATCCCTGAGGGGATCAGCATTGTGTGCGCCAAGCCTACTGAAGTTGTGATCGAAGGTCACGACAAGCAGCAGGTTGGTGAAGTGGCGGCTAAGATCCGCGACTGGCGCCGCCCCGAGCCCTACAAGGGGAAGGGTATTCGCTACAAAGGCGAATTCATCTTCCGCAAAGAAGGCAAGAAGAAGTAAATGAAAGTTATCCTTCTGGAACGTGTGGCCAAATTGGGCCAAATGGGCGAAGTCGTTGACGTCAAAGCCGGTTACGCACGTAACTTCCTGCTGCTGCAAGGCAAAGCACTGACCGCCTCGAAAGAGAACATCGCTCAGTTCGACGCCCAAAAAGCACAGCTTGAAGCGCGCAACCTGGAAACCAAAAAAGAAGCCGAAGCGCTTGGAGAGAAGCTCGATGGACAGCAGTTCATCGTGATTCGTTCTGCGTCTGACGGCGGCAACCTGTATGGTTCGGTCACCACCCGTGACGCGTCCGAAGTTGCCACCGAAGAAGGCTTCTCGATTGATCGCAAGCAGGTTGTGATCATCGATCCAATCAAAATCTTGGGTCTACACACCCTTGATGTTGTTCTGCACCCCGAAGTGACCGTTCAAATCCAAATGAACGTTGCACGCTCTGTTGAAGAAGCAGAACTGCAAGCCTCGGGTAAGTCGATCCAAGAACTGGCCGCTGAAGAAGAAGCGCAAGCTGAATTCGAAGTGTCCCAGTTGTTTGACGATTTGGGCTCCGCCGCC------GATGAAGATGGCGACGACGCTCCGGCTCAAGACGCTGAAGAGACATCCGAAGAC---------------TAAATGAGCGATAACGACGGTAAAAAGACATTGGGTTTGCGTGGTGGGGCCCGTTCGGGCAACGTGAAGCAGAGCTTCAGCCACGGGCGCACCAAGAACGTCGTGGTGGAAACCAAGCGCAAGCGCGTTGTGGTCCCCAAGCCGGGTGCTGCTGGCGGCAGAGGCCCTGGCGTAGGTCCCGGTAGCGCAGGCGGCAAACGCCCTGCGGGCATCACTGATGCCGAAATGGAACGTCGTCTCAAGGCGTTGCATGCTGCTAAGGCACGCGAAGCTGAAGAGACAGCGGCGCGCGCGGCAGAAGAAAAAGCACGCGAAGAAGAGCGTAATCGCAAGCGCGCTGAAAACGAAGCGAAGGCCAAAGAAGCGGCCATCGCCGAAGAGCGCGCGCGGCAAAAAGCCGACGACGAAAAGCGTAAAGTTGAGGAAGCCGCAGCAGCCGCCAAACGTGCAGCTGCTCCTGCTGCTCCGCCAGGTGACGATGCGCCGAATCGCAACGTCAACAAGCCGGAAGCGACTCCGCGCCGTGACGATCGTGGCAACGATCGCAACGCACGCAACACACGTGGCCGCGACGACAACCGTCGCTCGGGCAAATTGACGCTGAATCAGGCTCTTGGGGGTGGT---GGCAACCGTCACAAATCCATGGCTGCGATGAAACGTAAGCAAGAGCGTGCGCGTCAAAAAGCCATGGGCGGCCAGCAGGCGCGTGAAAAGGTTGTGCGTGACGTTCAGCTGCCAGAGGCAATTACGGTCGCCGAACTGGCGAACCGGATGACCGAAAAAGTCGGTGACGTGATCAAAGCACTGATGGCCAATGATATTATGGTCACTCAGAACCAATCAATTGACGCAGACACTGCGGAATTGATCATCGAAGAATTTGGTCACAAAATCACACGTGTTTCCGATGCTGACGTTGAAGACGTGATCCAGCAAGTGACAGACGATGCCGCGGACCTGAAGTCTCGTCCGCCGGTCATCACCATCATGGGCCACGTTGACCACGGTAAAACCTCGCTTTTGGACGCGATTCGCGACGCAAAGGTGACAGCAGGCGAAGCCGGCGGGATCACCCAGCACATTGGTGCGTATCAGGTGACCACTGACAACGGCAGCGTTCTCAGCTTCCTCGACACCCCCGGCCACGCGGCCTTTACTTCGATGCGCTCTCGCGGTGCTCAGGTAACGGATATCGTGGTTCTGGTTGTTGCGGCGGACGACGCTGTGATGCCTCAGACTGTTGAAGCGATCAATCACGCCAAAGCAGCGAACGTACCGATGATCGTGGCGATCAACAAAATCGACCGCCCGGCCGCCAACCCTACCAAAGTGCGCACAGACTTGTTGCAGCATGAAGTTGTGGTGGAACAAATGTCTGGTGACGTTCAGGACGTAGAAGTCTCGGCCATTACCGGCCAAGGCCTTGATCAACTTCTTGAAGCCATCGCGCTTCAGGCGGAGATCTTGGAGCTTAAGGCCAACCCTGATCGCAACGCCGAAGGCGCGGTGATCGAGGCGAAGCTGGATGTTGGCCGCGGCCCTGTTGCCACGGTTCTGGTTCAGAACGGTACGCTGAAACTGGGTGACATCTTTGTTGTCGGTGAGCAGTACGGTAAGGTCCGTGCGCTGATCAACGATAAAGGCGAGCGCATCAAAGAAGCCGGTCCTTCGGTTCCTTGTGAAGTTCTTGGCCTTAACGGCACTCCAGAAGCAGGCGACGTGTTGAACGTTGTTGAAACTGACGCACAAGCCCGTGAAATCGCGGAATACCGTGCAAACCTTGCCAAGGAAAAGCGCGCCGCAGCTGGTGCCGCAACCACCTTGGAGCAGCTGATGCAAAAGGCCAAAGAAGACGAGGATGTTTCCGAACTTCCCGTTTTGGTCAAAGCAGACGTTCAAGGGTCGTCCGAAGCCATCGTTCAGGCGCTGGAAAAAGTCGGCAACGGCGAAGTACGGGTGCGCGTTTTGCATGCCGGCGTTGGTGCGATCACCGAATCCGATGTCAGCCTTGCTGAAGCATCGGGTTGCCCGGTCATCGGCTTTAACGTTCGTGCAAACGCATCGGCCCGTAACTCGGCCAACCAAAAAGGTGTCGAAGTGCGCTACTATAGCGTTATCTACGACCTTGTGGACGACATCAAAGCGGCAGCCTCTGGCCTACTTTCGAATGAAATCCGCGAGAACTTTATCGGCTACGCGACCATCAAGGATGTGTTCAAAGTAACCGGTGTTGGCAAAGTTGCAGGCTGTCTGGTGACCGAAGGGGTTGCACGCCGCAGCGCCGGAGTTCGCCTGTTGCGCGACAACGTGGTTATCCACGAAGGTACGCTTAAGACTCTCAAGCGCTTCAAGGATGAAGTGTCCGAGGTTCAGTCTGGTCAAGAGTGCGGTATGGCATTCGAGAACTATGACGATATCCGTGCCAACGACGTGATCGAAATCTTCGAACGCGAAGAGATCACACGTACACTGGACTAAATGGCCAAAGTTAAAAAAGCTCCCCGCCCCAAGGCACAAACGCCCAAGGGATTCCGCGATTATTTCGGCACCGAGGTGACCGAACGCGCAGACATGCTCAAAACGATTGCCGAGGTCTACCATCAATACGGTTTTGATGCGCTGGAAAGCTCGGGTGTTGAAACAGTCGAGGCATTGGGCAAGTTCCTGCCAGACGTGGATCGCCCCAACGAAGGTGTGTTCGCTTGGCAA------GACGAAGATGATGCC---------TGGATGGCGCTGCGCTATGATCTGACCGCGCCTTTGGCCCGCGTTTATGCGCAATATCGTAATGATCTGCCGCTGCCTTACCGCCGCTATGCGATGGGGCCGGTTTGGCGCAATGAAAAGCCCGGACCCGGCCGCTATCGCCAGTTCTATCAATGCGATGCGGACACTGTGGGATCAGCGAACGTTGCGGCCGATGCCGAGATCTGTGCGATGTTGTCGGATACGCTTGAACGTGTTGGAATTCCGCGCGGCGACTATCTGGTGCGTGTCAACAACCGCAAAGTTCTGAACGGTGTGCTGGAAACCATGGGCTTGTCC---GATGAC------GCTCAACGTGACGCGGTTTTGCGTACCATCGACAAATTTGACAAGGTTGGCGAAGCAGGCGTGCGCGAATTGCTCGGCAAGGGGCGCTTGGACGCCTCGGGGGCCTATATCGACGGTGTCGGTCTGTCAGTGGATCAGGCGGAGCCGGTTGTGGCTTTCCTGACCTCCAAAAGCAGTGATGTTGCTAAAACCTTTGCCAATCTGCGCGATGCTATCGGGGCCAGCACAATTGGTGCCGAAGGCGTGGGCGAGCTGGAAAAGATCGGAGATCTTTTGGCGGTGCAGGGCTATAACGCAGACCGGATCGAGATCGACCCAAGCGTGGTGCGCGGGCTGGGGTACTACACCGGTCCTGTGTTCGAGGCTGAACTGACTTTTGAAATCCTTGATGAAAAGGGTCGCAAGCGCCAGTTCGGGTCTGTTGCGGGCGGCGGTCGATATGACGATCTTGTCAAACGGTTCACCGGTCAGGCTGTGCCTGCTACGGGTGTATCTATTGGTGTTGACCGCCTGTTGGCAGCGCTGCGCGAAAAGGGCCGGATTGGCGGCACAGTCCAAGGACCTGTTGTCGTCACTGTTATGGATAAGGACCGGATGGCCGACTACCAAACGATGGTGGCCGAATTGCGCAATGCCGGTATCCGCGCCGAAGTGTATCTGGGCAATCCCAAGAATTTTGGCAACCAGCTGAAATATGCAGACAAACGCGAAAGCCCGATTGCAGTGATCGAAGGCGGCGATGAACATGCCAATGGTATGGTGCAAATCAAGGATTTGATCCTTGGTGCGAAAATTGCCGAAAATGCGACGCTCGAAGAATGGAAAGAGCGCCCTTCGCAATATGAGGTTCCTCGTGACCAATTGGTTGCCAAAGTGCGCGAAATTCTGGACTTGAACCCATGAATGGACGATCTGCGCGACAAGTATCTGACCCTAATAAATGACGCTGGCGACGAAGCCGCGCTCGAAGAGCTGCGTGTGCAGGCCGTCGGTAAGAAGGGCGAGATTAGCCTGCAAATGCGGTCTTTAGGGAAAATGTCCCCTGAGGAACGTCAAGTTGCGGGCCCGGCGTTGAATGCACTCAAAGATGAAATCAACGCTGCCTTGGCCGCCAAAAAATCTGCGCTTGCGGACGCTGCCCTTGACGCCCGTCTGGCCACAGAATGGCTGGACGTAACACTGCCTGCACGGCCACAGCGCCAAGGGTCAATCCATCCGATCAGTCAGGTGACCGAAGAGGTCTCGGCCATCTTTGCCGACATGGGATTTGCAGTTGCCGAAGGTCCTCAGATCGACACAGACTGGTATGTGTTTGATGCACTGAACATCCCAAGCCATCATCCGGCCCGCGCCGAGATGGACACCTTCTATATGAGCCGCGCGGAAGGTGACGACCGCGCACCTCATATATTGCGGACCCACACATCCCCGGTGCAAATTCGGTCGATGGAAAAGCAAGGGGCACCAATCCGCATCATCGCACCCGGCCGTGTCTATCGCGCCGATTATGACCAGACGCACACGCCAATGTTTCATCAAGTCGAAGGTCTATGCATTGGCAAAGACGTTTCAATGGCAAACCTGAAATGGTGCCTCGAAGAATTCGTGAAAGCGTTCTTCGAAGTCGACCATGTGGAACTACGTTTTCGCGCATCACATTTTCCTTTCACGGAACCCTCGGCTGAGGTCGACATTCAGTGCAGTTGGGAAAATGGCGTTCTGAAGGTTGGCGAAGGCGACGATTGGATGGAAATCCTCGGCTCTGGCATGGTGCATCCAAAGGTTTTGGAGAATGCAGGTGTTGACCCCAAAGAATACCAAGGGTTCGCCTTTGGGATCGGGATTGACCGGTTGGCAATGCTCAAATACGGCATTCCAGATTTGCGGGCATTCTTCGATTCAGACCTGCGCTGGTTGAAGCACTACGGGTTCACACCACTCGATGTCCCGACATTGCACGGCGGTCTCAGCAAATAAATGGAAATGGCCGAACAGGCCGGGCTGGACCTCGTAGAAATTTCACCCAACGCCAATCCACCGGTCTGTAAGATCATGGACTACGGCAAGTTTAAGTACGAACAGCAAAAGCGTGAATCCGAAGCGCGTAAAAAGCAAAAGACTATTGAAGTCAAAGAGGTAAAGTTCCGTCCCGGGACCGATACTCATGACTACGACGTAAAAATGCGCAATGTTTTCAAGTTTCTTGAAGGCGGCGACAAAGTCAAAGTGACTTTGCGGTTCCGTGGCCGTGAAATGGCCCACCAACAGCTTGGACGTGAGCTGCTGGAGCGTGTTGCCGGTGATGTCAAAGAGCTTGGCAAAATCGAGAACATGCCGAAAATGGAAGGCCGTCAAATGGTCATGATGATCGGGCCGCTTCCCAAGTAAATGAAATTCACTTTCTCCTGGCTTAAAGACCATCTCGACACCACAGCCTCGGTTGATGAGATTGCCGAAGTGCTGACCGACCTTGGGCTTGAGGTCGAGGGCATCGAAAATCCGGCGCAAGCGCTGGCTGGCTTTACCCTTGCCAAAGTTACCCACGCCGAGCAGCATCCAGACGCGGACCGTCTGCGTGTCTGCACCGTGGCGACGGACGAAGGCGACAAACAGATCGTTTGCGGCGCGCCCAATGCTCGTGAAGGGATCACCGTGGTTCTTTGTAAGCCCGGTGATTATGTTCCCGGCTTGGACATCACGCTAAGCGTCGGCAAGATCCGCGGTGTCGAAAGCCACGGCATGATGGCGTCGATGAAGGAACTGCAGTTGGGGGATGACCACGACGGGATCATCGAACTGCCATCCGGCGATGTCGGAACAAAATTTGTCGATTGGCTGGCGGCCAATATGCCCGAAAAAGTTGATCCGGTGATCGAGATCGCGATTACGCCAAACCGTCAGGATGCGTTGGGCGTACACGGCATTGCCCGCGATTTGGCGGCACGCGGTTTGGGCAAGCTCAAACCGCTCAAGGTCGAAGCGGTTGAGGGCAGCTTTGAAAGTCCGATCACTGTGACGATTGATGAGGATACACGGGACGGCTGCGAAGTTTTTGCCGGACGTATGATCAAAGGGGTGAAGAACGGACCTTCGCCAGAATGGCTGCAACAGCGGCTCAAGGCGATTGGCTTGCGCCCGATTTCTGCCCTTGTGGATATCACCAACTTTTTCACCTTTGATCGCAACCGTCCATTACACGTGTTTGATGCGGATAAGGTTCGGGGCAACCTGCGCATTCATCGCGCAACAGCCGGCGATACGCTGGTTGGGCTGGATGAAAAGACTTATACCTTTGGAGAAGGTCAGGTCGTGATCTCGGACGACTCCGGCATCGAAAGCATCGCTGGCATCATGGGCGGCTTGGCGACGGGCTGCACCGAAGAAACCACAAATGTTTTTCTTGAGGCTGCCGTCTGGGATCACATCCAGATCGCCCACACGGGCCGCGCGCTGAAAATCAACTCGGATGCGCGTTACCGCAATGAACGCGGCATCGACCCTGCCTACAACATGCAGGCGATCGAAGATGCGACGCAGATGATCCTTGATCTTTGCGGCGGCGAAGCATCGAAAGTTGTCACCGCCGGGCAAGTCCCAGATGTGGCGCGCAGCTATCGTCTGGATGCCAAACGGGTGGTTTCGCTGGTTGGCATGGAAATCCCCGAGGCAGAGCAGCGTCAGACGCTGACCGCGCTAGGGTTCCGGATGGAAGGCGATCAGGCCCATGTGCCCAGCTGGCGTGCAGACGTCAAAGGCGAAGCCGATCTGGTTGAGGAGGTGGCGCGTATCGCCTCGCTGACCAAATTGGTGGGTAAACCTTTGCCGCGCCTGCAAAACGGTGTTCCAAAGCCGATCTTGTCACCCATTCAAAAGCGCGAGCGGACCGTCCGGCGGACTGTGGCGACGCTGGGGTACAATGAATGCGTGACCTATAGCTTTATTGATCAAGCCGCTGCGGCCCTGTTTGGTGGTGGCGATGATGTGACAAAACTAGCGAACCCGATTTCATCAGAGATGAGCCATATGCGCCCGTCACTGCTGCCCGGTCTTTTGCAAGCTGCGGCCCGTAATCAGGCTCGCGGCTACGCTGATTTGGCTCTTTTTGAAGTTGGCCACGCCTTCCAAGGCGGCGAACCGGGTGAACAGAACCTGCAGGTCGCTGGCCTGTTGGTTGGCAAGTCAGGTCCCAAGGATGTTCATGGCGCAATGCGAAGCGTCGATTTGTTTGATGCCAAGGCAGATGCCGAATCCGCTTTGGGCGCGATGGGTGCACCTGCCAAAGTGCAGATCCTGCGCGGAGGTGAAGGTTGGTGGCATCCCGGACGCCATGGCCAGATTTGTTTGGGACCGAAAAAGGTGTTGGGCGTCTTTGGCGAATTGCACCCCAAAGTGCTTGACGAAATGGGCGTCAAAGGTCCGGCTGTCGCCTTTGTATTGTATCCTGAGCAAGTGCCGTTGCCGCGAAAAACCGGTGCCACACGCGCCGCGCTGACACAGAACGACCTTCAGGCCGTTGAACGCGATTTTGCCTTTGTGGTGGATGCCGATGTTGAGGCGCTGACAGTGGTCAATGCGGCTGCCGGTGCCGACAAAGCCCTAATAGAAGAAGTGCGTGTTTTCGACGAATTTATCGGAGGCAACTTGGGTGAGGGCAAGAAATCTCTCGCTATCTCAGTGCGTTTGCAGCCGGTTGGTCAAACGCTGAAAGAAAAAGATATCGAAGCGGTCGCCGCCAAAATCATCGCGAAAGTGAGCAAAGCAACTGGCGGCGAATTGCGCGGGTAAATGCACGCCTACCGGAGCCACACCTGCGCCCAACTGACCAAGGACAATGTGGGCGATACCGTTCGCCTTTCGGGCTGGGTGCATCGCATCCGTGATCACGGCGGTGTGCTGTTTATCGATTTGCGCGATCATTACGGTATCACTCAGGTCATTGCGGACGCTGACAGCCCTGCCTTTGCGGATCTGGAAAAGGTGCGGTCCGAATGGTGTATCCGCGTTGATGGCAATGTGCTTGCGCGCGACGAAAGCCTTGTGAACCCGAACCTGCCCACAGGCGCTATCGAAGTGTTTGCCCGTGAGTTGGAAGTCTTGAGTGCAGCCGCCGAGCTGCCGTTGATGGTTTTTGGCGATCAAGAGTATCCCGAAGAAACGCGTTTGCGTCACCGCTATCTCGATCTGCGCCGTGAAGTGATGCAAAAGGCGATGACCCTGCGGTCTGACGTTGTTTCCAGCATGCGCAAGCGGATGTGGGATCTGAACTTTCGCGAATTCCAGACGCCGATCATCACAGCGTCGTCGCCCGAAGGCGCGCGCGACTTTCTGGTGCCATCGCGTTTGCATCCGGGTAAATTCTATGCGTTGCCGCAGGCGCCTCAGCAATTCAAACAGCTGATCATGGTGTCTGGTTTTGACAAGTATTTCCAGATTGCACCATGTTTCCGCGACGAAGACCCACGCGCAGACCGGTCGCCCACGGACTTCTACCAGCTCGACCTGGAGATGAGCTTTGTTGAGCAGCAAGACATCTTTGACACCATCGCGCCGGTTCTGGCAGGTGTGTTCGAGGAGTTTGGCGAAGGTAAAAACGTTGATGCAGACTGGCCGCAGATTTCCTATCGCGATGCTGCGAAGTGGTACGGCAGCGACAAGCCCGACCTGCGTAACCCGATCAAGATGCAAGATTGCTCGGAACATTTCCGTGGTTCCGGTTTTGCGATCTTTGCGAAACTTCTGGAACAAGACGGCACCGAAGTGCGTGCGATTCCGGCCCCCACGGGCGGCGGACGTAAATTCTGTGACCGGATGAACAAGTTCGCCCAAGGCGAAGGTTTGCCGGGCATGGGCTACATCTTCTGGCGCGACAAGACCGGCGAAGAAGTTGGTATGGAAGCTGCTGGCCCGCTTGCGAAGAACATCGGCCCGGAGCGTACCGAAGCAATTCGCCAACAGCTGGGTTTGACCGTCGGTGATGCGGCCTTCTTCCTTGGTGGCAAGCCAAAAGCCTTTGAGAAAATCGCGGGCCGTGCTCGTGATGTGATCGGCGAAGAGCTTGGGCTGACCGACAAAAACCGTTTCGCATTCGCATGGATCGTTGATTTCCCAATTTACGAACAAGACGAAGAAACCGGAAAAATCGACTTTGAGCACAACCCGTTTTCGATGCCACAAGGTGGTGCCGAAGCGTTGGAAGGCAACCCGCTTGACGTGCTTGGCTATCAATATGATCTGGCCTGCAACGGCTATGAATTGCTGTCCGGCGCAATTCGGAACCACAAGCCCGAGATCATGTTCAAAGCCTTTGAAATCGCGGGCTACGGCAAAGATGAGGTCGAAAAGCGTTTTGGGGCTTTGGTCAACGCGTTCCAATACGGCGCTCCGCCGCACGGTGGCTGCGCTGCTGGTATCGACCGCATCGTTATGCTTCTGGCTGATCAGCAGAACATCCGCGAAGTCGTGATGTTCCCAATGAACCAGCGCGCCGAAGACTTGATGATGAATGCACCATCTGATCCGATGTCAGACCAAATGATGGAATTGGGCCTGCGCGTCATTCCGCAGGATTAAATGACTCCACTTAATCGCATCCGCAACTTCTCCATTGTCGCCCATATCGACCACGGGAAATCCACCCTGGCCGACCGGTTGATTCAATCCACCAACACTGTCGCCGAACGGGACATGCAAGCACAGTTGCTTGACAGCATGGACATTGAGCGGGAGCGGGGCATTACCATCAAGGCCAACACCGTTCGCATCGATTATACGGCGAACGACGGGCTCGACTACGTTTTGAACTTGATCGACACCCCCGGCCACGTGGATTTTGCCTACGAGGTCAGCCGGTCCATGCGCGCCGTCGAAGGGTCTTTGCTGGTTGTTGACGCCTCCCAAGGGGTTGAGGCGCAAACACTTGCAAACGTCTACACTGCGATGGAAGCCGACCACGAAATTGTACCGGTTCTGAACAAAGTTGACCTACCGGCAGCCGAGCCAGAGCGGATCGCCGAGCAGATCGAAGACGTCATTGGCATTGATGCGTCCGACGCCTGCCTGATTTCGGCCAAAACCGGTATCGGCATCCCGGATGTTTTGGAAGCTATCGTTAAACGCCTGCCGCCGCCCAGCGGTGACCCTGATGCGCCGCTCAAGGCAATGCTGGTCGACAGCTGGTACGACGCCTACCTTGGCGTTGTTGTTCTGGTGCGCATAATGGACGGCACCTTGAAAAAGGGCGACAACATCAAAATGATGCAGACTGACGCAAAATACAGCGTTGACCGCATTGGTGTGTTTCGTCCGCAGATGGAAAACGTTCAGGAACTTGGCCCCGGCGAGATCGGTTTTATCACCGCTCAGATCAAACAGGTGCGCGACACCAAGGTCGGCGACACGATCACCCATGAGAAAAAGGGCGCAACCGAAGCGCTGCCGGGCTTTCAGCCCAGCCAGCCAGTGGTGTTCTGTGGTCTGTTCCCCGTGGACAGCGCCCAGTTCGAAGACCTGCGCGAAGCCATTGAGAAATTGGCGCTAAATGACGCCTCGTTCTCTTATGAAATGGAAACCTCTGCTGCGCTTGGCTTTGGCTTCCGCTGCGGTTTCCTTGGGCTTTTGCACCTTGAGGTTATTCGTGACCGTGTGGAACGTGAATACAACATCGACCTGATCACCACCGCGCCTTCGGTGATCTATCACATCTACCAGCGCGACGGGACCAAGCACGAGCTGCACAATCCCGCCGACATGCCCGATCCGTCGACCATCGACCACCTTGAGGAACCGCGCATCAAGGCAACAATCCTTGTGCCCGATGAATATCTTGGTGACGTGCTGAAACTGTGCCAAGACCGCCGCGGTATCCAGCTGGATCTGACCTATGCAGGTGCCCGCGCGATGGTTGTCTATGACCTGCCATTGAACGAAGTGGTTTTTGACTTCTACGACCGCTTGAAGTCCGTCACCAAAGGGTACGCGTCGTTCGACTATTCCATGGAAGGCTACCGCGAGGACAATCTCGTGAAAATGTCGATCCTTGTGAATGACGAACCTGTTGATGCCCTGTCGACAATGGTTCACCGTGATCGCGCCGAAATGCGTGGCCGTGCGATGGTTGAAAAGCTAAAAGACCTGATCCCGCGCCATATGTTCAAAATCCCGATCCAAGCGGCGATTGGCGGCAAGGTGATTGCCCGCGAAACTTTGTCAGCAATGCGCAAAGACGTGACGGCGAAATGCTACGGCGGTGATGCGTCGCGCAAGAAGAAGCTGCTGGAAAAGCAGAAGGCCGGTAAAAAGAAGATGCGCCAGTTCGGCAAAGTCGACATCCCACAAGAGGCGTTTATTTCCGCGCTTAAAATGGACAATTAAATGAGCCTGCCCCCCGGATTTCTTGATGAACTGCGCAATCGTATCAGCCTGTCCCAAGTGGCGGGCCGCAAGCTTATGTGGGACGCACGCAAGTCCAATCAGGGAAAAGGGGATTTGTGGGCTCCGTGCCCCTTTCATCACGAAAAAACAGCGTCTTTTCATGTGGATGACCGCAAGGGTTACTACTATTGCTTTGGGTGCCACGCCAAAGGCGACGCGATTTCCTTTGTGCGCGAGACCGAGAATGTCAGTTTCATAGAAGCGATTGAAATTCTTGCCACCGAAGCCGGGATGGAGATGCCCAAGGCTGATCCGCGTGCCAAAGAAAAATCTGATCGCCGCACTGTTTTGAGCGAGGTGATGGAGCAAGCGGTCCAGTTCTTCCGCTTGCAGTTGAAGGCGGAGGCAGGGAGCGCCGCGCGGGCTTATCTGGACAAACGGGGACTGTCGCAATCCGCGCGCGACACCTTTGAGATCGGGTTTGCGCCTGCGGGATGGGAAAACCTGCGGGAACATTTGAAGGCCAAGGGGATCGCCCCTGAGCTGATGCTGGCTTGCGGCTTGGTTAAGTCATCGGACAAGGGGCGCGAACCTTATGATGTGTTTCGCAACCGGATCATGTTCCCGATCCGCGATGCCCGCGGCAAGGCGATTGCCTTTGGCGGGCGGGCGATGGACCCCAATGATAACGCCAAATATTTGAACTCTCCGGAAACCGAGCTGTTTGATAAATCACGCAACCTATACAATATGCGCCCCGCCCGCGAGGCGGCGGGGCGCGGTCAGCCGTTGATCGTTGCCGAAGGCTATATGGACGTGATTGCCCTGTCCGAGGCCGGGTTTGGCGCAGCCGTAGCCCCGCTAGGGACCGCTGTGACAGAGCCACAGATGCAACTGATGTGGCGCATGTCCCAAGAGCCGCTCATTGCACTGGATGGTGACAAGGCCGGGCTGCGTGCGGCCTATCGGGTGATCGACATTGCTTTGCCTTTGCTGGAGGCGGGCAAGGGGTTGCGGTTCGCGATCATGCCTGAGGGCAAAGATCCGGATGATTTGGTGCGGTCCGGCGGGCCGGAAGCGGTCCAGAAGGTTTTGGATGACGCCATGCCCATGGTGCGCCTGCTTTGGCAAAATGAGACAGAAGGCAAGGTTTTCGACAGCCCTGAACGCAAAGCGGCGTTGGATAAACTGTTGCGGGCTCGGATCGCTAAAATCACGGACCCCCATCTGCGCCGTCATTACGGCGACGAAATCAAAGAGCTGCGCTATGCTCTGTTTCGCCCCAATCGGCAAAATGCGAGCGGATTTGGCCAG------------GCCGGAAAGTCCGAATGGTCTAATGCGCCTTTGCCCGCCACAGCAGGGGCAAAATCGTCTTTGATGGTGACC---GCAGGCGACAATGCTGAAACCGCGCTGCGCGAGGCGGTTATTTTGCTGTCGGTTCTAGCGACGCCGGATGTGGCAGCAGAATTTGAGGGCCAGTTGGAAAGGATGCCCTGCCATGATCCCGATCATGCGTTGCTGCGAGATGTGATTTTGCGCAACATCGGGGCC---GAAAATGATATGCGTTTTCGCGCAGAGGATGCAATCGGACCCGAAACCCTTGAAACCATGCTGTCGGCGCGCCATGTGGCGGTTGTGCCCTGCATCCGCACGCCCGGAAATCCCGAAATGGCCCGCATGACCGTGGCCGAAGAATTGGCAAAGCTGGATGCGTTGCATGGGTGGCACGTCGAATTGGCCGAAGCACAAGAAGATCTTGAGGATCAGGCTGACGAGGCGTTGACGTGGCGTCTGGCTGAAGCTGCAAAAGTACGTGCGCAGGCCGGACGTTTGAAAGATGAGAACAGTGCCGAATATGACGTCGGCCCCAACGGAGCCTCGATCGATCGAGAAGAACGCAGCGCACTTGATTCGTTGCTTTCGACAATTCGATTCGACAAGGGGCGGGGT------TAAATGTGCGCCGATACCCCCGAGTATAAACAGACCCTCAATCTGCCCAAAACCGATTTCCCCATGCGCGCTGGCCTGCCCAAGCGTGAACCGGCATGGCTGGACCGCTGGGAGAAAATCGGGGTCTATGACCGCCTGCGTGAAAAGGCCGAGCGCGCGCCCTTTACCCTGCATGATGGCCCTCCCTATGCCAACGGCCATCTGCATATCGGCCACGCGCTGAACAAAACCATCAAAGACATGATTGTGCGCAGCCATCAAATGATGGGCCGCGACGCGCGCTACATCCCCGGTTGGGATTGCCACGGCCTGCCGATCGAATGGAAAATCGAAGAGCAGTACCGCAAAAAAGGCAAAGACAAAGACGACGTCAATGTCATCGACTTCCGTCAAGAATGCCGCAAGTTTGCCGAAGGCTGGGTGGATGTGCAGCGCGAAGAATTCAAGCGTTTGGGCATCACCGGCAATTGGGCCGATCCCTATCTGACGATGAATTTCCACGCCGAACGCGTCATCGCCGAAGAATTCATGAAGTTTTTGATGAATGGCACGCTTTATCAAGGCTCCAAGCCCGTTATGTGGTCGCCGGTTGAAAAGACCGCCTTGGCCGAGGCCGAGGTTGAATACCACGATCACAAAAGCCACACGATCTGGGTGCCCTTCAAGGTCCAGAATGGC---------TCTGGCGATCTGGCCACGGCCCGCGTTGTGATCTGGACAACAACCCCGTGGACGATCCCGTCCAACAAGGCTGTCGCCTTTGGCAAAGGCATTTCCTATGGCCTGTACGAAGTCACCGGAACACCGGAAGAATGCTGGTGTGCGGCAGGCGAAACATACATTCTAGCCGATACTTTGGCAGCGGACGTCTTTACCGCAGCCCGCCTCGAAGAAGGCCAGTTCAAGCGCGTGCGCGATGTCGCAGCGGATGAGCTTGACGGCTTGGTTCTTGCCCACCCCTTCAACGGTATGGACGGTGCAGACGGGTATTGGGACTATGATGTTCCGATGATCGACGGCGACCACGTCACCGATGACGCGGGCACCGGATTTGTGCACACCGCCCCCAGCCACGGCCAAGAAGACTATGAATGCTTTGTTGCGCGCAACTGGCTGGACCGG---ATGACACATAACGTCGGCGAGGAATCCGAATTCCTGAGCCACGTTCCGTTCTTTGCGGGCATGAGGGTCTTTGACCAAAAAGGCAAAGAAGGAAAAGCCAACAACGCTGTGATTGCCAAGCTGGTCGAAGCGGGCGGCATCATCGCCCGCGGCCGCGTGGCGCACAGCTATCCGCATTCGTGGCGCTCCAAAGCACCGATCATCTATCGCAACACCCCGCAGTGGTTTGCGTCGGTTGATCGCGAAGTGGGTGACGGGCTGGACACGCATGGCAAAACCATCCGCTCGCGCGCGCTCAATTCGATCGACGAGGTGAAATGGTGGCCACAAACCGGCCGCAACCGCCTGTATTCCATGATCGAAGCGCGCCCGGACTGGGTGCTGTCACGCCAACGCGCTTGGGGGGTTCCGCTAACCTGCTTTACACGCAAAGGCCTGCTGCCCACCGATCCCGATTTTCTGTTGCGCAATGATGCCGTGAACGCCCGCGTTGCGGCCGCGTTTGAAGTCGAAGGCGCAGATTGCTGGTATGCCGATGGGGCAAAAGAACGCTTCCTTGGCAGCGACGTAAACCCCGACGACTACGATCAAGTGTTCGACGTTCTCGATGTGTGGTTCGACAGTGGCACCACCCACGCCTTTGTCTTGCGCGACCGCGAAGACGGCAGCGAAGACGGCATCGCCGATGTCTATATGGAAGGCACCGACCAACACCGAGGTTGGTTCCACTCATCGCTGCTACAAGCCTGTGGCACCTTGGGCCGCGCGCCCTATCGCAACGTGGTCACACATGGCTTTACGCTGGACGAAAAGGGCAACAAGATGTCCAAATCGCTCGGCAATACTATCGTGCCGGAAAAGGTCATCCAACAATATGGCGCGGATATCTTGCGTCTTTGGGTTGCCCAGACCGATTACATGGTCGACCAGCGGATCGGGCCGGAAATCCTGAAAGGCGTGGCAGACAGCTATCGCCGCTTGCGCAACACCATGCGCTATATGCTTGGCGCGCTGAACGATTTCAAACCAGAGCAGCGCGTTCCTGTCGAAGAGATGCCGGAACTTGAACGCTTGATGCTGCACCGTCTTGCCGAAATCGACCATCAGGTGCGCAAGGGCTACTCGGAATTCGATTTTCAGGGCGTTTTCTCGACTGTGTTCAATTTTGCGACGATTGATCTGTCGAGCTTCTATTTCGATATCCGCAAGGACGCGCTGTATTGTGATGGCGACAGCCACCGCGCTAATGCCTGCCGCACAGTGCTGGACATCTTGTTCCACCGCTTGACCACATGGCTTGCGCCAATCCTGGTCTTCACCATGGAAGAAGTCTGGCTAGAGCGCTTCCCCGGCGAGGCCAGCTCGGTCCACTTGATCGATATGCCTGAAACGCCCAAGGATTGGCTGGACGAACCTTTGGCCGCAAAATGGGCCGAGGTGCGCAAGGTGCGCCGTGTCGTGACCGCCGCGCTTGAGGTGCAACGGACGGACAAGGTTATCGGGGCCTCTCTCGAAGCGGCACCAGTTGTGCATGTGCGCGACGCCGATACTTTGGCTGCGCTGAAATCTGTCAACTTTGAAGACATCTGCATCACCTCGGCAATTTCGCTGTCAGGTGATCCACAGCCCGCCGAAGCCTTCCGCATGCCGGAAACCGAAGGCGTGGGCGTGGTGTTTGAAAAGGCCGATGGTGAAAAGTGTCAGCGCTGTTGGAAAATCCTGCCAGATGTCGGACAGCACAAACATGCCGGCACCTGCAAGCGGTGTAACGACGCCTTGGGCTAA------ATGTCTCAGAACATCCGTATCCGGCTTAAAGCGTTTGACTACCGTGTTCTCGACGCCTCTACACAAGAGATCGTCAACACAGCCAAACGTACCGGTGCCACGGTCCGCGGTCCTATCCCGCTGCCCAACAAGATCGAAAAGTTTACTGTTCTACGTGGTCCACACGTAAACAAAAAGTCGCGTGACCAGTTCGAGATCCGTACGCACAAGCGTATGCTCGACATTGTCGACCCGACTCCACAGACTGTTGATGCCCTGATGAAACTCGACCTCGCCGCCGGCGTTGACGTCGAGATCAAAGTA------TAAATGTCAGATCAGATCAATTCTTTGGAAGAGCTGGCCGGTGTTGCA---------------GGTGTTGAAGCTGCAGAAGCCGAAGTTGTCGCTCCTCGTGAGCCCGTTCGTGACGAACTGGGCCGCGCCTATGCAACTGGTAAACGTAAGGACGCCATCGCGCGCGTCTGGATCAAACCAGGTTCCGGCAAGGTAACTGTCAACGGCCGCGAGATGGACAAGTATTTTGCCCGTCCCGTTTTGCAGATGATCCTGCGTCAGCCGTTCCAGATTGCTGGCCGTGAAGGTGAATTCGACGTATATGCAACCGTCAAAGGTGGTGGCCTGTCCGGTCAGGCTGGCGCGGTTAAGCACGGCATTTCCAAAGCCCTGCAACTGTACGAACCCTCCTTGCGCGGCGCGCTGAAAGCGGCAGGCTTCCTGACCCGTGACAGCCGTGTTGTTGAGCGTAAGAAATTCGGTAAGCGCAAAGCGCGTCGTTCGTTCCAGTTCTCGAAGCGCTAAATGCCCACGATCCAACAGCTGATCCGCAAACCGCGCCAGCCTAAAGTCAAACGTTCGAAGTCTCAGCACCTCGAACAGTGCCCGCAAAAGCGCGGCGTTTGCACACGCGTCTATACAACCACTCCCAAAAAGCCGAACTCGGCGATGCGGAAAGTGGCCAAGGTTCGCCTGACAAATGGCTTTGAAGTCATTAGCTACATCCCAGGTGAAAGCCACAACCTTCAGGAACACTCTGTGGTTCTGATCCGCGGCGGTCGTGTAAAAGACCTTCCCGGTGTGCGTTACCACATCCTTCGCGGTGTTCTGGATACCCAGGGCGTCAAAGATCGTAAGCAGCGTCGCTCCAAGTATGGCGCGAAGCGTCCTAAATAAATGGCACGTGATACTCGCCGCGCTAAGAAAAAGGTCTCCAAGAACATCGCTGCTGGCGTTGCTCATGTAAACTCGACCTTCAACAACACCAAAATCCTGATCTCTGACGTACAGGGCAACGCCATTTCATGGTCGTCCGCCGGTACAATGGGCTTCAAAGGCTCGCGTAAGTCGACGCCTTATGCGGCTCAGCTTGCAGCAGAAGATGCTGGCAAGAAAGCTCAGGAACATGGTGTTAAAACTCTGGAAGTCGAAGTTCAGGGCCCCGGTTCGGGTCGTGAATCGGCTCTGCGCGCCTTGGCCGCAGCTGGGTTCAACATCACATCGATCCGTGATGTAACACCTATTGCGCACAACGGCTGCCGCCCGCCAAAGCGCCGCCGCGTTTAAATGAGCGAGAATCGTCGCGGCCTGTTGATTATCTTGTCATCACCTTCAGGGGCCGGAAAATCTACTTTGTCGCGCAGATTGCTGGATTGGGATCCAAGTCTGAGCTTTTCGGTGTCTGCCACCACCCGCGCGCCGCGCCCCGGTGAAGTCGACGGAGAGCACTACCATTTTCTTGAAGAAGACGCCTTTAAAAGGCAGGTCGCGGATGATGGCATGTTGGAACATGCGCATGTGTTCGGGAACTTCTACGGCTCTCCCAAGGCACCGGTGCAAAAGGCCATCGAATCCGGCCGCGATGTGTTGTTTGATATTGATTGGCAAGGCGCGCAGCAGATTGTGAACTCCTCGCTTGGGCAGCACACCTTGTCGGTTTTCATCCTGCCGCCATCGATTGTTGAACTGCGCCGCCGATTGATCGGACGCGGCCAAGACAGTGATGACGTGATTTCCAAACGTATGCAAAAAAGCTGGGACGAGATCAGCCATTGGGGCAGTTATGATTACGTGCTGGTCAATGATGATCTGGACCGGACGTTTGAGGATCTTAAAACCATTGTCACCGCCACGCGCCTGCGCCGTTTGCAACAGCCTAGGCTGGTTGAACATGCGCGCGGCTTGCAATCCGAATTTCAGGAGCTGTCATGAATGGCGCAAAGCTTCCTTGGCCAGAAACGTCTTCGCCGTTATTACGGCAAAATCCGCGAAGTTCTGGAAATGCCGAACCTTATTGAGGTTCAAAAATCCAGCTACGAGCTTTTCTTGAAATCAGGCGACCAGCCTACGCCGTCTGACGGCGAAGGCATCAAAGGCGTTTTCCAGTCGGTATTCCCGATCAAGGATTTCAACGAAACCGCGATTTTGGAATTCGTTAATTACGATCTGGAAAAACCAAAATACGACGTCGAAGAGTGCATGCAGCGCGACATGACCTACAGCGCTCCGCTTAAGGTCACTCTGCGCCTCATCGTATTTGATATCGATGAGGATACCGGTGCGAAATCGGTAAAAGACATCAAAGAACAAGATGTGTTCATGGGCGACATGCCCCTGATGACACCCAACGGTACGTTTGTTGTAAACGGCACCGAACGTGTGATCGTCAGCCAAATGCACCGCTCACCCGGTGTTTTCTTTGATCACGACAAAGGTAAGACGCACAGCTCGGGCAAGTTGCTGTTCGCCTGCCGTATCATCCCCTATCGCGGATCTTGGCTCGACTTCGAATTCGATGCCAAAGACATCGTATTTGCACGCATCGACCGTCGCCGTAAATTGCCGGTGACGACGCTGCTATACGCGCTTGGTCTCGACCAAGAAGCGATTATGGATGCGTATTACAACACTGTGTCATACAACCTGAAGAAAAGCGGTGGCTGGTCCACCAAGTTCTTCCCAAATCGTGTGCGCGGGACCCGCCCGGCCTATGATCTGGTGGATGCCGACAGCGGTGAAGTGATTGCTGAAGCAGGCAAAAAGGTAACGCCACGCGCAGTGAAAAAGCTGATCGACGAGGCCAAAGTTGAGAACCTGTTGGTTCCGTTCGACCAAATCGTCGGCAAGTTTGTGTCACAAGACATGATCAACGAAGAAACTGGTGCGATTTACGTCGAAGCCGGTGACGAGCTGACATGGGAAGTTGACAAAGACGGTGACGTAACCGGCGGTACACTCAAGGAGCTTGTTGACGCAGGCGTCACCGAGATCCCGGTGCTGGACATCGACAACGTCACTGTTGGCCCCTACATGCGCAACACAATGGCGATGGATAAGAACATGGGCCGCGATACCGCGCTTATGGATATCTACCGCGTTATGCGTCCGGGCGAGCCGCCCACCGTTGAAGCTGCTTCCGCCTTGTTCGAGACACTGTTCTTTGACGGTGAGCGTTATGACCTGTCTGCCGTTGGCCGTGTTAAGATGAACATGCGCTTGGCGCTGGAAAAAGAAGACACACAGCGTACGCTGGACCGCGAAGACATCGTCAAGTGTATCAAAGCGCTTGTTGATCTGCGCGATGGTCGCGGCGACATCGACGATATTGACCACCTCGGCAACCGTCGCGTGCGTTCTGTCGGCGAATTGATGGAAAACCAGTACCGTGTTGGTCTGCTCCGCATGGAGCGCGCAATCAAGGAACGCATGTCCTCTGTCGAAATCGACACTGTGATGCCGCAAGACCTGATCAATGCCAAACCTGCCGCAGCTGCGGTGCGTGAATTCTTCGGCTCTTCGCAGCTGTCGCAGTTCATGGACCAAACCAACCCGCTGTCTGAAGTCACCCACAAACGCCGTCTTTCGGCGCTTGGGCCAGGTGGTTTGACACGTGAGCGTGCTGGCTTTGAAGTTCGCGACGTTCACCCGACCCACTACGGCCGGATGTGCCCGATTGAAACACCGGAAGGACCGAACATTGGTCTGATCAACTCGCTGGCGACATTCGCCCGCGTGAACAAATACGGTTTCATCGAAACACCTTACCGTGTCGTAAAAGAGGGCGTCGTCACCGACGAAGTCCACTACATGTCGGCAACCGAGGAAATGCGTCACACCGTGGCTCAGGCGAACGCCAACCTCGATGAAAACATGAAGTTCAAGAACGAATTGGTGTCGACACGTAAATCTGGTGACTACACACTGTCACCCAGCGATGCGGTTGACCTGATTGACGTTAGCCCGAAGCAGTTGGTTTCTGTTGCGGCCTCGCTCATTCCGTTCTTGGAAAACGACGATGCGAACAGGGCCTTGATGGGCTCGAACATGCAACGTCAGGCTGTTCCAACTCTGCGCTCCGAAGCGCCGCTTGTCGGCACCGGGATCGAAGAAGTTGTGGCACGCGATTCCGGCGCGGCTGTTATGGCGAAACGCGCGGGTATCATTGACCAAGTTGACGCGCAGCGTATCGTTGTGCGCGCCACGTCTGACCTGGAAATGGGCGATGCGGGCGTAGACATCTACCGCATGCGCAAGTTCCAGCGTTCGAACCAGAACACCTGTATTAACCAACGTCCGTTGGTCAAAGTGGGTGACACGGTTCTGAAGGGCGAAGTTATCGCGGACGGTCCATCGACAGACATGGGTGAACTGGCCCTTGGTAAAAACGTGATCGTCGCGTTTATGCCTTGGAACGGCTACAACTACGAAGACTCGATCCTGATTTCTGAACGCATTGCCAAAGATGATGTGTTCACATCGGTCCACATCGAAGAATTCGAAGTCGCAGCCCGTGACACCAAGCTTGGCCCAGAAGAGATCACACGCGACATTCCCAACGTTGGGGAAGAAGCGCTGCGGAACCTCGACGAAGCTGGCATCGTGTACATTGGTGCGGACGTAGAGCCTGGCGATATTCTTGTTGGTAAAATCACCCCCAAGGGCGAAAGCCCGATGACGCCAGAAGAAAAACTGCTGCGCGCCATCTTTGGTGAAAAAGCTTCGGACGTGCGCGATACCTCGCTGCGTGTGAAGCCGGGTGACTACGGCACAATCGTCGAAGTGCGTGTCTTTAACCGCCATGGCGTGGAAAAAGACGAACGCGCGCTGCAGATCGAGCGTGAAGAAATCGAACGTCTTGCCCGCGACCGTGACGACGAACTCGCAATCCTTGACCGGAACATCTACGCTCGCTTGAAGGGTATGATCGAAGGTAAAGTGGCTGTTAAAGGCCCCAGAGGTGTACGCCCGAACGCGGAAATCAACGAAGAGTTGCTTGAAACTCTGACCCGCGGTCAGTGGTGGCAGTTGGCGCTTGGTGAAGAAGATGACGCCAAGCACGTCGAAGCCCTGCATGAGCAGTACGAAGTGCAAAAGCGCGCTTTGGATGCCCGTTTCGAAGACAAGGTCGAAAAAGTGCGCCGCGGCGACGATCTGCCTCCGGGTGTGATGAAGATGGTCAAAGTCTTCATCGCGGTGAAGCGCAAGCTTCAGCCTGGTGATAAAATGGCGGGTCGCCACGGGAACAAAGGTGTGGTTTCACGCGTTGTTCCAATGGAAGATATGCCGTTCCTTGCCGATGGTACGCCGGTTGACTTCTGTTTGAACCCGCTGGGCGTTCCATCGCGTATGAACGTTGGTCAGATCCTTGAAACCCACATGGGTTGGGCCTCGCGGATGTTGGGCATCAAGATCGACGATGCTCTGCAAGAGTATCGCCGGTCCGGTGACATGACGCCAGTTCGCGAAGCACTGCGCATTGGGTATGGCGACGACGTCTACGAAGATGGTTTTGCCGACATGTCCGAAGGTGATTTGCTGGAAGCTGCCGGAAATGTGACCTCGGGTGTTCCGATTGCAACACCGGTCTTTGACGGTGCGAAAGAAGCTGATGTGAACGATGCGCTGTTGCGCGCTGGTTTCTCGGAAAGCGGGCAATCGGTACTGTTTGATGGCCGCACCGGTGAGCAGTTTGCACGTGAAGTGACTGTGGGTGTTAAGTACCTGCTGAAACTGCACCACCTTGTGGACGACAAAATCCACGCACGTTCGACTGGACCATACTCGCTTGTTACTCAGCAGCCGCTGGGTGGTAAGGCGCAGTTCGGTGGCCAGCGTTTCGGGGAAATGGAAGTCTGGGCGCTCGAAGCTTATGGCGCTGCATATACCCTGCAAGAAATGCTGACTGTGAAGTCGGATGACGTGGCAGGCCGGACCAAGGTCTATGAAAGCATCGTTAAGGGTGAGGACAACTACGAAGCTGGCGTGCCAGAATCGTTCAACGTTCTCGTCAAAGAGGTCCGCGGCTTGGGCCTCAACATGGAACTCCTGGATGCCGAGGAAGATGAGTGAATGCCGCTATATGAGCATGTTTTCATTTCGCGTCAGGACTTGTCCAACGCGCAGGCTGAAAGCCTAGTCGAACATTTTGGCACAGTGCTTGCGGATAACGGCGGTAAAGTCGTTGAATCCGAGTACTGGGGTGTCAAAACGATGGCCTACAAAATCAACAAGAACCGTAAGGGCCATTATGCCTTCTTGCGCACCGATGCACCTGCACCGGCCGTAAAGGAAATGGAACGCCTGATGGGTCTGCATGATGACGTAATGCGCATCATGACCATCCGCGTCGACGAGCACGCCGAAGGTCCCTCGGTTCAGATGCAAAAACGTGACGAACGCAGCGACCGCCGCGAACGTCGCTAA---ATGTCGGACATTGCGATTGAAAAATTGACAGAAACAGAAGCCCGCGCTGAACTGGCGCGGCTTTCGTTGATTTTGTCCCAAGCAAACCAGGACTACCATCAACGTGATGCACCGGAACTATCCGATGCAGAATACGACCATCTCAAACGTCGCAATGCTGAGATCGAAACCAAATTTCCCCAGTTAATGCGTGCGGATAGCCCGTCTGATCAAGTCGGGTCGCCAGTATCCGATGGCTTTTCCAAAGTTGCACATGCGGTCCGTATGTTGTCGCTTGCAAATGCCTTTGACGATGAAGACGTTAACGATTTTGACATGTCCATTCGGCGATACCTCGGGTTGACCGAGGGGCAGGGTCTCGCTTACACGGCCGAACCCAAGATTGATGGCCTGTCTTTGTCATTGCGTTACGAAAACGGCCAGTTGGTACAAGCTGCCACGCGTGGTGATGGAACCATTGGTGAGAATGTCCTCGCAAATGCATTAACCATAGCCGACATCCCACAAATAATTGCCAACGCGCCTCACATCACCGAAATTCGCGGTGAAGTGTACATGAGCCACGACGATTTTGCTGCATTGAACCAAAGACAAGCAGCGCGGGGAGGGAAACTATTTGCCAATCCTCGCAATGCCGCAGCAGGATCTTTGCGGCAACTTGACTCCGAGATCACTAAAAACAGACCGTTGCGGTTTTTTGCATATTCATGGGGCGAGCTATCGGAACCGCTTTCCGATACTCAAATGGGGGCTGTCGAACGGATGAAAGCCTTGGGGTTTCAAGTGAATCCATTGACCCAGCTTTGCCCAACCCCACAAGCACTGTTGCAGCAATATGCCAAAATTGAAGAACAGCGTGCGACATTGGGTTATGATATTGATGGTGTGGTTTACAAAGTGAACGATCTGGCGTTGCAAGCGCGACTTGGTTTCAGATCGACCACACCAAGATGGGCTATCGCCCATAAGTTTCCCGCCGAATTGGCGTGGACGCGGTTAGAGGCAATAGACATACAAGTTGGCCGGACGGGTGCCTTGTCTCCGGTTGCCCGTTTGACGCCGGTAACAGTTGGCGGTGTCGTCGTGTCAAATGCGACGCTGCACAACGAAGACTATATTGCGGGTCGCGACTCTTTCGGTGGCGAAATCCGTTCCGGCAAAGATATTCGGGTGGGGGATTGGGTCCAAGTTTACCGCGCCGGTGACGTCATTCCAAAAATTGCTGATGTTGACCTGTCCAAACGGCCTGAGGCTGTAGAACCTTTTGCGTTTCCCAAAGTCTGCCCAGAGTGTGGGTCAGATGCCATTCGTGAAGATGGGGACGCAATTCGTCGGTGCTCTGGTGGTATGATTTGTCCGGCTCAAGCTGTCGAGCGCCTGAAACACTTTGTGTCCCGCGCAGTTTTCGACATTGATGGGTTGGGTGCCAAACAGATCGAACAGTTTTACACCGATGGATGGGTGAAAGAGCCCGCAGATATTTTTACGCTAGGCGCACGCTTTAGCACTGGTTTGCAGCAATTGCGAAATCGGGAAGGCTGGGGCGAAAAATCGGCGTTGAACTTGTTTGAAGCCATTGAAACCAGCCGCATAGTCCCGCTTGGCCGGGTGATTTTTTCACTAGGAATCCGTCATGTGGGAGAGAACGCGTCCAATCTTTTGGCGCGGCATTATGGGGCATGGTCGGATTTTGAAAGCGCTATGGTTGCTGCAGCTCCGCAAGAAGGTACCAGTTGGGACGATCTTTTGTCGATAGATGGCGTTGGTGTTGTCATGGCGCAATCGCTGGTGTCGGCGATGAACCAACCGGCAGAACGGGCGTCCATCGATCGATTGATTGCACAATTGACTGTTGAAGATGCCGCAAGACCAGACACAGAAGGCAGCCCTGTCGCAGGAAAAACAGTTGTATTTACCGGCGCATTGGAAAAAATGTCTCGTGCCGAAGCCAAGGCCCGGGCCGAGTCTTTGGGGGCAAAAGTAGCGGGATCTGTATCCAAAAAGACAGACATCGTTGTGGTGGGGCCTGGCGCTGGCAGCAAAGAAAAGAAGGCGCGCGAATTGGAGCTTATGGTACTGGATGAAGATGCTTGGTTGGAGCTGATCGAAAGA---TGAATGATCCACAAGAACTGGGCCGAATTGATCAAGCCAACACAGCTTGAAGTCAAACCCGGCAATGATCCAGCACGCAAAGCCACTGTTGTTGCCGAACCGCTGGAACGCGGCTTTGGTCTGACACTTGGTAATGCTCTGCGCCGCGTACTGATGTCGAGCCTGCAAGGCGCGGCCATCACAAGCGTTCAGATCGACAATGTTTTGCACGAATTTTCATCGGTTGACGGTGTTCGTGAAGACGTTACCGACATCATCTTGAACCTCAAAGGCGTTTCGATCCGCATGGAAGTCGAAGGCCCCAAGCGTTTGTCTGTGACCGCCAAAGGTCCGGGCGTTGTTACCGCTGGTGACATCACCGAAAGTGCTGGCATCGAGATTCTGAACCGCGATCACGTGCTCTGTCACGTGGACGAAGGTGCCGAAGTGTACATGGAATTCACTGTGAACACTGGCAAAGGTTATGTGTCCGCAGACAAGAACAAGCCTGAGGATGCGCCAATCGGACTGATCCCGATCGATGCAATCTATTCGCCGGTTAAGAAGGTCGCTTATGACGTTCAGCCGACACGCGAAGGTCAGGTTCTGGACTATGACAAGCTGACAATGAAGGTCGAAACTGACGGCTCGATCAGCCCCGAAGATGCAGTGGCCTACGCCGCGCGTATCCTTCAGGATCAGCTGTCGATCTTCGTCAACTTCGATGAGCCTGAGTCGGCTCAGCGTCAGGACGATGATGATGGTCTCGAGTTCAACCCGCTTCTGCTGAAGAAAGTGGATGAACTGGAACTGTCTGTCCGTTCTGCAAACTGCCTGAAAAACGACAACATCGTTTATATCGGCGATCTGATCCAGAAAACCGAAGCAGAAATGCTGCGCACACCTAACTTTGGCCGTAAGTCTTTGAACGAGATCAAAGAAGTGCTGTCGGGCATGGGTCTGCACCTTGGCATGGACGTTGAGGACTGGCCACCGGACAACATCGAAGATCTGGCCAAGAAGCTTGAAGACAACTTCTAAATGGCTGAACGTGATAACCGCCGGGGCAACCGTCGCGAC---CGTGACGAAGCACCGGAATTTGCAGATCGCCTAGTCGCGATCAACCGCGTATCTAAAACTGTAAAAGGCGGTAAGCGCTTTGGTTTTGCTGCGCTTGTTGTTGTTGGCGACCAAAAAGGCCGCGTTGGTTTCGGTAAGGGTAAAGCGAAAGAAGTGCCCGAAGCGATCCGTAAGGCGACCGAGCAGGCCAAACGTCAAATGATGCGCGTGCCTCTCAAAGAGGGCCGCACATTGCACCACGACATCGAGGGCCGTCACGGCGCTGGTAAAGTTGTGATGCGCACTGCACCACAGGGTACTGGTATCATCGCCGGTGGTCCAATGCGTGCTGTGTTCGAAATGCTTGGTGTTCAGGACGTCGTCGCGAAATCGATCGGCTCCCAGAACCCTTACAACATGATCCGCGCCACTCTGAACGGTTTGGGCAAAGAACAGTCTCCTCGCAACGTCGCTCAGCGTCGTGGCAAAAAGGTTGCTGATATTCTTCCCAAGCGTGACGAAGCGCCA---------------GTAGCAGAGGAAGCATAAATGAACGATCCTATCGGCGATATGCTAACTCGTATCCGCAACTCGCAGATGCGTGGTAAATCCACCGTTTTGACACCAGCTTCCAACAAGCGCGCACGCGTTTTGGACGTTTTGGCATCCGAAGGCTATATCCGCGGCTACGAAAGTGGCACGGATTCCGCAGGTCACCCGACCTTCGAAATCAGCCTGAAGTATTATGATGGCGAACCAGTTATTCGCGAATTGGCACGGGTTTCAAAGCCTGGCCGTCGTGTTTACATGGGCGTTAAGGAGATCCCACAGGTCCGTCAGGGTCTGGGTGTCTCGATTGTCTCCACCTCTCAGGGTGTGATGTCGGATGCAAAAGCACGCGCAGCCAATATTGGCGGCGAAGTGCTCTGCACCGTCTTCTAAATGCATGACATCCGCGCCATCCGTGACAACCCCGCTGCCTTTGATGCGGCTCTTTCGCGCCGTGGGATCGAAAATGCATCATCTCCGCTGCTGAAACTGGACGCGGAACGCCGTGCTGCGATCTTGGCTGCCGAAACCGCGCAAAGCGATCAAAAGAAAGCCGCCAAGGAAGTCGGTGCCGCCAAAGCCAAGGGCGATGACGCCGAATTCGAACGGCTGCGTGCGCTTGTGTCCGAGAAAAAGGCCGAAGTGGCCGATATGCAGACCCGCGCCAAGGAGCTGGACGAAAAACTGCAGTATGAACTGTCGGTGATTCCGAACCTGCCCTATGACGATGTACCGGACGGTGCAGACGAAGACGACAACGTCGAAGTTCGCACATGGGGCACACCGGCAACGCTCGATTTTGAGGCCAAAGAGCATTTTGAATTGGCGGGCGTTGCCGCAAGCATGGACTTCGAGCTGGCCGCCAAGATTTCCGGCGCGCGATTTGTCATGCTAAAGGGCGCTGTTGCCCGCATCCACCGTGCGCTGGCGCAATTCATGATCGACACGCATGTTGATGAAAACGGTCTGACCGAGATGAATTCGCCGGTGCTGGTGCGCGACGAAGCGATGTATGGCACCGACAAGCTGCCGAAATTCGGCGAAGACAGCTACCAGACAACAAACGGCTGGTGGCTGGTTCCGACCTCCGAAGTGCCTCTGACCTATACCGTTGCAGGCGACATTCTGGACGAAAGCACCCTGCCGATCCGCATGGCTGCGCACACGCTGTGTTTCCGCTCCGAAGCGGGCAGTGCTGGCAAAGACACGTCTGGCATGTTGCGTCAGCACCAGTTTGAAAAAGTCGAGATGGTTTCGATCACCCATCCGTCGCAATCGGACGCTGAGCAGCAACGGATGTTGGGCTGTGCACAGGGGATCCTGGAACGGCTCGACATTCCATATCGCACAGTTGAATTATGCACCGGGGACACCGGATTTGGCGCACGCCGCACCTTTGACATCGAAGCCTGGTTGCCCGGTCAGAACACCTACCGCGAGATCAGTTCGGTTTCGACCACAGGTGATTTTCAGGCACGCCGGATGAATGCACGGTTCCGCCCCGCCGACGGTGGCAAGCCCGAGTTTTTGCACACGCTGAACGGGTCGGGTCTTGCGGTTGGGCGGTGTTTGATTGCGGTTTTGGAAAACGGTCAGCAGGCGGATGGCTCAGTCACGCTGCCCGCTGCACTGGTTCCCTATTTGCGCGGCAAAACCACGCTGACAGCGGATGGCGTGCTGGCCTAAATGGCGATCACTGCTGCACAGGTAAAAGAACTGCGCGAAATCACTGCCGCTGGCATGATGGACGCGAAAAAAGCGTTGGTTGAAACCGACGGTGACATGGAAGCTGCCATCGATTGGCTGCGCACCAAAGGCCTTGCCAAAGCCGCCAAGAAATCGGGCCGCACGGCTGCCGAAGGTCTGGTTGCTGTGGCTGTTGCCGGCGGCAAAGGTGTTGCTGTTGAAGTAAACTCCGAAACCGACTTCGTTTCCAAGAACGCAGAATTCCAGGAAATGGTCGGCGGCATCGCCACCGTTGCTTTGAACGTTGCTGATGTAGAAGCGCTGAAAGTCGCTGATCTGGGCGGCAAGACCGTTGAGACTGTGATCACCGACAAAATCGCCACCATCGGCGAAAACATGTCAGTTCGCCGTATGGCGACAGTCACTGGCGAAACCGTTGTGACCTATGTTCACAATGCAGCCACCGCTGGCATGGGCCAGATCGGTGTTTTGGTTGCGCTGAACGGCGGCAACGAAGATTTTGGCAAGCAGATTGCGATGCACATCGCGGCTGCAAACCCTGCGTCTTTGTCCGAAGCAGATCTTGACCCGGCGGTTGTTGAAAAAGAACGCCAGATTCAGATCGATATCGCACGCGAATCCGGCAAGCCAGAGCAGGTTATCGAAAAGATGATCATCGGCCGCATGAAGAAATTCCTCGCCGAAGTGACTTTGCTGGGCCAGCAATTTGTTGTGAACCCTGACATTACTGTCGAGCAGGCCGCAAAAGACGCAGGCGCAGAAATCGTTGCCTACGTCCGCATGCAGGTTGGCGAAGGTATCGAGAAAAAAGAAGAAGATTTCGCAGCTGAAGTTGCCAAAGCCGTTCAAGGCTAAATGAACCAGGAACTGACCAACAATCCGTTCAACCCTGTTGCACCGATTAAAACTTTCGACGAAATCAAGGTGTCGCTTGCGTCGCCCGAACGGATTCTGTCGTGGTCTTTCGGTGAAATCAAAAAGCCCGAAACCATCAACTACCGGACGTTCAAACCTGAACGGGACGGTCTTTTCTGCGCGCGTATCTTTGGCCCGATCAAAGACTACGAATGCTTGTGCGGCAAATATAAGCGTATGAAATATCGCGGCGTTGTCTGCGAAAAATGTGGTGTTGAAGTTACGCTGCAAAAAGTACGCCGCGAACGTATGGGCCACATCGAACTGGCCGCTCCTTGCGCACACATCTGGTTCCTCAAGTCGCTGCCTTCGCGCATCGGCCTGATGTTGGATATGACATTGCGTGACCTTGAGCGCGTTTTGTATTTCGAAAACTACGTTGTGATCGAACCCGGTCTGACCGACCTGACTTATGGCCAAATGATGAGCGAAGAGGAATTCCTCGACGCACAAGACGCCTATGGTATGGACGCGTTCACCGCCAATATCGGTGCCGAAGCCATCCGTGAAATGTTGGCCAACATCGATCTGGAAAACGAAGCTGAACAGCTGCGCGCCGACCTCAAAGAGGCAACCGGTGAGCTGAAGCCCAAGAAGATCATCAAACGTCTGAAAGTTGTGGAATCCTTCTTGGAATCCGGCAACCGTCCGGAATGGATGATCATGACCGTTGTGCCTGTGATCCCACCAGAGCTGCGCCCGCTGGTGCCGCTGGACGGTGGCCGCTTTGCGACGTCGGATCTGAACGATCTGTATCGCCGGGTGATCAACCGGAACAACCGTCTGAAGCGTCTGATCGAACTGCGCGCGCCTGATATCATCGTTCGCAACGAAAAGCGGATGTTGCAGGAATCTGTTGACGCACTGTTCGATAACGGCCGTCGTGGCCGCGTGATCACCGGTGCCAACAAGCGCCCGCTGAAATCGCTGTCCGACATGCTGAAGGGTAAGCAAGGTCGCTTCCGTCAAAACCTTTTGGGTAAGCGCGTCGACTTCTCTGGTCGTTCGGTCATTGTGACCGGTCCTGAGCTGAAACTGCATCAGTGTGGATTGCCGAAAAAGATGGCCTTGGAACTGTTCAAGCCGTTTATCTATTCGCGCCTCGAAGCCAAAGGCCTAAGCTCGACTGTAAAGCAGGCGAAAAAGCTGGTTGAAAAAGAGCGTCCCGAAGTGTGGGATATCTTGGATGAAGTGATCCGCGAACACCCTGTAATGCTGAACCGTGCGCCAACATTGCACCGTCTTGGTATTCAGGCGTTCGAACCCACGCTGATCGAAGGTAAAGCCATCCAGCTGCACCCACTGGTTTGTTCGGCCTTCAACGCTGACTTTGACGGCGACCAAATGGCGGTCCACGTGCCTTTGTCGCTCGAAGCCCAGCTTGAAGCCCGCGTTTTGATGATGTCGACGAACAACGTTCTGTCGCCTGCAAACGGCGCACCGATCATCGTTCCGTCGCAGGATATGATTTTGGGTCTGTACTATGTGACCATCATGCGCGAAGGCATGAAGGGCGAAAACATGGTATTCTCGTCGCTCGAAGAGGTCGAACACGCCCTCAACGCCGGCGAAGTGCATTTGCACGCCAAAGTTCAAGTTCGTCTCAAGCAAATCGACGACGAAGGTGCCGAAGTCTATACACGCTTCGAAACCACGCCGGGCCGTGCCCGTCTTGGTGCCCTGTTGCCGATGAACGCAAAAGCACCGTTCTCGTTGGTCAACGACTTGCTGCGCAAAAAAGACGTGCAGCGCGTGATTGACACCGTTTACCGGTATTGCGGTCAAAAAGAGTCTGTTATTTTCTGTGACCAGATCATGACACTCGGTTTCCGCGAAGCGTTTAAGGCAGGCATTTCCTTTGGTAAATCCGACATGGTTGTTCCTGACAACAAGTGGGATATCGTTGGTCATACGCGCGATCAGGTAAAAGATTTCGAACAGCAGTACATGGACGGCCTGATCACTCAGGGCGAAAAGTACAACAAAGTTGTCGATGCTTGGTCAAAGTGTAACGACAAAGTCACCGAAGCGATGATGTCTACCATCTCGGCTGAAAAACGCGCCGAAGACGGCTCGGTTATGGAACCAAACTCGGTTTACATGATGGCCCACTCCGGTGCGCGTGGTTCGGTTACACAGATGAAACAGCTGGGCGGTATGCGCGGTTTGATGGCAAAGCCGAACGGCGACATCATCGAAACACCGATCATCTCGAACTTTAAAGAAGGTCTGACCGTTCTGGAGTACTTCAACTCCACCCACGGTGCCCGTAAGGGTCTGTCAGATACGGCTTTGAAAACAGCGAACTCGGGTTACTTGACCCGTCGGTTGGTTGACGTTGCCCAAGACTGTATCGTTCGTATGGTTGATTGTGGAACGGACCGTGCTGTTACGGCCTCTGCGGCTGTGAACGACGGCGAAGTTGTCGCAACTTTGGGTGAACGTGTTCTGGGCCGTGTTGCAGCCGAAGACATTTGCCACCCTGTCTCTAGTGAAGTGCTGGCACCAGAAGGCTCGCTCATTGATGAACGGACTGCGGATGCCATCGAAGACTCCGGTGTTTTGACAGCACGTATTCGCTCACCACTGACTTGTGAAGCGGAAGAAGGCGTATGTTCGCAGTGTTACGGTCGTGACCTTGCACGCGGTACGCGCGTCAACCTTGGTGAGGCCGTTGGCATCATCGCGGCGCAGTCGATCGGTGAACCGGGCACTCAGTTGACCATGCGGACATTCCACATGGGCGGCGTTGCGTCTGGTTCGTCCCGCTCGTTCCTTGAAGCGTCTCAGAACGGCAAGATCGCCTTCGCAAACCCAAGCGTTATTACAAACGCGTCGGGCGAGCAGATTGTGATGAGCCGGAACATGCAGGTTTTGATCCTGAACGAACAAGGCGAAGAAATCGCATCCCATAAAATGGGTTACGGTTCCAAGCTGTTCGTTACCGATGGTCAAACCATTGCACGCGGCGACAAAATGTTCGAATGGGATCCCTACACCCTGCCGATCATCGCGGAAAAGGACGCTGTTGCGAGACATGTTGACCTTGTGAACGGCCTCGCCGTGCGTGAGGAAACCGATGACGCGACCGGCATGACCCAGAAGATCGTGGTTGACTGGCGTGCGGCCCCCAAAGGCAATGAGCTTAAGCCTGAAATTATCCTGATGGATAAAGATGGTGAGCCCGTTCGCAACGATGCTGGCAATCCGATTACCTATCCTATGTCTGTTGATGCGGTTCTGTCTGTTGAAGACGGTCAGGACGTCAAAGCGGGTGACGTTGTTGCACGTATTCCGCGCGAAGGTGCCAAGACCAAGGACATTACAGGTGGTCTGCCACGTGTTGCGGAATTGTTCGAAGCCCGTCGTCCAAAGGATCACGCGATCATCGCTGAAATCGATGGTTATGTGCGCTTTGGTAAGGACTATAAAAACAAGCGTCGTGTTTCCATCGAGCCTCAAGACGAGACCATGGACGCCCGTGAATACATGATCCCGAAAGGTAAGCACATTCCTGTCATGGAAGGTGATTTTATCCAGAAGGGTGAATACCTCATGGACGGTAATCCGGCACCGCACGACATCCTCGGCATTATGGGGATCGAAGCGCTGGCGAACTACATGATCGACGAAGTTCAGGACGTTTACCGCCTGCAGGGTGTTAAGATCAACGATAAGCATATCGAAGTGATCGTGCGCCAAATGCTGCAAAAGTGGGAAATCCAGGACAGCGGTGACACAACCTTGCTGAAAGGCGAGCATGTGGACAAAGCTGAATTTGATGCGGCCAACGAAAAGGCTCTGTCGAAATCAGGTCGTCCTGCGAAGGGTGAGCCGATCCTATTGGGGATCACAAAGGCGTCCTTGCAGACCCGTTCGTTCATCTCGGCGGCATCGTTCCAGGAAACCACACGGGTTCTGACCGAAGCCTCTGTTCAGGGCAAGAAAGACAAACTTGTTGGCCTGAAAGAGAACGTCATCGTTGGTCGTTTGATCCCTGCCGGTACCGGTGGTGCCACGCAAGAAATGCGCCACATCGCATCGACACGTGACAACGTTGTTGTTGAAGCGCGCCGTATTGAAGCAGAAGCCGCAGCCGCTTTGGCTGCGCCTGAGCCAGTT---GCCGACGTCGTTGGTGGCAGCGAATTCGATACGCTGATTGTCACTCCGGAGAGCCGCGAATAAATGTCCCGTCGTCACGCTGCCGAGAAGCGCGAAGTCCTGCCCGACGCCAAATTTGGCGATCGCGTTCTGACTAAATTCATGAACAACCTGATGATTGATGGTAAAAAATCAGTCGCAGAAAGAATTGTTTATAACGCGCTCGATCGCGTTGAAACCAAAGTCAAGCGCGCCCCTGTGGAACTGTTCCACGAAGCGCTTGATAACATCAAACCATCGGTCGAAGTTCGTTCGCGCCGCGTGGGTGGTGCAACTTACCAGGTTCCCGTCGAAGTGCGCCCCGAGCGCCGCGAAGCCCTGGCAATTCGCTGGTTGATCACGGCCAGCCGTTCGCGCAATGAAAACACAATGGAAGAGCGTCTTGCTGGCGAACTTCTGGACGCTGTTCAATCGCGCGGTTCCGCCGTTAAGAAACGTGAAGACACCCACAAGATGGCCGACGCTAACAAAGCGTTCAGCCATTACCGCTGGTAAATGCCTTACGCCCATTCTGACAAATCT---------GCCTTGCTGCATTCTCCGGCCCCTGACATCAAAGACCGCGTGAAACTGGAAGGCGGCAAAGAATTTGTGCTGCACACGGAATTCAGCCCTGCGGGGGATCAACCGACAGCGATTGTAGAATTGACAGGTGGCATAAACGACGGCGACCGGTCGCAGGTTTTGTTAGGGGCCACTGGCACCGGCAAAACATTCACCATGGCGAAAGTCATCGAAGAAACGCAGCGCCCAGCCATCATCCTTGCCCCGAACAAGACCCTCGCGGCCCAGCTATATGGGGAATTCAAAGGATTTTTTCCGGACAATGCCGTCGAATACTTTGTCAGCTATTACGACTATTACCAACCCGAGGCCTACGTCGCGCGCTCTGACACCTTCATCGAGAAAGAAAGCCAGATCAACGAGCAGATCGACCGGATGCGCCATTCGGCCACTCGGGCGCTTTTGGAGCGCGACGATGTGATTATTGTGGCCTCGGTCTCGTGCATTTACGGGATAGGCTCGGTCGAAACCTACGGTGCGATGACCCAAGATTTGTTGGTCGGCACTGAATATGACCAGCGGAAGGTCATCGCCGATTTGGTCGCACAGCAGTATCGCCGCAACGATCAGGGGTTTCAGCGCGGTTCCTTTCGGGTCCGCGGCGACAGTTTGGAAATCTGGCCCGCCCACCTTGAAGATCGCGCTTGGAAACTATCCTTCTTTGGCGAGGAACTGGAAGGTATTACCGAATTTGACCCGCTGACCGGGGTGAAAACCGACACCTTTGACCGCATCCGCATCTACGCAAATTCGCATTACGTCACGCCCAAACCAACGATGAATCAGGCCGTCATCGGCATCAAAAAAGAATTGCGCCAGCGGCTGGATCAACTGGTCGGCGAAGGTAAATTGCTAGAGGCGCAACGTCTTGAGCAACGCTGTAATTTCGACGTCGAGATGTTGGAAGCCACCGGCGTTTGCAGTGGAATTGAGAACTATTCGCGTTATCTAACCGGCCGTGCACCTGGCGAGCCGCCCCCCACGCTGTTCGAATTCATCCCGGACAATGCCATCGTATTTGCGGACGAATCCCACGTTTCAGTGCCGCAAATCGGCGCGATGTACAAAGGCGACTTTCGGCGCAAGATGACACTGGCCGAACACGGGTTCCGCCTGCCTTCATGCATGGACAACCGCCCCCTCAAGTTTGAGGAATGGGACGCAATGCGGCCCCAGTCGATCTTTGTTTCAGCCACCCCTTCCAGATGGGAGCTGGAGCAATCCGGCGGTGTCTTCACCGAACAGGTGATCCGACCCACAGGCCTGTTGGATCCCCCGGTCGAAATCCGCCCGGTCGAAATGCAAGTCGATGATGTTCTTGATGAAATTCGCAAAGTGACCGCCGAAGGCATGCGCACCCTTGTCACAACTCTGACAAAACGCATGGCCGAAGATTTAACCGAATACCTGCATGAACAGGGCATCAAGGTCCGCTACATGCATTCGGATATCGACACCATTGAACGGATCGAAATCCTGCGCGACCTACGACTTGGGGCGTTTGACGTTCTCATCGGTATCAACCTGCTGCGAGAAGGGTTGGACATTCCGGAATGCGGATTGGTCGCCATTTTGGACGCGGACAAAGAAGGTTTTCTGCGCTCTGAAACATCGCTTGTGCAGACAATCGGACGTGCGGCGCGGAACGAGCATGGCCGGGTTATCATGTACGCTGACCGGATGACTGGCTCAATGGAGCGGGCAATTGGCGAAACCAACCGCCGCCGCGCCAAACAAGAGGCATACAACGTCGAACACGGCATCACCCCCGCAACTGTGAAAAAGAACGTCGAGGATATTCTAGCTGGTCTTTATAAGGGCGATACGGATCAATCACGGGTTACGGCCAAAATCGACAAGGGGCATGCTGGCGGCAACCTTCAAACCGTTCTTGAGGGCCTGCGCAACGACATGCGCAAGGCCGCCGAGAACCTTGAGTTCGAAGAAGCGGCGCGATTACGCGATGAAGTCAAGCGATTGGAGGCCGTCGATTTGGCTGTATCGGACGACCCGCTGGCCCGGCAATCTGCAGTTGAGGCAGCGTCAGAAGCGGCGGTGAAATCACGAGGTCGGTCTACAGCCGGTCGGCCGGGCCAACGTGGCGGGGTGAAGAGACGCAGA------TAGATGGCTGCAAAACCGTTTTTCCGCCGTCGCAAAGTCTGCCCCTTCTCGGGCGACAACGCACCTAAGATCGATTACAAAGACACCCGTCTTTTGCAACGCTACATCTCTGAGCGCGGCAAAATCGTTCCTTCCCGTATCACCGCAGTGTCTGCGAAAAAGCAGCGTGAATTGGCAAAAGCCATCAAACGCGCCCGCTTCCTCGCTCTGCTGCCATATGCTGTGAAATAAATGCCCAAGCGTATCCTTACTGGCACTGTAACCTCCACTGCCAACACCCAGACTGTAACTGTCTCGGTTGAGCGCCGTTTTAAGCATCCGGTCATGCAAAAGACCGTGAAGCGTAACAAGAAATACCGTGCTCACGACGAGGCGGAAAAGTTCGCAGTCGGCGATATGGTTCGCATCATTGAATGTGCGCCTCGCTCGAAAACGAAACGTTGGGAGGTTCTGGCTGAC------TAAATGGCTAATTCGCCCCAAGCAAAAAAACGCGCCCGTCAGAACGAAAAGCGTTTGGAAGTTAACAAAGCACGCCGTTCGCGTATCCGCACATTCCTGCGCGGCGTTGAAGAAGCAATCGCTTCCGGTGACAAAGCTGCTGCAACTGCTGCTTTGAAGGCCGCTCAGCCAGAGCTGATGCGCGGCGTCACCAAAGGCGTTTTCCACAAGAATACCGCGTCACGCAAAGTTTCTCGTCTCGCGGCACGTGTAAAAGCGTTGGGATAAATGTCTCGCTCTGTTTGGAAGGGCCCATTTGTCGATGCCTACGTCCTGAAAAAGGCCGAAAAAGCACGCGACTCGGGCAAGAACGAAGTTATCAAGATCTGGTCGCGTCGTTCGACAATCCTGCCGCAATTCGTCGGTCTGACTTTTGCCGTGTATAACGGTCACAAGCATATCCCAGTGAACATCACTGAGGACATGATTGGTCAGAAGTTCGGTGAATATTCGCCAACGCGTACCTATTATGGTCACGCGGCCGACAAAAAAGCCAAAAGGAAATAATTGGCACGTATTGCCGGCGTTAACATCCCGACCAACAAGCGGGTGCCCATCGCACTCACATATATCACAGGTATCGGCCACACTTCGGCTGCTGCTATCATCGAAGCGGTAGGCATCGACGCCACACGCCGCGTAAATGAGCTGTCCGACGCTGAAGTATTGGCGATCCGTGAGCACATCGATGCAACCTATACCGTAGAAGGCGACCTGCGCCGCGAAGTGACCATGAACGTTAAGCGTTTGATGGACCTCGGTTGCTACCGTGGCCTGCGTCACCGCCGCAATCTCCCAGTACGCGGTCAGCGTACTCACACCAACGCTCGTACCCGCAAAGGCCCCGCAAAGGCCATCGCAGGCAAGAAGAAGTAAATGGCTATTAAAATTCGTCTCGCCCGCGGCGGTTCCAAAAAGCGCCCCTTTTACCGGATCGTTGCTGCGGACAGCCGCATGCCACGTGACGGCCGCTTTGTAGAAAAGCTGGGCACATACAACCCCCTGCTGCCAAAAGACAGCGAAGAGCGTGTGAAAATGAACGTTGAGCGCATCCAGTACTGGCTCGGCGAAGGCGCTCAGACCACTGACCGCGTATCGCGCTTCCTCGAAGCGGCTGGCGTTGTTGCCAAAAAAGAGCGTTCCAACCTGAAAAAAGGCGAGCCAGGCAAAAAAGCCAAAGAGCGCGCCGAAGAAAAAGCATCCAAAGCTGCT------------------------GACGCCGCTGAAGCTGCTGCCGAGGCATAAATGTCGATTACACCTGAAGAAAAAACACGCTTGATGAAAGAATTCGGCACCAAAGAAGGCGACACTGGTTCGCCCGAAGTACAGGTTGCCATCCTCACCTCGCGTATCTCGACCCTGACCGAGCATTTCAAAACCCACAAAAAAGACAACCACTCGCGTCGTGGTCTTTTGAAGTTGGTTGCTCTGCGTCGTAAGCTTCTGGATTACACCAAAGCCAAAGATGTGGCCCGTTACCAAGACCTCATCAAACGCCTCGGCATCCGTCGCTAAATGGCGACGCTGAACGACATCCGGTCGACCTTTTTGAACTACTTCGCGAAACAGGGGCATACGATCGTCGATTCCAGCCCTCTGGTGCCGCGCAACGATCCGACGCTGATGTTCGTCAACTCGGGCATGGTGCAGTTCAAGAACCTGTTCACCGGACTTGAAACACGCGACTACAATCGGGCCACAACTGCGCAAAAATGTGTGCGCGCTGGCGGCAAGCACAACGATTTGGACAATGTCGGGTATACGGCACGTCATCACACGTTCTTTGAGATGCTCGGCAACTTTTCCTTTGGGGATTACTTCAAAGAGGAGGCAATCACCTTCGCATGGGAGCTGATCACCAAAGAGTACGGCATTCCCAAGGATAAACTGTATACCACAGTTTATCACACCGATGACGAAGCTTATAACATCTGGAAAAAACTG---GGTGTCCCCGAAGAGCGGATCATCCGGATCGCGACCTCTGACAACTTTTGGCAGATGGGGCCAACCGGTCCGTGTGGTCCATGTACCGAGATTTTCTACGATCACGGGGATCATATCTGGGGCGGCCCTCCCGGCTCGCCCGAGGAAGATGGTGACCGGTTCATCGAAATCTGGAACGTGGTTTTCATGCAGAACGAGCAGTTCGAAGATGGTTCGATGAAGGCGTTGGACATGCAGTCGATCGACACCGGCATGGGGCTGGAACGGATCGGCGCGCTGTTACAAGGCAGCCACGACAACTACGACACCGATTTGTTCAAAACATTGATCGAAGCCTCGGCCCATGCCACCTCCGTTGAGCCTTATGGTGACAAGAATGTGCATCATCGGGTTATTGCGGACCACCTGCGCTCGACCTCGTTCTTGATTGCGGATGGTGTGATGCCATCCAATGACGGGCGCGGTTACGTTTTGCGCCGGATTATGCGCCGTGCGATGCGCCATGCGCATTTGTTGGGCGCGAAAGACCCGGTGATGCATCAATTGGTGCCTTCGCTGGTCGGACAAATGGGGCAGGCCTATCCTGAACTTGGTCAGGCACAAGCCATGATCGAGGAGACATTGCTGCTTGAAGAAACCCGTTTCAAACAGACTTTGGACCGCGGCCTGAAATTGCTTGATGATGAAGTCAGCGGATTGGACGAAGGGGCGCAGCTTTCTGGTGAAGCCGCGTTCAAACTCTATGACACCTATGGCTTTCCTCTCGATCTGACCCAAGATGCGCTGCGCGAGCAGGGCCGCGAAGTCGACACCGACGGGTTTACAGCGGCAATGGAGCAGCAAAAAGCCAAAGCGCGGGCTGCTTGGTCGGGGTCTGGTGACGCTGCGGACAGCACGGTGTGGTTTGACGTGCTTGACGGTGCAGAGCCCACGGACTTTCTTGGTTATGACACCGAAAAGGCCGAAGGTCAGATTTTGGCTTTGGTGGCTGATGGCAAGCTGGTCGACAGTTTGGCTGAGGGGGCAACGGGTTGGGTTGTGACCAACCAGACGCCATTTTATGCCGAAAGTGGCGGTCAGGTTGGCGACGAAGGCGTGATCCGCAACCGCGACGATGCGGCCGCAGTTTCGGACACTCGTAAAGACAGCAAGATCATTGCCCACAAGGTTACGGTGTCCAAGGGCACATTGGCCACCGGCGAAGCCGTTGAATTGGAAGTGGGGCACGGCCGACGCAGCACCATTCGCGCCAACCATTCGGCGACTCACCTTTTGCACGAGGCCCTGCGCAATGCACTTGGCGATCACGTCGCGCAGCGCGGATCGTTGAATGCACCGGATCGGCTGCGGTTCGACTTTAGCCATGGCAAGGCATTGAGCGCTGCCGAACTGTCACAGGTTGAGGCCGAGGTGAACGCCTATATCCGTCAGAATTCCAGCGTCAGCACCCGCATCATGACGCCCGATGACGCCCGCGCCATTGGTGCGCAGGCGCTATTTGGCGAAAAATACGGTGACGAAGTGCGTGTCGTCTCGATGGGGCAGCAATCCGGATCGGGCAAGGGCAGCGATGGTTCAACCTATTCGCTGGAACTGTGCGGTGGAACCCATGTGCGCCAAACGGGCGATATCGGTGCCTTTGTTAGCCTTGGAGACAGTGCATCCAGCGCTGGTGTAAGGCGGATTGAAGCGCTGACTGGGCAAGCGGCAATGGACTACCTGTCTGAACAGTCTGCAGCGCTGAGTACGGTTGCGGGCGAGCTTAAAGCCCCCGTGTCCGAGGTGCCTGCACGGGTCAAGGCGCTGATGGACGAACGCAAAGCCCTGACCAACGAAGTGGCGCAACTGCGCCGAGAGTTGGCCATGGCGGGCGGCGCAGGGCAGGGCGGCGCGGAATCAACCGATGTCAACGGGGTCGCCTTTGTTGCGCAGGTTTTGACCGGTGTGTCCGGCAAAGATCTGCCGGCGCTGATTGATGAACACAAATCCCGCATTGGGTCGGGCGCAGTTCTGCTGATCGTGGATGCGGGCGGCAAGGCTGCTGTGGCAGCTGGTGTGACGGCTGACATGACCGGAAAGGTATCGGCGGTTGATTTGGTCAAGGCAGCCGTGGTTGAACTGGGCGGAAAAGGCGGCGGTGGCCGCCCTGATATGGCGCAAGGCGGGGCCAAGGATGTGGCCAACGCCGACGCGGCAATAAAAGCGGCTCAAGCCGTCATTGGAGGATAAATGGGCTTTCGGATGGGAATCGTGGGTCTGCCCAATGTGGGTAAATCGACGTTGTTTAATGCGCTGACCAAAACCGCCGCAGCGCAGGCGGCCAATTTTCCGTTCTGCACGATCGAACCCAATGTGGGTGACGTGGGCGTGCCGGACGCGCGACTTGATAAACTGGCCGCGATTGCCGGATCCAAACAGATCATTCCAACGCGGATGACGTTTGTGGACATTGCCGGTTTGGTCAAAGGGGCGTCAAAGGGCGAAGGTCTGGGCAACCAGTTTTTGGCCAATATTCGTGAAACAGACGCGATTGCGCATGTCTTGCGGTGTTTCGAAGACGAAGACGTGACCCATGTGGATGGCCGCGTTGATCCGGTGTCCGATGCTGAAACCATTGAGACCGAGCTGATCATTTCCGACATGGAAAGCTTGGAAAAGCGGTTGCAGAACATCACCCGTAAGGTCCGCGGTGGCGACAAAGAGGCGGTTCAACAAGAACGCCTGATGAAAGAAGCCATGGCGATGCTTGAGCAGGGCAAACCCGCCCGCTTGGTTGAGGTCGACGATGATGACCTCAAAGCTTGGAAAATGCTGCAATTGCTATCGACCAAGCCGGTTCTGTATGTCTGCAACGTTGACGAAGGCGAAGCCTCAACCGGCAACCATTTGTCTGAGCAGGTGGCGGAAATGGCGGCAGCGCAGGGTAACACCCACGTGGTGATCTCGGCCAAAATCGAAGAAGAGATCAGCCAGCTCGAAGATGACGAAGCCGAGATGTTTTTGAGCGAACTTGGTCTCGAAGAGGCCGGTCTCGACCGTTTGATCAAGGCTGGCTATGAACTGCTGCACCTTGAAACCTACTTCACGGTTGGCCCGAAAGAAGCGCGTGCCTGGACGATCAAATCCGGCACGCAGGCTCCGCAGGCGGCCGGGGTGATTCATGGCGATTTCGAACGTGGATTTATCCGCGCCGAAACCATCGCCTATGATGACTATCTGGCGTGCAACGGTGAAAACGGTGCCAAAGACGCGGGCAAGATGCGCGCTGAGGGTAAAAGCTATACCGTCAAAGATGGCGATGTGTTGCACTTCCTGTTCAACACCTGAATGGTCTGGAAAACCCTCGACGACATGGATCTGGCAGGCAAACGCGTGCTGACGCGTGTTGATATCAACGTGCCGTTCGAAGACGGAAAAGTCACTGACACAACGCGTATCGAACGCATTGCAGCAACGGTTCAAGATATCCTTGATCGCGGCGGAAAGCCTGTTTTGCTGGCGCATTTCGGCCGTCCCAAGGGCGAGCGGAACATGGACATGAGCCTGCAATCGCTGATTCCGGCGCTTGAATCTGTGTTTGGGTCTCCGGTGGTTTTTGCCTCTGATTGCGTTGGAGCGTCGGCTGTGGTGGATGCTTTGCAGGACGGTCAAGTCGCCTTGCTGGAAAACACCCGTTTTCATGCTGGCGAAACAAAAAACGACCCTGAATTGGCTGCGCAAATGGCCAAACTCGGTGACATCTATTGCAATGATGCCTTTTCAGCCGCGCACCGCGCGCATGCCTCCACCGAAGGGTTGGCTCGGTTGCTGCCCTCTTGCGCAGGGCGCTTGATGCAGGCTGAACTCTCCGCGCTGGAAAGTGCACTGTCCGAGCCCGAGCGTCCGGTGCTGGCAGTGGTTGGCGGTGCAAAAGTTTCGACCAAACTGGATTTGCTAGGAAATTTGGTCGCCAAAGTTGATATGCTGGTGATCGGCGGCGGGATGGCCAATACCTTTTTGGCAGCCCAAGGCATCGACGTCGGCAAATCCCTTTGCGAACATGAAATGGCTGACACCGCCCGTGAAATTCTGGCCAAAGCGGCGGACGCCGGATGCGAAATCATTTTGCCAACCGACGTTGTCGTGGCGCGTGAATTCAAAGAAGGTGCAGCGAATGAGACCGTGGCCGCC---------AACGCTTGCCCCGCTGATGCGATGATTTTGGACGCGGGCCCCGAAGCGGTTGCAGCCATCAAGGACGCGATCAACAAGGCAAAGACACTGATCTGGAACGGCCCGCTTGGCGCGTTTGAAATTGCGCCCTTTGACATTGCCACCAACGCCGCTGCGAAATTCGCAGCCGAGCGTAGTGCAGCCGGTAAACTTGTGTCTGTCGCCGGTGGCGGCGATACCGTTGCGGCCTTGAACAAGGCGGATGCAGCCAATGATTTCAGCTACATCTCAACCGCTGGTGGTGCCTTTTTGGAATGGATGGAGGGCAAAGAACTGCCCGGAGTTGCCGCGCTGAGC---TAGATGGCTCTTCCTGAGTTCACCATGCGTCAGCTGCTTGAAGCAGGCGTACACTTTGGTCACCAAACACAACGCTGGAACCCTCGTATGGGTCCGTTCATCTATGGCGGCCGTAATGGCATCCATATCATGGACCTCACACAGACTGTTCCAATGCTGGATCAGGCGCTGAAAGCGATCCGTGATTGCGTTGCAAAAGGCGGCCGCGTTCTGTTCGTTGGCACCAAGCGTCAGGCAGCTCAGCCTGTTGCAGACGCCGCAGAAAAATGCGCCCAGTATTACATGAACCACCGTTGGCTCGGCGGTACGCTGACCAACTGGCAGACCGTTTCCAAATCAATCGGCCGTCTTCGTCAAATCGACGAGCTGATGGAAACTGGTGCCGAAGGCCTGACCAAAAAAGAGCGTCTGGGCATGGAACGTGACCAGATCAAATTGCAAGCCTCTTTGGGCGGCATCCGTGAAATGGGCGGCGTTCCTGACATGCTGTTCGTCATCGACGTCAAAAAAGAAGCACTGGCCGTGGCCGAAGCCAACAAACTGGGTATCCCGGTTGTTGCAATCGTCGACACCAACTGCTCGCCCGATGGCATCGATTACATCATCCCAGGCAACGACGACGCGTCCCGCGCCATCACGCTGTACTGTGATCTTGCATCGCGCGCTGCTCTGGACGGTATGACCGGTCAGATGGAAGCCGCTGGCTACGATCTGGGTGCCTTCGAAGAAGCGCCGATCGAAGAAGTTGTTGCCGAAGAAGCA---CCTGCTGCC---------------GAGGCACCTGCA---------GAAGGCTAAGTGACCAAACGCACATCTGCCAAGTACAAAATTGACCGCCGCATGGGCGAAAACATCTGGGGCCGTCCTAAATCCCCGGTAAACCGTCGCGAATATGGCCCCGGCCAGCACGGTCAGCGCCGCAAGGGCAAAATGTCTGACTTCGGTCTGCAGCTTCGCGCCAAGCAGAAGCTAAAAGGCTACTACGGCGACCTGACCGAAAAGCAGTTCCGTCGCATCTTCGGCGAAGCCGAGCGTGTTAAAGGCGACACTGGTGAAAACCTGATCGCACTTCTGGAACGCCGCTTGGATGCGGTTGTTTACCGCGCCAAGTTCGTTGCGACCGTATTCGCCGCACGCCAGTTCGTGAACCACGGCCACGTTCTGGTCAACGGCAAGCGCGTAAACATTCCTTCGTACCGCGTCAAAGAAGGCGATGTTGTTGAAGTGCGTGAAAAGTCTCGCCAGATGGTCGTTCTGCTGGAAGCAACTCAGCTGTCCGAGCGTGACGTTCCTGACTACATCGAAGCCGACCACTCCAAAATGTCTGCGAAGTTCGTTCGTTCGCCTGGTCTGGCCGATGTGCCTTACCCAGTTGTGATGGAACCAAACCTCGTCATCGAATACTACGCTCAGAACTAAATGGGTAATAAAACAAACCCGATCGGTATGCGTCTGCAGGTGAACCGCACCTGGGACAGCCGTTGGTACGCTGACACCAAAGACTACGGTGATCTGCTGTTGGAAGACCTCGCGATCCGCGATTTCATCCACAAAGAGTGTAAGCAGGCCGGTATTGCCCGCGTGATCATTGAACGCCCTCACAAGAAGTGCCGTGTATCTGTACACACAGCGCGTCCGGGTGTGATCATCGGCAAAAAAGGTGCTGACATCGAAGTTCTGCGCAAGAAGCTGGCGGCTATGACCGACAGCGAAGTGCACCTGAACATCGTTGAAGTGCGCAAGCCCGAGCTGGACGCCCAGCTTGTTGGTGAGAGCATTGCTCAGCAGCTGGAACGCCGTGTGTCTTTCCGCCGTGCGATGAAGCGCGCTGTTCAAAACGCCATGCGTATGGGCGCCCTGGGTATCCGGGTGAACGTTGCTGGTCGTTTGGGCGGTGCAGAAATCGCGCGTACCGAATGGTACCGCGAGGGCCGAGTGCCCCTTCACACGCTGCGTGCTGACATCGATCACGCCCAAGTCGAAGCTTCGACCGCCTATGGTATCATTGGTATCAAGGTCTGGATCTTCAAGGGTGAGATCATGGAGCACGACCCGGCGGCACGCGATCGTAAATCGCAAGAAGTTCAAGATGGCCCAGCACCTCGCGGTGCAGGTGGTCGTCGTTAAATGGGACGCAAGCGCAAGGGCCGCGATATTTCAGGCTGGCTGATCGTAGACAAACCGGCTGGCATGACCTCGACTTCGGTTGTGAACAAGGTCCGCTGGGCAATGGATGCCAAAAAAGCCGGCCATGCAGGCACGCTCGATCCAGAGGCGACAGGTGTTTTAGCTGTCGCTTTGGGCGAAGCCACCAAGACCGTTCCCTATATTACAGAGGCTCTCAAAGCCTACCGGTTTACGGTGGTGCTGGGACAGGCCACAAACACTGACGACGCCGAGGGCGAAGTCATAGAGACCTCGGATTTGCGCCCGTCTGACGAAGAGATCAAAGAGGCTTTGCATCAATTTGTCGGTGACATCCAACAGGTGCCCCCACAGTTTTCCGCCGTCAAAATAGATGGAGAACGCGCCTACAAACGCGCCCGCGACGGCGAAGAAATGGAGATCGCTGCCCGGGCTTTGTTTGTCGAAGAACTGGTGATGATCTCGCGTCCCGACACCGACCATGTTGAACTGGAAATGATCTGCGGCAAAGGCGGATATGTCCGGTCCATCGCCCGTGATCTGGGAGAAACCTTGGGATGTAAGGGCCACGTGCTGCGCCTCCACCGTATCTGGTCAGGTCCTTTTGAAACCTCAGACGGGATCACCGTTGAACAAATCGATGAAATGGCCAAGACCAGCGCACTTGACGCCTATCTTAAACCGCTCGAAGTCGGTCTGAGCGATCTACCTGAATTACGCACCACCCCTGAAGGGGCGGTTAAACTGCGCAACGGCAACCCCGGCATGGTTTTTGCGGCTGATGCGGAATACGGCGACGAGGCTTGGGCCTCGCTAGACGGCGAACCCATTGCGGTCGGGATCTACAAAGCAGGTGAACTCCACCCCAAACGCGTCTTTGTCCGTGCAGAATAAAGTTTTAAGCTTGGACTGACCGGATCTATTGGAATGGGGAAAAGCACAACAGCCCAGATGTTTGCGGACTTGGGTTGCCCGGTCTGGGACGCGGATGCTGCGGTGCATCGTCTCTATGGAATTGATGGCGATGCGACCTTGGCAATTTCTGATATTTGGCCGGAGGCTGTCATAGACGGTATGGTTTCGCGGGATGAACTGCGCGCAATTATCGCCAAGGATCACACTGCCTTGCCCCGAATTGAAGCGATTGTACACCCTTTGGTTCAGGCTGATCGCACAAAATTTATTTCTCAACATATTGATAATATTTGTGTATTTGACATTCCTTTACTGTTTGAAACCGGCGGTGAATCGGAGATGGACGCGGTTGCCTGTGTGTCGGTGTCATCCCAGATACAGCGCGACCGGGTGTTGGCGCGCGGAACAATGACGGCCGCAGATTTGGATCGCATTTTAGCGCGTCAAATGCCGAACGAAGAGAAATGCGCCCGGTCGACCTATGTTATAGAAACCGATAGTCTGGAGCATGCCAAGGCGCAAGTTGCGGCAGTTGTGGCAGATATCAAAAGGCAGTTGGCTAATGCGTGAATG---TTGACCGATGCAATTCTAGAGGACGACCGTTGGCAGCCCATCGGGCTTGAACAGCTGTCCGAGCGCGCCGCAGTTGCAACACTGTTGCATCTGAATGTGGCAGTGGATGCGCATGAGATTGTGGTGATGGGCTGCAACGACTCCCGAATTGCGGCCCTGAACCAAGATTTCCGTGACAAACCCACTGCGACAAATGTGTTGTCTTGGCCGTATGAGGATCTCTCAAGCGAAGAATCAGGCGGAACTCCGCTTTTGCCGGAGGCAGAA---------------GAGCTGGGGGATATCGCAATTTCCTTTGAAACTTGCGAAAAAGAGGCGCGCGACCAAGGAAAACCGCTAACCGACCACGTGACACATCTTTTGGTTCACGGTGTGTTACATCTGCTGGGCTATGATCATATCCGTGACGCAGATGCCACGGTCATGGAACAGTTAGAGGTGGAAATACTTGGCAAGCTAGGGTTACCCGACCCATATAGAGGGTATGAAGGGTGAATGGCACGCTTTATCTTTATCACCGGCGGTGTGGTTTCTTCGCTTGGCAAGGGCCTTGCATCTGCGGCCTTGGGTGCCTTGCTGCAGGCACGTGGTTATTCGGTCCGTTTGCGCAAACTGGACCCTTACCTAAACGTCGATCCCGGCACGATGTCGCCTTTTGAACATGGCGAAGTGTTTGTCACCGATGATGGCGCTGAAACCGATCTGGATCTGGGCCACTACGAACGCTTCACCGGCGTTCCCGCGACACGTACGGATTCCATCAGCTCAGGCCGCGTTTATACCAATGTTCTGGAAAAAGAGCGCCGCGGCGACTATCTGGGCAAAACCATTCAGGTCATTCCGCATGTGACCAACGAAATCAAAGACTTCATCAGTATTGGCGAAGACGACGTAGATTTTATGCTGTGCGAGATTGGCGGCACTGTAGGCGATATCGAAGGCCTGCCTTTCTTTGAAGCCATCCGCCAATTCGCCAATGACAAGGCACGCGGCCAATGTGTCTTTATGCACCTGACGCTTCTGCCGTTTATCAAAGCCTCGGGCGAGCTAAAGACCAAGCCAACCCAGCATAGCGTCAAGGAACTGCGGTCCATTGGCCTTGCGCCCGACATTCTGGTGTGCCGATCCGAAGGGCCGATCCCAAAGAAAGAACGCGAAAAGCTGGCTTTGTTCTGCAACGTCCGCCCAGATAGCGTGATCGCGGCCCAAGACCTGAAGTCCATCTACGAAGCCCCGCTTGCCTATCACCGCGAAGGTATGGATCAGGCGGTTCTGGATGCATTCGGCATCGCGCCAGCCCCCAAGCCCAACCTGACAAAATGGGAAGACGTCGCGGATCGGGTGTTCAACCCCGAAGGCGAAGTCACAGTTGCCATCGTTGGCAAATATATCCAGCTTGAAGACGCGTATAAATCGATCGCCGAAGCTCTGACCCACGGCGGAATGGCCAACCGGGTCAAGGTCAACATCGAGTGGGTCGACGCCGAGATGTTCGAGCGCGAAGACGCCGCCCCCCATCTGGCTAAATACAACGCCATTTTGGTGCCGGGCGGGTTTGGCGAACGCGGAACCGAGGGCAAGATCAAAGCAGCGCAGTTCGCGCGTGAAAACAATGTGCCCTATCTTGGGATTTGTTTGGGCATGCAGGTCGCCGTGATCGAAGCCGCAAGAAATGTTGCGGGTGTGGCCAAAGCCGGATCAGAAGAGTTTGACCACGAAGCCGGAGAAAAACGATTTGAGCCAGTGGTTTACCACCTCAAAGAATGGGTTCAAGGCAACGAAAAGGTGAACCGCAAGGTGACTGATGCCAAGGGCGGCACAATGCGGCTGGGCGCGTATGATGCGGTTTTGACCGAAGGGTCGCTGGTTGAAAAAATCTACGGCAAACAAACGATTGATGAGCGCCATCGGCATCGCTACGAAGTTGACACAGCCTATCGTGAACAGCTTGAAAAGGTTGGCATGCGGTTCTCGGGAATGTCACCCGATGGTAAACTGCCCGAGATTGTGGAATGGCCAAACCACCCGTGGTTTATCGGCGTGCAATTCCACCCCGAACTAAAATCCAAACCGTTCGACCCGCACCCCTTGTTCAAAGATTTTGTACGGGCTGCCAAAGAAGCCTCACGCTTGGTATAAATGACATCTCCCCTTATA---CTTGGGCTGGAAAGCAGCTGTGACGACACCGCGGCAGCGCTTTTGCGT---------------GGACGTGACGTGTTGGCATCGGTGGTGCTTGGGCAAACGCAGCTGCACGCCGAATTCGGCGGTGTGGTTCCGGAGATTGCCGCGCGCGCGCATGCTGAGCGCCTTGATGGTGCAATTGAACAAGCTTTAGACGAGGCCAAGGTGACTTTGCCAGACATTGACGCGATCGCAGTGACCGCTGGACCGGGCTTGATCGGTGGTGTCTTGTCGGGTGTGATGATGGCCAAGGGGCTGTCAACCGGCCTGGGAAAACCATTGATCGGGGTGAATCATTTGGCAGGCCACGCATTGACCCCACGCCTGACAGACAGCCTGACCTACCCCTATTTGATGCTGCTTGTCTCTGGCGGGCATTGCCAGTTTTTGGTCGTACGCAGCGCCCAAGACTTTACCCGGCTTGGCGGAACAATCGACGACGCCCCGGGCGAAGCTTTTGACAAAACCGCGCGGCTGTTGGGGTTGCCGCAACCGGGTGGCCCGTCGGTGGAACACGTCGCCCGCAACGGCGATGAAAAACGGTTCCGGTTTCCCCGACCTTTGCTGGACCGCGCGGGTTGCGATCTGTCTTTTTCCGGACTTAAGACTGCATTGTTGCGACAGCGCGACCAACTGATTGCCGAAAAATCAGGGCTGACAGAGCAAGACCGCAATGACATGTGTGCCGGGTTTCAAGCGGCCGTGCGGGATGTTCTGGCAGAAAAAACCCGCCGCGCCATTGCTCTTTACTTGGCGGAATCGCCGGCCGAACCTGCCTTGGCGGTTGCTGGCGGTGTTGCGGCAAATATGGCCCTGCGCACTGTGTTAGAGTCTGTTTGTGCCGACAGCAGTATACGTTTTACCGCGCCGCCCTTAAAGCTATGCACAGATAACGCGGCCATGATTGCCTACGCAGGATCCGAGCTGTTTGCCGCTGGGATTACCGATGACATGACGCTGTCCGCCCGCCCCCGGTGGCCGCTGGACAAGACCGCCGTCCCCCTCATCGGGTCAGGAAAAAAAGGGGCAAAAGGATGAATGGCAAAAAACAGATCCAATGAACAATCCGGCCCCCGTCAACGGCAGTTGCGCGTCGCCGAACTAATCCGGCGCAAGCTGTCCGAGATTCTATTGCGCGGTGAAATCCACGACCCTGACCTGAACCGGCTGAACCTGACAGTCGGCGAAGTCCGTGTGTCGCCGGACCTGCGTATCGCAACCGTCTATGTTGTCCCTTTGGGCGGCAAAGGCGAAGACGAGATGAACGCGATCTTGCGTCGCAACAAGGGCGAGATCCGCCATCAAGTCGTGCATGGCTTAAAGCTGAAATTCGCCCCCGAACTGCGTTTCCGGTTTGACGACACATTTGACCGCATGGATGAAACCCGCCGCCTGTTTGAAAATGAAGACGTGCGCCGCGATCTGGACGAA---TAA

>'Roseobacter-denitrificansOch-114'
[truncated: 2,827,429 more chars]
