# Supplementary material for: Genome-based taxonomic classification of the genus Sulfitobacter along with the proposal of a new genus Parasulfitobacter gen. nov. and exploring the gene clusters associated with sulfur oxidation
Source: BMC Genomics. 2024 Apr 22;25:389. doi: 10.1186/s12864-024-10269-3 (PMC11034169; doi:10.1186/s12864-024-10269-3)
Supplement: Supplementary file 3 — Supplementary Material 3 [file 12864_2024_10269_MOESM3_ESM.docx]

>'Brevirhabdus-pacificaDSM-27767'

MNAKELNDKTPDQLKDQLAELKKEAFNLRFQQATQQLENTARMRTVRRDVARVKTVLNQKAAAAAAEEMASADLLTMTSKTSPDKQKALDSALAQIERQFGKGSIMKLGADNPVQEIEATSTGSLGLDIALGIGGIPKGRIVEIYGPESSGKTTLTLHCVAEEQKKGGVCAFVDAEHALDPQYAKKLGVDLDELLISQPDTGEQALEIVDTLVRSGAVNMVVVDSVAALTPKSELEGEMGDSSVGVHARLMSQAMRKLTGSISRSKCTVIFINQIRMKIGVMFGSPETTTGGNALKFYSSVRLDIRRIGAIKDRDEVVGNTTKVKVVKNKVAPPFKQVEFDIMYGEGISKMGELLDLGVKAGVVEKSGSWFSYGDERIGQGRENAKAFLKANNRIALSIEDKIRAAHGLEFDEEAGD--DVVEAMSRLNTAEVEPKWQSAWEEAGVFTATRDESKPKYYVLEMFPYPSGRIHMGHVRNYTMGDVIARYKISTGHNVLHPMGWDAFGMPAENAAIERGGHPATWTYDNIADMRAQMKPLGLSIDWSREFATCDPEYYGQQQAMFIDMLDKGLVYRKNAVVNWDPVDMTVLANEQVIDGRGWRSDAPVERRELTQWFFRISDFADELLSALDGLDNWPEKVRTMQANWIGRSRGLEFAFQLTAPTNGFAELPVYTTRPDTLLGASFVGISPDHPLAKSLEEGNPELAAFSADCRRMGTSEADMEKAEKKGFDTGLRVRHPLNPAWELPVWVANFILMDYGTGAVFACPAHDQRDLDFCRKYDLPVTDTFFALDNPRPVEDEAFVPPKTEPVRWVDHFAGLDVATGQEAIDATIDLAEKQGWGKGVTQFRLRDWGLSRQRYWGCPIPVVHCDACGVVPEKKENLPIELPRDVSFDKPGNPLDRHPTWRDCACPACGQPAKRETDTMDTFVDSSWYYARFTSPRAATPTDAEDAAYWMNVDQYIGGIEHAILHLLYSRFFARAMNQTGHLPEKAIEPFNALFTQGMVTHEIYVTQDDKGRPVYHLPESITESGARESGAPVQVIPSAKMSKSKKNVVDPVNIIEAYGADTARWFILSDSPPERDVEWTAAGAEAAFRHLNRVWRIATDAAEAS-DAAGDADQALDRATHRAIADVTEGVENFAFNAAVAKLYGFTNTLAKSKASGQARRRAVATLAQLMSPMTPHLSEEIWALLGNVGLVVRAPWPKADPALLVEDSVTLPIQINGKRKSEITVAADLPREEVEKIALADKAVQKALAGGQPRKLIVVPGRIVNVVIMAITSANQLELLQTAEAVAREKMIDPSLVIEAMEESLARAAKSRYGAEMDIRVSIDRKTGRATFTRVRTVVEDDAVENYQAELTVKEAKQYKEDPQIGDEIVDEVPPVEMGRIAAQSAKQVILQKVREAERDRQYEEFKDRAGTIINGVVKREEYGNVIVDIGRGEAVLRRNEKIGRESYRPNDRIRVYIKDVRREPRGPQIFLSRTAPEFMAELFKMEVPEIYDGIIEIKAVARDPGSRAKIAVISYDSSIDPVGACVGMRGSRVQAVVNELQGEKIDIIPWNEDQPTFLVNALQPAEVTKVVLDEEAERIEVVVPDEQLSLAIGRRGQNVRLASQLTGLDIDIMTEEEESKRRQAEFAERTQLFMDTLDLDEFFAQLLVSEGFTNLEEVAYVDADELLVIDGVDSDTAGELQARARDHLEAINKKALERAAELGVDQSLVDFEGLTPQMVEALAEDGILSLEDFATCADWELAGGWTTVEGERVKDDGLLEKFDVSLEEAQDMVMTARVMLGWVDPTEMLAEDTGEDAEGEEGQAAEGDAMAHKKAGGSSRNGRDSAGRRLGVKKFGGEAVIPGNIIVRQRGTKWWPGEGVGLGKDHTIFATAEGHVTFHKGLKGRTFISVMPAAEAAEMAKRWYSVSVLSNFEKKIAEQIRHDVAAAGLEDEIEEVLVPTEEVIEVRRGKKVTAERRFMPGYVLVRMEMSDRGYHLINSINRVTGFLGPQGKPMPMRDAEVNQILNRVEEGQEAPRQLISFEVGEKVKVNGGPFEGFDGMVEEVDDDNQRLKVSVSIFGRATPVELEYTEVSKEIMSFTLAIVGRPNVGKSTLFNRLVGRRLALVDDQPGVTRDLREGEARLGDLRFTVVDTAGLEEATDQSLQGRMRRLTERAVDMADICLFMIDARVGITPTDQVFAEILRKRSAHVIVAANKAEGRAGEAGMLEAYSLGLGEPLRLSAEHGEGMEDLLAVLTPLADEFALKAQDSAPETDVELDEEGGEEDPSPRPSRAKPLQVAVVGRPNAGKSTLINAILGEDRLLTGPEAGITRDAISVTTEWDG-APVRIFDTAGMRKRAKVQDKLEKLSVSDGLRAVRFAEVVVVLLDVEIPFEQQDLRIADLAEREGRAVVIAVNKWDIETDRQNKLKELKEEFTRLLPQLRGAPLITVSAKTGRGLDRLQEAILKAHEVWNRRVTTATLNRWLIGMLEAHPPPAPGGRRIKLRYMTQAKTRPPGFVVMCSHPEKLPESYSRYLVNGLRDSFDMPGTPIRLTFRSQADKNPYKDRKKSTPSRLRKHLGKGRADMRIVFMGTPDFSVPVLDALIAAGHEIAAVYCQPPRPAGRGKKPRPTPVHARAEALGLEVRHPETLRDADEQARFAALEADVAVVVAYGLLLPQAVLDAPRHGCLNIHASLLPRWRGAAPIHRAIMAGDARTGVCIMQMEAGLDTGPVLLRAEIPIGPTDTTGDLHDALSALGAARIVEALERLEGLTPVPQPEEGVTYAAKIDKAEARIDWSRPAAEVDRKIRGLSPFPGAWTEMAGERVKLLRSRAEGGAGAPGEVL-TGFTIACGEGAVEVLEAQRQGKRAAGAAEFLRGNTLP--ERMGD-MSDSTGYQVLARKYRPATFADLIGQDAMVRTLRNAFAADRIAQAFILTGIRGTGKTTTARIIAKGMNCVGTDGEGGPTTDPCGTCEHCVAIAEGRHVDVMEMDAASRTGVGDIREIIDSVHYRAASARYKIYIIDEVHMLSNSAFNALLKTLEEPPAHVKFIFATTEIRKVPVTVLSRCQRFDLRRIEPEVMIAHLQSIAGKESAQINDDALALITRAAEGSVRDAMSLLDQAISHGAGETTADQVRAMLGLADRGRVLDLYELVMAGDAAGALNELSAQYSDGADPLAVLRDLAEITHWISVIKITPEAAEDPTVGPDERARGQGLAQRLPMRAMTRMWQMLLKALEEVSAAPNAMMAAEMAVIRLTHVSTLPSPEELIRRLQD-TPAPPAAFAPGGGAAPGPSAGGQSGSQQGSASGSAPVTAGPATAAAPSQQTALARFATFQSVVELIRQNRDVKLLVEVETTLRLVSYQPGRIEFQPDDRAPRDLAQRLGQRLQGWTGARWAVSVTAEGGGETIAEGRDAERLAMEREAAKHPLVQAVMDAFPGAKISNVIPFETIQAEAAIQALPEVDDEWDPFEETMPKMKTKSSAKKRFKMTASGRVKAGQAGKRHGMIKRTNKFLRDARGTTLLSKPDENIVKKYMPYARMNLFAEMRALVLDSLSDLQDAGGLPRDLDMANVAVEPPRDAAHGDMATNAAMVLAKPAGMKPRDIADALAARLLEDGRIASADVAGPGFLNLRLSPAVWQGVVRAALREGVDFGRSDMGQGRKINVEYVSANPTGPLHVGHTRGAVFGDALCSLLDFSGHDVTREYYVNDGGAQVDVLARSVYLRYLEAHGQEVAFEDGTYPGDYLIEVGQALKDKVGDAYVGKGEDAWLEEVRDFATDAMLGLIREDLHLLGVKMDNFFSEKSLYGTGRIEAALNSLETKGLIYEGVLEPPKGKTPEDWEPREQTLFRSTAHGDDVDRPVKKSDGSWTYFAPDIAYHFDKIERGYDELIDVFGADHGGYVKRMKAAVSALSDGAVPLDIKLTQLVRLFKNGEPFKMSKRAGTFITLRDVVEQVGADVTRFVMLTRKNDAPLDFDFARVLEQSKDNPVFYVQYAHARIRSVMRKAEAAGIASDAATLAGADLDLASDPAELALAAKLAEWPRLIEIAARTHEPHRVAFYLYELASELHGLWNLGNARPELRFLQEGDIAATQAKMALIQATAVVISAGLGILGVTPVEEMRMAADDPLPPLRQVIAAHGLSARKSLGQNFLLDLNLTAKIARQAGDLAGSDVLEVGPGPGGLTRGLLAEGARKVLAIEKDSRCLPALEEISAAYPGRLEVLNADALEIDPTAHLTAPIRVVANLPYNVGTELLVRWLTPRDWPPFWSSLTLMFQKEVAERIVARPGSKAYGRLALLAQWRADPAIVMHLPPQAFIPAPKVHSAVVHLTRLDAPRYPADAAILSRITKAAFGQRRKMLRASLKGTVPDVENVLRDVGIDPTARAETLDLEAFCALARRVEELMDIIAQIEAEQIEALGKTFPDFKAGDTVRVGFKVTEGTRTRVQNYEGVCISRRNGAGIAGSFTVRKISFGEGVERVFPLHSTNIDSIEVVRRGKVRRAKLYYLRSRRGKSARIAEKTNYKPLSGAKAMANSKRTLFLKRRLRVRNKLRKMNAGRPRLSVHRSNKNISVQLIDDVNGVTLASASSLEKDLGVLGKNNVEAAAKVGTAIAERAKKAGVEECYFDRGGFLFHGKVKALADAARENGLKFMFAVLKTGGKQYKVQSGDVLRVEKLAAQAGDTVQFNDIMMLGGDKPVVGAPLISGAAVQAEVIDQIKGEKVIHFVKRRRKHSSKRTKGHRQQLTLLRVTEILAEGGDKSGVKAAVGAGSVAGV---AAAAA--APKTPAKKAAAPKAEAPKAEAAAPKAAKADDKADDLKKLSGVGPALEKKLHEAGVTTFAQIAGWSAADIAEMDEKLSFKGRIEREGWVDQAKELTKGMSRVKGGTTTHARHKKVIKAAKGYYGRRKNAFRTATQAVDKANQYATRDRKARKRNFRALWIQRINAAVRLHDDSLTYSRFINGLGLAGIEVDRKVLADLAVHEPEAFGAIVDQAKAALN-MQVTETKNEGLERAYTITVTAKELDDKVGEKLVEAQPEVELKGFRKGKVPMALLKKQFGQRLLGEAMQESVDAAMAKHFEDSGDRPAFQPKVEMTNQDWKEGDDVVVDMSYEALPEVPDADFSKIKLEKMVVKPEESAVDDALKNLAESAQNFTDRDAKEKSQDGDQVVIDFLGKVDGEAFEGGAAEDYPLVLGSNSFIPGFEEQLVGKKAGEEVEVKVSFPGEYGAAHLAGKDAVFECKVKAVKAPAAAEIDDELAKKYGSESLDALKEQIRERLAAEYNGAARAVMKRKLLDELDELVSFELPPTLVDAEAKQIAHQLWHEENPDVQGHDHPEIETTDEHKKLAERRVRLGLLLAELGRKQEITVNDQELTQAIMNQARQYPGQERQFFEFAQQNAEFRQQIQAPIFEDKVVDYIFELAKVEEKEVTKEELEKAVEELEQEMKLHELHDNPGATKKRKRVGRGPGSGTGKTAGRGIKGQKSRSGVSINGYEGGQMPLYQRLPKRGFTPPNRKKFAVLNLSLLQKFLDDKKIDGSKAITEDVLVESGLVRRKLDGVRILAKGEFNAKVNLEVTGASKSAIEAVEKAGGSLKVSTPAATSEQAMIQMQTNLDVADNSGARRVQCIKVLGGSKRKYASVGDIIVVSVKEAIPRGRVKKGDVRKAVVVRTAKEVRREDGTAIRFDRNAAVILNNNNEPVGTRIFGPVVRELRAKNFMKIISLAPEVLMRHARGYRRLNRTHEHRKALFANMAGSLIEHEQIKTTLPKAKELRRIVEKLITLGKRGDLHARRQAGAQLKQDEYVAKLFDVLGPRYAERQGGYVRVLKAGFRYGDMAPMAIIEFVDRDVDAKGAGDRARLEAEEAAE-MLQPKRTKFRKQHKGRIHGEAKGGSTLNFGTFGLKATQPERITARQIEAARRAMTRHMKRQGRVWIRIFPDTPVTSKPTEVRMGKGKGSVDYWAAKVKPGRVMFEIDGVSEAVAREALRLAAMKLPIKTRTVVREDWMSDIRLTNTATRSKELFRPIDPQNVRMYVCGPTVYDRAHMGNARPAIVFDVLFRLLRHVYGPEHVRYVRNITDIDDKINARAAETGRPIREITDETAAWYLEDMAALGNLTPTVTPRATEFVDGMIAMIEQLIASGHAYEAEGHVLFSVESYDRYGRLSGRSIDDMIAGARVEVAPYKRNPMDFVLWKPSGPDLPGWDSPWGRGRPGWHIECSAMSRDLLGESFDIHGGGNDLMFPHHENEIAQSCCANPNSDFARVWMHNEMLQVEGKKMSKSLGNFFTVRDLIDQGIPGEVIRFVFLSTHYGKPMDWTERKAQEAAATLRKWRALCRGVTAAPQPDAAVLACLADDLNTAGAITEMHRLASAGQAAELLASAAMLGLLEPGMGDWAEAPAADLSAFAEALAARRAEAMVSKDFSQVDALKQALVAAGVDVRMSKTGVDLVPGPGFDPAAIEGLIMAK----PKSDPNYKVVAENRRARYDYAIEEDLECGIMLEGSEVKALRQKSANIAESYAAVEDGELWLINSYIAPYEQARTFGHDERRRRKLLVSRKELARLWNATKREGMTLVPLVLYFNHRGMAKIKIAIAKGKKNQDKRANEAKRDWQRQKARLLRQG-MSAKAEHYDVIRKPIITEKATMASDAGAVVFEVAMDSNKPQIKAAVEGLFGVKVKAVNTTITKGKVKKFRGQPGRRKDVKKAYVTLEEGNTIDVTTGLMGKEKNPRRVADNEALAKTRMLRTSPQKLNLVAAMIRGKKVDKALADLTFSKKRIAADVKKCLQSAIANAENNHNLDVDELVVAEAYVGKNLTMKRGRPRARGRFGKIVKPFSELTIKVRQVEEQAMASAAEQMAANMSWGAFGKATELKQRILFTLALLIVYRLGTYIPVPGIDGTALRQFVQEAATGLGGILNMFTGGAIGRMGIFALGIMPYISASIIVQLLTAMVPSLEQLKKEGEQGRKKINQYTRYGTVFLATFQAYGLAASLEAGDLATDPGLYFKASAVITLVGGTMFLMWLGEQITSRGIGNGISLIIFVGIIAEVPAALAQFFASGRSGALSPAVIVGVIIMVILTIAFVVFMERALRKIHIQYPRRQVGMKVYDGGSSHLPIKVNPAGVIPAIFASSLLLLPTTISTFSGSQTGPVMSTILAYFGPGQPLYLLFFAAMVIFFTFFYTANVAFKTDDVAENLKNQNGFIPGIRPGKRTEEYLDYVVNRILVLGAGYLTLVTLLPEILRSQLAIPFYFGGTSVLIVVSVTMDTIQQVQSHLLAHQYEGLIEKSQLRG---KRRGKKGTARRMQFLDLAKVYIRSGGGGGGAVSFRREKYIEYGGPDGGDGGKGGSVVAEAVEGLNTLIDFRYQQHFFAKNGVPGMGKQRTGADGEDVILRVPVGTEILDEDEETVIADLTEVGQRVVLARGGNGGFGNLHFKSSTNQAPRRANAGQPGVERTIWLRLKLIADAGLLGLPNAGKSTFLAASSNARPKIADYPFTTLHPNLGVVGIDEVEFVMADIPGLIEGAHEGRGIGDRFLGHVERCSVLLHLVDGTSEDVAEDYRVIIHELESYGGALADKPRITALNKIDALDDEERAEKKAALEAATGGNVLMMSGVSREGLPEVLRAVRARIDADKLRQRKAEEPESWRPMAAKLKKGDKVVVLAGKDKGKEGEITRVMPAANKAIVDGVNVAIRHTRQSQNSQGGRVPTPMPIDLSNLALLDSNGKATRVGFRTEDGKKVRFAKTTGDVI--MLRSGVIAKKVGMTRLFMDDGKQIPVTVLQLDKLQVVGTRTADEHGYSAVQLGAGTAKAKRVSKAMRGVFSAVKVEPKRKIAEFRVAPENLIEVGEEITANHYFEGQFVDVSGTSIGKGFAGAMKRHNFGGLRATHGVSISHRSHGSTGQCQDPGRVFKGKKMAGHMGAARVTTQNLQVIRTDADRGLIMVKGAVPGSKGGWVTIKDAVKKPIPENVIYPAALKSAAEEAERLAKEAAEQAAAEAEAAEKAAAEAAAAEQAAALKEAEAS-----DDTAAPEGGDNNES--MALKSYKPTTPGQRGLVLIDRSELWKGRPVKSLTEGLTKKGGRNNTGRITMRRRGGGAKRLYRIVDFKRTKLDMTATVERIEYDPNRTAFIALIKYEDGQQAYILAPQRLAVGDSVVASAKADVKPGNAMPFSGLPIGTIVHNIELKPGKGGQIARAAGTYAQFVGRDGGYAQIRLSSGELRMVRQECMCTVGAVSNPDNSNQNLGKAGRNRHKGIRPSVRGVVMNPVDHPHGGGEGRTSGGRHPVTPWGKPTKGARTRNKNKASSKLIIRSRHAKKKGRMLDAATYTPRLKTLYTDEIRAKLKEEFGYANEMQIPRLDKIVLNIGCGAEAVRDTKKAKSAQEDLTAIAGQKALITKAKKSIAGFRVREEMPLGAKVTLRGERMYEFLDRLITVAMPRIRDFRGVSGKSFDGRGNYATGLKEHIVFPEINFDKVDEVWGMDIVICTTAGTDAEAKALLKHFNMPFNSMKADAIKLDGAKAGSVELDDAIFGLEPRADILHRVVRWQRNNAQQGTHKVKTRSETSYSTKKIYRQKGTGGARHGDRNAPIFRKGGIYKGPTPRSHGHELTKKFRKLGLRHALSAKQSAGELVILDTLEKDAKTAVLSRQVKDLGWKRALVIDGATVDENFARAARNLDGVDVLPSMGANVYDILRSDTLVLTKAGVEALEARLKMENVVLVIHLLLAMALIGVVLLQRSEGGGLGIGGGGGGVVSGRSAATALGKITWFLAAGFIATSITLTIFAAQNSAGQSVIDRLAPAPAAESGATDGALPPASDLLPPSSGSATPLTPPRAMSFFKKLKDRMLKSSSRLEAGLDAIVEEGQEPSGQAPAAPPSDA--------------APAPAPVPPADPA-----------SPATAPSGAPVP--------------------AAPTPEAQTPVSP---------EPESKGGLIDRLMGR-GGQEVARRTLDDPMLEQLEELLITADMGVDTALRVTANIAESHFGKRVSAEEVKRLMAEEIARIMEPVAQPMPIYSKKPQVVLVVGVNGSGKTTTIGKLASQFRAVGKSVIIAAGDTFRAAAVEQLQVWGERAGVPVLTAPEGSDPASLAFDAMLKAEAEGADLLLIDTAGRLQNRADLMEELAKIVRVIRKRDPEAPHNTLLVLDATTGQNALSQVETFRKLADVSGLVMTKLDGTARGGVLVALADRFGLPIHAIGVGEQIDDLAPFDPEDYACALTGLEVMTDDTWGRVSKLLKRSVGNNNYTTWIKPLRFLKEEAGVVTFGVPTVFIGDWVSRNFGDQIRQHLMAEGEAVERLRFVVPPAQASADQAPAGKAQAARAGRDPEDSAASVIPGAPLDARFTFDSFVVGKPNELANAAARRVASSGPVTFNPLFLYGGVGLGKTHLMHAIAWELQARSPEKKVVYLSAEQFMYRFVQSLRDRSTMDFKQLFRSVDVLMVDDVQFIAGKNSTQDEFFHTFNALVDQGRQIIISGDRSPSEMDGMDERIRSRLQWGLVVDLHPTDYELRLGILQSKVEQYRRDYPQLDIANGVLEFLAHRISTNVRVLEGALTRLFAFASLVGREITLDLTQDCLADILRASDRKVTIDEIIRKVSEHYNIRMSDILSPKRTRTIARPRQVAMYLAKQLTSRSLPEIGRRFGGRDHTTVLHAVRKIEELRSIDNQIDEDVELLRRMLEAMLGLGTIAKKVFGTPNDREVKARRPLVEKINALEPQFTDLTDEQIIEKTAEFRERLAKGEALDDLLPEAFANVREAARRTLGLRAFDVQLMGGIFLHQGNIAEMKTGEGKTLMATFPAYLNALTGRGVHIVTVNDYLARRDAEWMGKVYAALGLTTGVVYPQQPEQEKAEAYGADVTYATNNELGFDYLRDNMRMDLDEMNQRDHYFAIVDEVDSILIDEARTPLIISGPSEDRSELYKTIDTVVPSLSDEHFTIDEKTRNVTYTDEGNEFLEQELLRQGILPEGQSLYDPESTTLVHHVTQALRAHKLFQRDKDYIVRDDEVVLIDEFTGRMMSGRRLSDGLHQAIEAKENVSIQPENVTLASVTFQNYFRLYDKLGGMTGTAATEAEEFAEIYKLGVIEVPTNRPIARVDEHDAVYRTAKEKYDAIRETIEEAHKKGQPILVGTTSIEKSEFLSQMLKERGVPHNVLNARQHEQEAAIVADAGKPGAVTIATNMAGRGTDIQLGGNVDMKVLEALATDPEADPAALRERAEAEVAGDKKKVLEAGGLFVLATERHESRRIDNQLRGRSGRQGDPGRSAFYLSLEDDLMRIFGSERLDNVLGKLGMKEGEAIIHPWVNKSLEKAQAKVEARNFDIRKQLLKFDDVMNDQRKAIFGQRLEIMESKDVNEIVEDMRHQVIDDLVDFYIPARSYADQWDGEGLYAAVIEKLGVDAPVIAWTQEEGVDDSDIRERLYKATDEFMAGKAAKFGPEQMRRIEKQVLLQTIDAKWREHLVTLEHLRSVVGFRGYAQRDPLNEYKNESFQLFESLLNSLREEVTEKLAQLRPLSEEEQKQMLAQLIEQQRALQGAGAQP-----AAPAGPQ-AAAQPDGAGETAVAEVGRNDPCPCGSGKRYKHCHGAVTMNLSAELDQFSKRIGYRFRKPERLVESVTHASISTATRPDNQRYEFLGDRVLGLVMAEALLKADPAASEGRLAPRFNALVRKETCAEVARDADLGSVLKLGRSEQMSGGRRKEALLADAMEAVIAAVYLDGGFEAARDMILRLWGDRIEKVEADARDPKTALQEWAQARGQQPPAYREVGRDGPDHAPQFTIEARLADGLSEQATAGSKRQAEQAVARKLLERVEKNGMAKTGKRTAAAKAAFADKHDLTVEEAIALIKDNSKTKFDETVEIAMNLGVDPRHADQMVRGKVTLPNGTGKTVRVAVFARGAKADEAKEAGADIVGAEDLMETIQGGEINFERCIATPDMMPIVGRLGKVLGPRNLMPNPKVGTVTMDVAEAVKAAKGGEVQFRAEKAGVVHAGVGKASFEVDKLVENVRAFVDAVAKAKPTGAKGAYMKKVSLSSTMGPGVSVDITSATGNMADDIEIDLDDLGHRMDGAMTALRSEFQSLRTGRASASMLDTITVNAYDTVTPLNQVGTVNVPEPRMLTVTVWDKQLVNKVEKAIRESGLGINPVMDGTVIRLPIPELNEERRRELTKIAAQYAEAARVAVRNVRRDGMDQIKNAKSAGMSEDDQKIWSGEIQEMTDKHIAAIDAALESKQAEIMQVMAKKVAGTMKLQVPAGQANPSPPVGPALGQRGINIMEFCKAFNAKTQEMEQGAPCPTVITYYQDKSFTMDIKTPPASYLLKKAAKLKSGAKTPSRETAGYVTVAQVREIAETKMKDLSANDIEGAMQIILGSARSMGIEVKMFETLSERLSGVFDKLGKQGALSDEDVKTALREVRVALLEADVSLPVARDFVAAVQEKATGQAVTRSVTPGQQVVKIVHDELVHVLAGDGTAGQLKIDNPPAPILMVGLQGSGKTTTTAKLAKRLKEKSGKRVLMASLDVNRPAAMEQLAVLGVQVGVDTLPIVKGQTPVEIARRAKQQATMGGYDVYMLDTAGRLSIDEALMAEVEAVRDVTSPRETMLVVDGLTGQDAVHTAENFDARIGISGVVLTRMDGDGRGGAALSMRAVTGKPIKFVGLGEKLDALEEFHPERVAGRILGMGDIVSLVEKAQETIEAEQAERMMKRFQKGQFNMNDLKSQIEQMQKLGGMEGVMGMLPGMKKMSKQVEAAGMDDTLLKRQIALIQSMTKKERANPQLLQASRKKRIAKGAGLEVSELNKLLKMHRQMADMMKKMGKMGKKGMMRGALGQMFGKGG--PS----QADIEAAKAQMGGAGGQMPAGLPGMGGGLGLPPGLSGFGKKKMVPADKLAQILERFQYIEARMSQGS--GDIAQLGREYAALRPVVEQVEGYNRLIADIAEAEAMLDDPEMRALAEDELPALRTRLPEAEAAVRLALLPKDAADERAAIVEIRPGTGGEEAALFAGDLWRMYQRYAEGRGWSVSVIEESLTELGGLKELVANVRGQGVFARLKFESGVHRVQRVPSTESGGRIHTSAATVAVLPEAEEVDIDIPSTDIRIDTMRASGAGGQHVNTTDSAVRITHIPSGIVVTSSEKSQHRNREIAMQVLRARLYDAERQKVDSARAADRKAQVGSGDRSERIRTYNFPQGRMTDHRINLTLYKLDQVMQGDLDEIIDSLIEEDQARRLAEMEQMDRAQKEKLVEELGQIFESSGVVVVAHYEGLTVAEMQDLRARARDAGGAVRVAKNKLAKIALDGKPCASIADYLTGMTVLTYSEDPVAAAKVAEDFAKENKKFEILGGAMGENALDRAGVEAVSKMPSREELIATIAGMIGAPASNIAGAIGAPASNIASILSTIEERAEAMKTFTATPADIDKKWILIDAEGVVLGRLAAIVATRLRGKHKPSFTPSQDMGDNVIVINADKIQLTGNKRMKPNYWHTGYPGGIKSRTTGQILEGEHPERVVIQAVKRMLPGGKLSRQQMTNLRVYAGAEHGHEAQSPEVLDVKSMNSKNTRS-MTAAMAAILGAADDPDPSRPPTALGLAVSGGSDSLALMHLAAAWAAPRAIRLRVATVDHRLRPESGTEAREVAHAAAALDLPHEILEWTDGPGAGNLQANARDARRRLLGDWAARHGLTGVLTGHTADDQAETVLLRLARGSGVDGLAAMRPGRPGRSL-FLRPLLGHRREELRDLLRAKGLTWAEDPGNEDSVYDRVKARRALALLAPLGIDVEGVNRTADAMARAREALERRGAEAAAAMTREEGADLLIEVAPWRLLDDETRLRLLAAGLMWVGGQDYRPRLRALEDTVRAAAAGRRATLHGVVIHPAAGWLRLYREPSALIGVRARPGDTWDNRWILAREAGTTEGAQVEMLGEEGLTAL-DPRPAGVPRDSLLGLPAVWRAGRVICVPHLGMGGGFRATRAGEQE-FTRTLLSHMADLKKLAEEIVGLTLLEAQELKTILKDEYGIEPAAGGAVMMAGPADAGDAGEEQTEFDVILKAAGAQKINVIKEVRAITGLGLKEAKELVEAGGKVKEGVSKEEAEDIKGKLEAAGAEIELKMSRIGKKPVELPSGVSATVSGQTVEVKGPKGTRSFTATDDVTISVDDNVISIAPRGSSKRARQQWGMSRTMVGNLVTGVSTGFKKELEINGVGYRAQMQGNTLKLSLGLSHDVNFEVPEGVTVTAPKQTEIVVEGIDQQLVGQVAANIREWRKPEPYKGKGIKYKDEYIFRKEGKKKMDVILLERVAKLGQMGEVVSVKEGYARNYLLPQGKARRASDANLKMFEEQKAQLEARNLETKKEADSLAAKIDDQQYVVIRSASDAGALYGSVTTRDAADAINEDGVSIDRKQIVLSEPIKYLGLHTVTVVLHPEVTASVKLNVARSPEEAELQASGKSIQELAAEEEAEAEFEIAELFDDIGAAGLDDDDR------DERDSDDETEEN-MSDQDGKKTLGLRGGPRSGQVKQSFSHGRTKNVVVETKRKRVVVPKPGASKAAGAASNRGGDASKRPAGITDAEMERRLKALQAAKAREAEEAERREREERERAEDRERRRAEAEAKEREEREREERARQKAEEDERKQREAEEAKQ-AAQPA--PQERAEPQADAGPAATPRKERDDRPKREAKGAGDR--RRSGKLTLNQALSGGEGGRQRSMAAMKRKQERARQKAMGQNVEREKVVRNVNLPEAITVQELANRMAERVADVVKSLMTSGIMATQNQTIDADTAELIIEEFGHKVVRVSDADVEQVIDTIDDKPEDLRNRPPVITVMGHVDHGKTSLLDAIRNAKVVAGEAGGITQHIGAYQVQAADGQILTFLDTPGHAAFTSMRARGAQVTDIVVLVVAADDAVMPQTVEAIHHAKAAGVPMIVAINKIDRHEANPDKVRTDLLQHEVVVEKMSGDVQDVEVSAIKGTGLDELLEAIALQAEILELKANPDRAASGAVIEAQLDVGRGPVATVLVQNGTLRRGDIFVVGEQYGKVRALINDKGERVDEAGPSVPVEVLGLNGTPEAGDVLNVVETEAQAREIAEYREKAAKEKRAAAGAATTLEQLMAKAKDDENVSEMPILVKADVQGSAEAIVQAMEKIGNEEVRVRVLHSGVGAITESDIGLAEASGAPVFGFNVRANASARNSANQKGVEIRYYSVIYDLVDDVKAAASGLLSAEVRENFIGYAEIKDVFKVSNVGKVAGCLVTEGVARRSAGVRLLRDNVVIHEGTLKTLKRFKDEVAEVQSGQECGMAFENYDDIRAGDVIEIFEREEVERTLSMAKQKKTPRPKAETPKGFRDYFGAEVQGRKAMLDIIGQVYHRYGFDALESSAVETVEALGKFLPDVDRPNAGVFAWQEDAEGEKPGDWLALRYDLTAPLARVYAQHRNDLPTPYRRYAMGPVWRNEKPGPGRFRQFYQCDADTVGAPSVAADAEICAMLSDTLEAVGIQRGDYIVRVNNRKVLNGVMEVAGVADPDFADERGIVLRAIDKLDRLGTGGVRALLGAGREDESGDYTKGAGLSEAQADVVMGFMEARREDGAATVARLSELVGDSVVGREGVDELRQIADLLAAQGYGPDRIVIDPSVVRGLGYYTGPVFEAELTFEILDEKGRKRQFGSVAGGGRYDDLVKRFTGQSVPATGVSIGVDRLLAALAAKGLGETAEEGPVVVTVMDRERMADYQSMVAELRNAGIRAEVYLGNPKNFGNQLKYADRRGAPVAVIQGGDEAARGVVQIKDLKLGAEIAQSASLEEWKAQPAQSEVPRTDLVAAVRGILARTAMDELRQKYLAATADAADEAALEAIRVQALGKKGEISLKMRELGKMTPEERTTAGPALNALKDEINSALAAKRAALGDAALEARLRDEWLDVTLPGRPRRTGTIHPISQVTEEVTAIFADMGFSVAEGPQIESDWFNFDALNIPGHHPARAEMDTFYMARAEGDDRPPHVLRTHTSPVQIRTMQERGAPLRIIAPGRVYRCDYDQTHTPMFHQVEGLALDKDLSMANLKWVLEEFVRAFFEVDEVELRFRASHFPFTEPSAEVDIRCSWEGGQLRVGEGDDWLEILGSGMVHPKVLAAGGIDADTWQGFAFGMGIDRLAMLKYGIPDLRAFFDSDLRWLRHYGFASLDQPTLRGGLSRMELAEDAGLDLVEISPNAAPPVCKIMDFGKFKYEQQKRESEARKKQKTIEVKEVKFRPNTDTHDYEVKMRNVFRFLEAGDKVKITLRFRGREMAHQNLGRELLERVAEDVKELGKVENMPKMEGRQMIMMIGPIAKMKFTVSWLGDHLETDASVDEICDTLTDLGLEVEGVENPADTLGAFRICRVIEAGPHPDADRLRLCRVETPQGEVQVVCGAPNARTGLVGVFAPVGTHVPGTGVDLKPGVIRGVESNGMLCSERELMLSDNHDGIIDLPEAPLGERFIDYRGVN-----DPVIEIAITPNRPDALGVRGIARDLAARGLGRLKDAPAVSVASGGPSPINVSIDDDVLDGCPVFCGRMIRGVRNGPSPEWMQKRLRAIGLRPISALVDVTNFLTYDRNRPLHVFDVDKVQGDLRVHYAKGGERLVALDDKEYELGAGMMVISDDQGVESIAGIMGGAPTGCTEETVNVFVESAYWNPVAIAHAGRALKINSDARYRFERGIDPAFTPEGLDIATALILEICGGEASEVVCAGAVPDTDRAYRLDTDRVQSLVGMDIPADTQRQTLTALGFRLEGDMAHVPSWRPDVMGQADLVEEVARIASLTRLEGRPMPRV-PGVPRPILTPLQRRESAARRAGAALGYNECVTYSFIDQPTAAMFGGGDFATMLENPISSDMSHMRPSLLPGLLQAAARNQARGFTDLALFEVGPVFEGGEPEDQRLQMSGLLVGHNSPREPHGGRRPVDIYDAKADAEAVLAELGAPAKVQIARGAEEWWHPGRHGVMRLGPKKVLAVFGELHPKVLNALDIKGPAVAFTIWPSEVPVPKAKGATRPALEMRDLQAVERDFAFVVDEGVEALGLVNAAAGANKALIEDVRVFDEFTGTQMGEGKKSLAITVRLQPRDKTLTEADIEAVSAKIVEKVAKATGGTLRGMHAFRTHTCADLDKSNVGDTVRLSGWVHRVRDHGGILFIDLRDHYGVTQVLADPDSPVFADVEKVRSEWCIRIDGTVKARDPELVNPKIPTGEVEVFVRDIEVLGEAAELPLMVFGDQEYPEETRLRYRFLDLRREKLQRNMTLRSDVVASIRKRMWGADFREYQTPIITASSPEGARDFLVPSRLHPGKFYALPQAPQQFKQLIMVSGFDKYFQIAPCFRDEDPRADRSPTDFYQLDMEMSFVTQQDVFDTIQPVVQGIFEEFGGGRKVDTTWEQISYRDAAMWYGTDKPDLRNPIKMQDVSEHFRGSGFAIFAKLLEQDGTQIRAIPAPKGGSRKFCDRMNAFAQKEGLPGMGYIFWRET---DGQMEAAGPLAKNIGPERTEAIRQQLDLGVGDAAFFLGGKPSAFETVAGKARTEIGNELGLTDTDRFAFAWIVDFPMYEKDEETGAIDFSHNPFSMPQGGLEALQGDPLEVLGYQYDLACNGYELISGAIRNHKLDIMYKAFELAGYGPEEVDKRFGGMVKAFRYGAPPHGGCAAGIDRIVMLLADEANIREVIMFPMNQRAEDLMMEAPSEPTNEQLRELRLRVIPAEMTDLSHIRNFSIVAHIDHGKSTLADRLIQSTGTVQDRDMKEQLLDAMDIERERGITIKANTVRIDYVADDGRKYVLNLIDTPGHVDFAYEVSRSMRAVEGSLLVVDSTQGVEAQTLANVYQAIDADHEIVPVLNKIDLPAADCERVAEQIEDVIGIDASGAIQVSAKTGIGIHETLEAIVKLLPPPQGERDAPLKAMLVDSWYDAYLGVIVLVRIIDGVLKKGDRIRMMHNDSIHHVDRIGVFRPPMQTVDELGPGEIGFLTASIKQVRDTRVGDTITHEKKGADKALPGFKPSQPVVFCGLFPVDNAEFEDLRDAIEKLALNDASFSFEMETSAALGFGFRCGFLGLLHLEVIRDRIEREYDIDLITTAPSVIYNVHMRDGEMIELHNPADMPDLTHVDHIEEPRIKATILVPDEFLGDVLKLCQDRRGIQMDLTYAGSRAMVVYDLPLNEVVFDFYDRLKSVTKGYASFDYQMIGYRQDHLVKMQILVNDEPVDALSTMVHRDRAETRGRAMVEKLKDLIPRHMFKIPIQAAIGGRIIARETLAALRKDVTAKCYGGDATRKKKLLEKQKAGKKKMRQFGKVDIPQEAFINALKMDGMSLPPGFIDELRTRLSLSQVVGRKVMWDPRKSNQGKGDFWAPCPFHQEKSASFHVDDRKGFYYCFGCQAKGDAISFVRETENVGFMEAIRILAQEAGLPVPQSDPRAQARTDRRSALAEVMEQAVRFFTMQLGTAAGGAARQYLDGRGLDEGARKRFAIGYAPDARQALWSHLTGAGVAPDMIVDAGLAARPDDGGAPYDRFRGRIIFPIRDGRGRTIALGGRAMSANARAKYLNSPETELFDKGRSLYNLGPAREAAGKRGTLIVAEGYMDVIALVQAGFEATVAPLGTAITENQLRMLWQISPEPVIALDGDAAGLRAAQRLIDLALPLQEAGQSLRFAILPEGKDPDDLIRAEGAQAMQRVVDGAVPMVRLLWQRETEGRVFDSPERKAALDKSLRDILRRIRDRSIRGHYADEIKRLRWDLFDPNRGSGQGLGQGGGRGGNRRWGDAPAQPLDSTRRSLLAM-AEPGVEEHLREAVILAALVVHPAILRDFEAELETLELA-PAHEPVRAALLRHGFADLAELPERIATDAG-GALEKLFALSHVQISPPFRHRDDAELAAQTVRQELSKLASRRGVQREIDEASQDLEGLADEGLTWRLGQAAEARDRAERWGVDDS--PDMGEDRDALSK----MLQDLIDGEVWVKKTR--MCADAPDYKTTLNLPQTEFPMRAGLPKREPGWLERWARIGVYERLRDKETRQPFTLHDGPPYANGHLHIGHALNKILKDMVIRSQQMSGRDARYIPGWDCHGLPIEWKIEEKYRQKGLDKDAVPIVDFRQECRSFAEGWVDIQRDEFKRIGITGNWDNPYLTMDFHAERVIAEEFQKFLMNGTLYRGSKPVMWSPVEKTALAEAEVEYHDHKSHTIWVPFKIREG--APADMADARVVIWTTTPWTIPSNKAVAFNPKIAYGLYRVDGTEEESWTAQGDLYLLADKLADEVLSRARVTAS--TRLRDVSADELSGLTLIHPFNGVEGAEGFWDYDVPMIDGDHVTDDAGTGFVHTAPSHGADDFECFVRRNWLDR-MTYNVGEESEFLDHVPFFAGLQVFDRKGKEGKANAAVIDKLVAASGLIARGRVTHSYPHSWRSKAPVIFRNTPQWFAAIDRAVGDGQDEYGTTIRERALTSIDKVTWTPKTGRNRLYSMIEARPDWVLSRQRAWGVPLTCFVKKDTQPTDPDFLLRDPVVNARITEAFEAEGADAWYKDGAKARFLGDDHDHDAYEQVFDILDVWFDSGSTHAFVLRDREDGSEDGLADLYLEGTDQHRGWFHSSMLQACGTRGRAPYRGVLTHGFTLDGKGNKMSKSLGNTIAPEAVIKQYGADILRLWVAQSDYTADLRIGDEILKGVADSYRRLRNTMRFMLGSLSGFSEADRIAPEDMPELERWVLHRLAELDHRVRTGYAAYDFQGVFQALFNFATVDLSSFYFDIRKDVLYCDGDTVERRAARSVLDILYHRLTTWLAPILVFTMEEVWLERFPGDASSVHLQDIPATPGDWRDDPLAAKWAGVRRVRRVVTAALEEQRRDKVIGASLEAAPVVHVTDPAVLAQLRTLPFADICITSAVELTGDPIPDEAFRLPEIDGVGVVFEQAEGEKCQRCWKILPDVGHHAHAQVCGRCDAALKAIQSQNIRIRLKAFDYRVLDSSTQEIVNTAKRTGAQVRGPIPLPNKIEKFTVLRGPHIDKKSRDQFEIRTHKRLLDIIDPTPQTVDALMKLDLAAGVDVEIKV--MAEDIKTLDGLRDAVTGGVQGT-----ADMINREPQRDELGRSYATGKRKDAVARVWIKPGSGKVTVNGKPINAYFARPVLQMILKQPFQVAGVEGEFDVTATVKGGGLSGQAGAVKHGISKALQLYEPSLRGALKAAGFLTRDSRVVERKKYGRAKARKSFQFSKRMPTIQQLIRKPRQPKVKRSKSLHLEGCPQKRGVCTRVYTTTPKKPNSAMRKVAKVRLTNGFEVISYIPGESHNLQEHSVVLIRGGRVKDLPGVRYHILRGVLDTQGVKDRKQRRSKYGAKRPKMARDRRQTKRKVSKNIATGVAHVNSSFNNTKILISDVQGNAIAWSSAGTMGFKGSRKSTPYAAQMAAEDVGKKAQEHGVKTLEVEVQGPGSGRESALRALAAAGFNITAIRDVTPIAHNGVRPPKRRRVMRS-RRGLLLILSSPSGAGKSTLANRLRNWDPDIVFSVSATTRAPRPGELDGREYYFRSRESFLAMVEDGDMLEHAEVFGNLYGSPQGPVEEAIVEGRDVLFDIDWQGGQQIRNSALAEDVVSIFILPPSIAELERRLRSRDQDSDEVIAARMQKSRDEISHWAEYDYVLVNRDLDETEMKLRAILQAERLRRSRQVGLVDLVRELNKEFGDR-MAQSYLGQKRLRKYYGKIREVLEMPNLIEVQKSSYDLFLKSGDQTEPMDGEGIKGVFQSVFPIKDFNETAVLEFVKYELEKPKYDVEECQQRDMTYSAPLKVTLRLIVFDVDEDTGAKSVKDIKEQDVFMGDMPLMTPNGTFVVNGTERVIVSQMHRSPGVFFDHDKGKTHSSGKLLFACRIIPYRGSWLDFEFDAKDIVFARIDRRRKLPVTTLLYALGLDQEGIMDAYYDTVTYRLEKNKGWATKFFPERIRGTRPTYDIVDAGSGEVIAEAGKKVTPRAVKQLIDKGDVTEILVPYDQIVGRFVAKDIINEDTGAIYVEAGDELTQEFNKEGELTGGSLKDLTDAGITEIPVLDIDNINVGPYIRNTMVVDKNMGRDTALMDIYRVMRPGEPPTVEAASQLFDTLFFDSERYDLSAVGRVKMNMRLALDAPDTMRTLRREDIIACIKALVELRDGKGDIDDIDHLGNRRVRSVGELMENQYRVGLLRMERAIKERMSSVEIDTVMPQDLINAKPAAAAVREFFGSSQLSQFMDQTNPLSEVTHKRRLSALGPGGLTRERAGFEVRDVHPTHYGRMCPIETPEGPNIGLINSLATFARVNKYGFIETPYRKVENGKVTDDVSYMSATEEMRHTVAQANAKLDEQGRFVNDLVSTRQAGEYMLQPNENVDLIDVSPKQLVSVAASLIPFLENDDANRALMGSNMQRQAVPLLQAEAPLVGTGIEEIVARDSGAAIMARRGGIIDQVDSTRIVVRATQDLEPGDPGVDIYRLRKFQRSNQNTCINQRPLVKVGDTVGKNEVIADGPSTDLGELALGKNVVVAFMPWNGYNYEDSILISERIVRDDVFTSIHIEEFEVAARDTKLGPEEITRDIPNVGEEALRNLDEAGIVYIGADVGPGDILVGKITPKGESPMTPEEKLLRAIFGEKASDVRDTSLRLPPGDYGTIVEVRVFNRHGVDKDERALQIEREEVERLARDRDDELAILDRNIYARLKTQILGKTAVKGPKGVKPNSEITEELLETLSRGLWWQLALKEEADASIVEALNEQYQAQKLSLDARFEDKVEKVRRGDDLPPGVMKMVKVFVAVKRKLQPGDKMAGRHGNKGVISKVVPMEDMPFLGDGTPVDFVLNPLGVPSRMNVGQILETHMGWAARGLGEQIGEALGEYRRSGDMTPVRDAMKIAYGDDVYDEGIADMDETRLVEAADNVVRGVPIATPVFDGAKEADVNDALQRAGFDMSGQSVLFDGRTGEQFARQVTVGVKYLLKLHHLVDDKIHARSTGPYSLVTQQPLGGKAQFGGQRFGEMEVWALEAYGAAYTLQEMLTVKSDDVAGRTKVYESIVKGEDNFEAGVPESFNVLVKEVRGLGLNMELLDAEGDDMPLYEHVFISRQDLSNTQAEGLVEHFGTVLSDNGGKVIESEYWGVKTMSYKINKNRKGHYAFLRTDAPAPAVQEMERLMRLHDDVMRVLTIKVDEHAEGPSVQMQKRDE----RERRGAADTPVEALDEKTARAELARLAARIAEADRAYHTDDAPVIDDATYDALRRRNEAIEARFPALKRPDSPSEKVGGTIAEGFSKVEHEQRMLSLSNAFEDEEITEFVERVRKYLGLARDAELAFTAEPKIDGLSLSLRYEKGVLVTAATRGDGSVGENVTANARTIQGLPQKLSGAPDLLEVRGEVYMRHDDFAELNRTQLAQGKKPFANPRNAAAGSLRQLDAEITRARPLAFFAYGWGSLSEPLADTQMGAIERLAALGFETNPLTRRLSTPEDLLAHYHRIEEDRATLGYDIDGVVYKVDDLELQRRLGFRSTTPRWALAHKFPAELAWTRLEGIDIQVGRTGALSPVARLRPVTVGGVVVANATLHNEDYIAGRDSRGEEIREGKDIRIGDRVQVYRAGDVIPKIADVDLAARPDGAEPYVFPDTCPECGSEAIREEGDAVRRCTGGLICPAQAVERLRHFVSRAAFDIEGLGARQVEALYRDEWIAEPADIFRLRARYGSGPRQLKNREGWGEKSAANLLNAIDERRRIPLNRLIFALGIRHVGENAANLLARHYGTWEAFAQAMDAAAEGDAPAWQALNDIDGVGEVMATSLVTTLQQDRERASIERLVAELEVEAVAAPDPGDSVVAGKTVVFTGKLERMTRAEAKSRAEALGAKVAGSVSARTDYLVAGPGAGSKATKAAELGVEVLDEDAWLTLTGGLMIHKNWQELIKPTQLDVKPGNDPSREATVVAEPLERGFGLTMGNALRRVLMSSLQGAAITSVQIDNVLHEFSSISGVREDVTDVVLNLKGVAVRMEVEGPKRLSVNAKGPGVVTAGDIAETAGIEILNKDHVICHLDEGADLFMELTVNTGKGYVSADKNRPEDAPIGLIPIDAIYSPVKKVSYDVQPTREGQVLDYDKLTLKLVTDGSITPDDAVAYAARILQDQLSVFVNFDEPEAARSQDDEDDLEFNPLLLKKVDELELSVRSANCLKNDNIVYIGDLIQKTEAEMLRTPNFGRKSLNEIKEVLSGMGLHLGMDIVDWPPDNIEELAKKYEDHLMAREDNRRGNRRE-REETPEFADRLVAINRVSKTVKGGKRFGFAALVVVGDQRGRVGFGKGKAKEVPEAIRKATEQAKRQMVRVPLREGRTLHHDIEGRHGAGKVVMRTAPQGTGIIAGGPMRAVFEMLGVQDVVAKSIGSQNPYNMIRATLNGLGREASPRSVAQRRGKKVADILKK------PEAEAASESMNDPIGDMLTRIRNAQLRGKSTVETPASKLRAWVLDVLADEGYIRGYEKTTGKDGHPALSISLKYYEGTPVIREIKRVSKPGRRVYMGVKDIPSVRQGLGVSIVSTPRGVMSDANARTANVGGEVLCTVFMHDIRAIRENPDAFDAALSRLGLTNPSAEILQIDAARREAIAQAEEAQAARNAASKEVGAAKASGNEDEFNRLRALVAEKKQQIADLEEKAREGDAKLRDLLMRLPNLPLDEVPDGTDEEDNVELHRRGTPPAFDFKPLEHYQIPAAVPGLDFESAARLSGSRFVVLRGAMARVHRALAQFMLDVHATENGLEETWTPVLVREEMMYGTGQLPKFGEDSYQTTNGWWLVPTAEVTLTNTVNGQTVDESALPLRLCAHTQCFRSEAGSAGRDTSGMLRQHQFEKVEMVSITHPDTSRDELDRMTRCAEDILDRLGLAYRTVVLCTGDMGFGARRTHDIEVWLPGQDTYREISSISLVGDFQARRMNARFRPEGGGKPEFLHTLNGSGLAVGRTLIAVLENGQQEDGSVLLPEVLHPYLRGATRVTPEGALTMAITAAQVKELRDTTGAGMMDAKKALTENNGDMEAAVDWLRTKGLAKAAKKSGRTAAEGLVAVAIEGGEAVALEVNAETDFVAKNADFQAMVADFSKAALKVGSVDELKASDINGKKVEDILTDKIATVGENMAIRRMAKISGETVTAYVHNQAAENMGKIGVLIAMNGTDNGIGRQIAMHVAAANPASLGEADLDQALVEREKSVLTEQARESGKPEQVIEKMIEGRMKKFLSEVTLLGQAFVINPDQTVAEAAKEAGVEITGFVRLEVGEGIEKEAENFAEEVAKMNA-MNQELTNNPFNPLTPPKQFDEIKVSLASPERILSWSFGEIKKPETINYRTFKPERDGLFCARIFGPIKDYECLCGKYKRMKYRGVVCEKCGVEVTLQKVRRERMGHIELASPVAHIWFLKSLPSRIGLMLDMTLRDLERILYFENYVVIEPGLTDLTYGQLMTEEEFLDAQDAYGTDAFTAGIGAEAIREMLAAIDLESEAEQLRADLAEATGELKPKKIIKRLKIVENFIESGNRPEWMVLTVIPVIPPELRPLVPLDGGRFATSDLNDLYRRVINRNNRLKRLIELRAPDIIVRNEKRMLQESVDALFDNGRRGRVITGANKRPLKSLSDMLKGKQGRFRQNLLGKRVDFSGRSVIVTGPELKLHQCGLPKKMALELFKPFIYSRLEAKGLSSTVKQAKKLVEKERPEVWDILDEVIREHPVMLNRAPTLHRLGIQAFEPILIEGKAIQLHPLVCSAFNADFDGDQMAVHVPLSLEAQLEARVLMMSTNNVLSPANGAPIIVPSQDMILGLYYITLEREGMKGEGMVFADVDEVQHALDAGEVHLHSKITARLKQIDDEGNEVMRRFETTPGRVRLGALLPMNAKAPFDLVNRLLRKKEVQQVIDTVYRYCGQKESVIFCDQIMTTGFREAFRAGISFGKDDMTIPEAKWKIVDDVRGQVKEFEQQYMDGLITQGEKYNKVVDAWSKCSDEVAAAMMSEISAVRKDDAGAEMEPNSVYMMSHSGARGSPAQMKQLGGMRGLMAKPSGEIIETPIISNFKEGLTVLEYFNSTHGARKGLADTALKTANSGYLTRRLVDVAQDCIVRQHDCGTDLAVTAEPAVNDGEVVSSMAERILGRVAAENVLMPGTDEVLLSKGELIDERKADAVEQAGVLRMRIRSPLTCEAEEGVCAMCYGRDLARGTIVNEGEAVGIIAAQSIGEPGTQLTMRTFHIGGIAQGGQQSFLEASQDGKIEYRNAVVLKNDAGETIVMGRNMILAIVDGDGAERASHKLGYGTKIFVEDGAKVSRGDKLFEWDPYTLPIIAEKAGKAKFVDLVSGIAVRDETDDATGMTQKIVMDWRAAPKGNELKPEILIVGEDGEPVRNDNGNPVTYPMSVDAVLSVEEGQEVRAGDVVARIPREGAKTKDITGGLPRVAELFEARRPKDHAIIAEIDGYVRFGRDYKNKRRITIEPADESMEPVEYMVPKGKHIPVAEGDFVNKGDYIMDGNPAPHDILSIMGVEALAEYMINEVQDVYRLQGVKINDKHIEVIVRQMLQKWEISDSGETTLLKGEHVDKAEFDAANDKAIARGGRPAQGEPILLGITKASLQTRSFISAASFQETTRVLTEASVQGKRDKLVGLKENVIVGRLIPAGTGGATKRVRQIATERDRKVIEQRQAEAEAALALNAPDEAAVE-------DGFGMAPESRDMSRRHAAEKREVLPDAKYSDRVLTKFMNNLMIDGKKSVAESIVYNAMERVEERLKRAPIEVFHEALDNVKPSVEVRSRRVGGATYQVPVEVRPERREALAIRWLIKAARARNENTMEERLAGELSDAVNSRGTAVKKREDTHKMADANKAFSHYRWMPHAHTDRS---PVMHAPAPDVRNRKKLEGGRRFKLETEFSPAGDQPTAIAELSAGVMAGERDQVLLGATGTGKTFTMAKIIEETQRPAIILAPNKTLAAQLYGEFKGFFPDNAVEYFVSYYDYYQPEAYVPRSDTYIEKESQINEQIDRMRHSATRALLERDDVIIVASVSCIYGIGSVETYGAMTQDLIAGQEYDQRAIIADLVAQQYRRNDQAFQRGTFRVRGDSLEIFPAHLDSRAWRLSFFGNELESITEFDPLTGEKTDTFQQIRVYANSHYVTPKPTMKQAVNSIKKELRQRLDQLVADGKLLEAQRLEQRTNFDIEMLEATGVCNGIENYSRYLTGRAPGEPPPTLFEFIPDNAIVFADESHVSVPQIGGMYKGDYRRKFTLAEHGFRLPSCMDNRPLKFEEWDAMRPQSVFVSATPAGWELEQAGGVFTEQVIRPTGLLDPEIEIRPVGTQVDDLLDEVRRVTAAGYRTLVTTLTKRMAEDLTEYMHEQGIKVRYMHSDIDTLERIEILRDLRLGAFDVLIGINLLREGLDIPECGLVAILDADKEGFLRSETSLIQTIGRAARNVDARAILYADRITGSMERAMRETERRREKQIAYNTEHGITPATVKKNVEDILAGLYQGDVDMNRVTAKIDAPMAGANLQAHLDGLRDKMRKAAENLEFEEAARLRDEVKRLETVDLVVSDDPLARQQAVDRAVDAAQKASGRSTAGRGGMRGGVKRRKG-MAAKPFFRRRKVCPFSGDNAPKIDYKDTRLLQRYISERGKIVPSRITAVSAKKQRELARAIKRARFLALLPYAVKMPKRILNGTVTSDQNEQTVTVLVERRYTHPLLNKTVRASKKYRAHDPKNEFKVGDKVRIQECAPISKTKRWEVVAN--MANSPQAKKRARQNERRQNVNKARRSRIRTFLRKVEEAITSGDADAAKTALQQAQPELMRGVTKGVMHKSTASRKMSRLNSRVKALAMSRSVWKGPFVDSYVLKKAEKTKESGRNEVIKIWSRRSTILPQFVGLTFGVYNGQKHIPVNVTEDMIGQKFGEYSPTRTYYGHAADKKSKRKMARIAGVNIPTGKRVPIALTYITGIGNTSARAICDAVGIEPTRRVNELSDAEVLQIREHIDANFTVEGDLRREVQMNVKRLMDLGAYRGLRHRRNLPVRGQRTHTNARTRKGPAKPIAGKKKMSMKIRLARGGSKKRPHYSIVAADSRMARDGRFKEKLGTYNPLLPKDSEERVKMDVERVQYWLDQGAQPTDRVSRFLEAAGLKDKTERNNPNKAKPGKKATERAEEKAAKAAEAAEATAADAEEAAAE-MSITVEDKQRLMKEFATKEGDTGSPEVQVAILSSRIATLTEHFKTHKKDNHGRRGLLKMVAQRRKLLDYTRAKDEARYQDLIKRLGLRRMPSLNEIRSTFLNYFERQGHQVVPSSPLVPRNDPTLMFANSGMVQFKNLFTGVEKRDYTRATTAQKCVRAGGKHNDLDNVGYTARHHTFFEMLGNFSFGDYFKNEAIPFAWEMITKELDIPKDRLVVTVYHDDDEAAEIWKKVAGISDNRIIRIATDDNFWMMGPTGPCGPSSEIFFDHGDHIWGGPPGSPEEDGDRFVEIWNLVFMQYEQFEDGTREPLPNQSIDTGMGIERVAALLQGTNDNYATDLMRSLIEASAHATSTDPDGPGKTHHRVIADHLRSTSFLIADGVMPSNDGRGYVLRRIMRRAMRHAHLLGSQDPVMHRLVPALVQQMGAAYPELGQAQALIQETLRAEETRFKQTLERGLRLLDDELSDLPEDAPLPGEAAFKLYDTFGFPLDLTQDALREKGRSVDTDGFDAAMAEQKAKARAAWSGSGETADASLWFDLAEKHGTTEFLGYDTEVAEGQLLAIVRDGAAVKTAEAGQEVQFVLNQTPFYAESGGQVGDQGEIRTETGAARITDTRKTAGVFIHMGQVTEGTIETGQGAELEVDHSRRSAIRANHSATHLLHEALRRTLGDHVAQRGSLNAHDRLRFDFSHGKALSREELDQVETEVNEFIRRNEKVETRIMTPDDARAIGAQALFGEKYGDEVRVVSMGSLSGSGKGASGDTYSLELCGGTHVRQTGDIGMFVLLGDSASSSGVRRIEALSGADAFRYLADQGKHLSDAALALKARPDEVPERVRALLDERKALTNELAQLRRELAMGGGGGAAQPEAQEVNGIRFAAHVVNGVTGKDLPALVDEHKARLDSGAVLLIADTGGKAAVAAGVTKDLTDRLSAVDLVRAAVAELGGKGGGGRPDMAQGGGRDTANSDAAIAAARSHLETMGFKTGIVGLPNVGKSTLFNALTRTAAAQAANFPFCTIEPNVGEVAVPDARLDKLAAIASSKQIIPTRMTFVDIAGLVKGASKGEGLGNQFLANIRECDAIAHVLRCFENDDITHVDGRVDPVEDAETIETELMLADMESIEKRLQNLQRKLKGNDKDAAQQDRLLRRALAALEEGRPARTVEIDAEDEKAWAMLQLLTSKPVLYVCNVDEASAATGNDQTRRVAEMAEAQGAAHVVISAAIEEEISQLDPEEAREFLEELGLEEAGLDRLIRAGYELLDLQTYFTVGPKEARAWTVPAGSTAPKAAGVIHGDFERGFIRAETIAYDDYIANNGEQGAKDAGKLRVEGKSYIVKDGDVLHFLFNAMGWKRLEEMDLRGKRVLTRVDINVPVSDGRVSDATRIERIVPTVNAILEAGGTPILLAHFGRPKGRPDPALSLGVVLPALERALGRGVFFVEAPIGAREEIASIRAKDVILLENVRFYPGETANEDGFCDALAALGDVYCNDAFSAAHRAHASTEGLARRLPACAGRLMEAELSALEAALGKPARPVAAVVGGAKVSTKLDLLSNLVTRVDHLIIGGGMANTFLHAQGVAIGTSLAEKDLADTARRILDEAERAGCTIHLPVDLVVAREFRAEAPHEIVAR---DECPPDAMILDAGPESVRAIVELLGRCKTLVWNGPLGAFEIPPFDTATNGAAREAAVLTAAGQLVSVAGGGDTVAALNQAGAADGFSYVSTAGGAFLEWMEGKTLPGVAALGGMALPDFTMRQLLEAGVHFGHQTQRWNPRMGEFIYGDKNGIHILDLTQTVPMLDQALQVVRDTVAKGGRILFVGTKRQAQKPVADAAERCAQYYMNHRWLGGTLTNWKTVSNSINRLKAIDEQMQNGVEGLTKKERLGMEREQVKLEASLGGIREMGGVPDLLFVIDVNKEDLAIAEAKKLGIPVVAVVDTNASPDGVDYIIPGNDDAARAIALYCDLVSRAALDGMSAQMGAAGFDLGAMEEAPEEEAVAEEGNASSETVADDAVAKDAENMTKRTSAKYKLDRRMGENIWGRSKSPVVRREYGPGQHGQRRKGKLSDFGIQLRAKQKLKGYYGDLTEKQFRRIYAEAERVKGDTGENLIGLLERRLDAIVYRAKFVPTVFAARQFVNHGHVRVNGKKVNIPSYRVKEGDVIEVRDRSKQMAVLLEAVQLAERDVPDYLEVDHSKMTATFVRTPALGDVPYPVMMEPNLVVEFYAKNMGQKVNPIGMRLQVNRTWDSRWYADTKDYGDLLLEDIKIREFIKEECKQAGISKVIIERPHRKCRVTIHTARPGVIIGKKGADIETLRKKLASMTASELHLNILEVRKPELDAALVAESIAQQLERRVSFRRAMKRSVQNAMRMGSLGIRVNVAGRLGGAEIARTEWYREGRVPLHTLRADIDYALAEAKTPYGIIGIKVWIFKGEIMEHDPSARDRKQQELQEGPAPRGP--RRMGRRRKGRDISGWVVIDKPAGISSTAVVNKLRWAFDAKKAGHAGTLDPEATGVLAVALGEATKTVPYVTDALKAYRFTVRLGQTTNTDDAEGEVIAESDLRPSDAEIDAALPAFRGHIMQVPPQFSAVKVDGERAYALARGGEELELAARPLWVEELSLVERPDADHVTLEMVCGKGGYVRSIARDLGQALGCGGHVRELRRTWSGPFDAEDGLSMDQVEELARTPALDDHLLPLELGLADLPELPTTAEGATRLRNGNPGMVLTSDAEYGDEAWASFEGRAIAVGTYRAGELHPSRVFVG--PHLIGLTGSIGMGKSTTAGLFAAEGVPVWDADAAVHRLYGPGGAAVAPLARICPDAIVEDAVDRQRLKDWIARDDAALPRIEAIVHPLVGADRAAFLDEAAADIVVLDIPLIFETGAADRFDTLVVVSAPAEVQRARVLARGTMTEAEFENILARQVPDAEKRRRADHVIPTTTLEAAAAAVRQILEQIRGN-RDAMTPVDALIEDDRWCALGLEDLAARAAGAVLTHLDLDPEEFEISLLGCDDARIAVLNEEFRGKPAPTNVLSWPAEDLAPESPGAIPPPPEPMFDG--ALGDIAIAYDTCLREAEEQGKPLENHLLHLLAHATLHLLGYDHETDEDAAVMERLEREILVSLGVPDPYSGQTAMARFVFITGGVVSSLGKGLASAALGSLLQARGFSVRLRKLDPYLNVDPGTMSPFEHGEVFVTDDGAETDLDLGHYERFTGVAARKTDSVSSGRIYSNVLEKERRGDYLGKTIQVIPHVTNEIKDFISIGEDEVDFMLCEIGGTVGDIEGLPFFEAIRQFAQDKPRGQCIFMHLTLLPWIGASGELKTKPTQHSVKELRSIGIAPDILVCRSDKPIPEKEREKIALFCNVRKEDVIAAPDLRSIYEAPLAYHREGLDQAVLDAFGIHPAPAPNLSIWEDVADRVFNPEGEVRVAIVGKYTQLEDAYKSIAEALTHGGMANRVRVKIEWIDAEIFEREDPAPHLERFHAILVPGGFGERGTEGKIKAVEFARTRKVPYLGICLGMQMAVIEAARNVAGMTTAGSEEFDHEAGEKRFEPVIFHLKEWIKDNETIARTVLDDKGGTMRLGEYDATLTEGSNVASVYGTRHIKERHRHRYEVDIKYREALEKVGLCFSGLSPDGRLPEIVEWKDHPWFIGVQFHPELKSKPFAPHPLFRDFIRAAKDTSRLV-MDKLTVLGLESSCDDTAAAVVELTRAADGQVLASVVHGQTDLHAAFGGVVPELAARAHAEKLDLAVEQALSQAEVPLSAVDAIAVTSGPGLIGGVMSGVMCARGLAAGAGLPLIGVNHLAGHALTPRMTDGLEFPYLMLLVSGGHCQFLLVRGVDSFTRLGGTIDDAPGEAFDKCARLLGLEQPGGPAVEREARAGDPTAHRLPRPLLDRPGCDMSFSGLKTALLRARDGIVAQKGGLSVQDRADLCAAFQAAASDVLIEKTRRALDVAAGACGGVPALAVAGGVAANTALRKGLQSLADARGIALVAPPLRYCTDNAAMIAWAGAERLAADLVEPQDLVPRPRWPLDRTSPSLLGSGKKGAKA------------------------------------------------------------------------------------------------------------------------------------

>'Su-undariaeDSM-102234'

MIAKELHDKTPDQLRDELVNLKKEAFNLRFQQATGQLENPARLRTVKRDVARVKTVLNQKAATAATDAMATADLLNMD-KKSADKQKALDSALAQIERQFGKGSIMKLGTAGAIQDITACSTGSLGLDIALGIGGLPMGRIIEIYGPESSGKTTLTLHCVAEQQKMGGVCAFVDAEHALDPQYAKKLGVDIDELLISQPDTGEQALEIVDTLVRSGAVNMVVVDSVAALTPKSELEGEMGDSSVGVQARLMSKAMRKLTSSISRSNCMVIFINQIRMKIGVMFGSPETTTGGNALKFYSSVRLDIRRIGSLKDRDEVVGNQTRVKVVKNKVAAPFKQVEFDIMYGEGISKMGELLDMGVKAGIVDKSGSWFSCGDERIGQGRENAKTYLREHPEMAMDIEDKIRASHGLDFNGSENLDPDILDDMTRYTPSEIEARWQAAWEQNEIFKAVRSADKPKYYVLEMFPYPSGRIHMGHVRNYTLGDVIARYKLAKGFNVLHPMGWDSFGLAAENAAMQKGIHPGEWTFQNIEDMKNQMKPLGFSLDWSREIATCHPDYYQHQQAMFIDMIEAGLIYRKNAVVNWDPVDMTVLANEQVEQGRGWRSGALVERRELTQWFFKISDYSDELLGALDTLENWPAKVRLMQENWIGKSRGLQFSFSTVNAPGGHDQIEVYTTRPDTLLGASFVGISPDHPIAKLLERDSADVAAFVAECRKGGTTEEAIETGEKLGMDTGIRVRHPFDTSKELPVYIANFILMEYGTGAIFGCPAHDQRDFEFATKYDLPIISTYLPSEDASEELTEAYVPQKTEKVFYNRGFSGEPWQTGLEAIDAAIAFCESQGVGQGVTKFRLRDWGLSRQRYWGCPIPVVHCDDCGVVPEKKENLPVKLPEDVTFDIPGNPLDRHTEWRTTPCPSCGKAAQRETDTMDTFVDSSWYYARFTAPHADTPTNMDDAEYWMNVDQYIGGVEHAILHLLYARFFARAMQITGHLPQKAIEPFNALFTQGMVTHAIYKTIGADDRPVFHYPEEVKGDQAFEGGAEVEIIPSAKMSKSKNNVVDPLNIISAFGADTARWFVLSDSPPERDVEWTASGAEAAYKHLGRVWNICDKISQMEDSDAGDDDKELLKQMHKATHDVTMAIESFGFNAAIAKLYGFTATLQKSKASKAAQREAVMTLAQLMSPMTPHLAEDIWANQGGEGLIATAPWPVADEAMLKDDTVTLPIQVNGKRRGEIDVPADMPKDEVEKLALAHEAVIRILDGGTPKKVIVVPGRIVNVVVMAITSANQLELLQTAEAVAREKMIDPALVIDAMEESLARAAKSRYGAEMDIRVAIDRKTGRATFTRVRTVVADDELENYQAEFTVEQAKQYMADPEIGQTYVEEVPPVEMGRIAAQSAKQVILQKVREAERDRQYEEFKDRAGTIVNGLVKREEYGNVIVDVGAGEAILRRNEKIGRESYRPNDRIRVYIKDVRREQRGPQIFLSRTDPQFMAELFKMEVPEIYDGIIEIKAVARDPGSRAKIAVISYDNSIDPVGACVGMRGSRVQAVVNELQGEKIDIIPWNDDQPTFLVNALQPAEVSKVVLDEEAGKIEVVVPAEQLSLAIGRRGQNVRLASQLTGLDIDIMTEEQDSARRQAEFELRTKLFMDNLDLDEFFAQLLVSEGFTNLEEVAYVEVDELLVIDGVDEATAGELQARARDVLEAQSKAALDAARALGVEDSLFEFEGLTPQMIEALAKDDVKTLEDFATCADWELAGGWTTVNGERVKDEGTLEAFEMSLEDAQKLIMTARVLLGWVDPAELEAD-VEEDIDA----DEEAEAMAHKKAGGSSRNGRDSAGRRLGVKKYGGEVVVPGNIIVRQRGTTFWPGQNVGMGKDHTIFATVDGNVQFHKGLKNRTFISIVPAAEAAEMAKRWYSVSVLSNFEKKIAEQIRTTVAELELEDQIDEVLVPTEEVIEIRRGKKVTTERRFMPGYVLVHMEMSDRGYHLINSINRVTGFLGPQGRPMPMRDAEVTAILGRVQEGEEAPRTLIHFEIGERVKVADGPFEDFDGMVEQVDEENQKLKVMVSIFGRETPVELDFTQVNKQVMSFSLAIVGRPNVGKSTLFNRLVGKRLALVDDQPGVTRDLREGAARLADLRFTVIDTAGLEDVTDDSLQGRMRRLTERAVDMADVCLFMIDARTGLTPTDLVFADILRKRSANVILAANKAEGSAADAGVIEAYNLGLGEPIRLSAEHGEGLNDLYTQLMPLADAFAEKARDDAPEVDVDVSDEDEDEDAMPVPTRAKPLQVAVVGRPNAGKSTLINQIVKEDRLLTGPEAGITRDAISLMTEW--NVPMRIFDTAGMRKKAKVQEKLEKLSVSDGLRAVKFAEVVVVLLDAEIPFEQQDLRIADLAEREGRAVVIAVNKWDVEENRQEKLKELKESFERLLPQLRGAPLITVSAKTGRGLDRLHQAIMRAYEMWNRRVTTAQLNRWLSGMMEAHPPPAPQGKRIKMKYMTQAKTRPPGFVVMCSHPDKLPESYSRYLVNGLRVDFDMPGTPIRLWMRGQSDANPFKGRKKAPPSKLRKHTASKRRDMRIVFMGTPEFSVTVLDALVEAGHEIAAVYSQPPRPAGRGKKDRPTPVHARAEALGLEVRTPVSLKTPEALAEFSALTADVAVVVAYGLILPQAILDAPAQGCLNIHASLLPRWRGAAPIHRAIMAGDAQTGVCIMQMEAGLDTGPVLLCENLDIGAEETTAQLHDRLSILGAAAIVEALASLDDLTPQVQPEEGVTYAAKIDKAEARIDWHLPAGQVDRMIRGLSPFPGAWFEHGGVRVKVLGSRLVTGAGAAGIVLDEALHVACGEGAVALTRLQKAGKGAQDVEVFQRGMQIAVGANLNEGMTDTAAYRVLARKYRPETFADLVGQEAMVRTLKNAFAADRIAQAFIMTGIRGTGKTTTARIIAKGMNCIGPDGTSGPTTEPCGVCEHCTAIMEGRHVDVIEMDAASNTGVANIREIIDSVHYRAASARYKVYIIDEVHMLSTGAFNALLKTLEEPPEHVKFIFATTEIRKVPVTVLSRCQRFDLRRIEPEVMIALLRKIATAEAADIADDALALITRAAEGSARDATSLLDQAISHGAGETTAMQVRAMLGLADRARVLDLLDMILRGDAASALTEIGAQYAEGADPMAVLRDLAEITHWVSVVKITPDAAEDPTISPEERDRGRVMADALAIRVLTRLWQMLLKALEEVASAPNAMMAAEMAIIRLTHVADLPSPEELLRTLQN-TPAP--PAGGNGPS------SGQAVQHSAPQMTSTPNASGQATALAVDPASALAAFPTFEHVLELIRHNRDVKLLVEVETSLQLAAYQPGRVEFVPTDTAPRDLAQRLGAKLQLWTGNRWAVTVVNTGGAPTIASLRDAKDNAMRAEAEAHPLMMAVLAQFPRAKITAIRTAADIAAAAVSEALPEVEDEWDPFEDSMPKMKTKSSAKKRFKISATGKVIGGQAGKQHGMIKRSNKFIRNARGTTALSAPDAKIIKGFMPYDRMNLFADIRTLVLSSVDAMVASGDLPAGLTTDNITAEPPRDAAHGDMATNAAMVLAKPAGMKPRDIAEKLAVILVQDPRVTSADVAGPGFLNLRLADGVWQQVASTVLDAVTDYGRGDLGTGKTVNVEYVSANPTGPLHVGHTRGAVFGDALASLLDFAGWDVTREYVINDGGGQIGVLARSVYLRYLEAHGQEVAFPDGTYPGDYLIPVGQKLKDKVGDQYIDQPEDVWLGPIGDFATDEMMDLIRDDLAQLGVRMDRFFSEKSLYNTGKIEACLKKLDDMGLIYRGTLEPPKGKLPDDYEAREQTLFKSTDFGDDQDRAIQKHDGAWTYFAPDIAYHNDKVERGYDQLINVFGADHGGYVKRMKAAVHALSDGKVPLDIKLTQLVKLFKNGEEFKMSKRAGNFVLLSDLIKEVGKDVTRFVMLTRKNDAPLDFDFNKVMEQSRENPVFYVQYAHARVASVMRKATEAGIDVSDAALKAADLSKLDHTAELALLRKVAEWPRLVETAARSNEPHRIAFYLYELAGDLHGFWNLGNAETGLRFIQEDDPATSQAKIALARSVAIVIAAGLGILGVTPAEEMRMSTIDNLPSLRQVIDDHGLQARKSLGQNFLLDLNLTAKIARQAGDLTQCDVLEIGPGPGGLTRGLLAEGARRVLAIEKDSRCMPALAEIAAAYPDRLQVIDGDALEVNPLAHLTPPIRVAANLPYNIGTELLVRWLTPPEWPPFWQSLTLMFQREVAERIVAAPGSKAYGRLALLAQWRADAKIVINLPPEAFSPPPKVSSAVVHLTALPEPRFPADAAILSRTVAAAFNQRRKMLRAALKGTTPDIEDRLIAAGLKPTDRAEQISLEGFCALAREIAKSMNLIAEIEAEHIAELAKEIPDFRAGDTIRVGFKVTEGTRTRVQNYEGVCISRKHGKGIAGAFTVRKISFGEGVERVFPLHSTNIDSITVVRRGRVRRAKLYYLRERRGKSARIIENTHYKPLKG---MANSKRQLFIKRRLRVRNKLRRTNRGRMRLSVHRSNKNISVQLIDDVNGVTVASASSLEKDLGVVGKNNIEAATKVGAAIAERAKKAGVEVAYFDRGGFLFHGKVKALADAAREGGLKIMFAVLKTGGKQYKVQAGDMLRVERIAASAGETVQFNEVLMLGGDNPTVGAPMIEDAGVQAEVVDQIKGEKVIHFVKRRRKHSSKRTKGHRQKLTLVKITEILASGAGKSGVAAAIGTGSVSAAAVAA----KAAKPAKAEA---PKAE--KAEAKAKKAAKAE-G-DDLSEISGVGPVIVGKLNDAGITTFAQIAAWTDADVEEIEEKLSFKGRVGREDWIAQAKVLAKGMSRTKGGTVTHARHRKVVKAAKGYYGRRKSTFKVARQAVDKANQYATRDRKVRKRNFRALWIQRINAAVRAHDAELTYSRFINGLNLAGIEVDRKVLADLAVNEPEAFTAIVKQAQASLAAMQVKETLNEGLKRGYTINITAAELDVKVDEKLKEAQSEVEMKGFRKGKVPMALLKKQFGPKVLGEAMQEAVDGAMNDHFESTGDRPAMQPDVKMTNDDWKEGDDVEVEMSYEKLPAIPDVDLSKISLEKMVVKADEASIDEALASLAETAQDFKARKKGSKAKDGDQIVLDFVGKVDGEAFEGGAAEDYPLVLGSNSFIPGFEEQLVGVKAEEEKDVTVNFPDEYQAEHLAGKEAVFSCTIKEVKEPVAAEINDEMAKKFGAEDLAALKVQIGERLEAEYVGASRAVMKRGLLDALDGLVDFDLPPSLLDAEAGQIAHQLWHEDNPEVEGHDHPEIETTDEHKKLAARRVRLGLLLAELGQKAEVEVTDAEMTQAIMNQARQYPGQERQFFEFVQQNQQMQQQMRAPIFEDKVVDYVFEQATVAEKEVSKDDLQKAVEALEEEMKLHELSDNAGATKPRKRVGRGPGSGTGKMGGRGIKGQKSRSGVAIKGYEGGQMPLYQRLPKRGFTKPNRKTYSALNLGLIQKFVDAGKLDISAVITEDALVACGVLRRKRDGIRILAKGDVTSKLNLDVTGASKSAIEAVEKAGGSLTVKAAAAVEASEMIQMQTNLDVADNSGARRVQCIKVLGGSKRKYASVGDVIVVSVKEAIPRGRVKKGDVRKAVVVRTAKEVRRDDGTAIRFDRNAAVILNNNNEPVGTRIFGPVVRELRGKNFMKIISLAPEVLMRHARGYRRLNRTHEHRKALWANMAGSLIEHEQIKTTLPKAKELRPIIEKMITLAKRGDLHARRQARARLKEDQYVTKLFDILGPRYKDRQGGYVRVLKAGFRYGDMAPMAIIEFVDRDRDAKGAADKARVAAEEAAE-MLQPKRTKFRKQFKGSIKGLAKGGSDLNFGTYGLKAVEPERVTARQIEAARRAMTRHMKRQGRVWIRIFPDVPVTSKPVEVRMGKGKGSVDFWACKVKPGRVMFEIDGVNDDIAREALRLAAMKLPIKTRVIVREDWMTTLKLHNTKTRKREEFVPIDPQNVRMYVCGPTVYDRAHIGNARPVIVFDVLNRLLRHVYGESNVTYVRNFTDIDDKINARAARDGKDISEITAQTTQWFLDDMAAVGAIEPDHMPRATAYVPQMIAMIEDLIAKGHAYAAEGHALFAVDSYKDYGALSGRSTDDMIAGARVEIAPYKRNPMDFVLWKPSDADTPGWDSPWGRGRPGWHIECSAMAHELLGTHFDIHGGGNDLMFPHHENEIAQSCCAG--DDFANVWLHNEMLQVEGKKMSKSLGNFFTARDLLDKGVPGEVIRFVMLSTHYRKPMDWTEKKAQEASRSLKKWRDLTADIEAAPSIPAVVLTTLCDDLNTAGVLTLLHEFASNGDLASLKASAQLLGLLTEELGGWTERGGALLDGWTERLTVAREKAMETKDFVEVDRIKLLLTDAGVKVQMGKEGIVLAAGPEVDLAKLEALKMAQVKSSSKSDPNYKVIAENRRARFDYAIEDDVECGIILEGSEVKSLRMGGSNIAESYAAVEDGELWLVNSYIAPYKQAKTFGHEERRRRKLLVSRKQLVEMWNATQRKGMTLVPLVMYFNHRGKAKIKIGIAKGKQLHDKRADSAKRDWSRQKSRLLKEHSMSAKPEHYDVIRKPIITEKATMASEQNAVVFEVAIDSNKPMIKEAVEALFGVKVKAVNTSITKGKVKRFRGQLGTRKDVKKAYVTLEEGNTIDVSTGLMSKDKNPRRVADNEARAKLRMLKTSPQKLNLVAAMIRGKKVDKALTDLTFSKKRVAIDVKKCLQSAIANGENNHNLDVDELVVAEAYVGKNMTLKRGRPRARGRFGKIMKPFAEITIVVRQVEEQAMVSAVENMAANSSWSAFGKATDLRHRILFTLGLLIVYRLGTFIPVPGIDGAALRQFMDSAGQGIGGMVSMFTGGALGRMGIFALGIMPYISASIVVQLLTSMVPSLEQLKKEGEQGRKKINQYTRWGTVALATVQSYGLAVSLEAGDIASDPGMYFRIACMITLIGGTMFLMWLGEQITARGIGNGISLIIFVGIIAEVPAAIAQFFVSGRSGAISPAVIVAVLAMVVLTIMFVVFMERALRKIHIQYPRRQVGMKVYDGGSSHLPIKVNPAGVIPAIFASSLLLLPITISTFSGDTTSPVMSWLLANFGPGQPLYLLFFIAMIVFFAYFYTFNVSFKPDDVADNLKNQNGFVPGIRPGKKTAEYLEYVTNRILVLGSAYLAAVCILPEILRGQFAVPFYFGGTSVLIIVSVTMDTIQQVQSHLLAHQYEGLLEKSQLRGKSGKGRKKRSPARKMKFLDLAKVYIRSGAGGGGCISFRREKYIEYGGPDGGDGGGGGTVWAVAVDGLNTLIDFRYQQHFFAKNGQPGMGKQRTGKDGDDIILRVPVGTEILDEDQETVLADMTELGQRVELARGGNGGWGNLHFKSATNQAPRRSNPGQDGVERTLWLRLKLIADVGLLGLPNAGKSTFLAATSNARPKIADYPFTTLHPNLGVVGVDNTEFVVADIPGLIEGAHDGRGLGHRFLGHVERCAVLLHLVDGTSETITEDYQTIIGELEAYGGELADRPRVTVLNKIDALDEETLASASAELRKACSGEVMLMSGVAKTNTVEVLRALRAEIDDNRLRQTKGEEEAPWQPMAAKLKKGDTVIVLAGKDKGKEGTIASVDPKTNKAVVDGVNVYLRATRQTQTSQGGRIPKSMPIDLSNLAIKDANGKPSRVGFKMDGENKVRFAKTTGDVI--MLRSGVIAKKMGMTRLFMEDGKQIPVTVLQLDNLQVVAQRTIERDGYVAVQLGAGTAKVKRTSQAMRGHFAAAKVEPKRKVVEFRVDADAMLPVGEEIIADHYFAGQYVDVAGTSIGKGFQGAMKRHNFGGLRATHGVSVSHRSHGSTGQCQDPGKVFKGKKMAGHMGSARVTTQNLEVVKTDTARGLIMVKGAVPGSKGGWVTVKDAVKKPFPDAAIVPGALASAAREAAKAAEEAAAAAAAEAEAAAV----EAAAAEQAAMEAAEN---AEATPDAVAEA-EKKEGDAMALKSYKPTTPGQRGLVLIDRSELWKGRPVKALTEGLHKHGGRNNTGRITMRRKGGGAKRLYRIVDFKRNKLDVTATIMRIEYDPNRTAFIALVKYEDGEQAYILAPQRIAIGDQVVASAKADIKPGNAMPFSGMPIGTIIHNIEMKPGKGGQIARAAGTYAQFVGRDGGYAQIRLSSGELRLVRQECMATVGAVSNPDNSNQNYGKAGRMRHKGVRPSVRGVVMNPIDHPHGGGEGRTSGGRHPVTPWGKPTKGAKTRNKKKASSALIIRSRHAKKKGRMLDTAAYTPRLKADFKNRIRAALKEEFGYTNDMQIPRLDKIVLNIGCGAEAVRDSKKAKSAQEDLTLIAGQKALTTIAKKSIAGFRVREEMPLGAKVTLRGDRMYEFLDRLITVAMPRIRDFRGINGKSFDGNGNYAMGLKEHLVFPEIDFDKIDENWGMDIVIATTAKTDAEAKAMLKLFNMPFNSMKLDVINLEGASAGSIDLDEALFGLEPRADILHRVVRWQRNNAQAGTHKVKTRREVSYSTKKIYRQKGTGGARHGARSAPIFRGGGIYKGPTPRSHGHELTKKFRKLGLRMALSAKAKAGALVIIDDAASNGKTSALAKQVKSLGWKRALIIDGASVNENFLQAARNIEGLDILPTMGANVYDILKRDTLVITKAGIEALEARLKNENVILIIHLILALGLIAVVLLQRSEGGGLGM-GGGGGANSGRPAASPMAKVTWILGLAFVVTSIALTITSAQKSAGVSVLDRLTDSPPALEQSDPAAAQGLGDLLPPTQGDNAPLVPTVDMAFFKKLKDRLFTSSSKIDEGLEAIVSDGGQADVPAV-------ETPAQVVEVTPEAAQEAAQEVPDLDTAVHAALEEKQRQEAQAEAVEEPQA-------------PEPT---VEPLRTTLTPVAPVLEEAPT--EPVAKRGVLGRLMGRGADGEVVRRELDDAMLEQLEELLIASDMGVDTALRVTSNMAEGRLGKKLSVQEIKTLMADEIARIMEPVAKPLPLYAKTPQVVLVVGVNGSGKTTTIGKLASQFRGAGKKVVIAAGDTFRAAAVEQLQVWGERAGVPVLTAPHGSDPASLAFDAMVRAEADGADLLMIDTAGRLQNRGDLMEELAKIVRVIRKKDPEAPHNTLLVLDATTGQNAINQVKVFQDISDVSGLVMTKLDGTAKGGVLVSLADKFGLPIHAIGVGEQIDDLQPFDPQEFADALTGLDRMTRDEWGSVKQRLLKTVGQNNYTTWIDPLLLGALDSGIATVNVPTTFFGNYVSQNFADLILHEMRAFDDSLSRLKFEVAG----KTAKPATRQTDAIAAPRST-PKATTTYTAPLERRFSFDTFVVGKPNELAHAAARRVAEGGPVSFNPLFLYGGVGLGKTHLMHAIAQELQIRKPELNVLYLSAEQFMYRFVQALRDRKMMDFKEIFRTVDVLMVDDVQFIAGKDSTQEEFFHTFNALVDQNKQIIISADRAPQDIKDLEDRVKSRLQCGLVVDLHPTDYELRLGILQTKVDTHRASYPELAMEDGILEFLAHRISTNVRVLEGALTRLFAFASLVGRKIDMELTQDCLADVLRASERKVTVEEIQRKVSDHYNIRLSDMVGPKRLRSYARPRQVAMYLCKHMTSRSLPEIGRRFGGRDHTTVMHGVKRIEELKISDGQIAEDLELLRRALEAMLGLGTLTKKVFGSPNGRKIKAVRPLVEKINALEPEFEKLSDEAIKDKTEELSARALKGESLDALLPEAFANCREGARRTLGLRAFDTQLMGAIFLHQGNVAEQKTGEGKTLTATFAAYLNALTHKGVHIVTVNEYLVKRDAEWMSKVFASLGLTTGYIIPNMPDDLKRHAYECDITYATNNELGFDYLRDNMKAELSEIFQKQHNFAIVDEVDSILIDEARTPLIISGPSDDRSDMYQTIDTIIPTLLPEHYELDEKTRGVTFTDEGIEFLEEQLRARELLEGEATLYDPESTSLVHHVNQGLRAHTLFEKDKDYIVRDGDVVLIDEFTGRMMAGRRLGDGLHQAIEAKEGVKIQPENVTLASVTFQNYFRLYDKLAGMTGTALTEADEFMEIYGLGVVEVPTNVPVARQDADDAVYRTVAEKYAALIEDTKEAHAKGQPCLVGTTSIEKSEMLSKLLEKEGIKHSVLNARQHEQEAQIVGDAGKFGAVTIATNMAGRGTDIQLGGNVELKVLDALAADPEADPVAVRARIEAEHADEKKKVLEAGGLYVLASERHESRRIDNQLRGRSGRQGDPGRSSFYLSLEDDLMRIFGSDRLEKVLTSLGLKEGEAIIHPWVNKSLERAQAKVEGRNFDMRKQLLKFDDVMNEQRKVIFGQRREIMESQDLHEITEDMRHQMVDDLIDQYMPVNTYADQWDTQGFYAAVIEQLGVDVPVIAWCEEDGVDDDIIRDRLIEATDKLMAEKTEKFGPENMRNIEKHFLLQTIDAKWREHLLTLEHLRSVVGFRGYAQRDPLNEYKNESFQLFESMLDSLRSDVTQRLSRIEPPSEEQQQRLMEQMLAQQRAADDAVDDAVDQ---AEAAEA--IDGFDENDPSTWGSPARNDLCPCGSGKKFKHCHGQLTMKLSAELKAFEGRLGYQFEKPKLLNEAVTHASMTTPNRDDNQRLEFLGDRVLGLVMAEALLNLDTGASEGQLAPRFNALVRKEACADVAREIDIGAVLRLGRSEMLSGGRRKQALLGDAMEAVIAAVYVDGGFDAARDMIIRLWGTRTTSVKEDARDAKTSLQEWAQARGLEPPQYVLEKRSGPDHAPIFTIAARLSTGQTASATAGAKRAAEQDAAASLLAQLEQEIMAKLGKRTVAAREAFVGKENLTVEDAVALIKGNANAKFDETIEIAMNLGIDPRHADQMVRGVVGLPNGTGKDVRVAVFARGPKADEAKEAGADIVGAEDLMEIVQGGKIDFDRCIATPDMMPVVGRLGKVLGPRNLMPNPKVGTVTMDVAAAVKAAKGGEVQFKAEKGGVVHAGVGKASFDAAKLVENIRAFVSAVAKARPTGAKGAYMTKIALSSTMGPGVTVSVDNAASEMSEDFMLDTDDLDRRMKGAIASMRTEFASLRTGRGSASMLEPVMVDAYGQMTPINQVGTVNVPEPRMVTVNVWDKALVGKVEKAIRESGLGINPQLNGTIIMLPIPELNEERRAQLSKVAGGYAENARVSIRNVRRDGMDQIKKAKADGMSEDDQKIWEDEVQELTNKFIKAIDEGLETKQAEIMQVMAKKLVGTMKLQVKAGQANPSPPVGPALGQRGINIMEFCKAFNAKTADLEPGAPCPTVISYYQDKSFTMDIKTPPASYFLKKAAKVNSGAKTPSRETVGTITAKQLREIAEAKMADLSANDVEQAMKIIMGSAKSMGIEVKMFENLSERLSGVFDRLTKQGALSEDDVKTALREVRVALLEADVSLPVARDFVNAVQEKATGQAVTKSITPGQQVVKIVHDALIDTLRGEGEPGALKIDSPPAPILMVGLQGSGKTTTTAKLAKRLKEKDGKRVLMASLDVNRPAAMEQLAILGVQIGVDTLPIVKGETPVQIAKRTKTQASLGGYDVYMLDTAGRLSIDEELMQQVEAVRDVVTPRETLLVVDGLTGQDAVQTAQNFNDRIGISGVVLTRMDGDGRGGAALSMRAVTGKPIKYVGLGEKMDSLETFEPERVAGRILGMGDIVALVEKAQETIEAEQAEKMMRRMAKGQFNMNDLKMQLEQMIKMGGMQGMMGMMPGMGKMAKQVEDAGLDDKVLKQQIALINSMTKKERANPALLQASRKKRIAKGAGMQVSDLNKLMKMQRQMSDMMKKMGK-GKGGMMKAAMKQMMGKGGMDPAAMAQGMDPKALEAAAKQMGGKLPG----LGGGMGLPAGLSGFGKKK---MDRLAQITARFEYLEAAMSTA--GGDISKLAKEYSDLRPVVEEISSYRVLLDNLEGAKEMLADPDMKDMAREEISDIEAALPAAEASLQLALLPKDEADARPAMLEIRPGTGGDEAALFAADLLRMYHRYAESRGWKLDIIEEQTTELGGIKEVVAHITGENVFARLKYESGVHRVQRVPSTESGGRIHTSAATVAVLPEAEDVDIQIATTDIRIDTYRSSGAGGQHVNTTDSAVRITHIPTGIVVTSSEKSQHRNREIAMQVLKTRLYDAERQRIDTERSDSRASQVGSGDRSERIRTYNFPQGRMTDHRINLTLYKLDAVMQGDLDEIVDALTADAQAQMLAEMGQMDRAQKEQLVDELGQIFESSGVVVVSHYVGLTVAEMQDLRARATAAGGSVRVAKNRLAKIALEGKPCASIADLLTGMTVLTYSEDPVAAARVAQEFAKENDKLVILGGAMGENALDAAGVEAVSKMPSREELISTIAGMLGAPASNIAGAIGAPASNIASILSTIEDKAA-MKTFSATPADIDKKWIIIDAEGIVLGRLASIIAMRLRGKHKPSFTPHMDCGDNVIVINAEKIQMTGKKREEMFYWHTGHPGGIKERSKQDILDGKHPERIVTLAVKRMLPGNRLSRQIMTNLRVYAGGEHPHEAQEPTVLDVASMNKKNTRSAMQQEDAPLLHAVDVGFGAEPPKRIGIAVSGGGDSVALLHLFARWSAQCNHPIAAVTVDHGLRPESRAEAEGVAALCQKLGVSHDILTWERPEGAGNLPAAARDGRYALMADWAKAHDIGGIAVGHTIDDGAENFIMRLGRAAGIDGLAQMVPLFERYGLCWARPLWQFHRGALRDYLLRQDVSWAEDPSNDDPRYLRTKARRLLPQLKELGVDAQSIQQSASALRMAQSALQHYTVKEAETHVKEVAGDILIPQVIVPHIPSDIERRLLVAAVQWVGSNPYPPRKEFATTLEFTLSQQQRLTVAGCLVALRKGFFHITREYNAVKDLAGPTDAVWDTRWRLHGPH--APDLEVRALGE-AVSELPDWRATGLPRPTLMASPAVWRGKTLVAAPLAKYNVDWTAQIVAD---FTSFLLSHMADLKKLAEDIVGLTLLEAQELKTILKDEYGIEPAAGGAVMMAGPADGAAAAEEKTEFDVVLKNAGASKINVIKEVRGITGLGLKEAKDLVEAGGKIKEGVDKAEADEIKAKLEAAGAEVELAMSRIGKKAVNLPSGVSASVSGQTIEVKGPKGVRTFSATDDVTMTVEENAVSVTPRGKSKRARQQWGMSRTMIANLVTGVTDGFKKELEIQGVGYRAAMTGNTLKLNLGLSHDVDYIPPAGVTVTAPKQTEIIVEGIDEQLVGQVAANIRAWRKPEPYKGKGIRYKGEFVFRKEGKKKMKVILLQRVAKLGQMGDVVDVKPGHARNFLLPQQKALTASKANIEAFEGQKAQLEAQNLETKAEAEAMAERLGGQQFVVIRSASDAGALYGSVTTRDASDAATAEGFTVDRKQVVLIAPIKYLGIHEVKVILHPEVEATIEMNVARSPEEAELQAAGKSIQELAAEEEAAAEFEISELFDDLGSAASDDDDAAPAA-------AADEEA--MSDSDGRKTLGL-GGSRPSNVKQSFSHGRTKNVVVETKRKRVVVPKPGGQKPTGPGAGPVGDPSRRPAGITDSEMERRLKAVQAAKAREVEEAAARAAEEKARAEDRERRRAEMEAKEQEDREREESLKAKAEEEERAKRAAEAAAQAAPAPS-EPAQARATPNKALPAATPRKTERDREETNKKNRQDDD-RRSGKLTVNQALRGGEGGRQRSMAQMKRKQDRARAKAMGGNVEREKIIRDVQLPPAIVVSELAARMAEKTGAVVKALMQSGLMVTQNETIDADTAELIIEEFGHKVVRVSDADVEDVIKIEVDDEGDLRSRPPVITIMGHVDHGKTSLLDAIRNAKVVSGEAGGITQHIGAYQVTTDGGQVLSFLDTPGHAAFTSMRSRGAQVTDIVVLVVAADDAVMPQTIEAIAHAKAAKVPMIVAINKIDKPAADADRVRAALLQHEVIVEKMSGDVQDVEVSAVTGQGLDELLEAIALQSEILELKANPNRAAVGAVIEAQLDVGRGPVATVLVQNGTLRQGDIFVVGEQYGKVRALIDDQGNRVKEAGPSVPVEVLGLNGTPEAGDVLNVTSTEAQAREIAEYRANAAKDKRAAAGAATTLEQLMANAKADENVSELPILVKADVQGSAEAIVQAMEKIGNDEVRVRVLHSGVGAITETDVGLAEASGAPIIGFNVRANASARNTANQKGVEIRYYSIIYDLVDNVKAAASGLLSNEIKETFIGYASIKEVFKVTGIGKVAGCLVTEGVARRSAGVRLLRDNVVIHEGTLKTLKRFKDEVPEVQSGQECGMAFENYEDIRPDDVIEIFTREEVTRTLSMAKPKKTPRPKAETPRGFRDYFGTEVTQRAEMLAKIAGVYHRYGFDALESAGVEKVEALGKFLPDVDRPNEGVFAWQEDAEAEKPGDWLALRYDLTAPLARVYAQHRNELPTPYRRYAMGPVWRNEKPGPGRFRQFYQCDADTVGAGSVVADAEICAMLSDCLEEVGIERGDYIVRVNNRKVLNGVLEVAGLSGDDKENARGIVLRAIDKLDRLGVDGVRALLGAGRKDDSGDFTDGAGLDDAAADVVMGFMDAKRDDGAATCARLRELVGDSVVGLEGVAELETIAELLSAGGYGPDRIEIDPSVVRGLGYYTGPVYEAELTFEIKDEKGRPRNFGSVAGGGRYDDLVKRFTGQEVPATGVSIGVDRLLAALHAKGRMDTQAEGPVVVTVMDKARMADYQAMVAELRQAGIRAEVYLGNPKNFGNQLKYADKRGSPVAIIEGDEEHQNGMIQIKDLVLGAKIAENATLEEWRDRPSQYEVPRADLVTRVREILELYRMDDLKQKYLSQIANASDESGLEDIRLAAVGKKGEVALKMRELGKMTPEERQTAGPALNALKDEINSALAAKKAALGDAALDERLRSEWLDVTLPSRGRPMGTIHPVSQVTEELTAIFAEMGFSVAEGPRIDTDWYNFDALNIPGHHPARAEMDTFYMARAEGDDRPPHVLRTHTSPVQIRTMEAEGAPLRIICPGGVYRADYDQTHTPMFHQVEGLAIDKDISMANLKWTLEEFFSAFFEIDGIKTRFRASHFPFTEPSAEVDIQCSWVDGQLRIGEGDGWMEVLGSGMVHPKVLAAGGIDPEVYQGFAFGMGIDRIAMLKYGIPDLRAFFDSDLRWLRHYGFASLDQPNLHGGLSRMVMAEEAGLDLVEISPNANPPVCKIMDFGKFKYETQKREAEARKKQKIIEIKEIKFRPNTDSGDYEIKMRNVFKFLANGDKVKITLRFRGREMAHQNLGRELLERVAADTKDFGKVENFPKMEGRQMVMLIGPLPAMKFTLSWLKEHLDTTASVDEITYALTDLGLEVEGVEDRGAKLRDFTLGFVKSAEKHPDADRLRVCQVETDEGLQQIICGAPNAREGITVVIAKPGVYVPGIDTTIGVGKIRGIESFGMMASERELELSEEHDGIIELPSGNVGDSFTDWLAENDPAKVDPVIEIAITPNRPDALGVRGIARDLAARGLGKLKQRDVDVVAGAFPCPVSVSIDDDTLEHCPVFFGRVIRGVKNGPSPVWLQDRLRAIGLRPISFLVDVTNFFTFDRNRPLHVFDADKIAGDLRVHRAKGGETLVALDDKEYTLQDSMIVISDANGVESIGGVMGGLATGVTEDTVNVFLEAAYFDPVRTAYTGRALKINSDARYRFERGIDPEWTPYGIEHATQMILDHAGGEASDVVSAGKVPDTSRAYRLNAKRVVSLVGMEIPESTQRQTLTSLGFRLEGDMAHVPSWRPDVQGEADLVEEVARIASLTKLEGVPLPRLTTGVPRPVMSPVQRREVAARRTAAALGYHECVTYSFIDQPSAALFGGGTDATRLENPISNDMSHMRPALLPGLLAAAARNQARGFADMALFEVGPAFSGGEPGEQHMLVSGLLVGRTGPKDVLGAARDVDVFDVKADAEAILAAIGAPAKVQILRGADDCWHPGRHGKVCLGPKKVLAIFGEVHPRILAAMDVKGPAMAFTIWPAEVPLPRKAGATRPALKVSDLQAVERDFAFVVDADVEALTLVNAAMGADKVLIEDVRVFDEFIGGSLGEGKKSLALTVRMQPHEQTLKDADIEAVGAKVVEKVTKATGGVLRGMHAYRNQTCADLNTTNVGDKVRLSGWVHRVRDHGGVLFIDLRDHYGITQLICDGDSPAFAELEKVRAEWCIRVDGDVKARAAELVNEKIPTGGIEIYVRDVEVLGQVNDLPLQVFGEQEYPEETRLRYRYLDLRREKMQRNMILRSDVVSSIRQRMWDRKFKEFQTPIITASSPEGARDFLVPSRLHPGKFYALPQAPQQFKQLLMVSGFDKYFQIAPCFRDEDPRADRSPTDFYQLDLEMSFVEQQDVFDTIQPVLTGIFEEFGGGKAVDQEWPQISYKDAALWYGSDKPDLRNPIKMQVVSEHFAGSGFAIFAKLLEQEGTQIRAIPAPTGGSRKFCDRMNAFAQKEGLPGMGYIFWRDQ-GE--GMEAAGPLAKNIGPERTEAIRQQLGLGVGDAAFFLGGKPKAFESVAGRARNVIGEELGLTDQNRFAFAWIVDFPIYEKDETTGKIDFEHNPFSMPQGGMEALNGDPLEVLGYQYDLACNGYELVSGAIRNHRPEIMFKAFEIAGYGPEEVRKRFGGMVNAFQYGAPPHGGCAAGIDRIVMLLAEESNIREVILFPMNQRAEDLMMNAPNDPMPDQLMELGLRVIPQDMTPLSHIRNFSIVAHIDHGKSTLADRLIQETKTVAVRDMKAQMLDSMDIERERGITIKAQTVRINYQALNGEEYVLNLIDTPGHVDFAYEVSRSMRAVEGSLLVVDSTQGVEAQTLANVYHALDADHEIVPVLNKIDLPATDCDRVAEQIEDVIGIDASQAIRVSAKTGEGIVETLEAVVHRLPAPTGTLDAPLKAMLVDSWYDSYLGVIVLVRIMDGQLKKGDRITMMQNGSIHHVDRIGVFRPAMTEIDVLGPGEIGFLTASIKQVRDTRVGDTITHDKKGCETALPGFKPSQPVVFCGLFPVDAALFEDLRDSIEKLALNDASFSYEMETSAALGFGFRCGFLGLLHLEVIRDRIEREYDIDLITTAPSVIYNIHMKDGTMQQLHNPADMPDLTFVEHLEEPRIKATILVPDEYLGDVLKLCQDRRGIQMDLTYAGSRAMVVYDLPLNEVVFDFYDRLKSVTKGYASFDYQMIGYREDALVKMSILVNDEPVDALSTMVHRDRAEMRGRAMVEKLKDLIPRHMFKIPIQAAIGGKVIARETLSAMRKDVTAKCYGGDASRKRKLLDKQKAGKKKMRQFGSVNIPQEAFISALKMDGMSLPPGFLDELRTRSSLSQVVGRKVIWDARKSNQGKGDMWAPCPFHHEKSASFHVDDRKGFYYCFGCHAKGDAISFVRETENVSFMEAVEILAREAGMPIPQRDPQAQEKAEKRTELVDVMELAVRWFRLQLRTGAASAAREYLEKRGLNQEVCDRWEIGFAPDSWQGLWDALKGKDIADELIIGAGLAKPSSKGGKPYDTFRGRIMYPIRDARGRAIAFGGRAMDPEDKAKYLNSPETELFDKGRSLYNVKEARVAAGKGQPLLVAEGYMDVIALSEAGFGASVAPLGTAITENQLAMLWRISDEPIITLDGDTAGQRAALRLIDLALPLLEAGRSLRFAMMPEGKDPDDLLKSSGAGAVQALLDAAVPMVQLLWQREVEGKVFDSPERKAALDKNLREKIKLIKDPSIRSHYGQAIKDMRWDLFRAK----GSF---APRPWKSQWGKAPQGPSAGAKSSILATAADARATDHLREAVILAACISCPEAVEPFESGLETMACLDAEHARMRDMVLRYAPAGAAVLRGQISDVLGPDALENLLTQRHVAITPCIRRPGDVELTGMTIAEELAKLKAVRGLQEEVNDAADDLSGEADEGLTWRLAEAAKAADRAHRAGQEDKAEYVIADNGARLDRDQVTLSRNMFDQIDFSKGGKKDMCAETPDYKSTLNLPKTDFPMRAGLPKREPMWLEQWNEIGIYDRLREKETREPFTLHDGPPYANGHLHIGHALNKTIKDMIVRSHQMMGRDARYIPGWDCHGLPIEWKIEEQYRKKGRDKDQVPINEFRGECREFAKGWVDIQREEFKRLGVQGNWDKPYLTMDFHAERVIAEEFMKFLMNGTLYQGSKPVMWSPVEQTALAEAEVEYHDKDSFTIWVKFKVVGG-----DLEDAQVVIWTTTPWTMPSNKAVVYGAGISYGLYEITGTPDECWVNVGDKYLLADNMAADVLGRARLEEGQWARVRDVTTAELETISLKHPLHGVDGGNGEWDDLRDFRAADFVTDTDGTGFVHCAPSHGLDEYDLYRDLGMLDQVITYNVMPDGRYRDDLPLFGGKAILKPNGKEGNANAAVIDKLVEVGGLLARGKIKHSYPHSWRSKAPVIYRNTPQWFAAIDKTVGDGQDQFGTTIRERALTEIDNVNWTPKSGRNRLHAMMEARPDWVLSRQRAWGVPLTCFTRKGVLPTDADFLLRNAEVNQRIVEAFEVEGADAWYEDGAKARFLDGIVNPDDYDQVTDILDVWFDSGSTHAFTLRDREDGSEDGIADVYMEGTDQHRGWFHSSLLQSVGTTGRAPYRNVVTHGFTLDSKGMKMSKSIGNTIVPEKIIQQYGADILRLWVAQTDYTNDQRIGDEILKGTSDSYRRLRNTMRYMLGALSDFTEADRVDAADMPELERWVLHRLAELDTVVRDGYSRFDFQGVFRAIFEFATLDLSAFYFDIRKDALYCDGDTLRRRAARTVLDLLFHRLTTWLAPVLVFTMEEVWLERMGGEGSSVHLQDIPATPEGWLDTELAAKWAKVRAARRVVTAALEVQRTAKVIGASLEAAPVVYVEDAAQRAALESVSFDDVTITSQITVTGDAAPEDAFRMPETDGVAVSFVLAQGEKCARCWKVLPDVGTHKHAGVCARCDDAV-MAASQNIRIRLKAFDYRVLDASTQEIVNTAKRTGASVRGPIPLPNKIEKFTVLRGPHVDKKSRDQFEIRTHKRMLDIVDPTPQTVDALMKLDLAAGVDVEIKLQSMSDQINTLEDL-----ASVAGVAATPEVELTPREPVRDEFGRAYATGKRKDAVARVWIKPGSGKVIVNGKPQNEYFARPVLQMILQQPFGITGTDGQFDVYATVKGGGLSGQAGAVKHGVSKALQLYDPSLRGALKAAGFLTRDSRVVERKKYGKAKARKSFQFSKRMPTIQQLIRKPRQPKRKTSKSMHLQECPQKRGVCTRVYTTTPKKPNSAMRKVAKVRLTNGFEVISYIGGESHNLQEHSVVLIRGGRVKDLPGVRYHVVRGVLDTQGVKDRKQRRSKYGAKRPKMAREAKRTKKKVSKNIAAGVAHVNSSFNNTKILISDVQGNAIAWSSAGTMGFKGSRKSTPYAAQMAAEDAGKKAQDHGVKTLEVEVQGPGSGRESALRALAAAGFNITSIRDVTPMAHNGCRPPKRRRVMN-DRRGLLIILSSPSGAGKSTLAKRLMVWDETLSFSVSATTRAPREGEVDGKDYRFVQEDTFRQWVGEGEMLEHAHVFGNFYGSPKAPVEEAINAGNDVLFDIDWQGAQQIRNSPLGLYTLSIFLLPPSIAELHRRLISRGQDDEATISKRMQKSWDEISHWDGYDYVLVNDDLDVTELKLKTIISAERLKRTQQPQLSDIARRLQSQFEDLKMAQSFLGQKRLRKYYGKIREVLEMPNLIEVQKSSYDLFLNSGDAETPTDGDGIQGVFQSVFPIKDFNETSILEYVKYELEKPKYDVEECQQRDMTYAAPLKVTLRLIVFDVDEDTGAKSVKDIKEQDVFMGDMPLMTPNGTFVVNGTERVIVSQMHRSPGVFFDHDKGKTHSSGKLLFACRIIPYRGSWLDFEFDAKDIVFCRIDRRRKLPVTTLLYSLGLDQEAIMDAYYNTVTYRLEAGKGWVAPFFPERVRGTRPTYDIVDADSGEILFEAGKKVTPRAVKKLIDEGNVTSLLLPYDHIQGTFVSKDIINEETGAIYVEAGDEMTLEYDKDGTLIGGTAKELIDAGITEIPLLDIDNVNVGPYMRNTMAADKNMNRDTALMDIYRVMRPGEPPTVEAASNLFDTLFFDSERYDLSAVGRVKMNMRLALEKEDTQRTLDRDDIVACIKALVDLRDGRGDIDDIDHLGNRRVRSVGELMENQYRVGLLRMERAIKERMSSVEIDTVMPQDLINAKPAAAAVREFFGSSQLSQFMDQTNPLSEVTHKRRLSALGPGGLTRERAGFEVRDVHPTHYGRMCPIETPEGPNIGLINSLATFARVNKYGFIETPYRKVKDGIVSDDVQYMSATEEMRHTVAQANAHLDENMKFVNDLVSTRKSGDYTLSPSMNVDLIDVSPKQLVSVAASLIPFLENDDANRALMGSNMQRQAVPLLRAEAPLVGTGIEEVVARDSGAAYMARRAGVIDQVDATRIVIRATEDLELGDAGVDIYRMRKFQRSNQNTCINQRPLVKVGETVTKGQVIADGPSTDMGELALGKNVIVAFMPWNGYNYEDSILISERVSRDDVFTSIHIEEFEVAARDTKLGPEEITRDIPNVGEEALRNLDEAGIVYIGADVEPGDILVGKITPKGESPMTPEEKLLRAIFGEKASDVRDTSLRVKPGDFGTVVEVRVFNRHGVEKDERALQIEREEVERLARDRDDEMAILDRNIFARLREVILGKVAVKGPRGVKPNAQITEEVLEVLTRGQWWQIALEDEDDAKIVEALNEQYEIQKRALDARFEDKVEKVRRGDDLPPGVMKMVKVFVAVKRKLQPGDKMAGRHGNKGVISKVVPMEDMPFLEDGTPVDFCLNPLGVPSRMNVGQILETHMGWAARGLGINVDEALQEYKRSGDMTPVREAMKLAYGDDVYDEGIAGMEEDDLLEAAGNVTRGVPIATPVFDGAKEADVNDSLARAGFDTSGQSVLFDGRTGEQFSRKVTVGVKYLLKLHHLVDDKIHARSTGPYSLVTQQPLGGKAQFGGQRFGEMEVWALEAYGAAYTLQEMLTVKSDDVAGRTKVYESIVKGEDNFEAGIPESFNVLVKEVRGLGLNMELLDAEDEEMPLYEHVMIARQDLSNTQAEGLIEHFGTVLSDNGGALVDSEYWGVKTMAYKINKNRKGHYAFLRSDAPATAVQEMERLMRLHDDVMRVLTIKVDAHKELPSVQMQKRDERPERRERRDISELCVDTLNEEQARTALENLAAQLVAANTAYHTNDAPEISDAEFDLLKRLNADIEKRFPNLKRSDSPSDQVGAAVAAGFGKITHSVAMLSLSNAFSDEDVQEFDGSVRKYLGLAADASLSFTAEPKIDGLSLSLRYENGILVQAATRGDGSVGENVTANARTISDVPHEITGAPEVLEVRGEVYMSHADFAALNARQEERGGKTFANPRNAAAGSLRQLDAEITRSRPLRFFAYAWGSISAPLGNTQWESIEHLSKLGFSTNPLTALCAGPTEMINHYRDIEAQRAGLGYDIDGVVYKVNDLALQNRLGFRSTTPRWAIAHKFPAELSWTRLEGIDIQVGRTGALSPVARLTPVTVGGVVVSNATLHNEDYIKGLDSKGQVIREGKDIRIGDLVQVYRAGDVIPKVADVDITKRLDGVTPFEFPQTCPQCGSDAIREPGDAVRRCTGGLICPAQAVEKLKHFVSRGAFDIDGLGAKQVEQFHTDGWVTEPADIFTLQERFGSGVQQLKNREGWGDKSAAGLFAAIDAKRKIPLARLLFGLGIRHVGESASNMIALHYGTWAALEQAMDAAQPQEGAAWDDLVGIDGMGAVMAGSLVGAFAQEAERASINRLVAQLDVQEAVRADTSGSPVAGKTVVFTGTLEKMTRAEAKAKAERLGAKVSGSVSAKTDILVAGPGAGSKEKKAIELGIQILDEDGWLGLVDGLMIHKNWAELIKPTQLEVKPGNDPARQATVIAEPLERGFGLTMGNALRRVLMSSLQGAAITSVQIDNVLHEFSSVAGVREDVTDIILNLKGVSIRMEVEGPKRLSISAKGPGVVTAGDIGETAGIEILNRDHVICHLDDGADIYMELMVNTGKGYVSADKNKPEDAPIGLIPIDAIYSPVKKVSYDVQPTREGQVLDYDKLTMKVETDGSLTPDDAVAFAARILQDQLGIFVNFDEPESASRQDDDDGLEFNPLLLKKVDELELSVRSANCLKNDNIVYIGDLIQKTEAEMLRTPNFGRKSLNEIKEVLSGMGLHLGMDVEDWPPENIEDLAKKFEDNFMARDDNRGGNRRNQREEAPEFADRLVAINRVSKTVKGGKRFGFAALVVVGDQKGRVGFGKGKAKEVPEAIRKATEQAKRQMIRVQLREGRTLHHDMSGRHGAGKVIMRTAPEGTGIIAGGPMRAVFEMLGVKDVVSKSIGSQNPYNMIRATMDGLKKEQSPRSVAQRRGKKVADILPKREDASDSSAQVAEEAMTDPIADMLTRIRNSQLRGKSTVMTPGSKLRAWVLDVLADEGYIRGYEKTTGADGHPAIEISLKYYEGEPVIRELKRVSKPGRRVYMAAQDLPSVRQGLGVSIVSTPRGVMSDASARAANVGGEVLCTVFMHDIRAIRENPDAFDAALARRGDAPLSSSILELDTARRTKIQAAEAAQAEQNAASKNVGAAKAKGDDAEFERLRALVSEKKAEVAAMQAEAKDLDVQLTDMLARIPNTPADDVPDGADEAANVEVKRWGDVRAFDFAPREHFDLDSVAASMDFETAAKTSGARFVMLKRGVARVHRALAQFMLDTHVDENGLTEVNSPVLVRDEAMYGTDKLPKFGEDSYRTEEGMWLVPTSEVPLTYTVAGDTLDASDLPRRMTSHTLCFRSEAGSAGRDTSGMLRQHQFEKVEMVSITLPDESDAEQKRMLGCAEGILEKLGIPYRTLILCTGDMGFGARRTFDIEAWLPGQNAYREISSVSTTGDFQARRMNARFKPAEGGKPQFVHTLNGSGLAVGRCLIAVLENGQQADGTVKLPAVLAPYLGGKTVLGLDGQLAMAITASMVKELRDTTGAGMMDAKKALTENNGDMEASVDWLRTKGLAKAAKKSGRTAAEGLVAVQVEGGRGVAVEVNSETDFVGKNADFQKMVAGIAKVAVSAADIDALKAADMGGKTVEQTVTDAVAVIGENMSVRRMNSIEGDLVVSYVHNAAAPGMGNIGVLVAMTGGDEAFGKQIAMHIAATNPASLSEEDLDPAVIEKEKQVQIDIAKESGKPDAVIEKMIVGRMQKYMSEVTLVNQAFVINPDLTVAAAAKEAGATITGFVRLEVGEGIEVVKEDFAAEVAKVSQGMNQELTNNPFNPVAPTKTFDEIKVSLASPERILSWSFGEIKKPETINYRTFKPERDGLFCARIFGPIKDYECLCGKYKRMKYRGVVCEKCGVEVTLQKVRRDRMGHIELASPVAHIWFLKSLPSRIGLMLDMTLRDLERVLYFENYVVIEPGLTDLTYGQMLTEEEYMDAQDGYGMDAFTANIGAEAIREMLAAIDLEAEADQLREELKVATGELKPKKIIKRLKVVESFLESGNRPEWMVLTVIPVIPPELRPLVPLDGGRFATSDLNDLYRRVINRNNRLKRLIELRAPDIIVRNEKRMLQESVDALFDNGRRGRVITGANKRPLKSLSDMLKGKQGRFRQNLLGKRVDFSGRSVIVTGPELKLHQCGLPKKMALELFKPFIYSRLEAKGLSSTVKQAKKLVEKERPEVWDILDEVIREHPVMLNRAPTLHRLGIQAFEPVLIEGKAIQLHPLVCSAFNADFDGDQMAVHVPLSLEAQLEARVLMMSTNNVLSPANGAPIIVPSQDMILGLYYVTLEREGMKGQGKVFGTVDEVQHALDAGEVHLHAKIQARIKQIDNEGNEVIIRFDTTPGRMRLGALLPLNAKAPFDLVNRLLRKKEVQQVIDTVYRYCGQKESVIFCDQIMTLGFREAFKAGISFGKDDMLIPDSKWPLVEETRDQVRDFEQQYMDGLITQGEKYNKVVDAWSKCNDKVTDAMMGAISDTTYAEDGSENEPNSVYMMAHSGARGSVTQMKQLGGMRGLMAKPNGDIIETPIISNFKEGLTVLEYFNSTHGARKGLSDTALKTANSGYLTRRLVDVAQDCIVRMHDCGTDVAITATAAVNDGEVVSSLAERLLGRVVAEDIMRPGTEEVLIANGTIVDERLADIIDEASVASARIRSPLTCEAEEGVCAMCYGRDLARGTLVNQGEAVGIIAAQSIGEPGTQLTMRTFHIGGVAQGGQQSFQEAGQAGKIRFENSNTLQNSSDETMVMGRNMKLLIIDENGDERASHKVGYGTKLFVKEGQSIARGDKLYEWDPYTLPIIAEAKGIAKHVDLISGISVKDETDDATGMTQKIVIDWRSAAKGNELKPEIILVDADGEPVRNSAGNPITYPMSVDAIMSMEEGSDVEAGDVLARIPREGAKTKDITGGLPRVAELFEARRPKDHAIIAEIDGYVRYGKDYKNKRRIAIESSEDPDHKVEYMVPKGKHIPVAEGDFVQKGDYIMDGNPAPHDILAIMGVEALADYMIDEVQDVYRLQGVKINDKHIEVIVRQMLQKWEIQESGDTTLLKGEHVDKLEFDQANEKAISKGGRVAKGEPILLGITKASLQTRSFISAASFQETTRVLTEASVQGKRDKLVGLKENVIVGRLIPAGTGGATQQMRRVAADRDNVVVEARRIEAEKAAALAAPVAPASDVVGGDVFSDTSGDEESRDMSRRHAAEKREVLPDAKFGDLVLTKFMNNLMIDGKKSTAERIVYNAFDRVEAKIKRAPVEVFHEALENIQPSVEVRSRRVGGATYQVPVEVRPERRVALAIRWLIKAARARNENTMEERLAGELMDAVQSRGTAVKKREDTHKMADANKAFSHYRWMPYAQTDKSEATPILANPAPDVRTRPKLEGGKTFVLKTEFEPAGDQPTAIKELSEGIRNGERNQVLLGATGTGKTFTMAKMIEETQRPAIILAPNKTLAAQLYGEFKGFFPDNAVEYFVSYYDYYQPEAYVARSDTFIEKESQINEQIDRMRHSATRALLERDDVIIVASVSCIYGIGSVETYGAMTQDLTVGNSYDQRQVIADLVAQAYKRNDASFQRGAFRVRGDSLEIFPAHLDDRAWKLSFFGEELESITEFDPLTGEKTGNMDQVRIYANSHYVTPKPTMNQAVIGIKKELRMRLDQLVGEGKLLEAQRLEQRCNFDLEMLEATGVCNGIENYSRYLTGRAPGEPPPTLFEFIPDNAIVFADESHVSVPQIGGMYKGDFRRKMTLAEHGFRLPSCMDNRPLKFEEWDAMRPQSVFVSATPANWEMEQTGGVFTEQIIRPTGLIDPVIEIRPVEMQVDDLLDEVRKVAAEGMRTLCTTLTKRMAEDLTEYMHEQGIRVRYMHSDIDTIERIEILRDLRLGAFDVLIGINLLREGLDIPECGLVAILDADKEGFLRSETSLIQTIGRAARNSEGRVIMYADRITGSMERAMGETERRRVKQLAYNEEHGITPMTVKKNVEDILAGLYKGDTDQSRVTAKIDNPLAGGNLQSVLEGLRTDMRKAAENLEFEEAARLRDEVKRLESVDLAIADDPMARQYAVEKAVGEARVKSGRSTMGRGGMRGGVKRR-SRMAAKPFFRRRKVCPFSGDNAPAIDYKDTRLLQRYISERGKIVPSRITAVSAKKQRELARAIKRARFLALLPYAVNMPKRILTGTVTSDANEQTVTVSVERRFTHPVLKKTIRKSKKYRAHDENNTFKVGQQVRIIECAPRSKTKRWEVITAEAMANTPQAKKRARQNEKRFAINKARRSRIRTFIRKVEEAIESGVKDDAVAALKAAQPELMRGVTKGVYHKNTASRKMSRLAARVKAIAMSRSVWKGPFVDSYVLKKAEASRESGRNEVIKIWSRRSTILPQFVGLTFGVYNGHKHIPVNVTEDMIGQKFGEFSPTRTYYGHAADKKAKRKMARIAGVNIPTAKRVPIALTYITGIGNSSAQAICEAVKIDFTRRINELSDDEILKIREYIDEHYTVEGDLRRDTQMNIKRLMDLGCYRGLRHRRNLPVRGQRTHTNARTRKGPAKAIAGKKKMSMKIRLARGGSKKRPFYRIVAADSRMPRDGRYIEKLGTYAPLLPKDSEDRVKMNMERVEYWLGQGAQPTDRIQRMLEAAGVRPKTERNNPKKGTPGKKAQDRVEEKAAKATAAAEAAAAPAEEAAAEEMSITKEEKARLMSEFGAKEGDTGSPEVQVAVLSSRIATLTEHFKTHKKDNHGRRGLLKMVATRRKLLDYVKSKDEARYQDLIKRLGLRRMQTLNEIRSSFLNYFGDNGHAIVPSSPLVPRNDPTLMFTAAGMVQFKNLFTGVETRDYSRATSAQKCVRAGGKHNDLDNVGYTARHHTFFEMLGNFSFGDYFKEDAIPMAWDLLTKVFGIDASRLLVTVYHTDDEAVEIWKKHAGLSDDRIIRIATDDNFWSAGPTGPCGPCTEIFYDHGDHIWGGPPGSPEEDGDRFVEIWNLVFMQYEQFEDGTRRDLPNKSIDTGMGIERVAALLQGTNDNYATDLMRSLIEASANATSSDPDGPGKTHHRVIADHLRSTSFLMADGVMPSNDGRGYVLRRIMRRAMRHAHLLGVQDPLMHRLVPALVAQMGQAYPELGQAQSMIEQTLLQEETRFRQTLDRGLKLLDEELVLLPEGANLPGASAFKLYDTFGFPLDLTQDALREKGRAVDTDGFDTAMAEQKAKARAAWSGSGEAADAAIWFDVADKSGLTEFLGYDTETAEGKIAALVQDGKAVVQANAGDEVQIALNQTPFYAESGGQLGDTGLITTQTGTARITDTRKAAGVIIHFAKVEKGSISKDQSAVLDVDHTRRTAIRANHSATHLLHEALRHALGDHVAQRGSLNAQDRLRFDFSHNEAISSDDLARVETEVNAYIRQNTPVETRIMTPDDARALGAQALFGEKYGDEVRVVSMGLQDGSKKGADGNTYSLELCGGTHVRRTGDIGAFVMLGDSASSAGVRRIEALTGEAALKHLRDQDSLLAQTALELKSPSSSVPERVRALMDERRSLANEVAQLRRELAMSG--GSSDQDVREINGVSFVSQVLTGITGKDLPALMDEHKARIGSGAVLLIADTGDKAAVAAGVTDDLKGTLSAVDILRAAVAELGGKGGGGRPDMAQGGGASAENAQAAIAAAETVIKGMGFKMGIVGLPNVGKSTLFNALTRTAAAQAANFPFCTIEPNVGEVAVPDARLDKLVEIAKSQSIIPTRMTFVDIAGLVKGASKGEGLGNQFLANIRETDAIAHVLRCFEDDDVTHVEDRVDPVADAETIDTELMLADLESIEKRRAGLVRKIKGNDKDAVQQDRLLAQAQAAIEDGQPARVVEVDEDDAKAWRMLQLLTTKPVLYVCNVGVDDAAKGNAHSAAVAAMAEAQGNSAVVISAQIEEEISQLDADDAQMFLDDMNLEEAGLDRLIRAGYELLHLETYFTVGPKEARAWTIKQGTSAPKAAGVIHGDFEKGFIRAETIAYDDFVTLGGEGPAKEAGKMRAEGKSYIVKDGDVLHFLFNTMGWKSLDDMDLHGKRVLVRVDINVPVEKGQVTDATRIERIVPTVHDILAKGGSPILLAHFGRPKGKVNLDMSLRQVVPALRHALMRSVALVETLEAAEKMTAEVAAAEVELIENIRFHAGEEANDPEFAQRLAKLGDVYCNDAFSAAHRAHASTEGIAKYLPSCAGRLMQAELSALESALSKPERPVGAVVGGAKVSTKIALLENLVNRLDVLVIGGGMANTFLVAQGAQLGASLCEMDYLDTARDIMAQAAKAGCRVILPVDGLVATEFKSGAAHEVALLGPDTVLDADQMVLDAGPETIKQIKIAFEGLKTLIWNGPMGAFEIAPFDTATVAAAQCAAERTRNGALISVAGGGDTVAALNQAGVADDFTYISTAGGAFLEWMEGKTLPGVAALIKMALPEFSMRQLLEAGVHFGHQTQRWNPRMGPYIYGARNGIHIMDLTQTVPMLEDALKIIRDTVAKGGSVLFVGTKRQAAQPIAEAAEKCAQYYMNHRWLGGTLTNWQTVSKSIQRLKHIDEQSEMGFSGLTKKERLGMERDQGKLEASLGGIREMGGRPDLIFVIDVRKEQLAIAEANKLGIPVVAVVDTNCSPDGIDYIIPGNDDAARAIALYTDLAARAALDGMSAQLGAAGVDLGAMEEAPMEEAL-------AEEAAAEAPAAS---MTKRTAAKHKIDRRMGENIWGRAKSPVNRREYGPGQHGQRRKGKISDFGIQLRAKQKLKGYYGDLTEKQFRRIYGEAERVKGDTGENLIGLLERRLDAVVYRAKFVATVFAARQFVNHRHVRVNGKLVNIPSYRVKEGDVIEVRDRSKQMVALVEATQLAERDVPDYLEVDHSKMTATFVRTPALGDVPYPVVMEPNLVVEFYAKNMGNKVNPIGMRLQVNRTWDSRWYADTKDFGDLLLEDLAIRKFIKKECHQAGISRVIIERPHKKCRVTIHTARPGVIIGKKGADIEGLRKKLAAFTASELHLNIVEVRKPELDAALVGESIAQQLERRVSFRRAMKRAVQNAMRMGALGIRVNLAGRLGGAEIARTEWYREGRVPLHTLRADIDYAHVEAMTAYGIIGIKTWIFKGEIMEHDPAARDRKAQEMQDGPAPRGAGGRRMARRRKGRDISGWVVIDKPAGPTSTTVVNKVRWAFDARKAGHAGTLDPDATGVLAIALGEATKTVPYITDALKAYVFTVRLGVSTNTDDAEGEVLATTDLRPDDSAIKEALNGFVGDIEQVPPQFSAVKIDGERAYKRARDGEQMEIAARPLFVESLIMLDRPDVDHVTLEMVCGKGGYVRSIARDLGEVLGCKAHVRELRRTWSGPFDVDNAITLEQVDEMAKTLALDEFLGPLEMGLQDLPQVTASAEGANKLRHGNPGMVFAKDVEYGDECWAVYEGQAVAVGTYRAGELHPSRVFN-TGMFVLGLTGSIGMGKSTTAQMFVELGCALWDADAAVHRLYAQGGAAVAPFAAAFPDAIVDGAVSRLALKDIIGRDPAALKQIEQIVHPLVGEDRADFLTQTTADIVVLDIPLLFETGGDARVDATACVFTDDATQEARVMARGTMTREQFLSIKAKQMPAAEKCTRATYVIQTDTLEHARTQVQNVVETIRSQLNA-MDALDIVFEAGAWSTDEFTPLAQGAVIATLRHMALEADACEITLLACDDARIAELNAEFRGKPTATNVLSWPAQPVAPPSEGQAPPAVERGFDDMFELGDIALSYETCAREAAESGKPFSDHLTHLIVHGVLHLLGYDHETDGDAALMEGLEVEILGNLGLDDPYSVDIIMARYIFITGGVVSSLGKGLASAALGALLQARGFSVRLRKLDPYLNVDPGTMSPFEHGEVFVTDDGAETDLDLGHYERFTGVSARKTDSVSSGRIYSTVLEKERRGDYLGKTIQVIPHVTNEIKDFLAVGEDEVDFMLCEIGGTVGDIEGLPFFEAIRQFSHDKPRGQCIFMHLTLLPYLAASGELKTKPTQHSVKELQSIGIAPDILVCRSEHPIPEKEREKIALFCNVRKDSVVAAYDLKSIYEAPLAYHAQGLDQAVLDAFDISPAPRPDLSVWNDVYDRIHNTDGEVKVAIVGKYTQLGDAYKSIAEALTHGGMANRVRVNVEWVDAEVFDSDDVAQHLEGYHAILVPGGFGERGTEGKIKAAQYARENKVPYLGICLGMQMAVIEAARNVAGLSTAGSEEFDHESGKRRFEPVVYHLKEWVQGNHKVERKVGDDKGGTMRLGAYDATLKEGSKVAEVYGTTAIDERHRHRYEVDIAYREQLEAAGMTFSGMSPDGKLPEIVEWSDHPWFIGVQFHPELKSKPFKPHPLFKDFVRAAKDMSRLV-MTSLTLLGIESSCDDTAAAVLR-GQAGDAQVLSSIVMGQTALHADFGGVVPEIAARAHAEKLDLCVAQALEAAELSLDDIDAVAVTAGPGLIGGVVSGVMCAKGIALGRGLPLYGVNHLAGHALTPRLTDGVTFPYLMLLVSGGHCQFLIVSGPDQFQRLGGTIDDAPGEAFDKVARLISLPQPGGPAIETRAKAGDSKRFKLPRPLLDRQGCDMSFSGLKTAVLRQRDHLLKD-GTLSAQDQADLAAGFQAAVVDVLAEKTRRALAVYAPMTD-TPSICVAGGVAANMAIRSALETVAHKFDAAFIAPPLALCTDNAAMIAYAALEQMGTRAPDGMDLSARPRWPLDQSAPAMLGSGKKGAKAMAKNKSHGGAGPTQRQLRVGETTRRALSEILARGDVHDPELNRMSITVGEVRMSNDLRIATVYVLPLGGIGKEDVLKMLARNNYELRRMVAKRLTLKFAPELRFQLDMTFDQMDETSRMLSQDIVRRDADAD

>'Su-dubiusDSM-16472'

MKASELHDKTPDQLRDELVNLKKESFNLRFQQATGQLENPARLKTVKRDVARVHTVLNQKAAAAAAE-MAMADLLTMDSKKTAEKQKALDSALAQIERQFGKGSIMKLGAEGAIQDIKASSTGSLGLDIALGIGGLPMGRIIEIYGPESSGKTTLTLHCVAEQQKAGGVCAFVDAEHALDPQYAKKLGVDIDELLISQPDTGEQALEITDTLVRSGAVNMVIVDSVAALTPKSELEGEMGDSSVGVQARLMSQAMRKLTGSISRSNCMVIFINQIRMKIGVMFGSPETTTGGNALKFYSSVRLDIRRIGALKDRDEVVGNATKVKVVKNKVAPPFKQVEFDIMYGEGISKMGELLDLGVKAGVVDKSGSWFSYGDERIGQGRENAKNFLKENTAMASEIEDKIRAAHGLDFDGSGGDDADILEAMPRYTPAEIEARWQQAWEKDGIFQAVRNADKPKYYVLEMFPYPSGRIHMGHVRNYTMGDVIARYKIATGHNVLHPMGWDAFGMPAENAAMAIGGHPADWTYDNIAEMKKQMKPLGLSIDWSREIATCHPGYYGQQQALFLDFLKEGLVYRKNAVVNWDPVDMTVLANEQVENGCGWRSGAPVERRELTQWFFKISDHSEELLSALDSLDNWPAKVKLMQANWIGKSRGLQFAFSTIEAPEGFDRIEVYTTRPDTLLGASFVGISPDHPLAKTLERDDEAVAAFCAECRKGGTTEEAIETAEKLGYDTGIRVRHPFDTAHELPVYIANFILMDYGTGAIFGCPGHDQRDFDFASKYDLPIISTFLPSEDASPKLAEAFVPQKSEKVFYNRGFAGNQWQTGEDAVDAAIAFCEENGIGQGVTKYRLRDWGLSRQRYWGCPIPVIHCDDCGVVPEKKENLPIELPYDVTFDTPGNPLDRHPTWRNCACPACGKDALRETDTMDTFVDSSWYFARFTAPRAETPTVMEDAQYWMNVDQYIGGIEHAILHLLYSRFFARAMQITGHLPESAVEPFDALFTQGMVTHEIYQTRDGNGRPVYHLPEEVTEGKL-ADGTEVEIIPSAKMSKSKKNVVDPLHIISNYGADTARWFVLSDSPPERDVEWTASGAEASYKHLSRVWNICDRVSEMDRDATGTGDDDLLRAMHKTIHDVTMGVESFGFNAAIAKLYAFTATLQKSKAGYAAQREAIMTLAQLMSPMTPHLAEDIWAHQGGEGLITKAPWPKADEKMLVDDTVTLPIQINGKRRAEIQVPADMPKEEVEKIALAHEAVIRTLDGATPKKVIVVPGRIVNVVAMAITSANQLELLQTAEAVAREKMIDPGLVVEAMEESLARAAKSRYGAEMDIRVDIDRKTGKATFTRVRTVVEDDELENYQAEFTVDQAKQYMADPKVGDTYVEEVPPVEMGRIAAQSAKQVILQKVREAERDRQFEEFKDRAGTIINGLVKREEYGNVIVDVGAGEAILRRNEKIGRESYRPNDRIRVYIKDVRREQRGPQIFLSRTAPEFMAELFKMEVPEIYDGIIEIKAVARDPGSRAKIAVISHDGSIDPVGACVGMRGSRVQAVVNELQGEKIDIIPWNEDQPTFLVNALQPAEVSKVVLDEEAGKIEVVVPEEQLSLAIGRRGQNVRLASQLTGLDIDIMTEEQESQRRQAEFELRTKLFMDNLDLDEFFAQLLVSEGFTNLEEVAYVEVDELLVIDGVDEDTAGELQARARDVLEAQNKAALDNARALGVEDSLVEFEGLTPQMLEALAKDDVKTLEDFATCADWELAGGWTTVNGERVKDDGALEPFEVSLEDAQAMIMTARVMLGWVDPTELEADADEDDVETDGENAEEAEAMAHKKAGGSSRNGRDSAGRRLGVKKYGGEAVIPGNIIVRQRGTKFWPAEGVGMGKDHTIFAVVDGAVTFHKGLKNRTFISVLPRAEAAEMAKRWYSVSVLSNFEKKIAEQIRASVAEQELEDQIDEVLVPTEEVIEVRRGKKVTTERRFMPGYVLVHMEMSDAGYHLINSINRVTGFLGPQGRPMPMRDAEVQAILGRVQEGEEAPRTLIHFEIGEKVKVADGPFEDFDGMIEEVDEDNQRLKVSVSIFGRETPVELEFTQVNKQIMSFTLAIVGRPNVGKSTLFNRLVGKRLALVDDQPGVTRDLREGAAKLADLRFTVVDTAGLEEVTDDSLQGRMRRLTERAVDMADICLFMIDARVGVTPSDMVFAEILRKKSAHVILAGNKAEGKAADAGMIEAYSLGLGEPIRMSAEHGEGLNDLYTMLMPLADEYEDRAVKDAPETDVDLPEDDDDLETVPMPTRAKPLQVAVVGRPNAGKSTLINQILGEDRLLTGPEAGITRDAISLMTDWAGPVPMRIFDTAGMRKKAKVQEKLEKLSVSDGLRAVKFAEVVVVLLDAEIPFEQQDLRIADLAEREGRAVVVAVNKWDIEENRQEKLRDLKESFERLLPQLRGAPLITVSAKTGRGLDRLQAAIMRAYETWNRRVTTAQLNRWLSGMLEAHPPPAPQGKRIKLRYMTQAKTRPPGFVVMCSHPDKVPDSYSRYLVNGLRVDFDMPGTPIRLWMRGQSDANPYKNRKKAAPSKLRKHTAGRRKDMRLIFMGTPDFSVPILDALVQAGHDICAVYCQPPRPAGRGKKPRPSPVQQRAEEIGLLVRHPVSLKHPEPQEEFAELDADAAVVVAYGLILPQAILDAPKQGCLNIHASLLPRWRGAAPIHRAIMAGDEKTGVCIMQMEAGLDTGPVLLCEETKIGAAETTAELHDRLSAMGAVAINKALSQLLQLTPQPQPEEGVTYAAKIDKAEAKIDWTRPAVEVDRLIRGLSPFPGAWFEIDGQRVKVLGSVLDEGTGAAGEVLSDDLRIACGEGAVRLTRLQRAGKGAQDADVFQRGAQIAVGRRLGEGMTDTSAYRVLARKYRPETFADLVGQDAMVRTLKNAFAADRIAQAFVMTGIRGTGKTTTARIIAKGMNCIGPDGNGGPTTDPCGVCEHCTAIMEGRHVDVMEMDAASNTGVANIREIIDSVHYRAASARYKVYIIDEVHMLSTGAFNALLKTLEEPPEHVKFIFATTEIRKVPVTVLSRCQRFDLRRIEPEVMIVLLRKIATAEGAEITDDALALITRAAEGSARDATSLLDQAISHGAGETGAEQVRAMLGLADRGRVLDLFDMVLRGDAGAALTELSGQYADGADPMAVLRDLAEITHWVSVVKITPDAAEDPTIAPEERARGQQMAETLPMRVLTRLWQMLLKALDEVAAAPNAMMAAEMAVIRLTHVADLPSPEELVRKLQNSTPPPAPPVGGGGGGNGAPQGGAQAVQHAQQRMASNPGPQGQTIALAQDLNAALARFPTFEHVVELIRVNRDVKLLVEVETCLQLAAYQPGRIEFVPTDDAPRDLAQRLGQKLQLWTGNRWAVSLVNEGGAETIAQIRDARELALKKQAQEHPMMQAVLAQFPKARITAIRTPEDIAAAATAEALPEVEDEWDPFEDGMPKMKTKSSAKKRFKVSATGKVIGSQAGKQHGMIKRTNKFIRNARGTTALSEPDAKIIKGFMPYARMNLFAEIRHLIIATLEQMVAQDALPATLNFDPITAEPPRDPAHGDMATNAAMVLAKPAGMKPRDIAEALAAQLQNDPRITSAEVAGPGFINLRLAPSVWQNVARQVLSQGTDFGRAILGAGQRVNVEYVSANPTGPLHVGHTRGAVFGDALASLLDFAGYDVTREYYINDGGAQVDVLARSVYLRYLEANGKEVAFPDGTYPGDYLIPLGEALAKMYGDKLVDQPESEWLDHIREFATDAMMNLIREDLAALGVEMDVFYSEKSLYGTGQIEAAIASLEAKGLIYEGVLEPPKGKKPEDWEPREQTLFKSTEHGDDVDRPVKKSDGSWTYFAPDIAYHYDKVSRDFDMLIDVFGADHGGYVKRMKAAVSALSDGHVPLDIKLTQLVKLYKNGEPFKMSKRAGTFVTLRDVVDQVGPDVTRFVMLTRKNDAMLDFDFDKVLEQSRENPVFYVQYAHARVASILRKAQEAGVEVADATLMDADLTKLDHDAELGLLRKLAEWPRLVETAARSNEPHRVAFYLYELASDFHSLYHLGRSEDGLRALQEGDAATSQAKIALSRAVAIVIAAGLGILGVTPAQEMRMSAIDNLPPLREVINTHELAARKSLGQNFLLDLNLTAKLARQAGDMADCDVLEIGPGPGGLTRGLLAEGARHVLAIEKDRRCLPALAEVAEHYPGRLTVIEGDALEIDPLSHLTPPIRVAANLPYNVGTELLVRWLTPPEWPPFWQSLTLMFQREVAERIVATPGSKAYGRLAVLAQWRSDARIVMQLPPGAFTPPPKVSSSVVHLTALPEPRYPADPAVLSRVVAMAFNQRRKMLRSALKGAAPDIEDRLQAAGLKPTDRAEQIPLEGFCALAREIAKGMNLIAEIEAEQVAELGKEIPDFRAGDTIRVGFKVTEGTRTRVQNYEGVCISRKNGHGIAGSFTVRKISFGEGVERVFPLHSTNIDSITVVRRGRVRRAKLYYLRSRRGKSARIVENAHYKPRANA--MANTKRQLFLKRRMRVRNKLRKVNAGRMRLSVHRSSKNISAQLIDDVNGVTLASASTMEKDLGVVGKNNVEAATKVGALIAERAKKAGVEEAYFDRGGFLFHGKVKALAEAAREGGLKIMFAVIKTGGKQYKVQSGDMLRVERIAANAGETVQFNEVLMLGGDSPVLGAPMVKDAGVQAEVVDQIKGEKVINFVKRRRKHSSKRTKGHRQKLTLIKITDILASGAEKSGVAAAIGTGSVSA----AAVAA--AKPAKTKKAAAPKA---KAEKTAPKAKKADAGSDDLKELSGVGPALEKKLHEAGVTSFAQIAAWTEADIAEVDEKLSFKGRIQREGWVDQAKEKTKGMSRVKGGTVTHARHKKIIKAAKGYYGRRKNVFKVATQAVDKANQYATRDRKNRKRNFRALWIQRINAAVRSHDEALTYSRFINGLSLAGIEVDRKVLADLAVHEPEAFGAIVKQAQDALAAMQVNETLNEGLKRGYNITVTAAELEAKVNEKLAEAQPEVEMKGFRKGKVPMALLKKQFGQRIMGEAMQESIDGAMAEHFEKSGDRPAMQPEVKMTNEDWKEGDDVEVSMAYEKLPEIPEVDLSKIELEKMVVKADDAAVEEALASLAETAQDFKARKEGAKAEDGDQVVIDFKGSVDGEEFEGGAAEDYPLVLGSNSFIPGFEEQLVGVKAGEEKSVVVNFPEEYQAEHLAGKEATFACTVKEVKEPVAAEVNDEMAKKFGAEDLDALKGQIAERLEAEYAGASRAVMKRNLLDALDKEVSFDLPPSLVDAEAKQIAHQLWHEENPEVEGHDHPEVEPTDEHKTLAERRVRLGLLLAELGQKAEVQVTDAEMTQAIMNQARQYPGQERQFFEFVQQNQQMQQQMRAPIFEDKVVDHVVEQAKVTEKEISKDELQKAVEELEDEMKLHELSDNEGATKKRMRVGRGPGSGKGKMGGRGIKGQKSRSGVAIKGFEGGQMPIYQRLPKRGFNKPNRKSFAVVNLGLIQKFIDAKKIDAGNTIDGAALVASGLVRRELDGIRVLAKGDITSKIDLNVAGASQSAIEAVEKAGGKVTVTAPKAVEASEMIQMQTNLDVADNSGARRVQCIKVLGGSKRKYASVGDIIVVSVKEAIPRGRVKKGDVRKAVVVRTAKEVRRDDGTAIRFDRNAAVILNNNNEPVGTRIFGPVVRELRAKNFMKIISLAPEVLMRHARGYRRLNRTHEHRKALFSNMAGSLIEHEQIKTTLPKAKELKPIIEKMITLAKRGDLHARRQAASKLKEDQYVAKLFDILGPRYKDRQGGYVRVLKAGFRYGDMAPMAIIEFVDRDRDAKGAADKARLAEEEAAE-MLQPKRTKFRKQFKGSIKGLAKGGSDLNFGTYGLKALQPERVTARQIEAARRAMTRHMKRQGRVWIRIFPDVPVTSKPVEVRMGKGKGSVDFWAAKVKPGRIMFEIDGVGEDVAREALRLAAMKLPIKTRVVVREDWMTTIKLHNTATRKKEDFTPIDARNVRMYVCGPTVYDRAHLGNARPVIVFDVLFRLLRHVYGPEHVTYVRNFTDVDDKINARAATSGRSIGDITAETTQWYLDDMAAVGALEPDHMPRATQYIPQMVAMIRGLIDEGYAYEAEGHVLFRVRKYAEYGALSGRSVDDMIAGARVEVAPYKEDPMDFVLWKPSDEATPGWESPWGQGRPGWHIECSAMAHDLLGERFDIHGGGIDLQFPHHENEIAQSKCAG--HGFANYWLHNEMLQVEGKKMSKSLGNFFTVRDLLDQGVPGEVIRFVMLSTHYRKPMDWTEKKREEAEKTLRKWYLQAAEAEAATP-SPDLVTLLADDLNTHGALTECHRLSNAGDTAGLRGALKLLGLLDDLIPEWAAVQALDLSDVEAFLSDAHATAMETKDFAQVDRIKTALTSVGIEVQMSKDGVKLTPPPGFDRAQLEGLLMSQVKSSSKSDPNYKVIAENRRARFDYAIEEDIECGIILEGSEVKSLREGGANIAESYAAVEDGELWLVNSYVAPYKQAKTFQHEERRRRKLLVSRKQLADLWNATQRKGMTLVPLVMYFNHRGMAKIKIGVAKGKKLHDKRETAAKRDWSRQKQRLLKDHGMSAKHEHYDVIRKPIITEKATMASEQNAVVFEVAIESNKPMIKEAVEALFNVKVKAVNTSITKGKVKRFRGQMGRRKDVKKAYVTLEEGNTIDVSTGLMSKDKNPRRVADNEAMAKLRMLRTSPQKLNLVAALIRGKSVDKALTDLTFSKKRVAQDVKKCLQSAIANAENNHNLDVDELIVAEAYVGKNLTMKRGRPRARGRFGKIIKPFAEITIKVRQVEEQAMVSAVENMAANTSWAALGKATDLRNRILFTLGLLIVYRLGTFIPVPGIDGGALRDFMESAGQGIGGMVSMFTGGALGRMGIFALGIMPYISASIIVQLLTSMVPALEQLKKEGEQGRKKINQYTRYGTVALATLQSYGLAVSLQAGDIVADGQMGFGFACMITLVGGTMFLMWLGEQITARGIGNGISLIIFVGIIAEVPAAIAQFFASGRSGAISPAVIVGVLVMVIATIMFVVFMERALRKIHIQYPRRQVGMKMYDGGTSHLPIKVNPAGVIPAIFASSLLLLPVTVSTFSGNSTSPVMSWLLANFGPGQPLYLLFFVAMIVFFAYFYTFNVSFKPDDVADNLKNQNGFVPGIRPGKRTAEYLEYVVNRILVLGSFYLAAVCVLPEILRGQFAIPFYFGGTSVLIVVSVTMDTIQQVQSHLLAHQYEGLLERSQLRGKGAGTRKKRSPVRRMKFLDLCKVYIRSGGGGGGCVSFRREKYIEYGGPDGGDGGTGGSVWAEAVDGLNTLIDFRYQQHFFAKSGQPGMGKQRTGKDGDDIILRVPVGTEILDEDQETVIADLTELGQRVQLARGGNGGWGNLHFKSATNQAPRRANPGQEGVERTLWLRLKLIADVGLLGLPNAGKSTFLAATSNARPKIADYPFTTLHPNLGVVGVDNTEFVVADIPGLIEGASEGRGLGDLFLGHVERCAVLLHLIDGTSETVAEDYHTIIGELEAYGGDLAEKPRVTVLNKVDALDEDERATRLKELQKACGGDVMMMSGVAGEGVIEVLRTLRQNIDDDRLRFRISEEEETWQPMAAKLRKGDKVIVLSGKDKGKTGTISSVDPKSNKAIVDGVKIAIRATRQTQTSQGGRIPKAMPIDLSNLALVDANGKATRVGFKIEGDKKVRFAKTTGDVIDAMLRSGVIAKKVGMTRLFMEDGKQIPVTVLQLDKLQVVAQRTADRDGYTAVQLGAGSAKAKRTSQAMRGHFAAAKVEPKRKVAEFRVDAEAMLEVGEEIIADHYFAGQYVDVAGTSIGKGFAGAMKRHNFGGLRATHGVSISHRSHGSTGQCQDPGKVFKGKKMAGHMGAARVTTQNLEVVKTDSARGLIMVKGAVPGSKGGWVTVKDAVKKPFPEDAILPAALKSAAEEAAKAAEEAAAAAAAEAEAEAKRLAEEQAAQEAEALKAAEAEIAAEGSDADNSDADDKKEGDAMALKSYKPTTPGQRGLVLIDRSELWKGRPVKALTEGLTKSGGRNNTGRITMRRTGGGAKRLYRIVDFKRNKLDMSAVVARIEYDPNRTAFIALIQYEDGEQAYILAPQRLAIGDKIIAGAKVDIKPGNAMPFSGMPIGTIVHNIEMKPGKGGQIARAAGTYAQFVGRDGGYAQIRLSSGELRLVRQECMATVGAVSNPDNSNQNYGKAGRMRHKGIRPSVRGVVMNPIDHPHGGGEGRTSGGRHPVTPWGKPTKGAKTRNKNKASSKLIIRSRHAKKKGRMLDTATYTPRLQAEYREKIRAALKEEFGYKNDMMIPKLDKIVLNIGCGAEAVRDSKKAKSAQEDLTVIAGQKALTTVAKKSIAGFRVREEMPLGAKVTLRGDRMYEFLDRLITIAMPRIRDFRGVPGKSFDGRGNYAMGLKEHIVFPEIDFDKVDETWGMDIVIATTAKTDAEAKALLKAFNMPFNSMKLDVIKLDGGKAGSVDLDEALFGLEPRADILHRVVRWQRNNAQQGTHKVKTRSETSYSTKKIYRQKGTGGARHGDRNAPIFRKGGIYKGPTPRSHGHELTKKFRKLGLRHALSAKAKAGSLVIIDEATSEGKTAALAKQVSNLGWKRTLVIDGASVNENFAQAARNIEGLDILPSMGANVYDILKRDTLVITKAGIEALEARLKMENVVLIIHLILALGLIAVVLLQRSEGGGLGI-GGGGGAVSGRSAATALGKITWILAAGFIATSITLTIIAAEKSAGSSVIDRLSATPPAQNQDGSPALPAGDDLLPPSADGNTPLVPAADMAFFTKLKDRLFKSSSKIDEGLEAIVSDGGETEA---EAAPVDADQPGAASDVMPDGQTPEPAPMPE--PT--PAPMPEPT-APATIPEPTPQPADPEPAPEPEQVPPEPAPDEVPPLRQAMTPVAPALDE--A-EAAPAKPGLLGRLMGRSTAQPVVRRALDDEMLEQLEELLISADMGVDTALRVTANMAEGRFGKKLSVAEIKQLLASEVSRIMEPVARPLPIYSKTPQVVLVVGVNGSGKTTTIGKLASQFRAAGKKVVIAAGDTFRAAAVEQLQVWGERAGVPVLTAAQGSDPASLAFDAMGRAQEEGADLLLIDTAGRLQNRGDLMEELAKIVRVIRKKDETAPHNTLLVLDATTGQNALNQVKVFQEISDVSGLVMTKLDGTAKGGVLVALADRFGLPIHAVGLGEQIDDLSPFDPDEFAAALVGTDAMTLDHWGEIKERLLKTVGQNNYTTWIEPLVPGDVEDGIVTLKVPTNFFGNYVSQNFSDLILHEINAAGTDATRLNFALNQQPANAADKPAPAARQTTAAAKP--AANSQLSTAPLDPRFSFDNFVVGKPNELAHAAARRVAEGGPVTFNPLFLYGGVGLGKTHLMHAIARELHERKPEMNVLYLSAEQFMYRFVQALRDRKMMDFKEIFRSVDVLMVDDVQFIAGKDSTQEEFFHTFNALVDQHKQIIISADRAPGEIKDLEDRVKSRLQCGLIVDLHPTDYELRLGILQSKVEVQRKTYPDLEVADGVLEFLAHRITSNVRVLEGALTRLFAFASLVGREIDMGLTQDCLADVLRASERKISVEEIQRKVSDHYNIRLSDMIGPKRLRSYARPRQVAMYLCKQMTSRSLPEIGRRFGGRDHTTVMHGVRRIEELKVSDGQIAEDLELLRRALESMLGIGTIAKKVFGTPNDRKIKATRPLVARINALEPEFEKLSDEEIKARTEELAKRANAGESLDDLLPEAFANCREAARRTLGLRAFDTQLLGGIFLHQGNIAEQKTGEGKTLTATFAAYLNGLTHKGVHVVTVNEYLAKRDAEWMSKVFGALGLTTGVAYSGMPEDQKRAAYACDITYATNNELGFDYLRDNMKSNLSDMLQHGHNFAIVDEVDSILIDEARTPLIISGPSQDRSEMYQIIDTLIPSLTEEHYELDEKTRNVTFTDEGNEFLEEQLRARDLIEEGMTLYDPESTTIVHHVNQGLRAHKLFQRDKDYIVRDGAVTLIDEFTGRMMPGRRLSDGLHQAIEAKEGVDIQPENVTLASVTFQNYFRLYDKLGGMTGTALTEAEEFAEIYGLGVVEVPTNVPIARVDEDDAVYRTAREKYEAMIEKVKEAHAKGQPCLVGTTSIEKSEQLSAMLTADGIAHNVLNARQHEQEAQIIADAGKLGAVTIATNMAGRGTDIQLGGNVELKVLDALDADPEADPANIRAQIEAQHAEEKQKVLEAGGLYVLASERHESRRIDNQLRGRSGRQGDPGRTSFFLSLEDDLMRIFGSERLEKVLTTLGLKEGEAIVHPWVNKSLERAQAKVEGRNFDIRKQLLKFDDVMNEQRKVIFGQRREIMEAENLNEIVTDMREQVIDDLIDTYMPPKTYADQWDTQGFYAAVIEQLNVDVPIIAWCEEDGVDDEVIRERLMKATDELMAKKAEAFGEENMRNIEKQLLLQAIDTKWRDHLLTLEHLRSVVGFRSYAQRDPLNEYKNEAFQLFETMLDSLRQDVTQKLGQIQPMSEEQRREMMQEMADRQAAMQAAATEAADEA--SEQAEA-AAPGFDENDPSTWGNPGRNDLCPCGSGKKFKHCHGEIKMKLSGDLKAFEARIGHHFGKPELLVRAVTHASMSSANRDDNQRLEFLGDRVLGLVMAEALLALDPGATEGQLAPRFNALVRKETCADVAREIDLGKVLKLGRSEMISGGRRKQALLGDAIEAVIAAVYLDGGFDAAKDLVLRLWGNRLKTVKEDARDAKTALQEWAQARGLTPPRYVQTDRSGPDHAPVFTITARLDNGAEAAATAPSKRAAEQAAATTLLRQLEKNSMAKLGKRTRAAREAFAGKEEITVEEAVSLIKANANAKFDETIEIAMNLGVDPRHADQMVRGVVGLPNGTGKTMRVAVFARGAKAEEAEKAGADIVGAEDLMETVQSGKIDFDRCIATPDMMPIVGRLGKVLGPRNLMPNPKVGTVTMDVADAVKAAKGGEVQFKAEKGGVVHAGVGKLSFDEAKLAENIRAFVGAVSKAKPAGAKGTYMKKINLSSTMGPGVSVAVENATAEMSDEFMLDTDDLERRMNGAIASLRTEFASLRTGRGSASMLEPVMVEAYGQMTPVNQVGTVNVPEPRMVTINVWDKGLVGKVEKAIRESGLGINPQLNGTIIMLPIPELNEERRTQLTKVAGQYAEHARVSIRNIRRDGMDQIKKAKNDGMSEDDQKIWEGEVQDLTNRFITLIDDQLETKQAEIMQVMAKKLVGTMKLQVKAGQANPSPPVGPALGQRGINIMEFCKAFNAKTADMEPGAPCPTVISYYQDKSFTMDIKTPPASYYLKKAAKVNSGAKTPSRETVGTVTTKQLREIAEAKAADLSANDVEAAMKIILGSARSMGIEVKMFENLSERLSGVFDRLTKQGALSDEDVKTALREVRVALLEADVSLPVARDFVKAVQDKATGQAVTKSVTPGQQVVKIVHDALIDTLKGEGEPGALKIDSPPAPILMVGLQGGGKTTTTAKLAKRLKERDGKRVLMASLDVNRPAAMEQLAILGTQIGVDTLPIVKGESPVQIAKRAKTQAGLGGYDVYMLDTAGRLSIDEELMQQVKAVRDVANPRETLLVVDGLTGQDAVHTAENFDERIGITGVVLTRMDGDGRGGAALSMRAVTGKPIKFVGLGEKMDALETFEPERIAGRILGMGDIVALVEKAQETIEAEQAEKMMKRMAKGQFNMNDLKMQLEQMIKMGGMQGMMGMMPGMGKMAKQVEDAGFDDKILKQQIALIQSMTKKERANPALLQASRKKRIAKGAGMEVSDLNKLMKMHRQMSDMMKKMGKMGKGGMLKQAMKGMMGKGGMDPS----QMDPKALEAAAKQMGGKLPGGLGGMGGGMGLPGGLSGFGKKKMIPMHRLAQISQRFQFLEASMSAGSDGADFSALAKEYSDLKPVVDQIDLYQQLQRDLEEAELMLKDPEMAELAREELPRLKARLPEVEQGLQLALLPRDSADAKPAMLEIRPGTGGDEAALFAGDLLRMYQRYAEARGWGFDLIESQMTELGGVKEVVAHITGQNVFARLKFESGVHRVQRVPSTESGGRIHTSAATVAVLPEAEDVDIEINANDLRIDTMRSSGAGGQHVNTTDSAVRITHIPTGIVVTSSEKSQHRNRDKAMQVLKARLYDMERSRIDSARSADRAAQVGTGDRSERIRTYNFPQGRMTDHRINLTLYRLEAVMQGDLDEIVDALTADAQARQLSEMEGMDRAQKEQLVDELGQIFESSGVVVVSHYVGLTVAEMQDLRARARAAGGAVRVAKNRLAKIALEGKPCESIADLLTGMTVLTYSEDPVAAAKVAQEFSKENPKLVILGGSMGENALDAAGVEAVSKMPSREELISTIAGMLGAPASNIAGAIGAPASNIASILSTIEDKAAAMKTFSATPADIDKKWIIIDAEGVVLGRLASIIAMRLRGKHKPSFTPHMDCGDNVIVINAEKVQMTGKKREEHFYWHTGHPGGIKSRTKEQILEGAHPERVVTQAVKRMLPGNRLSRQIMTNLRVYAGSDHPHEAQSPEVLDVKSMNKKNTRSAMTAADAALAEGLLAGLGGALPPRLGVAVSGGGDSMALLSLLHGLCKVAGTHLEVVTVDHGLRPEAAAEADLVARCAGDLGLHHETLQWRGWDGQGNLQNAARHARYALMADWAARRDLPCIALGHTADDQAETVLMRLARRAGVDGLAAMAPQSQRQGVTWLRPLLSARREALRDYLRRAGLEWVDDPSNDDPRYTRIQTRQTLAALAPLGLDVETLAEVASNMARARDALDQQADHAAGNILRMEAGAWVIRADAFFAEPEEIRRRLMIRALGQISGGAYPPRRGPVAALIAGLANGQGATLDGCQTLLRRGEIWVFREYNAVRNLHVPADHLWDGRWRAVPSTVF-MGAELRALGPEGLAYCPDWRSFGRPRAMLLSTPAVWQGGRLLAAPLAGLDEKWHVLLERDAGWPKTTPLSHMADLKKLAEDIVGLTLLEAQELKTILKDEYGIEPAAGGAVMMAGPADGGAAEEEKTEFDVVLKNAGASKINVIKEVRGITGLGLKEAKDLVEAGGKIKEGVDKAEAEDIKGKLEAAGAEVELAMSRIGKKPVAMPSGVSAEVSGQTIEVKGPKGTRTFRATDDVTLTVEDSAITVTPRGKSKRARQQWGMSRTMIENLVTGVTTGFKKELEIQGVGYRAAINGNTLRLNLGLSHDVDYVAPEGVTVTAPKQTEIVVEGIDEQLVGQVAANIRAWRKPEPYKGKGIRYKGEFVFRKEGKKKMQVILLERVAKLGQMGEVVDVKSGYARNYLLPQGKALSASKANVEAFEGQKAQLEAQNLETKKEAEAMAEKLNGQQFVVIRSASDAGALYGSVTTRDAAEAATEEGFSVDRKQVVLGQPIKYLGVHEVQVVLHPEVTATIELNVARSPEEAELQASGKSIQELAAEEEAAAEFEISELFDDIGSAADE-DGDSDVVRTPEGDAQDDSNS--MSDSDGKKTLGLRGGARPGNVKQSFSHGRTKNVVVETKRKRVVVPKPGGQKPTGPGAGPIGDPKKRPAGITDAEMERRLKAVQAAKAREVEEAAAREAEEKARAEERERRRAEIEAKEREEREREESLKAKAEEDARRKAEAEA---AAAAPAPEPAAAREPGNKPMPAATPRKTERDRDETKKRSKGGDS-RRSGKLTVNQALNGGEGGRQRSMAQMKRKQERARQKAMGGQVEREKIVRNVNLPPAIVVSELANRMAEKTGAVVKALMQNGMMVTQNETIDADTAELIIEEFGHKVVRVSDSDVEDVIKEIVDKPEDLQGRPPVITIMGHVDHGKTSLLDAIRNAKVVAGEAGGITQHIGAYQVTTDNGAVLSFLDTPGHAAFTSMRSRGAQVTDIVVLVVAADDAVMPQTIEAIAHAKAAKVPMIVAINKIDKPAANPDKVRTDLLQHEVIVEKMSGDVQDVEVSAATGQGLDELLEAIALQSEILELKANPDRAAVGAVIEAQLDVGRGPVATVLVQNGTLRQGDIFVVGEQYGKVRALINDQGERVKEAGPSVPVEVLGINGTPEAGDVLNVTETEAQAREIAEYRANAAKDKRAAAGAATTLEQLMANAKANEDVSELPILVKADVQGSAEAIVQAMEKIGNDEVRVRVLHSGVGAITETDVGLAEASGAPIMGFNVRANASARNTANQKGVEIRYYSVIYDLVDDVKAAASGLLSAEIKENFIGYANIKEVFKVTGVGKVAGCLVTEGVARRSAGVRLLRDNVVIHEGTLKTLKRFKDEVPEVQSGQECGMAFENYDDIRPDDVIEIFEREEVTRTLAMAKPKKTPRPKAQTPKGFRDYFGTEVTHRAEMLSKIAAVYHRYGFDALESSGVETVEALGKFLPDVDRPNEGVFAWQEDSEGDKPGDWLALRYDLTAPLARVYAQHRNDLPMPYRRYAMGPVWRNEKPGPGRFRQFYQCDADTVGAPSVAADAEICAMLADCLEEVGIARGDYVVRVNNRKVLNGVLEVAGLAGDDKDIERGIVLRAIDKLDRLGPEGVRALLGDGRKDDSGDFTKGAGLADAQADVVMGFMQAKRDSGAETVARLRELVAGSDVGVQGVDELELISDLLAAGGYGPDRVEIDPSVVRGLGYYTGPVFEAELTFEIKDEKGRARNFGSVAGGGRYDDLVKRFTGQEVPATGVSIGVDRLLAALAAKGRLEAEATGPVVVTVMDRDRMADYQAMVAELRQAGIRAEVYLGNPKNFGNQMKYADKRQSPVVVIEGGDEKDRGVVQIKDMVLGAQLAQEASHDEWKERKNQYEVKRSDLVQEVRNILDRTNMDDLKQKYLSQIADAGDESALEDIRLAAVGKKGEVALKMRELGKMTPEERQTAGPALNALKDEINSALAAKKAALGDAALDDRLRSEWLDVTLPTRHQRQGSIHPVSQVTEELTAIFAEMGFSVAEGPRIDTDWYNFDALNIPGHHPARAEMDTFYMARAEGDERAPHVLRTHTSPVQIRTMEAEGAPLRIICPGGVYRADYDQTHTPMFHQVEGLAIDKDISMANLKWTLEEFFAAFFEIDGIKTRFRASHFPFTEPSAEVDIQCSWVDGQLRIGEGDGWLEVLGSGMVHPKVLQAGGIDPNEWQGFAFGMGIDRIAMLKYGIPDLRAFFDSDLRWLRHYGFASLDQPNLHGGLSRMEMADEAGLDLVEISPNANPPVCKIMDFGKFKYEQQKRESEARKKQKIIEIKEVKFRPNTDTNDYDVKMRNVFKFLEGGDKVKVTLRFRGREMAHQNLGRELLERVAADTKEIGRVENFPKMEGRQMVMVIGPLPNMKFTLSWLKDHLDTTASIDEITYALTDLGLEVEGVENPAAKLADFTLGYVQSAEKHPDADRLNVCQVETDEGVMQIICGAPNARPGITVVVAKPGVYVPGIDTTIGVGKIRGVESFGMMASEREMELSEEHDGIIELPSGKPGDRFIDWLAENDPAKVDPVIEIAITPNRPDALGVRGIARDLAARGLGKLKPRDCDAVEGSFASPISVSIDDDTLDGCPVFYGRVIRGVKNGPSPQWLQDCLRAIGLRPISFLVDVTNFFTFDRNRPLHVFDADKVKGNLRVHRAKGGEEIVALDEKTYTLQAGQMVISDDSGVESIAGIMGGEATGVTEDTVNVFVESAYWDPVQIAYAGRALKINSDARYRFERGVDPAWTPYGIEHATRMILDHAGGEASEVVVAGKVPDTSRAYKLDAAKVQSLVGMTIPESDQRQTLTALGFQLDGDMAQVPSWRPDVQGEADLVEEVARIASLTKLEGKPLPRLTTGVPRPVLSPMQRRVVTARRTAAALGYNECVTYSFIDQASAALFGGGTDETRLENPISSDMSHMRPDLLPGLLQTAARNQARGFADMALFEVGPAFSGGEPGEEQIMVSGLLVGRTGPRDVHGAARAVDVFDAKADAEAVLAAIGAPAKVQVLRGAADWWHPGRHGKICLGPKKVLGVFGEVHPRVLAAMDVKGPAMAFTIWPTEVPLPRKSGATRPALQISDLQAVERDFAFVVDADVEALTLVNAAKGADKSLIEDVRVFDEFIGGSVGEGKKSLAITVRLQPSDKTLKDADIEAVGAKVVEKVTKATGGLLRGMHAYRSHTCADLSLENKGDNVRLSGWVHRVRDHGGVLFLDLRDHYGITQVICDPDSPAFAEMEKVRAEWCVRIDGTVKARDESLVNPKLPTGAIEVYAREIEVLGSAAELPLQVFGDQEYPEETRLRYRYLDLRREKMQKNMTLRSDVVTSIRKRMWDQNFREFQTPIITASSPEGARDFLVPSRLHPGKFYALPQAPQQFKQLLMVSGFDKYFQIAPCFRDEDPRADRSPTDFYQLDMEMSFVTQQDVFDTIQPVIAGIFEEFGGGKKVDETWEQISYKDAALWYGSDKPDLRNPIKMQVVSDHFRDSGFAIFAKLLEQDGTEIRAIPAPTGGSRKFCDRMNAFAQKEGLPGMGYIFWRDQG---NGMEAAGPLAKNIGPERTEAIRQQLNLGVGDAAFFLGGKPKSFEAVAGRARNVIGEELNLTDKERFAFAWIVDFPIYERDEETGKIDFEHNPFSMPQGGMEALQGDPLKVLGYQYDLACNGYELVSGAIRNHKPEIMFKAFEIAGYGEDEVRKRFGGMVNAFQYGAPPHGGCAAGIDRIVMLLAEEANIREVILFPMNQRAEDLMMNAPSEPTSDQLMELGLRVIPQEMTPLENIRNFSIVAHIDHGKSTLADRLIQLTGTVAERDMQSQLLDNMDIERERGITIKANTVRIEYPAKDGKTYVLNLIDTPGHVDFAYEVARSMHAVEGSLLVVDATQGVEAQTLANVYTAIDADHEIVPVLNKVDLPASDPDRVREQIEDVIGIDASEACLISAKTGVGIPDVLEAIVNKLPAPGGDPDAPLKAMLVDSKYDQYLGVICIVRIIDGTLKKGDRIRMMKTGGTYDVDDVGVYRPKMTGVESLGPGEIGYLNASIKQVRDTRVGDTITHEKRKCETPLPGFKPSVPVVFCGLFPVDANDFEDMRDAIEKLALNDASFTYEMETSAALGFGFRCGFLGLLHLEVIRDRLEREYDIDLITTAPSVIYHVHMKDGTMQELHNPADMPDMTLVDHMQEPRIKATILVPDEYLGDVLKLCQDRRGIQEDLTYAGSRAMVVYDLPLNEVVFDFYDRLKSVTKGYASFDYQMIGYRQDNLVKMQILVNDEPVDALSTMVHRDRAEMRGRAMVEKLKDLIPRHMFKIPIQAAIGGKVIARETLSAMRKDVTAKCYGGDATRKKKLLEKQKAGKKKMRQFGKVDIPQEAFISALKMDSMSLPPGFLEELRDRASLSQVVGRKVIWDNRKSNQGKGDMWAPCPFHQEKSASFHVDDRKGFYYCFGCHAKGDAISFVRETENVSFMEAVEIIAREVGMPVPKQDPRAQAKADKRTQLAEVMEQAVQWFRLQLRTGAAGAARDYLAKRGLSEQAQAHWEIGFAPNSWQGLWDALKSKGVADELILAAGLAKPSSKGGKPYDTFRGRIMFPIRDARGRAIAFGGRAMDPEDKAKYLNSPETELFDKGRSLYNVKDARAAAGRGQPLIVAEGYMDVIALHGAGFEGAVAPLGTAITENQLQMLWRIAPEPIITLDGDAAGQRAALRLIDLALPLLEAGQSLRFAVMPEGQDPDDLLRAQGAGALQKLLDNALPMVRLLWQRETEGKVFDSPERKAALDKSLREKIKLIRDPSIRSHYGQEIKDLRWDLFRPQKKRPKGPGK--GKGGKGSWG-APLAPLASTKASALVAMSDEQVGMHLREAVILVALVDCPQMIETFETGLEGMGCADPDHARLRDLLLRFGHAGEDVLREEISYSLGWETLENMKGQRHVAITPCIRNPGNVEMTRLTVAEELAKLDAARGLNEEIAEAVEDLSGPADEGVTWRLSEAARAADMARRSAQEDNGEFDVGDNGMTMDRDERSALDALLGTIKYEKSKGRGMCAETPDYKATLNLPKTDFPMRAGLPKREPAWLERWEKIGVYDRLREKEGREPFTLHDGPPYANGNLHIGHALNKTIKDMIVRSHQMMGYDARYIPGWDCHGLPIEWKIEEQYRKKGRDKDQVPINEFRAECREFARGWVDVQREEFKRLGITGNWENPYLTMDFHAERVIAEEFMKFLMNGTLYQGSKPVMWSPVEKTALAEAEVEYHDKESHTVWVKFKVV--ETGDRTLDGANVVIWTTTPWTMPSNKAVVYGESISYGLYEITGRPEECWVSIGDRYLLADDLAADVFARARLDEGMYRRLCDVTQEDLAKIKLQHPLAGAEGANGEWDDLRDFRAADFVTSDEGTGFVHCAPSHGLEEYELYRDLGMLPQVITYNVMEDGRFRDDLPFFGGKAILKPNGKEGNANSAIIDKLVEVGGLLARGKIKHSYPHSWRSKAPVIYRNTPQWFAAIDKEVGDGLDQNGKTIRERALTCIDKVNWVPKSGRNRLHSMMEARPDWVLSRQRAWGVPLTCFVRRGVAPTDENFLLRNEAVNQRITEAFETEGADAWYAEGAKERFLEGIVDPAEFDQVTDILDVWFDSGSTHAFTLRDREDGTEDGIADVYMEGTDQHRGWFHSSLLQSVGTTGRAPYRNVVTHGFTLDAKGMKMSKSIGNTIVPEKIVQQYGADILRLWVAQTDYTADQRIGDEILKGVADSYRRLRNTMRYMLGALNDFSEADRVDPADMPELERWVLHRVAELDKVVRDGFARFDFQGVFQAVFTFATVDLSAFYFDIRKDALYCDGDTLRRRAARTVLDILFHRLTTWLAPVLVFTMEEVWLERFPGEESSVHLVDMPETPEAWLNPELAAKWSKVRAARRVVTAALEVQRTEKVIGASLEAAPVVHVDDAAQREALESVSFEDVAITSDITVTGDAAPAEAFRMPEAQGVAVVFEKAEGAKCERCWKVLPDVGTHEHPGVCGRCDEAVRAAPSQNIRIRLKAFDYRVLDSSTQEIVSTAKRTGASVRGPIPLPNKIEKFTVLRGPHVDKKSRDQFEIRTHKRLLDIIDPTPQTVDALMKLDLAAGVDVEIKLQSMADEIKTLEGLEAAVTGGVQGTE----TEMTPREPVRDELGRAYATGKRKDAVARVWIKPGSGKVIVNGKPQNEYFARPVQQLILAQPFGITNTEGQFDVFATVKGGGLSGQAGAVKHGISKALQLYDPSLRGALKAAGFLTRDSRVVERKKYGKAKARRSFQFSKRMPTIQQLIRKPRQPKVKRSKSMHLQECPQKRGVCTRVYTTTPKKPNSAMRKVAKVRLTNGFEVISYIPGESHNLQEHSVVLIRGGRVKDLPGVRYHILRGVLDTQGVKDRKQRRSKYGAKRPKMARDKTKTKRKVSKNIAAGVAHVNSSFNNTKILISDVQGNAISWSSAGTMGFKGSRKSTPYAAQMAAEDAGRKAQDHGVKTLEVEVQGPGSGRESALRALAAAGFNITSIRDVTPMAHNGCRPPKRRRVTKPNRRGLLIILSSPSGAGKSTMAHALRAWDPTINFSVSATTRAPRPGEEDGTDYRFVGEEDFRQAVAEGEMLEHAHVFGNFYGSPKAPVQAAIDQGQDILFDIDWQGAQQIRNSDLNTHTLSIFLLPPSITELKRRLESRGQDDAETIAKRMGKSWDEISHWDGYDFVLVNDDLDQTEARLKSIITAARLRLSQQPAIKDHVRRLQSEFEDLKMAQSFLGQKRLRKYYGKIREVLEMPNLIEVQKSSYDLFLNSGDAETPTDGEGITGVFQSVFPIKDFNETSVLEYVKYELEKPKYDVEECQQRDMTYSAPLKVTLRLIVFDVDEDTGAKSVKDIKEQDVFMGDMPLMTPNGTFVVNGTERVIVSQMHRSPGVFFDHDKGKTHSSGKLLFACRIIPYRGSWLDFEFDAKDIVFARIDRRRKLPVTTLLYALGLDQEAIMNAYYKTVTYTLEKNKGWVAPFFPDRVRGTRPTYDLVDAATGEILFEATKKVTPRAVKKLLDEGKVKDLLLPFDHIVGKFVARDIINEETGAIYVEAGDELTLEYDKDGTLIGGTAKELIDAGITEIPLLDIDNVNVGPYMRNTMAMDKNMNRDTALMDIYRVMRPGEPPTVEAASALFDTLFFDSERYDLSAVGRVKMNMRLALDKPDTQRTLDRDDIVACIKALVDLRDGRGDIDDIDHLGNRRVRSVGELMENQYRVGLLRMERAIKERMSSVEIDTVMPQDLINAKPAAAAVREFFGSSQLSQFMDQTNPLSEVTHKRRLSALGPGGLTRERAGFEVRDVHPTHYGRMCPIETPEGPNIGLINSLATFARVNKYGFIETPYRVVKDSTVTDEVHYMSATEEMRHTVAQANANLDENMKFVNELVSTRQSGDYTLAPTENVDLIDVSPKQLVSVAASLIPFLENDDANRALMGSNMQRQAVPLLQAEAPLVGTGIEEVVARDSGAAYTARRAGIIDQVDASRIVIRATEDLELGDAGVDIYRMRKFQRSNQNTCINQRPLVKVGEKVTKGQVIADGPSTDMGELALGKNVVVAFMPWNGYNYEDSILISERISRDDVFTSIHIEEFEVAARDTKLGPEEITRDIPNVGEEALRNLDEAGIVYIGADVEPGDILVGKITPKGESPMTPEEKLLRAIFGEKASDVRDTSLRVKPGDFGTVVEVRVFNRHGVEKDERALQIEREEVERLARDRDDELAILDRNIYARLKDMILGKIAVKGPKGVKANSQITEELLETLTRGQWWQLALEDEDDAKIVEALNEQYEIQKRTLDARFEDKVEKVRRGDDLPPGVMKMVKVFVAVKRKLQPGDKMAGRHGNKGVISKVVPMEDMPFLADGTPVDFCLNPLGVPSRMNVGQILETHMGWAARGLGINIDEALQEYKRSGDLTPVREAMQLAYGDDVYEEGITGMDEDTLLEVADNVRRGVPIATPVFDGAKEADVNDSLKRAGFDTSGQSVLFDGRTGEQFARPVTVGVKYLLKLHHLVDDKIHARSTGPYSLVTQQPLGGKAQFGGQRFGEMEVWALEAYGAAYTLQEMLTVKSDDVAGRTKVYESIVKGEDNFEAGIPESFNVLVKEVRGLGLNMELLDAEEDEMPLYEHVMIARQDLSNTQAEGLIEHFGTVLADNDGKLVDSEYWGVKTMAYKINKNRKGHYAFLRSDAPATAVQEMERLMRLHDDVMRVLTIKVDEHKELPSVQMQKRDERPDRRERRMTSQIEVAALDKAQAETELARLAELLSAANTAYHTEDAPEISDAEYDALKRRNAAIEQRFPELKRSDSPSEQVGAPVADGFGKVRHAVSMLSLANAFDAEDVTEFDARIRKYLGLGAKAPLAYTAEPKIDGLSLSLRYENGVLKQAATRGDGSVGENVTANARTIADIPHELKNAPDLLEVRGEVYMSHADFAALNARQAETGGKTFANPRNAAAGSLRQLDAEITRARPLRFFAYAWGALSTPLAETQKGAIDRLAELGFSTNPLTALCDGPNDMVAHYEQIEAQRATLGYDIDGVVYKVDDLALQERLGFRSTTPRWAIAHKFAAELAWTRLEGIDIQVGRTGALSPVARLQPVTVGGVVVSNATLHNEDYIKGLDSKGAEIRGGKDVRVGDWVQIYRAGDVIPKVADVDLSKRPEDAAPFVFPTTCPECGSDAIREPGDAVRRCTGGLICPAQAVEKLKHFVARGAFDIEGLGAKQVEQFYHDGWIAEPADIFTLKERYGSGVQQLKNREGWGPKSADNLFQAIEDKREIPMARLIFALGIRHVGEAASNLIALHYGDWDSFEAAMAEARGLEGPAWDDLIGVDGVGSVMAGSLVSAFAQEAERASIDRLVAHLTVVPAERPDTEGSPVAGKTVVFTGTLEKMSRAEAKARAERLGAKVSGSVSAKTDILVAGPGAGSKAKKAADLGIETLDEDGWLALIEGKMIHKNWAELIKPQQLDVKPGNDPARQATVMAEPLERGFGLTLGNALRRVLMSSLQGAAITSVQIDNVLHEFSSVAGVREDVTDIILNLKGVSLRMEVEGPKRLSISAKGPGVVTAGDISESAGIEILNREHVICHLDDGADVYMELTVNTGKGYVSADKNKPEDAPIGLIPIDAIYSPVKKVSYDVQPTREGQVLDYDKLTMKVETDGSITPDDAVAFAARILQDQLGIFVNFDEPESASRQDDDDGLEFNPLLLKKVDELELSVRSANCLKNDNIVYIGDLIQKTEAEMLRTPNFGRKSLNEIKEVLSGMGLHLGMDVEDWPPENIEDLAKKFEDSFMARDDNRGGNRRNQRDETPEFADRLVAINRVSKTVKGGKRFGFAALVVVGDQKGRVGFGKGKAKEVPEAIRKATEQAKRQMIRVQLREGRTLHHDMHGRHGAGKVIMRTAPEGTGIIAGGPMRAVFEMLGVKDVVSKSVGSQNPYNMIRATLDGLRKEQSPRSVAQRRGKKVADILPKRDDNVESSAQVAEEAMNDPIADMLTRIRNSSLRGKSTVSTPASKLRAWVLDVLADEGYIRGYEKVTGADGHPAIEISLKYYEGEPVIRELKRVSKPGRRVYMAVNDIPVVRQGLGVSIVSTSKGVMSDASARSANVGGEVLCTVFMHDIRAIRDNPEAFDAALARRGEAAMSEAVLSLDAARRAKIAAAETAKAEQNKASKEVGAAKAKGDEAEFDRLRALVSDKKAEVAAMNTEAQELDAKLTDMLARIPNSPADDVPQGANEEDNVEVKVWGDKPSFDFTPVEHYEIASVKPGMDFETASKTSGARFVMLKGGVARIHRALAQFMIDTHVDENGLTEYNTPVLVNNAAMYGTDKLPKFGGDSFDTQEDYWLIPTSEVTLTYSIAGETLEERSLPMRMTAHTLCFRSEAGSAGRDTAGMLRQHQFEKVEMVSITHPDESDAEQQRMVGCAEGILEKLGVPYRTVILCTGDMGFGARRTYDIEAWVPGQNCYREISSVSTTGDFQARRMNARFKPGGGGKPQFVHTLNGSGLAVGRCLIAVLENGQQADGSVILPEVLAPYLGGKTTLTAEGVLAMAITASMVKELRDSTGAGMMDAKKALTESNGDMEAAVDWLRTKGLAKAAKKSGRTAAEGLVAVKVEGGHGVAVEVNSETDFVGKNAEFQSMVSNIADAALKVDDVEALKAAEINGKTVETTLTDAIAKIGENMSLRRMQSIDGETVVSYVHNAAAPGMGKIGVLVAMNGGNEEFGKQVAMHIAAVNPASLSEADLDPAVVEKEKQVQIDIARESGKPEAVIEKMIVGRMQKYMSEVTLLNQSFVVNPDLSVGKAAEEVGATITGFVRLEVGEGIEVVKEDFAAEVAKAAKGMNQELTNNPFNPLTPQKAFDEIKVSLASPERILSWSFGEIKKPETINYRTFKPERDGLFCARIFGPIKDYECLCGKYKRMKYRGVVCEKCGVEVTLQKVRRERMGHIELASPVAHIWFLKSLPSRIGLMLDMTLRDLERVLYFENYVVIEPGLTDLQYGQMMTEEEYMDAQDAYGMDAFTANIGAEAIREMLAAIDLEAEAETLRADLKEATGELKPKKIIKRLKVVESFLESGNRPEWMVMTVIPVIPPELRPLVPLDGGRFATSDLNDLYRRVINRNNRLKRLIELRAPDIIVRNEKRMLQESVDALFDNGRRGRVITGANKRPLKSLSDMLKGKQGRFRQNLLGKRVDFSGRSVIVTGPELKLHQCGLPKKMALELFKPFIYSRLEAKGLSSTVKQAKKLVEKERPEVWDILDEVIREHPVMLNRAPTLHRLGIQAFEPVLIEGKAIQLHPLVCSAFNADFDGDQMAVHVPLSLEAQLEARVLMMSTNNVLSPANGAPIIVPSQDMILGLYYTTLERQGMVGEGMVFGSVDEVQHALDAGAVHLHSKIKARIKQIDAEGNEVMMRFDTTPGRVRLGALLPLNAKAPFDLVNRLLRKKEVQQVIDTVYRYCGQKESVIFCDQIMTMGFREAFKAGISFGKDDMLIPDTKWPLVEETREQVKDFEQQYMDGLITQGEKYNKVVDAWSKCNDKVTEAMMGSISATTYHENGSEKEPNSVYMMAHSGARGSVTQMKQLGGMRGLMAKPNGDIIETPIISNFKEGLTVLEYFNSTHGARKGLSDTALKTANSGYLTRRLVDVAQDCIVRMHDCGTETAITAEAAVNDGEVVSSLAERLLGRVAADDILAPGTEEVIVPAGGLIDERMADAIDAAAVQVARIRSPLTCEAEEGVCAMCYGRDLARGTLVNQGEAVGIIAAQSIGEPGTQLTMRTFHIGGVAQGGQQSFQEASQSGKIVFENAQTLENSSGEILVMGRNMKLSIVDESGDERSSHKVGYGTKLFVKDGDTIARGDKLFEWDPYTLPIIAEKPGMAKYVDLVSGIAVKDDTDDATGMTQKIVIDWRAAPKGNELKPEIILVGDDGEPVRNDAGNPVTYPMSVDAVLSVEDQTEIQAGDIIARIPREGAKTKDITGGLPRVAELFEARRPKDHAIIAEIDGYVRFGKDYKNKRRIAIESSDDPDVKVEYMVPKGKHIPVAEGDFVQKGDYIMDGNPAPHDILAIMGVEALAEYMIDEVQDVYRLQGVKINDKHIEVIVRQMLQKWEIQESGDTTLLKGEHVDKQEFDTANEKALKKGGRPAKGEPILLGITKASLQTRSFISAASFQETTRVLTEASVQGKRDKLVGLKENVIVGRLIPAGTGGATQQMRKVATDRDNVVIEARREEAEAAAALAAPTASTDDVVGGDVFNTPVGDDESRDMSRRHAAEKREVLPDAKYGDLVLTKFMNNLMIDGKKSVAERIVYNAMTRVEDKIKRAPIEVFHEALENIQPSVEVRSRRVGGATYQVPVEVRPERRQALAIRWLIKAARARNENTMEERLAGELMDAVQSRGTAVKKREDTHKMADANKAFSHYRWMPYAHSDKSEGMPMLANPAPDVRSRPKLEGGHKFKLVTEFAPAGDQPTAIKELTEGVNSGERDQVLLGATGTGKTFTMAKVIEETQRPAIILAPNKTLAAQLYGEFKGFFPDNAVEYFVSYYDYYQPEAYVARSDTFIEKESQINEQIDRMRHSATRALLERDDVIIVASVSCIYGIGSVETYGAMTQDLKAGESYDQRKVIADLVAQQYKRNDAAFQRGSFRVRGDSLEIFPAHLDDRAWRLSFFGEELESITEFDPLTGEKTDTFDQIRVYANSHYVTPKPTMSQAIIGIKKELRTRLDQLVADGKLLEAQRLEQRTNFDLEMLEATGVCNGIENYSRYLTGRAPGEPPPTLFEFIPDNAIVFADESHVSVPQIGGMYKGDYRRKFTLAEHGFRLPSCMDNRPLKFEEWDAMRPQSVFVSATPAAWEIEQTGGVFTEQIIRPTGLIDPQIEIRPVEMQVDDLLDEVRKVAADGYRTLCTVLTKRMAEDLTEYMHEQGIRVRYMHSDIDTIERIEILRDLRLGAFDVLIGINLLREGLDIPECGLVAILDADKEGFLRSETSLIQTIGRAARNAEGRVIMYADRITGSMERAMGETDRRRAKQLAYNEEHGITPATVKKNVDDILAGLYKGDVDMNRVTAKVDNPLAGGNLQTVLDGLRVDMRKAAENLEFEEAARLRDEVKRLEAVDLAVADDPMARQQAVDRAVDAAQKASGRSTSGRGGMRGGNVK--RRMAAKPFFRRRKVCPFSGDNAPAIDYKDTRLLQRYISERGKIVPSRITAVSAKKQRELARAIKRARFLALLPYAVKMPKRILTGTVTSDANAQTVSVSVERRFTHPVLKKTIRKSKKYRAHDENNTYKVGDSVRIIECAPKSKTKRWEVLTSDKMANTTQSAKRARQNEKRFAINKARRSRIRTYLRKVEEAITSGDKEAATAALKAAQPELMRGVTKGVFHKNTASRKMSRLAARVKALGMARSVWKGPFVDSYVLKKAEASREGGRNEVIKIWSRRSTILPQFVGLTFGVYNGHKHIPVNVSEDMIGQKFGEYSPTRTYYGHAADKKAKRKMARIAGVNIPTAKRVPIALTYITGIGTSSAKAICEAVGIDATRRVNELSDAEVLAVREHIDANYTVEGDLRRDTQMNIKRLMDLGCYRGLRHRRNLPVRGQRTHTNARTRKGPAKAIAGKKKMAMKIRLARGGSKKRPFYRIVAADSRMPRDGRFIEKLGTYNPLLPKDSEDRVKMDVEKIEAWIAKGAQPTERVVRMLEAAGVREKTERNNPKKGTPGKKAQERVQEKADKAAAAAEAANAPAEEASAE-MSITAEEKAKVMKDFGTKEGDTGSPEVQVAILTSRIVTLTEHFKTHKKDNHGRRGLLKMVATRRKLLDYVKAKDESRYQDLIKRLGLRRMKTLNEIRSTFLNYFDAQGHQIVPSSPLVPRNDPTLMFTAAGMVQFKNLFTGVETRDYSRATSAQKCVRAGGKHNDLDNVGYTARHHTFFEMLGNFSFGDYFKSEAIPFAWDLLTKEFGIDPNRLLVTVYHTDEEAVKIWKAHTGLPDDRIIRIATDDNFWSAGPTGPCGPCTEIFYDHGDHIWGGPPGSPEEDGDRFVEIWNLVFMQYEQFEDGTRQPLPNQSIDTGMGIERVAALLQGTNDNYATDLMRSLIEASAHASSTDPDGPGKTHHRVIADHLRSTSFLIADGVMPSNEGRGYVLRRIMRRAMRHAHLLGVKDPLMHQLVPSLVQQMGAAYPELGQAQSLIRETLLLEETRFRQTLDRGLKLLDDELSSLPEGATLPGEAAFKLYDTYGFPLDLTQDALREKGRAVDTDGFDTAMQAQKAKARAAWAGSGEAADATVWFDVADKSGTTEFLGYDTESAEGQIVALVQGSDKVDSAAVGSDVQVALNQTPFYAESGGQVGDTGVIRTQSGIVNVTDTRKSAGVFVHFGHVVEGEVKPGQTAVLNVDPARRTAIRANHSATHLLHEALRNALGDHVSQRGSLNAHDRLRFDFSHAKGLTQEELSQVEREVNDYIRQNTPVETRIMTPDDARRMGAQALFGEKYGDEVRVVSMGQLEGSGKGSDKSTYSLELCGGTHVRQTGDIGAFVLLGDSASSAGVRRIEALTGTEALAWLREQEAALSRVAAELKTSTSDVPDRVRALLDERRSLSNEVAQLRRELAMS-GGGAAAPEAREVNGVRFVGQVLSGVTGKDLPGLVDEHKAKLGSGAVLLIADTGGKAAVAGGVTKDLTDRLSAVDMVKAAVAELGGKGGGGRPDMAQGGGASAENAEAAIAAAENILKGMGFKMGIVGLPNVGKSTLFNALTRTAAAQAANFPFCTIEPNVGEVAVPDARLDTLAEIAKSKSIIPTRMTFVDIAGLVKGASKGEGLGNQFLANIREVDAIAHVLRCFEDGDVTHVEGRVDPVADAETIDTELMLADIESIEKRLQNIVRKVRGGDKEAVQQERLMRMALEALEAGNPARVVEVDEDDAKAWRMLQLLTTKPVLYVCNVGEAEAAEGNAHSAKVAEMAAAQGNSHVVISAQIEEEISQLEAEEAEMFLEEMGLKEAGLDRLIRAGYELLHLETYFTVGPKEARAWTIKSGTSAPKAAGVIHGDFEKGFIRAETIAYDDFVCLGGEGPAKEAGKMRAEGKSYIVKDGDVLHFLFNTMGWKSLDDMDLNAKRVLLRVDINVPVEDGRVTDATRIERIVPTVNDILSRGGKVTLLAHFGRPKGKVVEEMSLKQVLPALENALGRDVAFVPSLEAAA-----GAQDDLQLMENIRFYPGEEANDAEFAQRLADLGDVYCNDAFSAAHRAHASTEALARLLPACAGRLMQAELSALEAALAKPERPVGAVVGGAKVSTKIALLENLVNRLDVLVIGGGMANTFLAALGADLGKSLEEPDYYSTAKDIMAQADKAGCRVILPVDGLVARDFAKGAAHEVAQLGPDAKLAADQMVLDAGPDTVALVEAAFAGLRTLIWNGPMGAFEIPPFDTATVAAARAAAQQTRDGTLTSVAGGGDTVAALNQAGVADDFTYISTAGGAFLEWMEGKTLPGVAALGGMALPEFSMRQLLEAGVHFGHQTQRWNPRMGPYIYGARNGIHIMDLTQTVPMLDDALKVIRDTVAKGGSVLFVGTKRQAAQPIAEAAEKCAQYYMNHRWLGGTLTNWQTVSQSINRLKSIDEQSERGFEGLTKKERLGMERDQFKLEASLGGIREMGGRPDLLFVIDVKKEALAIAEANKLGIPVVAVVDTNCSPDGIDYIIPGNDDAARAISLYCDLAARAALDGMSAQLGAAGVDLGAMEEAPEEEAMTAESNASEETLHDDAMGKDAESMTKRTAAKHKLDRRMGENIWGRPKSPVNRREYGPGQHGQRRKGKISDFGIQLRAKQKLKGYYGDLTEKQFRRIYGEAERVKGDTGENLIGLLERRLDAVVYRAKFVATVFAARQFVNHGHVRVNGKKVNIPSYRVKEGDVIEVRDRSKQLASVLEAVQLPERDVPDYLETDHSKLTATFVRTPGLSDVPYPVVMEPNLVVEFYAKNMGNKVNPIGMRLQVNRTWDSRWYADTKDYGDLLLEDLAIRDFIKKECHQAGVARVIIERPHKKCRVTIHTARPGVIIGKKGADIETLRQKIAKMTNSELHLNIVEIRKPELDAHLVGESIAQQLERRVSFRRAMKRAVQNAMRMGALGIRVNLAGRLGGAEIARTEWYREGRVPLHTLRADIDYAHVEAATAYGIIGIKTWIFKGEIMEHDPAARDRKAQELQDGPAPRGAGGRRMARKRKGRDISGWLVVDKPAGPTSTAVVNKVRWALEAKKAGHAGTLDPEATGVLAIALGEATKTVPYITDALKAYEFTVRLGIATNTDDAEGEVIGTSDLRPDDAAIKDALSDFIGDIQQVPPQFSAVKIDGQRAYKRARDGEEMDIAARPLWVESLLLLDRPDADHVTLEMVCGKGGYVRSIARDLGQKLGCLGHVRELRRTWSGPFEAANALTLAQIDEIARTPELDTHLLPLAEGLVELPEVKATPEGATRLRNGNPGMVIAHDVEYGDECWASLDGRPVAVGRFKAGELHPSRVFNLSSMVLLGLTGSIGMGKSTTAQMFAEQGCAVWDADAAVHRLYAAGGAAVAPMQAAFPTAIEHGAVSRVALKEIIAADPTALPRIEAIVHPLVAQDRAAFLSSATNDIAVLDIPLLFETGGNAAMDAVVCVTIPDAVQRDRVLVRGTMTEAQFDAIRAKQMPAKEKCARSDYVIVTDTLDHARAQVRDVIRDIREKLRHAMEDFDLVIEDDRWNAVDLEPLAHAAARATLGHLGLNAEAAEMTLLACDDARIAVLNEDFRGKARATNVLSWPAEERGAAAPGGDPLPVSPGIDGMLELGDIALAYETCAAEAKAADKPLAAHVTHLIVHGLLHLLGYDHENDPDATLMEGLEREILGKMGYDDPYRENGPMARYIFITGGVVSSLGKGLASAALGALLQARGFSVRLRKLDPYLNVDPGTMSPFEHGEVFVTDDGAETDLDLGHYERFTGVAARKTDSISSGRVYSTVLEKERRGDYLGKTIQVIPHVTNEIKDFIDIGDDEVDFMLCEIGGTVGDIEGLPFFEAIRQFSQDKPRGQCIFMHLTLLPFVKASGELKTKPTQHSVKELRSIGIAPDILVCRSEGPIPAKEREKLALFCNVRPDSVIAAQDLKSIYEAPLAYHREGLDQAVLDAFQITPAPRPNLSRWEDVADRIYNPEGEVKVAIVGKYTQLEDAYKSIAEALTHGGMANRVKVKVEWVDAEIFDSEDAGPHLEGFHAILVPGGFGERGTEGKIKAAKYAREHKVPYLGICLGMQMAVIEAARNVAGLKTAGSEEFDHEAGKKRFEPVVYHLKEWVQGNHKVARKVDDDKGGTMRLGAYDATLVEGSRVAEAYGTTTIDERHRHRYEVDIAYKEQLEKAGLKFSGMSPDGKLPEIVEWSDHPWFIGVQFHPELKSKPFDPHPLFKDFVRAAKDVSRLVQSQTRLILGLESSCDDTAAAVVRIEEDGRGTVLASVVAGQTELHADFGGVVPEIAARAHAEKLDHCVEDALAGAGITLPQIDAIAVTAGPGLIGGVVSGVMCAKGLSAATGKPLYGVNHLAGHALTPRLTDDVPYPYLMLLVSGGHCQFLLVRGPESFDRLGGTIDDAPGEAFDKVARLLGLPQPGGPSIEKCAQEGDAKRFALPRPLLDREGCDMSFSGLKTAVLRARDKCVAAHGGLTRQDQADLAAGFQAAVVEVLAHKTRRAFAEYPAEGA--RGLCVAGGVAANQSIRAALETVAAEQEARFVAPPLALCTDNAAMIAFAAGEQAVLRAPDDLTLSARPRWPLDTARPSMLGSGKKGAKA------------------------------------------------------------------------------------------------------------------------------------

>'Pelagimonas-variansDSM-23678'

MNAQELMDKTPDQLREDLVQLKKEAFNLRFQAASGALENTARMREVRRSVARVNTILNQKAAAAAAEAMATADLLTM-NKKTADKQKALDSALAQIERQFGKGSIMKLGGENAIRDIEATSTGSLGLDIALGIGGLPKGRIIEIYGPESSGKTTLTLHVVAEEQKKGGVCAFVDAEHALDPQYAKKLGVDLDELLISQPDTGEQALEIVDTLVRSGAVNLVVVDSVAALTPKSELEGDMGDSSVGVQARLMSQAMRKLTGSISRSNCMVVFINQIRMKIGVMFGSPETTTGGNALKFYSSVRLDIRRIGAIKDRDEVVGNQTRVKVVKNKVAPPFKVVEFDIMYGEGISKMGELLDLGVKAGVVEKSGAWYSYGDERIGQGRENAKNYLRENSRISYEIEDKIRAAHGLEFD-----DPDLVEDMSLYDPAKTEPKWQAAWDEAGTFKATMTGDKPKYYVLEMFPYPSGRIHIGHVRNYTMGDVIARYKLATGHNVLHPMGFDAFGMPAENAAMAIGGHPKTWTYENIDTMVGQMKPLGFGLDWSRMFATCDPEYYGQQQALFIDFLAKDLVYRKNAVVNWDPVDMTVLANEQVIDGKGWRSGAEVERRELTQWFFKISDYSEELLDALDTLENWPAKVRLMQENWIGKSRGLEFGFERVD---GEEPITVYTTRPDTLMGASFVGISPDHPIAKKLEAENPEIAAEIAEMRKGGTTAEALEKAEKRGVDTGIRTKHPLDSNWELPVWIANFILMDYGTGAIFGCPAHDQRDYEFASKYELPIVPVF------EEEITEAFVPAKTEKVTFLRGFAGATEQTGDEAVDAAIAAAERDGWGSGVTKFRLRDWGLSRQRYWGCPIPVVHCDDCGVVPEKKENLPIELPDDVTLDIPGNPLDRHPTWRDCACPSCGKPAQRETDTMDTFVDSSWYFARFTAPDAKTPTDMEAASYWMNVDQYIGGIEHAILHLLYSRFFARAMNITGHLPDSAREPFHALFTQGMVTHAIYMTTGEDGRPVYHYPEDVRDGGGFKDGTQVKIIPSAKMSKSKNNVVDPVEIIKAFGADTARWFVLSDSPPERDVEWTASGAEAASKHLNRVWMLCDKIGKMPADYAGRDNEELMREMHKAIHDVTMAIDSFGFNAAIAKLYGFASTIAKSDAGTNEKKLAIKTLAQLMSPMTPHLAEDIWAHQGGEGLIANAPWPVADEKLMVEDSVTLPIQINGKRRGEMKVPRDMPKDEVEKAALAHEAVVKALDGGQPKKLIVVPGRIVNVVVMAITSANQLELLQTAEAVAREKMIDPGLVIEAMEESLARAAKSRYGAEMDIRVSIDRKNGKATFTRVRTVVAEEDLENYQSEFTVEQAKQYMAEPTIGDTYVEEVPPVELGRIAAQSAKQVILQKVREAERDRQFEEFQDRNGTIINGSVKREEYGNVIVDVGRGEGMLRRNEKIGRESYRPGDRIRCFIKDVRRETRGPQIFLSRTAPEFMAELFKMEVPEIYDGIIEIKAVARDPGSRAKIAVISHDGGIDPVGACVGMRGSRVQAVVNELQGEKIDIIPWNEDVPTFLVNALQPAEVTKVVLDEEAERIEVVVPDEQLSLAIGRRGQNVRLASQLTGLDIDIMTEEEESKRRQAEFETRTKLFIETLDVDEFFAQLLVSEGFTNLEEVAYVEKDELLVIDGVDDDTANELQTRAREFLEAQAKKALERAQELGVQDNLIGFEGLTPQMVEALAEDGVLSLEDFATCADWELAGGWTTEDGKRVKDDGLLEKFDLSLEDAQTMVMTARVLLGWVDPTELEPD-----ADGDEGEFESEEDMAHKKAGGSSRNGRDSAGRRLGVKLYGGQAAIPGNIVVRQRGTKFWPGEGVGMGKDHTIFATVEGAVTFRKGLKGRTFISVLPVAEAAEMAKRWYSVSVLSNFEKKIAEQIRTAVEEQELHDQIDEVLVPTEEVIEVRRGKKVTTERRFMPGYVLVHMEMSDQGYHLINSINRVTGFLGPQGRPMPMRDAEVDAMIGRVQENEESPRLEIRFETGEKVKVTDGPFEGFDGMVEGVDDDNQRLRVSVSIFGRETPVELEFTQVTKEMMSFTLAIVGRPNVGKSTLFNRLVGKRLALVDDQPGVTRDLREGDARVGDIRFTVIDTAGLENANDESLPARMRRLTERAVDMADVCLFLVDARAGILADDIVFAEILRRRAGKVILAANKAEGAAADAGVIEAYSLGLGEPIRLSAEHGEGLNDLYSFLLPIEEEFKARADANAPDIEVEVGDDDPD--APRPITADRPLQIAVVGRPNAGKSTLINQIIGEERLLTGPEAGITRDAISVQKEWDG-VHMRIFDTAGMRKRAKVQKKLEKLSVSDGIRAIKFAEVVVVLLDAEIPFEVQDLKIADLAEREGRAVVIAVNKWDIEDEKQEKLRNMREAFERLLPQLRGAPLVTVSARTGKGLDRLQAAIMKAHTVWNRRVSTGELNRWLSDMIERHPPPAPQGKRIKLRYITQAKTRPPGFVVMCSHPDKLPTSYSRYLVNGMREDFDMPGTPIRLVMRGQSDQNPFKGRRKKNAGALKKHLGSLPKAMRVIFMGSPDFSVPVLEALVEAGHEIAAVYCQPPRPAGRGKKDRPTAVHARAVKMGLEVRHPVSLKGDAEREDFAALGADVAVVVAYGLILPQAILDAPELGCLNIHASLLPRWRGAAPIHRAILEGDAETGVCIMQMEAGLDTGPVLLTESLRIGDEETTGALHDRLSEMGARMICKALGQLGNLSPQVQPEDGVTYAAKIDKSEAAVDWSKPAEVISRQIRGLSPFPGVWTLQDGQRIKLLGARLTTGQGNPGEALDDAMTIACGQGAVQITRAQRAGKGAQDTDVFLRGNPVAKGTVLGS-MTDTPEYQVLARKYRPETFVDLVGQDPMVRTLRNAFEADRIAQAFVMTGIRGTGKTTTARIIAKGMNCIGADGNAGPTTDPCGICEHCVAIMEGRHVDVMEMDAASNTGVANIREIIDSVHYRAASARYKVYIIDEVHMLSTGAFNALLKTLEEPPAHVKFIFATTEIRKVPVTVLSRCQRFDLRRIEPEVMIAMLRKIATGENAQITDDALALICRAAEGSARDATSLLDQAISHGAGETTADQIRAMLGLADRGRVMDLFERIMRGDAAGALQELSSQYADGADPLAVLRDLAELTHWVSVVKITPDAAEDPTIAPDERTRGLAFADGLGMRPLSRAWQMLLKAIEEVSNAPSSMMAAEMAVIRLTHVADLPSPEELIRKLQDTPPPTAP--GPQGGGMAQPSHNTSAQGLPAPPGTSGPVAYAGGAAAAVAVDQALAKYPTFEHVLELIRINRDVKLLVDVEGSVRLAAYQPGRIEFAPADNASPDLAARIGGALQRWTGNRWAVSIVSDCNAPTIVERRDAAKLALHAKAEEHPLVQAVIAAFPKAEIVDVRTAEDIAAVALEESLAEVEDEWDPFEEDMPKMKTKSSAKKRFKVTATGKVMGGQAGKRHGMIKRTRKFIRDARGTTTLSAPDAKTVKSFMPYDRMNLFTDIRELVLASINAMQADGALPGELNLAPVTVEPPRDPAHGDMATNAAMVLAKPAKQKPRDITQALAAKLAEDDRIQSAEVAGPGFLNLRLSPALWQKVPAEILKTGTDYGRSDMGAGKKVNVEYVSANPTGPMHVGHARGAIVGDAMASLLDFAGFDVTREYYINDGGAQVDVLARSVYLRYLEAHGQEVEFPEGTYPGDYLVAVGQALKDKVGDAYIDQPEDVWLADVRVYSTDAMMDLIREDLKALGVEMDYFFSEKSLYGTGKIEAAIDSLRDKDLIYRGVLEPPKGKKPEDWEPREQTLFKSTEHGDDVDRPIQKSDGGWTYFAPDIAYHYDKVERGYDLLIDIFGADHGGYVKRMKAAVSALSGGTVPLDIKLIQLVKLFKNGEPFKMSKRAGTFITLRDLVEMVGADVARFHMLTRKSDASLDFDFDKVVEQSSENPVFYVQYAHSRICSVLRNAAAQGISTDDATLAGADLSNL-HEAEIALIAKLADWPRQVEIAARVHEPHRIATYLQELASDLHGHYNRGNKETELRFLQD-DANVTLTKIALARAVAVVISAGLGILGVTPATEMRMTQIDSLPPLRDVITSHDLKARKALGQNFLLDLNLTSRIARIPGDLSQTDVIEVGPGPGGLTRGLLAEGARHVLAIEKDSRCLPALQQIADIYPDRFTFVEGDALKIDPLEHLTPPIAICANLPYNVGTELLVRWLTPKEWPPFWSSLTLMFQKEVAERIIAQPGSKAYGRLAILAQWRADPRIALTLPPEAFSPPPKVHSAVVHLTALPEPRFPAKAKVLERVVAAAFNQRRKMLRSALKTLSPDIEDRLLAAGIKPTERAEQVPLEAFCALARQFEDDMNLIAQLEAEQIAALGKDIPDFKAGDTIRVGFKVTEGTRSRVQNYEGVCISRKNGKGISGSFTVRKISFGEGVERVFPLHSTNIEYIEVVRRGRVRRAKLYYLRSRRGKSARIAEDSTYKPLKAKS-MANSKRVLFQKRRMRVRNKLRKVNAGRVRLSVHRSNKNISVQLIDDVAGRTLAAASTLEPTLGLVGKNNVEAATKVGALIAERAKAAGVEEAYFDRGGFLFHGKVKALADAAREGGLKIMFAVLKTGGKQYKVQAGDILRVEKLAADAGETVQFNDILMIGGESTAIGAPFVAGAGVQAEIVDQIKGEKTINFVMRRRKHGSQRTKGHRQQLTLLRITDILASGADASGVKAAVGAGSAP-------------------KAAAPAAAPQKA---AAAAGA-----DDLTEITGVGPAAAKKLVDAGITTFAQLAAVDAD---------TFEGAKVKAEWVEQAKTLA--MSRVKGGTVAHARHKKVIKQAKGYYGRRKNTFKVAAQAVDKANQYATRDRHNRKRNFRALWIQRINAAVRAHDEALTYSKFINGLNLAGIEVDRKVLADLAVHEPEAFAAIVDQAKGALAAMQVTETLNEGLKRGYSIVVSAAELDEKVTAKLVEAQPEVELKGFRKGKVPMPLLKKQFGQRLMGEAMQEAIDGAMNKHFEDSGDRPALQPEIKMTNDDWKEGDDINVDMSYEALPVVPEIDLSVIAVEKLVVKADDASVDEALASLAETAQDFET--KDGAAEDKDQVVIDFLGKVDGEAFEGGAAEDYPLALGTGSFIPGFEEQLIGVKAGDEKNVEVKFPEEYGAENLAGKDAVFEVKVKEVKAPKAAEINDELATKFGAEDLDGLKGQIRERLEAEYTGAARQVMKRAMLDKLDDLVSFDLPPSLVEAEAGQIAHQLWHEENPDVQGHDHDPVVPTDEHNKLAERRVRLGLLLAELGQKAEVEVSEAEMTQAIMNQARQYPGQERQFFDYVRENPQMQQQMRAPIFEDKVIDHIAEKAEVTEKEISKDDLQKAVEALDDEMKLNELRDNDGATKRRKRVGRGAGSGMGKTGGRGIKGQKSRSGVAINGYEGGQMPLYQRLPKRGFNKPNRKAWAVINLGLIQKFVDAGKLTAG-DISEDTLVSSGLVRRKLDGIRVLAKGEVTGALTINVTGASKSAIAAVEAKGGALTVTNAAAAE---MIQMQTNLDVADNSGARKVQCIKVLGGSHRRYASVGDIIVVSVKEAIPRGRVKKGDVRKAVVVRTAKEVRRDDGTAIRFDRNAAVILNTAGEPVGTRIFGPVVRELRAKNFMKIISLAPEVLMRHKRGYRRLNRTHEHRKALFSNMAGSLIEHEQIKTTLPKAKELKRIIDKLITLGKRGDLHARRQAAAQLKEDKDVAKLFEILGPRYAERSGGYCRVLKAGFRYGDMAPMAIIELVDRDVDAKGKADKARLEEAEAADAMLQPKRTKFRKMFKGRIKGLAKGGSDVTFGTYGLKALEPERVTARQIEAARRAMTRHMKRQGNVWIRIFPDTPITAKPIEVRMGKGKGSVDRWACKVKPGRIMFEIDGVSEDVAREALRLAAMKLPVKTRVVIREDWMDPIFLTNSKTRKKERFVPIDPKNVRLYLCGPTVYDRAHLGNARPVLVFDVLQRLLRHVYGADHVTYVRNFTDVDDKINAARKEAGAQVHERSSETIDWYHQDMDALGAARPDHEPRATEYIDQMVTMIADLIAKGHAYEAKGHVLFRVRSYKDYGQLSGRSVDDMIAGARVEVAPFKEDPMDFVLWKPSTDDLPGWPSPWGRGRPGWHIECSAMADDLLWKKFDIHGGGNDLTFPHHENEIAQSCCAHSEVGFANVWLHNEMLQVEGKKMSKSLGNFFTVRDLLDQGIPGEVIRFVMLSTHYRKPMDWTEKKAQHANSTLDSFVSQTVNMEPAKDIPVEFLEALADDLNTSKAITVLYSLAKRFD--ELLASLLFLG------GGWVGNNT--LEELVFELEKLRAEAMETKDFSQVDRLKTALIDAGVEVRMSKAGVELVSGPNFDAAKLEGL-MAK---KPVSDPNYKVIAENRRARYDYAIADDIECGIILAGSEVKSLRENSANIAESYAAVEDGELWLVNSYIAPYTRA-MFSHEERKRRKLLVSRKELSRLWNETQRKGMTLVPLVMYFNHTGKVKIKIGIAKGKKNHDKRETDAKRDWGRQKARLLKQS-MSAKAQHYDIVRKPIVTEKTTMASENGAVVFEVAIDSNKPQIKEAVEALFGVKVKAVNTTITKGKVKRFKGTTGRRKDVKKAYVMLEEGNTIDVTTGLMGKDKNPRRVADNEAMAKLRMLRTSPQKLNLVAQLIRGKKVDRALVDLTFSKKRIAEDVKKCLQSAIANAENNHGLDVDELVVAEAYVGKNLIMKRGRPRARGRFGRINKPFSELTIKVRQVEEQAMVSAAEQMAANTSWAALGKATDLRNRILFTLGLLIVYRLGTFIPVPGIDGAALQEFVDQAGQGIAGIVSMFTGGALGRMGIFALGIMPYISASIIIQLLTAMVPALEQLKKEGEQGRKKINQYTRYGTVVLATFQAYGLAASLEAGDLATDPGWYFRAACVITLVGGTMFLMWLGEQITARGIGNGISLIIFVGIIAEVPAALAQFFASGRSGAISPAVIVGVMLMVVAVIAFVVFMERALRKIAIQYPRRQVGMKMTEAQQSHLPLKVNPAGVIPAIFASSLLLLPVTISTFSGSETGPIMSTVLAYFGPGQPLYLLFFASMIVFFAYFYTFNVSFKPDEVADNLKNQNGFVPGIRPGKKTAEYLEYVVNRLLVVGAAYLAAVCLLPEILRAQFSIPFYFGGTSVLIVVSVTMDTIQQVQSHLLAHQYEGLIQKSQLRGKG-KARKRKGPARKMKFLDLCKVYIRSGGGGNGCISFRREKFIEYGGPDGGDGGRGGSVWVEAVDGLNTLIDFRYQQHWFAGNGQSGAGRGRTGKDGEDIILRAPVGTEIIDEDQETVIADLTELGQRVLLAKGGNGGWGNLHFKTSTNQAPRRANPGQDEINRTLWLRLKLIADVGLVGLPNAGKSTFLAATSNARPKIADYPFTTLHPNLGVVLVDGNEFVVADIPGLIEGAHEGRGLGDLFLGHVERCAVLLHLIDGSSGSLLEDYKTICSELEAYGEGLADKPRVTVLNKIDTLDAEERAFLKDELEAIGAENVLLMSGASGEGVTDVLRALRPHVDARRAADRPVEEDGTWHPMAAKLKKGDKVIVLAGKDKGKTGEITSVNPAAGKAIVDGVNVAIRHQRQSQTAQGGRLPKALPIQLSNLALVDANGKATRVGFRMEGDKKVRFAKTTGDVIDA-MRSGIIAKKVGMTRVFMEDGKQIPVTVLQLENLQVVAQRTAEDNGYTAVQLGAGTAKVKRTSKAMRGHFAKANVEPKRKVAEFRVAPENMIEVGAEITAEHYNEGQYVDVSGISIGKGFQGGMKRWNFGGLRATHGVSISHRSHGSTGQCQNPGKVFKGKKMAGHMGAARVTTQNLQVVKADADRGILMIKGAVPGSKGGWVTVKDAVKKPLPDDVPTPAGLRATA-------------APAEAPAEGG-----------------EA----------------------MALKSYKPTTPGQRGLVLIDRSELWKGRPVKALTEGLTKKGGRNNTGRITMRRKGGGAKRLYRIVDFKRNKFDVTATVARIEYDPNRTAFIALVQYDDGEQAYILAPQRLAIGDRVVASAKADIKPGNAMPFSGMPIGTIVHNIEMKPGKGGQIARAAGTYAQFVGRDGGYAQIRLSSGELRLVRQECMATIGAVSNPDNSNQNFGKAGRMRHKGIRPSVRGVVMNPIDHPHGGGEGRTSGGRHPVSPWGKPTKGKRTRNTNKASQKLIIRSRHAKKKGRMLDEQKYTPRLKTKFRDEIRAKLKEEFGYKNDMQIPRLDKIVLNIGCGAEAVRDSKKAKSAIEDLTTIAGQQAVGTKAKNSHAPFRLREGMIIGTKVTLRGDRMYEFLDRLTTVAMPRIRDFRGVK-PSFDGRGNFAMGLKEHIVFPEIDFDKVDEVWGMDIIITTTADDDAEAKALLKHFNMPFNAMKLDVIKLDGASVGSVDLDEALFGVEPRADILHRVVRWQRNNAQAGTHKVKTRSEVSYSTKKIYRQKGTGGARHGSRKAPIFRKGGIYKGPTPRSHGHDLQKKVRALGLKMALSAKAATGSLVVIEDINTDGKTKTLANQIKALGWKRALVIDGASVNEEFLKAARNIEGLDVLPTMGANVYDILRRDTLVLTKAGVEALEARLKMENVILIVHLLLALGLIGVVLLQRSEGGGLGMGGGGGGAMSQRSAATAMGKVTWALAIAFIITSISLTVISAKNASGSSILDRVGGAPAVEETADPAGE-LTDALLPPPSADDAPLVPTADMSFFGKLKNKLFKSSSKLDEGLDAIVQDGG--EDEVDVPAPEPVKTP-----------IPNDAEIPTDESESEVAPDQETDLDPLAE--------------APEEAS-----------------------------------GILGRLMGRGGESADLRRVLDDDMLESLEELLIQSDMGVDTALRVSANIAEGRMGKKLSVTEIKGLLAGEIARIMEPVAKPLPLYATKPQVVLVVGVNGSGKTTTIGKLASQFRAAGKKVVIAAGDTFRAAAVEQLQVWGDRAGVPVLTAPEGSDPASLAYDAMTKAQEDGADLLMIDTAGRLQNRQDLMEELAKIVRVIRKKDPDAPHNTLLVLDATTGQNAVTQVEVFRKISDVSGLVMTKLDGTARGGVLVSLADKFGLPIHAIGVGEQIDDLAPFDPEEFAAALTGLDAMTKEQWGALQEQICNTVGDNNYKTWIKPLSFAGMNDGVVTLHAPTSFFGSYVLQNYGDMLLAQISTVAPTVRRISYAV---------EQASASSEPKAPAKPAEAAPKTLPGAPLDGRFTFDNFVVGKPNELAHAAAKRVAEGGPVTFNPLFLYGGVGLGKTHLMHAIAHELSARNPHLSVLYLSAEQFMYRFVQSLRERKMMDFKEMFRSVDVLMVDDVQFIAGKDSTQEEFFHTFNALVDQNKQIIISADRAPDEIKDLENRIRSRLQSGLVVDLHPTDYELRLGILQTKVETYRTMYPSLSIDSGVLEFLAHRISTNVRVLEGALTRLFAFASLVGKPINMDLVQDSLSDVLRASERKISIDEIQRRVAEHYNIRLSDMIGPKRVRNFARPRQIAMYLCKQLTSRSLPEIGRRFGGRDHTTVMHGVRRIEELRVQDGQIDEDVEMLRRALEA--MFGKIARKVFGTPNDRKIKATRPLVQQINALEAEFEKLGDAGLIEKTEEFRKRVADGESLDSLLPEAFANCREAAKRALGLRAFDVQLMGGIFLHQGNISEMKTGEGKTLMATFAAYLNALTGRGVHVVTVNDYLARRDADWMSKVYGALGLTTGVVYPRQEDGEKKEAYASDITYATNNELGFDYLRDNMKSELDQIYQRDHYFAIVDEVDSILVDEARTPLIISGPAEDRSEMYVQIDKLIPDLDPDHYTVDEKTRNVTFTDEGNEFLEQHLQARGILPEGQSLYDPESTTIVHHVNQGLRAHVLFTKDKDYIVRDREVVLIDEFTGRMMAGRRLSEGLHQAIEAKENCDIQAENVTLASVTFQNYFRLYDKLGGMTGTAATEAEEFAEIYGLGVVEVPTNRPIARVDEDDKVYRTAREKFEAIVEEVKIAHEKGQPVLVGTTSIEKSEMLSNLLKDAGLPHNVLNARQHEQEAQIVADAGKRGAVTIATNMAGRGTDIKLGGNVDFTVMEAIAADPEGDPEAIRTRIEEEHKTDEAAVKEAGGLYVLATERHESRRIDNQLRGRSGRQGDPGRSSFFLSLEDDLMRIFGSERLEKVLSTLGMKEGEAIVHPWVNKSLERAQAKVEGRNFDIRKQLLKFDDVMNDQRKVIFSQRREIMEAEDLSEIVQDMRTDVIDELVDIYAPAKSYAEQWDMPGLYAASIEKLGVDLPIIAWADEDGVDQTVIRERLEEATDKAMAEKAEAFGPTIMRQIEKQFLLNTIDGKWREHLLTLEHLRSVVGFRGYAQRDPLNEYKTEAFQLFENLLDGLRETVTTQLSQVRPMNEEERAAMIAQEEQRQKMAQMMAQQSTPDATSAER---PLIAGFDETDPATWGNPGRNDACPCGSGDKFKHCHGKI-MKQSAELIALQDRLGYTFTKPELLRQSVTHSSMSSPGRNDNQRLEFLGDRVLGLVMSEALLLHDQQATEGVLAPRFNALVRKETCADVARQIDLGAALKLGRSEQMSGGRRKLALLGDAMEAVIAAVYMDGGFETARTLILRLWGTRIEAVEDDAKDAKTSLQEWAQARAQEPPRYILAERSGPDHAPVFTIQAVLASGETSTATAGSKRQAEQAAAKALLDKLG---MANLGKRTRAAREAFAGKDNLTVEDAVALLKGNANAKFDETIEIAMALGVDPRHADQMVRGVVSLPNGTGKTVRVAVFARGPKAEEAQAAGADIVGAEDLMEAVQGGTIDFDRCIATPDMMPIVGRLGKVLGPRNLMPNPKVGTVTMDVAQAVKDSKGGQVQFKAEKAGVVHAGVGKASFDEAKLVENIRAFVGAVSAAKPSGSKGTYMKKISVSSTMGPGVTLDVNSAVGSMSEDFELDTDDLIRRMDGAMANLRTEFASLRTGRASASMLEPIDVDAYGSMTPINQVGTVNVPEPRMVTINVWDKGLVGKVEKAIRESGLGINPQLNGTIIMLPIPELNEERRRELGKVAGNYAEHARVSVRNVRRDGMDKIKKAKNDGMSEDDQKLWESEVQDMTNTYIKKVDDLLETKQEDIMQVMAKKLAGTMKLQIAAGKANPSPPVGPALGQRGINIMEFCKAFNAKTQEMEPGAPCPTVITYYQDKSFTMDIKTPPASYYLKKAAGLKRGAENPGRETVASVTVAQVREIAEAKMKDLSANDVEAAMLIIVGSARSMGIEVKMFENLSERLGGVFDRLTKQGALSADDVKTAMREVRVALLEADVSLPVARQFIKAVEKKATGASVTKSVTPGQQVVKIVHDELIAVLAGEGEPGSLKIDNAPAPILMVGLQGSGKTTTTAKLAKRLTERDGKKVLMASLDVNRPAAMEQLEILGKQIGVTTLPIVKGEDPVAIAKRAKTQASLGGYDVYMLDTAGRLSIDEELMAQVEAVRDVANPRETLLVVDGLTGQDAVHTAENFDDRIGISGVVLTRMDGDGRGGAALSMRAVTGKPIKFVGLGEKMEALETFEPERVAGRILGMGDIVALVEKAQSTLEAEQAERMMKRFSKGQFNMNDLKMQLEQMIKMGGMEGMMQMMPGMGKMAKQVGDAGMDDKVLKQQIALIQSMTKRERANPQLLQASRKKRIAAGAGMQVSDLNKLLKMQRQMSDMMKKMGKMGKGGMLKQAMKGMMGKAGMDPS----QMDPAAMEAAAKQLGSRMPGGLPGM-GGMGLPGGLSGLGKKKMIPFERLQKIKERLEYVEARMALGE--GDIAQLGREYSELKPVVDQILDWERLVADLAEAEAMLDDPEMRELAEEELLGLRERLPEVEHAVQLSLLPKDAADARPAMLEIRPGTGGDEAALFAGDLARMYQRYVEQHGWRWEVIEEATSELGGIRELVVRIKGDGVFARLKFESGVHRVQRVPETESGGRIHTSAATVAVLPEAEEVDIAINPGDIRIDTMRASGSGGQHVNTTDSAVRITHMPSGIVVTSSEKSQHRNRDIAMQVLRARLFDLERQKVADERSAHRKSQVGSGDRSERIRTYNFPQGRLTDHRIGLTLYKLDQIMQGDLDDIIDALTAEHQAALLAELGTMDRAQKEKLVDELGQIFDSSGVVVVARYEGLTVADMQSLRGIAREAEASVRVAKNRLARIAVKGTQCESIDEFLGGMTVLTFSEDPVAAAKVVEDFAKTNKKFEILGGVMGGTALDRAGVAAVSKMPSRDELIASIVGCIGAPASNIAGAIGAPASNIASILSTIEEKAEAMKTFSAKPADIEKKWIIIDAEGVVLGRLASIIAVRLRGKHKPSFTPHMDCGDNVIVINAEKVQMTGKKREENFYWHTGHPGGIKSRTKAEILEGKHPERVVTQAVKRMLPGNRLSRQIMTNLRVYAGAEHPHEAQSPEVLDVKSMNKKNTRV------------MGGLLGPDFPGDIGLAVSGGGDSMAMLTLAHNWTRVWGVRLWVVTIDHGLRRESATEAAMVAQECAALGWPHATVKWH-WDGSGNLQDAARQARLDLIDRWRGV--LRHVLMAHTKDDLSETFLMRFKRGSGVDGLSAMRDEMQSDSFEVLRPCLSMRREDLRHYLTVLKTPWAEDPSNLDPKYERVRVRQSLPDLDRLGLSVDLIAETAKRLRRAQEALQQRAVQVWRDIGHARTGDILLTRSGFDGVERETQLRLLAAGLQYVSSAPYRPRAEPLEALLDRLLGGGGGTLHGCECRAEKEQLRIFREEKPLKNLTDPQHGFWDQRWRIPAPL--PDGAQIRNLGDEGWRLIENKRDTAIPYHAARSLPAVWKGDVLLACDAFGVGPGGGLALWRAGHSFARFLLSHMADLKALAESIVGLTLLEAQELKTILKDEYGIEPAAGGAVVMAGPADAGEAAEEKTEFDVILKAAGPKKINVIKEVRGITGLGLKEAKELVEAGGKVKEGVSKDEAEELKKKLEEAGAEVEVKMSRIGKKPVELPSGVTASVSGQVVEVKGPKGVQTFTATDDVSITVEDSAVKVAPRGKSKRARQQWGMSRTVVANLVHGVQNTFKKELEIHGVGYRAAVQGKTLKLNLGYSHDVDFAIPEGISIVCAKPTEVVIEGHDKQQVGEVAAKIRDWRRPEPYKGKGIRYKGEFIFRKEGKKKMKVILLERVAKLGQMGEVVDVKAGYARNFLLLQGKALTASKENIAQFDAQKAQLEARNLETKKEAEALGEKLDGQQFIVIRSASDGGNLYGSVTTRDASEVATEEGFSIDRKQVVIIDPIKILGLHTLDVVLHPEVTVQIQMNVARSVEEAELQASGKSIQELAAEEEAQAEFEVSQLFDDLGSAA--DEDGDDAPAQDAEETSED-----MSDNDGKKTLGLRGGARSGNVKQSFSHGRTKNVVVETKRKRVVVPKPGAAGGRGPGVGPGSAGGKRPAGITDAEMERRLKALHAAKAREAEETAARAAEEKAREEERNRKRAENEAKAKEAAIAEERARQKADDEKRKVEEAAAAAKRAAAPAAPPGDDAPNRNVNKPEATPRRDDRGNDRNARNTRGRDDNRRSGKLTLNQALGGG-GNRHKSMAAMKRKQERARQKAMGGQQAREKVVRDVQLPEAITVAELANRMTEKVGDVIKALMANDIMVTQNQSIDADTAELIIEEFGHKITRVSDADVEDVIQQVTDDAADLKSRPPVITIMGHVDHGKTSLLDAIRDAKVTAGEAGGITQHIGAYQVTTDNGSVLSFLDTPGHAAFTSMRSRGAQVTDIVVLVVAADDAVMPQTVEAINHAKAANVPMIVAINKIDRPAANPTKVRTDLLQHEVVVEQMSGDVQDVEVSAITGQGLDQLLEAIALQAEILELKANPDRNAEGAVIEAKLDVGRGPVATVLVQNGTLKLGDIFVVGEQYGKVRALINDKGERIKEAGPSVPCEVLGLNGTPEAGDVLNVVETDAQAREIAEYRANLAKEKRAAAGAATTLEQLMQKAKEDEDVSELPVLVKADVQGSSEAIVQALEKVGNGEVRVRVLHAGVGAITESDVSLAEASGCPVIGFNVRANASARNSANQKGVEVRYYSVIYDLVDDIKAAASGLLSNEIRENFIGYATIKDVFKVTGVGKVAGCLVTEGVARRSAGVRLLRDNVVIHEGTLKTLKRFKDEVSEVQSGQECGMAFENYDDIRANDVIEIFEREEITRTLDMAKVKKAPRPKAQTPKGFRDYFGTEVTERADMLKTIAEVYHQYGFDALESSGVETVEALGKFLPDVDRPNEGVFAWQ--DEDDA---WMALRYDLTAPLARVYAQYRNDLPLPYRRYAMGPVWRNEKPGPGRYRQFYQCDADTVGSANVAADAEICAMLSDTLERVGIPRGDYLVRVNNRKVLNGVLETMGLS-DD--AQRDAVLRTIDKFDKVGEAGVRELLGKGRLDASGAYIDGVGLSVDQAEPVVAFLTSKSSDVAKTFANLRDAIGASTIGAEGVGELEKIGDLLAVQGYNADRIEIDPSVVRGLGYYTGPVFEAELTFEILDEKGRKRQFGSVAGGGRYDDLVKRFTGQAVPATGVSIGVDRLLAALREKGRIGGTVQGPVVVTVMDKDRMADYQTMVAELRNAGIRAEVYLGNPKNFGNQLKYADKRESPIAVIEGGDEHANGMVQIKDLILGAKIAENATLEEWKERPSQYEVPRDQLVAKVREILDLNPMDDLRDKYLTLINDAGDEAALEELRVQAVGKKGEISLQMRSLGKMSPEERQVAGPALNALKDEINAALAAKKSALADAALDARLATEWLDVTLPARPQRQGSIHPISQVTEEVSAIFADMGFAVAEGPQIDTDWYVFDALNIPSHHPARAEMDTFYMSRAEGDDRAPHILRTHTSPVQIRSMEKQGAPIRIIAPGRVYRADYDQTHTPMFHQVEGLCIGKDVSMANLKWCLEEFVKAFFEVDHVELRFRASHFPFTEPSAEVDIQCSWENGVLKVGEGDDWMEILGSGMVHPKVLENAGVDPKEYQGFAFGIGIDRLAMLKYGIPDLRAFFDSDLRWLKHYGFTPLDVPTLHGGLSKMEMAEQAGLDLVEISPNANPPVCKIMDYGKFKYEQQKRESEARKKQKTIEVKEVKFRPGTDTHDYDVKMRNVFKFLEGGDKVKVTLRFRGREMAHQQLGRELLERVAGDVKELGKIENMPKMEGRQMVMMIGPLPKMKFTFSWLKDHLDTTASVDEIAEVLTDLGLEVEGIENPAQALAGFTLAKVTHAEQHPDADRLRVCTVATDEGDKQIVCGAPNAREGITVVLCKPGDYVPGLDITLSVGKIRGVESHGMMASMKELQLGDDHDGIIELPSGDVGTKFVDWLAANMPEKVDPVIEIAITPNRQDALGVHGIARDLAARGLGKLKPLKVEAVEGSFESPITVTIDEDTRDGCEVFAGRMIKGVKNGPSPEWLQQRLKAIGLRPISALVDITNFFTFDRNRPLHVFDADKVRGNLRIHRATAGDTLVGLDEKTYTFGEGQVVISDDSGIESIAGIMGGLATGCTEETTNVFLEAAVWDHIQIAHTGRALKINSDARYRNERGIDPAYNMQAIEDATQMILDLCGGEASKVVTAGQVPDVARSYRLDAKRVVSLVGMEIPEAEQRQTLTALGFRMEGDQAHVPSWRADVKGEADLVEEVARIASLTKLVGKPLPRLQNGVPKPILSPIQKRERTVRRTVATLGYNECVTYSFIDQAAAALFGGGDDVTKLANPISSEMSHMRPSLLPGLLQAAARNQARGYADLALFEVGHAFQGGEPGEQNLQVAGLLVGKSGPKDVHGAMRSVDLFDAKADAESALGAMGAPAKVQILRGGEGWWHPGRHGQICLGPKKVLGVFGELHPKVLDEMGVKGPAVAFVLYPEQVPLPRKTGATRAALTQNDLQAVERDFAFVVDADVEALTVVNAAAGADKALIEEVRVFDEFIGGNLGEGKKSLAISVRLQPVGQTLKEKDIEAVAAKIIAKVSKATGGELRGMHAYRSHTCAQLTKDNVGDTVRLSGWVHRIRDHGGVLFIDLRDHYGITQVIADADSPAFADLEKVRSEWCIRVDGNVLARDESLVNPNLPTGAIEVFARELEVLSAAAELPLMVFGDQEYPEETRLRHRYLDLRREVMQKAMTLRSDVVSSMRKRMWDLNFREFQTPIITASSPEGARDFLVPSRLHPGKFYALPQAPQQFKQLIMVSGFDKYFQIAPCFRDEDPRADRSPTDFYQLDLEMSFVEQQDIFDTIAPVLAGVFEEFGEGKNVDADWPQISYRDAAKWYGSDKPDLRNPIKMQDCSEHFRGSGFAIFAKLLEQDGTEVRAIPAPTGGGRKFCDRMNKFAQGEGLPGMGYIFWRDKTGEEVGMEAAGPLAKNIGPERTEAIRQQLGLTVGDAAFFLGGKPKAFEKIAGRARDVIGEELGLTDKNRFAFAWIVDFPIYEQDEETGKIDFEHNPFSMPQGGAEALEGNPLDVLGYQYDLACNGYELLSGAIRNHKPEIMFKAFEIAGYGKDEVEKRFGALVNAFQYGAPPHGGCAAGIDRIVMLLADQQNIREVVMFPMNQRAEDLMMNAPSDPMSDQMMELGLRVIPQDMTPLNRIRNFSIVAHIDHGKSTLADRLIQSTNTVAERDMQAQLLDSMDIERERGITIKANTVRIDYTANDGLDYVLNLIDTPGHVDFAYEVSRSMRAVEGSLLVVDASQGVEAQTLANVYTAMEADHEIVPVLNKVDLPAAEPERIAEQIEDVIGIDASDACLISAKTGIGIPDVLEAIVKRLPPPSGDPDAPLKAMLVDSWYDAYLGVVVLVRIMDGTLKKGDNIKMMQTDAKYSVDRIGVFRPQMENVQELGPGEIGFITAQIKQVRDTKVGDTITHEKKGATEALPGFQPSQPVVFCGLFPVDSAQFEDLREAIEKLALNDASFSYEMETSAALGFGFRCGFLGLLHLEVIRDRVEREYNIDLITTAPSVIYHIYQRDGTKHELHNPADMPDPSTIDHLEEPRIKATILVPDEYLGDVLKLCQDRRGIQLDLTYAGARAMVVYDLPLNEVVFDFYDRLKSVTKGYASFDYSMEGYREDNLVKMSILVNDEPVDALSTMVHRDRAEMRGRAMVEKLKDLIPRHMFKIPIQAAIGGKVIARETLSAMRKDVTAKCYGGDASRKKKLLEKQKAGKKKMRQFGKVDIPQEAFISALKMDNMSLPPGFLDELRNRISLSQVAGRKLMWDARKSNQGKGDLWAPCPFHHEKTASFHVDDRKGYYYCFGCHAKGDAISFVRETENVSFIEAIEILATEAGMEMPKADPRAKEKSDRRTVLSEVMEQAVQFFRLQLKAEAGSAARAYLDKRGLSQSARDTFEIGFAPAGWENLREHLKAKGIAPELMLACGLVKSSDKGREPYDVFRNRIMFPIRDARGKAIAFGGRAMDPNDNAKYLNSPETELFDKSRNLYNMRPAREAAGRGQPLIVAEGYMDVIALSEAGFGAAVAPLGTAVTEPQMQLMWRMSQEPLIALDGDKAGLRAAYRVIDIALPLLEAGKGLRFAIMPEGKDPDDLVRSGGPEAVQKVLDDAMPMVRLLWQNETEGKVFDSPERKAALDKLLRARIAKITDPHLRRHYGDEIKELRYALFRPNRQNASGFGQ----AGKSEWSNAPLPATAGAKSSLMVT-AGDNAETALREAVILLSVLATPDVAAEFEGQLERMPCHDPDHALLRDVILRNIGA-ENDMRFRAEDAIGPETLETMLSARHVAVVPCIRTPGNPEMARMTVAEELAKLDALHGWHVELAEAQEDLEDQADEALTWRLAEAAKVRAQAGRLKDENSAEYDVGPNGASIDREERSALDSLLSTIRFDKGRG--MCADTPEYKQTLNLPKTDFPMRAGLPKREPAWLDRWEKIGVYDRLREKAERAPFTLHDGPPYANGHLHIGHALNKTIKDMIVRSHQMMGRDARYIPGWDCHGLPIEWKIEEQYRKKGKDKDDVNVIDFRQECRKFAEGWVDVQREEFKRLGITGNWADPYLTMNFHAERVIAEEFMKFLMNGTLYQGSKPVMWSPVEKTALAEAEVEYHDHKSHTIWVPFKVQNG---SGDLATARVVIWTTTPWTIPSNKAVAFGKGISYGLYEVTGTPEECWCAAGETYILADTLAADVFTAARLEEGQFKRVRDVAADELDGLVLAHPFNGMDGADGYWDYDVPMIDGDHVTDDAGTGFVHTAPSHGQEDYECFVARNWLDR-MTHNVGEESEFLSHVPFFAGMRVFDQKGKEGKANNAVIAKLVEAGGIIARGRVAHSYPHSWRSKAPIIYRNTPQWFASVDREVGDGLDTHGKTIRSRALNSIDEVKWWPQTGRNRLYSMIEARPDWVLSRQRAWGVPLTCFTRKGLLPTDPDFLLRNDAVNARVAAAFEVEGADCWYADGAKERFLGSDVNPDDYDQVFDVLDVWFDSGTTHAFVLRDREDGSEDGIADVYMEGTDQHRGWFHSSLLQACGTLGRAPYRNVVTHGFTLDEKGNKMSKSLGNTIVPEKVIQQYGADILRLWVAQTDYMVDQRIGPEILKGVADSYRRLRNTMRYMLGALNDFKPEQRVPVEEMPELERLMLHRLAEIDHQVRKGYSEFDFQGVFSTVFNFATIDLSSFYFDIRKDALYCDGDSHRANACRTVLDILFHRLTTWLAPILVFTMEEVWLERFPGEASSVHLIDMPETPKDWLDEPLAAKWAEVRKVRRVVTAALEVQRTDKVIGASLEAAPVVHVRDADTLAALKSVNFEDICITSAISLSGDPQPAEAFRMPETEGVGVVFEKADGEKCQRCWKILPDVGQHKHAGTCKRCNDALG--MSQNIRIRLKAFDYRVLDASTQEIVNTAKRTGATVRGPIPLPNKIEKFTVLRGPHVNKKSRDQFEIRTHKRMLDIVDPTPQTVDALMKLDLAAGVDVEIKV--MSDQINSLEELAGVA-----GVEAAEAEVVAPREPVRDELGRAYATGKRKDAIARVWIKPGSGKVTVNGREMDKYFARPVLQMILRQPFQIAGREGEFDVYATVKGGGLSGQAGAVKHGISKALQLYEPSLRGALKAAGFLTRDSRVVERKKFGKRKARRSFQFSKRMPTIQQLIRKPRQPKVKRSKSQHLEQCPQKRGVCTRVYTTTPKKPNSAMRKVAKVRLTNGFEVISYIPGESHNLQEHSVVLIRGGRVKDLPGVRYHILRGVLDTQGVKDRKQRRSKYGAKRPKMARDTRRAKKKVSKNIAAGVAHVNSTFNNTKILISDVQGNAISWSSAGTMGFKGSRKSTPYAAQLAAEDAGKKAQEHGVKTLEVEVQGPGSGRESALRALAAAGFNITSIRDVTPIAHNGCRPPKRRRVMSENRRGLLIILSSPSGAGKSTLSRRLLDWDPSLSFSVSATTRAPRPGEVDGEHYHFLEEDAFKRQVADDGMLEHAHVFGNFYGSPKAPVQKAIESGRDVLFDIDWQGAQQIVNSSLGQHTLSVFILPPSIVELRRRLIGRGQDSDDVISKRMQKSWDEISHWGSYDYVLVNDDLDRTFEDLKTIVTATRLRRLQQPRLVEHARGLQSEFQELSMAQSFLGQKRLRRYYGKIREVLEMPNLIEVQKSSYELFLKSGDQPTPSDGEGIKGVFQSVFPIKDFNETAILEFVNYDLEKPKYDVEECMQRDMTYSAPLKVTLRLIVFDIDEDTGAKSVKDIKEQDVFMGDMPLMTPNGTFVVNGTERVIVSQMHRSPGVFFDHDKGKTHSSGKLLFACRIIPYRGSWLDFEFDAKDIVFARIDRRRKLPVTTLLYALGLDQEAIMDAYYNTVSYNLKKSGGWSTKFFPNRVRGTRPAYDLVDADSGEVIAEAGKKVTPRAVKKLIDEAKVENLLVPFDQIVGKFVSQDMINEETGAIYVEAGDELTWEVDKDGDVTGGTLKELVDAGVTEIPVLDIDNVTVGPYMRNTMAMDKNMGRDTALMDIYRVMRPGEPPTVEAASALFETLFFDGERYDLSAVGRVKMNMRLALEKEDTQRTLDREDIVKCIKALVDLRDGRGDIDDIDHLGNRRVRSVGELMENQYRVGLLRMERAIKERMSSVEIDTVMPQDLINAKPAAAAVREFFGSSQLSQFMDQTNPLSEVTHKRRLSALGPGGLTRERAGFEVRDVHPTHYGRMCPIETPEGPNIGLINSLATFARVNKYGFIETPYRVVKEGVVTDEVHYMSATEEMRHTVAQANANLDENMKFKNELVSTRKSGDYTLSPSDAVDLIDVSPKQLVSVAASLIPFLENDDANRALMGSNMQRQAVPTLRSEAPLVGTGIEEVVARDSGAAVMAKRAGIIDQVDAQRIVVRATSDLEMGDAGVDIYRMRKFQRSNQNTCINQRPLVKVGDTVLKGEVIADGPSTDMGELALGKNVIVAFMPWNGYNYEDSILISERIAKDDVFTSVHIEEFEVAARDTKLGPEEITRDIPNVGEEALRNLDEAGIVYIGADVEPGDILVGKITPKGESPMTPEEKLLRAIFGEKASDVRDTSLRVKPGDYGTIVEVRVFNRHGVEKDERALQIEREEIERLARDRDDELAILDRNIYARLKGMIEGKVAVKGPRGVRPNAEINEELLETLTRGQWWQLALGEEDDAKHVEALHEQYEVQKRALDARFEDKVEKVRRGDDLPPGVMKMVKVFIAVKRKLQPGDKMAGRHGNKGVVSRVVPMEDMPFLADGTPVDFCLNPLGVPSRMNVGQILETHMGWASRMLGIKIDDALQEYRRSGDMTPVREALRIGYGDDVYEDGFADMSEGDLLEAAGNVTSGVPIATPVFDGAKEADVNDALLRAGFSESGQSVLFDGRTGEQFAREVTVGVKYLLKLHHLVDDKIHARSTGPYSLVTQQPLGGKAQFGGQRFGEMEVWALEAYGAAYTLQEMLTVKSDDVAGRTKVYESIVKGEDNYEAGVPESFNVLVKEVRGLGLNMELLDAEEDEMPLYEHVFISRQDLSNAQAESLVEHFGTVLADNGGKVVESEYWGVKTMAYKINKNRKGHYAFLRTDAPAPAVKEMERLMGLHDDVMRIMTIRVDEHAEGPSVQMQKRDERSDRRERR-MSDIAIEKLTETEARAELARLSLILSQANQDYHQRDAPELSDAEYDHLKRRNAEIETKFPQLMRADSPSDQVGSPVSDGFSKVAHAVRMLSLANAFDDEDVNDFDMSIRRYLGLTEGQGLAYTAEPKIDGLSLSLRYENGQLVQAATRGDGTIGENVLANALTIADIPQIIANAPHITEIRGEVYMSHDDFAALNQRQAARGGKLFANPRNAAAGSLRQLDSEITKNRPLRFFAYSWGELSEPLSDTQMGAVERMKALGFQVNPLTQLCPTPQALLQQYAKIEEQRATLGYDIDGVVYKVNDLALQARLGFRSTTPRWAIAHKFPAELAWTRLEAIDIQVGRTGALSPVARLTPVTVGGVVVSNATLHNEDYIAGRDSFGGEIRSGKDIRVGDWVQVYRAGDVIPKIADVDLSKRPEAVEPFAFPKVCPECGSDAIREDGDAIRRCSGGMICPAQAVERLKHFVSRAVFDIDGLGAKQIEQFYTDGWVKEPADIFTLGARFSTGLQQLRNREGWGEKSALNLFEAIETSRIVPLGRVIFSLGIRHVGENASNLLARHYGAWSDFESAMVAAAPQEGTSWDDLLSIDGVGVVMAQSLVSAMNQPAERASIDRLIAQLTVEDAARPDTEGSPVAGKTVVFTGALEKMSRAEAKARAESLGAKVAGSVSKKTDIVVVGPGAGSKEKKARELELMVLDEDAWLELIER-MIHKNWAELIKPTQLEVKPGNDPARKATVVAEPLERGFGLTLGNALRRVLMSSLQGAAITSVQIDNVLHEFSSVDGVREDVTDIILNLKGVSIRMEVEGPKRLSVTAKGPGVVTAGDITESAGIEILNRDHVLCHVDEGAEVYMEFTVNTGKGYVSADKNKPEDAPIGLIPIDAIYSPVKKVAYDVQPTREGQVLDYDKLTMKVETDGSISPEDAVAYAARILQDQLSIFVNFDEPESAQRQDDDDGLEFNPLLLKKVDELELSVRSANCLKNDNIVYIGDLIQKTEAEMLRTPNFGRKSLNEIKEVLSGMGLHLGMDVEDWPPDNIEDLAKKLEDNFMAERDNRRGNRRD-RDEAPEFADRLVAINRVSKTVKGGKRFGFAALVVVGDQKGRVGFGKGKAKEVPEAIRKATEQAKRQMMRVPLKEGRTLHHDIEGRHGAGKVVMRTAPQGTGIIAGGPMRAVFEMLGVQDVVAKSIGSQNPYNMIRATLNGLGKEQSPRNVAQRRGKKVADILPKRDEAP-----VAEEAMNDPIGDMLTRIRNSQMRGKSTVLTPASNKRARVLDVLASEGYIRGYESGTDSAGHPTFEISLKYYDGEPVIRELARVSKPGRRVYMGVKEIPQVRQGLGVSIVSTSQGVMSDAKARAANIGGEVLCTVFMHDIRAIRDNPAAFDAALSRRGIENASSPLLKLDAERRAAILAAETAQSDQKKAAKEVGAAKAKGDDAEFERLRALVSEKKAEVADMQTRAKELDEKLQYELSVIPNLPYDDVPDGADEDDNVEVRTWGTPATLDFEAKEHFELAGVAASMDFELAAKISGARFVMLKGAVARIHRALAQFMIDTHVDENGLTEMNSPVLVRDEAMYGTDKLPKFGEDSYQTTNGWWLVPTSEVPLTYTVAGDILDESTLPIRMAAHTLCFRSEAGSAGKDTSGMLRQHQFEKVEMVSITHPSQSDAEQQRMLGCAQGILERLDIPYRTVELCTGDTGFGARRTFDIEAWLPGQNTYREISSVSTTGDFQARRMNARFRPADGGKPEFLHTLNGSGLAVGRCLIAVLENGQQADGSVTLPAALVPYLRGKTTLTADGVLAMAITAAQVKELREITAAGMMDAKKALVETDGDMEAAIDWLRTKGLAKAAKKSGRTAAEGLVAVAVAGGKGVAVEVNSETDFVSKNAEFQEMVGGIATVALNVADVEALKVADLGGKTVETVITDKIATIGENMSVRRMATVTGETVVTYVHNAATAGMGQIGVLVALNGGNEDFGKQIAMHIAAANPASLSEADLDPAVVEKERQIQIDIARESGKPEQVIEKMIIGRMKKFLAEVTLLGQQFVVNPDITVEQAAKDAGAEIVAYVRMQVGEGIEKKEEDFAAEVAKAVQGMNQELTNNPFNPVAPIKTFDEIKVSLASPERILSWSFGEIKKPETINYRTFKPERDGLFCARIFGPIKDYECLCGKYKRMKYRGVVCEKCGVEVTLQKVRRERMGHIELAAPCAHIWFLKSLPSRIGLMLDMTLRDLERVLYFENYVVIEPGLTDLTYGQMMSEEEFLDAQDAYGMDAFTANIGAEAIREMLANIDLENEAEQLRADLKEATGELKPKKIIKRLKVVESFLESGNRPEWMIMTVVPVIPPELRPLVPLDGGRFATSDLNDLYRRVINRNNRLKRLIELRAPDIIVRNEKRMLQESVDALFDNGRRGRVITGANKRPLKSLSDMLKGKQGRFRQNLLGKRVDFSGRSVIVTGPELKLHQCGLPKKMALELFKPFIYSRLEAKGLSSTVKQAKKLVEKERPEVWDILDEVIREHPVMLNRAPTLHRLGIQAFEPTLIEGKAIQLHPLVCSAFNADFDGDQMAVHVPLSLEAQLEARVLMMSTNNVLSPANGAPIIVPSQDMILGLYYVTIMREGMKGENMVFSSLEEVEHALNAGEVHLHAKVQVRLKQIDDEGAEVYTRFETTPGRARLGALLPMNAKAPFSLVNDLLRKKDVQRVIDTVYRYCGQKESVIFCDQIMTLGFREAFKAGISFGKSDMVVPDNKWDIVGHTRDQVKDFEQQYMDGLITQGEKYNKVVDAWSKCNDKVTEAMMSTISAEKRAEDGSVMEPNSVYMMAHSGARGSVTQMKQLGGMRGLMAKPNGDIIETPIISNFKEGLTVLEYFNSTHGARKGLSDTALKTANSGYLTRRLVDVAQDCIVRMVDCGTDRAVTASAAVNDGEVVATLGERVLGRVAAEDICHPVSSEVLAPEGSLIDERTADAIEDSGVLTARIRSPLTCEAEEGVCSQCYGRDLARGTRVNLGEAVGIIAAQSIGEPGTQLTMRTFHMGGVASGSSRSFLEASQNGKIAFANPSVITNASGEQIVMSRNMQVLILNEQGEEIASHKMGYGSKLFVTDGQTIARGDKMFEWDPYTLPIIAEKDAVARHVDLVNGLAVREETDDATGMTQKIVVDWRAAPKGNELKPEIILMDKDGEPVRNDAGNPITYPMSVDAVLSVEDGQDVKAGDVVARIPREGAKTKDITGGLPRVAELFEARRPKDHAIIAEIDGYVRFGKDYKNKRRVSIEPQDETMDAREYMIPKGKHIPVMEGDFIQKGEYLMDGNPAPHDILGIMGIEALANYMIDEVQDVYRLQGVKINDKHIEVIVRQMLQKWEIQDSGDTTLLKGEHVDKAEFDAANEKALSKSGRPAKGEPILLGITKASLQTRSFISAASFQETTRVLTEASVQGKKDKLVGLKENVIVGRLIPAGTGGATQEMRHIASTRDNVVVEARRIEAEAAAALAAPEPV-ADVVGGSEFDTLIVTPESREMSRRHAAEKREVLPDAKFGDRVLTKFMNNLMIDGKKSVAERIVYNALDRVETKVKRAPVELFHEALDNIKPSVEVRSRRVGGATYQVPVEVRPERREALAIRWLITASRSRNENTMEERLAGELLDAVQSRGSAVKKREDTHKMADANKAFSHYRWMPYAHSDKS---ALLHSPAPDIKDRVKLEGGKEFVLHTEFSPAGDQPTAIVELTGGINDGDRSQVLLGATGTGKTFTMAKVIEETQRPAIILAPNKTLAAQLYGEFKGFFPDNAVEYFVSYYDYYQPEAYVARSDTFIEKESQINEQIDRMRHSATRALLERDDVIIVASVSCIYGIGSVETYGAMTQDLLVGTEYDQRKVIADLVAQQYRRNDQGFQRGSFRVRGDSLEIWPAHLEDRAWKLSFFGEELEGITEFDPLTGVKTDTFDRIRIYANSHYVTPKPTMNQAVIGIKKELRQRLDQLVGEGKLLEAQRLEQRCNFDVEMLEATGVCSGIENYSRYLTGRAPGEPPPTLFEFIPDNAIVFADESHVSVPQIGAMYKGDFRRKMTLAEHGFRLPSCMDNRPLKFEEWDAMRPQSIFVSATPSRWELEQSGGVFTEQVIRPTGLLDPPVEIRPVEMQVDDVLDEIRKVTAEGMRTLVTTLTKRMAEDLTEYLHEQGIKVRYMHSDIDTIERIEILRDLRLGAFDVLIGINLLREGLDIPECGLVAILDADKEGFLRSETSLVQTIGRAARNEHGRVIMYADRMTGSMERAIGETNRRRAKQEAYNVEHGITPATVKKNVEDILAGLYKGDTDQSRVTAKIDKGHAGGNLQTVLEGLRNDMRKAAENLEFEEAARLRDEVKRLEAVDLAVSDDPLARQSAVEAASEAAVKSRGRSTAGRPGQRGGVKRRR--MAAKPFFRRRKVCPFSGDNAPKIDYKDTRLLQRYISERGKIVPSRITAVSAKKQRELAKAIKRARFLALLPYAVKMPKRILTGTVTSTANTQTVTVSVERRFKHPVMQKTVKRNKKYRAHDEAEKFAVGDMVRIIECAPRSKTKRWEVLAD--MANSPQAKKRARQNEKRLEVNKARRSRIRTFLRGVEEAIASGDKAAATAALKAAQPELMRGVTKGVFHKNTASRKVSRLAARVKALGMSRSVWKGPFVDAYVLKKAEKARDSGKNEVIKIWSRRSTILPQFVGLTFAVYNGHKHIPVNITEDMIGQKFGEYSPTRTYYGHAADKKAKRKMARIAGVNIPTNKRVPIALTYITGIGHTSAAAIIEAVGIDATRRVNELSDAEVLAIREHIDATYTVEGDLRREVTMNVKRLMDLGCYRGLRHRRNLPVRGQRTHTNARTRKGPAKAIAGKKKMAIKIRLARGGSKKRPFYRIVAADSRMPRDGRFVEKLGTYNPLLPKDSEERVKMNVERIQYWLGEGAQTTDRVSRFLEAAGVVAKKERSNLKKGEPGKKAKERAEEKASKAA--------DAAEAAAEAMSITPEEKTRLMKEFGTKEGDTGSPEVQVAILTSRISTLTEHFKTHKKDNHSRRGLLKLVALRRKLLDYTKAKDVARYQDLIKRLGIRRMATLNDIRSTFLNYFAKQGHTIVDSSPLVPRNDPTLMFVNSGMVQFKNLFTGLETRDYNRATTAQKCVRAGGKHNDLDNVGYTARHHTFFEMLGNFSFGDYFKEEAITFAWELITKEYGIPKDKLYTTVYHTDDEAYNIWKKL-GVPEERIIRIATSDNFWQMGPTGPCGPCTEIFYDHGDHIWGGPPGSPEEDGDRFIEIWNVVFMQNEQFEDGSMKALDMQSIDTGMGLERIGALLQGSHDNYDTDLFKTLIEASAHATSVEPYGDKNVHHRVIADHLRSTSFLIADGVMPSNDGRGYVLRRIMRRAMRHAHLLGAKDPVMHQLVPSLVGQMGQAYPELGQAQAMIEETLLLEETRFKQTLDRGLKLLDDEVSGLDEGAQLSGEAAFKLYDTYGFPLDLTQDALREQGREVDTDGFTAAMEQQKAKARAAWSGSGDAADSTVWFDVLDGAEPTDFLGYDTEKAEGQILALVADGKLVDSLAEGATGWVVTNQTPFYAESGGQVGDEGVIRNRDDAAAVSDTRKDSKIIAHKVTVSKGTLATGEAVELEVGHGRRSTIRANHSATHLLHEALRNALGDHVAQRGSLNAPDRLRFDFSHGKALSAAELSQVEAEVNAYIRQNSSVSTRIMTPDDARAIGAQALFGEKYGDEVRVVSMGQQSGSGKGSDGSTYSLELCGGTHVRQTGDIGAFVSLGDSASSAGVRRIEALTGQAAMDYLSEQSAALSTVAGELKAPVSEVPARVKALMDERKALTNEVAQLRRELAMAGGAGQGGAESTDVNGVAFVAQVLTGVSGKDLPALIDEHKSRIGSGAVLLIVDAGGKAAVAAGVTADMTGKVSAVDLVKAAVVELGGKGGGGRPDMAQGGAKDVANADAAIKAAQAVIGGMGFRMGIVGLPNVGKSTLFNALTKTAAAQAANFPFCTIEPNVGDVGVPDARLDKLAAIAGSKQIIPTRMTFVDIAGLVKGASKGEGLGNQFLANIRETDAIAHVLRCFEDEDVTHVDGRVDPVSDAETIETELIISDMESLEKRLQNITRKVRGGDKEAVQQERLMKEAMAMLEQGKPARLVEVDDDDLKAWKMLQLLSTKPVLYVCNVDEGEASTGNHLSEQVAEMAAAQGNTHVVISAKIEEEISQLEDDEAEMFLSELGLEEAGLDRLIKAGYELLHLETYFTVGPKEARAWTIKSGTQAPQAAGVIHGDFERGFIRAETIAYDDYLACNGENGAKDAGKMRAEGKSYTVKDGDVLHFLFNTMVWKTLDDMDLAGKRVLTRVDINVPFEDGKVTDTTRIERIAATVQDILDRGGKPVLLAHFGRPKGERNMDMSLQSLIPALESVFGSPVVFASDCVGASAVVDALQDGQVALLENTRFHAGETKNDPELAAQMAKLGDIYCNDAFSAAHRAHASTEGLARLLPSCAGRLMQAELSALESALSEPERPVLAVVGGAKVSTKLDLLGNLVAKVDMLVIGGGMANTFLAAQGIDVGKSLCEHEMADTAREILAKAADAGCEIILPTDVVVAREFKEGAANETVAA---NACPADAMILDAGPEAVAAIKDAINKAKTLIWNGPLGAFEIAPFDIATNAAAKFAAERSAAGKLVSVAGGGDTVAALNKADAANDFSYISTAGGAFLEWMEGKELPGVAALS-MALPEFTMRQLLEAGVHFGHQTQRWNPRMGPFIYGGRNGIHIMDLTQTVPMLDQALKAIRDCVAKGGRVLFVGTKRQAAQPVADAAEKCAQYYMNHRWLGGTLTNWQTVSKSIGRLRQIDELMETGAEGLTKKERLGMERDQIKLQASLGGIREMGGVPDMLFVIDVKKEALAVAEANKLGIPVVAIVDTNCSPDGIDYIIPGNDDASRAITLYCDLASRAALDGMTGQMEAAGYDLGAFEEAPIEEVVAEEA-PAA-----EAPA---EGMTKRTSAKYKIDRRMGENIWGRPKSPVNRREYGPGQHGQRRKGKMSDFGLQLRAKQKLKGYYGDLTEKQFRRIFGEAERVKGDTGENLIALLERRLDAVVYRAKFVATVFAARQFVNHGHVLVNGKRVNIPSYRVKEGDVVEVREKSRQMVVLLEATQLSERDVPDYIEADHSKMSAKFVRSPGLADVPYPVVMEPNLVIEYYAQNMGNKTNPIGMRLQVNRTWDSRWYADTKDYGDLLLEDLAIRDFIHKECKQAGIARVIIERPHKKCRVSVHTARPGVIIGKKGADIEVLRKKLAAMTDSEVHLNIVEVRKPELDAQLVGESIAQQLERRVSFRRAMKRAVQNAMRMGALGIRVNVAGRLGGAEIARTEWYREGRVPLHTLRADIDHAQVEASTAYGIIGIKVWIFKGEIMEHDPAARDRKSQEVQDGPAPRGAGGRRMGRKRKGRDISGWLIVDKPAGMTSTSVVNKVRWAMDAKKAGHAGTLDPEATGVLAVALGEATKTVPYITEALKAYRFTVVLGQATNTDDAEGEVIETSDLRPSDEEIKEALHQFVGDIQQVPPQFSAVKIDGERAYKRARDGEEMEIAARALFVEELVMISRPDTDHVELEMICGKGGYVRSIARDLGETLGCKGHVLRLHRIWSGPFETSDGITVEQIDEMAKTSALDAYLKPLEVGLSDLPELRTTPEGAVKLRNGNPGMVFAADAEYGDEAWASLDGEPIAVGIYKAGELHPKRVFVRAESFKLGLTGSIGMGKSTTAQMFADLGCPVWDADAAVHRLYGIDGDATLAISDIWPEAVIDGMVSRDELRAIIAKDHTALPRIEAIVHPLVQADRTKFISQHIDNICVFDIPLLFETGGESEMDAVACVSVSSQIQRDRVLARGTMTAADLDRILARQMPNEEKCARSTYVIETDSLEHAKAQVAAVVADIKRQLANAM-LTDAILEDDRWQPIGLEQLSERAAVATLLHLNVAVDAHEIVVMGCNDSRIAALNQDFRDKPTATNVLSWPYEDLSSEESGGTPLLPEAE-----ELGDIAISFETCEKEARDQGKPLTDHVTHLLVHGVLHLLGYDHIRDADATVMEQLEVEILGKLGLPDPYRGYEGMARFIFITGGVVSSLGKGLASAALGALLQARGYSVRLRKLDPYLNVDPGTMSPFEHGEVFVTDDGAETDLDLGHYERFTGVPATRTDSISSGRVYTNVLEKERRGDYLGKTIQVIPHVTNEIKDFISIGEDDVDFMLCEIGGTVGDIEGLPFFEAIRQFANDKARGQCVFMHLTLLPFIKASGELKTKPTQHSVKELRSIGLAPDILVCRSEGPIPKKEREKLALFCNVRPDSVIAAQDLKSIYEAPLAYHREGMDQAVLDAFGIAPAPKPNLTKWEDVADRVFNPEGEVTVAIVGKYIQLEDAYKSIAEALTHGGMANRVKVNIEWVDAEMFEREDAAPHLAKYNAILVPGGFGERGTEGKIKAAQFARENNVPYLGICLGMQVAVIEAARNVAGVAKAGSEEFDHEAGEKRFEPVVYHLKEWVQGNEKVNRKVTDAKGGTMRLGAYDAVLTEGSLVEKIYGKQTIDERHRHRYEVDTAYREQLEKVGMRFSGMSPDGKLPEIVEWPNHPWFIGVQFHPELKSKPFDPHPLFKDFVRAAKEASRLVMTSPLI-LGLESSCDDTAAALLR-----GRDVLASVVLGQTQLHAEFGGVVPEIAARAHAERLDGAIEQALDEAKVTLPDIDAIAVTAGPGLIGGVLSGVMMAKGLSTGLGKPLIGVNHLAGHALTPRLTDSLTYPYLMLLVSGGHCQFLVVRSAQDFTRLGGTIDDAPGEAFDKTARLLGLPQPGGPSVEHVARNGDEKRFRFPRPLLDRAGCDLSFSGLKTALLRQRDQLIAEKSGLTEQDRNDMCAGFQAAVRDVLAEKTRRAIALYLAESPAEPALAVAGGVAANMALRTVLESVCADSSIRFTAPPLKLCTDNAAMIAYAGSELFAAGITDDMTLSARPRWPLDKTAVPLIGSGKKGAKGMAKNRSNEQSGPRQRQLRVAELIRRKLSEILLRGEIHDPDLNRLNLTVGEVRVSPDLRIATVYVVPLGGKGEDEMNAILRRNKGEIRHQVVHGLKLKFAPELRFRFDDTFDRMDETRRLFENEDVRRDLDE-

>'Roseobacter-denitrificansOch-114'

MNAKELHDKTPDQLREELANLKKTSFNLRFQQATGQLENPAQIRKARRDAARVKTILNQKAASAAASEMATADLLSMNSKKSADKQKALDSALAQIERQFGKGSIMKLGAEGAVQDIQASSTGSLGLDIALGIGGLPMGRIVEIYGPESSGKTTLTLHCVAEQQKAGGVCAFVDAEHALDPTYAKKLGVDLDELLISQPDTGEQALEITDTLVRSGAVNMVIVDSVAALTPKSELEGDMGDSSVGVQARLMSQAMRKLTGSISRSNCMVIFINQIRMKIGVMFGSPETTTGGNALKFYSSVRLDIRRIGALKDRDEVVGNHTRVKVVKNKVAAPFKQVEFDIMYGEGISKMGELLDLGVAAGVVDKSGSWFSYGDERIGQGRENAKGFLRENVQMALEIEDKIRAAHGLDFDAHEKKDDDILEAMTRYTPAEIEARWQKAWRENEVFKAIRSADKPKYYVLEMFPYPSGRIHMGHVRNYTMGDVIARYKLATGHNVLHPMGWDAFGMPAENAAMAIGGHPKDWTYGNIKDMRDQMKPLGLSIDWSREFATCDPEYYGQQQALFLDFLEAGLVYRKNAIVNWDPVDMTVLANEQVEQGRGWRSGALVERRELTQWFFKISDHSEELLSALDTLENWPAKVRLMQENWIGKSRGLQFAFSTIDGPDGHDRIEVYTTRPDTLLGASFVGISPDHPLAKLLERDNDNVAAFCAECRKGGTTEEAIETAEKLGFDTGLRVRHPFDTAAELPVYIANFILMDYGTGAIFGCPAHDQRDFEFATKYDLPIISTFLPSQDADEVLSEAFVPAKTEKVFYNRGFAGAEFQTGLEAIDAAIDFCESNGIGQGVTKYRLRDWGLSRQRYWGCPIPVVHCDDCGVVPEKKENLPIELPYDVSFDTPGNPLNRHPTWRDTPCPACGKPAQRETDTMDTFVDSSWYFARFTAPRAETPTVMEDAQYWMNVDQYIGGIEHAILHLLYSRFFARAMQMTGHLPQSAIEPFDALFTQGMVTHEIYQTRDANGRPVYHLPEDVTDGRL-ADGTEVEIIPSAKMSKSKKNVVDPLGIIASYGADTARWFVLSDSPPERDVEWTASGAEAAFKHLTRVWTLSERIGKMDKDAAGQGDEDLLRAMHVCIHDVTMGIESFGFNAAIAKLYAFTAKLQKSKAGYGAQRTAIMTLAQLMSPMTPHLAEDIWAHQGGDGLVTTAPWPRADEAMLVSDTVTLPVQINGKRRAEIVISADLSKEEVEKIALADPAVIRSLNGATPKKIIVVPGRIVNVVVMAITSANQLELLQTAEAVAREKMIDPGLVVEAMEESLARAAKSRYGAEMDIRVHIDRKTGRATFTRVRTVVEDELLENYQAEFTVEQAKQYMENPEVGQQLIEEVPPVEMGRIAAQSAKQVILQKVREAERDRQYEEFKDRAGTIINGLVKREEYGNVIVDVGAGEAILRRNEKIGRESYRPNDRIRVYIKDVRREQRGPQIFLSRTAPEFMAELFKMEVPEIYDGIIEIKAVARDPGSRAKIAVISYDGSIDPVGACVGMRGSRVQAVVNELQGEKIDIIPWNEDQPTFLVNALQPAEVSKVVLDEEAGKIEVVVPEEQLSLAIGRRGQNVRLASQLTGLDIDIMTEEQESARRQAEFELRTKLFVDNLDLDEFFAQLLVSEGFTSLEEVAYVEVDELLVIDGVDEDTANELQARARDVLEAQNKAALESARALGVEDSLIEFEGLTPQMIEALAKDDVKTLEDFATCADWELAGGWTTVDGERSKDDGVLEPFDVTLEEAQDLIMTARVMLGWVDPADLEPD--EDVAE-DAE-AEEAEVMAHKKAGGSSRNGRDSAGRRLGVKKYGGEAVIPGNIIVRQRGTKFWPAAGVGMGKDHTIFATVDGAVTFHKGLKNRTFISVLPVAEAAEMAKRWYSVSVLSNFEKKIAEQIRTSVAEQELEDQIDEVLVPTEEVIEVRRGKKVTTERRFMPGYVLVHMEMSDKGYHLVNSINRVTGFLGPQGRPMPMRDAEVNAILNRVQEGEEAPRTLIHFEVGEKVKVADGPFEDFDGMIEEVDDENQRLKVSVSIFGRETPVELEFTQVNKQSMSFTLAIVGRPNVGKSTLFNRLVGKRLALVDDQPGVTRDLREGAARLADLRFTVIDTAGLEDVTDDSLQGRMRRLTERAVDMADICLFMVDARVGITPTDLVFADILRKRAGHVVLAANKAEGAAADAGVIEAYSLGLGEPIRLSAEHGEGLNDLYTHLMPLADAYAERAAEDAPETDVALDEDSGDMEALRMPTANKPLQVAVVGRPNAGKSTLVNQILGEDRLLTGPEAGITRDAISLRTDWVGVIPMRIFDTAGMRKKAKVQEKLEKLSVGDGLRAVKFAEVVVVLLDAAIPFEQQDLRIADLAEREGRAVVVAVNKWDIEENKQAKLNELRESFERLLPQLRGAPLVTVSARTGRGLDRLHKAVLRAYEVWNRRVTTAQLNRWLAGMLEAHPPPAPQGKRIKLRYMTQAKTRPPGFVVMCSHPDKVPDSYNRYLVNGLRLDFDMPGTPIRLWMRGQNDANPYKGRKKAPPSKLRKHTDGRRKDMRLVFMGTPDFSVPVLEALVAAGHEIACVYSQPPRPAGRGKKDRPSPVQARAEALGLPVRHPVSLRSDEALADFAGLQAEVAVVVAYGLILPQAILDAPTRGCLNIHASLLPRWRGAAPIHRAIMAGDAQTGVCIMQMEAGLDTGPVLAREAVDIGPEETTAQLHDRLSALGAALIVDTLARLDQLEAVPQPEDGVTYAAKIDKAEAQVDWDQPAEDVDRLIRGLSPFPGAWTLLGDTRVKLLGSRIAEASGVPGTVRLNPLRVVCGTGAVELTQVQRAGKAVQSAADFLNGSAIEDGTLFKGKMSETAPYQVLARKYRPETFADLVGQDAMVQTLKNAFEADRIAQAFIMTGIRGTGKTTTARIIAKGMNCIGPDGTGRPTTDPCGTCEHCTAIMEGRHVDVMEMDAASNTGVANIREIIDSVHYRAASARYKVYIIDEVHMLSTGAFNALLKTLEEPPEHVKFIFATTEIRKVPVTVLSRCQRFDLRRIEPEVMIDLMRKIATAEGAEIAQDAMALIARAAEGSARDATSLLDQAISHGAGETTAAQVRAMLGLADRGRVLDLFDMILRGDTAGALTELSAQYAEGADPMAVLRDLAEITHWVSVVKITPEAAEDPTVSPDERSRGLEMAGALPMRVLTRLWQMLLKALDEVASAPNAMMAAEMAIIRLTHVADLPTPGELVRQLKADNPPPAR--GNTATQSPAPQG--NATAHATAR-PAPPSGSGTVAALAEQPNEALARFVSFDHVVELIRANRDVKLLVEVEGCVRLAQYQPGRIEFAPTENAPADLAQRLGQRLQLWTGNRWAVTIVNDASAPTIAEKRDAAELDLQSKALEHPLMQAVLTQFPKARITSIKTPEQIAAAAVANALPEVEDEWDPFEDSMPKMKTKSSAKKRFKVTATGKVIGGQAGKRHGMIKRTKKFIRDARGTTVLSEPDAKIIKGFMPYDRMNLFAEMRALVLECVDQMVTAGQLPADLNMTNVTVEPPRDAAHGDMATNAAMVLAKPAGKKPRDIAEALAQLLAADARIASAEVAGPGFLNLRIAPTAWRAVLLSVLDKGTDFGRADIGAGQKVNVEYVSANPTGPLHVGHTRGAVFGDALASLLDYAGFDVTREYYINDGGAQVDVLARSVYLRYLEAHGQEVAFPDGTYPGDYLIPVGQQLKDLKGDSYVGQPEEAWLQDIRTFATDAMMDLIRADLKSLGVEMDVFYSEKSLYGTGRIEAAIEDLKSKGLIYDGVLEPPKGKKPEDWEPREQTLFKSTEHGDDVDRPVMKSDGSWTYFAPDIAYHYDKVSRGYDALIDVFGADHGGYVKRMKAAVSALSDGKVPLDIKLTQLVKLFKNGEPFKMSKRAGTFVTLRDVVDQVGPDVARFVMLTRKNDAMLDFDFDKVMEQSRENPVFYVQYAHARVASVMRKAEAAGIAVDMDTLKAADLSLLNHEAELGLIAKLSEWPRLVETAARSNEPHRVAFYLYELSGVFHGLWNRGNDVPELRFVQD-DPKVTQAKIALARACSVVIAAGLGILGVTPAQEMRMSTIDHLPPLRSVIRDHDLSARKSMGQNFLLDLNLTAKIARQAGDLSACDVLEIGPGPGGLTRGLLAQGARRVLAIEKDARCLPALAEIAAVYPGRLEVMNGDALEIDPLSALTPPIRVAANLPYNVGTELLVRWLTPQIWPPYWQSLTLMFQREVAERIVARPGSKAYGRLAILAQWRADARIVMQLPPDAFTPPPKVSSSVVQITALPAPRYPADPYVLSKVVAMAFNQRRKMLRAALKGLGPDIEDRLLAAGIEPTERAERVSLEGFCALARAVAATMDLIAQIEAEQIAALGKDIPDFRAGDTVRVGFKVTEGTRTRVQNYEGVCIARNNGHGIAGSFTVRKISFGEGVERVFPLHSTNIESITVVRRGRVRRAKLYYLRSRRGKSARIAENTNYKPKSGASVMANSKRQLFIKRRLRVRNKLRKVNAGRVRLSVHRSNKNISVQLIDDVAGKTLASASTLEKDLGVVGKNNIEAAAKVGAAIAERAKKAGVSEAYFDRGGFLFHGKVKAVAEAAREGGLKIMFAVLKTGGKQYKVQAGDMLRVEKLAADAGETVQFNEVLMLGGENTVVGAPFVKDAGVQAEVVDQIKGEKVINFVKRRRKHSSKRTKGHRQKLTLVKVTDILASGADKSGVKAAMGAGSVSA----TAVAA--AAPKPTKKAAPAKAE--KA-EAAPA-----AGADDLKKLSGVGPALEKKLIEGGVTSFAQIAAWTEADVAAMDEKLSFKGRIEREGWIEQAKELAKGMSRTKGGTVTHARHKKVIKAAKGYYGRRKSTFKVARQAVDKANQYATRDRKNRKRNFRALWIQRINAAVRSHDEALTYSRFINGLNLAGIEVDRKVLADLAVHEPEAFGAIVKQAQDALAAMQVTETLNEGLKRGYAIKVTAAELDAKVNEKLAEAQPEVEMKGFRKGKVPMALLKKQFGQKVMGEAMQESIDGAMNDHFTSSGDRPAMQPEVKMTNENWKEGDDIEVSMSYEALPDIPDVDFSAIELERMTVKADDAAIDEALGSLAETAQNFENRKKGSKAKDGDQIVMDFLGKVDGEAFDGGAAEDYPLVLGSNSFIPGFEEQLVGVKAGEEKAVTVTFPENYQAENLAGKEAVFECTVKEVKKPVPAEIDDELAKKFGAEDLAALKGQIAERLEAEYAGAARAIMKRSLLDALDDIVDFDLPPSLVSAEAGQIAHQLWHEENPDVQGHDHPEITPTEEHTKLAERRVRLGLLLADIGQKAKVEVTDAEMSQAIMNQARQYPGQERQFFEFVQQNQQMQQQMRAPIFEDKVVDHVFAGAKVTEKEVSKDDLQKAVEALEEEMKLHELSDNDGAAKKRKRVGRGPGSGTGKMGGRGIKGQKSRSGVAIKGYEGGQMPLYQRLPKRGFNKPNRKAYAVVNLGLIQKFVDAGKIDASAAITEDALIASGLVRRKKDGIRVLAKGDVTGKLNIEVTGASKSAIEAVAKAGGSLTVT-APVAETSEMIQMQTNLDVADNSGARRVQCIKVLGGSKRKYASVGDIIVVSVKEAIPRGRVKKGDVRKAVVVRTAKEVRREDGTAIRFDRNAAVILNNNNEPIGTRIFGPVVRELRAKNFMKIISLAPEVLMRHARGYRRLNRTHEHRKALWANMAGSLIEHEQIKTTLPKAKELRPIIEKMITLAKRGDLHARRQAASKLKEDQYVAKLFDVLGPRYKDRQGGYVRVLKAGFRYGDMAPMAIIEFVDRDRDAKGAADRARLAAEENAEEMLQPKRTKFRKQFKGSIKGLAKGGSDLNFGTYGLKAIEPERITARQIEAARRAMTRHMKRQGRVWIRIFPDVPVTAKPIEVRMGKGKGSVDRWAAKVKPGRVMFEIDGVNDDVAREALRLAAMKLPIKTRVVVREDWMT-IKLYNTKTRRKEDFEPINKDDVRMYVCGPTVYDRAHLGNARPVIVFDVLYRLLRHTYGPDHVTYVRNFTDVDDKINARAAESGRSIAEITAETTQWFLDDMAAVGALEPSAMPRATQYIAQMVTMIEDLIAKGHAYAAEGHVLFAVESYSKYGALSGRSIDDMIAGARVEVAPYKRNPMDFVLWKPSDDATPGWAGPIGRGRPGWHIECSAMAHDLLGATFDIHGGGNDLMFPHHENEIAQSTCAG--HNFANVWMHNEMLQVEGKKMSKSLGNFFTVRDLLDQGVPGEVIRFVMLSTHYRKPMDWTEKKREEAEETLWKWKKLTDGAESSRRPHLEIIAALKDDLNTPLAISILHRKFASDDKNRFLSTLLLLG------GTW--RDGVDTVSLIEALIESIEKARAQKDWATSDRTRDGLESAGVKVQISKDGVSWERGPSFDPAKLEALKMAQVKSSSKSDPNYKVVAENRRARFDYAIESDVECGIILEGSEVKSLREGGANIAESYAAVEDGELWLVNSYIAPYKQAKTFQHEERRRRKLLISRKQLSDFWNATQRKGMTLVPLVLYFNHRGMAKIKIGVAKGKKLHDKRETAAKRDWSRQKSRLMKDHGMSAKAEHYDVIRKPIITEKATMTSENGAVVFEVAIDSNKPQIKEAVEALFGVKVKAVNTTITKGKVKRFRGQLGKRKDVKKAYVTLEAGNTIDVSTGLMSKDKNPRRVADNEAMAKLRMLRTSPQKLNLVAGLIRGKKVEQALTDLTFSKKRIAHDVKKCLQSAIANGENNHNLDVDELIVAEAYVGKNLTMKRGRPRARGRFGKIIKPFSEITIVVRQVEEQAMVSAVESMAANTSWSALGKATDLRNRILFTLGLLIVYRLGTFIPVPGIDGVALREFMEQAGQGIGGMVSMFTGGALGRMGIFALGIMPYISASIIVQLMTAMVPKLEALKKEGEQGRKKINQYTRYGTVFLATLQAYGLAVSLEAGDIVTDPGMFFRASCVITLVGGTMFLMWLGEQITARGIGNGISLIIFVGIIAEVPAAMAQFFASGRSGAISPAVIIGVIIMVIATIMFVVFMERALRKIHIQYPRRQVGMKVYDGGSSHLPVKVNPAGVIPAIFASSLLLLPVTISTFSGNSTNPIMSWLLANFGPGQPLYLAFFTTMIVFFTYFYTFNVSFKPDDVADNLKNQNGFIPGIRPGKKTSEYLEYVVNRVLVLGSAYLAAVCLLPEILRGEFAIPFYFGGTSVLIVVSVTMDTIQQVQSHLLAHQYEGLLEKSQLRGKS-KGRKKRSPVRRMKFLDLCKVYIRSGAGGGGCVSFRREKYIEYGGPDGGDGGTGGSVWAEAVDGLNTLIDFRYQQHFFAKNGQPGMGRQRTGKDGDDIVLRVPVGTEILDEDQETVICDLTEVGQRVQLARGGNGGFGNLHFKSSTNQAPRRSNPGQDGVERTLWLRLKLIADVGLLGLPNAGKSTFLAATSNARPKIADYPFTTLHPNLGVVGVDNTEFVVADIPGLIEGASEGRGLGDLFLGHIERCAVLLHLFDGTSETLIEDYHTIIGELEAYGVGLADKPRITVLNKIDALDEERRAMALKQLNNVCGGGVMAMSGVAGDGVTDVLRKLRGEISDEPLRHKPVEEKEPWRPMAAKLKKGDKVIVLAGKDKGKTGSISSVDPKSGKAIVDGINIAIRATRQSQTEQGGRIPKAMPIDLSNLAYVDANGKATRVGFKMEGDKKVRFAKTTGDVIDAMLRSGVIAKKVGMTRLFMEDGKQIPVTVLQLDKLQVVAQRTSEKDGYSAVQLGAGTAKAKRTSQAMRGHFAAAKVEPKRKVAEFRVDPENLIGVGEEITANHYFEGQFVDVAGTSIGKGFAGAMKRHNFGGLRASHGVSISHRSHGSTGQCQDPGKVFKGKKMAGHMGAARVTTQNLQVVRTDANRGLIMVKGAVPGSKGGWVTVKDAVKKPFPENAILPAALKSAAEEAEKAAEAAAAAAAAEAEAAAA----AAAAEEQAAMEAAEA--AEAKTDTVAEAEAEKKEGDAMALKSYKPTTPGQRGLVLIDRSELWKGRPVKALTEGLSKHGGRNNTGRITMRRKGGGAKRLYRIVDFKRNKMDVAATVERIEYDPNRTAFIALVKYEDGEQAYILAPQRLAVGDTVVASQKADIKPGNAMPFSGMPIGTIIHNIEMKPGKGGQIARAAGTYAQFVGRDGGYAQIRLSSGELRLVRQECMATVGAVSNPDNSNQNYGKAGRMRHKGVRPSVRGVVMNPIDHPHGGGEGRTSGGRHPVTPWGKPTKGAKTRNKKKASSQLIIRSRHAKKKGRMLDSANYTPRLKAVYRDTIRAALKEEFGYKNDMQIPRLDKIVLNIGCGAEAVRDSKKAKSAQADLTLIAGQKAMTTTAKKSIAGFRVREEMPLGAKVTLRGDRMYEFLDRLITIAMPRIRDFRGISGKSFDGRGNYAMGLKEHLVFPEIDFDKIDENWGMDIVIATTAQTDAEAKSLLKAFNMPFNSMKLDVIKLDGAKAGSVDLDEALFGLEPRADILHRVVRWQRNNAQAGTHKVKTRSEVNYSTKKIYRQKGTGGARHGARSAPIFRGGGVYKGPKVRSHGHELTKKFRKLGLCHALSAKMKAGELVIIDEVSSDGKTAALAKQVANLGWKRALIIDGATVNETFAQAARNIDGLDILPTMGANVYDILKRDTLVITKAGVEALEARLKMENVVLIIHLILALGLIAVVLLQRSEGGGLGMGGGGGGAMTGRAAATALGKVTWLLAIAFICTSITLTIFAAENASGSSVIDRLGVTPPAADRGETPALPDSDLLLPPSADENAPLVPTLDMAFFKKLKDKLFKSSSRIDEGLEAIVDDGGVEETASAVAAPEAAAEPSDASAPEPDPAEPEPEPEPEPEPEPEPEPEPEPEPEPEPEPEPEPEPPEPEPEPEPERTPPTPEPEVVAPLPRATAPIASDLSEPTP--VVRDKPGLLGRLMGRQAPATVVRRTLDDDMLEQLEELLISADMGVDTALRVVANMAEGRVGKKLSVQEIKELLASEIAQIMEPVAKPLPLYPRTPQVVLVVGVNGSGKTTTIGKLASQFRAAGKKVVIAAGDTFRAAAVEQLQVWGDRAGVPVLTAPEGSDPASLAFDAMLKAQRDGADLLLIDTAGRLQNRTDLMEELAKIVRVIRKNDETAPHNTLLVLDATTGQNALNQVKVFSDISDVSGLVMTKLDGTAKGGVLVALADKFGLPIHAIGVGEQIDDLDAFDPEDFADALMGLERMTKEQWGQLQQRLLKTVGQNNYKNWIEPIEFGSTQDGVATFEVPTNFLGNYVSQNFADLILHEVRQEDPAVRRLRFAVSHVNATTK-PARPAQATPRAPAEKTP--RSTLSTAPLDARFTFDNFIVGKPNELAHAAAKRVAEGGPVTFNPLFLYGGVGLGKTHLMHAIAHELRLRRPEMNVLYLSAEQFMYRFVQALRDRKMMDFKEIFRSVDVLMVDDVQFIAGKGSTQEEFFHTFNALVDQNKQIIISGDRAPGEIKDMEERVKSRLQCGLVVDLHPTDYELRLGILQSKVEQQRKNYPGLDISDGVLEFLAHRISTNVRVLEGALTRLCAFASLVGREIDMELTQDCLSDVLRASERKITVEEIQRKVSDHYNIRLSDMIGPKRLRSYARPRQVAMYLSKKMTSRSLPEIGRRFGGRDHTTVMHGVKRIEELKIQDGQIAEDLELLRRALEEMLGIGTISRKIFGSPNDRKVKATRPVIAQINALEPEFEKLSDEGLKEKTEEFRKRALEGESLDALLPEAFANCREAARRALGLRAFDTQLMGAVFLHQGNIAEQKTGEGKTLTATFAAYLNGLTGRGVHIVTVNEYLAKRDAAWMGSVFGALGLTTGVAYSDMPEDEKRKAYASDITYATNNELGFDYLRDNMKSELDQIYQKQHNFAIVDEVDSILIDEARTPLIISGPSQDRSEMYKTVDAVIPQVRDDHFELDEKTRNVTFTDEGNEFLEEILHAQGLLEEGQSLYDPESTTVVHHVNQGLRAHKLFQRDKDYIVRDDNVVLIDEFTGRMMPGRRLSDGLHQAIEAKEGVDIKPENITLASVTFQNYFRLYDKLSGMTGTALTEAEEFMEIYGLGVVEVPTNKPIARVDEDDQVYRTAREKYEAMLEKIKESNAKGQPVLVGTTSIEKSEMLSNMLKQAGITHNVLNARQHEQEAQIVADAGKYGAVTIATNMAGRGTDIQLGGNVDLQVMNALTADPDADPEALRASIEAQHADEKAKVLAAGGLYVLASERHESRRIDNQLRGRSGRQGDPGRSSFFLSLEDDLMRIFGSERLEKVLKTLGLKEGEAIVHPWVNKSLERAQSKVEGRNFDIRKQLLKFDDVMNEQRKVIFGQRREIMEAKDLSEITTDMRHQVIDDFIEQYLPPNTYADQWDAEGLYAAVQEQLGIDVPVMDWVEEEGVDDEAIRERLVEATDKLMTEKAGQFGAENMRNIEKQLLLQAIDTKWREHLLTLEHLRSVVGFRGYAQRDPLNEYKNESFQLFESMLDSLREDVTQKLSQIRPMTEEEQQAMIEQIRAQQAAAQAAAAGPTVSAQAGPVGPADAAVAFDENDPSTWGNPGRNEPCPCQSGKKFKHCHGRLIMKLSAELKSFQKRLGHQFNQPALLAQAVTHASMSTANRGDNQRMEFLGDRVLGLVMAEALLRLDNNATEGQLAPRFNALVRKETCADVAREIDLGAVLRLGRSEMLSGGRRKQALLGDAIEAVIAAVYQDAGFDAARALILRLWGDRVHEVEEDARDPKTALQEWAQARGLQPPRYEEVSRKGPDHAPIFTISVRISTGETDQATAGSKRQAEQAAAQALLAKLETSGMAKLGKRARAAREAFAGKENVTVEEAVALIKANSSVKFDETVEIAMNLGVDPRHADQMVRGVVGLPNGTGKTVRVAVFARGAKADEATAAGADIVGAEDLMETVQSGKIEFDRCIATPDMMPIVGRLGKVLGPRNLMPNPKVGTVTMDVEAAVKAAKGGEVQFKAEKGGVVHAGVGKLSFDEAKLVENIRAFVGAVSKAKPTGAKGAYMKKIALSSTMGPGVSVAIEDANVEMSEDFMLDTDDIERRMDGAISSLKTEFASLRTGRASASMLEPVMVDAYGQRTPINQVGTVNVPEPRMVTINVWDKGLVGKVEKAIRESGLGINPQLNGTIIMLPIPELNEERRRELSKVAGQYAEHARVSIRNIRRDGMDQIKKAKADGLSEDDQKLWEGEVQEITDRYIKGIDDQLATKQAEIMQVMAKKLVGSMKLQVPAGQANPSPPVGPALGQRGINIMEFCKAFNAKTADMEQGAPCPTVITYYQDKSFTMDIKTPPASYYLKKAAKLSSGAKTPSREVVGYVSSKQVREIAEAKMKDLNATSIEAAMKIILGSARSMGIEVKMFENLSERLSGVFDRLTKQGALSEEDVKTALREVRVALLEADVSLPVARDFVKAVQEKATGQAVTKSITPGQQVVKIVHDALIDVLKGEGEPGTLKVDNPPASVLMVGLQGSGKTTTTAKLAKRLKEKDGKRVLMASLDVNRPAAMEQLAILGAQIGVDTLPIIAGQDPVAIAKRAKTQAGLGGYDVYMLDTAGRLSIDEELMQQVEAVRDVASPRETLLVVDGLTGQDAVHTAQNFDERIGISGVVLTRMDGDGRGGAALSMRAVTGKPIKFVGLGEKMDALETFEPDRVAGRILGMGDIVALVEKAQETLELEQSEKMVKRMMKGHFTMNDLRMQLEQMQKMGGMQGMMGMMPGMGKMAKQVEDAGFDDNILKRQIALIQSMTKKERANPQILQASRKKRIAKGAGLEVSELNKLLKMHRQMGDMMKKMGKMGKGGMMKQAMKGMFGGGGPSPQDMAAGMDPKALEAAAKQLGGGMPGQ---FGGGAALPPGLSGFGKKKMIPLDRLRQITERFQYLEAAMAAGDGAADIAALAKEYSDLRPVVEQISAYQQLLSDLEDAAEMLGDPDMAPLAEEEIPRLKSALPQAEAALQLALLPKDKADAKPAMLEIRPGTGGDEAALFAGDLFRMYQRYAETQGWKVDLIELQETELGGIKEVVAHIKGADVFARLKYESGVHRVQRVPSTESGGRIHTSAATVAVLPEAEDVDIHIDANDLRIDTMRSSGAGGQHVNTTDSAVRITHLPTGLVVTSSEKSQHRNREIAMQVLKARLYDLERSRVDDERSADRAAQVGSGDRSERIRTYNFPQGRMTDHRINLTLYRLDAVLQGDLDEIIDALTAHAQAQLMADMGQVDRAQKEQLVDELGQIFESSGVVVVSHYAGLTVAEMQDLRARARDAGGSVRVAKNRLAKIALEGKPCASIADLLTGMTVLTFSEDPVAAAKVAQDYAKENQKFVILGGAMGENALDAAGVEAVSKMPSREELIATIAGMLGAPASNIAGAIGAPASNIASILSTVEDKAAAMKTFSATPADIDKKWILIDAEGVVLGRLASIVATRLRGKHKPSFTPHMDCGDNVVIINADKVQLTGKKREESHYWHTGHPGGIKSRTKAQILEGKHPERIVTLAVKRMLPGNRLSRQIMTNLRVYAGSEHPHEAQSPEVLDVTSMNSKNTRSA----MCDLDAEIDAHFVNDIPKALGVAVSGGSDSMALLVLLCGFAERNGVALSCATVDHGLRPEAADEARSVAGYCAAHDVPHVTLKWEGWDGTGNTQNAARVARYRLLADWARGQGIADIALGHTQDDQAETVLMGLARASGVDGLSAMPARRDDRGLCWHRPLLATSRAGLRGFLSANGVAWCEDPSNDDAQFERIKARRALTTLAELGVDRASLAQVAQNMAQARAALEQQTRRAIGDLVSLLGGGAMMPWSGFQTLPSEIARRVLMAAIDWIAAPPHAPRQKALTAALSALRASGSATVAGCRLILKDDRFWMFREFQAVAHARADASQLWDNRWKVTGGQG-KQAREIRALGEIGLQQHPEWRTMGLPRALLLATPAVYQGDVLIAAPVVQNTARWRADVIKRPGCLFDEVFGHMADLKKLAEEIVGLTLLEAQELKTILKDEYGIEPAAGGAVMMAGPADAGAAEEEKTEFDVVLKSPGASKINVIKEVRGITGLGLKEAKDLVEAGGKIKEGCDKAEAEEIKGKLEAAGAEVELAMSRIGKKPVDLPAGVSASVSGQTIEIKGPKGTRAFKATDDVTLTVEDNVVTVTPRGKSKRARQQWGMSRTMVANLVTGVSEGFKKELEIQGVGYRAAMTGNTLKLNLGLSHDVDYTPPAGVTVTAPKQTEIVVEGIDEQLVGQVAANIRQWRKPEPYKGKGIRYKGEFVFRKEGKKKMQVILLERVAKLGQMGEVVDVKPGYARNFLLPQGKALSASKANIEAFEQQKAQLEARNLETRKEAEALAAKLDGQQFIVIRSASDSGALYGSVTTRDAAEAATEAGFTVDRKQVVLS-PIKELGLHAVQVVLHPEVDATIHLNVARSVEEAELQASGKSIQELAAEEEAAAEFEIQELFDDIGAAASEDEELAETAGVAPAEPS--EEDDSMSDTDGKKTLGLRGGARPGNVKQSFSHGRTKNVVVETKRKRVVVPKAGATTSAG-GKAPIGDPSRRPAGISDAEMERRLKAVKAAKAREAEEEAARIAEEKARAEERERRRAEQEERERAEREREESLKAKAEEDKRRKDEAEAAAKAAAAPAAEPVVQRPA-AKAAPEPAPRKQDRDRDNK-RGGKGNDDSRRSGKLTLNQALAGGEGGRQRSMAAMKRKQERARQKAMGGQVEREKVVRDVQVPEAIVVSELANRMSEKVGEVVKALMNNGMMVTQNQAIDADTAELIVQEFGHRIVRVSDADVEDVIKEVEDDEADLKTRPPVVTIMGHVDHGKTSLLDAIRKAKVVAGEAGGITQHIGAYQVKTDSGQLLSFLDTPGHAAFTSMRSRGAQVTDIVVLVVAADDAVMPQTIEAINHAKAAEVPMIVAINKIDRPAADPTKVRTDLLQHEVIVEQMSGDVQDVEVSAITGQGLDDLLEAIALQAEILELKANPNRAAQGAVIEAQLDVGRGPVATVLVQNGTLRQGDIFVVGEQYGKVRALINDQGERVKEAGPSVPVEVLGLNGTPEAGDVLNVTSTEAQAREIASYRANAAKDKRAAAGAATTLEQLMANAKADEDVSELPILVKADVQGSAEAIVQAMEKIGNDEVRVRVLHSGVGAITETDVGLAEASGAPIMGFNVRANASARNTANQKGVELRYYSIIYDLVDDVKAAASGLLSAEIRENFIGYATIKEVFKVTGVGKVAGCLVTEGVARRSAGVRLLRDNVVIHEGTLKTLKRFKDEVAEVQSGQECGMAFENYDDIRADDVIEIFEREEITRTLTMAKPKKTPRPKAQTPKGFRDYFGAEVSQRAEMLQRIAGVYHRYGFDALESSGVETVEALGKFLPDVDRPNEGVFAWQEDADADKPGDWLALRYDLTAPLARVYAQHQNDLPKPYRRYAMGPVWRNEKPGPGRFRQFYQCDADTVGASSVAADAEICAMLCDCLEAVGIERGDYVVRINNRKVLNGVLEVIGALGDETAAKVGIVLRTIDKLDRLGKEGVRSLLGPGRKDESGDLTFGADLSKAQIDVVMSFLEAKGGTNSETLINLREIVGRSRIGKEGVDELEKISELLSAGSYGPDRIVIDPSVVRGLGYYTGPVYEAELTFEIQDDKGRTRNFGSVAGGGRYDDLVKRFTGQEVPATGVSIGVDRLLAALHAKGRLDTTDVGPVVVTVMDRDRMADYQAMVAELRQAGIRAEVYLGNPKNFGNQLKYADTRNSPVAVIEGGDEHEKGVVQIKDLILGAEIAKSATLEEWKDRPSQFEVPRAELVAKVREILAQHGMDDLKQKYLSQIAAAQDEAGLEAIRLAAVGKKGEVALKMRELGKMTPEERQVAGPALNALKDEINSALAAKKAGLADAALDARLRDEWLDVTLPARGRPRGTIHPVSQVTEEVTAIFGEMGFSVAEGPRIDTDWYNFDALNIPGHHPARAEMDTFYMTRAEGDDRPPHVLRTHTSPVQIRTMEAQGAPLRIICPGGVYRADYDQTHTPMFHQVEGLAIDKDISMANLKWVLEEFFAAFFEIDGIKTRFRASHFPFTEPSAEVDIQCSWIDGQLRIGEGDDWLEVLGSGMVHPKVLAAGGIDPDQWQGFAFGMGIDRIAMLKYGIPDLRAFFDSDLRWLRHYGFASLDQPTLHGGLSRMEMAMDAGLDLVEISPNANPPVCKIMDFGKFKYEQQKRESEARKKQKIIEIKEVKFRPNTDTNDYDVKMRNVFRFLENGDKVKITLRFRGREMAHQNLGRELLERVAEDTKEAGRVENFPKMEGRQMVMLIGPLPRMKFTLSWLKEHLDTSATLDEITYALTDLGLEVEGVEDRAGKLRDFTIGYVKSAEKHPDADRLKVCQVATDEGDKQIICGAPNAREGITVVVAKPGVYVPGIDTTIGVGKIRGIESFGMMASEREMELSEEHDGIIELPSGEVGRSFVDWLAEHDPAKVDPVIEIAITPNRPDALGVRGIARDLAARGLGTLKPRDVAAVEGVFPCPVSITIEADTLEQCPVFFGRVIRGVKNGPSPQWLQDKLRAIGLRPISFLVDVTNFFTYDRNRPLHVFDADKIEGKLRVHRAKGGETLVGLDEKEYTFAEGMTLISDAANVESIAGVMGGLATGCTAETTNVFIEAAYFDPVRTAYTGRALKINSDARYRFERGIDPEWTPYGIEHATHMILEHAGGEPSEVIVAGKIPDTNRAYKLDAARVQSLVGMDIPESSQRQTLTALGFRLEGNMAHVPSWRPDVQGEADLVEEVARIASLTKLKGRPLPRLDEGVSRPILSPIQRREAMARRTCAALGYNECVSYSFIDQASAKLFGGGEDVTRLENPISSDMSHMRPALLPGLLQAAARNQARGNMNLALFEVGPAFHGGEPGEQHLLVSGVLIGRTEPKDVHGASRAVDVYDVKADAEAVLAAIGAPAKVQILRGAAPWWHPGRHGMICLGPKKVLGVFGEIHPRVLAAMDVKGPAMGFTIWPAEVPLPRKSGATRAALQISDLQAVERDFAFVVDSGVEALTLVNAAMGADKALIEDVRVFDEFIGGSLGEGKKSLALTVRMQPATQTLTDKDIEAVSAKVVEKVIKATGGTLRAMHMYRSHTCADLSAQDVGKTVRLSGWVHRVRDHGGVLFIDLRDHYGITQVLCDPDSPVFEQMEQVRAEWCIRVDGVVKARDASLINPKISTGEIELFVKDLEVLGASEELPLQVFGDQEYPEETRLKYRYLDLRRANMQANMKLRSDVVASLRQQMWKADFREFQTPIITASSPEGARDFLVPSRLHPGRFYALPQAPQQFKQLLMVSGFDKYFQIAPCFRDEDPRADRSPTDFYQLDIEMSFVTQQDVFDVIQPVLTQVFEQFGNGKAVDQEWPQISYKDAALWYGSDKPDLRNPIKMQSVSEHFAGSGFAIFANLLEQEGTEIRAIPAPGGGSRKFCDRMNAFAQKEGLPGMGYIFWRDQ-GA--GMEAAGPLAKNIGPERTEAIRQQLGLGVGDAAFFLGGKPKAFETVAGKARNHIGMELGLTDQNRFAFAWIVDFPIYEKDSETGRIDFEHNPFSMPQGGLEALQGDPLSVVGYQYDLSCNGYELVSGAIRNHKPEIMFKAFEIAGYGEDEVRKRFGGMVNAFQYGAPPHGGCAAGIDRIVMLLAEEANIREVILFPMNQRAEDLMMSAPSEPESEQLMELGLRVIPKDMTPLSHIRNFSIVAHIDHGKSTLADRLIQSTNTVADRDMKEQMLDSMDIERERGITIKAQTVRINYTAQNGEEYVLNLIDTPGHVDFAYEVSRSMRAVEGSLLVVDSTQGVEAQTLANVYHAIDADHEIVPVLNKIDLPASDCDRVAEQIEDVIGIDASGAIQVSAKTGQGIMETLESIVQNLPAPKGDRDAPLKAMLVDSWYDSYLGVIVLVRIMDGVLKKGDRIRMLSNNSTHNVDRIGVFRPEMTAIDELGPGEIGFLTASIKQVRDTRVGDTITHEKKGTEVALPGFKPSQPVVFCGLFPVDSSEFEELRDSIEKLALNDASFSYEMETSAALGFGFRCGFLGLLHLEVIRDRIEREYDIELITTAPSVIYHVYMKDGAMIELHNPADMPDLTLVDHLEEPRIKATILVPDDYLGDVLKLCQDRRGIQQDLTYAGSRAMVVYDLPLNEVVFDFYDRLKSVTKGYASFDYAMIGYRTDNLVKMSILVNDEPVDALSIMVHRERAEGRGRAMVEKLKDLIPRHMFKIPIQAAIGGKVIARETLSAMRKDVTAKCYGGDATRKRKLLDKQKAGKKKMRQFGKVDIPQEAFISALKMDGMSLPPGFLDELRTRLSLTQVVGRKVMWDARKSNQGKGDMWAPCPFHQEKTASFHVDDRKGFYYCFGCHAKGDAISFVCETENVGFMEAVRILAQEAGMPVPERDLQAQQKADRRTQLAEVMELAVKYFRLQLNTGLAAEARGYLERRGLSAQVLERWEIGFAPDQWQGLWDHLKAKSVADDLILAAGLAKPSTKGGRPYDTFRGRIMYPIRDARGRAIAFGGRAMDPNDNAKYLNSPETELFDKGRSLYNQKEARTAAGKGQPLIVAEGYMDVIALDAAGFTAAVAPLGTAITENQLQMLWRIAPEPIVALDGDTAGLRAAMRLIDLALPLLEAGRSLRFAIMPAGKDPDDLLREQGAGALQKLLDEALPMVQLLWQRETEGGVFDSPERKAALEKALRDKVALIKDPSIQNHYKQELKDLQWQLFRPQRQKNKGWRK----G--GRWGASVATPLQSTKASALASAQTIDVTENLREAVLLAAFATCPSVIEEFESDLERMTCSDHTHQALRDILLRHMSATAAEMRAVISETLGPDALENLLTQSHVAIVPCIRRPGDVEKTRMTVAEEFAKLSATRGLNAEIEEASEDMSGLADEGLTWRLSQAAKAAENARKSVHEDRAEYETGANGARISRDERNTLDALLEAIKSQKPHG--MCADTTDYKSTLNLPKTDFPMRAGLPKREPDWLARWERIGIYDRLREKDGRTPFTLHDGPPYANGHLHIGHALNKTIKDMIVRSHQMMGHDARYIPGWDCHGLPIEWKIEEQYRQKGRNKDDVPINDFRAECRKFAAGWVDIQREEFKRLGVTGNWEKPYLTMDFHAERVIAEEFMKFLMNGTLYQGSKPVMWSPIEQTALAEAEVEYHDKESFTIWVKFRV---GEMPEDLMGAHVVIWTTTPWTIPSNKAVVYGENISYGLYEITGRPQENWVKIGDRYILADKQAEEVLTRARLDEGMWRRLRDVTNDELAGIKLAHPLKEKEGADGEWDGLRDFRAADFVTDTEGTGFVHCAPSHGMEEYELYRDLGMLQDVITYNVTDEGMFRESLPFFGGKYILNRKGKEGDANTAVINKLAEVGGLLARGKIKHSYPHSWRSKAPIIYRNTPQWFAAIDKPMQIGDDEF--TIRARALREIDRVNWTPQSGRNRLHSMMEARPDWVLSRQRAWGVPLTCFTRKGALPTDPDFLLRNEDVNQRIVDAFEAEGADVWYEEGAKERFLGGIVDPDDYDQVMDILDVWFDSGSTHAFTLRDRADGSEDGIADVYMEGTDQHRGWFHSSLLQSVGTTGRSPYRNVVTHGFTLDEKGMKMSKSLGNTIVPEKIVQQYGADILRLWVAQTDYTLDQRIGPEILKGVADSYRRLRNTMRYMLGSLTDFTQSDRMDPADMPELERWVLHRLAELDKTVRDGYAAYDFQGVFQAVFTFATVDLSAFYFDIRKDALYCDGDTVQRRAARTVLDILFHRLTTWLAPVLVFTMEEVWLQRFDGPDSSVHLQDFPETPAAWLDPDLAAKWAGVRSARRVVTAALEVERTNKVIGASLEAAPTVHVSDAALRATLDAVPFEDICITSQITLTGDAAPDGAFRLPETDGIAVVFRKAEGGKCARCWKILPDVGSHSHAGVCGRCDSAMSMAQSQNIRIRLKAFDYRVLDTSTQEIVNTAKRTGASVRGPIPLPNKIEKFTVLRGPHVDKKSRDQFEIRTHKRLLDIVDPTPQTVDALMKLDLAAGVDVEIKLQSMAEEINTLEEL-----GAVAGVEAAPQVEAVVREPVRDDLGRSYATGKRKDAIARVWIKPGSGKVTVNGKPQNEYFARPVLQMILAQPFSVSGTDGQFDVVATVKGGGLSGQAGAVKHGISKALQLYDPSLRGALKAAGFLTRDSRVVERKKYGKAKARKSFQFSKRMPTIQQLIRKPRQPKIKRSKSMHLQECPQKRGVCTRVYTTTPKKPNSAMRKVAKVRLTNGFEVISYIPGESHNLQEHSVVLIRGGRVKDLPGVRYHILRGVLDTQGVKDRKQRRSKYGAKRPKMARDKVRVKKKASKNIAAGVAHVNSSFNNTKILISDVQGNAISWSSAGTMGFKGSRKSTPYAAQMAAEDAGRKAQEHGVKTLEVEVQGPGSGRESALRALAAAGFNITSIRDVTPMAHNGCRPPKRRRVMKPMRRGLLIILSSPSGAGKSTLARRLRDWDTDIEFSVSATTRAARPGEVDGREYHFVDEAAFKSQVSNGGMLEHAHVFGNFYGSPSGPVQEAINAGRDVLFDIDWQGAQQITNSALSAHTLSIFLLPPSIRELRNRLEKRAQDDAATIARRMDKSWDEISHWGSYDYVLVNDDLDTTEAHLKTIVTATRLRRSQQPGLTEHVRHLQSEFERLSMAQSFLGQKRLRKYYGKIREVLDMPNLIEVQKSSYDLFLNSGEAEVPTDGEGIAGVFQSVFPIKDFNETSVLEYVKYELEKPKYDVEECQQRDMTYSAPLKVTLRLIVFDVDEDTGAKSVKDIKEQDVFMGDMPLMTPNGTFVVNGTERVIVSQMHRSPGVFFDHDKGKTHSSGKLLFACRIIPYRGSWLDFEFDAKDIVFARIDRRRKLPVTTLLYSLGLDQEAIMDAYYDTITYKLEKNKGWVAPFFPDRVRGTRPTYDLVDAASGEVLFERGKKVTPRAVKKLIDEGKVTELLLPYEHIAGKFVAKDIINEETGAIYVEAGDELTLEYDKDGTLIGGTAKELVDAGITEIPVLDIDNVNVGPYMRNTMAQDKNMNRDTALMDIYRVMRPGEPPTVEAASALFDTLFFDAERYDLSAVGRVKMNMRLALDAEDTQRTLRREDIVACIKALVDLRDGRGDIDDIDHLGNRRVRSVGELMENQYRVGLLRMERAIKERMSSVEIDTVMPQDLINAKPAAAAVREFFGSSQLSQFMDQTNPLSEVTHKRRLSALGPGGLTRERAGFEVRDVHPTHYGRMCPIETPEGPNIGLINSLATFARVNKYGFIETPYRKVENRMVTDEVQYMSATEEMRHTVAQANAQLDEDGRFKNDLVSTRQSGDYTLAPSENVDLIDVSPKQLVSVAASLIPFLENDDANRALMGSNMQRQAVPLLQAEAPLVGTGIEEVVARDSGAAIMAKRAGIIDQVDAQRIVIRATSDLELGDAGVDIYRMRKFQRSNQNTCINQRPLVKVGDTVLKGQVIADGPSTDMGELALGKNVVVAFMPWNGYNYEDSILISERIARDDVFTSIHIEEFEVAARDTKLGPEEITRDIPNVGEEALRNLDEAGIVYIGADVEPGDILVGKITPKGESPMTPEEKLLRAIFGEKASDVRDTSLRVKPGDFGTVVEVRVFNRHGVEKDERALQIEREEVERLARDRDDEMAILDRNIYARLKSTILGKVAVKGPKGVSANAEITEDLLQMLPRVQWWQLALKDEGDAQVVEALNEQYEIQKRTLDARFEDKVEKVRRGDDLPPGVMKMVKVFVAVKRKLQPGDKMAGRHGNKGVISKVVPMEDMPFLADGTPVDFCLNPLGVPSRMNVGQILETHMGWAARGLGLNVDEALQEYRRSGDLTPVRDALSHAYGENVYDEGIAGMDEETLIEAAGNVTRGVPIATPVFDGAKEGDVNDALVRAGFSESGQSILFDGRTGEQFARPVTVGVKYLLKLHHLVDDKIHARSTGPYSLVTQQPLGGKAQFGGQRFGEMEVWALEAYGAAYTLQEMLTVKSDDVAGRTKVYESIVKGEDNFEAGVPESFNVLVKEVRGLGLNMELLDAEEDEMPLYEHVMIARQDLSNTQAEGLIEHFGTVLSDNGGKLVDHEYWGVKTMAYKINKNRKGHYAFLRSDAPAPAVHEMERLMRLHDDVMRVLTIKVDEHAELPSVQMQKREERGDRRERR-MDFAPLDSLTEKAAKKELSKLADLLDQANTAYHANDAPIISDADFDAYKRRNLAIEERFPHLKRADSPTDRVGAAPSAGFSKVVHSIAMLSLANAFDEADVSDFVQRIRKHLGLSDIEALSFTSEPKIDGLSLSLRYENGTLVQAATRGDGAIGENVTENARTIPDIPEQISNAPDVLEVRGEVYMSHADFEALNARQASAGAKLFANPRNAAAGSLRQLDARITQSRPLRFFAYAWGTLSEPLGKTQMQSIERLQSFGFQTNDLTEKCNGPEELLAQYRRIEERRASLDYDIDGVVYKVDDLDLQRRLGFRSTTPRWAIAHKFPAELAWTELQGIDIQVGRTGALSPVARLKPVTVGGVVVSNATLHNEDYIAGRDNKGDVIRGGKDIRVGDFVQVYRAGDVIPKVADVDLKKRPADAIPYVFPATCPECGSDALREAGDAVRRCTGGLICPAQAVERLKHFVSRAAFDIDGLGAKQIEQFYKDGWISEPADIFTLRDRFGSGIQQLKNRDGWGEKSANNLFDAIDDKRQIPLARLIFALGIRHVGEAASNLIAQHYSTFDAFEKSMLAAQDKDGEAWDDLLSIDGVGTVMAQSVIHAMGQAAERASIDRLVAQLDVQPSEAVVTDGSPVAGKTVVFTGTLSKMTRAEAKSRAESLGARVAGSVSAKTDFLVAGPGAGSKAKKAAELGVQTLDEDGWLALIEGLMIHKNWAELIKPQQLDVKPGNDPARQATVTAEPLERGFGLTMGNALRRVLMSSLQGAAITSVQIDNVLHEFSSVAGVREDVTDIILNLKGVSIRMEVEGPKRLSISAKGPGVVTAGDISESAGIEILNRDHVICHLDDGADVYMELTVNQGKGYVSAEKNKPEDAPIGLIPIDAIYSPVKKVSYDVQPTREGQVLDYDKLTMKVETDGSLTPDDAVAFAARILQDQLGIFVNFEEPESASRADEDDGLEFNPLLLKKVDDLELSVRSANCLKNDNIVYIGDLIQKTEAEMLRTPNFGRKSLNEIKEVLSGMGLHLGMDVEDWPPDNIEDLAKKFEDSFMAERDNRRGNRRD-RDEAPEFADRLVAINRVSKTVKGGKRFGFAALVVVGDQKGRVGFGKGKAKEVPEAIRKATEQAKRQMIRVQLREGRTLHHDMEGRHGAGKVVMRSAPEGTGIIAGGPMRAVFEMLGVKDVVSKSIGSQNPYNMIRATMDGLRKESSPRSVAQRRGKKVADILPKVD-AAPAPAETAEA-MNDPIADMLTRIRNSQLRGKSTVITPASKLRAWVLDVLADEGYIRGYEKITGADGHPAIEISLKYYEGEPVIRELKRVSKPGRRVYMGVNDIPVVRQGLGVSIVSTPKGVMSDQAARSANVGGEVLCTVFMHDIRAIRENPAAFDAALARRGEAPMSQAILALDTARRAKINAAENAQAEQNKASKEVGQAKAQGDEATFEKLRALVSEKKALVAEMQADAKALDAELTEKLSWIANIPADDVPDGADEDDNVEVRRWGHLPDFEFTPKEHYEIGGVAASMDFDTAAKTSGARFVMLKGAVARIHRALSQFMIDTHVDHNGLTEINSPVLVRDDAMYGTDKLPKFAEDSYQTTNGWWLVSTSEIPLTYTVAGDILDAAALPIRLTAHTLCFRSEAGSAGRDTAGMLRQHQFEKVEMVSITEPDQSEAEQQRMLRCAEDILERLGLPYRTVLLCTGDMGFGARRTFDIEAWLPGQNNYREISSVSTTGDFQARRMNARYKPADGGKPQFVHTLNGSGLAVGRCLIAVLENGQQADGSVVLPPVLAGYLGGKTVLSAEGTLVMAITAAMVKELRDSTGAGMMDAKKALTENNGDMEAAVDWLRTKGLAKAAKKSGRTAAEGLVAVKVQGGHGVAVEVNSETDFVGKNADFQKMVAGIADVALGASDIDALRAADMGGKTVEQAVIDAVAVIGENMSVRRMSSIDGENVVSYVHNAAAPGMGKIGVLVATNGGDEAFGKQVAMHIAAVNPASLSEADLDPAVVEKEKQVQMDIARESGKPEQVIEKMIIGRMKKYMSEVTLLNQSFVVNPDLTVGDAAKEAGATITGFVRLEVGEGIEVVKEDFAAEVAKAVKGMNQELTNNPFNPVAPLKTFDEIKVSLASPERILSWSFGEIKKPETINYRTFKPERDGLFCARIFGPIKDYECLCGKYKRMKYRGVVCEKCGVEVTLQKVRRERMGHIELASPVAHIWFLKSLPSRIGLMLDMTLRDLERVLYFENYVVIEPGLTELTYGQMMTEEEYMDAQDIYGMDAFTANIGAEAIREMLAAIDLEAEADQLRADLKEATGELKPKKIIKRLKVVESFLESGNRPEWMVLTVIPVIPPELRPLVPLDGGRFATSDLNDLYRRVINRNNRLKRLIELRAPDIIVRNEKRMLQESVDALFDNGRRGRVITGANKRPLKSLSDMLKGKQGRFRQNLLGKRVDFSGRSVIVTGPELKLHQCGLPKKMALELFKPFIYSRLEAKGLSSTVKQAKKLVEKERPEVWDILDEVIREHPVMLNRAPTLHRLGIQAFEPVLIEGKAIQLHPLVCSAFNADFDGDQMAVHVPLSLEAQLEARVLMMSTNNVLSPANGAPIIVPSQDMILGLYYTTLEREGMKGEGMVFGSVEEVQHALDAGMVHLHSKITARITQIDENGLEVMKRFDTTPGRIRLGALLPLNAKAPFDLVNRLLRKKEVQQIIDTVYRYCGQKESVIFCDQIMTMGFREAFKAGISFGKDDMLIPDTKWPIVGETRELVKDFEQQYMDGLITQGEKYNKVVDAWSKCNDKVTDAMMGSISASRKDADGSEMEPNSVYMMAHSGARGSVTQMKQLGGMRGLMAKPNGDIIETPIISNFKEGLTVLEYFNSTHGARKGLSDTALKTANSGYLTRRLVDVAQDCIVRMRDCGTESSITAVAAVNDGEVVSSLSERILGRVLAEDVVRPGTDEVLAAAGTLIDERMSDTIEEAGVASARIRSPLTCEAEEGVCAMCYGRDLARGTMVNTGEAVGIIAAQSIGEPGTQLTMRTFHIGGVAQGGQQSFLEASHSGKVVFDNAQTLENDAGEIMVMGRNMKLIIQDDNGEERASHKVGYGTKLFVTEGQKVARGDKLFEWDPYTLPIIAEKSGTAKFVDLVSGIAIKDETDDATGMTQKIVIDWRSATKGNELKPEIILVDADGEPVRNDAGNPVTYPMSVDAVLSIEDQAEVKAGDVVARIPREGAKTKDITGGLPRVAELFEARRPKDHAIIAEIDGYVRYGKDYKNKRRIAIESSEDPDHKVEYMVPKGKHIPVAEGDFVQKGDYIMDGNPAPHDILAIMGVEALADYMIDEVQDVYRLQGVKINDKHIEVIVRQMLQKWEIQESGDTTLLKGEHVDKQEFDQANAKALSKNGRPAKGEPILLGITKASLQTRSFISAASFQETTRVLTEASVQGKKDKLVGLKENVIVGRLIPAGTGGATMKVRRVAQDRDNVVIEARREEAEAAAALAAPVA--DDMVGGDVFDQPVHEEESRDMSRRHAAEKREVLPDAKYGDLVLTKFMNNLMIDGKKSVAETIVYNALTRVETKIKRAPIEVFHEALDNIQPSVEVRSRRVGGATYQVPVEVRPERRQALAIRWLIKAARSRNENTMEERLAGELLDAVQSRGTAVKKREDTHKMADANKAFSHYRWMPYAHSDTTE--AVLANAAPDVRAREKLEGGKRFKLATDFEPAGDQPTAIKELAAGVLSGERDQVLLGATGTGKTFTMAKVIEETQRPAIILAPNKTLAAQLYGEFKGFFPDNAVEYFVSFYDYYQPEAYVARSDTYIEKESQINEQIDRMRHSATRALLERDDVIIVASVSCIYGIGSVETYGAMTQDLKTGRMYDQRQVMADLVAQQYRRNDQSFQRGSFRVRGDSLEIFPAHLEDRAWKLSFFGEELESITEFDPLTGEKTNTFEQIRVYANSHYVTPKPTMQQAIISIKKELRTRLDQLVGDGKLLEAQRLEQRTNFDLEMLEATGVCNGIENYSRYLTGRAPGEPPPTLFEFIPDNAIVFADESHVSVPQIGGMYKGDYRRKFTLAEHGFRLPSCMDNRPLKFEEWDAMRPQSVFVSATPAAWEMDQTGGVFTEQIIRPTGLIDPEIEIRPVEMQVDDLLDEVRKVAADGFRTLVTTLTKRMAEDLTEYMHEQGIRVRYMHSDIDTLERIEILRDLRLGAFDVLIGINLLREGLDIPECGLVAILDADKEGFLRSETSLIQTTGRAARNAEGRVIMYADRITGSMERAIEETNRRRAKQIAYNEKHGITPTTVKKNVEDILAGLYKGDTDQSRVTAKIDKQMAGGNLQAVLDGLRTDMRKAAENLEFEEAARLRDEVKRLEAVDLAIADDPMARQYAVEKAVEDSAKASGRSTLGRGGMRGGVKRKRGRMASKPFFRRRKVCPFSGDNAPKIDYKDTRLLQRYISERGKIVPSRITAVSAKKQRELARAIKRARFLALLPYAVKMPKRILSGVVTSDANDQTVSVSVERRFTHPVLKKTIRKSKKYRAHDENNTFKKGDAVRIVECAPKSKTKRWEVLQAAEMANSPQAKKRARQNEARFQINKARRSRIRTFLRRVEEAIASGDKEAATAALRAAQPELMRGVTKGVFHKNTAARKMSRLAARVKVLGMSRSVWKGPFVDSYVLKKAEASRESGRNEVIKIWSRRSTILPQFVGLTFGVYNGRKHIPVNITEDMIGQKFGEYSPTRTYYGHAADKKAKRKMARIAGVNIPTAKRVPIALTYITGIGNTSAKQICEAVGIDPSRRVNELSDAEVLAVREHIDANYTVEGDLRREVQMNVKRLMDLGCYRGLRHRRNLPVRGQRTHTNARTRKGPAKAIAGKKKMAMKIRLARGGSKKRPFYRIVAADSRMPRDGRFIEKLGTYNPLLPKDSEERVKMDAERVQYWLGQGAQPTDRVARMLEAAGLREKAERNNPKKGTPGKKAQERAEEKAAK-------AAAPAEEEAAAEMSITAEEKARVMKEFATKEGDTGSPEVQVAILSSRIATLTEHFKTHKKDNHGRRGLLKMVATRRKLLDYTKAKDEARYQDLIKRLGLRRMQTLNDIRSTFLSYFEKQGHSVVASSPLVPRNDPTLMFANSGMVQFKNLFTGVETRAYKRATTAQKCVRAGGKHNDLDNVGYTARHHTFFEMLGNFSFGDYFKEDAIPFAWDLVTRELGIDKSRLYVTIYHTDEEAAAIWKKV-GVPEDRIIPIATNDNFWMMGPTGPCGPCTEIFYDHGDHIWGGPPGSPEEDGDRFVEIWNLVFMQYEQFEDGTRRDLAAQSIDTGMGIERVAALLQGTNDNYATDLMRSLIEASADASNTDPDGPCKTHHRVIADHLRSTSFLMADGVMPSNDGRGYVLRRIMRRAMRHAHLLGAKDPLMHQLVPALVQQMGAAYPELGQAQSMIIQTLLQEETRFKQTLERGLKLLDDELSALGEGDDLPGAAAFKLYDTYGFPLDLTQDALREKGRGVDTDGFDAAMAAQKAKARAAWSGTGEAADAAIWFDVADAQGTTDFLGYDTEVAEGQIVALVQGGTATQTAKAGETVQIALNQTPFYAESGGQVGDTGVIKTANGVANVTDCRKTADVFVHMAKVTEGALSVGEAAQLTVDPARRTAIRANHSATHLLHEALREALGDHVAQRGSLNAEDRLRFDFSHTQALSHEELARVEADVNAYIRQNAPVETRIMTPDAARELGAQALFGEKYGDEVRVVSMGRKEGSGKGLSGDTYSLELCGGTHVRQTGDIGAFVTLGDSASSAGVRRIEALTGAAALQHLRAQDQRLAAVAADLKAQLADVPERVRSLLEERRALSNEVAQLRRELAMSGGGGSAAPDVKDVAGTPFMAQVLSGVTGKDLPGLVDQMKEQMGSGVVLLIADADGKAAVAAGVTKDLTAKLSAVDIVKASVAALGGKGGGGRPDMAQGGAKDAANADAAIAAAETVLKGMGFKMGIVGLPNVGKSTLFNALTRTAAAQAANFPFCTIEPNVGEVAVPDARLDKLADIAKSKQIIPTRMTFVDIAGLVKGASKGEGLGNQFLANIREVDSIAHVLRCFEDGDVTHVEGRVDPVADAETIETELMLADLESIEKRLQNIVRKVRGGDKEAVQQQRLMEAAKAALEDGRPARVVEVDAEDRKAWNMLQLLTSKPVLFVCNVGEAEAATGNAHSAKVAEMAAAQGNSHVVISAQIEEEISQLEPEEAEMFLDEMGLSEAGLDRLIQAGYALLHLETYFTVGPKEARAWTIPQGTTAPKAAGVIHGDFEKGFIRAETIAYDDFVSLGGESAAKEAGKMRAEGKAYVVKDGDVLHFLFNTMSWKTLDDMDVAGKRVLLRVDINVPVEEGQVTDRTRIDRIIPTLQDILAKGGTPIMLAHFGRPKGKVVPEMSLRVTLPALEAALGRKVTFIEEPNAER--LSDLPEGIVVLLENTRFAVGEEANDPEMARFLATLGDVFCNDAFSAAHRAHASTTGVAHLLPSCAGRLMQAELSALEAALSTPQRPVGAVVGGAKVSTKIALLENLVARLDVLVIGGGMANTFLAAQGAKLGASLMEPDYMETARTIMQSAERAGCKLLLPVDGVVAREFKAGADFEVVALGKDTVLAEDQMVLDAGPQSVAAVMAAFRTLKTLIWNGPLGAFEITPFNQATNATALEAARLTKSGALISVAGGGDTVAALNQAGAAADFTYISTAGGAFLEWMEGKTLPGVAALQG-------MRQLLEAGVHFGHQTQRWNPRMGPFIYGARNGIHIMDLTQTVPMLDQALQAIRDTVAKGGSILFVGTKRQAQQPIADAAEKCAQYYMNHRWLGGTLTNWQTVSQSINRLKAIDEQSERGFEGLTKKERLGMERDQGKLQASLGGIREMGGRPDLIFVIDVKKEALAVAEANKLGIPVVAVVDTNCAPDGIDYIIPGNDDASRAISLYCDLAARAALDGMSAQLGAAGVDLGAMEEAPVEEAV------AAEAPAEEAQA-----MTKRTSAKHKIDRRMGENIWGRPKSPVNRREYGPGQHGQRRKGKISDFGIQLRAKQKLKGYYGDLTEKQFRRIYAEAERVKGDTGENLIGLLERRLDAVVYRAKFVATVFAARQFVNHKHVRVNGKIVNIPSYRVKEGDVIEVRDRSKQMVALIEATQLAERDVPDYIEADHSKMQATFVRTPALGDVPYPVMMEPNLVVEFYAKNMGNKVNPIGMRLQVNRTWDSRWYADTKDYGDLLLEDLAIRDFIKKECHQAGVARVIIERPHKKCRVTIHTARPGVIIGKKGADIEGLRQKIAKMTASELHLNIVEVRKPELDAALVGESIAQQLERRVSFRRAMKRAVQNAMRMGALGIRVNVAGRLGGAEIARTEWYREGRVPLHTLRADIDYAHVEAATAYGIIGIKTWIFKGEIMEHDPAARDRKAQELQDGPAPRGAGGRRMGRRRKGRDISGWLIVDKPAGLTSTAVVNKVRWALDAKKAGHAGTLDPDATGVLAVALGEATKTVPYVTGALKAYEFEIRLGQATNTDDAEGEVIAQSDLRPTEDEIKEALSAFIGDIEQVPPQFSAVKVDGERAYKRARDGEEMTLAARPLYVDSLLLIDRPDADHVLLEMVCGKGGYVRSIARDLGAALGCLAHVRSLRRIWSGPFDAKNAADLETIEALARTPELDAHVQPLETALDDMPEVKATAEGAVRLRNGNPGMVLASGIEYGETCWASFESRAIAIGRYKSGELHPVRVINAPDTFKLGLTGSIGMGKSTTAQLFVELGCALWDADAAVHRLYVEGGAAVAPIGAIYPTVIHKNSVSRDALRDLIQNDPSVLRRIEKIVHPLVGADRTEFLDQTKSDITVLDIPLLFETGADSDMDAVACVRTTAEEQERRVLARGKMTAKQFQQIRHKQMPNAEKCARADFVIETDTLEHARAQVQTITEQIRRAIADAM-DIDILIEDDRWAAMDLDVLVRDVLRAISDTLTLDLAAAEVSYLACDDARIALLNADFREKATATNVLSWPAADLAPVVAGAVPTTPTVDVTGALTLGDVAIAYETCEREAADLGKPLAEHVTHLIVHGTLHLLGYDHIRDSDAALMQGVEAKILGKMGFDDPYMV---MARYIFITGGVVSSLGKGLASAALGALLQARGFSVRLRKLDPYLNVDPGTMSPFEHGEVFVTDDGAETDLDLGHYERFTGVAARSTDSVSSGRIYSTVLEKERRGDYLGKTIQVIPHVTNEIKDFIAIGDDEVDFMLCEIGGTVGDIEGLPFFEAIRQFTHDKPRGQCIFMHLTLLPYLAASGELKTKPTQHSVKELQSIGIAPDILVCRSEHPIPEKEREKIALFCNVRKEAVVAAYDLKTIYDAPLAYHEQGLDQAVLDAFDISPAPKPDLTVWRDVSDRVHNPEGEVKVAIVGKYTQLEDAYKSIAEALTHGGMANRVKVIVEWVDAEVFDTQDVAPHLEGFHAILVPGGFGERGTEGKIKAAQYAREHNVPYLGICLGMQMAVIEAARNVANIKAAGSEEFDHEAGKKRFEPVVYHLKEWVQGNAKVSRRVGDDKGGTMRLGAYDAALTPGTRVADIYHTSAIDERHRHRYEVDIKYRAQLEDAGLVFSGMSPDGKLPEIIEWPNHPWFIGVQFHPELKSKPFDPHPLFADFIRAAKENSRLVMGRTQTVLALESSCDDTAAAVLRKGATGETQVLSSIVRGQDSLHAAYGGVVPEIAARAHAEILDICVEDALQAAQTSLHDVDAIAVTAGPGLIGGVVSGVMCAKGLALATGKPLYGINHLAGHALTPRLTDNVPFPYLMLLVSGGHCQFLLIKGPDDFKRIGGTIDDAPGEAFDKIARLLALPQPGGPSVERSARQGDAKRFSLPRPLLDRPGCDMSFSGLKTAVLRVRDGLVQDQGGLYPQDQADLSAGFQAAVVDVLAEKTSRAMQQYQQLSPTTATLCVAGGVAANMAIRTRLETVARDNGAVFVAPPLALCTDNAAMIGFAALERMADHPPDDLTLAARPRWPLDSKSPAMLGSGKKGAKA------------------------------------------------------------------------------------------------------------------------------------

>'Su-maritimusS0837'

MKASELHDKTPDQLRDELVNLKKESFNLRFQQATGQLENPARLKTVKRDVARVHTVLNQKAAAAAAE-MATADLLTMD-KKTAEKQKALDSALAQIERQFGKGSIMKLGAEGAIQDIKASSTGSLGLDIALGIGGLPMGRIIEIYGPESSGKTTLTLHCVAEQQKAGGVCAFVDAEHALDPQYAKKLGVDIDELLISQPDTGEQALEITDTLVRSGAVNMVIVDSVAALTPKSELEGEMGDSSVGVQARLMSQAMRKLTGSISRSNCMVIFINQIRMKIGVMFGSPETTTGGNALKFYSSVRLDIRRIGALKDRDEVVGNATKVKIVKNKVAPPFKQVEFDIMYGEGISKMGELLDLGVKAGVVDKSGSWFSYGDERIGQGRENAKNFLKENTAMASEIEDKIRAAHGLDFEGSGGDDADILEAMPRYTPAEIETRWQQAWEKDGVFQATRKADKPKYYVLEMFPYPSGRIHMGHVRNYTMGDMIARYKIATGHNVLHPMGWDAFGMPAENAAMAIGGHPADWTYDNIATMKEQMKPLGLSIDWSREIATCHPGYYGQQQALFLDFLKEGLVYRKNAVVNWDPVDMTVLANEQVENGCGWRSGAPVERRELTQWFFKISDHSEELLSALDSLDNWPAKVKLMQANWIGKSRGLQFAFSTIEAPEGFDRIEVYTTRPDTLLGASFIGISPDHPLAKTLERDDEAVAAFCAECRKGGTTEEAIETAEKLGYDTGIRVRHPFDTAHELPVYIANFILMDYGTGAIFGCPGHDQRDFDFATKYDLPIISTFLPSEDASPELAEAYVPPKTEKVFYNRGFAGDQWQTGEEAVDAAIAFCEENGIGHGVTKYRLRDWGLSRQRYWGCPIPVVHCDDCGVVPEKKENLPIELPYDVSFDTPGNPLDRHPTWRNCACPACGKDALRETDTMDTFVDSSWYYARFTAPRADTPTVMEDAEYWMNVDQYIGGIEHAILHLLYARFFARAMQITGHLPEGAAEPFDALFTQGMVTHEIYQTRDANGRPVYHLPEDVTDGKL-ADGTEVEIIPSAKMSKSKKNVVDPLHIISNYGADTARWFVLSDSPPERDVEWTASGAEASYKHLSRVWNICDRVSEMDREAAGTGDEDLLRAMHKTIHDVTMGVESFGFNAAIAKLYAFTATLQKSKAGYAAQREAIMTLAQLMSPMTPHLSEDIWAKQGGEGLIVNAPWPKADEAMLVDDTVTLPIQINGKRRAEIQVPADMAKEEVEKVALAHEAVIRTLDGAAPKKVIVVPGRIVNVVAMAITSANQLELLQTAEAVAREKMIDPGLVVEAMEESLARAAKSRYGAEMDIRVDIDRKTGKATFTRVRTVVEDDELENYQAEFTVEQAKQYMADPKVGDTYVEEVPPVEMGRIAAQSAKQVILQKVREAERDRQYEEFKDRAGTIINGLVKREEYGNVIVDVGAGEAILRRNEKIGRESYRPNDRIRVYIKDVRREQRGPQIFLSRTAPEFMAELFKMEVPEIYDGIIDIKAVARDPGSRAKIAVISHDGSIDPVGACVGMRGSRVQAVVNELQGEKIDIIPWNEDQPTFLVNALQPAEVSKVVLDEEAGKIEVVVPEEQLSLAIGRRGQNVRLASQLTGLDIDIMTEEQESQRRQAEFELRTKLFMDNLDLDEFFAQLLVSEGFTNLEEVAYVEVDELLVIDGVDEDTAGELQARARDVLEAQNKAALDNARALGVEDSLVEFEGLTPQMIEALAKDDVKTLEDFATCADWELAGGWTTVNGERVKDDGALEPFDISLEDAQEMIMTARVMLGWVDPTELEADADEDDAETDGETAEEAEAMAHKKAGGSSRNGRDSAGRRLGVKKYGGEAVISGNIIVRQRGTKFWPAEGVGMGKDHTIFATVDGAVTFHKGLKNRTFISVLPRAEAAEMAKRWYSVSVLSNFEKKIAEQIRASVAEQELEDQIDEVLVPTEEVIEVRRGKKVTTERRFMPGYVLVHMEMSDAGYHLINSINRVTGFLGPQGRPMPMRDAEVQAILGRVQEGEEAPRTLIHFEVGEKVKVADGPFEDFDGMIEEVDDENQRLKVSVSIFGRETPVELEFTQVNKQVMSFTLAIVGRPNVGKSTLFNRLVGKRLALVDDQPGVTRDLREGAAKLADLRFTVIDTAGLEEVTDDSLQGRMRRLTERAVDMADICLFMIDARVGVTPSDMVFAEILRKKSAHVILAGNKAEGKAADAGMIEAYSLGLGEPIRLSAEHGEGLNDLYTMLMPLADEFEDRAVQDAPETDVDLPEDDADLESIPMPTRAKPLQVAVVGRPNAGKSTLINQILGEDRLLTGPEAGITRDAISLMTEWAGPVPMRIFDTAGMRKKAKVQEKLEKLSVSDGLRAVKFAEVVVVLLDAEIPFEQQDLRIADLAEREGRAVVVAVNKWDIEENRQEKLRDLKESFERLLPQLRGAPLITVSAKTGRGLDRLQAAIMRAYETWNRRVTTAQLNRWLSGMMEAHPPPAPQGKRIKLRYMTQAKTRPPGFVVMCSHPDKVPDSYSRYLVNGLRVDFDMPGTPIRLWMRGQSDANPYKNRKKAAPSKLRKHTAGRRKDMRLIFMGTPEFSVPVLDALVQAGHDISAVYCQPPRPAGRGKKPRPSPVQQRAEALGLLVRHPISLKHPDTQEEFAELEAEAAVVVAYGLILPQAILDAPKHGCLNIHASLLPRWRGAAPIHRSIMAGDEKTGVCIMQMEAGLDTGPVLLCEETEIGAAETTAELHDRLSGMGALAINKALAQLADLTPKPQPEEGVTYAAKIDKAEARIDWTRPAVEVDRLIRGLSPFPGAWFEIDGQRVKVLGSVLDEGEGAPGEVLSEDLRVACGAGAVRLTHLQKAGKGAQKADVFQRGAQIPVGARLGEAMTDTSAYRVLARKYRPETFADLVGQDAMVRTLKNAFAADRIAQAFVMTGIRGTGKTTTARIIAKGMNCIGPDGNGGPTTDPCGVCEHCTAIMEGRHVDVMEMDAASNTGVANIREIIDSVHYRAASARYKVYIIDEVHMLSTGAFNALLKTLEEPPEHVKFIFATTEIRKVPVTVLSRCQRFDLRRIEPEVMIALLRKIATAEGAEITDDALALITRAAEGSARDATSLLDQAISHGAGETGAEQVRAMLGLADRGRVLDLFDMVLRGDAGAALTELSAQYADGADPMAVLRDLAEITHWVSVVKITPDAAEDPTIAPEERARGQQMAETIPMRVLTRLWQMLLKALDEVAAAPNAMMAAEMAVIRLTHVADLPSPEELVRKLQNSSPPST-PIGGGGGN-GAAQGGAQAVHQAQQRMASNPGPQGQTTALAQDLDAALARFPTFEHVVELIRVNRDVKLLVEVETCVQLAAYQPGRIEFVPTDDAPRDLAQRLGQKLQLWTGNRWAVSLVNEGGAQTIAQIRDARELALKAKAEDHPMMQAVLAQFPKARITAIRTPEDLAAAATAEALPEVEDEWDPFEDGMPKMKTKSSAKKRFKVSATGKVIGSQAGKQHGMIKRTNKFIRNARGTTALSEPDAKIIKGFMPYARMNLFAEIRHLIITTLENMVAQDALPATLNFDPITAEPPRDPAHGDMATNAAMVLAKPASMKPRDIAEALAAQLLQDKRITSAEVAGPGFLNLRLAPSVWQNVARQVLSQGTDFGRAILGAGQRVNVEYVSANPTGPLHVGHTRGAVFGDALASLLDFAGYDVTREYYINDGGAQVDVLARSVYLRYLEANGKEVAFPDGTYPGDYLIPLGEALAKMYGDKLVDQPESEWLDHIREFATDAMMNLIREDLAALGVEMDVFYSEKSLYGTGQIEAAIASLEAKGLIYEGVLEPPKGKKPEDWEPREQTLFKSTAHGDDVDRPVKKSDGSWTYFAPDIAYHYDKVSRDFDMLIDVFGADHGGYVKRMKAAVSALSDGHVPLDIKLTQLVKLYKNGEPFKMSKRAGTFVTLRDVVDQVGPDVTRFVMLTRKNDAMLDFDFDKVLEQSRENPVFYVQYAHARIASILRKAEAAEIDVADAALMEADLSKLDHEAEIGLLRKIAEWPRLVETAARSNEPHRVAFYLYELASDFHSLYHLGRSEDGLRALQEGDAETSQAKIALSRAVAIVIAAGLGILGVTPAQEMRMSTIDNLPPLREVIATHDLAARKSLGQNFLLDLNLTAKIARQAGDMADCDVLEIGPGPGGLTRGLLSEGARHVLAIEKDRRCLPALAEIADHYPGRLTVIEGDALEVDPLTHLTPPIRVAANLPYNVGTELLVRWLTPPEWPPFWQSLTLMFQREVAERIVATPGSKAYGRLAVLAQWRSDARIVLQLPPGAFTPPPKVSSSVVHLTALPEPRFPADPDVLSRVVAMAFNQRRKMLRSALKGAAPDIEDRLRAAGLSPTERAEQISLEGFCALAREIAKGMNLIAEIEAEQVAELGKEIPDFRAGDTIRVGFKVTEGTRTRVQNYEGVCISRKNGHGIAGSFTVRKISFGEGVERVFPLHSTNIDSITVVRRGRVRRAKLYYLRSRRGKSARIVENAHYKPRANA--MANTKRQLFLKRRMRVRNKLRKVNAGRMRLSVHRSSKNISAQLIDDVNGVTLASASTMEKDLGVVGKNNIEAATKVGAAIAERAKKAGVEEAYFDRGGFLFHGKVKALAEAAREGGLKIMFAVIKTGGKQYKVQSGDMLRVERIAANAGETVQFNEVLMLGGDSPVLGAPMVKDAGVQAEVVDQIKGEKVINFVKRRRKHSSKRTKGHRQKLTLIKITDILASGAEKSGVAAAVGTGSVSA----AAVAA--TQIKKAK-----KA---KATKAAPKAEKADAGADDLKALSGVGPALEKKLHEAGITSFAQIAAWTEADIAEVDEKLSFKGRIQREGWVDQAKEKTKGMSRVKGGTVTHARHKKIIKAAKGYYGRRKNVFKVATQAVDKANQYATRDRKNRKRNFRALWIQRINAAVRSHDEALTYSRFINGLTLAGVEVDRKVLADLAVHEPEAFGAIVKQAQDALAAMQVNETLNEGLKRGYTITVTADELDAKVNEKLAEAQPEVEMKGFRKGKVPMALLKKQFGQKVMGEAMQESIDGAMNEHFEKSGDRPAMQPEVKMTNEDWKEGDDVEVSMSYEKLPEIPEVDLSQIELEKLVVKADDAAVEEALANLAETAQDFKARDADAEAQDGDQVVIDFKGSVDGEEFEGGAAEDYPLVLGSNSFIPGFEEQLVGAKAGDEKSVVVNFPEEYQAEHLAGKEATFACTVKEVKEPVAAEVNDEMAKKFGAEDLDALKGQIAERLEAEYAGASRAVMKRNLLDALDKQVSFDLPPSLVDAEAKQIAHQLWHEENPDVQGHDHPEIEATDEHKTLAERRVRLGLLLAELGQKADVQVTDAEMTQAIMNQARQYPGQERQFFEFVQQNQQMQQQMRAPIFEDKVVDHVVEQAKVTEKEISKDELQKAVEELDDEMKLHELSDNEGAAKKRMRVGRGPGSGKGKMGGRGIKGQKSRSGVAIKGFEGGQMPIYQRLPKRGFNKPNRKSFAVVNLGLIQKFIDAKKLDAGNTIDGAALVASGLVRRELDGIRVLAKGDITSKIDLNVAGASKSAIEAVEKAGGKVTVAAPKAAEAAEMIQMQTNLDVADNSGARRVQCIKVLGGSKRKYASVGDIIVVSVKEAIPRGRVKKGDVRKAVVVRTAKEVRREDGTAIRFDRNAAVILNNNNEPVGTRIFGPVVRELRAKNFMKIISLAPEVLMRHARGYRRLNRTHEHRKALFSNMAGSLIEHEQIKTTLPKAKELKPIIEKMITLAKRGDLHARRQAASKLKEDQYVAKLFDILGPRYKDRQGGYVRVLKAGFRYGDMAPMAIIEFVDRDRDAKGAADKARVAEEDAAE-MLQPKRTKFRKQFKGSIKGLAKGGSDLNFGTYGLKALQPERVTARQIEAARRAMTRHMKRQGRVWIRIFPDVPVTSKPVEVRMGKGKGSVDFWAAKVKPGRIMFEIDGVGEDVAREALRLAAMKLPIKTRVVVREDWMTTIKLHNTATRKKEDFTPINAENVRMYVCGPTVYDRAHLGNARPVIVFDVLYRLLRHVYGPDHVTYVRNFTDVDDKINARAAESGRSIGEITAETTQWYLDDMAAVGALEPDHMPRATQYVPQMVAMIRGLIDDGYAYEAEGHVLFRVRKYAAYGALSGRSVDDMIAGARVEVAPYKEDPMDFVLWKPSEAGAPGWDSPWGQGRPGWHIECSAMAHDLLGERFDIHGGGIDLQFPHHENEIAQSKCAG--HGFANYWLHNEMLQVEGKKMSKSLGNFFTVRDLLDQGVPGEVIRFVMLSTHYRKPMDWTEKKREEAEKTLRKWYLQAAEAEAAVP-GPELVALLADDLNTHGALTECHRLSNAGDTVRLRGALTLLGLMDELIPEWAAVQALDLSDIEAFLSDARATAMETKDFAEVDRIKTALTSVGIEVQMSKDGVKLTPPPGFDRSQLEGVLMSQVKSSSKSDPNYKVIAENRRARFDYAIEDDIECGIILEGSEVKSLREGGANIAESYAAVEDGELWLVNSYVAPYKQATTFQHEERRRRKLLVSRKELGDLWNATQRKGMTLVPLVMYFNHRGMAKIKIGVAKGKKLHDKRETAAKRDWSRQKQRLLKDHSMSAKHEHYDVIRKPIITEKATMASEQNAVVFEVAIESNKPMIKEAVEALFNVKVKAVNTSITKGKVKRFRGQMGRRKDVKKAYVTLEEGNTIDVSTGLMSKDKNPRRVADNEAMAKLRMLRTSPQKLNLVAALIRGKSVDKALTDLTFSKKRVAQDVKKCLQSAIANAENNHNLDVDELIVAEAYVGKNLTMKRGRPRARGRFGKIIKPFAEITIKVRQVEEQAMVSAVENMAANTSWAALGKATDLRNRILFTLGLLIVYRLGTFIPVPGIDGGALRDFMESAGQGIGGMVSMFTGGALGRMGIFALGIMPYISASIIVQLLTSMVPALEQLKKEGEQGRKKINQYTRYGTVALATLQSYGLAVSLQAGDIVADGQMGFGFACMITLVGGTMFLMWLGEQITARGIGNGISLIIFVGIIAEVPAAIAQFFASGRSGAISPAVIVGVLVMVIATIMFVVFMERALRKIHIQYPRRQVGMKMYDGGTSHLPVKVNPAGVIPAIFASSLLLLPVTVSTFSGNSTSPVMSWLLANFGPGQPLYLLFFVAMIVFFAYFYTFNVSFKPDDVADNLKNQNGFVPGIRPGKRTAEYLEYVVNRVLVLGSAYLAAVCVLPEILRGQFAIPFYFGGTSVLIVVSVTMDTIQQVQSHLLAHQYEGLLEKSQLRGKGAGTRKKRSPVRRMKFLDLCKVYIRSGAGGGGCVSFRREKYIEYGGPDGGDGGTGGSVWAEAVDGLNTLIDFRYQQHFFAKNGQPGMGKQRTGKDGDDIILRVPVGTEILDEDQETVIADLTELGQRVQLARGGNGGWGNLHFKSATNQAPRRANPGQEGVERTLWLRLKLIADVGLLGLPNAGKSTFLAATSNARPKIADYPFTTLHPNLGVVGVDNTEFVVADIPGLIEGASEGRGLGDLFLGHVERCAVLLHLIDGTSETLAEDYHTIIGELEAYGGDLAEKPRVTVLNKVDALDEEDRATRLKELENACGGPVMMMSGVAGEGVTDVLRTLRQNIDDDRLRFRSTEEEEPWQPMAAKLRKGDKVIVLSGKDKGKTGTISSVDPKSNKAIVDGVKIAIRATRQTQTSQGGRIPKAMPIDLSNLALVDANGKATRVGFKIEGDKKVRFAKTTGDVIDAMLRSGVIAKKVGMTRLFMEDGKQIPVTVLQLDKLQVVAQRTADRDGYTAVQLGAGTAKAKRTSQAMRGHFAAAKVEPKRKVAEFRVDADAMINVGEEITADHYFAGQYVDVAGTSIGKGFAGAMKRHNFGGLRATHGVSISHRSHGSTGQCQDPGKVFKGKKMAGHMGAARVTTQNLEVVKTDSARGLIMVKGAVPGSKGGWVTVKDAVKKPFPEDAILPAALKSAAEEAAKAAEEAAAAAAAEAEAEAKRLAEEQAAQEAEALKAAEAEIAAEGSDADNSDADDKKEGDAMALKSYKPTTPGQRGLVLIDRSELWKGRPVKALTQGLTKSGGRNNTGRITMRRTGGGAKRLYRIVDFKRNKLDMSAVVARIEYDPNRTAFIALIQYEDGEQAYILAPQRLAIGDKIIAGAKVDIKPGNAMPFSGMPIGTIVHNIEMKPGKGGQIARAAGTYAQFVGRDGGYAQIRLSSGELRLVRQECMATVGAVSNPDNSNQNYGKAGRMRHKGIRPSVRGVVMNPIDHPHGGGEGRTSGGRHPVTPWGKPTKGAKTRNKNKASSKLIIRSRHAKKKGRMLDTATYTPRLQVEYREKIRAALKEEFGYKNDMMIPKLDKIVLNIGCGAEAVRDSKKAKSAQEDLTTIAGQKALTTVAKKSIAGFRVREEMPLGAKVTLRGDRMYEFLDRLITIAMPRIRDFRGIPGKSFDGRGNYAMGLKEHIVFPEIDFDKVDETWGMDIVIATTAKTDAEAKALLKAFNMPFNSMKLDVIKLDGGNAGSVDLDEALFGLEPRADILHRVVRWQRNNAQQGTHKVKTRSETSYSTKKIYRQKGTGGARHGDRNAPIFRKGGIYKGPTPRSHGHELTKKFRKLGLRHALSAKAKAGSLVIIDEATSEGKTAALAKQVSNLGWKRALVIDGASVNENFAQAARNIEGLDILPSMGANVYDILKRDTLVITKAGIEALEARLKMENVVLIIHLILALGLIAVVLLQRSEGGGLGI-GGGGGAVSGRSAATALGKMTWILAIGFIATSITLTIIAAEKSAGSSVIDRLSATPPAQNQEGSPALPSGNDLLPPSADSDTPLVPAADMAFFTKLKDRLFKSSSKIDEGLEAIVSDGGAAEAEA-EAAPVDADRPGLASDVMPDRQQPDPQPTPE--PA-QPEPQPDPSQEPTPAPDPNPEPQDPEPTPEPEPIPPEPTPDPVPPLRQAMTPVAPDLTGEAT-ETTTAKPGLLGRLMGR-TAKTTVRRTLDDDMLEQLEELLISADMGVDTALRVTANMAEGRFGKKLSVEEIKQLLATEVGRIMEPVARPLPIYSKTPQVVLVVGVNGSGKTTTIGKLASQFRAAGKKVVIAAGDTFRAAAVEQLQVWGERAGVPVLTAAQGSDPASLAFDAMGRAQEEGADLLLIDTAGRLQNRGDLMEELAKIVRVIRKKDETAPHNTLLVLDATTGQNAVNQVKVFQEISDVSGLVMTKLDGTAKGGVLVALADQFGLPIHAVGVGEQIDDLSPFDPQEFAAALVGVDAMTQDQWGEIKQRLLKTVGQNNYTTWIEPLVPGDVEDGIVTLRVPTNFFGNYVSQNFSDLILHEINAAGTDATRLNFALNQQPANAADKPAPAARQTTAAIKP--TANNQLSTAPLDPRFSFDNFVVGKPNELAHAAARRVAEGGPVTFNPLFLYGGVGLGKTHLMHAIARELHERRPDMNVLYLSAEQFMYRFVQALRDRKMMDFKEIFRSVDVLMVDDVQFIAGKDSTQEEFFHTFNALVDQHKQIIISADRAPGEIKDLEDRVKSRLQCGLIVDLHPTDYELRLGILQSKVEVQRKTYPDLEVADGVLEFLAHRITSNVRVLEGALTRLFAFASLVGREIDMGLTQDCLADVLRASERKISVEEIQRKVSDHYNIRLSDMIGPKRLRSYARPRQVAMYLCKQMTSRSLPEIGRRFGGRDHTTVMHGVRRIEELKVSDGQIAEDLELLRRALESMLGIGTIAKKVFGTPNDRKIKATRPLVAQINALEPEFEKLSDDEIKVRTAELAKRAEAGESLDDLLPEAFANCREAARRTLGLRAFDTQLLGGIFLHQGNIAEQKTGEGKTLTATFAAYLNGLTHKGVHIVTVNEYLAKRDAEWMGKVFGALGLTTGVAYSGMPEDQKRAAYACDITYATNNELGFDYLRDNMKSNLSDMLQRGHNFAVVDEVDSILIDEARTPLIISGPSQDRSEMYQIIDALIPSLTEEHYELDEKTRNVTFTDEGNEFLEQQLRARDLIEEGMTLYDPESTTIVHHVNQGLRAHKLFQRDKDYIVRDGAVTLIDEFTGRMMPGRRLSDGLHQAIEAKEGVEIQPENVTLASVTFQNYFRLYDKLAGMTGTALTEAEEFATIYGLGVVEVPTNVPIARVDEDDAVYRTAREKYDAMIEKVKEAHAKGQPCLVGTTSIEKSEQLSAMLTAAGIEHNVLNARQHEQEAQIIAEAGKLGAVTIATNMAGRGTDIQLGGNVEMKVLDALDADPDADPANIRAQIEAQHAEEKKKVLEAGGLYVLASERHESRRIDNQLRGRSGRQGDPGRTSFFLSLEDDLMRIFGSERLEKVLTTLGLKEGEAIVHPWVNKSLERAQAKVEGRNFDIRKQLLKFDDVMNEQRKVIFGQRREIMEADNLNEIVTDMREQVIDDLIDTYMPPKTYADQWDTQGFYAAVIEQLNVDVPIIAWCEEDGVDDEVIRERLMEATDKMMAEKAEKFGEENMRNIEKQLLLQAIDTKWRDHLLTLEHLRSVVGFRSYAQRDPLNEYKNEAFQLFESMLDSLRQDVTQKLGQIQPMSEEQRQEMMREMAARQAEMQAAATQAADSS--AEKAEA-AVAGFDENDPATWGNPGRNDPCPCGSGKKFKHCHGQITMKLSGDLKAFEARIGHSFGKPELLGRAVTHASMSSANRDDNQRLEFLGDRVLGLVMAEALLALDPGATEGQLAPRFNALVRKETCADVAREIDLGKVLKLGRSEMISGGRRKQALLGDAIEAVIAAVYLDAGFDAAKDLVLRLWGDRLKTVKEDARDAKTALQEWAQARGFNPPRYVQTGRSGPDHAPVFTITARLDNGAEAAATAPSKRAAEQAAATTLLRQLESNSMAKIGKRTRAAREAFAGKEDLTVEEAVSLIKANATAKFDETIEIAMNLGVDPRHADQMVRGVVGLPNGTGKTMRVAVFARGAKAEEAEKAGADIVGAEDLMETVQSGKIDFDRCIATPDMMPIVGRLGKVLGPRNLMPNPKVGTVTMDVADAVKAAKGGEVQFKAEKGGVVHAGVGKLSFDEAKLVENIRAFVGAVSKAKPTGSKGTYMKKINLSSTMGPGVSVAVENATAEMSDEFMLDTDDLERRMNGAIASLRTEFASLRTGRGSASMLEPVMVEAYGQMTPVNQVGTVNVPEPRMVTINVWDKGLVGKVEKAIRESGLGINPQLNGTIIMLPIPELNEERRTQLTKVAGQYAESARVSIRNIRRDGMDQIKKAKNDGMSEDDQKIWEDEVQALTNRFITTIDEQLETKQAEIMQVMAKKLVGTMKLQVKAGQANPSPPVGPALGQRGINIMEFCKAFNAKTADMEPGAPCPTVISYYQDKSFTMDIKTPPASYYLKKAAKVNSGAKTPSRETVGTVTTKQLREIAEAKAADLSANDVEAAMKIILGSAKSMGIEVKMFENLSERLSGVFDRLTKQGALSDEDVKTALREVRVALLEADVSLPVAREFVKTVQEKATGQAVTKSVTPGQQVVKIVHDALIDVLKGEGEPGSLKIDSPPAPILMVGLQGGGKTTTTAKLAKRMKERDGKRVLMASLDVNRPAAMEQLAILGTQIGVDTLPIVKGESPVQIAKRAKTQAGLGGYDVYMLDTAGRLSIDEELMQQVKAVRDVANPRETLLVVDGLTGQDAVHTAENFDERIGITGVVLTRMDGDGRGGAALSMRAVTGKPIKFVGLGEKMDALETFEPERIAGRILGMGDIVALVEKAQETIEAEQAEKMMKRMAKGQFNMNDLKMQLEQMIKMGGMQGMMGMMPGMGKMAKQVEDAGFDDKILKQQIALIQSMTKKERANPALLQASRKKRIAKGAGMEVSDLNKLMKMHRQMSDMMKKMGKMGKGGMLKQAMKGMMGKGGMDPS----QMDPKALEAAAKQMGGKLPGGLGGMGGGMGLPSGLSGFGKKKMIPMPRLAQITQRFQFLEASMSAGSDGADFAKLAKEYSDLKPVVDQIELYQQLQRDLEDAEEMLNDPEMAELAREELPRLRTRLPEVEAALQLALLPRDAADAKPAMLEIRPGTGGDEAALFAADLLRMYQRYAEARGWGFDLIEEQMTELGGVKEVVAHITGQNVFARLKFESGVHRVQRVPSTESGGRIHTSAATVAVLPEAEDVDIDINPNDLRIDTMRSSGAGGQHVNTTDSAVRITHIPTGIVVTSSEKSQHRNRDKAMQVLKARLYDMERSRMDSERSADRAAQVGSGDRSERIRTYNFPQGRMTDHRINLTLYQLDAVMQGDLDEIIDALTADAQARQLAEMEGMDRAQKEQLVDELGQIFESSGVVVVSHYVGLTVAEMQDLRARARAAGGAVRVAKNRLAKIALEGKPCESIADLLTGMTVLTYSEDPVAAAKVAQEFSKENPKLVILGGSMGENALDAAGVEAVSKMPSREELISTIAGMLGAPASNIAGAIGAPASNIASILSTIEDKAAAMKTFSATPADIEKKWIIIDAEGVVLGRLASIIATRLRGKHKPSFTPHMDCGDNVIVINAEKVQMTGKKREEHFYWHTGHPGGIKSRTKEQILEGAHPKRVVTQAVKRMLPGNRLSRQIMTNLRVYAGSEHPHEAQSPEVLDVKSMNKKNTRSAMTTDPAGLAEKLIAGLGGGLPPRLGVAVSGGGDSMALLSLLSGLFRAAGSHLEVVTVDHGLRAEAAVEADFVARHAAALGLAHETLRWRDWDGQGNLQDAARQARYRLMADWAKRRQLPCVALGHTADDQAETVLMRLARRAGVDGLSAMAPQSTRHDIQWLRPLLAVRRDTLRDYLRESRVEWIDDPSNDDLHYSRIRARQCLAGLAPLGIDAETLAEVAENMASVRAALDHQTDRAASEILRIEAGAVVARAEAFFAEPVEIRRRLILRALGWISGSRYTPRRNPVAALIAGLAQGQGATLDGCQVLLRRGEIWVFREYNAVREHVVPADHIWDTRWRATTKAAA-AGAELRVLGPEGLAQCPAWRETGRPRAVLLSTPSVWQGDRLLAAPLAGLDEKWHVQLERDAGWLKTAPLSHMADLKKLAEDIVGLTLLEAQELKTILKDEYGIEPAAGGAVMMAGPAEGGAAEEEKTEFDVVLKNAGASKINVIKEVRGITGLGLKEAKDLVEAGGKIKEGVDKAEAEDIKGKLEAAGAEVELAMSRIGKKPVAIPSGVSAEVSGQTIEVKGPKGTRSFRATDDVTLTVEDDAITVTPRGKSKRARQQWGMSRTMIENLVTGVSTGFKKELEIQGVGYRAAINGNTLKLNLGLSHDVDYVAPEGVTVTAPKQTEIVVEGIDEQLVGQVAANIRAWRKPEPYKGKGIRYKGEFVFRKEGKKKMQVILLERVAKLGQMGEVVDVKPGYARNYLLPQGKALSASKANVEAFEGQKAQLEAQNLETKKEAEQLAEKLNGQQFVVIRSASDAGALYGSVTTRDAAEAATEEGFSVDRKQVVLGQPIKYLGVHEVQIVLHPEVTVSIELNVARSPEEAELQASGKSIQELAAEEEAAAEFEISELFDDIGSAADEDDDDSNMVRTPEGDAQDDSNS--MSDSDGKKTLGLRGGARPGNVKQSFSHGRTKNVVVETKRKRVVVPKPGGQKPTGPGAGPIGDPKKRPAGITDAEMERRLKAVQAAKAREAEEAAQREAEEKARAEERERRRAEIEAKEREEREREESLKAKAEEDARRKREAEE---AAAAPVVEPTPAREQPSKSAPAATPRKTERDREETKKRSKGGDS-RRSGKLTVNQALAGGEGGRQRSMAQMKRKQERARQKAMGGQVEREKIVREVQLPPAIVVSELAVRMAEKIGAVVKSLMQNGMMVTQNEVIDADTAELIIEEFGHKVVRVSDADVEDVIKEVEDKPEDLKPRPPVITIMGHVDHGKTSLLDAIRNAKVVAGEAGGITQHIGAYQVKTDNGTVLSFLDTPGHAAFTSMRSRGAQVTDIVVLVVAADDAVMPQTIEAIAHAKAAKVPMIVAINKIDKPAANPDKVRTDLLQHEVIVEKMSGDVQDVEVSAATGQGLDDLLEAIALQSEILELKANPDRAAVGAVIEAQLDVGRGPVATVLVQNGTLRQGDIFVVGEQYGKVRALINDQGERVKEAGPSVPVEVLGINGTPEAGDVLNVTDTEAQAREIAEYRANAAKDKRAAAGAATTLEQLMANAKANEDVSELPILVKADVQGSAEAIVQAMEKIGNDEVRVRVLHSGVGAITETDVGLAEASGAPIMGFNVRANASARNTANQKSVEIRYYSVIYDLVDDVKAAASGLLSNEIKENFIGYANIKEVFKVTGVGKVAGCLVTEGVARRSAGVRLLRDNVVIHEGTLKTLKRFKDEVAEVQSGQECGMAFENYDDIRPGDVIEIFEREEVTRTLSMAKPKKNPRPKAQTPKGFRDYFGAEVTHRAEMLSKIAAVYQRYGFDALESSGVETVEALGKFLPDVDRPNEGVFAWQEDAEGDKPGDWLALRYDLTAPLARVYAQHRNDLPLPYRRYAMGPVWRNEKPGPGRFRQFYQCDADTVGAPSVAADAEICAMLADCLEEVGIARGDYVVRVNNRKVLNGVLEVAGLSGDDKETERGIVLRAIDKLDRLGPDGVRALLGEGRKDDSGDFTKGAGLAEAQADVVMGFMQAKRDSGAETVARLRELVTGSDVGVQGVDELKLISDLLAAGGYGPDRVEIDPSVVRGLGYYTGPVFEAELTFEIKDEKGRARNFGSVAGGGRYDDLVKRFTGQEVPATGVSIGVDRLLAALAAKGRLAKEATGPVVVTVMDRDRMADYQAMVAELRQAGIRAEVYLGNPKNFGNQMKYADKRQSPVVVIEGGDEQARGVVQIKDMVLGAQMAQDASHDEWKERKNQYEVPRGELVQAVRDILDRTSMDDLKQKYLSQIADAGDESALEDIRLAAVGKKGEVALKMRELGKMTPEERQVAGPALNALKDEINSALAAKKAALGDAALDERLRSEWLDVTLPTRHQRQGSIHPVSQVTEELTAIFAEMGFSVAEGPRIDTDWYNFDALNIPGHHPARAEMDTFYMHRAEGDERPPHVLRTHTSPVQIRTMEAEGAPLRIICPGGVYRADYDQTHTPMFHQVEGLAIDKDISMANLKWTLEEFFAAFFEIDGIKTRFRASHFPFTEPSAEVDIQCSWVDGQLRIGEGDGWLEVLGSGMVHPKVLAAGGIDPNEWQGFAFGMGIDRIAMLKYGIPDLRAFFDSDLRWLRHYGFASLDQPNLHGGLSRMEMADEAGLDLVEISPNANPPVCKIMDFGKFKYEQQKRESEARKKQKIIEIKEVKFRPNTDQGDYDVKMRNVFKFLDGGDKVKITLRFRGREMAHQNLGRELLERVAEDTKEHGRVENFPKMEGRQMVMVIGPLPNMKFTLSWLKDHLDTTASIDEITYALTDLGLEVEGVENPAAKLADFTLGYVQSAEKHPDADRLNVCQVETDEGVMQIICGAPNARAGITVVVAKPGVYVSGIDTTIGVGKIRGVESFGMMASEREMELSEEHDGIIELPSGKPGDRFIDWLAENDPAKVDPVIEIAITPNRPDALGVRGIARDLAARGLGKLKPRDCDAVEGSFASPINVSIDEDTLDGCPVFYGRVIRGVKNGPSPQWLQDCLRAIGLRPISFLVDVTNFFTFDRNRPLHVFDADKVKGNLRVHRAKGGEEIAALDEKTYTLQAGQMVISDDNGVESIAGIMGGEATGVTEDTVNVFVESAYWDPVQIAYAGRALKINSDARYRFERGVDPAWTPYGIEHATRMILDHAGGEASEVVVAGQVPDTRRAYKLDAAKVQSLVGMTIPESDQRQTLTALGFQLDGDMAQVPSWRPDVQGEADLVEEVARIASLTKLEGKPLPRLTAGVPRPVLSPMQRRVVTARRTAAALGYNECVTYSFIDQASAALFGGGTEETRLENPISSDMSHMRPDLLPGLLQAAARNRARGFADMALFEVGPAFSGGEPGEEQMMVSGLLVGRTGPRDVHGAARAVDVFDAKADAEAVLAAIGAPAKVQILRGAAEWWHPGRHGKICLGPKKVLGVFGEVHPRVLAAMDVKGPAMAFTIYPAEVPLPRKSGATRPALQISDFQAVERDFAFVVDADVEALTLVNAAKGADKNLIEDVRVFDEFIGGSLGEGKKSLAITVRLQPSDKTLKDVDIEAVGAKVVEKVTKATGGVLRGMHAYRSHTCADLSLENKGDNVRLSGWVHRVRDHGGVLFLDLRDHYGITQVICDPDSPAFAEMEKVRAEWCVRIDGTVKARDESLVNPKLPTGEIEVYARDIEVLGAAAELPLQVFGDQEYPEETRLRYRYLDLRREKMQQNMTLRSDVVASIRKRMWDQNFREFQTPIITASSPEGARDFLVPSRLHPGKFYALPQAPQQFKQLLMVSGFDKYFQIAPCFRDEDPRADRSPTDFYQLDMEMSFVTQQDVFDTIQPVIAGIFEEFGGGKKVDETWEQISYKDAALWYGSDKPDLRNPIKMQVVSEHFRDSGFAIFAKLLEQEGTEIRAIPAPTGGSRKFCDRMNAFAQKEGLPGMGYIFWRDQG---NGMEAAGPLAKNIGPERTEAIRQQLGLEVGDAAFFLGGKPKSFESVAGRARNVIGEELGLTDKDRFAFAWIVDFPIYEKDEETGKIDFEHNPFSMPQGGMEALQGDPLEVLGYQYDLACNGYELVSGAIRNHKPEIMFKAFEIAGYGEDEVRKRFGGMVNAFQYGAPPHGGCAAGIDRIVMLLAEEANIREVILFPMNQRAEDLMMDAPSEPTSDQLMELGLRVIPQDMTPLENIRNFSIVAHIDHGKSTLADRLIQSTKTVADRDMKEQMLDNMDIERERGITIKAQTVRINYTADDGQDYVLNLIDTPGHVDFAYEVSRSMRAVEGSLLVVDSTQGVEAQTLANVYHAIDADHEIVPVLNKIDLPAADCDRVAEQIEDVIGIDASDAIQVSAKTGIGIKETLEAIVTRLPAPKGTLDAPLKAMLVDSWYDSYLGVIVLVRIMDGQLRKGDRVRMMQNGSVHHVDRIGVFRPAMTEIDVLGPGELGFLTASIKQVRDTRVGDTITHEKKGTEEALPGFKPSQPVVFCGLFPVDAAEFEDLRDSIEKLALNDASFSYEMETSAALGFGFRCGFLGLLHLEVIRDRIEREYDIDLITTAPSVIYDIHMKDGSVEQLHNPADMPDLTHVDHIEEPRIKATILVPDEYLGDVLKLCQDRRGIQENLTYAGSRAMVVYDLPLNEVVFDFYDRLKSVTKGYASFDYQMIGYRTDALVKMSILVNDEPVDALSTMVHRDRAEMRGRAMVEKLKDLIPRHMFKIPIQAAIGGKVIARETLSAMRKDVTAKCYGGDATRKKKLLEKQKAGKKKMRQFGKVDIPQEAFISALKMDSMSLPPGFLEELRDRASLSQVVGRKVIWDNRKSNQGKGDMWAPCPFHQEKSASFHVDDRKGFYYCFGCHAKGDAISFVRETENVSFMEAVEIIAREVGMPVPKQDPRAQAKADKRTQLAEVMEQAVQWFRLQLRTGAAGAARDYLAKRGLSDQAQAHWEIGFAPDSWQGLWDALKAKGVADELIFGAGLAKPSSKGGRPYDTFRGRIMFPIRDARGRAIAFGGRAMDPEDKAKYLNSPETELFDKGRSLYNVKHARAAAGRGQPLIVAEGYMDVIALHGAGFEGAVAPLGTAITENQLQMLWRIAPEPIITLDGDAAGQRAALRLIDLALPLLEAGQSLRFAVMPEGQDPDDLLRAQGAGALQKLLDGALPMVRLLWQRETEGKVFDSPERKAALDKSLREKIKLIRDPSIRSHYGQEIKDLRWELFRPQKPRARGGGKG-GKGGKPAWG-ASQAPLASTKASALVAMGDDQVGIYLREAVILVALCNCPELVETFETGLEAMPCTDLDHARLRDLLLRYGHAGAEVLNEQISYSLGWEALENMKAQRHVAITPCIRKPGDVDMTRMTVAEELAKLDAARGLNEEIAEAVEDLSGPADEGVTWRLSEAARAADMASRSAKEESGEFDLGDNGMTMSREERSALDALLGTIRYDKSKGRGMCAETPDYKATLNLPKTDFPMRAGLPKREPGWLERWEKIGVYDRLREKEGRTPFTLHDGPPYANGHLHIGHALNKTIKDMIVRSHQMMGYDARYIPGWDCHGLPIEWKIEEQYRKKGRDKDQVPINEFRAECRDFAAKWVDVQREEFKRLGITGNWENPYLTMDFHAERVIAEEFMKFLMNGTLYQGSKPVMWSPVEKTALAEAEVEYHDKESHTVWVKFKVADVSQRAKDLIGAHVVIWTTTPWTMPSNKAVVYGEDISYGLYEVTATPEECWAKVGERFLLADDLAADVFARARLEDGMWQRVRGVENDELAKISLQHPLAGAEGANGEWDDLRDFRAADFVTSDEGTGFVHCAPSHGLEEYELYRDLGMLPQVITYNVMEDGRFRDDLPFFGGKAILKPNGKEGNANSAIIDKLVEVGGLLARGKIKHSYPHSWRSKAPVIYRNTPQWFAAIDKEVGDGLDQNGKTIRERALTCIDKVNWVPKSGRNRLHSMMEARPDWVLSRQRAWGVPLTCFVRKGVAPTDENFLLRNEEVNQRIVEAFEAEGADAWYAEGAKERFLKGIVDPAEFDQVTDILDVWFDSGSTHAFTLRDREDGTEDGIADVYMEGTDQHRGWFHSSLLQSVGTTGRAPYRNVVTHGFTLDAKGMKMSKSIGNTIVPEKIVQQYGADILRLWVAQTDYTADQRIGDEILKGVADSYRRLRNTMRYMLGALSDFTEADRVDPADMPELERWVLHRVAELDKVVRDGFARFDFQGVFQAVFTFATVDLSAFYFDIRKDALYCDGDTLRRRAARTVLDILFHRLTTWLAPVLVFTMEEVWLERFPGDESSVHLVDMPETPEAWLNPELAAKWAKVRAARRVVTAALEVQRTEKVIGASLEAAPVVHVDDAAQRAALESVSFEDVSITSDITVTRDAAPAEAFRMPETQGVAVVFEKAEGAKCERCWKVLPDVGTHEYPGVCGRCDEAVRAAPSQNIRIRLKAFDYRVLDSSTQEIVSTAKRTGASVRGPIPLPNKIEKFTVLRGPHVDKKSRDQFEIRTHKRLLDIIDPTPQTVDALMKLDLAAGVDVEIKLQSMADEIKTLEGLEAAVTGGVQGTE----TEMTPREPVRDELGRAYATGKRKDAVARVWIKPGSGKVVVNGKPQNEYFARPVQQLILAQPFGITNTEGQFDVFATVKGGGLSGQAGAVKHGISKALQLYDPSLRGALKAAGFLTRDSRVVERKKYGKAKARRSFQFSKRMPTIQQLIRKPRQPKVKRSKSMHLQECPQKRGVCTRVYTTTPKKPNSAMRKVAKVRLTNGFEVISYIPGESHNLQEHSVVLIRGGRVKDLPGVRYHILRGVLDTQGVKDRKQRRSKYGAKRPKMARDKTRTKRKVSKNIAAGVAHVNSSFNNTKILISDVQGNAISWSSAGTMGFKGSRKSTPYAAQMAAEDAGRKAQDHGVKTLEVEVQGPGSGRESALRALAAAGFNITSIRDVTPMAHNGCRPPKRRRVSKQSRRGLLIILSSPSGAGKSTMARALREWDPTINFSVSATTRAPRPGEVDGKDYRFVAEDDFRHAVAEGEMLEHAHVFGNFYGSPKAPVQAAIDEGQDILFDIDWQGAQQIRNSDLNTHTLSIFLLPPSITELKRRLESRGQDDAETIAKRMGKSWDEISHWDGYDFVLINDDLDQTEARLKSIITAARLRLSQQPAIKDHVRRLQSEFQELKMAQSFLGQKRLRKYYGKIREVLEMPNLIEVQKSSYDLFLNSGDAETPTDGEGITGVFQSVFPIKDFNETSVLEYVKYELEKPKYDVEECQQRDMTYSAPLKVTLRLIVFDVDEDTGAKSVKDIKEQDVFMGDMPLMTPNGTFVVNGTERVIVSQMHRSPGVFFDHDKGKTHSSGKLLFACRIIPYRGSWLDFEFDAKDIVFARIDRRRKLPVTTLLYALGLDQEAIMNAYYNTVTYTLEKNKGWVAPFFPDRVRGTRPTYDLVDAATGEILFEAGKKVTPRAVKKLLDEGKVKDLLLPFDHIVGKFVARDIINEETGAIYVEAGDELTLEYDKDGTLIGGTAKELIDAGITEIPLLDIDNVNVGPYMRNTMAQDKNMNRDTALMDIYRVMRPGEPPTVEAASALFDTLFFDSERYDLSAVGRVKMNMRLALDKPDTQRTLDRDDIVACIKALVDLRDGRGDIDDIDHLGNRRVRSVGELMENQYRVGLLRMERAIKERMSSVEIDTVMPQDLINAKPAAAAVREFFGSSQLSQFMDQTNPLSEVTHKRRLSALGPGGLTRERAGFEVRDVHPTHYGRMCPIETPEGPNIGLINSLATFARVNKYGFIETPYRVVKDSTVTDEVHYMSATEEMRHTVAQANANLDDDMKFVNEMVSTRQSGDYTLAPTENVDLIDVSPKQLVSVAASLIPFLENDDANRALMGSNMQRQAVPLLQAEAPLVGTGIEEVVARDSGAAYTARRAGIIDQVDASRIVIRATEDLELGDAGVDIYRMRKFQRSNQNTCINQRPLVKVGEKVTKGQVIADGPSTDMGELALGKNVVVAFMPWNGYNYEDSILISERISRDDVFTSIHIEEFEVAARDTKLGPEEITRDIPNVGEEALRNLDEAGIVYIGADVEPGDILVGKITPKGESPMTPEEKLLRAIFGEKASDVRDTSLRVKPGDFGTVVEVRVFNRHGVEKDERALQIEREEVERLARDRDDELAILDRNIYARLKDMILGKIAVKGPKGVKANSQITEELLETLTRGQWWQLALEDEDDAKIVEALNEQYEIQKRTLDARFEDKVEKVRRGDDLPPGVMKMVKVFVAVKRKLQPGDKMAGRHGNKGVISKVVPMEDMPFLADGTPVDFCLNPLGVPSRMNVGQILETHMGWAARGLGLNVDEALQEYRRSGDLTPVREALSLAYGDDVYEEGISGMDEDTLLEAAGNVRRGVPIATPVFDGAKEADVNDSLKRAGFDTSGQSVLFDGRTGEQFARPVTVGVKYLLKLHHLVDDKIHARSTGPYSLVTQQPLGGKAQFGGQRFGEMEVWALEAYGAAYTLQEMLTVKSDDVAGRTKVYESIVKGEDNFEAGIPESFNVLVKEVRGLGLNMELLDAEEDEMPLYEHVMIARQDLSNTQAEGLIEHFGTVLADNDGKLVDSEYWGVKTMAYKINKNRKGHYAFLRSDAPATAVQEMERLMRLHDDVMRVLTIKVDEHKELPSVQMQKRDERSDRRERRMTSQIEVTALDKAQAEAELARLAEVLNAANTAYHTHDAPEMSDADYDALKQRNAAIEARFPELKRGDSPSEQVGAAVAEGFSKIRHAVPMLSLANAFEVSEVAEFDARIRKYLGLSADAPLSYTAEPKIDGLSLSLRYENGQLVQAATRGDGAVGENVTANARTIADVPHQISDAPDLLEVRGEVYMSHADFAALNARQAAAGGKTFANPRNAAAGSLRQLDAEITRARPLRFFAYAWGALSAPLAETQKGAIDRLDRFGFATNPLTALCDGPDEMIAQYQKIEEQRATLGYDIDGVVYKVNDLALQDRLGFRSTTPRWAMAHKFAAELAWTRLEAIDIQVGRTGALSPVARLQPVTVGGVVVSNATLHNEDYIKGLDSRGAEIRGGKDIRVGDWVQIYRAGDVIPKVADVDLSKRPNDAQPFVFPTTCPECGSDAIREPGDAVRRCSGGLICPAQAVEKLKHFVARGAFDIEGLGAKQVEQFYQDGWIAEPADIFTLKDRYGTGVQQLKNREGWGTKSAEKLFQAIDEKREIPLARLIFALGIRHVGEAASNLIASHYGDWQSFEAAMAQARGQSGPAWEDLLGVDGVGTVMAGSLVSAFAQEAERASIDRLAAHLTVLPAERPDTKDSPVAGKTVVFTGTLEKMSRAEAKARAERLGAKVSGSVSAKTDLLVAGPGAGSKAKKAAELGIETLDEDGWLALINDIMIHKNWAELIKPQQLDVKPGNDPARQATVMAEPLERGFGLTLGNALRRVLMSSLQGAAITSVQIDNVLHEFSSVAGVREDVTDIILNLKGVSLRMEVEGPKRLSISAKGPGVVTAGDISESAGIEILNREHVICHLDDGADVYMELTVNTGKGYVSADKNKPEDAPIGLIPIDAIYSPVKKVSYDVQPTREGQVLDYDKLTMKVETDGSITPDDAVAFAARILQDQLGIFVNFDEPESASRQDDDDGLEFNPLLLKKVDELELSVRSANCLKNDNIVYIGDLIQKTEAEMLRTPNFGRKSLNEIKEVLSGMGLHLGMDVEDWPPENIEDLAKKFEDSFMARDDNRGGNRRNQRDETPEFADRLVAINRVSKTVKGGKRFGFAALVVVGDQKGRVGFGKGKAKEVPEAIRKATEQAKRQMIRVQLREGRTLHHDMHGRHGAGKVIMRTAPEGTGIIAGGPMRAVFEMLGVKDVVSKSVGSQNPYNMIRATLDGLRKEQSPRSVAQRRGKKVADILPKRDDNVASSAQVAEEAMNDPIADMLTRIRNSSLRGKSTVVTPASKLRAWVLDVLADEGYIRGYEKVTGADGHPALEISLKYYEGEPVIRELKRVSKPGRRVYMAVNDIPVVRQGLGVSIVSTSKGVMSDASARSANVGGEVLCTVFMHDIRAIRDNPEVFDAALARRGEAAMSEAVLSLDAARRAKIAAAETAKAEQNKASKEVGAAKAKGDEAEFERLRALVSDKKAEVAAMQAEAQELDAQLNDMLARIPNNPADDVPQGASEDDNVEVKVWGDKPSFDFTPVEHYEIKGVKTGMDFETAAKTSGARFVMLKGAVARVHRALAQFMVDTHVDENGLTEVNSPVLVRDEAMYGTDKLPKFGDDSYQTTNGWWLVPTSEVPLTYSVAGETLEQSALPIRLTAHTLCFRSEAGSAGRDTAGMLRQHQFEKVEMVSITHPDESDAEQQRMVGCAEGILEKLGVPYRTVILCTGDMGFGARRTYDIEAWVPGQNSYREISSVSTTGDFQARRMNARFKPEGGGKPQFVHTLNGSGLAVGRCLIAVLENGQQADGSVKLPEVLGPYLGGKTTLTAEGVLAMAITASMVKELRDSTGAGMMDAKKALTESNGDMEAAVDWLRTKGLAKAAKKSGRTAAEGLVAVKVEGGHGVAVEVNSETDFVGKNAEFQSMVSSIADAALKVDDVEALKAAEINGKSVETTLTDAIAKIGENMSLRRMESIKGESVVSYVHNAAAPGMGKIGVLVAMNGGNDEFGKQVAMHIAAVNPASLSEADLDPAVVEKEKQVQMDIARESGKPEAVIEKMITGRMQKYMSEVTLLNQSFVVNPDLTVGKAAEEIGATITGFVRLEVGEGIEVVKEDFAAEVAKAAKGMNQELTNNPFNPLTPQKAFDEIKVSLASPERILSWSFGEIKKPETINYRTFKPERDGLFCARIFGPIKDYECLCGKYKRMKYRGVVCEKCGVEVTLQKVRRERMGHIELASPVAHIWFLKSLPSRIGLMLDMTLRDLERVLYFENYVVIEPGLTDLQYGQMMTEEEYMDAQDAYGMDAFTANIGAEAIREMLAAIDLEAEAENLRADLKEATGELKPKKIIKRLKVVESFLESGNRPEWMVMTVIPVIPPELRPLVPLDGGRFATSDLNDLYRRVINRNNRLKRLIELRAPDIIVRNEKRMLQESVDALFDNGRRGRVITGANKRPLKSLSDMLKGKQGRFRQNLLGKRVDFSGRSVIVTGPELKLHQCGLPKKMALELFKPFIYSRLEAKGLSSTVKQAKKLVEKERPEVWDILDEVIREHPVMLNRAPTLHRLGIQAFEPVLIEGKAIQLHPLVCSAFNADFDGDQMAVHVPLSLEAQLEARVLMMSTNNVLSPANGAPIIVPSQDMILGLYYTTLEREGMVGEGMVFGSVDEVQHALDAGAVHLHTKIKARIKQVDEEGNEVMKRFDTTPGRVRLGALLPLNAKAPFDLVNRLLRKKEVQQVIDTVYRYCGQKESVIFCDQIMTMGFREAFKAGISFGKDDMLIPDSKWPLVEETREQVKDFEQQYMDGLITQGEKYNKVVDAWSKCNDKVTEAMMGSISATTYHENGSEKEPNSVYMMAHSGARGSVTQMKQLGGMRGLMAKPNGDIIETPIISNFKEGLTVLEYFNSTHGARKGLSDTALKTANSGYLTRRLVDVAQDCIVRMHDCGTETAITAEAAVNDGEVVSSLAERLLGRVAAEDIVAPGTDEVIVPAGGLIDERMADAIDAAAVQVARIRSPLTCDAEEGVCAMCYGRDLARGTLVNQGEAVGIIAAQSIGEPGTQLTMRTFHIGGVAQGGQQSFLEASQSGKIVFENAQTLVNSAGEILVMGRNMKLSIVDENGEERASHKVGYGTKLFVKDGDTIERGDKLFEWDPYTLPIIAEKAGMAKYVDLVSGIAVKDDTDDATGMTQKIVIDWRAAPKGNELKPEIILVGEDGEPVRNDAGNPVTYPMSVDAVLSIEDQTEVQAGDIIARIPREGSKTKDITGGLPRVAELFEARRPKDHAIIAEIDGYVRFGKDYKNKRRIAIESSDDPDVKVEYMVPKGKHIPVGEGDFVQKGDYIMDGNPAPHDILAIMGVEALAEYMIDEVQDVYRLQGVKINDKHIEVIVRQMLQKWEIQESGDTTLLKGEHVDKQEFDTANEKALKKGGRPAKGEPILLGITKASLQTRSFISAASFQETTRVLTEASVQGKRDKLVGLKENVIVGRLIPAGTGGATQQMRKVATDRDNVVIEARREEAEAAAALAAPSASTDDIVGGDVFNTGVGDEESRDMSRRHAAEKREVLPDAKYGDLVLTKFMNNLMIDGKKSVAERIVYNAMTRVEDKIKRAPIEVFHEALENIQPSVEVRSRRVGGATYQVPVEVRPERRQALAIRWLIKAARARNENTMEERLAGELLDAVQSRGTAVKKREDTHKMADANKAFSHYRWMPYAHSDKSEGMPLLANPAPDVRNRPKLEGGKKFNLVTEFDPAGDQPTAIKELTEGVMSGERDQVLLGATGTGKTFTMAKVIEETQRPAIILAPNKTLAAQLYGEFKGFFPDNAVEYFVSYYDYYQPEAYVARSDTFIEKESQINEQIDRMRHSATRALLERDDVIIVASVSCIYGIGSVETYGAMTQDLKAGEMYDQRKVIADLVAQQYKRNDAAFQRGSFRVRGDSLEIFPAHLDDRAWRLSFFGEELESITEFDPLTGEKTDTFDQIRVYANSHYVTPKPTMSQAIIGIKKELRTRLDQLVAEGKLLEAQRLEQRTNFDIEMLEATGVCNGIENYSRYLTGRAPGEPPPTLFEFIPDNAIVFADESHVSVPQIGGMYKGDYRRKFTLAEHGFRLPSCMDNRPLKFEEWDAMRPQSVFVSATPAAWEIEQTGGVFTEQVIRPTGLLDPKVEIRPVEMQVDDLLDEVRKVAADGYRTLCTVLTKRMAEDLTEYMHEQGIRVRYMHSDIDTIERIEILRDLRLGAFDVLIGINLLREGLDIPECGLVAILDADKEGFLRSETSLIQTIGRAARNAEGRVIMYADRITGSMERAMGETDRRRAKQIAYNEEHGITPATVKKNVEDILAGLYKGDVDMNRVTAKVDNPLAGGNLQAVLDGLRSDMRKAAENLEFEEAARLRDEVKRLEAVDLAVADDPMARQQAIEKAVDTAQQASGRSTSGRGGMRGGNVK--RRMAAKPFFRRRKVCPFSGDNAPAIDYKDTRLLQRYISERGKIVPSRITAVSAKKQRELARAIKRARFLALLPYAVKMPKRILTGTVTSDANAQTVSVSVERRFTHPVLKKTIRKSKKYRAHDENNTFKVGDTVRIIECAPKSKTKRWEVLTSDKMANTTQSAKRARQNEKRFAINKARRSRIRTYLRKVEEAITSGDKEAATAALKAAQPELMRGVTKGVFHKNTASRKMSRLSARVKALGMARSVWKGPFVDSYVLKKAEASREGGRNEVIKIWSRRSTILPQFVGLTFGVYNGHKHIPVNVSEDMIGQKFGEYSPTRTYYGHAADKKAKRKMARIAGVNIPTAKRVPIALTYITGIGNSSAKAICEAVGIDETRRVNELSDAEVLAVREHIDANYTVEGDLRRDTQMNIKRLMDLGCYRGLRHRRNLPVRGQRTHTNARTRKGPAKAIAGKKKMAMKIRLARGGSKKRPFYRIVAADSRMPRDGRFIEKLGTYNPLLPKDSEERVKMDVEKIQEWIAKGAQPTERVTRMLEAAGVKEKTERNNPKKGTPGKKAQERAQEKADKAAAAAEAANAPAEEASAE-MSITAEEKAKVMKDFATKEGDTGSPEVQVAILTSRITTLTEHFKTHKKDNHGRRGLLKMVATRRKLLDYVKAKDESRYQDLIKRLGLRRMKTLNDIRSTFLNYFDAQGHQVVPSSPLVPRNDPTLMFTAAGMVQFKNLFTGVETRDYSRAASAQKCVRAGGKHNDLDNVGYTARHHTFFEMLGNFSFGDYFKAEAIPFAWDLLTKEFGIDPNRLLVTVYHTDEEAVNIWKAHTGLPDERIIRIATDDNFWSAGPTGPCGPCTEIFYDHGDHIWGGPPGSPEEDGDRFVEIWNLVFMQYEQFADGTRQPLPNQSIDTGMGIERVAALLQGTNDNYATDLMRSLIEASAHASSTDPDGPGKTHHRVIADHLRSTSFLIADGVMPSNEGRGYVLRRIMRRAMRHAHLLGVKDPLMHQLVPSLVQQMGAAYPELGQAQSLIRETLLLEETRFRQTLDRGLKLLDDELSGLPEGATLPGEAAFKLYDTYGFPLDLTQDALREKGRAVDTEGFDTAMAAQKAKARAAWAGSGEAADATVWFNVADEHGVTEFLGYDTETAEGQIVALVQGGDLVEVAAISSDVQVALNQTPFYAESGGQVGDTGIIRTPTGRVEVTDTRKSAGVFVHFGHVVEGEIKPGQTAVLEVDSARRTAIRANHSATHLLHEALRNALGEHVSQRGSLNAHDRLRFDFSHAKGLTQEELSQVEREVNDYIRQNTPVETRIMTPDDARAIGAQALFGEKYGDEVRVVSMGQLEGSGKGSDKSTYSLELCGGTHVRQTGDIGAFVLLGDSASSAGVRRIEALTGTEALNWLREQEAALSRVAAELKTSTSDVPDRVRALLEERRNLSNEVAQLRRELAMA-GGGAAAPEAREVNGVKFVGQVLNGVTGKDLPALVDEHKAKLGSGAVLLIADTGSKAAVAGGVTKDLTDRFSAVEMVKAAVAELGGKGGGGRPDMAQGGGASAENAEAAIAAAENTLKGMGFKMGIVGLPNVGKSTLFNALTRTAAAQAANFPFCTIEPNVGEVAVPDSRLDTLAEIAKSKSIIPTRMTFVDIAGLVKGASKGEGLGNQFLANIREVDAIAHVLRCFEDGDVTHVEGRVDPVADAETIDTELMLADIESIEKRLQNIVRKVRGGDKEAVQQERLMRKALEALEAGKPARVVEVDEDDAKAWRMLQLLTTKPVLYVCNVGEAEAAEGNAHSAKVAEMAAAQGNSHVVISAQIEEEISQLEAEEAEMFLEEMGLKEAGLDRLIRAGYELLHLETYFTVGPKEARAWTIKSGTSAPKAAGVIHGDFEKGFIRAETITYEDFVSLGGEGPAKEAGKMRAEGKSYIVKDGDVLHFLFNTMGWKTLDDMDLNGKRVLLRVDINVPVEDGRVTDATRIERIVPTVNDILSRGGKVTLLAHFGRPKGKAVDAMSLKQILPALKEALDRDVSFVPSLEAAA-----TAEDDLQLMENIRFYPGEEVNDADFAGQLADLGDIYCNDAFSAAHRAHASTEALARLLPACAGRLMQAELSALEKALAKPERPVGAVVGGAKVSTKIALLENLVNKLDLLVIGGGMANTFLAALGADLGKSLQEPDYFDTANDIMAQAEKAGCRVILPVDGLVAREFAKGAAHEVAQLGPDAKLASDQMVLDAGPDTVALVETAFAGLRTLIWNGPMGAFEIPPFDTATVAAARAAAKQTRDGTLTSVAGGGDTVAALNQAGVAEDFTYISTAGGAFLEWMEGKTLPGVAALGGMALPEFSMRQLLEAGVHFGHQTQRWNPRMGPYIYGARNGIHIMDLTQTVPMLDDALKVIRDTVAKGGSVLFVGTKRQAAQPIADAAEKCAQYYMNHRWLGGTLTNWQTVSQSINRLKSIDEQSERGFEGLTKKERLGMERDQSKLEASLGGIREMGGRPDLLFVIDVKKEALAIAEANKLGIPVVAVVDTNCSPDGIDYIIPGNDDAARAIALYCDLAARAALDGMSAQLGAAGVDLGAMEEAPQEEALTEERNASEETLHDDAMSKDAESMTKRTAAKHKLDRRMGENIWGRPKSPVNRREYGPGQHGQRRKGKISDFGIQLRAKQKLKGYYGDLTEKQFRRIYGEAERVKGDTGENLIGLLERRLDAVVYRAKFVPTVFAARQFVNHGHVKVNGRKVNIPSYRVKEGDVIEVRDRSKQLASVLEAVQLPERDVPDYIEVDHSKLTATFVRSPSLGDVPYPVVMEPNLVVEFYAKNMGNKVNPIGMRLQVNRTWDSRWYADTKDYGDLLLEDLAIRDFIKKECHQAGVARVIIERPHKKCRVTIHTARPGVIIGKKGADIETLRQKIAKMTKSELHLNIVEIRKPELDAHLVGESIAQQLERRVSFRRAMKRAVQNAMRMGALGIRVNLAGRLGGAEIARTEWYREGRVPLHTLRADIDYAHVEAATAYGIIGIKTWIFKGEIMEHDPAARDRKAQELQDGPAPRGAGGRRMARKRKGRDISGWLVVDKPAGPTSTAVVNKVRWALEAKKAGHAGTLDPEATGVLAIALGEATKTVPYITDALKAYEFTVRLGIATNTDDAEGEVIGTSDLRPDDAAIKDALSGFIGDIQQVPPQFSAVKIDGQRAYKRARDGEEMDIAARPLWVESLLLVDRPDADHVTLEMVCGKGGYVRSIARDLGQKLGCLGHVRELRRTWSGPFEAKDALTLAQVDELARTPELDTHLLPLAEGLADLPEVKATPEGATRLRNGNPGMVIAHDVEYGDECWASLDGQPVAVGRFKAGELHPSRVFNLSTMFHLGLTGSIGMGKSTTAKMFAEEGCAVWDADAAVHRLYAKGGAAVAPMQAEFPAAIVEGAVSRGALKEAIAADPTALPRIEAIVHPLVAEDRATFLADAESDIAVLDIPLLFETGGDNAMDAVACVNIPDDIQRDRVLARGTMTEAQFDAIRAKQMPAKEKCARSDYVIETDTLDHARAQVRAVIRDIREKLRHAMEDFDVLIEDDRWDTVDLEPLAHRAAAATLGHLGLAPATAELTLLACDDKRIAALNEDFRGKPRATNVLSWPAEERGAAAPGGDPLPVSPGVDGMLELGDIALAYETCAAEAKAGDKPLADHVTHLIVHGLLHLLGYDHENDPDALLMEGLEREILGKMGLDDPYKESEPMARYIFITGGVVSSLGKGLASAALGALLQARGFSVRLRKLDPYLNVDPGTMSPFEHGEVFVTDDGAETDLDLGHYERFTGVAARKTDSISSGRVYSNVLEKERRGDYLGKTIQVIPHVTNEIKDFIEIGDDEVDFMLCEIGGTVGDIEGLPFFEAIRQFSQDKPRGQCIFMHLTLLPFVKASGELKTKPTQHSVKELRSIGIAPDILVCRSEGPIPAKEREKLALFCNVRPDSVIAAQDLKSIYEAPLAYHREGLDQAVLDAFQITPAPKPNLSRWEDVADRIYNPEGEVKVAIVGKYTQLEDAYKSIAEALTHGGMANRVKVKVEWVDAEIFDSEDAGPHLEGFHAILVPGGFGERGTEGKIKAAQYAREHNVPYLGICLGMQMAVIEAARNVAGLKTAGSEEFDHEAGKKRFEPVVYHLKEWVQGNYKVARKVDDDKGGTMRLGAYDATLVEGSRVAEAYGTTTIDERHRHRYEVDIAYKDQLEKVGLKFSGMSPDGKLPEIVEWTDHPWFIGVQFHPELKSKPFDPHPLFKDFVRAAKDVSRLVMPRPQLILGLESSCDDTAAAIVRIDEDGRGAVLSSVVAGQTALHADFGGVVPEIAARAHAEKLDHCVEDALAEAGIALPQIDAIAVTAGPGLIGGVVSGVMCAKGLSAATGKPLYGVNHLAGHALTPRLTDDVPYPYLMLLVSGGHCQFLMVRGPNNFERLGGTIDDAPGEAFDKVARLLGLPQPGGPSIEACAKTGDPERFALPRPLLDRDGCDMSFSGLKTAVLRARDKAMADAGGLTHRDQADLAAGFQAAVVEVLAKKTRRAFANYPQDAP--LGLCVAGGVAANQSIRAALEAVAAEQSARFIAPPLALCTDNAAMIAFAAAEQAAERGFDDLSLSARPRWPLDTVRPSMLGSGKKGAKA------------------------------------------------------------------------------------------------------------------------------------

>'Su-noctilucicolaNB-77'

MNAKELHDKTPDQLRDELVNLKKEAFNLRFQQATGQLENPARLRSVKRDVARVHTVLNQKAATAATEAMATADLLTMDNKKTAEKQKALDSALAQIERQFGKGSIMKLGSEGAIQDIQSSSTGSLGLDIALGIGGLPMGRIIEIYGPESSGKTTLTLHCVAEQQKAGGVCAFVDAEHALDPQYARKLGVDIDELLISQPDTGEQALEITDTLVRSGAVNMVIVDSVAALTPKSELEGEMGDSSVGVQARLMSKAMRKLTGSISRSNCMVIFINQIRMKIGVMFGSPETTTGGNALKFYSSVRLDIRRIGSLKDRDEVVGNATRVKVVKNKVAAPFKQVEFDIMYGEGISKMGELLDLGVKAGVVDKSGSWFSYGDERIGQGRENAKQFLKDNEAMAMDIEDKIRAAHGLDFHGSDGDDSDILEAMSRYSPAEIEARWQSAWEKNETFKAVRDAAKPKYYVLEMFPYPSGRIHMGHVRNYTMGDVIARYKIATGHNVLHPMGWDAFGMPAENAAMAIGGHPKDWTYGNIKDMRDQMKPLGLSIDWSREFATCDPEYYGQQQALFLDFLDADLVYRKNAIVNWDPVDMTVLANEQVEAGRGWRSGALVERRELTQWFFKISEHSEELLDALDSLDNWPAKVKLMQANWIGKSRGLQFAFSTVDAPDGHDRIEVYTTRPDTLLGASFVGISPDHPLAKVLEKDNPEVAAFCAECRKGGTTEEAIETAEKLGYDTGITVRHPFDTSHELPVYIANFILMDYGTGAIFGCPAHDERDFEFATKYDLPIISTYLPSEESDDTLNEAYVPAKTEKVFYNRGFAGDKWQTGNEAIDAAIAFCESNGVGQGVTKFRLRDWGLSRQRYWGCPIPVVHCDACGVVPEKKENLPIELPYDVDFDTPGNPLDRHPTWRDCACPSCGAPAKRETDTMDTFVDSSWYFARFTAPDAETPTNMEDAKYWMNVDQYIGGIEHAILHLLYSRFFARAMQITGHLPESAIEPFDALFTQGMVTHAIYQTPKQDGRPTYHYPEEVRDGKAFKDGNEVEVVPSAKMSKSKNNVVDPLNIISNYGADTARWFVLSDSPPERDVEWTASGAEAAFKHLGRVWNISDRIGAMDKDAAGSGDDELLRTMHKTIHDVTMGVESFGFNAAIAKLYAFTAALQKSKAGYAAQREAVMTLAQLMSPMTPHLAEDIWTQQGGDGMIVTAPWPKADEAMLVDDTVTMPIQINGKRRAEIQVPADMAKEEVEKIALAHEAVIRTLDGATPKKVIVVPGRIVNVVAMAITSANQLELLQTAEAVAREKMIDPALVIEAMEESLARAAKSRYGAEMDIRVSIDRKTGRAKFTRVRTVVTDEELENYQAEFTVEQAKQYMENPAVGDTFVEEVPPVEMGRIAAQSAKQVILQKVREAERDRQYEEFKDRAGTIINGLVKREEYGNVIVDVGAGEAILRRNEKIGRESYRPNDRIRVYIKDVRREQRGPQIFLSRTAPEFMAELFKMEVPEIYDGIIEIKAVARDPGSRAKIAVISYDGSIDPVGACVGMRGSRVQAVVNELQGEKIDIIPWNDDQPTFLVNALQPAEVSKVVLDEEAGKIEVVVPEEQLSLAIGRRGQNVRLASQLTGLDIDIMTEEQESQRRQAEFELRTKLFMDNLDLDEFFAQLLVSEGFTNLEEVAYVEVDELLVIDGVDEDTAGELQARARDVLEAQNKAALEAARALGVEDSLVEFEGLTPQMIEALAKDGVMTLEDFATCADWELAGGWTTVDGERVKDDGSLEPFEVSLEDAQNMIMTARVMLGWVDPAELEAEAVEE----DEEASEEIEAMAHKKAGGSSRNGRDSAGRRLGVKKYGGEAVIPGNIIVRQRGTKFWPADGVGMGKDHTIFATVDGSVTFHKGLKNRTFISVLPAAEAAEMAKRWYSVSVLSNFEKKIAEQIRTNVEEQGLEDQIDEVLVPTEEVIEVRRGKKVTTERRFMPGYVLVHMEMSDQGYHLINSINRVTGFLGPQGRPMPMRDAEVQGILGRVQEGEDAPRTLIHFEVGEKVKVGDGPFEDFDGMIEEVDDENQRLKVSVSIFGRETPVELEYTQVIKQSMSFTLAIVGRPNVGKSTLFNRLVGKRLALVDDQPGVTRDLREGAARLADLRFTVIDTAGLEEVTDDSLQGRMRRLTERAVDMADICLFMVDARVGITPSDLVFADILRKRSANVILAANKAEGKAGEAGVMEAYTLGLGEPIALSAEHGEGLNDLYTMLMPLADAFAERTEDDTPETDIDLEEDAED--HVPVPTDAKPLQVAVVGRPNAGKSTLINQIMGEDRLLTGPEAGITRDAISLRKDWNG-VPMRIFDTAGMRKKAKVQEKLEKLSVSDGLRAVKFAEVVVVLLDAEIPFEQQDLRIADLAEREGRAVVIAVNKWDIEEDRQAKLKGLKESFERLLPQLRGAPLITVSAKTGRGLDRLHDAIMRAYETWNRRVTTAQLNRWLAGMMEAHPPPAPQGKRIKLRYMTQAKTRPPGFVVMCSHPDKVPESYSRYLVNNLRVDFDMPGTPIRLWMRGQNDANPYKGRKKAPPSKLRKHTEGRRKDMRIVFMGTPEFSVTVLDALVDAGHEIAAVYCQPPRPAGRGKKDRPTPVHVRADALGLEVRHPVSLKTPEAQAEFAALKADIAVVVAYGLILPQAVLDAPSKGCLNIHASLLPRWRGAAPIHRAIMAGDKKTGVCIMQMDAGLDTGPVLLRREIEIGATQTTAQLHDDLCSLGATAIVDALAQIDTLTPQPQPEEGVTYAAKIDKGEARIDWAQPAAQVDRLIRGLSPSPGAWFEMDGTRIKVLGSVLAQGSGKPGEVLDADLRVACGKGAVQLLRLQRAGKAAQDTAVFQSGAQIAPGTELNGDMSDTAAYQVFARKYRPETFADLVGQDAMVRTLKNAFEADRIAQAFIMTGIRGTGKTTTARIIAKGMNCIGEDGNGGPTTEPCGVCEHCTAIMEGRHVDVMEMDAASNTGVANIREIIDSVHYRAASARYKVYIIDEVHMLSTGAFNALLKTLEEPPEHVKFIFATTEIRKVPVTVLSRCQRFDLRRIEPEVMIALMRKIATAENAEIADDALALITRAAEGSARDATSLLDQAISHGAGETTAMQVRAMLGLADRGRVLDLFDMILRGDAAGALTELGGQYADGADPMAVLRDLAEITHWVSVVKITPDAAEDPTISPEERSRGTQMATDLPMRVLTRLWQMLLKALDEVASAPNAMMAAEMAIIRLTHVADLPSPEELVRKLNKTAPPPPSSAPGSGSP-SAPSGGAQAVQQAQTRMQSAPTGNGQATAVAQDVEAALGRFPTFEHVVELIRANRDVKLLVEVETSLRLAAYQPGRIEFVPTDDAPRDLAQRLGHRLQGWTGNRWAVSIVNEGGSETIAEVRDAKDLALRASAQDHPMMQAVLSKFPNARITAIRTPEQIAADAVSEALPEVEDEWDPFEDGMPKMKTKSSAKKRFKISATGKVIGGQAGKQHGMIKRTKKFIRNARGTTALSEPDAKIIKGFMPYDRMNIFAEIRELILTTLEQMVTAGDLPDGLNLEPITTEPPRDPAHGDMATNAAMVLAKPAKMKPRDIADALAGKLAEDPRITSAEVAGPGFLNLRLAPTVWQGIAKTVLSEGTGFGRGSLGQGQKVNVEYVSANPTGPLHVGHTRGAVFGDALASLLDFAGYDVTREYYINDGGAQVDVLARSVYLRYLEAHGQEVAFPEGTYPGDYLIAVGEALKDKVGDDYLDKGEQYWLEDVRSFATEKMMDLIRSDLKALGVEMDVFYSEKSLYGTGRIEAALEDLKNKGLIYEGVLEPPKGKKPEDWEPREQTLFKSTEHGDDVDRPVMKSDGGWTYFAPDIAYHYDKVQRGFDQLIDVFGADHGGYVKRMKAAVSALSDGKVPLDIKLTQLVKLFKNGEPFKMSKRAGTFVTLRDVVDQVGPDVTRFVMLTRKNDAMLDFDFDKVLEQSRENPVFYVQYAHARVASVLRKAAEAGIATDDATLAAADLSLLNHESELGVLRKLAEWPRLVETAARTNEPHRVAFYLYELAGDLHGLWNKGNEVTSLRFIQD-DAATSQAKIALARSTAIVIAAGLGILGVNPAEEMRMSVIDTLPPLREVIATHGLSARKSLGQNFLLDLNLTAKIARQAGDLTECDVLEIGPGPGGLTRGLLSEGARHVLSIEKDTRCLPALADVAAAYPGRLTVVEGDALEIDPLAHLTLPIRVAANLPYNIGTELLVRWLTPKEWPPFWQSLTLMFQREVAERIVAQPGSKAYGRLAILSQWRADARIVLQLPPGAFTPPPKVSSSVVHLTALPEPRFPADAAVLSRVVAAGFNQRRKMLRASLKGIAPDIEDRLIAAGIKPTDRAEQIPLEGFCALAREVARAMNLIAEIEAEQIAELAKEIPDFRAGDTVRVGFKVTEGTRTRVQNYEGVCIARNNGQGIAGSFTVRKISFGEGVERVFPLHSTNIDSITVVRRGRVRRAKLYYLRSRRGKSARIVENSNYKPKKA---MANSKRQLFIKRRLRVRNKLRRVNAGRMRLSVHRSNKNISVQLIDDLNGKTVASASTLEKDLGFVGKNNIEAASKVGAAIAERAKKAGVEEAYFDRGGFLFHGKVKAVADAAREGGLKIMFAVIKTGGKQYKVASGDVLRVERIAANAGDKVLFNEVLMLGGDSPKVGAPLIEDAGVEAEVVDQIKAEKVIHFVKRRRKHSSKRTKGHRQKLTLIKVGEILASGAGKSKSMTAVGTGSVPAATVAAEMAKKKAKP-------APT-K-PKAEAAPKKAAKSD-G-DDLSEISGVGPVIVGKLHAEGITTFAQIAAWTDADVEAIEEKLSFKGRVGREDWIAQAKDLAKGMARVKGGTVTHARHKKIVKAAKGYYGRRKNVFKVATQAVDKANQYATRDRKNRKRNFRALWIQRINAAVRSHDEALTYSRFINGLNLAGIEVDRKVLADLAVHEPEAFGAIVKTAQDALAAMQVNETLNEGLKRGYAITVTAAELDAKVNEKLAEAQPEVEMKGFRKGKVPMALLKKQFGQRIMGEAMQESIDGAMAEHFEKSGDRPAMQPEVKMTNEDWKEGDDVEVEMSYEALPEIPELDMSKIKLEKLVVKADDAAIDEALANLAETAQDFKARKKGSKAKDGDQVVMDFVGKVDGEAFEGGSAEDFPLVLGSGNFIPGFEEQLVGVKAEEEKSVTVTFPEEYQAEHLAGKEAVFDCTIKEVKEPVAAEINDELATKFGAEDLAGLKAQIAERLEAEYAGAARAVMKRGLLDALDKMVDFDLPPSLLEAEASQIAHQLWHEDNPDVQGHDHPEIETTDEHKTLATRRVRLGLLLAEIGQKAEVEVSDAEMSQAIMAQARQYPGQERQFFEFVQQNQQMQQQLRAPLFEDKVVDHIAEGAKVTEKEVSKEDLQKAVEALEDAYKLNELSDNPGATKKRKRVGRGPGSGTGKMGGRGIKGQKSRSGVAINGYEGGQMPLYQRLPKRGFNKPNRKSYAVVNLGLIQKFIDEKKLDAKKPVDAAALIASGLVRRELDGIRVLAKGDITAKVDLHVAGASKSAVEAVEKAGGSVTLPA-KAEAAEAMIQMQTNLDVADNSGARRVQCIKVLGGSKRKYASVGDIIVVSVKEAIPRGRVKKGDVRKAVVVRTAKEVRREDGTAIRFDRNAAVILNNNNEPVGTRIFGPVVRELRGKNFMKIISLAPEVLMRHARGYRRLNRTHEHRKALWSNMAGSLIEHEQIKTTLPKAKELRPVIEKMITLAKRGDLHARRQAASKLKQDKYVAKLFDILGPRYKDRQGGYVRVLKAGFRYGDMAPMAIIEFVDRDRDAKGAGDKARVAAEEAAE-MLQPKRTKFRKQFKGSIKGLAKGGSDLNFGTYGLKALEPERVTARQIEAARRAMTRHMKRQGRVWIRIFPDVPVTSKPVEVRMGKGKGSVDFWACKVKPGRVMFEIDGVNDDVAREALRLAAMKLPIKTRVVVREDWMTEITLYNTKTRRKELFTPLREDDVRMYVCGPTVYDRAHIGNARPVIVFDLLYRLLRHVYGPDHVTYVRNFTDVDDKINARAAQSGRPISDITAETAQWFLDDMAAVGALQPSAMPRATAYIDEMIAMIEDLIAKDHAYAAEGHVLFRVRSYAQYGALSGRSVDDMIAGARVEVAPYKEDPMDFVLWKPSDAETPGWDSPWGRGRPGWHIECSAMAEALLGSEFDIHGGGNDLTFPHHENEIAQSKCAG--HGFANVWMHNEMLQVEGKKMSKSLGNFFTVRDLLDQGVPGEVIRFVFLSTHYRKPMDWTEKKRAEAEKTLRKWYTQVAGGETGQP-PAEVMALLGDDLNTHGVLTECHRLSAENDIAGLRAALSVMGLLADKIPDWAIEQAFDLTDIEAFLSDARVTAMETKDFAEVDRIKSALTAVGIEVQMSKDGVKLTAPAGFDRSDLEGILMSQVKSSTKSDPNYKVIAENRRARYDYAIEEDIECGIMLEGSEVKSLRMGGSNIAESYAAVEDGELWLVNSYIAPYKQAKTFGHEERRRRKLLVSRKQLADLWSATQRKGMTLVPLVMYFNHRGMAKVKIGIAKGKQNHDKREASAKRDWSRQKSRLLKDHGMSAKHNHYDVIRKPIITEKATMASEANAVVFEVAMESNKPMIKEAVEALFGVKVKAVNTTITKGKVKRFRGQLGKRKDVKKAYVTLEEGNTIDVSTGLMSKDKNPRRVADNEARAKLRMLKTSPQKLNLVAGLIRGKKVERALTDLTFSKKRIAQDVKKCLQSAIANAENNHNLDVDELIVAEAYVGKNLTMKRGRPRARGRFGKIMKPFAEVTIVVRQVEEQAMVSAVENMAANTSWSALGKATDLRNRILFTLGLLIVYRLGTFIPVPGIDGGALREFMDSAGQGIGGMVSMFTGGALGRMGIFALGIMPYISASIIVQLLGSMYGPWEQLKKEGEQGRKKLNQYTRYGTVALATLQAYGLAVSLEAGDIAADPGMYFRIACMITLVGGTMFLMWLGEQITARGIGNGISLIIFVGIIAEVPAAIAQFFASGRSGAISPAVIVGVLVMVVLTIMFVVFMERALRKIHIQYPRRQVGQKMYDGGSSHLPVKVNPAGVIPAIFASSLLLLPITISTFSGNSTGPVMSVLLANFGPGQPLYLLFFIAMIVFFAYFYTFNVSFKPDDVADNLKNQNGFVPGIRPGKKTAEYLEYVVNRVLVLGSGYLALVCVFPEILRGQFAIPFYFGGTSVLIVVSVTMDTIQQVQSHLLAHQYEGLLEKSNLRGKGKGTRKKRSPVRRMKFLDLCKVYIRSGAGGGGCISFRREKYIEYGGPDGGDGGGGGSVWAEAVDGLNTLIDFRYQQHFFAKNGQPGMGKQRTGKDGEDIVLRVPVGTEILDEDQETVIVDITEVGQRVELARGGNGGWGNLHFKSATNQAPRRSNPGQDGVERTLWLRLKLIADVGLLGLPNAGKSTFLAATSNARPKIADYPFTTLHPNLGVVGVDNTEFVVADIPGLIEGASEGRGLGDLFLGHVERCAVLLHLIDGTSDTVAEDYQTIITELEAYGGDLAEKPRVTVLNKVDALDEEERVTKRKELEKACGGPVMMMSGVAREGVTEVLRTLRGEIDDDRLRQKPAEEAVQWHPMAAKLRKGDKVIVLAGKDKGKTGNITSVMPSANKAVVEGINISIRATRQSQESQGGRIPKAMPIDLSNLALVDANGKATRVGFKMEGDKKVRFAKTTGDVIDAMLRSGVIAKKLGMTRLFMEDGKQIPVTVLHLDNLQVVAQRTVEKDGYVAVQLGAGTAKAKRTSQAMRGHFAAAKVEPKRKVAEFRVDADAMLPVGEEIIADHYFAGQYVDVAGTSIGKGFAGAMKRHNFGGLRATHGVSISHRSHGSTGQCQDPGKVFKGKKMAGHMGAARVTTQNLEVVKTDAARGLIMVKGAVPGSKGGWVTVKDAVKKPFPDNAIVPAALASAAREAAKAAEEAAAAAAAEAEAEAARLAEEQAAAEAEALKAAEAEIAEEGATPEAGDA-DKKEGDAMALKSYKPTTPGQRGLVLIDRSELWKGRPVKALTQGLTKSGGRNNTGRITMRRTGGGAKRLYRIVDFKRNKLDMSAVVARIEYDPNRTAFIALIQYEDGEQAYILAPQRLAVGDKVIASQKADIKPGNAMPFSGMPIGTIIHNIEMKPGKGGQIARAAGTYAQFVGRDGGYAQIRLSSGELRLVRQECMATVGAVSNPDNSNQNYGKAGRMRHKGIRPSVRGVVMNPIDHPHGGGEGRTSGGRHPVTPWGKPTKGAKTRNKNKASSRLIVRSRHAKKKGRMLDNAEYTPRLLTVYRDEIRAKMREEFGYSNDMMIPRLDKVVLNIGCGAAAVRDSKKAKSAQEDLTLIAGQKAMTTIAKKSIAGFRVREEMPMGAKVTLRGARMYEFMDRLITIAMPRIRDFRGISAKSFDGRGNYAMGLKEHLVFPEIDFDKIDENWGMDIVIATTAKTDAEAKALLTAFNMPFTKMKLDVIKLDGGKAGSVDLDEALFGLEPRADILHRVVRWQRNNAQAGTHKVKTRSEVDYSTKKIYRQKGTGGARHGARSAPIFRGGGVYKGPKVRSHGHELTKKFRKLGLRHALSAKAKAGELVIIEDATSEGKTAALAKQISNLGWKRALVIDGAAVNENFAQAARNIEGLDILPTMGANVYDILKRDTLVITKAGVEALEARLKMENVVLIIHLLLALGLIAVVLMQRSEGGGLGM-GGGGGAVSGRAAATALGKLTWLLGAAFIVTSITLTVLVAQKSSGASVIDRLGVTPPAAS---DTTVPSGDLLLPPSADDNAPLVPLADMAFFKKLKDRLFKSSSKIDEGLEAIVSDGGEEE-AAVDEVMIEA--PAAAVETEDRAAAREARAQAEEEALAERLAAEQAARDRAAEEEAEEKREEQAAAREAEAARAEEAAEQTEPLRTTLTPVAPDLAA---ATQSAAKPGLLGRLMGRSTPKTVVRRTLDDDMLEQLEELLITADMGVDTALRVTANMSEGRFGKKLSVAEIKELMASEIARIMEPVARPLPLYPKTPQVVLVVGVNGSGKTTTIGKLASQFRAAGKKVVIAAGDTFRAAAVEQLQVWGDRAGVPVLTAAQGSDPASLAFDAMTKAEADGADLLLIDTAGRLQNRGDLMEELAKIVRVIRKKDETAPHNTLLVLDATTGQNAINQVKVFQEISDVSGLVMTKLDGTAKGGVLVALADKFGLPIHAIGVGEQIDDLSPFDPQEFADALVGYERMTHEQWGEIRQRLLKTVGQNNYKNWIEPIIPGNVEAGIATLNVPTNFFGNYVSQNFSDLILHEINAEGIEVSRLNFAMNPEKVANAPALATKAVESPSKTAAS-SRTDITSTAPLDPRFSFDNFVVGKPNELAHAAARRVAEGGPVTFNPLFLYGGVGLGKTHLMHAIARELKERKPEMTVLYLSAEQFMYRFVQALRDRKMLDFKDLFRSVDVLMVDDVQFIAGKDSTQEEFFHTFNALVDQNKQIIISADRAPTDIKDLEERVQSRLQCGLVVDLHPTDYELRLGILQNKVEAQQKTYPGLDVADGVLEFLAHRITSNVRVLEGALTRLFAFASLVGREIDMDLTQDCLVDILRSSERKITVEEIQRKVSEHFNIRLSDMIGPKRLRSYARPRQIAMYLCKQLTTRSLPEIGRRFGGRDHTTVMHGVRRIEELKQSDGQIAEDLELLGRALKGMLGIGTIAKKVFGTPNDRKIKATRPLVEKVNALEPEFEKLSDDGLKDKTEELAKRAMAGESLDDLLPEAFANCREAARRTLGLRAFDTQVMAAIFLHQGNISEQKTGEGKTLTAAIAAYLNALPGKGVHVVTVNEYLVQRDADWMGKVFAALGLTTGAAISGMNTEAKRAAYGCDITYATNNELGFDYLRDNMKSNLDEILQKHHFFAIVDEVDSILIDEARTPLIISGPAQDRSEMYVTINKLIPSLKPEHYELDEKTRNVTFTDDGNEFLEELLRAEGLMEEGQSLYDPESTTIVHHVNQGLRAHKLFLKDKDYIVRDGEIMLIDEFTGRMMQGRRLSDGLHQAIEAKEGTSIMAENVTLASVTFQNYFRLYDKLAGMTGTALTEAEEFQEIYGLGVVEVPTNLPIARVDEDDAVYRTVGEKYKAMIDKVKEANAKGQPCLVGTTSIEKSEQLSQMLTAEGIPHNVLNARQHEQEAQIIADAGKLGAVTIATNMAGRGTDIQLGGNVDLKVLEAITADPEADPNEVRARIEAEHAEEKKKVLEAGGLYVLASERHESRRIDNQLRGRSGRQGDPGRTSFFLSLEDDLMRIFGSERLEKVLTTLGLKEGEAIVHPWVNKSLERAQAKVEGRNFDIRKQLLKFDDVMNEQRKVIFGQRRDIMESDDLSEITTDMRHQVIDDLIDVYMPPKTYADQWDSKGFHEATMEQLNINLPIVEWCEEEGVDDEVIRERMIEATDKMMADKADVFGAENMRNIEKQLLLNAIDTKWQEHLMTLEHLRSVVGFRGYAQRDPLNEYKNEAFQLFEGLLDSLRQEVTQKLGQIQPMSEEERAAMIEELRAQQEAAKAAATAE--EPLPEPTAAA-IENGFVEDDPTTWGTPGRNDMCPCGSGKKFKHCHGRVNMKLSGDLKAFEGRIGHQFSDPALLVRAVTHASMSSANRDDNQRLEFLGDRVLGLVMAQALLERDPGASEGQLAPRFNALVRKETCAEVAREIDLGKVLKLGRSEMISGGRRKMALLGDAIEAVIAAVYLDAGFDAAMAMILQLWGSRVDTVEDDARDAKTALQEWAQARKLQPPKYVQTDRSGPDHAPVFTIAARLETGEEAQAMAPSKRAAEQAAATSLLAQLENDTMAKLGKRTAAAREAFAGKEDVTVEEAVALIKANSKVKFDETVEIAMNLGVDPRHADQMVRGVVGLPNGTGKTMRVAVFARGPKAEEATAAGADIVGAEDLMEIVQSGKIEFDRCIATPDMMPIVGRLGKVLGPRNLMPNPKVGTVTMDVKEAVEAAKGGQVQFKAEKGGVVHAGVGKASFGEAQLVENIRAFVGAVAKAKPAGSKGTYMKKINLSSTMGPGVSVAVDNATGEMSEDFMLDTDDLERRMNGAIASLRTEFASLRTGRASASMLEPVMVDAYGSMTPINQVGTVNVPEPRMVTINVWDKGLVGKVEKAIRESGLGINPQLNGTIIMLPIPELNEERRTQLTKVAGTYAENARVSIRNIRRDGMDQIKKAKADGMSEDDQKIWESEMQDLTNKFTSMVDEQLEVKQAEIMQVMAKKLVGTMKLQIKAGQANPSPPVGPALGQRGINIMEFCKAFNAKTADMEPGAPCPTVISYYQDKSFNMDIKTPPASYYLKKAAKINSGAKTPSRETVGTVTAKQLREIAEAKMVDLSANDVEQAMKIILGSARSMGIEVKMFENLSERLSGVFDRLTKQGALSEEDVKTALREVRVALLEADVSLPVARDFVKAVQDKATGQAVTKSITPGQQVVKIVHDALIDVLSGEGEPGALKVDNPPAPILMVGLQGGGKTTTTAKLAKRLKERDGKRVLMASLDVNRPAAMEQLAILGVQIGVDTLPIIKGEDPVAITKRAKTQAGLGGYDVYMLDTAGRLSIDEELMQQVEAVRDVVTPRETLLVVDGLTGQDAVHTAENFDERIGISGVVLTRMDGDGRGGAALSMRAVTGKPIKFVGLGEKMDALETFEPERIAGRILGMGDIVALVEKAQETIEAEQAEKMMKRMAKGQFNMNDLKMQLEQMMKMGGMQGMMGMMPGMGKMAKQVEDAGLDDKVLLRQIALIQSMTKKERANPALLQASRKKRIAKGAGMEVSDLNKLMKMHRQMSDMMKKMGKMGKGGMLKQAMKGMIGKGGMDPAAMAGQMDPKAMEAAAKAMGGKLP----GMGGGMGLPSGLSGFGKKKMIPMDRLAQITQRFQFLEASMSAGSDGADFAALAKEYADLRPVVEQIEAYRELLDGRAECEAMLADPEMAELARDEMASIKEALPKAEEALQIALLPKDAADAKPAMLEIRPGTGGDEAALFAADLLRMYQRYAEARGWGFDMVEEQMTELGGVKEVVAHITGQNVFARLKYESGVHRVQRVPTTESGGRIHTSAATVAVLPEAEDVDIEINPNDLRIDTMRSSGAGGQHVNTTDSAVRITHLPTGLVVTSSEKSQHRNRDKAMQVLKARLYDMERSRVDSERSADRASQVGSGDRSERIRTYNFPQGRMTDHRINLTLYRLDAVMQGDLDEIVDALTADAQARQMAEMNQMDRAQKEQLVDELGQIFESSGVVVVSHYVGLTVAEMQDLRARARAAGGAVRVAKNRLAKIALEGKPCESIGDLLTGMTVLTYSEDPVAAAKVAQEFAKENDKLVILGGAMGENALDAAGVEAVSKMPSREELISTIAGMLGAPASNIAGAIGAPASNIASILSTIEDKAA-MKTFTATPADIDKKWIIIDAEGVVLGRLASIVAMRLRGKHKPSFTPHMDCGDNVIIINAEKVQMTGKKREEKFYWHTGHPGGIKERTKAQILEGAHPERVVTQAVKRMLPGNRLSRQIMTNLRVYAGTDHPHEAQSPEVLDVKAMNKKNTRSA----------------MPHPPQTMGVAVSGGSDSMALLHLLSEFCAIHDIKLRAVTVDHRLREASTEEADSVARHCAKIGVPHDTLVWQDWDGEGNLQNAAREARYSKMAAWASSNGIDTIAVGHTADDQAETVLMRIARRSGVDGLSAMRPRTVREGITWVRPLLQTKRQTLKTYLTNQDITWVSDPSNEDERYDRIRARKALDLLAPLGIDVDALGEVAKHMAQARKALDWHTFLAAKQIVQIKDGALLIEETGLRLQPDEVQRRLMVKCINWVSGARYAPRSSAIESLLSGVRKGKAGTLDGCHIRCIGDNIWVFREFNAVRNVACATDQLWDNRWKMVSQHPTHHDLRIKALGLEGLEQCPDWRATGLPHVMLQSTPAIWFGDSLIAAPHAGFPQNWRAELNELPDTLFAALLSHMADLKKLAEEIVGLTLLEAQELKTILKDEYGIEPAAGGAVMMAGPADGGGAAEEKSEFDVVLKNAGASKINVIKEVRGITGLGLKEAKDLVEAGGKIKEGVDKAEAEDVKAKLEAAGAEVELAMSRIGKKAVELPSGVTASVSGQSIEVKGPKGVRTFKATDDVDLKVEDNTVSVIPRGKSKRARQQWGMSRTMVANLVTGVTEGFKKELEIQGVGYRAQMQGNTLKLNLGLSHDVDYTAPDGVTVTAPKQTEIVVEGIDEQLVGQVAANIRAWRKPEPYKGKGIRYKGEFVFRKEGKKKMHVILLERVAKLGQMGEVVDVKPGYARNYLLPQGKALSASKANIEAFEGRKAQLEAQNLETKKEADKMAETLNGQQFIVIRSASDAGALYGSVTTRDAADAATEAGFTVDRKQVVLSAPIKELGLHDVSVVLHPEVEATIQLNVARSTEEAELQASGKSIQELAAEEEAAADFEIAELFDDIGAAASDDDDLAESISEDADEGTGDQDETAMSDSDGRKTLGL-GGSRPSNVKQSFSHGRTKNVVVETKRKRVVVPKPGGQKPTGPGAGPVGDPSKRPAGITDAEMERRLKAVQAAKAREVEEAAARAAEEKARAEDRERRRAEIEAKEQEDREREESLKAKAEEEERAKQEAVAAAQAAAAPS-EPVAARPTPNKAAPAPAARKTERDREETNKKNR-TDD-RRSGKLTVNQALAGREGGRQRSMAQMKRKQERARQKAMGGTVEREKIIRNVNLPPAIVVSELAARMAEKTGAVVKALMTNGMMVTQNETIDADTAELIIEEFGHKVVRVSDADVEDVIKEIKDDPKDLKPRPPVITIMGHVDHGKTSLLDAIRNAKVVAGEAGGITQHIGAYQVTTDGGQVLSFLDTPGHAAFTSMRSRGAQVTDIVVLVVAADDAVMPQTIEAIAHAKAANVPMIVAINKIDKPAANADKVRTDLLQHEVIVEKMSGEVQDVEVSATTGQGLDQLLEAIALQAELLELKANPDRAAVGAVIEAQLDVGRGPVATVLVQNGTLRQGDIFVVGEQYGKVRALIDDQGNRVKEAGPSVPVEVLGLNGTPEAGDVLNVTDTEAQAREIADYREKAAKDKRAAAGAATTLEQLMANAKADEDVSELPILVKADVQGSAEAIVQAMEKIGNDEVRVRVLHSGVGAITETDVGLAEASGAPIMGFNVRANASARNTANQKGVEIRYYSVIYDLVDDVKAAASGLLSNEIKENFIGYANIKEVFKVTGVGKVAGCLVTEGVARRSAGVRLLRDNVVIHEGTLKTLKRFKDEVPEVQSGQECGMAFENYDDIRPDDVIEIFEREEVTRTLSMAKPKKTPRPKAQTPKGFRDYFGPEVTHRTQMLRKIAAVYDRYGFDALESSGVETVEALGKFLPDVDRPNEGVFAWQEDADADKPGDWLALRYDLTAPLARVYAQHQNDLPKPYRRYAMGPVWRNEKPGPGRFRQFYQCDADTVGASSVAADAEICAMLADCLEEVGIDRGDYVVRVNNRKVLNGVMEVAGLAGDDKEAERGIVLRAIDKLDRLGTDGVRALLGEGRKDESGDFTKGAGLDNTQADVVMGFMQAKRDSGAQTVARLRELVADSKIGTEGVDELETIADLLGAGGYGADRIEIDPSVVRGLGYYTGPVYEAELTFEIKDDKGRPRNFGSVAGGGRYDDLVKRFTGQEVPATGVSIGVDRLLAALDAKGRLDKTAHGPVVVTVMDKDRMADYQKMVADLRSAGIRAEVYLGNPKNFGNQLKYADRRGSPVAVIAGGDEFDKGVVQIKDLILGAKIAENATLEEWQARENQYEVARDDLVTEVAKIINGTSMDDLKQKYLTLIADAADEAVLEDIRLTAVGKKGEVALKMRELGKMTPEERQVAGPALNALKDEINSALAAKKAALGDAALDERLRSEWLDVTLPVRPQRVGSIHPVSQVTEELTAIFAEMGFSVAEGPRIDTDWYNFDALNIPGHHPARAEMDTFYMARAEGDDRPPHVLRTHTSPVQIRTMEKEGAPLRIICPGGVYRADYDQTHTPMFHQVEGLAIDKDISMANLKWTLEEFFSAFFEIDGIKTRFRASHFPFTEPSAEVDIQCSWVDGQLRIGEGDGWMEVLGSGMVHPKVLQAGGIDPDIWQGFAFGMGIDRIAMLKYGIPDLRAFFDSDLRWLRHYGFASLDQPNLHGGLSRMVMAEEAGLDLVEISPNANPPVCKIMDFGKFKYETQKREAEARKKQKIIEIKEVKFRPNTDTNDYDVKMRNVFKFLENGDKVKVTLRFRGREMAHQNLGRELLERVAEDTKEHGRVENFPKMEGRQMVMLIGPLPKMKFTLSWLKDHLDTQASVEEICETLTDLGLEVEGVEDRGAKLRDFTIGYVKSAEKHPDADRLNVCQVETDEGVMQIICGAPNARAGITVVVAKPGVYVPGIDTTIGVGKIRGVESFGMMASEREMELSEEHDGIIELPSGEVGQSFVDWLAANDPAKVDPVIEIAITPNRPDALGVRGIARDLAARGLGTLKARDVEPVTGTFPCPITVTIDEDTLDQCPVFYGRVIKGVKNGPSPAWLQDALRAIGLRPISFLVDVTNFFTYDRNRPLHVFDADKIAGNLRIHRAKGGETLMALDEKEYTFDEGMTLISDANSVESIGGIMGGLSTGCTEETTNVFLEAAYFDTVRTAYTGRALKINSDARYRFERGIDPAWTPHGIEHATRMILDHAGGEASEVVVAGSIPDTSRAYKLDTDRVQSLVGMTIPAETQRETLTALGFEIKGDMAHVPSWRPDVQGSADLVEEVARIASLTKLEGVPLPRLSDGVPRPILSPQQRRITAARRAVAALGYNECVTYSFIDQASAALFGGGTDETRLENPISADMSHMRPALLPGLLQAAARNQARGFADMALFEVGPAFQGGEPGEQHSLVSGLLIGRTGPKDVHGASRPVDVYDVKADAEAALAALGAPAKVQILRGADDGWHPGRHGKICLGPKKVLGIYGEVHPKILAAMDVKGPAMAFTLWPDEVPLPRKSGATRAALQISDLQAVERDFAFVVDAGVEALTLVNAAMGADKTLIDEVRVFDEFIGGSLGEDKKSLAITVRLQPKGETLKDADIEAVGAKVIEKVTKATGGVLRGMHAYRSHTCADLNLSNKGDKVRLSGWVHRVRDHGGILFIDLRDHYGMTQVLCDPDSPVFKEVEKVRSEWCIRIDGEVKARDADLINSKIPTGEIEVFVRDIEVLGSAAELPLMVFGEQEYPEETRLKYRYLDLRREKMQKNMVLRSDVVSSMRQRMWNQGFKEFQTPIITASSPEGARDFLVPSRLHPGKFYALPQAPQQFKQLLMVSGFDKYFQIAPCFRDEDPRADRSPTDFYQLDLEMSFVTQQDVFDTIQPVIGGIFEEFGGGRKVDQTWEQISYKDAALWYGSDKPDLRNPIKMQVVSEHFAGSGFAIFAKLLEQEGTQIRAIPAPTGGSRKFCDRMNAFAQKEGLPGMGYIFWREKTGEEGGMEAAGPLAKNIGPERTEAIRQQLGLDVGDAAFFLGGKPKAFEGVAGRARTVIGDELGLTDKNRFAFAWIVDFPIYEKDEETGKIDFEHNPFSMPQGGMEALEGDPLKVLGYQYDLACNGYELVSGAIRNHKPEIMFKAFEIAGYGEDEVRKRFGGMVNAFQYGAPPHGGCAAGIDRIVMLLADEQNIREVILFPMNQRAEDLMMNAPSDPTSDQLMELGLRVIPQEMTPLSHIRNFSIVAHIDHGKSTLADRLIQSTHTVADRDMKEQMLDSMDIERERGITIKAQTVRINYTAKDGEDYVLNLIDTPGHVDFAYEVSRSMRAVEGSLLVVDSTQGVEAQTLANVYHAIEADHEIVPVLNKIDLPAADCDRVAEQIEDVIGIDATDAIRVSAKTGVGIAETLEAIVTKLPAPKGTRDAPLKAMLVDSWYDSYLGVIVLVRIMDGVLKKGERIRMLSNDSTHHVDRIGVFRPAMTEIDELGPGELGFLTASIKQVRDTRVGDTITHERKGTTERLPGFKPSQPVVFCGLFPVDANDFEDLRDSIEKLALNDASFSFEMETSAALGFGFRCGFLGLLHLEVIRDRIEREYDIDLITTAPSVIYDIHMKDGTVTQLHNPADMPDMTLVDHIEEPRIKATILVPDEYLGDVLKLCQDRRGMQQDLTYAGSRAMAVYDLPLNEVVFDFYDRLKSVTKGYASFDYQMVGYRTDNLVKMSILVNDEPVDALSMMVHRDRAETRGRAMVEKLKDLIPRHMFKIPIQAAIGGKVIARETLSAMRKDVTAKCYGGDATRKKKLLEKQKAGKKKMRQFGKVDIPQEAFISALKMDG--------------------------------------MWAPCPFHQEKSASFHVDDRKGFYYCFGCHAKGDAISFVRETENVGFMEAVEILAREAGMPVPKQDPRAQEKADKRTELADVTELAVRWFRLQLKTGAAEEARAYLERRGLNEAALERWEIGFAPDTWQGLWDALKGKNISDELILGAGLAKPSTKGGKPYDTFRGRIMFPIRDARGRAIAFGGRAMDPNDNAKYLNSPETELFDKGRSLYNVKEARTAAGKGQTLLVAEGYMDVIALQQAGFEAAVAPLGTAITENQLQMLWRIDPEPIITLDGDTAGQRAALRLIDLALPLLEAGKSLRFAMMPEGQDPDDLLKSAGKGAFQKLIEAALPMVQLLWQRETEGRVFDSPERKAALDKALREKIMLIKDPSIRSHYGQEIKDLRWQLFRPK----KSGSTPVA-RGKGKWDTPPYKASPAAKTSVLAAAGDLRATEHLREAVILAAFASCPQIIEEFESGLEGMTCHDPDHRILRDLMLRHGAQDAAVLREKIYSALGPDALENLMSQRHVAITPCIRNPGDTEMARLTVAEELAKSEAARGLDAEIEEAVEDLSGVADEGVTWRLSEAARAADLATRSGQEDKAEYHVGDNGARISKDERSAWDTLLGTIKFSKAKGDTMCADTPDYKSTLNLPKTDFPMRAGLPKREPDWLARWEKIGVYDRLREKEGREPFTLHDGPPYANGHLHIGHALNKTIKDMIVRSHQMMGRDARYIPGWDCHGLPIEWKIEEQYRKKGRDKDEVPINEFRAECRNFAAGWVDVQREEFKRLGVQGNWADPYLTMDFHAERVIAEEFMKFLMNGTLYQGSKPVMWSPVEQTALAEAEVEYHDKDSFTIWVKFEVVEARVLASDLKGASVVIWTTTPWTMPSNKAVVYGAGISYGLYEVTSTPDECWAAAGDRFLLADNLAADVFGRARLEDDMWTRVRDLTADELAGLALKHPLAGAEGGNGEWDDLRDFRAAEFVTDTEGTGFVHCAPSHGLEEYDLYRDLGMLEQVITYNVNPDGRYRDDLPFFGGKAILKPNGKEGNANAAVIEKLVEVGGLLARGKIKHSYPHSWRSKAPVIYRNTPQWFAAIDKVVGDGLDEHGKTIRERALTEIDNVNWTPKSGRNRLHAMMEARPDWVLSRQRAWGVPLTCFTRKGVLPTDPDFLLRHPDVNARIVEAFEVEGADAWYEDGAKERFLGGIVNPDDYDQVTDILDVWFDSGSTHAFTLRDREDGTEDGIADVYMEGTDQHRGWFHSSLLQSVGTTGHAPYRNVVTHGFTLDAKGMKMSKSIGNTIVPEKIVQQYGADILRLWVAQTDYTQDQRIGDEILKGVADSYRRLRNTMRYMLGSLSDFSEADRVDPADMPELERLVLHRLAELDKVVREGYARFDFQGVFQAVFTFATVDLSAFYFDIRKDALYCDGDTERRRAARTVLDILFHRLTTWLAPVLVFTMEEVWLERYPGDDSSVHLTDMPETPSEWLNPELAAKWAKVRAARRVVTSALEVQRVEKVIGASLEAAPVVYIEDAEQRAALESVSFEDVSITSDITVSGDAAPADAFRMPEVEGVAVSFALAEGEKCARCWKVLPDVGTHAHPGVCGRCDAAVSMAQSQNIRIRLKAFDYRVLDTSTQEIVNTAKRTGASVRGPIPLPNKIEKFTVLRGPHVDKKSRDQFEIRTHKRLLDIVDPTPQTVDALMKLDLAAGVDVEIKLQSMADEINTLEDLAKVVTGAVQG---TPETMEINREPVRDELGRSYATGKRKDAVARVWIKPGSGKVTVNGKPQNDYFARPVLQMILAQPFGITNTEGQFDVFATVKGGGLSGQAGAVKHGISKALQLYEPSLRGALKAAGFLTRDSRVVERKKYGKAKARKSFQFSKRMPTIQQLIRKPRQPKRKTSKSMHLEQCPQKRGVCTRVYTTTPKKPNSAMRKVAKVRLTNGFEVISYIPGESHNLQEHSVVLIRGGRVKDLPGVRYHILRGVLDTQGVKDRKQRRSKYGAKRPKMARDARKTKRKVSKNIAAGVAHVNSSFNNTKILISDVQGNAIAWSSSGTMGFKGSRKSTPYAAQMAAEDAGRKAQEHGVKTLEVEVQGPGSGRESALRALAAAGFNITSIRDVTPMAHNGCRPPKRRRVQA-DRRGLLIILSSPSGAGKSTLARKLRDWDTSLQFSVSSTTRPPREGEVEGLDYYFTTQEDFRNSVNQGDMLEHARVFGNYYGSPRAPVQEAIEAGRDVLFDVDWQGAQQISNSALKDYVLSIFILPPSIAELKRRLVSRGQDSPDTIALRMAASWDEISHWDGYDYVLVNDDLAETEARLKAIITAERFRRSQQPRLVDHVRKLQTEFEDDTMAQSFLGQKRLRKYYGKIREVLDMPNLIEVQKSSYDLFLNSGDAETPTDGDGIQGVFQSVFPIKDFNETSILEYVKYELEKPKYDVEECQQRDMTYSAPLKVTLRLIVFEIDEDTGAKSVKDIKEQDVFMGDMPLMTPNGTFIVNGTERVIVSQMHRSPGVFFDHDKGKTHSSGKLLFACRIIPYRGSWLDFEFDAKDIVFARIDRRRKLPVTTLLYALGLDQEGIMDAYYNTVTYTLKAKKGWVAPFFPDRVRGTRPTYDVVDAKSGEVLFEAGKKVTPRAVKKLIDEGEVKELLLPFDHIQGKFVAKDIINEETGAIYVEAGDELTLEYDKDGDLIGGTAKELVDAGITEIPLLDIDNVNVGPYMRNTMANDKNMNRDTALMDIYRVMRPGEPPTVEAASALFDTLFFDSERYDLSAVGRVKMNMRLDLDAEDTVRTLRREDIVACIKALVDLRDGRGDIDDIDHLGNRRVRSVGELMENQYRVGLLRMERAIKERMSSVEIDTVMPQDLINAKPAAAAVREFFGSSQLSQFMDQTNPLSEVTHKRRLSALGPGGLTRERAGFEVRDVHPTHYGRMCPIETPEGPNIGLINSLATFARVNKYGFIETPYRVVKDSTVTDEVHYMSATEEMRHTVAQANASLDVNGKFTNEMVNTRRSGDYTLAPTESVDLIDVSPKQLVSVAASLIPFLENDDANRALMGSNMQRQAVPLLQAEAPLVGTGIEEVVARDSGAAYMARRAGVIDQVDASRIVIRATEDLELGDAGVDIYRMRKFQRSNQNTCINQRPLVKVGETVTKGQVIADGPSTDMGELALGKNVVVAFMPWNGYNYEDSILISERVSRDDVFTSIHIEEFEVAARDTKLGPEEITRDIPNVGEEALRNLDEAGIVYIGADVEPGDILVGKITPKGESPMTPEEKLLRAIFGEKASDVRDTSLRVKPGDFGTVVEVRVFNRHGVEKDERALQIEREEVERLARDRDDEMAILDRNIFARLRDMILGKVAVKGPKGVSPNSQITEDMLNVLTRGQWWQLALEDEDDAKIVEALNEQYEIQKRTLDARFDDKVEKVRRGDDLPPGVMKMVKVFVAVKRKLQPGDKMAGRHGNKGVISKVVPMEDMPFLADGTPVDFCLNPLGVPSRMNVGQILETHMGWAARGLGLNVDEALQEYKRSGDLTPVREAMSIAYGEDVYSEGLEGMDEDTLLEAAGNVTRGVPIATPVFDGAKEADVNDALVRAGFNSSGQSVLFDGRTGEQFARPVTVGVKYLLKLHHLVDDKIHARSTGPYSLVTQQPLGGKAQFGGQRFGEMEVWALEAYGAAYTLQEMLTVKSDDVAGRTKVYESIVKGEDNFEAGIPESFNVLVKEVRGLGLNMELLDAEEDEMPLYEHVMIARQDLSNTQAEGLIEHFGTVLADNDGKLVDSEYWGVKTMAYKINKNRKGHYAFLRSDAPAPAVQEMERLMRLHDDVMRVLTIKVDEHKELPSVQMQKRDERPDRRERRMTLEIAVDKLTEKEAKQELSRLAELLGQANTDYHTKDNPVLSDAEYDALKRRNAAIEERFASLKRGDSPSDQVGAPLAEGFSKITHKVAMLSLGNAFDPEDVAEFDGRIRKYLGLDAEAPLAYTAEPKIDGLSLSLRYEGGTLIHAVTRGDGATGENVTANARTIDDIPEQIEDAPDVLEVRGEVYMSHADFDALNVRQEAAGGKPFANPRNAAAGSLRQLDSSITKSRPLKFFAYAWGDLSAPLAATQKQAIARLAELGFVTNPLTALCNSPEEMLAHYRDIEEKRATLGYDIDGVVYKVDDLGLQDRLGFRSTTPRWAIAHKFPAELAWTWLTGIDIQVGRTGALSPVARLQPVTVGGVVVSNATLHNEDYIKGLDSKGAVIRDGKDIRVGDWVQVYRAGDVIPKVADVDLSKRDAAAEAFAFPDKCPECGSDAIREPGDAVRRCTGGLICPAQAVEKLKHFVSRAAFDIDGMGAKQVEQFYADGWIAEPADIFTLKERYGSGMQQLKNREGWGEKSAANLFDAIEDKQRIPLDRLIFALGIRHVGEAASNLIALHFGDWPTFEAAMAEAAVADSPAWQDLIGIDGVGSVMAGSLVSAFAQERERASIDRLVAALDVQPVSRPDTSGSPVAGKTVVFTGTLTQMTRAEAKARAERLGAKVSGSVSAKTDILIAGPGAGSKAKKAAELGVETMDEDGWLRLISDLMIHKNWAELIKPQQLDVKPGNDPARQATVMAEPLERGFGLTMGNALRRVLMSSLQGAAITSVQIDNVLHEFSSVAGVREDVTDIILNLKGVSIRMEVEGPKRLSISAKGPGVVTAGDISESAGIEILNRDHVICHLDDGADVYMELTVNTGKGYVSADKNKPEDAPIGLIPIDAIYSPVKKVSYDVQPTREGQVLDYDKLTMKVETDGSITPDDAVAFAARILQDQLGIFVNFDEPESASRADEDDGLEFNPLLLKKVDELELSVRSANCLKNDNIVYIGDLIQKTEAEMLRTPNFGRKSLNEIKEVLSGMGLHLGMDVEDWPPDNIEDLAKKFEDNFMARDDNRGGNRRNNRDETPEFQDRLVAINRVSKTVKGGKRFGFAALVVVGDQKGRVGFGKGKAKEVPEAIRKATEQAKRQMIRVQLREGRTLHHDMEGRHGAGKVVMRTAPEGTGIIAGGPMRAVFEMLGVKDVVSKSIGSQNPYNMIRATMDGLRKESSPRSVAQRRGKKVADILPKTDAQVSEQAPVVEEAMNDPIADMLTRIRNSQMRGKSTVMTPASKLRAWVLDVLADEGYIRGYEKMTGADGHPAIEISLKYYEGEPVIRELKRVSKPGRRVYMGANDIPQVRQGLGVSIVSTPQGVMSDANARSANVGGEVLCTVFMHDIRAIRENPDAFDAALARRGDAPVSSEILKLDEARRAKIGAAETAQAEQNKASKLVGAAKAKGDDAEFERLRALVGQKKEEVATMQAEAKALDAQLTDVLARLPNNPADDVPDGKDEADNVEVNRWGDIPSFDFTPKEHFEIAGVAASMDFETAAKISGSRFVLLTGAVARIHRALAQFMLDTHVDENGLTEVNPPVLVGDDAMYGTDKLPKFGEDSYQTSEGMWLVPTSEVPLTYTVAGDVVEQSTLPRRYTAHTLCFRSEAGSAGRDTSGMLRQHQFEKVEMVSVTLPEESDNEQKRMLRCAEDILERLGIPYRTVILCTGDMGFGARRTFDIEAWVPGQDTYREISSVSTTGDFQARRMNARFKPADGGKPQFVHTLNGSGLAVGRCLIAVLENGQQADGSVELPQVLAPYLGGKTMLTAEGRLAMAITASMVKELRDSTGAGMMDAKKALTENDGDMEAAVDWLRTKGLAKAAKKSGRTAAEGLVAVKVDGGHGVAVEVNSETDFVGKNADFQKMVAGIADVALGANDIDALKAADMGGKSVEQTVTDAVAVIGENMSVRRMAAIDGDTVVSYVHNAAAPGMGNIGVLVAMTGGDEAFGKQVAMHIAATNPASLSEEDLDAAVVEKEKQVQMDIARESGKPEAVIEKMIVGRMKKFMAEITLINQQFVVNPDVTVGDAAKEAGATITGFVRLEVGEGIEVVKEDFAAEVAKVGQSMNQELTNNPFNPVAPMKTFDEIKVSLASPERILSWSFGEIKKPETINYRTFKPERDGLFCARIFGPIKDYECLCGKYKRMKYRGVVCEKCGVEVTLQKVRRERMGHIELASPVAHIWFLKSLPSRIGLMLDMTLRDLERVLYFENYVVIEPGLTDLTYGQMMTEEEYMDAQDAYGMDAFTANIGAEAIREMLAAIDLEAEADQLREELKEATGELKPKKIIKRLKVVESFLESGNRPEWMVLTVIPVIPPELRPLVPLDGGRFATSDLNDLYRRVINRNNRLKRLIELRAPDIIVRNEKRMLQESVDALFDNGRRGRVITGANKRPLKSLSDMLKGKQGRFRQNLLGKRVDFSGRSVIVTGPELKLHQCGLPKKMALELFKPFIYSRLEAKGLSSTVKQAKKLVEKERPEVWDILDEVIREHPVMLNRAPTLHRLGIQAFEPVLIEGKAIQLHPLVCSAFNADFDGDQMAVHVPLSLEAQLEARVLMMSTNNVLSPANGAPIIVPSQDMILGLYYTTLEREGMKGEGKVFGTVDEVQHALDAGEVHLHTKIKARIKQIDNEGNEVLVRFDTTPGRVRLGAMLPLNAKAPFDLVNRLLRKKEVQQVIDTVYRYCGQKESVIFCDQIMTMGFREAFKAGISFGKDDMLIPDSKWPLVEETREQVKDFEQQYMDGLITQGEKYNKVVDAWSKCNDKVTDAMMGSISAERRDENGAEMEPNSVYMMAHSGARGSVTQMKQLGGMRGLMAKPNGDIIETPIISNFKEGLTVLEYFNSTHGARKGLSDTALKTANSGYLTRRLVDVAQDCIVRMHDCGTDMAITAEAAVNDGEVVSSLAERLLGRVIADDITVPGTDEVIATAGTIIDERLADLIDEAAVQSTRIRSPLTCEAEEGVCAMCYGRDLARGTLVNQGEAVGIIAAQSIGEPGTQLTMRTFHIGGVAQGGQQSFLEASQEGKIVFENAMTLENADGGIMVMGRNMKMLIVGEDGAERANHKIGYGTTLFVKDGDTVARGDKLFEWDPYTLPIIAEKSGMTKFVDLVSGIAVKDETDDATGMTQKIVIDWRAAPKGNELKPEIILVGEDGEPLRNDAGNPVTYPMSVDAILSVEDQTEIKAGDIIARIPREGAKTKDITGGLPRVAELFEARRPKDHAIIAEIDGYVRFGKDYKNKRRIAIESSDDPDHKVEYMVPKGKHIPVAEGDFVQKGDYIMDGNPAPHDILAIMGVEALADYMIDEVQDVYRLQGVKINDKHIEVIVRQMLQKWEIQESGDTTLLKGEHVDKQEFDLANEKAASKGGRLAKGEPILLGITKASLQTRSFISAASFQETTRVLTEASVQGKRDKLVGLKENVIVGRLIPAGTGGATMQMRKVASDRDNVVIEARREEAEAAAALAAPVP--DDVVGGDVFTTPSFDEESRDMSRRHAAEKREVLPDAKYGDLVLTKFMNNLMIDGKKSVAERIVYNAMTRVEDKIKRAPIEVFHEALENIQPSVEVRSRRVGGATYQVPVEVRPERRQALAIRWLIKAARSRNENTMEERLAGELMDAVQSRGTAVKKREDTHKMADANKAFSHYRWMPYAQTDSSTIPPVMANPAPDVRARPKLEGGKTFKLKTEFEPAGDQPTAIKELTAGVADGDRNQVLLGATGTGKTFTMAKVIEETQRPAIILAPNKTLAAQLYGEFKGFFPDNAVEYFVSYYDYYQPEAYVARSDTFIEKESQINEQIDRMRHSATRALLERDDVIIVASVSCIYGIGSVETYGAMTQDLKAGSIYDQRQVIADLVAQQYRRNDAAFQRGSFRVRGDSLEIFPAHLDDRAWRLSFFGEELESITEFDPLTGEKTDTFDQIRVYANSHYVTPKPTMSQAIIGIKKELRMRLDQLVADGKLLEAQRLEQRTNFDLEMLEATGVCNGIENYSRYLTGRAPGEPPPTLFEFIPDNAIVFADESHVSVPQIGGMYKGDYRRKFTLAEHGFRLPSCMDNRPLKFEEWDAMRPQSIFVSATPADWEIEQTGGVFTEQVIRPTGLLDPKIEIRPVEMQVDDLLDEVRRVAADGYRTLCTTLTKRMAEDLTEYMHEQGIRVRYMHSDIDTIERIEILRDLRLGAFDVLIGINLLREGLDIPECGLVAILDADKEGFLRSETSLIQTIGRAARNSEGRVIMYADRVTGSMERAMGETERRRAKQMAYNEEHGITPETVKKNVEDILAGLYKGDVDMNRVTTQIDSKMAGSNMQAVLDGLRTDMRKAAENLEFEEAARLRDEVKRLEAVDLAIADDPMARQYAVDKAVDNAKKASGRSTMGRGGMRGGVKRKKGRMAAKPFFRRRKVCPFSGDNAPAIDYKDTRLLQRYISERGKIVPSRITAVSAKKQRELARAIKRARFLALLPYAVKMPKRILTGTVTSDANAQTVTVSVERRFTHPVLKKTIRKSKKYRAHDENNTFKVGDSVRIIECPPKSKTKRWEVITAEAMANTAQSKKRARQNEKRFQINKARRSRIRTYLRKVEEAIESGVKDDAVAALKAAQPELMRGVTKGVFHKNTASRKMSRLSARVKALSMSRSVWKGPFVDSYVLKKAEATRESGRNEVIKIWSRRSTILPQFVGLTFGVYNGRKHIPVNVSEEMIGQKFGEYSPTRTYYGHAADKKAKRKMARIAGVNIPTAKRVPIALTYITGIGNSSAEAICEAVGIDFSRRINELSDAEILKIREHIDENYTVEGDLRRDTQMNIKRLMDLGCYRGLRHRRNLPVRGQRTHTNARTRKGPAKAIAGKKKMSMKIRLARGGSKKRPFYRIVAADSRMPRDGRFIEKLGTYNPLLPKDSEDRVKMDVEKVKEWMAKGAQPTDRVRRMLEAAGELPKTERNNPNKGTPGKKAQERVQEKADKAAAAAEAAAAPAEEAPAEEMSITAEEKARVMKEFGTKEGDTGSPEVQVAVLSSRIATLTEHFKTHKKDNHGRRGLLKMVATRRKLLDYVKTKDEARYQDLIKRLGLRRMKTLNDIRSTFLSYFERNGHEVVPSSPLVPRNDPTLMFTAAGMVQFKNLFTGVETRDYSRATTAQKCVRAGGKHNDLDNVGYTARHHTFFEMLGNFSFGDYFKEDAIPLAWDLLTKGFGIDESRLLVTVYHTDEEAVQIWKKHAGLSDDRIIRIATDDNFWSAGPTGPCGPCTEIFYDHGDHIWGGPPGSPEEDGDRFVEIWNLVFMQYEQLEDGTRRDLPNQSIDTGMGIERVAALLQGTNDNYATDLMRSLIEASADASNTDPDGPGKTHHRVIADHLRSTSFLIADGVMPSNDGRGYVLRRIMRRAMRHAHLLGVQDPLMHKLVPALVGQMGAAYPELGQAQSLITETLQLEETRFKQTLERGLKLLEDEVSGLDKGAHLSGQAAFKLYDTFGFPLDLTQDALREKGMGVDTDGFDAAMAEQKAKARAAWAGSGEAADATVWFDVADKHGTTDFLGYDTESAEGQIVAIIKDGAVADMAKAGDTVQIALNQTPFYAESGGQVGDTGTIRTDGALIRVTDTRKAADVFIHFGTVEEGEVTPSKAAVLTVDHARRTSIRANHSATHLLHEALRGALGDHVAQRGSLNAADRLRFDFSHAKALSAQELSQVEQEVNDYIRQNTAVETRIMTPDDARALGAQALFGEKYGDEVRVVSMGREDGSGKGTDKSTYSLELCGGTHVRQTGDIGAFVLLGDSASSAGVRRIEALTGSEALAWLREQQMALAGIAEAMKTSSADVPERVKALMEERKSLNNEVAQLRRELAMVGGGGATAPEAVDVGGIKFMAQVLQGVTGKDLPALVDEFKASLGSGAVLLIADTSGKAAVAGGVTADLTDKVSAVDIVKAAVVELGGKGGGGRPDMAQGGGASAENAEGAIAAATAILKGMGFKMGIVGLPNVGKSTLFNALTRTAAAQAANFPFCTIEPNVGEVAVPDSRLDKLAAIAQSKSIIPTRMTFVDIAGLVKGASKGEGLGNQFLANIREVDAIAHVLRCFEDGDVTHVEGRVDPVADAETIDTELMLADLESIEKRRAGLVRKIKGNDKEAVQQDRLLAMAQDAIEAGKPARVVEVDEDDARAWRMLQLLTTKPVLYVCNVGESEASEGNAFSAAVAEMAASQGNAHVIISAQIEEEISQLEAEEAEMFLDEMGLKEAGLDRLIRAGYELLHLETYFTVGPKEARAWTIRTGTSAPKAAGVIHGDFEKGFIRAETIAYEDFITLGGEGPAKEAGKMRAEGKGYTVKDGDVLHFLFNTMGWKSLDDMELRGKRVLVRVDINVPVEDGRVTDATRIERIVPTVTDILAKGGKPMLIAHFGRPKGKVVPEMSLRQVLPALENALGVSVAFVETLEAAENLSDDATAAQVILLENIRFAPGEEANDPAFAARLANLGDVYCNDAFSAAHRAHASTEALARELPACAGRLMQAELHALEASLSQPERPVGAVVGGAKVSTKIALLENLVTRLDTLVIGGGMANTFWAALGADMGGSLKEDDYLQTAKDILAKAEKSGCRVILPEDGLVARTFAANAPYEVVKLTATTKLDADQMVLDAGPESIKTLTVAFEALKTLIWNGPLGAFEIAPFDTATVAAAQIAARRTKEDALISVAGGGDTVAALHKAGVADDFSYISTAGGAFLEWMEGKTLPGVAALSGMALPEFSMRQLLEAGVHFGHQTQRWNPRMGPYIYGARNGIHIMDLTQTVPMLDEALKLIRDTVAKGGSILFVGTKRQAAQPIADAAEKCAQYYMNHRWLGGTLTNWQTVSKSIQRLKHIDEQSEQGFSGLTKKERLGMERDQGKLEASLGGIREMGGRPDLIFVIDVKKEALAVAEANKLGIPVVAVVDTNCSPDGIDYIIPGNDDAARAIGLYCDLAARAALDGMSAQLGAAGVDLGAMEQAPAEEALAEESNASEETVSNDAMAKDAESMTKRTAAKHKLDRRMGENIWGRPKSPVNRREYGPGQHGQRRKGKISDFGIQLRAKQKLKGYYGDLTEKQFRRIYGEAERVKGDTGENLIGLLERRLDAVVYRAKFVATVFAARQFVNHGHVRVNGKKVNIPSYRVKEGDVIEVRDRSKQMVALLEATQLAERDVPDYIDADHSKMTATFVRTPSLGDVPYPVMMEPNLVVEFYAKNMGNKVNPIGMRLQVNRTWDSRWYADTKDYGDLLLEDLAIRAFIKKECAQAGIARVIIERPHKKCRVTIHTARPGVIIGKKGADIEGLRQKIAKITKSDLHLNIVEVRKPELDAVLVGESIAQQLERRVSFRRAMKRAVQNAMRMGALGIRCNLAGRLGGAEIARTEWYREGRVPLHTLRADIDYAHVEAATAYGIIGIKTWIFKGEIMEHDPAARDRKAQEMQDGPAPRGAGGRRMARTRKGRDISGWLIIDKPAGPTSTAVVNKVRWAMDAKKAGHAGTLDPTATGVLAVALGEATKTVPFITDALKAYEFTVRLGVRTNTDDAEGEVVQTSDLRPDDEAIKEALAGFIGDIEQVPPQFSAVKIDGERAYKRARDGEEMELAARPLFVESLLLIDRSDEDHVTLEMVCGKGGYVRSIARDLGEALGCHGHVRELRRTWSGPFDAADGLTLDQIDEMAKSPELDAHLLPLEQGLCDLPEVKATVEGAVRLRNGNPGMVIGQDVEYGDECWASLDGHAVAVGRFKAGELHPSRVFNT--MFLLGLTGSIGMGKSTTAEMFVQEGCALWDADAAVHRLYSRGGAAVPLFAADFPSSIVEGAVSREKLKEIIAADPTALKKIEKIVHPLVGEDRAAFIASANADIVVLDIPLLFETGGENRVDAVACVTIDDDTQKQRVMARGTMTEAQFEAIRAKQLPAGEKTARADYIIVTDTVAHAARQVQLIVQDIREKRLNA---LEIVIEDSRWDDLDLTALADVALTETLSHLSVEHALCEVSLLACDDARIAVLNADFRGKPSATNVLSWPAEERAASQAGERPEPVQPGPDGMIELGDIAISYDTCAAEAAAAGKPPSEHVTHLISHATLHLLGFDHENAQDAALMEGLEIEILGKLGLDNPYRES--MARYIFITGGVVSSLGKGLASAALGALLQARGFSVRLRKLDPYLNVDPGTMSPFEHGEVFVTDDGAETDLDLGHYERFTGVPARMTDSVSSGRIYSTVLEKERRGDYLGKTIQVVPHVTNEIKDFLKVGDNEVDFMLCEIGGTVGDIEGLPFFEAIRQFSHDKPRGQCIFMHLTLLPYLAASGELKTKPTQHSVKELQSIGIAPDILVCRSEHPIPEKEREKIALFCNVRKEAVVAAYDLKSIYEAPLAYHAQGLDQAVLDAFDISPAPRPDLSVWHDVFDRVHNPEGEVRVAIVGKYTQLEDAYKSIAEALTHGGMANRVKVKVEWVDAEVFDSEEVATHLEGFHAILVPGGFGERGTEGKIKAAQYAREHKVPYLGICLGMQMAVIEAARNVAGVKTAGSEEFDHEAGKKRFEPVVYHLKEWVQGNHKVERKVGDDKGGTMRLGAYDATLTPGSRVAEVYGTTTIDERHRHRYEVDMAYKQQLEKVGLSFSGMSPDGKLPEIVEWSDHPWFIGVQFHPELKSKPFAPHPLFKDFVRAAKEMSRLVMRPPLTILGLESSCDDTAAAVVHLPVSGPPAILSSIVADQTDLHAAFGGVVPEIAARAHAEKLDLCVEQALTQANMTLGAIDAIAVTAGPGLIGGVVSGVMCAKGLSAATGKPLYGVNHLAGHALTPRLTDGVEYPYLMLLVSGGHCQFLLVKDADDFTRLGGTIDDAPGEAFDKVARLIGLGQPGGPAIESAALNGDNRRFAFPRPLLDRAGCDMSFSGLKTAVLRTRDQLVKENGGLTVQDQCDLAAGFQAAVVDVLAAKTRNAFASYPDAAQ--RSLCVAGGVAANTAIRSALETVASDQKASFVAPPLALCTDNAAMIAYAAAEQSTLRSEDDLKLSARPRWPLDTAQPAMLGSGKKGPKA------------------------------------------------------------------------------------------------------------------------------------

>'Roseobacter-pontiDSM-106830'

MNAKELHDKTPDQLREELATLKKEQFNLRFQQATGQLENPAQLRIARRNAARVKTVLNQKAASAAASEMATADLLSMNNKKSADKQKALDSALAQIERQFGKGSIMRLGAEGAVQDIKASSTGSLGLDIALGIGGLPMGRIVEIYGPESSGKTTLTLHCVAEQQKSGGVCAFVDAEHALDPTYAKKLGVDLDELLISQPDTGEQALEITDTLVRSGAVNMVVVDSVAALTPKSELEGDMGDSSVGVQARLMSQAMRKLTGSISRSNCMVIFINQIRMKIGVMFGSPETTTGGNALKFYSSVRLDIRRIGALKDRDEVVGNHTRVKVVKNKVAAPFKQVEFDIMYGEGISKMGELLDLGVATGVVDKSGSWFSYGDERIGQGRENAKSFLKENTQMALEIEDKIRAAHGLDFDAHEKTDEDILEAMERYTPAEIEARWQDVWNRDEVFKAVRSADKPKYYVLEMFPYPSGRIHMGHVRNYTMGDVIARYKLATGHNILHPMGWDAFGMPAENAAMAIGGHPKDWTYGNIADMRDQMKPLGLSIDWSREFATCDPEYYGQQQALFLDFLNAGLVYRKNAVVNWDPVDMTVLANEQVEQGRGWRSGALVERRELTQWFFKISDYSEELLEALDTLEAWPAKVRLMQENWIGRSRGLQFAFSTVDAPDGHDRIEVYTTRPDTLLGASFVGISPDHPLAKLLERDDPAVAAFCAECRKGGTTEEAIETAEKLGYDTGIRVRHPFDTAHELPVYIANFILMDYGTGAIFGCPAHDERDFEFATKYGLPIISTYLPSEDAVEELTEAYVPPKTETVYYNRGFAGEAHQTGLEAIDAAIDFCEASGVGQGVTKYRLRDWGLSRQRYWGAPIPVVHCETCGVVPEKKENLPVELPYDVDFGTPGNPLDRHPTWRDTPCPSCGRPGKRETDTMDTFVDSSWYFARFTAPDAKTPTSMEDAKYWMNVDQYIGGIEHAILHLLYSRFFARAMAITGHLPETAIEPFDALFTQGMVTHEIYQTHGDNGRPVYHLPEDVTDGKL-SDGTEVEIIPSAKMSKSKKNVVDPVNIISAYGADTARWFVLSDSPPERDVEWTASGAEAAFKHLSRVWNLSERIAGMEKEDQGDGNDDLIRAMHVTIHDVTMGIESFGFNAAIAKLYAFTATLQKSKAGYAAQHAAIRTLAQLMSPMTPHLAEDIWARQGGEGLITTASWPVADDAMLVSDTITLPIQVNGKRRAEITVPADMSKEDVEKTALADQAVIRTLNGATPKKIIVVPGRIVNVVVMAITSANQLELLQTAEAVAREKMIDPGLVVEAMEESLARAAKSRYGAEMDIRVHIDRKTGRATFTRVRTVVEEEELENYQAEFTVEQAKQYMENPEVGQEFIEEVPPVEMGRIAAQSAKQVILQKVREAERDRQFEEFKDRAGTIINGLVKREEYGNVIVDVGAGEAILRRNEKIGRESYRPNDRIRVYIKDVRREQRGPQIFLSRTAPEFMAELFKMEVPEIYDGIIEIKAVARDPGSRAKIAVISYDNSIDPVGACVGMRGSRVQAVVNELQGEKIDIIPWNEDQPTFLVNALQPAEVSKVVLDEEAGKIEVVVPEEQLSLAIGRRGQNVRLASQLTGLDIDIMTEEQESARRQAEFELRTKLFVDNLDLDEFFAQLLVSEGFTNLEEVAYVELDELLVIDGVDEDTANELQARARDVLEARNKAALDNARSLGVEDSLVEFEGLTPQMIEALAKDDVKTLEDFATCADWELAGGWTTENGERVKDDGVLEPFDVSLEEAQDLIMTARVMLGWVDPADLEAEAEAE-AE-GEE-TEETGVMAHKKAGGSSRNGRDSAGRRLGVKLYGGQAAIPGNIIVRQRGTKFWPGAGVGMGKDHTIFATVDGAVTFHKGLKNRTFISVLPAAEAAEMAKRWYSVSVLSNFEKKIAEQIRTSVAEQALEDQIDEVLVPTEEVIEVRRGKKVTTERRFMPGYVLVHMEMSDQGYHLINSINRVTGFLGPQGRPMPMRDAEVNAILNRVQEGEDAPRTLIHFEVGEKVKVADGPFEDFDGMIEEVDDDNQRLKVSVSIFGRETPVELEFTQVNKQTMSFTLAIVGRPNVGKSTLFNRLVGKRLALVDDQPGVTRDLREGAARLADLRFTVIDTAGLEEVTDDSLQGRMRRLTERAVDMADICLFMIDARVGITPSDLVFADILRKRSAHVILAANKAEGAAADAGVIEAYSLGLGEPIRMSAEHGEGLNDLYTHLMPLADQFAERAAEDAPDTDVTLDEDEGDMDPIPMPTAKKPLQVAVVGRPNSGKSTLVNQILGEDRLLTGPEAGITRDAISLRIDWAGAVPMRIFDTAGMRKKAKVQEKLEKLSVGDGLRAVKFAEVVVVLLDAAIPFEQQDLRIADLAEREGRAVVVAVNKWDIEEDRQGKLKELKESFERLLPQLRGAPLITVSAKTGRGLDRLQAAIMRAYEVWNRRVTTAQLNRWLAGMVEAHPPPAPQGKRIKLRYMTQAKTRPPGFVVMCSHPDKVPESYSRYLVNGLRLDFDMPGAPIRLWMRGQNDANPFKGRKKAPPSKLRKHTDGRRRDMRVIFMGTPEFSVPVLEALVAAGHEIACVYCQPPRPAGRGKKERPVPVEARARDMGLEVRHPVSLKSEEAQTDFAALNADVAVVVAYGLILPQAVLDAPAQGCLNIHASLLPRWRGAAPIHRAIMAGDAETGVCIMQMEAGLDTGPVLLREATPIGAEETTGALHDRLSAMGAALITRALEHLPTLVPQPQPETGVSYAAKIDKAEARIDWTRPAEETDRRIRGLSPFPGAWTTQGETRIKLLASKPAEGGGAPGTVLHDPLRVACGEGAVELMRLQRAGGSAQNADEFLRGRLLAPGTVL---MTDSTEYQVLARKYRPETFADLVGQDAMVRTLKNAFEADRIAQAFVMTGIRGTGKTTTARIIAKGMNCIGPDGEGGPTTEPCGTCEHCTAIMEGRHVDVLEMDAASNTGVANIREIIDSVHYRAASARYKVYIIDEVHMLSTGAFNALLKTLEEPPAHVKFIFATTEIRKVPVTVLSRCQRFDLRRIEPEVMIALMSRIAKAEGAQITDDALALITRAAEGSARDATSLLDQAISHGAGETTADQVRAMLGLADRGRVLDLFDMVLRGDAAAALSELSGQYAEGADPMAVLRDLAEITHWVSVVKITPEAAEDPTVSPDERARGLAMAEKLPMRVLTRLWQMLLKALDEVATAPNAMMAAEMAVIRLTHVADLPAPEDLLRRLSGTTPPPGP--GA-SAGTAASSAPVRADAPAGSFSTAPRASGGPATALAADADAALARFPTFEHVVELIRANKDGKLLVDVETDLRLVTYQPGRIEFQPTDRAPRDLAQRLGQRLQNWTGNRWAVTVVSEGGAKTIDEIRNAARYAMEEQIRQHPLMQAVLAQFPKAEIGTVRTPGDIAAAARQEALPEVEDEWDPFEDSMPKMKTKSSAKKRFKVTATGKVLAGQAGKRHGMIKRTRKFIRDARGTTTLSAPDAKIVKGFMPYDRMNLFADIRDLVIETLTALQAEGALPDGLAMDNVTVEPPRDAAHGDMATNAAMVLAKPAGMKPRDIAGALAEKLLADDRITSADVAGPGFLNLRLAPSVWQSVVKTVLETGPDFGRGSLGQGTRVNVEYVSANPTGPLHVGHTRGAVFGDALASLLDFAGFDVTREYYINDGGAQVDVLARSVYNRYLEAHDLSVDWPEGTYPGDYLIEVGEKLKELKGDAFVDQPEEVWLEDVRNFATDAMMDLIRADLKALGVEMDVFYSEKSLYGTGRIEAAIDDLRGKGLIYEGVLEPPKGKKPEDWEPREQTLFKSTEHGDDVDRPVMKSDGSWTYFAPDIAYHYDKVSRGFDALIDVFGADHGGYVKRMKAAVSALSDGKVSLDIKLTQLVKLFKNGEPFKMSKRAGTFVTLRDVVDQVGPDVTRFVMLTRKNDAMLDFDFDRVLEQSRENPVFYVQYAHARVASVMRKAGEAGVDVSDATLAAADLSLLSHEAELAMAGKLAEWPRMVETAARSNEPHRIAFYLYDLAGSFHALWNRGNDETSLRFVQD-DPNVTQSKIALARAVSVVIAAGLGILGVTPAQEMRMSAIDTLPPLREVIRTHDLQARKSMGQNFLLDLNLTAKIARQAGDLSTCDVLEVGPGPGGLTRGLLAEGARHVLAIEKDARCLPPLAEIADAYPGRLTVIEGDALKTDPLASLRPPVRVAANLPYNVGTELLVRWLTPPDWPPFWESLTLMFQREVAERIVAQPGGKAWGRLAILAQWRADARIVMQLPPEAFTPPPKVSSSVVHLQALPEPRYPANAAVLSRVVAAAFNQRRKMLRAALKGTAPDIEDRLIAAGIKPTDRAEQIPLEGFCALARAIESPMDLIAEIEAEQIAALGKEIPDFRAGDTVRVGFKVTEGTRTRVQNYEGVCIARNNGKGIAGSFTVRKISFGEGVERVFPLHSTNIDNITVVRRGRVRRAKLYYLRSRRGKSARITENSNYKPRSGAEAMANSKRQLFIKRRLRVRNKLRRTNAGRMRLSVHRSNKNISVQLIDDVAGTTVASASSLEKDLGVVGKNNVEAATKVGAAIAERAKKAGVSEAYFDRGGFLFHGRVKAVADAAREGGLKIMFAVLKTGGKQYRVQPGDMLRVEKLAADAGDKVQFNDILMLGGDSPVVGAPFVKDAGVQAEVVDQIKSEKTINFVKRRRKHSSKRTKGHRQKLTLIKVTDILTSGADKSGVKAAIGGAGYAAVV--AEMAKKSAKPAKAKKAESPKAEATKA-KAAPK--KADAAGDDLKQLSGVGPALEKKLHEGGVTTFAQIAAWTEADIADMDEKLSFKGRIEREGWVEQAKELTKGMSRVKGGTVTHARHKKVIKAAKGYYGRRKSTFKVARQAVDKANQYATRDRKNRKRNFRALWIQRINAAVRSHDEALTYSRFINGLSLAGIEVDRKVLADLAVHEPDAFGAIVKQAQDALAAMQVTETLNDGLKRGYAITVTASELEAKVNEKLAEAQPEVEMKGFRKGKVPMALLKKQFGQKVMGEAMQESIDGAMNEHFEASGDRPAMQPEVKMTNEDWKEGDDVEVSMSYEALPEIPELDFSKIKLEKMVAKAGDEAIDEALANLAETAQDFKARKKGAKAKDGDQVVMDFVGRVDGEAFDGGAAEDYPLTLGSNSFIPGFEEQLVGVKAGEEKAVTVSFPEDYQAENLKGKEAVFDCTIKEVKEPVAAEINDELATKFGAEDLAALKAQIAERLEAEYAGAARAVMKRSLLDELDKMVDFDLPPSLVDAEAGQIAHQLWHEENPEVEGHDHPDIEATDEHKKLATRRVRLGLLLADIGQKAEVQVTDAEMTQAIMNQARQYPGQERQFFEFVQQNQQMQQQMRAPLFEDKVVDHVFENAKVSEKEVSKDDLQKAVEALEDEMKLNELSDNEGATKKRKRVGRGPGSGTGKMGGRGIKGQKSRSGVAINGYEGGQMPLYQRLPKRGFNKPNRKSFAVVNLGLIQKFVDAKKIDAKKAITEDVLIESGLVRRKLDGIRVLAKGDVTGKLNIEVTGASKSAVAAVEKAGGSLSVAAPVAETSEAMIQMQTNLDVADNSGARRVQCIKVLGGSKRKYASVGDIIVVSVKEAIPRGRVKKGDVRKAVVVRTAKEVRRDDGTAIRFDRNAAVILNNNNEPIGTRIFGPVVRELRAKNFMKIISLAPEVLMRHARGYRRLNRTHEHRKALWANMAGSLIEHEQIKTTLPKAKELRPIIEKMITLAKRGDLHARRQAASKLKQDQYVTKLFDVLGPRYKDRQGGYVRVLKAGFRYGDMAPMAIIEFVDRDRDAKGAADKARHEAEEVTADMLQPKRTKFRKQFKGSIKGLAKGGSDLNFGTYGLKAVEPERVTARQIEAARRAMTRHMKRQGRVWIRIFPDVPVTAKPIEVRMGKGKGSVDRWAAKVKPGRVMFEIDGVGDDVAREALRLAAMKLPIKTRVVVREDWMTTLTLYNTRTRAKEAFQPINPENVRMYVCGPTVYDRAHLGNARPVIVFDVLFRLLRHVYGPEHVTYVRNFTDVDDKINARRKDAGAPIRERSDETIAWYLEDTKALGTLEPDHMPRATEYIAQMVAMIETLIAQGHAYVAEGHVLFAVDSWEDYGRLSGRSVDDMIAGARVEVAPYKRNPMDFVLWKPSGEDLPGWDSPWSRGRPGWHIECSAMAKELLGDTFDIHGGGNDLMFPHHENEIAQSCCANETEAMANVWMHNEMLQVEGRKMSKSLGNFFTVRDLLDQGVPGEVIRFVMLSTHYRKPMDWTEKKRAEAEKTLRRWRGLAADIEPAASPAPAVIDALSDDLNVPGAIAALHGMARRRDVATFVASAQVLGLLEKSAGGWAQTKLTPLDGI-RRLAKVWREMRSNKDFLSADDLKIRLAEHGVELKATTEGVEVFVARPFDPAALEALRMAQVKTSSKSDPNYKVVAENRRARFDYAIEEDLECGIILEGSEVKSLREGGANIAESYAAVEDGELWLVNAYVAPYKQAKTFQHDERRRRKLLVSRKQLSNLWNSTQRKGMTLVPLVLYFNHRGMAKIKIGIAKGKKLHDKRETSAKRDWSRQKSRLLKEHSMNAKAEHYDVIRKPIITEKATMSSENGAVVFEVAMDSNKPQIKEAIEALFGVKVKAVNTTITKGKVKRFRGQLGKRKDVKKAYVTLEAGNTIDVSTGLMSKDKNPRRVADNEAMAKLRMLRTSPQKLNLVAGLIRGKKVEQALTDLTFSKKRIAQDVKKCLQSAIANAENNHNLDVDELVVAEAYVGKNLTMKRGRPRARGRFGKIIKPFSEITIKVRQVEEQAMVSAVESMAANTSWSALGKATDLRNRIFFTLGLLIVYRLGTFIPVPGIDGVALREFMEQAGQGIGGMVSMFTGGALGRMGIFALGIMPYISASIIVQLMTAMVPQLEQLKKEGEQGRKKINQYTRYGTVFLATLQAYGLAVSLEAGDIVTDPGLFFRMSCVITLVGGTMFLMWLGEQITARGIGNGISLIIFVGIIAEIPAAIAQFFASGRSGAISPAVIIGVLVMVVGTIMFVVFMERALRKIHIQYPRRQVGMKVYDGGSSHLPVKVNPAGVIPAIFASSLLLLPVTISTFSGNSTNPVMSWLLANFGPGQPLYLIFFVAMIVFFAYFYTFNVSFKPDDVADNLKNQNGFIPGIRPGKKTSEYLEYVVNRVLVLGSGYLAAVCLLPEILRGQFAIPFYFGGTSVLIVVSVTMDTIQQVQSHLLAHQYEGLLEKSQLRGKS-KGRKKRSPVRRMKFLDLCKVYIRSGAGGGGCVSFRREKYIEYGGPDGGDGGTGGSVWAEAVDGLNTLIDFRYQQHFFARNGQPGMGRQRTGKDGDDIVLRVPVGTEILDEDQETVITDLTEVGERIELARGGNGGFGNLHFKSSTNQAPRRANPGQDGVERTLWLRLKLIADVGLLGMPNAGKSTFLAATSNARPKIADYPFTTLHPNLGVVGVDNTEFVVADIPGLIEGASEGRGLGDLFLGHVERCAVLLHLVDGTSPTLIEDYQTIITELEAYGGDLATKPRVTVLNKIDALDEEERATAHIALADASGGDVMMMSGVAREGLTEVLRALRSQIDDDRLRHRPQEEAEPWHPMAAKLKKGDKVIVLAGKDKGKTGSITSVDPKSGKAIVDGVNIAIRATRQSQTSQGGRIPKAMPIDLSNLAFVDKNGKASRVGFKMDGDKKVRFAKTTGDVIDAMLRSGVIAKKVGMTRLFMEDGKQIPVTVLQLDKLQVVAQRTSDKDGYSAVQLGAGSAKAKRTSQAMRGHFAAAKVEPKRKVAEFRVDEANLIGVGEEITANHYFEGQFVDVAGTSIGKGFAGAMKRHNFGGLRATHGVSISHRSHGSTGQCQDPGKVFKGKKMAGHMGAARVTTQNLQVIRTDADRGLIMVKGAVPGSKGGWVTVKDAVKKPFPENAILPAALKSDAEAAAKAAEEAAAAAAAEAEAEAKRLAEEQAAQEAEQLKAAEAEIEAEKSDDAPAADEDKKEGDAMALKSYKPTTPGQRGLVLIDRSELWKGRPVKALTEGLTKNGGRNNTGRITMRRKGGGAKRLYRIVDFKRNKRDVTAAVLRIEYDPNRTAFIALLKYTDGEQAYILAPQRIAIGDTVVASEKADIKPGNAMPFSGMPIGTIVHNIEMKPGKGGQIARAAGTYAQFVGRDGGYAQIRLSSGELRLVRQECMATVGAVSNPDNSNQNYGKAGRMRHKGIRPSVRGVVMNPIDHPHGGGEGRTSGGRHPVTPWGKPTKGAKTRNKNKASGKLILRSRHAKKKGRMLDSATYTPRLKAVYADTIRAAMKEEFGYKNDMQIPRLDKIVLNIGCGAEAVRDSKKAKSAQEDLTLIAGQKAMTTIAKKSIAGFRVREEMPLGAKVTLRGDRMYEFLDRLITIAMPRVRDFRGVSGKSFDGRGNYAMGLKEHLVFPEINFDKIDENWGMDIVIATTAATDAEAKALLKAFNMPFNSMKLDVIKLDGGKAGSVDLDEALFGLEPRADILHRVVRWQRNNAQAGTHKVKTRKEVSYSTKKIYRQKGTGGARHGARSAPIFRGGGVYKGPVVRSHGHELTKKFRKLGLCHALSAKLKAGELVIIDDATSDGKTAALAKQVKNLGWKRALVIDGASVNENFAQAARNIDGLDILPTMGANVYDILKRDTLVITKAGVEALEARLKMENVVLIVHLILALSLIAVVLLQRSEGGGLGMGGGGGGAVSGRAAATALGKLTWLLAIAFICTSITLTIFAAENASGSSVVDRLGVTPPALNEPASPAGPEGDDLLPPAPGDNAPLVPTLEMAFFKKLKDRLFKSSSKIDEGLEAIVTDGGVEETEAAPEAESAAEAPAAAVEAVPEAVDPEPAPLPADEA-VPETIQPEP--EPEPEPEPEPEPEETR----------APLPRTVAS--------SP-LREDRP-AEPAAKPGMLGRLFGRAAPQTVVRRTLDDEMLEQLEELLISADMGVDTALRVVANMAEGHIGRKLSVAEIKELMSAEIARIMEPVARPLPLYPKTPQVVLVVGVNGSGKTTTIGKLASQFRAAGKKVVIAAGDTFRAAAVEQLQVWGDRAGVPVLTAPEGSDPASLAFDAMGRAQADGADLLLIDTAGRLQNRSDLMEELAKIVRVIRKKDESAPHNTLLVLDATTGQNALNQVKVFSDISDVSGLVMTKLDGTAKGGVLVALADKFGLPIHAIGIGEQIDDLDAFDPEEFADALMGVERMTNEMWGQLRQRLQKTVGQNNFKAWIEPIEFRSADDGIAVFHVPTNFLGNYVNQNYADIILHEVRARSPEVRRLKFVVARTVETE---AANTDTVHRAARQVQN--GSALTTAPLDTRFTFDSFVVGKPNELAHAAAKRVAEGGPVTFNPLFLYGGVGLGKTHLMHAIAHELRLRRPELNVLYLSAEQFMYRFVQALRDRKMMDFKEIFRSVDVLMVDDVQFIAGKGSTQEEFFHTFNALVDQNKQIIISGDRAPGEIKDMEERVKSRLQCGLVVDLHPTDYELRLGILQSKVEQQRVNYPGLEVENGVLEFLAHRISTNVRVLEGALTRLFAFASLVGREINMELTQDCLSDVLRASERKITVEEIQRKVSDHYNIRLSDMIGPKRLRSYARPRQVAMYLCKKMTSRSLPEIGRRFGGRDHTTVMHGVKRIEELKVQDGQIAEDLELLRRALEEMLGFGTITKKVFGTPNDRKIKATRPLIEQINGLEPEFEKLTDAGILEKTEELRARALKGESLDDLLPEAFANCREAARRALGLRAFDTQLMGAVFLHEGNIAEQKTGEGKTLTATFAAYLNALTRDGVHIVTVNEYLAKRDASWMGKVFGALGLSTGVAWSGMPEEDKREAYDSDVTYATNNELGFDYLRDNMRSELSQIFQKKHNFAIVDEVDSILIDEARTPLIISGPSQDRSGMYQTIDALIPSLNDTHFELDEKTRNVTFTDEGNEFLEQVLRDNELLEEGQTLYDPESTTIVHHINQGLRAHKLFQRDKDYIVRDDSVVLIDEFTGRMMAGRRLSDGLHQAIEAKEGVEIRPENITLASVTFQNYFRLYNKLAGMTGTALTEAEEFSEIYGLGVVEVPTNRPVARVDEDDQVFRTAVEKYRAMLEQIKESHEKGQPVLVGTTSIEKSEMLSKMLTDAGLKHNVLNARQHEQEAQIVADAGKFGAVTIATNMAGRGTDIQLGGNVDLQIMEALAADPEADPEALRQGIEASHAGEKAKVIEAGGLYVLASERHESRRIDNQLRGRSGRQGDPGRSSFFLSLEDDLMRIFGSERLEKVLKTLGLKEDEAIVHPWVNKSLERAQAKVEGRNFDIRKQLLKFDDVMNEQRKVVFGQRREIMEAEDLSEITTDMRHQVIDDLIDQYLPPNTYADQWDSEGLNEAVREQLGMDLPVADWTAEEGVDDDVMRERLIEATDAFMKDKAAQFGPENMRNIEKQLLLQSIDGKWREHLLTLEHLRSVVGFRGYAQRDPLNEYKNESFQLFEGMLDSLREDVTQKLAQIRPLTEEEQKQMVEQMRAQQAEAQAAGEQAA-ALSAEPEGDSDAVAGFVEDDPSTWGNPGRNETCPCGSGKKFKHCHGRLIMKLAGELKSFQDRLGYEFARPELLVEAVTHASVSTANRDDNQRLEFLGDRVLGLVMAEALLATDDKASEGQLAPRFNALVRKETCADVAREINLGAVLRLGRSEMLSGGRRKQALLGDAIEAVIAAVYLDAGFDQAREMILRLWGRRVHEVEEDARDAKTALQEWAQARKQQPPEYVETARTGPDHAPVFTITARLDTGESEAANAGSKRQAEQAAAAALLAKLESRSMAKLGKRTRAAREAFAGKEDVTVEEAVALIKGNSKVKFDETVEIAMNLGVDPRHADQMVRGVVGLPNGTGKTVRVAVFARGPKAEEAEKAGADIVGAEDLMETVQSGKIEFDRCIATPDMMPIVGRLGKVLGPRNLMPNPKVGTVTMDVEAAVKAAKGGEVQFKAEKGGVVHAGVGKMSFDEGKLVENIRAFVSAVSRAKPSGSKGTYMKKIALSSTMGPGVTVSVDNAVTEMSDDFMLDTDDLERRMDGAMSSLKTEFASLRTGRASASMLEPVMVDAYGQRTPINQVGTVNVPESRMVTINVWDKALVGKVEKAIRESGLGINPQLNGTIIMLPIPELNEERRRDLTKVAGQYAEHARVSVRNVRRDGMDQIRKAKGDGLSEDDQKLWEGEVQEITDRYIKMIDDLLATKQSEIMQVMAKKLVGSMKLQVPAGQANPSPPVGPALGQRGINIMEFCKAFNAKTADMEPGAPCPTVITYYQDKSFTMDIKTPPASYYLKKAAKLKSGAKTPSREVVGHVTTKQVREIAEAKMKDLNANSIEAAMQIILGSARSMGIEVKMFENLSERLSGVFDRLTKQGALSDEDVKTALREVRVALLEADVSLPVARDFVKAVQDKATGQAVTKSVTPGQQVVKIVHDALIDVLRGEGEPGHLKVDNPPAPILMVGLQGGGKTTTTAKLAKRLKEKDGKRVLMASLDVNRPAAMEQLAILGLQIGVDTLPIVKGEDPVAIAKRAKTQAGLGGYDVYMLDTAGRLSIDEELMSQVEAVRNVANPRETLLVVDGLTGQDAVHTAENFDERIGITGVVLTRMDGDGRGGAALSMRAVTGKPIRYVGLGEKMDALETFEPDRIAGRILGMGDIVSLVEKAQETLELEQSEKMVKRMMKGQFSMNDLRMQLEQMQKMGGMQGMMGMMPGMGKMARQVEDAGLDDSILKRQIALIQSMTRRERANPQILQASRKKRIARGAGLEVSELNKLLKMHRQMGDMMKKMGKMGKGGMLKQAMKGMFG-GGPSPEEMAAGMDPKALEAAARQMGGKLPG----MGGGAGLPPGLSGFGKKKMIPRDRLEQLTARFQYLEAAMADGAQGADIAQLAREYSELRPVVAQIVAWDQLCRDMDEAREMLSDPDMAGLAEEELQRLKAALPAAEDALQRALLPRDAADAKPAMLEIRPGTGGDEAALFAGDLMRMYQRYAETRGWAFEVIELQETELGGVKEMVAHIRGEGVFARLKYESGVHRVQRVPSTESGGRIHTSAATVAVLPEAEDVDIRIEPGDLRIDTMRSSGAGGQHVNTTDSAVRITHLPTGIVVTSSEKSQHRNRDRAMQVLKARLYDLERSRADSERSADRAAQVGSGDRSERIRTYNFPQGRMTDHRINLTLYRLDAVMQGDLDEIIDALTADAQARMLAEMGQMDRAQKEQLVDELGQIFESSGVVVVSHYAGLTVAEMQDLRAKARDAGGSVRVAKNRLAKIALEGKPCESIADLLTGMTVLTYSEDPVAAAKVAQEFAKGNDKFVILGGAMGENALDVAGVEAVSKMPSREELISTIAGMLGAPASNIAGAIGAPASNIASILSTIEDKAAAMKTFSATPADIDKKWILIDAEGVVLGRLASIVAQRLRGKHKPSFTPHMDCGDNVIVINADKIQLTGKKRDEHHYWHTGHPGGIKSRTKAQILEGAHPERVVTLAVKRMLPGNRLSRQVMTNLRVYAGGDHPHEAQSPEVLDVASMNSKNTRSA---MSAELISAVRDHLPTALPGRLGVAVSGGSDSLALLSLLQEICAGTATTLLAVTVDHGLRPGAAREAAGVARHCAQLKIAHETLVWSGWDGQGNTQDAARRARYGLIAGWAQKENIPAVALGHTLNDQAETVLMRLARGAGVDGLSAMSVRREAHGVAWLRPLLGTGRDRLREYLSAKGIVWSDDPSNDDDRYDRIRARRALEVLSPLGLDPQVLCAVADNMRSTRDALDRHLIEKAHDIVTVTAGAVCIDRAGLVSLPEETRRRLLSGVLRWIGGAAYAPRASAVAAFAQALEEEASATLAGCHARRVKETVWIFREFRAVAGLETSHDAAWDDRWRLSGPVP-PEGATLRALGDAGLKDCPDRRAAGLPRDLQRASPGLWLGERLLCAPQAGLQADWQIRAEGTRDAFFAALLSHMADLKKLAEEIVGLTLLEAQELKTILKDEYGIEPAAGGAVMMAGPADGGGAAEEKTEFDVVLKNAGASKINVIKEVRGITGLGLKEAKDLVEAGGKIKEGCDKAEAEEIKGKLEAAGAEVELAMSRIGKKPVDLPSGVSASVSGQTIEVKGPKGTRSFNATDDVTLKVEDNVITIEPRGKSKRARQQWGMSRTQIQNCVTGVTEGFKKELEIQGVGYRAQMQGNTLKLNLGLSHDVDYTAPDGVTVTAPKQTEIIVEGIDEQLVGQVAANIRQWRKPEPYKGKGIRYKGEFVFRKEGKKKMQVILLERVAKLGQMGEVVDVKPGFARNFLLPQGKALSASKANIAAFEGQKAQLEARNLETKKEAESLAEKIDGQQFIVIRSASDSGALYGSVTTRDAADVATEAGFSVDRKQVVLS-PIKELGIHPVQIVLHPEVDATIELNVARSPEEAELQAAGKSIQELAAEEEAAADFEIQELFDDIGAAASEDDDLAEAAGVAAGEASGDQEDTAMSDTDGKKTLGLRGNARPGNVKQSFSHGRTKNVVVETKRKRVVVPKAGATKSAGTSGAAGGDPSRRPAGITDAEMERRLKAVQAAKARESEEAAEREAEEKARAEDRERRRAEQEEKEREEREREESLKAKAQEDQRRKDEAEAAAKAAAAPAEEPVVQRPAREKAAPEAAPRKQDRDRDTN-KRGKGGDDNRRSGKLTLNQALSGGEGGRQRSMAAMKRKQERARQKAMGGQVEREKVVRDVQVPEAIVVSELANRMTEKVGEVVKALMNNGMMVTQNQTIDADTAELIVEEFGHKVVRVSDADVEDVIKEVEDDDKDLSPRPPVITIMGHVDHGKTSLLDAIRNAKVVAGEAGGITQHIGAYQVKTEGGQLLSFLDTPGHAAFTSMRSRGAQVTDIVVLVVAADDAVMPQTIEAINHAKAAKVPMIVAINKIDRPAADPNKVRTDLLQHEVIVEQMSGDVQDVEVSAITGQGLDELLEAIALQAEILELKANPNRAAVGAVIEAQLDVGRGPVATVLVQNGTLRQGDIFVVGEQYGKVRALINDKGERVKEAGPSVPVEVLGLNGTPEAGDVLNVTGTEAQAREIAEYRANAAKDKRAAAGAATTLEQLMANAKADENVSELPILVKADVQGSAEAINQAMEKIGNDEVRVRVLHSGVGAITETDVGLAEASGAPIMGFNVRANASARNTANQKGVEIRYYSVIYDLVDDVKAAASGLLSAEIRENFIGYATIKEVFKVTGVGKVAGCLVTEGVARRSAGVRLLRDNVVIHEGTLKTLKRFKDEVAEVQSGQECGMAFENYDDIRPDDVIEIFEREEVTRTLTMGKPKKTPRPKAETPKGFRDYFGAEVTQRTEMLRKIAGVYHRYGFDALESAGVETVEALGKFLPDVDRPNEGVFAWQEDADADKPGDWLALRYDLTAPLARVYAQHQNDLPKPYRRYAMGPVWRNEKPGPGRFRQFYQCDADTVGAPSVAADAEICAMLADCLEAVGIERGDYVIRVNNRKVLNGVMEVAGLAGDDKAAERGIVLRAIDKLDRLGPDGVRALLGQGRKDESGDFTEGAGLSALQTDKIMSFVNSHRDEKEQRVDHLKELIGGTGVGFSGISDLQEIHDLMTSAGYSDQRFLIDPSVVRGLGYYTGPVYEAELTFDIQDEKGRTRNFGSVAGGGRYDDLVKRFTGQEVPATGVSIGVDRLLAALQAKGRLESEAQGPVVVTVMDRARMSYYQELVADLRNADIRAEVYLGNPKNFGNQLKYADKRGSPAAVIAGSDEQERGVIQIKDLILGAKIAESATLEEWKDRPSQFEVPRDQLVAKVREILAAHSMDDLKQKYLGQIADAADENALEAIRLGAVGKKGEVSLKMRELGRMTPEERQVAGPALNALKNEINSALAARKAALSDAALDERLRTEWLDVTLPARPGRQGTIHPVSQVTEEVTAIFGEMGFTVAEGPRIDTDWYNFDALNIPGHHPARAEMDTFYMARADGDERPPHVLRTHTSPVQIRTMEAQGAPLRIICPGGVYRADYDQTHTPMFHQIEGLAIDKDISMANLKWVLEEFFSAFFGIEGIRTRFRASHFPFTEPSAEVDIQCSWIDGQLRIGEGDDWLEVLGSGMVHPNVLKAGGVDPDVWQGFAFGMGIDRIAMLKYGIPDLRAFFDSDLRWLRHYGFASLDQPTLHGGLHRMEMAEEAGLDLVEISPNANPPVCKIMDFGKFKYEQQKRESEARKKQKIIEIKEVKFRPNTDTNDYDVKMRNVFKFLENGDKVKITLRFRGREMAHQNLGRELLERVAEDTKDAGRVENFPKMEGRQMVMLIGPLPKMKFTLSWLKDHLETKATLDEILYALTDLGLEVEGVEDRAARLKDFTLGYVASAEKHPDADRLRVCQVDTDEGQKQIICGAPNAREGITVVIAKPGVYVPGIDTTIGVGKIRGIESFGMMASERELELSEEHDGIIELPSGEVGQSFVDWLAENDPAKVDPVIEIAITPNRPDALGVRGIARDLAARGLGKLKKRDVDAVPGAFACPVSVTIDGDTLDQCPVFFGRVIRGVTNGPSPQWLQDCLRAIGLRPISFLVDVTNFFTYDRNRPLHVFDADKIAGNLRVHRAKGGETLTGLDEKEYIFEEGMTLISDENGVESIGGVMGGMASGCTEETVNVFLEAAYFDPVRTAYTGRALKINSDARYRFERGIDPAWTPYGIEHATRMILDHAGGEASEVIVAGKIPDTDRAYRLDAARVRSLVGMDIPESEQRQTLTSLGFILEGNMAHVPSWRPDVQGEADLVEEVARIASLTKLKGKPLKRLTEGVPKPILSPMQRREAIARRTCAALGYNECVSYSFIDQASAALFGGGDDATRLENPISSDLSHMRPALLPGLLQAAARNQARGFADMALFEVGPGFHGGEPGEQHLLVSALLVGRTGPKDVHGASRAVDVFDVKADAEAVLAAMGAPAKVQIMRTGRDWWHPGRHGVICLGPKKVLGVFGEVHPRVLEAMEVKGPAMAFTLWPAEIPLPRKAGATRAALAISDLQAVERDFAFVVEAQTEALTLINAAMGADKALIEDVRVFDEFVGGSLGENRKSLAITVRMQPKDQTLTEKDIEAVSAKVIEKVTKATGGTLRGMHAYRSHTCADLTAANVGETVRLSGWVHRVRDHGGVLFIDLRDHYGMTQVLCDPDSAVFAQVEKVRAEWCIRIDGEVKARDASLVNAKIPTGEIEVFVRDIEVLGGAGELPLQVFGDQEYPEETRLRYRYLDLRREKMQANMKLRSDVVASVRRRMWDADFREFQTPIITASSPEGARDFLVPSRLHPGKFYALPQAPQQFKQLLMVSGFDKYFQIAPCFRDEDPRADRSPTDFYQLDMEMSFVEQQDVFDTIQPVITGIFEEFGGGRPVDQEWPQISYKDAALWYGTDKPDLRNPIRMQVVSEHFAGSGFAIFAKLLEQEGTQIRAIPAPGGGSRKFCDRMNAFAQKEGLPGMGYIFWRDQ-GQ--GMEAAGPLAKNIGPERTEAIRQQLGLEVGDAAFFLGGKPKTFEGVAGKARTVIGEELGLTEKDRFAFAWIVDFPIYEKDAETGKIDFEHNPFSMPQGGMAALEGDPLDVLGYQYDLACNGYELVSGAIRNHRPEIMFKAFEIAGYGEDEVRKRFGGMVNAFQYGAPPHGGCAAGIDRIVMLLADESNIREVILFPMNQRAEDLMMSAPSEPLSDQLMELGLRVIPQEMTPLSHIRNFSIVAHIDHGKSTLADRLIQSTNTVSAREMKEQMLDSMDIERERGITIKAQTVRINYTAENGEEYVLNLIDTPGHVDFAYEVSRSMRAVEGSLLVVDSTQGVEAQTLANVYHAIEADHEIVPVLNKIDLPASDCDRVAEQIEDVIGIDASEAIRVSAKTGQGITETLEAIVHKLPAPKGNPDAPLKAMLVDSWYDSYLGVIVLVRIMDGQLKKNDRIRFMQNGTVHHVDRIGVFRPAMTEIDSLNPGEIGFLTASIKQVRDTRVGDTVTHEKNPTDKALPGFKPSQPVVFCGLFPVDSAEFEDLRDAIEKLALNDASFSYEMETSAALGFGFRCGFLGLLHLEVIRDRIEREYDIELITTAPSVIYHIHMKDGEMMELHNPADMPDLTLVDHLQEPRIKATILVPDDYLGDVLKLCQDRRGIQLDLTYAGSRAMVVYDLPLNEVVFDFYDRLKSVTKGYASFDYQMIGYREDKLVKMSILVNDEPVDALSTMVHRDRAEQRGRAMVEKLKDLIPRHMFKIPIQAAIGGKVIARETLSAMRKDVTAKCYGGDATRKRKLLDKQKAGKKKMRQFGKVDIPQEAFISALKMDGMSLPPGFLDELRTRSSLSQVVGRKVTWDARKSNQGKGDMWAPCPFHQEKSASFHVDDRKGFYYCFGCHAKGDAISFVRETENVGFMEAVEILAREAGMPMPERDPQAQQKADRRTQLADVMEQAVRYFRLQLNTGAAAEARDYLERRGLRADALERWEIGFAPDQWQGLWDHLKEKNVEDDLILGAGLAKPSSKGGKPYDTFRGRIIFPIRDARGRAIAFGGRAMDPNDNAKYLNSPETDLFDKGRSLFNHGPARTAAGKGQHLIVAEGYMDVIALASAGFEAAVAPLGTAITESQLQMLWRISPEPVIALDGDQAGLRAAMRLIDLALPLVEAGQSLRFALMPDGQDPDDLLRAQGAPALQKILDAAMPMVRLLWQRETEGRVFDSPERKAALDKALREKIMLIRDPSIRSHYGQEIKDLRWQLFRPQRSA-QKPRT----GAQG-WNAPEKGAISSSRASFLVSAQGPRATERLREAVILATLATCPAVIEDFEGGIEQMRCGDPDHAALRGVLLSGLGLNAAQMRDKIDDVLGPEALENLLSQRHVSVVPCIRSPGDEEKARMTVAEELAKLQSARGLDAEIIEAAEDMSGEADEGLTWRLSEAARAAERAQRSVQEDRAEYETGENGVRISREERSTFNALLQAIRQHKPHD--MCADT-EYKSTLNLPRTEFPMRAGLPKREPDWLARWEEIGIYDRLREKDGRTPFTLHDGPPYANGHLHIGHALNKTIKDMIVRSHQMMGFDARYIPGWDCHGLPIEWKIEEQYRQKGRDKDDVPINEFRAECRQFAAGWVDIQREEFKRLGVTGNWADPYLTMDFHAERVIAEEFMKFLMNGTLYQGSKPVMWSPVEKTALAEAEVEYHDKDSFTIWVKFKVA-------DLLDAYVVIWTTTPWTMPSNKAVVYGEDISYGLYEITGRPEESWVTISDRYILADKRAADVMSRARLDETMYRRLRDVSSDELSRLSLNHPLAGADGAEGEWDDTRDFRAADFVTDDEGTGFVHCAPSHGMEEYELYRDLGMLQDVITYNVTDEGMFREGLPFFGGKYILNRKGKEGDANAAVIDKLVEVGGLMARGKIKHSYPHSWRSKAPVIYRNTPQWFAAIDKTVGDGQDEFGTTIRERALTEIDNVNWVPKSGRNRLHSMMEARPDWVLSRQRAWGVPLTCFTRKGLLPTDPDFLLRNEAVNQRIVEAFEAEGADAWYEEGAKERFLGGIVDPDDYEQVTDILDVWFDSGSTHAFTLRDRPDGTKDGIADVYMEGTDQHRGWFHSSLLQSVGTMGRAPYRNVVTHGFTLDEKGMKMSKSLGNTIVPEKIVQQYGADILRLWVAQTDYTLDQRIGPEILKGTADSYRRLRNTMRYMLGSLADFSEADRVTAEEMPELERWVLHRLAELDKTVRNGYAAYDFQGVWQAVFTFATVDLSAFYFDIRKDVLYCDGDTRERRAARTVLDLLFHRLTTWLAPVLVFTMEEVWLERFPGDESSVHLQDFPETPAGWQNADLARKWSGIRSARRVVTAALEIERTNKVIGASLEAAPVVYIADDALRSAVSDLPFQDICITSQITVSGDPAPEDAFRLPEVAGVAVAFARADGEKCARCWKVLPDVGTHAHPDVCGRCDAAVSAAQSQNIRIRLKAFDYRVLDASTQEIVNTAKRTGASVRGPIPLPNKIEKFTVLRGPHVDKKSRDQFEIRTHKRLLDIVDPTPQTVDALMKLDLAAGVDVEIKLQSMADEINTLEDLKDVVTGAVQGVAAEEAIQ---REPVRDDLGRSYATGKRKDAVARVWIRPGSGKVTVNGKPQNEYFARPVLQMILAQPFTVSGTEGQFDVVATVKGGGLSGQAGAVKHGVSKALQLYDPSLRGALKAAGFLTRDSRVVERKKYGKAKARKSFQFSKRMPTIQQLIRKPRQPKIKRSKSMHLQECPQKRGVCTRVYTTTPKKPNSAMRKVAKVRLTNGFEVISYIPGESHNLQEHSVVLIRGGRVKDLPGVRYHILRGVLDTQGVKDRKQRRSKYGAKRPKMAREKVRVKKKVSKNIAAGVAHVNSSFNNTKILISDVQGNAISWSSAGTMGFKGSRKSTPYAAQMAAEDAGRKAQEHGVKTLEVEVQGPGSGRESALRALAAAGFNITSIRDVTPMAHNGCRPPKRRRVMSATRRGLLIILSSPSGAGKSTLARRLRDWDPEIAFSVSATTRAPRPGEEDGREYHFVDDSAFKAQVAAEEMLEHAHVFGNFYGSPRAPVQKAIDSGRDVLFDIDWQGAQQITNSALSAHTLSIFLLPPSITELRSRLEKRGQDDAETIARRMDKSWDEISHWGSYDYVLVNDDLDATEAQLKTIISATRLRRSQQPGLTDHVRHLQSEFEDLTMAQSFLGQKRLRKYYGKIREVLEMPNLIEVQKSSYDLFLNSGDADVPTDGEGITGVFQSVFPIKDFNETSVLEYVKYELEKPKYDVEECQQRDMTYSAPLKVTLRLIVFDIDEDTGAKSVKDIKEQDVFMGDMPLMTPNGTFVVNGTERVIVSQMHRSPGVFFDHDKGKTHSSGKLLFACRIIPYRGSWLDFEFDAKDIVFARIDRRRKLPVTTLLYSLGLDQEAIMDAYYNTVTYKLKKNKGWVAPFFPDRVRGTRPTYDLVDAKTGEVLFEKGKKVTPRAVKQLVDEGKVTELLLPFEHIAGKFAAKDIINEENGAIYVEAGDEMTLEYDKDGTLIGGTAKELVDAGVTEIPLLDIDNVNVGPYMRNTMAQDKNMNRDTALMDIYRVMRPGEPPTVEAASALFDTLFFDSERYDLSAVGRVKMNMRLALDAEDTQRTLRREDIVACIKALVDLRDGRGDIDDIDHLGNRRVRSVGELMENQYRVGLLRMERAIKERMSSVEIDTVMPQDLINAKPAAAAVREFFGSSQLSQFMDQTNPLSEVTHKRRLSALGPGGLTRERAGFEVRDVHPTHYGRMCPIETPEGPNIGLINSLATFARVNKYGFIETPYRKVENRKVTDEVQYMSATEEMRHTVAQANANLDENMVFVNDLVSTRKSGDYTLSPSESVDLIDVSPKQLVSVAASLIPFLENDDANRALMGSNMQRQAVPLLQAEAPLVGTGIEEVVARDSGAAIMAKRAGVIDQVDASRIVIRATEDLELGDAGVDIYRMRKFQRSNQNTCINQRPLVKVGDRVSKGQVVADGPSTDMGELALGKNVVVAFMPWNGYNYEDSILISERIARDDVFTSIHIEEFEVAARDTKLGPEEITRDIPNVGEEALRNLDEAGIVYIGADVEPGDILVGKITPKGESPMTPEEKLLRAIFGEKASDVRDTSLRVKPGDFGTVVEVRVFNRHGVEKDERALQIEREEVERLARDRDDELAILDRNIYARLKDLILGKTAVKGPKGFKSNSEITEDALEMLTRGQWWQLALKDEADAQIVEALNEQYEIQKRALDARFEDKVEKVRRGDDLPPGVMKMVKVFVAVKRKLQPGDKMAGRHGNKGVISKVVPMEDMPFLADGTPVDFCLNPLGVPSRMNVGQILETHMGWAARGLGVNVDEALQDYRRSGDLTPVREALSLAYGEDVYEEGIAGMDEETLVEAAGNVTRGVPIATPVFDGAKEADVNDALKRAGFDTSGQSVLFDGRTGEQFARPVTVGVKYLLKLHHLVDDKIHARSTGPYSLVTQQPLGGKAQFGGQRFGEMEVWALEAYGAAYTLQEMLTVKSDDVAGRTKVYESIVKGEDNFEAGVPESFNVLVKEVRGLGLNMELLDAEEDEMPLYEHVMIARQDLSNTQAEGLIEHFGAVLSDNGGSLVDHEYWGVKTMAYKINKNRKGHYAFLRSDAPAPAVQEMERLMRLHDDVMRVLTIKVDEHAELPSVQMQKRDERGDRRERRMVPQKDPDNLTEDEARDELAHLAGVLAAANRDYHTLDAPELTDAEYDRLKRRNSAIEARFPALKRSDSPSDQIGAAPAEAFSKVTHAVAMLSLGNAFSDDDVRDFDQRIRKYLGLGETADLRYTAEPKIDGLSLSLRYEDGRLVQAATRGDGAVGENVTANALTIDDIPEVISGAPSVLEVRGEVYMAHADFEDLNVRQSKSDAKTFANPRNAAAGSLRQLDAAITRSRPLRFFAYAWGALSEPLSDRQSGAVARLADLGFSTSPLLKTCASPDEMIAHYQNIETQRADLGYDIDGVVYKVDDLSLQDRLGFRSTTPRWAIAHKFPAELAWTRLRSIDIQVGRTGALSPVARLEPVTVGGVVVSNATLHNEDYIAGRDSRGRQIRGGKDIRVGDLVQVYRAGDVIPKVADVDLSQRQEGSERYEFPRTCPECGSAAIREEGDAVRRCMGGLICPAQAVEKLKHFVSRAAFDIEGLGARQVEQFCTDGWIAEPADIFTLRDRYGSGVKQLKNREGWGEKSALSLFDAIDEKRQIPLARFIFSLGIRHVGEAASGLLALHYGTWPALRDAMLAARDKEGPAWDDLVSVDGVGTVMAGSVVDTLAQDAERASIDRLTAHLEIQKATAPQTGNSPVAGKTVVFTGTLEKMTRAEAKARAERLGARVSGSVSSKTDILVAGPGAGSKAAKAADLGIEILDEDAWLALIEGQMIHKNWAELIKPQQLDVKPGNDPARQATVTAEPLERGFGLTMGNALRRVLMSSLQGAAITSVQIDNVLHEFSSVAGVREDVTDIILNLKGVSIRMEVEGPKRLSISAKGPGVVTAGDISESAGIEVLNRDHVVCHLDDGADLYMELTVNQGKGYVSAEKNKPEDAPIGLIPIDAIYSPVKKVSYDVQPTREGQVLDYDKLTMKVETDGSLTPDDAVAFAARILQDQLGIFVNFDEPESASRADEDDGLEFNPLLLKKVDELELSVRSANCLKNDNIVYIGDLIQKTEAEMLRTPNFGRKSLNEIKEVLSGMGLHLGMDVEDWPPDNIEDLAKKFEDSFMAERDNRRGNRRD-RDETPEFSDRLVAINRVSKTVKGGKRFGFAALVVVGDQKGRVGFGKGKAKEVPEAIRKATEQAKRQMIRVQLREGRTLHHDMEGRHGAGKVVMRTAPEGTGIIAGGPMRAVFEMLGVKDVVSKSIGSQNPYNMIRATMDGLRKESSPRSVAQRRGKKVADILPKREDNVESSAQVAEEAMNDPIADMLTRIRNSSMRGKSTVSTPASKLRAWVLDVLADEGYIRGYEKTTGSDGHPAIEISLKYYEGEPVIRELKRVSKPGRRVYMGVNDIPVVRQGLGVSIVSTPKGVMSDQAARSANVGGEVLCTVFMHDIRAIREAPEAFDAALARRGDAPLSSEILALDQARREKISAAETARAEQNKASKLVGAAKASGDGAEFERLRTLVGEKKAEVSALQGKAAELDTELSEKLSWIANIPADDVPEGADENDNVEVRRWGDQPSFGFAPKEHFEIAGVAPSMDFETAARTSGARFVMLTGAVARIHRALAQFMIDTHVDENGLTEVNSPVLVRDEAMYGTDKLPKFAEDSYQTTNGWWLVSTSEIPLTYTVAGQILDESDLPRRLTAHTLCFRSEAGSAGRDTAGMLRQHQFEKVEMVSVTHPDHSDEEQKRMLRCAEDILERLGIAYRTVLLCTGDMGFGARRTYDIEAWLPGQNAYREISSVSTTGDFQARRMNARFRPA-GGKPQFVHTLNGSGLAVGRCLIAVLENGQREDGSVALPEVLSGYLGGKTTLSADGDLIMAITASMVKELRETTGAGMMDAKKALTENDGDMEAAVDWLRTKGLAKAAKKSGRTAAEGLVAVKVDGGHGVAVEVNSETDFVGKNAEFQSMVSGIADVALTVSDVEALKEADMGGKSVATVITDSIAKIGENMSVRRMSSIEGETVVSYVHNAAAPGMGKIGVLVAMKGGDEAFGKQVAMHIAAVNPASLSEDDLDPAVVEKEKQVQMDIARESGKPEQVIEKMIVGRMKKYMSEVTLLNQSFVVNPDHTVGEAAKEAGAEITGFVRLEVGEGIEVVKEDFAAEVAKAAKGMNQELTNNPFNPLTPPKVFDEIKVSLASPERILSWSFGEIKKPETINYRTFKPERDGLFCARIFGPIKDYECLCGKYKRMKYRGVVCEKCGVEVTLQKVRRERMGHIELASPVAHIWFLKSLPSRIGLMLDMTLRDLERVLYFENYVVIEPGLTDLTYGQMMTEEEYMDAQDAFGMDAFTANIGAEAIREMLAAIDLEAEAEQLRADLKEATGELKPKKIIKRLKVVESFLESGNRPEWMVLTVIPVIPPELRPLVPLDGGRFATSDLNDLYRRVINRNNRLKRLIELRAPDIIVRNEKRMLQESVDALFDNGRRGRVITGANKRPLKSLSDMLKGKQGRFRQNLLGKRVDFSGRSVIVTGPELKLHQCGLPKKMALELFKPFIYSRLEAKGLSSTVKQAKKLVEKERPEVWDILDEVIREHPVMLNRAPTLHRLGIQAFEPVLIEGKAIQLHPLVCSAFNADFDGDQMAVHVPLSLEAQLEARVLMMSTNNVLSPANGAPIIVPSQDMILGLYYTTLEREGMPGEGMVFGSVEEVQHALDAGMVHLHSKITARIPQIDENGLEVMKRFETTPGRIRLGALLPLNAKAPFELVNRLLRKKEVQQVIDTVYRYCGQKESVIFCDQIMTMGFREAFKAGISFGKDDMLIPDTKWPIVEETRELVKDFEQQYMDGLITQGEKYNKVVDAWSKCNDKVTDAMMGSISASQRNEAGAEMEPNSVYMMAHSGARGSVTQMKQLGGMRGLMAKPNGDIIETPIISNFKEGLTVLEYFNSTHGARKGLSDTALKTANSGYLTRRLVDVAQDCIVRMNDCGTETSITAVAAVNDGEVVSSLSERILGRVVAEDIMRPGTDEVLLAAGTLIDERMADAVEEAGVASARIRSPLTCEAEEGVCAMCYGRDLARGTMVNTGEAVGIIAAQSIGEPGTQLTMRTFHIGGVAQGGQQSFLEASHSGKVVFDNASTLENASGEIMVMGRNMKLIIQDENGEERASHKVGYGTKLFVTDGQDVKRGDKLFEWDPYTLPIIAEKSGTAKFVDLVSGIAVRDETDDATGMTQKIVIDWRAAPKGNELKPEIILVDGDGEPVRNDAGNPVTYPMSVDAVLSIEDGAEIQAGDVVARIPREGAKTKDITGGLPRVAELFEARRPKDHAIIAEIDGYVRYGKDYKNKRRIAIESAEDPDTKVEYMVPKGKHIPVAEGDFVQKGDYIMDGNPAPHDILAIMGVEALADYMIDEVQDVYRLQGVKINDKHIEVIVRQMLQKWEIQDSGDTTLLKGEHVDKQEFDQANEKALAKGGRPAQGEPILLGITKASLQTRSFISAASFQETTRVLTEASVQGKKDKLVGLKENVIVGRLIPAGTGGATMKVRRVAQDRDNVVIEARREEAEAAAALAAPSAD-DDMVGGDVFDQVIPDEDSRDMSRRHAAEKREVLPDAKYGDLVLTKFMNNLMIDGKKSVAERIVYNAMTRVEDKIKRAPIEVFHEALDNIKPSVEVRSRRVGGATYQVPVEVRPERREALAIRWLIKASRARNENTMEERLAGELLDAVQSRGTAVKKREDTHKMADANKAFSHYRWMPYAQTDSSEAPAVMANPAPDVRSRPKLEGGRKFVLKTEFEPAGDQPTAITELSDGLVSGERDQVLLGATGTGKTFTMAKVIEKTQRPAIILAPNKTLAAQLYGEFKGFFPDNAVEYFVSYYDYYQPEAYVARSDTYIEKESQINEQIDRMRHSATRALLERDDVIIVASVSCIYGIGSVETYGAMTQDLKVGSSYDQRQVMADLVAQQYRRNDQAFQRGSFRVRGDSLEIFPAHLEDRAWRLSFFGEELESITEFDPLTGEKTDTFDQIRVYANSHYVTPKPTMQQAVIGIKKELRIRLDQLVKDGKLLEAQRLEQRTNFDLEMLEATGVCNGIENYSRYLTGRAPGEPPPTLFEFIPDHAIVFADESHVSVPQIGGMYKGDYRRKFTLAEHGFRLPSCMDNRPLKFEEWDAMRPQSVFVSATPASWEIEQAGGVFTEQIIRPTGLIDPEIEIRPVEMQVDDLLDEVRKVAADGYRTLVTTLTKRMAEDLTEYMHEQGIRVRYMHSDIDTIERIEILRDLRLGAFDVLIGINLLREGLDIPECGLVAILDADKEGFLRSETSLIQTVGRAARNADGRVIMYADRITGSMERAIGETDRRRARQLAYNEEHGITPQTVKKNVEDILAGLYKGDVDMNRVTAKVDNPMAGGNLQAVLDGLRTDMRKAAENLEFEEAARLRDEVKRLEAVDLAIADDPMARQQAVDKAVDDAQKASGRSTMGRGGMRGGVKRKRG-MASKPFFRRRKVCPFSGDNAPKIDYKDTRLLQRYISERGKIVPSRITAVSAKKQRELARAIKRARFLALLPYAVKMPKRILSGVVTSDANAQTVTVSVERRFTHPVLKKTIRKSKKYRAHDENNTFKTGDTVRIIECAPKSKTKRWEVLEAAEMANSPQAKKRARQNEARFQVNKARRSRIRTFLRRVEEAIASGDKEAATAALRAAQPELMRGVTKGVFHKNTASRKVSRLASRVKALGMSRSVWKGPFVDSYVLKKAEASRESGRNEVIKIWSRRSTILPQFVGLTFGVYNGHKHIPVNVSEDMIGQKFGEYSPTRTYYGHAADKKAKRKMARIAGVNIPTAKRVPIALTYITGIGNTSAKAICEAVGIDMARRVNELSDAEVLAVREHIDANYTVEGDLRRETQMNVKRLMDLGCYRGLRHRRNLPVRGQRTHTNARTRKGPAKAIAGKKKMAMKIRLARGGSKKRPFYRIVAADSRMPRDGRFIEKLGTYNPLLPKDSEERVKMDVERVQHWIAQGAQPTDRVARMLEAAGATEKTERNNPNKGTPGKKAQERAQEKADKAAAAAEAAAAPA-EAPAEEMSITAEDKSRIMKDFATKEGDTGSPEVQVAILSSRIATLTEHFKTHKKDNHGRRGLLKMVATRRKLLDYVKGKDESRYQDLIKRLGLRRMQTLNDIRSTFLSYYQKQGHEVVPSSPLVPRNDPTLMFANSGMVQFKNLFTGVETRDYKRATTAQKCVRAGGKHNDLDNVGYTARHHTFFEMLGNFSFGDYFKDDAIRFAWELITRDLGIDKDRLYVTVYHTDDEAAAIWKKVAGLSDDRIIRIATNDNFWMMGPTGPCGPCTEIFYDHGDHIWGGPPGSPEEDGDRFVEIWNLVFMQYEQFEDGTRRDLAAQSIDTGMGIERVAALLQGTNDNYATDLMRSLIEASADATSTDPDGTGRTHHRVIADHLRSTSFLMADGVMPSNDGRGYVLRRIMRRAMRHAHLLGAKDPLMHRLVPALVRQMGAAYPELGQAQPMIEQTLLQEETRFRQTLDRGLKLLDDELSGLPEGADLPGEAAFRLYDTYGFPLDLTQDALREQGRAVDTDGFDTAMAEQKAKARAAWSGSGEAADATVWFDVADKHGTTDFLGYDTETAEGQIAAIVRDGVPTDTAGTGDSVQIALNQTPFYAESGGQVGDSGTLRTQDGEVTITDTRKTAGVFIHFGKVTSGSIATGEAAILEVDHSRRSAIRSNHSATHLLHEALREALGDHVAQRGSLNAEDRLRFDFSHTEALTLDQLATVEADVNRYIRQNAPVETRIMTPDDARAIGAQALFGEKYGDEVRVVSMGRKEGSGKGNDGATYSLELCGGTHVRQTGDIGAFVTLGDSASSAGVRRIEALTGAAAMSYLRAQDHRLAEVALELKAQAADVPERVRSLLDERRALSNEVAQLRRELAMSGGGTAA-PEARDVGGISFMAQVLSGVTGRDLPALVDQFKERLGSGAILLIAEADGKAAVAAGVTGDLTDTVSAVDMVRTAVAGLGGKGGGGRPDMAQGGAKDITNAEAAIAAVEDILKGMGFKMGIVGLPNVGKSTLFNALTRTASAQAANFPFCTIEPNVGEVAVPDARLNRLAGIANSRQIIPTRMTFVDIAGLVKGASQGEGLGNQFLANIREVDAIAHVLRCFEDGDVTHVEGRVDPVADAETIDTELMLADIESIEKRLQNIVRKVRGGDKEAVQQERLMKAALEALEDGRPARVVEVDEEDAKAWRMLQLLSTKPVLYVCNVGEDDAADGNALSARVAELAAQQGNAHVVISARIEEEISQLEPEEASLFLEEMGLSEAGLDRLIRAGYELLHLETYFTVGPKEARAWTIRSGTTAPRAAGVIHGDFEKGFIRAETVAYEEFVSHGGEAAAKEAGKMRSEGKSYIVKDGDVLHFLFNTMAWKTLDDMDLSGKKVLVRVDINVPVEDGRVTDTTRIDRIVPTIRDILAAGGLPVMLAHFGRPKGKFVPEMSLRVTVPALEKALGQEITFIERPDAAI--IAALPEGSVALVENTRFAAGEEANDPAMAQFLASLGDIYCNDAFSAAHRAHASTEGVARLLPSCAGRLMQAELSALEAALSSPERPVGAVVGGAKVSTKIALLENLVGRLDLLVIGGGMANTFLAAQGAELGASLQEPDYLVTARAIMAQAEKSGCRVLLPVDGLVARAFEAGADHEVVALGPDTVLDDDQMVLDAGPESVKAVAAAFEDLKTLIWNGPMGAFEIPPFDQATNAAAADAAARTRAGTLISVAGGGDTVAALNQAGASDDFTYISTAGGAFLEWMEGKTLPGVAALG-MALPEFTMRQLLEAGVHFGHQTQRWNPRMGPYIYGARNGIHIMDLTQTVPMLDAALNVIRETVAKGGSILFVGTKRQAQQPIADAAERCAQYYMNHRWLGGTLTNWQTVSQSISRLKNIDEQSENGFAGLTKKERLGMERDQGKLQASLGGIREMGGRPDLIFVIDVKKEALAVAEANKLGIPVIAVVDTNCPPDGIDYIIPGNDDASRAISLYCDLASRAALDGMSAQLGAAGVDIGEMEEAPAEEAV------AEEAPAAAAETAEA--MTKRTSAKYKIDRRMGENIWGRPKSPVNRREYGPGQHGQRRKGKISDFGIQLRAKQKLKGYYGDLTEKQFRRIYGEAERVKGDTGENLIGLLERRLDAVVYRAKFVATVFAARQFVNHKHVRVNGQIVNIPSYRVKEGDVIEVRDRSKQMAALIEATQLPERDVPDYIEADHSKMSAKFVRTPALGDVPYPVMMEPNLVVEFYAKNMGNKVNPIGMRLQVNRTWDSRWYADTKDYGDLLLEDLAIREFIKEECKQAGVARVIIERPHKKCRVTIHTARPGVIIGKKGADIEGLRQKIAKMTDSELHLNIVEVRKPELDAALVGESIAQQLERRVSFRRAMKRAVQNAMRMGALGIRVNVAGRLGGAEIARTEWYREGRVPLHTLRADIDYAHVEAVTAYGIIGIKTWIFKGEIMEHDPAARDRKAQELQDGPAPRGAGGRRMGRRRKGRDISGWLVVDKPAGITSTSVVNKVRWAFDAKKAGHAGTLDPDATGVLAVALGEATKTVPYVTDALKAYEFTVRLGQATNTDDAEGEVIAQSDLRPDDEAIKEALQSFIGDIEQVPPQFSAVKIDGERAYKRARDGEEMEIAARPLYVDSLLLTDRPDADHVMLEMVCGKGGYVRSVARDLGAALGCFGHVRELRRTWSGPFDAADGLTLQQIDEMAKTPALDAHIKPLEIAFTDLPEVRATQEGAVRLRNGNPGMVMAPGVEYGDTCWASFDGRAVAVGTYRSGELHPTRVLKIQDMFILGLTGSIGMGKSTTAKMFAEEGCAVWDADAAVHRLYVKGGAAVGPLQDALPQAITDGAVSRDALRGIIAGDPDALKRIEAIVHPLVAADRAAFLTETTADISVLDIPLLFETGGDRLVDAVACVSVPGEIQEKRVLDRGTMTPEQFAQIRDKQLPDAEKRARSDYVIITDTVEHARAQVQNIVRQIRENMTNAM-APDILIEDPRWQDCEFERLATRALATTLAHLGLADAVAEISLLACNDARIAGLNTDFRDKAGPTNVLSWPAAELAAAEPGRTPQPPVPGIDGTLELGDIAIAFETCRREATDQGIPMDGHVTHLIVHGLLHLLGYDHIRDADATLMQRLETEILGKMGLDDPYRT---MARYIFITGGVVSSLGKGLASAALGALLQARGFSVRLRKLDPYLNVDPGTMSPFEHGEVFVTDDGAETDLDLGHYERFTGVPARMTDSVSSGRIYSNVLEKERRGDYLGKTIQVVPHVTNEIKSFLDIGDDEVDFMLCEIGGTVGDIEGLPFFEAIRQYSHDKPRGQCIFMHLTLLPYLAASGELKTKPTQHSVKELQSIGIAPDILVCRSEQPIPEKEREKIALFCNVRKEAVVAAYDLSTIYDAPLAYHAQGLDQAVLDAFDISPAPRPDMTVWDDVSDRVHNPEGEVKVAIVGKYTQLEDAYKSIAEALTHGGMANRVRVKVEWVDAEIFDAEDVAPHLEGFHAILVPGGFGERGTEGKIKAAEFARTRKVPYLGICLGMQMAVIEAARNVADIAAAGSEEFDHEAGKKRFEPVVYHLKEWVQGNARVSRKVGDDKGGTMRLGSYDAVLKEGSRVAEVYGKTSIDERHRHRYEVDIKYREQLEAAGLCFSGMSPDGKLPEIVEWSDHPWFIGVQFHPELKSKPFDPHPLFRDFIRAAKEISRLVMAAPCTVLALESSCDDTAAAVLRRTAGGATEILSSVVRGQAELHAGFGGVVPEIAARAHAETLDHCVEQALEASGLGLDDIDAIAVTAGPGLIGGVVSGVMCAKGLAAATGKPLYGINHLAGHALTPGLTDAVPYPWLMLLVSGGHCQFLLVRGPEDFTRLGGTIDDAPGEAFDKIARLLGLPQPGGPAVEAAAKDGDPARFAFPRPLLDRAGCDMSFSGLKTAVLRTRDALIADQGGLTEQDRCDLCAGFQAAVADVFAEKTRRAMDNYPKVADQTPVICVAGGVAANITIRRALETVSQEFGARFIAPPLALCTDNAAMIGYAALEQMAISGPSGMSLPARPRWPLDQTSPAMLGSGKKGAKA------------------------------------------------------------------------------------------------------------------------------------

>'Su-donghicolaDSW-25'

MIAKELHDKTPDQLRDELVNLKKEAFNLRFQQATGQLENPARLRTVKRDVARVKTVLNQKAAAAATDAMATADLLNMD-KKSADKQKALDSALAQIERQFGKGSIMKLGSDDAIQDIKASSTGSLGLDIALGIGGLPMGRIIEIYGPESSGKTTLTLHCVAEQQKAGGVCAFVDAEHALDPQYARKLGVDIDELLISQPDTGEQALEITDTLVRSGAVNMVIVDSVAALTPKSELEGEMGDSSVGVQARLMSKAMRKLTGSISRSNCMVIFINQIRMKIGVMFGSPETTTGGNALKFYSSVRLDIRRIGSLKDRDEVVGNQTRVKIVKNKVAAPFKQVEFDIMYGEGISKMGELLDMGVKAGIVDKSGSWFSYGDERIGQGRENAKNYLREHEAMAIDIEDKIRAAHGLDFDGSEDMDPDILDGTTRYTPSEIEARWQAAWEKDEIFKAVRSADKPKYYVLEMFPYPSGRIHMGHVRNYTLGDVIARYKLAKGFNVLHPMGWDSFGLAAENAAMQKGIHPGEWTYQNIEDMKNQMKPLGFSLDWSREIATCHPDYYQHQQAMFIDMIDAGLIYRKNAIVNWDPVDMTVLANEQVEQGRGWRSGALVERRELTQWFFKISDYSEELLAGLDTLENWPAKVRLMQENWIGKSRGLQFAFSAINAPDGHDRIEVYTTRPDTLNGASFVGISPDHPIAKLLERDNPEVAAFTAECRKGGTTEEAIETAEKLGFDTGIRVRHPFDTSQELPVYIANFILMDYGTGAIFGCPAHDPRDFEFATKYGLPIISTFLPSEDANEELTEAFVPAKTEKVFFNRGFAGEQWQTGLEAIDAAITFCESQGVGQGVTKYRLRDWGLSRQRYWGCPIPVVHCDDCGVVPEKKENLPVKLPEDVTFDVPGNPLDRHTEWRTTPCPSCGKPAQRETDTMDTFVDSSWYYARFTAPHADTPTVMDDAEYWMNVDQYIGGVEHAILHLLYARFFARAMEITGHLPKSAAEPFDALFTQGMVTHAIYKTAGADGRPVFHYPEEVKGDKAFEGGSEVEIIPSAKMSKSKNNVVDPLSIISAFGADTARWFVLSDSPPERDVEWTASGAEAAYKHLTRVWNICDRIGAMEDGAKGEGDDDLLRQMHKAIHDVTMAIESFGFNAAIAKLYGFTATLQKSKASKAAQREAVMTLAQLMSPMTPHLAEDIWANQGGEGLIAKAPWPVVDESMLKDDNVTLPIQVNGKRRGEINVPAEMPKDEVEKLALAHEAVIRILDGGTPKKVIVVPGRIVNVVVMAITSANQLELLQTAEAVAREKMIDPALVIEAMEESLARAAKSRYGAEMDIRVVIDRKTGRATFTRVRTVVADEELENYQAEFTVEQAKQYMANPEIGQEFIEEVPPVEMGRIAAQSAKQVILQKVREAERDRQYEEFKDRAGTIINGLVKREEYGNVIVDVGAGEAILRRNEKIGRESYRPNDRIRVYIKDVRREQRGPQIFLSRTAPEFMAELFKMEVPEIYDGIIEIKAVARDPGSRAKIAVISYDGSIDPVGACVGMRGSRVQAVVNELQGEKIDIIPWNDDQPTFLVNALQPAEVSKVVLDEEAGKIEVVVPEEQLSLAIGRRGQNVRLASQLTALDIDIMTEEQESARRQAEFELRTKLFMDNLDLDEFFAQLLVSEGFTNLEEVAYVEVDELLVIDGVDEATAGELQARARDVLEAQNKAALDAARALGAEDSLIEFDGLTPQMVEALAKDDVKTLEDFATCADWELAGGWTTVNGERIKDDGVLEPFDMSLEEAQQMIMTARVLLGWVDPTELEPDAVEEEFDAE--TDEEAEAMAHKKAGGSSRNGRDSAGRRLGVKKYGGEAVIPGNIVVRQRGTKFWPGENVGMGKDHTIFATVEGNVQFHKGLKNRTFISIVPVAEAAEMAKRWYSVSVLSNFEKKISEQIRTTVAELEMEDQIDEVLVPTEEVIEIRRGKKVTTERRFMPGYVLVHMEMSDRGYHLINSINRVTGFLGPQGRPMPMRDAEVTAILGRVQEGEEAPRTLIHFEVGEKIKVADGPFEDFDGMVEHVDDENQKLKVMVSIFGRETPVELDFTQVNKQVMSFTLAIVGRPNVGKSTLFNRLVGKRLALVDDQPGVTRDLREGAARLADLRFTVVDTAGLEEVTDDSLQGRMRRLTERAVDMADICLFMVDARVGITPSDLVFADILRKRSAHVILAANKAEGKAADAGVIEAYSLGLGEPIRLSAEHGEGLNDLYTQLMPLADAYAEKAKDDSPEVDVDVSDEDEDEDAVRVPTRARPLQVAVVGRPNAGKSTLINQILKEDRLLTGPEAGITRDAISLTTEWAGPVPMRIFDTAGMRKKAKVQEKLEKLSVSDGLRAVKFAEVVVVLLDAEIPFEQQDLRIADLAEREGRAVVVAVNKWDIEEDRQAKLKELKESFERLLPQLRGAPLITVSAKTGRGLDRLHQAIMRAYEVWNRRVTTAQLNRWLSGMMEAHPPPAPQGKRIKMRYMTQAKTRPPGFVVMCSHPDKVPDSYSRYLVNGLRLDFDMPGTPIRLWMRGQSDANPYKGRKKNAPSKLRKHTAGRRKDMRIVFMGTPDFSVSVLNALVEAGHEIAAVYSQPPRPAGRGKKDRPSPVHARADALGLEVRTPVSLKTPESQAEFAALKADVAVVVAYGLILPQAILDAPARGCLNIHASLLPRWRGAAPIHRAIMAGDAQTGVCIMQMEAGLDTGPVLLRQSLDIGVSETTAQLHDRLSVMGSDLIVEALGKLDDLTAEVQPEEGVTYAAKIDKAEARIDWTLSAKEVDRQIRGLSPFPGAWFEHEGVRIKVLGSTVVDGAAAAGTVLSEALHVACGEGAVALTRLQKAGKGAQDVDVFQRGAQIAVGTQLGKGMSDATAYRVLARKYRPETFADLVGQEAMVRTLKNAFEADRIAQAFIMTGIRGTGKTTTARIIAKGMNCIGADGTSGPTTEPCGVCEHCTAIMEGRHVDVIEMDAASNTGVANIREIIDSVHYRAASARYKVYIIDEVHMLSTGAFNALLKTLEEPPEHVKFIFATTEIRKVPVTVLSRCQRFDLRRIEPEVMIALLRKIATGESAEIADDALALITRAAEGSARDATSLLDQAISHGAGETTALQVRAMLGLADRARVLDLLDMILRGDAAAALTEIGAQYAEGADPMAVLRDLAEITHWISVVKITPDAAEDPTISPEERDRGRMMADALPIRILTRLWQMLLKALEEVGSAPNAMMAAEMAIIRLTHVADLPSPDELVRSLQN-TPVPAPPVGGGGGG------GGQAMQPAQPQMTSAPNGAGQTTALARDPATALAHYPSFEHVLELIRHNRDVKLLVEVETSLQLAAYQPGRIEFVPTETAPRDLAQRLGAKLQLWTGNRWAVTVVNSGGAPTIASIRDAKENAQRDAAKSHPLMQAVVAQFPQAKITAIRTAEDIAAAAVEEALPEVEDEWDPFEDSMPKMKTKSSAKKRFKISATGKVIGSQAGKQHGMIKRSKKFIRNARGTTALSEPDAKIIKGFMPYDRMNLFADIRTLVLSALDAMVAAGDLPSGLSTNNVTVEPPRDAGHGDMATNAAMVLAKPAGMKPRDIAEKLAALLAEDPRVTSAEVAGPGFLNIRLAAPVWQGVAKDVLERGTDYGRGDLGAGKSVNVEYVSANPTGPLHVGHTRGAVFGDALASLLAFAGWDVTREYVINDGGGQIDALARSVFLRYQEAHGQEVAFPDGTYPGDYLKPVGEKLKAQVGAEYLDQPESVWLDPIRNFATDEMMDLIRDDLALLGVKMDRFFSEKSLYNTGKIEACLQKLDDMGLIYNGTLEPPKGKLPDDYEAREQTLFKSTEFGDDQDRAIKKHDGAWTYFAPDIAYHYDKVERGYDALINVFGADHGGYVKRMKAAVHALSGGKVPLDIKLTQLVKLFKNGDEFKMSKRAGNFVTLRDLIDEVGSDVTRFVMLTRKNDAPLDFDFDKVMEQSRENPVFYVQYAHARVASVMRKAEAAGIDVSEDALKAADLSKLDHAAELALLRKVAEWPRLVETAARSNEPHRVAFYLYELAGDLHGFWNLGNAEVGLRFIQEDDPATSQAKIALARAVAIVIAAGLGILGVTPAEEMRMSTIDNLPPLRQVINTHELQARKSLGQNFLLDLNLTAKIARQAGDLTECDVLEIGPGPGGLTRGLLAEGARRVLAIEKDRRCMPALAEIAAAYPDRLQVIEGDALEVDPLAHLTPPIRVAANLPYNIGTELLVRWLTPTDWPPFWQSLTLMFQREVAERIVAQPGSKAYGRLALLAQWRADAKIVLNLPPEAFTPPPKISSAVVHLTALPEPRFPADAAILSRTVAAAFNQRRKMLRAALKGTAPDIEDRLIAAGLKPTDRAEQVPLEGFCALAREIAKPMNLIAEIEAEHIAELAKEIPDFRAGDTIRVGFKVTEGTRTRVQNYEGVCISRKNGKGIAGAFTVRKISFGEGVERVFPLHSTNIDNITVVRRGRVRRAKLYYLRSRRGKSARIVENTNYKPLKG---MANSKRQLFIKRRLRVRNKLRRTNRGRMRLSVHRSNKNISVQLIDDVNGVTVASASSLEKGFGAVGKNNVETAAKVGAAIAERAKKAGVEEAYFDRGGFLFHGKVKALADAAREGGLKIMFAVLKTGGKQYKVQAGDMLRVERIAASAGETVQFNEVLMLGGDNPQVGSPMIEDAGVQAEVVDQIKGEKVIHFVKRRRKHSSKRTKGHRQKLTLVKITDILASGAGKSGVAAAVGTGSVSAAAVAA----KAAKPAKAAK---PAKEAPKAEAKAKKAAKSD-G-DDLSKISGVGPVIVGKLHAEGITTFAQIAAWTDADVEAIEEKLSFKGRVGREDWIAQAKELAKGMSRTKGGTVTHARHRKVVKAAKGYYGRRKSTFKVARQAVDKANQYATRDRKNRKRNFRALWIQRINAAVRMHDEALTYSRFINGLNLAGIEVDRKVLADLAVNEPEAFAAIVKQAQASLAAMQVKETLNEGLKRGYNITLTAAELEAKVTEKLKEAQPDVEMKGFRKGKVPMALLKKQFGQRVMGEAMQEAIDGAMNDHFESSGDRPAMQPDVKMTNEDWKEGDDVEVEMSYEALPAIPELDLSGIELEKLVVKADDASIEEALASLAETAQDFKARKKGSKAKDGDQVVMDFVGKVDGEAFEGGSAEDYPLVLGSNSFIPGFEEQLVGVKAEEEKDVTVSFPDDYQAEHLKGKEAVFACTIKEVKEPVAAEINDEMATKFGAEDLAALKVQIGERLEAEYLGASRAVMKRGLLDALDKMVDFDLPPSLLEAEAGQIAHQLWHEDNPEVEGHDHPEIEATDEHKTLATRRVRLGLLLAELGQKAELEVTDAEMTQAIMNQARQYPGQERQFFEFVQQNQQMQQQMRAPIFEDKVVDHVVEQAKVTEKEVSKDDLQKAVEALEDEMKLHELSDNPGATKPRKRVGRGPGSGTGKMGGRGIKGQKSRSGVAIKGYEGGQMPLYQRLPKRGFNKPNRKSYSAINLGLIQKFVDAGKLDIKSVITEDALVAAGVVRRKRDGIRILAKGDVTAKLNLDVTGASKTAVEAVEKAGGSLTVKAAAATEASEMIQMQTNLDVADNSGARRVQCIKVLGGSKRKYASVGDIIVVSVKEAIPRGRVKKGDVRKAVVVRTAKEVRRDDGTAIRFDRNAAVILNNNNEPVGTRIFGPVVRELRGKNFMKIISLAPEVLMRHARGYRRLNRTHEHRKALWANMAGSLIEHEQIKTTLPKAKELRPIIEKMITLAKRGDLHARRQARARLKEDQYVTKLFDILGPRYKDRQGGYVRVLKAGFRYGDMAPMAIIEFVDRDRDAKGAADKARVAAEEAAE-MLQPKRTKFRKQFKGSIKGLAKGGSDLNFGTYGLKALQPERVTARQIEAARRAMTRHMKRQGRVWIRIFPDVPVTSKPVEVRMGKGKGSVDFWACKVKPGRVMFEIDGVNDDIAREALRLAAMKLPIKTRVIVREDWMTTIKLHNTKTRKREEFVPIDPKNVRMYVCGPTVYDRAHLGNARPVIVFDVLNRLLRHMYGDTHVTYVRNFTDIDDKINARSAASGRPIGDITAETTQWFLDDMAAVGAIEPDHMPRATAFVPQMIALISDLIAKGHAYEAEGHALFAVDSYKEYGALSGRSVDDMIAGARVEVAPYKRNPMDFVLWKPSDADTPGWDSPWGRGRPGWHIECSAMAHELLGAHFDIHGGGNDLMFPHHENEIAQSCCSG--DKFANYWLHNEMLQVEGKKMSKSLGNFFTVRDLLDQGVPGEVIRFVMLSTHYRKPMDWTEKKAKEASRSLKKWRDLVIGIAAAPSIPEAVLATLGDDLNTAGVLALLHEYANNGDYALLKASAQLLGLLTDELGGWTDRGGVLLDGWTERLSAAREEAMATKNFVEVDRIKSLLTDAGVSVQMGKEGIVLAAGPEVDLAKLEASQMAQVKSSSKADPNYKVIAENRRARFDYAIEEDIECGIMLEGSEVKSLRMGGSNIAESYAAVEDGELWLVNSYIAPYKQAKTFGHEERRRRKLLVSRKQLADMWNATQRKGMTLVPMVMYFNHRGKAKIKIGIAKGKKLHDKREDAAKRDWSRQKSRLMKDHGMSAKPEHYDVIRKPIITEKATMASENNAVVFEVAIDSNKPMIKEAVEALFGVKVKAVNTTITKGKVKRFRGQLGTRKDVKKAYVTLEEGNTIDVSTGLMSKDKNPRRVADNEARAKLRMLKTSPQKLNLVAAMIRGKKVDKALTDLTFSKKRIAVDVKKCLQSAIANGENNHNLDVDELIVAEAYVGKNMTLKRGRPRARGRFGKILKPFAEITIVVRQVEEQAMVSAVENMAANSSWSAFGKATDLRHRILFTLGLLIVYRLGTFIPVPGIDGAALREFMESAGQGIGGMVSMFTGGALGRMGIFALGIMPYISASIIVQLLTSMVPSLEQLKKEGDQGRKKINQYTRWGTVALATVQSYGLAVSLEAGDIAADPGMYFRIACMITLVGGTMFLMWLGEQITARGIGNGISLIIFVGIIAEVPAAIAQFFASGRSGAISPAVIVGVLVMVIATIMFVVFMERALRKIHIQYPRRQVGMKVYDGGSSHLPIKVNPAGVIPAIFASSLLLLPVTISTFSGNSTSPVMSWLLANFGPGQPLYLLFFIAMIVFFAYFYTFNVAFKPDDVADNLKNQNGFVPGIRPGKKTAEFLEYVVNRVLVLGSAYLAAVCILPEILRGQFAIPFYFGGTSVLIVVSVTMDTIQQVQSHLLAHQYEGLLEKSQLRGKSGKGRKKRSPARKMKFLDLAKVYIRSGAGGGGCVSFRREKYIEYGGPDGGDGGGGGTVWAEAVDGLNTLIDFRYQQHFFAKNGQPGMGKQRTGKDGDDIVLRVPVGTEILDEDQETVIADMTELGQRVQLARGGNGGWGNLHFKSATNQAPRRSNPGQDGVERTLWLRLKLIADVGLLGLPNAGKSTFLAATSNARPKIADYPFTTLHPNLGVVGVDNTEFVVADIPGLIEGAHDGRGLGHRFLGHVERCAVLLHLIDGTSETITEDYQTIIGELEAYGGELADRPRITVLNKVDALDEEELASALKELTKACGGNVMPMSGVAQTNTVEVLRALRAEIDDDRVRQKPAEEQAPWQPMAAKLKKGDTVIVLAGKDKGKEGTISSVDPKSNKAIVEGVNIAIRATRQSQESQGGRIPKAMPIDLSNLAYKDANGKPTRVGFKMDGENKVRFAKTTGDVI--MLRSGVIAKKMGMTRLFMEDGKQIPVTVLQLDNLQVVAQRTIEKDGYVAVQLGAGTAKAKRTSKAMRGHFAAAKVEPKRKVAEFRVDADAMIAVGEEIVADHYFAGQYVDVAGTSIGKGFAGAMKRHNFGGLRATHGVSISHRSHGSTGQCQDPGKVFKGKKMAGHMGAARVTTMNLQVVKTDTARGLIMVKGAVPGSKGGWVTVKDAVKKPFPENAVVPAALKS------------AAAPAAEAPAETP-----------------------------------AEGGEAMALKSYKPTTPGQRGLVLIDRSELWKGRPVKALTEGLTKHGGRNNTGRITMRRKGGGAKRLYRIVDFKRNKLDVTATVMRIEYDPNRTAFIALVKYEDGEQAYILAPQRIAIGDQVVASAKADIKPGNAMPFSGMPIGTIIHNIEMKPGKGGQIARAAGTYAQFVGRDGGYAQIRLSSGELRLVRQECMATVGAVSNPDNSNQNYGKAGRMRHKGIRPSVRGVVMNPIDHPHGGGEGRTSGGRHPVTPWGKPTKGAKTRNKKKASSSLIIRSRHAKKKGRMLDTATYTPRLKADYKDRIRAALKEEFGYKNDMQIPRLDKIVLNIGCGAAAVRDSKKAKSAQQDLTAIAGQKALTTIAKKSIAGFRVREEMPLGAKVTLRGDRMFEFLDRLITIALPRVRDFRGVNGKSFDGRGNYAMGMKEHIVFPEIDFDKVDETWGMDIVIATTAKTDDEAKAMLKLFNMPFNSMKLDVIKLDGGKAGSVDLDEALFGLEPRADILHRVVRWQRNNAQAGTHKVKTRREVSYSTKKIYRQKGTGGARHGARSAPIFRGGGIYKGPTPRSHGHELTKKFRKLGLRHALSAKAKAGALVIIDDAASDGKTSALAKQVSALGWKRALIIDGASVNENFLQAARNIDGLDILPTMGANVYDILKRDTLVITKAGIEALEARLKMENVVLIIHLILALGLIGVVLLQRSEGGGLGI-GGGGGANSGRPPATPMSKVTWILGAAFVVTSITLTIVSAQKSAGVSVLDRLSASPPALNQGEDLTAPALDNLLPPAEGDNAPLVPTLDMAFFKKLKDRMFKSSSKIDEGLEAIVSDGGEVEDEALAQAKAKAEAERAQARAQAEQEAAQEARRVEAEAAARAAKEDEKRQEKQAEEAAEAEREQAKKEAEERKAA-------TEPLRTTLTPVAPMLDA---PDEPAPKRGILGRLMGRGGAAPVARRVLDDAMLEQLEELLITADMGVDTALRVTANMAEGRLGKKLSVTEIKTLMSDEIARIMEPVAKPLPIYAKKPQVVLVVGVNGSGKTTTIGKLASQFCAAGKKVVIAAGDTFRAAAVEQLQVWGERAGVPVLTAAHGSDPASLAFDAMVKAEEDGADLLMIDTAGRLQNRGDLMEELAKIVRVIRKKDPDAPHNTLLVLDATTGQNAVNQVKVFQDISDVSGLVMTKLDGTAKGGVLVALADKFGLPIHAIGVGEQIDDLQAFDPQDFADALTGLDRMTRDDWGNVKQRLLKTVGQNNYTTWIDPLALGQMEGGIATVHVPTTFFGNYVSQNFSDLILHEMRSFDTSLSRLKFEVAS----KSPAPVTRQTDAIQPPRSTTPSAATSYNAPLEKRFSFDTFVVGKPNELAHAAARRVAEGGPVTFNPLFLYGGVGLGKTHLMHAIAQELQARKPELNVLYLSAEQFMYRFVQALRDRKMMDFKEIFRTVDVLMVDDVQFIAGKDSTQEEFFHTFNALVDQNKQIIISADRAPQDIKDLEERVKSRLQCGLVVDLHPTDYELRLGILQTKVDSHRVSYPDLEMDDGILEFLAHRISTNVRVLEGALTRLFAFASLVGRRIDMDLTQDCLADVLRASERKITVEEIQRKVSDHYNIRLSDMIGPKRLRSFARPRQVAMYLCKHMTSRSLPEIGRRFGGRDHTTVMHGVKRIEELKVSDGQIAEDLELLRRSLEAMLGLGKIAKKVFGTPNDRKIKAVRPIVEKINALEPEFEKLSDEAIKDKTEELATRAMKGESLDALLPEAFANCREGARRTLGLRAFDTQLMGAIFLHQGNVAEQKTGEGKTLTATFAAYLNALTHKGVHIVTVNEYLVKRDAEWMGKVFASLGLTTGYIVPNMPDDLKRHAYECDITYATNNELGFDYLRDNMKAELSEIYQKEHNFAIVDEVDSILIDEARTPLVISGPSDDRSDMYQTIDGVIPSLVPEHYELDEKTRNVTFTDEGIEFLEEQLRARELLEEGFTLYDPESTSIVHHVNQGLRAHKLFEKDKDYIVRNNEVVLIDEFTGRMMAGRRLGDGLHQAIEAKEGVDIKPENITLASVTFQNYFRLYNKLGGMTGTALTEADEFMEIYGLGVVEVPTNVPVSRVDEDDAVYRTAAEKYAAMIEKVKEAHAKGQPCLVGTTSIEKSEMLSKMLDGEGIKHNVLNARLHEQEAQIVGDAGKLGAVTIATNMAGRGTDIQLGGNVELKVQEALSADPEADPIAIRAKIEAEHADEKKQVLESGGLYVLASERHESRRIDNQLRGRSGRQGDPGRSSFFLSLEDDLMRIFGSERLEKVLTSLGLKEGEAIIHPWVNKSLERAQGKVEGRNFDIRKQLLKFDDVMNEQRKVIFGQRRDIMESQDLHEITEDMRHQVIDDLVDQYMPRNTYADQWDTQGFYAAVIEQLGVDVPVIAWCEEDGVDDDIVRERLIEATDKLMEGKAERFGPENMRNIEKHFLLQTIDAKWREHLLTLEHLRSVVGFRGYAQRDPLNEYKNESFQLFEGMLDSLRSDVTQRLSRVEPASEEEQQRMMQQMLEQEQAAAAAADLTVNT---AEAAE-----GFDENDPSTWGNPGRNDLCPCGSGKKFKHCHGELTMKLSAELKAFEKRLGYQFQTPRLLNEAVTHASMSTPNRDDNQRLEFLGDRVLGLVMAEALLNLDTKASEGQLAPRFNALVRKEACADVAREIDIGEVLRLGRSEMLSGGRRKQALLGDAMEAVIAAVYLDGGFDAARALIIRLWGSRTTSVKEDARDAKTSLQEWAQARGLEPPSYVLTKRSGPDHAPIFTIAAKLSTGQTASATAGAKRAAEQDAAAVLLAQLEQEKMAKLGKRTTAAREAFAGKENVTVEEAVALIKGNSNTKFDETIDIAMNLGIDPRHADQMVRGVVGLPNGTGKDVRVAVFARGPKADEATAAGADVVGAEDLMEIVQGGKIDFDRCIATPDMMPIVGRLGKVLGPRNLMPNPKVGTVTMDVEAAVKAAKGGEVQFKAEKGGVVHAGVGKVSFDEAKLVENIRAFVGAVAKAKPAGAKGAYMTKIALSSTMGPGVTVAVDNAVTEMSEDFMLDTDDLERRMLGAIASLRTEFSSLRTGRGSASMLEPVMVDAYGSMTPINQVGTVNVPEPRMVTINVWDKALVGKVEKAIRESGLGINPQLNGTIIMLPIPELNEERRTELTKVAGGYAENARVSIRNIRRDGMDQIKKAKADGMSEDDQKIWESEVQDLTNKQIKAIDDQLEAKQAEIMQVMAKKLVGTMKLQVKAGQANPSPPVGPALGQRGINIMEFCKAFNAKTADMEPGAPCPTVISYYQDKSFTMDIKTPPASYYLKKAAKVNSGAKTPSRETVGSVTTKQLREIAEAKMADLSANDVDQAMKIILGSAKSMGIEVKMFENLSERLSGVFDRLTKQGALSEEDVKTALREVRVALLEADVSLPVARDFVNAVQEKATGQAVTKSITPGQQVVKIVHDALIDVLKGEGEPGALKIDSPPAPILMVGLQGGGKTTTTAKLAKRLKEKDGKRVLMASLDVNRPAAMEQLAILGTQIGVDTLPIVKGEDPVAIAKRTKTQASLGGYDVYMLDTAGRLSIDDELMAQVEAVRDVVTPRETLLVVDGLTGQDAVQTAENFNERIGISGVVLTRMDGDGRGGAALSMRAVTGRPIKYVGLGEKMDALETFEPERIAGRILGMGDIVALVEKAQETIEAEQAEKMMKRMAKGQFNMNDLKMQLEQMLKMGGMQGMMGMMPGMGKMAKQVEEAGFDDRILKQQIALINSMTKKERVNPALLQASRKKRIAKGAGMEVSDLNKLMKMQRQMSDMMKKMGK-GKGGMMKAAMKQMMGKGGMDPAAMAQGMDPKALEAAAKQMGGKLPG----LGGGMGLPPGLSGFGKKK---MDRLAQITARFEYLEAAMSTA--GGDISKLAKEYSDLKPVVDEIAAYRVLLDNLEGAQEMLSDPDMKDLASEEIAEIQAALPAAEASLQLALLPKDEADARPAMLEIRPGTGGDEAALFAADLLRMYQRYADTRGWKVELIEEQATELGGIKEVVAHITGENVFARLKYESGVHRVQRVPSTESGGRIHTSAATVAVLPEAEDVDIQIAPNDIRIDTYRSSGAGGQHVNTTDSAVRITHIPSGIVVTSSEKSQHRNREIAMQVLKTRLYDAERQRIDSERSDSRASQVGSGDRSERIRTYNFPQGRMTDHRINLTLYKLDQVMQGDLDEIVDALTADAQAQMLAEMAQMDRAQKEQLVDELGQIFESSGVVVVSHYVGLTVAEMQDLRARARDAGGSVRVAKNRLAKIALEGKPCASIADLLTGMTVLTYSEDPVAAAKVAQEFSKENDKLVILGGAMGENALDAAGVEAVSKMPSREELISSIAGMLGAPASNIAGAIGAPASNIASILSSIEDKAA-MKTFSATPADIDKKWIIIDAEGIVLGRLASIIAMRLRGKHKPSYTPHMDCGDNVIVINAEKIQMTGKKREEMFYWHTGHPGGIKERSKADILDGKHPERIVTLAVKRMLPGNRLSRQIMTNLRVYAGGEHPHEAQEPTVLDVASMNKKNTRSAMQQADAMLLHAIDVAFGSELPKRVGIAVSGGGDSVALLHLFARWSEQTGHPIAAVTVDHGLRAESRAEAEGVAALCTQLGVSHDIFTWSGPNSGGNVSAAARDARYDLMAKWAKTAKVGGIALGHTMDDTAENMLIRLGRSAGLDGLAQMQTLFRRNGILWSRPLWQQHRSTLRDYLKRQQIEWVDDPTNDDPHYMRTKARRLLPSLKELGIDADSLHHSAFALRQSQEALAHYTRQEARSHVTQEGGDLLLSADFTREVPADIERRLTAAALQWVGSLPYPPRKVFAGFLGRELEAQNRLNLAGCLVQRGKGHIRITREFNAVKDTVTKTNEIWDTRWRLVGPH--DDSLEVRALGE-GVLALPDWRAIGVPRHSLMSSPAVWKGENLVAAPLAGYNLDWSAQIVAD---FTSFLLLHMADLKKLAEDIVGLTLLEAQELKTILKDEYGIEPAAGGAVMMAGPADAGDAA-EKSEFDVVLKNAGASKINVIKEVRGITGLGLKEAKDLVEAGGKIKEGVDKAEAEDIKGKLEAAGAEVELAMSRIGKKPVDLPSGVTASVSGQTIEVKGPKGTRSFKATDDVTMAVEDNAVTVTPRGKSKRARQQWGMSRTMVSNLVAGVTEGFKKELEIQGVGYRAAMTGNTLKLNLGLSHDVDYVAPEGVTVTAPKPTEIVVEGIDEQLVGQVAANIRAWRKPEPYKGKGIRYKGEFVFRKEGKKKMQVILLQRVPKLGQMGDVVDVKPGFARNYLLPQKKALTASKANVEAFEGQKAQLEAQNLETKAEAEAMSEKLNGQQFVVIRSASDAGALYGSVTTRDAAEAATAEGFTVDRKQVVLVAPIKYLGVHDVTVVLHPEVEATIELNVARSPEEAELQAAGKSIQELAAEEEAAAEFEISELFDDIGSAASDDDDLAEAV-------SSDEEAPAMSDSDGRKTLGL-GGSRPSNVKQSFSHGRTKNVVVETKRKRVVVPKPGGQKQTGPGAGPVGDPSKRPAGITDSEMERRLKAVQAAKAREVEEAAARAAEEKARAEERERRRAEIEAKEAADRAREESLKAKAEEEARAKREAEAAAKAAPAPA--PAQAR-TPNKPAPAATPRKSERDREETNKKNRQSDD-RRSGKLTVNQALRGGEGGRQRSMAQMKRKQERARQKAMGGSVEREKVVREVQLPPAIVVSELAARMAEKTGAVVKALMTNGMMVTQNETIDADTAELIIEEFGHKVVRVSDADVEDVINIIEDDEADLQGRPPVITIMGHVDHGKTSLLDAIRNAKVVAGEAGGITQHIGAYQVTTDNGAVLSFLDTPGHAAFTSMRSRGAQVTDIVVLVVAADDAVMPQTIEAIAHAKAAQVPMIVAINKIDKPAADADKVRAALLQHEVIVEKMSGDVQDVEVSAITGQGLDELLEAIALQAEILELKANPDRAAVGAVIEAQLDVGRGPVATVLVQNGTLRQGDIFVVGEQYGKVRALIDDQGNRVKEAGPSVPVEVLGLNGTPEAGDVLNVTETEAQAREIAEYRANAAKDKRAAAGAATTLEQLMANAKADEDVSELPILVKADVQGSAEAIVQAMEKIGNDEVRVRVLHSGVGAITETDVGLAEASNAPIMGFNVRANASARNTANQKGVEIRYYSVIYDLVDDVKAAASGLLSNEIKENFIGYASIKEVFKVTGVGKVAGCLVTEGVARRSAGVRLLRDNVVIHEGTLKTLKRFKDEVPEVQSGQECGMAFENYEDIRPDDVIEIFTREEVARTLSMAKPKKTPRPKAETPRGFRDYFGAEVTQRAEMLAKIAGVYHRYGFDALESAAVEKVEALGKFLPDVDRPNEGVFAWQEDAESEKQGDWLALRYDLTAPLARVYAQHRNDLPTPYRRYAMGPVWRNEKPGPGRYRQFYQCDADTVGAGSVAADAEICAMLSDCLEEVGIARGDYVVRVNNRKVLNGVLEVAGLSGDDKEAERGIVLRAIDKLDRLGPDGVRALLGEGRKDDSGDFTDGAGLGDAQADVVMGFMQAKRGDGAATCARLRELVGDSAVGIEGVSELETIAELLAAGGYGPDRIEIDPSVVRGLGYYTGPVYEAELTFEIKDDKGRARNFGSVAGGGRYDDLVKRFTGQEVPATGVSIGVDRLLAALHAKGRMDTTSQGPVVVTVMDKARMADYQEMVAELRQAGIRAEVYLGNPKNFGNQLKYADKRGSPIAVIEGGEEHERGVIQIKDLILGAKMAEGATLEEWRDRPSQYEVARGDLVAKVREILELYSMDDLKQKYLSQIADAGDEAGLEDIRLAAVGKKGEVALKMRELGKMTPEERQVAGPALNALKDEINSALAAKKAALGDAALNERLRTEWLDVTLPSRERPAGTLHPVSQVTEELTAIFAELGFSVAEGPRIDTDWYNFDALNIPGHHPARAEMDTFYMSRAEGDERPPHVLRTHTSPVQIRTMEAEGAPLRIICPGGVYRADYDQTHTPMFHQVEGLAIDKDISMANLKWTLEEFFSAFFEIDGIKTRFRASHFPFTEPSAEVDIQCSWVDGQLRIGEGDGWMEVLGSGMVHPKVLAAGGIDPDVWQGFAFGMGIDRIAMLKYGIPDLRAFFDSDLRWLRHYGFASLDQPNLHGGLSRMDMADEAGLDLVEISPNANPPVCKIMDFGKFKYETQKREAEARKKQKIIEIKEVKFRPNTDTNDYDVKMRNVYKFLENGDKVKITLRFRGREMAHQNLGRELLERVAEDTKEHGKVENFPKMEGRQMVMLIGPLPSMKFTLSWLKEHLDTTASVDEITYALTDLGLEVEGVEDRGARLRDFTLGFVKSAEKHPDADRLRVCQVETDEGLQQIICGAPNAREGITVVIAKPGVYVPGIDTTIGVGKIRGIESFGMMASERELELSEEHDGIIELPSGNVGDSFVDWLAENDPSKVDPVIEIAITPNRPDALGVRGIARDLAARGLGTLKARDVDPVEGTFPCPVTVTIDEDTLDQCPVFYGRVIRGVKNGPSPAWLQDALRAIGLRPISFLVDVTNFFTYDRNRPLHVFDADKIAGNLRVHRAKGGETLVGLDEKEYTFAADMTLISDAEGVESIGGVMGGLPSGVTEETVNVFLEAAYFDTVRTAYTGRALKINSDARYRFERGIDPEWTPKGIDHATQMILDHAGGEASEVVVAGKIPDTSRAYRLNAKRVISLVGMDIPEATQRQSLTALGFRLEGDMAHVPSWRPDVQGEADLVEEVARIASLTKLEGVPLPRLTTGVPRPIMSPTQRREVAARRTAAALGYHECVTYSFIDQPSAALFGGGTDETRLENPISNDMSHMRPALLPGLLQAAARNQARGFADMALFEVGPAFHGGEPGEQHMLVSGLLVGRTGPKDVLGAARDVDVFDVKADAEAILAAIGAPAKVQILRGAADWWHPGRHGKICLGPKKVLGIFGEVHPKVLTEMGVKGPAMAFTIWPGEVPLKRKSGATRPALKVSDLQAVERDFAFVVDADVEALTLVNAAMGADKALIQDVRVFDEFIGGSLGEGKKSLALTVRLQPTDKTLKDADIEAVGAKVIEKITKATGGVLRGMHAYRSQTCADLNATNVGDNIRLSGWVHRVRDHGGVLFIDLRDHFGMTQLICDPDSAAFAEVEKVRSEWCIRIDGEVKARAADLVNSKIPTGEIEVFVRDIEVLGRVNDLPLQVFGEQEYPEETRLRYRYLDLRREKMQRNMVLRSDVVSSIRKRMWDQEFKEFQTPIITASSPEGARDFLVPSRLHPGKFYALPQAPQQFKQLLMVSGFDKYFQIAPCFRDEDPRADRSPTDFYQLDLEMSFVEQQDVFDTIQPVLTGIFEEFGGGKSVDQDWPQISYKDAALWYGSDKPDLRNPIKMQVVSEHFAGSGFAIFAKLLEQEGTEIRAIPAPKGGSRKFCDRMNAFAQKEGLPGMGYIFWRDQ-GE--GMEAAGPLAKNIGPERTEAIRQQLGLGVGDAAFFLGGKPKAFESVAGRARNVIGEELGLTDMDRFAFAWIVDFPIYEKDEETGKVDFEHNPFSMPQGGMEALEGDPLEVLGYQYDLACNGYELVSGAIRNHRPEIMFKAFEIAGYGEEEVRKRFGGMVNAFQYGAPPHGGCAAGIDRIVMLLAEESNIREVILFPMNQRAEDLMMNAPNDPMPDQLMELGLRVIPQDMTPLSHIRNFSIVAHIDHGKSTLADRLIQETNTVAERDMKAQMLDNMDIERERGITIKAQTVRINYTALNGEEYILNLIDTPGHVDFAYEVSRSMRAVEGSLLVVDSTQGVEAQTLANVYHAIDADHEIVPVLNKIDLPATDCDRVAEQIEDVIGIDASDAIRVSAKTGEGIIETLEAVVHKLPAPTGTLDAPLKAMLVDSWYDSYLGVIVLVRIMDGQLKKGERIKMLSNGSTHHVDRIGVFRPEMTEIDALGPGELGFLTASIKQVRDTSVGDTITHEKKGCTEALPGFKPSQPVVFCGLFPVDAAQFEDLRDSIEKLALNDASFSYEMETSAALGFGFRCGFLGLLHLEVIRDRIEREYDIDLITTAPSVIYDIHMRDGTVEQLHNPADMPDLTFVDHIEEPRIKATILVPDDYLGDVLKLCQDRRGIQEDLTYAGSRAMVVYDLPLNEVVFDFYDRLKSVTKGYASFDYQMIGYREDSLVKMSILVNDEPVDALSTMVHRDRAEMRGRAMVEKLKDLIPRHMFKIPIQAAIGGKVIARETLSAMRKDVTAKCYGGDASRKRKLLDKQKAGKKKMRQFGKVDIPQEAFISALKMDNMSLPPGFLDELRTRSSLSQVVGRKVMWDARKSNQGKGDMWAPCPFHQEKSASFHVDDSKGFYYCFGCHAKGDAISFVRETENVSFMEAVEILAREAGMPMPERDPRAQQKADKRTELADVMELAVKWFRLQLRTGAASAARDYLDRRGLPQKVCDRWEIGFAPDSWQGLWDALKGKNIPDELILGAGLAKPSTKGGKPYDTFRGRIMYPIRDARGRAIAFGGRAMDPDDKAKYLNSPETELFDKGRSLYNVKDARVAAGKGKPLLVAEGYMDVIALSEAGFEAAVAPLGTAITENQLAMLWRISDEPIITLDGDAAGQRAALRLIDLSLPLLEAGRSLRFAMMPEGQDPDDLLKSSGAGAVQSLLDAAVPMVNLLWQRETEGKVFDSPERKAALDKALREKIMLIKDPSIRSHYGQAIKDLRWELFRSR----KGAAQFQRR---AKWGKAPQGPSAGAKSSVLAQAGSDSATDHLREAVILAACISCPEVVEHFDSGLESMACVDNAHARLRDLILRHAHEGAEVLRSNIDDMMGYDTLENLMSQRHVAITPCIRKPGDVEITTMTITEELAKLTAVRGLLDEVSDAADDLTGEADEGVTWRLAEAARAADLAQRSGQEDRAEYVIADNGARLDREEVSKSRDFFEKIDFSKGGKKSMCAETP-YKTTLNLPKTDFPMRAGLPKREPVWLERWEKMGVYNQLREKAPRKPFTLHDGPPYANGHLHIGHALNKTIKDMIVRSHQMMGNDARYIPGWDCHGLPIEWKIEEQYRKKGRDKDQVPINEFRAECRSFAAGWVDIQREEFKRLGVQGNWEKPYLTMDFHAERVIAEEFMKFLTNGTLYQGSKPVMWSPVEQTALAEAEVEYHDKDSFTIWVKFDVV----GAADLEGAQVVIWTTTPWTIPSNKAVVYGAGISYGLYEVTGTPDECWLNVGDRYLLADNLAADVMGRGRLEEDQWTRVRDVTNEELETISLKHPFNGVEGGEGEWDDIRDFRAAEFVTDTEGTGFVHCAPSHGLEEYDLYRELGMLEQVITYNVNPDGRYRDDLPFFGGKAILKPNGKEGNANAAVIDKLVEVGGLLARGKIKHSYPHSWRSKAPVIYRNTPQWFAAIDRPVGDGQDTFGKTIRERALTEIDNVKWTPKSGRNRLHAMMEARPDWVLSRQRAWGVPLTCFTLKGKLPTDAEFLLRNDAVNKRIVEAFEAEGADAWYEEGAKERFLGGIVNPDDYDQVMDILDVWFDSGSTHAFTLRDREDGSDDGIADVYMEGTDQHRGWFHSSLLQSCGTYGRAPYRNVVTHGFTLDAKGMKMSKSIGNTIVPEKIIQQYGADILRLWVAQTDYTSDQRIGDEILKGTADSYRRLRNTMRYMLGSLPDFDASKAVAAEDMPELERLILHKLSLLDEVVRDGYARFDFQGVFRAIFDFATLDLSSFYFDIRKDALYCDGDTLRRLSALTVLDHLYSRLTTWLAPILPFTMEEVWLQRN-GEDTSVHLIDFPETPSTWRDDALAARAETVRAVRRVVTGCLEEQRTAKVIGSSLEAAPVVYLN-----AELAAIIFADLCITSQIKLVEGEGPADAFTLDDVKGVSVVFAKAEGQKCARSWKILPDVGTYSFPGVSKRCDEALRMAASQNIRIRLKAFDYRVLDASTQEIVNTAKRTGASVRGPIPLPNKIEKFTVLRGPHVDKKSRDQFEIRTHKRMLDIVDPTPQTVDALMKLDLAAGVDVEIKLQSMSDEINTLEDL-----ASVAGVEATPEVELTPREPVRDEFGRSYATGKRKDAVARVWIKPGSGKVIVNGKAQNDYFARPVLQMVLAQPFSVTGTEGQFDVYATVKGGGLSGQAGAVKHGVSKALQLYDPSHRGALKAAGFLTRDSRVVERKKYGKAKARKSFQFSKRMPTIQQLIRKPRQPKRKVSKSMHLQQCPQKRGVCTRVYTTTPKKPNSAMRKVAKVRLTNGFEVISYIGGESHNLQEHSVVLIRGGRVKDLPGVRYHIVRGVLDTQGVKDRKQRRSKYGAKRPKMAREAKRTKKKVSKNIAAGVAHVNSSFNNTKILISDVQGNAISWSSAGTMGFKGSRKSTPYAAQMAAEDAGKKAQDHGVKTLEVEVQGPGSGRESALRALAAAGFNITSIRDVTPMAHNGCRPPKRRRVMT-NRRGLLIILSSPSGAGKSTLAKRLMKWDDTLSFSVSATTRKPREGEVDGTDYRFTTEETFRKWVVENEMLEHAHVFGNFYGSPKGPVEKSINEGRDVLFDIDWQGAQQIRNSPLGLYTLSIFILPPSISELHRRLVSRGQDDSETISKRMQKSWDEISHWDGYDYVLVNDDLDSTEEKLKTIIAAERLKRTQQPQLSEIARDLQSQFEDLVMAQSFLGQKRLRKYYGKIREVLDMPNLIEVQKSSYDLFLNSGDAETPTDGDGIQGVFQSVFPIKDFNETSILEYVKYELEKPKYDVEECQQRDMTYSAPLKVTLRLIVFDIDEDTGAKSVKDIKEQDVFMGDMPLMTPNGTFVVNGTERVIVSQMHRSPGVFFDHDKGKTHSSGKLLFACRIIPYRGSWLDFEFDAKDIVFARIDRRRKLPVTTLLYSLGLDQEAIMDAYYNTVTYRLDAGKGWIAPFFPERVRGTRPTYDIVDAASGEVLFEAGKKVTPRAVKKLIDEGNVTELLLPYDHIQGTFVAKDIINEETGAIYVEAGDEMTLEYDKDGTLIGGTAKELIDAGITEIPLLDIDNVNVGPYMRNTMAADKNMNRETALMDIYRVMRPGEPPTVEAASSLFDTLFFDSERYDLSAVGRVKMNMRLALDKEDTQRTLDRDDIVSCIKALVDLRDGRGDIDDIDHLGNRRVRSVGELMENQYRVGLLRMERAIKERMSSVEIDTVMPQDLINAKPAAAAVREFFGSSQLSQFMDQTNPLSEVTHKRRLSALGPGGLTRERAGFEVRDVHPTHYGRMCPIETPEGPNIGLINSLATFARVNKYGFIETPYRKVTDGVVSDDVQYMSATEEMRHTVAQANANLDENMKFVNELVSTRKSGDYTLSPSSNVDLIDVSPKQLVSVAASLIPFLENDDANRALMGSNMQRQAVPLLQAEAPLVGTGIEEVVARDSGAAYMARRAGVIDQVDATRIVIRATEDLELGDAGVDIYRMRKFQRSNQNTCINQRPLVKVGDKVTKGQVIADGPSTDMGELALGKNVVVAFMPWNGYNYEDSILISERVSRDDVFTSIHIEEFEVAARDTKLGPEEITRDIPNVGEEALRNLDEAGIVYIGADVEPGDILVGKITPKGESPMTPEEKLLRAIFGEKASDVRDTSLRVKPGDFGTVVEVRVFNRHGVEKDERALQIEREEVERLARDRDDEMAILDRNIFARLREMILGKVAVKGPRGVKPNSQITEETLMVLTRGQWWQLALEDEDDAKIVEALNEQYEIQKRTLDARFEDKVEKVRRGDDLPPGVMKMVKVFVAVKRKLQPGDKMAGRHGNKGVISKVVPMEDMPFLADGTPVDFCLNPLGVPSRMNVGQILETHMGWAARGLGINVDEALQEYKRSGDLTPVREAMQLAYGDDVYEEGLTGLDEEELLEVAGNVRRGVPIATPVFDGAKEADVNDSLARAGFDRSGQSVLFDGRTGEQFSRKVTVGVKYLLKLHHLVDDKIHARSTGPYSLVTQQPLGGKAQFGGQRFGEMEVWALEAYGAAYTLQEMLTVKSDDVAGRTKVYESIVKGEDNFEAGIPESFNVLVKEVRGLGLNMELLDAEEDEMPLYEHVMIARQDLSNTQAEGLIEHFGTVLSDNGGSLVDSEYWGVKTMAYKINKNRKGHYAFLRSDAPAPAVQEMERLMRLHDDVMRVLTIKVDEHKDLPSVQMQKRDERPERRERRNIPELDVDVLTETQAASELARLSVLLAEANTAYHAEDAPQISDAAFDALRRRNAEIEARFPHLKRADSPSDTVGAAPASGFGKITHSVSMLSLANAFSDEDVGEFDTSIRKYLGLGAEAPLSYTAEPKIDGLSLSLRYEKGILTQAATRGDGSVGENVTANAKTVSDIPHHLEGTPDVLEVRGEVYMSHADFEALNARQEERGGKTFANPRNAAAGSLRQLDAEITRSRPLRFFAYAWGSLSEPLGVTQWQAIEKLKDLGFSTNPLTARCDGPSQLVKHYRQIEEQRPDLGYDIDGVVYKVDELELQARLGFRSTTPRWAIAHKFPAELAWTRLEGIDIQVGRTGALSPVARLAPVTVGGVVVSNATLHNEDYIKGLDSKGQEIRGGKDIRVGDLVQVYRAGDVIPKIADVDLGKRPDDAIAFAFPTACPECGSDAVREPGDAVRRCTGGLICPAQAVEKLKHFVSRGAFDIDGLGAKQVEQFYTDGWISEPAEIFTLKERFGTGMQQLKNREGWGDKSASGLFAAIDQKRKIPLARLLFGLGIRHVGEAASNMIALHYGTWDALAVAMDEARPMEGAAWDDLISIDGMGAVMAGSLVGAFAQEAERASIQRLIEQLDVQEAKQADTSGSPVAGKTVVFTGTLEKMTRAEAKARAERLGAKVSGSVSAKTDLLVAGPGAGSKAKKAAELGIETLDEDGWLELIDGLMIHKNWAELIKPTQLDVKPGNDPARQATVIAEPLERGFGLTMGNALRRVLMSSLQGAAITSVQIDNVLHEFSSVAGVREDVTDIILNLKGVSIRMEVEGPKRLSISAKGPGVVTAGDISESAGIEILNRDHVICHLDDGADIYMELMVNTGKGYVSADKNKPEDAPIGLIPIDAIYSPVKKVSYDVQPTREGQVLDYDKLTMKVETDGSLTPDDAVAFAARILQDQLGIFVNFDEPESASRQDDDDGLEFNPLLLKKVDELELSVRSANCLKNDNIVYIGDLIQKTEAEMLRTPNFGRKSLNEIKEVLSGMGLHLGMDVEDWPPDNIEDLAKKFEDNFMARDENRGGNRRNQRDEAPEFADRLVAINRVSKTVKGGKRFGFAALVVVGDQKGRVGFGKGKAKEVPEAIRKATEQAKRQMIRVQLREGRTLHHDMNGRHGAGKVVMRTAPEGTGIIAGGPMRAVFEMLGVKDVVSKSIGSQNPYNMIRATMDGLKKEQSPRSVAQRRGKKVADILPKREDASDSSAQVAEEAMTDPIADMLTRIRNSQLRGKSTVMTPASKLRAWVLDVLADEGYIRGYEKMTGADGHPAIEISLKYYEGEPVIRELKRVSKPGRRVYMGSQDIPAVRQGLGVSIVSTPQGVMSDAKARAANVGGEVLCTVFMHDIRAIRENPDAFDAALARRGDDALSSSILELDTARRSKIQAAEAAQADQNAASKQVGAAKAKGDEAEFESLRALVSEKKAEVAAMQTEAKELDAKLTDVLARIPNIVADDVPQGADEDENVEVKRWGDVPEFAFAPREHYDLDSVAASMDFETAAKTSGARFVMLKRGVARIHRALAQFMLDTHVDENGLTEVNSPVLVRDEAMYGTDKLPKFGEDSYKTEEGMWLVPTSEVPLTYTVAGDTLEASDLPRRMTSHTLCFRSEAGSAGRDTSGMLRQHQFEKVEMVSITLPEKSDEEQKRMLSCAEGILEALGVPYRTVLLCTGDTGFGARRTFDIEAWLPGQNAYREISSVSTTGDFQARRMNARFKPEGGGKPQFVHTLNGSGLAVGRCLIAVLENGQQEDGTVVLPAVLGPYLGGKTTLSLDGTLKMAITASMVKELRDATGAGMMDAKKALTETAGDMEAAVDWLRTKGLAKAAKKSGRTAAEGLVAVKVEGGRGVAVEVNSETDFVGKNADFQKMVAGIADVAVSAADVDALKAADMGGKSVEQTVTDAVAVIGENMSVRRMSSIEGDVVVSYVHNAAAPGMGKIGVLVAMSGGDDAFGKQVAMHIAAVNPASLSEADLDASVVEKEKQVQIDIARESGKPEAVIEKMIVGRMKKYMSEVTLLNQQFVVNPDLTVADAAKEAGATITGFVRLEVGEGIEVVKEDFAAEVAKAAQGMNQELTNNPFNPVAPMKTFDEIKVSLASPERILSWSFGEIKKPETINYRTFKPERDGLFCARIFGPIKDYECLCGKYKRMKYRGVVCEKCGVEVTLQKVRRERMGHIELASPVAHIWFLKSLPSRIGLMLDMTLRDLERVLYFENYVVIEPGLTDLTYGQMLTEEEYMDAQDAYGMDAFTANIGAEAIREMLAAIDLEAEADQLREELKEATGELKPKKIIKRLKVVESFLESGNRPEWMIMTVIPVIPPELRPLVPLDGGRFATSDLNDLYRRVINRNNRLKRLIELRAPDIIVRNEKRMLQESVDALFDNGRRGRVITGANKRPLKSLSDMLKGKQGRFRQNLLGKRVDFSGRSVIVTGPELKLHQCGLPKKMALELFKPFIYSRLEAKGLSSTVKQAKKLVEKERPEVWDILDEVIREHPVMLNRAPTLHRLGIQAFEPVLIEGKAIQLHPLVCSAFNADFDGDQMAVHVPLSLEAQLEARVLMMSTNNVLSPANGAPIIVPSQDMILGLYYVTLEREGMVGEGKVFGNVDEVQHALDSGEVHLHAKIKARIKQIDNEGNEVLVRFDTTPGRVLLGALLPLNAKAPFDLVNRLLRKKEVQQVIDTVYRYCGQKESVIFCDQIMTMGFRQAFKAGISFGKDDMLIPETKWTLVDETREQVKDFEQQYMDGLITQGEKYNKVVDAWSKCNDKVTDAMMGSISDTTYAENGSENEPNSVYMMAHSGARGSVTQMKQLGGMRGLMAKPNGDIIETPIISNFKEGLTVLEYFNSTHGARKGLSDTALKTANSGYLTRRLVDVAQDCIVRMHDCGTDASITATAAVNDGEVVSSLAERLLGRVVAEDILRPGTDEVLAAAGSIVDERMSDLIDEAGVASARIRSPLTCEAEEGVCAQCYGRDLARGTLVNQGEAVGIIAAQSIGEPGTQLTMRTFHIGGVAQGGQQSFQEAGQEGKIQFENAMTLQNSADETMVMGRNMKLLIIDENGDERASHKVGYGTKLFVKEGQDVARGDKLFEWDPYTLPIIAEAAGTAKTVDLISGISVKDETDDATGMTQKIVIDWRSAAKGNELKPEIILQDGNGEPLRNSAGNPITYPMSVDAILSVEDGAEVAAGDVIARIPREGAKTKDITGGLPRVAELFEARRPKDHAIIAEIDGYVRYGKDYKNKRRIAIESSEDPDHKVEYMVPKGKHIPVAEGDFVQKGDYIMDGNPAPHDILAIMGVEALADYMIDEVQEVYRLQGVKINDKHIEVIVRQMLQKWEVQESGDTTLLKGEHVDKLEFDQANEKALSKGGRVAKGEPILLGITKASLQTRSFISAASFQETTRVLTEASVQGKRDKLVGLKENVIVGRLIPAGTGGATQQMRRVAHDRDNVVVEARRIEAEKAAALAAPTAPADDIVGGDVFDNAPSEEESRDMSRRHAAEKREVLPDAKYGDLILTKFMNNLMIDGKKAVAERIVYNAFDRVEAKIKRAPVEVFHEALENIQPSVEVRSRRVGGATYQVPVEVRPERRQALAIRWLIKAARSRNENTMEERLAGELMDAVQSRGTAVKKREDTHKMADANKAFSHYRWMPYAQTDKSEVTEILSNAAPDVRNRPKLEGGKKFVLQTEFEPAGDQPQAIKELSEGIRSGERDQVLLGATGTGKTFTMAKMIEETQRPSIILAPNKTLAAQLYGEFKGFFPDNAVEYFVSFYDYYQPEAYVARSDTFIEKESQINDQIDRMRHSATRALLERDDVVIVASVSCIYGIGSVETYGAMTQDLKVGESYDQRQVIADLVAQAYKRNDAAFQRGTFRVRGDSLEIFPAHLDDRAWKLSFFGEELESITEFDPLTGEKTDTMDQVRVYANSHYVTPKPTMNQALVGIKKELRSRLDQLVAEGKLLEAQRLEQRTNFDLEMLEATGVCNGIENYSRYLTGRAPGEPPPTLFEFIPDNAIVFADESHVSVPQIGGMYKGDFRRKMTLSEHGFRLPSCMDNRPLKFEEWDAMRPQSVFVSATPADWELERAGGVFTEQIIRPTGLIDPEIEIRPVDMQVDDLLDEVRKVTANGMRTLCTTLTKRMAEDLTEYMHEQGIRVRYMHSDIDTIERIEILRDLRLGAFDVLIGINLLREGLDIPECGLVAILDADKEGFLRSETSLIQTIGRAARNAEGRVIMYADRITGSMERAMGETERRRAKQIAYNEEHGITPMTVKKNVEDILAGLYKGDTDQSRVTAKIDSKLAGGNMQAVLDGLRADMRKAAENLEFEEAARLRDEVKRLEAVDLTIADDPLARQYAVDKAVDDAQKKSGRSTMGRGGMRGGVKRR-GRMAAKPFFRRRKVCPFSGDNAPAIDYKDTRLLQRYISERGKIVPSRITAVSAKKQRELARAIKRARFLALLPYAVKMPKRILTGTVTSDANEQTVTVSVERRFTHPVLKKTIRKSKKYRAHDENNTFKVGQQVRIIECAPRSKTKRWEVITAEAMANTAQAKKRARQNEKRFAINKARRSRIRTFIRKVEEAIESGVKEDAVAALKAAQPELMRGVTKGVYHKNTASRKMSRLAARVKAIAMSRSVWKGPFVDSYVLKKAEASRESGRNEVIKIWSRRSTILPQFVGLTFGVYNGHKHIPVNVSEEMIGQKFGEYSPTRTYYGHAADKKAKRKMARIAGVNIPTAKRVPIALTYITGIGTTSAEKICEAVGIEMTRRVNELSDTEVVKIREHIDENYTVEGDLRRDTQMNIKRLMDLGCYRGLRHRRNLPVRGQRTHTNARTRKGPAKAIAGKKKMAMKIRLARGGSKKRPFYRIVAADSRMPRDGRFIEKLGTYSPLLPKDSEDRVKMNMERVEYWLGQGAQPTDRIQRMLEAAGARPKTERNNPKKGTPGKKAQERAQEKADKAAAAAEAAAAPAEEAAAEEMSITKEEKARVMKEFGAKDGDTGSPEVQVAVLSSRIATLTEHFKTHKKDNHGRRGLLKMVATRRKLLDYVKSKDEARYQDLIKRLGLRRMQTLNEIRSTFLNYFGDNGHAIVPSSPLVPRNDPTLMFTAAGMVQFKNLFTGVETRDYSRATTAQKCVRAGGKHNDLDNVGYTARHHTFFEMLGNFSFGDYFKEDAIPLAWDLLTKGLGIDESRLLVTVYHTDDEAVEIWKKHAGLSDDRIIRIATDDNFWSAGPTGPCGPCTEIFYDHGDHIWGGPPGSPEEDGDRFVEIWNLVFMQYEQFEDGTRQDLPNKSIDTGMGIERVAALLQGTNDNYSTDLMRSLIEASANATSSDPDGPGKTHHRVIADHLRSTSFLMADGVMPSNDGRGYVLRRIMRRAMRHAHLLGVQDPLMHRLVPALVTQMGQAYPELGQAQSMIEQTLLQEETRFRQTLDRGLKLLDDELIQLPEGADLPGAAAFKLYDTFGFPLDLTQDALREKGRAVDTDGFDAAMAEQKAKARAAWSGSGEAADAAIWFDVADKNGVTDFLGYDTESAEGKIVALVQGGKAVDEVGEGQEVQIALNQTPFYAESGGQLGDTGVIKTDSATIRVTDTRKAAGVFIHIATVEKGSVSNAQAAVLEVDHARRTAIRANHSATHLLHEALRHALGDHVAQRGSLNAQDRLRFDFSHNEAVSAEEMAKVETEVNQYIRQNSPVETRIMTPDDARALGAQALFGEKYGDEVRVVSMGQQDGSGKGIEKDTYSLELCGGTHVERTGDIGAFVLLGDSASSAGVRRIEALTGEAAMAHLRAQDQLLAQTALELKAPAAAVPERVRALMDERRSLANEVAQLRRELAMSG--GTNAPEAQEVNGISFHAQVLSGVTGKDLPALVDEHKTRLGSGAVLLIADTGGKAAVAAGVTDDLKGDLSAVDILRAAVAELGGKGGGGRPDMAQGGGASAENADAAIAAAKTVLEGMGFKMGIVGLPNVGKSTLFNALTRTAAAQAANFPFCTIEPNVGEVAVPDARLDQLAEIAQSKSIIPTRMTFVDIAGLVKGASKGEGLGNQFLANIRETDAIAHVLRCFEDDDVTHVEDRVNPVADAETIETELMLADLESIEKRRAGLVRKIKGNDKEAVQQDRLLAAAQDAIENGKPARTVEIDEDDAKAWRMLQLLTTKPVLYVCNVGESEASEGNDHSAAVAKMAAAQGNSAVIISAQIEEEISQLEDEEAEMFLTEMGLDEAGLDRLIRAGYELLHLETYFTVGPKEARAWTIKAGTSAPKAAGVIHGDFEKGFIRAETIAFDDFVTLGGEGPAKEAGKMRAEGKGYTVKDGDVLHFLFNTMGWKSLDDMDLHGKRVLVRVDINVPVENGKVTDATRIERIVPTVHDILAKGGTPILLAHFGRPKGKVNLDMSLRQVLPALEKALMRTVALVETLDAAESLTAEAAVAEVVLIENIRFHPGEEANDPEFAARLAKLGDIYCNDAFSAAHRAHASTEGVARHLPSCAGRLMQAELSALEAALSKPERPVGAVVGGAKVSTKIALLENLVNRLDLLVIGGGMANTFLAAQGAKMGASLSEPDYFDTARDIMAQAAKAGCRVLLPVDGLVAREFKAGAEHEVALLGPDTVLGDDQMVLDAGPETVSQVEIAFAGLKTLIWNGPMGAFEIEPFDTATVAAAKAAADHTRQGTLISVAGGGDTVAALNQAGAADDFTYISTAGGAFLEWMEGKTLPGVAALGGMALPEFSMRQLLEAGVHFGHQTQRWNPRMGPYIYGSRNGIHIMDLTQTVPMLDEALKVIRDTVAKGGSILFVGTKRQAAQPIADAAEKCAQYYMNHRWLGGTLTNWQTVSKSIQRLKHIDEQSELGFSGLTKKERLGMERDQGKLQASLGGIREMGGRPDLIFVIDVRKEQLAIAEANKLGIPVVAVVDTNCSPDGIDYIIPGNDDAARAIALYTDLAARAALDGMSAQLGAAGVDLGAMEEAPVEEAV-------AEEAAAEAPAES---MTKRTAAKHKIDRRMGENIWGRPKSPVNRREYGPGQHGQRRKGKISDFGIQLRAKQKLKGYYGDLTEKQFRRIYGEAERVKGDTGENLIGLLERRLDAVVYRAKFVSTVFAARQFVNHRHVRVNGKLVNIPSYRVKEGDVIEVRDRSKQMVALVEATQLPERDVPDYMEVDHSKMTATFVRTPGLSDVPYPVVMEPNLVVEFYAKNMGNKVNPIGMRLQVNRTWDSRWYADTKDYGDLLLEDLAIRDFIKKECHQAGISRVIIERPHKKCRVTIHTARPGVIIGKKGADIEGLRKKLAAFTDSELHLNIVEVRKPELDAALVGESIAQQLERRVSFRRAMKRAVQNAMRMGALGIRVNLAGRLGGAEIARTEWYREGRVPLHTLRADIDYAHVEAMTAYGIIGIKTWIFKGEIMEHDPAARDRKSQEMQDGPAPRGAGGRRMARKRKGRDISGWVIIDKPAGPTSTAVVNKVRWAFDARKAGHAGTLDPDATGVLAVALGEATKTVPFITDALKAYEFTVRLGVSTNTDDAEGEVLETTDIRPDDAAIKEALNQFVGDIEQVPPQFSAVKIDGERAYKRARDGETMEIAARPLFVESLILMDRPDADHVTLEMVCGKGGYVRSIARDLGEVLGCKGHVRELRRTWSGPFDVANAITLDQVEELATTPALDEFIGPLELGLQELPQVTASSDGAIKLRNGNPGMVFANDVEYGDECWASHEGTAVAVGIFKSGELHPSRVFNLPAMFLLGLTGSIGMGKSTTAKMFQELGCAVWDADAAVHRLYSKNGLAVEPFSKVFPESFVNGEVSRPALKQIIQSDPSALKRIETIVHPLVAKDRADFLADVKSDIVVLDIPLLFETGGDAAMDATACVFVDDETQEARVLERGTMTRDQFLAIKAKQMPAAEKCAKSTYVIQTDTLEHATSQVQKVVSTIRSQLNA-LQDLDILIEAGAWDAATLEPLAQRAIAATLTHMQVDPQACEMSLLACDDARIAVLNAEFRDKPTATNVLSWPAQPLAPEAEGQAPPAPQTGFDGMIELGDIALSFDTCKREAAESGKQMDDHLTHLIVHGVLHLLGYDHISDGDAALMEGLEVEILGNLEIDDPYNSDTTMARYIFITGGVVSSLGKGLASAALGALLQARGFSVRLRKLDPYLNVDPGTMSPFEHGEVFVTDDGAETDLDLGHYERFTGVAARKTDSVSSGRVYSTVLEKERRGDYLGKTIQVIPHVTNEIKDFLAVGEDEVDFMLCEIGGTVGDIEGLPFFEAIRQFSHDKPRGQCIFMHLTLLPYLAASGELKTKPTQHSVKELQSIGIAPDILVCRSEQPIPEKEREKIALFCNVRKESVVAAYDLKSIYEAPLAYHDQGLDQAVLDAFDISPAPRPDLAVWHDVYDRIHNTDGEVKVAIVGKYTQLGDAYKSIAEALTHGGMANRVRVNVEWVDAEVFDSDDVAPHLEGYHAILVPGGFGERGTEGKIKAAQYAREHNVPYLGICLGMQMAVIEAARNVAGLKTAGSEEFDHEAGRKRFEPVVYHLKEWVQGNHKVERKVGDDKGGTMRLGAYDATLKEGSKVAEVYGATSIDERHRHRYEVDIAYREQLEKAGLTFSGMSPDGKLPEIVEWSDHPWFIGVQFHPELKSKPFQPHPLFSGFVKAAKDNSRLV-MTAMTILGIESSCDDTAAAVLR-GQAGDAKVLSSVVMGQTALHANFGGVVPEIAARAHAEKLDHCVKQALDEADLGLGDIDAVAVTAGPGLIGGVVSGVMCAKGIALARGLPLYGVNHLAGHALTPRLTDHVTFPYLMLLVSGGHCQFLIVSGPDAFKRLGGTIDDAPGEAFDKVARLIALPQPGGPSIEQRAKAGDPKRFKLPRPLLDRAGCDMSFSGLKTAVLRQRDSLLVD-GAMAAQDQADLAAGFQAAVVDVLAEKTRRALAEYAPLAD-VRSICVAGGVAANMAIRGALETVAQEIDAAFVAPPLALCTDNAAMIAYAALEQMASREPDGMALSARPRWPLDQTSPSMLGSGKKGAKA------------------------------------------------------------------------------------------------------------------------------------

>'Su-delicatusDSM16477'

MKASELHDKTPDQLRDELVNLKKESFNLRFQQATGQLENPARLKTVKRDVARVHTVLNQKAAAAAAE-MATADLLTMDSKKTAEKQKALDSALAQIERQFGKGSIMKLGAEGAIQDIKASSTGSLGLDIALGIGGLPMGRIIEIYGPESSGKTTLTLHCVAEQQKAGGVCAFVDAEHALDPQYAKKLGVDIDELLISQPDTGEQALEITDTLVRSGAVNMVIVDSVAALTPKSELEGEMGDSSVGVQARLMSQAMRKLTGSISRSNCMVIFINQIRMKIGVMFGSPETTTGGNALKFYSSVRLDIRRIGALKDRDEVVGNATKVKIVKNKVAPPFKQVEFDIMYGEGISKMGELLDLGVKAGVVDKSGSWFSYGDERIGQGRENAKNFLKENTAMASEIEDKIRAAHGLDFEGSNSDDADILEAMPRYTPAEIETRWQQAWEKDGVFQATRNADKPKYYVLEMFPYPSGRIHMGHVRNYTMGDVIARYKIATGHNVLHPMGWDAFGMPAENAAMAIGGHPADWTYANIAEMKKQMKPLGLSIDWSREIATCHPGYYGQQQALFLDFLKEGLVYRKNAVVNWDPVDMTVLANEQVENGCGWRSGAPVERRELTQWFFKISDHSEELLSALDSLDNWPAKVKLMQANWIGKSRGLQFAFSTIEAPEGFDRIEVYTTRPDTLLGASFVGISPDHPLAKTLERDDEAVAAFCAECRKGGTTEEAIETAEKLGYDTGIRVRHPFDTAAELPVYIANFILMDYGTGAIFGCPGHDQRDFDFATKYDLPIISTFLPSEDASPKLSEAYVPQKSEKVFYNRGFAGDQWQTGEEAVDAAIAFCEDNGIGQGVTKFRLRDWGLSRQRYWGCPIPVVHCDDCGVVPEKKENLPVELPYDVTFDTPGNPLDRHPTWRNCACPACGKDALRETDTMDTFVDSSWYYARFTAPRADTPTDLEEAAYWMNVDQYIGGIEHAILHLLYARFFARAMQITGHLPEGAAEPFDALFTQGMVTHEIYQTRDGNGRPIYHLPEEVEEGYF-KEGQEVEVIPSAKMSKSKKNVVDPLHIISNYGADTARWFVLSDSPPERDVEWTASGAEASYKHLSRVWNICDRVGEMDREAAGQGDEDLLRAMHKTIHDVTMGVESFGFNAAIAKLYAFTATLQKSKAGYAAQREAIMTLAQLMSPMTPHLSEDIWAHLGGEGLIVNAPWPKADEAMLVDDTVTLPIQINGKRRAEIQVPADMPKEEVEKVALAHEAVIRTLDGASPKKVIVVPGRIVNVVAMAITSANQLELLQTAEAVAREKMIDPGLVVEAMEESLARAAKSRYGAEMDIRVDIDRKTGKATFTRVRTVVEDEELENYQAEFTVEQAKQYMADPKVGDTYVEEVPPVEMGRIAAQSAKQVILQKVREAERDRQFEEFKDRAGTIINGLVKREEYGNVIVDVGAGEAILRRNEKIGRESYRPNDRIRVYIKDVRREQRGPQIFLSRTAPEFMAELFKMEVPEIYDGIIEIKAVARDPGSRAKIAVISHDGSIDPVGACVGMRGSRVQAVVNELQGEKIDIIPWNEDQPTFLVNALQPAEVSKVVLDEEAGKIEVVVPEEQLSLAIGRRGQNVRLASQLTGLDIDIMTEEQESQRRQAEFELRTKLFMDNLDLDEFFAQLLVSEGFTNLEEVAYVEVDELLVIDGVDEDTAGELQARARDVLEAQNKAALDNARALGVEDSLVEFEGLTPQMIEALAKDDVKTLEDFATCADWELAGGWTTVNGERVKDDGALEPFEITLEEAQTMIMTARVMLGWVDPTELEGDADEDDAETDGENAEEAEAMAHKKAGGSSRNGRDSAGRRLGVKKYGGEAVIPGNIIVRQRGTKFWPAAGVGMGKDHTIFATVDGAVTFHKGLKNRTFISVLPRAEAAEMAKRWYSVSVLSNFEKKIAEQIRASVAEQELEDQIDEVLVPTEEVIEVRRGKKVTTERRFMPGYVLVHMEMSDAGYHLINSINRVTGFLGPQGRPMPMRDAEVQAILGRVQEGEEAPRTLIHFEIGEKVKVADGPFEDFDGMIEEVDEDNQRLKVSVSIFGRETPVELEFTQVNKQIMSFTLAIVGRPNVGKSTLFNRLVGKRLALVDDQPGVTRDLREGAAKLADLRFTVIDTAGLEEVTDDSLQGRMRRLTERAVDMADICLFMIDARVGVTPSDMVFAEILRKKSAHVILAGNKAEGKAADAGMIEAYSLGLGEPIRLSAEHGEGLNDLYSMLMPLADEYEDRAVQDAPETDVDLPEDDADLETVPMPTRAKPLQVAVVGRPNAGKSTLINQILGEDRLLTGPEAGITRDAISLMTDWAGAVPMRIFDTAGMRKKAKVQEKLEKLSVSDGLRAVKFAEVVVVLLDAEIPFEQQDLRIADLAEREGRAVVVAVNKWDIEENRQEKLRDLKESFERLLPQLRGAPLITVSAKTGRGLDRLQAAIMRAYETWNRRVTTAQLNRWLSGMMEAHPPPAPQGKRIKLRYMTQAKTRPPGFVVMCSHPDKVPESYSRYLVNGLRVDFDMPGTPIRLWMRGQSDANPYKNRKKAAPSKLRKHTAGRRKDMRLIFMGTPEFSVPVLDALVQAGHDIRAVYCQPPRPAGRGKKPRPSPVQQRAETLGLLVRHPVSLKHPDTQEEFAELDAEAAVVVAYGLILPQAILDAPKHGCLNIHASLLPRWRGAAPIHRAIMAGDAKTGVCIMQMEAGLDTGPVLLCEETKIGAAETTAELHDRLSDMGALAINKALAHLTDLTPQPQPEQGVTYAAKIDKSEARIDWTRPAVEVDRLIRGLSPFPGAWFEIDGQRVKVLGSVLDEGQGAPGEVLSDDLRVACGDGAVRLTRLQKASKGAQDADVFQRGAQIAVGARLAEVMTDTSAYRVLARKYRPETFADLVGQDAMVRTLKNAFAADRIAQAFVMTGIRGTGKTTTARIIAKGMNCIGPDGNGGPTTDPCGVCEHCTAIMEGRHVDVMEMDAASNTGVANIREIIDSVHYRAASARYKVYIIDEVHMLSTGAFNALLKTLEEPPEHVKFIFATTEIRKVPVTVLSRCQRFDLRRIEPEVMIALLRKIATSENAEITDDALALITRAAEGSARDATSLLDQAISHGAGETGAEQVRAMLGLADRGRVLDLFDMVLRGDAGAALTELSAQYADGADPMAVLRDLAEITHWVSVVKITPDAAEDPTIAPEERARGQQMAETLPMRVLTRLWQMLLKALDEVAAAPNAMMAAEMAVIRLTHVADLPSPEELVRKLQNSSPPPA-PV-GGGGN-GAAQGGAQAVHQAQQRMASNPGPQGQTTALAQDLDAALARFPTFEHVVELIRVNRDVKLLVEVETCVQLAAYQPGRIEFVPTDDAPRDLAQRLGQKLQLWTGNRWAVSLVNEGGAETIAQIRDARELALKAKAKDHPMMQAVLAQFPKARITAIRTPEDIAAAATAEALPEVEDEWDPFEDGMPKMKTKSSAKKRFKVSATGKVIGSQAGKQHGMIKRTNKFIRNARGTTTLSEPDAKIIKGFMPYARMNLFAEIRHLIITTLENMVAQDALPSTLNFDPITAEPPRDPAHGDMATNAAMVLAKPAGMKPRDIAEALAAQLLHDPRITSAEVAGPGFLNLRLAPSVWQNMARQVLSQGTDFGRAILGAGQRVNVEYVSANPTGPLHVGHTRGAVFGDALASLLDFAGYDVTREYYINDGGAQVDVLARSVYLRYLEANGKEVAFPDGTYPGDYLIPLGEALAKMYGDKLVDQPESEWLDDIREFATDAMMNLIRDDLAALGVEMDVFYSEKSLYGTGQIEAAIASLESKGLIYEGVLEPPKGKKPEDWEPREQTLFKSTEHGDDVDRPVKKSDGSWTYFAPDIAYHYDKVSRDFDMLIDVFGADHGGYVKRMKAAVSALSDGHVPLDIKLTQLVKLFKNGEPFKMSKRAGTFVTLRDVVDQVGPDVTRFVMLTRKNDAMLDFDFDKVLEQSRENPVFYVQYAHARVASILRKAEAAGIDVADATLRDADLSKLDHEAEIGLLRKLAEWPRLVETAARSNEPHRVAFYLYELASDFHSLYHLGRSEDSLRALQEGDAATSQAKIALSRAVAIVIAAGLGILGVTPAQEMRMSAIDNLPPLREVIQTHELAARKSLGQNFLLDLNLTAKIARQAGDMADCDVLEIGPGPGGLTRGLLSEGARHVLAIEKDRRCLPALAEIADHYPGRLTVIEGDALKIDPLAHLTPPIRVAANLPYNVGTELLVRWLTPPEWPPFWQSLTLMFQREVAERIVATPGSKAYGRLAVLAQWRSDARIVLQLPPGAFTPPPKVSSSVVHLTALPEPRFPADPEVLSRVVAMAFNQRRKMLRSALKGAAPDIEDRLNAAGLSPTDRAEQIPLEGFCALAREIAKGMNLIAEIEAEQVAELGKDIPDFRAGDTIRVGFKVTEGTRTRVQNYEGVCISRKNGHGIAGSFTVRKISFGEGVERVFPLHSTNIDSITVVRRGRVRRAKLYYLRSRRGKSARIVENAHYKPLSGAKAMANTKRQLFLKRRMRVRNKLRKVNAGRMRLSVHRSSKNISAQLIDDVNGVTLASASTMEKDLGLVGKNNVEAATKVGALIAERAKKAGVEEAYFDRGGFLFHGKVKALAEAAREGGLKIMFAVIKTGGKQYKVQSGDMLRVERIAANAGETVQFNEVLMLGGDKPVLGAPMVKDAGVQAEVVDQIKGEKVINFVKRRRKHSSKRTKGHRQKLTLVKITDILASGAEKSGVSAAVGTGSVSA----AAVAA--AQIKKAK-----KA---KATKAAPKAEKADAGADDLKALSGVGPALEKKLHEAGITSFAQIAAWTEADIAEVDEKLSFKGRIQREGWVDQAKEKTKGMSRVKGGTVTHARHKKIIKAAKGYYGRRKNVFKVATQAVDKANQYATRDRKNRKRNFRALWIQRINAAVRSHDEALTYSRFINGLSLAGIEVDRKVLADLAVHEPEAFGAIVKQAQDALAAMQVNETLNEGLKRGYNITVTAAELEAKVNEKLAEAQPEVEMKGFRKGKVPMALLKKQFGQKVMGEAMQESIDGAMNEHFEKSGDRPAMQPEVKMTNEDWKEGDDVEVSMSYEKLPEIPEVDLSKIELEKMVVKADDAAVEEALANLAETAQDFKARKKGSKAKDGDQVVIDFKGSVDGEEFEGGAAEDYPLVLGSNSFIPGFEEQLVGVKEGEEKSVVVNFPEEYQAEHLAGKEATFACTVKEVKEPVAAEVNDEMAKKFGAEDLEALKGQIAERLEAEYAGASRAVMKRNLLDALDKQVSFDLPPSLVDAEAKQIAHQLWHEENPDVQGHDHPEVEPTEEHKTLAERRVRLGLLLAELGQKADVQVTDAEMTQAIMNQARQYPGQERQFFEFVQQNQQMQQQMRAPIFEDKVVDHVVEQAKVTEKEISKDELQKAVEELEDEMKLHELSDNEGATKKRMRVGRGPGSGKGKMGGRGIKGQKSRSGVSINGYEGGQMPIYQRLPKRGFNKPNRKSFAVVNLGLIQKFIDAKKLDAGSTIDATALIASGLVRRELDGIRVLAKGDITSQIDLNVAGASKSAIEAVEKAGGKVTVAAAKAAEASEMIQMQTNLDVADNSGARRVQCIKVLGGSKRKYASVGDIIVVSVKEAIPRGRVKKGDVRKAVVVRTAKEVRRDDGTAIRFDRNAAVILNNNNEPVGTRIFGPVVRELRAKNFMKIISLAPEVLMRHARGYRRLNRTHEHRKALFSNMAGSLIEHEQIKTTLPKAKELKPIIEKMITLAKRGDLHARRQAASKLKEDQYVAKLFDILGPRYKDRQGGYVRVLKAGFRYGDMAPMAIIEFVDRDRDAKGAADKARLAEEDAAE-MLQPKRTKFRKQFKGSIKGLAKGGSDLNFGTYGLKALQPERVTARQIEAARRAMTRHMKRQGRVWIRIFPDVPVTSKPVEVRMGKGKGSVDFWAAKVKPGRIMFEIDGVGEDVAREALRLAAMKLPIKTRVVVREDWETTIKLHNTATRRKEVFTPIDPDNVRMYVCGPTVYDRAHLGNARPVIVFDVLYRLLRHVYGPQHVTYVRNFTDVDDKINARAAESGRSIGEITAETTQWYLDDMAAVGALEPDHMPRATQYVPQMVAMIRGLIEDGYAYEAEGHVLFRVRKYTEYGALSGRSVDDMIAGARVEVAPYKEDPMDFVLWKPSDEGTPGWESPWGQGRPGWHIECSAMAHDLLGERFDIHGGGIDLQFPHHENEIAQSKCAG--HGFANYWLHNEMLQVEGKKMSKSLGNFFTVRDLLDQGVPGEVIRFVMLSTHYRKPMDWTEKKRDEAEKTLRKWYLQAAEAENATP-SADLVALLADDLNTHGALTECHRLSNAGDTAGLRGALSLLGLMGELIPEWAAVQALDLTDVEAFLSDARATAMETKDFAEVDRIKKALTSVGIEVQMSKDGVKLTPPPGFDRSRLDGVLMSQVKSSSKSDPNYKVIAENRRARFDYAIEEDIECGIILEGSEVKSLREGGANIAESYAAVEDGELWLVNSYVAPYRQAKTFQHEERRRRKLLVSRKQLADLWNATQRKGMTLVPLVMYFNHRGMAKIKIGVAKGKKLHDKRETAAKRDWSRQKQRLLKDHGMSAKHEHYDVIRKPIITEKATMASEQNAVVFEVAIESNKPMIKEAVEALFNVKVKAVNTSITKGKVKRFRGQMGRRKDVKKAYVTLEEGNTIDVSTGLMSKDKNPRRVADNEAMAKLRMLRTSPQKLNLVAALIRGKSVDKALTDLTFSKKRVAQDVKKCLQSAIANAENNHNLDVDELIVAEAYVGKNLTMKRGRPRARGRFGKIIKPFAEITIKVRQVEEQAMVSAVENMAANTSWAALGKATDLRNRILFTLGLLIVYRLGTFIPVPGIDGGALRDFMESAGQGIGGMVSMFTGGALGRMGIFALGIMPYISASIIVQLLTSMVPALEQLKKEGEQGRKKINQYTRYGTVALATLQSYGLAVSLQAGDIVADGQMGFGFACMITLVGGTMFLMWLGEQITARGIGNGISLIIFVGIIAEVPAAIAQFFASGRSGAISPAVIIGVLVMVIATIMFVVFMERALRKIHIQYPRRQVGMKMYDGGTSHLPVKVNPAGVIPAIFASSLLLLPVTVSTFSGNSTSPVMSWLLANFGPGQPLYLLFFVGMIVFFAYFYTFNVSFKPDDVADNLKNQNGFVPGIRPGKRTSEYLEYVVNRILVLGSAYLAAVCVLPEILRGQFAIPFYFGGTSVLIVVSVTMDTIQQVQSHLLAHQYEGLLEKSQLRGKGAGTRKKRSPVRRMKFLDLCKVYIRSGAGGGGCVSFRREKYIEYGGPDGGDGGTGGSVWAEAVDGLNTLIDFRYQQHFFAKNGQPGMGKQRTGKDGDDIILRVPVGTEILDEDQETVIADLTELGQRVQLARGGNGGWGNLHFKSSTNQAPRRSNPGQEGVERTLWLRLKLIADVGLLGLPNAGKSTFLAATSNARPKVADYPFTTLHPNLGVVGVDNTEFVVADIPGLIEGASEGRGLGDLFLGHVERCAVLLHLIDGTSETVAEDYRTIITELEAYGGDLAEKPRVTVLNKVDALDDEERASRLEDLEKACGGPVMMMSGVAGEGVTEVLRTLRQNIDDDRLRFRSTEEEEPWQPMAAKLRKGDKVIVLSGKDKGKTGTISSVDPKSNKAIVDGVKIAIRATRQTQTSQGGRIPKAMPIDLSNLALVDANGKATRVGFKIEGDKKVRFAKTTGDVIDAMLRSGVIAKKVGMTRLFMEDGKQIPVTVLQLDKLQVVAQRTAERDGYTAVQLGAGTAKAKRTSQAMRGHFAAAKVEPKRKVAEFRVDADAMLEVGEEIIADHYFAGQYVDVAGTSIGKGFAGAMKRHNFGGLRATHGVSISHRSHGSTGQCQDPGKVFKGKKMAGHMGAARVTTQNLEVVKTDSARGLIMVKGAVPGSKGGWVTVKDAVKKPFPEDAILPAALKSAAEEAAKAAEEAAAAAAAEAEAEAKRLAEEQAAQEAEALKAAEAEIAAEGSDADNSDADDKKEGDAMALKSYKPTTPGQRGLVLIDRSELWKGRPVKALTEGLTKSGGRNNTGRITMRRTGGGAKRLYRIVDFKRNKLDMSAVVARIEYDPNRTAFIALIQYEDGEQAYILAPQRLAIGDKIIAGAKVDIKPGNAMPFSGMPIGTIVHNIEMKPGKGGQIARAAGTYAQFVGRDGGYAQIRLSSGELRLVRQECMATVGAVSNPDNSNQNYGKAGRMRHKGIRPSVRGVVMNPIDHPHGGGEGRTSGGRHPVTPWGKPTKGAKTRNKNKASSKLIIRSRHAKKKGRMLDTATYTPRLQAEYREKIRAALKEEFGYKNDMMIPKLDKIVLNIGCGAEAVRDSKKAKSAQEDLTTIAGQKALTTIAKKSIAGFRVREEMPLGAKVTLRGDRMYEFLDRLITIAMPRIRDFRGVPGKSFDGRGNYAMGLKEHIVFPEIDFDKVDETWGMDIVIATTAKTDAEAKALLKAFNMPFNSMKLDVIKLDGGKAGSVDLDEALFGLEPRADILHRVVRWQRNNAQQGTHKVKTRSETSYSTNKIYRQKGTGGARHGDRNAPIFRKGGIYKGPTPRSHGHELTKKFRKLGLCHALSAKMKAGSLVIIDEATSEGKTAALAKQVSNLGWKRALVIDGASVNENFAQAARNIEGLDILPSMGANVYDILKRDTLVITKAGIEALEARLKMENVVLIIHLILALGLIAVVLLQRSEGGGLGI-GGGGGAVSGRSAATALGKMTWILAIGFIATSITLTIIAAEKSAGSSVIDRLSATPPAQN--GSPALPAGDDLLPPSADGDAPLVPAADMAFFTKLKDRLFKSSSKIDEGLEAIVSDGGEPEAEAAEAAPVDADQPGVASDVMPDRQEPDPQPTPD--PV----PQPEPDREPTPVPDPEPDQQDPEPTPEPDPIPPEPVPEQVPPLRQAMTPVAPDLAGEAS-DSQQAKPGLLGRLMGR-TAKPTVRRTLDDDMLEQLEELLISADMGVDTALRVTANMAEGRFGKKLSVSEIKELLAAEVGRIMEPVARPLPIYAKTPQVVLVVGVNGSGKTTTIGKLASQFRAAGKKVVIAAGDTFRAAAVEQLQVWGDRAGVPVLTAAQGSDPASLAFDAMGRAQEEGADLLLIDTAGRLQNRGDLMEELAKIVRVIRKKDETAPHNTLLVLDATTGQNAVNQVKVFQEISAVSGLVMTKLDGTAKGGVLVALADRFGLPIHAVGVGEQIDDLSPFDPQEFADALVGVDAMTQDQWGEIKQRLLKTVGQNNYTTWIEPLVPGDVEDGIVTLKVPTNFFGNYVSQNFSDLILHEINAAGTDATRLNFALNQQPANVAEKPAPAARQTTAAVKP--SANNQLTTAPLDPRFSFDNFVVGKPNELAHAAARRVAEGGPVTFNPLFLYGGVGLGKTHLMHAIARELHERRPDMNVLYLSAEQFMYRFVQALRDRKMMDFKEIFRSVDVLMVDDVQFIAGKDSTQEEFFHTFNALVDQHKQIIISADRAPGEIKDLEDRVKSRLQCGLIVDLHPTDYELRLGILQSKVEVQRKTYPDLEVADGVLEFLAHRITSNVRVLEGALTRLFAFASLVGREIDMGLTQDCLADVLRASERKISVEEIQRKVSDHYNIRLSDMIGPKRLRSYARPRQVAMYLCKQMTSRSLPEIGRRFGGRDHTTVMHGVRRIEELKVSDGQIAEDLELLRRALESMLGIGTIAKKVFGTPNDRKIKATRPLVAQINALEPEFEKLSDDEIKARTEALAKRAEGGESLDDLLPEAFANCREAARRTLGLRAFDTQLLGGIFLHQGNIAEQKTGEGKTLTATFAAYLNGLTHKGVHIVTVNEYLAKRDAEWMGKVFASLGLTTGVAYSGMPEDQKRAAYACDITYATNNELGFDYLRDNMKSSLAEMLQRDHNFAIVDEVDSILIDEARTPLIISGPSQDRSEMYQIIDTLIPSLTDEHYELDEKTRNVTFTDEGNEFLEEQLRARELIEEGMTLYDPESTTIVHHVNQGLRAHKLFQRDKDYIVRDGSVTLIDEFTGRMMPGRRLSDGLHQAIEAKEGVEIQPENVTLASVTFQNYFRLYDKLAGMTGTALTEAEEFATIYGLGVVEVPTNVPIARVDEDDAVYRTAREKYDAMIEKVKEAHAKGQPCLVGTTSIEKSEQLSSMLTRAGIEHNVLNARQHEQEAQIIADAGKFGAVTIATNMAGRGTDIQLGGNVEMKVLEALDADPEADPANIRAQIEAQHAEEKQKVLEAGGLYVLASERHESRRIDNQLRGRSGRQGDPGRTSFFLSLEDDLMRIFGSERLEKVLTTLGLKEGEAIVHPWVNKSLERAQAKVEGRNFDIRKQLLKFDDVMNEQRKVIFGQRRDIMEAQDLNEIVTDMREQVIDDLIDTYMPPKTYADQWDTQGFYAAVIEQLNVDVPIIAWCEEDGVDDEVIRERLMKATDELMSKKAEAFGEENMRNIEKQLLLQAIDTKWRDHLLTLEHLRSVVGFRSYAQRDPLNEYKNEAFQLFETMLDSLRQEVTQKLGQIQPMSEEQRREMMQEMAARQAAMQAAATEAADEA--SEQAEA-AVTGFDENDPATWGNPGRNDPCPCGSGKKFKHCHGQITMKLSGDLKAFEARIGHSFAKPELLGRAVTHASMSSANRDDNQRLEFLGDRVLGLVMAEALLALDPGATEGQLAPRFNALVRKETCADVAREIDLGKVLKLGRSEMISGGRRKQALLGDAIEAVIAAVYLDSGFDAAKDLVLRLWGNRLKTVKEDARDAKTALQEWAQARGFNPPRYDQTGRSGPDHAPVFTITARLDNGAEAAATAPSKRAAEQAAATTLLRQLEKNSMAKLGKRTRAAREAFAGKEEITVEEAVSLIKANANAKFDETIEIAMNLGVDPRHADQMVRGVVGLPNGTGKTMRVAVFARGAKAEEAEKAGADIVGAEDLMETVQSGKIDFDRCIATPDMMPIVGRLGKVLGPRNLMPNPKVGTVTMDVADAVKAAKGGEVQFKAEKGGVVHAGVGKLSFDEAKLAENIRAFVGAVSKAKPAGAKGTYMKKINLSSTMGPGVSVAVENATAEMSDEFMLDTDDLERRMNGAIASLRTEFASLRTGRASASMLEPVMVDAYGSMTPINQVGTVNVPEPRMVTINVWDKGLVGKVEKAIRESGLGINPQLNGTIIMLPIPELNEERRTQLTKVAGQYAEHARVSIRNIRRDGMDQIKKAKNDGMSEDDQKIWEDEVQELTNRFITTIDEQLETKQAEIMQVMAKKLVGTMKLQVKAGQANPSPPVGPALGQRGINIMEFCKAFNAKTADMEPGAPCPTVISYYQDKSFTMDIKTPPASYYLKKAAKVNSGAKTPSRETVGTVTTKQLREIAEAKAADLSANDVEAAMKIILGSARSMGIEVKMFENLSERLSGVFDRLTKQGALSDEDVKTALREVRVALLEADVSLPVARDFVKAVQDKATGQAVTKSVTPGQQVVKIVHDALIDTLKGEGEPGALKIDSPPAPILMVGLQGGGKTTTTAKLAKRLKERDGKRVLMASLDVNRPAAMEQLAILGTQIGVDTLPIIKGESPVQIAKRAKTQAGLGGYDVYMLDTAGRLSIDEELMQQVKAVRDVANPRETLLVVDGLTGQDAVHTAENFDERIGITGVVLTRMDGDGRGGAALSMRAVTGKPIKFVGLGEKMDALETFEPERIAGRILGMGDIVALVEKAQETIEAEQAEKMMKRMAKGQFNMNDLKMQLEQMIKMGGMQGMMGMMPGMGKMAKQVEDAGFDDKILKQQIALIQSMTKKERANPALLQASRKKRIARGAGMEVSDLNKLMKMHRQMSDMMKKMGKMGKGGMLKQAMKGMMGKGGMDPS----QMDPKALEAAAKQMGGKLPGGMGGFGGGMGLPSGLSGFGKKKMIPMPRLAQITQRFQFLEASMSAGSDGADFAKLAKEYSDLKPVVDQIELYQQLQRDLEEAEAMLEDPEMAELAREELPRLRARLPEVEAALQLSLLPRDAADAKPAMLEIRPGTGGDEAALFAADLLRMYQRYAEARGWGFDLIEEQMTELGGVKEVVAHITGQNVFARLKFESGVHRVQRVPSTESGGRIHTSAATVAVLPEAEDVDIDINPNDLRIDTMRSSGAGGQHVNTTDSAVRITHLPTGIVVTSSEKSQHRNRDKAMQVLKARLYDMERSRMDSERSADRAAQVGSGDRSERIRTYNFPQGRMTDHRINLTLYRLEAVMQGDLDEIVDALTADAQARQLAEMEGMDRAQKEQLVDELGQIFESSGVVVVSHYVGLTVAEMQDLRARARAAGGSVRVAKNRLAKIALEGKPCESIADLLTGMTVLTYSEDPVAAAKVAQEFSKENPKLVILGGSMGENALDAAGVEAVSKMPSREELISTIAGMLGAPASNIAGAIGAPASNIASILSTIEDKAAAMKTFSATPADIDKKWIIIDAEGVVLGRLASIIATRLRGKHKPSFTPHMDCGDNVIVINAEKVQMTGKKREEHFYWHTGHPGGIKSRTKEQILEGAHPERVVTQAVKRMLPGNRLSRQIMTNLRVYAGSDHPHEAQSPEVLDVKSMNKKNTRSAMDPADAELAEALRAGLSDALPPRLGIAVSGGGDSLALLYLLHGVCAAAGTYLASVTVDHGLRPEAAAEADLVARHAAELGIPHETLKWRGWGGQGNLQNAAREARYTLMAEWAARGNLPVVALGHTADDQAETVLMRLARRAGVDGLSAMAPQSTRHGVTWLRPLLAVRREALRDYLRRRALDWVEDPSNDDLQYTRIQTRQALAAFEPLGIDADALADVARNMAVAREALEEQTDCTARRILRLDAGAVVLDADAFHSQPEEIRRRLMVRALGWITGNPYPPRRAPVAALIAGLAQGQAGTLDGCQTLLRRGEIWVFREYNAVRDRVAPADHLWDGRWRAVPPDTF-DGAELRALGPDGLLQCPDWRETGRPRAMLLSTPAIWQGENLLAAPLAGMGQKWHVQLERGAGWINSAPLSHMADLKKLAEDIVGLTLLEAQELKTILKDEYGIEPAAGGAVMMAGPADGGAAEEEKTEFDVVLKNAGASKINVIKEVRGITGLGLKEAKDLVEAGGKIKEGVDKAEAEDIKGKLEAAGAEVELAMSRIGKKPVAMPSGVSAEVSGQTIEVKGPKGTRSFRATDDVTLTVGDDAITVTPRGKSKRARQQWGMSRTMIENLVTGVTTGFKKELEIQGVGYRAAINGNTLRLNLGLSHDVDYVAPEGVTVTAPKQTEIVVEGIDEQLVGQVAANIRAWRKPEPYKGKGIRYKGEFVFRKEGKKKMQVILLERVAKLGQMGEVVDVKSGYARNYLLPQGKALSASKANVEAFEGQKAQLEAQNLETKKEAEKLAEKLNGQQFVVIRSASDAGALYGSVTTRDAAEAATEEGFSVDRKQVVLMNPIKELGVHEVQVVLHPEVNAVIELNVARSPEEAELQASGKSIQELAAEEEAAAEFEISELFDDIGSAAGDDDDLAEVVQTPEDDAKDDSNS--MSDSDGKKTLGLRGGARPGNVKQSFSHGRTKNVVVETKRKRVVVPKPGGQKPTGPGAGPIGDPKKRPAGITDAEMERRLKAVQAAKAREVEEAAAREAEEKARAEERERRRAEIEAKEREEREREESLKAKAEEDARRKAEAEA---AAVAPA-EPAAVREQSNKPLPAATPRKTERDRDDTKKRSKGGDS-RRSGKLTVNQALAGGEGGRQRSMAQMKRKQERARQKAMGGQVEREKIVRNVNLPPAIVVSELANRMAEKTGAVVKALMQNGMMVTQNETIDADTAELIIEEFGHKVVRVSDSDVEDVIKEIEDKPEDLQGRPPVITIMGHVDHGKTSLLDAIRNAKVVAGEAGGITQHIGAYQVTTDNGAVLSFLDTPGHAAFTSMRSRGAQVTDIVVLVVAADDAVMPQTIEAIAHAKAAKVPMIVAINKIDKPAANPDKVRTDLLQHEVIVEKMSGDVQDVEVSAATGQGLDELLEAIALQSEILELKANPDRAAVGAVIEAQLDVGRGPVATVLVQNGTLRQGDIFVVGEQYGKVRALINDQGERVKEAGPSVPVEVLGINGTPEAGDVLNVTETEAQAREIAEYRANAAKDKRAAAGAATTLEQLMANAKANEDVSELPILVKADVQGSAEAIVQAMEKIGNDEVRVRVLHSGVGAITETDVGLAEASGAPIMGFNVRANASARNTANQKGVEIRYYSVIYDLVDDVKAAASGLLSNEIKENFIGYANIKEVFKVTGVGKVAGCLVTEGVARRSAGVRLLRDNVVIHEGTLKTLKRFKDEVPEVQSGQECGMAFENYEDIRPDDVIEIFEREEVTRTLAMAKPKKTPRPKAQAPKGFRDYFGAEVTHRAEMLSKIAAVYQRYGFDALESSGVETVEALGKFLPDVDRPNEGVFAWQEDAEGDKPGDWLALRYDLTAPLARVYAQHRNDLPMPYRRYAMGPVWRNEKPGPGRFRQFYQCDADTVGAPSVAADAEICAMLADCLEEVGIARGDYVVRVNNRKVLNGVLEVAGLAGDDKDAERGIVLRAIDKLDRLGPEGVRALLGEGRKDDSGDFTKGAGLADAQADVVMGFMQAKRDSGAKTVARLRELVAGSDVGVQGVDELELISDLLAAGGYGPDRVEIDPSVVRGLGYYTGPVFEAELTFEIKDEKGRARNFGSVAGGGRYDDLVKRFTGQEVPATGVSIGVDRLLAALAAKGRLAGEATGPVVVTVMDRDRMADYQAMVAELRQAGIRAEVYLGNPKNFGNQMKYADKRQSPVVVIEGGDEQDRGVVQIKDMVLGAQMAQDASHDEWKERKNQYEVKRGDLVQAVRDILDRTSMDDLKQKYLSQIADAGDESALEDIRLAAVGKKGEVALKMRELGKMTPEERQVAGPALNALKDEINSALAAKKAALGDAALDERLRSEWLDVTLPTRHQRQGSIHPVSQVTEELTAIFAEMGFSVAEGPRIDTDWYNFDALNIPGHHPARAEMDTFYMARGEGDDRAPHVLRTHTSPVQIRTMEAEGAPLRIICPGGVYRADYDQTHTPMFHQVEGLAIDKDLSMANLKWVLEEFFSAFFEIDGIKTRFRASHFPFTEPSAEVDIQCSWVDGQLRIGEGDGWMEVLGSGMVHPKVLQAGGIDPNEWQGFAFGMGIDRIAMLKYGIPDLRAFFDADLRWLRHYGFASLDQPNLHGGLSRMEMADEAGLDLVEISPNANPPVCKIMDFGKFKYEQQKRESEARKKQKIIEIKEVKFRPNTDTNDYDVKMRNVFKFLEGGDKVKITLRFRGREMAHQNLGRELLERVAEDTKEMGRVENFPKMEGRQMVMVIGPLPNMKFTLSWLKDHLDTTASIDEITYALTDLGLEVEGVENPAAKLADFTLGYVQTAEKHPDADRLNVCQVETDEGVMQIICGAPNARAGITVVVAKPGVYVPGIDTTIGVGKIRGVESFGMMASEREMELSEEHDGIIELPSGKPGDRFIDWLAENDPAKVDPVIEIAITPNRPDALGVRGIARDLAARGLGKLKPRDCDAVEGSFASPIGVSIDEDTLDGCPVFYGRVIRGVKNGPSPQWLQDCLRAIGLRPISFLVDVTNFFTFDRNRPLHVFDADKVKGNLRVHRAKGGEEIAALDEKTYTLQAGQMVISDDSGVESIAGIMGGEATGVTEDTVNVFVESAYWDPVQIAYAGRALKINSDARYRFERGVDPAWTPYGIEHATRMILDHAGGEPSEVVVAGQVPDTRRAYKLDAAKVQSLVGMTIPESDQRQTLTALGFQLDGDMAQVPSWRPDVQGEADLVEEVARIASLTQLQGKPLPRLTTGVPRPVLSPMQRRVVTARRTAAALGYNECVTYSFIDQASAALFGGGTDETRLENPISSDMSHMRPDLLPGLLQAAARNQARGFADMALFEVGPAFSGGEPGEEQIMVSGLLVGRTGPRDVHGAARAVDVFDAKADAEAVLSAIGAPAKVQILRGAAEWWHPGRHGKICLGPKKVLGVFGEVHPRVLAAMDVKGPAMAFTIYPAEVPMPRKSGATRPALQISDLQAVERDFAFVVDADVEALTLVNAAKGADKSLIDDVRVFDEFIGGSLGEGKKSLAITVRLQPSDKTLKDADIEAVGAKVVEKVTKATGGVLRGMHAYRSHTCADLSLENKGDDVRLSGWVHRVRDHGGVLFLDLRDHYGITQVICDPDSPAFAEMEKVRAEWCVRIDGTVKARDESLVNPKLPTGAIEVYARDIEVLGSAAELPLQVFGDQEYPEETRLRYRYLDLRREKMQKNMTLRSDVVTSIRKRMWDQNFREFQTPIITASSPEGARDFLVPSRLHPGKFYALPQAPQQFKQLLMVSGFDKYFQIAPCFRDEDPRADRSPTDFYQLDMEMSFVTQQDVFDTIQPVIAGVFEEFGGGKKVDQTWEQISYKDAALWYGSDKPDLRNPIKMQVVSDHFRDSGFAIFAKLLEQDGTEIRAIPAPTGGSRKFCDRMNAFAQKEGLPGMGYIFWRDQG---NGMEAAGPLAKNIGPERTEAIRQQLGLEVGDAAFFLGGKPKSFEAVAGRARNVIGEELNLTDKDRFAFAWIVDFPIYEKDEETGKIDFEHNPFSMPQGGMEALQGDPLEVLGYQYDLACNGYELVSGAIRNHKPEIMFKAFEIAGYGEDEVRKRFGGMVNAFQYGAPPHGGCAAGIDRIVMLLAEEANIREVILFPMNQRAEDLMMDAPSEPTSDQLMELGLRVIPQEMTPLENIRNFSIVAHIDHGKSTLADRLIQSTKTVADRDMKEQMLDSMDIERERGITIKAQTVRINYTADDGQDYILNLIDTPGHVDFAYEVSRSMRAVEGSLLVVDSTQGVEAQTLANVYHAIDADHEIVPVLNKIDLPAADCDRVAEQIEDVIGIDASEAIRVSAKTGIGIKETLEAIVTRLPAPKGTLDAPLKAMLVDSWYDSYLGVIVLVRIMDGQLRKGDRVRMMQNGSVHHVDRIGVFRPAMTEIDVLGPGELGFLTASIKQVRDTRVGDTITHEKKGTDNALPGFKPSQPVVFCGLFPVDAAEFEDLRDSIEKLALNDASFSYEMETSAALGFGFRCGFLGLLHLEVIRDRIEREYDIDLITTAPSVIYDIHMKDGSVEQLHNPADMPDLTHVDHIEEPRIKATILVPDEYLGDVLKLCQDRRGIQENLTYAGSRAMVVYDLPLNEVVFDFYDRLKSVTKGYASFDYQMIGYRTDSLVKMSILVNDEPVDALSTMVHRDRAEMRGRAMVEKLKDLIPRHMFKIPIQAAIGGKVIARETLSAMRKDVTAKCYGGDATRKKKLLEKQKAGKKKMRQFGKVDIPQEAFISALKMDSMSLPPGFLEELRDRASLSQVVGRKVIWDTRKSNQGKGDMWAPCPFHQEKSASFHVDDRKGFYYCFGCHAKGDAISFVRETENVSFMEAVEIIAREVGMPVPKQDPRAQAKADKRTQLAEVMEQAVQWFRLQLRTGAAGAAREYLTKRGLSEQAQAHWEIGFAPDSWQGLWDALKGKGVADELILGAGLAKPSSKGGRPYDTFRGRIMFPIRDARGRAIAFGGRAMDPEDKAKYLNSPETELFDKGRSLYNVKDARAAAGRGQPLIVAEGYMDVIALHGAGFEGAVAPLGTAITENQLQMLWRIAPEPIITLDGDVAGQRAALRLIDLALPLLEAGQSLRFAVMPEGQDPDDLLRAQGAGALQKLLDSALPMVRLLWQRETEGKVFDSPERKAALDKSLREKIKLIRDPSIRSHYGQEIKDLRWELFRPQKPRARGPSKG-GKGGKPAWG-GPQAPLASTKASALVAMGDDQVGIYLREAVILVALCNCPTLVETFETGLEAMPCADPDHAQLRDLLLRYGHAGAEVLNEQISYSLGWEALENMKAQRHVAITPCIRKPGDVDMTRMTVAEELAKLDAARGLNEEIAEAVEDLSGPADEGVTWRLSEAARAADMASRSTQEESGEFDLGDNGMTMSREERSALDALLGTIRYDKSKGREMCAETPDYKATLNLPKTDFPMRAGLPKREPGWLERWEKIGVYDRLREKEGRTPFTLHDGPPYANGHLHIGHALNKTIKDMIVRSHQMMGYDARYIPGWDCHGLPIEWKIEEQYRKKGRDKDQVPINEFRSECREFARGWVDVQREEFKRLGITGNWENPYLTMDFHAERVIAEEFMKFLMNGTLYQGSKPVMWSPVEKTALAEAEVEYHDKESHTVWVKFKVAL-RHEKSDLEDANVVIWTTTPWTMPSNKAVVYGEGISYGLYEVTATPEECWANVGERFLLADDLAADVFARARLEDGMWQRVRGVENDELAKISLLHPLAGAEGANGEWDDLRDFRAADFVTSDEGTGFVHCAPSHGLEEYELYRDLGMLPQVITYNVMEDGRFRDDLPFFGGKAILKPNGKEGNANSAIIDKLVEVGGLLARGKIKHSYPHSWRSKAPVIYRNTPQWFAAIDKEVGDGLDMHGKTIRERALTEIDNVNWTPKSGRNRLHSMMEARPDWVLSRQRAWGVPLTCFVRKGVAPTDENFLLRNPEVNQRIVEAFEAEGADAWYAEGAKERFLEGIVDPAEFDQVTDILDVWFDSGSTHAFTLRDREDGTEDGIADVYMEGTDQHRGWFHSSLLQSVGTTGRAPYRNVVTHGFTLDAKGMKMSKSIGNTIVPEKIVQQYGADILRLWVAQTDYTADQRIGDEILKGVADSYRRLRNTMRYMLGALNDFSEADRVDPAEMPELERWVLHRVAELDKVVRDGFARFDFQGVFQAVFTFATVDLSAFYFDIRKDALYCDGDTLRRRAARTVLDILFHRLTTWLAPVLVFTMEEVWLERFPGDDSSVHLVDMPETPEAWLNPELAAKWAKVRAARRVVTAALEVQRTEKVIGASLEAAPVVHVADEAQRAALESVSFEDVSITSDITVTGDEAPAEAFRMPETQGVAVVFEKAEGAKCERCWKVLPDVGTHEYPGVCGRCDKAVRAAQSQNIRIRLKAFDYRVLDSSTQEIVSTAKRTGASVRGPIPLPNKIEKFTVLRGPHVDKKSRDQFEIRTHKRLLDIIDPTPQTVDALMKLDLAAGVDVEIKLQSMADEIKSLDGLEAAVTGGVQGTE----TEMTPREPVRDELGRAYATGKRKDAVARVWIKPGSGKVIVNGKPQNEYFARPVQQLILAQPFGITNTEGQFDVIATVKGGGLSGQAGAVKHGISKALQLYDPSLRGALKAAGFLTRDSRVVERKKYGKAKARRSFQFSKRMPTIQQLIRKPRQPKVKRSKSMHLQECPQKRGVCTRVYTTTPKKPNSAMRKVAKVRLTNGFEVISYIPGESHNLQEHSVVLIRGGRVKDLPGVRYHILRGVLDTQGVKDRKQRRSKYGAKRPKMARDKTRTKRKVSKNIAAGVAHVNSSFNNTKILISDVQGNAIAWSSAGTMGFKGSRKSTPYAAQMAAEDAGRKAQDHGVKTLEVEVQGPGSGRESALRALAAAGFNITSIRDVTPMAHNGCRPPKRRRVSKQSRRGLLIILSSPSGAGKSTMARALRDWDPTINFSVSATTRAPRPGEEDGKDYRFVAENDFRQAVAEGEMLEHAHVFGNFYGSPKAPVQDAIDQGQDILFDIDWQGAQQIRNSDLNTHTLSIFLLPPSIIELKRRLESRGQDDAETIAKRMGKSWDEISHWDGYDFVLINDDLDQTEARLKSIITAARLRLSQQPAIKDHVRRLQSEFEELKMAQSFLGQKRLRKYYGKIREVLEMPNLIEVQKSSYDLFLNSGDAETPTDGEGITGVFQSVFPIKDFNETSVLEYVKYELEKPKYDVEECQQRDMTYSAPLKVTLRLIVFDVDEDTGAKSVKDIKEQDVFMGDMPLMTPNGTFVVNGTERVIVSQMHRSPGVFFDHDKGKTHSSGKLLFACRIIPYRGSWLDFEFDAKDIVFARIDRRRKLPVTTLLYALGLDQEAIMDAYYKTVTYTLEKNKGWVAPFFPDRVRGTRPTYDLVDAATGEILFEAGKKVTPRAVKKLLDEGKVKDLLLPFDHIVGKFVARDIINEETGAIYVEAGDELTLEYDKDGTLIGGTAKELIDAGITEIPLLDIDNVNVGPYMRNTMAMDKNMNRDTALMDIYRVMRPGEPPTVEAASALFDTLFFDSERYDLSAVGRVKMNMRLALEKPDTQRTLDRDDIVACIKALVDLRDGRGDIDDIDHLGNRRVRSVGELMENQYRVGLLRMERAIKERMSSVEIDTVMPQDLINAKPAAAAVREFFGSSQLSQFMDQTNPLSEVTHKRRLSALGPGGLTRERAGFEVRDVHPTHYGRMCPIETPEGPNIGLINSLATFARVNKYGFIETPYRVVKDSTVTDEVHYMSATEEMRHTVAQANANLDENMKFVNELVSTRQSGDYTLAPTENVDLIDVSPKQLVSVAASLIPFLENDDANRALMGSNMQRQAVPLLQAEAPLVGTGIEEVVARDSGAAYTARRAGIIDQVDASRIVIRATEDLELGDAGVDIYRMRKFQRSNQNTCINQRPLVKVGEKVTKGQVIADGPSTDMGELALGKNVVVAFMPWNGYNYEDSILISERISRDDVFTSIHIEEFEVAARDTKLGPEEITRDIPNVGEEALRNLDEAGIVYIGADVEPGDILVGKITPKGESPMTPEEKLLRAIFGEKASDVRDTSLRVKPGDFGTVVEVRVFNRHGVEKDERALQIEREEVERLARDRDDELAILDRNIYARLKDMILGKIAVKGPKGVKANSQITEELLETLTRGQWWQLALEDEDDAKIVEALNEQYEIQKRTLDARFEDKVEKVRRGDDLPPGVMKMVKVFVAVKRKLQPGDKMAGRHGNKGVISKVVPMEDMPFLADGTPVDFCLNPLGVPSRMNVGQILETHMGWAARGLGLNVDEALQEYRRSGDLTPVREALSLAYGDDVYEEGISGMDEDTLLEAAGNVRRGVPIATPVFDGAKEADVNDSLKRAGFDTSGQSVLFDGRTGEQFARPVTVGVKYLLKLHHLVDDKIHARSTGPYSLVTQQPLGGKAQFGGQRFGEMEVWALEAYGAAYTLQEMLTVKSDDVAGRTKVYESIVKGEDNFEAGIPESFNVLVKEVRGLGLNMELLDAEEDEMPLYEHVMIARQDLSNTQAEGLIEHFGTVLADNDGKLVDSEYWGVKTMAYKINKNRKGHYAFLRSDAPATAVQEMERLMRLHDDVMRVLTIKVDEHKELPSVQMQKRDERPDRRERRMTSQIEVEALDEAQAKSELARLAEVLAEANAAYHTDDAPEISDAEYDTLKRRNAEIEARFPQLKRSDSPSEQVGAPVAEGFSKVRHAVSMLSLANAFDAEEVAEFDARIRKYLGLGPEAHLAYTAEPKIDGLSLSLRYEKGVLVQAATRGDGAVGENVTANARTIADIPHELKDAPDLLEVRGEVYMSHADFAAVNERQSAAGGKTFANPRNAAAGSLRQLDAEITRARPLRFFAYAWGALSAPLAETQKGAIDRLEALGFATNPLTALCDGPDEMIAHYEKIEEQRATLGYDIDGVVYKVDDLALQDRLGFRSTTPRWAVAHKFAAELAWTRLESIDIQVGRTGALSPVARLQPVTVGGVVVSNATLHNEDYIKGLDSKGAEIRGGKDVRVGDWVQIYRAGDVIPKVADVDLSKRPDDAQPFIFPTTCPECGSDAIREPGDAVRRCTGGLICPAQAVEKLKHFVARGAFDIEGLGAKQVEQFYQDGWIAEPADIFTLKDRYGTGVQQLKNREGWGPKSADKLFEAIDDKRKIPMSRLLFALGIRHLGEAASNLIALHYGDWDSFEAAMADARGLSGPAWDDLIGVDGVGSVMAGSLVSTFAQEAERASIDRLVAHLTVIPAERPDTADSPVAGKTVVFTGTLEKMSRAEAKARAERLGAKVSGSVSAKTDLLVAGPGAGSKAKKAADLGIETLDEDGWLALIDGKMIHKNWAELIKPQQLDVKPGNDPARQATVMAEPLERGFGLTLGNALRRVLMSSLQGAAITSVQIDNVLHEFSSVSGVREDVTDIILNLKGVSLRMEVEGPKRLSISAKGPGVVTAGDISESAGIEILNREHVICHLDDGADVYMELTVNTGKGYVSADKNKPEDAPIGLIPIDAIYSPVKKVSYDVQPTREGQVLDYDKLTMKVETDGSITPDDAVAFAARILQDQLGIFVNFDEPESASRQDDDDGLEFNPLLLKKVDELELSVRSANCLKNDNIVYIGDLIQKTEAEMLRTPNFGRKSLNEIKEVLSGMGLHLGMDVEDWPPENIEDLAKKFEDSFMARDDNRGGNRRNQRDETPEFADRLVAINRVSKTVKGGKRFGFAALVVVGDQKGRVGFGKGKAKEVPEAIRKATEQAKRQMIRVQLREGRTLHHDMHGRHGAGKVIMRTAPEGTGIIAGGPMRAVFEMLGVKDVVSKSVGSQNPYNMIRATLDGLRKEQSPRSVAQRRGKKVADILPKRDDNVESSAQVAEEAMNDPIADMLTRIRNSSLRGKSTVVTPASKLRAWVLDVLADEGYIRGYEKMTGADGHPAIEISLKYYEGEPVIRELKRVSKPGRRVYMAVNDIPVVRQGLGVSIVSTPKGVMSDASARSANVGGEVLCTVFMHDIRAIRDNPEGFDAALARRGEAAMSEAVLSLDAARRAKIAAAETAKAEQNKASKEVGAAKAKGDEAEFERLRALVSDKKAEVAAMQAEAQELDEQLTDMLARIPNNPADDVPEGANEDDNVAVKTWGDKPAFDFTPVEHYEIAGVKPNMDFETAAKTSGARFVMLKGAVARVHRALAQFMIDTHVDENGLTEVNSPVLVRDEAMYGTDKLPKFGEESYQTTNGWWLVPTSEVPLTYSVAGDTLEQSALPIRLTAHTLCFRSEAGSAGRDTAGMLRQHQFEKVEMVSVVHPDESDAEQQRMVGCAEGILEKLGVAYRTVILCTGDMGFGARRTYDIEAWVPGQDCYREISSVSTTGDFQARRMNARFKPEGGGKPQFVHTLNGSGLAVGRCLIAVLENGQQADGSVKLPEVLAGYLGGKTTLTAEGVLAMAITASMVKELRDSTGAGMMDAKKALTETDGDMEAAVDWLRTKGLAKAAKKSGRTAAEGLVAVKVEDGHGVAVEVNSETDFVGKNAEFQSMVSNIADAALKTDDVEALKAAEINGKTVETTITDAIAKIGENMSLRRMKSIDGETVVSYVHNAAAPGMGKIGVLVAMNGGDEAFGKQVAMHIAAVNPASLSEADLDPAVVEKEKQVQMDIARESGKPEAVIEKMITGRMQKYMSEVTLLNQSFVVNPDLTVGKAAEEAGATITGFVRLEVGEGIEVVKEDFAAEVAKAAKGMNQELTNNPFNPLTPQKAFDEIKVSLASPERILSWSFGEIKKPETINYRTFKPERDGLFCARIFGPIKDYECLCGKYKRMKYRGVVCEKCGVEVTLQKVRRERMGHIELASPVAHIWFLKSLPSRIGLMLDMTLRDLERVLYFENYVVIEPGLTDLQYGQMMTEEEYMDAQDAYGMDAFTANIGAEAIREMLAAIDLESEAETLRADLKEATGELKPKKIIKRLKVVESFLESGNRPEWMVMTVIPVIPPELRPLVPLDGGRFATSDLNDLYRRVINRNNRLKRLIELRAPDIIVRNEKRMLQESVDALFDNGRRGRVITGANKRPLKSLSDMLKGKQGRFRQNLLGKRVDFSGRSVIVTGPELKLHQCGLPKKMALELFKPFIYSRLEAKGLSSTVKQAKKLVEKERPEVWDILDEVIREHPVMLNRAPTLHRLGIQAFEPVLIEGKAIQLHPLVCSAFNADFDGDQMAVHVPLSLEAQLEARVLMMSTNNVLSPANGAPIIVPSQDMILGLYYTTLEREGMVGEGMVFGSVDEVQHALDAGAVHLHSKIKARIKQIDAEGNEVMKRFDTTPGRVRLGALLPLNAKAPFDLVNRLLRKKEVQQVIDTVYRYCGQKESVIFCDQIMTMGFREAFKAGISFGKDDMLIPDTKWPLVEETREQVKDFEQQYMDGLITQGEKYNKVVDAWSKCNDKVTEAMMGSISATTYHENGSEKEPNSVYMMAHSGARGSVTQMKQLGGMRGLMAKPNGDIIETPIISNFKEGLTVLEYFNSTHGARKGLSDTALKTANSGYLTRRLVDVAQDCIVREHDCGTEIAITAEAAVNDGEVVSSLAERLLGRVAAEDILKPGTEEVIVPAGGLIDERLSDAIDEAAVQSARIRSPLTCEAEEGVCAMCYGRDLARGTLVNQGEAVGIIAAQSIGEPGTQLTMRTFHIGGVAQGGQQSFLEASQSGKIVFENAQTLENSSGEILVMGRNMKLSIVDENGDERASHKVGYGTKLFVKDGDTIARGDKLFEWDPYTLPIIAEKPGMTKYVDLVSGIAVKEDTDDATGMTQKIVIDWRAAPKGNELKPEIILVGDDGEPVRNDAGNPVTYPMSVDAVLSVEDQTEIQAGDIIARIPREGAKTKDITGGLPRVAELFEARRPKDHAIIAEIDGYVRFGKDYKNKRRIAIESSDDPDVKVEYMVPKGKHIPVAEGDFVQKGDYIMDGNPAPHDILAIMGVEALAEYMIDEVQDVYRLQGVKINDKHIEVIVRQMLQKWEIQESGDTTLLKGEHVDKQEFDQANEKALKKGGRPAKGEPILLGITKASLQTRSFISAASFQETTRVLTEASVQGKRDKLVGLKENVIVGRLIPAGTGGATQQMRKVATDRDNVVIEARREEAEAAAALAAPEASADDIVGGDVFNAPVGDDESRDMSRRHAAEKREVLPDAKYGDLVLTKFMNNLMIDGKKSVAERIVYNAMTRVEDKIKRAPIEVFHEALENIQPSVEVRSRRVGGATYQVPVEVRPERRQALAIRWLIKAARARNENTMEERLAGELMDAVQSRGTAVKKREDTHKMADANKAFSHYRWMPYAHSDKSEGMPLLANPAPDVRNRPKLEGGKKFNLVTEFDPAGDQPTAIKELTEGVNSGERDQVLLGATGTGKTFTMAKVIEETQRPAIILAPNKTLAAQLYGEFKGFFPDNAVEYFVSYYDYYQPEAYVARSDTFIEKESQINEQIDRMRHSATRALLERDDVIIVASVSCIYGIGSVETYGAMTQDLKAGESYDQRKIIADLVAQQYKRNDAAFQRGSFRVRGDSLEIFPAHLDDRAWRLSFFGEELESITEFDPLTGEKTDTFDQIRVYANSHYVTPKPTMSQAVIGIKKELRTRLDQLVADGKLLEAQRLEQRTNFDIEMLEATGVCNGIENYSRYLTGRAPGEPPPTLFEFIPDNAIVFADESHVSVPQIGGMYKGDYRRKFTLAEHGFRLPSCMDNRPLKFEEWDAMRPQSVFVSATPAAWEIEQTGGVFTEQIIRPTGLIDPHIEIRPVEMQVDDLLDEVRKVAADGYRTLCTVLTKRMAEDLTEYMHEQGIRVRYMHSDIDTIERIEILRDLRLGAFDVLIGINLLREGLDIPECGLVAILDADKEGFLRSETSLIQTIGRAARNAEGRVIMYADRITGSMERAMGETDRRRAKQIAYNEEHGITPATVKKNVDDILAGLYKGDVDMNRVTAKVDNPLAGGNLQTVLDGLRTDMRKAAENLEFEEAARLRDEVKRLEAVDLAIADDPMARQQAIDKAVDTAQKASGRSTSGRGGMRGGNVK--RRMAAKPFFRRRKVCPFSGDNAPAIDYKDTRLLQRYISERGKIVPSRITAVSAKKQRELARAIKRARFLALLPYAVKMPKRILTGTVTSDANAQTVTVSVERRFTHPVLKKTIRKSKKYRAHDENNTFKVGDSVRIIECAPKSKTKRWEVLTSETMANTTQSAKRARQNEKRFAINKARRSRIRTYIRKVEEAIASGDKAAATAALKTAQPELMRGVTKGVFHKNTASRKMSRLSARVKALGMARSVWKGPFVDSYVLKKAEASREGGRNEVIKIWSRRSTILPQFVGLTFGVYNGHKHIPVNVSEDMIGQKFGEYSPTRTYYGHAADKKAKRKMARIAGVNIPTAKRVPIALTYITGIGTSSAKAICEAVGIDETRRVNELSDAEVLAVREHIDANYTVEGDLRRDTQMNIKRLMDLGCYRGLRHRRNLPVRGQRTHTNARTRKGPAKAIAGKKKMAMKIRLARGGSKKRPFYRIVAADSRMPRDGRFIEKLGTYNPLLPKDSEERVKMDVEKIEAWIAKGAQPTERVTRMLEAAGVREKTERNNPKKGTPGKKAQERVQEKADKAAAAAEAANAPAEEASAE-MSITAEEKAKVMKDFGTKEGDTGSPEVQVAILTSRIVTLTEHFKTHKKDNHGRRGLLKMVATRRKLLDYVKAKDESRYQDLIKRLGLRRMKTLNDIRSTFLNYFDAQGHQVVPSSPLVPRNDPTLMFTAAGMVQFKNLFTGVETRDYSRAASAQKCVRAGGKHNDLDNVGYTARHHTFFEMLGNFSFGDYFKAEAIPFAWDLLTKEFGIDPDRLLVTVYHTDEEAVKIWKAHTGLSDDRIIRIATDDNFWSAGPTGPCGPCTEIFYDHGDHIWGGPPGSPEEDGDRFVEIWNLVFMQYEQFEDGTRQPLPNQSIDTGMGIERVAALLQGTNDNYATDLMRNLIEASAHASSTDPDGPGKTHHRVIADHLRSTSFLIADGVMPSNEGRGYVLRRIMRRAMRHAHLLGVKDPLMHQLVPSLVQQMGAAYPELGQAQSLIRETLLLEETRFRQTLDRGLKLLDDELRDLPEGATLPGEAAFKLYDTYGFPLDLTQDALREKGRAVDTEGFDTAMEAQKAKARAAWAGSGEAADATVWFDVADKHGVTEFLGYDTESAEGQIVALVQGNNPVEVAAIDSEVQVALNQTPFYAESGGQVGDTGLIRTQTGRVKVTDTRKSAGVFVHFGHVVEGEVKPGQTAVLEVDASRRTAIRANHSATHLLHEALRNALGDHVSQRGSLNAHDRLRFDFSHAKGLSEDELRQVEREVNDYIRQNTPVETRIMTPDDARAMGAQALFGEKYGDEVRVVSMGQLDGSGKGSDKSTYSLELCGGTHVRQTGDIGAFVLLGDSASSAGVRRIEALTGTEALNWLREQEAALSRVAAELKTSTSDVPERVRALMDERRSLANEVAQLRRELAMS-GGGAAAPEAREVNGVKFVAQVLNGVTGKDLPGLVDEHKAKLGSGAVLLIADTGGKAAVAGGVTKDLTERVSAVDMVKAAVAELGGKGGGGRPDMAQGGGASAENAEAAIAAAENILKGMGFKMGIVGLPNVGKSTLFNALTRTAAAQAANFPFCTIEPNVGEVAVPDARLDTLAEIAKSKSIIPTRMTFVDIAGLVKGASKGEGLGNQFLANIREVDAIAHVLRCFEDGDVTHVEGRVDPVADAETIDTELMLADIESIEKRLQNIVRKVRGGDKEAVQQERLMRKALEALEAGNPARVVEVDEDDSKAWRMLQLLTTKPVLYVCNVGEAEAAEGNAHSAKVAEMAAAQGNSHVVISAQIEEEISQLEAEEAEMFLEEMGLKEAGLDRLIRAGYELLHLETYFTVGPKEARAWTIKTGTSAPKAAGVIHGDFEKGFIRAETIAFDDFVSLGGEGPAKEAGKMRAEGKSYIVKDGDVLHFLFNTMGWKTLDDMDLNGKRVLLRVDINVPVEEGRVTDATRIERIVPTVTDILAHGGKVTLLAHFGRPKGKVVEDMSLKQVLPALEKALGRNVTFVPSLDAAA-----EAQGDLQLMENIRFYPGEEANDEGFARQLAALGDIYCNDAFSAAHRAHASTEALARLLPACAGRLMQAELSALEAALAKPERPVGAVVGGAKVSTKIALLENLVNKLDVLVIGGGMANTFLAALGADLGKSLQEPDYYDTAKDIMAQAEKAGCRVILPVDGLVARDFAKGAAHEVAQLCPDARLAEDQMVLDAGPDTVALVESAFAGLRTLIWNGPMGAFEIPPFDTATVAAARAAAKQTRDGTLTSVAGGGDTVAALNQAGVAEDFTYISTAGGAFLEWMEGKTLPGVAALGGMALPEFSMRQLLEAGVHFGHQTQRWNPRMGPYIYGARNGIHIMDLTQTVPMLDDALKVIRDTVAKGGSVLFVGTKRQAAQPIAEAAEKCAQYYMNHRWLGGTLTNWQTVSQSINRLKNIDEQSERGFEGLTKKERLGMERDQFKLEASLGGIREMGGRPDLLFVIDVKKEALAVAEANKLGIPVVAVVDTNCSPDGIDYIIPGNDDAARAIALYCDLAARAALDGMSAQLGAAGVDLGAMEEAPEEEAVSEESNASEETVHDDAMGKDAESMTKRTAAKHKLDRRMGENIWGRPKSPVNRREYGPGQHGQRRKGKISDFGIQLRAKQKLKGYYGDLTEKQFRRIYGEAERVKGDTGENLIGLLERRLDAVVYRAKFVPTVFAARQFVNHGHVKVNGRKVNIPSYRVKEGDVIEVRDRSKQLASVLEAVQLPERDVPDYLETDHSKLTATFVRTPGLSDVPYPVVMEPNLVVEFYAKNMGNKVNPIGMRLQVNRTWDSRWYADTKDYGDLLLEDLAIRDFIKKECHQAGVARVIIERPHKKCRVTIHTARPGVIIGKKGADIETLRQKIAKMTNSELHLNIVEVRKPELDAHLVGESIAQQLERRVSFRRAMKRAVQNAMRMGALGIRVNLAGRLGGAEIARTEWYREGRVPLHTLRADIDYAHVEAATAYGIIGIKTWIFKGEIMEHDPAARDRKAQELQDGPAPRGAGGRRMGRKRKGRDISGWLVVDKPAGPTSTAVVNKVRWALEAKKAGHAGTLDPEATGVLAIALGEATKTVPYITDALKAYEFTVRLGIATNTDDAEGEVIGTSDLRPDDAAIKDVLSGFIGDIQQVPPQFSAVKIDGQRAYKRARDGEEMDIAARPLWVESLLLVDRPDADHVTLEMVCGKGGYVRSIARDLGQKLGCLGHVRELRRTWSGPFEVKDALTLAQIDEMARTPELDTHLLPLAEGLAELPEVKATPEGASRLRNGNPGMVIAHDVEYGEECWASLDGQPVAVGRFKAGELHPSRVFNLSTMFLLGLTGSIGMGKSTTAKMFAEEGCAVWDADAAVHRLYGKGGAAVAPMAAEFPAAIKDGAVSREALKETIAADPTALPRIEAIVHPLVAEDRATFLADAKSDIAVLDIPLLFETGGAAAMDAVACVTIPDDVQRDRVLARGTMTEAQFDAIRAKQMPAEEKCARADYVIETDMLDHARAQVRAVIRDIREKLRHAMEDFDVLIEDDRWETVDLERLAQGAAEATLRHLGLTPDAAELTLLACDDTRIAALNNDFRGKARATNVLSWPAEERGSAIPGGDPLPVSPGVDGMVELGDIALAYQTCAAEAEAADKPLADHVTHLIVHGLLHLLGYDHENDPDALLMEGLETEILGKMGLDDPYRENGPMARYIFITGGVVSSLGKGLASAALGALLQARGFSVRLRKLDPYLNVDPGTMSPFEHGEVFVTDDGAETDLDLGHYERFTGVAARKTDSISSGRVYSTVLEKERRGDYLGKTIQVIPHVTNEIKDFLDIGGDEVDFMLCEIGGTVGDIEGLPFFEAIRQFSQDKPRGQCIFMHLTLLPFVKASGELKTKPTQHSVKELRSIGIAPDILVCRSEGPIPAKEREKLALFCNVRPDSVIAAQDLKSIYEAPLAYHREGLDQAVLDAFEITPAPKPNLTRWEDVADRIYNPEGEVKVAIVGKYTQLEDAYKSIAEALTHGGMANRVKVKVEWVDAEIFDSEDAGPHLEGFHAILVPGGFGERGTEGKIKAAQYAREHKVPYLGICLGMQMAVIEAARNVAGLKTAGSEEFDHEAGKKRFEPVVYHLKEWVQGNHKVARKVDDDKGGTMRLGAYDATLVEGSRVAEAYGTTTIDERHRHRYEVDIAYKEQLEKAGLKFSGMSPDGKLPEIVEWADHPWFIGVQFHPELKSKPFEPHPLFKDFVRAAKEVSRLVMPKTRLILGLESSCDDTAAAVVQIDDEGRGTVLSSVVAGQTALHADFGGVVPEIAARAHAEKLDHCVEDALAEASVPLSQIDAIAVTAGPGLIGGVVSGVMCAKGLSAATGKPLYGVNHLAGHALTPRLTDNVPYPYLMLLVSGGHCQFLLVRGPESFDRLGGTIDDAPGEAFDKVARLLGLPQPGGPAIERCAETGDPNRFALPRPLLDRDGCDMSFSGLKTAVLRTRDKCVADAGGLTRQDQADLAAGFQAAVVEVLAKKTRRAFASYPADAP--RGLCVAGGVAANKPIRAALEAVAAEEDAAFIAPPLALCTDNAAMIAFAAAEQSGLRSADDLTLSARPRWPLDTARPSMLGSGKKGAKA------------------------------------------------------------------------------------------------------------------------------------

>'Su-algicola1151'

MNAQELRDKTPDQLREELANLKKESFNLRFQQATSQLENTARMKAVRRDTARVKTILNQKAADAAAEAMAMADLLSMDNKKNADKQKALDSALAQIERQFGKGSVMKMG-DGAIQEIESTSTGSLGLDIALGIGGLPKGRIVEIYGPESSGKTTLTLHCVAEEQKKGGVCAFVDAEHALDPQYAKKLGVNLDELLISQPDNGEQALEITDTLVRSGAVSMVVVDSVAALTPKSELEGDMGDSSVGVHARLMSQAMRKLTGSIARSNCMVIFINQIRMKIGVMFGSPETTTGGNALKFYSSVRLDIRRIGALKDRDEVVGNQTRVKVVKNKVAPPFKQVEFDIMYGEGISKMGELLDLGVKAGVVDKAGAWFSYGDERIGQGRENAKTFLKENTRIALEIEDKIRAAHGLEFDDEAKDDNDVVEAMSQYNAGQIEKKWQQAWEQADTFKAVRSDDKPKYYVLEMFPYPSGRIHMGHVRNYTMGDVIARYKISTGHNVLHPMGWDAFGMPAENAAMEIGGHPKTWTYDNIAVMRDQMKPLGLSIDWSREFATCDPEYYGQQQALFIDMLDAGLIYRKNAVVNWDPVDMTVLANEQVIDGKGWRSGAEVERRELTQWFFKISDYSEELLDAIDTLDNWPSKVKLMQANWIGKSRGLQFAFSVIDGPDGHDRVEVYTTRPDTLMGASFVGISPDHPMAKHLEKDNADIAAFCAECRKIGTSEEALEKAEKLGFDTGLRVRHPFDTAWELPIYIANFILMDYGTGAIFGCPAHDQRDLDFARKYDLPVVSTYAPAVDNPEDGGAAYVPPKLEKVHYVKGFAGDEMQTGDQGIDTAIAFCEANGVGQGVTKFRLRDWGLSRQRYWGCPIPVIHCNNCGVVAEKKENLPVQLPDDVSFDIPGNPLDRHATWRDTDCPSCGKPAKRETDTMDTFVDSSWYFARFTAPDAATPTNMADADYWMNVDQYIGGVEHAILHLLYSRFFARAMHITEHLPKKAIEPFNALFTQGMVTHETYSTPDSNDRRVWHLPEDVSDDGPKANGTPVEIGPVIKMSKSKKNVVDPVNIISTYGADTARWFVLSDSPPERDVEWTASGAEAAAKHLSRVWRIASDIAQND--AAGSGDDDLRRAMHKAIFDVTQGIESFGFNASIAKLYAFTNTLQKSKAGSAAKREAAKTLAQLMSPMTPHLSEEIWTMLGGEGLIANTPWPVADEAMLVDDKITLPIQINGKRKSEITVTKDLSKDEVEKLALQDKAVVRALDGGQPKKMIVVPGRIINVVVMAITSANQLELLQTAEAVAREKMIDPGLVVEAMEESLARAAKSRYGAEMDIHVSIDRKTGKATFTRVRTVVEEEELENYQAEFTLEQAKQYLDDPKIGDQLIEEVPPVEMGRIAAQSAKQVILQKIREAERDRQFEEFKDRAGTIINAQVKREEYGNVIVDVGSGEAVLRRNEKIGRESYRPNDRIRCYIKDVRREQRGPQIFLSRTAPEFMAELFKMEVPEIYDGIIEIKAVARDPGSRAKIAVISYDSSIDPVGACVGMRGSRVQAVVNELQGEKIDIIPWNEDQPTFLVNALQPAEVSKVVLDEEAERIEVVVPDDQLSLAIGRRGQNVRLASQLTGLDIDIMTESDESERRQAEFAERTKLFMDTLDLDEFFAQLLVSEGFTSLEEVAYVEVDELLVIDGVDSDTASELQARARDCLEEMNKKAMEKARELGVEDSLVEFEGLTPQMIEALAEDDIKTLEDFATCADWELAGGWTTVDGERVKDDGVLEKFDVSLEEAQNLVMTARVMLGWVDPTELGKE------DAPEQEQEETEAMAHKKAGGSSRNGRDSAGRRLGVKKFGGEAVIPGNIIVRQRGTKWWPGAGVGLGKDHTIFAKVEGAVQFHKGLKGRTFISVLPVAEAAEMAKRWYSVSVLSNFEKKIAEQIRTSVAEAELEDQIEEVLVPTEEVIEVRRGKKVTAERRFMPGYVLVRMEMTDQGYHLINSINRVTGFLGPQGRPMPMRDAEVNAILNRVEEGEAAPRTLIHFEVGEKVKVNEGPFEDFDGMVEEVDEDNQRLKVTVSIFGRATPVELEFTQVTKQIMSFTLAIVGRPNVGKSTLFNRLVGKRLALVDDQPGVTRDLREGDARLGDLRFTVIDTAGLEEVTDESLQGRMRRLTERAVDMADICLFMIDARAGVTATDQLFAEILRKRSKHIILVANKGEGRAADAGMIEAYSLGLGEPLRLSAEHGEGMSDLLSVLMPLADGMAKQHETIEPETDIDIDED------SPEITRTKPLQIAVVGRPNAGKSTLINKILGEDRLLTGPEAGITRDAISLKIDWDG-LPVRIFDTAGMRKKAKVQDKLEKMSVSDGLRAVKFAEVVVVLLDAEIPFEQQDLRIADLAEREGRAVVIAVNKWDVETEKQSKLKELLEEFERLLPQLRGAPLVTISAKTGRGIDRLKGAIERAYDVWNRRVTTAQLNRWLLAMVDAHPPPAPGGRRIKLRYATQVKTRPPGFVVMCSLPEKLPESYSRYLVNGLREDFDMPGTPIRLTMRSQADQNPYKDKKKSTPSRLRKHLGKKPLSMKVIFMGTPDFSVPVLDALMQAGHDICAVYCQPPRRAGRGKKERLTPVHARAEALGLTVRHPISLRNADAQAEFADLNADIAVVVAYGLILPQPILDAPTYGCLNIHASLLPRWRGAAPIHRAIMAGDAETGICIMQMEAGLDTGPVMLHESIPISDTETTGDLHDRLSKMGAGLIVKALADLPNLTPVVQSANGVTYANKIDKSEAQINWTKPAVDVDRLIRGLSPFPGAWTTLHGKRLKVLRSRVVEKNGMPGTVA-SGLTVCCGTGAIELLDVQFEGRSRQSQKEFLNGTQILPDTVLGENMSETTKYQVLARKYRPETFADLVGQDAMVRTLKNAFEADRIAQAFIMTGIRGTGKTTTARIIAKGMNCIGPDGTGGPTTDPCGQCEHCVSIMHGQHVDVIEMDGASQTKIDDIRNVLDSVYYAPASARYKIYIIDEVHMLSNSAFNALLKTLEEPPAHVKFIFATTEIRKVPVTVLSRCQRFDLRRIEPEDMIAMLRRIATAEGAQISDDALALITRAAEGSARDAQSLLDQAISHGAGETTADQVRAMLGLADRGRVLDLFDMIMRGDAAAALTELSGQYADGADPMAVLRDLAEITHWISVIKITPEAAEDPTIGPDERMRGQDMAAKLPMRVMTRMWQMLLKALEEVAMAPNAMMAAEMAVIRMTHVAELPSPEDLVRKLQD-TPPPA---APSGTSQPAPMGGSPAAAGASASAVTGSVTA-QATALAPQVDAGLERYARFEQVVDLIRANRDVKLLVEVETCLRLAKYSPGRIEFQPTDNAPGDLAQRLGSRLQAWTGNRWAVSIVQDGGAATIAEERDAQDTALKSKAAEHPLVKAVFDAFPDAEIIEIRTPKAIADAAATDALPEVDDEWDPFEEDMPKMKTKSSCKKRFKVTAKGRVKAGQAGKRHGMIKRSTKFIRDARGTTILSAPDEKIVKSMMPYSRMNLFASIRDLVTDSLTVLQSQGVLPAGLDFANVTVEPPRDATHGDMATNAAMVLAKPAQMKPRDIADALAAKLTDDPRIDLVEVAGPGFLNLRLANGVWQNVVKTALTDPT-FGQSQLGQGKRVNVEYVSANPTGPLHVGHTRGAVFGDALASLLSYAGYGVTREYYINDGGAQVDVLARSVYLRYLEAHGQQVAFEDGTYPGDYLIDVGQALKDKVGDQFVDKGEQYWLAEIREFATEKMMDLIRSDLKSLGVEMDVFFSEKALYGTGRIEEALQALDDKGLIYQGVLEPPKGKTPEDWEPREQTLFKSTEHGDDVDRPVKKSDGNWTYFAPDIAYHYDKIDRGFDQLIDVFGADHGGYVKRMKAAVSALSDGKVSVDIKLCQLVKLYKDGQPFKMSKRAGTFVTLSDVVDLVGPDVTRFVMLTRKNDAPLDFDFSKVLEQSKDNPVFYVQYAHARVCSVLRKAVEADIASDDATLSEVDFSKLDHEAELAVARKIAEWPRLVEIAARNNEPHRIAFYLYELASEFHALWNRGNDVPSLRFVQEGDAATSQAKMALARATAIVISAGLGILGVEPVQEMRMPTIDTLPPLRDVITTHGLSAKKSLGQNFLLDLNLTAKIARQAGDLTHCDVLEIGPGPGGLTRGLLAEGARRVLAIEKDSRCLPALAEIAATYPDRLHVINGDALDINPLDHLTPPIRIAANLPYNIGTELLVRWLTPPTWPPFWDSLTLMFQREVADRIVAKPGSKAYGRLAILAQWRADARIVMTLPPEAFTPPPKISSAVVHLTALPQPRYAADPAVLSRVVAMAFNQRRKMLRSSLKGLALDIEDRLVAADITPTDRAETLTIEQFCTLARQLN--MNLIAELEAEQVASLGNDIPDFKAGDTIRVGYKVTEGTRSRVQNYEGVCISRKNGEGIAGSFTVRKISFGEGVERVFPLHSTNIDSIAVIRRGRVRRAKLYYLRTRRGKSARIAEDTTYKPKKA---MANSKRDLFLKRRLRVRNKLRKRNTTGMRLSVHRSNKNISAQLIDDVNGVTVASASSLEKDLGVVGKNNVEAATKVGEALAKRAKKAKVSECYFDRGGFLFHGKVKALAEAAREGGLKFMFAVLKTGGKQYKVSSGDVLRVEKLAADAGETVQFNDILMIGS---TIGAPFVKGAGVQAEVIDQIKGDKVIHFVKRRRKHGSKRTKGHRQQLTLLRITDLMEKGADKSGVKAAMGAGSVSAAAVSADAKKADAKPAKKAKKAEPKKEPKKAVESATGAGKARGKPDDLKKISGVGPKLEGLLHDNGVFHFDQISAWTADEIAYMDDQLSFKGRIERDGWLDQAAKLAAEMSRTKGGTTTHARHKKVTDAAKGYYGRRKNTFKVARQAVDKANQYATRDRKNRKRTFRALWIQRINAAVRSHDEALTYSRFINGLALAGIEVDRKVLADLAVHEPEAFGAIVDQAKAALA-MQVTETLNDGLKRGYSIVVTAGELDEKVNEKLVEAQPDVEMKGFRKGKVPMALLKKQFGQRLLGEAMQETIDGAMTKHFEDSGDRPAMQPEVKMTNEDWKEGEDIHVDLSYEALPEIPEVDMKSIKLEKLVVKADDAEVDEALKNLAETAQDFDDRKKGSKAKDGDQIVIDFLGKVDGEAFEGGAADDYPLVLGSNSFIPGFEDQLVGAKVDDEVEVKVTFPEEYGAANLAGKDAVFTCKVKAVKAPKAAEINDELAKKFGAEDLAGLKAQIGERLEAEFAGAARAVMKRSLLDALDKKVSFELPPSLVDAEAGQIAHQLWHEDNPDVEGHDHPEIETTDEHKKLADRRVKLGLLLAELGQKAEVQVSDAEMTQAIMNQARQYPGQERQFFEFVQQNAQMQQQLRAPLFEDKVIDYIFELAKVSEKEVSKDDLQKAVEALEEEMRLNELHDNPGATKKRKRIGRGPGSGTGKTGGRGIKGQKSRSGVAIKGYEGGQMPLYQRLPKRGFNKPNRKKFAVVNLGLIQKFIDAKKLDTKGEITEDKLIESGLVRRKLDGVRILAKGDFSAKIKLSVTGASKSAVEVVEKAGGSLNVTTAAATE---MIQMQTNLDVADNSGARRVQCIKVLGGSKRKYASVGDIIVVSVKEAIPRGRVKKGDVRKAVVVRTAKEVRREDGTAIRFDRNAAVILNNNNEPLGTRIFGPVVRELRAKNFMKIISLAPEVLMRHARGYRRLNRTHEHRKALWANMAGSLIEHEQIKTTLPKAKELRRIVEKLITLGKRGDDHARRQAASQLKQHQYVEKLFDVLGPRYKERQGGYVRVLKAGFRYGDMAPMAIIEFVDRDVSAKGAADKARVAAEDTAE-MLQPKRTKFRKMHKGRIRGEAKGGSTLNFGTYGLKATEPERVTARQIEAARRAMTRHMKRQGRVWIRIFPDTPVTSKPTEVRMGKGKGSVDFWAAKVKPGRVMFEIDGVNDAVAREALRLAAMKLPVKTRVVVREDWMTTIKLTNTKTRKKEVFQPLDPKNVRMYVCGPTVYDRAHLGNARPVVVFDVLFRLLRHVYGADHVTYVRNFTDVDDKINARAAETGRSIENITAETAQWYLEDMAAIGAMEPTHMPRATQYIPQMVAMIEGLVAKGHAYEAEGHVLFAVESYENYGKLSGRSIDDMMAGARVEVAPYKRNPMDFVLWKPSTDDLPGWDSPWGRGRPGWHIECSAMADDLLGETFDIHGGGIDLQFPHHENEIAQSMCAHPKGDFARYWLHNEMLQVEGKKMSKSLGNFFTVRDLLDQGVPGEVIRFVMLSTHYSKPMDWTEKKREEAERTIRKWYDITQGVKPSIP-HECILDSLSDDLNLPLAIYYLHSFFDSDDLRGVFASAQLLGLLTDDLGAW-DIPSVDLSAYAKRLTELRETAIKTKNFVVLDAMKSALAEAGAEVRMGKTQIEVIAGPNLDVAKLEVLKMAQ----TKTDPNYKVIAENRRARYDYAIEEDLEVGIMLMGSEVKSLRGGQANIAESYVAVENGELWLTNSYVPLYSQAKTFGHEERRKRKLLASRREVARMWQATSRQGMTIVPLVMYFNHKGLVKLKIGIAKGKKNVDKRATEAKRDWGRQKQRLLRHGDMSVKPEHYDVIRKPIITEKATMASENGAVVFEVSMAANKPQIKEAVETLFGVKVKSVNTTITKGKVKRFRGQLGKRKDVKKAYVTLEEGNTIDVSTGLMGKDKNPRRVAENEAMAKTRMLRTSPQKLNLVAAMIRGKKVDKALNDLTFSKKRIAEDVKKCLQSAIANAENNHNLDVDELVVAEAYVGKNLTMKRGRPRARGRFGKIIKPFSELTIKVRQVEEQAMASAAEQMAANISWGALSKATELRQRIFFTIGLLIVYRIGTYIPVPGIDAEALRNFMDQAQSGIAGVLGMFTGGALSRMGILALGIMPYISASIIVQLLTAMVPQLEQLKKEGEQGRKKINQYTRYGTVILATFQAYGLAVSLEAGELVTDPGWFFRASCVITLVGGTMFLMWLGEQITARGIGNGISLIIFVGIVAEIPAALAQFFSQGRSGALTPAVIVGVIIMIFATLVFVVFMERALRKINIQYPRRQVGMKMYDGGSSHLPVKVNPAGVIPAIFASSLLLLPTTVSTFSGQDAGPIMATLLAYFGPGQPLYLLFFATMIIFFTYFYTFNVSFKTDDVADNLKNQNGFVPGIRPGKKTAEYLDYVVSRLLVLGSGYLALVCLLPEMVRSQLSITAYFGGTSILIIVSVGMDTIQQVQSHLLAHQYEGLLEKSQLRG---RKRKKKGPARKMKFLDLTKVYIRSGAGGGGCVSFRREKYIEYGGPDGGDGGRGGDVVAEAVEGLNTLIDFRYQQHFFAKNGVHGMGKQRTGADGADIVLKVPVGTEILDEDEETVIADLTEVGQRVILAKGGNGGFGNLHFKSSTNRAPRRGNPGQEGVERTLWLRLKLIADAGLLGLPNAGKSTFLAATSNARPKIADYPFTTLHPNLGVVGVDNAEFVMADIPGLIAGAHEGRGIGDRFLGHVERCSVLLHLVDGTSDTIAEDYHTIIGELEAYGGHLADKPRVTALNKIDALDDDERAAAKDELEDAVGAPVLMMSGVSREGLTEVLRAVRAEIQQDRLRIANAVEPEPWRPMAAKLKKGDKVIVLAGKDKGKEGTITSVNPKTGKAIVDGVNISVRHTKQSQATQGGRVPQAMPIQLSNLAIIDKDGKPTRVGFRMDGDKKVRFAKTTGDVIDAMLRSGVIAKKVGMTRLFMEDGKQIPVTVLQLDKLQVVAQRTAEKDGYSAVQLGAGTAKAKRTSQAMRGHFAIAKVEPKRKIAEFRVAPENLINVGEEITADHYFEGQFVDVAGTSIGKGFAGAMKRHNFSGLRASHGVSISHRSHGSTGQCQDPGKVFKGKKMAGHMGAARVTTQNLQVVKTDAGRGLIMVKGAVPGSKGGWVTIKDAVKKPFPENAILPAALKSAAEEAAKAAEEAAAQAAAEAEAAEKAAAEAAAAEQAAALKDAEASIEADKADDAAPEGGDKDES--MALKSYKPTTPGQRGLVLIDRSELWKGRPVKALTTGLTKNGGRNNTGRITMRRKGGGAKRLYRIVDFKRNKMDVSATVERIEYDPNRTAFIALIKYDDGEQAYILAPQRLAIGDKVIASSKADIKPGNAMPFQGMPIGTIIHNIELKPGKGGQIARAAGTYAQFVGRDGGYAQIRLSSGELRLVRQECMATVGAVSNPDNSNQNFGKAGRNRHKGIRPSVRGVVMNPVDHPHGGGEGRTSGGRHPVSPWGKPTKGARTRNKNKASSKLIIRSRHAKKKGRMLDTANYTPRFKAAFAETIRAAMKEEFGYKNDMMIPRLDKIVLNIGAGSEAVKDSKKAKSAQADLTAIAGQKAVITKAKKSIAGFRVREDMPLGAKVTLRGDQMYEFLDRLITIAMPRVRDFRGVSGKSFDGRGNYAMGLKEHIVFPEIDFDKIDEAWGLDIVICTTAKTDAEAKALLKLFNMPFNSMKADVIKLDGKKAGSADLDDAIFGLEPRVDILHRVVRWQRNNAQQGTHKVKTRSEVDYSTKKIYRQKGTGGARHGARSAPIFRGGGIYKGPTPRSHGHELTKKFRKLGLRHALSAKANAGELVVIDSIDKDLKTGALAKQIKELGWKRALVIDGAEVNENFAKAARNIEGLDVLPSMGANVYDILKRDTLVITKAGVEALEARLKMENVVLIILLILALALIVVVLLQRSEGGGLGMGGGGGGVMSGRSAATALGKLTWFLGISFMVASLAMTIISSNNSSGSSLFD--VPPPASDTPAAVPSIPLDGPILPPSENDNAPIVPRLDMAFFKKLKDRMFKSSSKLEEGLDAIVEEGPTE---------------------------------------------------------------------------------------------APDSGEETSIAEPEEKPGILGRLLGKVEDSTAPRRVLDDDMLEQLEELLISADMGVDTAMRVTANMAEGRFGKKLSVNEIKGLLADEVTRVMDPVAKPLPLYPKTPQVVLVVGVNGSGKTTTIGKLASQFRAAGKNVVIAAGDTFRAAAVEQLQVWGDRAGVPVLTAPEGSDPASLAFDAMTKAETDGADLLLIDTAGRLQNRADLMEELAKIVRVIRKKDETAPHNTLLVLDATTGQNAINQVEIFQKLANVSGLVMTKLDGTAKGGVLVALADKFGLPIHAIGVGEQIDDLDAFDPEDFAKALTGLDAMTNETWGQVRKELINTVGKNNYTTWIEPLEFHDLSDGVATFTVPTSFMGNYVSQNFGDQIKFQLKMSGAEVNRIAFQVANSTTRPA-KPAVAR--THASAQGSD-----LTGAPLDARYTFDGFVVGKPNELAHAASKRVAEGGPVTFNPLFLYGGVGLGKTHLMHAIAHELQTRRPELNVLYLSAEQFMYRFIQALRVNKMMDFKELFRSVDVLMVDDVQFIAGKDSTQEEFFHTFNALVSANKQIIISADRAPGEIKDLEDRIRSRMQCGLVVDLHPTDYELRLGILQSKVMQYRDQYPNIELNHGVLEFLAHRISKNVRVLEGALTRLFAFASLVGKPVTMELTQDCLADVLRDSDRKVSVEEIQRKVSEHYNIRLSDMIGPKRVRTFARPRQVAMYLCKQLTRRSLPEIGRRFGGRDHTTIMHGVRRIEELITTDSQIADDLELLRRALEEMLGIGTVAKKVFGTPNDRKIKATRPLVEQINALEPEYEALSDEGLKQKTEELATRAMNGENLDDLLPEAFANCREGARRALGLRAFDTQLMGGIFLHQGNISEMKTGEGKTLVATFPAYLNALTGKGVHIVTVNDYLAQRDSEWMSKVYNALGLTCSVVYSMQPDDQKKAAYSADVTYATNNELAFDYLRDNLKSSLRDMSQRGHNFAIVDEVDSILIDEARTPLIISGMAEDRSDLYGTLDKLIPELTDEHFTLDEKQKNVTFTEEGNEFLEARLHELGILPEDQSLYDPESITIVHHANQGLRAHRMFEKDKDYVVRDGEVVLVDEFTGRMTPGRRLSQGLHQAIEAKEGVEIKPENVTQASVTFQNYFRLYNKLSGMTGTALTEAEEFKEIYGLGVVEVPTNRPIARTDEDDKVFRTAREKYAAIAEEIKMAHEKHQPVLVGTTSIEKSEFLSNLLTQAGVPHNVLNARQHEKEAQIVADAGKLDAVTIATNMAGRGTDIQLGGNVEMRVLQALTADPEADPDQVRSRIEAEVAEEKEKVKEAGGLFVLATERHESRRIDNQLRGRSGRQGDPGRSSFFLSMEDDLMRIFGSERLEKILSALGMKEGEAIVHPMVNRSLEKAQAKVEGRNFDMRKQLLKFDDVMNDQRKVIFGQRREIMETEDVSEITQDMRHQVIDDLIDEFMPPKTYADQWDTKGMQEAVLERLGLDAPVADWAAEEGVDDEVMCERLEKAGDEFMASKAAQFGPENMRQVEKQVLLQIIDAKWREHIVVLEQLRSVIGFRGYAQRDPLNEYKTESFQLFESLLDSLREEVTQKLANIRPVTPEEQQAMLDQFKAQQLAMAQATEAA-----AAQT----AENGFVEDDPSTWGNPGRNDQCPCGSGKKFKHCHGRLAMKLAADLQAFAALIGHDFKRPEILIRAVTHASISSPTRDDNERLEFLGDRVLGLVMAEALLEADRNASEGQLAPRFNALVRKETCADVARQINLGDVLKLGRSEMISGGRRKEALLADAMEAVIAAVYRDAGFDAAKALVLRLWGDRINRVEADARDAKTSLQEWAQARGQTPPAYVELSRKGPDHAPIFTIEVQLSSGQTAIATAGSKRHAEQAAAKALLEKVQSE-MAKYGKRTTAAREAFAGKTDVTVEEAVALIKGNAKTKFDETLEIAMNLGVDPRHADQMVRGVVTLPNGTGKTVRVAVFARGPKADEAKEAGADIVGAEDLMEIVQGGKIEFDRCIATPDMMPIVGRLGKVLGPRNLMPNPKVGTVTMDVADAVKAAKGGEVQFKVEKAGVVHAGIGKVSFDESKLVENIRAFVGAVAKAKPAGSKGAYMKKIALSSTMGPGVTVSVDNATGNMSEDFELDTDDLQRRMDGAMASLRTEFASLRTGRASASMLEPVMVDAYGSMTPINQVGTVNVPEPRMVTINVWDKGLVSKVEKAIRESGLGINPQLNGTIIMLPIPELNEERRKELTKVAGTYAENARVSIRNIRRDGMDQIKKAKADGMSEDDQKLWEDEVQELTDKMISAIDEALETKQSEIMQVMAKKIAGTMKLQVPAGQANPSPPVGPALGQRGINIMEFCKAFNAKTQDLEPGSPCPTIITYYQDKSFTMDIKTSPASYFLKKAAKLKSGANNPSREVVGSVTVAQVREIAEAKMKDLNANDIEGAMKIIVGSAQSMGIEVKMFENLSERLSGVFDKLTKQGALSDEDVKTALREVRVALLEADVSLPVARDFIKAIQDKATGQAVTKSVTPGQQVVKIVHDELVHVLAGDEDPGALKIDNPPTPILMVGLQGSGKTTTTGKLAKRLTEKNGKRVLMASLDIYRPAAMQQLAVLGTQIGVDTLPIVAGETAVQIAKRAKQQATLGGYDVYMLDTAGRLQIDQTLMQEVEDVRDAVNPRETLLVVDGLTGQVAVEVAEEFDGKIGISGVVLTRMDGDGRGGAALSMRAVTGKPIKFVGLGEKMDALEEFHPERIAGRILGMGDIVSLVEKAQETIEAEKAERMMKRFQKGQFNMNDLKMQLDQMLKMGGMEGIMGMMPGAGKMAKQAEKAGLDDSILRRQIALINSMTKKERANPQILQASRKKRIAKGAGLEVSELNKLMKQHRQMSDMMK---KMGKGGMLKKAMAGMFGKGG--PS-------EAEIAAAQKQM-GQMPGGLPGLGGGMQLPPGLSGFGKKK--------------------MADGS--GDIAKLAKEYSDMKPVVEKIRSYRQLLDDIAEAEVMLADPEMKELAEDELPTLKARQPALEQALRLALLPKDAADARPAMIEIRPGTGGDEAALFAGDLLRMYQRYSETKGWKFEIIEEQATELGGIKEVTAHVQGDGVFAKLKYESGVHRVQRVPTTESGGRIHTSAATVAVLPEAEDVDIQVDAQDLRIDTMRASGAGGQHVNTTDSAVRITHIPTGLVVTSSEKSQHRNREIAMQVLKTRLYDLERQRVDNERSADRKAQVGSGDRSERIRTYNFPQGRMTDHRINLTLYKLDQIMQGDLDDIVDALTSEDQANLLAEMEAMDRAQKEKVVDELGQIFESSGVVVVAHYEGLTVAEMQDLRARMRDVDGSVRVAKNRLAKIALDGKPCASIADLLSGMTVLAYSEDPVAAAKVMQAYSKDNDKLEILGGAMGETALDVAGVKAVAAMPSREELIASIVGCIGAPASNIAGAIGAPASNIASILSSIEDKAAAMKTFSATPADIDKKWILIDAEGVVLGRLASIVAMRLRGKHKASFTPHMDMGDNVIVINADKIQLTGKKRDKPNYWHTGYPGGIKSRTTGQILEGAHPERVVMQAVKRMLPGNRLSRQVMTNLRVYPGADHPHEAQNPDVLDVKSMNKKNTRA-----------------------------------MALLHLMAQYSARTGVFLQSVTVDHRLRKGAADEAEMVGRAAAKLGVPHHILGWDDWDGRGNLQSQARQARYDLIGQWAQDNQILHVALAHTLDDQAETVLMRLARGSGVDGLSGMHPMRQHGPVAWLRPLLWAKRQELRDYLTGIDQPWADDPSNTDDRFDRVKARQIMGLLADLGLTQDRLAQTASHMAQARYALEIATQDLARRIVTLRAGNVVFQQNDFLDAPGELRDRLLAHGLGWVSRQFYKPRYKALSQVVDAVRNGQQSVLHGALIIPKKGQIWITREFKAVSDLRCSTDQMWDNRWRVSGPN--TNDHEIRALGETGLAQRPDWRDLQIPRNALISSPSIWHGSDLISAPIAGFVNNWTIKSIKSDNDFFTAIITHMADLKKLAEEIVGLTLLEAQELKTILKDEYGIEPAAGGAVMMAGPADGGAAAEEKTEFDVVLKNAGASKINVIKEVRSITGLGLKEAKELVEAGGKIKEGVDKAEAEDIKGKLEAAGAEVELAMSRIGKKPVELPSGVTASVSGQTVEVKGPKGTRSFTATDDVTLTVEDNVVTITPRGKSKRARQQWGMSRTMVGNLVTGVTDGFKKELEINGVGYRAQMQGNTLKLQLGLSHEVNFEVPQDVTVTAPKQTEIIVEGIDEQLVGQVAANIREWRKPEPYKGKGIKYKDEYIFRKEGKKKMQVILLERVAKLGQMGEVVDVKPGYARNFLLPQGKALTASQANITQFEEQKAQLEARNLETKKEAEDMAAKLDGQQFIVIRSASDSGSLYGSVTTRDAADAATADGFSVDRKQVALIEPIKELGLHDVKVSLHPEVEATITLNVARSQEEAELQASGKSIQELAAEEEAAAEFEISELFDDIGGAALDAEDAASV---EAQDAEETDEDDKMSEDDGKKTLGLRGGPRSGNVKQSFSHGRTKSVVVETKRKRVVVPKPGGAKASGNSSV--GDPSKRPAGISDAEMDRRLKALQAARARESQEAAEREAAEKAREQDRKRRREEQEIKEREEREREERAKAKVEEEERKKREVEETAKAAAQAA--PAKEVTTPSRAAP----REENNNDRNNRGKGRGDDSGRRSGKLTLNQALAGGEGGRQRSMAAMKRKQERARQKAMGGNEPREKVMRDVQLPEAIVVQELANRMAERVADVVKALMTNGIMATQNQTIDADTAELIIEEFGHKVVRVSDSDVEDVIHQIEDKDDELQPRPPVITVMGHVDHGKTSLLDAIRNAKVVSGEAGGITQHIGAYQVE-QQGHKLTFLDTPGHAAFTSMRARGAQVTDIVILVVAADDAVMPQTIEAINHAKAAQVPMIVAINKCDKPDANADKVRTDLLQHEVIVEKMSGDVLDVEVSAIKGTGLDQLLENILLQSELLDLKANPDRAASGAVIEAQLDVGRGPVATVLVQNGTLKQGDIFVVGEQYGKVRALINDQGDRVTEAGPSVPVEVLGLNGTPEAGDVLNVVDTEAQAREIAEYRENAAKDKRAAAGAATTLEQLMANAKADEDVSEMPILVKADVQGSAEAIVQAMEKIGNDEVRVRVLHSGVGAITETDIGLAEASGAPVFGFNVRANATARNVANQKGVEIRYYSVIYDLVDDVKQAASGLLSAEIKENFIGYAEIREVFKVSGVGKVAGCLVTEGVARRSAGVRLLRDNVVIHEGTLKTLKRFKDEVSDVQSGQECGMAFENYDDIRTGDVIEIFEREEIERSLDMAKQKKQPRPKAITPKGFRDYFGTEVTERAEMLQKIAGVYHRYGFDALESSAVETVEALGKFLPDVDRPNEGVFAWQEDDAKEG-QDWLALRYDLTAPLARVFAQYRNDLPTPYRRYAMGPVWRNEKPGPGRFRQFYQCDADTVGTSSMAADAEICAMLADCLEEVGIPRGDYVIRVNNRKVLEGVLEAVDLANDDMGEWQANILRTIDKFDKVGEQGVRDLLGEGRKDASGAFIDGIGLSDAQSEPVVAFLTSKGVNNAETLQNLSSAVGSSEIGYEGIRELEEIIALLNAQNYQADRIIIDPSVVRGLGYYTGPVYEAELTFEITDDKGRPRQFGSVAGGGRYDDLVRRFTGQEVPATGVSIGVDRLLSALHAKGRMDQTAKGPVVVTVMDRSRMADYQAMVAELRTAGIRAEVYLGNPKNFGNQLKYADKRNSPAAVIQGSDEHDKGIIQIKDLILGAKIAENATYEEWKERPSQFEVPRDQLVAKIREILDGQDMDDLRDKYLTAISGAADEAALEDLRVQAVGKKGEISLKMRELGKMTAEERQTVGPKLNALKDEINSALAAKKAGLADAALDERLRTEWLDVTLPGRPRRQGTIHPISQVWEEATVIFADMGFAVAEGPQIESDWYNFDALNIPGHHPARAEMDTFYMHRADGDNRPPHVLRTHTSPVQIRSMQDQGAPLRIICPGRVYRADYDQTHTPMFHQVEGLAIDKDISMANLKWVLEEFCRAFFEVDNVELRFRASHFPFTEPSAEVDIRCSWDGGQLKIGEGDDWMEILGSGMVHPKVLQAGNIDPNEWQGFAFGMGIDRIAMLKYGIPDLRAFFDSDLRWLRHYGFSSLDQPTLHGGLSG--MADEVGLDLVEISPNATPPVCKIMDFGKFKYETQKKEAEARKKQKIIEVKEVKFRPNTDTHDYEVKMRNVFKFLENGDKVKITLRFRGREMAHQNLGRELLERVAEDTKEHGKIENMPKMEGRQMVMMIGPMPKMKFTLSWLKDHLETDASLDDILYALTDLGLEVEEVLDPTAKLKDFTIGKVLSAEKHPDADRLRVCQVETDEGVKQIICGAPNAREGITVVVAKPGVYVPGIDTTIGVGKIRGIESFGMMASEREMELSDEHDGIIELPSGAVGESFANWLAANEPAKVDPVIEIAITPNRPDALGVRGIALDLAARGLGTMKPRDLEPVEGQFPCPIAVTIDYDTREACPVFYGRVIRGVKNGPSPQWLQDILRAIGLRPISALVDITNFFTYDRNRPLHVFDADKVQGNLRVHRAKGGETLVGLDEKEYTFQPDMTLISDDSGIESIAGVMGGLPTGCTEETVNVFLEAAYFDPVRTALTGRALKINSDARYRFERGIDPAWTPHGLEHATRMILDLCGGEASDVVIAGEIPDVSRAYKLDTDRVQSLVGMDIPAEDQRATLERLGFWLDGDMAHVPTWRPDVMGEADLVEEVARIASLTKLQGKPLARAQVGVPKPVLTPMQRREQAARRTCAALGYNECVTYSFIDKASAELFSGGDDATMLANPISSEMSHMRPALLPGLLQAAARNQARGFMDLALFEVGPAFHGGEPDEQHLLVTGLLIGSTGPKDVHGSARPVDVYDVKADADAVLSAIGAPAKVQILRGAQEWWHPGRHGMICLGPKKVLGIFGELHPKVLSAMDVKGPALAFTIWPGEVPLPRSKSATRPALEITDLQAVERDFAFVVNADVEALTLVNAAAGADKGLIEDVRVFDEFIGGALGEGKKSLAITVRLQPREKTLKDEDIEAVSVKIVEKVTKATGGTLRGMHAYRSHTCAELNKTHVGETVRLAGWVNRIRDHGGLLFIDLRDHYGVTQILADPDSPVFAQVEKVRSEWCIRIDGQVKARDESLINPNIATGEVEVFIRDIEVLGASDELPLMVFGDQEYPEETRLRYRYLDLRREKLQDNMKLRSDVVASIRKRMWDIGFREYQTPIITASSPEGARDFLVPSRLHPGKFYALPQAPQQFKQLIMMSGFDKYFQIAPCFRDEDPRADRSPTDFYQLDMEMSFVEQQDVFDTIQPVLQGVFEEFGSGRKVDADWPQISYRDAALWYGTDKPDLRNPIKMQVVSDHFRDSGFAIFAKLLEQDGTQIRAIPAPGGGSRKFCDRMNAFAQKEGLPGMGYIFWRKKDSQSELTEAAGPLAKNIGPERTEAIRRQLGLGVGDAAFFLGGKPESFETVAAKARVEIGNELGLTDHDRFAFAWIVDFPIYEKDDETGKVDFEHNPFSMPQGGMEALAGDPLDVLGYQYDLACNGYELVSGAIRNHKPEIMFKAFEIAGYGEDEVRKRFGGLVNAFQYGAPPHGGCAAGIDRIVMLLADEANIREVIMFPMNQRAEDLMMDAPSEPMNEQLRDLSLRVIDPDMTELSKIRNFSIVAHIDHGKSTLADRLIQETGTVKDRDMKAQLLDAMDIERERGITIKANTVRIDYKADDGQNYVLNLIDTPGHVDFAYEVSRSMRAVEGSLLVVDSTQGVEAQTLANVYQAIDADHEIVPILNKIDLPASDLDRVAEQIEDVIGIDASGAIPVSAKTGQGIHATLEAIVTHLPAPKGTRDAPLKAMLVDSWYDAYLGVIVLVRIIDGVLKKGDRIKMMSNGTIHPVDRIGVFRPQMDVIDELGPGEIGFLTASIKQVRDTRVGDTITHERKGTDTALPGFKPSQPVVFCGLFPVDNAEFEDLRDAIEKLALNDASFSFEMETSAALGFGFRCGFLGLLHLEVIRDRIEREYNIELITTAPSVIYHVYMRNDTMIELHNPADMPDLTHVDHLEEPRIKATILVPDDYLGDVLKLCQDRRGIQMDLTYAGSRAMVVYDLPLNEVVFDFYDRLKSVTKGYASFDYQMIGYRQDHLVKMQILVNDEPVDALSTMVHRDRAEARGRAMCEKLKDLIPRHMFKIPIQAAIGGRIIARETLAALRKDVTAKCYGGDVSRKKKLLDKQKAGKKKMRQFGKVDIPQEAFISALKMDGMSLPPGFLDELRTRISLTQVVGRKVMWDTRKSNQGKGDMWAPCPFHQEKTASFHVDDRKGFYYCFGCHAKGDAISFVRETENVGFMEAIEILARETGMPMPERDPQAQEKADKRSELAQVMEQAVQYYRLQLKTAAAQDAREYLTRRGLSEATQGRFDIGFAPDMRQGVFQHLTSKGVEADLVVDAGLAARPDDGGEPYDRFRGRIIFPIRDPRERCIGLGGRAMDPNARAKYLNSPETELFDKGRSLYNHGPAREAAGKGVPLVVAEGYMDVIALSEGGFEATVAPLGTAITEDQLRLLWRIAPEPIIALDGDKAGLRAAQRLVDLALPMLEAGQSLRFAIMPDGQDPDDLIRAQGAGAFQKLLDNALPMVQLLWRREIEGKVFDSPERKASLDKALREKIKLIRDPSIRSHYGEEIKRLRWDLFRPVRDFP---------KGRG-WQAPDV-PLPTTKNSALAA-GDGTFEDQLREAVILASLVLNPDILPEFESQLERMDCFSADHAVLRDALLRHVGLFTQKMRD----EIGGEALEKLFAQRHVAIAPPVRKAGNSEVAIMCVAEEFAKLDARRGHQREIDEAVEDLSGIADEGLTWRLSQAAHAMNRAGKTNNDDKADYELGPNGAKVKRDERTALDDLLGKIDFAKGGPRHMCADMPDYKDTLNLPQTDFPMRAGLPKREPDWLARWEKIGVYDKLRDKDGREPFTLHDGPPYANGHLHIGHALNKILKDMVVRSQQMMGRDARYIPGWDCHGLPIEWKIEEKYRQKGKDKDDVPVIEFRQECRDFANGWVDIQREEFKRLGITGTWDNPYLTMNYRAERIIAEEFQKFLMNGTLYQGSKPVMWSPVEKTALAEAEVEYHDKESFTVWVKFSVAEFHHAKLLLEKAFVVIWTTTPWTLPSNKAVVYGEDISYGLYRVDHSPVDNWAKQGEHYILADKLAGDVLSRARLEKGDYTRVSGVSHHELARMKLAHPLANCSGSNGEWDDTRDFRAADFVTDEEGTGFVHCAPSHGMEEYELYRDLGMLSEVITYNVEDDGKFRDNLPFFGGKYILSRKGGEGDANKAVIEKLVEVGGLLARGKIKHSYPHSWRSKAPIIYRNTPQWFAAIDRKIGDGQDTYGTTIRERALNSIDQVTWTPQTGRNRLYSMIEARPDWVLSRQRAWGVPMTCFIKKGALPTDDDFLLRDGAVNQRILDAFEAEGADAWYADGAKERFLGNDHNPDDYNQIFDILDVWFDSGSTHAFVLRDREDGSADGLADLYLEGTDQHRGWFHSSMLQSCGTQGRAPYRGVLTHGFTLDAKGNKMSKSLGNTVAPEKVVQQYGADILRLWVAQSDYTADLRIGDEILKGVADSYRRLRNTMRFMLGSLAQFSEADRMDPADMPELERWVLHRLAELDQTVRDGYTKYDFQGVFQAIFNFATLDLSAFYFDVRKDVLYCDGDTDRRRAARTVLDILFHRLTTWLAPVLVFTTEEIWLERYPGEDSSIHLTDIPETPADWRDKDLAAKWATIRKVRRVVTGALEIERREKTIGASLEAAPIVYVD-TETAAVLATVSFDDLCITSGIHVSTDATPDDAFTLDGADGIGVKFAAAKGEKCQRCWKILPDVGTHAHTAVCARCDAALG-MQSQNIRIRLKAFDYRVLDASTQEIVNTAKRTGAQVRGPIPLPNKIEKFTVLRGPHVDKKSRDQFEIRTHKRLLDIVDPTPQTVDALMKLDLAAGVDVEIKV--MADEIKSLDELNAVAE----GTE---VEVAAPREPVRDELGRAYATGKRKDAVARVWIKPGSGKVVVNGKEMKDYFARPVLQMILRQPFQVAGVEDQFDVMATVKGGGLSGQAGAVKHGVSKALQLYDPSLRGALKAAGFLTRDSRVVERKKYGKAKARKSFQFSKRMPTIQQLIRKPRQPKIKRSKSMHLQECPQKRGVCTRVYTTTPKKPNSAMRKVAKVRLTNGFEVISYIPGESHNLQEHSVVLIRGGRVKDLPGVRYHILRGVLDTQGVKDRKQRRSKYGAKRPKMAREARRAKKKVSKNIAAGVAHVNSTFNNTKILISDVQGNAIAWSSAGTMGFKGSRKSTPYAAQMAAEDVGKKAQEHGVKTLEVEVQGPGSGRESALRALAAVGFNITSIRDVTPMAHNGCRPPKRRRVNMIQRRGLLIILSSPSGAGKSTLAKRLMTWDPDIRFSISATTRAPRAGEKNGIDYHFSTDAEFKAQVASGEMLEHAHVFGNFYGSPKGPVETAISAGRDVLFDIDWQGAQQIRNSALGRHTLSIFLLPPSIAELHRRLVERAQDASDVIERRMQKSWDEISHWDGYDFVLVNDDLDATEDQLKTILKATRLRRSQQPGLTNHVRTLQQQFEDLPMAQSFLGQKRLRKYYGKIREVLEMPNLIEVQKSSYDLFLRSGDQLEPLDGEGIKGVFQSVFPIKDFNETAVLEFVKYELEKPKYDVEECQQRDMTYSAPLKVTLRLIVFDIDEDTGAKSVKDIKEQDVFMGDMPLMTPNGTFVVNGTERVIVSQMHRSPGVFFDHDKGKTHSSGKLLFTCRIIPYRGSWLDFEFDAKDLVFARIDRRRKLPVTTLLYSLGMDQEGIMDAYYDTVDYKYKKNKGWVTKFFPERVRGTRPTFDLVDAKTGEVIAEASKKVTPRAVKKLIDDGKVTELLVPFDHIVGKYVAKDIINEENGAIYVEAGDELTWEVDKDGDVIGGTVKELMDAGITDIPVLDIDNINVGPYMRNTMANDKNMGRDTALMDIYRVMRPGEPPTVDAASTLFDSLFFDSERYDLSAVGRVKMNMRLALDAEDTERTLRKEDIVSCIKALVDLRDGRGDIDDIDHLGNRRVRSVGELMENQYRVGLLRMERAIKERMSSVEIDTVMPQDLINAKPAAAAVREFFGSSQLSQFMDQTNPLSEVTHKRRLSALGPGGLTRERAGFEVRDVHPTHYGRMCPIETPEGPNIGLINSLATFARVNKYGFIETPYRKVENGQVTDEVHYMSATEEMRHTVAQANANLDESGKFVNDLVSTRQSGEYTLAPNENVDLIDVSPKQLVSVAASLIPFLENDDANRALMGSNMQRQAVPLLQAEAPLVGTGIEEVVARDSGAAIMAKRAGIIDQVDATRIVVRATADLELGDAGVDIYRMRKFQRSNQNTCINQRPLVKVGDTVQKGEVIADGPSTDMGELALGKNVVVAFMPWNGYNYEDSILISERIVRDDVFTSVHIEEFEVAARDTKLGPEEITRDIPNVGEEALRNLDEAGIVYIGAEVGPADILVGKITPKGESPMTPEEKLLRAIFGEKASDVRDTSLRLPPGDYGTVVEVRVFNRHGVEKDERALQIEREEVERLARDRDDELAILDRNIYARLKGMILGKTAVKGPKGVKPNSEITEDLLETLSRGQWWQLALGDEKDASQVEALNEQYEVQKRALDARFEDKVEKVRRGDDLPPGVMKMVKVFVAVKRKLQPGDKMAGRHGNKGVISKVVPMEDMPFLADGTPVDFVLNPLGVPSRMNVGQILETHMGWAARGLGIHIDEALGEYRRSGDLTPVRDAMKIAYGDNVYDEGIKDMEEDSLVEAAGNVTRGVPIATPVFDGAKEADVNDALVRAGFSESGQSVLFDGRTGEQFARPVTVGIKYLLKLHHLVDDKIHARSTGPYSLVTQQPLGGKAQFGGQRFGEMEVWALEAYGAAYTLQEMLTVKSDDVAGRTKVYESIVKGEDNFEAGVPESFNVLVKEVRGLGLNMELLDAEDEEMPLYEHVFISRQDLSNTQAEGLVEHFGTVLADNGGKLVDSEYWGIKTMAYKINKNRKGHYAYLRTDAPAPAVQEMERLMRLHEDVMRILTIKVDEHAEGPSIQMQKRDDR-DRGDRGDTADIDVDLLDHNQAQVELARLADLLSKANRDYHTADAPDISDADYDALKRRNIQIETRFPDLKRNDSPSEKVGAPIADGFSKVIHEIRMLSLGNAFNDKDVTDFDISIRKYLGLTDAPALTYTAEPKIDGLSLSLRYENGHLVQAATRGDGEVGENVTENARTISDIPQMLSDAPDILEVRGEVYMSHEDFSALNNRQTSAGLKTFANPRNAAAGSLRQLDASVTQNRPLRFFAYAWGALSDPLAETQMGAIERLAQLGFSVNPLTVLCVGPTEMIRHYVQIEEQRATLGYDIDGVVYKLDDLALQRRLGFRSTTPRWAIAHKFPAELAWTRLENIDIQVGRTGALSPVARLTPVTVGGVVVSNATLHNEDYIAGQDSGGQPIRGGRDIRVGDWVQIYRAGDVIPKIKDVDLTKRPDDTHPFDFPTQCPECGSDAVRETGDAVRRCTGGLICPAQAVEKLKHFVSRAAFDIEGMGAKQVEQFHNDGWVREPSDIFTLRDRYGSGLQQLQNREGWGEKSAQNLFQAIDEKRKISLGRVIFALGIRHVGDSSASLLANNYGTWQAFETAMTSATIGKGDEWDTLIAIDGVGSVMAASVVTAFHQEAERASIDRLIAHLDIQPAIKRVNTDSPVAGKTIVFTGALEKMTRAEAKARAEAFGAKVSGSVSAKTDLVIAGPGAGSKGKKAVELGIETIDEDGWLALIDA-MIHKNWAELIKPTQLDVKPGNDPARQATVVAEPLERGFGLTMGNALRRVLMSSLQGAAITSVQIDNVLHEFSSVAGVREDVTDIILNLKGVSIRMEVEGPKRLSISAKGPGVVKASDIVETAGIDILNRDHVICHLDDGADLYMELTVNTGKGYVAADKNRPEDAPIGLMPIDAIYSPVKKVSYDVQPTREGQVLDYDKLTLKLETDGSITPDDAVAYAARILQDQLSIFVNFDEPESATAMQEDDGLEFNPLLLKKVDELELSVRSANCLKNDNIVYIGDLIQKTEAEMLRTPNFGRKSLNEIKEVLSGMGLHLGMDVEDWPPDNIEDLAKKFEDSFMARDQNR--GRRD-REEAPEFADRLVAINRVSKTVKGGKRFGFAALVVVGDQKGRVGFGKGKAKEVPEAIRKATEQAKRQMIRVPLREGRTLHHDMEGRHGAGKVVMRAAPQGTGIIAGGPMRAVFEMLGIQDVVAKSIGSQNPYNMIRATIDGLQKEGSPRMVAQRRGKKVADILKK-DDAP-SKAPAEEAAMNDPLGDMLTRIRNAQMRGKSTVITPASKLRAWVLDVLADEGYIRGYENTTGKDGHPAIEISLKYYDGTPVIRELKRVSKPGRRVYMGVKDIPQVRQGLGVSIVSTPRGVMSDASARTNNVGGEVLCMVFMHDIRAIRENPVAFDAAMTRRGLEPQSSSILALDEARRIKIQAAEEAKAAQNAASKDVGAAKARGDNAEFERLRTLVAEKKDTVARLNDEAKAEDARLSDLLMGLPNVIYDDVPDGADESDNVEINRHGDPRVLDFPAKEHYELPGVIPGMDFETAAKLSGSRFVVLSGAVARIHRALAQFMLDTHVDENGLTETWTPILVREDMMLGTGQLPKFGEDSYQTTNGWWLVPTAEVTLTNIVNGLTVDEDYLPRRYVAHTQCFRSEAGSAGRDTAGMLRQHQFEKVEMVSIVHPDQSENEQKRMTRCAEGILEKLGLAYRTVNLCTGDIGFGAKRTYDIEVWLPGQGQYREISSVSTCGDFQARRMNARYKPSDGGKPQFVHTLNGSGLAVGRALIAVLENGQQTDGSVVLPDVLHSYLSGKLVLTTDGQLSMAITAALVKELRDSTGAGMMDAKKALTESNGDMEAAVDWLRTKGLAKAAKKSGRTAAEGLVAVKVDGGKGVAVEVNSETDFVAKNADFQGMVSDIADAAMTVSDIDALKAAEIGGKTVEATITDKIATIGENMSVRRMSTLEGGSVVSYVHNPAVAGMGKIGVLVAMNGDNEAFGRQVAMHIAAINPASLSAEDLDPALVDREKQVLTEQARESGKPEQVIEKMIEGRMKKFLSEVTLLGQDFVVNPDLTVAAAAKEAGVELTGYVRLEVGEGIEKVEEDFAAEVAKVGKAMNQELTNNPFNPLTPPKVFDEIKVSLASPERILSWSYGEIKKPETINYRTFKPERDGLFCARIFGPIKDYECLCGKYKRMKYRGVVCEKCGVEVTLQKVRRERMGHIELAAPVAHIWFLKSLPSRIGLMLDMTLRDLERILYFENYVVIEPGLTDLTYGQLMTEEEFMDAQDSFGMDAFTANIGAEAIREMLQAIDLEAEAEQLRADLKEATGELKPKKIIKRLKIVENFIESGNRPEWMILTVVPVIPPELRPLVPLDGGRFATSDLNDLYRRVINRNNRLKRLIELRAPDIIVRNEKRMLQESVDALFDNGRRGRVITGANKRPLKSLSDMLKGKQGRFRQNLLGKRVDFSGRSVIVTGPELKLHQCGLPKKMALELFKPFIYSRLEAKGLSSTVKQAKKLVEKERPEVWDILDEVIREHPVMLNRAPTLHRLGIQAFEPVLIEGKAIQLHPLVCSAFNADFDGDQMAVHVPLSLEAQLEARVLMMSTNNVLSPANGAPIIVPSQDMILGLYYVSLERKGMKGEGMIFGSVDEVQHALDAGEVHLHSKITARIKQIDDEGNEIYKRYETTPGRVRLGALLPLNAKAPFDLMNRLLKKGEVQQIIDTVYRYCGQKESVIFCDQIMTMGFREAFKAGISFGKDDMVIPDNKWDIVDGVREQVKEFEQQYMDGLITQGEKYNKVIDAWSKCSDQVADSMMSDISAMRVED-GVEQEPNSVFMMAHSKARGSPAQMKQLGGMRGLMAKPSGEIIETPIISNFKEGLTVLEYFNSTHGARKGLADTALKTANSGYLTRRLVDVAQDCIVREHDCGTENAITAEAAVNDGEIVSSLSERILGRVAADDVFKPNTDEVIVAKNELIDERKADAVEAAGIATMRMRSPLTCESEEGVCAMCYGRDLARGTLVNQGEAVGIIAAQSIGEPGTQLTMRTFHIGGIAQGGQQSFLEASQAGKIEFRNANILENASGDQIVMGRNMQIAIIDDNGEERASHKLGYGSKLLTKDGAKVNRGDKLFEWDPYTLPIIAEKAGMVRYVDLVSGIAVRDETDDATGMTQKIVTDWRAAPKGSDLKPEIIIVGSDGEPVRNEAGNPVTYPMSVDAILSVEDGQDIKAGDVVARIPREGAKTKDITGGLPRVAELFEARRPKDHAIIAEIDGYVRFGRDYKNKRRISIEAAEEGAEPVEYMVPKGKHIPVQEGDFVQKGDYIMDGNPAPHDILSIMGVEALADYMIDEVQDVYRLQGVKINDKHIEVIVRQMLQKWEILDSGETTLLKGEHVDKQEFDAANEKAIASGRRPASGEPILLGITKASLQTRSFISAASFQETTRVLTEASVQGKRDKLVGLKENVIVGRLIPAGTGGATQQMHRVAQERDNVVLEARRDEAEAAAALAAPTEAMSDV------DGSMITPESRGMSRRHAAEKREVLPDAKFGDTVLTKFMNNLMIDGKKSVAERIVYNALDRVEDKIKRAPVEVFHEALDNIKPTVEVRSRRVGGATYQVPVEVRPERREALAIRWLINASRSRNEHTMEERLAGELMDAVQSRGSAVKKREDTHKMADANKAFSHYRW--------------MSQPAPETRPRDKLEGGISFKLKTDFEPAGDQPTAIAELSGGLKNGDRDQVLLGATGTGKTFTMAKIIEETQRPAIILAPNKTLAAQLYGEFKGFFPENAVEYFVSYYDYYQPEAYVARSDTYIEKESQINEQIDRMRHSATRALLERDDVIIVASVSCIYGIGSVETYSAMTQDLIAGNQYDQRKVMADLVAQQYRRNDQAFQRGSFRVRGDSLEIWPAHLDDRAWRLSFFGEELEAITEFDPLTGQKTDTFEKTRIYANSHYVTPRPTMQQAIIGIKKELRMRLDQLVADGKLLEAQRLEQRTNFDLEMLEATGVCNGIENYSRYLTGRAPGEPPPTLFEFIPDHAIVFADESHVSVPQIGGMYKGDYRRKFTLAEHGFRLPSCMDNRPLKFEEWDAMRPQSVFVSATPAAWELEQAGGVFTEQVIRPTGLLDPEVEIRPVEMQVDDLLDEVRKVAADGMRTLVTTLTKRMAEDLTEYMHEQGIKVRYMHSDIDTIERIEILRDLRLGAFDVLIGINLLREGLDIPECGLVAILDADKEGFLRSETSLVQTIGRAARNVDGRVIMYADRMTGSMERALRETNRRREKQIAYNIEHNITPATVKKNVEDILAGLYKGDVDMNRVTAQIEKPMHGANLEAHLDGLRQQMRKAAENLEFEEAARLRDEVKRLETVDLVVSDDPLARQSAVDQAVGDAQKAAGRSTAGKPGQRGGVKRRKR-MAAKPFFRRRKVCPFSGENAPKIDYKDTRTLQRYISERGKIVPSRITAVSAKKQRELARAIKRARFLALLPYAVKMPKRILQGVVTSNQNEQTVTVSVERRFTHPVLKKTIRKSKKYRAHDEKNAFNVGDAVRIQECPPKSKTKRWEVIAE--MANSPQAKKRARQNETRFQINKARRSRIRTFLRKVEEAIETGDKDAASTALRAAQPELMRGVTKGIYHKNTVARKMSRLSARVKAIAMSRSVWKGPFVDAHVLKKAEKSRESGRNEVIKIWSRRSTILPQFVGLTFGVYNGRKHIPVNVSEDMIGQKFGEYAPTRTYYGHAADKKAKRKMARIAGVNIPTQKRVPIALTYITGIGPASAKSICEAVKIDPTRRVNELSDAEVLAVREHIDANHTVEGDLRREVQMNIKRLMDLGCYRGLRHRRNLPVRGQRTHTNARTRKGPAKAIAGKKKMAMKIRLARGGSKKRPFYRIVAADSRMPRDGRYVEKLGTYNPLLPKDSEDRVKMDLDRIKHWMGEGAKPTDRVARMLEAAGVMDKKERNNPNKAEPGKKAKERAEEKAAKAAAPAEEAPEDATEEAASEMSITVEEKARVMKEYATKDGDTGSPEVQVAILSSRIATLTEHFKTHKKDNHSRRGLLKLVAQRRKLLDYVKGKDEARYQDLIKRLGLRRMPTLNDIRSTFLTYFEKNGHAKVPSSPLVPRNDPTLMFANSGMVQFKNLFTGVETRDYTRATSAQKCVRAGGKHNDLDNVGYTARHHTFFEMLGNFSFGDYFKSDAIPFAWNLITKELDIPADKLVVTVYHDDDEAAEIWKKVAGFSDDRIIRIATDDNFWMMGPTGPCGPSSEIFFDHGDHIWGGPPGSPNEDGDRFVEIWNLVFMQYEQFEDGSRAPLPNKSIDTGMGLERVAALLQGTNDNYATDLIRSLIEASANATSTDPDGPGKTHHRVIADHLRSTSFLLADGVMPSNDGRGYVLRRIMRRAMRHAHLLGAKDPLMHQLVPALVSQMGAAYPELTRAQALITETLKLEETRFKQTLERGLRLLDDELTGLPEGAELPGDAAFKLYDTYGFPLDLTQDALREKGRSVDTDGFDAAMAAQKELARAGWSGSGETADAAIWFDIADQHGPTDFLGYDTETAEGQILAMVKDGKSINSIGHGDSCWVVLNQTPFYAESGGQVADHGFIRNEDMIAECSDVQKSEGVFAHYIKVLSGDFNVNDTVGLDVDHARRTSIRANHSATHLLHEALRKALGDHVVQRGSLNASDRLRFDFSHGKALSHDELKQVETEVNDFIRQNSPVETRIMTPDDARDIGAQALFGEKYGDEVRVVSMGRAA-TGKGTDGQTYSIELCGGTHVRQTGDIGVFVTLGDSASSSGVRRIEALTGAEAFQYLSLQDQRLGNVASQLKAQPGEVGDRVKALMDERKALQNEVAQLRRELAMSGGGQAAAPEIKTINGIDFVAQVLNGVTGKDLPALVDDHKVRIGSGAVLLIADADGKAAVAAGVTSDLTDRLSAVDLVKVAVIELGGKGGGGRPDMAQGGGRDAANADAAIKAAEQVLENMGFKMGIVGLPNVGKSTLFNALTRTAAAQAANFPFCTIEPNVGEVAVPDARLDKLAAIAASKEIIPTRMTFVDIAGLVKGASKGEGLGNQFLANIREVDAIAHVLRCFEDGDVTHVEGRVDPVSDAETIEMELMLADMESIEKRMQNVVRKIRGGDKDAVQQERLLKMALQALENGQPARVVDVDEEDAKAWKLLQLLTTKPVLYVCNVGEAEAADGNAHSAKVAEMAAAQGNSHVIISAQIEEEISQLDPDEAEMFLTEMGLTEAGLDRLIRAGYELLHLETYFTVGPKEARAWTIKQGTLAPQAAGVIHGDFEKGFIRAETIAYDDFVALNGEQGAKEAGKMRAEGKGYQVKDGDVLHFLFNTMGWKTLDDMDLAGKRILTRVDINVPMENGRVTDTTRIDRIVPTVKDILAAGGIPILLAHFGRPKGQVVPDMSLRQLIPTLETALEQSVKFADGNYG--ATAQALEPGDILLIENVRFAPGEEKNDPDFATRLASLGDIYCNDAFSAAHRAHASTEGIAHILPSCAGRLMQAELSALEAALGKPERPVVAVVGGAKVSTKLDLLGNLVGKVDHLIIGGGMANTFLAAQGIDVGKSLAEHDMTDTAREILDKAKTAGCQIVLPADVVVAREFKAGAASEVVAA---NACPADAMILDAGPDAVETISKVLETAKTLIWNGPLGAFEIEPFDTATNAAAQLAAQQTKAGKLISVAGGGDTVAALNKAGAADNFTYISTAGGAFLEWMEGKTLPGVAALERMAV-DFTMRQLLEAGVHFGHQTQRWNPRMQEFIYGSRNGIHIMDLTQTVPMLDAALHVIQETVAKNGRILFVGTKRQAQRPIAEAAEKCAQYYMNHRWLGGTLTNWKTVSQSINRLREIDEKMETGAEGLTKKERLGMEREQAKLQASLGGIREMGGVPDLLFVVDVNKEDLAIAEAKKLGIPVVAIVDTNCSPDGVDYIIPGNDDAARAISLYCDLAARAALEGMSTQLGAAGIDLGEMVDAPVEEAVAEDA-AAEEAPAAEAPA---ESMTKRTSAKYKIDRRMGENIWGRPKSPVNRREYGPGQHGQRRKGKLSDFGLQLRAKQKLKGYYGDLTEKQFRRIFREAERVKGDTGENLIGLLERRLDALVYRAKFVPTIFSARQFVNHGHVKVNGQKVNIPSYRVKEGDLIEVRDRSKQLAVVIEAAQLPERDVPDYLEVDHSKMTATFVRTPGLGDVPYPVMMEPNLVIEYYAQNMGHKVNPIGMRLQVNRTWDSRWYADTKDYGDLLLEDIKLREFIKEECKQAGISRVIIERPHKKCRVTIHTARPGVIIGKKGADIETLRKKLADMTDSELHLNVVEVRKPELDAQLVSESIAQQLERRVSFRRAMKRAVQNAMRMGALGIRVNVAGRLGGAEIARTEWYREGRVPLHTLRADIDYAASEAMTAYGIIGIKVWIFKGEIMEHDPQARDRRQQELQDGPAPRGAGGRRMARKRKGRDISGWLVIDKPAGITSTSVVNKVKWAMDAKKAGHAGTLDPEATGVLAVALGEATKTVPYITDALKAYRFTVRLGQATNTDDAEGEVIAESPNRPTDDQIKDALGQFVGDIEQVPPKFSAVKIDGQRAYKLARDGQDVDIAARPLWVEELVMTDRIDEDHVILEMICGKGGYVRSIARDLGVALGCHGHVLKLRRLWSGPFNAADGISLDLLDQHAKTTELDQYLRPLEDGLTDLPELRCPADAAAKLRNGNPGMVFAADVEYGDEAWVSHDGHAVAVGVYKAGQLHPSRVFNN--THIIGLTGSIGMGKSTTAAMFEKSGIPVWDADSTVHQLYAKGGAAVGPIGIEFPDAVKDGGINRSTLKSIIQSDPTVLKRIEQIVHPLVAQERQKFIQETQSDIIVLDIPLLFETGADQWVDTVVCVSVSADIQRNRVLERPGMTASQLDMILSKQMPNEEKCKQADFVVETETLNAAQIQVDAIIQKIKGQIANAM-LVEILVEDDRWQNVRLAALAETAASATLSRLGIEPGMFEVSLMGCDDARISELNAEFRAKQKPTNVLSWPAQALSTGRPGETPVSPEIGPMGPTELGDIALAYETCRQEADTADKSLDDHVTHLIVHAMLHLLGYDHINDQDAALMEELEVDILATLGLPDPYMQSNAMARFIFITGGVVSSLGKGLASAALGALLQARGFTVRLRKLDPYLNVDPGTMSPFEHGEVFVTDDGAETDLDLGHYERFTGVAARKTDSVSSGRIYSNVLEKERRGDYLGKTIQVIPHVTNEIKDFIKIGEDEVDFMLCEIGGTVGDIEGLPFFEAIRQFSQDKPRGQCIFMHLTLLPYIRASGELKTKPTQHSVKELRSIGIAPDVLVCRSEGPIPVKEREKLALFCNVRPDSVIAAQDLKSIYEAPLAYHREGLDQAVLDAFGISPAPKPDLTRWEDVADRIYNAEGEVKIAIVGKYTQLEDAYKSIAEALTHGGMANRVKVKIEWVDAELFDREDAAPYLEGYHAILVPGGFGERGTEGKIKAAEFARTRKIPYLGICLGMQMAVIEAARNLAGMENAGSEEFDHEAGKKRFTPVVYHLKEWVQGNHKVERKATDDKGGTMRLGAYDATLTKGSKVAQVYDSTAIEERHRHRYEVDIEYRDDLEKQGLIFSGMSPDGRLPEIVEVKDHPWFIGVQFHPELKSKPFDPHPLFADFIRAAVENSRLVMTKMTTILGIESSCDDTAAAVVR-----MTDILSSVVESQTELHALYGGVVPEIAARAHAEKLDHCIEHALDQASVGLRDLDAIAVTAGPGLIGGVLSGVMCAKGLSAATNLPIIGVNHLAGHALTPRLTDQLAYPYLMLLVSGGHCQFLIVRGALDFTRLGGTIDDAPGEAFDKTARLLGLPQPGGPSVERTAVDGDPSRFSFPRPLLDRDDCDMSFSGLKTALLRARDAAVADKGGLTTDDRANLCAGFQAAVTDVLIEKTRRAIRLYLAECPAIPAIAVAGGVAANQTIRVGLQNLADQMQTSFLAPPLALCTDNAAMIAYAGIELFRAGQTDDLTLSVRPRWPLDHKSAPMLGSGKKGVKA------------------------------------------------------------------------------------------------------------------------------------

>'Su-guttiforKCTC-32187'

MIAKELHDKTPDQLRDELVNLKKEAFNLRFQQATGQLENASRLRTVKRDVARVKTVLNMKAAAAATDAMATADLLNMD-KKTADKQKALDSALAQIERQFGKGSIMKLGAEGAVQDITASSTGSLGLDIALGIGGLPMGRIIEIYGPESSGKTTLTLHCVAEQQKAGGVCAFVDAEHALDPQYAKKLGVDIDELLISQPDTGEQALEIVDTLVRSGAVNMVIVDSVAALTPKSELEGEMGDSSVGVQARLMSKAMRKLTSSISKSNCMVIFINQIRMKIGVMFGSPETTTGGNALKFYSSVRLDIRRIGSIKDRDEVVGNQTKVKVVKNKVAPPFKQVEFEIMYGEGISKMGELLDMGVQAGIVDKSGSWYSYGDERIGQGRINAKTYLRENPAMANDIEDKIRASHGLDFNGSDSDEPNILDDMTRYTPSEIESRWQAVWESSEIFKAVRSADKPKYYVLEMFPYPSGRIHMGHVRNYTLGDVIARYKLAKGFNVLHPMGWDSFGLAAENAAMQKGIHPGTWTYQNIEDMKSQMKPLGFSLDWSREIATCHPDYYQHQQAMFIDMIEAGLIYRKNAVVNWDPVDMTVLANEQVEQGRGWRSGALVERRELTQWFFKISDYSDELLGALDTLENWPAKVRLMQENWIGKSRGLQFAFSTINAPDGHDRIEVYTTRPDTLLGASFVGISPDHPIAKLLERDNKDVADFVAECRKGGTTEEAIETGEKLGMDTGIRVRHPFDTSKELPVYIANFILMEYGTGAIFGCPAHDPRDFEFATKYDLPIISTYLPSEDAAEELSEAYVPPKTEKVFYNRGFAGEQWQTGLEAIDAAIAFCESQGVGQGVTKFRLRDWGLSRQRYWGCPIPVVHCDDCGVVPEKKENLPVKLPEDVTFDIPGNPLDRHTEWRTTPCPACGKSAQRETDTMDTFVDSSWYYARFTAPHADTPTVMEDAEYWMNVDQYIGGVEHAILHLLYARFFARAMQITGHLPKTAIEPFDALFTQGMVTHAIYTSVGADDRPVFHYPEEVKGDKAFQGGAEVKIIPSAKMSKSKNNVVDPLSIISSFGADTARWFVLSDSPPERDVEWTASGAEAAYKHLGRVWNISARIAAMQDGADGENDQDLLKQMHKATHDVTMAVESFGFNAAIAKLYGFTATLQKSNASKAAQREAVLTLAQLMSPMTPHLAEDIWATQGGEGLIATAPWPVADEAMLKDDTVTLPIQVNGKRRGEIDVPADMPKEEVEKLALAHPAVIRILEGGTPKKVIVVPGRIVNVVVMAITSANQLELLQTAEAVAREKMIDPVLVIEAMEESLARAAKSRYGAEMDIRVSIDRKTGRAKFTRVRTVVADAELENYQAEFTVEQAKQYMANPEIGQEFIEEVPPVEMGRIAAQSAKQVILQKVREAERDRQYEEFKDRAGTIINGLVKREEYGNVIVDVGAGEAILRRNEKIGRESYRPNDRIRVYIKEVRREQRGPQIFLSRTAPEFMAELFKMEVPEIYDGIIEIKAVARDPGSRAKIAVISYDNSIDPVGACVGMRGSRVQAVVNELQGEKIDIIPWNEDMPTFLVNALQPAEVSKVVLDEEAGKIEVVVPEEQLSLAIGRRGQNVRLASQLTGLDIDIMTEAQESERRQAEFELRTKLFMDNLDLDEFFAQLLVSEGFTSLEEVAYVELDELLVIDGVDEDTAAELQARAQDVLEAQNKAALDAARALGAEDSLINFEGLTPQMIEALAKDDVKTLEDFATCADWELAGGWTTVNGERVKDDGTLEPFEMTLEEAQAMIMTARVMLGWVDPADLETD-ADEG-DA----DEEAEAMAHKKAGGSSRNGRDSAGRRLGVKKYGGELVIPGNIVVRQRGTKFWPGENVGMGKDHTLFATVEGNVQFHKGLKNRTFISIVPVAEAAEMAKRWYSVSVLSNFEKKIAEQIRTTVAEQQLEDQIDEVLVPTEEVIEVRRGKKVTTERRFMPGYVLVHMEMSDRGYHLINSINRVTGFLGPQGRPMPMRDAEVTAILGRVQEGEETPRTLIHFEIGERVKVGDGPFEDFDGMVEEVDEENQKIKVMVSIFGRETPVELDFTQVNKQIMSFTLAIVGRPNVGKSTLFNRLVGRRLALVDDQPGVTRDLREGAAKLADLRFTVIDTAGLEEVTDDSLQGRMRRLTERAVDMADVCLFMVDARVGITPSDLVFAEILRKRSAHVVLVANKAEGNAAEAGVLEAYSLGLGEPIRLSAEHGEGLTDLYTVLMPLADGFAESRPDETPEIDVDLSEEDDDMDVVPVPTRAKPLQVAVVGRPNAGKSTLINQIVKEDRLLTGPEAGITRDAISLMTEWAGAVPMRIFDTAGMRKKAKIQEKLEKLSVSDGLRAVKFAEVVVVLLDAEIPFEQQDLRIADLAEREGRAVVIAVNKWDVEEDKQGKLKELKESFERLLPQLRGAPLITVSAKTGRGLDRLQQAIMRAYDMWNRRVTTAQLNRWLSGMLEAHPPPAPQGKRIKMKYMTQAKTRPPGFVVMCSHPDKVPESYNRYLVNGLRVDFDMPGTPIRLWMRGQSEANPYKGRKKAPPSKLRKHIGGKPNDMRIVFMGTPAFSVEVLDALVDAGHEIAAVYSQPPRPAGRGKKERPSPVDARAKELKLEVRTPVSLKSTEALAEFEALGAEVAVVVAYGLILPQGVLDAPVHGCLNIHASLLPRWRGAAPIHRAIMAGDPQTGVCIMQMEAGLDTGPVLLRGSLDIGPEETTAQLHDRLAGMGAKLIVEALAQLAELVPQVQPEEGVIYAAKIDKAEARIDWSRPAVEVDRMIRGMSPFPGAWFEVNGTRVKVLGSRLVEGEGAAGLVLDTSLHVACGDGAVALTVLQKAGKGAQDVNVFQRGMQIAVGTDLSEGMTDTAAYRVLARKYRPETFADLVGQEAMVRTLKNAFQADRIAQAFIMTGIRGTGKTTTARIIAKGMNCIGTDGTGKPTTEPCGACEHCTAIMEGRHVDVMEMDAASNTGVANIREIIDSVHYRAASARYKVYIIDEVHMLSTGAFNALLKTLEEPPEHVKFIFATTEIRKVPVTVLSRCQRFDLRRIEPEVMITLLQKIAGAEGAEIADDALALITRAAEGSARDATSLLDQAISHGAGETTALQVRAMLGLADRARVLDLLEMILRGDAAGALTEIGAQYAEGADPMAVLRDLAEITHWISVVKITPDAAEDPTISPEERDRGRQMADALPIRVLTRLWQMLLKALEEVASAPNAMMAAEMAIIRLTHVADLPSPEELVRSLQN-NPAP--PTAAPQGG------GTQAISHSQTRMVGQPNIAGQNTALARAPDGALAHYPTFEHVLELIRHNRDVKLLVEVETSLQLAAYRPGRIEFVPTDNAPGDLAQRLGNKLQLWTGSRWAVTLVNSGGAPTIASLRDAKDNALRADAAAHPMMLAVLAQFPQAKITAIRTPQDIAAAAVTEALPEVEDEWDPFEDSMPKMKTKSSAKKRFKISATGKVIGSQAGKQHGMIKRSNKFLRNARGTTALCPADAKIIKGFMPYDRMNLFADIRSLVLTALDAMVAAGDLPRGLITDNVTAEPPRDASHGDMATNAAMVLAKPAGMKPRDIAEKLAVHLADDPRITSAEVAGPGFLNLRLAPAVWQGVAASVLASGTDYGRGTLGAGKSVNVEYVSANPTGPLHVGHTRGAVFGDALASLLAFAGWDVTREYVINDGGNQIDALARSVFLRYQEAHGQKVAFPDGTYPGDYLVPVGQKLKDEVGDKYLDEAEDVWLIPIRNFATDQMMELIREDLALLGVKMDRFFSEKSLYNTGKIEACLKKLDDMGLIYRGTLEPPKGKLPDDYEAREQTLFKSTEFGDDQDRPIQKNDGTWTYFAPDIAYHNDKVERGYDQLINVFGADHGGYVKRMKAAVYALSGGSVPLDIKLTQLVKLFKNGEEFKMSKRAGNFVLLSDLIKEVGKDVTRFVMLTRKNDAPLDFDFNKVKEQSRENPVFYVQYAHARVASVLRKAAEAGIDVSDAALKTADLGKLDHDAELALLRKVAEWPRLVETAARSNEPHRIAFYLYELAGDLHGFWALGNSETGLRFIQEDDRATSQAKIALARAVAIVIASGLGILGVTPSEEMRMSAIDTLPTLRQVIDTHGLQARKSLGQNFLLDLNLTAKIARQAGDLTCCDVLEIGPGPGGLTRGLLSEGARRVLAIEKDSRCIPALDEIAAAYEGRLQIIEGDALEVNPLAYLTPPIRVAANLPYNIGTELLVRWLTPPEWPPFWESLTLMFQREVAERIVAKPGSKAYGRLALLAQWRADAKIVLHLPPEAFTPPPKVSSAVVHLTALAQPRFPADAAILSRTVAAAFNQRRKMLRSALKGTAPDIEDRLIAAGLKPTDRAEQVPLEGFCALARELAKPMNLIAQIEAEHIAELAKEIPDFRAGDTIRVGFRVTEGTRTRVQNYEGVCISRKHGKGIAGAFTVRKISFGEGVERVFPLHSTNIDSITVVRRGRVRRAKLYYLRTRRGKSARIIENSHYKAPKG---MANSKRQLFIKRRLRVRNKLRRTNRGRMRLSVHRSNKNISVQLIDDVNGVTVASASSLEKVLGVVGKNNIEAATKVGVAIAERAKAAGVEAAYFDRGGFLFHGKVKALADAAREGGLKIMFAVLKTGGKQYKVQAGDMLRVERIAASAGDTVQFNEVLMLGGDTPTIGAPMIEDAGVQAEVVDQIKGEKVIHFVKRRRKHSSKRTKGHRQKLTLVKITEILASGAGKSGVAAAIGTGSVSAAAVAA----KK---AKAEK---PAATAPKAKA-AKKAAKAD-G-DDLSQISGVGPVIVGKLHAEGITTFAQIAAWTDADVEAIEEKLSFKGRVGREDWIAQAKDLTKGMSRTKGGTVTHARHRKIIKAAKGYYGRRKSTFKVASQAVDKANQYATRDRKVRKRNFRALWIQRINAAVRAHDADLTYSRFINGLNLAGIEVDRKVLADLAVNEPESFAAIVKQAQASLAAMQVKETLNEGLKRGYTITVTAAELDAKVMDKLKEAQPDVEMKGFRKGKVPMPLLKKQFGPKVLGEAMQEAVDGAMNDHFESCGDRPAMQPDVKMTNDDWKEGDDVVVEMTYEKLPNIPDVDLSKIKLEKLVVKADDASIDEALASLAETAQDFKSRKKGSKAKDGDQVVMDFVGKVDGEAFEGGSAEDYPLVLGSNSFIPGFEEQLVGVKAEEKKDVTVNFPDDYQAEHLKGKEAVFACTIKEVKEPVAAEINDDMAKKFGAEDLAALKVQIGERLEAEYSGASRAIMKRGLLDALDGLVDFDLPPSLLDAEAGQIAHQLWHEENPDVQGHDHPEIETTDEHKKLATRRVRLGLLLAELGQKAEVEVTDAEMTQAIMNQARQYPGQERQFFEFVQKNQQMQQQMRAPIFEDKVVDYVFEQASVEDKEVSKDDLQKAVEALVDEMKLNELSDNPGATKPRKRVGRGPGSGTGKMGGRGIKGQKSRSGVAIKGYEGGQMPLYQRLPKRGFTKPNRKSYSAINLGLIQKFVDAGKLDIKSVINEDALVASGVVRRKRDGIRILAKGDVTSKINLDVTGASKTAIEAVEKAGGSLTIKAAAAAEASEMIQMQTNLDVADNSGARRVQCIKVLGGSKRKYASVGDIIVVSVKEAIPRGRVKKGDVRKAVVVRTAKEVRRDDGTAIRFDRNAAVILNNNNEPVGTRIFGPVVRELRAKNFMKIISLAPEVLMRHARGYRRLNRTHEHRKALWANMAGSLIEHEQIKTTLPKAKELRPIIEKMITLAKRGDLHARRQARARLKEDQYVTKLFDILGPRYKDRQGGYVRVLKAGFRYGDMAPMAIIEFVDRDRDAKGAADKARVAAMDASE-MLQPKRTKFRKQFKGSIKGLAKGGSDLNFGTYGLKAIEPERVTARQIEAARRAMTRHMKRQGRVWIRIFPDVPVTSKPVEVRMGKGKGSVDFWACKVKPGRVMFEIDGVGDDVAREALRLAAMKLPIKTRVVVREDWMTILKLHNTKTRKREEFVPIDATNVRMYVCGPTVYDRAHLGNARPVIVFDVLYRLLRHVYGAEHVTYVRNFTDVDDKINARSSETGRPIGEITAETTKWFLDDMAAVGALQPSAMPRATQYIPQMVAMIEGLVAEGYAYLNEGHVMFRVRNYTEYGALSGRSVDDMIAGARVEVAPYKEDPMDFVMWKPSDAQTPGWDSPWGRGRPGWHIECSAMAHDLLGAHFDIHGGGNDLMFPHHENEIAQSKCAG--HDFANVWLHNEMLQVEGKKMSKSLGNFFTVRDLLDQGVPGEVIRFVMLSTHYRKPMDWTEKKAAEASRTLKKWRKVTEGVVASPKIPQVVIDTLGDDLNTAGTLTLMHEFASAGAFAELKASAELLGILTEELGGWTELGGAQLDGWTELLSVARMKAVETKDFSEVDRLKAILTDAGVTVQMGKDGIVLAAGPEVDLAKLEALKMAQVKSSTKSDPNYKVIAENRRARFDYAIEDDVECGIMLEGSEVKSLRMGGSNIAESYAAVEDGELWLVNSYIAPYKQAKTFGHEERRRRKLLVSGKQLANMWNETQRKGMTLVPLVMYFNHRGMAKIKIGIAKGKKLHDKREDAAKRDWSRQKSRLLKDNGMSAKPEHYDIIRKPLITEKATMASEANAVVFEVAIAANKPMIKEAVEALFGVKVKAVNTSITKGKVKRFRGQLGTRRDVKKAYVTLEEGNTIDVSTGLMSKDKNPRRVADNEARAKLRMLKTSPQKLNLVAAMIRGKKVDKALTDLTFSKKRIAIDVKKCLQSAIANGENNHNLDVDELVVAEAYVGKNMTLKRGRPRARGRFGKIMKPFAEITIVVRQVEEQAMVSAVENMAANSSWSAFGKATDLRHRILFTLGLLIVYRLGTFIPVPGIDGGALRDFMEQAGQGIGGMVSMFTGGALGRMGIFALGIMPYISASIIVQLLTSMVPSLEQLKKEGEQGRKKINQYTRWGTVALATVQSYGLAVSLEAGDIAADPGMYFRIACMITLVGGTMFLMWLGEQITARGIGNGISLIIFVGIIAEVPAAIAQFFASGRSGAISPAVIVAVLVMVIATIMFVVFMERALRKIHIQYPRRQVGMKVYDGGSSHLPIKVNPAGVIPAIFASSLLLLPVTISTFSGNSTNPIMSWLLANFGPGQPLYLLFFIAMIVFFAYFYTFNVAFKPDEVADNLKNQNGFVPGIRPGKKTAEYLEYVVNRILILGSAYLAAVCILPEILRGQFAIPFYFGGTSVLIVVSVTMDTIQQVQSHLLAHQYEGLLEKSQLRGKSGKGRKKRSPARKMKFLDLTKVYIRSGAGGGGCVSFRREKYIEYGGPDGGDGGNGGSVWAEAVDGLNTLIDFRYQQHFFAKNGQPGMGKQRTGKDGDDIVLRVPVGTEILDEDQETVIFDMTELGQRVRLAGGGNGGWGNLHFKSATNQAPRRSNPGQDGVERELWLRLKLIADVGLLGLPNAGKSTFLAATSNARPKIADYPFTTLHPNLGVVGVDNAEFVIADIPGLIEGAHEGRGLGHRFLGHVERCSVLMHLVDGTSETIVEDYHTIINELEAYGGELADRPRITVLNKIDALDEEELASACENLKKASGGPVMQMSAVAKTNTVEVLRALRSQIDADRIRQSTSEEEAPWQPMAAKLKKGDTVIVLTGKDKGKEGKIASVDPKSNKAVVEGINVYVRATRQTQESQGGRIPKSMPMDLSNLAIKDANGKPSRVGFKMDGDTKVRFAKTTGDVI--MLRSGVIAKKMGMTRLFMEDGRQIPVTVLQLDNLQVVAQRTLEKDGYLAVQLGAGTAKVKRTSQAMRGHFAAAKVEPKRKVAEFRIDAEAMLPVGEEIIADHYFAGQYVDVAGTSIGKGFQGAMKRHNFGGLRASHGVSVSHRSHGSTGQCQDPGKVFKGKKMAGHMGSARVTTQNLEVVKTDSARGLIMVKGAVPGSKGGWVTVKDAVKKPFPDSAIVPGALASAAREAAKAAEEAAAAAAAEAEAEAKRLAEEAAAAEAEALKAAEADIASDKAEAGDQSETDKKEGDAMALKSYKPTTPGQRGLVLIDRSELWKGRPVKALTEGLHKHGGRNNTGRITMRRKGGGAKRLYRIVDFKRNKLDVTATIMRIEYDPNRTAFIALVKYDDGEQAYILAPQRLAIGDQVVSSKKADIKPGNAMPFSGMPIGTIIHNIEMKPGKGGQIARAAGTYAQFVGRDGGYAQIRLSSGELRLVRQECMATVGAVSNPDNSNQNYGKAGRMRHKGIRPSVRGVVMNPIDHPHGGGEGRTSGGRHPVTPWGKPTKGAKTRNKKKASSALIIRSRHAKKKGRMLDTAAYTPRLKADFKDRIRAAMKEEFGYTNDMQIPRLDKIVLNIGCGAEAVRDSKKAKSAQEDLTLIAGQKALTTIAKKSIAGFRVREEMPLGAKVTLRGDRMYEFLDRLITVAMPRIRDFRGVNGKSFDGNGNYAMGLKEHLVFPEINFDKIDENWGMDIVIATTAKTDAEAKAMLKLFNMPFNSMKLDVINLDGGNAGSIDLDEALFGLEPRADILHRVVRWQRNNAQAGTHKVKTRREVSYSTKKIYRQKGTGGARHGARSAPIFRGGGIYKGPTPRSHGHELTKKFRKLGLRHALSAKMKAGALVIIDDAMSNGKTAALAKQVKSLGWKRALIIDGATVNENFLQAARNIEGLDILPTMGANVYDILKRDTLVITKAGLEALEARLKMENVVLIVHLILALGLIAVVLLQRSEGGGLGM-GGGGGANSGRPPATPMSKVTWILGAAFVVTSITLTIVTAQKSAGSSVLDRLTATPPALNQG-SVPAPDLNNLLPPADGDNTPLVPTLDMAFFKKLKDRLFNSSSKIDEGLEAIVGDADPSQQAVD-------EAPAQAVDAVPDAKLTERQPTIETDPA----AETAARAETAAREEEQRAP-------------GAPIPQTLPP-------VIPDREQ---APDPAPTRGILGRLLGRDETPEIARRVLDDEMLEQLEELLIAADMGVDTALRVTANMAEGRLGKKLSVHEIKTLMSDEIARIMEPVAKPLPLYKKTPQVVLVVGVNGSGKTTTIGKLASQFRAAGKSVVIAAGDTFRAAAVEQLQVWGERAGVPVLTAPQGSDPASLAYDAMVKAEADGADLLLIDTAGRLQNRGDLMEELAKIVRVIRKKDPDAPHNTLLVLDATTGQNAIGQVKVFQEISDVSGLVMTKLDGTAKGGVLVALADKFGLPIHAIGVGEQIDDLQAFDPQEFADALTGLERMTRDDWGNLKQRLLKTVGQNNFTTWIDPLMLGKTTDGIATLNVPTNFFGNYVSQNFSDLILHEMKAFDADVSRLNFEVANTATKTSTKPVTRQTDAIAPPRSA-SNTSSVYTAPLEKRFSFDTFVVGKPNELAHAAARRVAEGGPVTFNPLFLYGGVGLGKTHLMHAIAQELQARKPELNVLYLSAEQFMYRFVQALRDRKMMDFKEIFRTVDVLMVDDVQFIAGKDSTQEEFFHTFNALVDQNKQIIISADRAPSDIKDLEDRVKSRLQCGLVVDLHPTDYELRLGILQTKVEVHRASYPELAMDDGILEFLAHRISTNVRVLEGALTRLFAFASLVGRKIDMDLTQDCLADVLRASERKITVEEIQRKVSDHYNIRLSDMIGPKRLRNFARPRQVAMYLCKHMTSRSLPEIGRRFGGRDHTTVMHGVRRIEELKVSDGQIAEDLELLRRALEAMLGLGKITKKVFGSPNDRKIKAARPLIEKVNALEPEFKALSDEGIKAKTEELATRALQGESLDDLLPEAFANCREGAFRTLGLRAFDTQLMGAIFLHQGNVAEQKTGEGKTLTATFAAYLNALTHKGVHIVTVNEYLVKRDAEWMGKVFASLGLTTGYIIPNMADDAKRAAYDCDITYATNNELGFDYLRDNMKSELSQIFQKQHSFAIVDEVDSILIDEARTPLIISGPSDDRSDMYLTIDAVIPVLQPDHYVVDEKTRNVTFTDDGTEFLEEQLRVRGLMEEGFTLYDPESTSLVHHVNQGLRAHTLFEKDKDYIVRDGEVVLIDEFTGRMMTGRRLGDGLHQAIEAKEGVDIKPENITLASVTFQNYFRLYDKLAGMTGTALTEADEFMEIYGLGVVEVPTNVAIARIDEDDAVYRTAVEKYAAIIEKTKEAHGRGQPCLVGTTSIEKSEMLSKLLQAEGIPHNVLNARQHEQEAQIVGDAGKLGAVTIATNMAGRGTDIQLGGNVELKVLEALAANPDADPVDTRKRIEAETADEKKKVLESGGLFVLASERHESRRIDNQLRGRSGRQGDPGRSSFFLSLEDDLMRIFGSEKLEKVLTTLGLKEGEAIIHPWVNKSLERAQAKVEGRNFDMRKQLLKFDDVMNEQRKVIFGQRRDIMESQDLHEITEDMRHQMIDDLIDQYMPPKTYADQWDTQGFYAAVIEQLGVDVPVIAWCEEDGVDDEIIRERLIEATDQLMAAKTEKFGPENMRNIEKHFLLQTIDAKWRDHLLTLEHLRSVVGFRGYAQRDPLNEYKNESFQLFEGMLDSLRQDVTQRLSRVEPASEADQQRMIEQMLAQQKAAAEAVDNAADG---AEAAPAAVADGFDETDPTTWGNPGRNDTCPCGSGEKFKHCHGRLTMKLSAELKAFQGRLGYSFQKPHLLNEAVTHASMSTPNRDDNQRLEFLGDRVLGLVMAEALLNLDTRASEGQLAPRFNALVRKEACADVAREIDIGEVLRLGRSEMLSGGRRKQALLGDAMEAVIAAVYIDGGFDAARAMILRLWGTRTTSVKDDARDAKTALQEWAQARGLEPPAYILTKRSGPDHAPIFTIAAKLSTGETASATAGAKRTAEQDAAASLMAQLEQDKMAKLGKRIRAAREAFVGKENLTVEEAVSLIKANSNTKFDETIEIAMNLGIDPRHADQMVRGVVGLPNGTGKDVRVAVFARGPKADEATAAGADIVGAEDLMDIIMGGKIEFDRCIATPDMMPVVGRLGKVLGPRNLMPNPKVGTVTMDVAAAVKAAKGGEVQFKAEKGGVVHAGVGKVSFDEAKLVENIRAFVSAVAKARPAGAKGSYLQQISLSSSMGPGVTVAVEKAIS-MSDDFMLDTDDLERRMNGAIASLRTEFASLRTGRGSASMLDPIQVDAYGQMTPINQVGTVNVPEPRMVTINVWDKALVGKVEKAIRESGLGINPQLNGTIIMLPIPELNEERRTQLSKVAGGYAENARVSIRNIRRDGMDQIKKAKADGLGEDDQKLWEGEMEDLTKKFIKAIDDQLETKQAEIMQVMAKKLVGTMKLQVKAGQANPSPPVGPALGQRGINIMEFCKAFNAKTADLEPGAPCPTVISYYQDKSFTMDIKTPPASYFLKKAAKVKSGAKTPSRENVGSITPKQLREIAEAKMVDLSANDVEAAMKIILGSAKSMGIEVKMFENLSERLSGVFDRLTKQGALSDEDVKTALREVRVALLEADVSLPVARDFVNAVQEKATGQAVTKSITPGQQVVKIVHDALIDVLKGEGEPGALKIDNAPAPILMVGLQGSGKTTTTAKLAKRLKDRDGKRVLMASLDVNRPAAMEQLAILGMQIGVDTLPIVKGETPVQIAKRTKTQASLGGYDVYMLDTAGRLSIDEELMAQVEQVRDIVSPRETLLVVDGLTGQDAVQTAENFNTRIGISGVVLTRMDGDGRGGAALSMRAVTGKPIKFVGLGEKMDALETFEPERVAGRILGMGDIVALVEKAQETIEAEQAERMMKRMAKGMFNMNDLKMQLEQMLKMGGMQGMMGMMPGMGKMAKQISDAGLDDKILKQQIALINSMTKKERANPALLQASRKKRIAKGAGMEVSDVNKLMKMQRQMSDMMKKMGK-GKGGMMKAAMKQMMGKGGMDPAAMAQGMDPKALEAAAKQMGGKLPG----M-GGMGLPSGLSGFGKKK--------------------MSTG--GGDISKLAKEYSDLRPVVEEIAAYRVLLENLEGAREMLSDPEMTSMAREEIYEIESAIPAAEASLQLALLPRDEADARPAMLEIRPGTGGDEAALFAGDLLRMYIRYAEGRGWKVDLIEQQMTELGGIKEVVAHITGENVFARLKYESGVHRVQRVPSTESQGRVHTSAATVAVLPEAEDVDIQIATTDIRIDTYRSSGAGGQHVNTTDSAVRITHIPTGIVVTSSEKSQHRNREIAMQVLKTRLYDAERQRIDTERSDSRASQVGSGDRSERIRTYNFPQGRMTDHRINLTLYKLDAVMQGDLDEIVDALTADAQAKMLAEMGQMDRAQKEQLVDELGQIFESSGVVVVSHYVGLTVAEMQDLRARATAAGGSVRVAKNRLAKIALEGKPCASIANLLTGMTVLTYSEDPVAAAKVAQAFAKDNPKLVILGGAMGENALDVAGVEAVSKLPSREELISTIAGMLGAPASNIAGAIGAPASNIASILSTIEDRAA-MKTFSATPADIDKKWIIIDAEGIVLGRLASIIAMRLRGKHKPSFTPHMDCGDNVIVINAEKIQMTGKKREEMFYWHTGHPGGIKERSKADILEGKHPERIVTLAVKRMLPGNRLSRQIMTNLRVYAGGEHPHEAQAPEVLDVASMNKKNTRSAMQQKDAVLLRAVDTAFQTLLPERIGVAVSGGGDSVALLHLVARWCVQTGHPVVAVTVDHGLRPESRSEADGVARLCRNLDIEHDVLDWDAPEGSGNLPAAARDGRYALMADWAKERGIGGIVLGHTIDDSAENFLIRLGRAAGVDGLAQMESLFTRNGVSWLRPLWQQSRADLRRYLHRQGVAWVEDPSNDDPRYRRTKARQILPDLADLGITADSINHSAHALRQAQKALSHYTRREAETHVCQEGGDLVFAEVITPPIPADIERRMLMGALQWISSAPYPPRKMRSGVLANEMLDQQRKTVFGCLVMRRKGRFRITREYNAVKDLRGPTDAVWDNRWRLVGPH--APDLEVRALGE-SVSILPDWRKTGLPRPTLMSSPAIWRDETLVAAPLAGYNSDWTAQIVAD---FASFLLSHMADLKKLAEDIVGLTLLEAQELKTILKDEYGIEPAAGGAVMMAGPADAGAAAEEKTEFDVVLKNAGASKINVIKEVRGITGLGLKEAKDLVEAGGKIKEGVSKAEAEEIMAKLVAAGAEIELAMSRIGKKPVDLPSGVTASVSGQTIEVKGPKGVRIFKATDDVTMTVEENAVTVTPRGKSKRARQQWGMSRTMVRNLVTGVTDGFKKELEIQGVGYRAAMNGNTLKLNLGLSHDVDYTPPEGVTVTAPKQTEIIVEGIDEQLVGQVAANIRAWRKPEPYKGKGIRYKGEFVFRKEGKKKMHVILLQRVAKLGQMGDVVDVKPGFARNYLLPQQKALTASKANIAAFEGQKAQLEVQNLETKKEAEDMAAKLNGQQFVVIRSASDSGALYGSVTTRDASEAATAEGFSVDRKQVVLLAPIKYLGIHEVQIILHPEVEATIELNVARSPEEAELQAAGKSIQELAAEEEAAAEFEISELFDDLGSAASDDDD-AEA---PRAKASDENEDN-MSDNDGRKTLGL-GGSRPGNVKQSFSHGRTKNVVVETKRKRVVIPKPGGQKPSGPGAGPVGDPSKRPAGITDSEMERRLKAVQAAKSREVEEAAAREADEIARAEDRERRRAEIEAKEAEDREREESLKAKAEEEERVKREAAEAAKAAAAPASEPAQSRAAPNKALPAATPRKAEREREETNKKNRQDDDSRRSGKLTVNQALRGGEGGRQRSMAQMKRKQDRARAKAMGGNVEREKIVRDVQLPPAIVVSELAARMAEKTGAVVKALMNSGLMVTQNETIDADTAELIIEEFGHNVVRVSDADVEDVIKLEIDDAADLQDRPPVITIMGHVDHGKTSLLDAIRKAKVVASEAGGITQHIGAYQVKTERGQVLSFLDTPGHAAFTSMRSRGAQVTDIVVLVVAADDAVMPQTIEAIAHAKAAKVPMIVAINKCDKPSADPDRVRTALLQHEVIVEKMSGDVQDVEVSALTGMGLDQLLEAIALQAEILELKANPARAAVGAVIEAKLDVGRGPVATVLIQNGTLRTGDIFVVGEQYGKVRALIDDQGNRIKEAGPSVPVEVLGLNGTPEAGDVLNVTDTEAQAREIAEYRANAAKDKRAAAGAATTLEQLMANAKADESVSELPIVVKADVQGSAEAIVQAMEKIGNEEVRVRVLHSGVGAITETDVGLAEASGAPIIGFNVRANASARNTANQKGVEIRYYSVIYNLVDDIKAAASGLLSNEIKENFIGYANIKEVFKVTGIGKVAGCLVTEGVARRSAGVRLLRDNVVIHEGNLKTLKRFKDEVSEVQSGQECGMAFENYDDVRAGDVIEIFTREEVTRTLSMANPKKTPRPKAETPRGFRDYFGTEVTQRADMLAKIAGVYHRYGFDALESAGVEKVEALGKFLPDVDRPNEGVFAWQEDADSDKPGDWLALRYDLTAPLARVYAQHRNDLPTPYRRYAMGPVWRNEKPGPGRYRQFYQCDADTVGAASVAADAEICAMLADCLEEVGIERGDYIVRVNNRKVLNGVLEVAGLSGDDKEAERGIVLRAIDKLDRLGVDGVRALLGAGRKDESGDFTDGAGLDDASADVVMGFMQAKRDSGAETCARLRELVGESVIGLEGVEELETIADLLAAGGYAADRIEIDPSVVRGLGYYTGPVYEAELTFEIKDEKGRSRNFGSVAGGGRYDDLVKRFTGQEVPATGVSIGVDRLLAALDAKGRLDTSAQGPVVVTVMDKARMVDYLAMVAELRQAGIRAEVYLGNPKNFGNQLKYADKRSSPVAIIEGDTEKENGMVQIKDLILGAQIAENATLEEWRDRPSQYEVLRSNLVAKVREILELYQMDDLKQKYLTQIANAADEAALEDIRLGAVGKKGEVALKMRELGKMTAEERQAIGPALNALKDEINSALAAKKMALGDAALDERLRSEWLDVTLPSRVRPAGTLHPVSQVTEELTAIFAEMGFSVAEGPRIDTDWYNFDALNIPGHHPARAEMDTFYMARAEGDDRPPHVLRTHTSPVQIRTMEAEGAPLRIICPGGVYRADYDQTHTPMFHQVEGLAIDKDISMANLKWTLEEFFAAFFEIDGIKTRFRASHFPFTEPSAEVDIQCSWVDGQLRIGEGDGWMEVLGSGMVHPKVLAAGGIDPDVYQGFAFGMGIDRIAMLKYGIPDLRAFFDSDLRWLRHYGFASLDQPNLHGGLSRMEMAEEAGLDLVEISPNANPPVCKIMDFGKFKYETQKREAEARKKQKIIEIKEVKFRPNTDTNDYEVKMRNVYKFLENGDKVKITLRFRGREMAHQNLGRELLERVAEDTKDQGKVENFPKMEGRQMVMLIGPLPKMKFTLSWLKEHLDTTASVDEITYALTDLGLEVEGVEDRGAKLRAFTLGFVKSAEKHPDADRLRVCQVETDEGLQQIICGAPNAREGITVVIAKPGVYVPGIDTTIGVGKIRGIESFGMMASERELELSEEHDGIIELPSGNVGDSFTDWLAENDPSKVDPVIEIAITPNRPDALGVRGIARDLAARGLGKLKARDVSMVEGAFACPVSISIDDDTLDQCPVFFGRVIRGVKNGPSPVWLQDKLRAIGLRPISFLVDVTNFYTYDRNRPLHVFDADKIVGGLRVHRARGGETMMGLDDKEYSFSDGMTLISDESGVQSIGGVMGGLATGVTEETVNVFVEAAYFDTVRTAYTGRALKINSDARYRFERGIDPAWTPDGLEHATRMIMEHAGGEASEVVSAGKVPDTSRAYKLNAKRVISLVGMEIPEATQRQTLTALGFRLEGDMAHVPSWRPDVQGEADLVEEVARIASLTKLEGRPLPRLTSGVPRPVMSASQRREVAARRTAAALGYNECVTYSFIDQPSAALFGGGTDATRLENPISNDMSHMRPALLPGLLQAAARNQARGFADMALFEVGPAFSGGEPGEQHMEVSGLLVGRTGAKDVLGAARDVDVFDVKADVEAILAAIGAPAKVQIMRDGDTWWHPGRHGRICLGPKKMLAVFGEVHPKVLAAMDVKGPAMAFTLWPAEVPLPRKTGATRPALKVSDLQAVERDFAFVVDTNVEALALVNAAMGADKTLITDARVFDEFIGGSLGEGKKSLALTIRLQPREQTLKDADIEAVGAAVVGAVTKATGGVLRRMHAYRSQTCADLNDTNVGDSVRLSGWVHRVRDHGGVLFIDLRDHYGITQLICDTDSPVFSDVEKVRAEWCIRIDGEVKARATELVNGKIPTGAIEVFIRDLEVLGRVNDLPLQVFGDQEYPEETRLRYRYLDLRREKMQRNMILRSDVVSSIRQRMWDRSFKEFQTPIITASSPEGARDFLVPSRQHPGKFYALPQAPQQFKQLLMVSGFDKYFQIAPCFRDEDPRADRSPTDFYQLDLEMSFVEQQDVFDTIQPVLTGIFEQFGGGKAVDQIWPQISYKNAALWYGSDKPDLRNPIKMQVVSEHFEGSGFAIFAKLLEQDGTQIRAIPAPTGGSRKFCDRMNAFAQKEGLPGMGYIFWRDQ-GD--GMEAAGPLAKNIGPERTEAIRVQLGLGVGDAAFFLGGKPKAFEAVAGRARNVIGEELGLTELDRFAFAWIVDFPIYEKDEESGKIDFEHNPFSMPQGGMDALNGDPLEVLGYQYDLACNGYELVSGAIRNHRPEIMFKAFEIAGYGKEEVEKRFGGMVNAFQYGAPPHGGCAAGIDRIVMLLAEESNIREVILFPMNQRAEDVMMNAPNDPMPEQLMELGLRVIPQDMTPLSHIRNFSIVAHIDHGKSTLADRLIQETNTVSLRDMKAQMLDSMDIERERGITIKAQTVRINYTAKNGEDYVLNLIDTPGHVDFAYEVSRSMRAVEGSLLVVDSTQGVEAQTLANVYHAIDADHEIVPILNKIDLPATDCDRVAEQIEDVIGIDASGAIRVSAKTGQGIVETLEAIVHHLPAPKGTLDAPLKAMLVDSWYDSYLGVIVLVRIMDGQLKKGDRITMMQNGSVHHVDRIGVFRPAMTEIDVLGPGEIGFLTASIKQVRDTRVGDTITHEKRKCDKALPGFKPSQPVVFCGLFPVDAALFEDLRDSIEKLALNDASFSYEMETSAALGFGFRCGFLGLLHLEVIRDRIEREYDIDLITTAPSVIYHIHMRDGTMQELHNPADMPDLTFVDHLEEPRIKATILVPDEYLGDVLKLCQDRRGMQMDLTYAGSRAMVVYDLPLNEVVFDFYDRLKSVTKGYASFDYSMIGYREDALVKMSILVNDEPVDALSTMVHRDRAEMRGRAMVEKLKDLIPRHMFKIPIQAAIGGKVIARETLSAMRKDVTAKCYGGDASRKRKLLDKQKAGKKKMRQFGSVNIPQEAFISALKMDGMSLPPGFLDELRTRSSLSQVVGKKVVWDARKSNQGKGDMWAPCPFHQEKSASFHVDDRKGFYYCFGCHAKGDAISFVRETENVTFMEAVEILAGEAGMPMPERDPRAVEKADVRKDLADVMELAVKWFRLQLRTGAAGAAREYLERRGLPQQVCDRWEIGFAPDSWQGLWDALKGKDIPDDLIIGAGLAKPSNKGSKPYDTFRGRIMYPIRDARGRAIAFGGRAMDPEDKAKYLNSPETELFDKGRSLYNVKDARVAAGKGQPLLVAEGYMDVIALSEAGFGASVAPLGTAITENQLAMLWRISDEPIITLDGDAAGQRAALRLIDLALPLLEAGRSLRFAMMPEGKDPDDLLKSSGAGAVQTLLDQAVPMVQLLWSREIEGKNFDSPERKAALDKSLREKTMLIKDPSIRSHYEQALKEMRWDLFRSK----GGMRVAQPRQW-GQWGKAPQGPSAGAKASMLATAADSRATDHLREAVILAACISCPQLVESFESGLERMACLDGDHRRLRDLVLNNAGEGVAVLRAKIAADLGADALETLMGQRHVAITPCIRKPGDIEITKMTIAEELAKLAAVRGLKDEVQDAADDLTGEADEGLTWRLAEAAKAADLAQRAGQEDKAEYVIADNGARLDREQVAKSRSMFDQIDFSKGGKKRMCADTPDYKPTLNLPRTDFPMRAGLPKREPEWLARWEELGIYDRLREKAGRKQFTLHDGPPYANGHLHIGHALNKTIKDMIVRSHQMMGRDARYIPGWDCHGLPIEWKIEEQYRQKGRDKDQVPINEFRAECREFASGWVDIQREEFKRLGITGNWEKPYLTMDFHAERVIAEEFMKFLMTGTLYQGSKPVMWSPVEQTALAEAEVEYHDKESFTVWVKFRVV----GGGDLENAQVVIWTTTPWTMPSNKAVVYGAGISYGLYEISETPDECWASVGDRYLLADNLAADVLGRARLEEGNWTRVRDVTNEELEAISLKHPLAGAEGGNGEWDDLRDFRAADFVTDTEGTGFVHCAPSHGLEEYDLYRELGMLEQVITYNVMPDGRYRDDLPFFGGKAILKPNGKEGNANAAVIDKLVEVGGLLARGKIKHSYPHSWRSKAPVIYRNTPQWFAAIDRPVGDGQDMFGKTIRERALTEIDNVNWTPKSGRNRLHAMMEARPDWVLSRQRAWGVPLTCFTLKGKLPTDPDFLLRNVDVNARIVEAFETEGADAWYEEGAKERFLGGIVNPDDYNQVMDILDVWFDSGSTHAFTLRDREDGSDDGIADVYMEGTDQHRGWFHSSLLQSVGTTGRAPYRNVVTHGFTLDAKGMKMSKSIGNTIVPQKIIDQYGADILRLWVAQTDYTNDQRIGDEILKGTADSYRRLRNTMRYMLGSLPDFDPSRAVTRDDMPELEQLMLHKLAVLDGVVREGYARFDFQGVFRAIFDFATLDLSSFYFDIRKDALYCDGDTTRRLAALTVLDHLYARLTTWLAPILPFTMEEVWLERH-GAETSVHLEDFPETPADWRDDVLAKRAETVRAVRRVVTGALEEQRTAKVIGSSLEAAPTVYLS-----TELAAVIFADLCITSQIMIVQGDAPEGAFKNADIAGVGVTFAKAEGEKCARCWKVLPDVGTHSHEAVCGRCDAAI-MAASQNIRIRLKAFDYRVLDASTQEIVNTAKRTGASVRGPIPLPNKIEKFTVLRGPHVDKKSRDQFEIRTHKRLLDIVDPTPQTVDALMKLDLAAGVDVEIKLQSMADEINTLEDL-----ASVAGIQATPEVELTPREPVKDEFGRAYATGKRKDAVARVWIKPGSGKVVVNGKPQNEYFARPVLQMILQQPFGITGTDGQFDVYATVKGGGLSGQAGAVKHGVSKALQLFDPMHRAALKAAGFLTRDSRVVERKKYGKAKARKSFQFSKRMPTIQQLIRKPRQPKRKYSKSMHLQECPQKRGVCTRVYTTTPKKPNSAMRKVAKVRLTNGFEVISYIPGESHNLQEHSVVLIRGGRVKDLPGVRYHILRGVLDTQGVKDRKQRRSKYGAKRPKMAREAKRTKKKVSKNIAAGVAHVNSSFNNTKILISDVQGNAIAWSSAGTMGFKGSRKSTPYAAQMAAEDAGKKAQDHGVKTLEVEVQGAGSGRESALRALAAAGFNITSIRDVTPMAHNGCRPPKRRRVMT-DRRGLLIILSSPSGAGKSTLAKQLMAWDPSLKFSVSATTRDPREGEVDKRDYFFVSEADFRKAVSQGEMLEHAHVFGNNYGSPKSPVEAAIGAGRDVLFDIDWQGAQQIRTSALGPYTLSIFILPPSIPELHRRLISRGQDTTETIAKRMRKSWDEISHWDGYDYVLVNDSLEETEEKLKTIIKAERMRVAQQPRLSDHVRRLQTEFEELKMAQSFLGQKRLRKYYGKIREVLEMPNLIEVQKSSYDLFLNSGDAETPTDGDGIQGVFQSVFPIKDFNETSILEYVKYELEKPKYDVEECQQRDMTYSAPLKVTLRLIVFDIDEDTGAKSVKDIKEQDVFMGDMPLMTPNGTFIVNGTERVIVSQMHRSPGVFFDHDKGKTHSSGKLLFACRIIPYRGSWLDFEFDAKDIVFARIDRRRKLPVTTLLYSLGLDQEAIMDAYYNTVHYKLEAGKGWIAPFFPERVRGTRPTYDIIDAASGEVLFEAGKKVTPRAVKQLIDEGSVTELLVPFSHIEGKFVSKDIINEETGAIYVEAGDEMTLEYDKDGTLIGGTAKELVDAGITEIPLLDIDNVNVGPYMRNTMAADKNMNRDTALMDIYRVMRPGEPPTVEAASNLFDTLFFDGERYDLSAVGRVKMNMRLALDKEDTQRTLDRDDIVACIKALVDLRDGRGDIDDIDHLGNRRVRSVGELMENQYRVGLLRMERAIKERMSSVEIDTVMPQDLINAKPAAAAVREFFGSSQLSQFMDQTNPLSEVTHKRRLSALGPGGLTRERAGFEVRDVHPTHYGRMCPIETPEGPNIGLINSLATFARVNKYGFIETPYRKVKDGIVSDEVQYMSATEEMRHTVAQANANLDENMKFVNDLVSTRKSGDYTLSPSMNVDLIDVSPKQLVSVAASLIPFLENDDANRALMGSNMQRQAVPLLQAEAPLVGTGIEEVVARDSGAAYMAKRAGVIDQVDATRIVIRATEDLELGDAGVDIYRMRKFQRSNQNTCINQRPLVQVGEVVTKGQVIADGPSTDMGELALGKNVVVAFMPWNGYNYEDSILISERISRDDVFTSIHIEEFEVAARDTKLGPEEITRDIPNVGEEALRNLDEAGIVYIGADVEPGDILVGKITPKGESPMTPEEKLLRAIFGEKASDVRDTSLRVKPGDFGTVVEVRVFNRHGVEKDERALQIEREEVERLARDRDDELAILDRNIFARLREMILGKTAVKGPKGVKPGSQITEETLEVLTRGQWWQLALEDEDDAKIVEALNEQYEIQKRTLDARFEDKVEKVRRGDDLPPGVMKMVKVFVAVKRKLQPGDKMAGRHGNKGVISKVVPMEDMPFLADGTPVDFCLNPLGVPSRMNVGQILETHMGWAARGLGINIDEALQEYKRSGDMTPVREAMKLAYGDDVYDEGIVGMDEDTLLEAAGNVTRGVPIATPVFDGAKEADVNDSLARAGFDTSGQSILFDGRTGEQFSRKVTVGVKYLLKLHHLVDDKIHARSTGPYSLVTQQPLGGKAQFGGQRFGEMEVWALEAYGAAYTLQEMLTVKSDDVAGRTKVYESIVKGEDNFEAGIPESFNVLVKEVRGLGLNMELLDAEDEEMPLYEHVMIARQDLSNTQAEGLIEHFGTVLSDNGGKLVDSEYWGVKTMAYKINKNRKGHYAFLRSDAPAAAVQEMERLMRLHDDVMRVLTIKMDEHKELPSVQMQKRDERPERRERRNNSSVNIDTLSEQDAAAELAALALKLGAANTAYHTQDAPEISDAEYDNLKRRNAAIEARFPALKRNDSPSEQVGASVASGFGKIAHEVRMLSLGNAFTNADVTEFDASIRKYLGLGADDPLKYTAEPKIDGLSLSLRYESGNLVQAATRGDGSVGENVTANARTIADIPQQISGAPEVLEVRGEVYMSHADFEALNRRQEATGAKTFANPRNAAAGSLRQLDAEITRARPLRFFAYAWGSLSAPLADTQSGAIAALEALGFSTNPLTVVCDGPAQLVDHYTKIEAARPQLGYDIDGVVYKVDDLALQSRLGFRSTTPRWAIAHKFPAELAWTTLEGIDIQVGRTGALSPVARLLPVTVGGVVVSNATLHNEDYIRGFDSKGQPIREGKDIRIGDMVQVYRAGDVIPKVADVDITKRPEAAAAFIFPTQCPECGSDAVREAGDAVRRCSGGIICPAQAVEKLKHFVSRAAFDIDGMGAKQVEQFYADGWIKEPADIFALQKRYGAGLQQLKNREGWGEKSASALFAAIEDKRKIPLARLIFGLGIRHVGEASANMIALHYGTWAALRRAMEEARSMEGPAWDDLVSVDGMGSIMAGSLVGAFGQEAELASIERLVSGLSVQDAVRADTSGSPVAGKTVVFTGTLEKMTRAEAKARAERLGAKVAGSVSAKTDLLVAGPGAGSKAKKAADLGIKVLDEDGWLALIDA-MIHKNWAELIKPTQLDVKPGNDPARQATVIAEPLERGFGLTMGNALRRVLMSSLQGAAITSVQIDNVLHEFSSVAGVREDVTDIILNLKGVSIRMEVEGPKRLSISAKGPGVVTAGDITESAGIEILNRDHVICHLDDGADIYMELMVNTGKGYVSADKNKPEDAPIGLIPIDAIYSPVKKVSYDVQPTREGQVLDYDKLTMKVETDGSLTPDDAVAFAARILQDQLGIFVNFDEPESASRQDDDDGLEFNPLLLKKVDELELSVRSANCLKNDNIVYIGDLIQKTEAEMLRTPNFGRKSLNEIKEVLSGMGLHLGMDVEDWPPDNIEDLAKKFEDNFMARDENRGGNRRNQREEAPEFADRLVAINRVSKTVKGGKRFGFAALVVVGDQKGRVGFGKGKAKEVPEAIRKATEQAKRQMIRVQLREGRTLHHDMQGRHGAGKVVMRTAPEGTGIIAGGPMRAVFEMLGIKDVVSKSIGSQNPYNMIRATMDGLKKEQSPRSVAQRRGKKVADILPKRDDATDSSTQVAEEAMTDPIADMLTRIRNSQLRGKSTVMTPASKLRAWVLDVLADEGYIRGYELMTGADGHPAIEISLKYYEGEPVIRELKRISKPGRRVYMGSQDLPSVRQGLGVSIVSTPRGVMSDASARAANVGGEVLCTVFMHDIRAIRDNPDAFDAALARRGDAALSASILTLDSSRRAKIQAAETAQADQNSASKLVGAAKASGNEAEFERLRALVSEKKAEVAAMQSEAKELDVQLTDMLARIPNMPADDVPMGADENDNVEVKKWGDVPVFDFEPKEHYDLGSVAASMDFETAAKTSGARFVMLKRGVARIHRALAQFMLDTHVDENGLTEVNSPVMVRDEAMYGTDKLPKFGDDSYRTTEGMWLVPTSEVPLTYTVAGDTLDAADLPRRMTSHTLCFRSEAGSAGRDTSGMLRQHQFEKVEMVSITLPEASDDEQKRMLACAEGILEKLGVPYRTVLLCTGDMGFGARRTFDIEAWLPGQNAYREISSVSTTGDFQARRMNARFKPADGGKPQFVHTLNGSGLAVGRCLIAVLENGQQADGTVILPAVLAPYLGGKTTLALDGILSMAITASMVKELRDSTGAGMMDAKKALTETDGDMEAAVDWLRTKGLAKAAKKSGRTAAEGLVAVNVAGGRGVAVEVNSETDFVGKNSDFQKMVSGIADVAISVADVDALKAADMGGKSVEQTVTDAVAVIGENMSVRRMSAIEGDVVVSYVHNAAAPGMGNIGVLVAMTGGDEAFGKQVAMHIAAVNPASLSEADLDPAVVEKEKQVQIDIARESGKPDAVIEKMIVGRMQKYMSEVTLLNQAFVINPDLTVGAAAAEAGATITGFVRLEVGEGIEVVKEDFAAEVAKVSKGMNQELTNNPFNPVAPTKTFDEIKVSLASPERILSWSFGEIKKPETINYRTFKPERDGLFCARIFGPIKDYECLCGKYKRMKYRGVVCEKCGVEVTLQKVRRERMGHIELASPVAHIWFLKSLPSRIGLMLDMTLRDLERVLYFENYVVIEPGLTDLTYGQMMTEEEFMDAQDAYGMDAFTANIGAEAIREMLAAIDLEAEADQLREELKEATGELKPKKIIKRLKVVESFLESGNRPEWMVLTVIPVIPPELRPLVPLDGGRFATSDLNDLYRRVINRNNRLKRLIELRAPDIIVRNEKRMLQESVDALFDNGRRGRVITGANKRPLKSLSDMLKGKQGRFRQNLLGKRVDFSGRSVIVTGPELKLHQCGLPKKMALELFKPFIYSRLEAKGLSSTVKQAKKLVEKERPEVWDILDEVIREHPVMLNRAPTLHRLGIQAFEPVLIEGKAIQLHPLVCSAFNADFDGDQMAVHVPLSLEAQLEARVLMMSTNNVLSPANGAPIIVPSQDMILGLYYVTLEREGMVGQGKVFGTVDEVQHALDAGEVHLHAKIKSRIKQIDAEGNEIDVRFDTTPGRVLLGALLPLNAKAPFDLVNRLLRKKEVQQVIDTVYRYCGQKESVIFCDQIMTMGFRQAFKAGISFGKDDMLIPDSKWPLVEETRDQVKDFEQQYMDGLITQGEKYNKVVDAWSKCNDKVTDAMMGAISDTTYAENGSENEPNSVYMMAHSGARGSVTQMKQLGGMRGLMAKPNGDIIETPIISNFKEGLTVLEYFNSTHGARKGLSDTALKTANSGYLTRRLVDVAQDCIVRMHDCGTDVAITATAAVNDGEVVSSLAERLLGRVVAEDLMRPGTDEVLVANGSMIDERLADILDEAGVASARIRSPLTCEAEEGVCAMCYGRDLARGTLVNQGEAVGIIAAQSIGEPGTQLTMRTFHIGGVAQGGQQSFQEASQSGKIHFENSNTLQNSSDETMVMGRNMKLLIIDENGDERASHKVGYGTKLFVKEGATVARGDKLYEWDPYTLPMIAEASGTIKHVDLISGISVKDETDDATGMTQKIVIDWRSAAKGNELKPEIILQDKDGEPLRNSAGNPITYPMSVDAVMSVEDGTEVEAGDVIARIPREGSKTKDITGGLPRVAELFEARRPKDHAIIAEIDGYVRYGKDYKNKRRIAIESSEDPDFKVEYMVPKGKHIPVAEGDFVQKGDYIMDGNPAPHDILAIMGVEALADYMIDEVQDVYRLQGVKINDKHIEVIVRQMLQKWEIQESGDTTLLKGEHVDKQEFDQANEKALSKGGRPAKGEPILLGITKASLQTRSFISAASFQETTRVLTEASVQGKRDKLVGLKENVIVGRLIPAGTGGATQQMRRVASDRDNVVVEARRIEAEKAAALAAPVAPAEDIVGGDVFNQAPSDEDSRDMSRRHAAEKREVLPDAKYGDLILTKFMNNLMIDGKKSVAERIVYNAFDRVESKIKRAPVEVFHEALENIQPSVEVRSRRVGGATYQVPVEVRPERRQALAIRWLIKAARSRNENTMEERLAGELMDAVQSRGTAVKKREDTHKMADANKAFSHYRWMPYAQTDKSEVTAYLANPAEDVRTRAKLEGGKTFVLHTEFEPAGDQPTAIRELSEGIRNGERDQVLLGATGTGKTFTMAKMIEETQRPAIILAPNKTLAAQLYGEFKGFFPDNAVEYFVSYYDYYQPEAYVARSDTFIEKESQINEQIDRMRHSATRSLLERDDVIIIASVSCIYGIGSVETYGAMTQDLKVGTSYDQRQVIADLVAQAYKRNDAGFQRGCFRVRGDTLEIFPAHLDDRAWRLSFFGEELESITEFDPLTGEKTGTFQTVRVYANSHYVTPKPTMNQAIIGIKKELRMRLDQLVGDGKLLEAQRLEQRCNFDLEMLEATGVCNGIENYSRYLTGRAPGEPPPTLFEFIPDNAIVFADESHVSVPQIGGMYKGDFRRKMTLAEHGFRLPSCMDNRPLKFEEWDAMRPQSVFVSATPAAWELEQTGGVFTEQIIRPTGLIDPQIEIRPVDMQVDDLLDEVRKVSAAGMRTLCTTLTKRMAEDLTEYMHEQGIRVRYMHSDIDTIERIEILRDLRLGAFDVLIGINLLREGLDIPECGLVAILDADKEGFLRSETSLIQTIGRAARNAEGRVIMYADRITGSMERAMGETERRRVKQVAYNELHGITPTTVKKNVEDILAGLYKGDTDQSRVTAKIDNPLAGGNLKSVLEGLRTDMRKAAENLEFEEAARLRDEVKRLEAVDLTIADDPMARQYAVDKAVDDAKTKSGRSTMGRGGMRGGVKRR-GRMATKPFFRRRKVCPFSGDNAPAIDYKDTRLLQRYISERGKIVPSRITAVSAKKQRELARAIKRARFLALLPYAVKMPKRILTGTVTSDANEQTVTVSVERRFTHPVLKKTIRKSKKYRAHDENNTFKVGEQVRIIECAPRSKTKRWEVIIAEAMANTPQAKKRARQNEKRFAINKARRSRIRTFIRKVEEAIESGVKDDAVAALRAAQPELMRGVTKGVYHKNTASRKMSRLAARVKAIAMSRSVWKGPFVDSYVLKKAEASRESGRSEVIKIWSRRSTILPQFVGLTFGVYNGHKHIPVNVTEDMIGQKFGEYSPTRTYYGHAADKKAKRKMARIAGVNIPTAKRVPIALTYITGIGNTSAEKICEAVGIEMTRRINELSDAEILKIREHIDENYTVEGDLRRDTQMNIKRLMDLGCYRGLRHRRNLPVRGQRTHTNARTRKGPAKAIAGKKKMSMKIRLARGGSKKRPFYRIVAADSRMPRDGRYIEKLGTYAPLLAKDSEDRVVMNVERIEHWLGQGAQPTERVQRMLEAAGVRPKTERNNPTKGTPGKKATARVEEKAAKAAAAAEAANAPAEETPAEEMSITKEEKTRIMSEFGAKEGDTGSPEVQVAVLSSRIATLTEHFKTHKKDNHGRRGLLKMVATRRKLLDYVKSKDDARYQDLIKRLGLRRMQTLNEIRSNFLTYFGDKGHTVVPSSPLVPRNDPTLMFTAAGMVQFKNLFTGVETRDYTRATTAQKCVRAGGKHNDLDNVGYTARHHTFFEMLGNFSFGDYFKEDAIPMAWDLLTKVFGIDASRLIVTVYHTDDEAVAIWKKHAGLSDDRIIRIATDDNFWSAGPTGPCGPCTEIFYDHGDHIWGGPPGSPEEDGDRFVEIWNLVFMQYEQFEDGTRRDLPNKSIDTGMGIERVAALLQGTNDNYSTDLMRSLIEASANATSSDPDGPGKTHHRVIADHLRSTSFLMADGVMPSNDGRGYVLRRIMRRAMRHAHLLGAKDPLMHRLVPALVAQMGQAYPELGQAQSMIQQTLLQEETRFRQTLDRGLKLLDEELVMLPEGGELAGATAFKLYDTFGFPLDLTQDALREKGRTVDTDGFTSAMAEQKAKARAAWSGSGEAADATIWFDVADKAGVTEFLGYDTETAEGKIAALVQDGSGVVNVSEGGEVQIALNQTPFYAESGGQLGDTGIIRTDTGTARITDTRKAAGVFIHFAKVEKGSISNGQSAVLEVDHGRRTAIRANHSATHLLHEALRHALGDHVAQRGSLNADDRLRFDFSHSETISAEDILTIEAEVNTYIRQNTAVETRIMTPDDARALGAQALFGEKYGDEVRVVSMGRQDASGKGADRNTYSLELCGGTHVARTGDIGAFVLLGDSASSAGVRRIEALTGEAAIAHLRAQDSLLAQAALELKSPASAVPARVRALMDERRALANEVAQLRRELAMSG--GTSAPVAREVNGISFHAQTLSGVTGKDLPALMDEHKARLGSGVVLLIADTGDKAAVAAGVTKDLTDRISAVDILRAAVSELGGKGGGGRPDMAQGGGASAQNAEAAIAAAENILKGMGFKMGIVGLPNVGKSTLFNALTRTAAAQAANFPFCTIEPNVGEVAVPDARLDKLAAIAKSKSIIPTRMTFVDIAGLVKGASKGEGLGNQFLANIRETDAIAHVLRCFEDDDITHVEDRVDPVADAETIDTELMLADLESIEKRRAGLARKIKGNDKDAAQQDRLLAMAQAAIEQGNPARTVSVDADDAKAWRLLQLLTTKPVLYVCNVGVSDAANGNAYSEAVTAMAEAQGNSAVIISAQIEEEISQLDAEDAQMFLDDMKLEEAGLDRLIRAGYELLHLETYFTVGPQEARAWTIKQGTSAPKAAGVIHGDFEKGFIRAETIAYDDFVSLGGEGPAKEAGKMRAEGKAYTVKDGDVLHFLFNNMGWKSLDDMDLHGKRVLVRVDINVPVENFEVTDATRIERIVPTVHDILSKGGTPILLAHFGRPKGKVNLDMSLRQVLPALKRALMRTVALVETLDAAEKFTAEVAAADVVLIENIRFNPGEEANDPAFAARLAQLGDVYCNDAFSAAHRAHASTEGIAHHLPSCAGRLMQAELSALEAALSRPERPVGAVVGGAKVSTKIALLENLVNRLDLLVIGGGMANTFLTAQGAQLGASLCEHDYLETARDIMAQAAKSGCRVLLPVDGLVATEFKAGAAHSVVQLGPDTVLDADQMVLDAGPQTIGQMRAAFENLKTLIWNGPMGAFEISPFDTATVAAAQVAAKQTRDGALISVAGGGDTVAALNQAGVADDFTYISTAGGAFLEWMEGKTLPGVAALGGMGLPEFSMRQLLEAGVHFGHQTQRWNPRMGPYIYGARNGIHIMDLTQTVPMLDDALKIIRDTVAKGGSILFVGTKRQAAQPIADAAEKCAQYYMNHRWLGGTLTNWQTVSKSIQRLKHIDEQSSMGFAGLTKKERLGMERDQAKLEASLGGIREMGGRPDLIFVIDVRKEQLAIAEANKLGIPVVAVVDTNCSPDGIDYIIPGNDDAARAIALYTDLAARAALDGMSAQLGAAGVDLGAMEEAPVEEAIEP--GASAEAAANDAIAKDAESMTKRTAAKHKIDRRMGENIWGRPKSPVNRREYGPGQHGQRRKGKISDFGIQLRAKQKLKGYYGDLTEKQFRRIYGEAERVKGDTGENLIGLLERRLDAVVYRAKFVATVFAARQFVNHRHVRVNGKLVNIPSYRVKEGDVIEVRDRSKQMVALIEATQLSERDVPDYLDVDHSKMTATFVRTPALGDVPYPVVMEPNLVVEFYAKNMGNKVNPIGMRLQVNRTWDSRWYADTKDFGNLLLEDLAIRKFIKKECHQAGISRVIIERPHKKCRVTIHTARPGVIIGKKGADIEGLRKKLSQFTASELHLNIVEVRKPELDAALVGESIAQQLERRVSFRRAMKRAVQNAMRMGALGIRVNLAGRLGGAEIARTEWYREGRVPLHTLRADIDYAHVEAMTAYGIIGIKTWIFKGEIMEHDPAARDRKAQEMQDGPAPRGAGGRRMARRRKGRDISGWVVIDKPAGPTSTTVVNKVRWAFDARKAGHAGTLDPDATGVLAIALGEATKTVPFITDALKAYVFTVRLGAATNTDDLEGEIIATSDLRPDDATIKEALHAFVGDIGQVPPQFSAVKIDGERAYKRARDGETMEIAARPLYVESLIMLERPDADHVTLEMVCGKGGYVRSIARDLGEVLGCKGHVRELRRTWSGPFDIANAITLAQVEEMAKTPALDDYLGPLEMGLQDLPQVTASSEGATRLRNGNPGMVFAKDVEYGDECWAVHEGTAVAVGIYKSGELHPSRVFN-PSMFLLGLTGSIGMGKSTTAKMFVDEGCALWDADAAVHRLYALGGAAVPPFLAAFPQAIVDGAVSRPALKEIIGGNPAALNQIEAIIHPLVGQDRADFLAKTKADIVVLDIPLLFETGGDARVHATACVYTDDATQETRVLARGTMTREQFLAIKAKQLPAADKCARATYVIQTDTLEHARAQVQTIVKTIRSQPHA-MNTLDILVECGGWDEKLIVPLAQRAVVATLHHMQLEPDDCEVTVMACDDARIAVLNAEFRGKPVATNVLSWPAQRLAPPAEGGVPPLPEKGFDGMLELGDIALSFETCTREAMEASKPVADHLTHLIVHGTLHLLGYDHITDGDAALMERIEVEILGNLGLDDPYTADITMARYIFITGGVVSSLGKGLASAALGALLQARGFSVRLRKLDPYLNVDPGTMSPFEHGEVFVTDDGAETDLDLGHYERFTGVSARKTDSISSGRVYSTVLEKERRGDYLGKTIQVIPHVTNEIKEFLSIGDDEVDFMLCEIGGTVGDIEGLPFFEAIRQFSHDKPRGQCIFMHLTLLPYLAASGELKTKPTQHSVKELQSIGIAPDILVCRSEHPIPDKEREKIALFCNVRKDSVVAAYDLKSIYEAPLAYHAQGLDQAVLDAFDISPAPKPDLTVWHDVYDRIHNPEGEVKVAIVGKYTQLEDAYKSIAEALTHGGMANRVRVKIEWVDAELFETDDVAPHLEGFHAILVPGGFGERGTEGKIKAAQYAREHKVPYLGICLGMQMAVIEAARNVAGLSTAGSEEFDHESGKKRFEPVVYHLKEWVQGNHKVERKVGDDKGGTMRLGAYDATLKEGSKVAEVYGTTAIDERHRHRYEVDIAYREKLEEAGMSFSGMSPDGKLPEIVEWSDHPWFIGVQFHPELKSKPFKPHPLFKGFVKAAKDMSRLVLMSSLTILGIESSCDDTAAAVLR-GHAGSADVMSSVVMGQTALHANFGGVVPEIAARAHAEKLDLCVVQALDKAGVGLEDIDAVAVTAGPGLIGGVVSGVMCAKGIALARDLPLYGVNHLAGHALTPRLTHGVAFPYLMLLVSGGHCQFLIVSGPDEFKRLGGTIDDAPGEAFDKVARLIALPQPGGPAIEACAFNGDASRFKLPRPLLDRAGCDMSFSGLKTAVLRQRDSLLVE-AKLKSQDQADLAAGFQAAVVDVLSEKTRRAMAIYTPLTS-TPAICVAGGVASNMAIRSAIQSIAAEFGAAFIAPPLALCTDNAAMIAYAALEQMGTRNPDGMALSARPRWPLDQTAPAMLGSGKKGAKA------------------------------------------------------------------------------------------------------------------------------------

>'Roseivivax-haloduransJCM-10272'

MNASDLRDKTPDQLRDQLVQLKKEAFNLRFQQATGQLENTARMKLVRRDVAKVKTILNEKAASAAATTMAAEDLLDMDSKRDANKQKALDSALAQIERQFGKGSIMKLGGDNPIKDIEATSTGSLGLDIALGIGGLPKGRVVEIYGPESSGKTTLTLHCVAEEQKKGGVCAFVDAEHALDPQYAKKLGVDLDELLISQPDTGEQALEITDTLVRSGAVSMVVVDSVAALTPKSELEGDMGDSSVGVQARLMSQAMRKLTSSISRSNCMVIFINQIRMKIGVMFGSPETTTGGNALKFYSSVRLDIRRIGSIKDRDEVVGNATRVKVVKNKVAPPFKQVEFDIMYGEGISKTGELLDLGVKAGVVEKSGAWYSYGDERIGQGRENAKKFLKENSATAYEIEDKIRAAHGLDFHMSEDDDDEVVEGMSRYAPAEIEPKWQKAWDEALTFRAERKDGKPKYYVLEMFPYPSGRIHIGHVRNYTMGDVVARYKRATGHNVLHPMGFDAFGMPAENAAMASDGHPKDWTYGNIDTMVEQMKPLGLSIDWSRMFATCDPDYYGQQQALFLDFLEQGLVYRKNAVVNWDPVDMTVLANEQVEDGRGWRSGALVERRELTQWFFRISDFAEDLLEALDGLDDWPAKVKTMQANWIGRSRGLQFAFSTIDAPEGHDRIEVYTTRPDTLLGASFVGISPDHPLAKQLERENEEVAAFNAECRRMGTSEEELETAEKKGFDTGITVRHPFDTSWELPVYIANFILMDYGTGAIFGCPAHDPRDLEFARKYELPVQTVFAPAMEGPEDGGPAYVPPKVETVRYLKPFAGEEAMTGDVAIDAAIAFCEENGVGQGVTKFRLRDWGLSRQRYWGCPIPVIHCETCGVVPEKKENLPVTLPYDIDFSVPGNPLDRHPSWRDVSCPSCGAAARRETDTMDTFVDSSWYFARFTAPKAETPTDMADAEYWMNVDQYIGGIEHAILHLLYARFFSRAMHLTGHLPEKSREPFDALFTQGMVTHAIFQTKDKAGRPVFHFPEDVRDNSAFKDGTEVEIVPSAKMSKSKKNVVDPVEIVTQYGADTARWFVLSDSPPERDVEWTAAGAEAASKHLGRVWRIASEAAASE-AASGEGDEPLLREMHKTIHDVTQGIDSFGFNAAIARLYGFTSTLSKSDAGADAKRQAARTLAQLMSPMTPHLAEEMWATLGGEGLVADAPWPVADESMMVEDTVTMPIQINGKRRGEVVVPKEMEKAEVEELALSHHAVLKALDGASPKKVIVVPGRIVNVVVMAITSANQLELLQTAEAVAREKMIDPSLVVEAMEESLARAAKSRYGSEMDIRVKIDRRTGRANFTRVRTVVEDEELENYQAELTVEQAKQYLEDPKVGDQYIEEVPPVEMGRIAAQSAKQVILQKVREAERDRQYEEFKDRAGTIINGVVKREEYGNVIVDIGRGEGILRRNEKIGREAYRPNDRIRCYIKDVRREARGPQIFLSRTAPEFMAELFKMEVPEIYDGIIEIKAVARDPGSRAKIGVISYDGSIDPVGACVGMRGSRVQAVVNELQGEKIDIIPWNEDMPTFLVNALQPAEVTKVVLDEDAERIEVVVPDEQLSLAIGRRGQNVRLASQLTGLDIDIMTEEEESKRRQAEFEERTKLFMDNLDLDEFFAQLLVSEGFTTLEEVAYVEIDELTVIDGVDDDTAEELQARARDVLEAQAKAALDNARALGAEDSLIDFEGLTPQMVEALAKDDIKTLEDFATCADWELAGGWTTVDGQRHKDDGLLEPFDVGLEEAQNMVMTARVLLGWVDPTEMEPEAED---DGETEETEEA--MAHKKAGGSSRNGRDSAGRRLGVKLYGGERAIPGNIIVRQRGTKFWPGNGVGMGKDHTIFATGEGSVTFHKGLKGRTYISVLPVAEAAEMAKRWYSVSVLSNFEKKVAEQIRTSVAEQGLEDQIEEVLVPTEEVIEIRRGKKVTAERRFMPGYVLVRMEMSDAGYHLINSINRVTGFLGPQGRPMPMRDAEVQAILGRVQEGEESPRTLIHFEVGEKVKVNDGPFEDFDGMVEGVDEDNQRLRVSVSIFGRETPVELEFTQVTKQGMSFTLAIVGRPNVGKSTLFNRLVGRRLAIVDDQPGVTRDLREGEAKLGDLTFTVLDTAGLEDATDDSLPARMRRLTERAVDMADVCLFVVDARTGITPTDEIFAEILRKRAGHVLLAVNKAEGRNTEGGVLEAWNLGLGEPIPLSAEHGEGMPDLYAALMPLADGFAERAPDE-PDLGLPVSEEA-DE-GMQVPTLEKPLQIAVVGRPNAGKSTLINKLLGEERLLTGPEAGITRDAISVMMDWEG-VPTRIFDTAGMRKKAKVQEKLEKLSVADGLRAVKFAEVVVVLLDAAIPFEQQDLRIADLAEREGRAVVVAVNKWDLEEDKGAKLKELREAFDRLLPQLRGAPLVTVSAKTGRGLDRLRGAVIRAHEVWNTRVPTAKLNRWLGEMIEAHPPPAPGGRRIRLRYMTQAKTRPPHFVVMCSHPDKMQDSYTRYLVNGLRADFDMPGTPIRLVMRGQGARNPYRGKREKNAGALQKHLDKRKG-MRLVFMGTPDFSVPVLDALIGAGHEIACVYCQPPRPAGRGKKDRPSPVQARAEALGLPVRHPVSLKGAEEQEAFAALGAEAAVVVAYGLILPRAILGAPERGCLNIHASLLPRWRGAAPIQRAIMAGDARTGVCIMQMEVGLDTGPVLLRRETEIGAEETTGDLHDRLSALGAEAVVEALARIDALSPETQPDEGVTYAEKVDKAEARVDWTEPAEIVARKIRGLAPFPGAWTEAGEARIKLLGARAVPGTGAPGEVL-GGFTVACGTGAVEVTRAQRAGKGAQEAQTFLLGMALP--ARLGA-MSDSPAYQVLARKYRPETFADLVGQDAMVRTLRNAFAADRIAQAFIMTGIRGTGKTTTARIIAKGMNCIGPDGTGGPTTDPCGICEHCVAIMEGRHVDVLEMDAASNTGVGDVREIIDSVHYRAASARYKIYIIDEVHMLSTSAFNALLKTLEEPPEHVKFIFATTEIRKVPVTVLSRCQRFDLRRIEPEVMLGLLRRIADKEGAEIADEALALITRAAEGSARDATSLLDQAISHAGGETTAEEVRAMLGLADRGRVMDLFELIMKGDAAGALSELSAQYSDGADPMAVLRDLAETTHWVSVVKITPDAAEDPTVGPEERTRGLAFAEGLGMRPLTRLWQMLLKALDEVAQAPNAMMAAEMAIIRMTHVAELPSPEDLVRKLQSETPPPPPP-NGGGGGAHGGATG--AQGAMTPSAPSGPVASGAATARAVAAEEALSRYPTFDHVVELIRANRDGKLLIEVETNVRLAAYQPGRIEFTPTDDAPGDLAQRLGAALARWTGNRWAVTLVNGAETATIYEQRNAERLAREAEAAEHPMVKAVLEAFPKAKIAEIRTEEEVAQEAQAEALPEVEDEWDPFEEEMPKMKTKSSAKKRFKVTSSGRVVAAQAGKQHGMIKRTKKFIRDARGTTTLSKPDEKIAKGFMPYDRMNLFTDIGGLVTDAIGALQADGTLPEGLNLGPVTVEPPRDPAHGDMATNVAMVLAKPAGKKPRDIAEALVPKLAADPRVASAEVAGPGFINLRLSDGVWQEVTKAVLADGERYGRSAMGQGQSVNVEYVSANPTGPLHVGHTRGAVFGDALASLYDYAGYDVTREYYINDGGAQVDTLARSVYLRYIEAHGRDVEFADGTYPGEYLIATGEALKAKVGDAYVDAPEEEWLAEVREFATDAMLDLIRGDLKLLGVEMDRFFSEKSLYGTGLIEQAIEALRGKDLIYRGTLEPPKGKTPEDWEPREQTLFRSTAHGDDVDRPIMKSDGAWTYFAPDIAYHWDKVSRGYDLLVDVFGADHGGYVKRMKAAVSALSDGNVPLEVKLCQLVKLYKNGEPFKMSKRAGTFITLRDVVEQVGPDVTRFVMLTRKNDAPLDFDFDKVLEQSKENPVFYVQYAHARVRSVQRKAEAAGIDVSDAALSGADLSSLTHEAELALARKIAEWPRQVEIAAKTSEPHRIAFYLYDLASELHALWNRGNDEPSLRFLQEDDPDTSRSKIALARAASVVISAGLGILGVTPAEEMR-MAIDELPPLREVIAAHGLSARKALGQNFLLDLNLTAKIARNAGDLSGFDVLEVGPGPGGLTRGLLAEGARRVLAIEKDPRCLPALEEIAEHYPGRLRVIEGDALDIDPLEHLTPPIAIAANLPYNVGTELLVRWLTPETWPPFWTSLTLMFQREVAERIVAEPGSKAYGRLAVLAQWRTDARIVMSLPPGAFTPPPKVSSAVVRLTALPEPKFPANPKVLERVVAMAFNQRRKMLRSALKGLHPDVEDLLKSAGISPTDRAEQVALEQFCALARAVEAEMDLIATLEAEQIASLGKSIPDFKAGDTVRVGYKVTEGTRTRVQNFEGVVISRKNGSGIAGTFTVRKISFGEGVERVFPLYSTNIESIDVVRRGKVRRAKLYYLRSRRGKSARIAEDTNYKPVQGSSTMAYSKRVQFLKRRLRVRNKLRAVNEGRMRLSVHRSNKNLHVQLIDDVNGHTVAAASTLEKDLGVVGKNSVEAAQKIGAAIAERAKKAGVEEAYFDRGGFLYHGRVKAVADAAREAGLKIMFAVMKTGGKQYTVRSGDTLRIEKLAADAGETVQFNEILMLGGDETTIGAPFVDGAAVQAEVVDQMKGEKVIHFVKRRRKHSSKRTKGHRQQLTTVRVTEILTSGGDKTGVKAAIGSGAAGAAGLGADMKA--ASEDQAQK---PTTE-------ATGAR------DDLKQLSGVGPALEKKLNEAGINTFAQIAAWTETDLEALDE--TIRNKAEKEGWVAQAADLQK-MSRTKGGTVTHARHKKITKAAKGYFGRRKNTFRTATQAVDKANQYATRDRKARKRNFRALWIQRINAAVRTHDETLTYSRFVNGLSLAGIEVDRKVLADLAVHEPEAFGAIVEKAKGALAAMQVTETLNDGLKRGYAITLTADELDAKVTEKLTEAQPDVEMKGFRKGKVPLALLRKQFGQRILGESMQEAIDGAMNQHFEDTGDRPAMQPEVKMTNEDWSEGDDVHVEMSYEKLPDVPEVDLTELSVEKPVAKASEADVDDALKSLAETATDFEDREDGAAAENDDQVVIDFVGKVDGEPFEGGAAEDYPLTLGSNSFIPGFEEQLVGVKAGDEKTVEVKFPEEYGAENLAGKDATFDVTVKNVKKPVAAEINDELATKFGAEDLEALKGQIRERLEAEYQGAARQVVKRRLLDALDEKVQFDLPPSLVEAEAKQIAHQLYHEDHPDDHGHDHGEIEPTEEHNKLAERRVRLGLLLAEIGNKHEVQVTDSEMTQAIMNQARQYPGQERQFFDFVRQNQQMQQQMRAPIFEDKVVDVILEKAEVSETEVGKEDLQKLLDDLDEVMKLHELRDNDGANKKAKRIGRGPGSGKGKMGGRGIKGQKSRSGVAINGYEGGQMPLYQRLPKRGFNKPNRKSYAVVNLGMIDKFVLAGKIDAGQPVTEEVLVASGLVRRKLDGVRVLAKGELSKAVNLEVTGASKSAVDAVEKAGGSLKLTGAQASE---MIQMQTNLDVADNSGARKVQCIKVLGGSKRKYASVGDIIVVSVKEAIPRGRVKKGDVRKAVVVRTAKEVRRDDGTAIRFDRNAAVILNNSNEPVGTRIFGPVVRELRAKNFMKIISLAPEVLMRHGHGYRKLNRTHEHRKALWANMAGSLIEHEQIKTTLPKAKELRRVIEKLVTLGKRGDLHARRQAAAQLKQDIHVAKLFDVIGPRYAERSGGYVRVLKAGFRYGDMAPMAIIEFVERDVDAKGKADRERVEAEESAEGMLQPKRTKYRKQQKGRIKGEAKGGSSLNFGSFGLKATQPERITARQIEAARRTMTRHMKRQGRVWIRIFPDVPVSAKPIEVRMGKGKGSVDRWACKVKPGRVMFEIDGVSDEVAREALRLASMKLPIKTRIVVREDW-MDIRLTNTKTRAKEVFTPIDPENVRMYVCGPTVYDRAHLGNARPVVVFDVLFRLLRHVYGADHVTYVRNFTDVDDKINARAEATGRDIGEITEETIGWFLEDMGALGALEPTHAPRATRYIGEMVAMIEKLIADGHAYEAEGHVLFAVESFEAYGALSGRSVDDMIAGARVEVAPYKRNPMDFVLWKPSGEGLPGWDSPWGRGRPGWHIECSAMADALLGETFDIHGGGNDLMFPHHENELAQSCCAHPEGGFAKVWLHNEMLQVEGKKMSKSLGNFFTVRDLLDEGIPGEVVRMVLMSAHYRKPMDWTAQKRREAEAALRKWYALVDGE--GPHAD--VVAALTDDLNTAGAISVLHQLAAAEDGPALRASMKFLGLMSGAVPDWASAPGADLSRWEDRLASARAQAMESKDFSEVDRLKGVLKDAGVEVRMLKAGVELLPGPDFDASRLEEA-MAK--DKSKESPNYKVIAENRRARFDYAIEDDVECGIVLTGSEVKSLRLNTAQIAESYAEVKDGELWLANGYIAPYEQA-MFGHEDRRPRKLLASKREIARMWNETARKGMTLVPLVLYFNHRGVAKIKIALAKGKRTVDKRETQAKRDWNRQKQRLLKEQNMSAKAEHYDVIRKPVITEKSTMASEHNGVVFEVAIDANKPQIKEAVEALFGVKVKAVNTLIAKGKVKRFRGQKGRRADTKKAYVTLVEGNTIDVTTGLMGKDKNPRRVADNEAMAKLRMLRTSPQKLNLVAALIRGKKVDRALNDLTFSKKRISDDVKKCLQSAIANAENNHGLDVDELIVAEAYVGKNLVMKRGRPRARGRFGKILKPFSEITILVRQVEEQAMASAVEQMAANTSWAALGKATDLRNRILYTLALLCIFRLGTFIPVPGIDGVALRDFMEGAQAGIGGILTMFTGGALGRMGIFALGIMPYISASIIVQLLTSMVPSLEQLKKEGEQGRKKINQYTRYGTVLLAVFQAYGLAMSLEAGDLAHDPGWYFRASTVITIVGGTMFLMWLGEQITQRGIGNGISLIIFVGIIAEVPAALAQFFASGRSGAISPAVIIGVVLFMVATIMFVVFMERALRKITIQYPRRQVGMKMTEAQNSHLPVKVNPAGVIPAIFASSLLLLPTTISTFSGESTGPVMSTIMAYFGPGQPLYLLFFAAMIVFFAYFYTFNVSFKPDDVAENLKNQNGFVPGIRPGKRTSEYLEYVVNRVLVLGSGYLALVCLMPEILRNQFAIPFYFGGTSVLIVVSVTMDTIQQVQSHLLAHQYEGLIQKSQLRGKG-KGRKKGGPARRMKFLDLCKVYIRSGSGGGGSVSFRREKYIEYGGPDGGDGGSGGSVYAEAVDGLNTLIDFRYQQHFFAENGRPGMGSQRTGAGGDDIVLRVPVGTEILDEDGETVLYDLSAVGDRICLAQGGNGGWGNVRFKSSTNQAPRRANSGQPGVERTIWLRLKLIADVGLLGLPNAGKSTFLAATSNARPKVADYPFTTLHPNLGVVGVDDVEFVVADIPGLIEGAHEGRGLGDLFLGHVERCAALLHLVDGTSGTLIEDYKTILTEIEAYGEGLADKPRVTVLNKIDALDDEERDFLKDELEAVAGP-VHLMSGVSKEGLTDVLRELRALIDDQRLRRRKEEE-GTWQPMAAKLRKGDKVVVLAGKDKGKEGTISAVMPKDGKAVVDGINMAVRHQRQTQSNQGGRQPKAVPIDLSNLALVDKNGKATRVGFRMDGDKKVRFAKTTGDAIDA-MRSGIIAKKVGMTRIFQEDGKQVPVTVLQLDKLQVVAQRTVDSHGYSAVQLGCGAIKPKNVSKPMRGHFAIAKVEPKRKVAEFRVAPENMIDVGEEITADHYFAGQFVDVCGTSIGKGFAGGMKRHNFRGLEATHGVSISHRSHGSTGQCQDPGKVFKGKKMAGHMGAVRVTTQNLQVVKTDADRGLIMIKGAVPGAKGGWVTIKDAVKKPAPEGIILPAALKSAADEAKRAAEEAAKQAEEEARQAEEARLAEEAAQQEEALKEAEAEIAEESADTDNSDA-EKKDGQAMALKSYKPTTPGQRGLVLIDRSELYKGRPVKSLTEGLTKKGGRNNTGRITMRRRGGGAKRLYRIVDFKRNKFDVQGTVARIEYDPNRTAFIALIQYDDGEQAYILAPQRLAIGDRVISGKKADIKPGNAMPFSGMPIGTIVHNIELKPGKGGQIARAAGTYAQFVGRDGGYAQVRLSSGELRMVRQECMATVGAVSNPDNSNQNFGKAGRMRHLGKRPHVRGVVMNPIDHPHGGGEGRTSGGRHPVTPWGKPTKGHRTRNTNKGSQKLIIRSRHARKKGRMLDEATYTPRLRQQFREEIRAKLKEEFGYTNDMQIPRLDKIVLNIGCGAEAVRDSKKAKSAQEDLTKIAGQHAVVTKAKKSIAGFRVREEMPLGAKVTLRGDRMYEFLDRLITIAMPRIRDFRGVK-PSFDGQGNFAMGIKEHIVFPEIDFDKVDEVWGMDVIIATTAGTDAEGKALLKHFNMPFTSMKLDVIKLDGGSAGSVDLADEIYDLEPRADILHRVVRWQRNKAMQGTHSTLGRSETSYSTKKIYRQKGTGGARHGDRNAPIFRKGGIYKGPKPRSHAHDLPKKVRRLGLMHALSAKAREGNLVVIDSAETDGKTKTLAKAIKDLGWKRTLVIDGAEVNGDFARAAANIDGLDVLPSMGANVYDILKRDTLVITKAGIEALEARLKMENVILIVHLLLALSLIGVVLMQRSEGGGLGMGGGGGGAMSARGAATALNKLTWILATAFICTSLVLTILAAADTSSGSIMDRLGSAPAAE---DGSGDGLTDSLLPPPSADDAPLVPRADMSFFGKLKSRLMRSSSKLEEGLDAIVEEGGSADPETP---------PAPDT-------DPPPDPAPDLPPEPPGVPEEAPVPDPVEIPDPAPGPADPAPE-------PVPEPSELTPMPQEELPSAPSAPAEVPAPPENRNTGLFGRLFGRGPSEPVVRRTLDDEMVESLEELLISADMGVDTALRVAGNMAEGRYGRRVSTQEIKELLAAEIARIMEPVAKPLPLYRSKPQVVLVVGVNGSGKTTTIGKLASQFTQAGKKVVIAAGDTFRAAAVEQLQIWGDRAGVPVLTAPEGSDPASLAFDAFTKAEADGADLLMIDTAGRLQNRQDLMEELAKIVRVLRKKDPDAPHNTLLVLDATTGQNALSQVETFQKLADVSGLVMTKLDGTAKGGVLVALADKFGLPIHAIGVGEQIDDLAPFDPEEFAAALTGLEQMTQEQWGALRDTLCETIGENNYKTWIEPLSFDGCENGIATFRAPTNFMGNYVSQNFGDVMLSHMNAISGDVRRINFRVQSANERGAPGPAATAGAGAAMPKSAVSADRSLPGAPLDQRYTFDSFVVGKPNELAHAAARRVSESGHVTFNPLFLHGGVGLGKTHLMHAIAWELRAKNPELNVLYLSAEQFMYRFVQALRDRRMMDFKSIFRSVDVLMVDDVQFIAGKDSTQEEFFHTFNALVDQNKQIVISADRAPTDIKDLENRIQSRLQSGLVVDLHPTDYELRLGILQTKVEQQAQHYPDVRIANGVLEFLAHRISNNVRVLEGALTRLFAFASLVGREVDLDLTQECLTDVLRANERKVSIEEIQRKVAEHYNIRLADMIGPRRTRTFARPRQVAMFLCKKMTARSLPEIGRRFGGRDHTTVMHGVKRIEELSRQDGQITEDLELLRRALEAMLGIGSITRKVFGTPNDRKIKATRPLVEKINALEPDFEALDDAGLVSKTQEFKGRLGKGESLDDLLPEAFANCREAAKRALGLRAFDVQLMGGIFMHQGNIAEMKTGEGKTLVATFPAYLNALAGP-VHIVTVNDYLARRDAEWMSKVYGALGLSTGVVYPRQPEDEKKEAYKADVTYATNNELGFDYLRDNMKSELDQMAQRGHSFAIVDEVDSILIDEARTPLIISGPAQDRSQLYEGIDRVIPELTDEHYTIDEKTRNVTFTDEGNEFLEERLSQMGLIEEGRTLYDPESTTVVHHLSQGLRAHKLFQRDKDYIVRDGEVVLIDEFTGRMMPGRRLSDGLHQAIEAKEGAKIQPENVTLASVTFQNYFRLYDKLSGMTGTASTEADEFAEIYKLGVVEVPTNKPIARVDEDDQVYRTAKEKYAAIVEEIGRAHAKGQPVLVGTTSIEKSEMLSQLLKDAGLEHNVLNARQHESEAQIVGDAGKFGAVTIATNMAGRGTDIKLGGNVDFKIMDALEADPNGDPEEIRARIEKEHEGEEAKVIESGGLFVLATERHESRRIDNQLRGRSGRQGDPGRSSFYLSLEDDLMRIFGSERLDNVLGKLGMKEGEAIVHPWVNKSLERAQAKVEGRNFDIRKQLLKFDDVMNDQRKVIFGQRREIMEAEDVTEITEDMRHQVIDDLVDAYCPPRSYAEQWNLEGLYAACIEKLGLDLPIMTWGQEDGVDQEVMRERIGEASDKLMSEKAEAFGEDTMRQIEKQMLLQTIDGKWRDHLLTLEHLRSVVGFRGYAQRDPLNEYKTESFQLFQGMLDSLREDVTTKLAHIQPMSEAQRQELIDQMRRQQEQMQQAAKQAGATNPEAEAGPAPLAEGFDETDPSTWGNPGRNEPCPCGSGNKFKHCHGRLSMKRSAALKDLEGRLGHRFDRPELLTRALTHGSMSGPTREDNQRLEFLGDRVLGLVMAEALLERDPDAAEGQLAPRYNALVRKETCAEVARDCAIGDAMKLGRSERLSGGRRKMALLGDAMEAVIAAVYVDAGFEVAKDMILRLWGDRVDRVEEDARDAKTALQEWAQARGLPPPSYTETGREGPDHQPVFTIEVRIRTGESDSAQAPSKRQAEQAAASALLGRLEGGAMGKIGKRLRAAREAFAGKENLSVEEAVSLIKSNSQTKFDQTLEIAMNLGVDPRHADQMVRGVVGLPNGTGKTIRVAVFARGAKADEAKEAGADIVGAEDLMEQIQNGQINFERCIATPDMMPVVGRLGKILGPRNLMPNPKVGTVTMDVGQAVKDAKGGQVQFKAEKAGVIHAGIGKVSFDEAKLAENVRAFVEAVSRAKPAGAKGTYMKKVVLSSSMGPGVSVDITNATQGMADDFELDTDDLERRMEGAMSNLRTEFASLRTGRASSSMLEPVQVEAYGQMTPINQVGTVNVPEPRMVTINVWDKSLVNKVEKAIRDSGLGINPQMNGTIIMLPIPELNEERRRELGKVAGQYAEGARVAIRNVRQDGMQTLKRNK-DNMSEDDQKFWENEVQTLTDTYISKVDKALETKQEEIMQVMAKKLAGKMKLQIPAGKANPSPPVGPALGQRGINIMEFCKAFNAKTQEMEPGAPCPTLITYYQDKSFTMDIKTPPASYYLKKAAGLQKGATTPGRETVASVTRKQVREIAEAKMRDLSANSVDDAMKIILGSAKSMGIEVKMFENLSDRLGSVFDRLTKQGALSEDDVKTALREVRVALLEADVSLPVVRNFVKSVEKKATGAAVTKSITPGQQVVKIVHDELIEVLRGEGEPGELRIGSAPAPILMVGLQGGGKTTTTAKLAKRLTERDKKKVLMASLDVQRPAAMEQLAVLGTQIGVDTLPIVKGEDPVAIAKRAKTQASLGGYDVYMLDTAGRLSIDEELMAQVEAVRDVTSPRETLLVVDGLTGQDAVHTAENFDERIGISGVVLTRMDGDGRGGAALSMRAITGKPIKFVGLGEKMDALETFEPERVAGRILGMGDIVALVEKAQQTLEAEQAERMMKRFQKGQFNMNDLKMQLEQMQKMGGMEGMMSMMPGMGKMAKQMEGAGIDDTMLRRQVALINSMTKKERANPALLQASRKKRIAKGSGLEVSELNKLLKQQRQMGDMMK---KMGKGGMLKQAMSGMFGKGGMDPS----KMDPKQLEAAAKAMGGKMPGGMGGAGGGPGLPPGLSGMGKKKMIPEDRLRQIVERFEYLEACMAEGR--GDIAALGREYAELRPVVERIAEWRALTAGIADADAMMDDPEMGELAEAESRELRARLPEAEHALNLALLPKDAADARSAVLEIRPGTGGEEAALFAGDLQRMYMRHAEAQGWSFEVVEEQATELGGVKELVARISGDGVFARLKFESGVHRVQRVPATESGGRIHTSAATVAVLPEAEEVDIAIDPNDLRIDTMRSSGAGGQHVNTTDSAVRITHLPTGIMVTSSEKSQHRNREIAMNVLRARLYDDERRRAAAERSDMRKGQVGTGDRSERIRTYNFPQGRLTDHRINLTLYKLDAVMAGDLDEIIDALTADAQATLLAEMSEMDRAQKEKVVEELGQIFESSGVVVVSRYQGLTVAEMQDLRGRAREGGASVRVAKNRLAKIALEGKDCESMSKYLEGMTVLTYSEDPVAAAKVVEDFAKDNKKLEILGGAMGGEALDRAGVEAVSNMPSRDELLAQIAGMIGAPASGIAGAIGAPASNIASILSTIEEKAEAMKTFSATPADIDKKWIVIDAEGLVLGRLASVVASRLRGKHKPSFTPHIDMGDNVIVINADKVQLTGKKRQEHFYWHTGYPGGIKSRTKEQILEGAHPERVVFQAVKRMLPGNKLAKTQLTNLRVYAGTEHPHDAQSPEVLDVKAMNPKNTRTAMTELETRFASEMGRLLGPDFPSEIGLAVSGGGDSMAMLALAHGWARVFGIRLWVVTIDHGLRADSASEAEMVARECALLGHSHAILRWH-WSGQGNVMDAARRARLRLIDGWRGR--LVHVAMAHTRDDLAETFLMRLARGAGVDGLAAMKSPAPGSAFTLIRPCLGMGREELRHYNRTLKVPWVDDPTNADPDYERARIRAALPAVAEAGIDVAALADAANRLGRAREALKRRASEAAERISSVPTGELHFDRDSFAALDRETQARLLVGALRWVAGGDYKPRAAPLEALLDRLLSGGGGTLAGCEAGCDRSALWVAREPAAVKGLS-AAGALWDGRWRLSGSA--AEGLEVRALGEEGWQQVPEKPADVPPHRRARALPALWDGDRLVASVHLGVGPGHDVEILRGANRFADFLLSDMADLKALAEQIVGLTLLEAQELKTILKDEYGIEPAAGGAVMMAGPGDAGAAEEEQTEFDVILKSAGASKINVIKEVRAITGLGLKEAKELVEAGGKVKEGASKDEAEEIKKKLEDAGAEIELKMSRIGKRPVELPSGVSASVSGQTVEVKGPKGTKTFHATDDVTITVEDDKISVTPRGNSKRARQQWGMTRTIVANLVHGVQNTFKKELEIQGVGYRAQVQGNILKLNLGLSHDVDFPIPEGVTVTATKPTELVVEGHDYQQVGQVAANIRDWRRPEPYKGKGIRYKGEYIFQKEGKKKMQVILLERVAKLGQMGDVVDVKPGYARNFLLLQGKALTASKENIARFESEKANLEAKNLETRKEAEAVAEKLDGEQFVVIRQASDGGNLYGSVTTRDAAEVCKDAGFDVDRKQVIIREPIKELGLHAVELHLHPEVIVEVTLNVARSPEEAEIQKAGKSIQDIAAEEEAQAEFEISELFDDMGAAQLDETETEQAADEPAVQDKDELDDRLMSDQDGKKTLGVRGGPRSGNVKQSFSHGRTKNVVVETKRKRTVAPKPGTGSGSGKSLAAAAAGAKRPAGISDAEMERRLKALQAAKAREADDAAKREAEEKARAEERDRMRAEKEQKEREQREAEERAKAKVEEEERKKREEADAAKAAEAPAQAPPAQQPRGGRAAP-SKQQERKTEREGRG-KSK-GDGNRRTGKLTVSQALSGGEGGRQKSMAAMKRKQERARQKAMGGPVQREKVMRDVRLPEAITVAELANRMTERVGDVVKALMQNGVMATQNQTIDADTAELIIEEFGHTVVRVSDADVEDVIATIEDKAEDLKPRAPIITIMGHVDHGKTSLLDAIRKTKVVSGEAGGITQHIGAYQVDPSTGATLTFLDTPGHAAFTSMRSRGAQVTDIVVLVVAADDAVMPQTIEAINHAKAAKVPMIVAINKCDKPEANPTKVRTDLLQHEIVVEEMSGDVQDIEVSALSGKGLPDLLEAIALQAEILELKANPDRAAEGAVIEAQLDVGRGPVATVLVQKGTLKQGDIFVVGEQWGKVRAMENDQGQRVKEAGPSVPVEVLGLNGTPEAGDVLNVVSTEAQAREIAEYRQQAAKDKRAAAGAATTLEALMQKAKEDENVAELPVLVKADVQGSAEAIVQALEKVGNDEVRVRVLHYGVGAITETDVGLAEASNAPILGFNVRANASARNSANQKGVELRYYSVIYDLVDDVKKAASGLLSAEVRENFIGYAKILETFRVSGVGTVAGCLVTEGVARRSAGVRLLRDDVVIHEGTLKTLKRFKDEVAEVISGQECGMAFENYDDVRKDDVIEIFTRQEIERTLEMAKVKKQPRPKAETPKGFRDYFGAEVTSRSEMLAKIAGVYHRYGFEALESSAVETVEALGKFLPDVDRPNEGVFAWQEADEGGN-GDWLALRYDLTAPLARVYAQHRNDLPTPYRRFAMGPVWRNEKPGPGRFRQFYQCDADTVGAPSVAADAEICALLADVLEEVGLG-GQYLIRVNNRKVLNGVLEAMGLE-DD--AQKDAVLRTIDKFDKVGESGVRELLGKGRLDASGAYIDGVGLSEAQAEPVIAFLTSKGETTEATLANLGAAVGASEIGAGGVAELREIADLLAAQGYEADRIVIDPSVVRGLGYYTGPVFEAELTFEITDEKGRPRQFGSVAGGGRYDDLVKRFTGQAVPATGVSIGVDRLLAALAASGKAEARAEGPVVVTVMEKARMAEYQAMVAELRQAGIRAEVYLGNPKNFGNQLKYADRRGSPAAVIAGSDEFEQGKIQIKDLILGARIAENATLEEWKERPSQFECPRSELVARVQDVLAGRGMDDLRTTYLGRIAEAEDEATLEALRVEAVGKKGEVSLKMRELGKMSAEERQVMGPKLNALKDEINSALAAKKEALADSALDERLRAEWLDVTLPGRPRRTGTIHPVSQVTEEVTAIFADMGFAVAEGPQIDTDWYNFDALNIPSHHPARAEMDTFYMHRAEGDDRPPHVLRTHTSPVQIRHMEKHGAPCRIIAPGRVYRADYDQTHTPMFHQVEGLALDRDISMANLKWVLEEFFAAFFEVDGIKTRFRASHFPFTEPSAEVDIQCSWEGGQLKIGEGDGWLEVLGSGMVHPHVLRSGGVDPDEWQGFAFGMGIDRIAMLKYGIPDLRAFFDSDLRWLRHYGFRSLDVPTLHGGLS-LELAEQAGLDLVEISPNAKPPVCKIMDFGKYKYEQQKREAEARKKQKTIEVKEVKFRPNTDIHDYDVKMKNVYKFLENGDKVKVTLRFRGREMAHQNLGRELLERVADDVKELGKVENMPKMEGRQMVMMIGPTTKMKFTLSWLKDHLDTTASVDEIAETLTDLGLEVEGVENPAERLGAFTLGKVVAAEKHPDADKLKVCRVETDEGEQQIICGAPNAREGITVVVAKPGTYVPGIDTTIQVGKIRGIESFGMMCSMREMELSDEHDGIIELPSGDVGQRFTDWLAEHDPAKVDPVIEIAITPNRPDALGVRGIARDLAARGLGTMKARDCDPVKGAFESPLKVTIDDDARDGCPVFYGRLIRGVKNGPSPEWLQTALRAIGLRPISFLVDVTNFFTFDRNRPLHVFDAGRVKGNLRVHLAQGGETLHALDERTYTLEPGMIAISDDAGVQSLGGIMGGEATGVTEETTDVFLEAAFFDPIRTAHTGRALKINSDARYRFERGIDPEWTPHGIEHATRMILDIAGGEASEVVVAGKIPDHSRAYHLDTERCSSLVGMDIPAETQRATLEALGFEMEGDMAHVPSWRPDVMGSADLVEEVARIASLTKLEGKPMPRPSPGVPKPILSPQQKREQAARRTMAALGYNECVTYSFIDQKAAELFGGGGDATMLANPISSEMSHMRPALLPGLLQAAARNQARGMSDLALFELGHAFQGGEPGEQHQLLTGLIVGRTGPKDVHGAARPVDLYDVKADAEAVLAAIGAPAKVQILRGASEWWHPGRHGMICLGPKKVLGVYGEIHPKVLKALDVKGPAVGFTIWPAKVPLPKQARTTRPALDLNDLQPVERDFAFVVDESVAALDLVNAAAGADKALIEDVRVFDEFVGGSLGEGKKSLAITVRLQPRDATLKEKDIEAVSAKIVEKVQKATGATLRGMHAYRSQTCADLRRDNVGQEVRLAGWVHRIRDHGGVLFVDLRDHYGVTQVLCDPDSPVFAEVEKLRSEWCVKIDGVVKARAESLVNPKLPTGEIEVYIRDVEVLGAAEELPLIVFGDQEYPEETRLRYRYLDLRREAMQENMVLRSDVVRSLRERMWKQNFREYQTPIITASSPEGARDFLVPSRLHPGKFYALPQAPQQFKQLIMVSGFDKYFQIAPCFRDEDPRADRSPTDFYQLDMEMSFVEQQDVFDAIQPAVQGVFEEFGGGRKVDTDWPQIAYKDSMLWYGTDKPDLRNPIKMQVVSDHFRGSGFAIFAKLLEQDGTEIRAIPAPGGGSRKFCDRMNAFAQKEGLPGMGYIFWRDK---DGEAEAAGPLAKNIGPERTEALRQELGLGVGDAAFFLGGKPASFERVAAKARTVIGEELGHTEQDRFAFAWIVDFPMYEADEESGDIDFSHNPFSMPQGGIEALDGDPLEVKGWQYDLACNGYELVSGAIRNHRLDIMFKAFEKAGYGEDEVRKRFGGMVNAFRFGAPPHGGCAAGIDRIVMLLADEQNIREVIMFPMNQRAEDLMMGAPTEPTSDQLMELRLRVMPDEMTSRALIRNFSIVAHIDHGKSTLADRLIQLTGTVAERDMQDQLLDQMDIERERGITIKANTVRIEYPAKDGNTYVLNIIDTPGHVDFSYEVARSMQACEGSLLVVDATQGVEAQTLANVYTAIDADHEIVPVLNKVDLPAADPERVRAQIEDVIGIDASEACLISAKTGIGIPDVLEAIVTKLPAPEGDAEKPLKAMLVDSKYDQYLGVICIVRIIDGVLRKGDRIRMMKTGGTYDVDDVGVYRPAMTAVKELGPGEIGYLNASIKQVRDTRVGDTITLEKRPCETPLPGFKPSVPVVFCGLFPVDANDFEDMRGAIEKLALNDASFTYEMETSAALGFGFRCGFLGLLHLEVIRDRLEREYGIELITTAPSVIYHLYTKDGERRDLHNPADMPDPSTVAHVEEPRIKATILVPDEYLGDVLKLCQERRGIQTDLTYVGGRAMAVYDLPLNEVVFDFYDRLKSVTKGYASFDYEMIGYREDNLVKMQILVNDEPVDALAMMVHRDRAEQRGRAMCEKLKDLIPRHMFKIPIQAAIGAKVIARETLSAMRKDVTAKCYGGDATRKKKLLEKQKAGKKKMRQFGKVEIPQEAFISALKMDSMSLPPGFLDELRNRTSLSQVVGRKVMWDQRKSQQGKGDMWAPCPFHQEKSASFHVDDRKGFYYCFGCHAKGDAISFIRETENVGFMEAVEILAGEAGMQMPAKDPQAKEKADRRTQLAEVMEAAVRFYRMQLGTGAAQEARAYLDRRKLGPAARDTFEIGFAPPGWQSLFDHLTGQGIAPELIMAAGLARESQKGGKPYDVFRHRIMFPIRDGRGRAIAFGGRAMDPDDKAKYLNSPETELFDKGRNLYNLAPAREASGKGAPLIVAEGYMDVIALSEGGFPAAVAPLGTAVTEAQLQTLWRVSDEPVIALDGDAAGIRAAYRVIDLALPLLEAGRSLRFAIMPPGKDPDDIIRGEGPEAIRRILDEAVPTVSLLWRRETEGRSFDTPERKAALDKSLRETVKRIPDETLRYHYGEEIKHLRWELFRP--AGQGGQRKRGAW-----WKNAPAVPSAETKNSLLVT-GQDRADQLIREVVILAALISTPEVLPDFEGQVEEMPCLDPTHAAMRDALLRIRPANGAEAREAVAAAIGAEALETALSAPHLAIVPCLRRPGDAEFAALTIGEELAKVTARFGRHAAETEAVEDLEAHGDEVLTWRLRQASEEFARAQRAVREDDIEYDLGDNGARIDRGERNAFAALMDTIRFDKGRK--MCADTPDYKDTLNLPQTDFPMRAGLPKREPGWLERWEEIGIYDRLRETAGRTPFTLHDGPPYANGHLHIGHALNKTIKDIIVRSHQMMGHDARYIPGWDCHGLPIEWKIEEQYRAKGQDKDQVNVVDFRQECRKFAESWIDVQREEFKRLGVTGCWDRPYLTMDFRAERIIAEEFQKFLMTGTLYQGSKPVMWSPVEKTALAEAEVEYHDKESFTIWVKFPVTEFAEARDAFVGTKVVIWTTTPWTIPSNKAVVFGSDFSYGLYEVTDTPGECWARAGERFILADKLAAQTFAKARLEDGMWRRVRDVTPEQLAGMELAHPLAGAEGGQGEWDDPRDFRAADFVTDEEGTGFVHCAPSHGMEEYELYRGLGMLAQVITYNVMDDGSFREDLPFFGGKRILKENGKEGDANKAVIDTLVANGGLLARGKIKHSYPHSWRSKAPVIYRNTAQWFAAIDRPVGDGQDENGKTIRERALTCIDQVKWTPQSGRNRLHSMMESRPDWVLSRQRAWGVPLTCFTKKGGKPTDPNFLLRDPAVNARILAAFEEEGADAWYKDGAKERFLGNDYDASEWDQVFDILDVWFDSGSTHAFVLRDREDGTEDGIADVYMEGTDQHRGWFHSSMLQACGTMGRAPYRNVVTHGFTLDEKGNKMSKSLGNTVGPEDVIKQYGADILRLWVAQADYTSDQRIGPEILKGTADSYRRLRNTMRFMLGALSHFREEDRVAHEDMPELERWVLHRVAELDERVRTGYRAFDFQGTFRALFDFATVDLSAFYFDIRKDALYCDGDTTRRRAARTVMDILFHRLTTWLAPILVFTTEEVWLERFPGERSSVHLQDMPETPQAWRDDALAAKWAEVRRARRVVTAALEVKRTEKVIGASLEAAPIVHVDSEDTLGALRSVAFEDVCITSDIALTNDPAPAEAYRLPEVDGIAVVFETADGAKCQRCWKILPDVGTHAHPDVCGRCDDALS-MQSQNIRIRLKAFDYRVLDSSTQEIVNTAKRTGADVRGPIPLPNKIERFTVLRGPHVNKKSRDQWEIRTHKRLLDIIDPTPQTVDALMKLDLAAGVDVEIKV--MADQINSLEELGEAA-----GT-AAVVEPEINREPKRDELGRSYATGRRKDATARVWIKPGSGKVTVNGKPLNEYFARPVLQMILNQAFTVAGVEGEFDVMATVKGGGLSGQAGAVKHGISQALQLYNPSLRPALKAAGFLTRDSRTVERKKYGRAKARRSFQFSKRMPTIQQLIRKPRQPKPKRSKAIHLEQCPQKRGVCTRVYTTTPKKPNSAMRKVAKVRLTNGYEVISYIPGESHNLQEHSVVLIRGGRVKDLPGVRYHILRGVLDTQGVKDRRQRRSKYGAKRPKMARDPRRGKRKVSKNIAAGVAHVNSSFNNTKILISDVQGNAISWSSAGTMGFKGSRKSTPYAAQLAAEDAGKKAQEHGMKTLEVEVQGPGSGRESALRALAAVGFNITAIRDVTPIAHNGCRPPKRRRV-MDDRRGLLIILSSPSGAGKSTLARRLRDWDPRIVFSVSATTRPPRPGEVDGKDYHFLTDSAFKHQVSDGGMLEHAHVFGNFYGSPKGPVREAIEGGHDVLFDIDWQGAQQIRNSELGLHTLSIFLLPPSIGELKRRLEARGQDGADVIEKRMQKSWDEISHWDGYDFVLVNADLDETEARLKSIVTAARLRRSQQPGLTAHVRKLQAEFEEGLMAQSYLGQKRLRRYYGKIREVLEMPNLIEVQKSSYDLFLDSGDQPTPQDGEGIKGVFQSVFPIKDFNETAVLEFVSYELEKPKYDVEECMQRDMTYSAPLKVTLRLIVFDIDEDTGAKSVKDIKEQDVFMGDMPLMTQNGTFIVNGTERVIVSQMHRSPGVFFDHDKGKTHSSGKLLFACRIIPYRGSWLDFEFDAKDLVYARIDRRRKLPVTTLLYALGLDQEAIMDAYYDTVDYTYEKGRGWKTKFFPERVRGTRPTHDLIDADSGEVIAEAGKKVTPRAVKQLIDEGKVESLLVPFDNIVGKYVAKDIINEETGAIYVEAGDELTWTVDKDGEVSGGTLKELMDAGVTEIPVLDIDNINVGPYMRNTMAQDKNMGRDTALMDIYRVMRPGEPPTVEAASALFDTLFFDSERYDLSAVGRVKMNMRLALDKPDTQRTLDRSDIVSCIKALVELRDGKGEIDDIDHLGNRRVRSVGELMENQYRVGLLRMERAIKERMSSVEIDTVMPQDLINAKPAAAAVREFFGSSQLSQFMDQTNPLSEVTHKRRLSALGPGGLTRERAGFEVRDVHPTHYGRMCPIETPEGPNIGLINSLATFARVNKYGFIETPYRIVKDGQVTDEVHYMSATEEMRHTVAQANASLDENGRFVNDLVSTRQSGDYTLAPNETVDLIDVSPKQLVSVGASLIPFLENDDANRALMGANMQRQAVPTLRSEAPLVGTGIEGVVARDSGASIMARRGGVIDQVDAQRIVVRATADLDPGDPGVDIYRLRKFQRSNQNTCINQRPLVKVGDTVEKGEVLADGPSTDMGELALGKNIIAAFMPWNGYNYEDSILISERIAQDDVFTSVHIEEFEVAARDTKLGPEEITRDIPNVGEEALRNLDEAGIVYIGADVEPGDILVGKITPKGESPMTPEEKLLRAIFGEKASDVRDTSLRVKPGDYGTVVEVRVFNRHGVEKDERALQIEREEIERLARDRDDELAILDRNIYARLKGMILGKTAVKGPKGVKANSEINEELLGTLTRGQWWQLALSDEEDAKNVEALNEQYEAQKRQLDARFEDKVEKVRRGDDLPPGVMKMVKVFVAVKRKLQPGDKMAGRHGNKGVISRVVPIEDMPFLEDGTPVDFCLNPLGVPSRMNVGQILETHMGWAARGLGIQVDEALKDYKRTGDLTPVREAMKIAYGDEVYSEGIEGMEEDRLVEAASNITGGVPIATPVFDGAKEPDVNDALRRAGFDESGQSRLFDGRTGEEFSRRVTVGVKYLLKLHHLVDDKIHARSTGPYSLVTQQPLGGKAQFGGQRFGEMEVWALEAYGAAYTLQEMLTVKSDDVAGRTKVYESIVKGEDNFEAGVPESFNVLVKEVRGLGLNMELLDAEEDEMPKYEHVFISRQDLSNTQAEGLIEHFSSILSDNGGTLVESEYWGVKTMAYKINKNRKGHYAFLRTDAPASAVQEMERLMRLHDDVMRVLTIKVDEHEEGPSIQMQKKDERDDRRERRAQGEIAVEALDEAGAREELERLARILLEANDAYHGRDDPKLTDADYDRLKRRNAEIEARFPGLKREDSPSERVGAEPALGFRKISHTVPLLSLENLFSESEVFDFDKRIKKYLNIQANKNIEYVADPKIDGLSVSVRYEYGKLKHAATRGDRRVGEDITENAKTIAEIPQSIPSKLSVLEVRGEVYMRKTDFAALNERGVEERQASFANPRNAAAGSLRQINPAITAKRPLKFFAYALGEVHGLTVETQSELLIWLKSLGFVTNELFRKCCGPDDLISYYKELEEQRSTLDYDIDGIVYKVDDLALQRRLGFRSTTPRWAIAHKFPAELAWTRLEAIEIQVGRTGALSPVARLTPVTVGGVVVSNATLHNEDYIAGRDSRGEPIRGGRDIREGDWVQVYRAGDVIPKIADVDLSKRPEGSEPYAFPEVCPECGSDAIREEGDSVRRCSGGMSCPAQAVEKLKHFVSRAAFDIEGLGSKQVEQFYRDGWIREPVDIFELRERYGQGGRQLKNREGWGEKSAQNLFAAIDERRRIPLGRLIFALGIRHVGEVNANLLARHYGTWAAFDAAMREAAPMEGAAWDELNGIDGIGQVLAQSLVATVNQDAERASIERLLAHLEVEEAARPQTDGSPVAGKTVVFTGSLERMTRAEAKARAEALGAKVSGSVSAKTDIVVAGPGAGSKEKKARELGLEIMDEDAWLALVEG-MIHKNWAELIKPTQLEIKPGNDPTRLATCVAEPLERGFGLTLGNALRRVLLSSLQGAAITSVQIDNVLHEFSSVAGVREDVTDIVLNLKGVSLRMEVEGPKRLSISAKGPGVVTAGDISESNGIEVLNKDHVICHLDEGADVFMELTVNTGKGYVSADKNKPEDAPIGMIPIDAIYSPVKKVAYDVQPTREGQVLDYDKLTLKVETNGAVTPEDAVAYAARILQDQLSIFVNFDEPESARSDDGDDGLEFNPLLLKKVDELELSVRSANCLKNDNIVYIGDLIQKTEAEMLRTPNFGRKSLNEIKEVLSGMGLHLGMDVEDWPPDNIEDLSKKMEDTFMAERDNRRGGRRD-RDEAPEFADRLVAINRVSKTVKGGKRFGFAALVVVGDQKGRVGFGKGKAKEVPEAIRKATEQAKRKMIRVPLREGRTLHHDIEGRHGAGRVVMRTASQGTGIIAGGPMRAVFEMLGVHDVVAKSNGSQNPYNMIRATLEGLTRQQSPRHVAQRRGKKVSDILPKREEQAEDHVRVSEEAMNDPIGDMITRIRNGAMRGKSTVRTPGSKLRAWVLDVLADEGYIRGYEKTTDERGHPQFEISLKYYEGEPVIRELKRVSRPGRRVYLGVDTIPQVRQGLGISIVSTSKGVMSDHTARAQNVGGEVLCTVFMHDIRAIRENPDAFDAALKRLGVEAASPSLLKIDEARRAAIHEAETAQAEQNAASKEIGKAKASGDEAEFERLRALVSEKKAAVAEMQARARDLDAELTDALMSLPNLPFEDVPEGADEEDNVEIRRWGEPRQFSFTPKEHYDLPCVQDGMDFETAAKLAGSRFVVMSGAVARLHRALAQFMLDTHVTENGLTETWTPVLVRDEMMYGTGQLPKFGEDSYRTREGWWLVPTAEVTLTNIVNGLTVDESYLPRRYVAHTQCFRSEAGSAGRDTAGMLRQHQFEKVEMVSVTHPDASLDEHARMTGCAEGILQRLGLPYRTVVLCTGDMGFGARRTHDIEVWLPGQQSYREISSISVCGDFQARRMNARMKPADGGKPVFLHTLNGSGLAVGRCLIAVLENGQEEDGSVTLPEALHPWLGGKTRITAQGELSMAITAAQVKELRETTGAGMMDAKKALTETDGDMDAAIDWLRTKGLAKAAKKAGRTAAEGLVAVKVKGGTGVAVEVNAETDFVAKNAEFQSMVGDIAEKATEVDDVEALKAADMGGKTVNDRLTDAIAKIGENMSLRRMAKLEGETVATYVHNAAANGMGQIGVLVALKGGDEAFGRQVAMHIAATQPAALSEAELDPATVEKERQLQMDIARESGKPEQVIEKMIEGRMKKYLAEITLLGQSFVVDPDKTVAQAAADAGAEIVGYVRLAVGEGIEKEKEDFAAEVAKAAQGMNQEITNNPFNPLTPPKVFDEIKVSLASPERILSWSFGEIKKPETINYRTFKPERDGLFCARIFGPIKDYECLCGKYKRMKYRGVVCEKCGVEVTLQKVRRERMGHIELAAPCAHIWFLKSLPSRIGLMLDMTLRDLERVLYFENYVVIEPGLTDLQYGQMMTEEEFMDAQDQYGMDAFTANIGAEAIREMLAGIDLESEAERLREELKEATGELKPKKIIKRLKVVENFLESGNRPEWMIMTVIPVIPPELRPLVPLDGGRFATSDLNDLYRRVINRNNRLKRLIELRAPDIIVRNEKRMLQESVDALFDNGRRGRVITGANKRPLKSLSDMLKGKQGRFRQNLLGKRVDFSGRSVIVTGPELKLHQCGLPKKMALELFKPFIYSRLEAKGLSSTVKQAKKLVEKERPEVWDILDEVIREHPVLLNRAPTLHRLGIQAFEPVLIEGKAIQLHPLVCSAFNADFDGDQMAVHVPLSLEAQLEARVLMMSTNNVLSPANGAPIIVPSQDMILGLYYLTIAREGMKGEGMVFGSVEEVEHALNAGEVHMHAKIQCRVKQIDDEGQEIVKRFETTPGRVRLGALLPMNAKAPFDLVNTLLRKKDVQRVIDTVYRYCGQKESVIFCDQIMSMGFKEAFKAGISFGKDDMVIPDSKWPIVEETRTHVKDFEQQYMDGLITQGEKYNKVVDAWSKCNDKVTEAMMTTISTSKTDENGAEAEPNSVYMMAHSGARGSVTQMKQLGGMRGLMAKPNGEIIETPIISNFKEGLTVLEYFNSTHGARKGLSDTALKTANSGYLTRRLVDVAQDCIIRMQDCGTDRSITAEAAINDGEVVASLGERVLGRVAAEDILKPGTDEVLVKHGTLIDERMSDAIEEAAVQTARIRSPLTCEAEEGVCANCYGRDLARGTMVNTGEAVGIIAAQSIGEPGTQLTMRTFHIGGVAQGAQQSFLEASQSGVIEYENPNTLVDSTGATLVMGRNMKVLITDGEGNERASHKVGYGTKLFVKEGQEIARGDKLFEWDPYTLPIIAEKDGTARHVDLVNGVAVREETDDATGMTQKIVTDWRAAPKGNELKPEIILQDENGEPVRNDQGNPITYPMSVDAVLSVEDGQKIQAGDVVARIPREGAKTKDITGGLPRVAELFEARRPKDHAIIAEIDGYVRFGKDYKNKRRIAIDPVDESLEPKEYMIPKGKHIPVAEGDYVQKGDYIMDGNPAPHDILSIMGVEALANYMIDEVQDVYRLQGVKINDKHVEVIVRQMLQKWEILDSGDTTLLKGEHVDKMEFDEANAKIEKKGGRAAQGEPILLGITKASLQTRSFISAASFQETTRVLTEASVQGKVDKLVGLKENVIVGRLIPAGTGGATQKVRKIATDRDNVVIEARRVEAEEAARLAAPEESVSDVSGGDEFDDLIVTPESREMSRRHSAEKREILPDAKYGDRVLTKFMNNLMVDGKKSVAEGIVYNALDRVENKVKRSPIEIFSEALDNIKPSVEVRSRRVGGATYQVPVEVRPERREALAIRWLIEASRKRNENTMEERLAGELLDAVNSRGSAVKKREDTHKMADANKAFSHYRW---MHNND-EAMPILHAPAPEVAQREKLEGGKRFVMHTEYSPAGDQPTAIKELSEQVMSGERNQVLLGATGTGKTFTMGKVIEATQRPAIILAPNKTLAAQLYGEFRQFFPENAVEYFVSYYDYYQPEAYVPRSDTYIEKESQINEQIDRMRHSATRALLERDDVVIVASVSCIYGIGSVETYGAMTQDLFVGKEYDQRAVMADLVAQQYKRNDQGFQRGSFRVRGDVLEVWPAHLEDRAWRFSFFGEELEAITEFDPLTGQKSTSFDRIRIYANSHYVTPRPTLKQAIEKIKTELRQRLDILVGEGKLLEAQRLEQRTNFDLEMLEATGTCNGIENYSRYLTGRAPGEPPPTLFEFIPDEAIVFADESHVSVPQIGAMYKGDYRRKFTLAEHGFRLPSCMDNRPLKFEEWDAMRPQSIFVSATPANWELDQSGGVFTEQVIRPTGLLDPQVEIRPVETQVDDLLDEIRKVAADGYRVLCTVLTKRMAEDLTEYLHEQGIRVRYMHSDIDTIERIEILRDLRLGAFDVLVGINLLREGLDIPECGLVAILDADKEGFLRSETSLIQTIGRAARNAEGRVIMYADRVTGSMERAIGETNRRREKQIAYNEEHGITPETIRKNVEDVLAGVYQGDTDMNRVTAKIEPAHQGSNMQAVLEGLRTDMRKAAENLEFEEAARLRDEVKRLEAVDLAVADDPLARQQAVEKASDEATKRSGRSTAGRPGQRGGVQRRKK-MAAKPFFRRRKVCPFSGENAPKIDYKDTKLLQRYVSERGKIVPSRITAVSSKKQRELSRAIKRARFLALLPYAVKMPKRILQGTVTSTANAQTVTVSVERRFKHPVLQKTIKKSKKYRAHDEGEAATVGQSVMIRECAPKSKTKRWEVMTPEMMANTPQSKKRARQNEKRYAVNKARRSRIRTHLRKVEEAIASGDQAAAQAALRDAQPELMRGVTKGVFHKNTAARKMSRLSSRVKSLAMTRSVWKGPFVDAYVLKKAEAARESGRNEVIKIWSRRSTILPQFVGLTFGVYNGHKHVPVNVSEDMIGQKFGEYSPTRTYYGHSADKKAKRKMARIAGVNIPTAKRVPVALTYIHGIGPSAAKDICAAVNIDESRRVNELSDAEVVAMREHIDANFTVEGDLRRERQVNIKRLMDLGCYRGLRHRRNLPVRGQRTHTNARTRKGPAKAIAGKKKMAVKIRLSRGGSKKRPHYSIVAADARMPRDGRFLEKLGTYNPLLPKGDERRVIMDVDRIKVWIEHGAVPTERIQRFLESAGAAEKKERNNPNKGTPGKAAQERAAAKAEKAAA--------AEETTSE-MSITQEDKQKVMKDFATKDGDTGSPEVQVAILTTRITALTEHFKTHHKDNHSRRGLLKLVAQRRKLLDYLKGNDEERYRTLIGKLGIRRMPSLNDIRSTFLGYFEKQGHTVVPSSPLVPRNDPTLMFANSGMVQFKNLFTGVEHRDYTRATTAQKCVRAGGKHNDLDNVGYTRRHHTFFEMLGNFSFGDYFKSDAIPFAWELVTKEFDIDPSRLLVTVYHTDDEAADLWKKVAGLPDDRILRIPTSDNFWQMGSTGPCGPCSEIFFDHGDKYWGGPPGSPEEDGDRFVEIWNIVFMQNELFEDGSMRELDAQSIDTGMGIERVAALLQGTNDNYATDLMRALIEASADVTGSDPDGPGETHHRVIADHLRSTSFLIADGVMPSNDGRGYVLRRIMRRAMRHAHLLGTKEPIMHRLVPALVQKMGAAYPELGRAQALIEETLKLEETKFKTTLDRGLKLLDEELGGLPEGAPLPGASAFKLYDTYGFPLDLTQDALRERERTVDTEGFDTAMAEQKAKARAAWSGSGEAADAAIWYDIADEHGATDFLGYDTETAEGQVKAVVKGGESVKTAAKGDEIQIVLNQTPFYAEAGGQVGDTGEIITDAGKARVTDTKKVAGVFIHFAQVTEGEIAPGAYAKLEVDHARRTSIRANHSATHLLHEALRRALGDHVAQRGSLNAPDRLRFDFSHAKALTDAELKSVEAEVNSYIRQNTPVSTRIMSPDDACAIGAQALFGEKYGDEVRVVSMGTQDGSGKGAYGATYSIELCGGTHVTQTGDIGVFVVTADSASSAGVRRIEALTGSEAWDYLAAQDRTVGDLASELKTSRSDVGARVKALMEERRALQNEVAQLRRELAMSGGTG-GAAEARDIGGVPFLAQIVSGVTGKDLPPLIDEHKARLGSGAVLLIADAGGKAAVAAGVTADLTDKVSAVDLVKAAVTELGGKGGGGRPDMAQGGGPSADNAENAIKAAEAVIGGMGFRMGIVGLPNVGKSTLFNALTRTASAQAANFPFCTIEPNVGEVAVPDKRLATLADIAKSKQVIPTRLTFVDIAGLVKGASKGEGLGNQFLANIREVDAIAHVLRCFEDGDVVHVDGRVDPVADAETIETELMIADMESIEKRLQNIVRKVRGGDKEAVQQERLLKMAMAALEEGKPVRTVEVAEDDRKAWRMLQLLTQKPVLYVCNVSEDEAATGNAQSEKVAAMAAEQGNAHVVISAKIEEEISQLDEEEAEMFLEELGLAEAGLDRMIRAGYELLHLETYFTVGPKEARAWTIPIGTLAPAAAGVIHGDFEKGFIRAETISYDDFVELGGEQAAKDAGKMRVEGKAYRVKDGDVLHFLFNTMAWKTLDDMDLAGKRVLTRVDINVPVEDGRVTDATRIERVVPTIKDILSKGGKPILLAHFGRPKGERVPEMSLQPLIPALEEAFGTRVVFAADCRGAQSAIEAMGDGEVVLLENTRFHPGEEKNDDALASEMAELGDIYCNDAFSAAHRAHASTEALARKLPSCAGRLMEAELSALEKALGEPKRPAVAVVGGAKVSTKLDLLSNLVTRVDTLVIGGGMANTFLAAQGLDVGKSLCEHDMTGTASEIAEKAAEAGCEILLPRDVVVAHRFEAGAPHQIVKT---EDVPSDAMILDTGPDSVAHIKEVLDRSETLIWNGPLGAFEIKPFDEATNAAAGYAAELTKAGKLISVAGGGDTVAALNKADASDHFTYVSTAGGAFLEWMEGKDLPGVAALG-MALPEFNMRQLLEAGVHFGHQTQRWNPRMGEFIYGARNGIHIVDLTQTVPMLDAALNAIRETVAKGGRVLFVGTKRQAQQPVAEAAEKCAQYYMNHRWLGGTLTNWQTVSKSIQRLKSIDEQMETGAEGLTKKERLGMEREQAKLQASLGGIREMGGLPDLLFVIDVKKEQLAIAEAKKLGIPVAAVVDTNCSPDGVDYIIPGNDDAARAIGLYTDLASRAALDGMTAQLEGAGVDLGALEDA--------ED--ATEAAPAEAPAEQA--MTKRTSAKYKIDRRMGENIWGRPKSPVNRREYGPGQHGQRRKGKMSDFGLQLRAKQKLKGYYGDITEKQFRRIYAEAERRRGDTGEILIGLLERRLDAVVYRAKFVATVFAARQFVNHGHVLVNGKRVNIPSYRVKEGDVIEVREKSKQLASVLEAAQLPERDVPDYLEVDHQKMSCTFVRQPGLGDVPYPVQMEPNLVIEFYAQNMGNKVNPIGMRLQVNRTWDSRWYADTKDYGNLLLEDLKIREFIHKECKQAGIARVIIERPHKKCRVTIHAARPGVIIGKKGADIETLRKKVGSMTASELHLNIVEVRKPELDATLVGENIAQQLERRVSFRRAMKRAVQNAMRMGAQGIRVNLAGRLGGAEIARTEWYREGRVPLHTLRADIDHASIEAATPYGIIGIKVWIFKGEIMEHDPQARDRKSQEIQDGPAPRGAGGRRMGRARKGRDISGWLVVDKPAGMTSTAVVNKVRWALAAKKAGHAGTLDPEATGVLAIAIGEATKTVPYVTDALKCYRFTVRLGSATNTDDAEGEVIATSEARPSDDEIKEALNGFVGEIMQVPPQFSAVKINGERAYKRARDGETMEIAARPLWVEELTMVSRPDADHVTLEMVCGKGGYVRSIARDLGETLGCLGHVRELRRTWSGPFEAEDGVTIEDVDALAKSPELDEKLLPVEAGLTDLPEAKCTDEGATRLRNGNPGMVIAPGLEYGEECWASHEGRAVAVGRFKAGEVWPDRVFR---TFRLGLTGSIGMGKSTTAAMFAERGCAVWDADAAVHRLYSKGGAAVEPIAAAFPDAIEDGAVSRARLRRIISADPDALSRIEAIVHPLVRTDRAQFAEAHPDGIVVFDIPLLFETGAEAEMDATACVSVSRETQEARVLDRGTMTRAEFEAILAKQMPNEEKLARADYRIDTSSLEAAARDVQDVLRKITGDP----MITDTIIDDERWHALDLPSVAETGAQAALVHLGLRPEDYEIAVLGCDDARIADLNAEFRDKPVPTNVLSWPAENLAAAEDGDAPLPPEADVFGSAELGDIAIAYETCLREAEAGQIGLPDHVTHLIVHGTLHLLGYDHVRDGDAALMERLETDILCKLGIADPCAEKAGMARFIFITGGVVSSLGKGLASAALGALLQARGFSVRLRKLDPYLNVDPGTMSPFEHGEVFVTDDGAETDLDLGHYERFTGVPARKTDSISSGRIYTNVLEKERRGDYLGKTIQVIPHVTNEIKDFISIGEDEVDFMLCEIGGTVGDIEGLPFFEAIRQFSQDKPRGQCIFMHLTLLPWIKASGELKTKPTQHSVKELRSIGIAPDVLVCRSEMPIPQKEREKLALFCNVRPDSVIAAQDLPSIYDAPLAYHREGLDQAVLDAFQITPAPKPNLTRWEDVSDRIHNPEGEVNVAIVGKYTQLEDAYKSIAEALTHGGLSNRVKVNIEWVDAEVFDREDPAPHLQGYHAILVPGGFGERGTEGKIKAAQFAREHKVPYLGICLGMQMAVIEAARNVAGVARAGSEEFDHEAGKKRFEPVVYHLKEWVQGNAKVNRRPDDDKGGTMRLGAYDAVLTEGSRVAEVYGSTAIDERHRHRYEVDITYKQALEKAGLTFSGMSPDGKLPEIVEWSDHPWFIGVQFHPELKSKPFDPHPLFRDFVRAAKEVSRLV--MPRTILGLESSCDDTAAAILR-----GREVLSSVVAGQGGLHAAYGGVVPEIAARAHAETLDGVVAEALREADLTLGEVDAIAVTAGPGLIGGVLAGVMMAKGLAAGSGKPLIGVNHLAGHALTPRLTDDLAYPYLMLLVSGGHCQFLIVRGPEDFTRLGGTIDDAPGEAFDKTARLLGLAQPGGPSVETEARHGDASRFAFPRPLLDRPGCDLSFSGLKTAILRQRDALVAAQRGLTRQDRADLCAGFQAAVRDVLSEKTRRALALYRAEHPAQPALAVAGGVAANGSLRAALRDVCADAGVVFTAPPLKLCTDNAAMIAYAGGELMEAGRASDMTLAARPRWPLDTAAAPLVGHGKKGAKAMAKNRQSGAEGPSQRQLRVGELIRRTLSDVLMRGDVHDPELSGRSITVGEVRVSPDLKVATAYVLPLGGHGQDDLIGALARNKSELRRQIAKKLTLKFAPDLRFRVDETFDRMDETRRIFEQDNVRRDLD-D

>'Su-aestuariiTSTF-M16'
[truncated: 667,249 more chars]
