# Supplementary material for: Genome-based taxonomic classification of the genus Sulfitobacter along with the proposal of a new genus Parasulfitobacter gen. nov. and exploring the gene clusters associated with sulfur oxidation
Source: BMC Genomics. 2024 Apr 22;25:389. doi: 10.1186/s12864-024-10269-3 (PMC11034169; doi:10.1186/s12864-024-10269-3)
Supplement: Supplementary file 4 — Supplementary Material 4 [file 12864_2024_10269_MOESM4_ESM.docx]

def chuli_seq(in_file_0):

import os

path = os.getcwd()

path = path + "/" + in_file_0 + "/"

files = os.listdir(path)

file_path = []

for file in files:

file_path_1 = path + file

file_path.append(file_path_1)

for file in file_path:

cmd = "sed -i 's/ /_/g' " + file

os.system(cmd)

return print("1. sequence preprocessing")

def protein_ortho(file):

import os

cmd = "proteinortho6.pl -project=" + file + "_work -e=1e-5 -cov=50 -identity=50 -clean " + file + "/*.faa"

os.system(cmd)

return print("2. homologous alignment completed")

def guoLv_result(in_file_1, num):

in_file_1 = open(in_file_1, 'r')

out_file_1 = open("work_ortho", 'w')

for line in in_file_1:

if line.split('\t')[0] == line.split('\t')[1] == num:

out_file_1.write(line)

out_file_1.close()

return print("3. file filtering completed")

def get_whole_protein(file_name):

import os

cmd = "cd " + file_name +" ; cat ./*.faa >> whole_protein ; mv whole_protein .. ; cd .."

os.system(cmd)

return print("4. genome integration completed")

def read_to_dict(in_file_2):

seq_dict = {}

ac = ''

seq = ''

for line in open(in_file_2):

if line.startswith('>') and seq != '':

seq_dict[ac] = seq

seq = ''

if line.startswith('>'):

ac = line.strip()[1:]

else:

seq = seq + line.strip()

seq_dict[ac] = seq

return seq_dict

def get_bacteria_name(in_file3):

file = open(in_file3)

line = file.readline()

names = line.replace('.faa', '').split()[4:]

return names

def get_single_seq(seq_dict):

i = 1

for line in open("work_ortho", 'r'):

lines = line.strip().split('\t')[3:]

out_file = open('%s.faa' % (i), 'w')

for key in seq_dict:

if key in lines:

out_file.write('>' + key + '\n' + seq_dict[key] + '\n')

out_file.close()

i = i + 1

return print("5. protein sequence extraction completed")

def get_protein_num():

import glob

path_file = glob.glob(r'*.faa')

protein_num = len(path_file)

return protein_num

def get_muscle():

import os

cmd1 = "for file in ./*.faa; do muscle -in $file -out $file.fas; done"

cmd2 = "cat ./*.fas >> protein_align.fas"

os.system(cmd1)

os.system(cmd2)

return print("6. sequence alignment completed")

def get_result(names, seq_dict, protein_num):

out_file_name = str(protein_num) + ".result.fasta"

out_file = open(out_file_name, 'w')

i = 3

for name in names:

seq_list = []

seq_align = ''

for lines in open("work_ortho", 'r'):

lines = lines.strip().split('\t')[i] # ll

seq_list.append(lines)

for key in seq_dict:

if key in seq_list:

seq_align = seq_align + seq_dict[key]

out_file.write('>' + name + '\n' + seq_align + '\n')

i = i + 1

out_file.close()

return print("7. genetate target file --> *.result.fasta")

def del_file():

import os

cmd1 = "rm -rf *.faa *.fas work_ortho whole_protein"

os.system(cmd1)

return print("8. end!")

###########################################################

if __name__=='__main__':

import sys

import os

import time

try:

file_name = sys.argv[1]

num = sys.argv[2]

test = int(sys.argv[2])

except:

print("input the correct parameter order")

print("usage: python3 protein_ortho.py <file_name> <bacteria_num>")

else:

chuli_seq(file_name)#sequence pretreament

in_file = file_name + "_work.proteinortho.tsv"

protein_ortho(file_name)

while True:

if os.path.isfile(in_file):

time.sleep(20) #make sure file .proteinortho.ts complete

guoLv_result(in_file, num) # obtain work_ortho

get_whole_protein(file_name) # generate whole_protein

whole_protein_dict = read_to_dict(in_file_2='whole_protein') #ll

get_single_seq(whole_protein_dict) # --

protein_num = get_protein_num()#--

get_muscle() # --

align_protein_dict = read_to_dict(in_file_2="protein_align.fas")

bac_names = get_bacteria_name(in_file)

get_result(bac_names, align_protein_dict, protein_num)

del_file()

break
