# Supplementary material for: Genome-based taxonomic classification of the genus Sulfitobacter along with the proposal of a new genus Parasulfitobacter gen. nov. and exploring the gene clusters associated with sulfur oxidation
Source: BMC Genomics. 2024 Apr 22;25:389. doi: 10.1186/s12864-024-10269-3 (PMC11034169; doi:10.1186/s12864-024-10269-3)
Supplement: Supplementary file 5 — Supplementary Material 5 [file 12864_2024_10269_MOESM5_ESM.docx]

>Arenibacterium-halophilumCAU-1492

--------MKFQGTKDYVATEDLTIAVNAAVALERPLLVKGEPGTGKTELARQVADALGMRMIEWNIKSTTRAQQGLYEYDAVSRLRDSQLGEEKVHDVSNYIRRGKLWEAFASDERVVLLIDEIDKADIEFPNDLLQELDKMEFHVYETGETVAAVNRPVVIITSNNEKELPDAFLRRCFFHYIRFPDEATMRKIVEVHHPGIKESLLTTALTQFYELRETSGLKKKPSTSEVLDWLKLLLAEDLTAEDLKRDGANAL-PKLHGALLKNEQDVHLFERLAFMAR--------AQ-NR------------MAALD--QTQVKEGVDQDTVDAVREVG-GAYKHGWNTD-IEMEYAPKGLTPDIVRLISEKNEEPQWMTEWRLQAYERWLTKEEPTWAMVDYPEIDFQDQYYYARPKSMEVKPKSLDEVDPKLLATYEKLGIPLKEQMILAGVEGAEDAPAEGR-------KVAVDAVFDSVSVGTTFQAELKKAGVIFCSISEAIREHPELVKKYLGSVVPVSDNFYATLNSAVFSDGSFVYVPPGVRCPMELSTYFRINAENTGQFERTLIIADKGSYVSYLEGCTAPARDIAQLHAAVVEIIVEEDAEVKYSTVQNWYPGDENGKGGIYNFVTKRADCRGDRAKVMWTQVETGSAVTWKYPSCILRGDDSQGEFYSIAIANNYQQADTGTKMIHLGKNTKSRIVSKGISAGHAQNTYRGLVSMHPKAKHARNYTQCDSLLIGDKCGAHTVPYIEVKNNSARVEHEATTSKVDDDQLFYCRSRGMDEEEAVALVVNGFCKDVLQALPMEFAMEAQQLVAISLEGSVGMKAAVIVFPGSNCDRDLAVAFEAA-GADVTMVWHKDSDLPRDVDIVGVPGGFSYGDYLRCGAIAAKSPVCTALRGHVERGGYAIGICNGFQVLTETGLLPGVLLRNAGLKYICRTVGLRVETSDSAFTQGYNAGDVLNIPIAHHDGNYYADEATLAALKGEGRIAFTYT--DNPNGSQYDIAGILSDNRRVLGMMPHPERAADAGHGGTDGTALFRALTGALTAAMTDLTPREIVSELDRYIIGQKDAKRAVAVALRNRWRRKQLSDDLRDEVYPKNILMIGPTGVGKTEISRRLAKLARAPFIKVEATKFTEVGYVGRDVEQIVRDLVDSAIALTREYMREDVKARAHSAAEERVISAIAGEDAREATREMFRRKLKNGELDDTVIELDLADTSNPM-SMFDIPG-QP-GANM-GMMNLGDLFGKAMGGRTQRRKLTVADSYEILIGEEADKLLDDETVTRTALEAVEQNGIVFLDEIDKVCARSDARGGDVSREGVQRDLLPLIEGTTVSTKHGPVKTDHILFIASGAFHIAKPSDLLPELQGRLPIRVELRALTEADFVRILTETDNALTRQYTALMGTEDVTVTFTDDGIAALAKIAAEVNQSVENIGARRLYTVMERVFEELSFTAPDKGGEAITVDADFVETNVGELARSTDVSRYVL--------MPSPLLTVTTGSSGLRIEKLRKSYRKKLVIRDVSMTLQRGEVVALLGPNGSGKTTSFYAIAGLVFPEAGTVAIDGQNVTNLPMYRRARLGIGYLPQEMSIFRGLSVEDNISAVLDITHDDPHKRRERLEDLLSDFSIEHLRRAPALALSGGERRRVEIARCLAAEPKYLLLDEPFAGVDPISVNDIRHLVSDLKKRGIGVLITDHNVRETLEIVDRAYILHDGQVLMSGTPADVVENENVRRVYLGENFRIS-MSDRPATPLLDRISRPADLKHLSDAELTQVAHELRAETISAVSVTGGHLGAGLGVTELTVALHAVFDTPRDKIIWDVGHQCYPHKILTERRDRIRTLRMKDGLSGFTKRSESPYDPFGAAHSSTSISAALGFAVARDLGGNPPEGLGDTVAVIGDGAMSAGMAFEAMNNAGHLKKRMFVILNDNEMSIAPPVGALSSHLSRLYAEAPLHDLKAAAKGAVSLLPGPFREGAKRAKEMLKGMAMGGSLFEALGFSYIGPIDGHDMDALLPILRTMRARATGPVLLHVLTRKGKGYAPAERARDKGHATAKFDVVTGEQKKAPSNAPSYTRIFGDELTRLAGEDDKICAVTAAMPDGTGLNLFAERFPSRCFDVGIAEQHGVTFSAALAAGGMKPFCAMYSTFLQRGYDQVVHDVAIQRLPVRFAIDRAGLVGADGATHAGSFDVAYLANLPGFVVMAAADEAELKHMVATAAAHDEGPIAFRYPRGEGTGVELPEKGEVLEIGKGRIVREGARVAILSFGTRLAEVEQAAEALAARGISPTVADARFAKPLDRDLILRLAADHEALITIEEGAIGGFGSHVAQLLAEEGQFDTGLKFRSMVLPDTFIDHASPADMYASAGMNAADIEAKVLDVLGVARIGE--K--RAMAKLGKRTRAAREAFAGKENLSVEDAVALVKANAAAKFDETVEIAMNLGVDTRHADQMVRGVVGLPNGTGKDVRVAVFARGAKADEAQAAGADIVGAEDLMETIQGGKIDFDRCIATPDMMPIVGRLGKVLGPRNLMPNPKVGTVTMDVAEAVKAAKGGEVQFKAEKGGVVHAGVGKASFDEGKLVENVRAFIGAVTRAKPTGAKGTYLKKIVLSSSMGPGVTVDVAGAGSE-------MADLNTLGLAEARDLLRKGETTSVELTEACLKAIDAADVLNAFVHKTPEIAMERARAADARLQAG-DAPAMCGLPIGMKDLFCTEGVPSQAASRILDGFRPEYESTVSANLRDAGAVMLGKLNMDEFAMGSSNETSAYGNAINPWRRDGDDTALTPGGSSGGSAAAVAADLCLAATGTDTGGSIRQPAALTGTVGIKPTYGRCSRWGIIAFASSLDQAGPMTKSVRDAAIMLGAMCSHDPKDSTSADLAVPDFESLLSGDIRGKTIGIPREYRVEGMPAEIEKLWNDGTEMLKAAGAKVVDISLPHTKYALPAYYVIAPAEASSNLARYDGVRYGHRAKLAQGDGITEMYEKTRAEGFGDEVQRRVMVGTYVLSAGFYDAYYNRARKVRTLIKRDFEQAFAAGVDAILTPATPSAAFGLGEMTEADPVQMYLLDVFTVTVNLAGLPGITVPTGRDAQGLPLGLQLIGRPWEEADLLNTALALESAAGFVAKPARWWMDKIPMTPAGHNALEVELKQLKSVERPAIIKAIAEAREHGDLSENAEYHSAREKQSFIEGRIKELEGVLSLAEVIDPAKLTGT-IKFGARVTLVDEDTDEEKTWQIVGEHEANVEKGLLNIKSPIARALIGKDVGDSVEVRTPGGEKSYEVLDIVYSMAKKLMGSLKLQVPAGQANPSPPVGPALGQRGINIMEFCKAFNAKTADMEPGAPCPTVISYYQDKSFTMDIKTPPASYYLKKAAKVKS--------GAKTPSRETVGTVTVAQVREIAEAKMKDLNANDVEGAMQIILGSARSMGIEVK-MAE-TRKTKVLIIGSGPAGYTAGVYASRAMLEPILVQGIEPGGQLTTTTEVENWPGDTEVQGPDLMVRMEAHAKAMGCEIIGDIISDLDLSQRPFTAKGDSGTVYEADAVILATGARAKWLGLPSEDKFKGFGVSACATCDGFFYRGQEIVVIGGGNTAVEEALFLTNFASKVTLIHRRDELRAEKILIDRLMKSPKIEPLWHHVLDEVVGTDAPLGVEAVRVKHVDTGEITEIPAKGVFVAIGHAPANELVKDVLELHNGGYVSVKPGSTETSVPGVFAAGDLTDHKYRQAVTSAGMGCMAALDAERFLAEQGDAEVAPDAPVAVVG-------------MRLSRYFLPVLKENPAEAQIVSHRLMLRAGMIKQAQAGIYSWLPLGYKVLRNVEAIVHQEQQRAGHIPLLMPTLQSADLWRESGRYDAYGEEMLRITDRQGRDMLYGPTNEELITDIFRSHVSSYKDLPLTLYHIQWKFRDEIRPRFGVMRGREFLMKDGYNFDVDYEAAMRAYDRHMVSYLRTYERMGLKAIPMRADSGPIGGEDTHEFLVLADTGESEVFYDAAITDLTLGDRAVDYDDAAQLRSICEEWTTPYARTDETHDESLFDA-IPEDRRKSSRGIEVGQIFYFGTKYSDPLNAAVQTADGGKVPVHMGSHGIGVSRLLGAIIEASHDDKGIIWPEGVTPFHCGIVNLRQGDEAADAECNRIYAALVAAGLEPLYDDTNERAGGKFATMDLIGLPWRITVGPRGLKNGVVELTSRRTGESEELSPEAAVQKVAEIYAPHLAAAAIL------------MDG----SK---GFEDALTGEQKIRNFNINFGPQHPAAHGVLRLVLELDGEIVERCDPHIGLLHRGTEKLMESRTYLQNLPYFDRLDYVAPMNQEHAWCLAIEKLTGVQVPRRASLIRVLYSEIGRVLNHLLNVTTQAMDVGALTPPLWGFEEREDLMIFYERACGARLHAAYFRPGGVHQDLPAGLLDDIEAWAKRFPNR-LDDIDELLTENRIFKQRNVDIGVVSEEDIQLYGFSGVMVRGSGLAWDLRRAQPYECYDEFEFQVPVGKNGDCYDRYLCRMEEMRQSTHIILQCIEKLRAPEGQGDVLARG-KITPPSRGDMKTSMESLIHHFKLYTEGFHVPAGEVYAAVEAPKGEFGVYLVADGTNKPYRAKLRAPGYLHLQAMDHVAGGHQLADVAAIIGTMDIVFGEVDRMASD-RQNLQDAFLNHVRKTKVPVTIFLINGVKLQGVITWFDNFCVLLRRDGQ-SQLVYKHAISTIMPAQPISLYEGEDAS-MSITADEKNRLIKEYATKEGDTGSPEVQIAILSSRIATLTEHFKTHKKDNHGRRGLLKMVAQRRKLLDYTKSKDEARYTDLIQRLGLRR-------MSDALNELGAHIEAKRPDCVLAWDVTHGELNIDVTPGNIPGFIEFLKADSACRFSSLVDITAVDYPERVKRFDVVYHLLSMYQNQRIRLRASVREEDMVPSIVAIHPSANWFEREVYDMFGILFSGHPDLRRILTDYGFRGFPLRKDFPTTGYTEVRYDEAQKRVVYEPVSLVQEYRQFDFMSPWEGAEYILPGDEKKE--------GAK------------------MTTQSPLRLGVNIDHVATVRNARGGATPDPVRAAQMAEAAGADGITAHLREDRRHISDADIDGLMAALRVPLNFEMAATAEMQAIALRHRPHAVCIVPEKREERTTEGGLEVAREENRLAHFIAPLREAGCRVSIFIAADRRQIEAAHRIGAEVIELHTGAYCDAHAEGRLAERDRELAALREMSAFAHSLGLEVHAGHGLTYDTVQPIAAFPEVRELNIGHFLIGEAIFRGLTPAIAEMRRLMDDARSQRAVS----------MAAKPFFRRRKVCPFSGENAPAIDYKDTRLLQRYISERGKIVPSRITAVSAKKQRELARAIKRARFLALLPYAVKMSKIALVDDDRNILTSVSMTLEAEGFEVETYNDGQAALDAFNKKLPDMAVLDIKMPRMDGMDLLQRLRQKTQMPVIFLTSKDDEIDEVLGLRMGADDYVKKPFSQRLLVERIRALLRRQEAVEGDA-TGTGPEAKVMERGHLRMDPLRHSVSWKGKDVSLTVTEFLLLQALAQRPGFVKSRDQLMDVAYDDQVYVDDRTIDSHIKRLRKKMRSADDDFSAIETLYGIGYRYNEE------MTAPRFTTLGCRLNAYETEAMKDLAGQAGLDNAVVVNTCAVTAEAVRKARQEIRRLRRENPTARLIVTGCAAQIEPERFAAMPEVDAVIGNTEKMQPATWAGLA----GDF--GTEPVQVNDIMSVTETAGHMIDGFGTRSRAYVQVQNGCDHRCTFCIIPYGRGNSRSVPAGVVVDQIKRLVGSGYHEVVLTGVDLTSWGADLPGTPRLGDLVMRILRLVPDLARLRISSIDSIEADENLMQAIATEPRLMPHLHLSLQHGDDLILKRMKRRHLRDDAIAFCEEARRLRPDMTFGADIIAGFPTETEAHFENSLKLVTDCGLTWLHVFPYSKREGTPAARIPSQVNGKVIKDRAARLRAACDAQVALHLAAQVGQTHAILMENATMGRTEQFTEVMLSEP-----AAEGSIVQRVISGHDGKVLLA-----MKLHELRDNDGATKRRQRVGRGPGSGKGKTGGRGIKGQKSRSGVAINGYEGGQMPLYQRLPKRGFNKPNRKAYAVVNLGLIQKFIDAKKLDAGAEITEDTLVASGLVRRKLDGVRVLAKGEFSSKVTLNVTGASKSAIDAVEKAGGSLTVAA-AAATE-----MARFIFITGGVVSSLGKGLASAALGALLQARGFSVRLRKLDPYLNVDPGTMSPFEHGEVFVTDDGAETDLDLGHYERFTGVAARMSDSVSSGRIYSTVLEKERRGDYLGKTIQVIPHVTNEIKDF-IAIGEDEVDFMLCEIGGTVGDIEGLPFFEAIRQFSQDRPRGQCIFMHLTLLPYIKASGELKTKPTQHSVKELRSIGLAPDILVCRSEGPIPAKEREKLALFCNVRPDSVIAAQDLKSIYEAPLAYHREGLDQAVLDAFQITPAPRPNLDRWEDVADRIYNPEGEVKVAIVGKYTQLEDAYKSIAEALTHGGMANRVKVRVEWVDAEIFD-TEDPAPHLEGFHAILVPGGFGERGTEGKIKAAEFARTRKVPYLGICLGMQMAVIEAARNVAGMKTAGSEEFDHEA-GKKRFEPVVYHLKEWVQGNHTVERKVDDDKGGTMRLGAYDAMLAEGSKVAEVYGSTQIEERHRHRYEVDLRYREKLEEVGLKFSGMSPDGRLPEIVEWQDHPWFIGVQFHPELKSKPFEPHPLFADFVRAAKETSRLV------MIDSA--PRSFQEIILRLQAYWASKGCAVLQPYDMEVGAGTFHPATTLRALGSNPWAAAYVQPSRRPTDGRYGENPNRLQHYYQYQVLIKPSPPDLQDLYLGSLRAIGIDMDLHDIRFVEDDWESPTLGAWGLGWEVWCDGMEVSQFTYFQQVGGHDCAPVSGELTYGLERLAMYVLGVEHVMDMPYNDPGAPIPLSYGDVFRQTEEEYARWNFDVANTEVLLDHFVEAEAECKAILDQDDTDPKTGKRIVMAHPAYDQCIKASHIFNLLDARGVISVTERQAYIGRVRALAKQCADAFVRTGAGGYEA--------------MTQDYIVKDIALADYGRKELDIAETEMPGLMALREEYGESKPLSGARIVGSLHMTIQTAVLIETLVALGADVRWASCNIFSTQDHAAAAIAAGGTPVFAIKGQTLTEHWDYLDKSFLFA---DG--P-NMILDDGGDATLYVLLGARAEAGED-IIPVPTSEEEEVIKAQIKKRMEASPGWFTKVRDQIKGVSEETTTGVNRLYQLVRDGQLPFPAINVNDSVTKSKFDNKYGCKESLVDGIRRATDTMMAGKVAVVMGYGDVGKGSAASLRGAGARVKVTEVDPICALQAAMDGFEVVRLEDVVDTADIFITTTGNKDVIRIEHMREMKNMAIVGNIGHFDNEIQVAALKNHKWTNIKEQVDMIELPSGNKIILLSEGRLLNLGNATGHPSFVMSASFTNQVLAQIELWTKGDEYKNQVYILPKHLDEKVARLHLDRIGVKLTELNPEQAAYIGVKPEGPFKPEHYRYMSDVADRVKKIVVEHLGVEEDKVTENASFIDDLGADSLDTVELVMAFEEEFGIEIPDDAAETIQTFGDAVKFINEAS--MGDWSKSEWRNKPRIQMP-DYTDADKLANVEAKLSKYPPLVFAGEARRLKRELGAAARGEAFVLQGGDCAEAFDQFSADAIRDTFKVMLQMAMVLTFAAKVPVIKVGRMAGQFAKPRSAPTETVDGVELPSYRGDIINDLAFTPEARIPDPDRMLQAYTQAAATLNLIRAFSTGGYADVHQVHAWTLGFTE-SGKADRYREMANRITDALDFMSAAGVTQDTAHTLQSVDYYTSHEALLLEYEEALTRVDTTSGNWLAGSGHMIWIGDRTRQPDGAHVEYCRGVQNPIGLKCGPSMTPEDLKVLMKRLNPKNEQGRLTLIARFGAGKVAEHLPRLIRTVREEGAEVVWVCDPMHGNTIKSSSGFKTRPFDSVLREVREFFAVHRDEGTIPGGVHFEMTGQDVTECTGGVREVTDEDLSDRYHTACDPRLNASQSLELAFLVAEELTSLREAR-RDRAAGMSES--MATPE-----EYGANSIKVLKGLEAVRKRPGMYIGDTDDGSGLHHMVYEVVDNGIDEALAGHADAVTVKIHADSSVSVHDNGRGIPVGIHEEEGVSAAEVIMTQLHAGGKFDSNSYKVSGGLHGVGVSVVNALSVWLELRIWRDGKEHYARF-EHGETAEHLKIVGDSEGK------TGTEVRFLASTDTFSNLEYDFHTLEKRLRELAFLNSGVRIILIDERPAEALRAELYYEGGVKEFVKYLDRSKTPAMAEPIFMTGERDDIGVEVAMWWNDTYHETVLPFTNNIPQRDGGTHMAGFRGALTRTINNYAQSSGIAKREKVSFTGDDAREGLTCVLSVKVPDPKFSSQTKDKLVSSEVRPAVESLVNEKLAEWFEENPNEAKMIVGKIIEAALAREAARKARELTRRKTAMDVNFLAGKLKDCSEKDPAQTEVFLVEGDSAGGSAQTGRDRRTQAVLPLRGKILNVERARFDRMLSSQEIGNLVMALGTGIGRDEFDIKKLRYHKIVIMTDADVDGAHIRTLLLTFFYRQMPELIEGGYLYIAQPPLYKVSRGKSEVYLKDQAAFDEYLINQGVDGAVLRLGSGEEISGQDLTRVVEEARQLKRVLDAFPTHYPRHILEQAAIAGAFVPGAVDSDLQGVADKVAQRLDLIAVEYERGWQGRITQDHGIRLARILRGVEEVRTLDGPMLRSGEARKTGSFTQSLMDVYGVAATLERRDRRQV-IHGPLGLLAAILEEGAKGLSLQRYKGLGEMNPDQLWETTLDPDARTLLQVKVDDMVEADDLFTKLMGDVVEPRREFIQQNALNVENLDF-----MDLKIAFMAGRSAAARAAFDTLVGQYGNVAPEAAEFIVALGGDGFMLRALHATQALSAPVYGMNRGTVGFLMNEYSESDLPDRLRAAVQEVINPLTMVAMDQAGMRHQALAINEVSLLRAGPQAAKLRISVDGRVRLDELVCDGALVATPAGSTAYNYSAHGPILPIGADVLALTAIAAFRPRRWRGALLPTTAHVRFDVIEADKRPVMADADSISIADIDWVEIRSDPRVTHRILFDPGHGLEERLISEQFH-MLGIGTLAKKVFGTPNDRMIKAVRPLVAQINDLEDEFAKLDDAGLKAKTEELAKRAMQGDSLDDLLPEAFANCREAAKRALGLRAFDTQLMGAIFLHRGNIAEQKTGEGKTLTATFAAYLNALPGKGVHVVTVNEYLARRDSEWMGKVFAALGLSSGVIYSEQPPEEKMAAYTSDITYGTNNEFGFDYLRDNMKSDLAQVHQKQHFFAIVDEVDSILIDEARTPLVISGMATDRSDLYQAIDALIPSLTEEHFTIDEKSRQVTFTDEGNEYLEDQLHARGFLEEGQTLYDPESTTIVHHVNQGLRAHKLFLKDKDYMVRNGEVVLVDEFTGRMMAGRRLSDGLHQAIEAKEGVDIKPENVTMASITFQNYFRLYEKLAGMTGTATTEAEEFQEIYGLGVVEIPTNVPIARVDEDDQVYRTAREKYLAMIEEVKAAHAKGQPVLVGTTSIEKSEMLSQMLTAEGIAHNVLNARQHEQEANIVADAGKLGAVTIATNMAGRGTDIQLGGNVESKVLEALEAN--------PDADPEQVRKDETARHAEENRKVKEAGGLCVLASERHESRRIDNQLRGRSGRQGDPGRTAFFLSLEDDLMRIFGSERLDKVLKTLGLKEGEAIVHPWVNKSLERAQAKVEGRNFDIRKQLLKFDDVMNDQRKVIFAQRREIMEAEDLAEIAADMRHQVIDDLVDEHIPAKSYAEQWDTHGLYAQVIEKLGMDVPVIDWAAEDGVDDEAIRERLVKASDEHMAKKASEFGPDNMRNIEKQVLLQSIDSKWREHLLTLEHLRSVVGFRGYAQRDPLNEYKNEGFALFENMLDSLRLTVTQTLSSLRLPSEDEQRKMMEDYLAQQGQTAAPAQPEP-------------------APTGEATAGFDENDP---------------ATWGNPSRNDPCPCGSGKKFKHCHGKVA-------------MGADLKARLVAQARDEGFVACRVCRPGDVPEVAGRLAEFLELGRHGQMGWMAERSHWRGDPAALWPEARSVIMLAESYTPEVDPRAVLERSERGAISVYAQNRDYHDLVKKRLKRLARWLID--AAGGA--EVKVFVDTAPVPEKPLGQAAGLGWQGKHTNLLSRDWGNWAFLGSIFTTLELPVDAPEVDHCGSCRACLDVCPTDAFPAPYQLDARRCISYLTIEHHGPVDPELRALMGNRIYGCDDCLAVCPWNKFAVAASDMRYTARPDLVAPELAELAVLEDAAFRAMFSGSPIKRIGRNRFVRNVLYAIGNSGRTALRAVAQGLVDDADPTVADAARWAAERLAEV--------------------------------MFDKILIANRGEIALRVVRAAREMGIATVAVHSTADSDAMHVRMADESVCIGPPPSPQSYLSVPAIISACEITGAQAIHPGYGFLSENANFVQIVEDHDLTFIGPTAEHIRVMGDKITAKDTMKALGVPCVPGSDGGVPDLAEAKRVGAEIGYPVIIKATAGGGGKGMKVAKSDAEMERAFQTARAEGKANFGNDEVYIEKYLTTPRHIEIQVFGDGKGKAVHLGERDCSLQRRHQKVFEEAPGPSITPEERARIGKICADAMAQINYIGAGTIEFLYENGEFYFIEMNTRLQVEHPVTEGIFGVDLVREQIRVAAGLPMSFGQDDLKINGHAIEVRINAEKLPNFSPCPGRITQYHAPGGLGVRMDSALYDGYSIPPYYDSLIGKLIVHGRDRPEALARLNRALSELIVDGVDTTVPLFDALLQEPDILSGNYNIHWLEHWLESALAKP---MAEPDPDLRPGERDEN-----DRALRPQMLDEFIGQAEARANLKVFIASARARGEAMDHTLFHGPPGLGKTTLAQIMARELGVGFRMTSGPVLAKAGDLAAILSNLEARDVLFIDEIHRLNPAVEEVLYPALEDFELDLVIGEGPAARTLRIELQPFTLVGATTRMGLLTTPLRDRFGIPTRLQFYTVDELHEIVSRNARKMGAPAEDAGALEIARRARGTPRIAGRLLRRVVDFAVVEGDGRITRDLADNALTRLGVDHLGLDGADRRYLRLIAESYGGGPVGIETLSAALSESRDALEEVIEPYLLQQGLIQRTPRGRMLAAAAWRHLGLEAP--RGQNDLF--G----MALDTQ-PP---LNSLGFAKPPAETRVVVAMSGGVDSSVVAAHLAEQGYDVVGVTLQLYDHGAALAKKGACCAGIDIHDARRVAEERGFPHYVLDYENIFKDAVIDEFADSYLAGATPVPCIRCNERVKFKDLLATARDLDADCMATGHYIQRKMGPNGPELHCAEDANRDQSYFLFSTTPEQLAFLRFPLGHLPSKNATRDMAAQYGLAVADKPDSQDICFVPNGDYASVIEKLRPGAGQPGEIVDVNGTVLGTHRGVLHYTIGQRRGLGIGGLADPLYVVKLDVDKAQVVVGPKEMLATRTIPVREINWLGDAPLTSRAEW--HVSVKVRSTRPPREAILRPISETEATVELLSPEEGVSPGQACVFYETE-GTRIFGGGWIWKGA------------MTES--TKTATLSFDGKTFELPILSPTAGPDVLDIRKLYAQAGVFTYDPGFTSTASCDSTITFIDGDKGELLHRGYPIDQLAESSHFLEVCYILLYGELPSPAELEDFEARVTNHTMIHEQMHNFFRGFRRDAHPMATMVGVVGAMSAFYHDSTDISDPWQREVASIRLIAKMPTIAAMAYKYSIGQPFVYPRNDLDYASNFLRMCFSVPAAEYEVNPILSRAMDRIFTLHADHEQNASTSTVRLASSSGANPFACIAAGIACLWGPAHGGANQACLEMLEEIGSVDRIPEFIARAKDKNDPFRLMGFGHRVYKNFDPRASVMKKSADEVLDLLGVEDNPKLQVAKELEKQALEDPYFKDKKLFPNVDFYSGIILEAMGFPTSMFTPIFAVARTVGWISQWKEQISDPQLKIGRPRQLYLGETARDYSDIENRMAELKHIEVRGAREHNLKNIDVDIPRDQLVVITGLSGSGKSSLAFDTIYAEGQRRYVESLSAYARQFLDMMEKPDVDHISGLSPAISIEQKTTSKNPRSTVGTVTEIYDYLRLLFARAGTPYSPATGLPIEAQQVQDMVDRIMDMEEGTRGYLLAPIVRDRKGEYRKEFLELRKQGFQRVKVDGEFYELDEPPTLDKKFRHDIDVVVDRIVVREGLETRLADSLRTALDLADGIAILE-T--APRAGSGENDDAG----APERITFSENFACPVSGFTIPEIEPRLFSFNAPFGACPSCDGLGVELFFDERLVVPDQNLKIYDGALAPWRKGKSPYFLQTIEAIAKHYGFNKNAKWKDLDAKVQQVFLYGSGKEEIPFRYDEGGRVYQVTRVFEGVIPNMERRYRETDSNWVREEFERYQNNRPCGTCGGYRLRPEALAVKIA-----G--------LHVGQVVEMSIREALAWVEGAPEGLSKQKNEIARAIFKEIRERLGFLNNVGLEYLTLSRSSGTLSGGESQRIRLASQIGSGLQGVLYVLDEPSIGLHQRDNDRLLTTLKNLRDQGNTVIVVEHDEEAIREADYVFDIGPGAGVHGGNVVAHGTPAQIAANPDSVTGQYLSGSRQIEVPAERRAGKK-GKSIKVVKASGNNLHDVTAEFPLGKFVCVTGVSGGGKSTLTIETLFKTASMRLNGARQTPAPCETIKGLEHLDKVIDIDQRPIGRTPRSNPATYTGAFTPIRDWFAGLPEAKARGYKPGRFSFNVKGGRCEACQGDGVIKIEMHFLPDVYVTCETCKGARYNRETLEIKFKGKSIADVLDMTVEDAQGFFQAVPSIREKMDALMRVGLGYIKVGQQATTLSGGEAQRVKLSKELSRRSTGRTLYILDEPTTGLHFEDVRKLLEVLHELVDQGNSVVVIEHNLDVIKTADHLIDIGPEGGHGGGHIVAVGTPEEVAEVEASHTGHYL------------------------------------KDMLKPRK-VAAE------MTDK-------TANQMWGGRFAAGPDAIMEAINASIGFDRRMAAQDIAGSRAHAAMLAATGVISDSDAEAIGEGLLTVLSEIEAGDFEFSTALEDIHMNVEARLKDLIGAPAGRLHTGRSRNDQVATDFRLWVRDQLDTAEGGLLALIRALLGQAEAGADWVMPGFTHLQTAQPVTWGHHMMAYVEMFGRDLSRMRDARARMNESPLGAAALAGTSFPIDREMTAKALGFDRPAANSLDAVSDRDFALEFLGAASICAMHLSRMAEELVIWSSAQFRFVALSDRFSTGSSIMPQKKNPDAAELIRAKVGRIFGANTALMMVMKGLPLTYSKDMQEDKEQVFDAADNLMLALAAMEGMVRDMSANRDSLAAAAGSGFSTATDLADWLVRVLGMPFRDAHHVTGSLVALAEGRGCDLPDLTLDDMQSVHAEITDDVFTVLGVENSVNSRMSYGGTAPAQVRAQIERWKSLLE------MKKVYESAADALDGVLFDGMLIAAGGFGLCGIPELLIAAIRDAGTKDLTVASNNAGVDDFGLGVLLQSRQVKKMMSSYVGENAEFMRQYLSGELELEFNPQGTLAERMRAGGSGIPGFYTKTGVGTVIAEGKEVKNFNGQDYILEEGIFADLSIVKAWKADDTGNLIFRKTARNFNPPAAMCGKICIAEVEEIVPRGELDPDLIHLPGIYVHRIIQGEHEKRIEQRTVRKKEEAMKTDIHPDYHVIDVKMTDGTVVQMKSTWGKEGDQLSLDIDPSVHPAWTGGGTRLMDTGGRVSKFKKKYEGLGFMGYRVVVAGATGNVGREMLNILAERQFPVDEITALASRRSLGTEVSFGDKTLTTKDLDTFDFTGWDIALFAIGSDATKKYAPIAAAAGCVVIDNSSLYRYDPDVPLIVPECNADAIEGYSKKNIIANPNCSTAQMVVALKPLHDRAKIKRVVVSTYQSVSGAGKDGMDELWDQTKAVYNPTDDKPAKKFTKQIAFNVIPHIDVFMEDGSTKEEWKMVAETKKILGSSIKVTATCVRVPVFVGHSEAVNIETEDFLDEDEARDILRESPGIMVIDKREDGGYVTPVECVGDFATFISRIRQDSTIDNGLNLWCVSDNLRKGAALNAVQIA-ELLGQRVLKKG--------MRIVFMGTPEFSVPVLEALVEAGHDIACVYCQPPRPAGRGKKDRPTPVHARAEALGLPVRHPVSLKGAEEQADFAALNADVAVVVAYGLILPQAVLDTPAQGCLNIHASLLPRWRGAAPIHRAIMAGDAETGVCIMQMEAGLDTGPVLLRDSVAIGDDTTTGQLHDTLSQMGAALILRALGDLES--LTPQVQPEDGVTYAAKIDKAEARIDWSDPAEVVSRQIRGLSPFPGAWCEID----GARVKLLGAHVVPG-Q-G--LPGEVLDDT----LVIACGAG-AIRPTRVQRAGKSAQDIDSFLRGNPVAPGTRL----MSIPQS----GGGPIERHEQLAEYLADGCKPKDQWRIGTEHEKFGYCKDTLRPIPYEGERSVHAVLAGLRDVHGWAPVEEGGKLIGLTKGGANVSLEPGGQLELSGAPVETIHETCDEVNEHLREVKDVADKVGVGFIGLGSAPEWTHEDMPLMPKGRYKLMDGYMQTVGTLGQTMMRRTCTVQVNLDFSSEADMVQKMRVAIALQPVATALFANSPFTEGKPNGVKSYRSKVWRNLDDARTGMVPFVFDESFGFEAWVQYALDVPMYFVYRDGKYINALGQSFRDFLKGELPALPGETPTLSDWGDHLTTLFPEARIKKFIEMRGADGGPWRRLCALPAFWVGLMYDQGALDAAWDMVRGWDAETRQALRVAASEQGLQAKV-NGIDMHALARDVVALSDAGLKARARTGAGGMVPDETHFLNALKESLETGQVPADELLERYYGAWNGDLSRLYAEYSYMVVLNKIYTRTGDKGTTALGNNDRVAKYAPRVAAYGTVDELNAQLGVARLAASG----------DVDTALMRIQNDLFDLGADLCRPDMEADATAEYPPLRMVQAQVDRLEREIDAMNANLAPLRSFVLPGGSALAAHLHVCRTVARRAERLAVELASEDQ--ANPAAIMYLNRLSDWFFVAARVANDNGADDVLWVPGANRGEGMRHARGYRRLNRTHEHRKALWANMAGSLIEHEQIKTTLPKAKELRPIIEKMITLAKRGDLHARRQAASKLKEDQYVTKLFDILGPRYKDRQGGYVRILKAGFRYGDMAPMAIIEFVDRDRDAKGAADKARVAAEMEAE-TAE---MRIALYPGTFDPITLGHIDIIRRGAALVDRLVIGVAINRDKGPLFSLEERVAMIEAECQKLSEQTGTEIVAHPFENLLIDCAADVGAQMIVRGLRAVADFEYEYQMVGMNRVLNNKIETVFLMAEAKHQAIASKLVKEIARLEGDVSKFVTPQVNEALKAKLASL--MFDLTGKCALITGASGGIGGEIAKALHGAGATVALSGTRVEPLEALAAELGERAHVLPCNLSDAEAVTALPKQAAEAMGAVDILVNNAGITRDNLFMRMSDDEWQSVLDVNLTSTFRLCKGVLRGMMKARWGRIVNISSVVGATGNPGQGNYAAAKAGMVGMGKSLAYEVASRGITVNTVAPGFITTAMTDKLTDDQKSGILGQVPAGRMGEPGEIAAAVLYLASPEAAYVTGATLHVNGGMAMLMALDTHSDIAPYDAPQKDLYEMGEMPPLGYVPKQMYAWAIRRERHGEPDTAMVQEVVDVPTLDSHEVLVLVMAAGVNYNGVWAALGKPISPFDGHGEPYHIAGSDASGIVWAVGDKVKRWKVGDEVVIHCNQDDGDDEECNGGDPMYSPSQRIWGYETPDGSFAQFTNVQAQQLMPRPKHLTWEESACYTLTLATAYRMLFGHEPHDLKPGQNVLVWGASGGLGSYAIQLINTAGANAIGVISDESKRQFVLDIGAKGVINRKDFNCWGQLPTVNTPEYAEWFKEARKFGKAIWDITGKGVNVDMVFEHPGEATFPVSTFVVKKGGMVVICAGTSGFNLTFDVRYMWMHQKRLQGSHFAHLKQASAANKLMLERRLDPCMSEVFTWADLPQAHMKMLRNEHKPGNMSVLVQAPKTGLRTLEEV-LDAKS--------MQGDFRTEFVREPGALRQHPALVLNADYRPLSYYPLSLWPWQEAVKAAWLDRVDIVAEYDEVVRSPSTTIRIPSVVVLKDYVKPQKRVAFTRFNLFLRDEFRCQYCGSKGDLTFDHVVPRASGGVTSWQNVVAACSPCNLKKGSKSLHRAGMHLRKPPRQPAAEDLRNMGRKFPPNHLHDSWIDFLYWDAELEA-MTYIVNDNCIACKYTDCVEVCPVDCFYEGENMLVIHPDECIDCGVCEPECPADAIRPDTDPGTE-TWVEFNRKYAELWPVIITRKDPLPEAEERDGETGKLEKYFSETPGEGGMTEH---------SPNDQFHASSFMQGANAAYLEQLYAQYAGDPNAVDAAWAEFFRALGDAELDVKAEARGPSWARSDWPPQPNDEITGALTGDWGPM--ADEAKAAGRKIADKAVEAGQVVSDDQIKRAVLDSIRALMLIRAYRIRGHLVADLDPLKMRDQT-PHPELDPKTYGFGEADLDRPIFIDNVLGLQIAPMRQIVEIVKRTYCGTFALQYMHISNPEEAGWLKERIEGFDKEITFTREGRKAILNKLVEAEGFEKFLHVKYMGTKRFGLDGGEALIPAMEQIIKRGGALGVRDIIVGMPHRGRLSVLANVMAKPYRAIFNEFQGGSFKPEDVDGSGDVKYHLGASSDREFDGNSVHLSLTANPSHLEAVNPVVLGKARAKQ----DQLSDSDRTKVLPILLHGDAAFAGQGVVAECFGLSGLRGHRTGGTMHIVVNNQIGFTTAPHFSRSSPYPTDIALMVEAPIFHVNGDDPEAVVHAAKVATEFRQKFHKDVVIDIFCYRRFGHNEGDEPMFTNPIMYKNIKTHKTTLSLYTERLVADGLIPEGEIEDMKAAFQAHLNDEFEAGKDYKPNKADWLDGRWSHLDRNQE-DYVRGTTEIAPETLAEIGKALSTAPDGFPVHKTVQRLLDAKAEMFETGKGFDWATGEALAFGSLLTEGYPVRLAGQDSTRGTFSQRHSGLVNQDTEERYYPLNNVRAGQSNYEVIDSMLSEYAVLGFEYGYSLAEPNALVMWEAQFGDFANGAQIMFDQFVSSGESKWLRMSGLVCLLPHGYEGQGPEHSSARLERFLQMCGQDNWIVANCTTPANYFHILRRQIHRSFRKPLILMTPKSLLRHKLAISSAEDFTTGSSFHRVLWDDAEKGNSD--TQLVADDKIKRVVMCSGKVYFDLLEERDARGINDVYLLRIEQFYPFPAISLVKELERFKEAEMVWCQEEPKNQGGWSFIEPNIEWVLNRIGARHQRPVYAGRATSASPATGLASQHKAQQAALVNEALSIEGN--------MIGLEHYLTVAATLFVIGIFGLFLNRKNIIILLMSIELMLLAVNINLVAFSSYLGDMVGQVFTLFVLTVAAAEAAIGLAILVCFFRNRGTIAVEDANVMKGMTKVKAR--GAQALPLNTILDGDCIEMMNSLPEASVDLIFADPPYNLQLRGELHRPDNSQVDAVNDHWDQFSSFAAYDEFTRAWLAAARRLLKPNGAIWVIGSYHNIFRVGAALQNEGFWILNDVVWRKSNPMPNFRGKRLTNAHETMIWASKSEGAKYTFNYEALKSLNEGIQMRSDWVLPICTGHERLKDENGDKAHPTQKPESLLHRVIVGSTNPGDVVLDPFFGTGTTGAVAKMLGREFIGIEREEAYRKAATARIAKVRKFDREALQVSTSKRAEPRVPFGQLVERGMLRPGEELFSMNNRHKAKVRADGTLIGDDIKGSIHQVGAHLEGAPSCNGWTYWCYKRDGKRV-PIDVLRQQIRAEMSA---MLDTAKYTPRLKAAFNDTIRAAMKEEFGYKNDMQIPRLDKIVLNIGAGAEAVRDSKKAKSAQEDLTAIAGQRAVITKAKKSIAGFRVREDMPLGAKVTLRGDRMYEFLDRLITVALPRVRDFRGVSGKSFDGRGNYAMGLKEHIVFPEINFDKVDEVWGMDIVIATTANTDAEAKALLKLFNMPFNSMAEV-EREAMEYDVVIVGAGPAGLSAAIRLKQL----DADLSVVVLEKGSEVGAHILSGAVLDPCGLDALIPDWKEKGA-PVTVPVKEDNFYILGEAGQMRIPNFPMPPLMHNHGNYIVSMGNVCRWMAEQAEELGVEIFPGMACSEIVYGENGEVKGVVAGEFGKEPDGSHGPSYEPGMELHGKYVFLGEGVRGSLAKQVIAKYDLQAGKEPQKFGLGMKEIWEIDPAKHREGTVTHTMGWPLG-SNAGGGSFIYHLDNNQ---VYVGFVVHLNYKNPHLFPYMEFQRFKHHPIVSELLKGGKRVAYGARAITEGGYQSMPKMVAPGVAMLGCSVGMVNVPRIKGNHNAMLSGKAAAEAAFAAIKDGRSADELSDYETEVRNGAIGADLKKVRNVKPFWSKYGLAASLALGGFDMWTNT-FGFSLFGTQSHGKTDAAATEPASKHEPIDYPKPDGKLSFDRLTNVSFSMTNHEESQPPHLTLKDPDVPVRVDYSEYAGPSQRYCPAGVYEFVEDD-NGKPKFVINFQNCVHCKTCDIKDPAQNIVWTTPQGGDGPNYPNMMTTGRVNPKFRLEDQGIEGLGQVHYNLTEPALVQAALMRGEGKLGNGGAFLVSTGKFTGRSPKDKHVVKTDSVAEKIWWENNAAMSPEGFDALYADMTEHMKGRDFFVQDLVGGADPANSIDVRMVTELAWHGLFIRHMLRRPDRADLDDFTADWTVINCPSFKADPAKHDCRSETVIAMNFDRKIILIGGTEYAGENKKSVFTLLNYLLPEKGIMPMHCSANHAVGNPVDTAIFFGLSGTGKTTLSADPARVLIGDDEHGWSDRGTFNFEGGCYAKTISLSPEAEPEIYATTSKFGTVIENMVFDPETFELDFEDDSLTANMRAAYPLEYISNASASALGGHPKNIIMLTCDAFGVLPPIARLTPAQAMYHFLSGFTSKVAGTERGVTEPEPTFSTCFGAPFMPRRPEVYGNLLRDKIAQHGATCWLVNTGWTGGAYGTGHRMPIKATRGLLTAALDGTLANAEFRKDPNFGFDVPVDVPGVPQLLLDPRRTWGNAEGYDQAAAKLVQMFADNFAQYVPYIDDDVKACAIG-----MTTYDDAWARAEEAKRKWMAENSLYTEETEHSSCGVGLVVSVDGKKSRKVVESGIKALKAIWHRGAVDADGMTGDGAGIHVEIPVPFFYDQIERTGHKPRKTELIAVGQVFLPRTDFGAQETCRTIVETEVLRMGYYIYGWRHVPVDTSCLGEKANATRPEIEQILISNAKGVDEETFERELYVIRRRIEKAAASAGINELYLASLSCRSIIYKGMMLAEQVAVFYPDLMDERFESAFAIYHQRYSTNTFPQWWLAQPFRMLAHNGEINTLKGNTNWMKSHEIRMASGTFGDMADDIKPIIAGGSSDSAALDAVFEVLVRAGRNAPMAKTMLVPESWSKQAVELPQAWRDMYSYCNSVMEPWDGPAALAMTDGRWVCAGLDRNGLRPMRYVVTGDGLLIAGSEAGMVPVDEARIVEKGALGPGQMIAVDMKNGQLFHDTEIKDQLAASQPFGDWVGKINDADEVLAGVQEKALFTGNDLRKRQIAAGYSLEELEQILSPMAEDGKEALASMGDDTPSAVLSKQYRPLSHFFRQNFSQVTNPPIDSLREFRVMSLKTRFGNLKNVLDEDSSQTEIVVLESPFVANAQFDKVIEQFNAPMIEIDCTFAPGE---R--ALHAALARIRSEAEDAVRSGAGHIVLTDQHSGPGKVAMPMILATSAIHSHLIRNGLRTFCSLVVRSAECIDPHYFAVLIGCGATVVNAYLAEDSIADRIERGLL-DGPLTEAVSRYRDAVDQGLLKIMAKMGISVVSSYRGGLNFEAVGLSRAMCAEYFPGMTSRISGIGVSGIQRKAEEVHARAWANDAAVLPIGGFYKARASGETHAWEATSMHMLQMACNKASFELWKQYSKKMQSNPPTHLRDLLDIKPLGKPVPIEEVESITSIRKRFVTPGMSLGALSPEAHKTLNVAMNRIGAKSDSGEGGEDPAHFVPEPNGDNPSAKIKQVASGRFGVTAEYLNQCEELEIKVAQGAKPGEGGQLPGMKVTDLIARLRHSTKGVTLISPPPHHDIYSIEDLAQLIYDLKQINPRCKVTVKLVASSGVGTIAAGVAKAKADIILISGHNGGTGASPATSIKYAGLPWEMGLTEAHQVLAMNKLRDRVTLRTDGGLRTGRDIVMAAMMGAEEFGIGTAALIAMGCIMVRQCQSNTCPVGVCTQDEALRGKFTGNADKVVNLITFYAQEVRELLAAIGARTLDEVIGRADLLAQVSRGSAHLDDLDLNPLLITVDGASKIKYNRDKPRNEVPDTLDAEIVRDAARFLKDGEKMQLSYAVQNTHRTVGTRTSSHIVRNFGMRNAFQADHLTVKLQGSAGQSLGAFAAPGLKLEVSGDANDYVGKGLSGGTIVVRPPMASPLVASDNTIIGNTVLYGATDGYLFAAGRAGERFAVRNSGAKVVIEGCGSNGCEYMTGGVAVILGEIGANFGAGMTGGMAYLYDPEGKARTLMNMETLVTCPVTV--PHWELQLEDLIERHLKETGSRRAADILQHWDLEKQNFLQVCPKEMLIHLAAPLSLEQG-AIPAE-MKPTVASFPRARLRRTRRTPALRGLVRETLLSPDDLIWPVFVREGDGVEEPIPSMPGVMRRSVDRIAEAAREAHALGIGAICIFPYTPLEARTEDCAEAWSPDNIANRAIRAVKAAVPDMAVMTDVALDPYNINGHDGFV--VDGEIVNDRTVEALVRMTLAQAEAGADIIGPSDMMDGRIAAMRDALEAKGHSHVSILSYAAKFASAYYGPFRDAVGASGALTGDKKTYQMDVANSDEAMRLIERDLMEGADMVMVKPGTPYLDICRRARDTFGAPTFAYQVSGEYAMIQAAAQNGWIDGEKVMMESLIGFKRAGCNGVLTYFAPEAARLLNG---MGIQAAEISAILKDQIKNFGQEAEVAEVGRVLSVGDGIARVYGLDNVQAGEMVEFPGGIMGMALNLETDNVGVVIFGSDRDIKEGDTVKRTNSIVDVPIGPEMLGRVVDGLGNPVDGKGPI-TTSQRGIADVKAPGIIPRKSVHEPMATGLKSVDAMIPIGRGQRELIIGDRQTGKTAVALDAILNQKTYNDRAGDDESKKLYCVYVAIGQKRSTVAQLVKKLEETGAIEYSIVVAATASEPAPMQYLAPYTATAMAEYFRDNGKHALIIYDDLSKQAVSYRQMSLLLRRPPGREAYPGDVFYLHSRLLERSAKLNEEFGAGSLTALPIIETQGGDVSAFIPTNVISITDGQIFLETELFYQGIRPAVNTGLSVSRVGSSAQTNAMKSVAGPVKLELAQYREMAAFAQFGSDLDAATQRLLNRGARLTELMKQPQYSPLTNAEIVAVIYAGTHGYLDKLPLNEVGRFEKGLLAHLRGKRQDVLDWLTTEDPKIKGEAEDKLKAALDEFAADYA------MTQTTDKNRVLIFDTTLRDGEQSPGATMTHAEKLEIAALLDEMGVDIIEAGFPIASEGDFKAVSEIAKNSVNSVICGLARAQLPDIDRCWEAVKHAAQPRIHTFIGTSPLH-RAIPNLSQDEMAERIHETVTHARNLCDNVQWSPMDATRTEWDYLARVVEIAIKAGASTINIPDTVGYTAPVESAELIRRLIAEVPGADEVIFATHCHNDLGMATANSLAAVAGGARQIECTINGLGERAGNTALEEVVMALKVRHDIMPWHTNVDTTKIMNISRRVATVSGFPVQFNKAVVGKNAFAHESGIHQDGMLKNVETFEIMRPEDVGLTESNLVMGKHSGRAALRDKLKHLGFEVGENQLKDVFVRFKELADRKKEVYDDDIVALMRA-GEDPENDHIKLKSLRVVCGTSGPQSADMVLEIGGSEQKTEQTGDGPVDAAFNAVKALFPHSARLQLYQVHAVTEGTDAQATVSVRLEEDGRVVTGQSADTDTVVASVKAYIHALNRLVVRRAKSAPGEDRREISYKD-VS---MA-AYDDRDGKIWLDGKMVDWRDANVHILSHALHYASSVFEGERCYNGKIFKGVEHSERLRKSAQMLDFEIPYTVEEIEKAKYDMLAANGWTDAYVRAVAWRGAGEDMGVSAKRNPVRLAITGWEWGNYYGDAKFKGAKLDISKWKRPSPETIPVHAKAAGLYMICTMSKHEAEAKGCSDALFMDYRGYVAEATGANIFFVKDGEVHTPLADCFLNGLTRQTVIQMLKDRQIVVHERHIMPEELESFEQCWLTGTAAEVTPVGQIGDFTFEVGALTRDISDSYEKLVRA-------MSP----KTLYDKIWDAHVAH-EAEDGTCLLYIDRHLVHEVTSPQAFEGLRMSGRKVRAPEKTIAVPDHNVPTTLDRDDATTMTE-DSRIQVEALDKNARDFGVHYYPVNDVRQGIVHIVGPEQGWTLPGMTVVCGDSHTATHGAFGALAHGIGTSEVEHVLATQTLIQKKSKNMKVEITGKLAPGVTAKDITLAVIGKTGTAGGTGYVIEYCGEAIRGLSMEGRMTVCNMAIEGGARAGLIAPDETTFEYCKGRPHAPKGAQWEAAMEWWKTLYSDDDAHWDKVLTIKGEDIAPVVTWGTSPEDVLPITAKVPAAADFTGG-KVGAAQRSLDYMGLTPGTPLNEVAIDTVFIGSCTNGRIEDLRAAAAILKGKKVKDGLRAMVVPGSGLVRAQAEEEGLADIFKDAGFEWRLAGCSMCLAMNPDQLAPGERCASTSNRNFEGRQGFKGRTHLMSPAMAAAAAITGKLTDVREMM-----------MLKPPYMM-----------SKPSDGDD-DTSVIVDVKPKTKRPPLYKVLLLNDDYTPMEFVVHVLERFFGLNHAQAFEIMLTVHKKGVAVVGVFSHEIAETKVGQVMDFARRHQHPLQCTMEKED-MSDTPFRPFETTLPLDEALVELRSATHGADDGEIFVERRRSESLVFDDGRLKSANYDASEGFGLRAVRGEVAGYAHSTEVSMAALKRAGDTARLAVGDGGGTLAEPPTPTNRRLYTDADPIGGTAFSVKIDTLREIDAFARGLDSRVVQVSAALSASVQEVEILRPDGVHVRDVRPMVRLNVSVIVEENGRRETGTAGGGGRLGLDGLIAPADWQAKTREALRIALVNLGAEPAPAGVMEVVLGPGWPGILLHEAIGHGLEGDFNRKGSSAFAGLMGQRIAAPGVTVLDDGTIPDRRGSITVDDEGTPSGKTTLIEDGILVGFMQDRQNARLMNTAPTGNGRRQSFAHKPMPRMTNTYMLGGDADPSDIVASMKDGIWAVGFGGGQVDITNGKFVFSCTEAYRVKNGKIGAPVKGATLIGDGATALNRIRALGNDMALDPGLGNCGKDGQWVPVGVGQPTVMIDGLTVGGAAT---MVPEERLEQIFQRFQYLEAAMSDGR--GDIAKLAREYSALKPVVEQIGEWRTLRDDMAEAKAMLA--DPDMKELAEEELPRLRARLPEVEHALQLALLPRDEADVRPAILEIRPGTGGDEAALFAADLLRMYHRYAEARGWSLDIIEEQATELGGIKEVVAHIKGDNVFARLKYESGVHRVQRVPSTESGGRIHTSAATVAVLPEAEDVDIQIDANDLRIDTMRSSGAGGQHVNTTDSAVRITHIPTGLVVTSSEKSQHRNREIAMQVLKTRLYDLERQRVDSERSANRAAQVGSGDRSERIRTYNFPQGRMTDHRINLTLYKLDQVMQGD-LDDIIDALIADGQARMLADMGQ------------------------------------------MLTKRAKYNLGQVVRHKKHPFRGVVFDVDPEFSNTEEWYQAIPEDSRPTKAQPFYHLLAENEQSYYVAYVSEQNLVADYSGEPVDHPDIPDMFGPFQDGSYPLHFHLN--MAWNTLDDM-ELNGKRVLVRVDINVPLDN-GRVTDDTRIQRIVPTLRDIMAAGGRPVILAHFGRPKGQHVPEMSLGQLAEPLAAATGKPVLFCADCRGHAAEAAIEAQ-----DDNQIVLLENTRFYPGEEKNDPELAAEFAKLGDIYCNDAFSAAHRAHASTEGVARLLPSCAGRLMQAELRALESALGTPERPVAAVVGGAKVSTKLDLLGNLVSKVDMLVIGGGMANTFLAANGIDVGKSLCEHDMADTAREIAAKAAEAGCEILLPRDVVVAREFKAGAKNRTC---GVEDVEADEMILDAGPETVAHVAMALERAKTLIWNGPLGAFEIAPFDAATNAAAQQAAELTRAGTLISVAGGGDTVAALNHAGAGEDFTYISTAGGAFLEWMEGKELPGVAALTRPA----------MQLKK---------LLLVDDDEDLREALSEQLVMTEDFDVFEASDGASAMERVKDSLFDLVILDVGLPDTDGRELCRLMRKQGVKSPILMLTGHDSDADTILGLDAGANDYITKPFKFPVLLARIRAQLRQHEQSEDAVFQLGPYTFKPAMKLLVTEDDRKIRLTEKETNILKFLYRSSEGVVARDVLLHEVWGYNAGVTTHTLETHIYRLRQKIEPDPSNARLLVTESGGYRLVAMAKST--TFVCTACGASSSKWSGRCEACGEWNTIIEEKPLSVGPASKSL---GGKRGRTIPLSDLSTRETPPPRTCSGIDELDRVLGGGLVDASAILVGGDPGIGKSTLLLQGAAKFAMAGLKTIYISGEEASAQVRMRAQRLGLAEAPVRLATETSLRDILTTLEAEKPDLVVIDSIQTMWADNVDSAPGSVSQVRAAAHELTTFAKRHGTSVILVGHVTKEGQIAGPRVVEHMVDTVLYFEGERGHQFRILRAVKNRFGPADEIGVFEMTGRGLSEVTNPSALFLSERGTPSPGSVVFAGIEGTRPVLVELQALVAPSPHSQPRRAVVGWDGSRLAMILAVLEARCGIPFAGLDVYLNVAGGMKISEPAADLAVAAALLSAREDAALPAETVVFGEISLSGALRPAPQTENRLKEAQKLGFTAAIAPAGGKSGTDAGMKITRATDLTGFVGEVFGA--G---MAKEDILEFPGVVKELLPNATFRVELENGHEIIAHTAGKMRKNRIRVLAGDKVQVEMTPYDLTKGRINYRFK---------------MTAPKKLFVKTYGCQMNVYDSERMAEALGGQGYVTTDRADDADMILLNTCHIREKAAEKVYSELGRFKGLKEEKPD-LKIGVAGCVAQAEGAEIMRRQPLVDLVVGPQSYHRLPELEAKA-RSGEKALDTDFPEEDKFEKLKGK-RKAQRGPTAFLTVQEGCDKFCAFCVVPYTRGAEVSRPADRILDEARDLVERGVREITLLGQNVNAYHGAGD--GG--DWSLARLIWALDKIDGLERIRFTTSHPNDMGDDLIAAHGECSKLMPYLHLPVQAGSDKILKRMNRSHTAESYLRLIERIRAARPDILMSGDFIVGFPEETEEDFQATMALIEEVNYGYAYSFKYSTRPGTPAAERA-QVPEDEKDDRLQRLQALITRQQRACQDGMVGREVGVLFEKPGRFAGQMVGKSDYLHAVHVTAPDVAVGEIHRVRIVDSGANSLAGEVIAPV----MGHSSVTPLKVAVLMGGPSSEREVSLSTGRECAAALRGEG-YEVVEVDAGPDLAQRLSAIAPDVAFNALHGRWGEDGCVQGILEWLRIPYTHSGVLASAMAMDKQRTKAAYESAGLPVVPSLIAARDDIAARHLMEPPYVVKPNNEGSSVGIYFVHDAADSPPQ----IADDMPARMMVEAYVPGRELTVTAMDGRALTVTEIITD-GWYDYDAKYAPGGSRHVVPAEVPADIFDLCMDYALHAHRALGCRGISRTDFRWDDTRGAKGLFLLETNTQPGMTPTSLSPEQAGAAGMSFGALCRWLVEDASCDRMAA-----KD-NDDSKTEDQDAEIS--LDMSQAAVKKMIGEAREKGYITYDQLNQVLPPDQVSSEQIEDVMSMLSEMGINIIE-DEEAEEEE--QKGSTDVVAQNDAREITLGSGTQEKLDRTDDPVRMYLREMGSVELLSREGEIAIAKRIEAGRNTMIAGLCESPLTFQAITIWRDELLSEDILLRDVIDLETTFGNQMGEDGDVEEPLV----------AGASTSETAKEKTEDSPELDADGNPIAR---DDDEDEDDQANMSLAAMEAALKDQVLTTLEVIANNYAQLSEMQDSRISATLNEDGSFSDQDESTYQSLRSEIVELVNGLHLHNNRIEALIDQLYGINRRVMSIDSSMVKLADQARINRREFVDEYRGRELDPNWLSDMADKPGRGWQMFIERSTDKVEELRADMAQVGQYVGLDISEFRRIVQQVQKGEKEARQAKKEMVEANLRLVISIAKKYTNRGLQFLDLIQEGNIGLMKAVDKFEYRRGYKFSTYATWWIRQAITRSIADQARTIRIPVHMIETINKLVRTGRQMLHEIGREPTPEELAEKLQMPLEKVRKVMKIAKEPISLETPIGDEEDSQLGDFIEDKNAVLPLDSAIQENLKETTTRVLASLTPREERVLRMRFGIGMNTDHTLEEVGQQFSVTRERIRQIEAKALRKLKHPSRSRKLRSFLDQ--MAQNASMEEFEALLNESFEMDTPEEGSVVKGKVIAIEAGQAIIDVGYKMEGRVELKEFANPGEAPDISVGDEVEVYLRQVENARGEAVISREMARREEAWDRLEKAYASEERVEGAIFGRVKGGFTVDLGGAVAFLPGSQVDVRPVRDAGPLMGLKQPFQILKMDRRRGNIVVSRRAILEESRAEQRAEVIGNLSEGQAVDGVVKNITEYGAFVDLGGVDGLLHVTDMAWRRVNHPSEILSIGETVKVQVIKINKETHRISLGMKQLQEDPWDLVGAKYPLSSVHKGRVTNITDYGAFVELEPGVEGLVHVSEMSWTKKNVHPGKIVSTSQEVDVMVLEIDGAKRRVSLGLKQTMRNPWEVFAETHPEGTEVEGEVKNITEFGLFIGLEGEIDGMVHLSDLSWDERGEDAIQNYRKGDVVQAVVSEVDIEKERISLSIKALGGDRFAEAV-------GGVKRGSIITVAVTSIEDGGIEVEYE--GMKSFIRRSDLSRDRAEQRPERFSVGDKVDVRVTNVDSKTRRLGLSIKAREIAEEKEAVEQYGSSDSGASLGDILGAAL-------------------------KG--DE-------------------------------------------------------------MQELTHYIGGEHVKGTSGRFADVMNPATGEVQAKVPLASADELDRAVQIAAEAQPAWAAVNPQRRARVLMKFVDLLNRDMDKLAEALSREHGKTLPDAAGDVQRGLEVVEYCIGAPQLLKGEYTDSAGPGIDMYSMRQALGVTAGITPFNFPAMIPMWMFAPAIACGNAFILKPSERDPSVPLMLAELMEEAGLPKGILQVVNGDKEAVDAILYHDVIQSIGFVGSTPIAEYIYGTGCAQGKRVQCFGGAKNHMIVMPDADMDQAADALIGAGYGAAGERCMAISVAVPVGDETADRLIEKLVPRIEKLKVGPYTAGADVDYGPVVTAAAKENILRLVQSGVDQGAELVVDGRDFNLQGYENGFFVGAHLFDKVTPDMDIYKKEIFGPVLSTVRAGSYEEALKLAMDHEYGNGTAIFTRDGDTARDFASRVNVGMVGVNVPIPVPLAYHTFGGWKKSVFGDLNQHGPDAFKFYTRTKTVTSRWPSGLKEGGEFHFKPMD-----MNYDAKLDDAIGRLHEEGRYRTFIDIERRNGMFPHAVWTRPDGSERDITVWCGNDYLGMGQNPVVLDAMKEAVDAAGAGSGGTRNISGTTVYHKRLEAELADLHGKEAALLFTSAYIANDATLSTLPKLFPGLIIYSDALNHASMIEGVRRNGGAKRIFRHNDVAHLRELLEADDPKAPKLIAFESVYSMDGDFGPIEAICDLADEFGALTYIDEVHAVGMYGPRGAGVAERDRLMHRLDIINGTLAKAYGVMGGYIAASARMCDAIRSYAPGFIFTTSLPPAVAAGAAASVAYLKRATD--LREKHQTGAKILKTRLKAMGLPIIDHGSHIVPVIVGNPVHTKILSDMLLEDHGIYVQPINFPTVPRGTERLRFTPSPVHGPKEMDALIRAMDKLWSHCALNRAELAG---MARYLITSAIPYINGIKHLGNLVGSQLPADLFARYQRGRGNEVLFLCATDEHGTPAELAAAKAGKPVAEYCAEMWEVQARIADGFGLSFDHFGRSSSEQNRKLTQHFAARLDDAGLIREVSEKQMYSHADGRFLPDRYIEGTCPNCGFDSARGDQCDNCTKQLDPVDLINPHSTISGSTDLEMRETKHLYLAQSQMKDELEQWIDTREG---------------------------WPVLTTSIAKKWLNDGDGLQDRGITRDLDWGVPVMKGD-APWPGMEGKVFYVWFDAPIEYIACAQEWVDA-GKGD-DWARWWRTDKGADDVTYVQFMGKDNVPFHTLSFPVTIL---------GSKEPWKLVDYIKSFNYLNYDGGQFSTSRGRGVFMDQALDILPADYWRWWLLSHAPESSDAEFTWDNFQATVNKDLADVLGNFVSRITKFCRSKFGEVVPEGGTQGEAEAALLAELTTRVRAYEGFMQAMEVRKSAQELRAIWAAGNEYLQAAAPWTTFKENPEQAAMQVRLGLNLIRLYAVLSAPFIPEASARMLSAMN-TLDM-------EWPGDIDAALAALPAGHAFTVPDVLFAKITDDQREDWQARFAGVRT---MQPVEIYTTPLCGFCHAAKRLLTQKGVSFAEVDVAAQPERRAEMTQRANGGRTVPQIFIGDTHVGGCDDLYALERAGKLDPLLAGK--------------MKTVLVSAVALIDRDGRVLLAQRPEGKAMAGLWEFPGGKVEPGETPEAALIRELHEELGIETWQSCLAPLTFASHSYDSFHLLMPLFACRKWQGTPQPREGQRLAWARPNEMRSYPMPEADVPLIAILRDWL--------MARKNYIFTSESVSEGHPDKVCDRISDAVLDAFLSEEAEARVAAETFATTNRVVIGGEVGLSDQDKLHDYMGRIEGIARECIRDIGYEQDKFHWKTCEITNLLHEQSAHIAQGVNAAEN--KDEGAGDQGIMFGFATNETEALMPAPIQYSHAILRRLAEVRKNGTEPALGPDAKSQLSVIYRDGKPVGIHSLVLSTQHLDESLTSEDIRAIVAPYFHEVLPEGWLTDDTIWHVNPTGKFVIGGPDGDAGLTGRKIIVDTYGGAAPHGGGAFSGKDPTKVDRSAAYAARYLAKNVVAAGMADKCTIQLSYAIGVSEPLSIYANTH-DTGEVPPAAIEKAIRKVMSLTPRGIREHLQLNKPIYQRTAAYGHFGRAPEADGGFSWERTDLVEALQNAV-MAVTNETPLVTDVQGSGQQADRVKSGPSSLAPYA---AGADREVATSALNAELQDKGFLLTSSEDLINWARTGSLHWMTFGLACCAVEMMHTSMPRYDAERFGIAPRASPRQSDVMIVAGTLTNKMAPALRKVYDQMPEPRYVISMGSCANGGGYYHYSYSVVRGCDRIVPVDIYVPGCPPTAEALLYGLLQLQRKIRRTGTIVRMALPEFTMRQLLEAGVHFGHQTQRWNPRMAPYIYGDRNGIHIMDLTQTVPMLDQALQAIRDTVARNGRVLFVGTKRQAAKAVAEAAEKSAQYYMNHRWLGGTLTNWKTVSQSINRLKHIDEQMEQGFG-GLTKKERLGMERDQAKLNASLGGIREMGGTPDLLFVIDVKKEALAIAEANKLGIPVVAVVDTNCSPDGVDYVIPGNDDAARAIALYCDLASRAALDGMSAQLGAAGVDIGAMEDAVVEEALAE--------------------------GEAETTEA------------------MTMREEPVRQRVSKVRSAARLSVAPMMDWTDRHCRFLHRQFSARTLLYTEMVTAPALVRGGATHLLDHDAREQPVALQLGGSDPGELAEAAALGADAGYPEINLNCGCPSDRVKSGLFGAVLMADPARVAACVSAMAQSVG-----DRAEITVKCRIGIDDQTPATVLPDFLQQIADAGVSRVTVHARKAWLQGLSPKENRDIPPLDYDLVLRMKDRFPNLHLSVNGGITTLDQAQGFLDRGIDGVMIGRAAYHDPASVLSRADPEIYGQGTAA-DPDKVVTAMRPYIAAHLEQGGKLHQISRHMLGLFTGRPGARIWRRHLSEGAHRPGAGLEVVDAAQTARTEA--EATTAETL----------MLLLIDNYDSFTYNLVHYLGELGAEVVVRRNDALSVDDAMAMNPAAILLSPGPCDPDRAGICLGLTQAAAQSGTPLLGVCLGHQTIGQAFGGRVVRAGEIVHGKMGAIRHDGGGVFAGLPSPFAATRYHSLVVDRESLPDALEITAALEDGTIMGLSHRTLPIHGVQFHPESIASEHGHALLGNFLKLMKVPAMIRSELIQKIAEENPHLYQRDVERIVNTVFEEVTGAMARGDRVELRGFGAFSVKKRDARVGRNPRTGETVTVEEKHVPFFKTGKLLRDRLNGKEE----MGFFDFLGGLFTADMAIDLGTANTLVYVKGRGVILSEPSVVAYHVKDGVKKVLAVGEDAKLMLGRTPGSIEAIRPMREGVIADFDTAEEMIKHFIRKVHKRSTFSKPKIIVCVPHGATPVEKRAIRQSVLSAGARRAGLIAEPIAAAIGAGMPITDPTGNMVVDIGGGTTEVAVLSLGDIVYARSVRVGGDRMDEAIISYLRRQQNLLVGESTAERIKTSIGTARMPDDGRGTSMQIRGRDLLNGVPKEIEISQAQVAEALAEPVQQICEAVMTALEATPPDLAADIVDRGVMLTGGGALLGDLDLALREQTGLAVSIADESLNCVALGTGKALEYEKQLRHAIDYDS---------------------------------------------------MAELEQLAKQLGQANQAYHGDDAPVMSDDDYDRLKRRNAAIEEKFPDLKRGDSPSDQVGARPSDGFSKVTHAVRMLSLGNAFDDDDVTDFDGSVRKYLNIGPDTPLAYTSEPKIDGLSLSLRYESGVLVQAATRGDGSVGENVTANARTISDIPERLNGA--PDVLEVRGEVYMAHDDFAALNARQSENGDKTFANPRNAAAGSLRQLDSGVTRKRPLRFFAYAWGEVSEPLSDTQYGAIKRLAELGFSTNPLTQRCNGPDEMIAHYRSIEEQRATLGYDIDGVVYKVDDLALQSRLGFRSTTPRWAIAHKFPAELAWTRLESIDIQVGRTGALSPVARLTPVTVGGVVVSNATLHNEDYIAGRDSRGGDIRGGKDIRVGDWVQVYRAGDVIPKV-ADVELEKRPKDAQPFQFPETCPECGSPAVREEGDSVR-----RCSGGLICPAQAVEKLRHMVSRAAFDIEGLGAKQVEQFYADG--WIAKPADVFRLRDTY-GSGIQQLKNREGWGETSARKLFDAIDEKRRIDLARFIFSLGIRHVGEVAARDLALHYLGWDALTQALDAARPAALAHRAADEAETIERRVAQDEGRRARIAEVRAAAIAAVAPGDVAQAAWDDLIGVDGIGPALAASLSDALANPDERTAIEDLAQQLNIQPPDAPAAD-SPVAGKTVVFTGTLEKMTRAEAKARAEALGAKVAGSVSAKTDILIAGPGAGSKAKKAAELGIDTLDEDGWLALIGDTMRTLNDIRSTFLSYFARNGHEVVDSSPLVPRNDPTLMFANSGMVQFKNLFTGVETRDYSRATTAQKCVRAGGKHNDLDNVGYTARHHTFFEMLGNFSFGDYFKSDAIPFAWEMVTRELGIPKDKLLVTVYKSDDEAAEIWKKVAGLTDDRIIRIGDKPDGGSDNFWQMGPTGPCGPCTEIFYDHGEKFWGGPPGSPEEDGDRFIEIWNLVFMQFEQFEDGSRRDLEAQSIDTGMGIERVAALLQGTNDNYATDLMRSLIEASADATSTDPDGPGKTHHRVIADHLRSTSFLLADGVMPSNDGRGYVLRRIMRRAMRHAHLLGAKDPLMHRLVPALVRQMGAAYPELGRAQALIEETLKLEETRFKQTLDRGLKLLDDELADLPEGAELPGAAAFKLYDTYGFPLDLTQDALREKGRAVDTDGFDAAMAEQKAKARAAWSGTGEAADAAVWYDVADAHGATDFLGYDTETAEGQIAALIVD-GAQAEQ-AGEGTSVQIALNQTPFYAESGGQVGDTGLIRT---ETG---------LARVSDTKKT-AG--VFVHIAEVTEGTVSQGQAATLEVDHTRRSMIRANHSATHLLHEALRGALGDHVAQRGSLNAPDRLRFDFSHGKALAADELSRVEGEVNQFIRQNSAVDTRIMTPDDARAMGAQALFGEKYGDEVRVVSMGQAP-TGKGANKQTYSIELCGGTHVARTGDIGAFVLLGDSASSAGVRRIEALTGQAALAHLRDQDQRLGDVAAALKAQPSDVPARVRALLDERKALANEVAQLRRDLAMSGGAG--KAGEEAAAREVNGVPFLAQVLSGVSGKDLPALIDEHKSRLESGVVLLIADTGGKAAVAAGVTGDLTGRVSAVDLVRAAVAELGGKGGGGRPDMAQGGGADAANAEAAIKAAETVLEG---MSAKAEHYDVIRKPIITEKSTMASENGAVVFEVAMDSNKPQIKEAVEALFNVKVKAVNTTITKGKTKRFRGLMGKRKDVKKAYVTLEEGNTIDVTTGLMPSLKDLKNRIESVKSTRKITKAMQMVAAAKLRRAQEAAEDARPYTERFNAVMAGLAASVGGSDSAPKLLGGTGSDQVHLLVVLTAERGLCGGFNSNIVRLARTHAAKLKAAGKTVKILTVGKKGRDALKRDSADLMVGHVDLSEVKRLSYADAQ-GIAQNVLSRFDAGEFDVATIFYSKFVNVVSQVPTAQQVIPADFPAEDGAQDS---VSTVYDYEPGEEEILADLLPRGVATQIFSALLENGASEQGARMSAMDNATRNAGDMIDKLTIQFNRSRQAVITNELIEIISGAEAL--------MNDLLS--T--APSKEYDASSIQVLEDMEHVRLRPGMYIGGKDDRALHHMVAEIIDNSMDEAVAGHATWIEVELHENGHVSVRDNGRGIPIGMHPKDPTKSALEIIFCTLNAGGKFSGDSYETSGGLHGVGSSVVNALSDHLRVEVAQNRELYAMEFSRGVPQTKLEKIGAAPNRRGTSVTFHPDPEIFGS-LQLKPHRLFKMARSKAYLFSGVEIRWKSA----IPDGDTPLEAKFHFPGGLADYLSETLGQASTYADSPFAGTVSF-EKFNAPGKVEWAINWTPS----RDGFIQSYCNTVPTPEGGTHEAGFWAAILKGIKAYGELVSNKKAATITREDLTTGAGALVSCFIREPEFVGQTKDRLATTEAQRMVENSVRDHFDNWLAADTKSAGAILDFLVLRAEERLRRRQEKETARKSATKKLRLPGKLVDCSATNRDGTELFIVEGDSAGGSAKMARDRKTQALLPLRGKILNVLGAASSKLGSNAEISDLTQALGVGLGSKFNIDDLRYDKIIIMTDADVDGAHIASLLMTFFFTQMRPMIDAGHLYLACPPLFRLTQGARRVYCVDEAERDRWMEKGLGGKGKIDVSRFKGLGEMDAKDLKETTMDPKSRKLIRVTIDEDEPGET--GDLVERLMGKKPELRFQYIQENARF--VEELDV-----MSAPKKVVLAYSGGLDTSIILKWLQTEYGCEVVTFTADLGQGEELEPARAKAELLGVK--EIYIEDLREEFVRDFVFPMFRANALYEGEYLLGTSIARPLISKRLVEIAAETGADAVSHGATGKGNDQVRFELAAYALNPDIKVIAPWRLWDLSSRTKLIDFAEKNQIPIAKNKRGEAPFSVDANLLHTSSEGRVLEDPAEQAPDYVFQRTVSPEDAPNEPEIIEVTFEKGDAIAINGQELSPATILTKLNELGGKHGIGRLDFVENRFVGMKSRGIYETPGGTVLLAAHRGIEQITLDSGAGHLKDSIMPRYAELIYNGFWFSPERDMLQALIDKSQEHVSGTVRLKLYKGSASVIGRWSDRSLYSEAHVTFEDDAGAYDQKDAAGFIQLNALRLKLLAARNRRLK-----------------------MSGQSGKGITYAQAGVDIDAGNALVERIKPAAKRTARPGVMSALGGFGALFDLKGAGYSDPILVAATDGVGTKLRIAIDTGLVDGVGIDLVAMCVNDLVCQGAEPLFFLDYFATGKLETDKAARIVEGIAEGCLRSGAALIGGETAEMPGMYPEGDFDLAGFAVGAMERGADLPR--DVAEGDVLLGLASDGVHSNGYSLVRRLVDISGLGWDAPSPFG-EGALGEALLAPTRLYVRPVLDAI-RAGGVHALAHITGGGLTENLPRVLPEGMGADIDLDAWALPPVFGWMAKVGEIEPGEMLKTFNCGIGMVVVCAPDRAEALAQLLSEAGETVCCLGTVGGAAG-IRYTGSLAMYQYNDFDTEFLAQRNAQFRAQVERRIDGSLTEDEFKPLRLMNGVYLQLHAYMLRVAIPYGTLNSAQMRQLALLAEKWDKGYGHFTTRQNIQYNWPKLRDIPDMLDALGEVGLHAIQTSGNTIRNVTSDHFAGATADEVEDPRPLAELLRQWSTDHPEFQFLGRKFKIAVIASEQDRAVIRAHDIGLRIL-RRD-GDTGVQVIVGGGLGRTPMIGKVIAEFLPKADLLPYLEAILSVYNRIGRRDNKYKARIKITVHENGIDTIREEVEKEFIERRKAFS-GVDQKILAQIETMFAAPAFRRAPV--EPFDAAYASDPLFRSWADTNLTAHKAPGYAIVSISLKAHGKTPGDATADQMRVMADLAAEFGHDELRISHEQNVILPHVHKSDLPALYARLRANGLGTANIGLISDIIACPGMDYCALATARSIPIAQDIATRFDELKLEHDIGELKLKISGCINACGHHHVGHIGILGLDRAGVENYQITLGGDATETAAIGERAGPGFSADDIVPAIERLVLGYLDLRASPEETFLQSYRRLGAAPFKAALYP-------EA-----RANAA--MAKTIVVKQIGSPIRRPAKQRATLIGLGLNKMHKTRELEDTPSVRGMVASISHMVEIIEEKE-------MPESE-----DLVAAIETVEAKLAEAKASITRRFIGQEQVVDLTLTALLCGGHALLIGLPGLGKTRLVETLSTVMGLHGNRIQFTPDLMPADILGSEVLDTAADGTRNFRFVQGPIFCELLMADEINRASPRTQSALLQAMQERMVTVAGENRDLGAAFHVLATQNPIEQEGTYPLPEAQLDRFLVQIDVAYPDRDTERAILLATTGVAEDEAVAVFTREELLAAQKLLRRMPVGDSVVEMILDLVRAFRPDEPDASDKVRDTVAWGPGPRAAQALMLTVRARAMLQGRLAPNAEDVLAMARPVLSHRMALNFAARARGDSLSELIDQTARALGKTEAAA-MPKRILQGTVTSDANAQTVTVLVERRFTHPVLKKTIRKSKKYRAHDEANSFKVGDTVRIIECAPKSKTKRWEVLTSETTEA-------------------------MTDQTKD-----DVVARAAQLLKGH-RESIDRLDAILVYTLGERFKHTQAVGKLKAENDLPPSDPAREKQQIARLEDLAIQADLDPEFAKAFLNFIIQEVIHHHQKHQS----MAHANPNIGESR------PVIRQLDDAAINRIAAGEVVERPASAVKELVENAIDAGATRIRIDIADGGKTLIRVTDDGCGIAPDDLPLALARHATSKI----DGT-DLLNIHTFGFRGEALPSLGAVGRLSVTSRAPGHD-AAVIRVSGGRMEPVRPAALSA----GTVVELADLFHATPARLKFMRSDRAETQAIADTVRRLAMAEPAIGFTLRDVSGGGEGRSLFRADAESGDLFDALHGRLSGVIGRDFADNALRIDATRDGLRLFGYAALPTYSRGAAVTQFLFVNGRPVRDKMLYGALRAAYFDFLSRDRHPAAALFIDCDPTLVDVNVHPAKSEVRFRDPGTARGLIVSGLRHALAEAGHRASTTVAGATLGAM--RP-------------------E---PTG-A---RVYQMD-----R------------PSPAARGLG--YAAQRPAEPAPQ------GFDELNAGFS----ARVV-EPEPMPEV----------------T--PAEHLPLGAARGQVHENYIIAQTETGMVIVDQHAAHERLVYEKLKRQMAENGVAAQALLIPEIVELSASDRDSLLDIAPDLVALGLTIEAFGGTAVAVRETPAILGEVNAAALVADILDELADAGSATTLQARIEAILSRVACHGSIRSGRRMRAEEMNALLREMEATPHSGQCNHGRPTYVELRLSDIERLFGRT-MNALQLKKGATSHSAYNLRPSFSEVEERHTLAVLVENEPGVLARVIGLFSGRGYNIESLTVAEVDHTGHLSRITIVTKGTPQVIEQIKAQLGRIVVVHDVHDLTVEGASVERELAMFKVIGDGDKRVEALRLADIFRANVVDSTLNSFIFEITGAPEKIDAFADLMRPLGLAEVARTGVAALSRGD---------------------MPSYTAPTKDMQFILHEMLKVSQ-SDVPGYDELEPEFTSAVLEEAGKISSEVLHPLNVVGDTQGC-TLENGVVRTPEGFKAAFDQMRDGGWPGLDMPEEFGGQNMPYVLNTAVGEMFSSSNQAFTMYQGLTHGAASAILAHGSDQQKATYLPKMVSCEWTGTMNLTESHCGTDLGLMRTKADPQDDGSYKITGQKIFISSGEHDMADNIIHLVLAKIAGGPEGIKGVSLFIVPKFLVNEDGSLGARNGVACGNIEEKMGIHGNSTCVMNYDGATGYLLGEEHKGMRAMFTMMNEARLGVGMQGLAQAEIAYQNALFYAKDRLQGRDVTGAKNPDGPADPLIVHPDIRRSLMDQKSFAEGARAFILWGASMIDSAHRKGDKDAD----GLVSLLTPVIKGFLTDQGYDMTVQAQQVYGGHGYIEEWGMSQFTRDARIAMIYEGANGVQALDLVGRKLAQDGGKHVMAFFEMVKSFAKENAGKDEAFDRDFLEPLKSASKDLQAAGMYFMQQGVKNPNNALAGSYDFMHMFGHVCLGLVWAQMALAARAALDAGTQDPTFYETKLATGRYYMARRLPATKMHLARIESGADPVMTLDAANF----------MSFDPK------------TLRFDSAGLIPAIAQDDETGEVLMMAWMNAQSLARTVDTGRVTYWSRSRQAFWAKGESSGHVQTLVDLRVDCDRDCLLLRVRQVGPACHTNRRSCFYTAVRDGQEVELMGPMKDT----MAKPTTIKIRLNSTAGTGHFYVTKKNARTMTEKMSVRKYDPVARKHVEYKEGKIK-------------MGNARNIAFWVVLFLLVLALFNLFSGSGGT--LQSREITYSDFVTAVKSGEVGQATLDGEQVRFRGTDNQDYVT-IKPEDA--EITSLLIDNNVPIRAEKQQQSM-FQSFLITLLPFLLLIGVWVYFMNRMQGGGKGGAMGFGKSKAKMLTEKHGRVTFDDVAGIDEAKEELEEIVEFLRNPQKFSRLGGKIPKGALLVGPPGTGKTLLARAIAGEAGVPFFTISGSDFVEMFVGVGASRVRDMFEQAKKNAPCIVFIDEIDAVGRHRGAGYGGGNDEREQTLNQLLVEMDGFEANEGVIIIAATNRKDVLDPALLRPGRFDRQVTVGNPDIKGREKILAVHARKTPLSPDVDLRVIARGTPGFSGADLANLVNEAALMAARLGRRFVAMVDFENAKDKVMMGAERRSMVLTSEQKEHTAYHEAGHAIVGLELPKCDPVYKATIIPRGGALGMVVSLPEIDRLNWHRDECTQKLAMTMAGKAAEVIKWGEDAVSNGPSGDIQQASGLARAMVMRWGMSDKVGNIDYQQAHEGYM--GNGAGGFSVSAHTKELIEEEVKRLVDEAYAWARQILTDKHDEWERLAQGLLEYETLTGEEIKRVIRGEPPHADDGEDDSA-DAGNAPSVTAIPKT--KPRKTPP-EG-G-MEPEPSAMAKITYIEHNGTRHEVDVPNGLTVMEGARDNNIPGIEADCGGACACSTCHVYVDPAWVEKLPAKEDMEEDMLDFAFEPDSARSRLTCQLRVSDDLDGLVVQMPEKQIMSRVCELTGKGPMTGNNVSHANNKTRRRFLPNLNDVTLQSEALGRGIKMRISAAALRSVDHRGGLDKFLAKAKDTELSARVLKVKKEIQKSGGNAVEAAVSE----MTRRAVVKGVGHYLPERVVLNSEFEATLDTSDEWIRSRSGIERRHFAAEGETTSALATHAARNALADAGIEADSIDAIILATSTADMTFPSAATMVQAALGMTRGFAFDVQAVCAGFVYALANANAMIASGQADRVLVIGAETFSRIMDWTDRSTCVLFGDGAGALVLEATEGAGTSDDRGILATDLNSDGRYRDLLYVDGGV-ATQNTGFLRMQGNQVFRHAVEKLAKTAETALERAGLTNADVDWIVPHQANIRIIQGTAKKMGVPMDRVVVTVQDHGNTSAASIPLALSVAKGRGQIKPGDLLVTEAIGGGLAWGAVVLRW--MPIADQPQQTERLGADAPGLARAAELLRAGQLVALPTETVYGLGADARNGAAVAAIYAAKGRPRFNPLIVHLASSDAAEPYVEWTDTARDIARRFWPGPLTLVLPLSEGHGLSSLVTAGLPTVALRVPAHPTARALLAQAGIPVAAPSANPSGRISPTNADHVLA----GLDGAIAAVLDDGPCSVGLESTIL--SLAGA--PTLLRPGGLPAQEIEAALGQPLLRPETGGP--ITAPGQLTSHYAPGAPVRLNAGSARPGELFLGFGT-CSGTGPDDLNLSPRGDLAEAAAHLFDHLHRLDRLGK-----P-----IAVAPVPATGLGLAINDRLTRAAAPRG-MSRVKGGTVTHARHKKVIKAAKGYYGRRKNTFKVAAQAVDKANQYATRDRKNRKRNFRALWIQRINAAVRSHDEALTYSRFINGLSLAGIEVDRKVLADLAVHEPEAFGAIVEQAKGALAA----MSQKIAFQGEPGAYSHEACREARPDMEPLPCRTFEDAIEAVRSGEADLAMLPVENSTYGRVADIHSMLPQSGLHIIDEAFVRVRIALMALPGTQLADITHAMSHTVLLGQCKTFLKDNGLQRITGADTAGSAKHVAEEGNPKIAALAAELAGEIYGLDVLARDIEDNKHNTTRFLIMSREADRTRRGANGMITSFVFQVRNIPAALYKAMGGFATNGVNMTKLESYMVDGSFTATQFYADIQGHPDDANVKLALDELGYFTTELEILGVYPAAKPR--------MTTNPPDLRPDLAPRAKIG---DTPRPG-QPTIGMVSLGCPKALVDSERILTRLRAEGYGISPDYKGADAVIVNTCGFLDSAKAESLDAIGEALSENGRVIVTGCLGAEPEYITGAHPKVLAVTGPHQYEQVLDAVHGAVPPAPDPFVDLLPASGVSLTPRHYSYLKISEGCNHRCKFCIIPDMRGRLVSRPAHAVVREAERLVEAGVKELLVISQDTSAYGADRKHD----TERGIRTH-IDDLARELGGLGA----WVRLHYVYPYPRVRQLIPMMAEGL--ILPYLDIPFQHAHPETLKRMARPAAGIRTLDEIAAWRDICPDITLRSTFIVGYPGETEEEFQTLLDWLDEAQLDRVGCFQYENVAGARSADLPDHVAEDVKQDRWDRFMAKAQAISEAKLAQKVGQRIEVIVDEVDAEA-ATC-RTKGDAPEIDGNLFIDEGFEALSPGDIVTVEVDEAGEYDLWGRLV---------MSIDESTAARVAKLARIRVEPEALPELAEDFNRILGFIEQLNEVDVEGVEPMTSVTPMRLKRREDQVDDGGHAAQVLSNAPDAREGFFAVPKVVE---MRLGDITRKTAETEITVQINLDGTGTYDNQTGIGFFDHMLDQLARHALIDMTVRAKGDLHIDDHHTVEDTGIAIGQALSQALGDKRGIRRYGECRLPMDDAQVACALDLSGRPFLVWNIDLPTQKIG-TFDTELVREFFQALSTHGGITLHLDLLHGFNSHHIAEATFKATARALRAAVAQDPRAAQAVPSTKGSLMSRRHAAEKREVLPDAKYGDIVLTKFMNNLMVDGKKSVAERIVYSAMDRVEGKIKRSPLEVFHEALDNIQPSVEVRSRRVGGATYQVPVEVRPERRQALAIRWLINASRARNENTMEERLAGELLDAVQSRGAAVKKREDTHKMADANKAFSHYRWMAAYQYVYHMSGVSKAYPGGKKCFENINLNFLPGVKIGVVGVNGAGKSTLMKIMAGMDKDFTGEAWSAEGARVGYLPQEPQLDENLSVRDNVMLGVKAKKDILDRYNELAM----NYSDETADEMAALQDRIDAENLWDLDSQVDVSLEALRCPPDEANVATLSGGERRRVALCKLLLEAPDMLLLDEPTNHLDAETIAWLQQHLIEYKGTILIVTHDRYFLDSITGWILELDRGRGIPYEGNYSAWVEQKAKRLEQEAREDKSKQKTLERELQWMRQGAKARQAKSKARIQAYEKLADQSMREKIGKAQIIIPPGQRLGGKVIEVENLGKAMGDKLLIENLSFSVPPGAIVGVIGPNGAGKSTLFKMITGLEKPDSGTIEIGNTVDLSYVDQSRDDLNPGDNVWEAISGGAEIIELGDAQINSRAYCGAFNFKGSDQQKKVSLLSGGERNRVHMARLLKSGGNVLLLDEPTNDLDVETLRALEDALSDFAGCAVVISHDRFFLDRICTHILAFEGEAHVEWFEGNFEDYEEDKKRRLGADALEPKRLKHKKFSR--MILYPAIDLKDGQAVRLLRGDMEKATVFNDDPTAPARGFVEAGCQWLHLVDLNGAFAGTPVNAAAVEAILAACP-VPAQLGGGIRDMATIESWISKGLARVILGTVAVENPDLVRDAARAFPGKIAVGIDARDGRVATKGWAEETDVQATDLARSFEDAGVAAIIYTDINRDGAMQGPNIESTAALAQSVSIPVIASGGVSSLDDLIALRDCGTQLDGAISGRALYEGAIDLKAALAALS---------MSRYSAADIEPKWQAAWDKLGVFTAKRDES---RPKYYVLEMFPYPSGRIHMGHVRNYTMGDVVARYKMSTGHNVLHPMGWDAFGMPAENAAMAIGGHPKDWTYNNIADMRAQMKPLGLSIDWSREFATCDPEYYGQQQALFLDMLDAGLVTRKNAVVNWDPVDMTVLANEQVIDGKGWRSGADVERRELTQWFFKISDMAEELLEALDGLDNWPAKVRLMQENWIGKSRGLEMEFTLSA-------PAHGH-DTLKIYTTRPDTLAGASFMAISPDHPLSKALESDNADLAAFNADCRRMDTTEAAMEKAEKRGFDTGLTVRHPLDDSRTLPIWVANFVLMDYGTGAIFGCPAHDQRDLDFARKYDLPVIDTFVAM--D----SDARVENTAFT-PPKTEKVRWIDQPAGV-TEATGQEAIDATIDWAEARGLGQGKTQFRLRDWGLSRQRYWGCPIPVVHCADCGVVPEKKENLPVQLPDD------VTFYIPGNPLDRHPTWRDCACPSCGKPAKRETDTMDTFVDSSWYFARFTSPHAATPTEKADAEYWMNVDQYIGGIEHAILHLLYSRFFARAMNVTGHLPAKSREPFDALFTQGMVTHEIYQT--RDA-------KGRP---------------------VYHLPEDV--------------------SDGK--L---ADGTE------------------VEIIPSAKMSKSKKNVVDPVSIISAYGADTARWFVLSDSPPERDVEWTAAGAEAAHKFLTRVWRMARELAD-QRDAGVGDLDKSLQDLMDSMQLGMNPLDDELMRATHKAIDDVTKGVESFGFNAAIARIYTYVNALSKSQ---ASARMKWTSMRILAQLMSPMTPHLAEEMWALLGGEGLVVETPWPTPDDNMLRDNTVTMPIQINGKRRGEISVPVDMSKEDVEKQALASDPVQKALAGGTPRKVIVVPGRIVNVVI--MAQYDVIIIGSGPGGYVCAIRCAQLGLKTACVEGRDTLGGTCLNVGCIPSKALLHASHMLHEAEHNFATMGLKGKSPSVDWKQMLSYKDDTIGQNTKGIEFLFKKNKVDWIKGWASIPEAGKVKVGD-----DVHEAKNIIIASGSEPSSLPGVEVDEKVVVTSTGALELPKIPKKMVVIGAGVIGLELGSVYARLGAEVTVIEYLDAITPGMDGEVQKTFQRTLKKQGMTFVMGAAVQKTEASKTKAKVTYTLKKDDSTHEIDADTVLVATGRKPYTEGLGLDALGVDLSKRGQIQVGKDWQTSVKGIYAIGDCIDGPMLAHKAEDEGMAVASVIAGKHGHVNYNVIPGVIYTWPEVANVGKTEEALKEEGRAYKVGKFSFMGNGRAKAMFAAEGFVKILADKDTDRVLGAHIIGPNAGELIHEICVAMEFGASAEDIALTCHAHPTCSEAVREAALACGDGPIHS--MKAALIQLCSSDDPAANLAMVQDRVAQAAAQGAGFVLTPEVTNCLSTSRKHQQEVLKLEEEDQTLPALRDQARDLGIWLLIGSLGLKTGD-ADGRFANRSFLIGPDGAVAAWYDKIHMFDVDVTPEETYRESDGYRPGTRAVVQDAGFATIGMSVCYDVRFPALYRALAQAGAQVITVPAAFSPVTGAAHWHSLLRARAIETGCYVLAPAQCGTHPSSGSKQRSTYGHSLAVSPWGEVLVDGG-TEPGIHFVDIDVKTVVEARRKVPSLSHGRPFDGP-----MT---------SKRDEWTKRAEAELR---GRPLDSLTWNTLEGIDVQPLYTAEDLERADHLGSLPGEAPFTRGVKATMYAGRPWTIRQYAGFSTAEESNAFYRRNLAAGQQGVSVAFDLATHRGYDSDHPRVTGDVGKAGVAIDSVEDMKILFDGIPLDTVSVSMTMNGAVIPVLASFIVTGEEQGHDKSVLSGTIQNDILKEFMVRNTYIYPPEHSMRIIADIIEYTSNEMPKFNSISISGYHMQEAGANLVQELAYTLADGKEYVKAAMSAGMDVDKFAGRLSFFFCIGMNFFMEAAKLRAARLLWYRIMDE-VGAKNERSKMLRTHCQTSGVSLQEQDPYNNVIRTAYEAMSAVLGGTQSLHTNALDEAIALPTDFSARIARNTQLILQEETGVTNVVDPLAGSYYVESLTKELADKAWALIQEVEEMGGMTKAVASGMPKLRIEETAARRQAMIDRGDEVIVGVNKYRKDKEDPIDILDVDNVKVRDSQIARLEKIRASRDESACQAALDELTR-RAGEGGNLLEAAVEAARARASVGEISMAMEKVFGRHRAEVKTLAGVYGAAYEGDEGFAAIQKSVEDFAEDEGRRPRMLVVKMGQDGHDRGAKVIATAFADIGFDVDVGPLFQTPEEAAQDAVDNDVHVIGISSQAAGHKTLAPQLVEALKAADAEDIIVICGGVIPQQDYDYLYKHGVKAIFGPGTNIPKAAEDILRLI----REARG---MRVYYDRDCDINLIKDKKVAILGYGSQGHAHALNLRDSGAKNLVVALREGSASAKKAEAEGLKVMGIAEAAAWCDLIMFTMPDELQAETYKKYVHDNLREGAAIAFAHGLNVHFGLIEPKAGVDVIMMAPKGPGHTVRGEYTKGGGVPCLVAVHNDASGKALELGLSYCSAIGGGRSGIIETNFREECETDLFGEQAVLCGGLVELIRMGFETLVEAGYAPEMAYFECLHEVKLIVDLIYEGGIANMNYSISNTAEYGEYVSGPRILPYDETKAKMKAVLRDIQTGKFVRDFMQENAAGQPYFKGTRRLNDEHQIEQVGEKLRAMMPWISAGKMVDKQKN---MYDVNQIRAQFPILAREVNGQPLTYLDNGASAQKPQVVIDAITQAYAMEYANVHRGLHFLSNLATEKYEAVRGTVARFLNAADENEIVLNSGTTEGINMVAYGWAMPRFEPGDEIILSVMEHHANIVPWHFLRERQGVVLKWVDVDATGALDPQAVLDAITPRTKLIAVTHMSNVLGTLVDVKAICHGARERGVPVLVDGSQGAVHLPVDVEDIGCDFYAITGHKLYGPSGSGAIHVRKERLAEMRPFMGGGDMIREVSKDAVTYADPPMKFEAGTPGIVQTIGLGVALEFLMDLGMENVAAHEAMLRDYTVEKLAGLNWLQVQGTTPDKGAIFSFTLDGAAHAHDISTILDKKGVAVRAGHHCAGPLMDHLGVSATCRASFGLYNTTEEVDRLVSALELAHELFA---MSSNRDIDTLIDLMARLPGLGPRSARRAVLHLIRKRGLLLSPLADSIARVAATARECVNCGNVGTADVCDICTDETRANGEICVVEDVADLWAMERAGVFKGRYHVLGGTLSAMDAIGPEELRIPRLLDRIGEETVGEVILALNATIDGMTTAHYIADQLGD-GIRLTSLAQGVPIGGELDYLDDGTISAALKARRVLMRAETQNTVAEIEKSLALLAQRMDRETAPHRLEEFNARVEDPNLWDNPDAAQKLMRDRQMLVDAMDTYDGIKQDLTDNLDLIELGEMEDDTEVVKEAEAALKTLAERAAQKELEALLDGEADGNDTFLEINAGAGGTESCDWASMLARMYVRWAEKKGYKVELQSESAGEEAGIKSATYKISGHNAYGWLKSESGVHRLVRISPYDSAAKRHTSFSSVWVYPVVDDNIEIEVNPADIRIDTYRSSGAGGQHVNTTDSAVRITHHPTGIVVTS-SEKSQHQNRDIAMKALKSRLYQMELDRRNAAINEAHENKGDAGWGNQIRSYVLQPYQMVKDLRTNHETSDTKGVLDGDLDGFMAATLALDVSGKSRAEAQG-G---MSD-PALLLDGICKSYNRGTPAEVRVLQGASLTLRKGEAVALVAPSGAGKSTLLHIAGLLDTADAGRVVVGGTDMSAAGDAARTAMRRQDIGFIYQFHHLLPEFSAVENVVLPQRANGVRGSEAEARALTLLKMVGVDHRAGHRPAALSGGEQQRVAFCRAMANAPGVLLADEPTGNLDPATSDQVFEALMRLVRENGMAALVATHNPALAARMDRVIHLAEGQLRDGPFSPAA-------MNQMTSVEKTV----STEA----NKITAEGVQVYYGDTHAIKDVNVQIEDKTVTAFIGPSGCGKSTFLRCLNRMNDTIDICRVEGQILLDGENIYDRKVDPVQLRAKVGMVFQKPNPFPKSIYDNVAYGPRIHGLARNKAELDEIVEKALRRGAIWNEVKDRLHAPGTGLSGGQQQRLCIARAVATEPEVLLMDEPCSALDPIATAQVEELIDELRQNYSVVIVTHSMQQAARVSQKTAFFHLGNLVEFGETGQIFTNPTDPRTESYITGRIG-MYRIAALYHFTRFADPAALKPSLEAVCRANDVRGTLLLAGEGINGTIAGP-AQGIDAVLAHIRALPGCAGLEWKEAQAPKPPFAKLKIRLKREIVTMGQPDIDPVARVGHYVEPGDWNELIRRDDVVVIDTRNDYEVAIGTFQGAIDPETKSFGELPAWWEANKTRF----HNKKVAMFCTGGIRCEKSTNYLLGQGVDEVFHLKGGILNYLEKVPERDSAWEGACFVFDNRVAVEHGLKESGHILCHGCRRPIEPADRTRPEYEQGVSCHLCIDETSDEDKTRFRQRQKQIALARERGELHIPLQD--------MNILILGSGGREHALAWAVMQNPKCDKLIVAPGNAGIAGIADCASLDVNDGGAVVEFAGENSIDLVIVGPEAPLAAGVADDLRAAGVLVFGPSAAAARLEASKSFTKEICDAAGAPTAAYGHFTDAEAARAHVRSNGAPIVVKADGLAAGKGVIVAITEDEALAAIDDMFGGEFGSAGAEVVIEEFMEGEEASFFVLCDGDTVLPVGTAQDHKRIGEGDTGPNTGGMGAYSPAPVMTDDVVARAMDEIVRPTVAEMARRGTPYQGVLYVGLMIKDGAPRLVEYNVRFGDPECQVLMMRLGAQALDLMHAAAEGRLSEMQVNWA-----DD--HAMTVVLAAKGYPGSYVKGTEIKGLEALPETSTQMCFHAGTTR-QD-----GRTLATGGRVLNVTARGATLAEARARAYAMADAIDWPEGVFRRDIGWRALG-----MYDPVDTYMNTLVPMVVEQTSRGERAYDIFSRLLKERIVFINGPIHDGMSHLVVAQLLHLEAENPSKEISLYINSPGGVVTSGLSIYDTMQYIRPKVSTLCIGQAASMGSVLLAGGTPGMRFSLPNSRIMVHQPSGGYQGQASDIMIHAAETQKLKDRLYDIYVKHTGQTKKAVEKALDRDNFMSPEEAKDWGHIDEIVENREKAEPSAG------------MKARVHVMLKTGVLDPQGEAVRHALGALGFEGVNGVRQGKVIELDLA----EGATEDQVKDMCEKLLANTVIESYSVELV--MPELPEVETVRRGLAPAMEGAVIARAEVNRPDLRWPFPPRMADRLSGQRVERLRRRSKYILADLTSGESLLIHLGMSGRMTVSGDPL---------GQFVHEHPMPEKHDHVVFHMSNGARITFNDPRRFGAMDLLPTATAEAHKLLASIGPEPLGNAFSEAYLVEMLKGRNTPIKSALLDQRIVAGLGNIYVCEALYRARIAPSRKAGRIAAIRVAGLVPIIRDVLTEAIEAGGSSLKDYRRTDGELGYFQHSFDVYGRESEPCRTPGCTAHIARIVQSGRSSFYCPQCQR------MTKLAIIGGSGIYDIDGLEDAQWISVDTPWGAPSDQLLTGRLSGLDMVFLPRHGRGHVHSPSTVPYRANIDALKRLGVTDVVSVSAVGSFREKMAPGDFVLVDQFIDRTVHRESSFFGTGCVAHVSLAHPTCARLSAACLAAALDADITVHDGGTYLAMEGPQFSTLAESRMFRESWGADVIGMTNMPEAKLAREAELCYASVAMVTDYDSWHPDHGEVDVTRIIEILTGNAGKARAMVARLPAALAARDGEARPPCEHGCDRALEYALLTAPDKRAPEVMARLDAVAGRVL------MTQYLDFEKPLAEIEGKAEELRALGRANAEMDVESEAKALDAKAVQLLNDLYKDLTPWRKCQVARHPERPHCQDYIAALFTEFTPLAGDRNFADDHAVMGGLARFEDQPVMVIGHEKGNDTKSRIDRNFGMARPEGYRKAIRLMEMAGKFGLPVITLVDTPGAYPGKGAEERGQSEAIARSTEKCLQIGVPLVSVIVGEGGSGGAVAFATANRVAMLEHSVYSVITPEGCASILWKDSEKMREAAEAMRMTADELKALGVIDRIIPEPRGGAHRDKAATIAAVGKSISAMLKELAKKDGGTLVNDRRQKFLNMGAKGLAAMILGIGTDLANIDRIAGTLDRFGDRFRNRVFTDIEQAKAERRADTPGTYAKRWAAKEACSKALGTGL-RMGIAWKDMAVSNLRTGQPVMHMTGWAKERLDSMTPPGHEAIIHVTLTDDHPWAQAFVVIEARPKAPDTPET---------MPALCRDCLSTCDAAR-------------RCPSCGSPRMVANAELFDLSIAHMDCDAFYASVEKRDDPSLADKPVIIGGGKRGVVSTACYIARIRGVRSAMPMFQALKLCPEAVIIKPRMDVYVEVSRQIRAMMEDLTPAIQPLSLDEAFMDLGGTARLHGAPPAVMLARLTKRMKSELGLTGSIGLSHNKFLAKVASDLEKPRGFSVIGKAETGAFLHDKPVRLIWGIGPSAQESLDRAGIRTFSDLLRWDRLALSGRFGSMGDRLWHLARGEDKRRVSAHEPVKSISNETTFHEDTGDPDILDGHIWRLAEKVSDRAKARGLAGRVVTLKLKRANHTTLTRRVTLREPTQIADTIYRTARAVFDQSA-QQAPFRLIGTGLSDLCS-ASEANFSGDLLDPQAARRAQAERAADTLRGRFGASAIVKGRALR----------------MTKFSDLNLNPKVLKAVEEAGYETPTPIQAGAIPPALEGRDVLGIAQTGTGKTASFTLPMITALARGRARARMPRSLVLCPTRELAAQVAENFDTYAKHVKLTKALLIGGVSFGEQDRLIDKGVDVLIATPGRLLDHFERGKLILSDVKIMVVDEADRMLDMGFIPDIERIFGLTPFTRQTLFFSATMAPEIERITNTFLSAPERIEVARQATTSETITQGVVLFKGSRKDREASEKRAVLRALIDGEGDKLTNGIIFCNRKTDVDIVAKSLKKYGYDAAPIHGDLDQSQRTRTLDGFRNGALKILVASDVAARGLDVPSVSHVFNFDVPSHAEDYVHRIGRTGRAGREGTAMMICVPRDEKNLADVEHLVQREIPRL--ENPLK--SSAPTEEADTASK------------------------DEPRKSSRSRSSSSRRSAARDE----------------------VADETPRAEAAKAE----------PAKSEPAKK-------------------------------DNPSRSD--------------DRGRSRK----------------SSDRRDQDVVGLGDHMPSFIALSFAERRAG--MTDIQPIRRALLSVSDKTGLIDLGRALADRGVELLSTGGTAKALREAGLEVRDVSDVTGFPEMMDGRVKTLHPMVHGGLLALRDNDDHRAAMETHGIGAIDLLVVNLYPFEETVAKGADYDTCIENIDIGGPAMIRAAAKNHAFVNVVVDVADYEALLAELDANDGGTALAFRQRLAQIAYARTGAYDAAVSTWMAGALDDSAPRRRAVAGTLAQSLRYGENPHQAAAFYTDGSARPGVATATQHQGKELSYNNINDTDAAFELVSEFAAGDGPAVAIIKHANPCGVARADTLIDAYRRAFDCDRTSAFGGIVALNGPLDGATAEEIAGIFTEVVIAPEADDDARAVFAAKKNLRLLTTGALADPRAAALTIRQVSGGYLMQDKDTGFVTEADLKVVTKRAPDAREMADLLFAWKVAKHVKSNAIVYARDGATVGVGAGQMSRVDSANIAAIKAQRMADVLGLPESPAKGAVVASDAFFPFADGLMEAAAAGATAVIQPGGSMRDAEVIAAADEAGLAMVFTGLRHFRHMNAKELRDKTPDQLREELANLKKESFNLRFQQATGQLENAARIKVARRNAARVLTVLNEKAASAAASE-MLTKKQLDLLEFIHKRVQRDGVPPSFDEMKAALDLRSKSGIHRLITALEERGFIRRLAHRARAIEIVRLPDSMNGTA--G----FQPRVIDGDKPDVS---RPANAAPVVAS-AIELPVMGRIAAGVPIEAISHVSHQVAVPGAMVSSKGEHYALEVKGDSMIDAGINDGDVVVIRETSVADNGDIVVALVEDAEATLKRFYRRGNAIALEAANPAYETRVLPEDKVKVQGKLVGLIRTY-----MSSFDDIDAFEAAS---LSARAMAARPA-PYLDGLNPAQRDAVEHLDGPVLMLAGAGTGKTRALTARIVHLLSVGKARPNEILAVTFTNKAAREMKNRVGDMLGQSIEGMPWLGTFHAICVKLLRRHAELA---------------------G-------------LKSNFTILDTDDQIRLLKQLIQAEGIDDKRWPARMLANIIDGWKNRALTPDKVPTADAGAY-NHRGAELYAQYQTRLRELNAVDFGDLLLHMVTIFQTHADVLEQYQRWFRYILVDEYQDTNVAQYLWLRLLASAHKNICCVGDDDQSIYGWRGAEVGNILRFEKDFPGAHVVRLEQNYRSTPHILAAASGVIRGNQGRLGKELW-------TEAEEGEKLRLIGHWDGEEEARWIGEEIEAMQRGTRGM-----------------------------------------------------------------------RPMGLDQMAILVRASHQMRAFEDRFLTIGLPYRVIGGPRFYERLEIRDAMAYFRVVTSPDDDLAFERIVNTPKRGLGDKAQQVIQRVARANGVNLVEGARMAVDAGEIKGKGGGELRRLIESIDRWSAMARGPQRVLDRDDDSVLDD--------GPTPME--PGGLDHLELAETILDESGYTAMWQNDKTPEAPGRLENLKELIKALESFDNLQGFLEHVSLIMDNEQADAEQ-KVTIMTLHAAKGLEFPAVFLPGWEDGLFPSQRSMDESGL---------KGLEEERRLAYVGITRAEEICTISFAGNRRVFGQ----WQSALPSRFIDELPEQHVEVLTPPGLYGGGYGAAG-----------SVSGGMQQRMADADGYNSPGWKRLQSRAGQRGTAQPSEVRSTVIDMTAVSAFAMGDRVFHQKFGYGAVTGIEGDKLEVDFEKAGVKKVVARFVT---SSDDVPF----MSH-PPRAWQRMLSGRRLDLLDPTPMDIEVEDIAHGLAFVARWNGQTRGDFAYSVAEHSLLVETLFRRIVPK-APVKWQLAALLHDAPEYVIGDMISPVKAAVGPGYGTLDDRLTAAIHIRFGLPAAIPKTVKQQIKRADRLSAWMEATQIAGFSEAEANKFFGRPDPALIDGLSILLRP--PVQVRADFVARHAELLAALR-----------MTFAHRHLLGIEHLAPSDITAILDLADTYADLNRQSDKHGTALAGLTQINMFFENSTRTQASFELAGMRLGADVMNMSMQTSSVKKGETLIDTAMTLNAMHPDLLVVRHPHSGAVDLLAQKVNCAVLNAGDGRHEHPTQALLDALTIRRAKGRLHRLNIAICGDIAHSRVARSNILLLGKMENRIRLIGPPTLMPAGIADFGVEVY-QDMAAGLEGVDVVMMLRLQKERMDGGFIPSEREYYHRYGLDAEKLACAREDAIVMHPGPMNRGVEIDGTLADDINRSVIQDQVEMGVAVRMAAMDLLARNLRNRAA----A-------MANSKRQLFLKRRLRVRNKLRKV--NAGRVRLTVHRSNKNISVQLIDDVKGVTLASASSLEKTLGIVGK----NNIEAATKVGSAIAERAKQAGVEEAYFDRGGFLYHGKVKALADAAREGGLKI----------MAKQLNYHTIREIFTRFHAAE---------PEPKGELDHVNAYTLVVAVALSAQATDAGVNRATAELFKIADTPQKMLDLGLEGLTEHIKTIGLFRQKAKNVIKLSQILVDDYGGEVPNSRAALQSLPGVGRKTANVVLNMWWQQPSQAVDTHIFRLGNRSGIAPGKTVEAVERAIEDNIPADFQLHAHHWMILHGRYTCVARKPKCPACLIKDLCQFEEKTL------------------------------MAIDKPATR--------PANPRFSSGPCAKPPAFALEKLAHAPLGRSHRAAVGKDKLKAAIETTRDILGVPADYRIGIVPASDTGAFEMAMWNLLGERPAEMLAWESFGAGWVTDVVKQLKI-EAEVKTAEYGEIVDLAGVDFDKDVCFTWNGTTSGVRVANGDAIPADRAGLTLCDATSAAFAMDLPWDKLDVTTFSWQKVLGGEAAHGMLILSPRAVERLESYTPAWPLPKIFRLTKGGKLIEGVFNGETINTPSMLAVEDYLLALDWARDVGGLKGLIARADANTAAVSDFVAAHDWIDFLATDPATRSNTSVCLKFTDARIA--DGAA---FAKAVAKRMEVENAALDVGAYRDAPPGLRIWCGSTVETADIQAMLPWLEWAFNAEIEAQKAAA--------MAKTNSGRFFEDYSLGQTLVHAVPRTVKMGERALYHALYPARHALYSSDAFAQACGLPFSPLDDMIGFHVVFGKTVPDVSLNAVANLGYAEGRWLAPVWPGDTLRSESEVIGLKENSNGKTGVVYVRTRGLNQHDEPVLEYVRWVMVRKGNVDAPAPEPVVPDLAKVVAPGDLV-IPKGLDFTGYNFALAGEPHRWGDYEVGETIDHVDGVTIEEAEHMLATRLWQNTAKVHFDATFRPD---GQRLIYGGHVISMARALSFNGLANAQMVVGLNGGAHANPCFSGDTVKAWSEVLDKAETDAPGVGALRLRLVATKGGEP--FRLRGDDGKYLPEVLLDLDYWALVPL-----MTKSKKLEFRPNDYVVYPAHGVGQIVSIEEQEVAGLSLELFVISFEKDKMTLRVPTHKATEIGMRSLSSPDVISQAMKTLKGKAKVKRAMWSRRAQEYEQKINSGDLISIAEVVRDLHRTDDQREQSYSERQLYEAALERLTREVAAVGGGDEVVAAKQVDDVLMSR---AA-MLKDQDRIFTNLYGMHDRTLKGAQSRGHWDGTAGIIEKGRDWIIQQMKDSGLRGRGGAGFPTGLKWSFMPKGSDGRPHYLVVNADESEPATCKDREIMRHDPHTLIEGCLIASFAMGAHAAYIYIRGEYIREKEALQAAIDECYDAGLLGRNAAKSGWDFDLYLHHGAGAYICGEETALLESLEGKKGMPRMKPPFPAGAGLYGCPTTVNNVESIAVAPTILRRGPEWFSSFGRPNNVGTKLFALTGHVNNPCVFEEQMSLPVRELIEKHGGGVRGGWDNLKAIIPGGASCPILPKDAL-EDAIMDFDWMR-ENKSSFGTGCMIVMDQNTDVIKAVWRLSKFFKHESCGQCTPCREGTGWMMRVMDRLVTGDAEVEEIDMLLDVTKQVEGHTICALGDAAAWPIQGLIRHFRNEIEDRIKHKRTGR-----VSAVAAEMAEYFA--YTDGACSGNPGPGGWGVLLQAKQGDSVVKERELNGGEPQTTNNRMELLAAINALETLGRATEITVVTDSNYVKNGITGWIHGWKRNGWKNAAKKPVANAELWQRLDAAQARHKVTWKWVKGHAGHPENERADELARKGMKPFKS------------MPVFLDAAAPDFETRFAALLGAKREDSPDVDAAVAKIIADVRARGDQAVIDLTARFDRIELTPATMAFSPEEIAAAVEAVSPEDRAALEMAAERIRAYHVRQMPADQMWTDPDGATLGWRWSAVSAAGLYVPGGLASYPSSVLMNAIPAKVAGVGRLAITVPTPDGVVNPLVLLAAQLAGVDEVYRIGGAQAIAALAYGTDTIAPVDKITGPGNAYVAAAKRRVFGKVGIDMIAGPSEILVIADADNDPDWIALDLLSQAEHDESAQSILIADDAEFGQRVAAAVDKRLETLERRAIAGASWRDFGAVITVSDMAQAAALSNRIAPEHLELCVADPVALAEHTVHAGAIFLGHWTPEAIGDYVGGPNHVLPTARSARFSSGLSVMDFLKRTTMAQMTPEALRAIGPAAERLATSESLEAHGLSVRARLDHLNR-------MTDYPS---SFDKDDLLKCARGELFGPGNAQLPEPPMLMMDRITEISADGGASGKGHVVAEFDITPDLWFFKCHFPGNPIMPGCLGLDGLWQLTGFNLGWRGMKGRGMAMGVGEVKLKGMVRPDRKMLTYYVDFTRIID-RKLKLGVADGRVVADGEEIYSVKDMKVGLATSDD--MSNAQLETAIEAAWDARDTITPGTTGETREAIESTLNALDSGTLRVAERQDNGDWHVNQWAKKAVLLGFRLKDMEAQSGGPQGGGWWDKVDSKFKGWGDDQWKQAGFRAVPNCVVRKSAYVAPGVVLMPSFVNLGAYVDSGTMVDTWATVGSCAQIGKNVHLSGGVGIGGVLEPMQAGPTIIEDNCFIGARSEVVEGVIVREGSVLGMGVFIGQSTKILDRESGEVFYGEVPPYSVVVAGSMP-SKNGVHLYCAVIVKRVDEKTRSKTGVNELLRDMADFFTTTYLGIA-LLTLGQILLLVLPLMVALAFLMYADRKIWAAVQLRRGPNVVGVFGLLQSFADLLKYVVKEVVFPAGADKAVFLLAPMVSLVMALIAWAVIPFNDG------WVISDINVAILYVMAISSLEVYGVIMGGWASNSKYPFLGSLRSAAQMISYEVSLGLIIIGVIISTGSMNFGDIVNAQRG---DFGLFSWYWLPH----FPMVFLFFISALAETNRPPFDLPEAESELVAGYQVEYSSTPFLLFMIGELVAVVLMCALVSLLFLGGWLSPIPGLPD-------------GILWMIAKMLVVFFMFAMIKAITPRYRYDQLMRLGWKVFLPFSLVWVVFVSFAAKFDWFWGAFARWSTGG--MDHFLYRDGALFAEDVPVTEIAAAVGTPFYVYSTATLLRHYHLFDEALAGTEHLVCYAMKAAGNMAILRTLAQAGAGMDVVSQGEYLRARAAGVPGDKIVFSGVGKTEEEMRVALEGGIRQFNVESEPEMEALSRVAQSLGKVAPITIRVNPDVDARTHAKIATGKSENKFGIPISRAREVYAMAAALPGLEIVGIDVHIGSQLTELEPFEQAYGKVAELTEALRADGHTIGRLDLGGGLGIPYTRSNAAPPLPTDYGAMIKRTLGHLGCEIEIEPGRLIAGNAGLLVSSVIYVKNGEGRDFLILDAAMNDLIRPAMYDAHHDIIPVIEPVPGAEQHPYDVVGPVCESGDTFARQRDLPELASGDLVAFRSAGAYGAVMSSEYNARPLIPEVLVNGDQFAVIRRRPSFDEMMNRDTIPSWL-----------MTRDLTVLGLESSCDDTGAAVVRLSGD------TGGSILSNVVYGQAELHRDFGGVVPEIAARAHTSKLDLAVRAALTEAGTSLAGIDAIAVTAGPGLIGGVMAGVMCAKGLAAATGLPLIGVNHLAGHALTPRLTDGLTYPYLMLLVSGGHCQFLIARGPDSFARLGGTIDDAPGEAFDKAARLLGLPQPGGPAVERAALDGDPKSFRLPRPLLDRSGCDMSFSGLKTALLRARNTLIADQGGLFRQDVADLCAAFQSSVCDVLAEKTRRALAEY-MVLNPTQPALAVAGGVAANNAIRAVLETVSSDAGAQFCAPPLALCTDNAAMIAYAGGELFRSGHI--DDMTLTPRPRWPLDRSSP---AMLGSGKKGAKA-----MSDTREAALASKAWPFEEARRVLKRYEKA-----PPEKGYVLFETGYGPSGLPHIGTFGEVARTTMIKRAFEEIS--DIPTRLICFSDDLDGMRKVPGNVPNQDMLREHLQKPLTSVPDPFGTHDSFGDHNNAMLRRFLDTFGFEYEFISATDFYRSGQFDTILRRAAERYDDIMKIMLKSLREERQQTYSIFLPIHPETGRVLYVPMKEVNAAEGTITFDDED-GREWILPVTGGNVKLQWKPDFGARWAALGVDFEMYGKDHSTNTPIYDGICRALGVPAPEHFTYELFLDENGQKISKSSGNGVSIDEWLTYASTESLSYFMYQKPKTAKRMHFDVIPKAVDEYHQQLRAYPTQDVKAQLNNPVWHIHGGNVPESTLVVPFGMLLNLASVSGAQDKAQLWGFINRYAPEASAETHPDLDQAAGFAVRYFNDFVKPTRQFRAPTEQEREALEALRAELSTYNGPL----------------------DDEALQSVVYSVGRDR-FDPLRAWFTALYEVLLGASQGPRFGGFIALYGVQETIALIDKALAGELA---MRCPFCGNIDTQVKDSRPAEDHVSIRRRRFCPACGGRFTTYERVQLRDLVVIKSSGKREDFDRDKLERSIRISMQKRPIEPERIDQMISGIVRRLESMGETDIPSHKIGEIVMEALARIDTVAYVRFASVYKNFQAADDFEDFVHELRPPSPTEE-----MPVVVVESPAKAKTINKYLGPGYTVLASYGHVRDLPPKDGSVDPEHDFDMKWEVASDSRKHVKAIADALAEDGELILATDPDREGEAISWHLQEALTKRKSIKKDTPVSRVTFNAITKDAVAQAMANPRQVDMPLVEAYLARRALDYLVGFNLSPVLWRKLPGAKSAGRVQSVCLRLIVEREMEIEAFRAREYWSVKALLATPRGQEYEARLTVLAGKKLDRYDIENGTQAELAVQAITSRDLTVTSVEAKPASRNPSAPFMTSTLQQEASRKFGMGARQTMSAAQRLYEAGHITYMRTDGIDMAPEAVSAARDAIAARFGKDYVPGSPRIYKNKAKNAQEAHECIRPTDMTADADALKLTDADQRKLYDLIWKRTLACQMESARLERTTVDVGSRDGQVELRATGQVVLFDGFLRVYEEGRDDA-VTDDDDKRLPQISQGEKA-----------------------------------------------DKRSVTPEQHFTQPPPRYTEATLVKRMEELGIGRPSTYASIVTTIQDREYVRKDKNRLIPEDKGRLVTAFLQNYFQKYVGYDFTAGLEDELDHVSAGDADYKAVLNRFWRDFSAAIAETSELRITDVLEKINEVLEPHLFPPTE-DGS---DPRRCPNCGEGRLSMRTARSGGAFIGCSNYPECRYTRAFGPPGAE-DDGAIPPDGKLLG--EDMGDEIRVFKGRFGPYVQRGPVTEE-NKKPPRQSVPK----DWPAEELELERAVMLLSLPREIGPHPEDGVMVWSNIGRYGPYIKHAPSTSDRGGTNANLESLDEVFTVGMNRAVQLLAEKVASRGGR-GRAAAPLRELGEHPEAGGPVSIMSGKYGPYVKWEKINATLPDTIEPDQVTMEQAVELIAERAAKSGKK----AP----KKAAA----KKPAAKKAP-----------AKKAAAKK-APAKKAPKAEE----------------MIQMQTNLDVADNSGARRVQCIKVLGGSKRKYASVGDIIVVSVKEAIPRGRVKKGDVRKAVVVRTAKEVRRDDGTAIRFDRNAAVILNNNNEPVGTRIFGPVVRELRAKNFMKIISLAPEVL-MD-NLLSIVTFIPALAALILAVFLR---------GEDEAAQRNAKWVALFATAITFLVSIGIYAGFNPDDTGFQFVEEANWLGGLKYKMGVDGISVLFVMLTTFIMPLTILASWE-VKTRVKEYMIAFLLLETLMLGVFMALDLVLFYLFFEAGLIPMFLIIGIWGGANRIYASFKFFLYTFLGSVLMLVAMVAMYADAGTTDI-------EVLLTHEFSSASFSLLGIQVFGGLQTLLFLAFFASFAVKMPMWPVHTWLPDAHVQAPTAGSVVLAAILLKMGGYGFLRFSIPMFPIGSEILTPLVLWMSAIAIVYTSLVALVQEDMKKLIAYSSVAHMGFVTMGIFAGNQQGIDGAIFQMLSHGFISAALFLCVGVIYDRMHTRDIDAYGGLVVRMPAYALVFMLFTMANVGLPGTSGFIGEFLTLMGTFQTNTWVAAVATTGVIFSAGYALWLYRRVVFGDLIKGSLKTIQDMTTRERAIFAPLVVMTLLLGVYPALITDIIGPSTAALVDHYQAAVSAGE-AATQHASN------MSDEFMLDTDDLKRRMDGAMANLRTEFASLRTGRASASMLEPVMVDAYGSQTPINQVGTVNVPEPRMVTINVWDKGLVGKVEKAIRESGLGINPQLNGTIIMLPIPELNEERRRDLTKVAGQYAEGARVSVRNVRRDGMDQIKKAKADG-MSEDDQKFWESEVQDLTDKMIKAVDEALETKQAEIMQVMTE------PD--ASF-----DRRMLEALICPQTHATLEYDAAAQELISKPAGLAFPIRNGIPVLLIDEARELD--------MQDPAITPDLIAA---------HGLKPDEYERILEIIGRAPTFTELGIFSAMWNEHCSYKSSKKWLRTLPTDGPQVICGPGENAGVVDIGDGQAVVFKMESHNHPSYIEPYQGAATGVGGILRDVFTMGARPIAAMNSLSFGATTHRKTRQLVNGVVEGIGGYGNCFGVPTVGGEVRFHAAYNGNCLVNAFAAGLADADAIFYSAASGVGMPVVYLGAKTGRDGVGGATMASAEFDDTIEEKRPTVQVGDPFTEKRLMEATLELMKTGAVISIQDMGAAGLTCSAVEMGDKGGLGIRLDLERVPVREQNMTAYEMMLSESQERMLMVLRPEKEAEAKAVFDKWDLDFAIVGETIAEDRFLIQLNGETKADLPLSKLASSAPEYDRPWVEPAAPAPLGDVTAIDP---IAGLTALIASVNYAGKQWVYEQYDTMVMADSARVPGLG--AGIVRVHGTDKALAFTSDVTPRYVRANPVEGGKQAVAEAYRNLTAVGARPLASTDNLNFGNPEKPEIMGQFVGAIKGIGAAVAALDMPIVSGNVSLYNETDGAAILPTPTIGAVGLIENVADIIGCD-VRDGHVLLVVGDTAGHLGQSALLAELW-GREE---GDAPAVDLEAEKRNGDFIRD--NRALIKACTDVSDGGIALAAFELAEGAGVGVQIDSDDT-------GFLFGEDQARYLIGCNFDQAEALMGAAAQAGVPIAHIGKFTG----STVRFGKSEA-ALDALAATFRG---SFAAAVA-MKTFSATPADIDKKWILIDAEGVVLGRLASIVAMRLRGKHKASFTPHMDMGDNVIVINADKIQMTGKKR-EEHFYWHTGHPGGIKSRTKQQILEGAHPERVVFQAVKRMLP-GNRLSRQQMTNLRIYAGGEHPHEAQSPEVLDVKSMNKKNTRS--MAPRVLISDKLSDAAVQIFKDRGIDVDFQPDLGKDKDKLAEVIGQYDGLAIRSATKVTEKILENATNLKVVGRAGIGTDNVDKDAASKKGVIVMNTPFGNMITTAEHAIAMMFAVARQIPEASASTHAGKWEKSKFMGVELTGKTLGVIGAGNIGGIVCDRARGLKMKVIAYDPFLGEEKAAKMGVEKVELDALLARADFITLHVPLTDQTKNILSRENLAKTKKGVRIINCARGGLVDEEALAEMLQSGHVAGAAFDVFSEEPAKENALFNLPNVVCTPHLGAATTEAQENVALQVAEQMANYLLTGAVENALNMPSVTAEEAKVMGPWLKLSGHLGAFIGQMTDEPIKAINILYDGVVSSMNLAALNCGVIAGIMKKSNPDVNMVSAPVVAKERGIQISTTNQDKSGAFDGYIKVTVVTEKRE-------RSIAGTVFSDGKPRFIQIKGINIDAEVGAHMLYTTNEDVPGIIGTLGNTLGTNGVNIANFTLGRSDRGGEAIALLYVDDEVPADVRQKLADTGLFRQIKPLQFDVA--MKTQD-MNRDWTNTAETLSRAVPYLQRYDDAIVVIKLGGHAMGSDEAMDEFARDVVLLRLCGVNPVVVHGGGPQINALLKQLEIKSDFVNGKRVTDKATVDVVEMVLSGSVNKSIVQAIQRQGGKAVGLSGKDANLMVCDQT-----DP--------ALGLVGTPADMDPSLLKLL--FEKEIIPVIAPIGAGRNG-ETYNVNGDTAAGAYAKALKADRLLLLTDVAGVKNAEGDVVTELTAAEIRQMTADGVIAGGMIPKTETALDAVEGGVRAAVIIDGRAPNACLLELFTEHGAGSMIRR------------MTPATKLPGDVGPIHFVGIGGIGMSGIAEVLINHGYTVQGSDLKTSRITDRLAGLGATIFE-GQRAENLENAEVVVISSAIKTGNPELDEARRRGLPVVRRAEMLAELMRLKSNVAIAGTHGKTTTTTMVATLLDAGGLDPTVINGGIIHAYGSNARVGLGEWMVVEADESDGTFNRLPATIAIVTNIDPEHMEHWGSFDALRDGFHEFVSNIPFYGLAVCCTDHVEVQRLVGRITDRRVVTYGFNAQADVRAVNLHYEKGVAHFDIALQADE----IVIEGCTLPMPGDHNVSNALSAVAVARHLGMKAEEIRTALANFAGVNRRFTRVGE------VDGVTIIDDYGHHPVEIAAVLKAARQATEGR-VIAVHQPHRYSRLSSLFDDFCNCFNEADVVAIAEVYAAGEDPIPGASRDDLVSGLIRHGHRHARAIVSEDDLERLVREQARPGDMVVCLGAGTISAWANALPERLTN-KGAAA-MAITAAMVKELRESTGAGMMDAKKALTENDGDMEAAVDWLRTKGLAKAAKKSGRTAAEGLVAVVV--EGGKGVAIEVNSETDFVAKNTEFQSMVGAIAQAAVSADD-IEALKAAEV--NGKTVESTLTDAIAKIGENMSLRRMVKIEGG-QVVAYVHNAAAPGMGKIGVLVAMTGGD--EAFGKQVAMHIAATNPAALNEAELDPAVVEKEKQVQMDIARESGKPEEVIEKMIVGRMKKFMAEVTLLSQQFVINPDLTVGAAAKEAGAEITGFVRLEVGEGIEKKVEDFAAEVAKTAAGG-MSDILIVDDERDIRELVSDILEDEGYATRLAGNSDECMNAINAEPPALLILDIWLKDSRMDGIDILKTVKRDNPDVPVVIISGHGNIEIAVAAIKQGAYDFIEKPFNIDQLMVVIRRAMETSRLRRENSSLKRREVTSAEMIGQSAAFRTLVGQLDKVTKSNGRVMLTGPAGSGKEIAARYIHAQSNRASGPFITVNCAGVEPERMEEVLFGRESAE-RGIESGLLEEAHGGVIYFDEVADMPLGTQSKILRVLVDQQFQRVGGSDKVRVDLRVISSTNRDLEHEIRAERFRQELYHRLNVVPISVPSLEERREDIPVLAEHFIAACHAAQGLPLRPLSDEAVALMQTMTWPGNVRQLKNLVERLLIL--GEGNGPIEARELPGEDDA-GGESGRVVLSGALATLPLREAREAFEREYLLTQINRFGGNISRTASFVGMERSALHRKLKSLGVVTSTKMGARVAHVDDIDDQVEAAE-MLQPKRTKFRKQHKGRIHGEAKGGSTLTFGTYGLKALQPERVTARQIEAARRAMTRHMKRQGRVWIRIFPDLPVTSKPTEVRMGKGKGSVDYWAAKIKPGRVMFEIDGVDDDIAREALRLAAMKLPLKTRVVVR-EDWMFSKYKKPG---PKAPAAAAPAANTAPKPKEVHAAPQ-AAPTPTPAPTPAPSMRRKVEQVPADTAAGDKERKRKERMGEIKIEMHRALLDNLNLAALEHATEKELRNEIATIAGELLDERGIVLNREDRQTLNKELYDEVTGLGPLETLLQDDTVSDILVNGPQQVFVERGGKLQLSDVTFKDERHLLRIIDKIVSAVGRRVDESNPYVDARLADGSRFNAMVPPIAVDGSLVSIRKFKKDKLQIDDLVNFGAFTEEMAAYLQAAVATRLNIIVSGGTGSGKTTTLNALSSFIDNSERILTIEDTAELQLQQTHVGRMESRPPNVEGKGEVSPRDCLKNALRMRPDRIIVGETRGEEVIDMLQAMNTGHDGSMTTIHANNPRDGVSRLENMIAMAGIEMPLKAMRSQISSAVNLIVQASRLQDGSRRMTSITEVTGMEGDVISMQEIFKFQRVGLTPDNKIIGHFTATGVRSHYSERFRMWGYDLPASIYEPTVMESR---MDD---LKSKYLGQIADAADENALEQIRLAAVGKKGEVALKMRELGRMTPEERQTAGPALNALKDEINSALAAKKAALEDAALDERLRSEWLDVTLPGRPRH-QGSVHPVSQVTEELTAIFAEMGFSVAEGPRIDTDWYNFDALNIPGHHPARAEMDTFYMHRAEGDNRPPHVLRTHTSPVQIRTMEKMGAPLRIICPGGVYRADYDQTHTPMFHQVEGLAIDKNISMANLKWTLEEFFAAFFEIDGIKTRFRASHFPFTEPSAEVDIQCSWVDGQLRIGEGDGWMEVLGSGMVHPKVLAAGGIDPDKWQGFAFGMGIDRIAMLKYGIPDLRAFFDSDLRWLRHYGFASLDQPTLHGGLSR-MSD----TDGKKTLGLR--GG-SR--PGNVKQSFSHGRTKNVVVETKRKRVV-VPKPGAVKS-ASAGVSA---GDPSR-------RPGGISDAEMERRLKALQAAKAREADEAAQREAEEKAREEERDRRRAEAEAKEREEREREEA-LKAKAEEEERQKREAEEAAR-RAAEPA---PA--PEEANRGGGA-----AKA-AP---SSPRKVDTEKDREQRTTR-----GKGRD-DNRRSGKLTLNQALSGGEGGRQRSVAAMKRKQERARQKAMGGNVEREKVVRDVQVPEAIVVSELANRMAERVGAVVKALMQNGMMVTQNQTIDADTAELIVEEFGHNVVRVSDSDVEDVITQVEDKADDLSPRPPVITIMGHVDHGKTSLLDAIRNAKVVAGEAGGITQHIGAYQV-KTEGGQMLTFLDTPGHAAFTSMRSRGAQVTDIVVLVVAADDAVMPQTIEAINHAKAAKVPMIVAINKIDKHEANPTKVRTDLLQHEVVVEQMSGDVQDVEVSAHTGQGLDNLLEAIALQAEILELKANPDRAAQGAVIEAKLDVGRGPVATVLVQTGTLKQGDIFVVGEQWGKVRAMENDKGERVKEAGPSVPVEVLGLNGTPEAGDVLNVVSTEAQAREIAEYREKAAKD--KRAAAGAATTLEQLMANAKADE-NMSELPILIKADVQGSAEAIVQAMEKIGNEEVRVRVLHSGVGAITETDVGLAEASNAPIMGFNVRANASARNTANQKGVEIRYYSVIYDLVDDVKNAASGLLSAEIREHFIGYAEIREVFKVTGVGKVAGCLVTEGTARRSAGVRLLRDNVVIHEGTLKTLKRFKDEVSEVQSGQECGMAFENYDDIRNGDVIEIFQREEVTRTL-MAAESQTISLTLPDGSARQYDAGITPGDVAADISKSLAKKAISATVDGKHWDLQWPIQSDAAIAIHTMQDEAQANELVRHDLAHIMARAVQEIWPDTKVTIGPVIDNGWYYDFDRAEPFTPEDLGAIEKKMKEIINARDAVRTEVWDRARAVKHYADNNELYKVELIDAI--PGD-EPLRMYWHGEWQDLCRGPHLQNTGQVPGDSFKLMSIAGAYWRGDSSRQMLQRIYGVAFTNKEKLKAHLHMLEEAAKRDHRKLGREMDLFHMQE-EAPGQVFWHPNGWTVYTQLQDYMRRKQRAGGYREINTPQVVDRKLWEASGHWDKYQHHMFLVEVDESRDGENDDASKAKNRDQTRINALKPMNCPCHVQVFNQGLKSYRDLPLRLAEFGSCARFEPSGALHGIMRVRGFTQDDAHIFCTEDQIQAECARFIDFLADIYRELGFPDFEIRFATRPEKRVGSEESWDYVEGALENAIKAVGRDYVLEPGDGAFYGPKLDFYLTDAIGRVWQCGTFQVDPNLPERLNASYIAPNGEKTRPFMLHRATLGSFERFIGILIEEHAGKLPFWLAPRQVVVASITSE-AEEYADEVVAQLTAAGIRAEADTRNEKINYKVREHSVGKVPVILAVGAREVEERTVSVRRLGEKQTSVQALADVTAALKREATPPDLL-----------------------------------MSDTP------------SPSRSFGRKSIRASLQPRDLMTDGDTLAGVWTARIITLFPEAFPGVLGQSLTGRALKDGLWQLETIDLRGFGIGKHRNVDDTPAGGGAGMVLRADVVGPAIEAAQSGV---RG----DWPVIGLSPRGQPMTQALMHRLSRADGVTLLCGRFEGIDERALEHYGVQEISLGDFVMTGGEIAAQALIDATVRLIPGVLGNAASTDEESFSDGLLEHPQYTRPASWQGRDIPDVLMSGNHGEIARWRRAAAKELTRIRRPDLWAHRTSGSNDQE--------MSKDKNPRRVADNEAMAKLRMLRTSPQKLNLVAAMIRGKKVERALTDLTFSKKRIAVDVKKCLQSAIANAENNHNLDVDELVVAEAWVGKNMTMKRGRPRARGRFGKIVKPFSEITILVR---QVEEQA--MDSIIVTGNGPLSGQIPIAGAKNACLTLMPATLLSDEPLTLTNAPRLSDIRTMTDLLGSLGAEVASLQGGKVLALSSHDLNNHTADYDIVRKMRASILVLGPMLARDGHAVVSLPGGCAIGARPVDLHLRALESLGAEMDLRDGYVHAKAPAGGLRGGVIEFPFPSVGATENALMAATLAKGTTVIKRAAREPEIVDLADCLRKMGAQIEGDGTDTITVQGVDRLGGATHPVVTDRIELGTYMLAPAICGGEVELLGGRRDLLAAFCDKLDEAGVDV--TETEAGLSVRRRSDHISAVDVATEPFPGFPTDLQAQMMALLCTADGVSNLEETIFENRFMHAPELMRMGARIDVHGGSAKVTGVERLKGAPVMATDLRASVSLILAGLAAEGDTKVSRVYHLDRGYEHVVRKLRGVGAQIERVVGQ----MSASTPAKPTACLALADGTIFYGNGFGATGQTVAELCFNTAMTGYQEIMTDPSYAGQIVTFTFPHVGNVGVNPDDDETGDPVAE----GLVVKWDPTAPSNWRSAENLKTWLERRGRIAIGGVDTRRLTRAIRQSGAPHVALAHDPDGNFDIAALVAAARGFKGLEGVDLAKDVTCAQSYRWNEMRWAWPEGYSEQKDPKFKVVAVDYGAKRNILRCLATVGCDVTVLPASATAEEVLAHAPDGLFLSNGPGDPAATGAYAVPMIREVMEQRDMPVFGICLGHQMLALALGARTVKMNHGHHGANHPVKDLETGKVEITSMNHGFAVDAQSLPAGVIETHRSLFDGSNCGIRMADRPVWSVQYHPEASPGPQDSYYLFERFAAAMAERQPA--------MRPSGRQLDQMRPITIETGFTRHAEGSCLIKAGDTHVLCTATIEDRVPPFIKGTGLGWVTAEYGMLPRATNTRMRREAASGKQGGRTVEIQRLIGRALRAGVDRVALGERQITVDCDVLQADGGTRCASITGGWVALRLAVNKLLKAGDITSDPLVDPVAAVSCGIYAGQPVLDLDYPEDSEAGVDGNFIMTGSGKLIEVQMSAEGSVFTRDQMNTLLDLAVKGTGELVAAQQAACA-MATVAVTDATFDAEVKNSDIPVVVDFWAEWCGPCKQIGPALEELAAEYEGKIKVAKVDVDSNPNAAAAMGVRGIPALFIFKDGQVVSNRAGAAPKAALQSWINESIMPTIQQLIRKPRQPKVKRSKSMHLQECPQKRGVCTRVYTTTPKKPNSAMRKVAKVRLTNGFEVISYIPGESHNLQEHSVVLIRGGRVKDLPGVRYHILRGVLDTQGVKDRKQRRSKYGAKRPK--MTYWLFKSEPSTWSWQNQAEKGAEGEEWDGVRNYQARNFMREMKLGDLGFFYHSQSEKAVVGIVEVCAEVHPDSKADDPRWECVDIRAVRQFTQPVTLDQIKADPRLGEMVLVKNSRLSVQPVLPDEWAAICEMGQTQP---------------------------MSTRAGFVALIGEPNAGKSTLLNRMVGAKVSIVTHKVQTTRARIRGVAMEGDAQIVFVDTPGLFQPRRRLDRAMVAAAWGGAGDADVIVLMVE--------------AHRGITDGVTRILETLAEVGQGRTVALAINKIDRVPSEALLGLTKDLNETYDFAETFLISAERGHGTDDLRKWLATQLPEGPWLYPEDQIADLPMRMIAAEMTREKLTLRLHQELPYQMTVETEAWEDRKDGSARIEQVIYVVRDGHKGIVLGHKGETVKAVGKAAREELETFLGRKVHLFLQVRVRPNWLEESERYSQMGLDFKDGN-MSDIVDDPNMEPPEGVGEVAEPLRRAIGERYLTYALSTIMHRALPDARDGLKPVHRRIIYAMNRLRLASNGKFLKSAKISGDTMGDFHPHGDAAIYDAMARLAQDFAVRYPLVDGQGNFGNIDGDNPAASRYTEARMTFVAEALLNGLDEDAVDFRDNYDGRLTEPVVLPAEFPNLLANGSAGIAVGMATNIPPHNIAELCDACLHLIRTPDARDETLLNYVPGPDFPTGGVIVEPPENIAQAYRTGRGSFRLRCRWEVETLERGTWQIVVTEIPYQVQKSKLIERIADLIQTKKVPILADVRDESAEDIRVILEPRSKNVDPEVLMGMLFRNSELEVRFSLNMNVLIDGVTPKVCSMKEVLRAFLDFRQEVLLRRSRHRMGKIDHRLEVLEGFIVAFLNLDRVIDIIRYDDDPKAALMREDWGVDH----ARAMTEADYVPPAP-S------------------------------------DVQSLSEVQVDAILNMRLRSLRRLEELELLREQAELMEERAGLEDLLEDEGLQWARIADQLKATKKTFGKDYE------GGARRTRLEEAGEVEEV-PLEAMIDREPITVVCSQMGWIRAMTGHIDLTRELKFKDGDAPRFMFHAETTDRLLVFGSNGRFYTLSAANLPGGRGMGEPLRLMVDLPNEAEIVDLFIHRPDRRLLVASSAGDGFVVPENDVIAQTRSGKQVLNVRGD-VRARVCTPVQG--DAVAVVGENRKVLVFMVEELPELGRGKGVRLQKYK-----------DGG--MSDATTFVLSEGLSWLDPAGRTRT--QTELEEWTGKRAGSGRMAPRGFPRDNRFT---------------MQVSQSTVDLAEVSKFEAMAAEWWDPNGKFKPLHMLNPCRLDYITRQIAGEFGRDLTGAKPFDGLRLLDIGCGGGLLSEPMARLGADVVGADAAAGNIPVAQVHAEQSGLNIDYRNTTAESLAEAGE-QFDVVLNMEVVEHVADPLAYLTACHDLLRPGGLHICSTINRNPKSFAVAIVGAEVVMRWLPRGTHDWRKFITPDELFDLLSRAGLTPVDRKGFVFNPITWSWSLSDRDLSVNYVTASLRP-----------MKIAFQMDPITGVNIDADSTFRLAEEAQARGHDLFYYSPDDLSFQEGRIMARGHDMTVQRVKGDPAKLGPRREVDLA-DFDVVWLRQDPPFDMHYITSTHLLDRLAGTTLVVNDPFWVRNYPEKLLVLDFPELTPPTTIARDLGVIREFKARHGDIILKPLYGNGGAGVFRLDENDRNLSSLHELFTGFSREPLIVQKFLPAVSKGDKRVILVDGEPVGAINRVPAEGETRSNMHVGGRPEKIELSARDREICAAIGPLLREKGQVFVGIDVIGDYLTEINVTSPTGIQELERFDGVNIAEKVWEAIEARVAAMA-----MADREFIPVRIAVLTVSDSRTLAEDRSGQVLVDRLAEAGHELADRAILPDERADIADKLRAWIADPDVDVVISTGGTGLTGRDVTVEAHRDVYEKEIDAFGTVFTIVSMKKIGTSAVQSRATGGVAGGTYLFALPGSPGACKDAWDEILVHQLDYRHKPCNFVEIFPRLTENQRRK--MALPFPLAETPDQTTAEAGRLLFARESTFVKGVVAMSGLPPADRIEVCFAGRSNVGKSTLINALTGTKALARASNTPGRTQEINYFNQG-----PDLYLVDLPGYGYANAPLPVVEKWQRLLKQYLSGRPTLRRAFVLIDARH-GVKDVDDQILRLLDTSAVTFQCVLTKVDKVKAKDMENTLKQVRAALAKHPAAYPEIVLTSSEKGDGIETLRGIIAGLS----------MHPRLILGSGSPRRLDLLAQLGLTPADVRPPDIDESPLRAELPRPYCARIARAKVQAVVADPDDVVLCADTTVALGRRILGKPADAAEAAQFLTLLSGRRHQVITSVAVRRGD-RIWQRDVVSQVRMKRLSDAELNGYLASNDWQGKAGGYGIQGPAGALIPWISGSFTGIVGLPLAETANLLRSAGL--TPLEGL---MSNPSLLILPGDGIGPEVMAEVTRIIDWFGAKRGIAFDVSEDLVGGCAYDKHGTPLHDDTMAKALEVDAVLLGAVGGPKYDKLDFSVKPERGLLRLRKEMDLFSNLRPAQCFDALADFSSLKKDVVAGLDIMIVRELTSGVYFGEPRGIFKEG-NERVGINTQRYTESEIARVARSAFELARKRNNKLCSMEKANVMESGILWRDVVTEVHEAEYSDVELTHMYADAGAMQLTRWPKQFDVIVTDNLFGDMLSDLAAMLTGSLGMLPSASLGAPTENGRPKALYEPVHGSAPDIAGQGKANPIACILSFAMALRYSFDMGDEATRVEKAVEAVLADGKRTADLLGEEGVTPVSTSEMGDAILAALDASL--MTIDQKLTDLGLALPD---APAPAANYVPFVRVGTTLYVSGQISSDASGPIRGKLGESLTVEQGAAAAQRCALSLLAQVRAACDGDLGRLERVVKLTGFVNSTGDFTDQPKVINGASDLLVELLG-DKGRHARSAVSAAALPLGVAVEIEGIFEIR-MADDLFNSFMTGPDENGRFGDFGGRFVSETLMPLILQLEAEYEKAKTDQSFWDEMDDLWANYVGRPSPLYYAERLTEHLGGAKIYLKRDELNHTGAHKINNVLGQIILARRMGKTRIIAETGAGQHGVATATVCAKFGLKCVVYMGAHDVERQKPNVFRMRLLGAEVVPVTSGRGTLKDAMNDALRDWVTNVRDTFYCIGTVAGPHPYPAMVRDFQAIIGKEVRWQLAEQEGEGRLPDTVIAAIGGGSNAMGLFFPFLDDTEVNIIGVEAGGKGVTEKMEHCASLTGGRPGVLHGNRTYLLQDDDGQILEGFSISAGLDYPGIGPEHAWLHDVGRAQYVSITDKEALEAFQLSCALEGIIPALEPSHALAHVMKIAPELPKDHIIVMNMCGRGDKDIFTVAKHLGFDMAE-------MKLDVIKLDGDKAGSVDLDEALFGLDP-RADILHRVVRWQRAKAQAGTHKVKTRSEVSYSTKKIYRQKGTGGARHGSRKAPIFRKGGIYKGPTPRSHAHDLTKKFRALGLRHALSAKAKAGELVVIDTAES-DGKTAILAKQIASLGWKRALIIDGASVNEAFAKAARNIEGLDILPTMGANVYDILKRDTLVITKAGVEALEARLK-------MHAYRSHTCADLTAKNTGETVRLSGWVHRVRDHGGILFIDLRDHYGVTQVLCDPDSAAFADVEKVRSEWCIRIDGEVKARDPELVNPKLPTGEIEVFVREIEVLGAAK-ELPLIVFGDQEYPEETRLRYRYLDLRREAMQRNMTLRSDVVASLRKRMWDRKFREYQTPIITASSPEGARDFLVPSRLHPGKFYALPQAPQQFKQLIMVSGFDKYFQIAPCFRDEDPRADRSPTDFYQLDMEMSFVTQQDVFDTISPVIAGVFEEF----GGGHAVDAPGEWPQISYKDAALWYGTDKPDLRNPIKMQVVSEHFAGS--GFAIFAKLLEQDGTEIRAIPAPGGG--SRKFCDRMNAFAQKE-GLPGMGYIFWRDQ--------------------------------GE---GMEAAGPLAKNIGPERTEAIRQQLGLGVGDAAFFLGGKPATFQTVAGKARTVIGEELGLTDKNRFAFAWIVDFPIYERDEETGKLDFEHNPFSMPQGGMEALEG-----DPLDVRGYQYDLACNGYELVSGAIRNHRPEIMFKAFELAGYGEDEVRKRFGGMVNAFQYGAPPHGGCAAGIDRIVMLLADEANIREVIMFPMNQRAEDLMMNAPSEPQGDQLMELGLRVIPRD--MFTGSMPALVTPFNNGKVDLDALKHLVEWHVKEGSTGLVPVGTTGESPTLSHEEHELVVEEVVRAAAGRLPVIAGAGSNNTDESIRFMTFAKKVGASAALVVTPYYNKPTQRGLIAHFTAVHDCCDLPIIIYNIPPRSVIDMTPATMGQLAKLPRIIGVKDATGKLERVSQQRASCG--KEFVQLSGEDATALGFNAHGGVGCISVTANVAPKLCAEFQAATLAGDYKLALEYQDRLMPLHEAIFIEPGLVGAKYGLSRLGLCSEEVRSPLTGLEDSTKAAIDAAMAHAGIEL-MAI-TSANQLELLQTAEAVAREKMIDPSLVIEAMEESLARAAKSRYGAEMDIRVSIDRKTGRATFTRVRTVVEDEELENYQAEFTVEQA------------------------------KQYM------------------ADPVVGDTYVEEVPPVEMGRIAAQSAKQVILQKVREAERDRQYEEFKDRAGTIINGQVKREEYGNVIVDVGAGEAILRRNEKIGRESYRPNDRIRCYIKDVRREPRGPQIFLSRTAPEFMAELFKMEVPEIYDGIIEIKAVARDPGSRAKIAVISYDGSIDPVGACVGMRGSRVQAVVNELQGEKIDIIPWNEDQPTFLVNALQPAEVTKVVLDEEAERIEVVVPEEQLSLAIGRRGQNVRLASQLTGLDIDIMTEEEESARRQAEFEQRTKLFMDTLDLDEFFAQLLVSEGFTNLEEVAYVEVDELLVIDGVDEDTASELQARARDYLDAQNKAALEAARELGVEDSLVEFEGLTPQMLEALGKDGVKTLEDFATCADWELAGGWTT-VDGERVKDDGILEPFDVSLEEAQSLVMTARIALGWVDPAELED----EDTEGDV------------------DGADAE-TEEAGA-----------------------------MTGFSFSLSATDGKARTGAITTLRGTIRTPAFMPVGTAATVKAMLPESVAATGADILLGNTYHLMLRPGAERVDRLGGLHEFMNWSKPILTDSGGFQVMSLAGLRKLTEEGVAFKSHIDGSRHMLTPERSMEIQR-LLGSDIVMCFDECPALPAEEKAVAESMRLSMRWAQRSRDAFGDRPGHALFGIQQGGVTRDLRAESAEALQEIGFDGYAVGGLAVGEGQEIMFDVLDFAPDMLPVDKPRYLMGVGKPDDIVGAVKRGIDMMDCVLPSRSGRTGQVFTRRGVVNIKNARHADDPRPLDEGCSCPACRGYSRAYLHHVFRAQEMISGMLLTWHNLQYYQDLMAGMREAIAAGTFCDWENQFHADRALGDIEPV---MADLF--D-TGATPT---APSPRNRPLADRLRPAALDQVIGQAHILGPDGPLGAMLAANSLSSLIFWGPPGVGKTTIARLLADATDLHFVQISAIFTGVPDLKKVFEAARIRAGNGQGTLLFVDEIHRFNKAQQDGFLPHMEDGTILLVGATTENPSFELNAALLSRAQVLVLERLDHDDLERLAGRAEAELGKPLSLTPSARERLIEMADGDGRALLNLVEQVAAWTVD-APLDPQALGARLMRRASKYDKSGEEHYNLISALHKSVRGSDPDAALYWYARMLTGGEDPRFLARRLTRMAVEDIGLADPQAQTVCLHAWETYERLGSPEGELALAQAVIYLALAPKSNAGYVAYKAAMRSARETGSTPPPMHIRNAPTKLMKEQGYGAGYAYDHDAEDAFSGQNYFPDDMPRAEFYAPVERGFERDLKKRLDWFTRQRATRE-GQT--MKHFLDIHKTDADALRGIIDQAGAMKTARQGRSRGAVDDEQPLAGRMVALIFEKPSTRTRVSFDVGVRQMGGQTMVLSGSDMQLGHGETIADTARVLSRYVDMIMIRTFDESVLTEMAEFSDVPVINGLTDRTHPCQIMADVMTFEEHRGPIAGKKVVWTGDGNNVCASFLHAAGQFGFDMTFTGPAQLDPEAEFVGFARNKGSRIEIERDAAKAVQGADLVVADTWVSMHDAQSARERRHNLLRPYQVNAELMAQAKPDALFMHCLPAHREEEVTSAVMDGPNSVIFDEAENRLHAQKAIMRWCLGV-MKRDILIHPDPRLKKVCAPVP-DLSDELRRLADDMLETMYDAPGIGLAAPQIGVLDRLIVLDCVKEDGVPPRPLVMFNPEIIASSDETSVYEEGCLSIPEQYAEVTRPAEVQVRWIDRDGKEQTEGFDGLWATCVQHEIDHLNGKLFIDYIGPMKRQMITRKMQKLKRERARG------MPD------------DALIHDATAGIERLLEIMRRLRDPETGCPWDIEQDFASIAPYTIEEAYEVADAIERSDWTELEGELGDLLLQTVYHTQMGEEAGHFTFQSVVRAISDKMVARHPHVF-GEDSRDKSAEDQTRDWERIKAAERAGKAQGG----TLDGVAPNLPALLRALKLQKRAARVGFDWPDASHVIDKIREESAELVEARDTLGPDEIEEEFGDLLFVMANLGRHLGLDPEQALRRANAKFTRRFEGVEARLAAMGKTPAQSDLAEMDALWDAVKADEKAG----------------------------MMAQYLEIKAAHADALLFYRMGDFYEMFFDDAVAAAQALDIALTKRGKHDGDDIPMCGVPVHAAEGYLLTLIRKGFRVAVCEQLESPAEAKKRGSKSVVKRDVVRLVTPGTLTEDALLEARRHNFLAAFANV-RDTFALAWTDISTGAFHVVALPAVRLGPELARLAPSELVVA--DTPEAPY-ELAEDLGIALTPIGRSSFDSTGAEKRLCALFQVATLESFGSFDRAEVSALGALVEYLDMTQKGKLPLLQPPRKEAQNRVVQIDAATRRNLELTRSLSGTRDGSLLSVIDRTVTPGGARMLDQRLSSPTRDLETVQSRLAALDFARDNARIAADLREALRKAPDLDRALSRLALDRGGPRDLAAIRAALTQADHLNTRLSSLAPPELLASAAA--DLTGFEDLLALLDA---ALVADPPLMVRDGGFIARGFDPELDETTDLRDQGRGVIAQMQSRYAEQTGISSLKIKHNNVLGYFIETTATHAPKMLSAPLSETYIHRQTTANQVRFTTVELSELETKILNAGNRALELEKRLYERLRSEILGHAPRIHTAARALAEIDLATALADLSISENWCRPIVDTSRSFEITGGRHPVVERALKRDGGTGFVANDCTLNASDGA----A-IWLLTGPNMAGKSTFLRQNALIAVLAQIGSYVPADHAHIGMISQLFSRVGASDDLARGRSTFMVEMVETAAILNQADDRALVILDEIGRGTATYDGLSIAWATLEHLNETNRTRALFATHYHELTTLATKLDGVENATVAVKEWQGEVIFLHEVRKGAADRSYGVQVAQLAGLPDSVVARARVVLEALEKGEREGGNRQKALIDDLPLFAAAPPPPP--PVVTAK-SPVDDLLATIHPDELSPRDALDLLYKLKEASTT---MKDILTELETRRAEARLGGGQARIDAQHKRGKLTARERIELLLDEGSFEEFDMFVAHRCTDFGMEKQRPAGDGVVTGWGTINGRMVYVFSQDFTVFGGSLSETHAQKICKIMDMAMQNGAPVIGINDSGGARIQEGVASLAGYAEVFQRNIMASGVVPQISVIMGPCAGGAVYSPAMTDFIFMVRDTSYMFVTGPDVVKTVTNEVVTAEELGGASTHTKKSSVADGAFENDVEALAEVRRLVDFLPLNNREKPPVRPFFDEPDRVEASLDTLIPENANSPYDMKELIVKTADEGDFYEIQEDFAKNIITGFIRLEGQTVGVVANQPMVLAGCLDIDSSRKAARFVRFCDCFEIPILTFVDVPGFLPGTGQEYGGVIKHGAKLLFAYGEATVPKVTVITRKAYGGAYDVMASKHIRGDFNYAWPTAEIAVMGAKGATEIIHRADLGDAEKIAAHTRDYEDRFANPFVAAEKGFIDEVIMPHSTRRRVARAFASLRNKKLTNPWKKHDNIPLMAGHSKWANIQHRKGRQDAARSKLFSKLSKEITVAAKMGDPDPDKNPRLRLAVKEAKSNSVPKDVIDRAIKKSQAGEGDDYEEIRYEGYGPNGVAVIVEAMTDNRNRTASNVRSTFTKNGGNLGETGSVGFMFERKGEITYGPDAGDADTVMMAAIEAGAEDVESTEDGHTIWCADTDLNDVSAALEAELG---ESQSTKLAWRPSTTTELDLENMQKLMKLVDALEDDDDVQRVTTNFEASDEVMAQL--------MIPSVLPTYSRAPLSFVKGEGAWLVEADGRRFLDLGAGIAVNSLGHAHPTLVAALTEQAGALWHTSNLYNIPQQQALADKLVEHSFADTVFFTNSGTEACELAVKMARKYWYEKGQPERTDIITFEGSFHGRSAAGIAAAGSEKMTKGFGPLLPGFVHLPWGDEDALRAAITDKTA-AILIEPVQGEGGIRPMEDSALKALRALCDETGTLLVLDEVQCGVGRTGRLFAHEWAG-ITPDIMMVAKGIGGGFPIGAVLATENAASGMTAGTHGSTYGGNPLGCAVGCAVMDVVTAPGFLDDVGRKAGRLRQKLEGLVAAHPEVFESVRGSGLMLGLRCKAVNADVVKAGYGAGVLTVPAGDNVIRLLPPLTISDDEIDEAITRLDQAASTVSAG---------MAQQNEPKLIAGNANLPLAKAIARRMTMHRGVHTGLVDARVERFNDGEIFVEVFENVRGEDMFIIQPTSNPANDNLMELLIMADALRRSSAARVTAVLPYFGYARQDRRTKARTPISAKLVANMMVGAGIERVLTMDLHAAQIQGFFDIPVDNLYASPVFALDIKTNFKGKLDQLMVVSPDVGGVARARELAKRI-NSPLSIVDKRREKPGEVAEMTVIGDVKDKICLIVDDMCDTAGTLCKAAEVLMQNGAKEVHSYISHGVMSGPAVERVTKSVMKSLVITDSIRPSEAVKGAKNIRIVPTAPIFAQAILNIWNGTSVSSLFEDDTLSPIYESLYAMD---MEPIVIAGMARTPMGGFMGALSRIPAPKLGAAAISAALSRAGVAGDAVDQVVMGNVLPAGLGQAPARQAALGADLPLSVRCQTVGKVCGSGMQAIMNAHDTLALGQSDIIVAGGMESMSGAPYLLEKARTGYRAGHGRVIDHMFLDGLEDAYDTG-----RAMGTFAEDCAEAYQFTREAQDGYALQSLDRANAAISSGAFSAEVTPVG--------DVTTDEQPGRARPEKIPTLKPAFREGGTVTAANSSSISDGAAALVLTRAGLAESQGMTPIAVIRGHAGHAQAPGLFTTAPIPAIRKVLDRVGWAVEDVDLFEINEAFAVVAMAAIRDLGLDPEKVNVNGGACALGHPIGCSGARIVVTLIAAMQARGAKKGVASLCIGGGEATALAVELL-MTETTDIAA--QIQDTISANDVVLYMKGTKSMPQCGFSSRVAGVLNYMGVDFADVNVLADEGIRQGIKDFSDWPTVPQLYVKGEFVGGCDIITEMTLSGELDTLFDSKGVSYDKDAANKIREA-NGMTE---TPVTDLSFEQAMKELESVVDKLERGDVALDQSISLYERGAALKKRCDEELRRAEEKVAAITLDGNGAPTGTKPVEGMMSATAPITQDVHTIPRKAPE-GPVNLVGLTREGMRTALIEAGTPEKQAKMRVGQIWQWIYQWGVRDFAQMTNLAKAYRADLAEKFVIEIPEVVSKQVSNDGTRKYLVRIAGGHEVEVVYIPEEDR-GTLCVSSQVGCTLTCSFCHTGTQKLVRNLTAGEIVGQVMMARDDLGEWPVPGA-PK--DETRLLSNIVLMGMGEPLYNFDNVRDAMKIAMDAEGIQLSRRRITLSTSGVVPEIARTAEEIGCQLAISFHATTDETRDILVPINKRWNIDTLLQALASYPKVSNSERITFEYVMLNGVNDTDADAHRLIKLIRDHNIPAKINLIPFNEWPGAPYKRSSNNRIRAFADIIYKAGYASPIRTPRGEDIMAACGQLKSATERARKS---RRQIEAEAGL---------------MKQESFLPDDYQPAEDEPFMNERQVEYFRRKLLNWKQELLAGSRDTIEGLQDGTRNIPDVADRASEETDRALELRTRDRQRKLVTKIDAALRRIDEGEYGYCEVTGEPISLKRLDARPIATMSLEAQERHERREKVHRDD--MSRIFDIALDDAGLPAPTPEIEQERRVAIFDLLEDNSFTLPSRDGRDAPDGPYRLNLSIREKRLVFDVETESDDKAAEFHLSLGPFRQVVKDYFQICESYFAAVKTLPPSQIETIDMARRGIHNEGSRVLQERLEGKVDVDTDTARRLFTLICVLHFGG----------MTAPEHIT-------VGGAPEGFDARLILNEVARSGAPVLHVARDDKRMAAMRAALAFFAPEMPVLTFPGWDCLPYDRVSPNADISAARMATLAALVHQM--PAQFVLLTTLNAATQRVPAREVLREAAFSARVGDRIDEAGLRAFLVRMGFSQSPTVMEPGDYAVRGGIIDIYPPGEGGPVRLDLFGDVLDGARRFDPATQRTTEKLEAVELAPVSEVILDEAAITRFRQNYRIEFGAAGTDDPLYEAVSAGRKHQGIEHWLPFFHEKLETLFDYLPKAVV-TLDDQSTATRLARWDSIADQYETRKIAMQQKSRIDSVYKPTPPGLLYLDDAAWETAVAAHRVLKFHPLAQASGPGVIDAGGRIGRNFAPERQQENISLFSALADHLRKRLEVG-PVVVASYSEGARERLTGLIEDEGLAEVIPIPDGRRI-------GKR---GLHLAVWALEHGFETA------------DLTVISEQDVLGDRLIRSTRKRRKAENFLTEAQSLSPGDLVVHVDHGIGRYHGLEVITAAGAAHECLALEYAESARLYLPVENIELLSRYGHE--EGLLDKLGGGAWQAKKARLKERIREIADRLIRVAAERALRKAPVLEPEHHAWEAFSARFPYEETEDQLRAIGDVMDDMTSGQPMDRLICGDVGFGKTEVAMRAAFVAAMSGTQVAVIAPTTLLARQHYASFAERFRGFPVNVRQLSRFVSAKEAAATREAVSRGTVDIVIGTHALLAKGIRFANLGLLIIDEEQHFGVSHKERLKSLRTDIHVLTLTATPIPRTLQLSLSGVRDLSIIGTPPVDRLAIRTYVSEFDAVTIREALLRERYRGGQSFYVVPRISDLPEIEDFLRDQVPEVSVVVAHGQMAAGELDDRMNAFYDGKYDVLLATTIIESGLDIPTANTMVVHRADMFGLAQLYQIRGRVGRSKTRAYAYLTTKPRAKLTATAEKRLRVLGSLDTLGAGFTLASQDLDIRGAGNLLGEEQSGQMRDVGYELYQSMLEEAIAKIRAGEAEGLSEVDD-QWAPQINLGVPVLIPEAYVPDLDVRLGLYRRLSSLSTKVELEGFAAELIDRFGKLPREVNTLLLVVRIKAMCKRAGIAKLDGGPKGATIQFHNDKFAAPEGLVQFIQDQKGLAKVKDNKIVVRRDWKNDADK-IKGAFAIARDL-----------AEKVVAKKKAG--------------------MLDLTYEAPKVKTIAGAKHDWELVIGMEVHAQVSSRAKLFSSASTKFGAEPNSNVSFVDAAMPGMLPVINEFCVDQAVRTGLGLKAAINLNSAFDRKNYFYPDQPAGYQISQLYHPIVGEGEVLVEMG----EGIARRVRIERIHLEQDAGKSIHDMDPTMSFVDLNRAGVALMEIVSRPDIRGADEAAAYITKLRQILRYLGTCDGNMQNGNLRADVNVSVCLPGQYEKYLESGDFSHLGTRCEIKNMNSMRFIQQAIDYEARRQIAIVEGGGTVDQETRGFDPDTGETRTQRSKEEAHDYRYFPDPDLLPLEIEQAWVDDIAASLPELPDEKKARFMTGFGLSDYDASVLTAEVEAAGYFEQAAE-----------------------------GRD-GKLAANWIINELYGRLKKD-ETQLADSPVSPAQIGQIVDLIKSDAISGKIAKDVFEICYTSGRDPEEIVETEGMKQVTDTGAIETAVDEIIAANPAQVEKAKQNPKLAGWFVGQVMKATGGKANPKAVNDIVSAKLGL---------MSDQLKGKRGLVMGVANDRSIAWGIAKAMSEAGAELAFTYQGEAFGKRLEPLAASVGSDFMVDVDVTDDASLDAAFEQLGARWPTIDFVVHAIAFSDKSELTGRFL-NTSRANFKNSMDISAYSFIEVARRAYPLMKDNGGTLLTLTYQGSNRVVPNYNVMGVAKAALEAATRYLANDLGPEGIRVNAISPGPMKTLAGAAIGGARKTYKHTDQNAPLRANATLEAVGGTAVYLASDAGACTTGEIIRVDGGFHVLGMPQAEHL---MTVFS--FYLFALSAIAGGFFTVVSRQPVHSVLWLILAFLSSAGLFVLLGAEFVAMLLVIVYVGAVAVLFLFVVMMLDVDFAELKAEMARYMPLALLIGLVILMQFVMAFGAWEANEAAEGLRTQVMPV--DRHNTEALGMILYDQYFLLFQLAGLILLVAMIGAIVLTLRHRQDVKRQDVVAQMMRDPAKAMELRDVKPGQGL-MAFFTKLKDRLFKSSSKLEEGLDAIVNDGGTPEH-----DPAP--------------------------------------------------------DVTPSPAPQEAPQPKPETQPAPPVEQPPAPAPEVP--------------------------------------------------------ARPEPAE-----------APQPEPELPQP--NPA-PRP--VDPTPAP-PPAEVPPAPAPRPASPP----AEAPQP-----------------------------RDPAPEQTKSGLLGRLL-GR--------TAPA----------------APVVRRTLDDAMLEQLEELLITADMGVDTALRVTANMAEGRFGRKLSVDEIKRLMADEIARIMEPVARPLPLYPKR-PQVVLVVGVNGSGKTTTIGKLASQFRAAGKKVVIAAGDTFRAAAVEQLQVWGDRAGVPVLTAPEGSDPASLAFDAMTRAQADGADLLMIDTAGRLQNRADLMEELAKIVRVIRKKDDTAPHNTLLVLDATTGQNAVSQVDTFRKISDVSGLVMTKLDGTAKGGVLVALADKFGLPIHAIGVGEQIDDLAPFDPQEFAAALTGLEPGPRGMKISIERGALLKAVAQAQSVVERRNTIPILANVLIEAEGDTVQFRATDLDIEVVDKAPAQVERAGATTVAATTLHEIVRKLPDGALVTLSADSAAGRLSVEAGRSSFSLATLPREDFPVMASSEYQSNFSAPAKVLRRLFDKSKFAISTEETRYYLNGVYMHVSDG-DGGRVLRCVATDGHRLARIDADLPQGAEDMPGVIVPRKTVGELRKLLEDDDMAIAVSVSETKVRFATPDITLTSKVIDGTFPDYTRVIPQGNTKRLEVDASEFAQAVDRVATVSSERSRAVKLQLDEDRLILSVNAPDSGAAEEELAVAYGDERLDIGFNAKYLLEIASQVDRENAVFLFNSSGDPTLMREGNDETAVYVVMPMRV-----MTGVCPNQTIEQMIARGEIAAAPAILPDQVQPASLDLRLGTVAYRIRASFLAGQGRSVAERLEE--FEMHRIDLSA-GAVLEKGCVYLVPLMERLDLAEGITAVANAKSSTGRLDLLTRTITDGGTEFDRITDGYSGPVYAEICPRSFSVLVRPGMRLNQIRFRKGQSVLSDADLAALHARMPLVDTPPV-IEQGLGFSVDLALP-GSTLVGYRAKPHTGVIDLDRIGHYDPTDYWEEVHATNGSIILDPGAFYILVSRESVHIPPDYAAEMAPYLAMVGEFRVHYAGFFDPGFGHAAAGGTGSRGVLEVRCHEAPFVLEHGQVVGRLVYERMSETPTQLYGAGIASNYQGQGLKLAKHFKS--VG-----------------MADLRSLFVTRLYQARLSELGGKVDPDELRASCYSIAEDDEAGQDWCEENGFPGYTSYASLTDLPWRFPIFADVVACLDAHVAAFAADLGFDLGDKALKLEDLWINILPEGGAHSSHIHPHSVISGTTYVAMPDGASALKLEDPRLPMMMAAPTRVKDVREELKSFVYVAPKVGDVLLWESWLRHEVPMNMAEDD-RISVSFNYAWE-------------------------MPVLVMKFGGTSVATLDRIRRAAKRVGVEVAKGYDVIVIVSAMSGKTNELVGWVNETSP------FYDAREYDAVVSSGENVTAGLMALTLQEMDIPARSWQGWQVPVKTTSSHSAARIEDIPPANIMNKFAEGMKVAVVAGFQGVSPEGRITTLGRGGSDTTAVAFAAAFDAERCDIYTDVDGVYTTDPRICDKARKLDRIAFEEMLELASLGAKVLQTRSVELAMRYKVKLRVLSSFEEQSDEA-GTLVCDEEDIMESKVVAGVAYSRDEAKLTLLSVADRPGIAAAIFTALSEAKVNVDMIVQNIS-E-------EGR-TDMTFSCPTDQVARAEKALAEAKSKEVLNFSEVVADTGVCKVSVVGIGMRSHTGVAAKMFRVLSQEAINIKVITTSEIKISVLIDRKYMELAVQALHDAFDLDKAA-MFENLSERLSGVFDRLTKQGALSEDDVATALREVRVALLEADVSLPVARDFVKAVQDKATGQAVTKSVTPGQQVVKIVHDALIDVLRGEG-DPGHLKIDNPPAPILMVGLQGSGKTTTTAKLAKRMKDREGKRVLMASLDTNRPAAMEQLAILGAQIG--VDTLPIVKGEDPVAIAKRAKTQASLGGYDVYMLDTAGRLHIDQELIAQAAAVRDVANPRETLLVVDGLTGQDAVNVATEFDDKIGVTGVVLTRMDGDGRGGAALSMRAVTGKPIRFVGLGEKMDAIETFEPERIAGRILGMGDIVALVEKAQETIEAEQAEKMMKRMMKGQFNMNDLRMQLEQMQKMGGMEGMMSMMPGMGKMAKQVEEAGFDDKILARQIALIQSMTKKERAAPQILQASRKKRIAAGAGMDVSDLNKLLKMHRQMGDMMKKMGKMGKGGMLKQAMKGMF-GK----GGGMP-----DPSQMDPAALEAAARQMGGKMP-GGLPGL----GGG--G------LPPGLSGF---GKKKMALDDIKSRIAAAVEK-----AGRAPDSVTLIAVSKVQPDARVRTVLEQGHRSFGENKVQEAAGKWPAFREEFDGIDLHLIGPLQTNKARQAMELAQAIHSLDRPKLANTLARLAQDTGACPDLFIQVNTGEEQQKAGILPADADAFVAECR-GLDLPVRGLMCIPPVDEEPSLHFALLAKIAARNGLSGLSMGMSGDFESAIALGATHVRVGSAIFGERVPG----------MTYHPKSDFMAVMMQRGFLADCTDYQGFDEALAAGVVPGYIGFDATAKSLHVGSLIQIMTLRWLQKTGHKPITLMGGGTTKVGDPSFRADERPLLTPAQIDDNISGIKQVFAKYLDYG-DGP-----------TDALMINNAEWLDHLNYLEFLRDIGRHFSVNRMLSFESVKSRLDREQSLSFLEFNYMILQAYDFLELNRRYGCLVQFGGSDQWGNIVNGIDLTRRVLDTQIFGLTTPLLTTSDGKKMGKTADGAIWLNGDMRAPYEFWQFWRNTTDADVGRFLKLYTEIDIDECDRLGALAGSEINDAKIILANAVTTLCHGAEAAAAAAATAREVFERGGIGD----------DLPTLTLAKDE-VGDG------------------ISIVQVIVRAGLAKSGKEAKRLIAENGARIDDAPLTDAGLMLDAAAL-ASPVKLSAGRKRHALVQLG-MSTIIDIHAREILDSRGNPTVEVDVILEDGTMGRAAVPSGASTGAYEAVEKRDGDKSRYMGKGVLEAVAAVNGEIAEALAGFDATEQESIDAAMIELDGTDNKGRLGANAILGVSLAVAKAAADFTTQPLYRYVGGTSARVLPVPMMNIINGGEHADNPIDIQEFMIMPVAADTIRDAVRMGSEVFHTLKKELSAAGMSTGLGDEGGFAPELGSTRDALDFILRSIEKAGYTPGDDIHLALDCAATEYFKDGKYVLSGEGKTLSPEENADYLAALVADYPIISIEDGMSEDDWDGWKALTDRLGNKVQLVGDDLFVTNPERLAMGIERGCANSMLVKVNQIGSLSETLRAVEMAHRARYTNVMSHRSGETEDATIADLAVATNCGQIKTGSLARSDRLAKYNQLIRIEEALGESAIYAGRSILR--MARSVWKGPFVDAYVLKKAEAARESGRHEVIKIWSRRSTILPQFVGLTFGVYNGKKHVPVNVSEDMIGQKFGEYSPTRTYYGHAADKKAKRKMSDLKKIIIDGNELEVDGAMTLIQACEQ-AGVEVPRFCYHERLSIAGNCRMCLVEVVGGPPKPAASCAMQVRDLRPGPEGQPPVVKTNSPMVKKAREGVMEFLLINHPLDCPICDQGGECDLQDQAMAYGVDFSRFREAKRASEDLNLGPLVETHMTRCISCTRCVRFTTEVAGITQMGQTGRGEDAEITSYLGETLDSNMQGNIIDLCPVGALVSKPYAFTARPWELTKTETIDVMDALGSNIRVDTKGREVMRILPRNHDGVNEEWISDKTRFVWDGLRRQRLDTPYIRENGKLRKAGWGEALTAAAAAMK-G-KTVAGIVGDLAPVEAAFALKQLIEGQG-GVVECRTDGAKL-PAGNRSGYAGTASIEDLDDANGILLIGTNPAIESPVLNARIRKAWLRGA-TVGVVGEAPDLSYDYHHFGNDRAALAEHVKTAKSE-PVGEDERGIVIVGQGALREADGAAVLGAAMEIATARQ------AKLLILHTAASRVGAMDVGAV-NDRGMDAV---S-EAEVIYNLGADEVEIAE--GAFVIYQGSHGDRGAHRADIILPGAAYTEESGLFVNTEGRPQLALRAGFAPGEAKENWAILRALSGELGSALPFDSLAQLRQKLVAE------------VPHLADIDEVPENEWVA-VEP-GSLGEASFVPAISDFYLTNPIARASALMAELSANAKARRAEKIAAEMAEIKDPENTILLELKDGTVVIELLPDVAPKHAERMKELARSGAYDNVCFHRVIDGFMAQTGDVQHGDME-DGFNIRMAGTGGSDLPNLPAEFSKLPHDRGTLGAARSQNPDSANSQFFINFKDNHFLNGQYTVYGRVISGMEHVDAITRGEPPANPDRMVSVKVAADASMLDAASNRSELPAEIARRRTFAIISHPDAGKTTLTEKFLLYGGAIQMAGQVRAKGEARRTRSDFMQMEKDRGISVSASAMSFDY----ANYRFNLVDTPGHSDFSEDTYRTLTAVDAAIMVIDGAKGVESQTQKLFEVCRLRDLPILTFCNKMDRESRDVFEIIDEIQENLAIDVTPASWPIGMGRDFLGCYDILRDRLELMDR--ADRNKVAQSIEIKGLDDPKLAEHVPAAQLEKLREELEMARELLPPLDAKSMAEGSLTPIWFGSAINSFGVKELMEGIANYGPEPQIQSATPR----EILPEETKVSGFVFKVQANMDPKHRDRVAFVRMASGHFRRGMKLTHVRSKKPMTISNPVMFLASDRELAEEAWAGDIIGIPNHGQLRIGDTLTEGEDLRVTGIPSFAPELLQGVRAGDPMKAKHLEKALMQFAEEGAAKVFKPSIGSGFVVGVVGQLQFEVLASRIELEYGLPVRFEASQFTSARWVGGERA-AVEAFANANKQHIAHDHDGDIVYLTRLQWDIDRVERDYPKVKLTATKEMMV--MAQKSDNPADPFKKALAEATKVMANDPELTVSYTVDPSGLSGEAMRLPQISRRMSREEVLLARGTADALAMHRKYHDNATHARYAPPGDMARDLYEAMEVARCEAMGARDMPGTAGNIDAKIGNDALRRGYDQITQSSEAPLPMAAGYLVRHLATGRDLPRGAENVMNLWRGFIENQAGGSLEQINEMLADQSAFARLARQVIKDLGFGDQLGDDPDANDDEQEDQAEEDQSDDQ-EPESTGDDDSD-DDAADAD--PER---SQDDQQDAADAQVSMDDLADDELGEDAEMPD-AEAPLDPPGPPPASEADPDYKVFLTTHDEEIAAEDLAEPAELERLRAYLDQQLEPLKGAVSRLANKLQRRLQAQQNRSWEFDREEGILDAGRLARVVANPTTPLSFKVEKDTEFRDTVVTLLLDNSGSMRGRPISIAAICADVLARTLERCNVKVEILGFTTRAWKGGQAREAWLNDGRPQQPGRLNDLRHIIYKGADAPWRRARPNLGLMMKEGLLKENIDGEALEWAHRRMINRREQRKILMVISDGAPVDDSTLSVNPANYLEKHLRDVIAMVEKRKLVELLAIGIGHDVTRYYDRAVTITDVEQLAGAMTEQLAALFDSD-PRARARVM-----------------GM----RKAS-------------------MSDPIRRTGFAPRFWERKQMSELTQPEWEALCDGCGKCCLNKLEDEDTGEIALTRIACRLLDDSTCRCGQYDIRHQFVPECIVLRPDNIEDHLYWMPQTCAYRLIHEGRPLYDWHPLISGRADSVHHAGVSVRGITVPEFEIAEEDWEDHLIE----EPTMAEL-DAHLLIVDDDERIRDLLKKFLMRHGFLVTSARDAAHARRILSGLDFDLIVLDVMMPGEDGISLTRALRE--TKATPILLLTARGETENRISGLEAGADDYLSKPFEPKELLLRINAILRRMPELPAAATAPKVLTLGPIRYDIERGEMWQGEEIVRLTATESQLMRIFAARPGEAVSRTKLVEDLGRDRGQAQERAVDVQITRLRRKIEADPKQPRYLQTVRGAGYMLAPDMSEKTLTRMDLSEAVFREVGLSRNESAQLVESLLEHMSDALVRGEQVKISSFGTFSVRDKSARIGRNPKTGEEVPIQPRRVLTFRPSHLMKDRVASGNKA-MTQ--TPEALEGA----PLIA-PSSTDHPLYDQVVEACRTVYDPEIPVNIYELGLIYTIDINQENEVRIIMSLTAPGCPVAGEMPGWLADAIEPIPGVKQVDVQLTWEPPWGMEMMSDEARLELGFMMAHAKN-HDYHILAPSIAPFMGSVAGFIMLFGAVLWMHE----------VTPYVFWIGFVGVLYTMFTWWSEVVKESHIGDHTPVVRIGLRYGFILFIMSEVMFFFAWFWSFFKHAIYPMSS-----YVGTEY-----------------IPPEIHAIDPFHLPLINTLLLLLSGCAVTWAHHGLVHGNDRKALVNGLAIGIAFGIAFTALQAYEYILLLTHDGWEFGGDQFYSNFFMATGFHGAHVLIGTIFLIVCLIRAMRGDFTPEKHIGFEAAAWYWHFVDVVWLFLFVAVYV--W---GTSGF---------------MRAPYASDPQASRGRLVAEEESSFRSCFQRDRDRIIHASAFRRLKHKTQVFIEHEGDYYRTRLTHSIEVAQVARTISGALGLNPELTEAVALAHDLGHPPFGHTGEDALHVLMAPYGGYDHNAQAIRIVTDLERHYADFDGLNLTWETLEGIAKHNGPV-----TG--------DLPWALAAYDARHDLELATYASAEAQVAAIADDVAYNHHDLHDGLRAELFSTDELAELPILDRCFAEVDRLYPGLNYYRRRHEALRRFFGVLVEDVINFARVRLAEIDPKSAADVRHAGRGLIRFSDPVFNDLKQIREFLFHRMYRAPSVVLMREKVTQVVHDLFPHYMAHPDQLPKQWRKDV-EEARNETALARIVSDYVAGMTDRFAIQQHAAIV-GTKIVPDGSYQ-----MTIQKIGVIGAGQMGNGIAHVMAMAGFDVIMNDISQDALDAAVKRIEGNLARQVGRGKVSESDMTAAMSRITTTLHLKDIGQTDLVIEAATERESVKQAIFEDLQPHLKPETILTSNTSSISITRLASRTDRPERFMGFHFMNPVPVMQLVELIRGIATDEATYKACLGVVEKLGKTAASAEDFPAFIVNRILMPMINEAVYTLYEGVGSVKSIDESLKLGANHPMGPLELADFIGLDTCLAIMNVLHDGLADTKYRPCPLLTKYVEAGWLGRKTNRGFYDYRGE-HPVPTRMSKRDFYDVLGVAKSASADEIKKAYRKKAKELHPDRNADNPEAEAQFKEANAAYEVLKDAEKKAAYDRFGHAAFD-GGMGGGGGARPGQGY-GGG--DFSSAFSDVFDDLFGDFMGGRQGGGGGGR-RAARGADLRYNLRISLDEAFSGMQKTINVPTAVACSSCNGSGAEGGAEPTTCPTCSGMGKVRAQQGFFTVERTCPTCSGLGQIIKNPCKSCGGQGRVEKNRALNVNIPAGVETGTRIRLAGEGEAGMRGGPPGDLYIFVEVGPHDLFERDGVNLYCRVPVSMAKAALGGAIEVPTIDGGRGRVQIPAGSQSGRQMRLRGKGMPPLRGTG-NGDMYIELAVETPVNLSSRQKELLKEF-EDLGEDNNPESKSFFSSVKSFWDGMKG-----MDKFDKLTGVAAPM-----PLVNIDTDMIIPKQFLKTIQRSGLGANLFDEMRYDREGNEVPDFVLNQPAYREAEILVAGENFGCGSSREHAPWAIKDFGIRCVIAPSFADIFYNNCFKNGILPIALPQEAVDVLMKDAEKGSNARMSVDLEAQTVTTSDGEVFTFELDSFKKHCLMEGLDDIGLTMAKEASIDSYEAQAAQARPWV------MATLTTARRIVVKIGSALLVDRRSGQLRVDWLHALAQDVAMLRARGADVLLVSSGSIALGRSVLGLGNSDLALEQSQAAAAVGQIRLARAYEEALAPHDITTAQVLVTLEDSADRRRYLNTRATLGTLLGMNVVPIVNENDTVATDEIRYGDNDRLAAQIAVTVGADLLVLLSDIDGFYSANPSQDPSARRFDVIDRITPEIEAMA--GDAGSGLSKGGMKTKLMAAHMAMTGGCGMVITEGSPNRPLSLLEQGANATWFTAQEDPQLARKRWI-GAMKPRGEVYVDAGAERALRRGTSLLPAGVTRLGGDFGRGDPVSIIGPGG-PLGLGLIRYTAEEAGRIRGCHSSEIEERLGYPGRAALIHRDDMAVSLAVETGMPEVIFPGPEGRLEGRYHPQKEKDAPIAIVLHPHPQFGGTMNNKVVYNLHYAFYNMGFTVLRFNFRGVGRSQGEYDQGIGELSDAASALDYLQSMNNNSKHCWVAGFSFGAWIGMQLLMRRPEITGFISVSPPANMYDFSFLAPCPSSGLIINGSSDRVAPPADTTSLVGKLHEQKGITITHQEVPGADHFFQDQHMDTLIGSVSDYVKRRLTENTR---------------------------MTAPVYEKVALIGLGLIASSMFWAMKRHGNLAGTVVGYARSDATRDTARRIGLCDTVCDTLAEAVEGADLVVLCVPVGAMEAVAQDMAPHLKPGCTVSDVGSVKRDVIRSVGPHLPEGVVFIPAHPLAGTEHSGPESGFAELFDNRWSLLVPADAEVPE--EDMARLRALWEGMGANVEVMDADHHDLVLAVTSHTPHLIAYTMVGVADDLRRVTDSEVVKYSAAGFRDFTRIAASDPTMWRDVFLTNKDATLEILGRFTEELFALQRAIRTGDGDLLFDYFTRTRAIRRGIIDAGQDTAAPDFGRSPRKAT-----MQLPPKVT--DRAFERLSEI-GAAQQGKA-LRIAVEGGGCSGFQYEIALDAPAD-DDLVLEGQGERVVVDSVSLPFLSNAVIDFTEELIGARFVIENPNATSSCGCGTSFSM----MDQAEIEITVHPAIAGIGADDWDACACPEAAD--------GGAPNDPFTTYRFLSALEDSGSVGPGTGWQPQYISAR-SGGQVIGVAPMYVKSHSQGEYIFDHNWAHAYERAGGRYYPKLQIAVPFTPATGRRFLLRPGHEGIAL-PALVQGAVQLAANNNISSLHATFCTQAEAEAGAQMGLMHRVTQQFHWHDDGYGDFDGFLSSLSSRKRKNIRKERAQAQGFGGTIHTFQGDDIQPEHWDAFWRFYQDTGARKWGSPYLTRQFFDIAQETLRDDIALVLAERDGRWVAGALNFIGRAALFGRYWGCTEHHPCLHFELCYYQAIDIALARGLSRVEAGAQGEHKLARGYLPSETHSLHWIADPGFSDAVARYLEAEREAVAEEIEILTEYGPFR-KANVEEQE----------MMALTHFDDTGAAHMVDVSDKASTARIATAEGHVRMAPETLALIAEGRAGKGDVMGIARLAGIMGAKQTSGLIPLCHPLPITKVSVDLTTDASLPGVRISATVKTTGQTGVEMEALSAVSIAALTVYDMVKAADKAMEIGGVRVTLKDGGKSGRYEAQ-------------------MTAQRVLELGRAAGLRVATAESCTGGMVAAALTDIAGSSAVVDCGFVTYSNDAKQAMLGVSSDTLARFGAVSEEVAREMALGALAHSRADVAVSITGIAGPGGSEH-KPEGRVCFARA---RRGGACTAETVEFGALGRAKVRAAARDHALDLLATAISETDQ-----------------------MLRIDDKTAIEDWELTEQFVRASGPGGQNVNKVSSAVELRFEAARSPNLTPPVKARLKRLAGRRWTKEGALVIQCDETRSQARNREIARTRLAELIASALTPPKKRIRTRPTKGSIRRRLDAKTRRGEVKALRGKVEDP-------------------MPSFSSTLEQAIHSALALANERRHEFATLEHLLLALLDEPDATRVMKACSVDLDELRATLVEFVDEDLSNLVTDIDGSEAVPTAAFQRVIQRAAIHVQSSGRTEVTGANVLVAIFAERESNAAFFLQEQDMTRYDAVNFIAHGVAKDPAFGESRPVSGAPEPDE----STQ-GVTEGEQKESALSKYCVDLNAKSREGDIDPLIGRESEVERCIQVLCRRRKNNPLLVGDPGVGKTAIAEGLARKIVAGETPEVLSKTTIYSLDMGALLAGTRYRGDFEERLKAVVSELENHRDAILFIDEIHTVIGAGATSGGAMDASNLLKPALQGGKLRTMGSTTYKEFRQHFEKDRALSRRFQKIDVNEPSVEDTIKILKGLKPYFEDHHSVKYTADAVKTAVELASRYINDRKLPDSAIDVIDEAGAAQHLVAESKRRKTIGTKEIENVVAKIARIPPKNVSKDDAEVLKDLEGSLKRVVFGQDKAIEALASAIKLARAGLREPEKPIGNYLFAGPTGVGKTEVAKQLSDVLGVEMLRFDMSEYMEKHAVSRLIGAPPGYVGFDQGGMLTDGVDQHPHCVLLLDEMEKAHPDVYNILLQVMDHGKLTDHNGRTVDFRNVVLIMTSNAGATEQAKAAIGFGRDRREGEDTAAIERTFTPEFRNRLDAVISFAPLPKSVILQVVEKFVLQLEAQLLDRNVTIELTRPAAEWLADKGYDDKMGARPLGRVIQENIKKPLAEELLFGKLVKGGVVKVGVKDGEIDLRI----EGPENPRLS-G-NKPPLLTADMNEITSFDGKT------ADIQSPETMPHSVEAEQQLLGAVLTNNDVYDRIASIIGPQHFYDPVHARIFEIAAARIAKNALASPVTLKAFMEDDAGLKELGGPAYLARLAANAISAFAVRDYAQMVYDLAIRRELIGLGREISEKAARVEVSSEPREQIVEAEQMLYKLSEQGRAESGFQSFLSAVTEAVNVANAAYQREGGLAGISTGLIDMDKKLGGLHPSDLLILAGRPSMGKTSLATNIAFNVAKAYKRGMRPDGTEGTIEGGVVGFYSLEMSAEQLAARILSEASEVPSEQIRRGDMTEAEFRRFVDAAKALEACPLYIDDTPALPISQLAARARRLKRTHGLDVLIIDYLQL--VRPASAKD-SRVNEVSEITQGMKAIAKELDIPVIALSQLSRQVESREDKRPQLSDLRESGSIEQDADVVMFVFREEYYKEREKPGDHDLD-----KMAAWQDEMERLHGKAEVVIGKQRHGPIGTVELSFEGRFTRFGNLIKPWQ--QS---GEQEF--MAKRWYSVSVLSNFEKKIAEQIRTSVTENGLEAEIDEVLVPTEEVIEVRRGKKVSTERRFMPGYVLVHMEMSDRGYHLISSINRVTGFLGPQG--RPMPMRDAEVNQILNRVQE-GEEAPRTLIRFEVGEKVKVSDGPFEDFDGMVEEVDEDNERLKVTVSIFGRETPVELEFTQVSKQGMTRKFDGKTILIATHNAGKLEEMRALFAPLGVTVVGAAEKNLPEPIETEDNFVGNARIKAHAASQATGLPALSDDSGIEVDALGGAPGVYTADWAETPNGRDFVMAMERTHAELEAKNAAHPRTARFCCTLVLAWPDGHDEVFEGVVNGQVVWPMRGELGHGYDPIFMPDGHDITFGEMDADTKNGMSHRANALRKLVDACLA----MAGIPHDHYEPKSNPEKWLHSRLPVVGLLYDTLM-IPTPKNLNWMWIWGIVLTFCLALQIVTGIVLVMHYTPHVDMAFASVEHIMRNVNGGFMLRYLHANGASLFFVAVYLHIFRGLYYGSYKAPREVTWIIGILIFVLMMGTGFMGYVLPWGQMSFWGATVITGLFGAIPFIGEPIQTWLLGGP--AVDNATLNRFFSLHYLLPFVIAGLVIVHIWAFHSTGNNNPTGVEVRRASKAEAEKDTLPFWPYFVMKDLFALAVVMLVFWAIVGFMPNYLGHPDNYIEANPLSTPAHIVPEWYFLPFYAILRAFTSEVWVVQIASFLTGGIVDAKFFGVVAMFGAIAVMALVPWLDTSNVRSGRYRPMFKWWFALLVVDFMALMWLGAMPAEE------------------------------------------------------------PYASFSLIASAYWFAYFLVILPILGVIEKPDALPATIEEDFDSHYGKSDTADAA--GAATPAE--MKKLSLLAASAALIAL----PTLGLAQE----------TAEAA---EPATD---FAFVLNTLLFLIGGFLVMWMAAGFAMLEAGLVRSKNVAMQLTKNVALFSIAAIMYWLIGYSIMYPGD-----------SWAIPGYL-GNLFSPYAIN----GDPDLD-TGY-SVASDFFFQLMFCATTASIVSGTLAERIKLWPFLIFVVVLTGIIYPIEASWQWGGGWL--SEAGFNDFAGSTLVHAAGGWAALAGALILGPRIGKY-KENRTIPMPGSNLALATLGTFILWLGWFGFNGGSELALGANGSANNVSIIFANTNMAAAAGAVTALILTQVLYK-KPDLTMVLNGALAGLVSITAGPLDPTMFGALWIGAIGGVIVVFTVPMLDKLKIDDVVGAIPVHLLAGIWGTLAVPF---YTEGA-----------------------TFGAQLLGIIAVGIFVFVVSGVVWFILKAIMGIRVSEEDEINGLDMAELGMEAYPEFSKG---MTTTRFAPSPTGYIHIGNLRTALMNFLIARKSGGEFILRIDDTDPERSKEEYVEGIKEDLTWLGLHWDRIERQSLRLDRYAEAADALRAAGRFYEAFETPVELDLKRKKQLNMGKPPVYDRAALALSEAEKDKLRAERGQGVWRFKLDRERIVWADGILGDISIDAASVSDPVLIRGDGQVLYTLASVVDDTEMGVTHVVRGSDHVTNTATQIQIMQALGHGH-PQFAHHSLLTGPQGESLSKRLGTLSLRDLRAKGVEPMALLSLMARLGSADPVELQTDMGRLIDGFDVTRFGAAPTKFDEADLYPLTAHYLQTLPLSEVRDHVAALGVPDDIAEPFWLMARENITTLGDLGQWWALCRDGADPLIAPEDADFIAQAMAMLPSGPFDATTWGTWTSAVKDATGRKGKQLFMPLRKAVTGQERGPEMAALLPLM---QVIRARG--------------MSFETPGPVEAGLSDAISPITDIIAAARRGEMFILVDHEDRENEGDLVIAAEFADAKAINFMATHGRGLICLPMTAERVDRLGLPMMAVNNSSRHETPFTVSIEAREGVSTGISAADRALTIATAINEQNTMASLATPGHIFPLRARRGGVLVRAGHTEAAVDIARLAGLKPAGVICEIMREDGTMARLPDLVEFAKQHGLLIGTISDLIAYRSRNDNLVDETGRETVVSEF-GGEWDMRLFTDQTHNIEHVVLIKGDITTPEPVLTRTHAMHEASDILG-IGQKS-ARELPRAMEIIAEEGR-GVVCLFRQ--P-----RRTLYAQDEEGVRT-IKHTGLGAQILSKLGVHKLVLLTDSPETRYVGLDAYDITIAGTRRILSGD-----MTTEVRVPTLGESVTEATVATWFKKPGDSVAVDEMLCELETDKVTVEVPSPASGKLADIVAAEGETVGVDALLATIAEAGEAGAETVEERPSQKAKDTKEAKDEPAKSA--------------GGGSTDVMVPTLGESVTEATVSTWFKAVGDTVAQDEMLCELETDKVSVEVPSPAAGVLAEITAAEGTTVSASAKLGVISGSGASSAPK---VQEEGANGEAYAAPA-PGASN----------RG----DVENAPAAKKAMAEAGISADKVTGSGRDGRIMKEDVQKAI-ASA-SAAP---AA-SSAPTAP-RAPVPADDAAREERVKMTRLRQTIARRLKDAQNTAAILTTYNEVDMTEVMALRNEYKDLFEKKHGVRLGFMSFFTKACCHALREVPEVNAEIDGTDIVYKNFVHMGIAAGTPQGLVVPVIRDADSMSFAEIEKAINEKGKRARDGKLSMAEMQGGTFTISNGGVYGSLMSSPILNPPQSGILGMHKIQDRPMAINGKVEIRPMMYLALSYDHRIVDGKGAVTFLVRVKEALEDPRRLLMDLMTDASI-----PTQATAGEAGFGDYFALLKPRVMALVVFTAFAGLLAAP----GSVHPLVGFCAILFIAVGGGASGALNMWWDADIDRIMRRTRNRPIPSGRVQEGEALSLGLALSGFAVVMLALATNILAGALLAFTIFFYVVVYSMWLKRSTPQNIVIGGLAGAFPPLIGWVAVTGE-MAMEPWLMVALTFMWTPPHFWALALFMRSDYDDAGVPMLTVTHGRRATRVHILVYTILLALLAVGMGFTSLGGPLYLSVAVVLNALFLLGAVRIW--RRDETDAEADNFATERRFFRLSLLYLFLHFGAILAE--AVLRPYGLG-----GWA---MADQINTLEDLKDAVTENVGGVQ-GTET-TLDPREPVRDDLGRSYATGKRKDAVARVWIKPGSGKVTVNGKPINDYFARPVLQMILRQPFSVAGVEDQFDVQATVKGGGLSGQAGAVKHGISKALQLYDPSLRSALKAAGFLTRDSRVVERKKYGKAKARKSFQFSKRMLEIKNLHVKLEEED--KQILKGVNLSVEAGKVHAIMGPNGSGKSTLSYVLSGRDGYEVTEGSATLEGTDLLELEPEERAAAGLFLAFQYPVEIPGVGNMTFLRTAVNAQRKARGQDEMSAADFLKEVRARAKDLKIDAEMLKRPVNVGFSGGEKKRNEILQMAMLEPRMCILDETDSGLDVDAMKLVSDGVNALRSEGRGFLVITHYQRLLDHIQPDVVHIMANGQIIKTGGPELALEVEKNGYADILNEVA----MIGRLNHVAIAVPDLDAAAEQYRSALGA-KVGAPQDEPDHGVTVIFIELPNTKIELLYPLGENSPIQAFLDKNPSGGIHHVCYEVDDILAARDHLKSTGARVLGSGEPKIGAHGKPVLFLHPKDFNGCLVELEQV-----------------------------MAHIIVVGNEKGGAGKSTVSMHVATALARMGFKVSALDLDLRQRTMGRYVENRREFIEQSGLELASADCHDLPEIDASTLQPGENIYDHRLSAAVARLEPDSDFILIDCPGSHTRLSQVAHSLADTLITPLNDSFIDFDLLAHTDAAGEKITGPSVYSEMVWNARQLRAQAGLKPIDWVVMRNRVGTQRMVNKEKMERAVNMLAERVGFRIVPGFSERVIFRELFPRGLTLLDLKDIGVK-QLNISNVAARQELRDMMKGLRLP---GVTVDFMEFT-SHMEAALAEARAAADRDEVPVGAVIVAPD-GQVVAQAGNRTRELCDPTAHAEILALREACAKAGSERLPGHVLWVTLEPCAMCAAAIAAARIARVCYGAADPKSGGVAHGARVFSHPQAHHAPE-VVDGLAADQSQALLRAFFAARRDA----------MKIEVRRDVFRLAEVFTISRGSRTEAQVLTVHLDDGTHRGWGECVPYARYGETLDNVQAQIEG----LPSGITRAALVDHLPAGAARNAVDCALWDLECKRAGKRAWDLAGLAAPGPRITAYTLSLGTPEAMMAQAARNAMRPLLKIKLGTPDDMPRLEAVRAGAPTARIIVDANEGWSAEVYADLAPHLTRLGVELVEQPLPAGEDEALIGMARPVPVCADESCHDRESLPKLKGKYDVVNIKLDKTGGLTEALALRTEALAAGYDVMVGCMVGSSLAMAPAVLVAQEASVVDLDGPLLLAEDRAQPLRFDEAGVHPPEPGLWG-------MPKDPDHAPIAGPRHVAIIMDGNGRWAQARGRPRLFGHHAGARRVREVVEACPDLGIKYLTIFAFSTENWKRTQVEVAGLMSLFRRYIAKEARALDSRNVRVRFIGDRVRLDAKLTGLMDQLETLTAENDGVELTIALNYGARDEVARAIRRLAEDVEAGSLRSDEVDETTLTRYLDTHVLPDPDLVIRTSGEARISNFLLWQSAYAEYEFIDTLWPDFTAAELETLCRSYGARDRRFGAVKV----------MPEVNDIAPDFTLPRDG-------GGEVSLASLRGAPVVLFFYPKDDTPGCTKESIGFSADIGAFQDAGAQVFGISKDSVAKHDKFVAKHDLKTGMLSDENGTTCEDYGVWKEKKMYGKTFMGIERSTFLIDAEGRIAKIWPKVKVDGHVAEVLDAVRAL------------MNSTTPP----------------SDGERIAKVLARRGVASRRDAERMILAGRVQVDGKRVDTPAVNIAPDARITVDGTPVRAAEPTRLWLYHKAPGLVTTERDEQGRDTVFDALRADLPRVMSVGRLDLNSEGLLLLTNDGELKRRLELPATGWLRRYRVRINGRPQDSAFDPLREGIEVDGEQFLPMQITLDRQKGANAWLTIGLREGRNREIRRAMEDIGFTVNRLIRLSYGPFQLGQLKAGEVLEIKRRVLRDQLGDELSEGLDLSGEGTGEGGGKRDKPTRRPDPR-GDRGPRKDFGGKPGYKRDDRDGDARPDRGPRKDFGKGPRKDFGGKPGYKRDDRDGDARPDRGPRKEFGNKGPRKDFGGKPGFKRDDRDGDARPDRGPRKDFGKGPRKDFGGKPHGKPGDTGGGKPAHRGKPSGS-RPQQRGKPGGQNTGRDGGKRHMDAADT-----VFDSAFWITTGGIFLLLFMSAFFSGSETALTAASRGKLRAQADKGSKGAELALKITEDNERLIGSVLLGNNLVNILAASLATSLFTRALGDSGVAL--ATLVMTLLVLVFAEVMPKTYAIINSEAAASFVAPIIALLVRVLSPIVSAVRLFVRGLLRLFGVRVDPDSNILAVREEIAGALHLGHSEGVVEKEDRDRILGALDLGDRAVEEIMLHRSGIEMIDAAQPAQAILQQCLDSPHTRLPVYRDEPENIIGVVHAKDLLRAMYKAASAPD--ADPAALRNFAITDVAMPPYFVPETTTLDDQMRQFLRMRTHFALVVDEYGSLQGLITLEDILEEIVGEITDEFDVDAEHPLKQSSDGTYLVDGAMTIRDLNRATDWNLPDEEANTVAGLVIHEAQMIPTVGQVFSFHGFRFEVVAREGNRVTQLRIRPLHPAD------MSGHGTAIPMMSSPCGPLSGTAEVPGDKSISHRSLILGAMAVGETRITGLLEGEDVLDTAAAMRAFGAEVTDHGGGAWSVHGVGVGGFAEPGQVIDCGNSGTGVRLIMGAMATSPITATFTGDASLNKRPMARVTDPLALFGTRAVGRAGGRLPMTLVGAAEPVPVRYTVPVPSAQVKSAVLLAGLNAPGQTVVIEKEATRDHTERMLAGFGAEITTEE--TEEG----RVITLTGQPELKPQVIAVPRDPSSAAFPVCAALIVPGSDVLVPGIGLNPTRAGLFTTLREMGADLSYENERTEGGEPVADLRARFSPDMQGIEVPPERAASMIDEYPILSVVAANARGVTMMRGVKELRVKESDRIDAMAVGLRTCGVDVDEGEDWWSVAGLGPGAVPGGATCESHLDHRIAMSFLVMGMAARSAVSVDDGAPIATSFPIFEPLMARLGADLRRA-----------------------------------------------------MAAQTQPDSETLLARILSSLDDDKAEDVVQIDLRGKTAIGDYMVIASGRSTRQVAAMAEKLVDRLKQDYGRLCRVEGKDTGDWVLIDTGDVVVHIFRPEVREFYQLEKMW--APTTTN-------------MSFLSDTLARVKPSPTIAVTTMAAELKAAGRDVIGLGAGEPDFDTPENIKQAGIAAINAGKTKYTAPDGIPELKQAICDKFARENGLTYTPKQISVGSGGKQNLYNALVATLNPGDEVIIPAPYWVSYPDMVLLAGGTPVAIEATLENSFKITAEQLEAAITPKTKWFIFNSPSNPTGAGYSRDQLKALTDVLMRHPHVWVMTDDMYEHLAYDGFEFCTPAQVEPGLYDRTLTCNGVSKAYAMTGWRIGYAAGPEKVIAAMRKVQSQSTSNPCTISQWAAVEALNGTQEFLAPNNATFKRRRDLVVKALNAIDGITCPVPEGAFYVYPSIAGLIGKTTAKGVKIENDEVFATALLEDTGVAVVFGAAFGLSPNFRVSYATSDENLTEACKRITEFCAALA--MTVHQPS-------PFPGTSAQVAFDRRELQDILGLYGRMVAAGEWRDYGISCLRDSAVFSVFRRTAENPLYRIEKHPRLRLRQGMYAVVGMDGQILKRGHELKSVLRVLERKLIRAVD--------------MIELDNVGYSY-GGGDLLSDISVQLAPGSFHFLTGPSGSGKTTLMKLCYGALLPTSGQVRAFDADMASLDRDQMALLRRRIGVVHQDCRFLDHLPLADNVALPLTVSGRDRGAESENLAELLAWVGLGSRTDALPPELSGGERQRAALARAVIMSPDVVLADEPTGNVDWEMSQRLLQLLVELN-RMGKTVLIASHDLTLIRAAKSHV--------------QARVLRISNRRLQLAGADLMFGIPGKQAVTLTPAAATQISRLMQRAGHSGLRIGVKKGGCAGMEYTMDYVSDTDPNDEVVEQDGARVMIAPMAQMFLFGTEIDYQTSLLESGFKFNNPNVAEACGCGESIKFN--DMTLPTGN-----MIGSANL-NIMIKAARRAGRSLVKDFREVENLQVSMKGAGDFVSRADTAAEEIIKEDLMGARPTYGWLGEETGET-EGEDPTRRWIVDPLDGTTNFLHGLPHWAISIALEHKGQIVAGVVFDAAKDEMFFAEKGAGAWMNDQ-RLRVSGRSRMIESIFATGLPFGGRADLPATL--KDLGRLLPACAGVRRWGAASLDLAYVAAGRYDGFWERRLHAWDIAAGLIILREAGGLAEPMTKGGDILSDGEVVAANEAIFASFAKVIRG----------MKIATFNINGIKARAAALPDWLDSAQPDVVLLQEIKSVDEAFPRELFEERGYQVETHGQKSFNGVAILSKLPLEDVTRGLPGDD-------SDEQARWIEATVVGKQ-AIRLCGLYLPNGN---------PAPGPKYDYKLGWMERLRARAQQLLDGEEIALMAGDYNIIPQAEDAKRPDAWREDALFRLESREAFRRIVNLGFTDAFRARVGGPGHYSFWDYQAGAWNRDDGIRIDHLLLTPQCADLLRDVGIDKEIRGFDKPSDHVPVWAEIDIMFTGIITDVGRIAALEQQGDL-RARIETAYDTSGIDIGASIASDGVCLTVIDLG---------PNWYDVQISAETVSKTNIG--AWAVGSRVNLERALRVGDELGGHIVSGHVDGVAEVIAVTPEGDSTRIILRAPDALARFIAAKGSVALNGTSLTV-NDVAGTEFGINVIPHTQAETTWGGVTVGDRVNLEIDTLARYVARLAEVA------MFKKILIANRGEIACRVIKTARKMGIQTVAIYSDADRQALHVQMADEAIHIGPPPANQSYIVIDKVMEAIRASGAEAVHPGYGFLSENAKFAEALDAEGVAFVGPPVKAIEAMGDKITSKKIAQEAGVSTVPGYMGLIEDADEAVKISNEIGYPVMIKASAGGGGKGMRIAWNDEEAREGFQSSKNEAANSFGDDRIFIEKFVTQPRHIEIQVLCDSHGNGIYLGERECSIQRRNQKVVEEAPSPFLDEATRKAMGEQAVALAKAVGYASAGTVEFIVDG-SRNFYFLEMNTRLQVEHPVTELITGIDLVEQMIRVAKGEALTITQDDIKLTGWAIENRLYAEDPYRGFLPSIGRLTRYNPPAEVAAGPLLEQGKWQGDAPSGVNAVRNDTGVYEGGEISMYYDPMIAKLCTWAPTRAEAIEAMRVALDSFEVEGIGHNLPFLSAVMDHPKFVSGDMTTAFIAEEYPEGFEGVTLPGDDLRRIAAACAAMHRVGEIRRTRVSGRMDNHERKVGTDWAVTLQGERFDVVVD-ADPKGATIRFADGPALRVEGDWTPGRSLAQMTVDGSPLVLKVGKVSGGFRIRSRGADLKVHVRTPRQAELADLMPEKVPADTSKLLLCPMPGLIVKVDVAVGDEVQEGQALCTVEAMKMENILRAEKKGIVSKVNAAAGDSLAVDDVIMEFE----MREIVLDTETTGFDPEQGDRIVEIGGVELLNHVATGRTYHQYINPERDMPQEAFEVHGLSIDFLK-------------DKPVF--AKIGQEFLDFVG-DAKLVIHNAAFDMKFLNAELKWMGLPQLPWEQALDTLAIARKRFPGSPASLDALCRRFGIDNSSRTLHGALLDSEILADVYLELIGGRQPDFALSQDTTDT-QSAATETWRPQPRPAPLPSRISQAEKAAHDAFVEKLGAEALWRRA---MADLKKLAEEIVGLTLLEAQELKTILKDEYGIEPAAGGAVMMAGPADGA-GAAAEEEKTEFDVVLKNAGASKINVIKEVRGIT-GLGLKEAKDLVEAGGK-IKEGVDKAEAEDIKAKLEAAGAEVELAMTEK--------LSDETRGPLLTPLFEAGWSLTDD-RDAIAKTFVFDDFIAAFAWMTKVAFWAEKWNHHPEWRNVYKTVHVELITHDVDGLSALDAKLARKMDSLMET------MARDKTRT-KRKVSKNIAAGVAHVNSSFNNTKILISDVQGNAISWSSAGTMGFKGSRKSTPYAAQMAAEDAGRKAQEHGVKTLEVEVQGPGSGRESALRALAAVGFNITSIRDVTPMAHNGCRPPKRRRV-------------------MHPALRAALDDRGYSTLTPVQTAVLDPALEGRDLLVSAQTGSGKTLGFGLAIAPTLLGEDDAFA-PGATPLALVIAPTRELALQVKRELGWLYAKAGVVLASCVGGMDMRDERRALERGAHIVAATPGRLRDHIMRGSIDLSAVRAVVLDEADEMLDLGFREELEFILDELPADRQTLLFSATVPPAIAALAKGYQTD-AERIVAKSETPQHTDIEYRALMAAERDMDNAVINTLRYYEAPSALVFANTRATVNRLAARLSNRGFNAVALSGELSQSERTHALQSMRDGRAQVCVATDVAARGIDLPRLDLVVHAELPSNQETLLHRSGRTGRAGRKGVSVLVVTPRTRKKAERLLGWAKL-RADWGMAPSAADVRAMDEERLFSDPSWQEVATEDEQESVNRLLEAFSPEQLAAGYLRLLHTLRAPPEELADLDAK-------PERAR------EF----------------------------------GPATWFAVSGGRDAGAEPRRLLPMLCKAGGLSKDDIGAIRVQSDQSFVQISNAAVAGFLATVGESMELEKGAQITQLASPPDLA-------GKGGGFK--------RDGA--SRK--------------TTSAKPHRHKAQ--A-SARQEAD---EAA-TPD-----RDRTY----------RASDS--------------RKDAIESPAA-----------------EP-----GNAARPY-R---KPKGPPP--PK----------------------------------------------GKPSS------KKN---RAR----------AAVTA-------------------------LGKGSGK-----PRSKAS--------------SAKRPSKS-IKPT-------------------RGGK----------------------------MTLISPACRDALQNWLDGRRALAGAADHTITAYSRDVAGFLAFMAEHHGGGQGLAALERITVSDMRAWMARERGEGLSSRSLARKLSAVKSFYRWLAEREGFEPTAVLSTRAPRFQRKLPRPLAEPAARDVIDTMGMQS-ETPWVAARDVAVVTLLYGCGLRISEALGLTGADAPLPQVLRILGKGGKERLVPVLPAARDAVENYTRLCPFKAEPTAPLFLGVRGGALLPRAIQGAMAQARMQLGLPSSATPHALRHSFATHLLAAGGDLRAIQELLGHASLSTTQAYTGVETAQLIEVYNRAHPRA----------MSLTPDLLLQGYAMGIFPMAEHREDPEIFWVDPKRRGVIPLKGFHISRSLRRRLLRGTFTATINRDFEGVLDGCAD----RAETWINAEIRDLYVTLHHQGHAHSLEIWE-GDDLVGGVYGVALGAAFFGESMFSRRTDASKAALAYLVHLLRRDGFTLFDTQFLTEHLASLGAIEISRADYHAELARALGRFARFGNGPIP------------------------------------------SAQDVVQFSTHRSMARVTVEDCVDKVPNRFELVMLAAHRAREISAGAPITVDRDNDKNPVVSLREIADETQRADDLRERLIESNQSQIEVDEPEEDSMALLM-GAEN-DKPVEDDMSEERLLRALMEAQGQG-----MVSAAEQMAANTSWAALGKATDLRNRILFTLGLLVVYRLGTFLPVPGIDGVALREFMEQAGQGIGGMVSMFTGGALGRMGIFALGIMPYISASIIVQLMTAMVPQLEQLKKEGEQGRKKINQYTRYGTVLLATAQAYGLAVSLESGDLATDP--GLYFRLACMITLVGGTMFLMWLGEQITARGIGNGISLIIFVGIIAEVPAAIAQFFASGRSGAVSPGVIVGVIVMVIATIAFVVFMERALRKIHIQYPRRQVGMKVYDGGSSHLPVKVNPAGVIPAIFASSLLLLPTTISTFSQGAASGT-------VMSWMLANFGPGQPLYLLFFVAMIVFFAYFYTFNVSFKPDDVADNLKNQNGFVPGIRPGKKTAEYLEYVVNRILVLGAAYLAAVCLLPEILRGQFS-IPFYFGGTSVLIVVSVTMDTIQQVQSHLLAHQYEGLIEKSQLR----GR--GKKRTKRSPARRMVSRVIPVDPFDLVIFGGTGDLAQRKILPGLFRRYCSGQMPVDARIIGAARSEHDSAEYRAFAAQAIREFSGGRRCEDGTLDDFLNRLEYVTIDARGTDG-WEQLASLMR--DGVVRAFYFSVAPALFGDLAQRIHEHGMATEESRIVVEKPFGHDLASARALNRTLATHFDESQIYRIDHYLGKETVQNLMAVRFGNMLFEPLWNSQYVDHIQITVAETVGVGGRGEYYDKSGAMRDMMQNHLMQLLCLIAMEPPAKFDPDAVRDEKLKVIRALDPVLPHH----IVRGQYGAQDG---EHPSYREAVGNPR-TRTESYIALKTHISNWRWAGTPFYLRTGKRLKARSSVINVLFKDTPHSIFGEEAGRHANLLTIRLQPNEGITLQVTIKEPGPGGMRLIDVPLDMTFSDALGPEEADFPDAYERLIMDVIRGNQTLFMRGDEVEAAWAWTDPIIAGWEARNDVPKPYDSGSAGPTDAEMLIQRDNRNWRGITPMKILFLGDVMGRAGRRAVAENLPRLRDEWKLDFVVVNGENASNGMGLSGDHAKGLLDAGADCLTLGDHAFDQRDMLQFIEQEPRIIRPVNY--AKGAPGRGHRLFTARNGRKVLVAQVLGQVFMKRAFDDPFSALDGVLKSYPLGGQAAAVLVDVHCEATSEKMAAGHFCDGRASLVVGTHTHVPTADAQILPGGTAFLSDAGMCGDYDSVIGMDKAEPMRRFITGMPKGRFT--PALGEATLSGVYVETDDQTGKAKRIAMIRQ----GGRLAQAAP------------------------------------------MKLTDFDFDLPEDLIATRPAVPRTSARLLLAQG---PA-LSDRRVSDLPDILRPGDRLVLNDTRVIPARLSGQR-FRSGKAGDTSARIEVTLLEPRAD-GT---WAALLKPLKKLRVGETVEFTGGLSATLTGVQDGQGLLR-FNLVGEDFDAALAKAGQMPLPPYIAQRRPADERDRTDYQTVFA-RNPGAVAAPTASLHFDDELLAALAARGVAFSHVTLHVGAGTFLPVKVDDVTTHKMHAEWGEVGETAAQEITQTRREGGRVIPVGTTALRLIETAARG-GEIAPWTGETEIFIYPGFTFHATDALMTNFHLPKSTLLMLVSALIGHDRMNAIYAHAVAERYRFFSYGDSSLLIPGE-----------------------MLTAIIDYESGNLHSAEKAFQRMAQETGAGKVVVTDKAEVVAGADRIVLPGDGAFPACAAALRGHSGLFDALVEAVETRGRPFLGICVGMQLMATTGHEYEETPGLGWIDGDVRRITTDDPSLKIPHMGWNDLMIEQTHPVLDGISTGDHAYFVHSYQMDTHAP-DQRLASVDHGGAVTAIVGRGTMIGTQFHPEKSQATGLRLIANFLSWAPMNRFVLAALGGTLAGVIATTQVA-----GPLIAQENARSSSVYEQLDLFGDIFERIRAQYVEPVDESKLIEAAINGMLTSLDPHSSYLAPDDAAQMRVQTRGEFGGLGIEVTQEEGFVKVVSPIDGTPADEAGIEAGDFITHVDGESVLGLTLDKAVEMMRGPVGSEIVITVVREGEDEPFDVSIIRDTIKLTAVRTRTQ-GETVVLRVTTFNDQTTPNLESGLKEQIEAAGGIDNINGVVLDLRNNPGGLLTQAIKVSDSFLESGEIVSTRGRNPEDGERFNATPGDLIEGKPMVVLINGGSASASEIVAGALQDHRRAIVVGTKSFGKGSVQTVMPLRGD-GAMRLTTARYYTPSGRSIQALGVSPDIVVAQPRR----PANAEQEEEPASAVSR-SRSEADLRGRLNNDSLSEDEVRQIEEDRAKAEAAAQLREEDYQLAYAIDILK-GLSAIDHRN--------------------------MADGGIDINAKPTEEISVREVFGIDTDMKIKAFPERTDRVPEVDSTYKFDRDTTLAILAGFSHNRRVMIQGYHGTGKSTHIEQVASRLNWPTVRVNLDSHISRIDLIGKDAIKLRDGKQVTEFHEGILPWALRNPTAIVFDEYDAGRADVMFVIQRVLEHDGKLTLLDQNEIITPNKYFRLFATSNTVGLGDTTGLYHGTQQINQAQMDRWSLVATLNYLSHDAEVAIVLSKAPHYNNEKGRKTVSQMVTLADLTRTAFMNGDLSTVMSPRTVITWAQNAEIFRDVGYSFRLSFLNKCDELERQTVAEFYQRCFDEELPESAASVSLG----MSD-PLTSGATIGILGGGQLGRMLSAAASRLGLRTHIYEPGANPPAGDVAHRVTTASYEDEAALAAFADSVDVVTYEFENIPTAALDLLD-SKAMIRPGREALRISQDRLTEKTFLQGLGLKTAPFADISDLPGLEAALATIGAPAILKTRRFGYDGQGQARLKTPDDAASALAEMRGAPAILEGFVTFSREISVIAARGVTGEIACYDPGENVHVDGILHTTDVPARLTPAQRSDAVLLAANILNALDYVGVLGVELFVTDAG-LVVNEIAPRVHNSGHWTQNGCAVDQFEQHIRAVAGWPLGDGTRHSDVQMENLIGEAVLGAHDLAKESATALHLYGKTEVKAGRKMGHVNRIRSAGK-------MRRKLAAGNWKMNGLSGDLGVLADLAAAH-G-EGDV-----DILICPPHTLVNRAAQTVADGKIRIGGQDCHAAETGAHTGDVSAAMLVDAGAKWVIVGHSERRTDHDESDSDVRNKARAAIDAGLGVIVCVGESLAQREAANTLDIIGGQLSGSIPDLVTGQNLVVAYEPIWAIGTGKVPTTDEIGEVHDFIRARLERRFGAGVARSVRLLYGGSVKAGNAADIFAVSNVDGALVGGASLTAADFSPIVTALEQAAKAMTT----TVLDKIKAYKLEEIAADKAAKPMEEIEAEARAASPVRPFADAIAKATREG-YGLIAEVKKASPSKGLIRADFDPAALAAAYEQGGATCLSVLTDTPSFQGAKPFLAEARDACELPVLRKDFMYDTYQVAEARALGADCILIIMASVSDAQAAELEEAATEWGMDALIEVHTAAELERAAKLNSRLIGINNRDLKTFETSLDTSRSLSKLVPTDRIIVSESGLSTPDDLSEMARYGIRSFLIGESLMRQSDVAQATTDLLANPLTP--GAM-MTFTLAIVGRPNVGKSTLFNRLVGKRLALVDDQPGVTRDLREGAARLADLRFTVIDTAGLEDANDDSLQGRMRRLTERAVDMADVCLFMVDARTGITPTDEMFAEILRKRAGHVLLAANKAEGAAADAGVIDAWGLGLGEPIRLSAEHGEGLNDLYTQLM-PLADAF---AERASED---A-PD------------------TDVDLDED---DDT---DEAAP--ARKPTKSRPLQVAVVGRPNAGKSTLINRIIGEERLLTGPEAGITRDSISLQIDWQ-G-----TPMRLFDTAGMRRKAKVQEKLEKLSVSDGLRAVKFAEVVVVLLDAAIPFEQQDLRIADLAEREGRAVVIAVNKWDIEEDKQDKLRELKEAFERLLPQLRGAPLVTVSAKTGRGLDRLHGAILRAYDVWNRRVTTAQLNRWLTGMLEQHPPPAPQGKRIKLRYMTQAKTRPPGFVVMCSHPDKMPDSYSRYLVNGLREDFDMPGTPIRLHMRGQSDKNPYKGR-RERNAGALTKHLGKNRKG----------MQDDNSPIVVIGGGPAGLMAAEVIAAAGQPVLVAEAKPSLGRKLLMAGKSGLNLTKSESRDVFDAAYAEAAP-WLSPMLDAFGPDQVQDWARGLGVDLFTGTSGRVFPMAMKASPLLRAWSARLGSAGVEFRTRWRWAGWDESGA----LTFDTAQGRETITPAATVLALGGASWARLGSDGAWAGLLAARGVPLAPFAPANAALAVDWSRFM-GPVLGTPIKGVAFRSGA-----YTSRGEGVIAAKGLEGSGIYAVSRGVRE------GHMLSIDLMPDVPLPEIAARLARPRGKTSLSNHLRKVLSLSPAKAALLNEFAR-PLPNMPEVLAATIKGLTVHHAGLRPIDEAISTAGGVPQAALNDHLMLRALPGVFCAGEMLDWEAPTGGYLLTGCLATGQAAGRGVLRHLGI------------------------MSDD----WTNRTKAIHAGTRRSQYGELSEAIFLTQGFAYDSAESAEARFIQTGDDE-FIYARYGNPTVRMFEERIAALEGAEDAFATASGMAAVSGALTAMLRAGDHVVSARALFGSCLYILEEVLGRYGVEVTFVDGTDLDQWRDAIRPDTKAVFFESVSNPTLEVIDVAGVAELAHAAGAKVVIDNVFATPVFSKAFALGADVVIYSATKHIDGQGRALGGVILGSREFIRKTVEPYMKHTGGSMSPFTAWIMLKGLETMSLRVNAQADSAQAIAEALVDHPALDRVIYPGLSGHAQHALCAQQM-GRGGTVLSLDLKGGQKAAFAFLNALQIILISNNLGDAKSIVTHPATTTHQRLSDAQKTTLGITPGLIRLSLGIEDTGDLLADIRQALDAAAG---MDIASNPKPH-----DRLADALAEDMAAVNVLIRERMKSEHAPRIPEVTAHLIDAGGKRLRPMLTLAAARLCGYQGSDHVKLAATVEFIHTATLLHDDVVDESKQRRGRPTANLLWDNQSSVLVGDYLLARSFLLMVETGSLRVLEILSNASATISEGEVLQLT--AASDLKTDESIYLQVARGKTAALFSAATEVGGVISGAPDDQVAALRAYGDGLGVAFQIADDLLDYQGDSAATGKNVGDDFRERKLTLPVIKAVAQATEEERAFWVRTIEKGNQQDGDLEHALELMGKYGTLEATRLDAVAHAERAKQALSAL--PDSDMRAMLMDLADYVVDRLS-------------------MSKKMLIDATHAEETRVVVVDGNKVEEFDFESENKRQLAGNIYLAKVTRVEPSLQAAFVDYGGNRHGFLAFSEIHPDYYQIPIADRQALMEEERAYAEAQQARE---------------EGDEDKPKS-KSRSRSRSRS-KSSASAVTSD------------DAVETREV-------ETEINGMETIDL--------------SDGDAPAEDETSAAVEPQSEAEA--------------------------DASPVQDESAEADK-------------------------------------------------------------------------------------------------------------AKHDDDQDIEDDGAA-ASASDKDSTIESVADEDDPEDLRPPRKPRPRRYKIQEVIKVRQVLLVQVVKEERGNKGAALTTYLSLAGRYCVLMPNTARGGGISRKITNAADRKKLKEIASEIDVPTGAGLIVRTAGAKRTKAEIKRDYEYLLRLWEQIRELTLKSIAPTKIYEEGDLIKRSIRDLYNRDIDEVFVEGERGYRIAKDFMKMIMPSHAKNVKLYNETLPLFARYQVESYLAGMFNPTVQLKSGGYIVIGVTEALVAIDVNSGRATKEGSIEETALKTNLEAADEVARQLRLRDLAGLIVIDFIDMDERKNNASVEKRMKDRLKTDRARIQVGRISGFGLMEMSRQRLRPGMIEATTQPCTACHGTGLIRSDDNMALSILRQVEEEGTRRRSREVLVKCPVGIANFLMNQKREHIAQIEARYGMSVRIEGDPHLVSPDFSLEKFK---TASRSIPVSTAPVVSANAALMSDLDEEEDDTVEAEEAPEAPQA------ETPEA-----------------DGETKPKRKRRRRRR-RSKSSS--------DSD----GDTGDNGENGDTD----------DSEPDEAP-----AAEAEAA-ADP------VVADAAPEAEEEA-PKPAK-RS--RSSRSRSSKS--------KAKEAEVAEGAEPSAADAP-AEDAPAEEK--PKVKRTRSSSRSKTAK-------AVAEAEAAPEATAEAAPEVKSATAPETA-------EAPAVEAAPEPVETPA----------------------AEVSPEPAEAQTAE----VTPEPETA-PEAEATP------EPVA---------EAAP---EPVAAPEP-------------EPVPAEADEAP----KPKRRGWWSLGAKMPLTYLHTMVRVKDLEKSMAFY-ELLGLRETRRHESEEGRFTLVFMAPE-------DQPEC--P-------VELTYNWDGDDALPDDSRHFGHLAYGVDNIYDTCQFLMDNGVTINRPPRDGRMAFVRSPDNVSVELLQNGDSLPKAEPWASMGNTGHW--MTSDSQNTTHFGRETIPETEKAGRVRGVFNSVASKYDVMNDVMSVGIHRIWKDAMMDWLAPRPGQRLLDVAGGTGDISFRFL-------KRAGE---GHSTVLDLTEPMLIEGRKRAEAEQMADSLDWVVGDAMALPFADNTFDVYTISFGIRNVTRPAEALAEAYRVLRPGGRLMVLEFSQLPNEGLQKLYDLYSFNVIPRMGQIIANDRDSYQYLVESIRNFPDQETFLSMIRAAGFEQARYRNLSMGIAALHSGWKIMAKQPMLKFVGIERDMPEKREAEERRADFREIYAEYADAKAKEQASRCSQCGVPYCQSHCPLQNNIPDWLRLTAEGRLQEAYAVSQATNTFPEICGRICPQDRLCEGNCVIEQSGHGTVTIGSVEKYITDTAWEEGWVTPANPSVERAESVGIIGAGPGGLAAADVLRRAGVQVTVYDRYDRGGGLLTYGIPGFKLEKDVVMRRVEQLEKGGVTFKLNCAVGEDISFAEIREAHDAVIIATGVYKSRDLEMPGSGATGIERAIDFLTASNRK-SFGDTVDDFESGRMNAAGKRVVVIGGGDTAMDCVRTSIRQGAESVKCLYRRDRANMPGSQREVANAEEEGVVFEWLSAPKGFT-SKGE--------------NVAGVMVQKMRLGAPDASGRQAPELIEGSDYVEDADLVIKALGFEPEDLPALWDQPELTVTRWGTIRAEFTTGQTDLPGVYAVGDIVRGASLVVWAIRDGRDCADAILTSFDAA-PQ--MAAEMRVHICAVGRLRKGPEKALLDDYLTRFDRTGRALGL-GPANVIEVEDRKGGGMAAEANLLRRAIPQGAVTLALDERGKPMTSPDFATRLADWRDQGRGDLALLIGGADGLDPSLRDEADALLSFGAMVWPHMLVRVMLSEQLYRAATILAGTPYHRV-MPMMDSPMGILALCAAIGYLLGSIPSGIVLARLMGLGDLRKIGSGNIGATNVLRTGNKGAAALTLILDALKGVVAVLIARRLGGG-DAAQMAALAAFLGHCYPVWLGFRGGKGVATFLGVWLALAWPVGLAC-CATWAVAAAVGRISSASALVAAALSTVWAVLLGYGDMLVLG--AVLTLLVFWRHRANIARLRAGTEPKIGKK-------------MTDLTLKIVNEKGLHARASAKLVEVVEAFDAQAEVSRDGMSASGDSIMGLLMLAASRGTSIDVRTSGPDAEALANAIQALVADRFGE--PMMPPLQEAVQAADDA---------ALRQRVATLNDRWRHHSATDVMRAALRD--TGKIALVSSFGAESVVLLHMAAVIERNVPVLFIDTEMLFTETLVYQTEVSERLGLTNLRVIRAADIAAE--DPDNTLHLRDTDACCDLRKTRPLNAALAEFDGWITGRKRFQSGTRAALDFFEVEDGTG----RIKVNPLAHWAPEDVRSYMEENRLPRHPLVAKGYPSIGCAPCTSPVKPGEDPRAGRWRDQNKDECGIHFVNGKMVR----------------LGAGQ--------------MQVILLERVAKLGQMGDVVDVKPGYARNYLLLQGKALTASQANIDSFEAQKAQLEARNLETRKEAEDMGNRLDGQQFVVIRQASDGGSLYGSVTTRDAAEAASEAGFSLDRKQVVIEQPIKELGLHTVNVTLHPEVDVTITLNVARSPEEAELQASGKSI-QELAAEEEAAAEFEISELFDDIGGAAADDFGDDAP-------------AAQPEAEE----EPKE---------MTTGDTADTRAWVLHPDLKSDPDRRPSGPALEEAVALGAALPGLQIVGGTVVPLPKAHAGLLFGSGKIEELATLFQAQEIELVLIDGPLSPVQQRNLERKWKVKILDRTGLILEIFSDRARTREGVLQVEMAALSYQRTRLVRAWTHLERQRGGLGFVGGPGETQIEADRRAIDTQVVRLKRQLDKVVKTRALHRASRAKVPYPIVALVGYTNAGKSTLFNRLTGAEVMAKDMLFATLDPTMRRVELEN-GPEIILSDTVGFISDLPTELVAAFRATLEEVLAADLVLHVRDISHPATEEQAHDVAAILASLGVEND---RPQFEVWNKLDLLTPDERDAARARAERQDD--VFAISALDGEGLDVLLARIADQLAGAMFEEEITLGFDAGRQRAWLFEQDVVIEERQTED-GFAFTVRWSGIQKARFDAL-------------MEGDIAQLGQFIGAGLAAIGSGTAAIGVGHVAGNFLAGALRNPSGAASQTATLFIGIAFAEALGIFSFLVALLLMFAVMTLEILTSDLL---APLRHGFFTRRGGASSGVFTGLNCGPGSSDQADVVTINRTRVAEALGLPLTGLMSVDQRHTATVVTV-TEPTE-TRPVADGMVTNHPGIALGVLTADCQPVLFADHQAGVIGAAHAGWRGALDGVLEATLDAMETLGATREDTVAVIGPCISQSAYEVGPEFLDDFMAEDPANARFFANGEGDRMMFDLPGFGLHKLREAGVGTAEWSRHCTYADEARFYSYRRSCHRKEADYGRLISAIRL--MAQSETHYSLPRPEFDAPVKLLIVVAPYYKDIADQLVAGAKAEIEAAGGTWDLVEVPGALEVPSAIALADRLSNFDGYVALGCVIRGETTHYDTVCNDSSRAL-QLMGLQGLAIGNGILTVENRKQAEVRAEAAGQNKGGGAAAAALHLIALARKW---GAQRKGIGFKPESGS--YRIAGD-DNGTPTAMSDFDMMGLPAKLVARLAAMGIAEPTPIQAQSIPHALNGRDVMGLAQTGTGKTAAFGVPLVARMLEYGKKPEKHTVRGLILAPTRELAGQISVHLRGLCEDTPMKVGMVVGGQSIGAQIKRLERGTDILVATPGRLIDLLERNALTLESCDFLVLDEADQMLDLGFIHALRKIAEMLPSERQTMLFSATMPKQMNELAQSYLKSPIRIEVSPPGKAADKVTQAVHFIAKAEKTSLLIELLDTHRDELALVFGRTKHGSEKLMKTLESKGFAAASIHGNKSQGQRDRALAAFRAGDIKVLVATDVAARGLDIPDVKHVYNFDLPNVPENYVHRIGRTARAGRDGAAIAFCAPDEMGELKAIQKVMGITI-PVASGRAWEPMEEA---KPAGRRAGPSRRRPGGKPGQKQGQGQN-----RGGGK----PAHAGGKPAG--ANA----GAKSGRPRRAKS---------NRGRSAA---MANAKGKVTQVIGAVVDVQFDDALPEILNALTLDNNGKKLVLEVAQHLGENTVRTIAMDATEGVVRGQEVLDTDGPITVPVGTATLGRIMNVIGEPVDEKGPVEADEHRPIHAPAPDFAEQSTEAEVLVTGIKVIDLLAPYAKGGKIGLFGGAGVGKTVLIQELINNIAKVHSGLSVFAGVGERTREGNDLYHEMIESGVLTPDNLPDSKIALVFGQMNEPPGARARIALTGLTMAEQFRDATGTDVLFFVDNIFRFTQAGSEMSALLGRIPSAVGYQPTLATDMGAMQERITSTKNGSITSIQAVYVPADDLTDPAPATTFAHLDATTVLNRAISELGIYPAVDPLDSTSRLMDPQVIGEEHYQVARDVQGILQRYKSLQDIIAILGMDELSEEDKLTVARARKIQRFLSQPFDVAKVFTGSDGVQVPLEDTISSFKAVVAGEYDHLPEGAFYMVGGIDDVKAKAERMAADAA---------------MTTLDALAATIESRK-SADPDSSWTAKLLSRGPEKCAEKFGEEAIEAIIEAVKGDRARLTSEAADVLYHLLVMLAARDVPLSDVLSELDRRQGTSGIVEKSARTT---MAIGELTPPHPTAPQEVLIEFPDNRLMVDLCGEHSRNLAIIEDALGVQIVHRGNQLAMFGS--AQDRAAEVLRALYARLEAGREVTPGDIDRELRM-G---AGRAVPEGDQLEMFRG---GKFEIKTRKKTVEPRTDAQKAYVQSLFENELAFGIGPAGTGKTYLAVAVGVSMFISGQVDRIILSRPAVEAGEKLGYLPGDMKDKVDPYMQPLYDALNDFLPGKQLAKLIEEKQIEIAPLAFMRGRTLSRAFVVLDEAQNATTMQMKMFLTRLGEGSRMVITGDRTQIDLPRGVSSGLADAERLLKSIPKISFSYFTSKDVVRHPLVAAIIEAYEADAKV--------------------MSFTVAIDGPAAAGKGTISRAVAAHFGFAHLDTGLLYRAVGARVLAGEAPLEA-------AHALVAEDL-DADGLRTPEVAQAASRVAALPDVRSALVDFQRAFAARAGGAVLDGRDIGTVICPDAPAKLFVTASAECRATRRYDELRGNGHDVTRDEVLADVIARDKRDSERATAPLIAAEDAVTIDTSDMSIEEAVAAAVAAILSKDGRPEN----MTDDHR-SPFRDAHADRATATHVPDTPQTRAPAYRLAFADDDFLCREELRPVRLQLELLKPALMLDENNIESTVVLFGGARIPEPSKRDTARTQTLADLSEFYAEAQEFARLMTEKSNA-SGGR---KHVIVTGGGPGVMEAGNRGAADAGGKSIGLNIVLPHEQAPNRYVTPDLCFNFHYFAIRKMHFLMRAKAICVFPGGFGTLDEMFEALTLIQTGRMERVPFLLFGRDFWEKIINWDALAEAGTISADDLDLFRFVETAQDAVRLIDNWEPA-PPRDRIPGR--------MGEMRFTILGCGSSGGVPRLGGHWGDCDPENPKNTRRRCSLLVERETEDGI-TTVLIDTTPDLRQQLLDTGTGRLDGVVYTHSHADHVHGIDDLRMIVFNMRARLPVWADGDTQNALFSRFGYAFVQPEGSPYPPILNMK-TIS-GP-FTVTGDGGPITFRPFRVSHGSIDSLGFRIGDLAYLPDVADIPEDAWAELQGLDTFIVDALRRAPHPTHSHLDNTLDWIAQLDPRQAVLTNMHIDLDYDTVCAETPDNVTCAYDGMILRVPV------MPVSSEYTPPDTPLDILHEDADLLVVNKPAGLLSVPGKGEHLADCLISRLAGAFPQVLLVHRLDRDTSGVMVFALTAHAQRHLGLQFEKRMTRKTYHARVAGRVEPRSGTVDLPLCVDWENRPRQKVDHEQGRAALTDWRVVRHE-------GDSTRIRLFPKTGRSHQLRVHMLSLGHPILGDPLYASGEALN-HDRMMLHAEELRLKHPDGGRSLSFRVKAPFMIGIVIVAHGGLAREYLAAMEHVVGHQPGLKAVSIEPDHDRDAKQQEICAAADEVDLGDGVVVVTDLFGGSPSNLSLLACRPNNRRILYGANLPMLIKLAKSRQ-LPVGDAVRTAMDAGKKYINTQNVSTD------------MSKLLHPKATAVWLVDNTTISFKQIADFVGMHELEIQGIADGDVAAGVKGFDPVANNQLEQREIDQAQNSPLHRLKL-KPNAAAAGEEKRRGPRYTPLSKRQDRPASILWLVKFHPELSDGQISKLVGTTKPTIQAIRERTHWNIANIQPIDPVALGLCKQSELDTAVQKAAAKRAAEGAVMTDDERRRLVSTEQSLSMDTEPRLPTAIEGLETFTLSPSS----DDDEDEDRKSPADFSDADSFFNLPDN---DDDEDEDDR-------MAYHSRS-RDPLFDSNMQEAIEKRGKELFGIALICLGLAAAAMIGSYTPDDPNWMVSTDAPVQNWLGRFGASLAAPLFMIVGWGSWGIALVLGVWGLRFAFHNGEDRAMGRVIFAPIAIALASIYAATLVPGEPWRATHSFGLGGMFGDTVMGALLTLLPVGSHLMVK---LTSLVMAVAMLAMGAYVLGFERRDMARGGRFALIGLILGYSGLRTLLGRGASGSISALGTQYARAAER---RAQRRADKAEALAARAEYE-------------DDVIYD------------DEDDDDGYEPIRIVSHQTAE-----------PRGGLLSRVPG----------LIRRA--------DPVPELVEPEPVE-GDFDEMPGD-DRISAKIANIVKSR-RPAEP----PAPDPDR--PLTKG--RGRGPEPLLLNRG--G--SQGRVEPPLTAA--HAA--------------PR-ASDPAVAA---------PALAPVVE-------------------------------RATPRVEAPMAEIEPD---------------PLPLAEP----VAPP-------MAAIPVAEPRK-VVQNPARKAPQPSRRAQAEAQPALAFD--ESA-TEFDLPPLGLLSDPKAIQRHHLSDEALEENARMLENVLDDYGVKGEIVSVRPGPVVTMYELEPAPGLKASRVIGLADDIARSMMALSARVSTVPGRSVIGIELPNDNREKVVLREILAARDFGDSNQSLPLALGKDIGGDPIVANLAKMPHLLIAGTTGSGKSVAINTMILSLLYKLTPDECRLIMIDPKMLELSVYDGIPHLLSPVVTDPKKAVVALKWLVGEMEDRYRKMSKMGVRNIEGYNGRVRDALAKGEMFSRTVQTGFDDDTGEPVFETEEFAPEAMPFIVVIVDEMADLMMVAGKEIEACIQRLAQMARASGIHLIMATQRPSVDVITGTIKANFPTRISFQVTSKIDSRTILGEMGAEQLLGMGDMLYMAGGAKITRCHGPFVSDEEVEEVVNHLKAYGPPSYVNTVLEGPDDDSASD--IDAVLGL--GGNTDGEDALYDTAVQIVIKDRKCSTSYIQRKLAIGYNKAARLVEQMEDQGVVSPANHVGKREILVPE-QH------MPKMKTKSSAKKRFKVTATGKVLTAQAGKRHGMIKRTKKFIRDARGTTTLSAPDAKIVKGYMPYDRMARPKIALIGAGQIGGTLAHLAALKELGDVVLFDIAEGTPEGKALDIAESGPSEGFDATLKGTQSYADIAGADVCIVTAGVPRKPGMSRDDLLGINLKVMKSVGEGIRDNAPDAFVICITNPLDAMVWALREFSGLPHEKVCGMAGVLDSARFRHFLATEFNVSMKDVTAFVLGGHGDTMVPLTRYSTVAGVPLPDLVSMGWTTQEKLDGIVQRTRDGGAEIVGLLKTGSAFYAPATSAIEMAEAYLKDQKRVLPCAAYCDGVLGLKGMYVGVPTVIGAGGIERIIDIKMTKDEQAMFDNSVNAVKGLVEACKGIDSSLA------MTTIQMIDAAAARLKGHARRTPILTSPFLDEIAGRRVLLKAECLQHTGSFKFRGGWSAVSALEPEVRAKGVIAFSSGNHAQGVAAAAKGHGVPAVIIMPEDAPALKIANTRALGAEVVLYDR-ATGDRDAIGADLAQRRGLTLIRPFDEPLVIAGQGTVGLEIADDA---GVEQADVLVCCGGGGLTSGIALALEARAPGLRVRPVEPQGFDDVARSLAAGAIQRNDRLSGSICDAIITPQPGDITFPIMSRLCGPGMAVTEEEALRAMALAFQRLKVVLEPGGAVALAAALFH-GDSIEGDAVIAVASGGNVDAAMFARALETLD-------------------MSELATLYDQRIAQGKLTRDPAQLAVLEEFDRIHAGLDQPVKRG----FFRRSTPEPVKGLYLWGGVGRGKSMLMDLFVESLSDVAVRRVHFHAFMQEIHAGMHAAR--------------KANTQDALAPVVQALVQDLRILAFDEMQITDITDAMIVGRLFEALFEAGVTVVTTSNRPPEDLYKNGLNRQLFLPFIDLINNRMAVRELASDTDHRQNRLSGSQVYFTPIGPQSRADIDAVWDDLA-GGADTPLVLTVNKREVTLPRYRNGVARAKFYDLCGKPLGPADFLAIADALRVLVLEDIPRLSRSNFNEAKRFVTLVDALYEARVRLIASAAAEPEMLYVEGEGTFEFERTASRLREMQDKDWGTTD---------------MTFDYDLFVIGGGSGGVRAARVAAGDTGARVALAEEDRYGGTCVIRGCVPKKLMVFASGYGRMAEEARSYGW-DMQAVGFDWLAFRGKLHAELDRLEGIYRRLLANAGVETFDARAVVKDAHTVQL-STGEVKTAKHILIAVGGWPTLA---EIPGAELAITSNEIFHLDALPKSMLIVGGGFIACEFAGVMARLGVQVTQFYRGEQILRGFDDEAREMVAQSLRDQGVTLLTGINVAAMRRDGEG------------------------------------------IQVDDTEGKIHHFDKVMFATGRSPNTAGLGLEEVGVKLAKNGAIEVDAYSQTAIPSIYAIGDVTDRVNLTPVAIREGMAFVETVFKGNPTPVDHDLIPSAVFTQPELGTIGLTEAEAAKQ-EPIEVYSTSFRPMQSSFAGGEEKVLMKLVVSKATRRVLGCHIVAPDAGDMIQLAGIAVKMGATKEDFDRTVAVHPTMAEEIVTMKAP-VRTT-----MSGQNAVT-----PSAEGIAASVRAAG-----KSRGLPPVHLWNPEFCGDLDMRIARDGTWFYLGTPIGRPELVRLFSTILKKEGD-DYFLVTPVEKVGITVDDAPFVAVDFEATGE-GRDQVLTFATNVGDKVSAGADAPLRVERDAETGEPSPYVLVRANLEALIDRKSFYRLVEIGEHHD-G---WFGLWSGGDFFPVIPSKELPT---MALTPDFDSFARAYSAGENQVVYTRLAADLDTPVSLMLKLAGNQTDAFMLESVTGGEVRGRYSIIGMKPDLIWRCRGTTSEVNRQARFDPEAFTAMEGNPLDALRALISESRIDLPDDLPQASAGLFGYLGYDMVRLVEHLPHINPDPIGAPDAMMLRPSVVAVLDGVKGEVTVVSPVWVSEAQSARAAYAQAAERVMDAVRDLERAMPGDSRDLGDEEDMAPPSSNFTPEGYRAAVDTAKEYIRAGDIFQVVPSQRWTQEFRHPPFSLYRSLRRTNPSPFMFYFDFGGFQVIGASPEILVRVFGREVTIRPIAGTRPRGATPEQDRALEAELLADAKERAEHLMLLDLGRNDTGRVSKIGTVRPTEQFIIERYSHVMHIVSNVVGELDDGKDALDAFFAGMPAGTVSGAPKVRAMEIIDELEPEKRGLYGGGVGYFSAGGDMDMCIALRSAITQNGKLYIQAGGGVVYDSDPEAEYQETVHKSNAIRRAAADAARFR-TGGNRMPYRAPVSDYEFLLRHVVGYDRVSGTERFAEADADVASAILTEAGKMCEEIMAPLQRNGDLTPARLENGVVRTSPGFAEGWKAIAEGGWIGMSAPEAYGGMGLPMTLTTSVNEMMSAACLSLQLAPLMSQGQLEALEHHASDQLKELFMPKLISGEWSGTMNLTEPQAGSDVGALSSKAEPKGDGTYAISGQKIYISWGDNDFADNVCHLVLARLPDGVPGTKGISLFLVPKYLPREDGTPGEANSLKVVSLEHKMGLHGSPTCVMQYDGATGWLVGAEHGGMAAMFTMMNNARLGVGGQGVGAGEGAYQHALAYATERKQGRTP-KGDSGTIADHADVRRMLTAMKGDLFAARAIMLACAVAIDMSTATGAPEWKA----RAALLTPIAKAFGTDVGIDVAQMGVQVHGGMGFIEETGAAQYYRDVRVTAIYEGTNGIQAMDLVGRKMMDG-GEAAGALMDEIEAAAEAARAT----HPALAEAVWQATETLRETTEWLVGQ---DMQDRFAGAVPYLRAFARVLGAHFHLSAAVAEP-G----GPRAA---LAAFYIQRLLPEHAGLLTQVHVGAEGLYDLSLDDLVA---------------MASTNTVHVALGARSYDVQIGPGLIARAPDLMAPLLKRPRVAIVSDENVAARHLGALEQVLKDAGIKVDSLILPAGESTKSWPHLTRCVEWLLDQKIERRDIVIALGGGVIGDLVGFASAILRRGVRFVQIPTSLLAQVDSSVGGKTGINTSHGKNLVGAFHQPALVLADTDILQTLEPRDFLAGYGEVVKYGLLGDAAFFDWLESHAVD-MAGGDMSARVHAVTRSVQMKAEIVARDETEQGDRALLNLGHTFCHALEAATGYSDRLLHGEGVAIGCALAFDLSAALGLCSQEHPSRVRAHLLGLGMKTELSDIPGDLPDAQGLLNLMAQDKKVQDGQLRFILARDIGAAFVADDVPPETVHSILTEALAARG---MTFKLGLTGSIGMGKSTTAQMFRDEGCEVWDADAAVHRVYAAGGSAVGPMRAAFPQAIVDGA-----VDRGALKTIIAADPTALKTIETIVHPLLKQDRADFLANAT---ADILVYDIPLLFETGGNAGMDAVACVSVDAQTQEARVLARGTMTAEQFQMIREKQMPDAEKRARADYVIVTDT-LEHARAQVQNIVSTIRAGLRNA--MRSFDFAPLHRATVGFDQFADLMDRLLTSDVSQPTYPPYNIEKTAADAYRISIAVAGFSESDLMVEVKEKALVVSARKA--EED-TNRSFLHRGIATRAFERRFHLADHVRVTGATHVDGMLHIDLEREVPEALKPRRIQIASTT-PTAQETDVV--------EAKSVN------MPFTLATWNINSVRLREPIVLKLLAEEAPDVLCLQECKSPIDKIPLQGFHALGYTHMIGRGQK-GYNGVAILSRLPIEEVGAADYAALGHARHIAGRLENGVTIHNFYVPAGGDVPDREVNEKFGQKLDYLTEMRDAFHA-DAPEKSILVGDLNIAPREDDVWDHKKLLKIVSHTPIEVEHLAQAQDAGKWVDVTRADIPDGL-LYSWWSYRARDWDAADKGRRLDHVWATPDISGAAHSS--RVLRDAR-GWDKPSDHAPVFATFDL---------MTIEKLNLSDLKAQSPKDLLAMAEELEIENASTMRKGEMMFQILRERADEGWEVSGDGVLEVLQDGFGFLRSPEANYLPGPDDIYVSPDMIRQYSLRTGDSIDGVIKAPDDNERYFALTNVTQINFEDPERAKHKIAFDNLTPLYPDERLKMEIE-DPTIKDRTARIIDLVAPIGKGQRSLIVAPPRTGKTVILQNIAASIEKNHPECYLIVLLIDERPEEVTDMQRSVKGEVVSSTFDEPATRHVAVSEMVIEKAKRLVEHKRDVVILLDSITRLGRAFNTVVPSSGKVLTGGVDANALQRPKRFFGAARNIEEGGSLTIIATALIDTGSRMDEVIFEEFKGTGNSELVLDRKIADKRVFPAIDILKSGTRKEDLLVDKVDLQKTFVLRRILNPMGTTDGIEFLLSKLKQTKSNAEFFDSMNTMARDYPLQRYRNFGIMAHIDAGKTTCSERILFYTGKSHNIGEVHDGAATMDWMEQEQERGITITSAATTTFWQRQEEPT------KDGTSDTKYRMNIIDTPGHVDFTIEVERSLAVLDGAVCVLDANAGVEPQTETVWRQADRYKVPRIVFVNKMDKIGADFFNCVRMIEDRTGARAVPVALPIGAENELEGLVDLVTMEEWVWKGEDLGASWEKNPIRDSLKDVADEWRAKMIEAAVEEDDEAMMEYL-EGNEPDVDTLRKLLRKGTLALHFVPVLGGSAFKNKGVQPLLNAVIDYLPSPLDVVDYMGFKPGDEEEVRNIARRADDDMPFAGLAFKIMNDPFVGSLTFTRIYSGQLRKGDTMLNSTKGKKERVGRMMMMHSINREEIEEAFAGDIIALAGLKDTTTGDTLCNEKDPVVLETMTFPDPVIEIAIEPKTKNDQEKMSQGLQRLAAEDPSFRVETDIESGQTIMKGMGELHLDILVDRLKREFKVEANIGAPQVAYRETISKEVEHTYTHKKQSGGSGQFAEVKLIITPTEPGEGYSFESRIVGGAVPKEYIPGVEKGIKSVMDSGPLAGFPVIDFKVALIDGKFHDVDSSVLAFEIAARMGMREGMRKAGAKMLEPIMKVEVITPEDYTGGIIGDLTSRRGQVTGQEPRGNAIAINAFVPLANMFGYINTLRSMSSGRAQFTMQFDHYDPVPQNISDEIQAKYA---MDYGFTTAA--YVVAAVLFILSLGGLSGQESAKRAVWYGIVGMALAVFATLI----GPGSGLWLLSLLLIAAGGAIGWYVAKKVEMTEMPQLVAAMHSLVGLAAVFVGFNAHFTMKAVQKAHAA-------------------------------GEAI-----TGEFAQL-VAHKTPVELSILSVELFLGIFIGAITFTGSVIAYGKLAG-------KVTSTATK--LPG---------GHVLNASAAAISVLCLFWYAQSGG--FLPLLLMTLAALFIGYHLIMGIGGADMPVVVSMLNSYSGWAAAAIGFSLGNDLLIVVGALVGSSGAILSYIMCKAMNRSFISVILGGFGGTT-GPAMEVEGEQVAI-----EADGVAQALNEADSVIIIPGYGMAVAQAQQGVAELVRKLRAKGKNVRFAIHPVAGRLPGHMNVLLAEAKVPYDIVMEMDEINDDFPDTDVAIVIGSNDIVNPAAQEDPNSPIAGMPVLECWKAKQVFVSKRGQGTGYSGIENPLFFKDNTRMFYGDAKASLDKLLPMID-----MRARIFKPARNAMQSGTAKTRDWVLEYA-PASARSVDPLMGWTSSSDTQSQVRLRFATKEAALEYAESKGIDAQVIEPKTRKPNVRPGGYGENFATNRRAVWTH-----------------------------------------------MGKTLAVSPLAP-ASFPELPVISGVRFASAEAGVRY-KN--RTDVMLAVLAPGSTVAGVFTRSATRSAPVLDCQSKLA----AGGGASD--AGAAILVNSGNSNAFTGARGQTSVAELCMAAEKATGVPQSRIFTASTGVIGEPLPH-ERIVAKLDELTGNLSEDAIKGSARAIMTTDTFPKGACKSVTIDGKTVQIAGIAKGSGMIAPDMATMLVYIFTDAVVEQGALQSMLSSLTDRTFNCITVDSDTSTSDSLMLAATG-ASGV------DAS-NSDAFRTALSDVMLDLAHQVVRDGEGATKFVEIRVTGAASDADAKVHGLAIANSPLVKTAIAGEDPNWGRVVMAIGKSGAAADRDLLSIWFGDVLVAEKGWVSPDYKEADGAAHMKGQDLVIRVDLGLGDGASTVWTCDLTHGYIDINADYRS----MAMTGPGKTVVKLVGVVALMGGLAWASVPFYSWFCKVTGFGGVPGVSASGADHVLDRTIKVRFDASTDRDMAWDFKPVERTMEIKIGETGLAFYEAHNPTDRPIAGTATYNVFPYEAGGYFIKIQCFCFNEQVLQPGETVQMPVTFYVDPEIVDDIDAKHVNAITLSYTFY----------ETDLPEGL-ASLD----AGQQNNLN-------MLRIQDISYSVAGRPLFETAAATIPTGHKVGLVGRNGTGKTTLFRLIRGELALEGGS--ISLPSKARVGGIAQEVPSSDVSLIDTVLSADTERSALMAEAETATDPHRIAEVQTRLADMDAWSGEARAASILKGLGFDDEDHLRPCADFSGGWRMRVALAGVLFAQPDLLLLDEPTNYLDLEGALWLESYLARYPHTVIIISHDRGLLNRAVGSILHLEDRKLTFYQGNYDTFAETRTARLAVASAEARKQEARRAHLQSFVDRFRAKASKAVQAQSRLKMIAKLKPITTPQEAALRAFTFPEPEE-LSPPIINIEGGSTGYED-KTILSRLNLRIDQDDRIALLGKNGQGKSTLSKLLSERLSLMSGKMARSSKLRIGYFAQHQVDELHIDETPLDHLRRLRPTESP-GKWRSRLAGFGLNANQADTLVGQLSGGQKARLSLLIATLDAPHMLILDEPTNHLDIESREALVEALTGYSGAVVLVSHDMHLLSLVADRLWLVSEGTVKPYDGDLESYRELLLSRDR----PQKKN-----EPVKTA----EKPKKLG--REAMAALRGEVKKGEQRIEKLNEMRDKLATKLADPSLYETERKGEIEVWQKKYAEVMEGLDRAEALWLSAMEKLETAQSAGS-------------------------MDRAQKEKVVEELGQIFESSGVVVVAHYVGLTVAEMQDLRARAREAGGSVRVAKNRLAKIALDGKPCASIADLLTGMTVLTYSEDPVAAAKVAEDFAKENSKFEILGGAMGENALDRAGVTAVSKMPSREELIASIVGCIGAPASNIAGAIGAPASNIASILSTIEDKAAA---------------MSDMETEKARAAAWFRDLRDQIVAGFEGLEDSHGTGPLSDAEPGRFEVTPTRRAAPDGGDAGGGLMSVMRGGRVFEKVGVNVSTVFGTLGERAQAAMAARKGLPGMAEDPR-FWASGISLVAHMQNPHAPAVHMNTRMFWTPHAWWFGGGSDLNPCIEYP-----EDTAHFHAAQETALAPHGADLYPRLKAWADDYFFIPHRGRARGVGGIFLDDHNSGDWAADFALIQDIGRAFLPAFVPLVETRRTQDWSEADKHTQLVHRGLYAEYNLVYDRGTKFGLETGHDANAVLMSLPPMAKWV-----------------------------MAGPDDSFERAAMARGFLRIAGVDEVGRGPLAGPVTAAAVVLDMAKLPEGLNDSKKLTARARDRLAKAIHAQAEV-SVAHASVAEIDELNILRASHLAMVRAVAAL-----------D-P---APDYVLIDGNMVPVGLA---IAHETIVKGDARSVSVAAASIVAKTCRDAIMVDLAQQHPGYGWETNAGYPSKRHILALSEIGVTPHHRRSFKPVHNILYQDKIVNGMSGIEQKTTPKGASRGAERLR-KTVVLVGMMGAGKTAVGKALALRLGVPFLDSDHEIERAANMTVPEIFARDGEPFFRLKERQVISRLLDEARGVLSTGGGAFLAEENRRMISERGVSIWLNANTDVLWQRVRHRDSRPLLRTANPRETLENLFAERQQFYAQADIAVVSDAGASIEDMVDRVIAALGT-RPDVLTPQ---------MDKILFSFGHGYSAQALARLLLPRGWRIIGTTRTAEKAESLRETGIEPVIFP--GDGGVPPGLEQATHLLISAGPTADGDPVLNALGDTIARIAPRLDWAGYLSTTGVYGDHDGGWVDETTPLAPSTRRGRWRMQAEADWQAV---PGLPLHIFRLAGIYGPGRGPFAKVRAGTARRIIKKGQVFSRIHVDDIAQIVAASIDRPDPGAIYNCCDDDPAPPQEVIAHAAELLGLPVPPAVLYDEAEMTPMARSFYAESKRVRNHRIKQALGIRLLYPDYRSGLRALLEQDPPTE------------------MGKVIGIDLGTTNSCIAIMDGSQPRVIENSEGARTTPSIVAFTDD-ERLVGQPAKRQAVTNPDNTIFGVKRLIGRRNDDADLAKDKKNLPFNVIDGGNGDAWVEAKGEKYSPSQISAFILGKMKETAESYLGEEVTQAVITVPAYFNDAQRQATKDAGKIAGLEVLRIINEPTAAALAYGLDKENTQTIAVYDLGGGTFDVTILEIDDGLFEVKSTNGDTFLGGEDFDMRIVNYLADEFKKEHQVDLTKDKMALQRLKEAAEKAKIELSSSSQTEINQPFISMG-SNGQPLHMVMKLTRAKLESLVGDLIKASLKPCQAALKDAGISASDIDEVVLVGGMTRMPKVVEEVTKFFGKEPHKGVNPDEVVAMGAAIQAGVLQGDVKDVVLLDVTPLSLGIETLGGVFTRLIDRNTTIPTKKSQIFSTAEDHQSAVTIRVFQGEREMAADNKILGQFNLENIPPAPRGMPQIEVTFDIDANGIVSVSAKDKGTGKEQKITIQASGGLSDDDIERMVKDAEENAESDKARRELVDAKNQAESLIHSTEKSMEEHSDKVDPTTIEAIELAIAALKDELETEN--ADKIKSGIQNVTEAAMKLGEAIYKASQDEGE--DEPKGADMGGS---DDDIVDADFEDLDDNKRA--MPNPTAAMLVIGDEILSGRTRESNMNHLAVQLSAHGVDLKEARMVADDHPRIVEAVRALSSHYDHVFTSGGIGPTHDDITADAIAEAFGDRIDVREDARALLAEHYARTGRELNAARLRMARIPDSAALIENPVSIAPGFTIGNVHVMAGVPSVFEAMVASVLPTITGGAPLVSESLRVDR-GEGDIAEALGELAARYPDVSIGSYPF-LLDGRHGSNIVFRGADGARVRSALAELAALMGEPE------------------------------------------MTVTDNTPDPQEAERRSALPEAAQRALAEADDRRRAEAARP---PLPVELGGR-DGPEPVRYGDWEKKGLAIDF---------------------------MEGGAIAQFLPLILIFGIMYFLLIRPQQKKVKQHQAMVEALRRGDKVVTQGGIIGEVVKVKE--GGEIEVEIAEGVKVRVIKSTIATVMSKTEPAK---------MSEVPQITDRAALTRNR---ARA--APE--ALFLHRAAIDEVQDRLAMVNRTF----------------------TSPAVVTPFPGLWSSAMPEA---TIVADDSHLALEPGAHDLVIHAMALHWANDVVGQVIQCRRALQADGLFLAVTLGGQTLAQLRAVLAHAESTVSGGLSPRVAPMPEIRDLGAILQRSGLNLPVADSVPLTAEYRDLRHLMHDLRAMGEANALSGRLRHPTRRALFDVANRLYIDQFAT----GQG-------------RILATFELVCLTGWAPDESQPKPLRPGSAQARLADAL-------------GTSETP---L---PD--MNIILLGPPGAGKGTQASKLVETRGMVQLSTGDMLREAKASGTEMGKRVAEVMDRGDLVTDEIVIGLIREKLEAGGS-AGFIFDGFPRTLKQADALGELLEDMGQKLDHVIELRVEDEALVHRIVNRA------EEARAAGQPV-----------------RADDNEDSLRTRLAAYYKMTSPLVGYYYAKGLYCRVDGMAQMEAVQAEIGQVLDGTRAMADADA----MPFA-FA-DTAE--NAIPLHVIETAALD----DWCVDQTETVVNWVRLNGFTGKLGSALLVPGETGP--IAVVGYGDADARARGRYALAGAVKKLPEGTYDIVW--GLPE---HLLETECLGWLLAGYRFDRYNKKKDRDVRLVAPAGVDADRVRIIAEAETLLRDLVNTPTSDMGPDALEAAARDLAEKHGAAFSSVVGDDLLDKNFPMIHAVGRASATPPRLIEFTWGSDG-PLLTLVGKGVCFDTGGLDLKGASNMLLMKKDMGGAANVLGLAHMIMALKLRLRLRVLIPAVENSVAGNAFRPGDILTSRKGLTVEINNTDAEGRLVLADALALAAEDEPELVISMATLTGAARVALGSDIAPFFTDDDAGADTLSRAAREIDDPVWRMPFHTPYESKIEPGIANLDNAPSGGFAGAITAALFLRRFAGEGR-YMHFDIYSWSSFNLPARPKGGCGQGIRALLQAMPDLLAK------------------------MNYKQVRLAPGLHFVATPIGTARDITLRALDTLASADVLAAEDTRSLRHLMDIHGVPLSGRRIIAYHDHSGASVQDRLVAEAVQGKSVAYASEAGMPLIADPGFELGRAMAAAGQMVTVAPGASAVPAALVLAGLPTDSFFFAGFLPNASAARQTRLMDLAQIPGTLVFYESPKRLRASLADMAESLGGERQAAICREITKKFEDVRRGTLQDLVAGMADAPDPKGEIVVVVDRAGSEKISELDLENDLRTALETHSVRDAAEIVSKIHGLPKRDVYQMALQMGRP------MILLVGLGNPGPKYARNRHNIGFMALDQIAEDHGFAPWRGRFQGSLTEGRLGDDKALLLKPETFMNLSGQAVGEAMRFYKLTPEDVWVVHDEIDLPPGKVKLKRGGGHAGHNGLRSVHQHIGPEYGRVRIGVGHPGRKEAVPGYVLHDFAKADEAWIDDVLRGLSDGAPDLARGDAARASNAIALRVAPPRPSTGQ-------KKPKAAPE---------AAVKETEGESRDTATEPPA-----------DTRSPLEKLLDRFK--MPDLLIELFSEEIPARMQTRAAEDLKKRMTDALVEAGLTYAGAASFSTPRRLVLTVGGLLAASPAQREERKGPRFDAPEKAIEGFLRGAGVT--REALIERDTPKGAVFFAVIDKPGRPAAEIVAEALEQVIRDFPWPKSMRWGSGSLRWVRPLHSILCILTDEAG-AEVVPLNIDGIPSGNTTRGHRFMAPDAFSVSSFEDYEAKLK-RAHVTLSAQERADQIKSDAENLAFAAGLEVVEDAALLAEVAGLVEKPVVLMGDIGADFLTLPPEVLQTSMREHQKFFSVRNAKTGRIE-KFITVANRETADNGATILAGNQKVLAARLSDAKFFWENDLRVAKA----DINAWVQSLENVTFHNKLGSQAERISRIAALARELAPVTGADAEAADQAARLAKADLSSEMVYEFPELQGLMGRYYIESAGLPEDVAAVAQDHYSPLGPSDTVPTAPLSVTVALADKLDTLAGFWAIDEKPTGSKDPFALRRAALGVIRLVLENGLALSLGEAIARANSGAD--------------------------------------------------------------------------------------------------------------------------------------------------VADLTGFFHDRLKVYLRDQGIRHDIIDACIAMPGNDDLALLARRAQALSDVLKTEDGENLLQGFKRANNILSQAEAKDGVEYSFGADRKLARD--PAEVALFDALDAAEARIAPALKAQDFAEAMAAMAALRGPVDGFFDAVMINDENDIVRRNRLNLLSQIRQTCTRVADLTKIEG----MKGA-MSRVRLI---GAVIAVSVL--AACGGGGIGGGPAA---------DRSESLEAYSPDQIYERGEYELSRNRTDDAAYYFSEVERLYPYSEWAKQGLIMTAFAFHQGKDYEESRAAAQRYIDFYPTEEDAAYAQYLLALSYYDQIDEVGRDQGLTFQALQALRTVIEVYPDSEYATSAVLKFDLAFDHLARKEMEVGRYYLKRSHFTAAINRFRVVVEDFQTTSHTPEALHRLVEAYLALGLDEEAQSAGAILGHNFQSTEWYEDSYALLTGRGLQPRALGDNWLARIYRQTVKGAWLMRIASDLADAIGNTPLIRLRRVSEETGCTILGKAEFMNPGQSVKDRAALFIIKDALEKGTLKPGGTIVEGTAGNTGIGLALVGASMGFKTVIVIPETQSQEKKDMLRLAGAELVQVPAAPYKNPNNYVRYSQRLAEELAQSEPNGAIWANQFDNVANKRAHIEGTGPEIWEQTGGKVDGFVAAVGSGGTLVGVAEVLQPK--GVKVALADPMGAALYSFYTEGEFK-SEGNSITEGIGQGRITENLKGFTPDYAYQIPDEEALPYVFDLLSEEGLCLGGSSGINVAGAVRLARDLGPGHTIVTILCDYGNRYQSKLFNPEFLAEKGLPVPTWLDRAPASIPGVFVDG--MYVVTCLTSPKNPGLDHTLVDSLRAAWGGHQVRWLALNEAAEFTLAEVP-----SNRWEVWEDLQKLG-VDLVVQPVEG-RRKRMLLADMDSTMIQQECIDELADEAGVGARVKDITARAMNGELDFEGALIERVGLLANLPEAVIARVLDTRITLMPGGPELVGTMRANGAYAALVSGGFTAFTRVVAAKLGFDENRANTLIAENGALTGEVGRPILGRQAKIEALEQITQRLGISESEVIAVGDGANDLGMLGRAGTGVALHAKPVVAAECDVRINFGDLSALLFIQGYARADFVTG-MAE---IDE--QIVDATPEDDGEDDAYALNRKAVAAILYAVDIDDRDKLTELMEPLHAADIADLLEQINSYDRARLIRLYDREFDGEILSELEESVREEVLGILSPQVLAEAVRELDSDDVVDLVEDLEEDQQDAILNALDESDRLAVQQALSYPEGSAGRLMQREVVMAPEHWNVGQAIDHLRGS--ENLPDQFYHIILVDPRLHPIGNVTLGRLMAARRDVLLADLTEDMFRTIPVDQDEEDVAYAFNQYHLISAAVVDDEGRLVGQITIDDAMIVLDEEHEEDILRLAGV-GESSLADRVVDTVKGRFPWLAVNLVTAILASMVISQFDVVIAQFVALAVLMPIVASMGGNAGTQSLTVAVRAIATRDLTGSNVWRVIRREVLVGLANGVFFAVAMGIIGLIWFNSFGLALVIGAAMVINLVVAGLAGTVIPVLLDKFGVDPALASGAFVTTVTDVVGFFAFLGFAALYLLMATEILMPALSPTMEEGTLAKWLVKEGDTVSSGDILAEIETDKATMEFEAVDEGTVGKILVAEGTEAVKVNTPIAVLLDEGESADDIETSSGGDATKSEQAPEPD-------------KPAAQQPQAAPEIKA---ADTSPDWPEGTAMKQQTVREALRDAMAEEMRRDGDVYLMGEEVAEYQGAYKISQGLLDEFGPKRVIDTPITEHGFTGIAVGAAFGGLKPIVEFMTFNFAMQAIDQIINSAAKTLYMSGGQMGAPMVFRGANGAAARVGAQHSQDYAAWYMQIPGLKVAMPYSASDAKGLLKSAIRDPNPVVFLENEILYGQSFDVPDLDDYTVPFGKARIWREGTDVTIVSFGIGMRYALEAADKLADDGIDAEVIDLRTLRPMDTASILQSVKKTNRCVTVEEGWPQPSVGSYISSVIMQEAFDYLDAPVINCTGKDVPMPYAANLEKHALVTTEEVIEAVKKVTYK-MFAVIKTGGKQYKVASGDILRVEKLAADAGETVQFNDILMLG-GDKTTVGAPLIEGAGVQAEVIEQIKGDKVINFVKRRRKHGSQRTRGHRQKLTLVKITEILASGADKSGIKAAIGASAAGTSATVSE------------------------------------------------KAPAKKAKP---AAKAEGA----------------DDLTKLSGVGPALEKKLHGAGVTTFAQIAAWTDADVAAMDEQLSFKGRIEREGWIEQAKELSKG---------MDN-YINLIKLSVGTDSVEQLDEWQAMRRATAHADGLNRHVTRMWPKRADDILKGGSIYWVIKGLVQCRQRIVRLDEVTGEDGTRRCAIVLDPSLIRTQTAPKRPFQGWRYLPASDAPPDLPDHRQGEDSLPDDLNRALAEIGVLMTAQPQTAPAYPAQKA-----RRIVISVDAMGGDSGAQAVVAGMAESAAINPDIAFLLHGPRAELEPLVARR--SAQLDGRVDYCDVPDVVTMEDKPSHVVRHGNGTSMWSAVQSVRDGAAAAAVSCGNTGALMALSMLRLRRLPGVNRPAIAILWPSRNPQGFNVMLDVGADVRADAGDLLQFALMGAAYARTGLALERPRVGLLNVGTEEHKGRAELKEAHGLISTCAAAGGFDFVGFVEGSDIPGDVADVIVTDGFTGNVAIKTGEGTAGLIGDLLREAFRYSFLSRLASLLALTSLNRLKTRIDPRRVNGGVFLGLNGTVVKSHGGADATGVSAAIKLAFKLAQSGFSEKLAARVASAAELTQNARETPEKTADEQKEGRA-MALEKAFNAADAEDRLYRAWEEAGCFKAGANAKPGASAYSIMIPPPNVTGSLHMGHAFNNTLQDILIRWKRMKGFDVLWQPGTDHAGIATQMVTEREMAANGEPTRAEMGREEFLKRVWDQKVKSRGTIIGQLKRLGASADWSREAFTMSGAPQ-APEGEGG--NFHDAVIKVFVDMYNKGLIYRGKRLVNWDPHFETAISDLEVENIEVAGHMWHFKYPLA------GGETYEYVEKDEDGNVTLRETRDYISIATTRPETMLGDGAVAVHPSDERYAPIVGKLCEIPVGPQEFRRFIPIITDEYPDPSFGSGAVKITGAHDFNDYGVASRNDIPMYRLMDTKGRMRDDGAPYAEAAARAQEIA----------NG-----AAFTGAEVDTLNLVPDELRGLDRFEARKQVVAQITAEGLAVMVPG--------------EPGEDGEVHP-------IPLVEAKPIMQPFGDRSKVVIEPMLTDQWFVDTAQIVGPALDAVKNG---------TVRIMPESGERTYYHWLENIEPWCISRQLWWGHQIPVWYGFDLGVHR----FKDDEGDGALDAVELGRLLNEGGM------------LHA-GEVAHCGATLDDVTRAFLHELDD-TP--------TPINHSRVVEVADRQAA----IETLAASLAEYNV-------------TQD---PTH-LVYPVWRDPDVLDTWFSSGLWPIGTLGWPEQT------PELARYFPTSTLVTGQDILFFWVARMMMMQLAVVDQIPFSTVYLHGLVRDAKGKKMSKSLGNVVDPLDIIDEFGADALRFTNAAMASLGGVLKLDTQRITGYRNFGTKLWNFARFAEMNGVFEAAA------AGVP-----APAQTVNRWIVGETARVRAEVDEALENFRFDDAASALYRFVWGKVCDWYVEFSKPLLQGED--AAAMAETRETMKWVLDQCLVLLHPIMPFITEELWQTSGQ----RDGMLMLTDWPAYG-PEAINAEADREMTWVIALIESIRSARMQMHVPAGLQVPMVVSDIDAAGQAAWDRNQTLIMRLARIESLTPVDSFPKGTITIAAEGASFGLPLAEIIDIAEEKARLEKSLGKLAKELGGLRGRLNNPKFAESAPEDVVAETRANLAAREEEEARLKEALARLAELG-MTEKKHEQDVAFIKALAELLRENDLTEVQVKRDYGEDDSLNVRVSRMTAPAPV----AAAPVAAAPAA-AAPAPASAPVAA---AAEASEDPASHPGAVTSPMVGTVYMQPEPGAPSFISVGAQIGEGDTLLIVEAMKTMNHIPAPRAGKIKRILVEDGAAVEFGTPLVIIE-----------------------------------------MTCLDGLAPLP---LSDAMHHACR-GGKGLRGFLVMESARLHGVDAARALWPAAAIEAMHAYSLVHDDLPAMDDDDLRRGLPTVHIKWDEATAILAGDALQALAFELVSDARCGE-AEARAALSLGLARAAGARGMVLGQALDIAAEAAGTPLDLDAITRLQAGKTGALIEWAATAGAVMA---GADTGPLGRYARDLGLAFQIADDILDVEGDEAKTGKRLQKDAAAGKATFVSLLGLDAAKRRARDLVTSACDALSSYGAGAETLRETARFVISRES------MSKIDTPDSFRAESFDDAKAAVDRLIELYDEATDFLCSRFAQVLQEG-APGHRLRA---F-YPEIRFTTTSFAQVDS-RLSFGHVTTPGTYSTTVTRPDLFRAYLTQQIGLLIRNHGQPITIGPSHTPIPIHFAVARRPDLSVPQQGASNFTLRDVFDVPDLATTNDDIVNGT-FTVSD-GVGPLSLFTAQRVDYSLARLAHYTATDAEHFQNHVLFTNYQFYVAEFEAYARAMLADPDSGYSSFVATGNVEITDADTEI-----EPPLKLPQMPTYHLKRADGSGITLVNIGVGPSNAKTATDHIAVLRPHAWLMVGHCAGLRNTQALGDFVLAHGYLREDHVLDDDLPVWTPIPALAEIQTSLEQAVAEVTELSGYELKRIMRTGTVATIDNRNWELRDQSGPVQRLSQSRAIALDMESATIAANGYRFRVPYGTLLCVSDKPLHGELKLPGMASDFYNTQVSRHLNIGIRAMEMLRTMPLERLHSRKLRSFDETAFLMDLNKFTERARGFVQAAQTIAMRDGHQRLVPEHLLKALMDDDQGLASNLISRAGGQPARVVEVLTLNLGKQPKVGG-DAGQVYLDSATGKVLAEAEKLASKAGDSFVPVERVLMALC-MVKSGAKDALDAGNVSAQKLNEAINDIRKGRTADSATAEDGYDALKKYARDLTEAAEQGKIDPIIGRDEEIRRAMQVLSRRTKNNPVLIGEPGVGKTAIAEGLALRIVNGDVPESLRNKRLMALDMGALIAGAKYRGEFEERLKSILTEVTEAAGEIILFIDEMHTLVGAGKSDGAMDAANLIKPALARGELHCIGATTLDEYRKYVEKDAALARRFQPVTVSEPTVEDTISILRGIKEKYELHHGVRISDSALVSAATLSHRYITDRFLPDKAIDLVDEAASRLRMEVDSKPEELDQLDRQILQMQIEVEALKLEDDTASKDRLEKLEHELAELQEKSAEMTARWQAERDKLAGARDLKEQLDRARADLEIAKREGNLAKAGELSYGVIPQLEKQLAEAENRE--ENGMMVEEAVRPEQIAAVVERWTGIPTSKMLEGEREKLLRMEDDLHRRVIGQNLAVTAVANAVRRARAGLNDENRPLGSFLFLGPTGVGKTELTKAVAEFLFDDDQAMVRIDMSEFMEKHSVARLIGAPPGYVGYDEGGVLTEAVRRRPYQVVLFDEVEKAHPDVFNVLLQVLDDGVLTDGQGRTVDFKQTLIILTSNLGAQALSQLPDGADAAQAKRDVMDAVRAHFRPEFLNRLDETIIFDRLGRQDMDAIVDIQLRRLTRRLASRKITLDLDQSAKTWLADEGYDPVFGARPLKRVIQSALQNPLAEQLLAGDILDGTTVPVTAGAEGLIIGDRVAATNRPRPDDAVVHMEFERPSL-ARPLGIGTRGSPLALAQARETRDRLAIAFDLPAEAFSIVVIQTTGDAV----QDRPLKEIGGKGLFTREIEQALLTGQIDIAVHSMKDMPVLQPEGLVLDCYLPREDPRDAFVSPTLTGLDGLTDGAVVGTSSLRRRAQVLARRPDLRVVEFRGNVQTRLRKLDEGVAGCTFLACAGLNRLGR-SDVPATPIPPEAMLPAVAQGAIGIERRAD-DSRAAAMLEAIHDSETATRLAAERAFLAALDGSCETPIAGLADLSGGTLRLRGEILRPDGSEVLTEDATCPTEDGGK----LGREMALRLLDQAG---PGFFDWRGATG---MFLKKVEGPRTITLPDGTTMSRADLPPANTRRWVASRKAAVVRGVIYGLITRGDALNTYALSDEEFDGWVAAVADHGEEALKVTATKKFRQV----------MTIALVILAAGKGTRMNSDLPKVLHPIAHAPMLAHALRAGAALEPARTVVVAGHGADQLQAALAEIDEMAEVALQSEQLGTAHAVDMARETLAGFEGDIVVLYGDTPFVRPETLERMIAARQS-HDMVVLGFDTADTTARYGRLVM-EGARLQRIVEYKDASEDERALTLCNSGLLACDATVLFDLIAEVGNENASGEYYLTDVVELANARGLSVTAIACDESETLGVNSRADLAAASAVFQANARAEALDNGVTMAAPETVYFAFDTVIGRDCTIEPNVVFGPDVTVESGATIRAFTHLEGCHVSRGATVGPFARLRPGAELSENAHVGNFVEIKNAIIGEGAKINHLSYIGDADLGARTNVGAGTITCNYDGVMKHRTTIGTDVFIGSDTMLVAPVTVGDGAMTATGTVVTRDVEPGAMAVARARQENKPGFATRLFDKLRAAKARKSEG----MTRIDAKFADLKSKGQKAFVAYVMAGDPDYDTSLEIVRGLPGAGVDVIELGLPFTDPMADGPTIQLAGQRALEGGMTLDKTLALARAFREGDTTTPIVMMGYYNPIYSRGVPKFLEQAKEAGIDGLIIVDLPPEEDSELCIPAQEAGLNFIRLATPTTDDARLPRVLQNTSGFVYYVSITGITGSAEAQADDVAPEVARIKAGTDLPVIVGFGVKTPETAKAIAGVADGTVVGSAIVSQIEHGKSVADVLAFVRGLAD----------GAHSA------MRILGIDPGLRNMGWGVIEQDGPRLRHIANGICHSE-GADLAVRLMSLYDQMSEVVRAYAPDVAAVEQTFVNKDGAGTLKLGQARAIALLVPAQAGLQVGEYAPNSVKKTVVGVGHADKRQVLHMVCLQLPGCKPAGPDAADALAIAICHAHHAGHG--GAARLRRKIA-----------------------------MAERDGIFAGDDPFAIARDWLAAAQDSEVNDPNAMALSTVDSDGLPNVRMVLLKDIEPDAFVFYTNYGSIKGAELAASGKAAFVIHWKSLRRQIRVRGLVEKEDGAKADAYFASRSLQSRLGAWASEQSQPLKSRATLMAEVAKVTATKGPNP--ARPPFWGGFRITPLEIEFWADGAFRLHDRFRWRR-AD--PTASWGIQRLNPMSTLTKFLMIGSML-AMAACT-NAGRFGAD---------GTG--ASGVGSG-AIVPGSANDPTSPAYFNQTIGDRVLFLVDQSNLSPEAQQILNGQAQWLSQNVDYVAVIEGHADEQGTREYNLALGARRANAAREYLISRGVAGNRLKVVSYGKERPIEICSEEACYAKNRRAVTVLSAG-VLG-------MTVTETIRDRLQAAFAPHALDVVDDSERHRGHAGYQDGGESHFNVRIRAAAFEGQSRIARHRAVHDALGKDLVSRIHALSLDIDS--------MTSLTPITFAETDIDRIATAEGRVVVIVTPDGK-LDVAARRANRLSKGAIARLLESERFSKAKAGSVITLEMPVGLAAEALDVL--VLPRNAD-PLAARKAGATLGA--LK-AGKPALVMLGGLKNAADLALGLALKDYTFDAH----KTGEDTGKAPA-EVTLCHNAHEELAQAAHPLLAVAEGVHFTRDLVNEPANVLTTTEFANRLDEMKALGLKVEVLEEDELEKLGMRTLLSVGHGSDSPSKVVVMQWNGGEKGEAPLALIGKGVVFDTGGISLKPAAGMEDMTMDMGGAGVVSGVMRALALRKAKANVVGLVGLVENMPSARATRPGDVVRSMKGDTVEIINTDAEGRLVLCDVMWYAQDRFSPRGMIDLATLTGAIIIGLGHENAGVFSNDDALCNAFLKAAEAEGEGAWRMPLGKAYDDQLKSRIADMKNVGGRPAGSITAAQFLQRFVKEGTPWVHLDIAGVASVKSA-TDYAPKGATGWGVAALNRLVADMLEKG----MTRIFVDADACPVRAEAERVATRHRIPLVVVSNGGIRPSPNPLVETMIVSAGADEADKWIADACGPADVVITADIPLAARCVEQGARVLRHNGEAFNEMNIGQQLAMRDLMADMRAANPLGQGGGGKPFTKADRSRFLDALEREVRAAHRT-------------------------MTQ-THDRLLIIDFGSQVTQLIARRLRELNVYCEIHPYQNVSDAFLAEFAPKAVIFSGGPDSVTREGSPRPPQSVYDLGVPILGICYGQQVMMHDLGGKVERGH-----GTAEFGRAYVTPDDQRIDLLAGWFL--EAREQVWMSHGDHVSQIAPGFEVYGTSPNAPFAITADLSRNFFAVQFHPEVHHTPNGKTLYENFVR-LAGFKGDWTMDAYRDEAVRRIREQVGDAKVICALSGGVDSSVAAALIHEAIGDQLTCVFVDHGLLRLNEAEEVVGMFRDHMNLSVIHAQEQDLFLGELEGVSDPEVKRKTIGRLFIDVFQKYADQIDGARFLAQGTLYPDVIESVSFSGGPSVTIKSHHNVGGLPEKMGLTLVEPLRELFKDEVRALGHELGLPASFIGRHPFPGPGLAIRCPGEITRDKLEILRKADAVYIDQIRKHGLYDDIWQAFVAILPVRTVGVMGDGRTYDFACALRAVTSVDGMTADYYPFSHDFLGETATRIINEVPGINRVTYDITSKPPGTIEWEMDPRNRPYRSALYIPASKERALDKARGLACDAIIFDLEDAVTPEEKPAARATLAKAIETGGYGNRARIVRINGLDTQWGAEDAEAVAAMGVDAVLLPKVESPDHLTALAEIT-----GE-LPIWAMMETPVGMLNATQIAAHP---KLEGMVMGTNDLAKELQCRFRPDRLPMLTGLGLCVLAAKAHGRVIVDGVYNAYLDEAGLEEEATQGRDMGFDGKTLIHPAQVEIANRAFAPSEDEIDLARRQIAAFEETEASGQGVAVVDGRIVENLHVVTAREILAKAEAIAELAAQG---------------MTQ----------------------------------APIALSCGEPAGIGPEIAARAWDALRDSC--PFVWIGDPRHLPEGT-PVEVVSAPADAI-ALSARALPVLHHPFAAAATPGQPTPENAAGVISAIERGVALVQSGECAAICTAPIHKKALIDGAGFAYPGHTEFLAALAGVD-RV----VMMLASDQLRVAPATIHIALSEVPRCLTPDVLRDTIRITHDGLRARFGIAAPRIAVAGLNPHAGEGGAMGREELDWIAPLIDEMRAEGLDISGPRPADTMFHTAARAGYDAAIAMYHDQALIPIKTLDFDRGVNVTLGLPFVRTSPDHGTAFDIAGRGIANPTSLIEALKLAQRMAQGAA------------MLYWLTALSDGGDFFNLFRYITFRAGGAFLTALVFGFLFGRPLIAVLRRTQGKGQPIRDDGPEGH-FVKAGTPTMGGLLIVGALTTATLLWARLDN---PFVWLVLFVTLAYGLIGFADDYAKVSKQNTKGVPGKLRILLGILIAGVAAF-------------------------LASAFHP-------------AELQNQLALPVFKNTLINLGILYIPFAICVIVGSANAVNLTDGLDGLAIMPVMIAASTLGVIAYAVGRVDFTEYLDVHYVPGTGEILIFTAALFGGGLGFLWYNAPPAAVFMGDTGSLALGGALGAIAVVTKHELVLAVVGGLFVVEALSVIIQVLYFKRT------GRRVFLMAPIHHHYEKKGWAEPTIVIRFWIISLILAMIGLATLKVRMAMQAHEIEDLIRASFPAAQITITDLAGDGNHYAAEVIDESFRGQNRVQQQRAVYAALKGKMDGSSGELHALALTTKAPE--------------------------------------------MRHTLPIA--------PQFYVTAPQPCPYLDGRMERKLFTALQGENADRLNNSLSQQGFRRSQNVLYRPSCAECSACLSARIDVRRMTLSRSQRKILRRNEHLTRKTTSPWATEEQFTLFRRYLDERHADGGMADMDVFEFAAMIEETPIRSRVIEYT---DELRE------LQAVSLTDVLDDGLSMVYSFYTPDRPRAGLGTFMILDHVRLAQELDLPYVYLGYWVPGSQKMGYKANFSGLEIYLGGEWQPMNDPEAY-SHETHPLSTDPIAEQVANIQLPDRHPTGQRG------MARLTADFWVRAYLARLRLADIPAFVTSRGDATAGAVLVKLNTLDGQATAFQREFNLMTGARAWVELA----HGPEAEVDAAITRQRSFDPDLWVIEVEDRSGRHLL------DQDGLSE-MKELLRSTDPTILAFASALLQGEDIDCFQLDVNMSVLEGGIGIFPRRLMVRADDHDRAVRVMVDNDI-PLGRD--------MAEEVWKRDEVDSPCVKICVVHPVERICTGCLRSIDEIARWSRMSAEERRAVMAELPARAPRLAKRRGGRAARMSRGEG---------MSHIVVVGAGQAGSSLVAKLRNSGFDGRVTLIGAEPVPPYQRPPLSKAYLLGDMEAERLFLRPESFYADNDIDLRMGASVTAIDRHGKTLHIG-DE-----VLSYDQLALTTGSEPRRLPCAIGGDLEGVFVVRTLADIDALAPRVTEGARALIVGGGYIGLEAAAVAAKRGVQVTLVEMADRILQRVAAPETSDYFRALHMKHGVTIREGVGLDRLV----------GDGDRVTGAVLADGSEIEVDFVIAGVGIAPSTTLAELAGVTLENGIRVDAYGRTSDPHIWAAGDCASFP--YRDGRIRLESVPNAIDQAEVVARNMMGEQIAYQAKPWFWSDQYDVKLQIAGLNTGYDHIVTRPGDGT-T------VSFWYYAGETLLAVDAMNDPRAYMVGKRLIEAGKSPDPEAIANPETELKTLLKP----MSALEQIGLAALRQIDPERAHGLALRALRAGLAPM----PGVVTSHRLRTQMAGLDLPNPIGLAAGFDKNADCLAPLVRSGFGFVEVGAATPRPQPGNPKPRLFRLSEDRAAINRFGFNNDGMEAIAARL-----AARPTEGVIGLNLGANKDSTDKSADFARVLRHCGAHLDFATVNVSSPNTEKLRDLQGKEALKALLT--GVMQARRELPAPLPIFLKIAPDLSESEIEDIASLALDSG--VSAIVATNTTLSRD-GLRSAHRDQAGGLSGAPLFDRSTRVLARLYRLTEGEVPLIGVGGIGSAADAYTKIRAGASAVQLYTALVYQGLSLGARIAQELDALLARDGFASVEAAVGTENGKWT---MPRERMSVVVTRRLPEQVETRLSELFDVRLRDDDTPMTREELTEAMKSADVLVPTVTDQIDAALIAQGGDRLKLIANYGAGVDHIDVATARQRGVLVSNTPGVLTDDTADMTMALILAVTRRIPEGLAVMQRGDWQGWAPTSFLGGRVGGRRLGILGMGRIGQAVARRAAAFGMQVHYHNRKRLRPEIEESLEATYWESLDQMVARMDVLSINCPSTPSTFHLMNARRLKLMKDTAVIVNTSRGEVIDENALTRMLRAGEIAGAGLDVFEHGAAFNPRLRELENVVLLPHMGSATREGRVEMGEKVIINIKTFADGHRPPDQVVPSMLMSRIGKKPVDLPSGVSASVSGQTIEVKGPKGTRSFTATDDVTITVDDNVVKIDPR---------------GKSKR-----------ARQQWGMSRTMVANLVTGVTDGFKKELEITGVGYRATVQGN-SLKLNLGLSHDVDFVAPEGVTVTAPKQTEIVVEGIDEQLVGQVAANIRAWRKPEPYKGKGIRYKNEYIFRKEGKKK--MLVYGLKSCDTCRKALKSLP----EASFVDVRDKGVPAEVVGRAIADFG-HELVNKRSTTWRNLSEEDRAADV----ATLIASHPTVMKRPLIVNGDQMHLGWSDEVRAALGVG--MPRYALKIEYNGAPFAGWQRQADQPSVQGAIEAALARLEPRAHTIAAAGRTDAGVHAIGQVAHCDMARDWDPFRLSEALNFHLKPAPVAITACAAVNEDWHARFSAVERRYLFRLLMRRAPAVHDAGQVWQVSRELDIAAMQAGADRLIGRHDFTTFRSSICQAASPVKTLDSFTVSLSQGVSGPEARFELRARSFLHNQVRSFVGTLERVGAGAWSPDDVTAALEARNRAACGPVCPPQGLYLQSVGYPEDPFTA------MDKYPDQKRAVQYLHPPIDPFDQRLLDVGDGHTLYVEQCGNPNGIPVVVLHGGPGGGCSPAMRRYFDPEVYRVVLFDQRGCGRSRPHASVQDNTTWHLVADIELIRTTLGIDSWLVFGGSWGATLALIYAQTHPERARHLILRGVFLMTQAELDWFYGGGAGRFWPESWARFVAPIPEDERHDLIGAYHRRLFSGNLAEETKLARLWAGWENALASVHSNGTIGDAP----GDYARSFARLENHYFQNAGFLEFDGQIL--ANMDRIAHIPGVIVQGRYDMICPPVSAWSLAKAWPGSDYRLVRNAGHALSEPGISAELVRIMDDIAHSKVGAA--------MRIYKILRAHEWAAFQAQGETAGAPVDEADGFIHFSTAAQAAETAAKHFAGADDLMLLTLESEALGPDLKWEPSRGGALFPHLYGPLRLADVI---VAQDLPLIDGAHSFPEGME-------MSNNAQGAARRGLLIILSSPSGAGKSTLARRLRDWDSDIVFSVSATTRAPRPGEVDGADYHFTSESAFKAQVANGGMLEHAHVFGNFYGSPKAPVQAAIDEGRDVLFDIDWQGAQQIRNSALGAHTLSIFLLPPSIGELRRRLESRAQDDADTIARRMQRSWDEISHWDGYDFVLVNDDLDATEAHLKTIVTATRLRRLQQPGLTDHVRALQSEFEDL---MTLKLGVPSKGRLMDKTFAWFARHGIELSRAGSDREYAGAVQGIDGVSLVLLSAGEIPRELAAGRIHLGVTGTDLVHERLPRWEQQVEEIEPLGFGRADLIIAVPNCWADVDNLDDLDAAAAAFRARHGFRLRIATKYHRLVRDFLRDSGVADYALVDSQGATEGTVANETAEAIADITSTGETLRANHLKILEDGLILQSQATLWRSRVAEYDTTQAAALKALLSRIHGH----------MAPDRPDTSSDT--PKAP-HVPVLLRPLIERVSPVSG-VWLDGTFGAGGYTRALLQAGAERVIAVDRDPLAFQMAQGWAGDYG--------DRLVMQPGVFSRMDDYAQD-----LDGVVLDLGVSSMQLDLAERGFSFMKDGPLDMRMSQDGPSAADIVATASEAQLADILFHYGEERASRRIARAILRAREEAPIKTTLQLAKLVESCLPRAKPGQSHPATRTFQALRIAVNGEYEELYRGLMAAERALKPGGLLAVVSFHSVEDRMVKRFLAARAGQSGQANRYAPVQDESP-AQFVAEPKKSIAPDAEELEQNPRARSARLRIARRTDAPAGTVEPRE-ISMPQVGEGRT----------MTASPFTCLSHSAEETAEFARRIGARLESGDTILLTGPVGAGKSHFCRSLIQSRL---SEP-EDVPSPTFTLVQMYDTTKDQ-IWHADLYRLTGTDEIEELGLFDAFEQAICLVEWPDRLGGTAPPDALALTL--SASPEDENHRTLEFTWSAPKWAAKLDGVAHG--------MTVSLGSTSLTPPVFLAPMAGITDRPFRDIVSGFGAGLVVSEMIASQEMVQAKAGMREKAELGFDAGQTAVQIAGREEYWMAEAARMAEANGARIIDINMGCPAKRVTNGASGSALLKTPDHALRLIEAVVDAVDV-PVTLKTRLGWDDTLLNAPDVAARAEAAGVQMITIHGRTRCQFYKGRADWAAIRSVRDAVRVPVIANGDIVTTGDAKQALKQSGAEGVMVGRGAQGQPWRLAQIAAALWGAPAPEVPQGAALADLVGRHYEAILGFYGTQIGLRTARKHLGWYMEAANT--PA-PLRRDILTE----ATPARVLAYLPDALSGPIP-----RAA---MTKP----P---LTLYLAAPRGFCAGVDRAIKIVEMAIQKWGAPVYVRHEIVHNKFVVDGLRDKGAVFVEELDECPADRPVIFSAHGVPKSVPAEAEHRNMVYVDATCPLVSKVHIEAQRHADAGLQIIMIGHRGHPETIGTMGQLPEGDVLLVETEADVTDVVVRDPARIAYVTQTTLSVDDTAGIVAALQARFPAIVGPHKEDICYATTNRQEAVKAIAPRAQAMLVVGAPNSSNSKRLVEVAARAGCSYSQLVQRATDIDWRALDGVRTMGLTAGASAPETLVSEVVDALADRFDLTVEPVETAVENVEFKVPRVLRQPA----------------------MAAIWHPSPNFGVRRDGLRPTLIVLHYTAMTSAQAALDRLCDPKAEVSAHYLIGGDGTLWQMVDEDQRAWHAGAGAWAGQGDVNSRSIGIELDNRGDH---------PFSEPQMAALERLLPGIMQRHGIAPQGVIGHSCMAPGRKADPGPRFDWTRLARQGLATRP---GMGETAK-----PTPDSFRQMARARGFT--------APC-DDATLLAAVRLRFRPGA-SG---PLTAEDLAVLHMA----------------MLLAKRKLRIETERLTLRPPVHSDFRAWSALRLESRDFLTKWEPTWASDHLSRKSFTNRVYWAQRSVTSGSALPLFLFRRSDEVLVGAITLDNIRRGPAQAGTLGYWTGERFARSGFMREAAAAVVHHAFTRLDLSRIEAACLPENAASRGLLESSGFKYEGVAQSYLQIDGRWRTHVLYAALRSDRRGRTETG------MSGK-RSIFEEVGETT-LTRPEPQ-----TGIIDRGRTGARRGIRVWLMVLFALVVAMIVVGGLTRLTDSGLSITEWRPVTGALPPMNEADWQSEFEKYKQIDQFQLQHAWMELSDFKQIYWWEWSHRQLGRVIGVIWAVGFLGFLIARQIPVGWTGRLLWIGALGGAQGAIGWWMVASGVTQGEGVTTVASYRLATHLGLAFAILGFIAWYVFLLGRTERDLMQARRGRETRLFGLGTGLMHFAILQILIGALVAGIDAGRTYNDWPLMAGQFFPPNAFPEVPLWRNLFESPGLVQFIHRCAGYLLFIFGIVALLKGRRSANPATRFAFTAAFAALAVQVALGIMTVLYEAQLHVAITHQLVAVILWVLILRARFMAAYPVATSIRER----MSDRFRVTLAQLNPAVGDLAGNAAKAREAWEQGRDAGADLVAMTEMFITGYNAQDLVKKPAFYLDAISHIRQLAVDCADGPALAIGGPWIEGTQLYNAYFILKGGQIANTVLKHHLPNETVFDEVRIFDSGPLGGPYSV--GNTRVGSPICEDAWHEDVAETLAETGAEFLLVPNGSPYYRGKHDTRLNHMVARVVETGLPLIYLNMVGGQDDQVFDGASFGLNP-GGTLAFQMPQFDVAITHVDLHRTPDGWRIKQGEEHAHASEWEQDYRVMVQSLRDYMGKTGFRKALLGLSGGVDSAIVATIAADAIGPENVRCVMLPSEYTSAESLEDAEAAARALGCPYDHVPISQARAAVTETLAPIFAGTKPDLTEENIQSRLRGLLLMAMSNKFGEMLLTTGNKSEVAVGYATIY-GDMAGGYNPIKDLYKTRVFETCRWRNENHR-DWMMGPKGTVIPERIITKPPSAELRPDQKDSDSLPDYPDLDAMLEILVDRDGSIADCVAAGFDREVAQKVERLIYLSEYKRFQSAP--GARLT--KGAFWLDRRYPIVNRWRDPS--------MALD--------IVTIPCRTDNYAFLGHNPETGDTFLVDAPEAAPIVEELAKRGWSLKTILITHHHPDHVEGLPGILAAHTARVVGARADAHRLPPLDIEVAEGDMVDAAGEMAHVIDVSGHTVGHVAFHFPDSGVVFTADSLMALGCGRVFEGTMEQMWGSLSKLAALPPDTIVCSGHEYTQANAKFALTIDPDNPELKSRAEAIDLARSRNQPTVPSPLSLELATNPFLRAADPGIRRNLGMQTEPDAAVFAEIRGRKDRF---------------MDLTGKLLIAMPGMGDPRFEHSVVFMCSHTEDGAMGLIVNKPARDVRLSDLLDQLDIGG--AARPE-----MPVHFGGPVDGVRGFVLHSAEYASRLHTLAVPGGFCMTATLDILEDIAAGTGPQRALMMLGYAGWGPDQLEDEISRNGWLTADAAPELVYDMPDDAKWAAALNSIGVDPLGLSASAGRAMNDATRLL---NEMNDRSREVFRRVVESYLATGDPVGSRTLTRSLNEKVSAATIRNVMQDLEYLGLLDSPHVSAGRIPTQMGLRMFVDGLLEVGDLDEADRLQIDSSLTNNSASGVESALDRVGAVLSGVTQGASLVLTPKHEAAIRHIEFVSLNQTKSLVVLVFADGHVENRLFTPPPGQTPSSMREAANFLNALIDGRTLSDVRDLMKTEIARRKQEIDSLAQAMVDSGLAAWESGAEDGETAR-LIVRGRANLLSGGDPEVELERIRTLFNDLERKRDIAEFLQLAEDGDGVRIFIGSENKLFSLSGSSLVVSPYMNADRKIIGAVGVIGPTRLNYGRIVPIVDYTAQVLGRLVSDRS-----MTDLLDDPAP----GANTPEFTVSEVSGAVKRTLEGEFGRIRVRGEVGRVFRARSGHLYYDIKDDRNVLACTTWKGQISQLSVEPEEGLEVVVTGRLTAFGAQSKYNLNVEEVTVAGQGALMALLEKRKKQLAAEGLFDATRKRPLPFLPEIIGVVTSPSGAVIRDILHRLRDRFPRKVLIWPVAVQGANCAPEVARAIAGFNALTPGGALPRPDLIIVARGGGSVEDLWGFNEEIVARAAAASEIPLISAVGHETDTTLIDFVSDRRAPTPTAAAELAVPVRLDLLAWVEAQGARLTRAAGNATAQRRQRLADLARALPRPEGLLDTPRQRLDRAADRLP---------------------RALIGGLQTRRLHLSETTASLRPATLQQRLAARQDRLDGWARQLHPA-----------------------LLRLVARRREALAAT--RLRAQPLTMALATGRQDLDRLSKRLDAATTARLERLDTRLASSARMLETLSYQATLDRGYAVVRAG-DALVTSRDTAAGAPQLDIQFADGHVTA----------------VPTDPAP--------------RKPKKTVPKPPDQGSLF---MDKKQKPRG----LGRGLSALMADV-----------------GPTVSTAPGGPSPAPARPDMTVPIEQIIANPDQPRRRFSQDALDDLSNSIREKGIIQPLIVRRL--DSGQYEIVAGERRWRAAQQAQLHDIPVLVRDFDDTEVLEVAIIENIQRADLNAVEEAAGYRQLMDRFGHTQEKMAEALGKSRSHIANLLRLLALPEEVQDMLREGTISAGHARALITAENPVELARIVVRDGLSVRATEALVKKSQQPEDGPKPA---KVRKSAEKDADTRALEGDLGANIGMKVSINH-EPGSERGTMVLQYESLDQLDQLCNLLSGN-------------------------------------------------------------------------------------------------------------------------------MKLTGFFVAAALALCGGGLHAETCRDDRVQLRGDWGSAGFSVEVVDTVEDRARGLMFREEMPRGAGMLFVYDTPGPVSFWMKNTLIPLDMVFVDDAGTVQRVHSNAIPGDLTPIDGGDD-ILVVLEINGGLAQRYGIGPGSQLRHPVFSDNGPVWPC------MPKRTDISSIMIIGAGPIVIGQACEFDYSGAQACKALREEGYRVILVNSNPATIMTDPGLADATYIEPITPEVVAKIIEKERPDALLPTMGGQTGLNTSLALEEMGVLAKYGVEMIGAKREAIEMAEDRKLFREAMDRLGIENPKATIVTAPTLDNG-----KKDLAAGVRLAMDALDEIGLPAIIRPAFTLGGTGGGVAYNRDDYEYYCRSGMDASPMGQILVDESLLGWKEYEMEVVRDTADNAIIVCSIENVDPMGVHTGDSITVAPALTLTDKEYQIMRNGSIAVLREIGVETGGSNVQWAINPEDGRMVVIEMNPRVSRSSALASKATGFPIAKIAAKLAVGYTLDELDNDITKVTPASFEPTIDYVVTKIPKFAFEKFPGSKSELTTAMKSVGEAMAIGRTIHESLQKALASMESGLTGFDEVEIPG------------APDK-----------------AAVVKAIAQQTPDRMRTIAQAMRHGLSDDEIHGVTMFDPWFLARIREIVETEEQLKKTGLPTSEEGLRALKMMGFTDARLGKLTGRDESNVRRARRNLGVKAVFKRIDTCAAEFEAQTPYMYSTYEHPMMGE--VECEARPSDRKKVVILGGGPNRIGQGIEFDYCCCHACFALTDAGYETIMINCNPETVSTDYDTSDRLYFEPLTFEHVLEILRVEQENGTLHGVIVQFGGQTPLKLANALEAEGIPILGTTPDAIDLAEDRERFQDLVNRLGLKQPRNGIAHSDAEAMAIASDIGFPLVIRPSYVLGGRAMEIVRDQAQLERYISEAVVVSGDSPVLLDSYLSGAVELDVDALCDGTAVHVAGIMQHIEEAGVHSGDSACSLPPYSLGKDVIAEIKQQTEALAHGLNVVGLMNVQFAIKDG-----EIYLIEVNPRASRTVPFVAKATDSAIASIAARVMAGEPLSNFPLRPPYDADAAYDDVLPLGDPMTLADPDMPWFSVKEAVLPFARFPGVDTILGPEMRSTGEVMGWDRSFPRAFLKAQIGAGNPLPRDGR--AFISIRDDDKTPQMLEAAQILVEQGFDIVATRGTQAWLAAQGVACEVVNKVYEGRPDIVDMMKDGAVHLVMNTTEGAQAVEDSKGIRSVALYDKIPYFTTAAGAHAAALAIKAQAEGDVGVKALQA---------MTPEQIAQLPYRPCVGVMLANAEGAIFVGQRRDRDTD-AWQMPQGGIDAGETPVQAALRELGEETGLTPDKVKIVAETEAWLTYDLPHDLVPNIWKGRFRGQEQKWVLMRFTGEDADVNINTEHPEFSEWQWLPHADLVANIVPFKREVYERVLAEFTPALAQL-----------MRRVSIFGATGSVGQNCIDLIDR----NPASYDVVALTGGRNVAQLAQDARRLNADIAVTAFDECLPDLRVALEGTQIEAAAGTTALSEAGARPADWVLSAIVGSAGLAPGLAALGQGATLALANKESLVCAGALVMETARANSARILPVDSEHSAIFQALGGEDIDRVERIVITASGGAFRDWPMDKLRHATPELAATHPNWDMGQRITIDSASMFNKALEVIETREFFGIAPDRIEVLVHPESIVHALVGFHDGAMMAHLGAPDMRHAIGYALHHPARQPLPVARLDLARIGQLNFTTPDEARFPALRLARAVMARGGLTGAVFNAAKERSLDAFIAGRIGFMDMADIVERVLD--RFEAESDLIDAAMTLDNVMQTDH----LARMWSDEAMAKQAG-----MSAET-------PDYKSTLNLPKTEFPMRAGLPKREPDWLARWEKIGVYDRLREK---------QG---RPDFVLHDGPPYANGHLHIGHALNKTIKDMIVRSHQMMGHDSRYVPGWDCHGLPIEWKIEEQYRAKGRDKDDVPIVDFRQECRKFAEGWVDIQREEFKRLGITGNWADPYLTMNFHAEAVIAEEFMKFLMNGTLYQGSKPVMWSPVEETALAEAEVEYHDKESFTVWVKFKVV---------------APADH------------------KLSGAQVVIWTTTPWTMPSNKAVVYGEGISYGLYKVTDTPEEC----WANVGDTYLLADNLAADVFKRARL-GEDQYTRVGDV-TAQDMAGIGLAHPLAGAEGANGEWDDIRDFRAADFVTDTEGTGFVHCAPSHGMEEYELYRGLGMLEQVITYNVMDDGTFRPDLPF-FGG-----------KAILRNKPKNGSWEGDANAAVISKLAEVGGLLARGKIKHSYPHSWRSKAPVIYRNTPQWFAAIDRKIDDGQGTHGDTIRERALTSIDELVQWTPKTGRNRLHSMIESRPDWVLSRQRAWGVPLTCFTKKGALPTDADFLLRNPD-VNARIAAAFEEEGADAWYVEGAKERFLGDSVNPDDYDQVFDILDVWFDSGSTHAFVLRDRP-DGTPDGIADVYMEGTDQHRGWFHSSMLQACGTKGRAPYRNVVTHGFTLDEKGAKMSKSLGNTIVPEEVIKQYGADILRLWVAQTDYTADQRIGPEILKGVADSYRRLRNTMRYMLGSLADFSEADRV---------------DPAEMPELERWVLHRLAELDKVVKDGYRAFDFQGVFSTVFNFATVDLSAFYFDIRKDALYCD----------------GDTLRRRAARTVLDLLFHRLTTWLAPVLVFTMEEVWLE-RFPGDDSSVHLVDMPDTPTDWLNPDLATKWAAIRRARRVVTAALEVQRTDKVIGASLEAAPVVHVADAEALEA-------LKSVSFDDLCITSGIVLT-ADPAPAEAFRLPEVEGVGVVFERAEGEKCQRCWKIL-PDVGQHAHPNVCGRCDSALG------------MTIALQSLMMSHFRSHRVVRMEVDARPVALFGPNGAGKTNILEAVSLFSPGRGLRRASAAEMTRRPEALGWKLSAVVSGQGAPREIETFSEGGAARQVTIDGKAASQVALGALVRVLWLVPAMDRLWIEGADGRRRFLDRMVLSFDPGHAEASLAYEKAMRERNRLLKDMVRDAHWYGALERQMAEAGHRIDVARRAALAELAAAQEGAQTA-FPAAALELVQT--EG--EMPETEGD--------LREALAESRFRDLSAGRTLVGPHRSDLYGVYADKGVPARDCSTGEQKALLVSLILANARAL-QTRIGQAPLLLLDEVAAHLDAGRRAALYDEICALGAQAWMTGTGPELFAELGERAQMLEVTETGGESRVRLA-------------MAAKLRKGDKVIVLAGKDKGKEGTIASVDPKANKAVVDGINIAIRATRQSQTSQGGRLPKAMPIDLSNLALID-ANGKPTRVGFKIEG-DKKVRFAKTTGDVIDA-------MTAPTSDIILERMMALHPKVMDLSLDRMWRLLDALGNPEQSLPPVIHVAGTNGKGSTLAMIRAGLEGAGLTAHAYTSPHLARFHERIRVAGQLIPEPDLTAVLDDCYAANKGAPITYFEITTCAALLAFSRTPADYTLLEVGLGGRLDATNVVDSPRLTVITPVSIDHQQYLGTTLTEISGEKAGILKRNVPCVVGPQDPDAFDAIEARAIRLGAPLLVRGQHWHARRDAGRLIYEDETGLADLPLPNLLGAHQIDNAGAAIAALRALGLGQD--A----LEAAV----TRAEWPARMQRLKTGPLVDMAEGCELWLDGGHNPAAGEALAAVLASLPPRPTHLICGMLNTKDIGGYLRPLAPHVASLTALSI-PGEANTLPAETTAEHAQQAGIASQTADSAADAVQMIRQSDPDARILICGSLYLAGAILRENG-------MTGTAIKFCGLTQAADIDAAVAADARYVGFVFFPASPRNITREVARDLALMVPPGVMKVALTVNADDALLDDIVADVP-LDMLQLHGTEAAERVAEVRARYGLPVMKAVGVATADDLPALDTYGRVANQLLVDAKPPPGADLPGGNGLAFDWRLIAGRR-WPVPWMLAGGLTPENVAQAVAMTGARQVDVSSGVERAPGVKDARRMADFAAALA--------------------------------MTGLPTPKGTLTPGRDLSELTWLRVGGPADFLFQPKDKADLAEFLRNLDASVPVFPMGVGSNLIVRDGGLRGVVIRL-GRGFNQIE-CDGTRVTAGAAALDAHVARRAADAGL-DLTFLRTIPGSIGGAIRMNAGCYGSYVADRLVAATVITREGAEVRL-------TRDDLNLRYRQSDLPEGAVLISAEFEAP-QGDPEALHARMEDQLARRDATQPTRERSAGSTFRNPAGFSSTGQADDTHDLKAWKLIDDAGLRGARRGGAQMSEKHSNFMINTGGATAADLESLGEDVRKKVYASSGITLEWEIMRVGDPLA------------------MPAPI-----------VSFEFFPPQNLEASFRLWDTVQTLAPLDPRFVSVTYGAGGTTRDLTRDAVATLHKSSGLNVAAHLTCVNATREETLGIARSFAEAGVNELVALRGDPPKGTG-AFQPHPDGFANSVELIAALKQAGE-----FTIRVGAYPDTHPDAADMAADIDWLKAKLDAGADEALTQFFFEAESFLRFRDACAKAGIDKPITPGILPIENWKGASKFAKACGTYIPDWVDDAFTTAAR--DGREDLLATAICTELCSDLIDEGVDKLHFYTLNRPELTRDVCHALGVT-PKLNLQNVA-MNYETLIPLIRKLAIEAGEKIMEIYDGDDFEVKAKSDDSPVTAADEAADALISAGLRAELPDVMLVTEEQADSH--AAKGDTFLIVDPLDGTKEFINRRGDFTVNIAYVENGVPTRGVVYAPAKKRMFYTHPSGQSVEEIGSFDPETPGELREIHV-SKPDNGALMVVASKSHRDQATDDYIGKYDVKDMKSAGSSLKFCLVATGEADLYPRVGRTMEWDTAAGHAVLAGAGGKVVRFDNHKPLTYGK--EDFANPFFIAYAPGVELKDAMAQTRTETDSFGPLEVPADKYWGAQTQRSIMNFPIGWEKQPVPIIRALGVVKQACAMANKANGALEPELAEAIIAAAGEVIEGKFDDNFPLVVWQTGSGTQSNMNANEVIGNRAIEMLGGEMGSKKPVHPNDHCNMGQSSNDTFPTAMHVGIAMQARDVLLPGLEKLAAALEAKRDEFKDIIKIGRTHTQDATPLTLGQEFSGYAHQVRKGIARVNACLPDIYELAQGGTAVGTGLNTKKGWGEAVAANMAEITGLPFVTAPNKFEALAAHDAMVMFSGALKTVAASLFKIANDIRLLGSGPRSGLGELILPENEPGSSIMPGKVNPTQAEALTMVCAHVMGNDAAVGMAGSQGHFELNVYNPMMSYNVLQSMQLLGDSASAFTDNMVVGIQANEARIEKLMKESLMLVTALAPTIGYDNATKVAKTAHKNGTTLKEEAIALGFVDEETFDRVVRPEDMIGPK--------MTQDLKWAALAVRENAYAPYSRFKVGAAIRGASGKIYAGCNVENVAYPEGTCAEAGAIAAMIADGETVLTEVYVVADAPDPVPPCGGCRQKLAEFGA-AAVPVTLSTTAGDERGVTIGDLLPGAFGSAHMPGF-------MIQADLNVILPEIVLAAFAMLALVGAVYTGKDKTAPAIVWATGILMLGLAVWIGTRGTA-TTVAFNGMFIDDSFARFAKVAILLSAASVLVMSQEYMQRRGILRFEYPLLVALAAVGMMMMVSAGDLMSLYMGLELQSLALYVVASLRRDSLKSTEAGLKYFVLGALSSGLLLYGASLVYGYSGTTLFSGIIQTAVHGEVSLGLLFGLIFLISGLAFKVSAVPFHMWTPDVYEGSPTPVTAFFATAPKVAAMGLFARLMHDAFGAAVSDWSQVIALIAVASMFLGAVAAIGQTDIKRLMAYSSIAHMGFALMGLAAGTELGVQAMLIYMAIYATMNVGTFAFILTMERDGAPVTDIRALNQYARRQPGRALAMLILMFSLAGVPPMLGFFGKFYVLRAAYEAGLGWLAVAGVVASVIGAFYYLRIVYYMYFGEEGHE-IEANGSPVLWGFLMASAAVMLLGV-----INMFGVEGAAAAAAATLVN-----MSPSLDTVFSALADPTRREILRMLLEDDMAVTDVAEPFEMSLAAISKHLVILTRAGLISQEKRGRVKWCKLEPGGMRAASVWMQGFGQFEPVNLDAFERFLEREF--AEGGDGAE------------MNDTEDGSS-AAQSALSQS---SATDSAESQPEE----RS--GFFGRIIEALTPVEEV-EDDGANRRDAPR-------------GLANLRRMRVEDVAVPTAEITAVPSTISKSDLVDVFRDSGMTRVPVYDGTLDTPVGMIHLKDFALNHGFNG-GSDEFDISTLLRPLLYVPPSMPIGVLLTKMQAERRHMALVIDEYGGVDGLVTIEDLIEQVVGEIEDEHDIDEDKSWVKEKPG-QYIALAKTPLEDFEAEIGRSLTDHDEVDEEEIDTLGGLVFMLSGRVPTRGEVVAHPDGPEFEVVDADPRRIKRLRVRLVGPAE-------------------------------------------------MHANGPTPD---------------GQVADAPAGNWVDRWAPPAARPYLRMSRADRPIGTWLLLLPCWWGLALAMLWDQSPRWEDAWIFFGCGLGAWLMRGAGCTWNDIADRDIDGRVARTRSRPIPSGAVTPKQAAAWMILQALVALCILLTFNRAAIALGVLSLLPVAIYPFAKRFTWWPQVFLGLAFNWGALLAWTAHAGTLNWPAVILYVSGICWTLFYDTIYAHQDAEDDALIGVKSTARLFGANSARYLRYFLVACVALMGLAIIQAVMPNASVLALVIALAGPWAMGWHMAWQLRGFDPEDGARMLFLFRLNRDTGFIPLIFFGAALFA-----------------------------------------------------------------MAVQQNKVSKSRRNNRRAHDALVAANPNECSNCGELKRPHHICAACGHYDDKEVVAQVDEIDLDEDAA------MTEQPGIVVTGASGRMGQMLIRTIAESGKARLVGAIERPGHDWIGRDAGEAAGIGALGVAVTDDAAAALSGAQVVVDFTAPAATL-RFAELAAQAGVAHVIGTTGMSEDEIAALAPFARH-TAIVRAGNMSLGVNLLAQLTRKVAAALDEDFDIEVIEAHHHHKVDAPSGTALMFGEAAAEGRGVTLSDVSDRGRDGITGARKRGDIGFTAIRGGDIVGEHDVLFAGPGERVILRHVATDRGIFARGALRAALWASGREPGQYDMIDVLGL-----MADVNR-GNRPLSPFMLGQYYRPQMTSISSIMVRITGIASLGTAVLLVLWLLAAATSDGAFNVIDGILGSFIGDILMLLATWAIWYHVLGRLRHVIWDF-GYAVELDISEKMGAIMFIGATVMAILTVIIV--------------MPVSWLILILAAL-TLGGYIAGRARARASVQGDVRRLHSLPSYYGYNVALFALVP-ALAVLAL---WLLAQPMLIDARVSGMIPDT-------------------LIPEGSNQSLVMSDVRRVADGLELAVSSGAMSADEVAALDADATDIRARLGEMGVALGSDVRPEVLEAAQAYRAMSSTGTLWMTGVVLVLALA-GLAWSYSRTHSEFRARNVVERGVLTLLILAASIAILTTVGIVLSMLFETINFFS-------------------------------------------------------LHDWK--DFFLGATWAPN------FRGD--------SELSILPLLWGTLYISLIALIVAVPIGLFAAIYLSEYASKPVRAFAKPLLEILAGIPTIVYGLFALLTVGPLLVDIFGRGEEGLLGLN-------WMSGATSVLTAGVVMGIMLIPFVSSLSDDIINSVPQSMRDGSLGLGATKSETVRQVVMPAALPGIVGAILLAASRAIGETMIVVMGAGAIAK-------FSLNPLESMTTITTRIVSQLTGD-TDFASPETLVAFALGLTLFVLTLGLNILALYIVRKYREQYEMAQSFLGQKRLRKYYGKIREVLEMPNLIEVQKSSYDLFLNSGDQPDPSDGEGIMGVFQSVFPIKDFNETSVLEFVKYELEKPKYDVEECQQRDMTYSAPLKVTLRLIVFDIDEDTGAKSVKDIKEQDVFMGDMPLMTPNGTFIVNGTERVIVSQMHRSPGVFFDHDKGKTHSSGKLLFACRIIPYRGSWLDFEFDAKDIVYARIDRRRKLPVTTLLYALGLDQEGIMNAYYNTIQYKYQ-KGKGWVTKFFPERVRGTRPPFDLVDAATGEVIAEAGKKITPRAVKKLIDEGNVTELLVPFENITGKFVAQDIINEDNGAIYVEAGDELTLEYDKDGDLIGGTAKELIDAGITDIPVLDIDNINVGPYMRNTMAMDKNMNRDTALMDIYRVMRPGEPPTVEAAGTLFDTLFFDSERYDLSAVGRVKMNMRLAL------DKPDTQRTLDRDDIVACVKALVELRDGKGDIDDIDHLGNRRVRSVGELMENQYRVGLLRMERAIKERMSSVEIDTVMPQDLINAKPAAAAVREFFGSSQLSQFMDQTNPLSEVTHKRRLSALGPGGLTRERAGFEVRDVHPTHYGRMCPIETPEGPNIGLINSLATFARVNKYGFIETPYRVVKEGQVTDEVHYMSATEEMRHTVAQANATLDDDGKFVNEMVNTRQSGDYTLAPVESVDLIDVSPKQLVSVAASLIPFLENDDANRALMGSNMQRQAVPLLRAEAPLVGTGIEEVVARDSGAAIMARRAGIIDQVDAQRIVIRATEDLELGDAGVDIYRMRKFQRSNQNTCINQRPLVKVGQQVGKGEVIADGPSTDLGELALGKNVVVAFMPWNGYNYEDSILISERIARDDVFTSIHIEEFEVAARDTKLGPEEITRDIPNVGEEALRNLDEAGIVYIGADVEPGDILVGKITPKGESPMTPEEKLLRAIFGEKASDVRDTSLRVKPGDFGTVVEVRVFNRHGVEKDERALQIEREEVERLARDRDDELAILDRNIYARLKGMIMGKTAVKGPKGVKAGSEITDDLL-DTLSRGQWWQLALEDEKDAQIVEALNEQYEVQKRALDARFEDKVEKVRRGDDLPPGVMKMVKVFVAVKRKLQPGDKMAGRHGNKGVISKVVPMEDMPFLADGTPVDFCLNPLGVPSRMNVGQILETHMGWAARGLGLNIDEALGEYRRSGDLTPVREAMRLAYGDDV-YDEGIESMDETALIDAAGNVARGVPIATPVFDGAKEADVNDALARAGFDTSGQSDLFDGRTGQQFSRKVTVGVKYLLKLHHLVDDKIHARSTGPYSLVTQQPLGGKAQFGGQRFGEMEVWALEAYGAAYTLQEMLTVKSDDVAGRTKVYESIVKGEDNFEAGIPESFNVLVKEVRGLGLNMELL-------DAEEE-------MATNTGGDSKNTLYCSFCGKSQHEVRKLIAGPTVFICDECVELCMDIIREETKASGLKATDGVPTPKDICEVLDDYVIGQATAKRVLSVAVHNHYKRLNHAQKAGNDIELAKSNILLIGPTGCGKTLLAQTLARILDVPFTMADATTLTEAGYVGEDVENIILKLLQASEYNVERAQRGIVYIDEVDKITRKSENPSITRDVSGEGVQQALLKLMEGTVASVPPQGGRKHPQQEFLQVDTTNILFICGGAFAGLDRIIAQRGKGSAMGFGADVKDPDARGVGEIFKELEPEDLLKFGLIPEFVGRLPVLATLEDLDEDALVTILTQPKNALVKQYQRLFDLEDTELTFTDDALRAIAKRAIQRKTGARGLRSILEDILLDTMFDLPSLDSVTEVVVNEEAVMSDAAPLMIYADA-EKDSASAG--MAGSVNKVILIGNLGRDPEVRTFQNGGKVCNLRIATSENWKDRNTGERRERTEWHSVAIFNEGLVRVAEQFLRKGSKVYIEGQLQTRKWQDQSGQDRYSTEVVLQGFGSTLTMLDGRGES------GGAGGGGRDSYGGGNDGGYDDR----FGGGNDGGY--GGGSSSGGGRSG--------------GGGSRDMDDEIPF-MKLQSKALPSSDAFKSNIDAHLAALSEIREAAQAAAAGGGERARDRHLSRGKMLPRERVANLLDPGSPFLEIGATAAHGMYDGAAPGAGVIAGIGRVHGQEVMVVCNDATVKGGTYYPVTVKKHLRAQEIAMQNHLPCVYLVDSGGANLPNQDEVFPDRDHFGRIFYNQANMSAMGIPQIAVVMGSCTAGGAYVPAMSDVTIIVKEQGTIFLAGPPLVKAAIGEVVSAEDLGGGDVHTRLSGVADYLAEDDTHALALARRAVAGL-NRTRPATVQWQADEEPAYDPDEILGVVPPDLKTPYDIREVIARVVDGSRFDEFKARFGETLVCGFAHVRGCPVGIIANNGVLFSESAQKGAHFVELCSQRNIPLVFLQNITGFMVGRKYEAEGIARHGAKLVTAVATTKVPKITLLVGGSYGAGNYGMCGRAYSPRFLWTWPNSRISVMGGEQAAGVLATVKRDGIERQGGSWSAEEEAAFKQPTLDMFAEQSHPLYASARLWDDGVIDPRKTRDILALSLSASLNAPIAPTRFGVFRM--MRRVVVTGLGLVTPLADGVEESWSRILDGQSGAGPITQFD--TEGLTTTYACEVPRGDGSDG------TFNADKYMEPKEQRKVDTFIMFGIAAAQQAVEDSGWLPTDKDGQERTGVLIGSGIGGLNSIANTAVMMEEKGPRRVSPFFVPGALINLISGQVSIKYGFKGPNHSVVTACSTGAHAIGDASRLIAFGDADVMVAGGAEAAICRIGIAGFNACKALSTKRADDPKKASRPYDADRDGFVMGEGAGCVVLEEYEHAKARGAKIYAEVVGYGLSGDAYHITAPSEDGDGAERSMRAALKRAGLEPKDIDYINAHGTST-MADTIELGAVERMMG-DAASKVTMSSTKSATGHLLGAAGAIEAIFSILAIRDQVAPPTINLDNPAVETKVDLAPNAKRERAIKYALSNSFGFGGTNASVIFGKID-MPITVGQDTAKTRKTISVNGKTISYYSI-PAATEAGLGDFSKLPAALKVVLENMLRFEDGK-TVSVDDIKAFSEWADKGGNNPREIAYRPARVLMQDFTGVPAVVDLAAMRDGIVALGGDAEKINPLNPVDLVIDHSVMIDEFGNPRAFQMNVDREYERNMERYVFLKWGQKAFNNFRVVPPGTGICHQVNLEYLAQTVWTDVDQNGDEVAYPDTLVGTDSHTTMVNGAAVLGWGVGGIEAEAAMLGQPISMLIPEVIGFELTGKMVEGTTGTDLVLKVVEMLREKGVVGKFVEFYGAGLDTLPLADRATIANMAPEYGATCGFFPIDDETIRYLTNTGRDKDRIALVEAYAKENGFWRDADYAPVYTDTLSLDMGTIVPAISGPKRPQDYIALDGAAKAFGDYVKGVRGGVDTSKSSEVRWEGEGGAPEPREIPGDEGHHKRGYVT-----TEDGHYQLHDGSIVIASITSCTNTSNPYVMIGAGLVARKAAALGLTRKPWVKTSLAPGSQVVSAYLEAAGLQEDLDKVGFNLVGYGCTTCIGNSGPLEAPISKAINDYDLIATSVLSGNRNFEGRISPDVRANYLASPPLVVAYALVGDMNVDITTAVLGQDKDGNDVYLKDIWPSQKEIADLVEATVTREAFQSKYADVFKGDEKWQGVEITDSLTYDWPAASTYVQNPPYFQGMGKEPGTISNIEGARVLAVLGDMITTDHISPAGSFKDTTPAGKYLVDRQVPVREFNSYGSRRGNHEVMMRGTFANIRIRNEMLDGVEGGYTKGPDGEQTSIYDASMAYQEAGTPLVIFGGELYGAGSSRDWAAKGTALLGVKAVIAESFERIHRSNLVGMGVIPFEFTGSDTRISLGLTGEETVSIKGLD-TIKPLQEVPCEITMADGSVKTITVKCRIDTAIEIEYIEHGGVLHYVLRNLASAA------MFLPFFENLRKAGVPVSLREFLSFLEGMKGGLATYDVEAFYYLARVTMVKDERNLDKFDQAFAASFKGLEDISFDQVLEAVDLPEDWLRKMSEKHLTDAEKAEIEALGGFEKLMETLRERLREQQKRHQGGNKWIGTGGTSPFGAYGYNPEGVRIGQKEGRHGRAVKVWDKREFKNLDDTVELGTRNIKVALKRLRRWAREGAADELDLDGTIRSTAENGYLDVKTRPERRNAVKVLLFLDVGGSMDPHVKVVEELFSAARSEFKHMEHFYFHNCLYEGVWRDNRRRWDAQTPTHEVLRTYGPDYKAIFVGDASMSPYEIAYPGGANEHWNAESGQTWLKRARDQWSSTLWINPIPEKYWSYTQSIQMVQDIF-ENQMVPMTLAGLEKGMRELTR-MARRKKIYEGKAKILYEGPEPGTIVQYFKDDATAFNAAKKDVIDGKGVLNNRLSEFFMTGLNQIGVPTHFIRRLNMREQLVRSCEIIPLEIIVRNFAAGSLSKRLGIEEGTQLPRPIVEYCYKDDKLGDPLVTEEHIAAFGWASQQDMDDVLSLALRVNDFMSGLMLGVGIKLVDFKIEIGRVFEG--DFQRLVIADEISPDSCRLWDIETGQKLDKDVFRRDLGSLTDAYSEVARRLGVLPQNATQ-MTKPKLIN------MKFLDLAKVYIRSGGGGAGCISFRREKFIEYGGPDGGDGGGGGSVYAEAVDGLNTLIDFRYQQHFFAKSGQPGMGRQRTGKDGDDIVLKVPVGTEILDEDQETVIADLTEVGMRVELARGGNGGWGNLHFKSSTNQAPRRANPGQEGVERTLWLRLKLIADVGLLGLPNAGKSTFLAATSNARPKIADYPFTTLHPNLGVVGVD-NVEFVVADIPGLIEGAHEGRGIGDRFLGHVERCAVLLHLVDGTSEDVAADYNTIIGELEAYGGELADKPRLTVLNKIDALDEEQRAEAAAALEEASGGPVMQ-MSGVARDGVTDVLRALRSRI---DADRLRRKPET--------EDTE--WRP-MTKTYEYETHEYDVVVIGAGGAGLRATLGMAEQGLRTACVTKVFPTRSHTVAAQGGIAASLSNMGPDNWQWHMYDTVKGSDWLGDTDAMEYLAREAPKAVYELEHYGVPFSRTEEGKIYQRPFGGHTTEFGEGPPVQRTCAAADRTGHAILHTLYGQSLKNNAEFYVEYFAIDLIMSEDGQCQGVVCWKLDDGTMHVFNAKMVVLATGGYGRAYFSATSAHTCTGDGGGMVARAGLGLQDMEFVQFHPTGIYGSGCLITEGARGEGGYLTNSEGERFMERYAPTYKDLAPRDYVSRSMTMEIREGRGVGEHGDHIHLNLSHLPAEALAERLPGISESAKIFAGVDVTKEPIPVLPTVHYNMGGIPTNYWGEVINPTADDKNAIVPGLMAVGEAGCASVHGANRLGSNSLIDLVVFGRASAIRAGQVVDPSTKNPVLNQASIDKAFDRFDHYRNASGGTPTAELRLEMQRTMQADAAVFRTSKTMAEGVEKMTAIAAKLSDLKVTDRTLVWNSDLMETLELTNLMPNALATIVGAEARKESRGAHAHEDFTERDDEKWRVHTIAHVDG---NKVDLSYRDVIKDPLSTEDEGGISLKKIAPKARTF---MTDLAHIRNFSIVAHIDHGKSTLADRLIQSTNTVSEREMKEQLLDAMDIERERGITIKANTVRIEYIADNGELYVLNLIDTPGHVDFAYEVSRSMHAVEGSLLVVDSTQGVEAQTLANVYQAIDADHEIVPVLNKIDLPASEPDRIKEQIEDVIGIDASNALLVSAKSGIGIKETLEAICARLPAPT-GDRDAPLKAMLVDSWYDPYLGVVVLVRVMDGVMKKNDRVKFMRNGTVHQIDKIGVFRPAMQDVAELGPGEIGFITASIKQVRDTKVGDTITTEKKGTDKPLPGFKPSIPVVFCGLFPVDSAEFEDLRDAIEKLALNDASFSYEMETSAALGFGFRCGFLGLLHLEVIRDRLEREYDIELITTAPSVVYHVHMKDGSMQELHNPADMPDMTYVDHIEEPRIKATIMVPDEYLGDVLKLCQDRRGIQQELTYAGTRPLVVYDLPLNEVVFDFYDRLKSVTKGYASFDYQLTGYRQDTLVKMSILVNDEPVDALSMMVHRDRAEMRGRAMCEKLKELIPRHMFKIPIQAAIGGKVIARETLSAMRKDVTAKCYGGDATRKRKLLDKQKAGKKKMRQFGKVDIPQEAFISALKMDN-MANTPQSKKRARQNQTRFAVNKARRSRIRTYLRKVEEAIASGDKDAAANALRAAQPELMRGVTKGVLHKNTVSRKLSRLSARVKALA-MVQFSLPKNSRITTGKTWPKPDGATNVRKFQIYRWSPEDGANPRVDTYFLDMDKCGPMVLDALIKIKNEIDPTLTFRRSCREGICGSCAMNIDGINTLACIYGLDEITGD-VKIYPLPHMPVVKDLIPDLTHFYAQHASIMPWLETKTNRPAKEWRQSIEDRKKLDGLYECVMCASCSTACPSYWWNGDRYLGPAALLHAYRWIIDSRDEATGERLDQLEDPFKLYRCHTIMNCTKTCPKGLNPANAIAQIKKMMVERAVMPHDGQDTAM-TPALLMP-DLLDVTGAALAPVETLFDAAREALRSRVS-ENGKLSVALIESEQTATHGLAWLATYAESLRQMQKWATGLQSEGRFGEIEQLIHQIAFGEYLAQIAGGIPMSQGEIVRAADLGLGAEEMTAFQTPEVSRLIATGNTQAARTRLAELMQEQAANICFGASGLDEELEMIREQFRRYVVDKVEPHAHDWHLKDELIPMEVIEELAEMGVFGLTIPENLGGFGLSKASMVVVSEELSRGYIGVGSLGTRSEIAAELILGGGTDEQKSHWLPRIASGEILPTAVFTEPNTGSDLAALRTRAVKGEDG-DYAITGNKTWITHAARAHVMTVLARTDPATTDHTGLSMFLAEKEPGTEDNAFPSEGMTGGEIEVLGYRGMKEYELGFDNFRVKGENLLGGEEGQGFRQLMRTFEAARIQTAARAIGVAQSALDVGLQYAQDRKAFGKPLLAFPRVSGKLAMMAVEIMVARQLTYFSAYEKDHDRRCDLEAGMAKLLGARVAWACADNALQIHGGNGFALEYKVSRILCDARILNIFEGAAEIQAQVIARRLLG---MNIHEYQAKALLRSYGAPVSDGRAILKAEDAKNAAGELDGPLWVVKAQIHAGGRGKGSFKEADAGEKGGVRLAKSVEEAAEEAKKMLGKTLVTHQTGPAGKQVNRIYIEDGSDIDRELYLALLVDRQTSRVSFVCSTEGGMDIEEVAASTPEKILSFSVDPATGYQPYHGRRVAFSLGLEGAAVKQCVQLMGILYKAFIEKDMEMLEINPLIVMPG-NQLKVLDAKLGFDGNAMYRQPEVAELRDTTEEDPKELEASKYDLNYIALDGEIGCMVNGAGLAMATMDIIKLYGAEPANFLDVGGGATKEKVTEAFKIITSDPNVKGILVNIFGGIMRCDVIAEGVIAAVKEVGLKVPLVVRLEGTNVEKGKEIIQNSGLDVIAADDLKDGAEKIVKAVKG-------MPDKPDIGP------DKSLLLVDDDEPFLKRLAKAMEKRGFEIETAGSVAAGRAIATARPPAYAVVDLRLEDGNGLDVVEVLREKRPDSRIVVLTGYGAIATAVAAVKIGATDYLSKPADATDITHALLATGDEMPPPPENPMSADRVRWEHIQRVYELCDRNVSETARRLNMHRRTLQRILAKRSPR-----MDLRNIAIIAHVDHGKTTLVDELLKQSGTYRENQATTERAMDSNDLERERGITILAKATSVEWKGTRINIVDTPGHADFGGEVERILSMVDGVVLLVDAAEGPMPQTKFVTSKALALGLRPIVVLNKVDKPDAEPDRALDECFDLFANLGADDDQLDFPSMYASGRSGWADMELDGPRKDLSALFDLIVKHVPAPAQIAQRDEPFRMLATTLGADPFIGRILTGRVESGTLRTGESVKALSRDGKQIENFRVTKILAFRGLSQQPIDVAEAGDIVSLAGMAKATVADTIAATDVSEPIQAKPIDPPTITVTFGINDSPLAGRDGKKVQSRVIRDRLLKEAETNVAIRVTDTPGGEAFEVAGRGELQMGVLIENMRREGFELSISRPQVLFREDENGKTLEPVEEVTIDVDDEYSGAVIEKITGARKGELAEMKPAGTGKTRIIAHVPSRGLIGYQGEFLTDTRGTGVLNRVFHGWTPHKGPIPGRRAGVLISMENGTSVAYALWNLEERGKMFIGAQADVYTGMIIGEHSRDNDLEVNPLKGKKLTNVRASGTDDAVRLTTPVTMSLEEAIAYIDNDELVEVTPNAIRLRKRHLDPHERKRHAR-AAS------------------MTKEMTGAKMVVQALKDQGVDTIFGYPGGAALPIYDEIFQQNDIRHVLVRHEQGAVHAAEGYARSTGKVGVVLVTSGPGATNTVTGLTDALLDSIPILVLSAQVPTFLIGSDAFQEADTVGITRPCTKHNWLVKDTDRLPAVLHEAFHVARSGRPGPVLVDIPKDVQFASGTYTPP-KPSTSHYQPRQKGSMEEITELVAAIEKAKRPVFYTGGGVINSGPAASQLLRELVDATGFPVTSTLMGLGAYPASGKNWLGMLGMHGLYEANMATHGCDLLINIGARFDDRITGRIDAFSPKSTKAHIDIDPSSINKVIKTDIPIVGDVGHVLEDILKVWKSRGRKTDAEALVKWNRQIDEWRKIECLRYTPSTTSIKPQYALQRLEALTKDRKDRFITTEVGQHQMWAAQFLNFEDPNHWMTSGGLGTMGYGFPASIGVQMAHREALVINVAGEASWLMNMQEMGTAVQYNLPVKQFILNNERLGMVRQWQQLLHGERYSQSWSEALPDFVKLAEAFGAKGILCKDPADLDDAIMEMLNYDGPVIFDCLVEKHENCFPMIPSGKAHNEMLLG-DAETKGAIDAKGSVLVMALKSYKPTTPGQRGLVLIDRSELWKGRPVKALTEGLTKSGGRNNTGRITSRRRGGGAKRLYRIVDFKRNKFDVSAVVARIEYDPNRTAFIALIQYDDGEQAYILAPQRLAIGDKVVAGSKVDIKPGNAMPFSGMPIGTIVHNIEMKPGKGGQIARAAGTYAQFVGRDGGYAQIRLSSGELRMVRQECMATVGAVSNPDNSNQNYGKAGRMRHYGKRPSVRGVVMNPIDHPHGGGEGRTSGGRHPVTPWGKPTKGAKTRNRNKASSKLIIRSRHAKKKGRMNQELTNNPFNPLTPPKVFDEIKVSLASPERILSWSYGEIKKPETINYRTFKPERDGLFCARIFGPIKDYECLCGKYKRMKYRGVVCEKCGVEVTLQKVRRERMGHIELAAPVAHIWFLKSLPSRIGLMLDMTLRDLERVLYFENYVVIEPGLTDLQYGQMLTEEEYMDAQDAYGMDAFTANIGAEAIREMLAQIDLESEAEQLRADLAEATGELKPKKIIKRLKVVESFLESGNRPEWMVMTVIPVIPPELRPLVPLDGGRFATSDLNDLYRRVINRNNRLKRLIELRAPDIIVRNEKRMLQESVDALFDNGRRGRVITGANKRPLKSLSDMLKGKQGRFRQNLLGKRVDFSGRSVIVTGPELKLHQCGLPKKMALELFKPFIYSRLEAKGLSSTVKQAKKLVEKERPEVWDILDEVIREHPVMLNRAPTLHRLGIQAFEPVLIEGKAIQLHPLVCSAFNADFDGDQMAVHVPLSLEAQLEARVLMMSTNNVLSPANGAPIIVPSQDMILGLYYVTLEREGMKGEGMVFGSIEEVQHALDAGVVHLHSKIQARITQIDDEGNEVQKRFETTPGRVRLGALLPLNAKAPFELVNRLLRKKEVQQVIDTVYRYCGQKESVIFCDQIMSMGFKEAFAAGISFGKDDMLIPETKWPIVEETREQVKDFEQQYMDGLITQGEKYNKVVDAWSKCNDRVTEAMMGSISASQRDADGSEREPNSVYMMAHSGARGSVTQMKQLGGMRGLMAKPNGDIIETPIISNFKEGLTVLEYFNSTHGARKGLSDTALKTANSGYLTRRLVDVAQDCIVRMRDCGTDNAITAEAAVKEGEVVATLAERVLGRVAAEDIKRPGTDEVLIAHGQLIDERMADTIDEANVQSARIRSPLTCEAEEGVCAMCYGRDLARGTMVNSGEAVGIIAAQSIGEPGTQLTMRTFHIGGVAQGGQQSYLESSQAGKVVYENAQTLENANGEILVMGRNMKLLIQDEDGEERASHKIGYGTKLFVKDGQMIKRGDKLIEWDPYTLPIIAEKAGTAKFVDLTSGIAVRDETDDATGMTQKIVIDWRAAPKGNELKPEIILVGEDGEPVRNEAGNPVTYPMSVDAILSVEDGNEVKAGDVVARIPREGAKTKDITGGLPRVAELFEARRPKDHAIIAEIDGYVRYGRDYKNKRRISIEPADESMEPVEYMVPKGKHIPVQEGDFVQKGDYIMDGNPAPHDILAIMGVEALADYMINEVQEVYRLQGVKINDKHIEVIVRQMLQKWEIQESGDTTLLKGEHVDKQEFDQANE---KALAKGGRPAQGEPILLGITKASLQTRSFISAASFQETTRVLTEASVQGKKDKLVGLKENVIVGRLIPAGTGGATQQVRRVAQDRDNVVIEARREEADAAAALAAPTD----EDIIGTDATYDPLIDTPESRD-------MSDVLHIVGGGMAGSEAAWQAAQMGVDVVIHEMRPEVETFAHKTGNLAEMVCSNSFRSDDSEQNAVGLLHWEMRAAGGIIMAQADAHRLPAGGALAVDRDPFAEAVTAQLMAHPQISVSYGEITELPDQGHW---IFATGPLTSSALGRAIQAETGSEALAFFDAIAPIVYFDSIDMSRAWMQSRYDKGDTEEERTAYLNCPMDRDQYEAFIDALLAADKAEFHEGETAG------YFDGCLPIEVMAERGRETLRHGPMKPVGLTNPHQPDVKAHAVVQLRRDNALGTLYNIVGFQTKMKYGAQADVFRMIPGLEDARFARLGGIHRNTFLNSPTLLDARMRLKSRPNIRFAGQITGVEGYVESSAMGLLAGRMAAGDILGTNIAPPPPTTAMGALVTHITGG--ADARTFQPMNVNFGLFPPVE----------GLKGGRRGRKDRYKAYTDRAKSDWRDWL-DATQSEAIA--MSKALIFPGQGAQTIGMGQALAEAYPVARAVFDEVDEALGEKLSELIWDGEADVLTLTQNAQPALMATSIAALRALEAE-GFKVSDAA-FVAGHSLGEYSALAAAGSLSVADAARLLRTRGRAMQEAVPVGLGAMAALLGLDLATAREVAAEAA---GDQVCEAANDNDPAQVVVSGHKAAVERAVEIAKTRGAKRAVLLPVSAPFHCALMAPAADVMAAALAEVEINAPVVPLVANVLAAPVTDPEQIRDLLVQQVTGAVRWRESVEAMAAAGVTEMYEVGAGKALSGMVRRINRDIACKPVGTPDDVKAALA------------------------MRDLKI------------------------PAQRHPEKAHRPDNAQPKKPAWIRVKAPGGKGYAETAKIMRDNKLTTVCEEAGCPNVGECWSQGHATMMIMGEVCTRACTFCNIATGKPPEALDAFEPGRVAHAVEKLGLNHVVITSVDRDDIEDGGADHFAQTIRAIRHRSPDTTIEILTPDFIRCGPEALEVVVEARPDVFNHNLETVPGLYPEVRPGARYFHSLRLLQRVKELDPSMFTKSGIMVGLGETRQQVVQVMEDMRAADIDFLTIGQYLQPTPKHHAIDRFVTPEEFANYEKTAFNKGFLMVSATPLTRSSYHAGDDFARLRDARQKKLGIA----MADLLTMD-KKSADKQKALDSALAQIERQFGKGSIMKLGAEGAIQEIEASSTGSLGLDIALGIGGLPMGRIVEIYGPESSGKTTLTLHCVAEQQKKGGICAFVDAEHALDPQYARKLGVDIDELLISQPDTGEQALEITDTLVRSGAVNMVIVDSVAALTPKSELEGDMGDSSVGVQARLMSQAMRKLTGSISRSNCMVIFINQIRMKIGVMFGSPETTTGGNALKFYSSVRLDIRRIGSVTDRDEVVGNATRVKVVKNKVAPPFKQVEFDIMYGEGISKMGELLDLGVNAGVVAKSGAWFSYGDERIGQGRANAKTFLKENPKIALEIEDKIRASHG-LEFDMPP---GSED-SDDDVLEA--MTQIDYTRAAKYFLLQDFAKGFMLGLKYMFKPKATVNYPHEKGPLSPRFRGEHALRRYPNGEERCIACKLCEAICPAQAITIDAEPR-EDGSRRTTRYDIDMTKCIYCGFCQEACPVDAIVEGPNFEFATETREELFYDKAKLLENGERWEAEIARNLEMDAPYRMPIKIPADLPAYEVLTREGVMVMSDDDADRQDIRPLRIGLLNLMPKKIQTENQFARLIGATPLQIELSLIRMSDHQSRNTAAEHMESFYRTFEEVEATGEKFDGLIITGAPIEHLPFEEVTYWDELTRVMDWTQAHVHSTFGVCWGGMAMAWHFHRVPKHILTDKAFGCFRHRNLAPASPFLRGFSDDVMMPVSRWTEMRQEDIDAA-GLTTLVTSDQTGPCLVHDPDHRAVYIINHLEYDSDTLKQEYDRDVDSGTPINVPANYYPDDDPSRRPMNRWRSHAHLLYGNWINQIYQTTPFDPAKIGG-------------MANYANLPAPTPEGGLNRYMQEIRKFPLLEPEEEYMLAKRWVEEQDTEAAHKMVTSHLRLAAKIAMGYRGYGLPQAEVISEANVGLMQAVKRFDPEKGFRLATYAMWWIRASIQEYILRSWSLVKLGTTSAQKKLFFNLRKAKARIGALEEGDLHPDNVKQIATDLGVTEDEVISMNRRMSGGDASLNATVGAEGEGTMQWQDWLEDEDADQASDYEARDELEARRDLLVQSFDVLNDREKDILTQRRLTEEAVTLEDLSGQYNVSRERIRQIEVRAFEKLQKRMRELASERGMIGAA-MAREDNRRG---NRRDR-------EETPEFADRLVAINRVSKTVKGGKRFGFAALVVVGDQKGRVGFGKGKAKEVPEAIRKATEQAKRQMIRVQLREGRTLHHDMEGRHGAGKVVMRTAPEGTGIIAGGPMRAVFEMLGIKDVVSKSIGSQNPYNMIRATIDGLKKEASPRSVAQRRGKKVADIL--PKREDAPQE---------SAQVAEEA--MNDPIADMLTRIRNSQLRGKSTVMTPASKLRAWVLDVLADEGYIRGYEKVTGADGHPAIEISLKYYEGIPVIREIKRVSKPGRRVYMGVSDIPQVRQGLGVSIVSTPKGVMSDANARAANVGGEVLCTVFMGKTTFGKGCHLHLIDGSAFIFRAYHALPPLTRKSDGLPIGAVSGFCNMLQRYVEDSTGDDAPTHVAVIFDKGSHTFRNDMYDQYKANREAMPEDLRPQIPLTRQATLAFNIACEEMEGYEADDIIATLSCQAREAGGRVTIISSDKDLMQLVGDGVEMLDAMKNKRIDREGVFEKFGVYPDRVVDVQALAGDSVDNVPGAPGIGIKTAALLINEYGSLEDLLERADEIKQPKRRQTLIDHRDQIELSKKLVQLDCDTPLTFTLDDLEVKEPDPDKLMPFLAEMEFRTLTKRIADKLGVEAPVVADAPAPAAE--SPEQAADAP---DFAKANYECIRDAKALQGWIERIREVGHVAVDTETTGLNEMTADLVGISLCVDAGHACYIPLTH---KDGNADDLFGSDTLLEGQMPLQEALDLLKPVLEDESVLKIGQNMKYDAKIFARNGVQVAPVDDTMLMSYALHGGLHNHGMDALSERYLGHTPIPIKPLLGSGKSAITFDRVPIDDAVPYAAEDADITLRLWQMFKPRLHAAQVTTVYETMERPMIPVLARMEMQGIKVDRDTLSRMSNAFSQKMAALEDEIHTLAGESFNVGSPKQLGEILFDKMNM--PGGKK---GKTGAYATGVDVLEDLATE-HELPARVLDWRQVSKLKSTYTDALQTHINDETGRVHTSYSIAGASTGRLASTDPNLQNIPVRTEEGRRIREAFVAEKGNVLVSLDYSQIELRILAHVADIPELKQAFADGVDIHALTASEMFDVPLEDMTPDIRRQAKAINFGVIYGISGFGLARNLRIPRAEAQGFIDRYFERFPGIRTYMDDTVAFAKEHGHVQTLFGRVIHTPEINAKGPHAGFAKRAAINAPIQGTAADVIRRAMVRMPAALADS--PARMLLQVHDELLFEVPEDGADALIDTARGVMENAADPAVQLSVSLSVDAGRGANWAEAH-MANHLYQNDLPDGLKLGPVVAIDCETMGLNPHRDRLCVVQLSDGDGNAHIIQIAKGQTSAPNLCAMLTDPDTLKLFHFGRFDIAAMFNAFGALAAPVYCTKIASRLVRTYTDRHGLKNLTQELLGLDISKHQQMSDWGAETLTEAQLEYAASDVLHLHQLRDRLNERLAREGRTELAQSCFDFLPARAQLDLAGWPEIDIFAHAMAKKAMIEREKKRERLVEKYAAKRAELKAIVNDESKPMEERFRASLKLAKLPRNSSATRLHNRCQLTGRPHAYYRKLKISRIALRELGSAGQIPGMVKSSW------------------MEWIVSDGLTPYDTALSWMEARVADIIAGRAEECIWLLEHPPLYTAGTSARPEDLTDPDRFPVFTARRGGQYTYHGPGQRVAYVMLDLNQRGRDVRAFVERLERWIIAALAEFNVTGDIREGRVGVWVERPDRPLRPDGTLSEDKIAAIGIRLRKWVSFHGISVNVDPDLSHFDGIVPCGISD--FGVTSLVELGLPVTMEDLDVALMHSFDATFGAEPVI-----------------MANVVVVGAQWGDEGKGKIVDWLSERADVIARFQGGHNAGHTLVIDGKVYKLNALPSGVVRGGKLSVIGNGVVLDPWHLVKEIASIREQGVEITPETLMIAENTPLILPIHGELDRAREEAASAGTKIGTTGRGIGPAYEDKVGRRSVRVADLADAETLSARVDRALQHHNPLRRGLGIDEIDRDALIAQLQEIAAEILPYAAPVWKVLNEKRKSGKRILFEGAQGALLDIDFGTYPFVTSSNVIAGQAATGVGIGPGSIDYVLGIVKAYTTRVGEGPFPSELHDADGQRLGERGREFGTVTGRKRRCGWFDAALLRQTCATSGVNGIALTKLDVLDGFETLKICVGYDLDGTRLDYLPMAAEQQARCTPVYEEMPGWSESTEGARSWADLPAEAIKYVRRVEELIECPVALLSTSPERDDTILVTDPFAD-MGFKMGIVGLPNVGKSTLFNALTRTAAAQAANFPFCTIEPNVGEVAVPDARLDRLAAIASSKQIIPTRMTFVDIAGLVKGASKGEGLGNQFLANIREVDAIAHVLRCFEDGDVTHVEGRVDPVADADTIETELMLADLESIEKRRANLVRKLKGNDKEAQQQDRLLAAAQAALEDGRPARTVEVDAEDARAWKGLQLLTTKPVLYVCNVAEDESATGNAHSARVAEMAAAQGNAHVIISARIEEEISQLETDEAEMFLSEMGLEEPGLDRLIRAGYELLQLETYFTVGPKEARAWTIRQGTSAPQAAGVIHGDFEKGFIRAETIAFDDFVALGGEQGAKEAGKMRAEGKSYVVKDGDVLHFLFNT---MQNVKVGIIMGSQSDWPTMKEAAEILDALSVAYEKRIVSAHRTPDRLWDYGKGAVDRGLQVIIAGAGGAAHLPGMMASKTRVPVIGVPVQTRALSGVDSLYSILQMPRGYPVATMAIGAAGAANAGLMAAGILALGDPELAQRLDTWRADLSASIPEEPRDE--MSINSFGHLFRVTTWGESHGPALGATVDGCPPGIAIDEAAIQHWLDRRKPGQNKYTTQRREPDAVRILSGVFEG------QTTGTPVQLMIENTDQRSKDYSDIMEKFRPGHADITYWQKYGIRDYRGGGRSSARETASRVAAGGLARAALASLAPSVEIQAYMVQVGPHGIDREQFDAGQIEANPFWCPDAGAASQWADYLDGLRKSG-NSVGAIVEVVARGVPAGLGAPIYGKLDTDLAAAMMSINAVKGVEIGEGMAAAALTGEDNADEIFMGNDGKPVYSSNHAGGILGGISTGQDVVVRFAVKPTSSILKTRQTITKSGQSAEIITKGRHDPCVGIRAVPVGEAMMACVILDHLLLHRGQIGGAVG-ETRGTIG---MFN---AVMQFDLGEDVNSLRDMVHRWAQERLKPMAAQIDSSNAFPPELWREMGDLGILGVTVPEEYGGAGMSYLAHVVAVEEVARASASVSLSYGAHSNLCVNQIKLNGTDAQRAKYLPGLVSGEHVGALAMSEAGAGSDVVSMKLRAEKRNDHFRLNGNKYWITNGPDADTLVVYAKTDPEAGSKGITAFLIEKSMKGFSTSPHFDKLGMRGSNTAELIFEDVEVPFENVLGEEGKGVRVLMSGLDYERVVLAGIGLGIMAACLDEVMPYMASRKQFGQPIGNFQLMQGKIADMYTAMNSARAYVYEVAKACDRGDVTRQDAAACCLYASEQAMVQAHQAVQAMGGAGFLNDAPVARIFRDAKLMEIGAGTSEIRRMLVGRELMGAMA-MSSTDFPG-WHGTTIIGVKKGGEVVIAGDGQVSLGQTVIKGTARKVRRLSPGGYDIVAGFAGSTADAFALLERLEAKLESTPGQLARASVELAKDWRTDKYLQKLEAMLIVSDGADIFVITGAGDVLEPEHDVTAIGSGGNYALAAARGMMDSDRSAEQVARDAMAIAADICVYTNGNLTVETIRK------------MKRTFQPSNLVRKHRHGFRARMATKAGRKILNARRARGRKSLSAMGNKVNPIGMRLQVNRTWDSRWYADTKDYGDLLLEDLKIRDFINEECKQAGVARVIIERPHKKCRVTIHTARPGVIIGKKGADIEVLRKKIAAMTNSELHLNIVEVRKPELDARLVGESIAQQLERRVSFRRAMKRAVQNAMRMGALGIRVNVAGRLGGAEIARTEWYREGRVPLHTLRADIDYAHVEATTAYGIIGIKTWIFKGEIMEHDPQARDRKAQE-----LQDGPAPRGA-GGRR-----MFYKDERLALFIDGSNLYAAAKALSFDIDYKLLRQEFMRRGKLLRAFYYTALLENDEYSPIRPLVDWLHYNGFTMVTKPAKEYTDSMGRRKVKGNMDIELAVDAMELAPRVDHIVLFSGDGDFRPLVESLQRQGVRVSVVSTIRSQPPMISDELRRQADNFIELEELKEVIGRPPRDHS-QDDSRVAAAGAR--MTKRTSAKYKIDRRMGENIWGRPKSPVNRREYGPGQHGQRRKGKLSDFGIQLRAKQKLKGYYGDLTEKQFRRIYGEAERVKGDTGENLIGLLERRLDAVVYRAKFVATIFAARQFVNHGHVKVNGQRVNIASYRVKEGDVIEVRDKSKQLAVLLEATQLPERDVPDYIEADHSKMTATFVRTPGLGDVPYPVMMEPNLVVEFYAKN---MIHKNWQELIKPTQLEVKPGNDPARQATVVAEPLERGFGLTLGNALRRILMSSLQGAAITSVQIDNVLHEFSSVAGVREDVTDIILNLKQVSLRMEVEGPKRLSINAKGPAVVTAGDISESAGIEVLNRDHVICHLDDGADLFMELTVNTGKGYVSADKNKPEDAPIGLIPIDAIYSPVKKVSYDVQPTREGQVLDYDKLTMKVDTDGSITPDDAVAYAARILQDQLSIFVNFDEP--ESASRQDDDDGLEFNPLLLKKVDELELSVRSANCLKNDNIVYIGDLIQKTEAEMLRTPNFGRKSLNEIKEVLSGMGLHLGMDVEDWPPDNIEDLAKKFEDAF-----MPFAHSDK----TMPMLNSPAPDVRSRE---------------KLEGGKRLVMNTEFEPAGDQPTAIAELSQGILDGERDQVLLGATGTGKTFTMARIIEQTQRPAIILAPNKTLAAQLYGEFKGFFPDNAVEYFVSFYDYYQPEAYVPRSDTYIEKESQINDQIDRMRHSATRALLERDDVVIIASVSCIYGIGSVETYGAMTQDLKVGHEYNQRQVMADLVAQQYKRNDQAFQRGSFRVRGDSLEIFPAHLEDRAWKLSFFGEELESITEFDPLTGERTGSFDQIRVYANSHYVTPKPTMQQAMVSIKKELRQRLDQLVGEGKLLEAQRLEQRTNFDLEMLEATGVCNGIENYSRYLTGRAPGEPPPTLFEFIPDNAIVFADESHVSVPQIGGMYRGDYRRKFTLAEHGFRLPSCMDNRPLKFEEWDAMRPQSVFVSATPGKWEIEQTGGVFTEQVIRPTGLLDPQVEIRPVT----TQVDDLLDEVRRVTEAGYRTLVTTLTKRMAEDLTEYMHEQGIKVRYMHSDIDTLERIEILRDLRLGAFDVLIGINLLREGLDIPECGLVAILDADKEGFLRSETSLIQTIGRAARNADGRVIMYADSITGSMERALAETDRRREKQIAYNEEHGITPATVRKNVDDIMAGL------YKGDTDMNRVTAKIDPKAS-----GGNLQTVLEGLRADMRKAAENLEFEEAARLRDEVKRLEAVDLAIHDDPMARQSAVEAASEAATKGKGRSTAGRPGQHGGNVR---RRKR---MQSQNIRIRLKAFDYRVLDASTQEIVNTAKRTGANVRGPIPLPNKIEKFTVLRGPHVDKKSRDQFEIRTHKRLLDIVDPTPQTVDALMKLDLAAGVDVEIKLQS-----------------------MFNISTKSMQWGEETLTLETGKVARQADGTVIATLGETSVMANVTFAKEQKPGQDFFPLTVHYQEKYYAAGKIPGGFFKREARPTEKETLTARLIDRPIRPLFVPGFKNEVLVMCTVLSHDLVNDPDVVAMIAASAALTLSGAPFMGPIAGCRVGYEDGEYILNPTVDDMQDLRLNPEQRLDLIVAGTKDAVMMVESEAYELTEAEMLGAVNFAHEQIQPVIDLIIALAEDAAKEPFTFTPPDYSALFDAVKAAGEEQMKAAYAILDKQERVAAVSAAKEAIIASLSEEQQDDANLG-----SALKKLESTVLRSTVVKDGKRIDGRALDQVRPIVAETGLLPRTHGSALFTRGETQGLVVTTLGTGDDEQFIDALHGNFKSNFLLHYNFPPYSVGEVGRVGSPGRREIGHGKLAWRALQAVLPAATDFPYTIRVVSEITESNGSSSMASVCGGSLSMMDAGVPLKAAVAGVAMGLVLEDDGSYAVLTDILGDEDHLGDMDFKVAGTENGITSLQMDIKVAGITPEIMEKALAQAKEGRLHILGEMSKAIT-GAGEFSVHAPRIETMQIPTDKIREVIGSGGKVIREIVEVSGAKVDINDDGIIKIASPNGDAIQKAYDMIHSIVAEPEEGAVYTGTVVKIVDFGAFVNFFGKRDGLVHVSQIENRRLNHPSDVLKEGQEVKVKLLGFDDRGKVRLSMKVVDQETGEEIK--KEESAE----MQIREALTFDDVLLVPAASSVLPSTADTRTRVTRAISMNIPLLSSAMDTVTESRMAITMAQAGGIGVIHKNLDVEAQAREVRRVKRFESGIVYNPVTLRPDQTLADAKALIERYNFTGFPVVDEQGRVVGIVTNRDMRFATSDETPVRVMMTSKDLAILTEPADRDEAISLMKSRRIEKLLVTDKNGKLTGLLTLKDTEKSVLNPTACKDLLGRLRVAAATSVGDAGFERTEALIDSGVDIVVIDTAHGHSAGVIEAVSRAKKLSNEVQVIAGNVATAEATRALIDAGADAIKVGIGPGSICTTRMVAGVGVPQLTAIMDCAAAAG--DVPVIADGGIKFSGDFAKAIAAGASCAMVGSMIAGTDESPGEVILYQGRSFKAYRGMGSIGAMARGSADRYFQKDAASDKLVPEGIEGQVPYKGSAGTVIHQLVGGLRAAMGYTGCATVEQMRSNCQFVKITGAGLKESHVHDVQITRESPNYRI--G-MAVLINENTKVICQGLTGSQGTFHTEQAIA-YGTKMVGGVTPGKGGQ-----THLDLPVFNSVHEAKHVTEANATVIYVPPPFAADSIMEAIDAEMEVIVCITEGIPVLDMMKVKRALEGSKSRLIGPNCPGVITPDACKIGIMPGHIHRRGSVGVVSRSGTLTYEAVKQTTDVGLGQSTCVGIGGDPIKGTEHIDVLEWFLADDETTSIIMIGEIGGSAEEEAAQFLADEKKKGRWKPTAGFIAGRTAPPGRRMGHAGAIVAGGKGDAESKIEAMKSAGIVVADSPAGLGEAVLKAI--G--MALERTFSIIKPDATRRNLTGKINAKFEDAGLRIVAQKRIHMTKEQAGVFYAVHAERPFYGELCDFMSSAPVVVQVLEGENAIAKNREIMGATNPADAAPGTIRAEFAESVGENSVHGSDAPETAAVEIAYFFSGLELVG-MSLNHVRPWRNIYRRKSRQIMVGSVPVGGDAPISVQTMTNTLTTDVAATVAQVQAAAEAGADIVRVSVPDQESSRALREIVAESPVPIVADIHFHYKRGIEAAEAGAACLRINPGNIGSPERVREVIQAARDHNCSIRIGVNAGSLEKHLLEKYGEPCPDAMVDSGLEHIRILQDNDFHNFKISVKASDVFMSAAAYQQLAEATDAPIHLGITEAGGLMSGTIKSAIGLGNLLWMGIGDTIRVSLSADPVEEVKVGYDILKSLGLRHRGVNIISCPSCARQGFDVIKTVEALEKRLEHIKTPMSLSIIGCVVNGPGEALMTDVGFTGGGAGSGMVYLAGRQSHKMSNDQMIDHIVEQVEQKAEQI--------ELETKA----AAEAAEMIPRYSRPDMVAIWSPETKFRIWYEIEAHACDAMADLGVIPRENADAVWKAKDVEFDVARIDEIEAVTKHDVIAFLTHLAEHVGSEEARFVHQGMTSSDVLDTCFNVQLVRASDILIEDVKQLLAALKRRAFEHKDTVRIGRSHGIHAEPTTMGLTFARFYAEMDRNLNRLEKARFEIATGAISGAVGTFANIDPAVEEHVCKKLGLEPEPISTQVIPRDRHAAFFAALGVVASSIENIAIEIRHMQRTEVLEAAEFFSMGQKGSSAMPHKKNPVLTENLTGLARLVRMTVVPAMENVALWHERDISHSSVERNIGPDATITLDFALARLTSVIDKLLVYPQNMLDNMNKFPGLVMSQRVLLALTQAGVSREDAYRLVQRNAMKVWDHRTDFREELLADEEVRAALSEDEINEKFDLGYHTKHVDTIFARVFKDS-MAHKKAGGSSRNGRDSAGRRLGVKLYGGQSAIAGNIIVRQRGTKFWPGEGVGLGKDHTIFATTDGAVTFHKGLKGRTFISVLPVAEAAE------------MLTGKRILLIIGGGIAAFKSQILIRKFQEVGATVTPVLTRAGAEFVTPLSLSGLSGQKTHTELFSLTDEVEMGHIELSRSADLIVVAPATADLMAKMAQGMANDLASTLLLATDTAVLAAPAMNVRMWQHPATQRNVAQLRADGVKMVGPGDGAMACGEFGPGRMAEPDEIVAAAAGML--GAG--PLAGKRIIVTSGPTHEPIDPVRYIANRSSGAQGTAIARALAALGAHVVFVTGPADVAPPEGVEVVRVQTAQQMSDAVDAALPAEAAVFAAAVADWRVASQSDRKLKKTRDG-MPKLEFAENPDILARVAQMATG-RPGLVVGFAAETNDVIENATAKRARKGCDWIVANDVSAATGIMGGTENAVVLISDDGAESWPRMGKDAVARQLADRIAAALA-----------MEEMLR---EYLPILVFLAVAIGFGLVLILAAVVIAVRNPDPEKVSAYECGFNAFDDARMKFDVRFYLVSILFIIFDLEIAFLFPWAVAFKDISMAGFWSMMVFLGVLTIGFAYEWKKGALEWQ-MSNKAVLKLIKDEDIEYVDIRFTDTRGKLQHVTVVADLVDEDFLEEGFMFDGSSVAGWKSIEASDMKLMPDTESAYIDPFYAEKTLCIHCSVVEPDTGESYERDPRGTAEKAEAYLKESGIGDVAYMGPEAEFFLFDNVKFSNTINKVSYEVDATDASWNTDADFE----MGNMGHRPGLKGGYFPVNPVDEAQDLRAEMLSTMKRLGMKVDKHHHEVASCQHELGLIFSTLTKQADELQKYKYVIHNVAHAYGKSATFMPKPIYGDNGSGMHVNMSIWKDGKPLFAGDKYADLSQEALYFIGGILKHAKSLNAFTNPGTNSYKRLVPGFEAPVLRAYSARNRSGCVRIPWTESPKAKRVEARFPDPSANPYLAFAALLMAGLDGIKNKIDPGEAMDKNLYDLPAEELAGIPTVCGSLREALTELEGDMDYLLAGDVFTKDQIEGYIELKMEEVLKYETTPHPVEFGMYYSC

>Brevirhabdus-pacificaDSM-27767

--------MKFTGTDSYVATEDLTIAVNAAVTLERPLLVKGEPGTGKTELARQVSAALGMPMLEWHIKSTTKAQQGLYEYDAVSRLRDSQLGDARVHDVANYIRKGKLWQAFEAEERVVLLIDEIDKADIEFPNDLLQELDRMEFHVYETGQTVRAVNRPVVIITSNNEKELPDAFLRRCFFHYIRFPGIETMRRIVEVHYPGIKDELLTTALTQFYEIRDTAGLRKRPSTSEVLDWLKLLLAEDLSAADIRREGKDAL-PKLYGALLKNEQDVQMFERLAFMAR--------GG--R------------MAALD---AEIREGVDRETVETVQSMA-GKYKHGWNTE-IEMEYAPKGVNPDIVRLISNKNEEPEWMTEWRLQAFERWTQLEEPTWAMVNYPEIDFQDQYYYARPKSMEEKPKSLDEVDPKLLATYEKLGIPLKEQMLLAGVEGAEDAGE--R-------KVAVDAVFDSVSVGTTFQAELKKAGVIFCSISEAIREHPELVKKYLGTVVPASDNFYATLNSAVFSDGSFVYVPPGVRCPMELSTYFRINAENTGQFERTLIIADKGSYVSYLEGCTAPQRDTAQLHAAVVEIVIEEDAEVKYSTVQNWYPGDENGKGGIYNFVTKRADCRGDRAKVMWTQVETGSAVTWKYPSCILRGDDSQGEFYSIAITNNYQQADTGTKMIHLGKNTKSRIVSKGISAGHAQNTYRGLVSMHPKAKNSRNYTQCDSLLIGSECGAHTVPYIEVKNNSSRVEHEATTSKVDDDQLFYCRQRGMDEEEAVALVVNGFCKEVLQALPMEFAMEAQQLVAISLEGSVGMKAGVIVFPGSNCDRDMAVAFEKA-GADVQMIWHKDSSLPSGLDVLGVPGGFSFGDYLRCGAIAAQSPICRAVVDFAGKGGHILGVCNGFQVLTETGLLPGALMRNSNLKFICKNVELTVATDDSAFTSGYERGAQITVPVAHHDGNYVIDQDGLSRLRDEDRIAFTYA--SNPNGSVADIAGVLSENRRVLGMMPHPERRVEAAHGGEDGAAMFRGLIDALVPAMTDLTPREIVSELDRFIIGQKDAKRAVAVALRNRWRRKQLGPELRDEVYPKNILMIGPTGVGKTEISRRLAKLARAPFIKVEATKFTEVGYVGRDVEQIVRDLLDSAIAMTREHMREDVKSAAHKAAEDRVIDALVGDQAREGTREMYRKKLKSGELDNTEIEIEVADAGPSL-PMMHIPG-QPGGGGM-GGMNLGDIFGKAFGGRTTKKRTTVAESYDILIGEEADKLLDDETVNRAAIEAVEQNGIVFLDEIDKVCARADARGGDVSREGVQRDLLPLIEGTTVSTKHGPVKTDHILFIASGAFHIAKPSDLLPELQGRLPIRVELRALTEEDFVRILTETDNALTRQYAALMATEEVAVTFTPEGIAALARIAADVNQSVENIGARRLYTVMERVFEELSFHAPDRSGAEVTVDAQFVEDNLGELTRSSDLSRYVL-------------MTLAQADGGLRIEHLRKSYRKRPVIRDVSMHLGRGEVVALLGPNGSGKTTCFYSIAGLVTPEGGKVLIDGRDATTLPMYRRAKMGIGYLPQEVSIFRGLSVEQNILAVLEVVEPKPNRRRERLEQLLSEFSIEHLKRAPALSLSGGERRRVEIARCLAANPKYLLLDEPFAGVDPIAVSEIRNLVADLKTRGIGVLITDHNVRETLEIVDRAYILHDGKVLMSGTTDEVVRDEHVRRVYLGQNFRIS-MSDRPLTPTLDRVNVPADMKSLSDAELHRLADELRRETISAVSETGGHLGAGLGVVELTVALHAVFDTPRDRIIWDVSHQCYPHKILTGRRDRIRTLRQKDGLSGFTKRSESPYDPFGAAHSSTSISAALGFAVARDLGGAPDPGLGDAIAVIGDGAMSAGMAYEAMNNAGHLGKRLIVILNDNEMSIAPPVGAMSAYLSRLYAGAPFQEFKAAAKGAVSLLPEPFQQGARRAKEMLKGMAVGGTMFEELGFSYVGPIDGHDLDQLLPVLRTVKARATGPILIHALTRKGKGYAPAEAAIDRGHGVARFDVVTGKQKKSPSNAPSYTSVFAKSLIRAGEADDRIVAITAAMPDGTGLNLFAERFPTRCFDVGIAEQHAVTFSAALAAGGMRPFCALYSTFLQRGYDQVVHDVAIQRLPVRFAIDRAGLVGADGATHAGAFDIAFLANLPDFVVMAAADEAELVHMVATAAAYDAGPIAFRFPRGEGVGVELPEKGQLLEIGKGRIIREGARAAILSFGTRLAEVEAAAETLAQMGITPTVADARFAKPLDRDLILDLAARHEAVITIEEGAVGGFGSHVAQLLSDEGVFDKGLRFRSMVLPDTFIDQASPADMYAMAELDARHIVQRVLDALGIADISS--R--RAMAKTGKRTAAAKAAFADKHDLTVEEAIALIKDNSKTKFDETVEIAMNLGVDPRHADQMVRGKVTLPNGTGKTVRVAVFARGAKADEAKEAGADIVGAEDLMETIQGGEINFERCIATPDMMPIVGRLGKVLGPRNLMPNPKVGTVTMDVAEAVKAAKGGEVQFRAEKAGVVHAGVGKASFEVDKLVENVRAFVDAVAKAKPTGAKGAYMKKVSLSSTMGPGVSVDITSATGN-MSADTKSSDLAALTIAGARDALRKGEVTSVELTQACLSRIEGADALGAFVHKTPEIALERARAADDRLAKG-DAPAMCGIPIGIKDLFCTEGVPSQAASRILEGFLPQYESTVSAKLRDAGSVMLGKLNMDEFAMGSSNETSVYGNAVNPWRRDGDDTALTPGGSSGGSASAVAADLCLGATGTDTGGSIRQPAAFTGITGLKPTYGRCSRWGVVAFASSLDQAGPMTKTVRDAAIMFGAMAGHDPKDSTSADLAVPDFEAMLTGDIKGKVIGLPREYRMDGMPAEIEKLWAEGADMLRDAGAEIRDISLPHTKYALPAYYVIAPAEASSNLARYDGVRYGHRATLAQGDGITEMYEKTRAEGFGPEVQRRVMVGTYVLSAGFYDAYYNRARKVRALIKRDFDQAFEAGVDAILTPTTPSAAFGLGEMTEADPVAMYLNDVFTVTVNLAGLPGIAVPTGTDAKGLPLGLQLIGRPWEEGELLNAAYALESAAGFVSKPAKWWMEKIPMTRSGHQALEAELKNLKSVQRPAVIRAIAEAREHGDLSENAEYHAAREKQSFIEGRVKELESIISRADVIDTSKMSGN-IKFGATVKLVDEDTDEEKTYQIVGEPEADIEKGRLNIKSPLAHALIGKEEGDSVEVRTPGGTRSYEVLEIGYHMAKKVAGTMKLQVPAGQANPSPPVGPALGQRGINIMEFCKAFNAKTQEMEQGAPCPTVITYYQDKSFTMDIKTPPASYLLKKAAKLKS--------GAKTPSRETAGYVTVAQVREIAETKMKDLSANDIEGAMQIILGSARSMGIEVKGMSE-SRHSKVLIIGSGPAGYTAAVYASRAMLEPVLIQGIQPGGQLTITTEVENWPGDIEVQGPDLMARMEAHARSVGTEIIGDIITELDLGSRPFTAKGDSGTTYTCDALILATGAQAKWLGLPSEEKFKGFGVSACATCDGFFYRGQEVVVIGGGNTAVEEALFLTNFASKVTLIHRRDELRAEKILQNRLFKNPKVEMLWNHEVTEVLGSENPLGVEAVRARNVQTGETTDVPCKGFFVAIGHAPASELVKDKLETHMGGYVVTKPDSTATSIPGVFAAGDLTDHIYRQAVTSAGMGCMAALEAERFLAEHEEQ----DDKDVSEPLGYGAPVEG----MRLSRYFLPVLKENPAEAQIVSHRLMLRAGMIKQSAAGIYSWLPLGLRVLKKIEAIVHEEQQRAGHIPLLMPTMQSADLWRESGRYEDYGEEMLRITDRHGRDMLFGPTNEELITDIFRSHVNSYKDLPLTLYQIQWKFRDEIRPRFGVMRGREFYMKDGYNFDIDRDSAIHAYNRHMVSYLRTYERMGLQAIPMRADSGPIGGEDTHEFLVLADTGESEVFYDSAITDLTLGERAVDYDDRTQCADIVTEWTTPYARTDETHDAALFDG-VPEDRRRTSRGIEVGQIFYFGTKYSEPMGATVVNDKGERVPVHMGSHGIGVSRLLGAIIEASHDDKGIIWPEGVTPFHCGIVNLRQGDAATDAACEDLYKALTAAGLDPLYDDRDERAGAKFATMDLIGLPWRITVGPRGLKSGVVELTDRRSGEAEELSPELAVTRLREIYAAHGI-----------------MDG---------QFDDALTGEQKIRNFNINFGPQHPAAHGVLRLVLELDGEIVERCDPHIGLLHRGTEKLMESRTYLQNLPYFDRLDYVAPMNQEHAWCLAIEALCGIEVPRRASLIRVLYSEIGRILNHLLNVTTQAMDVGALTPPLWGFEEREKLMVFYERASGARLHAAYFRPGGVHQDLPPELIDDIDTWADEFPAV-LADIDGLLTDNRIFKQRNVDIGVVNEQDILDWGFSGVMVRGSGLAWDLRRAQPYECYDEFDFQIPVGTAGDCYDRYLVRMQEMRESLKIIKQAIEKLR--VTPGDVLARG-KLTPPPRAEMKTSMESLIHHFKLYTEGFHVPAGEVYAAVEAPKGEFGVYLVADGTNKPYRAKLRAPGFLHLQAMDYISKGHQLADVAAIIGTMDVVFGEIDRMAAD-KQNLQDTFLNHVRKTKVPVTIFLINGVKLQGVITWFDNFCVLLRRDGQ-SQLVYKHAISTIMPAQPINLYDGDD---MSITVEDKQRLMKEFATKEGDTGSPEVQVAILSSRIATLTEHFKTHKKDNHGRRGLLKMVAQRRKLLDYTRAKDEARYQDLIKRLGLRRMAERLRERTEQLSDLAAHISGREEDAVLRTELLRGELTIEVAPASILTFTEFLLSDATSRFSTLVDITAVDYPDREKRFDVVYHFLSMYRNQRLRVRVSIREDEVLSSIIPVHPSANWFEREVFDMFGILFSGHPDLRRILTDYGFRGHPLRKDFPTTGYSEVRYDEALKRVVYEPVNLVQEYRQFDFMSPWEGADYILPGDD-KVAADGHVSGAAPAKPAAKG-----------MDKDGAQRLGVNIDHVATLRNARGSAYPDPLRAALLAEEAGADGITAHLREDRRHIVDADIDRLMERLRLPLNFEMAATDEMQAIALRHRPHAVCLVPERREERTTEGGLEVAGELDRLTRFIAPLREAGCRVSLFVAADEGQIDAAARSGAQVVELHTGAYCDLHAEGRLPERDAELARLAAAARQADDLGLEVHAGHGLTYDTVTPIAALPQVMELNIGHFLIGEAVFRGLGPAIAEMRRLMDAARA---------------MAAKPFFRRRKVCPFSGDNAPKIDYKDTRLLQRYISERGKIVPSRITAVSAKKQRELARAIKRARFLALLPYAVKMSKIALVDDDRNILTSVSMTLEAEGFEVETYNDGQTALDAFAKNMPELAVLDIKMPRMDGMDLLQRLRQKSKMPVIFLTSKDDEIDEVLGLRMGADDYVKKPFSQRLLIERIRALLRRQEALDGRA-EPETPDQTVMIRGQLQMDPLRHAVSWKGQEVTLTVTEFLLLQALAQRPGFVKSRDQLMDVAYDDQVYVDDRTIDSHIKRLRKKMRSVDPEFSAIETLYGIGYRYNEE--------------MGCRLNAYETEAMKELTARAGLENAVVINTCAVTAEAVRKARQEIRRMRRDHPEARIIVTGCAAQTEPETFAAMDEVDLVLGNNRKMQPETWANLA----ADFAGPTEKLLVDDIMSVTETAGHLIDGFGTRSRAYVQVQNGCDHRCTFCIIPYGRGNSRSVPAGVVVDQIRRLVDSGYNEVVLTGVDLTSWGADLPSTPQLGNLVQRILKLVPDLPRLRISSIDSIEADPALIEAIASEPRLMPHLHLSLQAGDDMILKRMKRRHLRDDAIRFCEDMRRARPGMTFGADIIAGFPTETEAMFENSMKLVEECGLTWLHVFPYSPRQGTPAARMP-QVHGTAIRERAQRLRDLGQARVGAHLNAQVGQAHRVLMENPRMGRTEQFAEVLFDSD-----RPEGQIVDARVTGVRDAQLLGA----MKLHELHDNPGATKKRKRVGRGPGSGTGKTAGRGIKGQKSRSGVSINGYEGGQMPLYQRLPKRGFTPPNRKKFAVLNLSLLQKFLDDKKIDGSKAITEDVLVESGLVRRKLDGVRILAKGEFNAKVNLEVTGASKSAIEAVEKAGGSLKVST-PAATSEQASEMARFVFITGGVVSSLGKGLASAALGSLLQARGFSVRLRKLDPYLNVDPGTMSPFEHGEVFVTDDGAETDLDLGHYERFTGVAARKTDSVSSGRIYSNVLEKERRGDYLGKTIQVIPHVTNEIKDF-ISIGEDEVDFMLCEIGGTVGDIEGLPFFEAIRQFAQDKPRGQCIFMHLTLLPWIGASGELKTKPTQHSVKELRSIGIAPDILVCRSDKPIPEKEREKIALFCNVRKEDVIAAPDLRSIYEAPLAYHREGLDQAVLDAFGIHPAPAPNLSIWEDVADRVFNPEGEVRVAIVGKYTQLEDAYKSIAEALTHGGMANRVRVKIEWIDAEIFE-REDPAPHLERFHAILVPGGFGERGTEGKIKAVEFARTRKVPYLGICLGMQMAVIEAARNVAGMTTAGSEEFDHEA-GEKRFEPVIFHLKEWIKDNETIARTVLDDKGGTMRLGEYDATLTEGSNVASVYGTRHIKERHRHRYEVDIKYREALEKVGLCFSGLSPDGRLPEIVEWKDHPWFIGVQFHPELKSKPFAPHPLFRDFIRAAKDTSRLV------MSASQSTPRSFQEIILRLQAYWASKGCAMLQPYDMEVGAGTFHPATTLRALGDRPWAAAYVQPSRRPTDGRYGENPNRLQHYYQYQVLIKPSPPDLQELYLGSLAAIGIDMAMHDIRFVEDDWESPTLGAWGLGWEVWCDGMEVSQFTYFQQVGGHDCKPVSGELTYGLERLAMYVLGVDHVMDMPYNDPDAPIPLSYGDVFRQTEAEYSRWNFDVADTETLRRHFEDAEAECQRILASPAEDPKTGRTIIMAHPAYDQAIKASHLFNLLDARGVISVTERQAYIGRVRALAKACADAFVQTPAGGVAA--------------MTKDYIVKDISLADYGRKELAIAETEMPGLMALREEYGASQPLKGARIAGSLHMTIQTAVLIETLTALGAEVRWASCNIFSTQDHAAAAIAAAGIPVFAIKGETLPEYWDYADKIFDFG---DQ--PANMILDDGGDATLYILIGARVEAGETDLIATPTSEEEEALFAQIRKRLKDSPGWFTRQRDAIKGVSEETTTGVHRLYELVKAGQLPFPAINVNDSVTKSKFDNKYGCKESLVDGIRRATDTMMAGKVAVVCGYGDVGKGSAASLRGAGARVKVTEIDPICALQAAMDGFEVVVLEDVVADADIFVTTTGNKDVIRIEHMREMKDMAIVGNIGHFDNEIQVANLKNHKWTNIKDQVDMIEMPSGNRIILLSEGRLLNLGNATGHPSFVMSASFTNQVLAQIELWTKGDEYKNDVYILPKHLDEKVARLHLAKIGVKLTELKKEQADYIGVPQEGPFKPEHYRYMSDVEDRVKKIVVEHLGVEEEKVTENASFIDDLGADSLDTVELVMAFEEEFGIEIPDDAAETIQTFGDAVKFIKEAQ-MTRAWSKSDWRNKPRVQMP-EYTDEAALQAVESQLSKYPPLVFAGEARKLKKSLAEVSEGRAFLLQGGDCAESFSEFNADNIRDTFKVMLQMAMVLTYGAKMPIVKVGRMAGQFAKPRSAPTETVNGVELPSYRGDIINGFEFTEADRVPDPQRMLQAYTQAAATLNLLRAFSHGGYADVHQVHAWTLGFTD-REEAEKYRDMASRISDTLDFMKAAGVTQDNAHTLQSVDFYTSHEALLLEYEEALCRTDSTTGLPVAGSGHMIWIGDRTRQPDGAHVEFARGVQNPIGLKCGPSTTADDLKVLMKKLNPANEAGRLTLIARFGAGQVGEHLPRLIRAVREEGAKVVWSCDPMHGNTIKSSTGYKTRPFESVLREVQEFFAIHNAEGTNPGGVHFEMTGRDVTECTGGVRAVTDEDLSNRYFTACDPRLNASQALELAFLVAEELSSRQET--IAAASMMAEQ--LQNAE-----EYGADSIKVLKGLEAVRKRPGMYIGDTDDGSGLHHMVYEVVDNGIDEALAGHADAVTVRIHADSSVSVSDNGRGIPVGIHEEEGVSAAEVIMTQLHAGGKFDQNSYKVSGGLHGVGVSVVNALSDWLELRVWRDGKEHYARF-ERGDTAEHLTVVGDANGR------KGTEVRFLASTDTFSNLEYSFKTLEHRLRELAFLNSGVKIVLEDLRPAEPLRTELVYEGGVREFVKYLDRSKTPVMDAPIYVSGERDGIGVEVAMWWNDSYHETVLPFTNNIPQRDGGTHMAGFRGALTRTINNYAQSSGIAKKEKISFTGDDAREGLTCVLSVKVPDPKFSSQTKDKLVSSEVRPAVEGLMNEKLAEWFEENPNEARQIVGKIVEAAMAREAARKARELTRRKSAMDVNFLAGKLKDCSEKDPSKTEVFLVEGDSAGGSAQTGRDRRTQAVLPLRGKILNVERARFDRMLGSQEIGNLVMALGTGIGRDEFDISKLRYHKIVIMTDADVDGAHIRTLLLTFFFRQMPELIEGGYLYIAQPPLYKVARGKSEVYLKDQTALEDYLIQQGVDGAVLRLGTGEEIMGQDLARVVEEARQTRRILQAFPTHYPSHILEQAAIAGAFKPGVVDADLQGTADRVAERLDLIAVEYERGWQGRQTQDRGLRLNRTLRGVEEVRTLDGQVLRSGESRRLGQMTDALQEVYDKPAKLVRKDREQL-IYGPVDLLDAILTEGEKGLSLQRYKGLGEMNPDQLWETTLDPEARTLLQVRIEDLAEADDIFTKLMGDVVEPRREFIQTNALNVENLDA-----MALKIAFQASESPEARQAFDRLTARYGNMPPEDANVIVALGGDGFMLQTLHGSRSLGLPTYGMNCGTIGFLMNEYSEEALNERLLAAEQEVINPLAMTATTADGQIVEALAVNEVSLLRAGPQAAKLRITVDGKERLSELVCDGALVCTPAGSTAYNYSAHGPILPIGSDVLALTAMSAFRPRRWRGALLPKTAKVRFDVLEHEKRPVMADADSRSVRNVVRVDIESEPTVAHRILFDPGHGLEERLIQEQFT-MLGLGTIAKKVFGTPNDREVKARRPLVEKINALEPQFTDLTDEQIIEKTAEFRERLAKGEALDDLLPEAFANVREAARRTLGLRAFDVQLMGGIFLHQGNIAEMKTGEGKTLMATFPAYLNALTGRGVHIVTVNDYLARRDAEWMGKVYAALGLTTGVVYPQQPEQEKAEAYGADVTYATNNELGFDYLRDNMRMDLDEMNQRDHYFAIVDEVDSILIDEARTPLIISGPSEDRSELYKTIDTVVPSLSDEHFTIDEKTRNVTYTDEGNEFLEQELLRQGILPEGQSLYDPESTTLVHHVTQALRAHKLFQRDKDYIVRDDEVVLIDEFTGRMMSGRRLSDGLHQAIEAKENVSIQPENVTLASVTFQNYFRLYDKLGGMTGTAATEAEEFAEIYKLGVIEVPTNRPIARVDEHDAVYRTAKEKYDAIRETIEEAHKKGQPILVGTTSIEKSEFLSQMLKERGVPHNVLNARQHEQEAAIVADAGKPGAVTIATNMAGRGTDIQLGGNVDMKVLEALATD--------PEADPAALRERAEAEVAGDKKKVLEAGGLFVLATERHESRRIDNQLRGRSGRQGDPGRSAFYLSLEDDLMRIFGSERLDNVLGKLGMKEGEAIIHPWVNKSLEKAQAKVEARNFDIRKQLLKFDDVMNDQRKAIFGQRLEIMESKDVNEIVEDMRHQVIDDLVDFYIPARSYADQWDGEGLYAAVIEKLGVDAPVIAWTQEEGVDDSDIRERLYKATDEFMAGKAAKFGPEQMRRIEKQVLLQTIDAKWREHLVTLEHLRSVVGFRGYAQRDPLNEYKNESFQLFESLLNSLREEVTEKLAQLRPLSEEEQKQMLAQLIEQQRALQGAEAGAQPGAAPAAG-----------PQAAAQPDGAGE------------------TAVAEVGRNDPCPCGSGKRYKHCHGAVTA--------------------------------MGVCRPDAVPELAGRLEAFVAAGRHGQMGWMEERRNWRGNPAALWPEARSIVMLADLYTPHADPLEVLERPDRGAISVYARNRDYHDVVKKRLKKVGRWLLE--QSRGE--EIKVFVDTAPVMEKPLAAAAGLGWQGKHTNLLSRDLGNWFFLGAIFTTVELPPDTPGRENCGSCTACLDICPTAAFPAPFQLDARRCISYLTIEHHGPVPEELRPALGNRIYGCDDCLAVCPWNKFARDAQEIRYHARDDLDAPELSRLAALDDAGFRALFSGSPIKRIGRARFLRNVCYAIGNSGRPDMLPSLGPLLDDPDPALADAARWAVARLRDHAA------------------------------MFEKILIANRGEIALRVIRACREMGIRSVAVHSTADADAMHVRMADETVCIGPPSSTDSYLSIPAIISACEITGAQAVHPGYGFLSENQNFVQILEDHDITFIGPTSEHIRVMGDKITAKETMRKLGVPCVPGSEGGVPDLATAQTVAEEIGYPVIIKATAGGGGRGMKLARTAEELPDAFRTARAEGKSNFGNPEVYIEKYLGTPRHIEIQVFGDGKGNAVHLGERDCSLQRRHQKVFEEAPGPSIDAETRARIGKTCADAVAAINYAGAGTIEFLYENGEFYFIEMNTRLQVEHPVTEAIFGVDLVREQIRVAEGLPMSFTQDDLEINGHAIEVRINAEKLPNFAPCPGKITQYHAPGGLGVRIDSALYDGYRIPPYYDSLIGKLIVHGRDRNEALARLHRALAELIVDGVDTTVPLFHALLQEPDIQKGDYNIHWLEKWLETNLGS----MSEPDALLRPQAGAEDARAEADRALRPQALDEFVGQAEARANLRVFIQSARQRGEAMDHTLFHGPPGLGKTTLAQIMARELGVNFRMTSGPVLAKAGDLAAILTNLEARDVLFIDEIHRLNPAVEEVLYPALEDFELDLVIGEGPAARTVRIELQPFTLVGATTRLGLLTTPLRDRFGIPTRLQFYTVDELHLIVTRGARLLGIPCEDEGAREIAKRARGTPRIAGRLLRRVVDFALVEGDGRLTREIADSSLTRLGVDHLGLDGADRRYLSLIAENYAGGPVGIETIAAALSETRDALEEVIEPFLLQQGLIQRTPRGRMLTQGAWRHLGLAAPEPPRQQGLF--ND---MPLDTPAHP---INSLGFAKPPSETRVVVAMSGGVDSSVVAAMLAEEGYDVVGVTLQLYDHGAALAKKGACCAGRDIHDARRVAEAMGFPHYVLDYENVFRDAVMDEFADSYLGGATPVPCIRCNERVKFKDLLETAKDLEADCMATGHYIARADGPGGPELHSAADPARDQSYFLFSTTPEQLSFLRFPLGHLKSKAETRALAAKYGLMVADKPDSQDICFVPDGNYASVIEKLRPGAAEPGEIVDTEGRVLGTHRGVIHYTIGQRRGLGIGGLSDPLYVVKLDVDNKRVVVGPKSDLATRQVPVREINWLGEAPFDSREEW--HVAVKVRSTRPPTPAVIRPLSPTTATVELAEAEEGVSPGQACVFYDTE-SSRIFGGGWIWRGY------------MADQ--KKHATLEFGDTRLELPMMSPTLGPDVIDIRKLYAEGDVFTYDPGFTSTASCESTITFIDGEKGELLHRGYPIDQLAEKSHYLEVCYLLLYGELPSVEMLEDFEARVNRHTMLHEQMMNFFRGFRRDAHPMAIMTGVVGAMSAFYHDSTDISDPWQREVASIRMIAKLPTIAAMAFKYSVGQPFVYPRNDLDYASNFLRMCFAVPAEDYVVDPILSRAMDRIFTLHADHEQNASTSTVRLAGSSGANPFACIAAGIACLWGPAHGGANQACLEMLREIGTVDRIPEYIARAKDKSDPFRLMGFGHRVYKNFDPRAKVMKQSADEVLDLLGFENNPTLQVAKELEAQALQDPYFTDKKLFPNVDFYSGIILEAMGFPTSMFTPIFALSRTVGWISQWKEMIDDPVQRIGRPRQLYTGAPRRDYVDIENRMAEQKKIEVRGAREHNLKSIDVDIPRDELVVITGLSGSGKSSLAFDTIYAEGQRRYVESLSAYARQFLDMMEKPDVDHISGLSPAISIEQKTTSKNPRSTVGTVTEIYDYMRLLFARAGTPYSPATGQPIEAQQVQDMVDRVMAMEEGSRAYLLAPIVRDRKGEYRKELLELRKQGFQRVKIDGAFHELDEAPKLDKKFRHDIDVVVDRIVVRKGLETRLADSLRTALDLADGIAILE-T--AP-------REEGA---EPERITFSEKFACPVSGFTIPEIEPRLFSFNAPFGACPECDGLGVELFFDERLVVPDAALQIGDGALAPWRKGKSPYFTQTIEAIARHYEFDRATPWKDLPAHVQQVFLHGSGEEEIRFRYDEGGRVYEVSRPFEGVIPNMERRYRETDSAWVREEFERYQNNRPCHVCEGYRLRPEALAVRIA-----G--------CHIGQVVEMSIREAHAWCEDVPAHLSKQKNEIARAILKEIRERLGFLNNVGLEYLTLSRAAGTLSGGESQRIRLASQIGSGLTGVLYVLDEPSIGLHQRDNDRLLLTLKNLRDQGNTVIVVEHDEEAIREADYVFDIGPGAGVHGGQVVAHGTPAEIIANPDSVTGDYLAGRRAIEVPGSRRKGNG--KKVTVVKASGNNLRDVTVDFPLGRFVCVTGVSGGGKSTLTIETLFKTASMRLNGARQTPAPCETIKGLEHLDKVIDIDQRPIGRTPRSNPATYTGAFTPIRDWFAGLPEAKARGYKPGRFSFNVKGGRCEACQGDGVIKIEMHFLPDVYVTCETCNGARYNRETLEIRFKGKSIADVLDMTVEDAQEFFKAVPSIREKMDALCRVGLGYIKVGQQATTLSGGEAQRVKLSKELARRSTGRTLYILDEPTTGLHFEDVRKLLEVLHELVEQGNTVVVIEHNLDVVKTADWVVDIGPEGGDGGGQVVATGTPEKIAGVEGSHTGQYL------------------------------------APMLKPRK-MAAE------MTDK-------TQNTMWGGRFAAGPDAIMEAINASIGFDKRLYAQDIAGSRAHAAMLAASGILSEADADAIGEGLTAILGEIEQGDFPFSTALEDIHMNVESRLRERIGDAAGRLHTARSRNDQVATDFKLWTRDQIDAATRGLEALIDALLGQAEAGADWVMPGFTHLQTAQPVTWGHHMMAYVEMFGRDLSRFRDARVRMNECPLGAAALAGTSFPIDRHVTAEALGFDRPSANSLDAVSDRDFALDYLSAATICAMHLSRFAEELVIWSSAQFRFVALSDRFSTGSSIMPQKKNPDAAELIRAKIGRILGAMVALFTVMKGLPLAYSKDMQEDKEQVFDAADSLMLALAAMEGMVRDMTANRESLAAAAGSGFSTATDLADWLVREAGLPFRDAHHATGALVAMAEKKGCDLPDLTLEEMQSVNPAITQGVFDVLGVENSVASRVSYGGTAPAQVRKQVARWREVQAGWTA--MKKIYGSAAEALEGVLSDGMFIAAGGFGLCGIPENLIAAIRDAGTQNLTVASNNAGVDDFGLGVLLQTRQVKKMISSYVGENAEFMRQYLSGELELEFNPQGTLAERMRAGGAGIPGFYTKTGVGTQIAEGKEHKDFDGETYILERGIVADLSIVKAWKADDTGNLVFRKAARNFNPPAAMCGRICIAEVEEIVPRGSLDPDNIHLPGIYVHRLIQGEHEKRIEQRTIRAA---MKKDTHPDYHLIDVKLTNGDIVQMKSTWGAEGETLALDVDPSVHPAWTGGGARLMDTGGRVSKFKKKYEGLGFMGYKVAVVGATGNVGREMLNILAERQFPVDEICALASRKSLGSEVSFGDKTLKTKDLDTFDFTGWDIALFAVGSDATKIYAPKAAKAGCVVIDNSSLYRYDADVPLIVPEVNADAITGYTKKNIIANPNCSTAQMVVALKPLHDRARIKRVVVSTYQSVSGAGKAGIDELWDQTKSIYNPVDNKPPTKFTKQIAFNVIPHIDVFMEDGSTKEEWKMVVETKKIVDKDIKVTATCVRVPVFVGHAESINIEFEEFLDEDEAREILREAPGVMVIDKREDGGYVTPTECVGDFATFISRIRQDGTIENGLNLWCVSDNLRKGAALNAVQIA-ETLGQRCLKKG--------MRIVFMGTPDFSVPVLDALIAAGHEIAAVYCQPPRPAGRGKKPRPTPVHARAEALGLEVRHPETLRDADEQARFAALEADVAVVVAYGLLLPQAVLDAPRHGCLNIHASLLPRWRGAAPIHRAIMAGDARTGVCIMQMEAGLDTGPVLLRAEIPIGPTDTTGDLHDALSALGAARIVEALERLEG--LTPVPQPEEGVTYAAKIDKAEARIDWSRPAAEVDRKIRGLSPFPGAWTEMA----GERVKLLRSRVAEG-GAG--APGEVL-TG----FTIACGEG-AVEVLEAQRQGKRAAGAAEFLRGNTLP--ERMGD--MSIPQS----GGGPIERHEQLAEYLAEGCKPRDQWRIGTEHEKFGYNRETLLPLPYEGDCSVRAILEGLRDRFGWAPVEEGGKIIGLLKDGANVSLEPGGQLELSGAPLETIHETCDEVNVHLREVQSVADDIGAGFIGLGAAPEWTHDQMEMMPKGRYRLMTDYMDRVGSMGKVMMYRTCTVQVNLDFESEADMVRKLRVALALQPVATALFANSPFFEGKPNGHKSWRSRVWRDLDPARTGMLPFVFDEGFGFEAWVDYALDVPMYFVYRDGTYIDALGQSFRDFMRGELPALPGETPSLSDWADHLTTIFPEARLKKFIEMRGADGGPWRRLCALPAFWAGLTYDQGALDAAWDLVRGWSAEQREALRVAASVDGLQAEA-GGIRMHDLAREVLGIAEQGLKSRARPGAGGLVPDETHFLNALRESVESGTVPADELLAKYHGEWDGDLSRIYAEYSYMVVLSKIYTRTGDAGDTALGDGMRVRKDAARVEAYGTVDETNATVGLARLHAPKGQSAAPDGALDMDARLAAIQNDLFDLGADLCRPGHDRDAEAEYPPLRVTEAQVTRLEHEIDTMNTALTPLRSFVLPGGSALAAQLHLCRVVSRRAERLTVTLAVNEP--VNPAALRYLNRLSDWFFCAARIANDNGAADVLWVPGANR-EGMRHARGYRRLNRTHEHRKALFANMAGSLIEHEQIKTTLPKAKELRRIVEKLITLGKRGDLHARRQAGAQLKQDEYVAKLFDVLGPRYAERQGGYVRVLKAGFRYGDMAPMAIIEFVDRDVDAKGAGDRARLEAE---E-AAE---MRIGLYPGTFDPITLGHIDIIRRAALLVDRLVIGVAINRDKGPMFTLEERVAMIEAECAELSAESGTEIVVHPFENLLIDCARDVGAQIIVRGLRAVADFEYEYQMVGMNRVLDSSIETVFLMADARHQAIASKLVKEIARLGGDVSKFVTEPVDTALRDRYAQG--MFDLSGKNALITGASGGIGGSIARALHGAGATVALSGTREAPLQELAQELGERAHVLPCNLSDAEAVEGLPKAAAAAMGSVDVLVNNAGITRDNLFMRMSDEEWASVLEVNLTSTMRLCRGVMRGMMKARWGRIVNISSVVGATGNPGQANYAASKAGMVGMSKSIAYEVASRGITVNCVAPGFITTPMTEKLTDDQKAGILQQVPAGRMGEADEIGAAVLYLASPEAAYVTGATLHVNGGMAMIMALDTNPPIAAYDAPEKDLYGLGEMPPLGHVPAKMHAWAIRRERHGEPDESFQLETVETPAIDSNEVLVLVMAAGVNYNGVWAGLGVPISPFDVHKAPYHIAGSDASGIVWAVGDKVTRWKVGDEVVIHCNQDNGDDEECNGGDPMFSDSQRIWGYETPDGSFAQFTRVQAQQLMPRPRHLTWEESACYTLTLATAYRMLFGHHPHELKPGQNVLVWGASGGLGSFAIQLINTAGANAIGVISEEDKREFVMSLGAKGVINRKDFDCWGQLPKVNTDEYKAWFAEVRKFGKAIWEITGKGNNVDMVFEHPGEATFPVSTFVCKKGGMVVICAGTTGYNLTLDARYLWMHQKRVQGSHFAHLKQAAAANKLMVERRLDPCMSEVFPWEDIPAAHIKMLRNEHKPGNMAVLVQAPRTGLRTFEDT-LEASRQAR-----MDGDFRTSFVREPGGLKRHPALVLNADFRPLSYYPLSLWPWQEAVKAAVLDRVGILAEYDEVVRSPTTTIRIPSVVVLKDYVKPQKRVAFTRFNLFLRDGFCCQYCGSRGDLTFDHVVPRARGGVTSWSNVVAACSPCNLRKGSRMLAQTGMQLRKPPRRPRAEELKNMGRRFPPNFLHDSWLDFLYWDAELDS-MTYIVNDNCIACKFTDCVEVCPVDCFYEGENMLVIHPDECIDCGVCEPECPADAIRPDTEPDTD-KWVEFNRKYSEMWPVIITKKDPLPDAEKHDGEEGKLEKYFSEKPGEGGMTDQ---------SNNDAFHASSFLQGHNAEYIEQLHARYAEDPNAVDEAWQQFFRQMGDTEVDAKREAAGPSWARRDWPPQPDDELTHALDGQWA----GEEQKHAGEKIRAKAQAAGVSVSDEAVRRAVLDSVRAIMIIRAYRIRGHLVADLDPLGMRDQT-PHPELDPRSYGFTEADMDRPIFIDNVLGLEVASMREILAIVKRTYCGTFALQFMHISDPEQSSWLKERIEGFNKEIRFTREGRRAILNKLVEAEGFEKFLHVKYMGTKRFGLDGGEALIPAMEQVIKRGGALGVEEIVIGMPHRGRLSVLANVMGKPYRAIFNEFQGGSFKPDDVDGSGDVKYHLGASSDREFDGNTVHLSLTANPSHLEAANPVVLGKARAKQ----NQINDADRRRVLPLLLHGDAAFAGQGVVAECFGLSGLKGHRTGGTMHIVVNNQIGFTTAPHFSRSSPYPTDIALMVEAPIFHVNGDDPEAVVHAAKVATEFRQKFCKDVVIDIICYRRFGHNEGDEPMFTNPVMYKRIKRHKTTLQLYTETLVRDGLIPEGEIEDMKAAFQSTLNTEFEAGKDYRPNKADWLDGRWSHLDPKGK-KYQRGKTAIKPEVYDALATALTTAPDGFELHRTVERLLETKARMFETGEGFDWATGEAMAFGSLMLEGYPVRLSGQDSTRGTFSQRHSALVDQKTEERYYPLDNIRAGQARFEVIDSMLSEYAVLGFEYGYSLAEPNALVGWEAQFGDFANGAQIMFDQFISSGERKWLRMSGLVMLLPHGFEGQGPEHSSARLERFLQMSAEDNWIVANCTTPANYFHILRRQLHRSFRKPLVLMTPKSLLRHKLAVSRKEDFVTGSSFHRVLWDDAQQGNSE--TKLVGDDKIRRVVMCSGKVYYDLLEERDARGIDDVYLLRIEQFYPFPALSLVKELERFKDAEMVWCQEEPKNQGAWTFIEPNIEWVLTRIKARLQRPVYVGRNASASPATGLASQHKAQQEALVDEALTIEG---------MIGLEHYLTVAAALFVIGIFGIFLNRKNVIIILMSIELMLLAVNINFVAFSAFLNDLAGQVFTLFVLTVAAAEAAIGLAILVCFFRNRGTIDVEDVNVMKGMNKVIRE--AATILPLNQILDGDCIDLMNGLPAESVDLIFADPPYNLQLKGDLHRPDNSRVDAVDDHWDQFASFQVYDDFSREWLKAARRLLKPNGAIWVIGSYHNVFRLGAALQDAGFWILNDVVWRKSNPMPNFRGKRLTNAHETLIWAAKSEDSKYTFNYEALKALNEGIQMRSDWVLPICTGHERLKDDNGDKAHPTQKPESLLHRILIGTTNAGDVVLDPFFGTGTTGAVAKKLGREYIGIEREAAYREVAEKRLKSVRKFDRSSLEVTTSKRAEPRVPFGQLVERGMLSPGEELLSMNGRYRAKVRADGTLIGNDVKGSIHQVGAALEGAPSCNGWTYWCYKRDGKRV-PIDLLRQQIRAEMVERPNMLDAATYTPRLKTLYTDEIRAKLKEEFGYANEMQIPRLDKIVLNIGCGAEAVRDTKKAKSAQEDLTAIAGQKALITKAKKSIAGFRVREEMPLGAKVTLRGERMYEFLDRLITVAMPRIRDFRGVSGKSFDGRGNYATGLKEHIVFPEINFDKVDEVWGMDIVICTTAGTDAEAKALLKHFNMPFNSMSDS-ARETMEYDVVIVGAGPAGLSAAIRLKQL----NADLDVVVLEKGSEVGAHILSGAVLDPSGLDALLPDWRNMGA-PINVEVKRDEFHMLGESGSVRIPNFLMPPLMSNHGNYIVSMGNVCRWMAEQAEALGVEIFPGMSCSALVYREDGSVKGVVAGEFGLNTDGTPGPNYEPGMELHGKYVLLGEGVRGSLSKQVIEKFALDGDSDVQKYGLGMKEIWEIDPAKHQQGKVVHTMGWPLG-GNAGGGSFIYHLDNNQ---VYVGFVVHLNYANPYLFPYMEFQRFKHHPMVAELLEGGKRVAYGARAISEGGWQSLPKLAFPGGALLGCSAGMVNVPRIKGNHNAMLSGKAAAEAIVEALAAGRSADVLDGYDGAVRDGAIGRDLRPVRNVKPLWSRFGLLASLTVGGLDMWTNNLFGFSLFGTLKHQKTDAEATELAEQHKPIDYPRPDGVLSFDRLTNVSFSMTNHEENQPCHLKLRDADTPVKVNLPLYAGPSARYCPAGVYEFVGE--GEAVKFQINFQNCVHCKTCDIKDPSQNIVWTTPQGGDGPNYPNMMTTGRVNPAMTLEKQGISGLKAVHYNLLEPALIEESLKRGESTLGKGGTILATTGAHTGRSPKDKFTVKSESNADKIWWENNPPMEAEAFDRLYEDMIAHMQGGEYFVQDLYACADPEHRLNVRVVNELAWHQLFIRYMLRRPETSELDSFVPDFTVINCPSFKADPERHGCRSETVIAVNFDRKMILIANTAYAGENKKSVFSLLNYMLPEKGVMPMHCSANHAKGNPVDTAVFFGLSGTGKTTLSNDPNRTLLGDDEHGWSDRGTFNFEGGCYPKTINLSQEAEPDIYATTTMFGTVIENMVHDPYTKELDFDDDSLTANMRCAYPMEFIDYASDTGLGGHPKNIVMLTCDAFGVLPPISRLSPAQAMYHFLSGFTSKVAGTERGVTEPEPTFSTCFGAPFMPRRPEVYGKLLSEKIAKHGATCWLVNTGWTGGAYGTGSRMPIKATRALLTAALDGSLNDVEFRKDPNFGFEVPVSVPGVDDKLLDPRNTWDDPAAYDAQARKLVQMFADNFAQYEANIDEDVRAVAIG-----MTKFDDAWVAAEEAKRKFMAENSLYHEESEHASCGVGLVVSVDGSKSRAVVENGIAALKAIWHRGAVDADGKTGDGAGIHVQIPVEFFYDQIERTGHKPNKNHQIAVGQVFLPRTNFGAQEICRTIVESEVLRMGYYIYGWRHVPVNIDCLGEKANATRPEIEQILISNSKGVDEETFERELYVIRRRIEKAAAAAQVPTLYLASLSCRSIIYKGMMLAEQVAEFYPDLMDERFESAFAIYHQRYSTNTFPQWWLAQPFRMLAHNGEINTLKGNVNWMKSHEIRMASNYFGDMAEDIKPIVAAGSSDSAALDSVFEVLVRAGRNAPMAKTMLVPESWSKQARDLPQAWRDMYSYCNSVMEPWDGPAALAMTDGRWVCAGLDRNGLRPMRYVVTGDGLVIAGSEAGMVPIDEARVKEKGALGPGQMIAVDMKEGKLFHDTEIKDKLANARPYADWVKRIVDLEAKTTLDQGKPIYAGSELRKRQIAAGFSVEELEQVLAPMAEDGKEMVASMGDDTPSAVLSSKYRPLSHFFRQNFSQVTNPPIDSLREYRVMSLKTRFGNLKNVLDESSSQTEILSLDSPFVSNAKFEALLEQFGEAVVTIDCTFAYDA---GPAALSEGLTRIRAEVEDAVASGAAHIVLTDQNQGPERVAMPMILATSAVHSWLTRKGLRTFCSINVRSAECVDPHYFAVLIGAGATTVNAYLAQDSLADRVERGLL-DCTVTEAVRNYRTAIDQGLLKIMSKMGISVISSYRGGLNFEAVGLSRAMCAEYFPGMLSRISGIGVSGIQRKLEEVHALGWRGERDILPIGGFYKLRRSGEKHAWEAQTMHLLQAACNKASYEMWKQYSKAMQSNPPIHLRDLLAIKPMGTPVPIEEVESITSIRKRFVTPGMSLGALSPEAHKTLNVAMNRIGAKSDSGEGGEDPAHFVPEANGDNPSAKIKQVASGRFGVTAEYLNQCEELEIKIAQGAKPGEGGQLPGMKVTELIARLRHSTPGVTLISPPPHHDIYSIEDLAQLIYDLKQINPRCKVTVKLVASSGVGTIAAGVAKAKADVILISGHNGGTGASPATSIKYVGLPWEMGLTEAHQVLAMNNLRGRVTLRTDGGLRTGRDIVMAAMMGAEEFGIGTAALIAMGCIMVRQCQSNTCPVGVCTQDEALRGKFTGNADKVVNLITFYATEVREILASIGARSLDDVVGRADLLSQVSRGSAHLDDLDLNPLLITVDGAADIVYDRDKPRNAVPDTLDAQIVNDASRFLNDGEKMQLQYAVQNTLRTVGTRTSSHIVRKFGMRNSLQPDHLTVKLNGSAGQSLGAFAAPGLKIEVSGDANDYVGKGLSGGTIVVRPPMASPLVASQNTIIGNTVLYGATDGCLFAAGRAGERFAVRNSGAQVVIEGCGTNGCEYMTGGTAVILGSVGANFGAGMTGGMAFLYDPEGKAETMINRETVVTCPVTV--DHWMTLLHGLVRRHHEETGSVKAADLLQHWDLERDNFLQVCPKEMLSRLPYPLSEEDT-AIPAE-MQPVTAPFPASRPRRLRRTGAIRDLVRETRLATGDLIWPLFVRDGEGIEEPVASLPGVSRLSIDRLVDAAATARDLGIPAICLFPYTDPALKTETCEEAWNPDNLSNRAIRAIKKAVPEIAVMTDIALDPYNANGHDGIV--RDGVIVNDETVEALVRMALAQAEAGADILGPSDMMDGRIGAMRAALEEAGHNDISILSYSAKFASGFYGPFRDAVGASGALKGDKSTYQIDPANADEAMRMIARDLAEGADMVMVKPGMPYLDICRRAKDRFGAPTFAYQVSGEYAMIEGAARQGWIDGDKVMMESLLAFKRAGCDGILTYFAPRAARMLAETRQMGIQAAEISAILKEQIKNFGQEAEVAEVGRVLSVGDGIARVYGLDKVQAGEMVEFPGGIQGMALNLENDNVGVVIFGSDSDIKEGDVVKRTNSIVDVPAGDELLGRVVDGLGNPLDGKGPI-NASERRVADVKAPGIIPRKSVHEPMATGLKSVDAMIPVGRGQRELIIGDRQTGKTAIALDTILNQKSYNEAAGDDESKKLYCIYVAVGQKRSTVAQLVKKLEETGAINYSIVVAATASDPAPMQFLAPYAATAMAEYFRDNGRHALIVYDDLSKQAVSYRQMSLLLRRPPGREAYPGDVFYLHSRLLERSAKLNEENGSGSLTALPIIETQGGDVSAFIPTNVISITDGQIFLETELFYQGIRPAVNTGLSVSRVGSSAQTNSMKSVAGPVKLELAQYREMAAFAQFGSDLDAATQKLLNRGARLTELMKQPQYSPLTNAEIVTIIYAGTHGYLDKLPVKDVGRFERDLLAFLRGKHKDLLDWITTEDPKIKGDAEDRIKAVLDEFAKDFA------MTQSTERDRVLIFDTTLRDGEQSPGATMSHAEKLEIASMLDEMGVDIIEAGFPIASEGDFKAVSEIARASRNAVICGLSRANIGDIDRCWEAVRHAARPRIHTFIGTSPLH-RAIPNLTQDEMVERIHQTVTHARNLCDNVQWSPMDATRTEEDYLCRVVETAIKAGATTINIPDTVGYTAPRESAELIRMLIEKVPGADEVVFATHCHNDLGMATANALAAVEGGARQIECTINGLGERAGNTALEEVVMALRVRGDIMPFTTGVDSTKLMAISRRVATVSGFPVQFNKAIVGKNAFAHESGIHQDGMLKNAETFEIMRPADVGLKETNIVMGKHSGRAALRSKLSELGFDLADNQLKDVFVRFKALADRKKEVYDDDLLALMRD-QADETQDRIQVKFLRVVCGTQSPQTAELVLTIDGEDHEVTATGDGPVDATFNAVKQLFSHEARLQLYQVHAVTEGTDAQATVSVRMEENGRIVTGQSADTDTVVASARAYVNALNRLLVRREKSAPDSDVKTVNYLD-AN--MSG-TYDDRDGVIWMDGKLIPWRDANVHILTHAMHYASSVFEGERAYNGKIFESRRHSERLRKSAEMIDFEIPWTVDEIEAAKMAVLEANGQTDAYVRAVAWRGVGEDMGVASARNPVRLAIASWAWGNYYGDAKMKGAKLDIAKWRRPDPATAPSQAKAAGLYMICTMSKHAAEAKGCSDAMMFDYRGYVAEATGANIFFVKDGEVHTPDPDCFLNGITRQTVIGMLKDRQIKVHERHIMPEELESFEQCWLTGTAAEVTPVGKIGDYNFEVGSLTRDISQSYEELVRK------MTAP----KTLYDKIWDAHVAH-EADDGTTLLYIDRHLVHEVTSPQAFEGLRMNGRKVRAPEKTIAVPDHNVPTTEGRDTYIE-NE-ESRIQVEALDRNAREFELVYYPVSDIRQGIVHIVGPEQGWTLPGMTVVCGDSHTATHGAFGALAHGIGTSEVEHVLATQTLIQKKSKNMKVEITGKLRPGVTAKDITLAVIGETGTAGGTGYVIEYCGEAIRDLSMEGRMTVCNMAIEGGARAGLIAPDEKTFDYVMGRPHAPKGAQWEAALAWWKTLYTDEGAHFDKVLTLKGEDIAPVVTWGTSPEDVLPITASVPSPSDFEGG-KSEAVKRSLDYMGLTPGTPLDQVEIDTVFIGSCTNGRIEDLRAAAAILKGKKVKDGLRAMVVPGSGLVRAQAEEEGLADIFKEAGFEWRLAGCSMCLAMNPDQLSPGERCAATSNRNFEGRQGRGGRTHLLSPAMAAAAAITGRLTDVRELM-----------------MM-----------ADNPDKPDDDAGVITKAKSKTQRPPLYKVMLLNDDYTPMEFVVHVLERFFGINHAQAVEIMLVVHKKGLAVVGIFSHEIAETKVAQVMDFARRHQHPLQCTMEKE-MPDQTPFRPFETVLDEARALETLRQATNGADDGELFLERRRSEVLSFDDGRLRTASYDASEGFGLRAVRGETAGYSHSTEISEAALTRAAETARLAVGDGGGTLAEGPRPTNVRLYGDFDPLSGAPFPVKVDLLREIDDFARGLDRRVVQVSATLAASLQEVEILRPEGTRVRDVRPMARLNVSVIVEQDGRREGGGTGGGGRIELSGLMEPDHWKGVTREALRIALVNLEAEPAPAGVMDVVLGAGWPGILLHEAVGHGLEGDFNRKKTSAFAGLMGQRVAAPGVTVLDDGTIPDRRGSISVDDEGTPSGKNVLIEDGILVGYMQDRQNARLMGVEPTGNGRRESHAHTPMPRMTNTYMLGGQADPAEIVADLRDGIYAVGFGGGQVDITNGKFVFSCTEAYRVRDGKIGAPVKGATLIGDGATAMQKIRAIGNDMALDPGIGNCGKAGQWVPVGVGQPTLMIGGLTVGGSAT---MVPADKLAQILERFQYIEARMSQGS--GDIAQLGREYAALRPVVEQVEGYNRLIADIAEAEAMLD--DPEMRALAEDELPALRTRLPEAEAAVRLALLPKDAADERAAIVEIRPGTGGEEAALFAGDLWRMYQRYAEGRGWSVSVIEESLTELGGLKELVANVRGQGVFARLKFESGVHRVQRVPSTESGGRIHTSAATVAVLPEAEEVDIDIPSTDIRIDTMRASGAGGQHVNTTDSAVRITHIPSGIVVTSSEKSQHRNREIAMQVLRARLYDAERQKVDSARAADRKAQVGSGDRSERIRTYNFPQGRMTDHRINLTLYKLDQVMQGD-LDEIIDSLIEEDQARRLAEMEQ------------------------------------------MIKLKAKYHLGQVVRHKRHPFRGVVFDVDPRFSNTEEWYDAIPEDSRPRRDQPFYHLLAENDQSYYVAYVSEQNLIADETGEPVEHPDLPDLFGDFENGRYPLHFQLN--MGWKRLEEM-DLRGKRVLTRVDINVPVSD-GRVSDATRIERIVPTVNAILEAGGTPILLAHFGRPKGRPDPALSLGVVLPALERALGRGVFFVEAPIGDGAREEIASIRAKDGADAPVILLENVRFYPGETANEDGFCDALAALGDVYCNDAFSAAHRAHASTEGLARRLPACAGRLMEAELSALEAALGKPARPVAAVVGGAKVSTKLDLLSNLVTRVDHLIIGGGMANTFLHAQGVAIGTSLAEKDLADTARRILDEAERAGCTIHLPVDLVVAREFRAEAPHEIV---ARDECPPDAMILDAGPESVRAIVELLGRCKTLVWNGPLGAFEIPPFDTATNGAAREAAVLTAAGQLVSVAGGGDTVAALNQAGAADGFSYVSTAGGAFLEWMEGKTLPGVAALGG-----------MSNVKK---------ILLVDDDDDLREVLSEQLVMTEDFEVIEGNSGQSAMERVKESLYDLVILDVGLPDTDGRELCRLLRKQGVKCPVIMLTGHDSDADTILGLDAGANDYVTKPFKFPVLLARIRAQLRQHEQSEDAVFQLGPYTFKPSMKMLIDENERKIRLTEKETNILKFLYRSSEGVVPRDVLLHEVWGYNAGVTTHTLETHIYRLRQKIEPDPSNASLLVTESGGYRLVAMAKSSS-SFVCANCGARHKKWAGNCDACGEWNTISEDVPLSSGPTRGTL---GGVRGRQIPLSDLATNETPPPRTRSRMEELDRVLGGGLVPASAVLVGGDPGIGKSTLLLQAAASFANSGMSCVYVSGEEATAQVRMRAQRLGLADAPVRLASETNLRDILTTLDAERPDLAIIDSIQTMWSDTVDSAPGSVSQVRAASHELTQFAKQRNTAVILVGHVTKEGQIAGPRVVEHMVDTVLYFEGERGHQFRILRAVKNRFGPADEIGVFEMTGEGLAEVANPSALFLSDRDRPAPGSVVFAGIEGTRPVLVEFQALVAPSSLSQPRRSVVGWDSGRLSMVLAVLEARCGISFAGLDVYLNVAGGMRISEPAADLAVAAALLSAREDAALPADCVVFGELSLSGALRPVGQTENRLKEAQKLGFTAAIAPDGSKISGDSGLSVRKIADLTGFVGEVFGA--G---MAKEELLEFPGVVKELLPNATFRVELENGHEIIAHTAGKMRKNRIRVLAGDKVQVEMTPYDLTKGRINYRFK---------------MGEAKKLFIKTYGCQMNVYDSERMAEALGGAGYVATDRADDADMILLNTCHIREKAAEKVYSELGRLKPLKAERPD-LKIGVTGCVAQAEGEEIMRRQPLVDLVVGPQSYHRLPQMEARV-AEGHKALDTDFPEEDKFETLQKR-PRAARGPSAFLTVQEGCDKFCAFCVVPYTRGAEVSRPAARILSEARDLVSRGVREVTLLGQNVNAYHGAAP--EGEGPWSLADLARGLAAIEGLERIRYTTSHPNDMADDLIQAHADCDKLMPYLHLPVQSGSDRILKAMNRKHTARQYLDLIARIRARRPDIMLSGDFIVGFPGETDEDFEQTLALIEAVNYGQAYSFKYSPRPGTPAAERAGHVDDAVATERLRRLQALLERQQKAVQQAMVGREVGVLFEKPGRMPGQIVGKSDYLHAVHVPGPQDLIGQVRRVRIVESTSNSLAGELI---------MSSRTSPTVAVVMGGSSAERDVSLSSGRECAKALKEAG-YRVEVIDAGPDLVEQLRAAKPDVVFNALHGRWGEDGCVQGVLEWLRIPYTHSGVLSSALAMDKIKAKEAYEAAGLPVAQSRIAGRDEIEAGHVLPPPYVVKPVAEGSSVGVFLIMEESDAPPR----LNAEMPERLMVETFVPGRELTTTVMGDRALAVTEILTD-GWYDYDAKYVAGGSRHEVPADLPPEITQACLDYALRAHECLGCRGVSRTDFRWDPERGLAGLFLLETNTQPGMTPTSLVPEQAAHLGISFPELVRWMVEDASCNRMAA-----KD-TEDRKQDDQDNEAM--LDMSQAAVKKMISEARARGYITYDQLNQVLPPEQVSSEQIEDVMSMLSEMGINIIE-DDEAEDGE---QKSTELTTTSDSKEITVATTSSETLDRTDDPVRMYLREMGSVELLSREGEIAIAKRIEAGRNTMIAGLCKSPLTFQAITIWRDELLSEDILLRDVIDLETTFGRSMGEDGEAGEENV----VAGLDVGAATTTTPKPEKKSAEPELDADGNPIRS--TDDDDDEDEQANMSLAAMEAALKPRVLETLDRIAADYARLSDMQDVRISATLNEDESFSAGEEEAYQSLRGEIVELVNELHLHNNRIEALIDQLYGINRRIMAIDSNMVKLADQARINRREFIDTYRDHELDPTWLDQIAAKPGRGWSAFLERSRDKIEELRGEMAQVGQYVGLDISEFRRIVQQVQKGEKEARQAKKEMVEANLRLVISIAKKYTNRGLQFLDLIQEGNIGLMKAVDKFEYRRGYKFSTYATWWIRQAITRSIADQARTIRIPVHMIETINKLVRTGRQMLHEIGREPTPEELAEKLQMPLEKVRKVMKIAKEPISLETPIGDEEDSQLGDFIEDKNAILPLDSAIQENLKETTTRVLASLTPREERVLRMRFGIGMNTDHTLEEVGQQFSVTRERIRQIEAKALRKLKHPSRSRKLRSFLDQ--MCAKASMEEFEALLNESFEMDTPDEGSVVKGKVIAIEAGQAIIDVGYKMEGRVDLKEFANPGEAPEISVGDEVEVYLDRVENARGEASISRDKARREEAWDKLEKAYADDARVEGAIFGRVKGGFTVDLGGAVAFLPGSQVDVRPVRDAGPLMGMKQPFQILKMDRRRGNIVVSRRAILEESRAEQRAEVIGKLTEGDAVDGVVKNITEYGAFVDLGGVDGLLHVTDMAWRRVNHPNEILAIGETVKVQVIKINKETHRISLGMKQLQDDPWNAVEGKYPLNSVHTGRVTNITDYGAFVELEPGVEGLVHVSEMSWTKKNVHPGKIVSTSQEVEVMVLEIDTAKRRVSLGLKQTMRNPWEVFAETHPVGTEVEGEVKNITEFGLFIGLENDIDGMVHLSDLTWEGRGEDVIGDYRKGDMVKAIVTEVDVEKERISLSVKALD-NSFSGAV-------EGVKRGSIITVEVTAIEDGGIEVEYE--GAKSFIRRSDLSRDRAEQRPERFGVGDKVDVRVTNVDPKSRRLGLSIKAREIAEEKEAVEQYGSSDAGASLGDILGAAL-------------------------KG--DDN------------------------------------------------------------MTELTHWIDGKHVKGTSGRFADVFNPATGEVQAKVPLASKAELDAAVESAARAQPAWAATNPQRRARVLMKFVDLLNRDMDKLAEALSREHGKTLPDAAGDVQRGLEVVEFCIGAPHLLKGEYTDSAGPGIDMYSMRQALGVVAGITPFNFPAMIPLWKMAPALACGNAFILKPSERDPSVPLMLAELMQEAGLPDGVLQVINGDKESVDAILDNETVQAVGFVGSTPIAQYIYSRGTANGKRVQCFGGAKNHMIIMPDADLEQAADALVGAGYGAAGERCMAISVAVPVGDETADRLIEKLVPRIEALKVGPYTAGKDVDYGPVVTAAAKQNIARLVQTGIDQGAELVVDGRDFNLQGYEDGFFVGPHLFDRVTKDMDIYTQEIFGPVLSTVRAKDYEEALSLAMDHEYGNGTAIYTRDGDTARDFANRINIGMVGINVPIPVPLAYHTFGGWKKSAFGDLNQHGPDAFKFYTRTKTVTARWPSGIRQGGEFSIPVME-----MDHSAAIDAALSRLHEEGRYRTFIDIERRRGQFPTATWTRPDGTETEITVWCGNDYLGMGQHPAVLSAMHDALDATGAGSGGTRNISGTTVHHNALEAEIADLHRKEAALLFTSAYIANDATLSTLPRIFPGLVILSDELNHASMIEGIRRAGGDKRVFRHNDVAHLRSLLEELPADVPKLIAFESVYSMDGDFGPIEAICDLAEEFGALTYIDEVHAVGMYGPRGGGITERDRLAHRIDIINGTLAKAYGVMGGYIAGKANLVDAIRSYAPGFIFTTSLPPAVAAGAAASVRHLKTDQA--LRDLQQERAAALKLRLKGLGLPIIDHGSHIVPVHVGDPVKCKMISDRLLADHGIYVQPINFPTVPRGTERLRFTPSPVHTPEMMDRLVAGMDDLWSQCALNRAELSA---MARVLITSAIPYINGIKHLGNLVGSQLPADLYARYMRQRGHEVLFLCATDEHGTPAELAAAKAGKPVAEFCAEMHAVQAKLGEGFRLSFDHFGRSSSPQNHALTQHFAGKLAEAGLIEEVSERQVYSHADGRFLPDRYIEGTCPNCGYDKARGDQCENCTKQLDPTDLIEPRSAISGSTDLEVRETKHLYLRQSQIRDRLRDWIDGQSD---------------------------WPILTTSIARKWLDDGDGLRDRGITRDLDWGVPVKKGT-QDWPGMEGKVFYVWFDAPIEYIASAKEWTDADGRPDADWERWWRTDKGAEDVRYVQFMGKDNVPFHTLSFPATIM---------GSGEPWKLVDYIKSFNYLNYDGGQFSTSQGRGVFQDQALEILPADYWRWWLLSHAPESSDSEFTWENFQAGVNKDLADVLGNFVSRVTKFCRSKFGEVVPEGGTTGPAEEALVAELTRRLAAYQDHMEAIEIRKAAAELRAIWVAGNEYLQSAAPWTLFKTDPEAAAAVVRLSLNLIRLYAVLSRPFIPDAAQELMRAMN-CDDW-------TWPEDIGAALTTLPAGHAFTVPDVTFRKITDDEREEWKARFSGTRD---MKPVEIYTSPLCGFCHAAKRLLKGKGVSFTEYDISRDPAKRKEMMQRANGRHTVPQIFIGDTHVGGSDELHAMERSGKLDPMLAA-------------------------MIDVDGRVLLTQRPEGKSMAGLWEFPGGKVEPGETPEAALIRELKEELDIDTWQSCLAPLTFASHSYENFHLLMPLFACRKWQGTPRGLEGQEMAWVRPNALRDYQMPPADVPLIPILRDWL--------MSRQNYIFTSESVSEGHPDKVCDRISDAVLDAFLDQEPEARVACETFATTNRVVIGGEVGLSDKSKLKEYLGRIEQIARDCVRDIGYEQDAFHWKTLTVDNLLHEQSAHIAQGVDSSND--KEEGAGDQGLMFGYAIDETEELMPAPIHYSHAILRRLAEVRKSGAEPALGPDAKSQLTVRYENGRPVGVTSLVLSTQHMDPDLSSADVRAIVEPYITEVLPEGWLSDETEWWVNPTGKFVIGGPDGDAGLTGRKIIVDTYGGAAPHGGGAFSGKDPTKVDRSAAYAARYLAKNVVSAGLARRCTIQLSYAIGVARPLSIYADTH-GTGEVDESRIEKAVNEVMDLTPRGIREHLGLNRPIYQRTAAYGHFGRAPEADGGFSWERRDLAEALKAAV-MGVS-TALNT---------------------------AGPDREVTTAALNRDLQDKGFLVTSTEDVINWARTGSLHWMTFGLACCAVEMMHTAMPRYDMERFGTAPRASPRQSDLMIVAGTLTNKMAPALRKVYDQMPEPRYVISMGSCANGGGYYHYSYSVVRGCDRIVPVDLYVPGCPPTAEALLYGILQLQRKIRRTGTIVRMALPDFTMRQLLEAGVHFGHQTQRWNPRMGEFIYGDKNGIHILDLTQTVPMLDQALQVVRDTVAKGGRILFVGTKRQAQKPVADAAERCAQYYMNHRWLGGTLTNWKTVSNSINRLKAIDEQMQNGVE-GLTKKERLGMEREQVKLEASLGGIREMGGVPDLLFVIDVNKEDLAIAEAKKLGIPVVAVVDTNASPDGVDYIIPGNDDAARAIALYCDLVSRAALDGMSAQMGAAGFDLGAMEEAPEEEAVAE-----GEGVEVGNASSETVADDAVAKDAPYDLENSEPGEKV------------------------------------MMDWTDRHCRYLHRVMSRRTLLYTEMVTAPAVVHGKREMLLGYDPAEHPVALQLGGSEPELLAEATRIAVGYGYDEINLNVGCPSDRVQSGAFGAVLMKDPGLVAECVAAMQGAVPGGSGPDGPEITVKCRIGVDEQEPEEVLPAFLEAMVGAGIGRVAIHARKAWLQGLSPKENRDVPPLDYDLVLRMKRAFPALHMSINGGIADLDQAEHFLAQGMDGVMIGRAAYHAPAQILLDADRRIFGEEGAI-TAEETVRRMLPYIEAHLADGGRLHSITRHMLGLFLGRPGARAWRRVLSEGAHREGAGVALVEEALARVED----AAQPACT----------MLLLIDNYDSFTYNLVHYLGELGAEVDVRRNDALSAEEALALAPSAIVLSPGPCDPARAGICLDLTRAAAREGVPLLGVCLGHQTIGEAFGGTVSRHHEIVHGKTAQVRHAGKSVFAGLPDPLESTRYHSLVVDRDTLPDALEVTAWLEDGTIMGLQHREHPIHGVQFHPESIASDAGHELLRNFLRMAKVAAMIRSELIQIIADENPHLYQRDVERIVNTLFEEIINAMASGDRVELRGFGAFSVKKRDARIGRNPRTGESVEVEEKYVPFFKTGKLLRDRLNGKAD------MFG-FGSLFSADMAIDLGTANTLVYVKGKGVILNEPSVVAYHVKDGRKQVLAVGEDAKLMLGRTPGSIEAIRPMREGVIADFDVAEEMIKHFIRKVHKRTTFTKPKIIVCVPHGATPVEKRAIRQSVLSAGARKAGLIAEPIAAAIGAGMPITDPTGNMVVDIGGGTTEVAVLSLGDIVYARSVRVGGDRMDEAIISYLRRQQNLLIGEATAERIKTSIGTSRMPDDGRGASMQIRGRDLLNGVPKEIDINQAQVAEALSEPVQQICEAVMTALEATPPDLAADIVDRGVMLTGGGALLGDLDLALREQTGLSISVADESLNCVALGTGKALEYEKQLRHVVDYESMGTDSLRSGQGEDDTGEGTTAPRGDEGTAGADPAAKGAADTPVEALDEKTARAELARLAARIAEADRAYHTDDAPVIDDATYDALRRRNEAIEARFPALKRPDSPSEKVGGTIAEGFSKVEHEQRMLSLSNAFEDEEITEFVERVRKYLGLARDAELAFTAEPKIDGLSLSLRYEKGVLVTAATRGDGSVGENVTANARTIQGLPQKLSGA--PDLLEVRGEVYMRHDDFAELNRTQLAQGKKPFANPRNAAAGSLRQLDAEITRARPLAFFAYGWGSLSEPLADTQMGAIERLAALGFETNPLTRRLSTPEDLLAHYHRIEEDRATLGYDIDGVVYKVDDLELQRRLGFRSTTPRWALAHKFPAELAWTRLEGIDIQVGRTGALSPVARLRPVTVGGVVVANATLHNEDYIAGRDSRGEEIREGKDIRIGDRVQVYRAGDVIPKI-ADVDLAARPDGAEPYVFPDTCPECGSEAIREEGDAVR-----RCTGGLICPAQAVERLRHFVSRAAFDIEGLGARQVEALYRDE--WIAEPADIFRLRARY-GSGPRQLKNREGWGEKSAANLLNAIDERRRIPLNRLIFALGIRHVGENAANLLARHYGTWEAFAQAMDAAAEGT------------------------------------------DAPAWQALNDIDGVGEVMATSLVTTLQQDRERASIERLVAELEVEAVAAPDPGDSVVAGKTVVFTGKLERMTRAEAKSRAEALGAKVAGSVSARTDYLVAGPGAGSKATKAAELGVEVLDEDAWLTLTGGLMPSLNEIRSTFLNYFERQGHQVVPSSPLVPRNDPTLMFANSGMVQFKNLFTGVEKRDYTRATTAQKCVRAGGKHNDLDNVGYTARHHTFFEMLGNFSFGDYFKNEAIPFAWEMITKELDIPKDRLVVTVYHDDDEAAEIWKKVAGISDNRIIRIATD-----DNFWMMGPTGPCGPSSEIFFDHGDHIWGGPPGSPEEDGDRFVEIWNLVFMQYEQFEDGTREPLPNQSIDTGMGIERVAALLQGTNDNYATDLMRSLIEASAHATSTDPDGPGKTHHRVIADHLRSTSFLIADGVMPSNDGRGYVLRRIMRRAMRHAHLLGSQDPVMHRLVPALVQQMGAAYPELGQAQALIQETLRAEETRFKQTLERGLRLLDDELSDLPEDAPLPGEAAFKLYDTFGFPLDLTQDALREKGRSVDTDGFDAAMAEQKAKARAAWSGSGETADASLWFDLAEKHGTTEFLGYDTEVAEGQLLAIVRD-GAAVKT-AEAGQEVQFVLNQTPFYAESGGQVGDQGEIRT---ETG---------AARITDTRKT-AG--VFIHMGQVTEGTIETGQGAELEVDHSRRSAIRANHSATHLLHEALRRTLGDHVAQRGSLNAHDRLRFDFSHGKALSREELDQVETEVNEFIRRNEKVETRIMTPDDARAIGAQALFGEKYGDEVRVVSMGSLSGSGKGASGDTYSLELCGGTHVRQTGDIGMFVLLGDSASSSGVRRIEALSGADAFRYLADQGKHLSDAALALKARPDEVPERVRALLDERKALTNELAQLRRELAMGGGGAGGAAQPE--AQEVNGIRFAAHVVNGVTGKDLPALVDEHKARLDSGAVLLIADTGGKAAVAAGVTKDLTDRLSAVDLVRAAVAELGGKGGGGRPDMAQGGGRDTANSDAAIAAARSHLETI--MSAKAEHYDVIRKPIITEKATMASDAGAVVFEVAMDSNKPQIKAAVEGLFGVKVKAVNTTITKGKVKKFRGQPGRRKDVKKAYVTLEEGNTIDVTTGLMPSLKDLKNRIESVKSTRKITKAMQMVAAAKLRRAQEAAEAGRPYAERFNAVLGGLAASVGTGDNAPRLLAGTGSDQTHLLVVMTSERGLAGGFNSNIVKLAKQHIERLRGEGKTVKVLTVGKKGREQLRREYGDTLIGHVDLSEVKRIGYDTAQ-GIARDLLSRFDTGEFDVATLFFARFGSVISQVPTAQQIIPAVYETAAEEDAGA--DTTLYDYEPSEEGILADLLPRGVATQIFSALLENAASEQGARMSAMDNATRNAGEMIDKLTIQYNRSRQAVITNELIEIISGAEAL--------MNDLLT-GS--APEQTYDASSIEVLEGLEPVRKRPGMYIGGTDERAMHHLVAEVLDNSMDEAVAGHASRIEVELISDNSVTIRDNGRGIPVDPHPKFPDKSALEVILCTLHAGGKFSGKAYQTSGGLHGVGISVVNALSDQLRVEVARNRELFAQEFSRGHPQGPVEMVGAAPNRRGTSVTFRADSEIFGS-HKFKPARLMKMVRSKAYLFSGVEIRWKSA----IDDGETPTEATFHFPGGLADYLKESLGNAATYADAPFAGKVSFQEKFGKPGSVEWAINWTPS----RDGFIGSYCNTVPTSEGGTHEAGFWAAILKGIRAYGELVGNRKAAQINRDDLIAGGCALVSCFITEPEFVGQTKDRLATVEAGKLIEGAVRDHFDNWLASDTKSAGAILDYLVLRAEERLRRRSEKETARKTATKKLRLPGKLVDCSATNREGTELFIVEGDSAGGSAKMARDRKNQALLPLRGKILNVLGAASAKMGQNAEINDLCQALGVGMGTRFNLEDLRYDKIIIMTDADVDGAHIASLLMTFFFTQMRPMIDAGHLYLACPPLFRLTQGARRVYCLDEAEKAAMLERGLGGKGKIDVSRFKGLGEMDAKDLKDTTMNPETRKLIRVTISEDMPGET--ADLVERLMGKKPELRFQYIQQNAQF--VEELDV-----MSAPKKVVLAYSGGLDTSIILKWLRTEYDCEVVTFTADLGQGEELEPARKKAEMMGVS--EIYIEDLREEFTRDFVFPMFRANALYEGLYLLGTSIARPLISKRLIEIAEATGADAVAHGATGKGNDQVRFELSAYALNPDIKVIAPWREWDLTSRTKLLDFAERNQIPIAKDKRGEAPFSVDANLLHTSSEGKVLEDPADEAPDYVYQRTVHPEDAPDTPEYIEVGFEKGDAVSINGEAMSPATILTRLNELGGKHGIGRLDLVEGRFVGMKSRGIYETPGGTILLEAHRGIEQITLDRGAAHLKDQIMPQYAELIYNGFWFSPEREMLQALIDKSQEHVTGTVRLKLYKGLARTVGRWSDHSLYSEEHVTFEDDAGAYDQKDAAGFIKLNALRLRLIAMRNRRLKG-----------------------MCSDKTGLTYADAGVDIDAGNALVERIKPAAAATRRSGVMAGLGGFGALFDLKEAGYSDPVLVAATDGVGTKLRIAIDTGHYDTIGIDLVAMCVNDLVCQGAEPLFFLDYFATGKLELDAAAAVIEGIAEGCKRSGAALIGGETAEMPGMYGAGDFDLAGFAVGAMERGCALPD--GVKEGDVLLGLASDGVHSNGYSLVRRIVEKSGLSWSDDAPFA-DGPLGAALLTPTRLYVTQVLAAI-RAGGVHALAHITGGGLTENIPRVLPEGMGVRIDLDAIELPPVFAWLAREGGLDQAEMLKTFNCGIGMVLAVERDRAEALRTLLIGRGETVCTLGEVVAGEG-VAYTGSLGMYVYSEFDRDFVRDRNAQFREQVARRLSGALTEDEFKPLRLMNGVYLQLHAYMLRVAIPYGTLNSAQMRKLAYLAQRWDKGYGHFTTRQNIQYNWPALTDIPDMLDALAEVGMHAIQTSGNTIRNVTADHFAGAAADEIADPRPVAELLRQWSTDHPEFQYLPRKFKIAVTGSPLDRAVIRAHDIGLRLVRDAD-GAPGYEVIVGGGLGRTPMVGQVLTPFLPQADLLPYVEAIVSVYNLLGRRDNKYKARIKITVHENGIDEIRRRVEARFARLRKEFR-GVDQQMLASIEAQFAPPAFEVGRS-ALPYEDAREIDPVFQAWSDTNIHPHKRDDHAIVTVSLKRPGETPGDASAEQMRVLADLAQEFGHDELRISHEQNVILPHVHRADLPTLHARLRRAGLATANAGLISDIIACPGMDYCALATARSIPVAQEIASRFEALKLEHEVGPMKIKISGCINACGHHHVGHIGILGLDRAGVENYQITLGGDAGPDMVLGERAGAGFPADRIVDAVEALVRGYLDLRAGPAETFLDTYRRLGAEPFRAILYP-------TE-----KVHAA--MAKTIVVKQIGSPIRRPAKQRATLIGLGLNKMHKTRELEDTPSVRGMVNSISHMVEIIEERD-------MADPQ-----ELLEDIEALGAKLAAARQSISARVIGQPQVVDLALTTLLCGGHGLLIGLPGLAKTRLVETLAVVMGLDGNRIQFTPDLMPADILGSEILDVAADGSRSFRFLEGPVFCQLLMADEINRASPRTQAALLQAMQEKHVTIAGQERPLGRPFHVLATQNPIEQEGTYPLPEAQLDRFLLQIDVDYPDRETERAILLATTGTEEATATPVFDAESLIAAQRTVRQMPVGDAIVDSILDLVRACRPGEADAPELVRETVSWGPGPRAAQALMLAVRARALLDGRLAPSVEDVVALARPVLEHRMALSFAARARGQDLAQVIASVSDRIGRAEDAA-MPKRILNGTVTSDQNEQTVTVLVERRYTHPLLNKTVRASKKYRAHDPKNEFKVGDKVRIQECAPISKTKRWEVVAN------------------------------MHDSDPNQA---DPVALAADLLRDH-RGSIDRLDAILVYTLGERFKHTQAVGRLKAKHDLPPSDPDREARQIARLEELALAADLDPAFARKFLNFIIQEVIRHHEEHQK----MNELSPKITAEL------PVIRQLDDAAVNRIAAGEVVERPASAVKELVENALDAGARRIEVDYADGGKTLIRVTDDGHGIPAEALPLALQRHATSKI----DGS-DLLNIHTFGFRGEALPSLAAVGRLSLTSRASGHDEAAEILVSGGSAPVLRPAALGR----GTVVALRDLFHATPARLKFLRSDRAEAQAIGDVVKRLAMAEPSVGFTLRDVSGGGEGRVLLRLDPCNGDLFDALLERLRAILGREFAENALPIEAEREDLSLTGFAALPTFSRGSAVGQFLFVNTRPVRDKLLVGALRAAYSDFLSRDRHPVAVLNIGCHPELVDVNVHPAKSEVRFRDPALARGLVVSGLKHALAGAGHRASTTVAAATLGAL--RPGDGPMAGGWNPAAARDQAAEGSALEGAP---RVYQMD-----RPRGYGTSGYAGQPSRGVLEAA--HAAQA---P---------GFAEMGVALS----GRLA-TDEPGHDVARAE------------ADEAARDLPLGAARGQVHENYIIAQTATGIVIVDQHAAHERLVYEKLKAQRADARVPSQALLIPEIVELDEADAARLLDEAEALAGLGLVIEPFGGGAVAVRETPAILGQADPARLVRDVVDELADQGSSASVQEKIDAILSRMACHGSIRSGRRMRGEEMNALLREMEATPHSGQCNHGRPTYVELRLADIERLFGRT-MSPLKIKKGTSKHSAYDLRSNYADTQETHTLAVIVDNEAGVLARVIGLFSGRGYNIDSLTVAEIDHQGHRSRITVVTTGTPQVIEQIKSQLSRMVPVYEVHDLTVEGPSVERELALLKVVGEGEKRVEALRLADIFRANVVDSTLESFVFEITGPPEKIDAFAELMRPLGLEEVARTGVAALSRGGSGSLGVASDKVTADVTGGANAMPSYTAPVRDMQFLLHDVLKVTE-TDIPGYDDLEADFTAPVLEEAGKLASDVLAPLNPVGDHEGC-TLENGVVRTPKGFSEAFDKLREGGWTALDCDPEYGGQGMPYLLGTAVGEIFVSANMAFNMYQGLTHGAYSAIHAHGTDAQKQTYLPKMVTCEWTGTMNLTEPHCGTDLGLMRSKAEPQDDGSYRITGQKIFISAGEHDMADNIIHLVLARITGAPAGIKGISLFIVPKFMVNEDGSLGDRNAVTCGNLEEKMGIHGNATCVMNYDGATGYLLGTADKGMRAMFTMMNEARLGVGLQGYAQAEAAYQNAVAYAKDRLQGRDVTGAKNPDGPADPIIVHPDIRRSLMDQKSFVEGARALTFWGAHLIDRAHRAGDAEAD----GLISLLTPVIKGFQTDKGFDMTVQAQQVFGGHGYIEEWGMSQFARDARIAMIYEGANGVQALDLVGRKLALDGGKHVMAFFDLVKTFIAENKD-EERLAKDFLDPLKAASKDLQAAATYFMQTAAKNPNHALAGSTDFLHLFGHVCLGLMWARMGKAALEGLDAGASETDFLNAKLATGRYYMARQLPATGMHLARIQSGADTVMALEADAF----------MEFDPK------------SLRYDDRGLIPAIAQDADSGEVLMMAWMNAESLARTLDTGRVTYWSRSRQSFWIKGESSGHVQELVDLRVDCDRDCLLLRVRQTGPACHTNRRSCFYTAVREGAEVELMAPEA------MAKPTTIKIRLNSSAGTGHFYVTKKNARTMTEKMVVKKYDPVVRKHVEYKEGKIK--------MRRSFLGNARNLAFWVVLFLLVLALFNLFSGGSST--MSSRSMSYSEFIEKVDSGAVRSVVLDGERILIRDAAGQQYNT-VKPEGV--DVTPKLAARNVDIEVKAQEQSG-FFSVLSLWLPFLVLIGIWIFFMNRMQGGGRGGAMGFGKSKAKLLTEKHGRVTFDDVAGIDEAKDELEEIVEFLRTPQKFSRLGGKIPKGALLVGPPGTGKTLLARAIAGEAGVPFFTISGSDFVEMFVGVGASRVRDMFEQAKKNAPCIVFIDEIDAVGRSRGVGYGGGNDEREQTLNQLLVEMDGFEANEGIIIVAATNRPDVLDPALLRPGRFDRQVQVPNPDIKGREKILGVHARKVPLGPDVDLRIIARGTPGFSGADLANLVNEAALMAARVGRRFVTMDDFENAKDKVMMGSERRSMVMTEDEKKLTAYHEAGHAIVGLNVPQHDPIHKATIIPRGRALGLVLSLPERDQLSVTYTKYKSKIAMAMGGKVAEELIFGKENVTSGASSDIQQVSKIARAMVTQFGFSEELGNIDYANEQQSYL--GNYQGQTAISPETQQKIDAEVRRIVDEGYERAKQILTDKNDDLHRLAQGLLEYETLTGSEITKVIAGQALNRGEDDDDA--TGGNTPSITAIPKT----KPRPKGDS-G-LEPEPSSMAKITYIEHNGTEHVVEVANGMTVMEGARDNSIPGIEADCGGACACSTCHVYVHPDWVDRLPGKDSMEEDMLDFAYEPDTQRSRLTCQLKVTDALDGLVVQLPEKQIMSRRCELTGKGPMVGNNVSHAKNRTRRRFLPNLNDTTLVSESLGRSFSLRISASALRTVDHRGGLDAFLAKAKDADLSDKALKIKKDIAKAGATA-----------MIKRAVVRGVGHYLPDRVVPNSHFETFLDTSDAWIRGRSGIERRHFAAEGQTTSDLGARAARAALEMAGMQPDDIDAVIVATSTPDLTFPSAATMIQGQLGMTRGFAFDVQAVCAGFVYALSNANALILSGQAERVLVIGAETFSRIMDWTDRGTCVLFGDGAGALVLEAEDGAGETADRGILSTDLHSDGRYRDLLYVDGGVSSSQSTGVLRMEGKEVFRHAVEKLAQTAHAALDKAGVSAEQVDWIVPHQANLRIIKATAQKMKLPMERVVVTVQDHGNTSAASIPLALSVGVERGQIKKGDLLVAEAIGGGLAWGSVVLRW---------MKTLHLSADGQGLDRAAELLRGGALVAFPTETVYGLGADAADDRAVARIFEAKGRPSFNPLIVHVSSVDQARRYAEFSPEAERLAAAFWPGALTLVLPLRPEAPLSRLVTAGLETVAIRMPAHPLAHELLERVGRPLAAPSANASGRISPTSAAHVLGTGDGGLGGRIEAVLDGGPCDVGVESTILRPGTAGAP-ARLLRPGGIPAEAIEAALGAPLEPEAPVAENAPSAPGQLASHYAPEARVRLNASEVGPDEVLLGFGA-DKDRTA-ALNLSPSGDLHEAAANLFDMLRRVDAQAR-----QAGLSRIAVAPVPEHGLGKAINDRLRRAAAPRDAMSRVKGGTTTHARHKKVIKAAKGYYGRRKNAFRTATQAVDKANQYATRDRKARKRNFRALWIQRINAAVRLHDDSLTYSRFINGLGLAGIEVDRKVLADLAVHEPEAFGAIVDQAKAALN-----MTDRIAFQGELGAYSHQACRDARPGHEVLPCRTFEDVIDAVRQGEADLAMLPVENSTYGRVADIHHLLPESGLHIVDEAFVRVHINLLAVPGTPLSGVRRAMSHPVLLGQCRDFLAQHGIERVTGADTAGSAQAVARDGRPEVAALASELAGEIYGLEVLARHIEDHDKNTTRFLIMAREADTTRRGDGPMMTSFVFKVRNIPAALYKAMGGFATNGVNMTKLESYMVGGSFSATQFYADIEGHPEDANVKLALDELAYFCSEQTILGVYPSDPNRR-------MSQNPPELRPDLAPRARIDIAESAPRPH-QPRIGMVSLGCPKALVDSERILTRLRAEGYAISPDYAGAEAVIVNTCGFLDSAKAESLEAIGEALTENGRVIVTGCLGAEPEYITGRHPNVLAVTGPHQYEQVLDAVHRAVPPAPDPFVDLLPARGVNLTPRHYSYLKISEGCNHKCRFCIIPDMRGRLVSRPVNAVLREAERLVDAGVKELLVISQDTSAYGLDRRHDRHEWQGRDVRSH-ITDLAREMGSLGA----WVRLHYVYPYPHVRDLIPLMAEGL--VLPYLDIPFQHAHPDTLKRMARPAAGSKTLDEIAAWRASCPDIALRSTFIVGYPGETEAEFQTLLDWLDEAQLDRVGCFQYENVAGARSNDLPDHVPAEIKQERWDRFMEKAQAISEAKLAAKVGRRIEVIVDEVDADA-ATC-RTKADAPEIDGNLFIDEGFQDLSPGDIVTVEVDEAGEYDLWGRLVS--------MSIDIETARRVAKLARIKVEDDHLPDLAQEFNNILGFIEQLNEVDVEGVEPMTSVTPMRLKRRTDEVTDGGMQDRILANAPDAREGFFAVPKVVE---MRTAKITRKTAETDITVEIDLDGSGRYDNQTGVGFFDHMLDQLARHSLIDMTIRATGDLHIDDHHTVEDTGIALGQALGKALGDKRGIRRYGACLLPMDDALVRCALDLSGRPFLVWNVALPTAQIG-RFDTELVREFFQALATHGGITLHVDALHGINSHHIAEAAFKSVARALREAVETDPRKADEIPSTKGALMSRRHAAEKREVLPDAKYSDRVLTKFMNNLMIDGKKSVAESIVYNAMERVEERLKRAPIEVFHEALDNVKPSVEVRSRRVGGATYQVPVEVRPERREALAIRWLIKAARARNENTMEERLAGELSDAVNSRGTAVKKREDTHKMADANKAFSHYRWMAAYQYVYHMDGVSKTYPGGKKTFENIRLSFLPGVKIGVVGVNGSGKSTLLRIMAGIDKDFSGEAWSAEGAKVGYLPQEPELDPALDVRGNVMAGVKAKRDILDRYNELAM----NYSDETADEMAKLQDEIDSQNLWDLDSQIDVAMEALRCPPDDADVSTLSGGEKRRVALCKLLLEAPDMLLLDEPTNHLDAETIAWLQQHLMDYKGTILVVTHDRYFLDDITSWILELDRGRGVPWEGNYSSWLEQKAKRLEQEAREDKSKQQTLARELEWMRQGAKARQAKSKARINAYNELANQSEREKLGRAQIIIPNGPRLGGKVIEVENLQKAMGDKLLVEDLSFSLPPGGIVGVIGPNGAGKTTLFRMLTGQEEPDGGSVTYGDTVKLAYVDQSRDALDPGKTAWEEISGGAELIELGDAQMNSRAYASAFNFKGGDQQKKVGMLSGGERNRVHMAKLLRSGGNVLLLDEPTNDLDVETLRALEDALVEFAGCAVVISHDRFFLDRICTHILAFEGEAHVEWFEGNFEDYEEDKKKRLGADSLEPKRVKHKKFTR--MILYPAIDLKDGQCVRLFKGEMDEATVFSDNPAAQARAFVDAGCEWLHLVDLNGAFEGRPVNAEAVEAILAAVD-VPAQLGGGIRDMATIDAWLEKGIRRVILGTVAVENPELVREAARKHPGRIAVGIDARHGKVATRGWAEETEIDATDLARQFEDAGVAAIIYTDINRDGAMQGPNVAETAALANAVSIPVIASGGVSSLADLKALRDSGAPLDGAISGRALYDGALDLAEALRVLA---------MSRLNTAEVEPKWQSAWEEAGVFTATRDES---KPKYYVLEMFPYPSGRIHMGHVRNYTMGDVIARYKISTGHNVLHPMGWDAFGMPAENAAIERGGHPATWTYDNIADMRAQMKPLGLSIDWSREFATCDPEYYGQQQAMFIDMLDKGLVYRKNAVVNWDPVDMTVLANEQVIDGRGWRSDAPVERRELTQWFFRISDFADELLSALDGLDNWPEKVRTMQANWIGRSRGLEFAFQLTA-------PTNGF-AELPVYTTRPDTLLGASFVGISPDHPLAKSLEEGNPELAAFSADCRRMGTSEADMEKAEKKGFDTGLRVRHPLNPAWELPVWVANFILMDYGTGAVFACPAHDQRDLDFCRKYDLPVTDTFFAL--D----NPRPVEDEAFV-PPKTEPVRWVDHFAGL-DVATGQEAIDATIDLAEKQGWGKGVTQFRLRDWGLSRQRYWGCPIPVVHCDACGVVPEKKENLPIELPRD------VSFDKPGNPLDRHPTWRDCACPACGQPAKRETDTMDTFVDSSWYYARFTSPRAATPTDAEDAAYWMNVDQYIGGIEHAILHLLYSRFFARAMNQTGHLPEKAIEPFNALFTQGMVTHEIYVT--QDD-------KGRP---------------------VYHLPESI------------------EWTESG--ARLGESGAP------------------VQVIPSAKMSKSKKNVVDPVNIIEAYGADTARWFILSDSPPERDVEWTAAGAEAAFRHLNRVWRIATDAAEASDAAGDAA------------------EDQALDRATHRAIADVTEGVENFAFNAAVAKLYGFTNTLAKSK---ASGQARRRAVATLAQLMSPMTPHLSEEIWALLGNVGLVVRAPWPKADPALLVEDSVTLPIQINGKRKSEITVAADLPREEVEKIALADKAVQKALAGGQPRKLIVVPGRIVNVVI--MSSYDVIVIGSGPGGYVCAIRCAQLGLKTACVEGRETLGGTCLNVGCIPSKALLHASHQLHEAEHNFAKMGLKGKTPSVDWKQMLAYKQETIDTNTKGIEFLFKKNKVDWIKGWASIPGAGKVQVGD-----EVHEAKHIVIASGSQVSTIPGVTVDEKTVVTSTGALELPKVPKTMAVIGAGVIGLELGSVYARLGAEVTVLEYLDGITPGMDLELARAFQKTLTKQGLKFVLGAAVQKVAPGKSNATVTWRAKGSDKDETLKTDVVLVATGRKPFTDGLGLEALGVEMTERGQIRTGAEWQTNVKGIHAIGDAIEGPMLAHKAEDEGMAVAEVLAGKHGHVNYGVIPAVVYTHPELSSVGETEEQLKAAGRDYKVGKFSFMGNGRAKANFAGDGFVKILADKESDRILGAHIIGPMAGDLIHEICVAMEFGASAQDLAMTCHAHPTYSEAVREAALACGDGAIHA--MRVGLCQLNASDAPPENLERTLEMLRQGVAQGARFLLTPEVTNCVSADRDHQRRVLSTEEDDATLTAIRAFALENSVWILIGSLALKTED-ADGRFANRSFLIGPDGAIRARYDKIHMFDVAISDRESYRESAGFRPGAQAVLATTPFGNLGLSICYDLRFPSLYRGLAQAGAQILTVPSAFSPVTGEAHWEVLLRARAIENGAFVLAPAQTGTHRASEGRARRTHGHSMVVAPWGEVLLDAG-TEPGVHVLDIDLAAVTEARQRVPSLEHDRDF-GPVEE--MT---------ERKKAWRKLAEDELR---GRSPEDLTWKTLEGIDVAPVYFPEDIEGLDHLGGVPGAAPFTRGVKGTMYAGRPWTIRQYAGFSTAEESNRFYRQALDAGQQGVSVAFDLATHRGYDSDHPRVVGDVGKAGVAIDSVEDMKILFDGIPLDKVSVSMTMNGAVIPVLAAFIVTGEEQGVDRAQLSGTIQNDILKEFMVRNTYVYPPEPSMRIVADIIEYTANEMPRFNSISISGYHMQEAGANLVQELAFTLADGREYVRTAIARGMDVDRFAGRLSFFFAIGMNFFMEAAKLRAARLLWHRIMAE-FEPKNPKSSMLRTHCQTSGVSLQEQDPYNNVVRTAFEAMSAVLGGTQSLHTNSFDEAIALPTEFSSRIARNTQLILQEETGVTNVVDPLAGSYYVEKLTADLAEQAWALIEEVEELGGMTRAVESGMPKLRIEESAARRQAAIDRGDEVIVGVNKYRRSKEDPIDILDIDNDKVREAQVARLERIRASRDEAACTAALDELTR-RTSEGGNLLEAAVEAARARATVGEISMAMEKTFGRHRAEVKTLAGVYGAAYEGDEGFAAIQQSVEEFARDEGRRPRMLVVKMGQDGHDRGAKVIATAFADIGFDVDVGPLFQTPEEAAQDAIDNDVHVIGISSQAAGHKTLAPKLVQALRDQGAGEILVICGGVIPQQDYQYLYDHGVQAIFGPGTNIPEAAQKILKLV----REARAE--MRVYYDRDCDINLIKDKKVAILGYGSQGHAHALNLRDSGAKNLVVALREGSPSAKKAEGEGLKVMGIAEAAAWCDLMMFTMPDELQAETYKKYVHDNLREGSAIAFAHGLNVHFGLIEPKPGVDVIMMAPKGPGHTVRSEYTKGGGVPCLVAVDQDASGKALETALSYCSAIGGGRSGIIETNFKEECETDLFGEQAVLCGGLVELIRMGFETLVEAGYAPEMAYFECLHEVKLIVDLIYEGGIANMNYSISNTAEYGEYVSGPRVLPYDETKARMKGILRDIQTGAFVRDFMQENQAGQPFFKGTRRMNDAHQIEEVGEKLRGMMPWISAGKMVDKAKN---MFDVNKIRGDFPILSRTIHGKPLVYLDSGASAQKPQVVIDAILRGYGEEYANVHRGLHHLSNLSTERYEAVRGTIARFLNAADPESIILNTGTTEGINMVAYGWAMPRMEAGDEIVLSVMEHHANIVPWHFLRERQGVNLRWVDIDQDGGLDPQAVIDAITPRTKLVAITQLSNVLGTRVDVAAITRAAHEKGVPVLVDGSQGAVHAPVDLQAIGCDFYVITGHKLYGPSGSGAIYVAPERMAEMRPFMGGGDMIREVRKDHVTYNDPPMKFEAGTPSIVSAIGLGAALEYLMDLGMENVAAHEGDLRDYAVQRLSGLNWLNLQGTTADKAAIFSFTLNGGAHPHDISTVLDKKGVAVRAGHHCAQPLMEHLGVSATCRASFGLYNTREEVDVLVEALELCHELFAMGAATDTGAIDALIDLMARLPGLGPRSARRAVLHLVKHRGRQLAPLAEAMRTVADTARECLNCGNIGTADLCPICQNNARANGQICVVGDVADLWAMERSGVFKGRYHVLGGTLSALDDIGPEELRIPKLLDRVVSEHVSEVVLALNATVDGQTTAFYIAEQLGE-GVEVTSLAQGVPIGGELDYLDDGTITAALKARRKFMRAEAQNHAEAVRKSLALLRQRMDWETAPHRLEEFNAMTEDPDLWNDPDKAQKLMRDRQSLSDAIDTHNAIQQELEDSIELIEMGEAEGDTDVVKEAEEALKALVGRAREKELEALLNGEADGNDTFLEINSGAGGTESCDWASMLARMYVRWAERKGYKVELQSESAGEEAGIKSASYKISGPNAYGWLKSESGVHRLVRISPYDSAARRHTSFSSVWVYPVVDDNIEIEVNPADIRIDTYRSSGAGGQHVNTTDSAVRITHAPTGIVVTS-SEKSQHQNREIAMKALKSRLYQMELDKRTAAINEAHENKGDAGWGNQIRSYVLQPYQMVKDLRTNVETSDTQGVLDGDLDRFMAATLALDVAGKSRAEARGEE---MND-VMLRLEGISKSYNPGTPAEISVLRGATLEVARGEAVALVAPSGAGKSTLLHIAGLLDTADAGMVEIGGVATGDLGDRSRTRMRRNDVGFIYQFHHLLPEFTARENIMLPQLANGVARGAARERAEELLASVGLTERGGHRPAEMSGGEQQRVAFCRAMANAPRVLLADEPTGNLDPDTSDRVFGALMGLVRSTGLSAVIATHNLELAARMDRVVRLEHGRLVERVERVDAGRAAD--MNDPRQISAQTEKALDSND----IKIRARDVQVYYGDNHAIKDVDVDILDKTVTAFIGPSGCGKSTFLRCINRMNDTIDIARIEGEILLDGQDIYDPSIDPVQLRAKVGMVFQKPNPFPKSIYDNIAYGPRIHGLARNRAELDEIVEKSLRRGAIWDEVKDRLDAPGTSLSGGQQQRLCIARAVATEPEVLLMDEPCSALDPIATAQVEELIDELRARYSVVIVTHSMQQAARVSQRTAFFHLGNLVEYGLTSDIFTNPRDSRTESYITGRIG-MITVAALYHFARFSDPDALRAPIRALCEGHGLRGTLLLAPEGVNGTVAGS-RGGIAALIAHLRALPGCAGLEWKESHAEEMPFLRLRVRLKREIVTMGQPDVDPTAAVGRYVAPADWNELIGQEDVVVIDTRNDYEVGIGTFEGAVDPGTRSFGEFPAWWRANRERF----HNKRVAMFCTGGIRCEKSTNFLLGEGVDEVFHLKGGILKYLEEVPAEDSRWQGECFVFDQRVSVGHGLEQGSHVLCHACRRPLAMPDLGRPEYEAGVQCHQCVDQFSEADRERFRERQRQIALARKRGERHLGSPD--------MNILILGGGGREHSLAWAIQQNPKCDRLIVAPGNAGIAEIAECARLDIEDGGAVCTFAEENAVDFVVIGPEAPLVSGVADALRAAGILCFGPDREAARLEGSKSFMKEVCTAAGAPTADYARFTEAEPARAHIREQGAPIVIKADGLAAGKGVIVAMTEDEALAAVDEIFGGAFGSAGAVVVIEEFMEGEEASFFVLCDGTDVLPIGTAQDHKRVHEGDEGPNTGGMGAYSPAPVLSPEVEQRALDEIIRPTVAEMARRGTPYSGVLYAGLMIKDGAPRLVEYNARFGDPECQVLMMRLGAQVLDLLLACAREKLSDARVNWA-----ED--HAITVVMAARGYPGSYAKGEVIGGLEDLPEDSMRMVFHAGTVR-EDAANGRGALLSNGGRVLNVTARGASLKEAQARAYETVAGIDWPGGFYRRDIGWRALTD----MFNPTQQVQNFIIPTVEETTSRGSLRYDIFSRLLKERIVFVSGPVHDEMATVIVAQLLFLEADNPSKEISMYINSPGGVVTSGLSIYDTMQYIKPKVSTVCIGQAASMGSLLLAAGEPGMRYSLPNSRIMVHQPSGGYQGQATDIMIHAQETQKLKDRLNQIYVKHTGRKLEEVEKALERDNFMEAQAAKDWGLIDEIVESRPRGDDPKS------------MKARVHVMLKAGVLDPQGEAVRHALGALGFDQVGGVRQGKVIDLEVD-ASSEEAARASVTEMCEKLLANTVIEKYDIEISAAMPELPEVETVRRGLEPVMEGRRILRADVRRPDLRRPFPERMAERLTGAEVLRLRRRSKYLLLDLSTGETLIVHLGMSGRMLISIDEE-----RHGPGQFHHAHSAPGKHDHVVLDMEGGARITFNDPRRFGSMDLAPTEGLEGHPLLAVIGPEPLGNGFSGTYLAERLRGRNTPVKSALLDQGIVAGLGNIYVCEALFRAGISPRRKAGALSSARAEALVPIIREVLQEAIASGGSSLRDYRQADGELGYFQHSFQVYGREDQPCATPGCGARIRRITQAGRSTFYCAQCQR--MGEAKTVIGVIGGSGVYEIDGLEDARWQDVQTPWGAPSDQILTGRIGEVAMAFLPRHGRGHVHSPSSVPYRANIDALKRLGVTDVISVSACGSFQEAMAPGDFVIVDQFVDRTFAREKSFFGPGCVAHVSVAHPTCARLGDACEAAAAATGVRTHRGGTYLAMEGPQFSTLAESRLYREVWGCDVVGMTNMPEAKLAREAELCYASLAMITDYDSWHPDHGEVDVTEIIATLTGNASNARDLVARLPAGL----GPDRAPCPCGCDRALEFALMTAPDKRDPEVLARLDAVAGRVLGAPDKGMQNYLEFEKPLAEIEGKAEELRAMARANSEMDVEKEAAALDAKADKMLRELYAELTPWRKCQVARHPNRPHCMDYVNRLFTDFMPLAGDRNFADDHAVIGGLARLEDRPVMVIGHEKGHDTKTRIERNFGMARPEGYRKAIRLMDLADRFRLPVVTLVDTPGAYPGKGAEERGQSEAIARSTERCLSIGVPLVSVIIGEGGSGGAVAFATADRLVMLEHSIYSVISPEGCASILWKDAEKMREAAEALRLTAQDLKALGVIDRIVKEPVGGAQRARDAAIDAVGRSIVELLEELSGKDGKALRDARRQKFLDMGSKGLAAMILGIGTDLANIDRIAGTLERFGDRFRNRVFTPEEQARAARRRDVAGTYAKRWAAKEACSKALGTGL-RMGIAWRDMQVTNLATGQPVMHVTGWAAERLRAMTPPGHESVIHVTLTDDHPWAQAFVVIEALPKKDKGDTGGG-------MPALCRDCLTSFPGPETAPGAVAVPATPLRCPACRSPRVVSHPELESLGIAHMDCDAFYASVEKRDNPELRDKPVIVGGGRRGVVTTACYIARIRGVRSAMPMFKALRLCPEAVIVKPRFEVYVETSRAIRQMMEDLTPAVEPLSLDEAFMDLTGTARLHGHPPAVMLAGLIKRMQSELGIAGSIGLSHNKFLAKIASDLDKPRGFSVIGVAETDAFLRDRPVRLLSGVGDSFQSALDAAGIRTFADLLRWDRADLAARFGSGGDRLWHLARGIDRRPVSARATVKSISNETTFAEDISDPELLDGHLWRLAEKVSDRAKAQDRAGRVVMLKLKSADYAVLTRRLSLREPTQMADTIYRTAAGLYQQLD-RRGPWRLIGTGLSDIVQ-AADADRSGDLLDQGAERRNRAERATDAIRRKFGKDAIVKGRSLR----------------MTKFSDLKLDPKVLKAVEEAGYETPTPIQAGAIPPALEGRDVLGIAQTGTGKTASFTLPMISMLRRGRARARMPRSLVLAPTRELAAQVAANFDIYAKNTKLTKALLIGGVSFKEQDQLIDKGVDVLIATPGRLLDHFERGKLLLTGVQIMVVDEADRMLDMGFIPDIERIFGLTPFTRQTLFFSATMAPEIERITNTFLSNPAKIEVARAATTSETIEQALIMHTPKRRDMTAKDKRAILRSIIDSEGEGCSNAIIFCNRKTDVDVVAKSLKKHGYDAEPIHGDLDQSHRTRTLTGFREGKIRFLVASDVAARGLDIPMVSHVFNFDVPSHAEDYVHRIGRTGRAGRKGKAYTIATPHDDKLLAAVESLVKMTIPRL--DSPLG--GGAPASDAGPAAE----------SGDEKPTT------DRKR--SRRRGKSDTKADAKPDAKPEAETRSEEAEAPVAVAEDNAGEKPKRRRRSRRSDAVEGAEAVQAAKSDEKADAAEVSEAPKAEKVEKAAEEASTDARAESRSAPRSEPRS--------------EARSAPRAERDQNQVRNRDGNRGRDNGDRKGVVGMGDHMPEFLTRNFMDQAG---MTDLVPVRRALLSVSDKTGLIDLGRALAERGVELLSTGGTAAALRDAGLDVRDVSEVTGFPEMMDGRVKTLHPMVHGGLLALRDDPSHVAAMDEHGIGAIDLLVVNLYPFEQTVARGALYDECIENIDIGGPAMIRAAAKNHGFVNVVVDVEDYEPLLAEMAANDGATSLNFRRRLAQNAYARTGAYDAAVSNWMAGALELAAPRRRVVAGELSQTLRYGENPHQSAAFYVDGRDRPSIGTARQHQGKELSYNNINDTAAAYELVAEFDPAEGPACAIIKHANPCGVARGDSLLDAYRAAYDCDRTSAFGGIVALNQPLDAATAEEITQIFTEVVIAPGADADAMEIFAAKKNLRLLTTDGLPDVTAAALSFRDVPGGMLVQDRDVGRITLDDLKVVTKRAPTEEQMRDLLFAWTVAKHVKSNAIIYASGGATVGVGAGQMSRVDSTRIAARKAQDMAEALGLSKSPAEGSVVASDAFFPFPDGLLTAADAGARAVIQPGGSMRDDEVIAAADKAGLAMVFTGMRHFRHMNAKELNDKTPDQLKDQLAELKKEAFNLRFQQATQQLENTARMRTVRRDVARVKTVLNQKAAAAAAEE-MLTRKQLELLSFINDRLNSDGVPPSFDEMKEALDLRSKSGIHRLITALEERGFIRRLAHRARAIEIVKLPDAIGGQP-AG----FEPRVIEGDRRD-----PPAGAMAISSIHAMELPVMGRIAAGTPIEAISEVSHNVAVPGSMVSNAGNHYALEVKGDSMIEAGINDGDIVVIRDQNSADNGDIVVALVDGQEATLKRLRRKGGMIALEAANPAYETRVLPADRVKVQGRLVGLIRSY-----MSDFDDIDAFDGAAAASLSARAMAARPA-PYLDDLNPAQRAAVEALDGPVLMLAGAGTGKTKALTTRIAHLLNSGRAWPNEILAVTFTNKAAREMKTRVGRLLGQAVEGVPWLGTFHAICVKLLRRHAELV---------------------G-------------LKSSFTILDTDDQIRLLKQLIAAAQIDEKRWPARQLAHIIDGWKNRAWTPANVPASEGTAY-NGQGVPLYAAYQERLRTLNAVDFGDLLLHVVTIFQTHEDVLEQYRRRFKYILVDEYQDTNVAQYLWLRLLAQGHRNICCVGDDDQSIYGWRGAEVGNILRFEKDFPGATVVRLEQNYRSTPHILAAASGVIAANKGRLGKTLW-------TAETEGEKLRLIGHWDGEEEARWIGEEVESLQRGSRGM-----------------------------------------------------------------------EPVSLDAMAILVRASHQMRAFEDRFLTIGLPYRVIGGPRFYERMEIRDAMAYFRVATSPEDDLAFERIVNTPKRGLGDKAQQKIQQAARSNGVSLVEGARILLQAKGLGGKGAGELQRLVEGIGRWHG--------------AILDG------------------RQSHIEIAETILEESGYTEMWQNDKTPEAPGRLDNLKELVKALENFDNLQGFLEHVSLVMDNETDAGEE-KVSIMTLHAAKGLEFPVVFLPGWEDGLFPSQRSMDESGL---------KGLEEERRLAYVGITRAEQLCTISFAANRRVFGQ----WQSALPSRFIDELPEAHVEVLTPPGLYGGGYGAAGMATSASPASV-GIGSDLHEKASRADVYNSPGWRRMQSRASERGMSQPRESRNVVIDLEAVSAFSEGDRVFHQKFGYGAVAGIEGDKLEIAFDKAGTKKVVARFVVAAAEAGDVPF---------------MLSGRRLDLLDPTPMDIEIEDIARGLAFVARWNGQTEGEFAYSVAEHSLLVEEIFGRLNPA-APVRWRMAALLHDAPEYVIGDMISPVKSSVGPGYAELDQRLTAAIHLRFGLPAAVPKTIKAKIKRADRVSAWLEATCIAGFTEAEADRLFGKMPAPLREGLSIRLRP--PVEVRRDYTARHRTLLASMEG----------MTFRHRHLLGIEPLHQEDIRTILDLSERYVDLNRQSKKHLDVLSGLTQINMFFENSTRTQASFELAGKRLGADVMNMAMQASSLKKGETLIDTALTLNAMHPDLLVVRHPHSGAVNLLADKVNCAVLNAGDGRHEHPTQALLDALTIRRAKGRLHRLSIAICGDIAHSRVARSNLLLLGKMENRVRLIGPPTLMPSRIEEFGVEVF-DDMREGLRDVDVVMMLRLQKERMDGGFIPSEREYYHRFGLDEEKLAHAKPDAIVMHPGPMNRGVEIDGTLADDINRSVIQEQVEMGVAVRMAAMDLLSRHGREGED--QA--------MANSKRTLFLKRRLRVRNKLRKM--NAGRPRLSVHRSNKNISVQLIDDVNGVTLASASSLEKDLGVLGK----NNVEAAAKVGTAIAERAKKAGVEECYFDRGGFLFHGKVKALADAARENGLKF----------MRQPLPYETQVEIFSRFRAQE---------PEPRGELEHVNAYTLLVAVALSAQATDAGVNRATRALFQVADTPRKMLDLGEEALVEHIRTIGLFRNKARNVIKLSRILVEEHEGEVPSSRAALQALPGVGRKTANVVLNMWWGHPAQAVDTHIFRIGNRTGICPGRDVVAVERAIEDHVPVEFQRHAHHWLILHGRYICKARKPLCANCLIRDLCPFEEKTV-----------------------------MLDDTKPAAR--------PANPRFSSGPCAKPPAWTLDKLRDAPLGRSHRAAVGKERLLKAIEDTREILGIPADYRIGIVPASDTGAVEMAMWSMLGQRPVEMVAWESFGAGWVTDAVKQLKL-DARVHEAPYGQIVDMNALDYDRDVVFTWNGTTSGVRMPDGQAIPADRDGLTICDATSAAFAMDLPWDKLDVTTFSWQKVLGGEAAHGMLILSPRAVERLESYTPDRPLPKIFRLTKGGKLIEGIFKGETINTPSMLCVEDYLLALDWARSVGGLKGLIARADANAGAVADFVAAHDWIENLAEDPATRSTTSVCLKFTDARIT--DGAA---FAKAVAKRLEGEGVALDVGAYRDAPAGLRIWCGSTVETSDIQAMLPWLDWAFNAEIDAQRG--------MAAQKTDAGRFFEDYRLAQVIEHAVPRTVSGGERALYHALYPARHALYSSDEFARGCGLPASPLDDLAGFHLVFGKSVPDISINAVANLGYAEGRFLRPVTPGDTLRATSEVIGLKQNSNGRTGVVWVRTRGLDQNGDCVMEFVRWVMVRKRDEDAPAPETVVPDLAHAVAASDLP-LPEGLDFSTYDFTAAGEPHRFDDYEVGEKIDHVDGVTVEEAEHMLATRLWQNTAKVHFDASSRPD---GRRLIYGGHVISMARALSFNGLANAQMIVALNGGSHANPCFAGTTIRAWSEVLDKAPTDRPGVGALRLRLVATSGGEA--FALRDEDGKYLPHVLLDLDYWALVPV-----MSKSKKSEFRPNDHVVYPAHGVGQIISIEEQEVAGLTLELFVISFEKDKMTLRVPTNKATEVGMRSLSSPDVVSKAMDTLKGKAKVKRAMWSRRAQEYEQKINSGDLISIAEVVRDLHRTDDQREQSYSERQLYEAALERLTREIAAVSGGNEDEAQKRVGDVLVSRA--VAAMLTDKDRIFTNLYGMHERTLAGARARGHWDGTGDIIKRGRDAIVEEMKASGLRGRGGAGFPTGLKWSFMPKESDGRPSYLVVNADESEPGTCKDREIMRHDPHTLIEGCLIASFAMNAHTCYIYLRGEYIREREALQAAIDEAYDAGLVGPNAAGSGWDFDIYLHHGAGAYICGEETALLESLEGKKGMPRMKPPFPAGAGLYGCPTTVNNVESIAVVPTILRRGAEWFSGFGRPNNAGTKLFAISGHVNNPCVVEEAMSIPLQELLDKHCGGVRGGWKNLKAVIPGGSSVPLLPADIC-DEAIMDFDWLR-EQRSGLGTAAVIVMDQSTDVIKAIWRLSKFYKHESCGQCTPCREGTGWMMRVMDRLVRGDAEPHEIDMLLDVTKQVEGHTICALGDAAAWPIQGLIRHFRDEIEERIAHKRRGR-----APAVAAEMTELFA--YTDGACSGNPGPGGWGALLIAREDGKVIKERELKGGEADTTNNRMELLAAISALESLTRPSTLVIVTDSAYVKNGVTSWIHGWKRNGWKTSTRKPVKNEDLWRRLDEAQARHNVEWRWIKGHAGHEENERADELARAGMAPFKPAARRPAGAAGRRMPQFLDAREPGFEARFQALLGSKREDAPDVDDTVAAIIADVRQRGDEALVELTAKFDRLQLTPDTLAFTDDEIDAYCAQVPAAERAALELAAARIRAYHDRQLPRDESWTDEAGATLGWRWSPVSAAGLYVPGGLASYPSSVLMNAIPAQVAGVGRLVVTVPTPDGAVNPLVLLAARLAGVHEVYRIGGAQAIAALAYGTATVRPVDKITGPGNAFVAAAKRRVFGKVGIDMIAGPSEILVIADRDNDPDWIAADLLSQAEHDESAQSILITDDEAFGQAVARAVEARLETLERRAIAGPSWRDYGAVITVRDLAQAAELSDRIAPEHLELCVADADALAARITHAGAIFIGAWTPEAIGDYIGGPNHVLPTARSSRFSSGLSVLDFMKRTTLARMSPEALGAIGPAAETLARSESLEAHGLSVRLRLDRLNGAGQ----MADYPR---QFDKDGLLACARGELFGEGNPQLPEPPMLMMDRVTDISADGGLHGKGHVVAEFDIDPDLWFFACHFPGDPVMPGCLGLDALWQLTGFNLGWRGMQGRGRALGVGEVKFTGMVTPETKLVTYHVDFTRVID-RRLKMGIANGRMLADGEEIYTTENMKVGLFQDQEG-MSNAQLETAIEAAWEGRDQITPQTKGETRDAIETTLNALDSGTLRVAERRESGDWHVNQWAKKAVLLGFRLNDMEMQSGGPQGSGWWDKVDSKFKGWNEADWKKAGFRAVPNCVVRRSAYIAPGVVLMPSFVNLGAYVDEGTMVDTWATVGSCAQIGKGVHLSGGVGIGGVLEPMQAGPTIIEDNCFIGARSEVVEGVIVREGSVLGMGVFLGQSTKILDRETGKVTYGEVPSGSVVVAGSMP-SSNGVHLYCAVIVKRVDEKTRSKTSINELLRDMAEFF-STGAGIA-LIIVAQVLAMVAFVMISLLFLVYGDRKIWAAVQMRRGPNVVGVFGLLQSVADALKYVVKEVVVPAGADKAVFLLAPLVSFVLALVAWAVIPFTDT------WVLADLNVAILYVFAISSLEVYGVIMGGWASNSKYPFLGSLRSAAQMISYEVSIGLIIVGVILSTGSLNFGDIVRAQDG---DYGFFNWYWLPH----LPMVVLFFISALAETNRPPFDLPEAESELVAGYQVEYSSTPFLLFMAGEYIAIFLMCALITLLFFGGWLSPIPGVPD-------------GALWMVAKMAFFFFMFAMVKAIVPRYRYDQLMRLGWKVFLPFSLGWVVFVAFAAQFGWFGGAYARWIVGV--MDHFLYRDGQLHAEDVPVSEIARAVGTPVYIYSEATLLRHFRLFDEALSGMDHLVCFAMKSNSNQAVLKLLADAGAGMDVVSGGEYARARAAGVPGERIVFSGVGKTEAEMRQALEGGIRQFNIESEPEMALLSGVAESMGKVAPVTIRINPDVDARTHEKIATGKSENKFGIPISRASAVFAEAAALPGLEVVGIDVHIGSQLVELEPFEQAYTKVAELTRQLRAEGHDIRRLDLGGGLGIPYTRSNEVPPLPQDYGDMVRRTLGHLDCEIEIEPGRLIAGNAGILVSSVIYLKSGEGRDFLIVDAAMNDLLRPSMYGAYHDIVPLIEPSSDTPLAPIDIVGPVCESGDTFAKQRPMPPLASGDLVAFRSAGAYGAVMSSEYNTRPLIPEVLVRGDQFAVIRPRPTYDDIINRDKLPEWL------------MDKLTVLGLESSCDDTAAAVVELTRAADGQVTGPGRVLASVVHGQTDLHAAFGGVVPELAARAHAEKLDLAVEQALSQAEVPLSAVDAIAVTSGPGLIGGVMSGVMCARGLAAGAGLPLIGVNHLAGHALTPRMTDGLEFPYLMLLVSGGHCQFLLVRGVDSFTRLGGTIDDAPGEAFDKCARLLGLEQPGGPAVEREARAGDPTAHRLPRPLLDRPGCDMSFSGLKTALLRARDGIVAQKGGLSVQDRADLCAAFQAAASDVLIEKTRRALDVAAGACGGQVPALAVAGGVAANTALRKGLQSLADARGIALVAPPLRYCTDNAAMIAWAGAERLAADLV--EPQDLVPRPRWPLDRTSP---SLLGSGKKGAKA-----MSELRELAMSSKAWPFEEARRLVKRYSKA-----PPEKGFVLFQTGYGPSGLPHIGTFGEVQRTTMVRRAFEALS--DIPTKLLCFSDDMDGLRKVPGNVPNQEQMKEDLNLPLTKVRDPFGTHAGFAQHNNARLCAFLDSFGFEYEFASATEYYTSGRLDEMLLRALERFDKIMEIMLPSLGADRQATYSPFLPISPKTGHVLQVPTLERNLSRGTIVYEEPD-GERVEVPVTGGNVKMQWKPDWALRWAALGVDYEMSGKDLIDSVTLSTKICRALGETPPESLSYELFGDEKGQKISKSKGNGLSMEEWLTYAPPESLSYFMYQKPKTAKRLYFDVIPKAVDEYHQQLRAFPEQDLKSQLANPVWHIHEGKPPVSSLTVPFAMLLNLASAASAEDKTALWGFINRYAPEATPESHPDLDRAAEFAVRYFNDFVRPAKTYRAPTEQERAALADLAGRLRDWQGGT----------------------DAEELQSLVFAVGKDHGFEQLRDWFMAIYEVLLGATQGPRFGGFIALFGVTETADLIDAALAGKLG---MRCPFCGNVDTQVKDSRPAEDHVAIRRRRFCPACSGRFTTYERVQLRDLVVIKTNGKRENFDRDKLERSIRIALQKRPVEPERMDQMISGIVRRLESMGDTDISSKLIGEIVMEALARIDTVAYVRFASVYKNFQAADDFEDFVAELRPPSTPEG-----MPVVVVESPAKAKTINKYLGDNYTVLASYGHVRDLPPKDGSVDPEQNFEMKWEVGTDSRKHVKAIADALADDNNLILATDPDREGEAISWHLEEALRKRKAIKKDTPVSRVVFNAITKSAVTEAMKNPRQVDMELVEAYLARRALDYLVGFNLSPVLWRKLPGAKSAGRVQSVCLRLIVEREMEIEAFDPREYWSVKALLATPRGQEFEARLTVMGGKKLDKFDLANETAAEMAVQAINSRDLSVVSVEAKPASRNPSAPFMTSTLQQEASRKFGMGARQTMSAAQRLYEAGHITYMRTDGIDMAPEAVMAARDAIKDRYGADYLPKSPRMYKNKAKNAQEAHECIRPTDMTRDAASLRLSEPDQRKLYDLIWKRTLASQMEAARMERTTVDVGSRDEQVVLRATGQVMLFDGFIRVYEEGRDDT-GDDEDGNRLPQIAEGEPA-----------------------------------------------EKRGVTPEQHFTQPPPRYTEATLVKRMEELGIGRPSTYASVVTTIQDREYVRKEKNRLIPEDKGRLVTVFLLNYFRKYVGYEFTANLEDELDDITAGNRDWQDVLARFWREFSAAIAETSELRITEVLEKINEVLEPHLFPAKE-DGS---DPRLCPNCGQGRLSMRTARSGGAFIGCSNYPECRFTRPFGPPGVEGDE--IGPDGKLLG--EDQGDAITLRKGRFGPYVQRGEATEE-QPKPPRASLPK----GWAQDDIDLEKALLLLNLPREIGHHPEDGELVEAGIGRYGPYVKH-------GRVYANLPEVDEVFTIGMNRAVEVLAQKATRGRGR-AAPAKPLKELGEHPESGGAINVLDGRYGPYVKWEKVNATLPKGTEPGDVTLEMAVQLIEEKAAKGGKK----KAAA--KKPAA----KKTAAKKPA-----------AKKATTKKAAPKKAAKTEE-----------------MIQMQTNLDVADNSGARRVQCIKVLGGSKRKYASVGDIIVVSVKEAIPRGRVKKGDVRKAVVVRTAKEVRREDGTAIRFDRNAAVILNNNNEPVGTRIFGPVVRELRAKNFMKIISLAPEVL-MD-NLLSFVTFIPLLAAGILALFLR---------GDDAAAQRNAKWLALAATSATFLISLFLLAEFDPGNTKFQFVEEGEWLGGLKYKMGVDGISILFVMLTTFLMPLVIASCWN-VTYRVKDYMVALLVLETLMIGVFCALDLILFYLFFEAGLIPMFLIIGIWGGANRIYASFKFFLYTLLGSVLMLVAMVMMYVDAGTTDI-------PTLLNHQFDSQALNILGIHVVGGLQTMMWLAFFASFAVKMPMWPVHTWLPDAHVQAPTAGSVVLAAILLKMGGYGFLRFSLPMFPAGSEVMAPMVLWLSVIAIIYTSLVALAQHDMKKLIAYSSVAHMGYVTMGIFTANQQGIDGAIFQMLSHGFISGALFLCVGVIYDRMHTREIDAYGGLVNRMPAYALIFLLFTMANVGLPGTSGFVGEFLTLMGVFQVNTWVAVLATTGVILSAAYALWLYRRVVLGDLIKESLRSITDMTGRERAIFAPLVVMTLLLGVYPALVTDIIGPSVEALVADYETALVAG--SDTRLAQN-----MADNDIEIDLDDLGHRMDGAMTALRSEFQSLRTGRASASMLDTITVNAYDTVTPLNQVGTVNVPEPRMLTVTVWDKQLVNKVEKAIRESGLGINPVMDGTVIRLPIPELNEERRRELTKIAAQYAEAARVAVRNVRRDGMDQIKNAKSAG-MSEDDQKIWSGEIQEMTDKHIAAIDAALESKQAEIMQVMN-------TR--PDF-----DRHMLEALVCPVTQAPLSYDAERQELVSKAAHLAYPIREGIPIMLESEAREVE--------MDEPQITEDLIAA---------HGLKPDEYQRILEIIGREPSFTELGIFSAMWNEHCSYKSSKHWLRTLPTDGPQVICGPGENAGIVDIGDGQAVVFKMESHNHPSYIEPYQGAATGVGGILRDVFTMGARPVAAMNSLSFGAPDHPRTRTLVNGVVAGIGGYGNCFGVPNVGGEVRFDPAYNGNCLVNAFAAGIADTDKIFYSAASGVGMPVVYLGAKTGRDGVGGATMASAEFDDTIEEKRPTVQVGDPFTEKRLLEACLELMQTGAVISIQDMGAAGLTCSAVEMGDKGDLGVKLELDRVPVREDHMTAYEMMLSESQERMLMVLNPELEAEAKAIFDKWDLDFAIVGETIAEDRFLILHGNEVKADLPLKALSGTAPEYQRPWVPTPAATPLADVPEVDP---IDALRALVSSPNYASKQWVWEQYDHMVMADTIVAPGLG--AGVVRVHGTDKALAFTSDVTPRYVRANPVEGGKQAVAEAFRNLCAVGARPLATTDNLNFGNPEKPEIMGQLVGAIQGIGAACTALDMPIVSGNVSLYNETDGEPILPTPTIGAVGLVDHVDHLIAGT-AREGDLAILLGETVGHLGQSALLAEVF-NRHE---GDAPPVDLEAERRHGEFLRD--NRTLVQAATDLSDGGLALAAFEMAEAAGVGVTLDDGAT-------AHAFGEDQGRYLLACSFDQAEALMVAAARAEVPVATVGRFGG----DRVRIGASQA-ELAELAQIYRT---SFAAAVA-MKTFTATPADIDKKWILIDAEGVVLGRLAAIVATRLRGKHKPSFTPSQDMGDNVIVINADKIQLTGNKR-MKPNYWHTGYPGGIKSRTTGQILEGEHPERVVIQAVKRMLP-GGKLSRQQMTNLRVYAGAEHGHEAQSPEVLDVKSMNSKNTRS--MAPKVLVSDKLSETAVQIFRDRGIEVDFDPALGKDKDRLLEVIGQYDGLAIRSATKVSDKVLAAATNLKVIGRAGIGVDNVDIPAASRKGVIVMNTPFGNSITTAEHAIAMMFAVARQIPEASTSTHAGKWEKSRFMGVELTSKTLGVIGAGNIGSIVIERAHGLKMKVVAYDPFLSQERADKMGVEKVELDELLKRADFITLHVPLTDKTRNILSRENIAKLKPGVRIINCARGGLVDEAALAEALTDGRVAGAAFDVFETEPATDSPLFNLPNVVVTPHLGAATTEAQENVALQVAEQMSDYLLSGAVTNALNMPSVTAEEAAVMGPWIKLAEHLGAFAGQMTDEPIEAINILYDGAVAEMNLAALNCAAVAGIMKASNPDVNMVSAPVVAEERGITVSKTTQNKSGVFDAYMKLTVRTSKRE-------RSIAGTVFSDGKPRFIQIKGINVDAEIGRHMLYTTNMDVPGIIGVLGQTLGENGVNIANFTLGRHD--GQAIALLYVDDAIGEDVLDQIRATGKFQSVAPLTFDVE--MKTQTMMNRDWIATARTLSQALPYLQRYDGARVVIKLGGHAMGSQEAMESFARDVVLMQQVGVNPVIVHGGGPMINEMLAKLDITSEFVNGKRVTDEATIEVVEMVLSGRVNKRIAQAINDQGGRAVGLSGKDANLMVCDYA-----SP--------ELGFVGDPNSVNPDVLHRM--HDAEIIPVVAPIGAGRNG-ETFNINGDTAAGAIAAALKADRLLLLTDVAGVKGSDGEVLTEMTPQQIRDLTRDGVIAGGMIPKTETALAAIEGGVRAVVILDGRAPNACLLELFTDHGAGSLIRAG-----------MNAATKLPTQLGPIHFVGIGGIGMSGIAEVLLNHGYSVQGSDLKGSKITERLEGLGAVIFE-GQKAENLQNAAVVVISSAIKPGNPELDEARAQGLPVVRRAEMLAELMRLKSNIAVAGTHGKTTTTTMVATLLDAGGVDPTVINGGIIHAYGSNARMGQGEWMVVEADESDGTFNRLPATIAIVTNIDPEHMEHWGDFDRLRQGFTDFVSNIPFYGLAVCCTDHPEVRSLVGRITDRRVRTFGFNAQADVRAVNLTYEAGVAHFDIALQYED----EVIEGCTLPMPGDHNVSNALAAVAVSRHLGMTADEIRAALAGFKGVNRRFTRVGE------VDGVAIIDDYGHHPVEIAAVLKAARQACEGR-VIAVHQPHRYSRLHSLFEDFCSCFHDADVVAIAEVFAAGEDPIEGAGRDDLVAGLIRHGHRHARALLSEDDLERLVREQARPGDMVVCLGAGTISAWANGLPQRLEK----AA-MAITAAQVKELRDTTGAGMMDAKKALTENNGDMEAAVDWLRTKGLAKAAKKSGRTAAEGLVAVAI--EGGEAVALEVNAETDFVAKNADFQAMVADFSKAALKVGS-VDELKASDI--NGKKVEDILTDKIATVGENMAIRRMAKISGE-TVTAYVHNQAAENMGKIGVLIAMNGTD--NGIGRQIAMHVAAANPASLGEADLDQALVEREKSVLTEQARESGKPEQVIEKMIEGRMKKFLSEVTLLGQAFVINPDQTVAEAAKEAGVEITGFVRLEVGEGIEKEAENFAEEVAKMNA--MMSDILIVDDEKDIRELIADILQDEGYSTRLAANSGECMAEINRTPPGLMVLDIWLKDSDMDGIDILKTVKRDNPDIPIVIISGHGNIEIAVAAIKQGAYDFIEKPFNIDQLMVVIGRAMETSRLRRENAELKRADGGATEMIGSSAVFKSLKSQLDKVTGSNGRVMLSGPAGSGKEVAARYIHQHSNRASAPFVCVNCASIEPDRMEEVLFGKESAD-RSVEPGLLEQAHGGIIYFDEVADMPLGSQSKILRVLVDQQFQRVGGMDKVRVDLRVVASTSRDLKAEMAAGRFREELYHRLNVVPIAVPSLEERREDIPELARHFIDDFHRSQGLPLRELSEEAEALLQTMVWPGNVRQLRNVIERVLIL--GPDSGEVSVKELPSSDTG-PEDPDRVILSGSLATLPLREARELFEREYLMTQINRFGGNISRTATFVGMERSALHRKLKSLNVVTSAKAGTRVARVDEME-EAEADGAMLQPKRTKFRKQHKGRIHGEAKGGSTLNFGTFGLKATQPERITARQIEAARRAMTRHMKRQGRVWIRIFPDTPVTSKPTEVRMGKGKGSVDYWAAKVKPGRVMFEIDGVSEAVAREALRLAAMKLPIKTRTVVR-EDWMFSRYKKDA---TKPGAAKV-----NSAPVAAAPRPAAAAPRPQPKAVPT----------PARPQPVDKEQKRREKLTEVKVELHKRLLESLNLSALEHASETELRQEIVSISTEALTEMAVVLNKEERILLYQDLYDEVTGLGPLEPLLKDDTVNDILVNGPKRVFVERNGKLTLSDTTFRDEKHLLRIIDKIVSAVGRRVDESNPYVDARLADGSRFNAMVPPIAVDGSLVSIRKFKKEKLGVPDLVNFGAFSPEMAMYLEAAVACRLNIIVSGGTGSGKTTTLNALSSFIDNSERILTIEDTAELQLQQVHVGRMESRPANVEGKGAVSQRDCLRNALRMRPDRIIVGETRGEEVIDMLQAMNTGHDGSMTTIHANSARDGVSRLENMIAMTGIEMPTKAVRSQISSAVNLIVQASRLQDGSRRMVSITELTGMEGEVISMQEVFRYQRTGLAPDGKIIGHFTATGVRSYYADRFRQWGFDLPSNIYEPVSE------MDE---LRQKYLAATADAADEAALEAIRVQALGKKGEISLKMRELGKMTPEERTTAGPALNALKDEINSALAAKRAALGDAALEARLRDEWLDVTLPGRPRR-TGTIHPISQVTEEVTAIFADMGFSVAEGPQIESDWFNFDALNIPGHHPARAEMDTFYMARAEGDDRPPHVLRTHTSPVQIRTMQERGAPLRIIAPGRVYRCDYDQTHTPMFHQVEGLALDKDLSMANLKWVLEEFVRAFFEVDEVELRFRASHFPFTEPSAEVDIRCSWEGGQLRVGEGDDWLEILGSGMVHPKVLAAGGIDADTWQGFAFGMGIDRLAMLKYGIPDLRAFFDSDLRWLRHYGFASLDQPTLRGGLSR-MSD----QDGKKTLGLR--GG-PR--SGQVKQSFSHGRTKNVVVETKRKRVV-VPKPGASGK-AAGA-ASNRGGDASK-------RPAGITDAEMERRLKALQAAKAREAEEAERREREERERAEDRERRRAEAEAKEREEREREER-ARQKAEEDERKQREAEEAKQ-RAAQPA-PQERAEPQADAGPAGP-----RGGLKP---AATPRKTERDDRPKREAK-----GAG---DRRRSGKLTLNQALSGGEGGRQRSMAAMKRKQERARQKAMGQNVEREKVVRNVNLPEAITVQELANRMAERVADVVKSLMTSGIMATQNQTIDADTAELIIEEFGHKVVRVSDADVEQVIDTIDDKPEDLRNRPPVITVMGHVDHGKTSLLDAIRNAKVVAGEAGGITQHIGAYQV-QAADGQILTFLDTPGHAAFTSMRARGAQVTDIVVLVVAADDAVMPQTVEAIHHAKAAGVPMIVAINKIDRHEANPDKVRTDLLQHEVVVEKMSGDVQDVEVSAIKGTGLDELLEAIALQAEILELKANPDRAASGAVIEAQLDVGRGPVATVLVQNGTLRRGDIFVVGEQYGKVRALINDKGERVDEAGPSVPVEVLGLNGTPEAGDVLNVVETEAQAREIAEYREKAAKE--KRAAAGAATTLEQLMAKAKDDE-NVSEMPILVKADVQGSAEAIVQAMEKIGNEEVRVRVLHSGVGAITESDIGLAEASGAPVFGFNVRANASARNSANQKGVEIRYYSVIYDLVDDVKAAASGLLSAEVRENFIGYAEIKDVFKVSNVGKVAGCLVTEGVARRSAGVRLLRDNVVIHEGTLKTLKRFKDEVAEVQSGQECGMAFENYDDIRAGDVIEIFEREEVERTLS----MTQISLTFPDGNSRSFPAGVTAAEVAADISTSLAKKAISATVDGRHWDLQWPIEGDAAIAINTMKEAEPALELIRHDLAHIMARAVQELWPDVKVTIGPVIKDGWYYDFDREEPFTPEDLGAIEKKMKEIINSRDPVRTEVWDRPRAIAHYESLGEPYKVELIEAI--PGD-EPLRMYWHGDWQDLCRGPHLAHTGQVPADAFKLMSVAGAYWRGDSSRQMLQRIYGVAFQNKEQLKAHLHMLEEAAKRDHRKLGREMDLFHMQE-EAPGQVFWHPGGWTVYTTLQDYMRRKQRAGGYVEVNTPQVVDRKLWEASGHWEKYQDHMFIVEVDEEHAREKS------------VNALKPMNCPCHVQIFNQGLKSYRELPLRMAEFGSCNRYEPSGALHGIMRVRGFTQDDGHIFCEEDQIEAETATFINFLSEVYRDLGFESFKVKFSDRPETRAGSDEVWDKAEAALLNATRAAGIEPELNPGEGAFYGPKLEFVLTDAIGRDWQCGTHQVDFVLPERLDANYIGRDGEKHRPVMLHRATLGSFERFIGILIEEHAGKLPFWLAPRQVVVAAIVSD-ADAYVHEVVATLRAAGIRAEADTRNEKINYKVREHSVAKVPAILAVGMSEVADKTVSVRRLGQKQTQVRTLDEIRDELALEATPPDLRG---MNDDKDDPARPAKGVAPSENTPSDATASAPAPSDPPSGPAPGEGVRRAVPERSHGRLSISASRSPRSLMDETPRLKNAWCARVITLFPEAFPGLLGESLTGKALKEGIWALEPIDLRIFGEGKHRNVDDTPAGGGAGMVLRPDIVDAALNFAARGTPTDRD----EWPVVYLSPRGRPFDQAMARRFSRARGLTLLCGRFEGVDQRVLDHHHVEEVSLGDFVLTGGEIAAQALIDASIRLIPRVLGNQASTEEESFSDGLLEHPQYTKPATWADRDIPEVLLSGHHAKISSWRQAMAERLTKERRPDLWRAYCETHGRDPEEDREL--MGKEKNPRRVADNEALAKTRMLRTSPQKLNLVAAMIRGKKVDKALADLTFSKKRIAADVKKCLQSAIANAENNHNLDVDELVVAEAYVGKNLTMKRGRPRARGRFGKIVKPFSELTIKVR---QVEEQA--MDAILIKGGAELRGRIPIAGAKNACLTLMPATLLSEEPLTLTNAPRLSDIKTMTQLLQSLGTEVTALQGGKVLALSSHDMTSHTADYDIVRKMRASILVLGPMLARDGQAVVSLPGGCAIGARPVDLHLKGLEALGAELELRDGYVHAKAP-GGLKGGTVDFPLVSVGATENVLMAATLAKGTTVINNAAREPEIVDLADCLRRMGAQIEGDGTSTITVQGVDRLSGATHPVVTDRIELGTYMLAPAFTGGEVELLGGRLDLVEAFVEKLDAAGIDV--TETDEGLKVRRRGDRVQAVDVTTEPFPGFPTDLQAQMMALLCTAEGTSRLEEKIFENRFMHAPELQRMGAHIEVHGGTAVVHGVERLRGAPVMATDLRASVSLILAGLAAEGETVVRRVYHLDRGYEKLEEKLAACGAQVERIRES----MSDKSTNHPTACLALADGTLFFGKGFGATGETTAELCFNTAMTGYQEIMTDPSYAGQVVTFTFPHVGNVGVNPDDDESGEPVAA----GIVVKWDPTEPSSWRAAEDLSDWMAKRGRIGIGGIDTRRLTRAIRQQGAPHVALIHAPDGNFDIEALVAAARGFPGLVGLDLAKEVTCAQTYQWNEMRWAWPDGFQPRTEPGHRVVAIDYGAKRNILRSLASAGCDVTVLPATATAEEILAHDPQGLFLSNGPGDPAATGEYAVPTVQEVLAKSQIPVFGICLGHQILARALGARTVKMNHGHHGANHPVKNVETGKVEITSMNHGFTVDSQSLPEGVMETHVSLFDGSNCGIRMTDRPVYSVQYHPEASPGPQDSLYLFERFSDAIREQA----------MRPSGRQISELRPVSIETGVMKHAEGSCLIRCGDTHVLCTATLEERVPPFLKNTGLGWVTAEYGMLPRATNTRMRREAKNG-QSGRTQEIQRLIGRSLRAGIDRSALGERQISIDCDVIQADGGTRCASITGGWVALRLAVNKLMKAGDITSDPLTDHVAAVSCGVYAGQTILDLDYAEDSQAGTDANFVMTGSHGLIEIQGSAEGATFSRTQFDELMNLAEAGITQLVEAQKAAI--MATVAATDANFDEIVRQSDVPVVVDFWAEWCGPCKQIGPALEELSDQYDGKVKIVKVNVDENPNSPAQLGVRGIPALFMFKDGEVISNKVGAAPKAALQSWIDEAIMPTIQQLIRKPRQPKVKRSKSLHLEGCPQKRGVCTRVYTTTPKKPNSAMRKVAKVRLTNGFEVISYIPGESHNLQEHSVVLIRGGRVKDLPGVRYHILRGVLDTQGVKDRKQRRSKYGAKRPK--MARWLFKSEPSTWSWQDQQARGAKGEEWDGVRNYQARNFMREMKLGDQGFFYHSQSEKAVVGIVEVIAESHPDSTTDDARWECVDIRAIRPLPTPVTLEQIREEPRLAEMVLVRNSRLSVQPVADDEWDLICELGGIPKA---------------------MLTQPENSRAGFVALIGEPNAGKSTLLNRMVGAKVSIVTHKVQTTRARIRGVALEGASQIVFVDTPGLFRPRRRLDRAMVAAAWSGAADADVVVLLIE--------------AHRGLTEGVEAILEALNERAPDRPVALAINKIDRVKSEALLALTEKLNAAYPFTKTFMISAERGHGVDHLREWLAQEVPAGPWLYPEDQIADLPMRMIAAEITREKLTLRLHQELPYQLTVETESWEERKDGTVRIDQLIYVIRDGHKGIVLGNKGETIKAVSQASRAELTEFLGRTVHLFLQVKVRPNWLEERERFSEMGLDFNDGD-MNDITDTAPSD------ELSESLSRAIGERYLTYALSTIMHRALPDARDGLKPVHRRILYAMRELRLSSNGGFRKSAKISGDVMGNYHPHGDAAIYDAMARLAQDFNVRYPLVDGQGNFGNIDGDNPAASRYTEARMTAVAEALLEGLNEDAVDFRENYDGTLTEPVVLPAAFPNLLANGSSGIAVGMATNIPPHNIEELCNACLHLIKSPNARDETLLEHVPGPDFPTGGVLVEPRENILEAYRTGRGSFRLRCRWEVEELGRGQWQVVVTEIPYQVQKSKLIEKIAEIIQTKKIPILGDVRDESADDVRLVLEPKSKNVSPDVLMNMLYRHSDLEVRFSLNMNVLIDGLTPKVCSLREVLQAFLAHRREVLLRRSRYRLDKIDHRLEVLEGYIVAFLNLDRVIEIIRTEDEPKPVMMAEF----------------------------------------------------------------DLTDVQTEAILNMRLRSLRRLEEMELKREHEALLLERAGLEDLLASEELQWQRIAEQLRETRRQFGRTYE------GGARRTTFAEAGVVEEV-PLEAMIDREPVTVVCSQMGWIRAMKGHIALDQEFKFKDGDGPRFAFHAETTDRLLVFGTNGRFYTVSASTLPGGRGMGEPLRLMVDLPNEAEIVDLFTHEAGRRLLVASSAGDGFIVPEVDVLAQTRTGRQVLNVRGD-TVAKVCHPVSG--DHVAVVGENRKVLVFPIDELPEMGRGKGVRLQKYK-----------DGG--LSDLTTFTLTEGLSWKDPAGRTRT--QTELEEWTGKRAGAGRMAPRGFPRDNRFN---------------------------------MAAEWWDPSGKFKPLHMLNPCRLDYITRQIAAEYGRDLSVPRPFEGLRILDIGCGGGLLSEPMARLGAEVVGADAAERNIPVAQVHAAQSGLDIDYRHTTAEAMAEAGE-TFDAVLNMEVIEHVSDPAAYLRACHALLRPGGVMVCSTLNRNPKSFLFAIVGAEQVMRWLPRGTHEWSKFITPDELYDLLRQAGFEPVDRKGFVFNFALWDWSLSDRDLSVNYVTTSLRPGG-------MALKVAIQMDPIEHVNIDADSTFRIAEEAQKRGHSLFYYTPDKLSYRTGRIVAGGHRLTVRREQGNHAELGPFEEVDLA-DWDVVWLRQDPPFDMGYITTTHLLDMITPGTLVVNDPFWVRNYPEKLLVLRFPELTPPTTIARDLATLRAFKQEHGDIILKPLYGNGGAGVFRLGPDDRNLASLHELFAGINREPLIAQKFLPAVSRGDKRIILVDGEPVGAINRVPAAGETRSNMHVGGRPEKVGLTERDREICAAIGPLLREKGQIFVGIDVIGDWLTEINVTSPTGIQELERFDGSNIAALIWEAIEARRAG---MSRIDENAQFVPVRIAVLTVSDTRSIEEDRSGQTLVDRIEEAGHQLADRRILPDERAQIAAQLRDWCADPAIDVVISTGGTGLTGRDVTVEAHRDVYEKEIEAFATVFTIVSMKKIGTSAVQSRATGGVAQGTYLFALPGSPGACKDAWDEILKLQLDLRHRPCNFVEIMPRLDEHLRRK--MKLPFPQAPAPTPEEEEAGRMLFAQGAEFLKGVVAMDGLPPADRIETCFAGRSNVGKSSLINALTGRKGLARASNTPGRTQEINYFTLT-----DDHYLVDLPGYGFANAPVAVVEKWQKLLKSYLSGRPTLRRAFVLVDHRH-GVKPVDDQIMTLLDRSAVPFQVVMTKADKVKAAEREKILDQVRAALQTHPAAFPELVVTSSEKGDGIATLRAIINSLV------------MKLVLGSGSPRRLELLAQIGITPDAVRPPDIDETPHRAELPRPYCARMAAEKAAAGTLADDELMLCADTTVALGRRILGKPATAGEAAEFLLALSGRRHKVITAVALRWGT-RMWQADVVTTVRMKRLSDEELNAYLATGDWQGKAGAYGIQGPAGALIPWISGSFTGVVGLPLAETAGLLRAAGYLPRPQTQPEAPMSNPTLLILPGDGIGPEVMTEVRRIIDWFGDKRGLKFDVAEDLVGGAAYDAHGTPLTDKTMAHAQSVDAVLLGAVGGPKYDDLDFSVKPERGLLRLRKEMDLYANLRPAQCFDALADFSSLKRDIVAGLDIMIVRELTSGIYFGEPRGIQADNEGGRVGVNTQRYTTNEIRRVARSAFELARRRSNRLCSMEKANVMESGILWREEVQWVHDNEYPDVELTHMYADAGAMQLVRAPKQFDVIVTDNLFGDLLSDAAAMLTGSLGMLPSASLGAPAENGRPKALYEPVHGSAPDIAGQGKANPCACILSFAMALRYSFDQGTEADRLEAAVESVLRDGVRTADLMQAGDRDPASTTQVGDAVLKALDASLMPGTFETRLADLGETLPD---APAPAANYVPYVLLGDVLYVSGQISQDRDGFITGRLGDDLGVEQGARAARACALSLLAQVRAACDGDLDRLVRVVKLTGFVNSTPDFTDQPKVINGASDLFAEVLG-DAGRHARAAVSAASLPLGVAVEIEGIFQVR-MPEDIINSFMNGPDEKGRFGDFGGRFVSETLMPLILELEEQYEHAKTDQSFWDEMNDLWTNYVGRPSPLYYAERLTKHLGGAKIYLKRDELNHTGAHKINNVLGQIILARRMGKTRIIAETGAGQHGVATATVCAKFGLKCVVYMGAHDVERQAPNVFRMKLLGAEVVPVTSGRGTLKDAMNDALRDWVTNVRDTFYCIGTVAGPHPYPAMVRDFQAIIGKEAREQM--MAAEGRLPDTLIAAIGGGSNAMGLFYPFLDDKDVRIIGVEAGGKGVNAKMEHCASLTGGRPGVLHGNRTYLLQDDDGQILEGYSISAGLDYPGIGPEHSWLHDTKRAEYVAITDVEALKAFQLCCELEGIIPALEPSHALAHVMKIAPELPADHLICMNLCGRGDKDIFTVAKALDVNMGPSD-----MKADAIKLDGAKAGSVELDDAIFGLEP-RADILHRVVRWQRNNAQQGTHKVKTRSETSYSTKKIYRQKGTGGARHGDRNAPIFRKGGIYKGPTPRSHGHELTKKFRKLGLRHALSAKQSAGELVILDTLELKDAKTAVLSRQVKDLGWKRALVIDGATVDENFARAARNLDGVDVLPSMGANVYDILRSDTLVLTKAGVEALEARLK-------MHAFRTHTCADLDKSNVGDTVRLSGWVHRVRDHGGILFIDLRDHYGVTQVLADPDSPVFADVEKVRSEWCIRIDGTVKARDPELVNPKIPTGEVEVFVRDIEVLGEAA-ELPLMVFGDQEYPEETRLRYRFLDLRREKLQRNMTLRSDVVASIRKRMWGADFREYQTPIITASSPEGARDFLVPSRLHPGKFYALPQAPQQFKQLIMVSGFDKYFQIAPCFRDEDPRADRSPTDFYQLDMEMSFVTQQDVFDTIQPVVQGIFEEF----GGGRKVDT--TWEQISYRDAAMWYGTDKPDLRNPIKMQDVSEHFRGS--GFAIFAKLLEQDGTQIRAIPAPKGG--SRKFCDRMNAFAQKE-GLPGMGYIFWRETD-------------------------------GQ----MEAAGPLAKNIGPERTEAIRQQLDLGVGDAAFFLGGKPSAFETVAGKARTEIGNELGLTDTDRFAFAWIVDFPMYEKDEETGAIDFSHNPFSMPQGGLEALQG-----DPLEVLGYQYDLACNGYELISGAIRNHKLDIMYKAFELAGYGPEEVDKRFGGMVKAFRYGAPPHGGCAAGIDRIVMLLADEANIREVIMFPMNQRAEDLMMEAPSEPTNEQLRELRLRVIPAED-MFKGSMPALVTPFKDGAVDFGTLESLVEWQIGEGTDGLVPVGTTGESPTLSHAEHEAVIDAVIRAAKGRVPVIAGAGSNNTTEAVRLVNHAQGAGADAALVVTPYYNKPTQEGLYAHYARLNE-VGLPIIIYNIPGRSVVDMSPATMGRLAELENIIGVKDATGDLARVSQQRAACG--PEFIQLSGEDATALGFNAHGGVGCISVTANVAPRLCAEFQKATLAGDYAKALEYQDRLMPLHEAIFMEPGVAGAKYALSLLGRCADSVRLPLLPLGEVTRLAIKDAMRHAGLIG-MAI-TSANQLELLQTAEAVAREKMIDPSLVIEAMEESLARAAKSRYGAEMDIRVSIDRKTGRATFTRVRTVVEDDAVENYQAELTVKEA------------------------------KQYK------------------EDPQIGDEIVDEVPPVEMGRIAAQSAKQVILQKVREAERDRQYEEFKDRAGTIINGVVKREEYGNVIVDIGRGEAVLRRNEKIGRESYRPNDRIRVYIKDVRREPRGPQIFLSRTAPEFMAELFKMEVPEIYDGIIEIKAVARDPGSRAKIAVISYDSSIDPVGACVGMRGSRVQAVVNELQGEKIDIIPWNEDQPTFLVNALQPAEVTKVVLDEEAERIEVVVPDEQLSLAIGRRGQNVRLASQLTGLDIDIMTEEEESKRRQAEFAERTQLFMDTLDLDEFFAQLLVSEGFTNLEEVAYVDADELLVIDGVDSDTAGELQARARDHLEAINKKALERAAELGVDQSLVDFEGLTPQMVEALAEDGILSLEDFATCADWELAGGWTT-VEGERVKDDGLLEKFDVSLEEAQDMVMTARVMLGWVDPTEMLA----EDTGEDA------------------EGEEGQ-AAEGDAAPSDDETATPAGSGA-------------MTQRVTFQLQARDGKARRGVIQTPRGEIRTPAFMPVGTAATVKAMLPESVRATGADVLLGNTYHLMLRPTAERIAALGGLHKFMNWERPILTDSGGFQVMSLAELRKLTEKGVTFRSHVDGARHELTPERSMEIQA-LLGSDIVMSFDECTPYPATEEVAASSMRLSMRWAQRSRDAFGDRPGHALFGIQQGSTFRELREESAQALQQIGFDGYAVGGLAVGEGQEAMFDVLDYAPDMLPEDKPRYLMGVGKPDDIVGAVARGIDMMDCVLPSRSGRTGQVFTHRGVVNIKNARHRDDPRPLDESCTCPACRSYSRAYLHHVFRAGEMISGMLLTWHNLHYYQVLMDGMREAIEAGDFAGFERDFHARRAEGDIEPI---MADLF--DTPADGSTDRAPSGAGPRPLADRLRPRRLDAVIGQDHVLGPEGPLGVMLASGSLSSLILWGPPGVGKTTIARLLADETDLAFVQISAIFTGVPELRKVFEAARIRHANGQGTLLFVDEIHRFNKAQQDGFLPHMEDGTILLVGATTENPSFELNAALLSRAQVMVLNRLTPRDLERLAQSAEKELDRPLPLDGPAREALLEMADGDGRALLNLIEQVMAWKVA-GPLDREGLATRLMRRATKYDKSGDEHYNLISALHKSVRGSDPDAALYWLARMMKGGEDPRYLARRITRMAVEDIGLADPQAQAVCLQAWETYERLGSPEGELALGQAIVYLALAPKSNAGYAAFKNAMSAATKTGSEPPPKHILNAPTSLMKEQGYGEGYAYDHDAEAGFSGQNYFPESMKRGVYYTPVERGFERELKRRLDYFAKLRDKRQGG----MPHFLDIHKTDADALHRMLDQARSMKDARRTFPKAVPDEGKPLDGRMIALIFEKPSTRTRVSFDVGVRQLGGQSMVLSGADMQLGHGESIADTARVLSRYVDAIMIRTFEETTLEELAEHATVPVINGLTNRTHPCQIMADLMTYEEHRGPIKGRRVVWAGDGNNVCASFLHAAGQMGFDFTFTGPEVLDPEQVFVDYARSKGSDVRIERDPRVAVEGADLVVTDTWVSMHDSQTARERRHNLLRPFQVDEDLMARAAPDALFMHCLPAHREEEVTSTVMDGPQSVIFDEAENRLHAQKAVLRYCLGVMSKRTILIHPDPRLKTVASPVT-DVNGALISLADDMLETMYAAPGIGLAAPQVGVLTRALVMDCVKEEGAEPRPMVMFNPEVIASSDERNVYEEGCLSIPEQYADVERPAEVTVRWIGRDGEEHQEDFAGLWATCVQHEIDHLDGRLFIDYLKPLKRQMITRRMQKLKRERARS--------------------------------------MRRLRDPQTGCPWDIEQDFASIAPYTIEEAYEVADAIERQSWDELRGELGDLLLQSIYHAQMADEAGLFDFEDVAREIGDKMVARHPHVF-GSDTTPKTAEQQTADWEAVKAAERAGKAETR----TLDGVALGLPALLRALKLQKRAARVGFDWPDTGGVMDKIVEESRELAEAIDSKDTDHIAEEYGDLLFVMANLGRHLGLDPEAALRAANAKFVRRFNAVEDRLQQLGKRPEQSTLEEMDALWDAAKRAERDISD--------------------------MMAQFLEIKSEHQDALLFYRMGDFYEMFFDDAVAAAEALDISLTKRGKHLGDDIPMCGVPVHAAENYLLTLIRKGFRVAVCEQTEDPAEARKRGSKSVVRREVVRLVTPGTLTEDALLDARRHNYLSAWAQV-RDTAALAWVDISTGAFHVMPCPAVRLSPELARLTPREVIVA--EGAQEPLRDIAREMGAALTPLAASAFDSTAAEHRLTDLFKVGTLEAFGRFDRAELAAMGGIVEYLTLTQKGNLPLLRAPVREQARDAVQIDGATRRNLELTQALSGGRAGSLISAIDRTATAGGARLLERRLSSPSRHLETVRARHDAVEWLVENRAIAADLRDTLRKVPDLSRALSRLSLERGGPRDLAAVRNGLEQADRIADSLCRLEMPPVLAEAVQ--SLMGHDALLERLQS---ALVAEPPLLVRDGGFIAPGVDPELDEARQLRDEGRGVIARLQAHYAEETGIQSLKVKHNNVLGYFIEATATHSAKLLAEPLSQRFIHRQTTANAIRFTTVELSELETRILNAGGRAQEIEKRLFSTLCDEVLALAPQLGDAAAGLAEVDHSRALADLAVECDWSRPQMEDSRAFDIRGGRHPVVERALRQQGGAAFIANDCDLSGEDGTGADPARIWLLTGPNMAGKSTFLRQNALIALLAQAGSYIPATSGRIGLVSQIFSRVGASDDLARGRSTFMVEMVETAAILNQADENALVILDEIGRGTATYDGLSIAWATLEHMHDTNRCRALFATHYHELTGLSAKLEGVENATVAVKEWEGEVIFLHEVRRGAADRSYGVQVARLAGLPASVVERARTVLEALEQGEREGGKRKQTLIDDLPLFSAT-PPPPVRPAPPAA-DPLAERLEAVNPDQLTPMEALNLVYEMKALGRDRG-MKDILQELENRRETARMGGGERRIAAQHAKGKLTARERIELLLDEGSFEEFDMFVAHRCTDFGMEQDRPAGDGVVTGWGTINGRQVYVFSQDFTVFGGSLSETHAQKLCKIMDMAMQNGAPVIGLNDSGGARIQEGVASLAGYADVFQRNIMASGVVPQISVIMGPCAGGAVYSPAMTDFIFMVKDSSYMFVTGPDVVKTVTNEVVTAEELGGASTHTRKSSVADGAFENDVEALAEVRRLVDFLPQNNRSKPPVRPFFDDVNRIEDSLDTLIPDNPNAPYDMKELITKLGDEGDFYEIQEEFAKNILTGFIRLEGSTVGVVANQPMVLAGCLDIDSSRKAARFVRFCDAFEIPILTLVDVPGFLPGTGQEYGGVIKHGAKLLFAYGEATVPKVTVITRKAYGGAYDVMASKHLRGDFNYAWPTAEIAVMGAKGATEILYRSELGDAEKIAARTKDYEDRFANPFVAAEKGFIDEVIMPHSTRRRVCRAFASLRNKSLANPWKKHDNIPLMAGHSKWANIQHRKGRQDKLRSKLFSKLSKEITVAAKMGDPDPDKNPRLRLAVKEAKSQSVPKDVIDRAIKKSQGGDAENYDEIRYEGYGPGGVAVIVEAMTDNRNRTASSVRSTFAKNGGNLGETGSVSFMFERKGQVTYPAEVGDEDTVMMAAIEAGAENVESSEDGHVIICADTDLNEVSTALEAELG---ESDSTKLVWMPTTTTELDLDGMQKLMKLIDALEDDDDIQNVTANFEASDEVLSQL--------MIPSVLPTYNRAPLAFVKGEGSWLIEADGTRYLDLGAGIAVNALGHANPELVEALTAQAGALWHVSNLYQIPAQQALADALVAASFADTVFFTNSGTEACELAVKMARKHFHDAGQPERVEILTFEGAFHGRSSAGIAAAGSEKMTKGFGPLLPGFRQLPFGDHDALRAAVTDKTA-AILVEPIQGEGGIRALPDVCLKGMRDLCDEHGLLLIFDEVQCGMGRTGRLFAHEWAG-IAPDIMMVAKGIGGGFPLGAVLATEAAASGMIAGTHGSTYGGNPLACAVGGKVMEIVSDPDFLAKVNRKAGLLRQKLEALVAEHDDIFDGVRGSGLMLGLHCVVENSRVVSAGYAEHVLTVPAADNVLRLLPALNITDEEIAEAVARLDRAASRLKADRAA------MPANTEPKLMSGNANRTLAQSIARRMSMHRGMNIGLVDARIERFNDAEIFVEVYENVRGEDMFIIQPTSNPANDNLMELLVMTDALRRSSAARITGVIPYFGYARQDRRTKARTPITAKLVANMMVEAGIERVLTMDLHAAQIQGFFDIPVDNLYASPIFALDIQHHFKGKLDQVTVVSPDVGGVARARELAKRI-GCALAIVDKRREKPGEVAEMTVIGNVKDQTCIIVDDICDTAGTLCKAAEVLMEAGASQVHSYITHGVLSGPAVERITNSVMESLVITDSIEPTDEVKGAHNIRIVPTAPMFAQAILNIWSGTSVSSLFEHETLAPIYEGLYTGA---MERIVIAGAMRTPMGGFQGDFDGVPAARLGGEAIAAALSGAGVRPEAVDELLMGCVLPAGQGQAPARQAGFSAGLSRHVPATTLNKMCGSGMKAVMMAHDQLALGGASVIVAGGMENMSNAPYILPRMRCGARLGHDKVVDHMFLDGLEDAYESG-----RLMGTFAEDCAGKFAFSREHQDAFALQSLENALEAQRSGAFDDEIVPVTVESRKGAVEVRQDEQPAKARPDRIPHLKPAFRAEGTVTAANSSSISDGAAALVLTPERVAGERGYASRARIVAHASHAQEPGWFTTAPVPAARKVLERAGWQVGDVDLWEVNEAFAVVPMAFMKEMGIPRDRLNVNGGACALGHPIGASGARIIVTLLNAMERRGARRGVAAICIGGGEGTAIAIERD----MSDAES--RIKQTITANDVVLFMKGTKAMPQCGFSSRVAGVLNYMGVDYADVNVLEDDGIRQGIKDFSDWPTIPQLYVKGEFVGGCDIITEMTLSGELDQMFEKSGVTFDKDAADKIREA-NGMTE---TPIEEMSFEQAMRELETVVGKLERGEVELEESIRLYERGARLKKRCDEKLKEAEEKVAAITTDAEGNATGATPVDGL-----------MTIPRKLPE-GPTNLVGLTRDGLAEALRAAGTPERQVKMRTGQVWQWIYEKGVRDFEAMTNLAKPYRTLLAENFVIAVPEVVSRNVSTDGTRKYLVRIAGGHEVEVVYIPETDR-GTLCVSSQVGCTLTCSFCHTGTQKLVRNLTAGEIVGQILVARDDLGEWPVQGA-PK--NETRLLSNVVLMGMGEPLYNFDNVRDAMKIAMDPEGISLSRRRITLSTSGVVPEIARTAAEIGCMLAISFHATTDEVRDKLVPINRKWNIEKLLKALKEYPKASNSERITFEYVMLKDVNDSDEDARRLIRLI--EGIPAKINLIPFNEWPGSPYQRSDWKRIKAFADIIYKAGYASPIRTPRGEDIMAACGQLKSATERARKS---RAEIEADIARG--------------MKAEIFLPEDYRPAEDEPFMNDRQLEYFRRKLNAWKNDLLSESRDTIEGLQDGTRNIPDVTDRASEETDRALELRTRDRQRKLISKIDAALRRIDEGEYGYCDETGEPISLKRLDARPIATLSLEAQERHERREKVHRDD-MTDRLIHVEIDDSNLPAPTPEIEQERRVAIFDLLEANRFSLPGREGREAPPGPYRLNLAIRDRRLVFDLQTEDESKAAEFHLSLGPFRQVVKDYFQICQSYFDAVKKLPTNQIETIDMARRGIHNEGARILQERLEGKAEVDTDTSRRLFTLICVLHFGG----------MSSKDHIT-------VSGAPEGFDAKIVLRELERAGGPVIHVARDDRRLEAMRAALAFFDPNVVALTFPAWDCLPFDRVSPNADVSAARMATLAALAAGF--TGRFVLLTTVNAVTQRLPAREVLRGASFAARVGERIDEEQLRQFLVRMGFTQSPTVTEPGDYAVRGGIIDIYPPGQDGPVRLDLFGDVLDGARRFDPATQMTTEKLDAVELAPVSEVILDDEAIARFRQSYRTEFGAAGTDDPLYEAVSAGRKHQGIEHWLPFFHERLETLFDYLPGAPV-VLDDQTTAIRLARWETIADQYDNRREALVKKARLDSVYKPAPPGLLYLDEGAFDGALGPRRRLDLSVLPQATGPGVIDAGGRIGRSFAPERQQDSINLFEALAAHVKARRASG-PVIIASYSEGARERLQGLLEDEELADTRLISDFRDVPDSDATPGSRKSGGVHLAVWPLEAGFQTD------------GLTVISEQDVLGDRLIRSAKRRKRADNFLTEAQSLSPGDLVVHVDHGVGLYRGLETVTAMGAPHECLLLEYAGGDRLFLPVENVELLSRYGHE--EGLLDKLGGGAWQAKKAKLKERIRQVAERLIRVAAERALRTAPMLEPPEDMWDAFSARFPYQETDDQQNAIMDVVADLASGQPMDRLICGDVGFGKTEVAMRAAFIAAASGMQVAVIAPTTLLARQHAKSFSDRFRGFPMEVRQLSRFVSAKDAADTRDGISRGTVDIAIGTHALLAKGVRFKNLGLLIIDEEQHFGVQHKERLKEMRSDIHVLTLTATPIPRTLQLSLSGVRDLSIIGTPPVDRLSIRTYVSEFDTVTIREALLRERYRGGQSFFVVPRLTDLPEIEAFLTEQVPEVSYVVAHGQLAAGDLDDRMNAFYDGQYDVLLATTIVESGLDIPTANTMIVHRADMFGLAQLYQIRGRVGRSKTRAYAYLTTKPRAKLTATAEKRLRVLGSLDTLGAGFTLASQDLDIRGAGNLLGEEQSGHVRDVGYELYQQMLEEAIAKIKAGEGEGLSQTDD-QWAPQINLGVPVLIPEAYVPDLDVRLGLYRRLSSLATKVELEGFAAEMIDRFGPLPREVNTLLLIVRIKAMCKRAGIAKLDGGPKGVTIQFHNDHFPNPQGLVEFVHDQKGQAKVRDNKIVVMRDWTKDAEK-IKGAFAIARDL-----------AQKAAPAKPSGKTASKSVARGGKA-------MLDLSYESPAPKVIAGAKHDWELVIGLEVHAQIASKAKLFSGASTQFGAEPNSNVAFVDAAMPGMLPVINEFCVEQAVRTGLGLKAQINLRSAFDRKNYFYPDLPQGYQISQLYHPIVGEGEVLVNMA----PGIARRVRIERIHMEQDAGKSIHDMDPTMSFVDLNRTGVALMEIVSRPDIRGPEEAAAYVVKLRQILRYLGTCDGNMQNGNLRADVNVSVCRPGDYEKYQETQDFSHLGTRCEIKNMNSMRFIQQAIDYEARRQIAILEDGGSVVQETRLYDPDKGETRSMRSKEEAHDYRYFPCPDLLPLEIEQGWVDDIQANLPELPDEKRARFVTEYGVTEYDADVLTADVESGHYFDEVAK-----------------------------GRD-GKLAANWVINELFGRLKKE-DHGIADSPVSAAQLGGILDLIAKGDISGKIAKDLFEIVYTEGGDPAQIVEDRGMRQVTDTGAIEAAVDKIIAENPAQVEKAKQNPKLAGWFVGQVMKATGGKANPKAVNEIVAAKLAG---------MAELMKGKRGLIMGVANERSIAWGIARQMAAEGAELAFSYQGEAFGKRVEPLAASVGSDILVDVDVTDDASLDAAFETLGTHWDRLDFVVHAIAFSDKAELTGRFI-NTSRENFKNSLAISCYSLIDVARRARPMMSE-GGTILTLTYQGSNRVTPFYNVMGVAKAALESTVRYLANDLGPEGIRVNAVSPGPMKTLAGAAIGGARKTYRQTEMNAPLRSNATLEAVGGTAVWLASDYGACTTGEIVRVDGGYHVLGMNQPENI-MSVADFA--FYLFAITVITGGLMTVLARNPVHSVLWLILAFLSSAGLFVLLGAEFVAMLLVIVYVGAVAVLFLFVVMMLDVDFAELKAEMARYMPLGLLIGVVLLMELGLVFGSWTFADQAQALRGAVTPDLNEVHNTAALGLLVYTKYIYLFQAAGLILLVAMVGAIVLTLRHRTNVKRQNILAQIYRDPAEAMELRDVKPGQGLQMSFFKKLKDRMLKSSSRLEAGLDAIVEEGQE-------------------------------------------------------------------------PSGAEVPQA-----------------------------------------------------------------------------PDAAPPS-----------DAAPAPA---P--VPP-ADP---AESPAT-APSGAPVPAAPQTPEAQ----T----------------------------------PVSPEPESKGGLIDRLM-GR---------GG-----------------QEVARRTLDDPMLEQLEELLITADMGVDTALRVTANIAESHFGKRVSAEEVKRLMAEEIARIMEPVAQPMPIYSKK-PQVVLVVGVNGSGKTTTIGKLASQFRAVGKSVIIAAGDTFRAAAVEQLQVWGERAGVPVLTAPEGSDPASLAFDAMLKAEAEGADLLLIDTAGRLQNRADLMEELAKIVRVIRKRDPEAPHNTLLVLDATTGQNALSQVETFRKLADVSGLVMTKLDGTARGGVLVALADRFGLPIHAIGVGEQIDDLAPFDPEDYACALTGLEV----MKISIERGTLLKAVAQAQSVVERRNTIPILANVLIEADANSVSFRATDLDIEVVDKTVAHVERPGATTVSAVTLHEIVRKLPDGALVQLTDDGTSGRLTVEAGRSNFALATLPKEDFPVMASSEYQSNFSAPAPVLRRLFDKSKFAISTEETRYYLNGVYMHVAEA-EDGRMLRCVATDGHRLARIDTPLPEGAADMPGVIVPRKTVGELRKLLDDDDAEIAVSVSETKVRFATPEITLTSKVIDGTFPDYTRVIPMGNTRRLEVDAQEFAKAVDRVATVSSERSRAVKLSLDEDRLILSVNAPDSGAAEEELAVAYADEKLEIGFNAKYLLEIASQVDRENAVFMFNSSGDPTLMREGNDSSAVYVVMPMRV---MAMTGVLPSQALRQMIEAGEIRAEEPLTTAQIQPASIDLRLGRIAYRVRASFLAGKGRTVAERLSE--FEMHRVDLDE-GAVLEKGCVYVVPLMESLDLPAEVQAVANAKSSTGRLDLLTRTITDEGVEFDRIPAGYSGPLYAEICPRSFSVLVRPGQTLNQIRFRAGQAILDDDELTRLHEQTTLVSGPAH-IDAGLGFSVDLEPA-SGSLVGFRAKPHTGVIDLSLIDHYDPTEFWEEVRSTEGRIILDPGAFYILVSRESVHIPPDYAAEMAPYLAMVGEFRVHYAGFFDPGFGHSEAGGTGSRGVLEVRCHEAPFVLEHGQIVGRLVYERMSARPEEVYGAGIASNYQGQGLKLSKHFKPFQPG-----------------MAQIDSLFVTRLYRAALAEHGKPVDPGELEAACLSIAEDDEAGQDWCEENGFPGYTSYASLTDLPWRFPIFADLVKVLDKHVASFAKDLEFDLADKKIKLDSLWINILPEGGIHTSHLHPHSVISGTTYVAMPTDTSAIKFEDPRLAMMMAAPSRTKGARAELRNFIYVAPEVGEVLLWESWLRHEVPMNMAEDD-RISVSFNYGWE-------------------------MPRLVMKFGGTSVGTLDRIRRAAKRVGVEVAKGYEVIVIVSAMSGKTNELVGWVEETAP------LFDAREYDAVVSSGENVTAGLMALTLQEMDVPARSWQGWQVPLRTTSAHSSARIVDIPTDNLDAKFAEGMKVAVVAGFQGISDEGRITTLGRGGSDTTAVAFAAAFNAERCDIYTDVDGVYTTDPRISSKARKLDRIAFEEMLELASLGAKVLQTRSVELAMRYNVKLRVLSSFGEQSDDA-GTLVCDEEDIVESNVVSGVAYSRDEAKMTLISVADQPGIAAAIFGPLSEAGVNVDMIVQNIS-E-------EGR-TDMTFSCPVSEVKRAEKAMMEAKEAGAIDFQELVADTDVCKISVVGIGMRSHAGVASKMFKALSSEGINIKVITTSEIKISVLIDRKYMELAVQALHDAFELEKAA-MFETLSERLSGVFDKLGKQGALSDEDVKTALREVRVALLEADVSLPVARDFVAAVQEKATGQAVTRSVTPGQQVVKIVHDELVHVLAGDGDTAGQLKIDNPPAPILMVGLQGSGKTTTTAKLAKRLKEKSGKRVLMASLDVNRPAAMEQLAVLGVQVG--VDTLPIVKGQTPVEIARRAKQQATMGGYDVYMLDTAGRLSIDEALMAEVEAVRDVTSPRETMLVVDGLTGQDAVHTAENFDARIGISGVVLTRMDGDGRGGAALSMRAVTGKPIKFVGLGEKLDALEEFHPERVAGRILGMGDIVSLVEKAQETIEAEQAERMMKRFQKGQFNMNDLKSQIEQMQKLGGMEGVMGMLPGMKKMSKQVEAAGMDDTLLKRQIALIQSMTKKERANPQLLQASRKKRIAKGAGLEVSELNKLLKMHRQMADMMKKMGKMGKKGMMRGALGQMF-GK----GGP---------SQADIEAAKAQMGGAGGQMP-AGLPGM----GGGLGGAA----LPPGLSGF---GKKKMSLDEIRTRLRAAESA-----AGRPEGSVELIAVSKVQPLPRIEAVLEAGHRLFGENRVQEAAGKWPELRARHEGVAVHLLGPLQTNKARQALDLFEAIHSLDRPKLARTLARLAQEEGRCPELFVQVNTGEEEQKAGILPAEADAFIAECR-AMDLPVRGLMCIPPVEEEAALHFALLAKIAARNGLEGLSMGMSADFEKAVAFGATHVRVGSAIFGARTTEG---------MAYHPKSEFLRIMIERGFLADCTDYQGLDEALLEGGMPGYIGFDATARSLHVGSLIQIMMLRWMQKCGQKPIVLMGGGTTKVGDPSFRADERPLLTPAQIDDNIAGIRQVFSRYLDFG-DAP-----------TGALMLNNAEWLEDLNYLEFLRDIGRHFSVNRMLAFESVKSRLDREQSLSFLEFNYMILQAYDFLELNRRYGCRLQMGGSDQWGNIVNGIDLTRRVLDHEIYGLTSPLLTTSDGKKMGKTADGAIWLNADMRAPYEFWQFWRNTNDADVGRFLKLYTELPVEECDRLGALEGSEINAAKIRLANEVTTLAHGPEAAAAAEATAREVFEKGGVGD----------DLPTVTLAPGDFPIDGH----------------SISAQQMFVAAGLTKSGKDTKRLFAEGGARIDDAPADDPARLFTAADL-ARPVKLSAGRKRHALVTLADMTHIIDIHAREILDSRGNPTIEVDVLLEDGTMGRAAVPSGASTGAHEAVEMRDGDASRYMGKGVLKAVAAVNGEIAEALVGFDATEQTAIDMAMIELDGTPNKGRLGANAILGVSMAVAKAAADMTTQPLYRYIGGTAARVLPVPMMNIINGGEHADNPIDIQEFMIMPVSASSIAEAVRMGAEVFHTLKKELSAAGLSTGIGDEGGFAPNLSSTRDALDFILKSIQKAGYTPGEDIYLALDCASTEYFRDGRYEMTGEGKSLTPDENVAWLEALVNDYPIISIEDGCAEDDWDGWASLTRALGDRVQLVGDDLFVTNPERLSRGIEERAGNSLLVKVNQIGTLTETLAAVDMAHRARMTCVMSHRSGETEDATIADLAVATNCGQIKTGSLARSDRLAKYNQLIRIEEALGETALYAGRSILRRGMSRSVWKGPFVDSYVLKKAEKTKESGRNEVIKIWSRRSTILPQFVGLTFGVYNGQKHIPVNVTEDMIGQKFGEYSPTRTYYGHAADKKSKRKMSELRKIIIDDREVEVDPAMTLIQACEQ-VGIEIPRFCYHERLSIAGNCRMCLVEVVGGPPKPAASCAMQVRDLRPGPEGQPPVVKTNSPMVKKAREGVMEFLLINHPLDCPICDQGGECDLQDQAMAYGVDFSRYREPKRAVDDLDLGPLVETHMTRCISCTRCVRFTTEVAGITQMGQTGRGEDAEITSYLNQTLNSNLQGNIIDLCPVGALVSKPYAFTARPWELSKTESIDVMDALGSAIRVDTKGREVMRILPVNHDAVNEEWISDKTRFVFDGLRRQRLDRPYIRENGKLRPASWPEALGAAANALR-A-GKVAGVVGDLAPVEAIFALKQLVEGLG-GSVECRTDGAAL-PAGQRGAYVGNAAIEDIDSAEMIQLIGTDPRTEAPVLNARLRRAWIRGA-DIGLVGPAVDLTYDYAHLGDDRAALDKLCDRQVSDETR--AKRTLVIVGQGALTGEDGAAVLERVMRLVRNSD------SKMMILHSAASRVGAMDVGAV-TAGGLDAA---LDGAATVFNLGADEIDVPA--GPFVIYQGSHGDRGAHRADVILPAAAYTEEPGLFVNTEGRPQLAQRASFAPGDAKENWAILRALSAEAGQTLPYDSLAQLRQALIAA------------VPHLGDVDQLADNQLPADLPA-GDLGQGAFGGAVRDFYLSNPIARASALMAELSAGAKARANSPMAAEMAEIKDPENTVIVELKDGRVVIELLADVAPKHAERMKELARAGAYDNVAFHRVIDGFMAQTGDVQNGNME-KDFNIRMAGTGGSDLPNLPAEFSKLPHDRGTLGAARSANPDSANSQFFINFSDNHFLNGQYTVYGRVIEGMEHVDKIARGEPPASPDRMISVKVAADA-MLDRPTNRPDLPEEIARRRTFAIISHPDAGKTTLTEKFLLYGGAIQMAGQVRAKGEARRTRSDFMQMEKDRGISVSASAMSFDY----SGYRYNLVDTPGHSDFSEDTYRTLTAVDAAVMVIDGAKGVESQTQKLFEVCRLRDLPILTFCNKMDRESRDVFEIIDEIQENLAIDVTPASWPIGMGREFIGCYDLLRDRLELMDR--ADRNKVAASIEISGLDDPKLEQHVPANLLEKFREELEMARELLPPMDPQALRDGTLTPIWFGSAINSFGVKELMEGIAGFGPEPQIQNAEPR----KIAPEEPKVTGFVFKVQANMDPKHRDRVAFVRMCSGHFKRGMKLTHVRSKKPMAVSNPVLFLASDRELAEEAWAGDIIGIPNHGQLRIGDALTEGETLRFTGIPSFAPELLQTVRAGDPMKAKHLEKALMQFAEEGAAKVFKPAIGSGFIVGVVGALQFEVLASRIELEYGLPVRFEPSQFTSARWVTGPDK-EMDRFINVNKGHIAHDNDGAVVFLTRLQWDIDRVVRDYPELTLSATKELMV---MKQSDNPADPFKKALAEATKVMADDPDLTVSYSVDPPSLVNDTVRLPQVSRRMSRDEVMLARGTADAYALRHKFHDEATHNRYVPRGTMARDIYEAMETARCEAMGARVMPGTAGNIDAKVADEAARKGYAQITQASDAPLAVAAGYLVRHLATGRPLPPGAQNVMELWRGFIEEQAAPTLENLDEALSDQRAFARFARQVIDDLGYGDQLGEDPDAADDDQEDEGQSEEDED--DAESSGQENQE-DDEQDADAAPEQ---SQDQQQDAAQAQLSMDDSDPSDDAEAAELPD-GEAPMEPPAPAPPSEADPHYQVFHAEFDEEIRAEDLAEPAELERLRAYLDQQLEPLKGSVSRLANKLQRRLQAQQNRSWEFDLEEGTLDAGRLARVVANPTTPLSFKTERDTEFRDTVVTLLLDNSGSMRGRPISIAAICADVLARTLERCQVKVEILGFTTRAWKGGQSREKWLAEGRPQQPGRLNDLRHIIYKRADAPWRRARPNLGLMMKEGLLKENVDGEALEWAHRRMVGRPEARKILMVISDGAPVDDSTLSVNPANYLEKHLRDVIAMVEKRKAVELIAIGIGHDVTRYYGKAVTITDVEQLAGAMTEQLASLFDRD-PRGKARLK-----------------GI----RNA------------------------------------------MAPKEWEALCDGCGKCCLNKLEDPDTAEIAFTDVACRLLDDETCRCAQYEIRKQFVPDCVVLTPANIAEIAYWMPQTCAYRLLFEGKALYDWHPLISGTAETVHEAGISMRGRTVSEFDVDEEEWEDHIIE----EPVMSELADIHLLVVDDDERIRTLIQKFLIRHGFCVSTARDAAHARRILAGLEFDLIVLDVMMPGEDGISFTAALRE--SVTTPILLLTAKGETNDRIAGLEAGADDYLTKPFEPRELLLRINAILRRMPQNPPEDDGPRVLHLGPLRYDVDRGELWHGTDPVRLTATESQLMRIFSGRVGEPIGRDKLVEDLGRDRGQAQERAIDVQITRLRRKLEADPRQPRYLQTVRGAGYMLAPDMGSTTLTRMDLSEAVFREVGLSRNESAQLVESILQHMSDALVKGETVKISSFGTFSIREKAARVGRNPKTGEEVPIHPRRVLTFRPSYLMKDRVAAGNRGMTSQ--TTEKLEGA----PLIR-PSSTDHPLYETVVEACRSVYDPEIPVNIYDLGLVYTIDINGENDVSILMTLTAPGCPVAGEMPGWVAEAVEPLPGVRTVAVELTWEPPWGMEMMSDEARLELGFMMAHVKN-HDYHILNPSVWPFIGAVSGFAMLFGAVLWMHD----------NGPWLFFIGLAGVLYTMFSWWSDVVEESMVGDHTPVVRIGLRYGFIFFIMSEVMFFAAWFWSFFKHAMYPMAT-----FSEGQW-----------------PPPSIETFDPWHLPLINTLILLCSGAAATWAHHALVHDNNRRDLKMGLIIAIVLGLVFTVFQAYEY----MHASFGFAGNIYGANFFMATGFHGAHVIIGTIFLFICLLRVYRGHFTPEKHIGFEAAAWYWHFVDVVWLFLFAAVYI--W---GG------------------MLAPFACRPEETRGRLHPEPEAAFRSPFQRDRDRIIHASAFRRLKHKTQVFVEHEGDYYRTRLTHSIEVAQVARTIAGVLKLNAELTEAVALAHDLGHTPFGHTGEEALDALMQGHGGFDHNAQALKIVTSLERHYAEFDGLNLTWETLEGIAKHNGPV-----TGPRARHKDRPLPFALSEYNARHDLELDSFASAEAQVAALADDVAYNNHDLHDGLRAKLFSLAELAELPVLDRCFAEVDAAYPGLNSYRRQHEALRRFFGVLVGDVLDVSRARLERLDPRSAEDVRRADGPVIRFSDPLWRDLKEIREFLFQRMYRAPTVVEMRRRVTEVVRALFALYMASPQELPKQWRKDV-AAASGQTELARIVADYIAGMTDRFAFQEHERLC-GPAPR-----------MEIKSIGIVGAGQMGNGIAHVCALAGYDVLMTDITQEALDSALALIRSNLERQASRGKIEEDEVSAALDRIRCTLKLSDLGQTDLVIEAATERETVKQAIFEDLLPHLKPETILTSNTSSISITRLASRTDRPEKFMGFHFMNPVPVMQLVELIRGIATDEATFKACLQVVERLNKSAATAEDFPAFIVNRILMPMINEAIYTLYEGVGNVKSIDTSMKLGANHPMGPLELADFIGLDTCLAIMNVLHDGLADTKYRPCPLLTKYVEAGWLGRKTRRGFYDYRGEGDPVPTRMAKRDFYDVLGVSRGASEAEIKKAYRTKAKELHPDRNADKPEAESQFKEVNEAYDALKDPSKKAAYDRYGHAAFE-GGNGASAGHGRGGFHPGGG--DFASAFSDVFDDLFGDFVGGG-PRGRGGQPRAARGSDLRYNLRVTLEDAYAGVQKSINVPTSVRCGTCEGSGAEGGAVPEACPTCSGMGKVRAQQGFFTVERTCPTCGGIGQIIKNPCKSCGGAGRVEKERALSVNIPAGVETGTRIRLAGEGEAGLRGGPAGDLYIFIEVAEHTLFERDGENLFCRVPVSLTAAALGGDIEVPTIDGGRSRVKVPAGSQSGRQMRLRGKGMPRLRGGG-SGDMFIEMAVETPVNLTSKQKDLLREF-ERLSEDNNPHGSSFFDSVKSFWDGMKG-----MDKFEKITGVAAPM-----PLINVDTDMIIPKQYLKTIARTGLGKSLFDEMRYDDDGNEIESFVLNQPAYRNAEILVAGDNFGCGSSREHAPWALKDFGISCVIAPSFADIFYSNCFKNGILPVILPQEQVDVLMKDAEKGANARMTVDLENQTITTSEGESFGFEIDAFKKHCLLNGLDDIALTLEKGAAIDAFEANVNQQRPWV------MAALSDARRLVVKIGSALLVERGSRALRREWLEGLAADVAELRAAGTSVVLVSSGSIALGRGVLNLPEGPLALEQSQAAAAVGQIGLARAYEEALQPYGFPTAQVLLTLDDSANRRRYLNIRATLQQLLKLGAIPIVNENDTVATDEIRFGDNDRLAAQVALTVGADQLVLLSDVDGLYTADPTRDPDARHLPQVDAITPEIEAMA--GDPVSGLSKGGMKTKVMAAKTATAGGCAMAIAEGRVLRPLSALRDGARATWFLPEQDPTTARKGWI-AAMKTRGAIGVDAGAAAALGQGKSLLPAGVTGVEGRFHRGDLVEIRSPEGLVLGVGLTRYDTDEARRIIGLRSEEIEDVLGYPGRAALIHRDDMVL-------MPEVIFPGPEGRLEGRYHAQKEKDAPIAIILHPHPQFGGTMNNRVVYNLHYTFLNMGFTVLRFNFRGVGRSQGEYDQGIGELSDAASALDYLQSMNQNAKHCWVAGFSFGAWIGMQLLMRRPEITGFVSVSPPANMYDFSFLAPCPSSGLVINGSADRVAPPADTRNLVEKLHDQKGITITHEEVEGAGHFFEDPHMDGMLETVRSYVSRRLTESTR----------------------------MSVIYQRVALVGLGLIASSMAHAMRRAG-LAGHIAGTARSAETRQIALEIGLCDSVAETAAEAVRDADLVVLAVPVGAMGTLASEIAPHLKPGATLTDVGSVKQAVIDAVGPHVPEGVHFVPAHPLAGTEHSGPRAGFAELFENRWCLLVPAEGSDEA---AVERLAELWHAMGSRVDRMDAAHHDLVLAVTSHAPHLIAYTMVGVADDLGRVTDSEVIQYSAAGFRDFTRIAASDPTMWRDVFLTNRDATLEILGRFTEELFALQRAIRTGDGEMLHDYFTRTRAIRRGIIEAGQDTDAPDFGRLPKPGKD----MNLPPKVT--DRAFERLAEINGTAEQPQA-LRVAVEGGGCSGFQYDIRLDQPAE-DDLVLEKNGQKVIVDTVSLPFLADAVIDFSEELIGARFVIENPNATSSCGCGTSFSI----MQSTEIEIHVHPSLGDIPAAEWDACACPEVVQSDGPDPDKAARPVDPFTTHRFLLALERSGSVGRGTGWQPRYLVAR-SEGRVIACAPLYAKSHSQGEYVFDHSWAHAFERAGGDYYPKLQLAVPFTPATGRRFLTAPGWHEAGM-AALVQGMVQIAADNDLSSAHATFCTPQEAAAGEAMGLMHRTGQQFHWQNREYADFDAFLATLSSRKRRNIRKEREGAARFGGRIVRLTGDEIEPEHWDAFWRFYQDTGARKWGHPYLTRAFFDEVQETMRRDVLLVLALRGDEPVAGALNFIGRDTLYGRYWGCVEDHPFLHFELCYYQAMDHAIAHGLARVEAGAQGEHKLARGYLPVATHSLHWIADAGFRRAVAQFLEAEREAVEDEIEVLTSYGPFR-KTTATERE----------MSGLTHFDTKGDAHMVDVSDKEATDRTAVAEGAVRMSPETLAIITEGRAKKGDVLAVARLAGIMGAKRTSDLIPLCHPLAITKVAVELVPDSDLPGVRITATVRTTGQTGVEMEALTAVSVAGLTLYDMVKAVEKTMVIENVRLLSKDGGKSGRFTASGFAAPDAEMKTPADRPDAETPEARLLELSRAHGIRLACAESCTGGLVAARLTRLAGSSDMFDRGIVTYSDDAKMSLLGVRKATLDAVGAVSEEVAREMVEGLRAGAKAGLAVSITGIAGPGGSEH-KPEGRVCFGLAFAESSGLATRTETVEFGALGRENVRQASCEHALQLLIAGFAELFHD----------------------MLRIDDRITIEDWELTEQFTRASGPGGQNVNKVSTAVELRFEAARSPNLPEPVKTRLRRLAGRRWTTDGAVVIRAESHRSQARNREDARNRLADLIRMATVKPKRRIPTRPTKGSERRRLAAKAQRGTVKSLRGKVTGEE------------------MPSFSNTLEQAIHSALAEANQRRHELATLEHLLLALVDEPDAAKVMQACGVELEILRKSLIDFIEDDLSSLVTDVDGSEAVPTAAFQRVIQRAAIHVQSSGRSEVTGANVLVAIFAERESNAAFFLQEQDMTRYDAVNFIAHGVAKDPSYGEQRPVQGAAEFGEE--QTAG-AEPEDDGKESALAKYCVDLNAKARKGDVDPLIGRDQEVERCVQVLCRRRKNNPLLVGDPGVGKTAIAEGLARKIVDGETPEILSDATIYSLDMGALLAGTRYRGDFEERLKAVVTELEEHPDAVLFIDEIHTVIGAGATSGGAMDASNLLKPALQGGKLRCMGSTTYKEFRQHFEKDRALSRRFQKIDVNEPSVDDTIKILKGLKPYFEEHHDIKYTTDAIKTAVELSSRYIHDRKLPDKAIDVIDEAGAAQHLVAESKRRKTIGAKEIEAVVAKIARIPPKSVSKDDAEILKDLEKTLKRVVFGQDNAIEALSSAIKLSRAGLREPEKPIGNYLFAGPTGVGKTEVAKQLADTLGVELLRFDMSEYMEKHAVSRLIGAPPGYVGFDQGGLLTDGVDQHPHCVLLLDEIEKAHPDVYNILLQVMDHGKLTDHNGRQVDFRNVVLIMTSNAGAAELAKSAIGFGRDRREGEDTAAIERTFTPEFRNRLDAVISFAPLGREVILQVVEKFVLQLEAQLMDRNVSIELTKPAAEWLAERGYDDRMGARPLGRVIQEHIKKPLAEELLFGKLAKGGVVKVGVKNDKIDLRI----EAPDKPRLA-S-KKPPLLTAE-MTVASANLPL------AGVAGAESMPHNIEAEQQLLGAILTNNDLYDRIASIVGAEHFHEPVHARIFEIAAARIQKNALASPVTLKPFLEEDPGLGELGGVAYLARLAGAAISAFAARDYAQMIYDLAIRRELIRLGGDISAKAREMEVASEPKDQIVEAEQALYKLGEQGKTDSGFQSFLKAVTDAVNVANAAYQREGGLAGVSTGLIDMDKKLGGLHRSDLLILAGRPSMGKTSLATNIAFNIAKAYKRGRTPDGSDGTVDGGVVGFYSLEMSAEQLAARILSEAAEVPSEQIRRGDMTETEFRRFVEAAKSLESCPLYIDDTPALPISQLAARARRLKRTHGLDVLIIDYLQL--VRPATAKD-SRVNEVSEITQGLKAIAKELDIPVIALSQLSRQVENREDKRPQLSDLRESGSIEQDADVVMFVFREEYYKEREKPGDHDLE-----AMATWQTEMERLHGKAEVIIGKQRHGPIGTVELSFEGQFTRFGNLVRPWQNNEGGGGPDVGF--MAKRWYSVSVLSNFEKKIAEQIRHDVAAAGLEDEIEEVLVPTEEVIEVRRGKKVTAERRFMPGYVLVRMEMSDRGYHLINSINRVTGFLGPQG--KPMPMRDAEVNQILNRVEE-GQEAPRQLISFEVGEKVKVNGGPFEGFDGMVEEVDDDNQRLKVSVSIFGRATPVELEYTEVSKEI-MRRFSGDTLLIATHNQGKLEEIRRLLAPYDVNVTSAAEHDLPEPEETEDSFVGNARIKAHAAARATGLPALSDDSGIEIEALGGKPGVYTADWAETPEGRDFEMAMRRTWDELERIAAPFPRRARFCCTLVLAWPDGHDEVFPGKMDGQIVWPMRGEQGHGYDPIFQPDGFDQTFGEMERWQKNRISHRADAFAQLVAGCFANPDDMSGIPHDHYEPSTSGGRWLHKRLPIVGLLYDTLM-IPTPKNLNWMWIWGIVLAFCLVLQIATGIVLVMHYTPHVDMAFASIEHIMRNVNGGYMLRYLHMNGASLFFVAVYAHIFRGLYYGSYKAPREITWIIGMLIYLLMMGTAFMGYVLPWGQMSFWGATVITGLFGAIPLIGEPIQTWLLGGP--AVGNPTLNRFFSLHYLLPFIIAGLVIVHIWAFHTTGNNNPTGVEVRRGSRAEAERDTVPFWPYFVIKDLFALVVILVVFFAIVGFMPNYLGHPDNYIEANPLATPAHIVPEWYFLPFYAILRAFTSDVWVVQIASFVTGGVIDAKFFGVLAMFGAIAVMALVPWLDTSSVRSGRYRPMFKWWFALLVVDFVVLMWLGAMPAEE------------------------------------------------------------PYATFSLIASAYWFAYFLVILPLLGVIEKPLARPETIEEDFEAHYGP-VDDALN--AGSTPAE--MILKKYLLTAAALAALPALAPTVALAQE-----------AAAE-ATAPAAVPTDVVFILNSVLFLVGGFLVFWMAAGFAMLEAGLVRGKNVTMQLTKNVVLFSLASIFYWLIGYNLMYPLG-----------DWAIEGWLSGLFPSWGVLEAVGVTADKVDDYSYASTGSDFFFQLMFAAATASIVSGTLAERIKLWPFLLFTIVLTSIIYPLQASWKWGGGWL--DAMGFQDFAGSTVVHSVGGWAALTGAIILGPRLGKY-KDGRTVPMPGSNLALATLGTFILWLGWFGFNGGSQLAMGSIGDVADISRIFANTNAAAAGGAVAAVILTQLLYK-KPDLTMILNGALAGLVSITAEPLNPSLGLATVIGMAGGTLVVFAVPMLDKLRIDDVVGAIPVHLVAGIWGTLVVPL---SNPDA-----------------------SFMVQITGIVSVGVFVTVCSAVLWLVLKAVTGIRVSEEAEINGLDMAELGMEAYPEFSKG---MTVTRFAPSPTGLLHVGNLRTALFNWLIARKAGGTFILRLDDTDPDRSKDEYAEAIQEDLTWLGLGWDRIERQSARLDRYAEAADKMRASGRLYEAFETPTELDLKRKKQLNMGKPPVYDRAALRLSDAEKDALRAER-SGHWRFKLDHERIEWTDGILGDISIDAASVSDPVLIRGDGQVLYTIASVVDDTEMGITHVVRGSDHVTNTATQIQIINALG-GTVPEFAHHSLLTGPQGEALSKRLGTLSLRDLRARGVEPMALLSLMARLGSSQPVELQPDLDAIVEGFDLSHFGAAPTKFDEADLDPLTARHLQSLPLEAVREDVAAAGVPDALAEDFWDAVRENVGTRAEIAPWWTMFRDGAEPVIEEDDAAFVAEAMEMLPKGPLTRESWGEWTDAVKERTGRKGRNLFRPLRLALTGQERGPDIAKVLPLL---QVIRARG--------------------MSDHDSYSDAISPIEEILDDARNGRMFILVDHEDRENEGDLVIPAQMATPEAINFMATHGRGLICLSLPGERIDALGLPLMASYNSSRHETAFTISIEAREGVTTGISAYDRARTVAVAIDPAKAAADIATPGHIFPLRARDGGVLVRAGHTEAAVDVARLAGLNPSGVICEIMNDDGSMARLPDLVSFAQLHNLKIGTISDLIAYRRRNDNLVKVSTERTVTSEF-GGEWQMRVFTDETHGDEHIALVKGDLSGDDPVLVRMHALDAMHDVIG-VGPAGRSQEFGRAMEIIAQEGR-GALVLLRD--T-----TLKLTTDGAASPQT-LRQYGLGAQILSNLGLSRLVLLTNSPTPRVVGLDAYGLSIEGTRPIPLDPAATKDMSTEVRVPTLGESVTEATVATWFKKKGDSVAVDEMLCELETDKVTVEVPSPAAGVLTEIVAAEGDTVGVDALLAAIAESGEK-GASP--APAAAEKPADSAPKAKADAAPAPSGG--------AGGSIDVMVPTLGESVTEATVSSWFKKPGDQVAQDEMLCELETDKVSVEVPAPAAGILSEILAAEGSTVEAGGKLAILTGADGA-------AA--AASTAGQTAPA-PAAQPTGTASNDATARG----DVEDAPSARKLMAEHGLSPEQVQGSGRDGRIMKEDVQRAVSEGARKPAPAASAP-APAPQAP-RAPVAAEDSAREERVKMTRLRQTIARRLKDSQNTAAMLTTYNEVDMTSVMELRNEYKDLFLKKHGVKLGFMSFFVKACVHALAEVPDVNAEIDGNEVIYKNYVHMGVAVGTPNGLVVPVVRNADQMSFAAIEKKIAELGVRARDGKLSMAEMQGGSFTISNGGVYGSLMSSPILNPPQSGILGMHKIQDRPVALGGQVVIRPMMYLALSYDHRIVDGKGAVTFLVRVKEALEDPRRLLMDLMTDTS------ILPPQSGEAGFGDYFALLKPRVMSLVVFTALVGLLVAP----VPVHPMVAFASVLFIAIGAGASGALNMWWDADIDQIMKRTKGRPVPSGRVTPGEALQIGMWLSCFAVVMLLLSANPVAAGLLAFTIFFYAVVYTMWLKRWTPQNIVIGGAAGAFPPMIGWAVATGG-VSVESVLMFALIFMWTPPHFWALALFMKSDYADAGVPMLTVTHGRRETRRQILIYTLILVPVALGTGLTSIGGPLYMAVALVLNLAFLKGAVDIW--RRKEEEAEADGYAVEKKVFRLSLYYLFLHFGALLAQ--AVLRVYGIG-----GW----MAEDIKTLDGLRDAVTDDIGGVQ-GTA--DMINREPQRDELGRSYATGKRKDAVARVWIKPGSGKVTVNGKPINAYFARPVLQMILKQPFQVAGVEGEFDVTATVKGGGLSGQAGAVKHGISKALQLYEPSLRGALKAAGFLTRDSRVVERKKYGRAKARKSFQFSKRMLKITDLHVQLEEED--KKILKGVNLTVETGKVHAIMGPNGSGKSTLSYVLSGREGYEVTQGSAELDGEEILEMDPEERAAAGLFLAFQYPVEIPGVGNMTFLRTAVNAQRKARGQEEMSATDFLKEVRAKAKSLKIDADMLKRPVNVGFSGGEKKRNEILQMAMLEPRMCILDETDSGLDVDAMKLVADGVNALRDEGRSFLVITHYQRLLDHIKPDVVHIMADGRIIKTGGPELALEVENNGYADLLEGTA----MIGRLNHVAIAVPDLEAAAAQYRGVLGA-EVGPPQAEPDHGVTVVFITLPNTKIELLHPLGEGSPIAGFLEKNPAGGIHHICYEVEDIRAAAARLTESGARVLGSGEPKIGAHGKPVLFLHPKDFNGTLVELEEV-----------------------------MAHIIVSGNEKGGSGKSTMSMHVATALARMGFRVGAMDLDLRQRTFGRYIENRVANAQRHDLDFASPELRELPEIDQASLAPGENIYDVRLSTAVAEMEEAYDFIVIDCPGSHTRLSQVAHSLADTLITPLNDSFVDFDLLARIDPDNGNILGPSVYSEMVWSARQLRNQAGLPPIDWIVLRNRLGTQYMHNKKKVGDALESLSKRIGFRVAPGFSERVIFRELFPRGLTLLDLKDIGIQ-QLNMSNIAARQELRDLMIALNLP---GVTVEFMSFT-SHMHRALAEARAAADRAEVPVGAVVVDPA-GRIVAAAGNRTRMNSDPTAHAEILALRAACAAAGSERLPGHDLYVTLEPCPMCAAAISFARIRRLYYGAADPKSGGVARGPRIFEHPQAHHVPE-VYGGIAEAEAEALLTDFFRARRGADASGGDI-MPSSLTVTAESFRLSETFTISRGSRDRAEVLCVRIAADGFEGRGECVPYARNGESLASVTAQIAA----LDPVPDRRSLQEMLSPGAARNAVDCALWDLEAKRTGRRAWELAGLEAPGPVQTAFTLSLAAPDAMEAAARRHAHRPLLKVKLGTPDDMPRLEAVRRGAPRARIIVDANEGWSAGIYADLAPHLARLGVALVEQPLPAGQDAELDGLARPVPLCADESCHTRAGLAELSGRYDMVNIKLDKTGGLTEALALRVEARAAGFGIMVGCMVGTSLAMAPATLLAQGAELTDLDGPLLLAEDRNRPLHYDQAGVHPPEAELWG--------------------------MDGNGRWATQRKRPRLFGHQAGARRVREIVEACPELGVHYLTIFAFSTENWKRTQSEVAGLMSLFRRYIAKEMQEFMRQGVRVRFIGDRVRLDAKLRALMDEVEALTIANDRTHLTIALNYGGRDEVARATRRLAQEVAADRLHPDEVDETTLARFLDTCVLPDPDLVIRTSGEARISNFLLWQSAYAEYEFIDTLWPDFTPEVFESVLENFGNRERRFGAVPA----------MPETGQTAPDFALPQVGPEQTGDNAETLRLSDLRPAPVVLYFYPRDDTPGCTREAREFSEMLPKFAALGARVVGISRDTPAKHERFRDKHDLSVTLLSDEDGAVCEAYGVWVEKNMYGRRSMGIERATFLIDGEGIVRRVWRKVKVAGHAEEVLDAVAAL------------MTDQE------------------KQGDRIAKVLARAGVASRRDAEKMILEGRVRVDGKRIDSPALNVTGRERITVDGTELAPPEPPRLWLYHKPLGLVTTERDEKGRTTVFESLPEEMPRVMSVGRLDLNSEGLLLLTNDGAIKRQLELPATGWLRKYRVRVKGTPEDAALEPLRKGLVVEGERFQGMIVQLDRQQGANAWLTVGLREGRNREIRRAMEAVGLTVNRLIRVSYGPFQLGDLKPGEVSEIKGRVMRDQLGLTPEE-EPT---------GTARKARRPGKPD-GRKPGARKPAGT------GAQGGRMQ----------------GKVLQRPRRNAAETAERDAERTEPRGDAGPKGRPQGKPGGKPRARTGSKPAASKPGNRGTNDRGFGERGFAERGAGKRPGGPSPRKPGKPG--GPRRG---------------------------MLDTGFWITSGAILLLLVMSAFFSGSETALTAASRGKLRSQSDKGSRGATTALAITEDNERLIGSVLLGNNLVNILATSLATALFTRLLGDSGVAL--ATLVMTLLVLIFAEVLPKTYAITNAEAAAALVARPIAFVITVLSPVVSAVRALVRLLLSVFGVRTDPDSHILAVREEIAGALQLGHSEGVVEKEDKDRILGALDLSDRAVEEIMLHRSKIEMINVDDAPGDILSQLLESRYTRLPLYRDDPENIIGVVHAKDLLRAMDKLSRGPE--ASEDALEKFNVLDVSMKPYFVPETTPLDDQMREFLRRRTHFALVVDEYGSLQGLITLEDILEEIVGEITDEFDVDAEHPIRKTDDGAYLVEGGMTIRDLNRATEWNMPDEEANTVAGLVIHEAQMIPTQGQVFSFHGFRFEVVQRKDNRITKLKIRPL----------MSAHGQPVPMHARRAGPLRGQADVPGDKSISHRSLILGALSVGETRITGLLEGQDVLDTAAAMAAFGAQVENHGGGSWSVHGVGVGGFAEPSQVIDCGNSGTGVRLVMGAMATTPVTASFTGDASLNKRPMGRVTDPLALFGARAYGREGGRLPMTLVGAAEPVPVTYRLPVASAQVKSAVLLAGLNAPGETVVIERVPTRDHTERMLAGFGAEIETVERPAEDGQGSEHVITLRGRPELRAQDIAVPRDPSSAAFPVCAALITEGSDVLVPNIGLNPTRAGLFTTLQEMGADLTFENMREEGGEPVADLRARYSPDMKGIAVPPARAASMIDEYPILSVVASFAEGETVMDGVEELRVKECDRIDAMARGLEACGVRIEEGPARMVVHGMGPGGVPGGATAATRLDHRIAMSFLCLGMAARQPVAVDDAGPVVTSFPIFETLMRDLGADLRRGGADAGGQGA-----------------------------------MTAARHAGAESAADNATSKQLLDEILDSLASDKAEDVVTIDLRGRTELADHMVIASGRSTRQVAAISEKLVDRLKQEFGRLSKVEGKDAGDWVLIDTGDVIVHVFRPEVREFYALEKMW--LPTEPR-------------MAFLSDRLARVKPSPTVAVTTLAQQLKAAGRDVIGLGAGEPDFDTPEHIRQAAVRAIDEGRTKYTAPDGMPELKEAVCAKFRRDNDLVYTPEQISVGTGGKQVLFNALLATLNPGDEVVIPAPFWVSYPDIVRLCGGEAVIVPTTMADGFKLSAEALEAAITPATKWFIFNSPSNPTGAAYTHDEIRALTQVLLRHPHVHVLSDDIYEHLTYDGFRFATPAQVEPALMDRTLTLNGVSKTYAMTGWRIGYAGGPVDLIAAMRKIQSQSTTNPSTISQWAAVEALNGKQDFLEENRAIFVRRRDLVVSALDAIPGIDCPVPQGAFYVYPSIAGLIGRRSAAGTLIADDEAFARALLEETGVAVVFGAAFGLSPCFRVSYATSDDTLTEACARIRRFCSGLS--MNTHTPVPL-----TGQAPDDKVHFQRRELGAIMGLYGRMVAAGEWRDYGISSLREVAIFSIFRRTAEHPIYRIEKRPRLALRQGQYSVIGMDGRILKRGHDLKSVLRVLERKLIRAVE--------------MIELENAAYSY-GGAELLSDMSLQLGAGSFHFLTGPSGAGKTTFLKLCYAELVPSAGTVRLLGQDVRQMDRDDVARLRRRVGVVHQDCQFLDHLSLRENIALPLEVSGHDMNADSINLDELLGWVGLAERARALPPELSGGERQRAALARAVIMSPDVIIADEPTGNIDWEMSLRLLTLLVELN-KMGKTIVIATHDLNLIRATKAEV--------------SARVLRIANRRLQLAGSDLMFGIPGKQAVTMTPAAQAHIRRLMEKDGSVGLRIGVKKGGCAGMEYTMEYVREIAQADEVVELDGARVMIAPMAQMFLFGTEIDYETSLLESGFKFRNPNVADACGCGESIKFK--DVAELQAEGGEGSMQGSANL-NLMIKAARRAGRSLVKDFREVENLQVSMKGAGDFVSRADLAAEKILKEDLMDGRPNYGWLAEESAEEIQGKDPTRRWIVDPLDGTTNFLHGLPHWAVSIGLEHKGEIVAAVVFDPAKDEMFVAEKGFGAFMNES-RLRVSGRRSMIEAIFATGLPFGGRVDLPDTL--RDLGRLLPQCAGVRRWGAAALDLAYVSAGRFDGFWERDLKPWDMAAGILLVREAGGFVQSIDPEAGMLESGEVIAGNEQIFDKFAKAIRGA---------MKIATFNINGIKARVTALGDWLDEAQPDVALLQEIKSVDEGFPRELFEDRGYQVETHGQKGFNGVAILSKLPLEDVVRGLPGDD-------EDEQARWIEATVVGRT-AVRLCCLYLPNGN---------PAPGPKYLYKLAWMDRLYDRAQALLDAEEPAMMAGDYNIIPQDEDAARPDAWRKDALALPESRHAHRRVLNLGFTDAFRARTEGPGHYSFWDYQAGAWNRNDGIRIDHLLLTPQCADLLQDCQIDSEIRGREKPSDHVPVWVELDAMFTGIVTDIGRLIAVERRGDM-RARIATAYDAAGIDIGASIACDGVCLTVVDKGRVDGAGVGPENWFDVDISAETLSATNLG--AWQDGRHINLERALRVGDELGGHIVSGHVDGTATLEELRPEGDSTRLTFRAPAALTRFIAPKGSVALNGTSLTV-NEVEGERFGVNLIPHTMAATNWSEARAGDAINLEIDTLARYVARLAEAG------MFDKILIANRGEIACRVIKTARRMGIKTVAIYSDADRNALHVRMADEAVHIGPAPANQSYIVIDKVMDAIRQTGAQAVHPGYGFLSENARFAEALEAEGVAFIGPPKGAIEAMGDKITSKKIAQEAGVSTVPGHMGLIADAEEAVRIASQIGYPVMIKASAGGGGKGMRIAWNDDEAREGFQSSKNEAANSFGDDRIFIEKFVTQPRHIEIQVLSDAHGNALYLGERECSIQRRNQKVIEEAPSPFLDEETRRAMGEQAVALAQAVGYTSAGTVEFIVDG-DRNFYFLEMNTRLQVEHPVTELITGIDLVEQMIRVADGQALTLKQSDIKLNGWAMESRLYAEDPYRNFLPSIGRMTRYRPPEEVV---------------RDTLVVRNDTGVFEGGEISMYYDPMIAKLCTWAPSRLEAIEAMRVALDSFEVEGIGNNLPFLSAVMDHPRFVSGNITTAFIEEEYPEGFEGATLPEDELRRIAAATAAMNRVAEIRRTRVSGRMDNHERHVGRDWVVTLQGQEYAVEIE-ADREGATIRFDDGESLRVTSDWQPGQPLAMLQVGEAPLVLKVGKISGGFRVRSRGADLKVHVRTPRQAELARLMPEKQAPDTSKLLLCPMPGLVVKIDVEEGQEVQEGQALCTIEAMKMENILRAERKGTVSKINAEAGDSLAVDEIIMEFD----MREIVLDTETTGLDPFDGHRIVEIGAVELLNHMPTGRTFHKYINPQRSMPQEAFQVHGLGDEFLA-------------DKPLF--AAIADEFLSFVG-EARLVIHNASFDMKFLNAELDWCKRTKLPMTQALDTLAIARKRFPGAPSSLDALCRRFAIDNSARTLHGALLDSEILAEVYLELIGGRQPDFALNVRSSGSGDDQGAGDWRPAPRPTPLPSRLTAAEAEAHAAFVAKLGDAPLWLKEGRGMADLKKLAEEIVGLTLLEAQELKTILKDEYGIEPAAGGAVMMAGPAGDA-GGDAGEEQTEFDVILKAAGAQKINVIKEVRAIT-GLGLKEAKELVEAGGKAVKEGVSKEEAEDIKGKLEAAGAEIELKMTDKNQKTPQDMLEREAREEALAPLLDNGWRLDEG-RDALSKRFEFKNFVEAFGWMTRAAIWAEKWNHHPEWSNVYNRVDVTLTSHDAGGLTQRDVKLARKMDALNS-------MARDRRQT-KRKVSKNIATGVAHVNSSFNNTKILISDVQGNAIAWSSAGTMGFKGSRKSTPYAAQMAAEDVGKKAQEHGVKTLEVEVQGPGSGRESALRALAAAGFNITAIRDVTPIAHNGVRPPKRRRV-------------------MIQTISDALEQQGYTTLTPVQEAVTDPALEASDLLVSAQTGSGKTVGFGLAIAPTLLGDETRFG-PAATPLALIIAPTRELALQVRRELTWLYGKAGAVVTSCVGGMDMRDERRALDRGAHVVVATPGRLRDHIQRGSLDMSTLRAIVLDEADEMLDLGFREDLEFILSEAPEDRRTLLFSATVPPAIAQLAGSYQRD-AVRVSTTAGTSQHADIEYRALTVQSREAENAIINVLRFYEAKNAIVFCNTRAMVNRLTTRFSNRGFAVVALSGELTQTERTHALQAMRDGRAQVCVATDVAARGIDLPNLELVIHAELPSNSDTLLHRSGRTGRAGRKGVSALIVTAQSKRKAERLLGMAKV-KAEWGSPPSAAEVLARDEERLMADPAWQEPVAKDERAFVDRLTSEFAPEQLAAAYLRLYRSRVSAPEELSDGKVE--------TRPR--E---AF----------------------------------GPGTWFSVSIGRDRNAEPRWLLPLLCRKGDITRDDIGAIRIQQRETYVEILTSSVPAFLAAIGPSMQLEDGASLRQMDSAPDLA-----APPRAGGKGRP----ERAERP--AYKGKPGADKGAKPR--REAPAADNGWQP--D-QGAPSEAADIAPRPAPK-----KPREA----------RIEGAGDDP----------RADKTGKPFKPGKATRPERFDPKADRSGPHDGKSGSKGKFG-KPGGKPDGKPGGKPRDFDNEDRPRGDKWEG----------------KGGGKFAGKPGGKPSSKPGG--KPGGKPAGGFAPRPGKGDGPDAPASRGAPDWASDPSRSLKPRGAGKGKPGPKGAGKPGGK-----PGG------------KSFGKPGGKPGGKPFAKPGGKPG---GKPAG------KGGSAAPRRPKP-----MAGAARQAARAGADGAALLSPGARDLLQRWLESLSALDDASANTIEAYRGDLVSFLSFLTLHRAEGQGADRLGAIEPSDMRSWLAHERASGLGARSLARRLSAVKGFYRWLAERDGTEATAVLSARAPRFQKKLPRPLAPDAAREMITTVSLQA-PEPWVALRDAAVVTLLYGCGLRISEALGLKGRDHPLDEVLRITGKGGKERLVPVLPVAREAVAAYVAACPHGIAPDMPLFRGVRGGPLLPRIVQKVVAEARMQLGLPATATPHALRHSFATHLLEAGGDLRTIQELLGHASLSTTQTYTAVDTARLMEVYDRAHPKA-------MTDRALTSTLLLQAYARGIFPMAESRDDDAVFWVDPHLRGVLPLEAFHISRRLARRIRRGDFRVTVDRAFEDVVRGCAD----RDTTWISDQIFRLYTELHEAGHAHSVEVWE-GEELVGGVYGVAIGGAFFGESMFSRRTDASKVALAYLVSRLREGGFTLLDTQFLTEHLASLGGQEITRAEYRRALAAALERPADFMAQPPEP-----------------------------------------DVASVLQRSTHTSMARVTVEDCVDKVPNRFELVMLAAHRAREVAAGSPLTVDRDNDKNPVVSLREIAEETQSADDLRERMIEAHQTQIEVDEPEEDAMALLM-GVEA-DKPADDDMDEEKLLRALMEAQGQG-----MASAAEQMAANMSWGAFGKATELKQRILFTLALLIVYRLGTYIPVPGIDGTALRQFVQEAATGLGGILNMFTGGAIGRMGIFALGIMPYISASIIVQLLTAMVPSLEQLKKEGEQGRKKINQYTRYGTVFLATFQAYGLAASLEAGDLATDP--GLYFKASAVITLVGGTMFLMWLGEQITSRGIGNGISLIIFVGIIAEVPAALAQFFASGRSGALSPAVIVGVIIMVILTIAFVVFMERALRKIHIQYPRRQVGMKVYDGGSSHLPIKVNPAGVIPAIFASSLLLLPTTISTFS-GSQTGP-------VMSTILAYFGPGQPLYLLFFAAMVIFFTFFYTANVAFKTDDVAENLKNQNGFIPGIRPGKRTEEYLDYVVNRILVLGAGYLTLVTLLPEILRSQLA-IPFYFGGTSVLIVVSVTMDTIQQVQSHLLAHQYEGLIEKSQLR--------GKRRGKKGTARRMVSRVIPVETFDLVIFGGTGDLARRKILPALFRRFQAGQMPEESRIIGAARSEMGTKEFRAFVGEAIGEFIPAEEAPAKAVENFLRHISYVAVDARGTDG-WSDLSAQVR--QDVIQAFYFSVAPALFGDLAERLHEHGIAGPESRIVVEKPFGRDLESAHALNATLAAHFDEHQIYRIDHYLGKETVQNLMAVRFGNILFEPLWNAQYVDHVQITVAETVGVEGRGAYYDLSGAMRDMVQNHMMQLLCLIAMEPPSQFEPDAVRDEKLKVIRALDPVAPAE----IVRGQYKAGN----GRPSYVEGVENPD-SRTESFIAMRVDIANWRWKGTPFYLRTGKRLKSRMSEIVVTFREPPHSIFGEEAGKSANVLVIRLQPDEGITLRVTIKEPGPGGMRLVDVPLDMTFAEALGPDADDIPDAYERLIMDVIRGNQTLFMRGDEVEAAWAWTDPIIAAWSDKGDRPLPYETGSTGPEDALMLLHRDGRRWREIEVMRILFLGDVVGRAGRKAITEHLKPLREAWKLDFIVVNGENATGGAGLSAAHAKTLLDAGADCLTLGDHAFDQRDMLSFIEQEPRILRPLNY--AKGAPGRGHRLFTATGGRKVLVTQVLGNVFMKRAFDDPFSAVEPVLKSHPLGGMAQAILVDMHCEATSEKMGMGHWCDGRASVVVGTHTHVPTADAQILPRGTAFQSDAGMCGDYNSVIGMEKEEPMRRFVTGMGRGRFT--PAMGEATMSGLLVETDDRTGLAKSVRMIRH----GGRLESAAP------------------------------------------MKLSDFDFDLPETLIATRPAVPRTAARLLLAEG---PG-IEDRQVADLPDILRPGDVLVLNDTKVMPVHLDGAR-HRDSAQGPVTARIRVNLLEPLPD-GR---WTALLKPLRKLREGEEIVFAPGFAARFEGVEGDHAILH-FDLEGEDFDTALETAGAMPLPPYIAARRAPDAQDMTDYQTVFA-RQRGAVAAPTASLHFDRALLDRLEAAGVEFVHVTLHVGAGTFLPVKVDDITQHRMHSERGEITAEAAERLNRARAEGRRVIPVGTTALRLIESAANEDGTLAAWQGSTDIFITPGYRFRVTDGLMTNFHLPKSTLMMLVSALMGADTIRNIYRHAIDTGYRFFSYGDSSLLLPETGAGARKVAQRS------------MLTALIDYDSGNLHSAEKAFQRMAREVDAGDVVVTSDPEVVRRADRIVLPGDGAFPACRRALLDVPGLFEAVEEAAITRARPFLGICVGMQMLATTGHEYSQTPGFDWIGGEVVPIRPADPSLKVPHMGWNDLVIDNPHPVLEGIGAGAHAYFVHSYHMAVADPGAHRIAHVDYAGDITAIIGRDNLLGMQFHPEKSQADGLRMIANFLRWAPMKRYVMAAVGGTLAGIVMTTQVA-----GPLIAQESEQKTNVYEQLDLFGDIFERIRAQYVEEVDEADLIEAAINGMLTSLDPHSSYLPPKDFDDMQVQTRGEFGGLGIEVTQEEGFVKVVSPIDGTPADEAGIEAGDFVTHVDGESVLGLTLDDAVEMMRGPVGSEIVITVVREGKAEPFDVTIVRDTIRLTAVRARLE-GTTPVLRVTTFNDQTYPNLETELKKLVEEAGGIDKVNGFILDLRNNPGGLLTQAIRVSDAFLDKGEIVSTRGRNPQDSDRVNASPGDLAEGKPMVVLINGGSASASEIVAGALQDHHRAVVVGTKSFGKGSVQTIMPLRGE-AAMRLTTARYYTPSGRSIQALGVSPDIVVEQPPRPEAAAEGEDGDEKANGTRRP-NRSEADLRGAISNDSMTDAEREQLKKEQEAAAAAAKLREEDYQLSYAIDLLK-GLKVLGPQE--------------------------MADGAMELSAKPTEELSVREVFGIDTDMKVKGFADRTDRVPELDSTYKFDPDTTLAILAGFSHNRRVMIQGYHGTGKSTHIEQVASRLNWPCVRVNLDSHISRIDLIGKDAIKLKDGKQVTEFQEGILPWALRTPTAIVFDEYDAGRADVMFVIQRVLEVDGKLTLLDQNKVITPHPYFRIFATANTVGLGDTTGLYHGTQQINQGQMDRWSLVATLNYLSHDAETAIVLAKNPHYNTAAGRRTIGQMVTVADLTRTAFMNGDLSTVMSPRTVLAWAQNAEIFRDVGYAFRVSFLNKCDELERQTVAEFYQRCFDEELPESAASMSLGMTERATDTPLPAGSTIGILGGGQLGRMLSAAASRLGLRCHIYEPGANPPAADVAHALTTASYDDHEALAAFAASVDVVTYEFENIPAPALDLLA-DSAPIRPGRRALEVSQDRLTEKTFLCDLGLRTAPFVACDDAGALRDALAQIGPRGILKTRRFGYDGKGQVRLDAPDQCEDAIAQMAGAPAILEGFVDFSAEVSVIGARSASGDIACFDPGENEHRDGILHRTLVPSRLSPSLRSDAVLLAARILNALDYVGVMGVELFVTPDG-LVVNEIAPRVHNSGHWTQLGCAVDQFEQHIRAVAGWPLGDGKRHADVEMLNLIGDDILTAHEHAGQTGTQLHLYGKAEARPGRKMGHINRLRSAG--------MRRKLAAGNWKMNGCAPALEQISKLCEGL-A-DGNANATGTDVLICPPSTLLHRMSERA-GGRIALGGQDCHPETSGAHTGDLSAVMLADAGASHVILGHSERRADHGESDELVRRKARAAIAAGLTAVICVGESKQERDEERALEVVGRQLAGSVPDAVTARNLVVAYEPIWAIGTGATPTLEEIAEVHDHMRARLEERFGGGIGRGVRLLYGGSVKPANAGEIFGVENVDGALVGGASLLARDFLPIIKALEAAS--MSD----NILSQIKSYKLREVAEAKAECDLSGMEQRAREADTVRPFTEALQAASRTG-YGLIAEIKKASPSKGLIRADFDPALLAKAYEEGGATCLSVLTDGPSFQGAPEYLVEARAAVSLPVLRKDFLFDPYQVVESRAMGADCILIIMAAVTDAEAAALEETATTWGMDVLIEVHDAEELERAALLKSPLIGINNRDLRSFVTDLDVTRRLAKLVPEDRLIVSESGMSTSDDLADMAMNGARIFLIGEALMRKADVAAATREIL-RPATSAMGGM-MSFTLAIVGRPNVGKSTLFNRLVGRRLALVDDQPGVTRDLREGEARLGDLRFTVVDTAGLEEATDQSLQGRMRRLTERAVDMADICLFMIDARVGITPTDQVFAEILRKRSAHVIVAANKAEGRAGEAGMLEAYSLGLGEPLRLSAEHGEGMEDLLAVLT-PLADEF---ALKAQDS---A-PE------------------TDVELDEEGALPGEEDADEDGVLPSPRPSRAKPLQVAVVGRPNAGKSTLINAILGEDRLLTGPEAGITRDAISVTTEWD-G-----APVRIFDTAGMRKRAKVQDKLEKLSVSDGLRAVRFAEVVVVLLDVEIPFEQQDLRIADLAEREGRAVVIAVNKWDIETDRQNKLKELKEEFTRLLPQLRGAPLITVSAKTGRGLDRLQEAILKAHEVWNRRVTTATLNRWLIGMLEAHPPPAPGGRRIKLRYMTQAKTRPPGFVVMCSHPEKLPESYSRYLVNGLRDSFDMPGTPIRLTFRSQADKNPYKDR-KKSTPSRLRKHLGKGRADE------MSDFDEVTPDALVIGGGPAGLMAAEELSRAGHRVLVADARPSLGRKFLMAGKSGLNLTRDEDDARFLAAYEEASA-PLAPMLAEFGPRAVVSWAEGLEQEVFTGTSLRVFPKAMKASPLLRAWLARLAGQGVETRTRWRWTGFAEGGA----VCFEAPDGPLTLRPRVTVLALGGASWPRLGSDGAWVGALEEAGVAIAPLRPANAGLAVDWSDHM-TRHHGAPVKGVALLAQGDNGQRHLSRGEFVITARGLEGGGVYSISKPVRE------GARLCLDLCPDLGAQRVAERLGRPRGKQSRANHIRKALGLDPVKLALLQEFGR-PLPEEAGDLAALIKALPVAHQGLRPIEEAISTAGGIRWDALDEDLMLRALPGVFAVGEMLDWEAPTGGYLLTACLATGRHAGRAAAARLAGSPKD------------MSDTESSKPAAGAGSAWRPRTRAVHSGSRRSGYGETSEAIFLTQGFVYDSASQAEQRFVELGDDE-FIYARYGNPTTRMFEDRIAAIEGAEAAFATASGMAAVNGALCSMLKAGDHVVSSRALFGSCLYILEEILTRYGVEVTFVDGADNEQWRAALRPDTRAVFLETVSNPTLEVVDLRAVCDMAHAVGATVVVDNVFATPIFQPVIEMGADVVIYSATKHIDGQGRCLGGVILGTEEFIRKTAEPYLKHTGGAMSPFNAWVMLKGLETLHLRCNAQADSALTIATALQGHEKLNKALYPLVESHPQFDLARQQM-SRGGTVLALDLKGGKEAAFRFLDALEIIIISNNLGDAKSLVTHPATTTHQRLSEEQRATLGITDGLVRVSIGLEDTDDLLEDIQAALAAV----MGLDTAIAKPH-----DELRSLLAGDLDAVNHLIRERMASEHAPRIPEVTAHLVEAGGKRVRPMLTLAAARMCGYDGAHHVKLAATVEFIHTATLLHDDVVDESGQRRGRPSANLLWDNKSSVLVGDYLFARSFQLMIETGSMRVLDILANASATIAEGEVLQLT--AAQDLATDEATYIQIVRGKTAALFAAATEVGGVIAGAPEDQVAALHAYGDALGIAFQIADDLLDY-GGTAAIGKNIGDDFRERKMTLPVIHAVAAAEGEEAAFWNRVIARGRQQEGDLEQAIELFRRHGSLDATRDKALGWAKKAKDALAPL--PEGRIRELMRDLADYVVERIN-------------------MAKKMLIDATHAEETRVVVVDGNKVEEFDFESVNKRQLAGNIYLAKVTRVEPSLQAAFVDYGGNRHGFLAFSEIHPDYYQIPVADRQALLAEERAYAASLEDGD-----------------EEGQPRK-----RSRSRS-RTKAADASGG------------DPTTTAEL-------TDDLTAEGGEGG----DESATEDAARDEGRKPRSRSRSRRGAKKDTGTTEATGTDIPGMGVVDLDETDGDPLVADENSDTDASPDAGDSPSRKTRG------------------------------------------------------------------------------------------------------RAPSEDDGGEDDGNGALDAADKDSEIESIADEDVADEIRQPRKARPRKYKIQEVIKVRQILLVQVVKEERGNKGAALTTYLSLAGRYCVLMPNTARGGGISRKITNVADRKKLKEIAQDITVPEGAGLIIRTAGAKRTKSEVKRDYEYLLRLWEQIRELTLKSIAPAPIYEEGNLIKRSIRDLYNREIDEVFVEGEAGYRVAKDFMKMLMPSHAKNVKLYNEPLPLFARYQVESYLAAMFNPTVQLKSGGYIVIGVTEALVAIDVNSGRATKEGSIEETALKTNLEAAEEVARQLRLRDLAGLIVIDFIDMDERKNNAAVEKRFKDKLKTDRARIQVGRISGFGLMEMSRQRLRPGMIEATTQPCPSCHGTGLIRSDDSLALSILRQLEEEGVRRRSREVLLKAPIGIVNFLMNQKREHVASIEGRYGLSIRVEADPFLISPDFSIEKFK---TATRVVAEVPSPVVSIDMSDMPDAIEEDEIEEDAAEAATESSE------AENRGAESG-------------DDEGQPKKRRRRRRRRRGGRSD--------EQGFENGDQSGEERSRESDDSDGAEGDAPLAATTDADP-----EQSRAGE-AVTAEAREGQDAEATAEAADAE-EAPRKKRT--RAPRRRKSET-EG-----TEAAEAEAAQGDSAKADAE-APEAAADEA--EKPKRAPRKRAPRKKK------TDAEGAAELPEVVEKVVEDAAEKPAPKPR-------TRRSRA-KPKADAVEPATPV------------------DTPEPVPAEAAAPMT---PGTEPAAA-AMASAEA------VTGE---------DTRA---EPEISHEDATETGAVAVADEKVEETAEPASD-----RPKRRGWWSLGR---MKFLHTMVRVRDVDASLDFYCNKLGLVETRRTENEAGRFTLIFLAAPRD--RETAEAEQAPE-------LELTYNWDGDEVYQT-GRSWGHLAYRVSDIYATCQKLMDAGVTINRPPRDGRMAFIKSPDDVSIELLQEGKALAPAEPWASMENTGSW--MTDEQKNTTHFGFRTVPEDEKAGMVHGVFTNVASRYDVMNDVMSGGVHRLWKDAMMDWLAPRPGQRLLDVAGGTGDISFRFL-------DRAGD---AEAVVLDMTEQMLVEGRRRAEAGARADRLSWIVGDAMQLPFEDDSFDVYTISFGIRNVTRIPDALSEAYRVLKPGGRLMVLEFSQIPNPAMQWAYDRYSFNVIPAMGQVIANDRDSYQYLVESIRKFPDQESFAAMIRQAGFGQVKYRNLTFGVAALHSGWKLMAKQPMLKFVNIERDMPEKRPPDLRSRDFDEIYAEFADAKAAEQSSRCSQCGVPYCQSHCPLHNNIPDWLRLTAEGRLREAYEVSQATNTFPEICGRICPQDRLCEGNCVIEQSGHGTVTIGSVEKYITDTAWEEGWVTPAAPRQERSESVGIIGAGPGGLAAADMLRQQGIQVTVYDRYDRAGGLLTYGIPGFKLEKDVVMRRIDQLEQGGVTFRLDCNVGEDIGFDEIREAHDAVVIATGVYKSRDLQAPGIGAQGIVRALDYLTASNRQ-NMGDTVPEYESGELNAKGKRVVVIGGGDTAMDCVRTAIRQGAESVRCLYRRDRANMPGSQREVANAEEEGVEFVWLSAPKGFT---GD--------------TVEGVMVSRMRLGAPDATGRQMPEVIEGADYVEEADLVIKALGFEPEDLPTLWSQPELEVTRWGTVKADFRTHQTAMPGVYAVGDIVRGASLVVWAIRDGREAADAIIAEFASR-EV--VAAEMRVRICAVGRLRAGPERLLLDDYLSRFDKTGRALGL-GPAQVVEVEDRKGGGMTAEAALLERAVPEAAALWVLDERGRKLSSRDFAQELARHRDDGRGELALMIGGADGIDPALRARADMAISLGAMVWPHMLARVMLSEQLYRAASILSGSPYHRD-MPVFDTPIAALLLTALLGYLLGSVPFGLVMTRLFGLGDLRRIGSGNIGATNVLRTGNKTAAFLTLVLDAGKGAIAVLLARALLGE-DAAQIAGFFAFLGHCYPATLGFKGGKGVATFLGTLLALYWPAGLAA-CGVWLVMAAIFRISSLAALVAAAAAPLLLYLLGMPQAVLFG--AALAALIFLRHRANIARLVTGTEPRIGQK-------------MPERVLKIVNEKGLHARASAKLVEVVEAHDAEATVSKDGMDVSGDSIMGLLMLAASKGSEIRIATKGNAAEDLAAALEELVTNRFGE--DRMPLEAPPA---------------PVADRARALNHRHRNHAATTVLEHALNDPEMGRVALVSSFGAESVVLLHMVACIDRTTPVLFLETGMLFAETLEYQRDLVARLGLTGLRHLAPDRTELFTEDHDGLLHLAEPDACCALRKTRPLERALGSFDAWITGRKRFQGGQRQALEFFEPDGDT-----RLKINPLAHWSARDLSDYIDNNRLPRHPMVARGYPSIGCAPCTGPVEPGRDPRAGRWPGMAKTECGIHFTAGGVRR----------------DGGATPAKAATEQDAKGTQMDVILLERVAKLGQMGEVVSVKEGYARNYLLPQGKARRASDANLKMFEEQKAQLEARNLETKKEADSLAAKIDDQQYVVIRSASDAGALYGSVTTRDAADAINEDGVSIDRKQIVLSEPIKYLGLHTVTVVLHPEVTASVKLNVARSPEEAELQASGKSI-QELAAEEEAEAEFEIAELFDDIGAAGLDDDDDRGS-------------DERDSDDE----TEEN------MSTPFSTDAPVTRAWVIHPDIRNDRQRRQALAALDEAAALGDALPGLEVVGKEVVALPKARAGLLFGSGKIEELAKRFEEAEVDLVLIDGPVTPVQQRNLEKAWKVKILDRTGLILEIFSDRARTREGVLQVEMAALSYQRTRLVRAWTHLERQRGGLGFVGGPGETQIEADRRAIDDAMVRLRRQLDKVVKTRALHRDARKKVPFPIVALVGYTNAGKSTLFNRMTGAEVMAEDMLFATLDPTMRAVELPS-GPRVILSDTVGFISDLPTELVAAFRATLEEVLDADLVVHVRDIAHPQSEEQAADVRHILADMGIDDQ---APLLEVWNKIDALPAEARDPVLATAERDPG--VLALSALTGEGIEALYGRIADMLAAPRTRETLTLGFSEGRRRAWLFEQGVVEEDRQVED-GYEMRVYWTERQKQQFSAL-------------MEGDIVQMGAYLGAGLACIGMGGAAVGVGNVVASFITGALRNPSAAASQTATMFIGIAFAEALGIFSFLVALLLMFAVMTLHILTNDLL---MPLAHGFFTRRGGASTGIYAELNGGAGSSDQAHVVSLNRARVAEALDLAPASLFSVHQYHSAEVFVLDGEPPA-EKPRADAIVTRQPGYALTILTADCQPVLFADHEAQVIGAAHAGWQGALGGVLEATIDRMVELGAQRERIVAAIGPSISQKNYEVGPDFMERFLDDDPENQRFFAQGKGDRVQFDLPGYGLHRLRSAGIRDAEWIRHCTYEDPGAFYSYRRSVHLNEADYGRLMSSIRL--MAGTETHHVLPLPGFDRPLRLLIVVAPYYKDIADDLLAGAIATLDKAGATHEVVEVPGALEVPGAIAIARRGANFDGFVALGCVIRGETTHYETVCNDSSRAL-TLMGLDGACIGNGILTVETRNQAEVRADPEGQNKGGGAAAAALHLVALSRRWPAGGSETDGASGTTGTQQAPFRMAGQ-DKGTDIAMNYFEELGLTEALAKRVTDAGLTDPTPIQAKAIPLVLEGRDVMGLAQTGTGKTAAFGLPLIDQLSRIGSKPDAQTCRGLILAPTRELAKQISVSLEQFTRNTHMRVELVVGGASIGAQINRLRRGTDVLVATPGRLLDILDRRALRLDQTRFLVLDEADQMLDLGFIHALRKIAALLPKERQTLLFSATMPKQMAELAETYLTNAARVQVNPPGKAADKILQSVHYVQKGDKPALLMDYLKKHPGELAIVFGRTKHGSEKLKKSLVAAGFSAVSVHGNKSQGQRERAIAAFRSGEEEILVATDVAARGLDIPEVRHVYNFDLPNVPENYVHRIGRTARAGADGKAVAFCAPDEMGELKQIQKVMKITI-PVAGGTPWEGGAVEEG-SKNGRRGGGGGGGGGRRRFG------------AGGGN----GGPKGGNGGGGNGGQ----RRQQQR----------------RRRTAA---MANAQGKVTQVIGAVVDVQFDDQLPEILNALETENNGKRLVMEVAQHLGENTVRCIAMDATEGLVRGQAVTDTGGQIRMPVGNATLGRILNVTGDPVDEKGPVDSTETRAIHGEAPAFADQSTETEILVTGIKVIDLLAPYTKGGKIGLFGGAGVGKTVLIMELINNIAKVHSGLSVFAGVGERTREGNDLYHEMIESGVIVPDNLTESKIALVYGQMNEPPGARMRIALSGLTLAEQFRDESGSDVLFFVDNIFRFTQAGSEVSALLGRIPSAVGYQPTLATDMGAMQERIASTKAGSITSVQAVYVPADDLTDPAPATSFAHLDATTVLSRAISELGIYPAVDPLDSTSRLLDPAVIGDEHYKTATDVQQILQRYKSLQDIIAILGMDELSEEDKLTVSRARKIQRFLSQPFDVAKVFTGSDGIQVPLDVTIASFKAVVAGEYDHLPEGAFYMVGGIEDVIAKAEKMAADAA-MTDQPTDPLDPLVRLAPLAELARTVAARK-GADPDTSWTAKLLSQGPDKCAEKFGEEAVEAVVAAVRGDRAHLTAEAADVIFHLLVMCAARDVDLADIADELARRAGTSGIAEKAARPRG-MGIDTLTPEPPQDQADTPRLEFPDNRLLIELCGEFDSHLAHLETALGVQVVRRGNQLEVAGEPAARAKALEVLRGLYQRLESGKAVDVGVIDAMIRM--GAESDSQSRDGDQLEMFKG---GEIEIKTRKKSVEPRTAAQKAYARALFDNELVFGIGPAGTGKTYIAVAAAVNMFIGGHVDRIILSRPAVEAGEKLGYLPGDMKDKVDPYMQPLYDALNDFLPGKQLAKMIEEKRVEIAPLAFMRGRTLSNAFVVLDEAQNATSMQMKMFLTRLGEGSRMVITGDRTQVDLPRGVQSGLADAERRLGGIDRIAFNYFSAKDVVRHPLVARIIEAYDATDTDAG------------------MRFTVAIDGPAAAGKGTISRAVAERFGLAHLDTGLLYRATGRRVMAGEDPVAA-------ARALRPADL-EADGLRSPEVAQAASRIAALPAVRAALVAFQRDFARREGGAVLDGRDIGTVICPRAEVKLFVTARADIRARRRFDELCAGGYEGAYEQVLADVEARDMRDRERADAPLRPARDAVAIDTSELDIEAAVARAVAEVDAALGRRGTPGTD-MDDRRRQVFRDSHQDVETMRETPDTPQTRASAYRLAFVDGDFLCRDELRPVRLQLELLKPQMLLEEAGIESTIVLFGGARIPAPAERATARTQTLAELSHFYDEARRFSQLMTERSMA-AGGG---ESVIVTGGGPGVMEAGNRGAADAGGKSIGLNIVLPHEQAPNEYVTPELCFNFHYFAIRKMHFLMRARAICCFPGGFGTLDEMFESLTLIQTGRMARIPFLLFGEKFWRSIINWEALAEAGTISDQDLELFRFVETAEEAVELIENWPLPETPRDHVPGRPDAG----MRRRRFTILGCGSSGGVPRLGGIWGACDPDNPRNRRRRCSLLIEQEGPGGV-TRVLVDTSPDMRDQLLDADVGTLDGVVYTHAHADHVHGIDDLRMIVFNTAKRLGVWADGATQNDLLNRFGYAFAQPEGSPYPPILDLH-TIN-DG-LRIEGAGGEIAMLPLRVHHGTIDALGFRVGDLAYLPDVSEIPAETWPRLEGLDCFILDALRRTPHPSHAHLARSLEWIERAAPRRAILTNLHIDLDHDTLEAETPGHITPAHDGMTVEYEL-------MHGADYDPPQDPLAVVHHDHQILLVDKPAGLLSVPGKGAHLADCLIARVQAVFPEALLVHRLDRDTSGIMVFALTHSAQRHLGLQFEKRQTRKTYVARLTGRLEERRGTVDLPLGVDWPNRPRQHVDHENGRAAVTDWRVVRYEEADEDLPEGATRVRLTPHTGRSHQLRVHMLEIGHPILGDPLYASGAALR-APRMMLHAEKLKLRHPDGGEWQSYSAKCPFMIGIVIVAHGGLAREYLAAVEHVVGDQPGIRAISIEPDHDRGVKQREICAAADAVDEGEGVVVVTDMFGGSPSNLSLMACHPVNRKILYGANLPMLIKLAKSRR-LPVAEAVTAAMNAGRKYINSFDGSAG------------MNKPIMAKATAVWLVDNTTLTFRQIAEFVGMHELEVQGIADGDVAQGVKGFDPVANNQLDQADIDKAEKNPLINLKL-KFNAAAVGEEKRRGPRYTPLSKRQDRPASILWLVKFHPELTDGQISKLVGTTKPTIQAIRERTHWNIANIQPIDPVALGLCRQSELDAAVQKAAAKRARDGEVMTDDERRKLVSTEQSLGMDTEPRIPTAISGLETFSLSDKE---EPEEEDDDPRKGGDIVDADSFFNLPSDQPDLDDEDDK--------MASYQARS-RDPLLDRKMQAAIERRGKELLGIALIALGLCVALVLGTYVPDDPSWLSATDEPARNFFGRFGASLASPLFVIVGLGGWGIALALLVWGGRFLFHIGEERALGRVIFAPIAVALGAVLASTHVPFEGWS--HSFGLGGLFGDTILGAFLGVIPVSASLGLK---SLSVLVAAITVVTALFVLGFSMAELRGIGRFMLVGVILAYDHTVRLASQGAMRAAQGAQGLQDRHAAG---REARAAARAASSAERDARQP------------DPSLSR--------TARVTRARTEPEVEPDLLDDEDFDDPA--------PRRGLLSGVTS----------LIRRT--P-----DPVPELVSPQVAP-DLEVAAPDD-DRIREKIASVIKSR-VRAAP----ILTTRSEPPVVAAARRHPRGPQPLIADRG----PRAGVAESGTTAAALAASIAGAGVGSAAGAAGAGIGAASAAASGAMNAFDAAPAHGDSSDPFGDDPFAGDPYA--------AAYEEGLDQYGHEDPYEGYDSLADSEDDHDALSQQSAPGGAPRPMFQPVVSRTAPDAAP----VPGIPAYEPRK-VVQHSPRKPAQPSRQARQEAQPDLRFE--DAPLPEYESPPLSLLTNPAEIQRHHLSDEALEENARMLENVLDDYGVKGEIVSVRPGPVVTMYELEPAPGLKASRVIGLADDIARSMSALSARVSTVPGRSVIGIELPNEHREKVVLREILSHRDFGDGNQRLPLALGKDIGGDPIVANLAKMPHLLIAGTTGSGKSVAINTMILSLLYKLSPDECRLIMIDPKMLELSVYDGIPHLLSPVVTDPKKAVVALKWVVGEMEERYRKMSKMGVRNIEGYNGRVRDALEKGEMFKRTVQTGFDDETGEPVFETDEFKPETIPYIVVIVDEMADLMMVAGKEIEACIQRLAQMARASGIHLIMATQRPSVDVITGTIKANFPTRISFQVTSKIDSRTILGEQGAEQLLGMGDMLFMAGGAKITRVHGPFCSDEEVEEIVNHLKSYGPPDYVGGVVEGPDEEKGSD--IDAVLGL--GGNTDSEDALYDQAVAIVIKDRKCSTSYIQRKLAIGYNKAARLVEQMEEEGLVSPANHVGKREILVPE-QS------MPKMKTKSSAKKRFKMTASGRVKAGQAGKRHGMIKRTNKFLRDARGTTLLSKPDENIVKKYMPYARMARPKIALIGSGQIGGTLAHLAAIKELGDVVLFDIAEGTPQGKALDIAESGPSEKFDAALTGTNDYADIAGADVCIVTAGVPRKPGMSRDDLLGINLKVMKSVGEGIAKHAPNAFVICITNPLDAMVWALREFSGLPHEKVVGMAGVLDSARFRHFLSLEFDVSMRDVTAFVLGGHGDTMVPLARYSTVGGIPLPDLVKMGWTTQEKLDAIIQRTRDGGAEIVGLLKTGSAFYAPATSAIEMAEAYLKDQKRLLPCAAWVDGAFGLKGMYVGVPTVIGAGGVERVVDIKLNKDEQAMFDKSVESVQGLVSACKEIDETLA-------MDINLIEAADRRLEGHVRRTPLLSSPFLDEIAGRRVLVKPECLQHTGSFKFRGGWAAITALDPEQRARGVLATSSGNHAQGIAKAAAAHGVRAVIVMPSDAPEIKIANTRALGAEVVLYDR-ATEDRDLLAADRIEADGLSFIPPFDHPQVIAGQGSVGLEIAAQAQEEGVTEGDVLVCCGGGGLTSGIALALEARAPGLRPRTVEPEGFDDFARSLDEGRICRNPATSGSICDAILTPEPGRLTFPVVSRLAGPGLVVSEDECLRAMALAFARLRIVLEPGGAAALAAALFR-PEEIAGEAVIAVATGGNVDPAVFRQALDRHG-------------------MASLKEIYAARVAEGRLKQDPAQEDALALLEDLRRSLEEVPAPGGLMRFFRAA-PPPPRGLYIWGGVGRGKSMLMDLFYEHAPVEARRRVHFHAFMQEVHAKLHAAR--------------QTGVEDAIAPVAEGLARDVRLLCLDEMQITDITDAMIVGRLFQKLFDAGVTVVTTSNRPPDDLYKDGLNRDLFLPFIGLLKERMQVHELESPRDHRQHRLQGEARYFTPINANARARIEAIWTGLT-GGASSPLVLRVNGREVTVPAYRNGAGRAAFWDLCGQPLGPADYLAIAGALRVLVLEDIPCLGRSNFNQAKRFVTLIDALYEARVTLICSAEAPPHRLYLEGEGSFEFERTASRLAEMQSADWGRPASEAASA---------MTFDYDLFVIGGGSGGVRAARLASSEAGARVGLAEEYRMGGTCVIRGCVPKKLMVFASGYSSVVDDARNFGW-ELSTGPFQWETFAGHMNRELDRLEAAYRNTLAKAGVEVFDARAVLEDAHTVRL-STGERFSARHILVCTGGRPVLP---PIEGAEYGITSNDIFLMPHMPKRMLIVGGGYIASEFACIMNGLGTEVTQFYRGEQILRGFDTETALFVQEQIVERGVNLRLNTNVTRLEKLDDGR-----------------------------------------VRAHATDGTAEEYDQVFFATGRHPNTENMGLEELGVALGKRGEILVDEFSQTGVPSVYAVGDVTGRIDLTPVAIREGAAFVETVFHGRPTPVDHALVPSAVFTQPEMGTVGLTEEEAAEH-GPVEVYTTRFKPMHRAFAGRSDRVLMKLLVQKDTRKVLGCHIVADQAGEMIQLVGIAVKMGATKEEFDRTCAVHPTMAEELVTMKAP-TRTI-----MSGQNIVT-----PSAEGLARAARAAA-----KHDRLPPVHQWNPPFCGDLDMRIARDGTWFYQGTPIGRPELVRLFSTILRRDGE-DYFLITPVEKVGITVDDAPFVAVDFTREEG-PDGPVLTFQTNVGDRCAAGPAHPIRVAHDPESGEPAPYVLVRDRLEALIDRKSFYRLVEIGEHAEHEGRSWFGLRSGGGFFPILPSERV-----MTLTPGFDGFKATFDAGRNQVVAMRLAADLDTPVSVMLKLAHARKDTFILESVTGGEVRGRYSVVGMNPDLIWECRGTASRINRHAETRADAFEDQEGGPLDNLRALIAESRIDMPEDLPPIAAGLFGYLGYDMIRLVERLPNVNPDPLGLPDAVLVRPSVVAVLDGVKGEIILVSPVWAGGDLDARAAYDRAVARIDAAVADLDGGIPDQGHVLGDAAEVAEPVSNFTREGYVAAVEKAKEYIRAGDIFQVVPAQRWTQDFSLPPFALYRSLRRTNPSPFMFFFNFGGFQIVGASPEILVRLRGTEVTIRPIAGTRPRGATPEEDLALEKDLLSDPKELAEHLMLLDLGRNDTGRVSQIGTVRPTEKFVIERYSHVMHIVSNVVGEIAPEHDALSALLAGLPAGTVSGAPKVRAMEIIDELEPEKRGVYGGGCGYFAANGDMDMCIALRTAVLKDEKLYIQAGGGVVFDSDPDAEFEETVNKSRALRRAARDAGLFA-RSTKGMAYRAPVTDYQYILDHVVDFPLLSDTERFGDATSETSLAIISEAGRLCEEVLAPLQREGDLHPAHLENGVVRTSPGFAEGYAAVAEGGWVGISAPEEYGGMGLPLAITSVVNDMMSSACLALQLNPLMTQGQIEALEHHASDEIKRVYLPRLISGEWSGTMNLTEPQAGSDVGALRSRAVDNGDGSFAITGQKIFISWGDNDFTGNVCHLVLARLPGAPEGTRGIGLFMVPKRIPDAEGQPGAANSLRVVSLEHKMGLHGSPTAVMEFDGATGWMIGEPNKGMAAMFTMMNNARLGVGLQGIGVAEGAYQHALAHALERRQGRTPLGDGAGPIADHADVRRMLMQMKAEVFGARAIALGCAVSADMARATGKPEWRD----RAAFLTPIAKAFGTDIGVEVSQMGIQVHGGMGYIEETGASQYLRDVRVTAIYEGTNGIQAMDLVGRKLADG-GEAATRLLDDIEACAEGARAD----MPELAEPIWQAAENLREACDWLVAR--EDLNDRFAGATSFLRAFARVLGACAHLRAAMAEK-AQGEAGPRRA---LARFYVDRLLPEAASLCVQATRGAGDLYGLSHEELAG-MTAGTRTDAPSATTGRAGAVVHVPLGTRAYDVHVGGGLLARSGELIAPLLDRPRVAVISDETVAALHLETLRAGLASHGIEMVALALPPGEATKSWPQFTRATEWLLEQKIERRDMVVALGGGVIGDLAGFAAAVLRRGVRFVQIPTSLLAQVDSSVGGKTGINAPQGKNLIGAFHQPALVLADTDVLDTLPRRDFLAGYGEVVKYGLLGDADFFDWLVAHGPGLLDPGAEADRIEAVRRSVAMKAEIVVRDETEQGDRALLNLGHTFCHALEAATGYSDRLLHGEGVAIGCALAFELSARLGLCAQEEPGRVRAHLREMGMKTDLADIPGPLPDARGLLGLMGQDKKVVQGKLRFILARGIGEAFVTGDVPDKAVLEVLDDALSMR----------------MGKSTTAGLFAAEGVPVWDADAAVHRLYGPGGAAVAPLARICPDAIVEDATGQAIVDRQRLKDWIARDDAALPRIEAIVHPLVGADRAAFLDEAARAGADIVVLDIPLIFETGAADRFDTLVVVSAPAEVQRARVLARGTMTEAEFENILARQVPDAEKRRRADHVIPTTT-LEAAAAAVRQILEQIRGN-RDA--MRNLDFAPLYRATVGFDQIADLMDRVLTNDVAQPSYPPYNIEKTADDAYRISIAVAGFSDSDLSVEVRENALIVSARKSETEED-AGRTYLHRGIATRAFERRFHLADHVRVEGATHADGMLHIDLVREIPEALKPRQIQIARSA---DVDVKAVTDGSAAKKDEKTVN------MAFTLATWNINSVRLREPIVSRLLRENAPDVLCLQECKSPVEKIPLEAFRALGYQHMVARGQK-GYNGVAILSKLPIEEAGAHDFATLGHARHVAARLENGVTVHNFYVPAGGDIPDRERNDKFGQKLDYLTEMRDWFHA-EAPRKSVLVGDLNIAPREDDVWNHKQLLKVVSHTPVEVEHLAQAQDAGGWVDITRQDHPTGQ-LYSWWSYRARDWDAADKGRRLDHVWATPDISAAGHSS--RVLREAR-GWEKPSDHAPVFASFDL---------MTTERLNLSDLKAKSPKDLLSMAEELEIDNASTMRKGDMMFQILKEQAEDGWVIGGDGVLEVMQDGFGFLRSPEANYLPGPDDIYVSPEMIRQFSLRTGDTIEGVIIAPRDTERYFGLTQVEKINFEDPERARHKVAFDNLTPLYPDERLKMELD-DPTTKDRSARIIDLVSPIGKGQRSLIVAPPRTGKTVLLQNIAHSIEKNHPECYLIVLLIDERPEEVTDMQRSVKGEVVSSTFDEPATRHVAVSEMVIEKAKRLVEHKRDVVILLDSITRLGRAFNTVVPSSGKVLTGGVDANALQRPKRFFGAARNIEEGGSLTIIATALIDTGSRMDEVIFEEFKGTGNSEIVLDRKVADKRVFPAMDILKSGTRKEELLVDKIDLQKTYVLRRILNPMGTTDAIEFLISKLKQTKSNSEFFDSMNTMARDYPLERYRNFGIMAHIDAGKTTCSERILFYTGKSHNIGEVHDGAATMDWMEQEQERGITITSAATTTFWERTED--------GQSADTPKHRLNIIDTPGHVDFTIEVERSLAVLDGAVAVLDANAGVEPQTETVWRQADRYKVPRIVFVNKMDKIGADFFNCVHMIADRTGAVPAPIQIPIGAENELEGIVDLVTMKEWVWRGEDLGASWVQEDIRESLQDVAAEWRAKLVETAVEMDDVAMEAYL-EGEEPDVATLRKLIRKGTLSMSFVPVLCGSAFKNKGVQPLLNAVIDYLPSPLDVVDYMGFKPGDETETRNIPRRADDDMPFSGLAFKIMNDPFVGSLTFTRIYSGVLKKGDTMLNSTKGKKERVGRMMMMHSINREEIDEAFAGDIIALAGLKDTTTGDTLCAVNDPVVLETMTFPDPVIEIAVEPKTKADQEKMSQGLARLAAEDPSFRVETDLESGQTIMKGMGELHLDILVDRLKREFKVEANIGAPQVAYRETVSREAEHTYTHKKQSGGSGQFAEVKMIISPTEPGEGFSFESRIVGGAVPKEYIPGVEKGVKSVMDSGPLAGFPVIDFKVALIDGKFHDVDSSVLAFEIAARQWMREAMKKAGAKLLEPIMKVEVVTPEEYTGGIIGDLTSRRGQVQGQDTRGNAIAIDAFVPLANMFGYINTLRSMSSGRAQFTMQFDHYEPVPQNISEEIQAKYA---MEFGFTTAA--YVVAAVLFILSLGGLSGQESAKRAVWYGIAGMALAVFATLI----GPGQGLWVLSVLLIIAGGAIGFVVAKRVQMTEMPQLVAAMHSLVGLAAVFVGYIAHIELARVLGMDPG-------------------------------ARAE-----LEGFAGL-LAHKDGIEISILRVELFLGIFIGAVTFTGSVIAYGKLAG-------KVGSAATK--LPG---------GHLLNATAALISLLCLVWYFNTGG--LLPLALMTLAALFIGYHLIMGIGGADMPVVVSMLNSYSGWAAAAIGFSLGNDLLIVVGALVGSSGAILSYIMCKAMNRSFISVILGGFGGTS-GPAMEVEGEQVAI-----DADGVATALNDADSVVIIPGYGMAVAQAQQAVSELTRKLRAAGKTVRFAIHPVAGRLPGHMNVLLAEAKVPYDIVLEMDEINDDFPDTDVAIVIGSNDIVNPAAQEDPNSPIAGMPVLEVWKAKQVFVSKRGQGTGYSGIENPLFYKENTRMFYGDAKASVDSLLSKID-----MRARIFQPARSATSSGMAKTRSWYLEFL-PADAREVDPLMGWTSSGDTQSQVRLKFDSCEQAQEYARDHGIDAVVMQSHKRRPNIRGGGYGENFATNRRTVWTHMGANDMLRQLSLRLKAAGQALRQPLPETSAGTTGAQADQAAVKPAKPAKAPPPVSPLAPKGGFPALPVIEGARFAAAAAGVRYTTG--RLDVALMELCAGSTVAAVFTRSATRAAPVLDGEEKLARIATGSAGSGD--AGFAIIVNAGNANAFTGAAGSGSVEAVSKAVGERLGIPAGHVFMSSTGVIGEPLKH-ERIVAVLDGLKSDLDGAALPRAAEAIRTTDTFAKGASAEVAIGGGTVRIAGIAKGSGMIAPDMATMLVYLFTDAPIDQPLLQAMVSRASDRTFNCITVDGDTSTSDTLICGATGKAKGVARLTDAESD-DARAFEKALHGVMLDLAHQVVRDGEGATKFVEVRVTGARSDEDARKVALATANSPLVKTALAGEDPNWGRIVMAVGKSGAEADRDRLAIRLGDLTIAEKGWVAEGYREEDGAAYMKRDELVIGIDLGLGDGESVVWTCDLTHQYITINADYRS---------------MLVGVVVTMGALAWAAVPFYDWFCRVTGFGGTTSVAEAGADRVLDQTIKVRFDGSLDRGMPWTFKPVQREIELKIGETAMAFYEAYNPTDRVVAGTATYNVTPYAAGGYFTKIDCFCFTQQVLQPGERVQMPLTFYVDPEIVEDADAKFVKVITLSYTFY----------ETELPEEQ-ASLALRPQEPTNKSLYRGIKWPTMLRIENISYSIEGRPLLHQASAVIPTGHKVGIVGRNGTGKTTLFKLIRGELSLDDGE--ITLPSRARIGGVAQEVPSSETSLLDTVLAADTERASLLAEAETATDPNRIAEVQTRLADIDAWSAEARAATILQGLGFDTKAQAMPCSAYSGGWRMRVALAAVLFSAPDFLLLDEPTNYLDLEGALWLETYLARYPHTVLIVSHDRGLLNRAVGSILHLEDRKLTLYPVPYDLFAQQRAQRLALAESEMRKQEARRQHMQAFVDRFRYTASKARQAQSRLKMLEKMQPISTPEEARSRSFTFPTPEE-LSPPIIALEQGVVGYDG-KPVLSNLNLRIDPDDRIALLGRNGEGKSTLSKMLAGRLELMDGKRVSSSKLRIGYFAQHQLDELDAGATPIAHVMRRLKDVPP-PKARARLAGFGLGAAQADTEVGRLSGGQKARLSLLMATLDNPHMLILDEPTNHLDIESREALVEALTAYTGAVILVSHDMHLLSMAADRLWLVQGGRVTPYEDDLEAYRKMLLSGDK----SQSGHNAKPEKPAASPDKQKQKAKRAP--RDRVLALRAEVRKCEERVEKLADMRQRIERRLADPKMYEKSTPDEIENWKRKYAEVVDGVARAEALWVSALEKLETAEA----------------------------MDRAQKEKLVEELGQIFESSGVVVVAHYEGLTVAEMQDLRARARDAGGAVRVAKNKLAKIALDGKPCASIADYLTGMTVLTYSEDPVAAAKVAEDFAKENKKFEILGGAMGENALDRAGVEAVSKMPSREELIATIAGMIGAPASNIAGAIGAPASNIASILSTIEERAEAA-------------MTDNFDDRKRTASAWFRQLRDRIVAAFEGLEDSQRDGPFAALPAGRFEMTETRRRSDDGSDAGGGLMSVMRGGRLFEKVGVNISTVHGELGERAQAAMAARKGIPGMKEDPR-FWASGISLVAHMQNPHTPSVHMNTRMFWTPHAWWFGGGTDLNPALEYD-----EDTAFFHKVLEEACAPHDAGYYPRFKEWADEYFYVPHRHRARGVGGIFYDDLNTSDWERDFAFTKAVGEAFLPAYQPLVEKRRTTAWSKDDKQRQLEHRGLYAEYNLVYDRGTKFGLETGHDATAVLMSLPPLASWP-----------------------------MSGPDLSFELRARAAGARCIIGVDEVGRGPIAGPVTAAAVCLDLDCIPEGLNDSKKLPQSRREALEVILTECAIV-SVAHATVREIEEVNILRASHLAMMRAVAGL-----------RFATGERPDHLLIDGRDLPRDCP---CRAEAIIGGDGISLSIAAASIMAKVARDRLMVDLAQQHPGYGWETNAGYPTKSHISALNDLGVTPHHRRSFRPVHNILYQEENLSC---------------MANELA-KTVVLVGMMGSGKTAIGTALAKRLDVPFLDSDAEIEVAASMSIAEIFARYGEPFFREKEEQVIARLLDGAPSVLSTGGGAFLSPRIRQLVSDRGVSVFLDADLPLLWSRVRHKTTRPLLRTDDPRRTLGELFRARAPVYALADLAVEARAEYSIADMTEAVADTLS--RAGIIKETT--------MEKTLFIFGHGYSAAALTRRLVGKGWTVHGTTRDAGRAEEIASAGAIPVVLNEGGGEPWLEGLARADHLLISAGPDADGDPVLNMAAQRIAALAPRIRWAGYLSTTGVYGDRGGDWVDEASALDPATARGRARVEAESGWQALAARSGLPLHIFRLAGIYGPGRGPFAKLRQGTARRIVKPGQVFSRIHVEDIARVLDASIAAPRPGAVYNVCDDDPAPPQDVIAHAARLLDLPVPPEVDFESAELSPMARSFYSESKRVSNRLMHEELGVALAYPDYRSGLAALLRDDPHG-------------------MAKVIGIDLGTTNSCVAIMDGSQPRVIENQEGARTTPSIVAYTDD-ERLVGQPAKRQAVTNPENTVFAVKRLIGRRADDPDLAKDLKNLPYAVVDGGNGDAWVQVHGEKFSPAQVSALILQKMKETAEKYLGEDVTQAVITVPAYFNDAQRQATKDAGKIAGLEVLRIINEPTAAALAYGLDKKDSKTIAVYDLGGGTFDITILEIDDGLFEVKSTNGDTFLGGEDFDMRIVNYLADEFKKENGVDLTKDKMALQRLKEAAEKAKIELSSSTQTEINQPFISMDPKGGQPLHMVMKLTRAKLESLVGDLIKASIKPCKEALKDAGLSTNDIDEIVLVGGMTRMPKVKEEVTKFFGKEPHQGVNPDEVVAMGAAIQAGVLQGDVKDVVLLDVTPLSLGIETLGGVFTRLIDRNTTIPTKKSQIFSTAEDNQNAVTIRVFQGEREMAADNKMLGQFNLENIPPAPRGMPQIEVTFDIDANGIVSVAAKDKGTGKEQTITIQASGGLSDDEIEQMVKDAEANADADKERRELVEARNQAESLIHSTEKSMEEHSDKVDPTTIEAIELAIAALKDDLEKDD--PAKIKSGIQNVTEAAMKLGEAIYKTQQESAA-GEAPEGESEESPRSVDDDIVDADFEDIDDDKRA--MQNPTAAMLAIGDEILSGRTQDANMHHLAQELTAAGIDLRECRVVADDHDAIVEAVRALSSRWDHVFTSGGIGPTHDDITADAIAAAFDRPIDVRADARALLAAHYAASGRELNAARLRMARIPDGAALIENPISTAPGFILENVHVMAGVPAIFRAMLASVLPGLTGGAPIQSRTLRVER-PEGDVAEVLGAVAAECPDLSIGSYPF-VEAGVLGTNLVLRGPDPDRLEAALAELKRRFGGLA----------------------------------------MTTPDHPERQNVSSEAASRDLPPAARRALAEAEERRRLAEAGEK--DRPVELGGR-EGPEPVRYGDWEKKGLAIDF--------------MFVTPAYAQAAAGGAAGAFTSFVPLILIFAIMYFLLIRPQQKKVKEHKAMVEALRRGDTVITQGGIIGKVVKVKE--GDEIEVEIAEGVKVRVLRSTVGQVLSKTEPAAS--------MPAPPEIFDRAAVRLHR---DRARHSGDPGALFLHDIAIDEIQQRLSEVKRTF----------------------TDIVIVTGHPETWAPAFPGA---RVIEDTATLEVAEGQADLVIHAMALHLANDPVGQIVQCGRALRPDGLFLAAAPGGETLTELRSTLAEAEIALSGGLSPRVSPMAEIRDMGGLLQRAGLALPVADAVKHRASYPSLTALMHDLRAMGEANALAGRLRHATRRALFERAQTLYQQHFPDTGTPPDG-----------APRLLATFEILFLTAWRPDSSQPRPARPGSASHSLAEALNAIDIQGLNGQSAGMTESERGQV---DE--MNIILLGPPGAGKGTQARILVEERGMIQLSTGDMLREAKSSGTEMGNLVADVMARGQLVTDEIVIGLIEEKLNAESG-GGFIFDGFPRTLAQADALGELLERKGQKLDAVIEMRVDDVALVRRITGRFTCGNCGEVYHDDTKPTAKEGVCDVCG-STDLQRRADDNEESLRQRLMEYYKKTSPLIGYYHAKGSLRAVDGLGEIDAVANEIRGILDRG------------MTPA-FAPRTADASDAIPVHLLDEGDIEARIAGWRESGQANLADWAEATKFTGKAGQVQVVRAATGQPLAVLAGHGTPATRRRGRFATAGIAAGLPAGTYRLEC--DLDG---PALEEAALGWLLSAYRFDRYKSMPPAEALLLAPDGVDAKRLEILAAAEFLTRDLVNTPASDLGPDALEAALRDLAEAHGAGVSVTAGDQLLTDNFPLIHTVGRAAAQAPRLLDMRWGSEG-PTLTLVGKGVCFDTGGLNIKPGTSMGLMKKDMGGAANVMGLASAIMALKLPLRLRVLIPAVENAIDGSAFRPGDILPSRKGLTVEVNNTDAEGRLVLADALALACEEPPEMLISMATLTGAARVAVGPDLAPFYTDDDDLARELSEAGGRMADPVWRLPFWDPYEALIEPETADLDNAPSGGFAGSITAALFLRRFVGEGQRYCHFDIFAWRPTGKPGLPKGGLGQGPRALLHTIERVLLA------------------------MDPHPAPLAPALYFVSTPIGSARDITLRALDILKSADVIAAEDTRNTRHLMEIHGISLGGRPLVAYHDHNGKAQRPRLIGYIKDGKSVAYVSDAGTPLVADPGYALGRAVIDAGLPVTAAPGPSAVLAALSVSGLPTDRFLFAGFPPPAAGARLSFLKELSKVPATLVFYESPNRLGKLLAAMVETLGGDRPAAICRELTKRFEETRRGTLDELVAALPEMT-LKGEIVVLCGRPGETRADAEDVEAALRRAMETSRLKDAAREVAEALNLPRREVYQIGLTLKSPE-----MRLFVGLGNPGAKYAANRHNIGWMAVERIAEDHGFAPWRAKFQGEMTEGRLGAEKVVLLKPGTFMNLSGQSVGEAMRFFKLSPEDIVVFHDEIDLAPGKLRCKTGGGHAGHNGLRSLHQHVGPDYHRVRMGVGHPGHKDRVPGYVLSDFAKAEAGWLDDMLRGVSDGAPALAEGDTGAFTNAVARRLAPPRPSTGT-------KGPAAAKQAPKTTGDAAAAPGARPVKAAPPAPDKED--------AAPAPRSALQRLADKFR--MPDLLLELFSEEIPARMQQRASEDLRKLVADGLVEAGLTYGSAASFSTPRRLALTIEGLTDKSPTLREERKGPRTDAPEKALEGFLRSTGLS--REDLETRADKKGEVFFAVIEKPGRPAEEIVAEVVAAAIHNFPWPKSMRWGSGSLRWVRPLHSILCILHDEGG-ARVVDPQIADVPAGDSTAGHRFMAPARFTVSSFEDYAVKLK-KAHVVLDPAERADHIRADAENAAFAQGLELVEDKGLLAEVAGLVEWPVVLMGDIGADFLDLPPEVLQTSMKEHQKFFSVRNPSSGRIE-KFVTVANRETADQGATILAGNQKVLAARLSDAKFFWENDLRVARA----GMGDWLESLANVTFHNKLGSQAQRISRIAALAREIAPLVGAEPDLAERAAQVAKADLASEMVYEFPELQGVMARYYAAQAGLAPEVAAATQEHYAPLGPSDDVPSAPVSVAVALADKLDTLAGFWAIDEKPTGSKDPFALRRAALGVIRLVLENDLRVSLTGNDGLLRLAAD-------------------------------------------------------------------------------------------GVGARKTDNGEQSAD----------------------------------------INDLLSFIHDRLKVYLRDRGIRHDVIDASLAMPGSDDLTLLVKRAQALSDFLGTEDGTNLLQGFKRANNILTQAEEKDGVEYSYGADLKLAEG--PEEKALFVALDQAEPQIRDAMQAEDFAAAMGAMAALRAPVDAFFEAVQVNADSAIIRRNRLNLLHRIRAVCSGVADLTRLEG----MALV----RRVS---SAVTLVSVL--ALAGCGDSGGLGLGDLFAT----APEASDEALTPEALFEKAEFQLSSSEPDRAATTFAEVERLYPYSELAKRAIIMQAFAYHQDKDYENSRASAQRYIEFYPADEDAAYAQYLLALSYYDQIDEVGRDQGLTFQALQALRTVIERYPDSEYARSAILKFDLAFDHLAAKEMEIGRYYLKRGHYAAAINRFRVVVEEFQTTTQTAEALHRLVEAYLSLGLDAEAQTAGAILGHNFKSTEWYEDSYRLLTGRGLKPQSIGDNWLSAIYRQVVRGEWLMPIYRDLPDAIGHTPLFRLNGASEATGCEVLGKAEFLNPGQSVKDRAALYIIRDAIARGTLRPGGTIVEGTAGNTGIGLALVGAAMGFRTVIVIPETQSEEKKDMIRLAGAELVQVPAAPYRDPNNYVRYSERLAARLAETEPDGAVWANQFDNVANRQAHVETTAPEIWEDTDGQVDGFICAVGSGGTLAGVAEGLRARKADVKIGIADPMGAALYHYYTDGELK-SEGSSITEGIGQGRITTNLEGLKVDYAYQVPDDEAVQVVFDLLRDEGLCLGGSSGINVAGAIRMARDMGPGHRIVTVLCDYGTRYQSKLFNPAFLKEKGLPVPEWLDRAPVVMPDVFEKE--MFTVSLLTDPAGAPLDPALVEALRNAWGGGDVLWLNPRCAAEFGLAHAP-----QDFERHWAELQSMK-TDLAIQPTAN-REKRLLIADMDSTMIRQECIDELADMAGVGAHVADITARAMNGELDFEEALRERVGLLKGLDRAVIGQVLRERITLMPGGATLLATMAARGHHSILVSGGFTDFAAEIARQLGFAEHRANVLLAEDETLSGLVQEPILGREAKVTALQETAAKLGLTPDDAIAVGDGANDLGMLQLAGTGVALHAKPAVAAQAKVRINHGDLSALLYLQGYASDTHVTA------------MTDESPDLEEREDAGYALDKATVHAIHEALLSDDDAALRALIDPLHPADVADLLEQVSGTERRQLLTRWSGSMAGEILSELDESLREEVIEYLEPGILAEAVRELDSDDVVDLVEDLEQDQQDLILDSLSDADRVAVELSLSYPEESAGRLMQREVVRVPEDWTVGETIDFLRRE--TQLPEQFYHVILVDPRMRPTGYVTLGKLLGSPRAAPLSDIVEESFRTIPVSQPEEDVAYAFNQYHLISAPVVDEDDRLVGVITIDDAMAVLDEEAEEDLLRLAGV-GDERVSDRTLKIARQRFPWLFVNLATAILASLVISQFEAALTQVVALAVLMPIVASMGGNAGTQSLTVAVRALATRDLTGSNAVRVLTREAVAGLLNGAAFALIMGVIAILWFDTPILGAVIAISMVVNLVVAALAGILVPLALEKVGIDPALASGAFVTTVTDVVGFFVFLGLAVILMLMAIKVLMPALSPTMEEGTVAKWLVKEGDKVSSGDILAEIETDKATMEFEAVDEGTLGRILVAEGTEGVAVNTPIAILLEEGESADDAQMDDAGDAEPQAASATPKAKEEQPTQA----PDTPERSPAAPRTPA---PSAEPDYPEGTETRSMTVREALRDAMAEEMRRDETVFLMGEEVAEYQGAYKVSQGLLDEFKEKRVIDTPITEHGFAGIGVGAAFGGLRPIVEFMTFNFALQAIDHIINSAAKTLYMSGGQMGCPIVFRGANGAAARVGAQHSHDFAAWYSSVPGLKVVMPYTAADAKGLLKTAIRDPNPVVFLENEILYGRSFDVPVLDDYTVPFGKARIARPGNDVTIVSFGIGMQYALEAAEKLAEEGIDAEVIDLRTLRPLDMDTVLNSVRKTNRCITVEEGFPVCSIGSYLSSRIMTEAFDHLDAPVLNCTGKDVPMPYAANLEKLALVTTQEVVDVAKQVTYR-MFAVLKTGGKQYKVQSGDVLRVEKLAAQAGDTVQFNDIMMLG-GDKPVVGAPLISGAAVQAEVIDQIKGEKVIHFVKRRRKHSSKRTKGHRQQLTLLRVTEILAEGGDKSGVKAAVGAGSVAGVAAAAA-------APKTPAKKAAAP-------KAEKPKAEKKAAA------PKAEKKAAAPKA---AKADDKA----------------DDLKKLSGVGPALEKKLHEAGVTTFAQIAGWSAADIAEMDEKLSFKGRIEREGWVDQAKELTKG---------MVD-HINLVKLCVGAKSVEDLVAWQKTKRSRN-ADGLPCHVTRMWPKREAELLAGGSIFWVMKGLIQARQRISSLEERVGADGIRRCAIVLDPRIIRTEAAPRRAFQGWRYLPVDQAPRDLPEGRASEASLPPALLAALAEIGVRMADPISDTDDLTRIDG-----SRIVVSVDAMGGDRGPEVVVAGLALAARGNPELHFILHGPEATLSPMVGRH----GIADRCTLRDAPGVVSMDEKPSHVMRKGKDTSMWSTIESVRAHEADVAVSCGNTGALMAVSMVRLRKLPGVNRPAIACLWPSRNPGGFNVMLDVGADIRADADDLLQYALMGASYARNGLDLACPRIGLLNVGTEEHKGRAELKAAHELLQSAAAAGNFDYVGFVEGGDIPSARVDVIVTDGFTGNVALKTGEGTAMLIRDFLKESLTSTIVSKLAALLAMKSLKRLSQRIDPRQVNGGVFLGLNGTVVKSHGSADATGVEAAIKLAAALSKSGFSERVAARVASFNAAREGATADDADNKPGTLHD---MPMEKTFDSAEAEARLYDKWESGGAFAAGANAKPGAEAFSIVIPPPNVTGSLHMGHAFNNTLQDILVRWHRMRGFDTLWQPGQDHAGIATQMVVERKLAAEGQPPRRELGREAFLDKVWEWKDESGGTIINQLKRLGASCDWSRNAFTMDE-------------HFQKAVIKVFVDMYDKGLIYRGKRLVNWDPHFETAISDLEVENIETPGHMWHFKYPLA------SGETYEYVERDEDGNVTLRETRDYISIATTRPETMLGDGAVAVHPSDERYAAIVGKLCEIPVGPKEYRRLIPIITDEYPDKNFGSGAVKITGAHDFNDYQVARRNNIPMYALMDTKGAMRADGRPYSDEAETAQRIA----------RGE----EEFDENKIAAMNLVPDELRGLDRFEAREKVIEAITAEGLAVMVPLNDPRLGKAA-KKPVAPEEGGEAREDQL----VPFVEAKAIMQPFGDRSKVVIEPMLTDQWFVDTDKIVGPALDAVRTGMDRPAGSTEGTKILPERDAKTYFHWLENIEPWCISRQLWWGHQIPVWYGPQLIDGH-----VDAEA----------------------------------EWRPFCAATPEEALSQMAAYYG----------------HDDVRLVEDRTEA----LSLLAGLAPDTRN-------------EGVIRSPQGPVAIPAYRDPDVLDTWFSSGLWPIGTLGWPEQT------PELAKYFPTSVLVTGFDIIFFWVARMMMMQYAVVDEKPFHTVYVHALVRDEKGKKMSKSLGNVLDPLELIDEYGADAVRFTLTAMAAMGRDLKLSTQRIAGYRNFGTKLWNAARYAEMNGVFGDDVPAPG--AMPP-----AASQTLNRWIIGETARVREEVDAALGNYRFNDAATALYAFVWGKVCDWYVEFSKPLLLGED--AAAAAETRATMRWVIDQCLVLLHPIMPFITEELWDTLAS----RDTMLVHADWPTYGTEALLDPEADREMNWVISLIEAIRSIRGEMHVPVGAKLEMIQLDLDAAGQAAWDRNAGLIQRLARIESLSKAEAAPKGAVTVAVEGGTFCLPLADVIDIAAERERLEKTLGKLAKEKGGLAGRLKNPKFVDSAPAEVVAETRELLAQKDEEESRLRAALDRLAALG-MTDKPHDADVAFIQALAELLRENDLTELEVRRDYGEDDSLKVRVSRRKEVVSH----VQAAPAPAQAPAAAPAPAAAPAAAPA-APAASDDPAQHPGAVTSPMVGTVYLQGEPGAPAFVSVGDKVSEGQTLLIIEAMKTMNQIPAPRAGTVKRIVVEDGAAVEFGAPLMIIE------------------------MFRDALERSAGAVRDRLQQAILAQPGGE---LRDAMAYATR-GGKGLRGFLVVEGARLHGLNGAGPLNAGAAVEALHAYSLVHDDLPCMDDDDLRRGQPTVHVKWNEATATLAGDALQTLAFELLARPETAADPAIRIALVARLAAASGAAGMVHGQALDIAAETAATPLDLAQITRLQQAKTGALIEWSATAGAVLA---GADPAPLARYSRALGLAFQLADDILDIEGDADAAGKRLRKDASAGKATFVSLLGLEGARSRARELVAEAEAALMPFGADGETLRQAARFVIERDS---MPTQLPVVTPDLPEAQTFDNAEAAVARLEELYAGGTRFLAERFTEAAAQH-SPTRRYRA---Y-YPEIRLTTTSYVQVDS-RLSYGHVSQPGSYSTTVTRPDLFRQYLIQQIGLLMRNHRVPVRIGLSDTPIPLHFAVAGDPDLSVPQEGAMAFPLRDVFDVPDLNTTNDAIVNGYGFTHPD-GSGPLAPFTAQRIDYSLARLEHYTATRAEHFQNHVLFTNYQFYVEEFEAYARMKLDEDDSPYVAFVGPGNQEITEPHSPL-----PGPEKLPQMPAYHLKRADGQGITLVNIGVGPSNAKTATDHIAVLRPHAWLMVGHCAGLRNSQRLGDFVLAHAYLREDHVLDDDLPVWVPIPALAEIQIALENAVEQVTELTGFELKRIMRTGTVATIDNRNWELRDQSGPVQRLSQSRAIALDMESATIAANGFRFRVPYGTLLCVSDKPLHGELKLPGMASDFYRTQVLRHLLIGIRAMEELSEMPLERLHSRKLRSFEETAFLMNLEKFTERSRGFLQAAQTIAMRESHQKLAPEHLLKALLDDEEGLATNLIRRAGGEPERVVESVELALGKLPKVTG-DAGQTYMDQQTGKVLDEAEKIAKKAGDSYVAVERILTALA-VVKSKAREALEAGAITAQKLNTAINDVRKGRTADSANAEEGYDALKKYARDLTEAAEQGRIDPIIGRDEEIRRTMQVLSRRTKNNPVLIGEPGVGKTAIAEGLALRIVNGDVPESLRNKRLMALDMGSLIAGAKYRGEFEERLKAILKEIEAAAGEIILFIDEMHTLVGAGKTDGAMDASNLLKPALARGELHCVGATTLDEYRKHVEKDAALARRFQPVMVEEPTVEDTISILRGIKEKYELHHGVRISDAALVAASTLSHRYITDRFLPDKAIDLVDEAASRLRMEVDSKPEALDALDRDILQKQIEAEALKKEDDDASKGRLEKLEKELSDLMERSSEMTAKWQAERDKLESARTVKERLDRARAELEQAKREGNLQKAGELSYGVIPDLERQVKGSDGGE--AEDMMVEEAVRPEQIAQVVERWTGIPTSKMLEGEREKLLKMEEQLGRRVVGQDRAVKAVANAVRRARAGLNDENRPLGSFLFLGPTGVGKTELTKAVAEYLFDDDSAMVRIDMSEFMEKHAVSRLIGAPPGYVGYDEGGVLTEAVRRRPYQVVLFDEVEKAHPDVFNVLLQVLDDGVLTDGQGRTVDFKQTLIVLTSNLGSHALGQQAEGEDSAAAHAAVMEAVRGHFRPEFLNRLDETIIFDRLSREDMTGIVDIQLERLTKRLAARNITLEMDEAARKWLADEGYDPVFGARPLKRVIQRALQDQLAEMILSGDVHDGDTIPVSAGTDGLVVGDRVATSNRRPPSEAVVHMTADRPTP-DAPLKIGTRGSPLALAQAHETRDRLARAFDLDPAAFEVVVIKTTGDIV----LDRPLKEIGGKGLFTKEIEEAMLSGAIDLAVHSMKDMPVEQPAGLVLDTYLPREDPRDAFVSREVSAIAELPQGATVGSSSLRRRAQLAARRPDLNLVEFRGNVQTRMRKLDDGVALATFLAMAGLNRLGM-AEVAKGAIDTEEMLPAVAQGAIGIERRAD-DHRVAAMLEAIHDADTALQLAAERAFLAALDGSCETPIAGLARLEGGTLTLRGEILRPDGSDVLDDKAEAPAEDGAA----LGREMGLRLLERGG---PGFFDWRG------MQIEKEITEEEDAAGDGA----AGLPPRSVRRWVASRKAAVVHAVLGGKLTAEQACRSYDLSGEELQGWIAAVRNHGEGALKATSLRKFR------------MNDALIILAAGQGSRMNSELPKVLHQVAGAPLLVHAMESGAVLEPSRTVIVAGHGAEAVEAAARDHDPDVTVVRQEEQLGTAHAVDQARAALSDFDGDAYVLYGDTPFIRPETLSRMAEARAAGSAVVVLGFRAGD-PGRYGRLVM-NGDKLERIVEFKDATDEERRIDLCNSGVVCADAATLFELISEVGNDNGSGEYYLTDIVAVARKRGLGTTVVTCDETETMGVNTRADLAAAERAFQDRARAALQEDGVTLTAPETVHLSRDTHVGRDAVIEPNVVFGPGVTVESGATIRAFSHLEGCHVGAGAIVGPYARLRPGAELGNDVRIGNFVEVKNATFGEGAKANHLAYVGDSTVGDGANIGAGTITCNYDGVMKHHTHIGAGAFIGSNTALVAPVRVGDGAMTGSGSVVTTDVPDGALALGRAKQVNKPGLALRIMQNLRAIKARKSSSGE--MTRIDAKFAELRKAGRKAFVTYVMAGDPDYERSLEVVRGLPGAGVDIIELGLPFTDPMADGGTIQLAGQRALEAGQTLARTLELAHAFREQDDTTPIVLMGYYNPIYSRGVERFLAEAKEAGIDGLIVVDLPPEEDDELCIPAQKAGLNFIRLATPTTDDKRLPKVLTNTSGFVYYVSITGITGSAAAEAGDVGPEVARIKAATDLPVIVGFGIRTPEAAERIASVADGAVVGSAIVDRIGKGEPTEKVLEFVAGLAE----------GAHRG---MGSRRVLGIDPGLGNLGWGLIDVDGPRITHVANGTCKSS-GKDLAVRLLSLFDQLSAVVTAHSPDVAAVEQTFVNKDGAGTLKLGQARGVAMLVPARAGLPVSEYAPNAVKKTVVGVGHADKRQVLHMVRLQLPGIEIDGPDAADALAIAICHAHHGGAQAPGNDTLARAIARADGRAASGVRA-----------------MTQRKGIFSGSDPFAIARGWLQEATRTEPNDPNAMALATVDADGLPNVRMVLLKEIEAEAFVFYTNYQSAKGEELALNPKAAFVLHWKTLRRQIRVRGIVSREDGKDADAYFASRSVKSRLGAWASAQSRPLSGRAEMLAKVAQYAAKYGTSP--PRPPYWGGFRIRPVEIEFWADGEFRLHDRFRWRRNPD--ATGQWKIERLNPMRHLTKTVLLLGAL-TLAACSGNPFNRGNG-------PVGAN--AGAGYGA-G-NPAGTVSPNSVEFFQQNVGDRVLFAVDQSTLSPEARTTLAGQSQWLMQNIEYAVVIEGHADEQGTREYNLALGARRAASVRDFLVSQGINANRVRTVSYGKERPIEICSTEACYSKNRRAVTVVTAG-ASS-------MTVTEEIRNRLEAGLSPRHLEIEDQSEAHRGHAGFREGGESHFHVEIHAAQFGGMSRIARHRAVHEALGKDLMGRIHALSLTLEE--------MTKPVAVSFDTLDTDAIQKTQGRLAVFATCDGK-LDRAGRRVNRLTRGALERFLASEGFEKLKEGEGHGFAYPSGMEAEAVMLV--KLDRRPKAPAPARKAGATLAK--FM-AGAPLTVLADGKYALADVAYGVALRAYAFTDH----KTRDDNGAAEARDARVLCTEPEAEAERFADMRAVAEGVFLTRDLVSAPSNILTTTSFAERLQKLEALGAKVEILEEPELEKLGMGALLGVGQGSDSPSKVAVIEWQGGEKGGAPFALVGKGVVFDTGGISLKPAAGMEDMTMDMGGAGVVAGVMHALASRKARANVVALIGLVENMPDGKAQRPGDVVTSMKGDTIEVINTDAEGRLVLADVLWYAQERFKPSGIVDLATLTGAIIIGLGHENAGVFSNDDTLCNAFLKAAGDEGEGAWRMPLSDGYDKLIKSDIADIKNVGGRAAGSITAAQFLQRFVKPDVPWIHLDIAGTASVKTE-TDFAPKGATGWGVRALDRMIRDYYEG------MTIYVDADACPVKAEAEAVATRHRTPVKFVSNGGLRPSANPLVEMVYVPEGPDLADMWIADRAGPGDVVVTGDIPLAAKCVEAGARVLRHNGEALTRANIGQTLATRDLMADLRAADPFRQ-GGGKGFGKADRSRFLDALERALRAAAQDLADGAVPRGQDGEQGE---------MTDQNHDRLLIIDFGSQVTQLIARRLRELNVYCEIHPFQNVTDDFLEDFAPRAVILSGGPASVIDTDSPRPPARVFEMGVPVLGICYGQQVMMQMLGGEVLRGH-----GTAEFGRAYVQPEEDRIDLLTGWFL--EQREQVWMSHGDHVSRLAPGFAVYGTSPNAPFAVTADLSRNFFAVQFHPEVHHTPNGRSLFQNFVR-LAGFKGDWTMDAYRDEAIRQIREQVGDAKVICGLSGGVDSSVAAVLIHEAIGDQLTCVFVDHGLLRQGEAEEVVTMFRDHYNMQLIHADEQELFLGELDGVSDPETKRKIIGRLFIDVFQKHANEVGGAKFLAQGTLYPDVIESVSFSGGPSVTIKSHHNVGGLPEKMGLKLVEPLRELFKDEVRALGRELGLPDAFIGRHPFPGPGLAIRCPGEITREKLAILRKADAVYIDQIRRHGLYDEIWQAFVAILPVRTVGVMGDGRTYDFACALRAVTSVDGMTADYYPFSHDFLGETATRIINEVPGINRVTYDITSKPPGTIEWEMSHRSHPHRSVLYIPGSKPRALEKARGLPVDAIIFDLEDAVAPDEKAQARETLAEALVAGGYGSRVRLVRINGFDTEWGEADLARMADVGADGILLPKVGRPEHVADLRQRMAAAGYPD-TPVWAMIETCEGVLNAAAIAGAE---GIAGFVLGTNDLAKELGCRNRADRLPLMTALQHSLLAARCHGLVALDGVYNAFRDEEGLRAECEQGRDLGFDGKTLIHPGQVAITNQVFAPSEAEVDLARRQIAAFEAACAEGQGVAVVDGSIVENLHVETARKTLAAADAIAAMEGQDQ--------------MNVPASRGRDGPTPDKAADGAEAAPEADLELSEPSDDLPVALTSGDPAGIGPEIAVAARLALGAEV--PFFYIGDPAHLPEGT-ALAEIAAPAEAL-LVAPDTLPVLVQEFAAPARPGTLDPANAAGTIAAIRRAVELVRDGQASAVCTNPIHKKALKDGADFPYPGHTEFLAALAGID-RV----VMMLASDALRVVPVTIHIPLSEVTAALTPELLEETLRITHAGLIRDFGIAAPRIAVAGLNPHAGEGGAMGHEESTLIAPVLDRLRAEGMELSGPASADTMFHAPARARYDAAVAMYHDQALIPIKTLDFDRGVNVTLGLPFVRTSPDHGTALDIAGQGVASASSLVEALRMAHRMGRNRRQAA---------MLYWLTELSDGGDLFNLFRYITFRAGGAFFTALVFGFLFGRPLINLLKRRQRNGQPIREDGPESHLLTKAGTPTMGGVLILGAIVVATVLWARWDT---PYVWIVLFVTMSFGAVGFVDDYQKITQSHAAGVPGRVRLAIGFVIAGIAAY-------------------------LASLVHP-------------AELTNQLAVPVFKDALLNLSWFFLPFAMFVIVGSANAVNLTDGLDGLAIMPVMIASAALGVIAYAVGRVDFSEYLDVHYVPGTGEILIFCSALIGGGLGFLWYNAPPAAVFMGDTGSLALGGALGAIAVATKHEIVLAIVGGLFVVEALSVIIQVLYFKRT------GKRVFLMAPIHHHFEKKGWAEPQIVIRFWIISLILALIGLATLKLRMPMQAREIEALIRASFPDARITVTDLAGDGNHYAAEVIDASFKGQNRVQQQRAVYAALKGKMDGNNGELHALALTTKAPD--------------------------------------------MRHTLPIA--------PQFYVTAPQPCPYLDGRMERKLFTALQGDYAGKLNDSLSKQGFRRSQNVLYRPSCSDCSACLSARIRVADFKPSRSQRRTLNRNRSLRREATSPWATEDQYALFRSYLEARHASGGMADMDIFEFAAMIEETPVRSRVVEYTQPADPATGSDQR--LAAVCLTDVMDDGLSLVYSFYDPNMTERSLGTYVILDHIEIAREAGLPYVYMGYWVPGSSKMGYKARFDPVEIYHEGEWKELSDPATF-EADPHPLSTDPIAEQVARINLPDSRPTD---------MTRLTAEFWVDAYMRRLQLADIPAYLMRRGDATAGAVLVRLDTMDGQARVFQRSFDLMTGERRWVVLA----EGPEAEVGQTLARQAERDPDLWIVEIEDRQGRHML------DQPGLDG-MKELLRSNDPTVIAFASALLKGEDIETFQMDVHMSVLEGSIGVLPRRLMVRGEDLHLARAILRDNDLHPSAG--MSDGTDNTAGPERRSARVSSPCVKLCLVHPVEGICTGCLRTLEEITGWSQLTEAERRTIMKELPARAPRLAKRRGGRAAKLNRLG----------MTHFVVVGAGQAGASLVARLRAKGFEGRITLVGDEDAPPYQRPPLSKGYLLGEISRERLFLRPERFYGEQKIDLLTGRKVTAIDTSVRSLTLA-GPDGAEESLSYDALALTTGSRPRHLPAAIGGALKGVHVVRTLADVDAMAPEFAEGRRALIVGGGYIGLEAAAVACKLGVAVTVVEMGERILQRVAAPETSERFRQLHAAHGVDLREGVGLERLTEGEADAGDGRGDG-RVHAAQLTDGSTLEVDFVIVGVGILPETELAEAAGLAVEDGIRVDALGRSSAPDVWAAGDCANFP--LGEGRLRLESVQNAIDQAEAVADNMMGAETPYVPRPWFWSDQYDAKLQIAGLNTGYDRVVVRAAPEA-AEDLSAPVSYWYFRGDTLLAVDAINDPRSYMVGKRLIEAGRSPEPAQVARHDLPVKELLG-----MSLTERLGLPLLRRIDPELAHGLSIRALESGLSGMLTGNAGPVTSDRLRVRLAGMDLANPVGLAAGFDKNAQAIPALARAGFGFVELGAATPRPQDGNPRPRLFRLPEDRAAINRFGFNNQGAEVIAGRLEASRPAATDAGVPVGLNLGANKDSADRAADFGRVLATCGAHVDFATVNVSSPNTERLRDLQGAEALAALLS--QVLAVRDGLERRVPVFLKIAPDLTAEELADIARVAREVA--IDGIIATNTTLDRE-GLRSPHRDERGGLSGAPLFEKSTRVLAHLSHLTEGNIPLIGVGGISTAEQALAKIRAGATAVQLYTAMVYGGLSLAREIATGLDRLLEQRGFASVAEAVGTGRDEWLL--MARERLSVVVTRRLPEAVETRMRELFDARLREDDTPMGREELAGAMRGADVLVSTVTDTIDAALLAQAGERLKLIANYGAGVDHIDVQTARQRGVLVSNTPGVVTDDTADMTMALILAVTRRIPEGLALMQAGAWTGWSPTALLGGRVGGRRLGILGMGRIGQAVARRAKAFGMQIHYHNRRRLRPETETELDATWWESLDQMVSRMDILSINCPHTPSTFHLMNARRLALMKPTAVVVNTSRGEVVDENALTRMLRSGAIAGAGLDVYERRQDINPRLRELNNVVLLPHMGSATREGRAEMGEKVLINIKTFADGHRPPDLVVPSMLMSRIGKKPVELPSGVSATVSGQTVEVKGPKGTRSFTATDDVTISVDDNVISIAPR---------------GSSKR-----------ARQQWGMSRTMVGNLVTGVSTGFKKELEINGVGYRAQMQGN-TLKLSLGLSHDVNFEVPEGVTVTAPKQTEIVVEGIDQQLVGQVAANIREWRKPEPYKGKGIKYKDEYIFRKEGKKK--MRMLGLKTCDTCRKALRALAADGHEITLRDVRQEPLSPDEIARFEGRFG-EALVNRKSTTWRALSDEERARPA----TELLADHPALMKRPVITDGDRMTLGWTGDVREVWLG---MPRYALKIEYDGGPFSGWQRQVAFPSVQGAIEAALARIAPEKPLIQGAGRTDAGVHAHAQVAHVDLAREWDPFRLAGAINYHLKPAPVAILDVAPVADDFHARFSASGRRYIFRLLSRRAPVTLERGQVWQVGHPLELAPMQEAARLLLGHHDFTTFRSSTCQAASPMKTLDRLEITRRDLTVGAEFTFDVRARSFLHNQVRSFVGTLERVGVGAWHPEDVARALEARDRSACGPVSPPQGLYLAGVDYPRDPFASAAS---MDKSSSQNRAYQHLYPLIDPFDQKMLNVGDGHRIYVEQCGNPQGVPVVVLHGGPGGGCSPTMRRYFDPAVYRIILFDQRGCGRSRPHASVTANTTWHLVRDIETIRERLDIDRWIVFGGSWGATLALAYAETHPDRAAFLALRGVFLMTRSELDWFYGGGAGHFWPDLWADFLSLLPEAERGDPIAGYNRRLFSGDLTTEIRFGRAWAAWENALASINSDGRGGESP----PEYARAFSRLENHYFINGGFFEADGQLL--DNIGLLRNTPAAVVQGRYDMICPPRAAYDLASAMPMARLHMVTRAGHALSEPGISAELVRVMDGLRDDLGALGLATHA---MLIYKIFRPAEWQALSSDGRTDGAPVDLADGYIHFSTAEQAQETAAKHFAGAGELVLAAVDADRLGESLRWEPSRGGALFPHLYRPLELTDVL---WHRPLPERDGQHHFPAEMT-------------MRSRRGLLLILSSPSGAGKSTLANRLRNWDPDIVFSVSATTRAPRPGELDGREYYFRSRESFLAMVEDGDMLEHAEVFGNLYGSPQGPVEEAIVEGRDVLFDIDWQGGQQIRNSALAEDVVSIFILPPSIAELERRLRSRDQDSDEVIAARMQKSRDEISHWAEYDYVLVNRDLDETEMKLRAILQAERLRRSRQVGLVDLVRELNKEFGDR---MSIRLGVPSKGRLMEKTFDWFGERGVDIRRTGSDREYAGAVGGIDGVELVLLSAGEIPRELAAGRIHLGVTGSDLVREKLPRWDRLVQELAPLGFGHADLVIAVPKFWVDVDTLDDLDAAAAAFRARHGHRLRLATKYHRLVREYLRRAGVADYQLVDSQGATEGTVKHGTAEAIADITSSGETLRANHLKILEEAPIHQSQATLFRSLTAPTDEGQDRTLDALSRTLGLAG-------------MMAGPASDTPRKAP-HIPVLLRPILGIAAPVSG-TWLDGTFGAGGYARGLLDAGAERVIAVDQDPMVFEMARAWAGEYGATEEGGEDSRLVFRHGNFANLDEYAQD-----LDGIVLDLGVSSMQLDMADRGFSFSRSGPLDMRMSQSGPTAADLVNTAEESELADILYGYGEERASRRIARAIVAKR---PFTTTGELAEVIQSVLPRPKPGQSHPATRSFQAIRIAVNDELGALVRGMEAAERALRPGGVLAIVTFHSIEDRIVKRFFQAGTGKAGRANRYAPEAPEQV-ATFENLTRRAVAPDEDELADNPRARSARLRMGRRTEAPARAPDRGN-LGVPGIARLDDKDSERSGQMPDRRIIKEITLGSEAATLAFATALAPMLRAGDTLLLEGPVGAGKSFFARSVIQARLAALGRS-EDVPSPTFTLVQVYDLDDVE-LWHCDLYRLTDPAEAYELGLDEAFDQAICLVEWPGRLGGLQPEGALRLDLRADAGEDGNPDRRIATLSGSAEWARRLETEE----------------------------MAGITDLPFRTLVRRFGAGLVVSEMVGSQEMVQAKPSVRAKAELGLGVEGTAVQIAGREAHWMAEAARMAEAGGARIIDINMGCPAKKVTSGYSGSALMKTPDHALSLIEAVVNAVSV-PVTLKTRLGWDDASLNAAPLARRAQEAGVQMVTIHGRTRCQFYKGTADWAAIADVVQALDLPVIANGDIVDTATARKALSLSGAGGVMVGRGARGRPWQLAAIAAGLEGRGQCPEPQGHDLADLVAEHHAAILSFYGQELGMKNARKHLGWYLDAAGQ--GAHPERHALLTE----THPDRIAPRLRRILAEAPVE----VAA---MSRP----P---LTLYLAAPRGFCAGVDRAIKIVEMALEKWGAPVYVRHEIVHNRYVVDGLRDKGAVFVEELSDCPPDRPVIFSAHGVPKSVPAEAREREMVFVDATCPLVSKVHIEAERHAEAGRQMVMIGHQGHPETIGTMGQLPEGEVLLVETAEDVDRLEVRDPDQLAFITQTTLSVDDTAGIVEALKRRFPAIVGPHKEDICYATTNRQQAVKAVAPKVDAMLVIGAPNSSNSRRLVEVGSAAGCAYSQLVQRAADIDWRALGDIRSVGITAGASAPEVLIEEVINAFRDRYDVTVELVETARENVEFKVPRVLREPA---------------------------------------------------MTSGAAALARLCDPGAEVSCHYLVHEDGAVRQLVDEEERAWHAGAGAWGAVGDVNSRSLGIEIVNRGDH---------PFAAHQMAALIALLGGILSRHSIPPERVIGHSDMAPGRKADPGRRFDWRGLAREGLSVWPGAGAAIEAPAQ----DLPALFRARARAFGYP---------DV-ADAPLLEAFRQRFRPWD-HG---PLCAADLSAIDDLARRFPVDRPPTRA---MLRRRSKLRIETERMILRLPQHGDFAAWANLRRESASFLQPWEPAWSTDHLSRKAFVHRVHWAHRAALDGSAMPLFLIGR-DETFYGALTLDNIRRGPAQAGTLGYWIGERHHRQGLMREAIEAVVHYAFTNLEVSRLEAACLPENVASRGVLERSGFKYEGVAQSYLQINGRWRNHVLYACLRTDRRGKTVVGLE----MTTK-RSIFEDVPAES-SPRGRPQGEGPGPGLIDRGTGRGRRATRIWLFILFAMVVVMIAVGGLTRLTDSGLSITEWRPVSGALPPMSIEAWQVEFDKYRATPEYRLQNQGMSMAEFQFIYWWEWGHRQLGRAIGLVWALGLGLLWATGRIPRDWTGRLLLLGALGGAQGAVGWWMVSSGL--EGSMLDVASYRLATHLGLAFVILGVIAWFAFKLGRQSGDLLQARRHGEAKLFGMSTGLMHFAFLQILLGALVAGIDAGRTYTDWPLMAGEFLPPDPLDLAPVWRNFFENEGLVQFLHRMAGYLLFIYGLVVWRRSRSSGNDGTRRAFDMMAGMMVVQVLLGIATVIAAAPWHLGLLHQLGAVTLWVLILRARFLSRYPQRQNLRGKGATAMTERFRLTLAQLNPTVGDFAGNSAKALEAWRAGRDAGADLVALPEMFLSGYQLQDLVLRPAFLARSEEALEELARACADGPPLAIGAPYMEGLRAYNSYAVLEGGRIATRVLKHHLPNADVFDEVRVFGSGPISGPYRV--GPMRIGSPICEDAWFDDVAETMAESAAEMLLVPNGSPYHRGKHEVRQNLMVARVVETGLPLVYLNLVGGQDDQVFDGASFVLNP-GGGLAVQMPAMEEAIQHVDFTLGDDGWRAEQGPRASLPDPWEADYKAMVLALRDYCGKTGFGKVLLGLSGGVDSALVAAIATDALGAENVRCVMLPSRYTSQHSLQDAAACARALGTRLDELPITEAQNAVNATLAPLFAGRDEDLTEENIQSRLRGVMLMALSNKFGEMLLTTGNKSEVAVGYATIY-GDMAGGYNPIKDLYKMRVFETCRWRNANHR-DWMQGPEGEVIPTRIIDKPPSAELREDQKDADSLPPYEELDVILEMLVDQDASVDAVVAKGFDAEVVRRVERLVYLSEYKRFQSAP--GTRLT--ARAFWLDRRYPIVNRWRDPS--------MPLD--------IRVIPCRTDNYAYLLHDPASGETALLDAPEAAPILAALKDTGWSLGQILITHHHADHVEGVEELRAATGARVLGAGEDVHRLPPLDRELAGGDSVEVGGSRGEVIEVPGHTVGHIAFHFPDSRAVFTADSLMALGCGRLFEGTAPMMWESLSRLKELPGDTLVYSGHEYTASNAKFALTIEPDNESLILRSEAIAKARSEDRPTVPALLEDELRTNPFLRADLPEVKTLLDMDGYSDADVFAEIRQRKDDFMNEISE---------SPLCGKLLIAMPGMGDPRFDRSVIFVCAHSDEGALGLIVNKPTPDLSARDLLEQLGITPA-GDLPP-----INVHFGGPVEHGRGFVLHSADYCSDNSTMQVSTSFGMTATLDILEDIARGDGPRQSLLALGYSGWGPGQLEDELQRNGWLTGDATPEIVFGSDARGKWEEALKSIGIDPLMLSSEGGRAMTDSGDIL---KQMNDRSREVFRRVVEGYLITGDPVGSRTLTRSMSERISAATVRNVMQDLEHLGLLDSPHVSAGRIPTQQGLRMFVDGLLEVDDLTPDDRLRIEATVGNSNGD-VGSILDRVGAALSGVTRGASLVLAPKHEAPIKHIEFVSLAPHRALVVLVFADGQVENRVFTPPPGLTPSAMREAANFVNAVAEGLTLSELGARMTKEIARRRQEIDVLARDLVESGLAIWDDADGQNE--R-LIVRGRANLLGEGGSEADLDRIRSLFDDLERKRDIAEFLELAEAGDGVRIFIGSENKLFSLSGSSLVVSPYMNADRKIVGAVGVIGPTRLNYGRVVPIVNYTAQLVGKLISDRG-----MSDLIDDPEP----GQNAPEFTVSELSGAVKRSIEGEFARVRVRGEVGRVSRPRSGHVYLDLKDDRSVLAGVIWKGVCSRLATQPEEGMEVVATGRLTTFPGQSKYQMVIEEIAPAGVGALMALLEKRRQMFQAEGLFDAGRKRALPFLPEVIGVVTSPSGAVIRDILHRLRDRFPRKVLIWPVAVQGKSCAPEVARALAGFNALTPGGALPRPDLLIVARGGGSIEDLWGFNEEEVVRAVAASRIPVISAVGHETDTTLIDHVADRRAPTPTAAAELAVPVRLELMAGLDQMGARLSRCVAQAVATRRQRAGDLARAMPRLESLLDSARQRNDGAEERLK---------------------SALRHATQHKRTRLEGIAGPLRPGLLRRAMRAEGERLSKAAERLRPC-----------------------LDTGLRERRSALAKLSAGLRPRELRRDIANRAEALARLDDRMRAAFEQAQGRRRDRLEATERLRQTLGYVETLGRGYAVIRSG-EHVLTDRAAAAAAGRFEVEFRDGRLDVAVTSGAPGTGSGQGGKGPSRPEPAPAQAERAAKPAPAPARKPKAP-PPGQGSLFD-MSDKKQERRG----LGRGLSALMADV-----------------DLQPGKARSEPAAA----ERVVPIEKIAANPDQPRRDFSAQALQELTDSVREKGIIQPLIVRPHPAGADRFEIVAGERRWRAAQRAQLHEIPVIVRDLDDTEVLELAIIENIQRADLNAVEEAAGYKQLMEKFGHTQERLSASLGKSRSHIANLMRLLQLPEDVQDHLRQGRLSAGHARALITTENPSELARQVIAKGLSVRQTEDLAKGGGG--SSAAPK-ASRKSSGRTTDPDTLALEDQLTANLGLEVRIEH-QAGQESGRMVLNYKDLDQLDGLIALLNVRPMDVSR-----------------------------------------------------------------------------------------------------------MGQTPPAGASSAAEPGVVAVEPAAARKRTPETS-ADACRPDRVSLRGGWGRTRFIVELADSDASRAQGLMNRTRMERTHGMLFVYPRPQRAAFWMRNTLIPLDMIFADAQGVVTRVHSNANPLDETVIDGGRG-VQYVLEINGGLARRYGIAPGTQLRHPAIGP-EPAWPCR-----MPKRTDIKTIMIIGAGPIVIGQACEFDYSGAQACKALREEGYRVVLVNSNPATIMTDPDMADATYIEPITPEIVARIIEKERPDALLPTMGGQTGLNTSLALADMGVLEKFGVELIGANREAIEMAEDRKLFREAMDRIGLENPKATI------------------ANNMQECMDAIEYVGLPAIIRPAFTLGGTGGGVAYNRDDYEHYCKTGLDASPVNQILIDESLLGWKEFEMEVVRDRNDNAIIVCAIENVDPMGVHTGDSITVAPALTLTDKEYQIMRNGSLAVLREIGVETGGSNVQWAVNPEDGRMVVIEMNPRVSRSSALASKATGFPIAKIAAKLAVGYTLDELDNDITKVTPASFEPTIDYVVTKIPRFAFEKFPGAQNNLTTAMKSVGEAMAIGRTIHESLQKALASMETGLTGFDEIDIPG------------APDM-----------------AAITKALSAQTPDRIRVIAQAMRHGLDNDAIQAATSFDPWFLARIREIVEAEAAIRRDGLPLTEDGLRKLKMMGFTDARLAKLTGRDEGTVRRARRNLGVNAVFKRIDTCAAEFEAQTPYMYSTYEADAMGD--VECESRPSDRKKVVILGGGPNRIGQGIEFDYCCCHACFSLTDAGYETIMVNCNPETVSTDYDTSDRLYFEPLTFEHVMEILRVEMSNGTLHGVIVQFGGQTPLKLANALEAEGIPILGTSPDAIDLAEDRERFQALVQRLGLKQPHNGIASTDAQAMMIAQEIGFPLVIRPSYVLGGRAMEIVRDMPQLERYIAEAVVVSGDSPVLLDSYLSGAVEIDVDAICDGTDVHVAGIMQHIEEAGVHSGDSACSLPPYSLPQSIIDELHVQTRALALALGVVGLMNIQFAVKDG-----EIYLIEVNPRASRTVPFVAKAVGSPIASIAARVMAGEKLSAFDLK----------------------DPQIEGFAVKEAVLPFARFPGVDTLLGPEMRSTGEVMGYDASFARAFLKAQLGAGTVLPQSGR--VFLSIKDADKTPELVETAKILRELGFEIVATRGTAEFLTTNKVESTVVNKVYEGRPNIVDMMKDGGIALVMNTTEGAQAVEDSRSMRAVALYDKIPYFTTAAASHAAALAMRSAREGEIGVQALQG-MTDNSPTAPCAEEIARLPYRPCVGIVLINRDGLIFAGERLDTPG--AWQMPQGGVDPGESPRQAALRELSEETGLTDEQVTVLAETREARCYDLPHDLVPKVWKGRFRGQSQHWFLMQLTGSENGIDINGGHPEFARWQWMRRADLVEAIVPFKRDIYVSVLAEFADHLR------------MTRKVSVFGATGSIGVSTVDLLTRGRDVDPSAYDVVALTGGANVALLAQQARALDASLAVTAHPSCLPELRERLAGTRIECASGADAIIEAARRPVDWAMSAIVGAAGLPPGLAVLEQGATLALANKESLVAAGPLMMATARRHGATLLPVDSEHSGIFQCLGQEDPAGVERVILTASGGPFRDWSMERLRQVTPAQAATHPNWDMGQRITIDSASMFNKAMEIIETHEFFGLDPARIEVLVHPQSLVHALVGFRDGGILAHLGPADMRHAIGYALHWPGRGDVPVERLDLAALGRLDFEAPDENRFPALRLAREVMEAGGLAGAAFNAAKETALDLFIDKRIGFLDMAVLVEHVLD--TLSAETRLGSAEFNLDNVLNVDH----LARKAAIDSARVRA------MCADTPDTPEA-PDYKTTLNLPQTEFPMRAGLPKREPGWLERWARIGVYERLRDKAKA------ET---RQPFTLHDGPPYANGHLHIGHALNKILKDMVIRSQQMSGRDARYIPGWDCHGLPIEWKIEEKYRQKGLDKDAVPIVDFRQECRSFAEGWVDIQRDEFKRIGITGNWDNPYLTMDFHAERVIAEEFQKFLMNGTLYRGSKPVMWSPVEKTALAEAEVEYHDHKSHTIWVPFKIR---------------EGAPA------------------DMADARVVIWTTTPWTIPSNKAVAFNPKIAYGLYRVDGTEEES----WTAQGDLYLLADKLADEVLSRARV---TASTRLRDV-SADELSGLTLIHPFNGVEGAEGFWDYDVPMIDGDHVTDDAGTGFVHTAPSHGADDFECFVRRNWLDR-MTYNVGEESEFLDHVPF-FAG-----------LQVFDRKGK----EGKANAAVIDKLVAASGLIARGRVTHSYPHSWRSKAPVIFRNTPQWFAAIDRAVGDGQDEYGTTIRERALTSIDKLVTWTPKTGRNRLYSMIEARPDWVLSRQRAWGVPLTCFVKKDTQPTDPDFLLRDPV-VNARITEAFEAEGADAWYKDGAKARFLGDDHDHDAYEQVFDILDVWFDSGSTHAFVLRDRE-DGSEDGLADLYLEGTDQHRGWFHSSMLQACGTRGRAPYRGVLTHGFTLDGKGNKMSKSLGNTIAPEAVIKQYGADILRLWVAQSDYTADLRIGDEILKGVADSYRRLRNTMRFMLGSLSGFSEADRI---------------APEDMPELERWVLHRLAELDHRVRTGYAAYDFQGVFQALFNFATVDLSSFYFDIRKDVLYCD----------------GDTVERRAARSVLDILYHRLTTWLAPILVFTMEEVWLE-RFPGDASSVHLQDIPATPGDWRDDPLAAKWAGVRRVRRVVTAALEEQRRDKVIGASLEAAPVVHVTDPAVLAQ-------LRTLPFADICITSAVELT-GDPIPDEAFRLPEIDGVGVVFEQAEGEKCQRCWKIL-PDVGHHAHAQVCGRCDAALKDAGV-------MSRLILSHLSLSRFRSHRTLDLALDGRPVAIFGPNGAGKTNILEAISLLSPGRGMRRAAMEEIARKPEALGWKLRAELTGPGVSHEIAMRSEAGTPRTLSIDEKPAAQMALGRMIRILWLIPAMDRLWIEGAEGRRRFLDRVTLSFEPAHGEASLAYEKAMRERNRLLKDQARDPAWYGALESQMARLGAEIRQNRAEALTALSLAQTDATTA-FPTAQLRMEEAGQEGVEDPAMDEAS--------LREALARGRARDMAAGRTLVGPHRADLVAIYEAKGMEARHCSTGEQKALLISLILANARAL-SARLDAPPILLLDEVAAHLDEARRAALYDEITGLGAQAFMTGTEPGLFRAVGDRAMAVAVDETDGASTTRILARLPDDRSPEERPMAAKLKKGDKVVVLAGKDKGKEGEITRVMPAANKAIVDGVNVAIRHTRQSQNSQGGRVPTPMPIDLSNLALLD-SNGKATRVGFRTED-GKKVRFAKTTGDVI---------------------MMTLHPKVIDLTLDRVWRLLAALGHPEKNMPPVIHVAGTNGKGSTQAMIRAGLEAGDKRVHAYTSPHLARFHERIRLAGELISESYLSEILDECYAANDGAPITYFEITTVAAILAFSRVPADYTVLEVGLGGRLDATNVVESPALSIITPVSIDHQQYLGETLALIAGEKAGIIKRGVPCIVGAQEEAGLDVIEARARALDAPLLVRGQHWHVWEERGRLVFQDETGLLDLPLPNLIGAHQVQNAGMAIAALRHLGFGEA--E----CEAAM----RDAYWPARLQRLTTGPLIAAAGEAELWLDGGHNPAAGEALAEALARLPERPLHLICGMLDTKDIGGYLRPLAGLAQDLIAVSI-PGEAATLPAERTAEAARKAGIPATTAESVDEAVAELKAQAPGGRILICGSLYLAGRVLRENG-----MTSADPRIKFCGLRTEADIAVAVAAGATYVGFVFFDRSPRAVSVDEARALALAVPAGIAKVALTVDPDDATLDRIVQQVP-LDMIQLHGHETPERVAELRARHGLPVMKAIGVADAADLEAIERYEGVADQLLIDARPPRDAVLPGGNGLKFDWRLIEGRD-WRGPWMLAGGLDVANVAEAIARTGARQIDVSSGVESAPGQKDAAKMAAFAAAARGESHA--------------MSGAGKAATKGTTAPAMPEVRGNLTADRPLADLTWLRVGGPADWFFQPADLDDLRAFLRDLPENVQVFPMGVGSNLIVRDGGLRAVVIRM-GRGFNGIE-IEGSRVTAGVAALDAHVARKAAAAGV-DLTFLRTIPGAIGGAVRMNAGCYGSYVADVFVSARAVTRAGELVTL-------TADELNFAYRQTELPEGWVLVDATFEGP-SGEPEVLEQRMTDQLAKRDATQPTKDRTAGSTFRNPAGFSSTGRADDSHELKAWKVIDDAGMRGARLGGAQMSEMHPNFLVNTGDATAADLEGLGEEVRKKVFQNSGLTLQWEIMRVGEPTDPKPGQ------------MPSAPS-----------VSFEFFPPQSLDASFRLWETVQALAPLSPEFVSVTYGAGGTTRKLTHEAVETIHKNYGLRVAAHLTCVDATREETMEIVRGYKEAGVTDIVALRGDPPKGAA-RFTPHPEGFASSVELIEALAAEGG-----LTLHVGAYPDPHPEATNPVADVEYLKRKIDAGASTAITQFFFEKETFLRFRDRCVAAGIDAPIVPGILPIDNWKRVRNFAARCGAFIPDWVDEAFDKADR--DDRSELFATAMCTELCSDLIDEGVDTLHFYTLNRPYLTRQVCNALGVH-STQGLEKVA------MERVFRKLALEAGEKIMEIYGSDDFGVMSKADESPVTAADLAADAIISAGLAQAFPDIPVVTEEQAASH--SGAGTEFIIVDPLDGTKEFVKRRGEFTVNIAYVRDGVPLRGVVYAPARKRMFYTLADGTSIEEQGPFGEE-PGVCAPIRV-SDPDNDALIVVASKSHRDEATDAYISRYEVAEAASAGSSLKFCLVATGEADLYPRLGRTMEWDTAAGDAVLRGAGGRVVDFDRLEPLTYGK--PGHANPFFIAHAPGVELREG-MNTRTETDSFGPLEVPSDKYWGAQTQRSLMNFPIGWERQPIAIVRALGVIKKACAQANAASGKLPAELGQAIADAAQEVVDGKLDDNFPLVVWQTGSGTQSNMNANEVIANRAIEMLGGTIGSKDPVHPNDHVNMGQSSNDTFPTAMHVATAMTARDVLLPGLEKLHAALQAKATAFQDIIKIGRTHTMDATPLTLGQEFSGYTHQVAMGIERIKGALPRINELAQGGTAVGTGLNTVEGWDVTVAANMAEITGLPFVTAPNKFEALAAHDAMVEMSGALKTVAASLFKIANDIRLLGSGPRCGLGELMLPENEPGSSIMPGKVNPTQAEALTQVCAHVFGNDAAVGFAGSQGHFELNVYKPMMAYNVLQSMQLLGDAAVAFTDNCVSGIEANRERIDKLMRESLMLVTALAPQIGYDNATTVAKTAHKNGTTLREEAINLGFVDGETFDRVVRPEQMIGPKSK--------MSLLDAAIKVRENAHAPYSGFRVGAALRGSDGRIYVGCNVENVAYPEGTCAEAGAIAAMVAAGVTRLEEVVVVAGGAAPVAPCGGCRQKLREFGG-PDVTVTMRNLDGAEVRQTLAELLPGAFDAAQMAEG-------MIQADLSIVLPEVVLAVYAMLALLVAVYTGKDRVAPLVLWVTAGVFALLAFWIGVGGGG-SRQAFGGAFIDDGFARFAKVLILLSAAAILVMSQRMLERLEIMRFEYPLLLALSVVGMMMMVSAGDLMALYMGLELQSLALYVVASIHRDSTRSTEAGLKYFVLGALSSGLLLYGASLIYGFAGTTLFTGI-TAAVTGQLPLGLLFGLVFLSVGLAFKVSAAPFHMWTPDVYEGSPTPVTAFFATAPKVAAMALFARVAHDAFGGAVGDWTQIVAFLSVLSMVIGAVAAIGQRNIKRLMAYSSISHMGFALMGLAAGTVAGVQAMLIYMVIYVTMNIGTFAFIMSMERDGEPVTDIRALNQYSKQEPLRALALLVLMFSLAGIPPLVGFFAKFYVLTAAVDADLTWLAVAGALASVVAAFYYLRIVFYMYFGEEG-EALDRSMTPVNWLALMASAAFMVLGV-----VNLFGIDGMAAMAAEALVGR----MTQDLDRVFSALADATRRRILAMLLEDDMAVTDVAEPFEISLAAISKHLGVLTAAGLISQERRGRVKWCKLEPDALRAASVWMQGFGQFEPVNLDQFERFLEAEF--GTDPSAGSPDAG--------MGDQTDGSSSAALSAQDDD-----------SDTE----QR--GLWQRFVSTISGSDTG-DDGAEGDREKEN----ADPHATDSMGILNLRRMRVEDVAIPKVEIVAVPNTIKKEELVDVFRESGFTRLPVYKGTLDTPMGFVHLKDLALRHGFSA-NGTKFSLKAMLRPLLYAPPSMGTGVLLQKMQSERIHMALVIDEYGGVDGLVTIEDLIETVIGEIEDEHDLTEGPLWRRDKQG-GYVVDARAPLDEFEAEIGVKLGSPEA--DEDVDTLGGLVFMMTGRVPVRGEMVPHPTGAEFEVVDADPRRLKRLRVRLPGALEDGAA--------------MRLVPVQRNRLPRPERVIYIKANLGTHGPDHMTDQTEAPEAGHPSRPGQAGPGTDVQVADAVRRNWVDTRAPAAWRPYLRLSRLDRPIGTWLLLLPCWWGLLLAAFSTGRFGFHDLWILAACGVGAIVMRGAGCTWNDITDRNIDDKVARTRSRPLPSGQVSTRGALVWMAAQCLVGLAILLTFNWAAVGLGVASLGLVAIYPFAKRFTWWPQVFLGLAFNWGALLAWTAHTGSLGWPAVLLYLAGIAWTLFYDTIYAHQDKEDDALIGVHSTARLFAERTPLWLRGFLVISVVLMMLSVMIAAGPGANPLALALALGGAWGFGWHLAWQLGRLNIDDTDCCLRLFRSNRNAGLIPVLFLAVAVLIRLNAGG-----------------------------------------------------------MAVPQNKVTKSRRNMRRAHDALVAANPNECANCGELKRPHHVCGACGHYDDREVVAATNEVDLDEDAA------MSELPGIVVTGASGRMGQMLIRIIRESDRARLVGAIERSGHEWVGQDVGRAMGGADCGVIVTDEPLEAFAKAQAVIDFTAPEATL-EFAKLAAQARAVHVIGTTGMSDEQIAKLEPAARH-APIVRAGNMSLGVNLLTQLTRQVAAALGEEFDIEVIEAHHRHKVDAPSGTALMLGEAAAEGRGVALEDVSDRGRDGITGARKPGDIGFVAVRGGDIVGEHDVLFAGEGERIILRHVATDRAIFARGALRAALWGQDKGPGQYDMIDVLGLRRD--MPDVNR-GNRPLSPHL--TIYRPQLTSMTSILTRLTGNALLLVALMIVWWFLAASIGPEYFAFADWLITSWFGDLVMTLSLWALWYHSLAGLRHLYWDT-GRGLELETAETLGWAVIAGSFVLTLVTLFFV---------------MLGTTFVVILAL-SAGWYFLLRRRAMAAAGPRLADLHSRPSYHGFYAALGSFLAGTAVLLVVGIGWGLWNDMQLQNQIGQALPES-------------------SAIE---RQLVLSDAKAVADG----------------ALPSRSGELRS------------------AVAERHATLETARQWGTAALALAAALA-FGLLAWRRSDRDFRARNRTERMVTIVLWATAAIAVLTTLGIVLSLIFETANFFS-------------------------------------------------------NIGWRIDKFLFGTTWSP-------LSGVQSGQMNP-DKVGAVPLFAGTFLITLIAMIVAVPIGLMAAIYLADFASPRTRSVVKPMLEILAGIPTVVYGFFAAITVAPFIRQS-GEA----LGIS---------VASESALAAGVVMGIMIIPFISSLSDDVINAVPQSLRDGSYGLGATKAETIRQVVLPSALPGIVSAVLLGVSRAVGETMIVVMAAGQGAN-------LTLNPLEAVTTVTVQIVMLITGD-TE-ASTAAGPAFTLGFTLFCITLLMNIIALRIVRRYRELYDMAQSYLGQKRLRKYYGKIREVLEMPNLIEVQKSSYDLFLKSGDQTEPMDGEGIKGVFQSVFPIKDFNETAVLEFVKYELEKPKYDVEECQQRDMTYSAPLKVTLRLIVFDVDEDTGAKSVKDIKEQDVFMGDMPLMTPNGTFVVNGTERVIVSQMHRSPGVFFDHDKGKTHSSGKLLFACRIIPYRGSWLDFEFDAKDIVFARIDRRRKLPVTTLLYALGLDQEGIMDAYYDTVTYRLE-KNKGWATKFFPERIRGTRPTYDIVDAGSGEVIAEAGKKVTPRAVKQLIDKGDVTEILVPYDQIVGRFVAKDIINEDTGAIYVEAGDELTQEFNKEGELTGGSLKDLTDAGITEIPVLDIDNINVGPYIRNTMVVDKNMGRDTALMDIYRVMRPGEPPTVEAASQLFDTLFFDSERYDLSAVGRVKMNMRLAL------DAPDTMRTLRREDIIACIKALVELRDGKGDIDDIDHLGNRRVRSVGELMENQYRVGLLRMERAIKERMSSVEIDTVMPQDLINAKPAAAAVREFFGSSQLSQFMDQTNPLSEVTHKRRLSALGPGGLTRERAGFEVRDVHPTHYGRMCPIETPEGPNIGLINSLATFARVNKYGFIETPYRKVENGKVTDDVSYMSATEEMRHTVAQANAKLDEQGRFVNDLVSTRQAGEYMLQPNENVDLIDVSPKQLVSVAASLIPFLENDDANRALMGSNMQRQAVPLLQAEAPLVGTGIEEIVARDSGAAIMARRGGIIDQVDSTRIVVRATQDLEPGDPGVDIYRLRKFQRSNQNTCINQRPLVKVGDTVGKNEVIADGPSTDLGELALGKNVVVAFMPWNGYNYEDSILISERIVRDDVFTSIHIEEFEVAARDTKLGPEEITRDIPNVGEEALRNLDEAGIVYIGADVGPGDILVGKITPKGESPMTPEEKLLRAIFGEKASDVRDTSLRLPPGDYGTIVEVRVFNRHGVDKDERALQIEREEVERLARDRDDELAILDRNIYARLKTQILGKTAVKGPKGVKPNSEITEELL-ETLSRGLWWQLALKEEADASIVEALNEQYQAQKLSLDARFEDKVEKVRRGDDLPPGVMKMVKVFVAVKRKLQPGDKMAGRHGNKGVISKVVPMEDMPFLGDGTPVDFVLNPLGVPSRMNVGQILETHMGWAARGLGEQIGEALGEYRRSGDMTPVRDAMKIAYGDDV-YDEGIADMDETRLVEAADNVVRGVPIATPVFDGAKEADVNDALQRAGFDMSGQSVLFDGRTGEQFARQVTVGVKYLLKLHHLVDDKIHARSTGPYSLVTQQPLGGKAQFGGQRFGEMEVWALEAYGAAYTLQEMLTVKSDDVAGRTKVYESIVKGEDNFEAGVPESFNVLVKEVRGLGLNMELL-------DAEGDD------MATNSGTDSKNTLYCSFCGKSQHEVRKLIAGPTVFICDECVELCMDIIREETKTSGLKSHDGVPTPKEICEVLDDYVIGQMHAKRVLSVAVHNHYKRLNQAGKTG-DIELAKSNILLIGPTGCGKTLLAQTLARILDVPFTMADATTLTEAGYVGEDVENIILKLLQASEYNVERAQRGIVYIDEVDKITRKSDNPSITRDVSGEGVQQALLKIMEGTVASVPPQGGRKHPQQEFLQVDTTNILFICGGAFAGLEKIIAQRGKGSAMGFGADVREEEKQGIGETFKSLEPEDLLKFGLIPEFVGRLPVIATLEDLDAEALVTILTAPKNALVKQYQRLFELEDAELTFTEDALKAIASRAIERKTGARGLRSIMEDILLDTMFDLPGMDDVEEVVVNEEAVLAEAKPLLIYADRAKEEPATAG--MAGSVNKVILVGNLGRDPEVRTFQNGGKVCNLRIATSENWKDRNTGERKERTEWHSVAIFSEPLARIAEQYLRKGSKVYLEGQLETRKWQDQSGQDRYSTEVVLRPYKGELTLLDSRGGE------GGGG------------GGYMADQ---SGGGYSGGG--SGGANDGGGYGG-------------PSGAASDLDDEIPFMSVLKSRIRPGDDSFVANRKAHLTLLETAAEAARAAAAGGGDKARERHLSRGKMLPRDRVAGLLDPGSPFLEVGATAAHGMYDGAAPGAGIIAGVGRVHGRDVMVVCNDATVKGGTYYPMSVKKHLRAQEIAAECRLPCVYLVDSGGANLPNQDEVFPDRDHFGRIFYNQARMSGAGIPQIAVVMGSCTAGGAYVPAMSDVSIIVRDQGTIFLAGPPLVRAATGEVVSAEDLGGGDVHTRLSGVADYLAEDDAHALALARQAVANL-NLGPQPGMPVQPPEPPAYDPDEILGVVPADLRTPYDIREVIARLVDGSRFDEFKARFGETLVTGFAHVEGMPVGLIANNGVLFSEAAQKGAHFIELCSQRRIPLVFLQNITGFMVGRKYENEGIARHGAKMVTAVASTEVPKVTMIVGGSFGAGNYGMSGRAYQPRFLWSWPNSRIGVMGGEQAAGVLATVRRDAIERGGGSWTPEEETAFKQPTIEMFERQSHPLYASARLWDDGIIDPRHSRRVLALSLRAALNAPIPETRFGVFRM--MRRVVVTGLGAVTPLACGVEETWSKILDSQSGAGPITRFD--ASGLATTYACEIPLGDGSNG------TFNADDWMEPKERRKVDDFILYGMAASVQAVRDSGWEPANEDERQRTGVMLGSGIGGLSTIADTAVLIKERGPRRVSPFFIPGALINLISGQVSIRFGFKGPNHSVVTACSTGAHAIGDAARLIMLGDADVMVAGGAESPISEIGIAGFNACKALSTKRPDAPKTASRPYDIDRDGFVMGEGAGVVVLEEYEHAKARGAKIYAEVLGYGLSGDAYHITAPSEDGEGGERAMRAAIKRAGIEPGAIDYINAHGTST-MADVIELAAVERVLG-DAAERATMSSTKSAIGHLLGAAGAVEAIFSILAIRDQVAPPTLNLDNPAVETPINLAPKVKQPREINVALSNSFGFGGTNASLVLGKV--MTITVGQDSSNTRKTLNVGGSSFAYYSI-AAAEAAGLGSFGNLPAVLKVVLENLLRFEDGI-TVSQDDIRAFSEWGANGGRNPREIAYRPARVLMQDFTGVPAVVDLAAMRDGIVALGGDAQKINPLNPVDLVIDHSVMIDEFGNPRAFQMNVDREYERNLERYTFLKWGQKAFNNFRVVPPGTGICHQVNLEYLSRTVWSDTDQNGEMVAYPDTLVGTDSHTTMVNGAAVLGWGVGGIEAEAAMLGQPISMLIPEVIGFKLTGKMVEGTTATDLVLRVVQMLREKGVVGKFVEFYGDGLDTVPLADRATIGNMAPEYGATCGFFPIDDETLRYLTQTGRDEETIALVEAYAKENGMWRNPGYAPVYTDTLELDMGTVVPAISGPKRPQDHIALTSAATAFGDYVKGVRNGQDASASAEIRWEGEGGQPEPQDIPGDEGHHQRGYVQ-----TDDGHYQLHDGSIVIASITSCTNTSNPYVMIGAGLVARKARALGLTRKPWVKTSLAPGSQVVSAYLEAANLQEDLDAIGFNLVGYGCTTCIGNSGPLEPEISKAINDYDLIATSVLSGNRNFEGRISPDVRANYLASPPLVVAYALVGDMNVDIATASLGKDKDGNDVYLKDIWPSAAEISELVEKTVTREAFQSKYADVFKGDEKWQAVKTTDSQTYDWPASSTYIQNPPYFRDMPAEAGTISDVKDARVLAILGDMVTTDHISPAGSFKETTPAGQYLTQRQVPVREFNSYGSRRGNHEVMMRGTFANIRIRNEMLDGVEGGYTKGPDGSQKAIFDAAQAYQEQGTPLVIVAGEQYGAGSSRDWAAKGTALLGVKAVIAESYERIHRSNLVGMGVIPFEFTGGDNRKSLGLTGDETFSITGLEGDLKPGAMVPCTITSPDGTTREIQLKCRIDTVVEKEYVENGGVLHYVLRDLARA-------MFLPFFEKLRESGIPVSLREYLAFLAGMKAGIVTYDVEGFYFLARTSMVKDERHLDRFDRAFASAFKGLEDIGLDQVLEAMDLPADWLEKMAEKHLSAEEKAEIEALGGFEKLMDTLRERLKEQQKRHQGGSKWIGTAGTSPFGAYGYNPEGVRIGQDGSRHQRAVKVWDRREFRNLDGDVELGTRNIKVALKRLRQWARDGAAEELDLNGTIRATAEHGYLDVVTRPERRNAVKVLLFLDVGGSMDPHVQVVEELFSAARAEFKHMEYYYFHNCLYEGVWKDNRRRHAERIPTWDVLRTFGSDYRCIFVGDASMSPYEIAFAGGASEHWNEEPGSTWLSRAREQWPATVWINPMPERFWPYTQSIAMIQELF-EGRMFPMTLDGIGRAVDELRR-MARRKKIYEGKAKILYEGPEPGTFVQYFKDDATAFNAEKKDVIEGKGVLNNRLSEFFMNGLTAVGIPNHFIRRINMREQLIRQVEIIPLEVIVRNYAAGSLSKRLGIEEGTALPRPIIEFCYKDDALGDPLVAEEHIIAFGWAAQQDLDDMVALALRVNDFLSGVMFGVGIRLVDFKIEIGRVWDN--DFQRLIVADEISPDSCRLWDMETGQKLDKDVFRRDLGNLADAYTEVARRLGVLPSNVTH-AVKPTLIN------MQFLDLAKVYIRSGGGGGGAVSFRREKYIEYGGPDGGDGGKGGSVVAEAVEGLNTLIDFRYQQHFFAKNGVPGMGKQRTGADGEDVILRVPVGTEILDEDEETVIADLTEVGQRVVLARGGNGGFGNLHFKSSTNQAPRRANAGQPGVERTIWLRLKLIADAGLLGLPNAGKSTFLAASSNARPKIADYPFTTLHPNLGVVGID-EVEFVMADIPGLIEGAHEGRGIGDRFLGHVERCSVLLHLVDGTSEDVAEDYRVIIHELESYGGALADKPRITALNKIDALDDEERAEKKAALEAATGGNVLM-MSGVSREGLPEVLRAVRARI---DADKLRQRKAG--------EEEPESWRP--MAAYEYETHVYDVVVIGAGGAGLRATLGMAERGLRTACVTKVFPTRSHTVAAQGGIAASLGNMGPDSWQWHMYDTVKGSDWLGDTDAMEYLAREAPKAVYELEHYGVPFSRTEEGKIYQRPFGGHTTEFGEGPPVQRTCAAADRTGHAILHTLYGQSLKQKAEFFVEYFAIDLLMSDDGKCEGVVCWKLDDGTIHVFAAKMVVLATGGYGRAYFSATSAHTVTGDGGGMVARAGLPLQDMEFVQFHPTGIFGAGCLITEGARGEGGYLTNSEGERFMERYAPTYKDLASRDVVSRCMTIEIREGRGVGADGDHIHLHLNHLPPETLAERLPGISESARIFAGVDLTKEPIPVIPTVHYNMGGIPTNYWGEVLNPTPEAPDAVTPGLMAVGEAGCASVHGANRLGSNSLIDLVVFGRAAAIRAAEVVDPASPTPVPSPRAIEGALSRFDGLRHASGATPTAALRLEMQRAMQADAAVFRTDKTLAEGVDKMTDIAGKMNDLQVTDRSLIWNTDLMETLELANLMPNALATIVSAEARKESRGAHAHEDYPDRDDVNWRKHTLATVDG---SKVTLDYRPVHLDPLTTEDEGGIQLKKIAPKARVY---MTDLSHIRNFSIVAHIDHGKSTLADRLIQSTGTVQDRDMKEQLLDAMDIERERGITIKANTVRIDYVADDGRKYVLNLIDTPGHVDFAYEVSRSMRAVEGSLLVVDSTQGVEAQTLANVYQAIDADHEIVPVLNKIDLPAADCERVAEQIEDVIGIDASGAIQVSAKTGIGIHETLEAIVKLLPPPQ-GERDAPLKAMLVDSWYDAYLGVIVLVRIIDGVLKKGDRIRMMHNDSIHHVDRIGVFRPPMQTVDELGPGEIGFLTASIKQVRDTRVGDTITHEKKGADKALPGFKPSQPVVFCGLFPVDNAEFEDLRDAIEKLALNDASFSFEMETSAALGFGFRCGFLGLLHLEVIRDRIEREYDIDLITTAPSVIYNVHMRDGEMIELHNPADMPDLTHVDHIEEPRIKATILVPDEFLGDVLKLCQDRRGIQMDLTYAGSRAMVVYDLPLNEVVFDFYDRLKSVTKGYASFDYQMIGYRQDHLVKMQILVNDEPVDALSTMVHRDRAETRGRAMVEKLKDLIPRHMFKIPIQAAIGGRIIARETLAALRKDVTAKCYGGDATRKKKLLEKQKAGKKKMRQFGKVDIPQEAFINALKMDG-MANSPQAKKRARQNERRQNVNKARRSRIRTFLRKVEEAITSGDADAAKTALQQAQPELMRGVTKGVMHKSTASRKMSRLNSRVKALA-MVQLTLPKNSRIKTGRTWPKPAGATNLRKFRIYRWSPEDGENPRVDTYFVDMDSCGPMILDALIKIKNEIDPTLAFRRSCREGICGSCAMNIDGVNTLACTRGLDEVASE-VRVYPLPHMPVVRDLIPDLTHFYAQHASIKPWLETKTNEPQKEWRQSIEDRKKLDGLYECIMCASCSTACPSYWWNGDRYLGPAALLHAQRWIVDSRDEATGERLDDLEDPFKLYRCHTIMNCTKTCPKGLNPAKAIAEIKKALVERIVMPHDGQDSTAIQAKSRLDGELLAVTSAALGAVSPLLERATQLGRERTV-RDGRVAADLVEAEQSASHGLAWLATYIEALTQMQAWAERLDAEGTLGEVERLILAIAFGEYLGQLAGGIPMSQGEILRLHDMGLTRADAACLEHGEVRLLIEQGNTQARRTRLVELMQEQSANVTVGRTGLDDELEMIREQFRRFAVEKVEPEAHGWHLRDELIPMPIIEEMAELGVFGLTIPEEYGGFGLSKASMCVVSEELSRGYIGVGSLGTRSEIAAELILCGGTEAQKQHWLPKIASAEILPTAVFTEPNTGSDLGSLRTRAVREGD--DYLVTGNKTWITHAARAHVMTLLARTDPATSDYKGLSMFLAEKEPGTDADPFPTPGMTGGEIEVLGYRGMKEYELGFDGFRVSGDNLLGGVEGQGFKQLMQTFESARIQTAARAIGVAQSALDTGLRYAQDRKQFGKALIAFPRVSGKLAMMAVEIMIARQLTYHSARQKDGGKRCDLEAGMAKLLGARVAWAAADNALQIHGGNGFAQEYAISRILCDARILNIFEGAAEIQAQVIARRLLG---MNIHEYQAKRLLRDYGAPVADGRPVLKAEDAKSAAGELDGPVWVVKAQIHAGGRGKGSFKEADAGEKGGVRITKSVEEAATEAKKMLGRTLVTHQTGPAGKQVNRIYIEDGSGIARELYLALLVDRQTSRIAFVCSTEGGMDIEEVAASTPDKILSFSVDPAAGYQAFHGRRIAFALGLEGQQVKQCVQLMGTLYKLFNDKDMEMLEINPLVVTDK-GDLMCLDAKMGFDGNAIYRHQDIAQLRDETEEDPKELAASKYDLNYIALDGEIGCMVNGAGLAMSTMDIIKLEGAEPANFLDVGGSATTEKVTEAFKIITSDPNVKGILVNIFGGIMRCDVIAEGVVEAVKEVGLQVPLVVRLEGTNVEEGKKIINESGLNVIAADDLGDAARKIVKAVKG-----MSDRELREIGP------DKSLLLVDDDEPFLRRLARAMEKRGFDVETADCVAAGRAIATARPPAYAVVDLRLEDGNGLDVVEVLREKRPDSRIVVLTGYGAIATAVTAVKIGATDYLSKPADANEVTAALLADGSDLPPPPENPMSADRVRWEHIQRVYELCDRNVSETARRLNMHRRTLQRILAKRSPR-----MDLRNIAIIAHVDHGKTTLVDELLKQSGTYRENQATTERAMDSNDLERERGITIFAKPTSVEWKGTRINIVDTPGHADFGGEVERILSMVDGVVLLVDAAEGPMPQTKFVTSKALALGLRPIVVLNKVDKPDAEPDRALNEVFDLFASLDADDDQLDFPHLYASGRSGWADAELDGPRKDLAALFNLVVRHVPPPRQQKHQDEPFRMLATTLGADPFMGRVLTGRVESGKLKVGATVQALSRIGEKIEQFRVTKIQAFRGLAQQDIEEAQAGDIVSIAGMTKATVADTICALAVEDPLPAQPIDPPTITVTFGINDSPLAGRDGKKVQSRVIRERLMKEAESNVAIKVTDTPGGEAFEVAGRGELQMGVLIENMRREGFELSISRPQVVFRELD-GQQMEPIEEVTIDVDDEYSGAVIEKITGSRKGDLIEMRQAGAGKTRIIAHVPSRGLIGYHGEFLTDTRGTGVLNRVFHSWAPHKGAIPGRRQGVLISMENGTSVAYALWKLEDRGKFFIGAQEPVYQGMILGEHSRDNDLEVNPLKGKQLTNVRASGTDEAVRLTTPVTLTLEQAIAYIDDDELVEVTPNAIRLRKRFLDPHERKRQSR-AG-------------------MSRQMTGAQMVVKALKDQGVDTIFGYPGGAVLPIYDEIFQQNDIRHVLVRHEQAAVHAAEGYARSTGKPGVALVTSGPGATNAVTGLTDALMDSIPIVVLTGQVPTFMIGNDAFQEADTVGITRPCTKHNWLVKETEKLPGVIHQAFHVATKGRPGPVLVDIPKDVQFASARYTSPEKAPVGHYQPRTKGDATAIAELARMMETAERPILYTGGGVINSGDEATRLLRELAGATNFPVTSTLMGLGAYPASGDNWLGMLGMHGLYEANMAMHGCDLMICVGARFDDRITGRVDAFSPNSKKAHIDIDPSSINKVIHVDVPIIGDVASVLGDLLEVWKERGAKTNEEGIARWWSQISEWKKTRCLDYAPSKETIKPQHAVERLEALTRDHPKRYITTEVGQHQMWAAQYLHFNEPNRWMTSGGLGTMGYGFPASVGVQMAHPDALVINVAGEASWLMNMQEMGTVAQYRLPVKQFILNNERLGMVRQWQELLHGERYSHSWSESLPDFVKLAESFGIKGLTVSDPADLDDAIREMLDHDGPVIFDCLVEKHENCFPMIPSGAPHNEMLLG-NASTKDAIGSKGAVLVMALKSYKPTTPGQRGLVLIDRSELWKGRPVKSLTEGLTKKGGRNNTGRITMRRRGGGAKRLYRIVDFKRTKLDMTATVERIEYDPNRTAFIALIKYEDGQQAYILAPQRLAVGDSVVASAKADVKPGNAMPFSGLPIGTIVHNIELKPGKGGQIARAAGTYAQFVGRDGGYAQIRLSSGELRMVRQECMCTVGAVSNPDNSNQNLGKAGRNRHKGIRPSVRGVVMNPVDHPHGGGEGRTSGGRHPVTPWGKPTKGARTRNKNKASSKLIIRSRHAKKKGRMNQELTNNPFNPLTPPKQFDEIKVSLASPERILSWSFGEIKKPETINYRTFKPERDGLFCARIFGPIKDYECLCGKYKRMKYRGVVCEKCGVEVTLQKVRRERMGHIELASPVAHIWFLKSLPSRIGLMLDMTLRDLERILYFENYVVIEPGLTDLTYGQLMTEEEFLDAQDAYGTDAFTAGIGAEAIREMLAAIDLESEAEQLRADLAEATGELKPKKIIKRLKIVENFIESGNRPEWMVLTVIPVIPPELRPLVPLDGGRFATSDLNDLYRRVINRNNRLKRLIELRAPDIIVRNEKRMLQESVDALFDNGRRGRVITGANKRPLKSLSDMLKGKQGRFRQNLLGKRVDFSGRSVIVTGPELKLHQCGLPKKMALELFKPFIYSRLEAKGLSSTVKQAKKLVEKERPEVWDILDEVIREHPVMLNRAPTLHRLGIQAFEPILIEGKAIQLHPLVCSAFNADFDGDQMAVHVPLSLEAQLEARVLMMSTNNVLSPANGAPIIVPSQDMILGLYYITLEREGMKGEGMVFADVDEVQHALDAGEVHLHSKITARLKQIDDEGNEVMRRFETTPGRVRLGALLPMNAKAPFDLVNRLLRKKEVQQVIDTVYRYCGQKESVIFCDQIMTTGFREAFRAGISFGKDDMTIPEAKWKIVDDVRGQVKEFEQQYMDGLITQGEKYNKVVDAWSKCSDEVAAAMMSEISAVRKDDAGAEMEPNSVYMMSHSGARGSPAQMKQLGGMRGLMAKPSGEIIETPIISNFKEGLTVLEYFNSTHGARKGLADTALKTANSGYLTRRLVDVAQDCIVRQHDCGTDLAVTAEPAVNDGEVVSSMAERILGRVAAENVLMPGTDEVLLSKGELIDERKADAVEQAGVLRMRIRSPLTCEAEEGVCAMCYGRDLARGTIVNEGEAVGIIAAQSIGEPGTQLTMRTFHIGGIAQGGQQSFLEASQDGKIEYRNAVVLKNDAGETIVMGRNMILAIVDGDGAERASHKLGYGTKIFVEDGAKVSRGDKLFEWDPYTLPIIAEKAGKAKFVDLVSGIAVRDETDDATGMTQKIVMDWRAAPKGNELKPEILIVGEDGEPVRNDNGNPVTYPMSVDAVLSVEEGQEVRAGDVVARIPREGAKTKDITGGLPRVAELFEARRPKDHAIIAEIDGYVRFGRDYKNKRRITIEPADESMEPVEYMVPKGKHIPVAEGDFVNKGDYIMDGNPAPHDILSIMGVEALAEYMINEVQDVYRLQGVKINDKHIEVIVRQMLQKWEISDSGETTLLKGEHVDKAEFDAAND---KAIARGGRPAQGEPILLGITKASLQTRSFISAASFQETTRVLTEASVQGKRDKLVGLKENVIVGRLIPAGTGGATKRVRQIATERDRKVIEQRQAEAEAALALNAPDEAA--QPVEDGFGMGAP-----ESRDE------MEKRLNIVGGGMAGSEAAWQAVNMGVPVTIHEMRPGVETFAHKTGDLAEMVCSNSFRSDDDEANAVGLLHWEMRAADGLIMEMADRHSLPAGGALAVDRDAFSAAVTERLRAHPLVKVEPAEITTLPDDGTW---IIATGPLTSGNLAEAIRGVTGQQSLAFFDAIAPIVYADTVDMDTAWLQSRYDKGETEEERTAYLNCPMTRDQYEAFIDALLAADKTEFKPGETAG------YFDGCLPIEVMAERGRETLRFGPMKPVGLTNAHQPDVKPYAVVQLRRDNALGTLYNIVGFQTKMKYGAQTEVFRMIPGLEKAGFARLGGIHRNTFLNSPTLLDDRMRLKTHPHIRFAGQITGVEGYVESAAMGLLAGRMAAAQILGRDLPPPPPQTATGALIHHITGG--AEAKTFQPMNVNFGLFPPLT----------DVKGGRRNRKDRYKAYTDRAKADYSAWL-AHVPEPA-----MRAFVFPGQGAQTIGMGRDLARAYPAAQAVFDEVDEALGEELSALIWEGDQDELTLTQNAQPALMATSLAAVRALEAE-GVDMSVAS-YVAGHSLGEYSALAAAGALSVADTARLLRLRGQAMQKAVPVGQGAMAAILGLDYAAVRTLAAAAAAGGEGDVCQAANDNDPGQVVVSGHRAAVERAVDLAKEQGAKRALLLPVSAPFHCELMAPAAEAMAEALAGVEIKRPAVPLVANVRAEAVSDPDLIRNLLIEQVTGAVRWRESVLWMAAEGVKEIWELGAGKALIGMVRRIDRTVGTRAVGSPEDVVAAAQSLTPDEDQDGDDTDTGAAGNKE--MRDLRL------------------------PQERHPEKAHRPDNAQPKKPDWIRVKAPTSEGYRQTRDIMREHKLSTVCEEAGCPNVGECWSQGHATMMIMGEICTRGCTFCNVATGK-PQALDVFEPGRVADAVKKLGLKHVVITSVDRDDVDDGGAEHFAQTIRAVRRQAPGTTIEILTPDFLKCDPSVVKIVVDARPDVFNHNLETVPALYPGVRPGARYFHSLRLLQRVKEMDPSMFTKSGIMVGLGEDRQSVLQVMDDMRAADIDFLTIGQYLQPTPKHHRVDRFVTPDEFAGYERAAFGKGFLMVSATPLTRSSYHAGDDFERLRAARQKKLGIS----------MTSKTSPDKQKALDSALAQIERQFGKGSIMKLGADNPVQEIEATSTGSLGLDIALGIGGIPKGRIVEIYGPESSGKTTLTLHCVAEEQKKGGVCAFVDAEHALDPQYAKKLGVDLDELLISQPDTGEQALEIVDTLVRSGAVNMVVVDSVAALTPKSELEGEMGDSSVGVHARLMSQAMRKLTGSISRSKCTVIFINQIRMKIGVMFGSPETTTGGNALKFYSSVRLDIRRIGAIKDRDEVVGNTTKVKVVKNKVAPPFKQVEFDIMYGEGISKMGELLDLGVKAGVVEKSGSWFSYGDERIGQGRENAKAFLKANNRIALSIEDKIRAAHG-LEFDMPP------EEAGDDVVEA-----MNITRAAKYFLLFDFIKGFGLGFKYFFAPKATLNYPHEKGPLSPRFRGEHALRRYPNGEERCIACKLCEAICPAQAITIDAEPR-EDGSRRTTRYDIDMTKCIYCGFCQEACPVDAIVEGPNFEFATETREELYYNKEKLLENGDRWEAEIARNLATDAPYRMPITLPENLPAFDVLSNEGVMVMSDTDAARQDIRPLHIGLLNLMPKKIQTENQFARLIGATPLQIELSLIRMSEHQARNTAPAHMEEFYRPFQEV--VDQKFDGLIITGAPIEHLPFEEVTYWDELRQVMDWTRTNVHSTFGVCWGGMALIHHFHGVGKHMLDAKAFGCFRHRNLAPSSPYLRGFSDDLVVPVSRWTEMRQQEIDARPGLTTLLGSDEVGPCLVEDPANRALYIFNHLEYDSGTLKEEYDRDVAEGRAINVPCNYYPDDDPSMAPQNRWRSHAHLLYGNWINQIYQSTPFDRNAIGS--------------MSYANLPAPSPEQGLNRYLQEIRKFPMLEPEEEYMLAKRWVEDQDTEAAHKMVTSHLRLAAKIAMGYRGYGLPQAEVVSEANVGLMQAVKRFDPEKGFRLATYAMWWIRASIQEYILRSWSLVKMGTTSGQKKLFFNLRKAKARIGALEDGDLHPDTVKKIATDLSVTEDEVISMNRRMSGGDASLNALVGSDGEGATQWQDWLEDTDADQAEDYAERDELEARRELMAEAMSVLNDREKDILMQRRLKDRPVTLEELSGQYDVSRERIRQIEVRAFEKLQDRMRSLAKEKGLLTPA-MAREDNRRG---NRRER-------EETPEFADRLVAINRVSKTVKGGKRFGFAALVVVGDQRGRVGFGKGKAKEVPEAIRKATEQAKRQMVRVPLREGRTLHHDIEGRHGAGKVVMRTAPQGTGIIAGGPMRAVFEMLGVQDVVAKSIGSQNPYNMIRATLNGLGREASPRSVAQRRGKKVADILKKPEAEAASES--------------VEA--MNDPIGDMLTRIRNAQLRGKSTVETPASKLRAWVLDVLADEGYIRGYEKTTGKDGHPALSISLKYYEGTPVIREIKRVSKPGRRVYMGVKDIPSVRQGLGVSIVSTPRGVMSDANARTANVGGEVLCTVF---MNFGKGHHLHLIDGSAFIFRAYHALPPLTRKSDGLPVGAVSGFVNMLFKYVQDNSGPDAATHVAVIFDKGSQTFRNEMYDLYKANREAMPEELRPQIPLTRRATEAFNIACKEVEGFEADDIIATLSCQARDLGGRVTIVSSDKDLMQLVGDGVEMLDPMKNRRIDRDGVQEKFGVPPERVVDVQALAGDSVDNIPGAPGIGVKTAALLINEYGDLESLLERAEEIKQPKRRQTLVEFAEQIRLSRRLVQLDCSMTLDFTLDDLEVCDPVPDTLLEFLNEMEFRTVTRRVAETLGVEAPPAPEIAAPSRDAGDAAHPGAAVAQLPFDASAYECVRDLAALQVWIDRIRAAGHVAVDTETTSLNDMRAELVGISLCVEPGQACYIPLTH---RAAAADDLFGSDALAEGQIPMAEALGALRPVLEDDGILKIGQNMKYDAKILGRNGIDVAPIDDTMLMSYALHAGLHGHGMDTLCDRYLGHTPIPIKPLLGSGKSAITFDRVPIDKATAYAAEDADVTLRLWRHFKPLLHRSSVTTVYETLERPLVPVLAGMERAGILVNRDVLSRMSNAFSQKMAALEAEVHEMAGMPFKLGSPKQLGEVLFDHLSL--PGGKR---GKSGAYATGADILEDLATE-HELPRRLLDWRQLDKLKSTYTDALQTHIHPETGRVHTCYSIAGASTGRLASTDPNLQNIPVRSEEGRRIREAFVAAPGKRLVSLDYSQIELRILAHVADIPALKEAFRDGLDIHAMTASEMFGVPLEGMDPMIRRQAKAINFGVIYGISGFGLARNLRIPREEAQGFIDRYFERFPGIRTYMDDTVAFAKEHGYVRTLFGRRIHTPEIGAKGPRAGFAKRAAINAPIQGTAADVIRRAMTRMPAAIEGL--PATMLLQVHDELVFEVDEGAVDTLIDRARQVMENAHAPVVDLSVPLIVDAGQGANWAEAH-MANHLYRGDLPDGLDLGPIVAIDCETMGLNPHRDRLCVVQLSGGDGNAHLVQIEKGQTSAPNLCRMLADPAVLKLFHFGRFDIAAMLHAFGVTAAPVYCTKIASKLVRTYTDRHGLKNLLQELLNIDISKQQQSSDWGAPNLTDAQLSYAASDVLYLHKVKEALDTMLEREGRTELAQAAFDFLPTRARLDLAGWAEIDIFSH-MAKKAMIEREKKRQALVEKYAAKRAELKEIARDESKPMEERFKARLKLAKLPRNSSATRLHNRCQLTGRPHAYYRKLKMSRIALRDLGSNGQIPGLVKSSW-----------------MVEWITSPRPVDYEEALRVMEDRVDRIAQGTADEAIWLLEHPPLYTAGSSARPADLVDPDRFPVFEARRGGQYTYHGPGQRVVYVMLDVGSRGRDVRRFVRQLEAWVIETLARFGVTGHLHPGRVGVWVNRPERPAAPDGSPAEEKIAAIGIRLRRWISFHGISINVEPDLEHFSGIVPCGIAD--RGVTSLVDLGLPVTMEDLDLALRANFDRTMGPRAAPAPAAASGTAPPPDPAPRMANVVVVGAQWGDEGKGKIVDWLSERADVIARFQGGHNAGHTLVIDGEVYKLSLLPSGIVRQGKLSVIGNGVVLDPWHLVSEIAKLREQGVEISPETLMVAENAPLILPIHGELDRARESQASV-AKIGTTGRGIGPAYEDKVGRRSVRVADLADAATLELRVDRALVHHDALRRGLGLEPVDRDALLASLREIAPEVLRYAAPVWKVLNEKRKAGKRILFEGAQGALLDIDFGTYPFVTSSNVIAGQAATGTGIGPGSIDFVLGIVKAYTTRVGEGPFPAELNDANGQRLGERGHEFGTVTGRQRRCGWFDAVLVRQTCATSGVNGIAFTKLDVLDGFDELKICVAYELDGERLDHLPIAADQQARCTPVYETMPGWSESTEGARSWADLPANAIKYVRRVEELIDCPVALLSTSPEREDTILVTDPFAD-MGFKTGIVGLPNVGKSTLFNALTRTAAAQAANFPFCTIEPNVGEVAVPDARLDKLAAIASSKQIIPTRMTFVDIAGLVKGASKGEGLGNQFLANIRECDAIAHVLRCFENDDITHVDGRVDPVEDAETIETELMLADMESIEKRLQNLQRKLKGNDKDAAQQDRLLRRALAALEEGRPARTVEIDAEDEKAWAMLQLLTSKPVLYVCNVDEASAATGNDQTRRVAEMAEAQGAAHVVISAAIEEEISQLDPEEAREFLEELGLEEAGLDRLIRAGYELLDLQTYFTVGPKEARAWTVPAGSTAPKAAGVIHGDFERGFIRAETIAYDDYIANNGEQGAKDAGKLRVEGKSYIVKDGDVLHFLFNA---MSRVEVGIIMGSQSDWSTMKEAAQILDELNVPYEARIVSAHRTPDRLWDYGRGAVEAGLKVIIAGAGGAAHLPGMMASKTRVPVIGVPVQTKALSGVDSLYSIVQMPKGYPVATMAIGAAGAANAGLMAAAILANGDPALARRLDEWRAALSDSIPEVPRDD--MSINGFGHLFRVTTWGESHGPALGATVDGCPPGIPLDAEMIQTFLDRRKPGQNRYTTQRREADEVEILSGVFEG------QTTGTPIQLMIRNTDQRSKDYGDIASKFRPGHADITYWQKYGIRDYRGGGRSSARETAARVAAGGVARAALSVLMPGLRISGYMTRMGVHATDPARHDAEEIARNPFWCPDPVAASEWEGYLDGLRKSG-SSVGAVIEVTASGLPAGLGAPVYAKLDTDLAAGMMSINAVKGVEIGEGMAAATLTGEENADEITMGPDG-PVFSSNHAGGILGGISTGQDVVVRFSVKPTSSILTPRRTVNIAGEETEIVTKGRHDPCVGIRAVPVGEAMMACILLDHLLLHR----GQVGDGPRGHIG---MFT---ASMRFDLGEDVNALREMVHRWAQERIKPMAGQIDASNEFPSELWSEMGELGLLGITVPEEFGGAGMSYLAHTVAVEEIARASASVSLSYGAHSNLCVNQIRLNGTQAQKEKYLPGLVSGQHVGALAMSEASAGSDVVSMRLRAEKRNDHYRLTGNKYWITNGPDADTLVVYAKTDPQAGSRGITAFLIEKSMKGFTTSPHFDKLGMRGSNTAELIFDDVEVPFENVLGDEGKGVRVLMSGLDYERVVLAGIGTGIMAACLDEVMPYLRERQQFGRPIGEFQLMQGKIADMYTAMNTARAYVYEVARACDRGEVTRQDAAACVLYSSEEAMKQAHQAVQALGGAGFLADSPVARLFRDAKLMEIGAGTSEIRRMLIGRELMAAD--MAETDFPG-WHGTTIIGVRRGGRVVVAGDGQVSLGQTVIKGTARKVRRLSPGGHDVVAGFAGSTADAFTLLERLETKLEATPGQLARASVELAKDWRTDKYLQKLEAMLIVTDGTDLLVITGAGDVLEPENDVAAIGSGGNFALAAARGLMETDLDAEQVARKAMAIAADICVYTNGNLTVESIDK------------MKRTFQPSNLVRKRRHGFRARMATKAGRKILNARRARGRKSLSAMGQKVNPIGMRLQVNRTWDSRWYADTKDYGDLLLEDIKIREFIKEECKQAGISKVIIERPHRKCRVTIHTARPGVIIGKKGADIETLRKKLASMTASELHLNILEVRKPELDAALVAESIAQQLERRVSFRRAMKRSVQNAMRMGSLGIRVNVAGRLGGAEIARTEWYREGRVPLHTLRADIDYALAEAKTPYGIIGIKVWIFKGEIMEHDPSARDRKQQE-----LQEGPAPRGP---RR-----MFYRDERLALFIDGSNLYAAAKALGFDIDYKLLRQEFMRRGKMLRAFYYTALLENDEYSPIRPLVDWLHYNGFSMVTKPAKEYTDSQGRRKVKGNMDIELTVDALELAPHVDHIVLFSGDGDFKPLIEALQRKGVRVSVVSTIRSQPPMISDELRRQADNFIELDELKEVIGRPPREPR-AEEEQRSEEMADI-MTKRTSAKYKLDRRMGENIWGRSKSPVVRREYGPGQHGQRRKGKLSDFGIQLRAKQKLKGYYGDLTEKQFRRIYAEAERVKGDTGENLIGLLERRLDAIVYRAKFVPTVFAARQFVNHGHVRVNGKKVNIPSYRVKEGDVIEVRDRSKQMAVLLEAVQLAERDVPDYLEVDHSKMTATFVRTPALGDVPYPVMMEPNLVVEFYAKN---MIHKNWQELIKPTQLDVKPGNDPSREATVVAEPLERGFGLTMGNALRRVLMSSLQGAAITSVQIDNVLHEFSSISGVREDVTDVVLNLKGVAVRMEVEGPKRLSVNAKGPGVVTAGDIAETAGIEILNKDHVICHLDEGADLFMELTVNTGKGYVSADKNRPEDAPIGLIPIDAIYSPVKKVSYDVQPTREGQVLDYDKLTLKLVTDGSITPDDAVAYAARILQDQLSVFVNFDEP--EAARSQDDEDDLEFNPLLLKKVDELELSVRSANCLKNDNIVYIGDLIQKTEAEMLRTPNFGRKSLNEIKEVLSGMGLHLGMDIVDWPPDNIEELAKKYEDHL------------------MPVMHAPAPDVRNRK---------------KLEGGRRFKLETEFSPAGDQPTAIAELSAGVMAGERDQVLLGATGTGKTFTMAKIIEETQRPAIILAPNKTLAAQLYGEFKGFFPDNAVEYFVSYYDYYQPEAYVPRSDTYIEKESQINEQIDRMRHSATRALLERDDVIIVASVSCIYGIGSVETYGAMTQDLIAGQEYDQRAIIADLVAQQYRRNDQAFQRGTFRVRGDSLEIFPAHLDSRAWRLSFFGNELESITEFDPLTGEKTDTFQQIRVYANSHYVTPKPTMKQAVNSIKKELRQRLDQLVADGKLLEAQRLEQRTNFDIEMLEATGVCNGIENYSRYLTGRAPGEPPPTLFEFIPDNAIVFADESHVSVPQIGGMYKGDYRRKFTLAEHGFRLPSCMDNRPLKFEEWDAMRPQSVFVSATPAGWELEQAGGVFTEQVIRPTGLLDPEIEIRPVG----TQVDDLLDEVRRVTAAGYRTLVTTLTKRMAEDLTEYMHEQGIKVRYMHSDIDTLERIEILRDLRLGAFDVLIGINLLREGLDIPECGLVAILDADKEGFLRSETSLIQTIGRAARNVDARAILYADRITGSMERAMRETERRREKQIAYNTEHGITPATVKKNVEDILAGL------YQGDVDMNRVTAKIDAPMA-----GANLQAHLDGLRDKMRKAAENLEFEEAARLRDEVKRLETVDLVVSDDPLARQQAVDRAVDAAQKASGRSTAGRGGMRGGNVK---RRRKG--MQSQNIRIRLKAFDYRVLDSSTQEIVNTAKRTGAQVRGPIPLPNKIEKFTVLRGPHIDKKSRDQFEIRTHKRLLDIIDPTPQTVDALMKLDLAAGVDVEIKV-------------------------MFKVSKKSMEWGEDTLTLETGKIARQADGCVIATYGETSVMAAVTFAKEQKPGQDFFPLTVHYQEKYYAAGKVPGGFFKREARPSEKETLTARLIDRPIRPLFVPGFKNEVLVMCTVLSHDLVNDPDMVAMIAASAALTLSGAPFMGPIAGCRVGYEDGEYILNPEVSDMDGLRNNPDQRLDLVVAGTQDAVMMVESEAYELSEAEMLGAVKFGHEQIQPVIDLIIDLAEEAAKEPFDFTPPDYSALYDAVKKAGEKDMRAAFALTDKQERTNAISAARETVKASLSEEQLSDANLG-----SAFKKLESSILRGDVVKGGKRIDGRALDTVRPIVSEVGILPRTHGSSLFTRGETQALVVTTLGTGEDEQIIDALHGNSRSNFLLHYNFPPYSVGEVGRVGPPGRREIGHGKLAWRALQAVLPAATDFPYTIRLVSEITESNGSSSMATVCGSSLAMMDAAVPLKAPVAGVAMGLILEDDGSYAVLTDILGDEDHLGDMDFKVAGTEEGITSLQMDIKVAGITPEIMEKALEQARAGRLHILGEMSKALSEGRQEFSAHAPRIETMTVPTDKIREVIGSGGKVIREIVETSGAKVDINDDGVIKIASPNNESIQKAYDMIYSIVAEPEEGKVYKGKVVKIVDFGAFVNFFGKRDGLVHVSQIENRRLNHPSDVLKEGQEVWVKLLGFDDRGKVRLSMKMVNQETGEEET---KAGEEA---MEIREALTFDDVLLVPAASSVLPSTAETVTHVTKSIKLNIPLLSSAMDTVTEARMAIAMAQAGGMGVVHRNLGVDEQADQVRQVKRFESGVVYKPITLRPDQTLADAKALQERYRVTGFPVVDESNRVLGIVTNRDMRFAHDLDTPVHAMMTSERLAMLQEPADLDEARLLMKERRIEKLLVVDGTGKLTGLLTLRDSEQAVLNPQACKDELGRLRVAAATTVGDAGFERSEALIDAGVDMIVVDTAHGHSASVAEAVERIKRVSNKVQVVAGNVATGSATKALIDAGADGIKVGIGPGSICTTRMVAGVGVPQLTAIMDCAEAAMQAGVPVIADGGIKFSGDFAKAIAAGASCAMVGSMIAGTDESPGEVILYQGRSYKSYRGMGSLGAMARGSADRYFQKDAASDKLVPEGIEGQVPYKGSAGTVIHQLVGGLRAAMGYTGNRTVEEMRRNCQFVRITGAGLKESHVHDVQITRESPNYRI--G-MSVLVDKNTRVICQGITGSQGTFHSEQAIA-YGTNMVGGVTPGKGGQ-----QHLNLPVFNSVHEAMAATDANATVIYVPPPFAADSILEAIDAKIPLVVAITEGIPVLDMMRVKRALEGTDTILIGPNCPGVITPGECKIGIMPGHIHRKGTVGVVSRSGTLTYEAVKQTSDVGLGQSSCVGIGGDPIKGTEHIDVLEWFLADDDTHSIIMIGEIGGSAEEEAAQFLKDEAKRGRKKPVAGFIAGRTAPPGRRMGHAGAIVAGGKGGADDKIEAMKSAGIVVAESPAGLGEAVLKAI--G--MATERTLSIIKPDATKRNLTGKINAKFEDAGLRIVAQKRLHLTKAQAGKFYEVHAERPFYDELCEFMASGPVVAQVLEGEGAIAKNREVMGATNPADAAAGTIRAEFAESVGENSVHGSDAAETAKEEISFFFSGLEIVG-MSLNSVRPWRSIDRRKSRQIHVGRVPVGGDAPISVQTMTNTDTSDVAATVAQIQAAAEAGADIVRVSTPDESSTRALREIVAESPVPIVADIHFHYKRAIEAAEAGAACLRINPGNIGDKTRVAEVIRAARDHGCSIRIGVNAGSLERHLLEKYGEPCPEAMVESGLEHIRILEDADFHEFKISVKASDVFLAAAAYQGIAEATDAPIHLGITEAGGLMSGTIKSAVGLGNLLWSGIGDTIRVSLSADPVEEVKVGFEILKSLGLRHRGVNIISCPSCARQGFDVIRTVETLEKRLEHIRTPMSLSIIGCVVNGPGEALMTDVGFTGGGKDAGMVYMAGKQSHKMSNEQMVDHIVGLVETRAAEIQKTLDAEAEAEEKARDSGDVAKAGMIPRYSRPEMVAIWSPETKFRIWYEIEAHACDAQADLGVIPRDNAAAVWKARDVEFDVARIDEIEAVTKHDVIAFLTHLAEIIGNDEARFVHQGMTSSDVLDTTFNVQLVRATDLLLADMDKLLDALKRRALEHKDTVRIGRSHGIHAEPTTMGLTFARFYAEMDRNRTRLRAARAEIATGAISGAVGTFANIDPAVEAHVCEKLGLEPEPISTQVIPRDRHAAFFATLGVIGSSIENVATEIRHMQRTEVLEAEEFFSKGQKGSSAMPHKRNPVLTENLTGLARLVRMAVVPAMENVALWHERDISHSSVERNIGPDTTITLDFALNRLTQVIDKLVIYPDNMLANMNKFRGLVMSQRVLLALTQAGVSREDSYKLVQRNAMKVWEEGKDFKTELLGDKEVLAALSAEEIEEKFDLGYHTKHVDTIFKRVFGD--MAHKKAGGSSRNGRDSAGRRLGVKKFGGEAVIPGNIIVRQRGTKWWPGEGVGLGKDHTIFATAEGHVTFHKGLKGRTFISVMPAAEAAE------------MLGGKRILLVIGGGIAAFKSLDLIRRLRDEGATVIPVLTRAATEFVTPLSVSSLAAQRVWTDLFDLTDEAEMGHIELSRAADLVVVAPCTADLMAKMAGGHADDLASTLLMATDKRVLIAPAMNVRMWEHPATRRNLNTLTGDGILCVGPDVGNMACGETGPGRMSEPPAIVDAIRAALSGDTGDRPLAGRHVIVTSGPTHEPIDPVRYIANRSSGAQGTAIAQALVALGARVTFVTGPATVPPPAGVDLRAIETAEQMRQAVEGALPADAAVLAAAVADWRIESPAQGKIKKDRNGAPPALSFAENRDILAELSRPGPD-RPALVVGFAAETDDVIANATAKRARKGCDWIVANDVSPGTGIMGGAENAVTLIRADGAETWERMSKPEVAARLARRMAEALGHGTAEEAAQ--MDELLR---EYLPIVVFLGVAIGLGVVLILAATIVAVRNPDPEKVSAYECGFNAFDDARMKFDVRFYLVSILFIIFDLEVAFLFPWAVAFKDVGLLGFWSMMVFLAVLTIGFAYEWKKGALEWE-MSNQDVLKTIKDEEVEYVDIRFTDPRGRLQHVTIIVDEIDEDFLEEGFMFDGSSIAGWKSIDQSDMKLMPDTDSAYIDPFYAEKTLCIHCTVVEPDTNEPYDRDPRGTAEKAEAYLKSSGIGDEAFFGPEAEFFLFDDVRYSNSINKVSYEVDAADASWNTGTEYE----MGNMGHRPGVKGGYFPVNPTDAAQDLRSEMLSTMKRMGMKVDKHHHEVASCQHELGLIFGSLTKQADEIQKYKYVIHNVAHAYGKTATFMPKPIAGDNGTGMHVNMSIWKDGKPLFAGDKYADLSQEALWFIGGILKHAKSLNAFTNPSTNSYKRLIPGFEAPVLRAYSARNRSGCVRIPWTESPKAKRVEARFPDPAANPYLAFSALLMAGLDGIKNKIDPGAASDKDLYDLPPEELAQIPTVCASLREALDELEADHEYLLAGDVFTRDQIKGYVNLKWEEVYAYEHTPHPIEFQMYYSC

>Hyphomonas-polymorpha-PS728
[truncated: 5,754,780 more chars]
